# Identification and Validation of a Proliferation-Associated Score Model Predicting Survival in Lung Adenocarcinomas

**DOI:** 10.1155/2021/3219594

**Published:** 2021-10-21

**Authors:** Yunyi Bian, Qihai Sui, Guoshu Bi, Yuansheng Zheng, Mengnan Zhao, Guangyu Yao, Liang Xue, Yi Zhang, Hong Fan

**Affiliations:** Department of Thoracic Surgery, Zhongshan Hospital, Fudan University, Shanghai, China

## Abstract

**Aim:**

This study is aimed at building a risk model based on the genes that significantly altered the proliferation of lung adenocarcinoma cells and exploring the underlying mechanisms.

**Methods:**

The data of 60 lung adenocarcinoma cell lines in the Cancer Dependency Map (Depmap) were used to identify the genes whose knockout led to dramatical acceleration or deacceleration of cell proliferation. Then, univariate Cox regression was performed using the survival data of 497 patients with lung adenocarcinoma in The Cancer Genome Atlas (TCGA). The least absolute shrinkage and selection operator (LASSO) model was used to construct a risk prediction score model. Patients with lung adenocarcinoma from TCGA were classified into high- or low-risk groups based on the scores. The differences in clinicopathologic, genomic, and immune characteristics between the two groups were analyzed. The prognosis of the genes in the model was verified with immunohistochemical staining in 100 samples from the Department of Thoracic Surgery, Zhongshan Hospital, and the alteration in the proliferation rate was checked after these genes were knocked down in lung adenocarcinoma cells (A549 and H358).

**Results:**

A total of 55 genes were found to be significantly related to survival by combined methods, which were crucial to tumor progression in functional enrichment analysis. A six-gene-based risk prediction score, including the proteasome subunit beta type-6 (PSMB6), the heat shock protein family A member 9 (HSPA9), the deoxyuridine triphosphatase (DUT), the cyclin-dependent kinase 7 (CDK7), the polo-like kinases 1 (PLK1), and the folate receptor beta 2 (FOLR2), was built using the LASSO method. The high-risk group classified with the score model was characterized by poor overall survival (OS), immune infiltration, and relatively higher mutation load. A total of 9864 differentially expressed genes and 138 differentially expressed miRNAs were found between the two groups. Also, a nomogram comparing score model, age, and the stage was built to predict OS for patients with lung adenocarcinoma. Using immunohistochemistry, the expression levels of PSMB6, HSPA9, DUT, CDK7, and PLK1 were found to be higher in lung adenocarcinoma tissues of patients, while the expression of FOLR2 was low, which was consistent with survival prediction. The knockdown of PSMB6 and HSPA9 by siRNA significantly downregulated the proliferation of A549 and H358 cells.

**Conclusion:**

The proposed score model may function as a promising risk prediction tool for patients with lung adenocarcinoma and provide insights into the molecular regulation mechanism of lung adenocarcinoma.

## 1. Introduction

Lung cancer is one of the most common malignant cancers characterized by a high incidence and the highest mortality worldwide, with an average 5-year survival rate of <15% [1, 2]. Lung adenocarcinoma (LUAD) is currently the major subtype of lung cancer, accounting for nearly 60% of new cases, characterized by poor survival [3]. Early surgical excision is the standard treatment strategy now. For patients with high-risk LUAD, they should receive radiation, chemotherapy, or targeted immunotherapy after surgery to improve survival [2]. Nearly 50% of patients are at risk of postoperative recurrence, and tumor recurrence in high-risk patients is an important cause of death [4, 5]. Therefore, accurate identification of high-risk patients and early intervention with adjuvant therapy mentioned earlier are very important for improving the prognosis.

Currently, the TNM staging system plays a critical role in risk assessment and therapy guidance. However, these risk assessment factors based on clinical pathological characteristics can not achieve early identification of patients with poor prognosis and can not be accurate to predict patients' response to adjuvant treatment; more precise risk prediction models need to be established, such as scoring models that contain molecular characteristics of LUAD.

In recent years, public databases such as The Cancer Genome Atlas (TCGA) and Gene Expression Omnibus (GEO) provide large lung cancer datasets. At the same time, techniques such as high-throughput sequencing, combined with machine learning methods, have been used to explore more types of biomarkers, identify the signaling pathways, reveal molecular mechanisms, and make clinical prognosis predictions based on large datasets [6]. Several prognostic models for LUAD based on the above techniques have been published, but these models still have limitations. The biomarkers selected had a minimal relationship with tumor proliferation and cannot fully reflect the proliferation potential of LUAD [7–9]. The cancer dependence of genes is the basis of prognosis prediction and drug target research.

Recently, the Cancer Dependency Map (DEPMAP), using genome-scale CRISPR screens in hundreds of cell lines, was used to establish a comprehensive and systematic identification of the genetic and pharmacological dependence of cancer and its prediction biomarkers [10–12]. Therefore, genes closely related to LUAD's proliferation were identified from DEPMAP, and a risk prediction score model was built based on the expression of these genes and survival information in this study. The proposed model could successfully predict prognosis in patients from TCGA and GEO. According to the model, somatic mutations, differentially expressed genes, microRNAs, and immune infiltration patterns were further analyzed to reveal the regulatory factors, cellular processes, and signaling pathways associated with the model-related genes in LUAD. Finally, the effects of some model-related genes on tumor proliferation were verified *in vitro*. The study was meaningful for elucidating the molecular mechanism of proliferation in LUAD and accurately predicting patient prognosis to provide individualized treatment.

## 2. Materials and Methods

### 2.1. Data Preprocessing

First, data of 60 LUAD cell lines and the CERES dependency score of genes from DEPMAP (https://depmap.org/) were obtained. The CERES dependency score of the genes represented the effect on cell survival by knocking out individual genes with CRISPR-Cas9 genetic perturbation reagents. A lower CERES score indicated a higher likelihood that the gene of interest was essential in a given cell line. A score of 0 indicated that a gene was not essential; correspondingly, a score of -1 was comparable to the median of all pan-essential genes [13].

The public datasets of LUAD patients (*n* = 497) were downloaded from the UCSC Xena Browser, and the expression data (FPKM form) was matched with patients' survival information downloaded from TCGA. The patients with missing survival data were excluded. The miRNA data, the somatic mutation data, and the copy numbers' variation were obtained from the Xena Browser.

The public datasets from the GEO (https://www.ncbi.nlm.nih.gov/geo/) were used for the validation cohort. A total of 930 samples from the datasets GSE30219 [14], GSE31210 [15, 16], GSE3141 [17], GSE37745 [18–21], GSE50081 [22], and GSE68465 [23] representing different independent studies of LUAD were enrolled. The batch effect caused by the heterogeneity among different studies was eliminated with the COMBAT empirical Bayes method using the sva package [24], and background adjustments and quantile normalization were conducted using the limma package [25].

Next, paraffin-embedded specimens of the tumor and adjacent healthy tissues were collected from 100 patients with LUAD who underwent radical surgery in the Department of Thoracic Surgery, Zhongshan Hospital, Fudan University, from September to November 2015. The survival information of the 100 patients was collected by the follow-up until December 2020, excluding the ones who succumbed. All participants signed informed consent according to the ethical requirements in the Declaration of Helsinki. Ethics approved by the ethical committees of Zhongshan Hospital (B2019-035).

### 2.2. Gene Selection and Prediction Score Model Construction

The data were processed by the R software (Version 3.5.3) and the GraphPad Prism software (version 7.0). The independent hazard rate of each gene was calculated using univariate Cox regression with the survival package in R, and *P* value < 0.05 was considered statistically significant. The ClusterProfiler package [26] was also adopted to analyze the functional enrichment of the selected genes. The cutoff of GO and KEGG terms comprised the adjusted *P* value < 0.05 and the false discovery rate (FDR) < 0.05.

The LASSO Cox regression analysis, a penalized method to select data with high dimensions and reduce the impact of overfitting, was used to build the predictive score model [27, 28]. Tenfold cross-validation was adopted using the glmnet package [29] in R to determine the optimal model parameter *λ* and corresponding coefficients. The optimal *λ* was determined as the smallest partial likelihood deviance. A multivariate Cox regression of the six genes was conducted, and their coefficients were applied to build the score model. Harrell's concordance index (C-index) [30] was applied to measure the predictive accuracy of the score model preliminarily.

### 2.3. Survival Data Analysis

The survival curves were visualized using the ggplot2 package by the Kaplan–Meier method. Log-rank tests exhibited the difference in overall survival (OS). The rms package in R was used to build a nomogram of the 497 LUAD samples from TCGA, and the calibration plots were shown. The univariate and multivariate Cox proportional risk analyses were conducted to show the score model's prognostic value when age, gender, and stage were adjusted. C-index was also calculated to identify the value of the score model.

### 2.4. Differentially Expressed Genes, microRNAs (miRNAs), and Somatic Mutation Distribution

The limma package [25] was adopted to identify differentially expressed genes (DEGs) and miRNAs between high and low score groups. The moderated *t*-test was used to calculate DEGs and miRNA expression changes, and the *P* value was adjusted as FDR by Benjamini and Hochberg method [31]. The log fold change was set as >0.5 and the adjusted *P* value < 0.05 as the cutoff criteria.

We used the maftools package basing on the Kruskal-Wallis test to compare the distribution of somatic mutations and the types of copy number variations. The adjusted *P* value < 0.01 was used to assess the significance of the mutational frequency.

### 2.5. Immune Cell Infiltration in the Two Groups

We selected the gene markers reported by Bindea et al. according to the previous studies [32–34]. A synopsis of genes associated with microenvironment cell sets was constructed precisely, which contained 585 genes depicting 24 tumor microenvironment- (TME-) infiltration cell populations related to innate immunity and adaptive immunity. The subsets included B cells, dendritic cells (DCs), immature DCs, activated DCs, neutrophils, mast cells, eosinophils, macrophages, natural killer (NK) cells, NK CD56bright cells, NK CD56dim cells, cytotoxic cells, T cells, CD8 T cells, and Th1, Th2, Th17, Tfh, Tgd, T*γδ*, T helper, Tcm, Tem, and Treg cells (Table S1). We employed a seven-gene panel introduced in the POPLAR (patients with previously treated non-small-cell lung cancer) trial as a surrogate index to identify infiltration pattern of effector T-cell (CD8A and CXCL10) and IFN-*γ* associated cytotoxicity (IFNG, GZMA, GZMB, EOMES, and TBX21) [35]. The CYT (cytolytic activity) score was defined by Rooney et al. [36]. We used it to calculate the geometrical mean of PRF1 and GZMA, which can reflect the significance of the response to antitumor. The pheatmap package was used to plot the 24 immune cell infiltrating patterns from different patients.

### 2.6. Immunohistochemistry

The tissue specimens were collected from both tumor and tumor-adjacent areas of 100 patients with LUAD who received lung surgery from September to November 2015 in the Zhongshan Hospital. The paraffin-embedded tissues were dewaxed, rehydrated, and stained using a GTVision + Detection System/Mo&Rb Immunohistochemistry kit (GK500710, GeneTech, Shanghai, China) following the manufacturer's protocol. Anti-PSMB6 (1 : 50, abs116436, Absin Bioscience Inc., Shanghai, China), anti-HSPA9 (1 : 50, abs135628, Absin), anti-DUT (1 : 50, abs102198, Absin), anti-CDK7 (1 : 50, abs136079, Absin), anti-PLK1 (1 : 100, ab17056, Abcam, Cambridge, UK), and anti-FOLR2 antibodies (1 : 50, abs107177, Absin) were used. The detailed procedure can be found in a previous study [37].

### 2.7. Cell Culture and siRNA Transfection

Two LUAD cell lines (A549 and H358) were purchased from the Chinese Academy of Science Cell Bank and cultured in high glucose Dulbecco's Modified Eagle's Medium (Hyclone, UT, USA) supplemented with 10% fetal bovine serum (Every Green, Hangzhou, Zhejiang, China), 100 U/mL penicillin, 0.1 mg/mL streptomycin, and 0.25 *μ*g/mL amphotericin B (Sangon Biotech, Shanghai, China) in a humidified 5% CO_2_ atmosphere at 37°C.

Two small interfering RNAs (siRNAs) targeting PSMB6 (si-PSMB6-1 and si-PSMB6-2), two siRNAs targeting HSPA9 (si-HSPA9-1 and si-HSPA9-2), and two negative control siRNAs (siCtrl-1 and siCtrl-2) were designed and purchased by Guangzhou RiboBio Co., Ltd. (RiboBio). Target sequences of the siRNAs can be found in Table S7. SiRNAs were transfected with a 100 nM Lipo8000 transfect reagent (Beyotime, Haimen, Zhejiang, China) and Opti-MEM (Thermo Fisher Scientific, MA, USA) following the manufacturer's protocol.

### 2.8. RNA Extraction and Quantitative Real-Time Polymerase Chain Reaction

TRIzol reagent (Tiangen Biotechnology Co., Beijing, China) served as an RNA extraction reagent. A PrimeScript RT Reagent Kit (TaKaRa, Tokyo, Japan) was used to synthesize the cDNA template, and SYBR Premix Ex Taq (TaKaRa) was used to perform quantitative real-time polymerase chain reaction following the manufacturer's protocol. All reactions were analyzed in a QuantStudio 5 (Thermo Fisher Scientific). The 2^-*ΔΔ*CT^ method using GAPDH as an endogenous calibrator was adopted to relatively quantify the mRNA. All primers were synthesized by Sangon Biotech, and the sequences can be seen in Table S2.

### 2.9. Western Blot Analysis

Western blot analysis was performed as described earlier [37]. RIPA buffer (Beyotime) with protease and phosphatase inhibitor cocktail (Topscience Co., Shanghai, China) was used to extract proteins from cells. Proteins were quantified using an Enhanced BCA Protein Assay Kit (Beyotime), separated with SDS-PAGE, and transferred onto polyvinylidene fluoride membranes (Merck-Millipore, MA, USA). Furthermore, 10% nonfat milk was used to block the membranes for 2 h and then incubated with specific primary antibodies for 12 h at 4°C. Tris-buffered saline-Tween 20 (TBST) solution was used to wash the membranes three times, and the secondary antibody dilutions were incubated on the membranes at room temperature for 1 h. Finally, the protein bands were visualized using a Moon Chemiluminescence Reagent kit (Beyotime). In this study, the following antibodies were used: anti-HSPA9 (1 : 1000, abs135628), anti-PSMB6 (1 : 1000, abs135628), anti-tubulin (1 : 1,000, AT-819, Beyotime), horseradish peroxidase- (HRP-) labeled goat anti-mouse IgG (H + L) (1 : 1,000, A0216, Beyotime), and HRP-labeled goat anti-rabbit IgG (H + L) (1 : 1,000, A0208, Beyotime).

### 2.10. Cell Proliferation Analysis

Green fluorescent protein- (GFP-) overexpressing cells were first transfected with siRNAs (si-PSMB6-1, si-PSMB6-2, si-HSPA9-1, si-HSPA9-2, si-NC-1, and si-NC-2) at a 100 nM final concentration using Lipo8000 transfection reagent (Beyotime) and Opti-MEM (Thermo Fisher Scientific). Then, 1,500 cells in the logarithmic growth phase were digested and inoculated in blank 96-well plates (Life Science, NY, USA) with 100 *μ*L of cell suspension in every well. Following incubation for 24, 48, 72, 96, and 120 h at 37°C, cell proliferation was measured according to corresponding fluorescence intensity using a Celigo cytometer (Cyntellect Inc., CA, USA), which was equipped with a 4-megapixel CCD camera with an F-theta scan lens.

## 3. Results

### 3.1. Gene Selection

**Figure 1:**
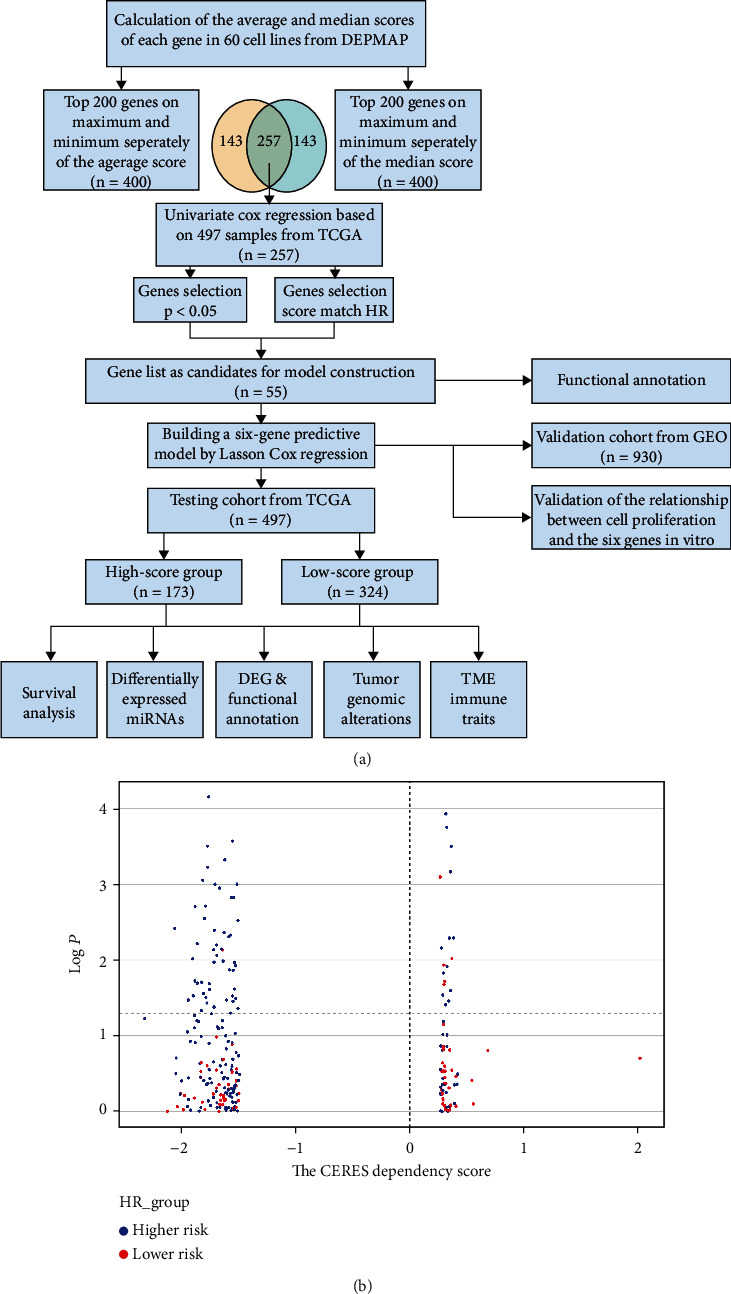
(a) Design of the study. (b) The scatter dot plots show the relationship between the CERES dependency scores and the *P* value of the 257 genes we selected before.

The design of this study is shown in Figure 1(a). First, the CERES dependency score of genes with 60 LUAD cell lines from DEPMAP was obtained. A score less than zero showed that the gene knockout inhibited cell proliferation; the smaller the score, the more pronounced the effect. A score greater than zero showed the opposite effect.

The average and median scores of each gene were calculated in 60 cell lines. The top 400 genes on the minimum and maximum of the average and median, respectively, were selected to match, and 257 genes were finally obtained, which meant that cell proliferation was dramatically accelerated or deaccelerated when the genes were knockout. Next, univariate Cox regression was performed on the 257 genes in 497 samples from TCGA. The genes significantly related to survival were retained (*P* < 0.05). The genes that showed the same tendency in cell proliferation (the CERES dependency score) and survival (HR) were matched, and 55 genes finally remained (Figure 1(b), Table S3). The results showed that all these genes greatly influenced cell proliferation and significantly correlated with the survival of LUAD patients.

**Figure 2:**
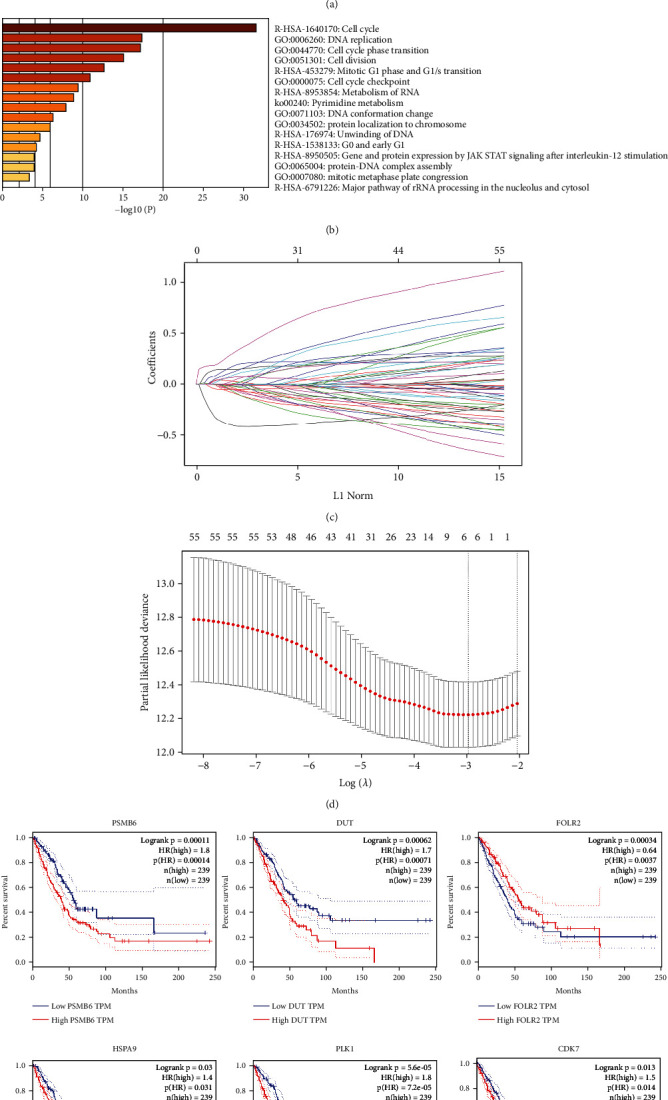
(a) The network showed the interactions among the enriched pathways of the 55 genes. The circle nodes' size represents the number of input genes that fall into that pathway, and its color represents its cluster identity. The description of each cluster was shown in the label. The same enrichment network has its nodes colored by *P* value, as shown in the legend. The dark the color, the more statistically significant the node is (see legend for *P* value ranges). (b) GO and KEGG functional enrichment analyses of the enriched terms. (c) Coefficient profiles of variables in the LASSO Cox regression model. (d) Tenfold cross-validation for turning parameter selection in the LASSO Cox regression model. *λ* is the turning parameter. The partial likelihood deviance is plotted in log(*λ*), in which vertical lines are shown at the optimal values by minimum criteria and 1 − SE criteria. (e) Kaplan-Meier curves of overall survival (OS) stratified by the six genes in TCGA.

The analysis of the enrichment of GO and KEGG on these 55 genes was performed using the R cluster profile package. These genes were significantly related to tumor progression, including DNA replication, nuclear division, and cell cycle (Figures 2(a) and 2(b)).

### 3.2. Construction of the Score Model

After LASSO Cox analysis, six genes, including PSMB6, HSPA9, DUT, CDK7, PLK1, and FOLR2 (Figures 2(c) and 2(d), Table S4), were selected to construct the optimal prognostic model. All the six genes were significantly related to survival (Figure 2(e)). The CERES dependency scores and HRs from univariate Cox regression of the six genes indicated that PSMB6, HSPA9, DUT, CDK7, and PLK1 served as oncogenes, while FOLR2 served as a tumor-suppressor gene. The expression values of most of them were significantly correlated (*P* < 0.05) (Fig. S1A). The correlation between clinical characteristics (sex, age, stage, and smoking) and gene expression is shown in Figure S1B.

**Figure 3:**
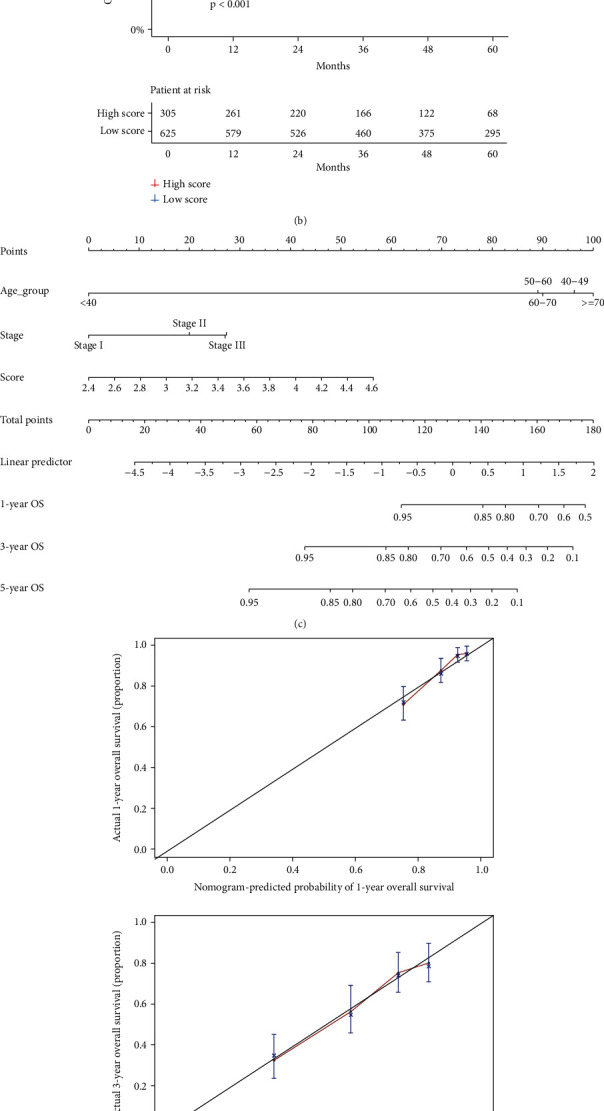
(a) Kaplan–Meier curves of OS in patients from TCGA in the two groups. (b) Kaplan–Meier curves of OS in patients from GEO in the two groups. (c) The nomogram of the overall survival prediction model. (d) Calibration plots for the nomogram: 1-, 3-, and 5-year nomogram.

A risk-predicted score model was built based on their coefficients with multivariate Cox regression (Additional file 1). Based on the risk predicting score model, 497 patients with LUAD from TCGA were assigned to low score (*n* = 324) and high score (*n* = 173) groups by the optimal cutoff value (3.539). Patients with a high score had a significantly poorer OS (*P* value < 0.0001, Figure 3(a)) compared with those with a low score, indicating the accuracy of the prediction model.

### 3.3. Validation and Clinical Significance of the Score Model

The univariate and multivariate Cox regression analyses were performed to demonstrate that the score model was an independent prognostic factor in patients with LUAD compared with other clinicopathological factors such as age and stage (Table 1). A nomogram based on the multivariate analysis (*P* < 0.05) of the OS of patients with LUAD from TCGA was used to show the prediction (Figure 3(c)). Great calibration plots were shown for the 1-, 3-, and 5-year OS rates of patients with LUAD (Figure 3(d)). The corresponding C-index showed that the combination of the score model, age, and stage performed remarkably (Table 2).

The results of immunohistochemistry further demonstrated the functions of genes. The overexpression of PSMB6, HSPA9, DUT, CDK7, and PLK1 was seen in resected tumor tissues compared with adjacent normal tissues, while FOLR2 was expressed more in normal tissues (Figure 6(d)). One hundred patients from the institution were divided into high and low expression groups according to the expression of the six genes. Patients with high expression of PSMB6, HSPA9, DUT, CDK7, and PLK1 had a significantly poorer OS (*P* value < 0.0001, Figure 6(e)), while patients with high expression of FOLR2 showed the opposite result.

GEO samples were obtained to verify the score model. Furthermore, 930 patients with LUAD from GEO were also classified into high and low score groups; the survival analysis showed a significant difference in OS between the two groups (*P* value < 0.0001, Figure 3(b)), which was consistent with the data from TCGA.

### 3.4. Somatic Mutation, DEGs, and Differentially Expressed microRNA (miRNA) in the Two Groups

**Figure 4:**
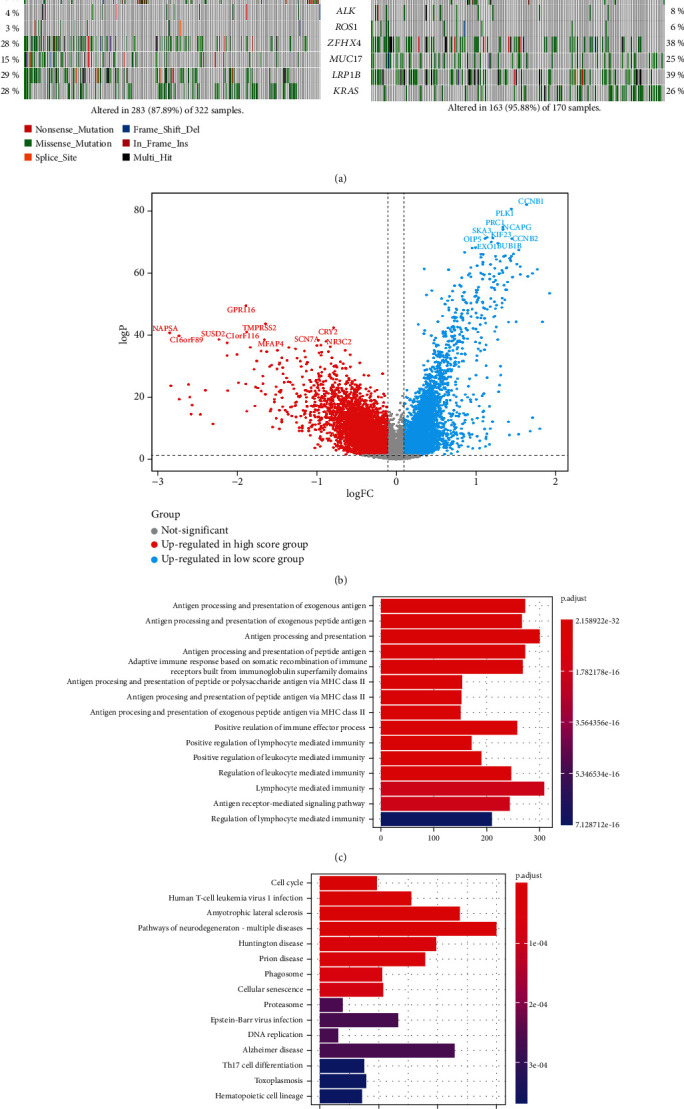
(a) The waterfall plots show the somatic mutations and copy numbers' variations in the two groups. (b) The volcano plot displays the DEGs of the two groups. (c, d) GO (c) and KEGG (d) functional enrichment analyses of the DEGs.

As reported before, the number of somatic mutations had relationships with survival. The distribution of somatic genomic mutations and the copy numbers' variation in the high and low score groups were analyzed. The average somatic mutation numbers of each sample in high and low score groups were 95.88 and 87.89, respectively (Figure 4(a)). TP53 was highly mutated in the high score group (65% in the high score group, 39% in the low score group, *P* value < 0.001). Other genes, such as EGFR (15% in the low score group, 7% in the high score group, *P* value = 0.013), had lower mutation rates in the high score group (Figure 4(a) and Table S5).

The expression of DEGs was analyzed to draw the landscape of the difference in biological characteristics between the two groups. In total, 4,863 genes, including GPR116, TMPRSS2, and CYR2, were upregulated (all adjusted *P* < 0.01) while 5,001 were downregulated in the high score group, including CCNB1, PLK1, and PRC1 (Figure 4(b), Table S6). Functional enrichment of GO and KEGG in the 9864 DEGs was analyzed, and the pathways were related to the high score group. The antigen processing and presentation and positive regulation of the immune effector process corresponded to the high score group (Figures 4(c) and 4(d)). Several classic metabolic pathways ranked top in the low score group.

Also, the miRNA expression and distribution of somatic mutations were analyzed. In conclusion, 75 miRNAs, including miR-99a-5p, miR-497-5p, and miR-29c-3p, were upregulated (all adjusted *P* < 0.01), and 63 were downregulated in the high score group, including miR-106b-5p and miR-128-1-5p (Fig. S1C and Table S7).

### 3.5. TME Infiltration Characteristics in the Two Groups

Based on the high scores and low scores of 497 patients from TCGA, the infiltration patterns of each patient in 24 immune cell populations associated with innate immune and adaptive immune processes were determined.

**Figure 5:**
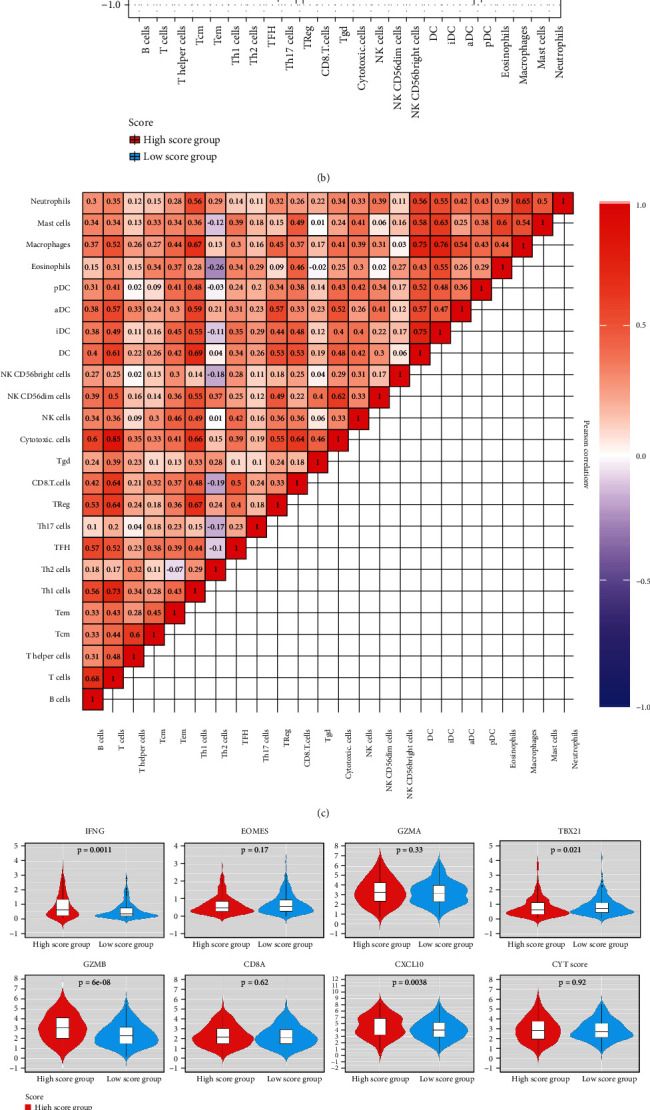
TME characteristics of the two groups. (a) Infiltration patterns of immune cells for 497 LUAD patients from TCGA. Clinical and pathological features contain age, gender, stage, smoking status, and score groups. (b) The proportion of immune cells in the two groups. The scattered dots show the immune cells' score. The median, third, and first quartile values are shown in the boxplots. ^∗^*P* < 0.05; ^∗∗^*P* < 0.01; ^∗∗∗^*P* < 0.001; ^∗∗∗∗^*P* < 0.0001. (c) Relationships between the 24 immune cells in LUAD patients from TCGA. (d) Violin plots show the expression profiling of the f7 immune-related genes in the POPLAR study and cytolytic activity (CYT) score. (e) Relative expression level of molecules associated with the innate immune activity (shown at left) and MHC-I/II antigen-presenting process (shown at right). (f) Violin plots displaying the CD8+ T cells/Treg ratio of the infiltration groups. (g) Relative expression level of immune coinhibitors (shown at left) and costimulators (shown at right).

The results showed high infiltration of T cells (*P* = 1.26*E*^−06^), Th1.cells (*P* = 1.62*E*^−05^), cytotoxic cells (*P* = 0.001491), and pDC cells (*P* = 3.54*E*^−07^), except for Th2.cells (4.45*E*^−26^), which exhibited low infiltration in the low score group (Figures 5(a) and 5(b)). The Wilcoxon test was used to verify the different infiltration patterns (Table S8). The nearly comprehensively positive relativity among the enrichment level of 24 microenvironment cell populations can be seen in Figure 5(c), which was related to the coinfiltration effect.

The TME characteristics in the high and low score groups were described by conducting a full analysis of the expression level of several genes and cytokines associated with immunity from the data of 497 patients with LUAD. A seven-gene panel designed in the POPLAR trial was chosen as a substitute indicator to quantify the cytotoxicity related to IFN-*γ* (IFNG, EOMES, GZMA, TBX21, and GZMB) and effector T cells (CD8A and CXCL10) [36]. Then, the score was examined according to the score of cytolytic activity reported previously [38], representing the geometric meaning of GZMA and PRF1, to reflect the importance of the antitumor response (Figure 5(d)). The high score group had higher expression levels of GZMA, IFNG, GZMB, CD8A, and CXCL10. (most *P* < 0.05), demonstrating that these patients had a more efficient cytotoxic function. As for the molecules, the low score group showed more activity of the innate immune response. TLR9, AIM2, and NLRP6 showed similar tendencies (Figure 5(e), left). Furthermore, compared with the high score group, the low score group had an enriched abundance of MHC-I/MHC-II-related antigen-presenting molecules(most *P* < 0.001; Figure 5(e), right).

The low score group had enrichment with active innate and adaptive immune cells and immunosuppressor cells such as Tregs and iDCs (Figures 5(a) and 5(c)). Based on this result, the CD8+ T cell/Treg cell ratio was used to estimate the importance of activated and suppressed immunity (Figure 5(f)). The low score group had a higher ratio, which meant that the TME in the low score group was more activated. To verify the result, the expression of several immunoregulators in the two groups was revealed, which included checkpoint molecules (*n* = 15) (Figure 5(g), left) and costimulating molecules (*n* = 20) (Figure 5(g), right). The heat map showed that more costimulating molecules and coinhibitory molecules were expressed in the low score group (most *P* < 0.05). Based on the result, it was concluded that patients in the low score group might benefit from immune checkpoint inhibitors (ICI).

### 3.6. Silencing of PSMB6 and HSPA9 Inhibited the Proliferation of LUAD Cells

**Figure 6:**
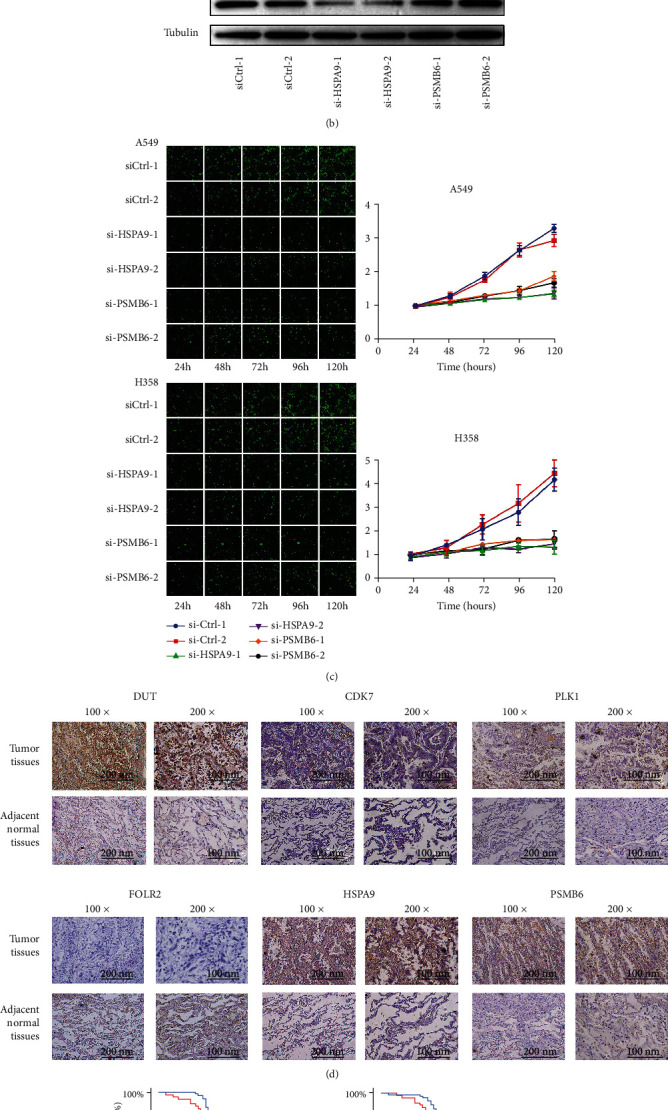
(a, b) Quantitative RT-PCR (a) and western blotting analyses (b) verifying the PSMB6 and HSPA9 knockdown efficiency in A549 and H358 cells. (c) The effects of PSMB6 and HSPA9 knockdown on cell proliferation in A549 and H1299 cells. ^∗∗∗∗^*P* < 0.0001. (d) Representative IHC staining images indicating the expression of the six genes in lung adenocarcinoma and adjacent normal tissues. (e) Kaplan-Meier curves of overall survival according to immunohistochemical staining of the six genes in patients from our institution.

According to the CERES dependency score from DEPMAP, the perturbation of PSMB6 and HSPA9 by knockout in different LUAD cell lines inhibited proliferation. To validate the result and explore the function of PSMB6 and HSPA9 in tumor formation and growth, siRNAs targeting PSMB6 and two siRNAs targeting HSPA9 each were transfected into two LUAD cell lines (A549 and H358). Two different siRNAs targeting each gene were used to attenuate the off-target effects. The stable knockdown efficiencies of siRNAs were verified by comparing them with those in the control cells at both mRNA and protein levels (Figures 6(a) and 6(b)). The results of cell counting demonstrated that the proliferation ability of A549 and H358 cell lines after PSMB6 and HSPA9 knockdown significantly decreased compared with that in the control cells (Figure 6(c)).

## 4. Discussion

In this study, six genes were identified via comprehensive analysis, including PSMB6, HSPA9, DUT, CDK7, PLK1, and FOLR2, which were significantly related to cell proliferation, to construct a risk prediction score model used as an independent predictor. The prediction score model based on these six genes predicted the prognosis of patients with LUAD accurately in the testing cohort from TCGA and the verifying cohorts from GEO. Based on previous findings, it was concluded that silencing the least reported genes PSMB6 and HSPA9 inhibited cell proliferation *in vitro*, consistent with the results of DEPMAP.

The DEPMAP was created to systematically identify genetic alterations of cancer and their influence by collecting genetic information of hundreds of cancer cell line models [12]. Project Achilles provided the foundation for DEPMAP, which systematically identified and cataloged the essentiality of genes across hundreds of genomically characterized cancer cell lines. Lentiviral-based pooled RNAi or CRISPR/Cas9 libraries were used as highly standardized genome-scale pooled loss-of-function screening, which guaranteed the stable suppression of individual genes. Computational models such as DEMETER [39] for RNAi screening and CERES [13] for CRISPR screening were adopted to determine gene essentiality more accurately. Recently, Szalai et al. [40] explored the mechanisms behind cell death and confounding factors of transcriptomic perturbation screens based on Project Achilles of the DEPMAP. Also, Zhou et al. [41] used the CERES score to identify the prognostic values of solute carrier (SLC) family genes for patients with LUAD. Other studies related to oncology drug discovery were dependent on the DEPMAP [11, 12].

In this study, the six genes used to build the predicted model strongly correlated with cell proliferation and survival of patients with LUAD. As previously reported, high levels of CDK7 mRNA and protein and overexpression of PLK1 were related to poor prognosis in NSCLC [42, 43]. The CDK7 and PLK1 inhibitors played a critical role in immunotherapies for lung cancer [44, 45]. PSMB6 regulated proteasome structure and function, variations in which affected the treatment of multiple myeloma [46]. Shi et al. [47] demonstrated that PSMB6 played a more important role in the proteasome structure than in functional activity. The mitochondrial HSP70 chaperone mortalin (HSPA9/GRP75) was often upregulated in MEK/ERK-deregulated tumors [48]. Wu et al. [49] demonstrated that the depletion effect of HSPA9 was sensitized by KRAS activity, suggesting that HSPA9 was a potential target for KRAS-mutated tumors.

The “cold tumor” in the high score group had a low level of infiltration. In contrast, the low score group was enriched in cytotoxic T cells (as immune activation) and Tregs and others (as immune suppression). As for immunomodulators, the high score group had a relatively low level while the low score group was abundant in immune-related cytokines or markers reported earlier [36, 38, 50, 51]. As showed earlier [38, 52], preexisting immunity, defined by the presence or absence of CD8+ T effector cells, can be used to discriminate immunotherapy-sensitive versus insensitive patients. In summary, we speculated that patients in the low score group could reap more benefits from ICI, and restoring preexisting immunity was crucial to a higher response rate. However, we fail to reveal the relationship between the mutation load and the cytotoxic factors. The low score group had a lower level of genomic mutation but higher immune infiltration comparing with the high score group. As reported before, mutational burden of the tumor may affect the ICI efficiency by enhancing tumor immunogenicity [53, 54].

This study had several limitations. First, although hundreds of samples from the GEO database and the institution were used as validation cohorts, more patients in prospective cohorts are still needed to verify the proposed risk prediction model. Also, the identification of immune subtypes in clinical samples was required to be performed to validate the function of immune cells in the training cohort. Besides, the roles of these genes and the molecular mechanisms in the tumorigenesis of LUAD could not be further explored due to the limitations on research funds and time in the present study. Future research should explore the interaction relationship between the relevant genes in the model and the pathways involved and downstream signaling factors.

In summary, the CERES score of genes from DEPMAP was used to identify six genes with a combination strategy and build a risk prediction score model that could effectively predict the survival of patients with LUAD from TCGA, GEO, and the institution. The study demonstrated that the genes in the prediction model were significantly related to cell proliferation *in vitro*. Moreover, the study described a comprehensive landscape of the regulator factors, signaling pathways, and immune infiltration patterns behind the model, which might help identify the high-risk patients and interfere with individualized treatment early.

## Figures and Tables

**Table 1:** Univariate and multivariate analysis of overall survival in LUAD patients from TCGA database.

	Univariable		Multivariable	
	HR (95% CI)	*P* value	HR (95% CI)	*P* value
Age				
<60	—	—	—	
60-70	0.82 (0.56-1.19)	0.29	0.97 (0.66-1.43)	0.88
>70	1.26 (0.87-1.83)	0.22	1.61 (1.09-2.36)	0.02
Stage				
Stage I	—	—	—	—
Stage II	2.32 (1.60-3.36)	<0.001	2.35 (1.61-3.43)	<0.001
Stage III	3.30 (2.24-4.85)	<0.001	2.91 (1.96-4.32)	<0.001
Stage IV	3.61 (2.08-6.26)	<0.001	3.20 (1.82-5.62)	<0.001
Gender				
Female	—	—	—	—
Male	1.09 (0.81-1.46)	0.59	0.92 (0.68-1.25)	0.595
Score groups (high vs. low)	2.83 (1.95-4.11)	<0.001	2.86 (1.94-4.22)	<0.001

**Table 2:** Comparison of the accuracy of survival prediction between the factors with and without the score.

	Age + gender+stage		Score + age + gender+stage	
	C-index	95% CI	C-index	95% CI
Training cohort	0.678	0.632-0.723	0.711	0.667-0.756

## Data Availability

All datasets were adopted in this study are available in the TCGA (http://portal.gdc.cancer.gov/), GEO (http://www.ncbi.nlm.nih.gov/geo/), and DEPMAP (https://depmap.org/).

## Supplementary Materials

Supplementary 1 Additional file 1 score: the score was used in the risk-predicted model to calculate the score of patients with LUAD.

Supplementary 2 Table S1: the table showed genes associated with microenvironment of the 24 immune cell subsets. Table S2: the table showed the sequences of all the siRNAs and primers used in this study. Table S3: the table showed 55 genes selected for LASSO Cox regression; all the 55 genes showed the same tendency in cell proliferation (the CERES dependency score) and survival (HR). Table S4: the table showed six genes used in the model and their LASSO coefficient after LASSO Cox regression. Table S5: the table showed the summary of genomic alterations in the two groups, including the somatic mutation numbers of each gene in high and low score groups. Table S6: the table showed the differentially expressed genes (DEGs) between high score group and low score group identified by limma. Table S7: the table showed the differentially expressed miRNAs between high score group and low score group identified by limma. Table S8: the table showed the comparison the abundance of 24 types of immune cells between the two groups by Wilcoxon test.

Supplementary 3 Figure S1 (A–C) (A) The scatter dot plots show the relationships of expression values between the six genes we selected in the score models. (B) The correlation between clinical characteristics (sex, age, stage, and smoking) and gene expression. The scattered dots show the immune cells' score. The median, third, and first quartile values are shown in the boxplots. ^∗^*P* < 0.05; ^∗∗^*P* < 0.01; ^∗∗∗^*P* < 0.001; ^∗∗∗∗^*P* < 0.0001. (C) The volcano plot displays the differentially expressed miRNAs of the two groups.
