# Supplementary material for: Identification and Validation of a Proliferation-Associated Score Model Predicting Survival in Lung Adenocarcinomas
Source: Dis Markers. 2021 Oct 21;2021:3219594. doi: 10.1155/2021/3219594 (PMC8554523; doi:10.1155/2021/3219594)
Supplement: Supplementary 1 — Additional file 1 score: the score was used in the risk-predicted model to calculate the score of patients with LUAD. [file 3219594.f1.docx]

**Additional information: The formula for the risk predicting score by the least absolute shrinkage and selection operator (LASSO) Cox regression**

(0.07371)×PSMB6 + (0.23058)×HSPA9 + (-0.0392)×FOLR2 + (0.14739)×DUT+ (0.17565)×CDK7 + (0.26704)×PLK1
