# Supplementary material for: Identification and Validation of a Proliferation-Associated Score Model Predicting Survival in Lung Adenocarcinomas
Source: Dis Markers. 2021 Oct 21;2021:3219594. doi: 10.1155/2021/3219594 (PMC8554523; doi:10.1155/2021/3219594)
Supplement: Supplementary 2 — Table S1: the table showed genes associated with microenvironment of the 24 immune cell subsets. Table S2: the table showed the sequences of all the siRNAs and primers used in this study. Table S3: the table showed 55 genes selected for LASSO Cox regression; all the 55 genes showed the same tendency in cell proliferation (the CERES dependency score) and survival (HR). Table S4: the table showed six genes used in the model and their LASSO coefficient after LASSO Cox regression. Table S5: the table showed the summary of genomic alterations in the two groups, including the somatic mutation numbers of each gene in high and low score groups. Table S6: the table showed the differentially expressed genes (DEGs) between high score group and low score group identified by limma. Table S7: the table showed the differentially expressed miRNAs between high score group and low score group identified by limma. Table S8: the table showed the comparison the abundance of 24 types of immune cells between the two groups by Wilcoxon test. [file 3219594.f2.zip › Table S1.pdf]

Table S1. Gene signatures of the 24 immune cell subsets.

**Cell type**					
B cells	MS4A1	TCL1A	MS4A1	TCL1A	HLA-DOB
T cells	PRKCQ	CD3D	CD3G	CD28	LCK
T helper cells	ICOS	LRBA	ITM2A	FAM111A	PHF10
Tcm	CDC14A	ATM	USP9Y	PCNX	ATM
Tem	TRAL	PRKY	VIL2	GDPD5	CCR2
Th1 cells	IFNG	LTA	APBB2	DOK5	IL12RB2
Th2 cells	PMCH	AHI1	PTGIS	AHI1	CXCR6
TFH	CHI3L2	CXCL13	MYO7A	CHGB	MYO7A
Th17 cells	IL17A	IL17A	IL17RA	RORC	
Treg	FOXP3				
CD8 T cells	CD8B	CD8A	PF4	PRR5	SF1
Tgd	TRDl	TARP	C1orf61	TRGV9	CD160
Cytotoxic cells	KLRD1	KLRF1	GNLY	KLRB1	KLRD1
NK cells	LOC643313	GAGE2	ZNF747	XCL1	XCL2
K CD56dim cel	KIR3DL2	KIR3DL2	SPON2	KIR2DL3	GZMB
K CD56bright ce	DUSP4	RRAD	XCL1	PLA2G6	PLA2G6
DC	CD209	CCL17	HSD11B1	CCL13	CCL22
iDC	CD1B	VASH1	F13A1	CD1E	MMP12
aDC	CCL1	EBI3	INDO	LAMP3	OAS3
pDC	IL3RA				
Eosinophils	IL5RA	KCNH2	TKTL1	IL5RA	EMR1
Macrophages	MARCO	CXCL5	SCG5	SULT1C2	SULT1C2
Mast cells	PRG2	CTSG	TPSAB1	SLC18A2	TPSAB1
Neutrophils	CSF3R	CYP4F3	VNN3	FPRL1	KCNJ15

PNOC	KIAA0125	CD19	CR2	IGHG1	FCRL2
TRAT1	PRKCQ	BCL11B	CD2	LCK	TRBC1
NUP107	SEC24C	NAP1L4	BATF	ASF1A	FRYL
FOXP1	KLF12	ST3GAL1	INPP4B	CASP8	MLL
MEFV	C7orf54	FLI1	TBC1D5	DDX17	AKT3
APBB2	APOD	ZBTB32	CD38	CSF2	CTLA4
EVI5	AHI1	IL26	NEIL3	GSTA4	PHEX
ICA1	HEY1	CDK5R1	ST8SIA1	PDCD1	BLR1
LIME1	DNAJB1	ARHGAP8	GZMM	SLC16A7	SFRS7
TARP	FEZ1				
KLRK1	NKG7	GZMH	KLRD1	SIGIRR	ZBTB16
AF107846	SLC30A5	NM_014114	MCM3AP	TBXA2R	CDC5L
KIR3DS1	KIR3DL1	FLJ20699	TMEPAI	KIR3DL2	IL21R
NIBP	FOXJ1	DUSP4	PLA2G6	MADD	BG255923
PPFIBP2	NPR1				
FABP4	CLEC10A	SYT17	MS4A6A	CTNS	GUCA1A
KCNH2	CCR3	ACACB	THBS1	GALC	TKTL1
MSR1	CTSK	PTGDS	COLEC12	GPC4	MSR1
MS4A2	CPA3	TPSB2	TPSAB1	NM_003293	TPSAB1
MME	IL8RA	IL8RB	MME	FCGR3B	DYSF

BLK	IGHG1	COCH	OSBPL10	IGHA1	TNFRSF17
TRBC1	TRAC	ITM2A	SH2D1A	CD6	cells
FUSIP1	TRA@	TRA@	RPA1	UBE2L3	ANP32B
PCM1	RP11-74E24.2	PHC3	NFATC3	LOC202134	TIMM8A
EWSR1	TBCD	CCR2	NFATC4	LTK	
CD70	DPP4	EGFL6	BST2	DUSP5	LRP8
SMAD2	CENPF	ANK1	ADCY1	AI582773	LAIR2
KIAA1324	ICA1	TSHR	C18orf1	HEY1	TOX
APBA2	C4orf15	LEPROTL1	ZFP36L2	GADD45A	ZFP36L2
RUNX3	APOL3	RORA	APBA2	SIGIRR	WHDC1L1
LOC730096	FUT5	FGF18	MRC2	RP5-886K2.1	SPN
KIR3DL2	KIR3DL3	KIR2DS5	KIR2DS2	GTF3C1	KIR2DS1
MPPED1	MUC3B				
CARD9	CD1E	ABCG2	CD1A	PPARG	RAP1GAP
RNU2	CLC	THBS1	HIST1H1C	CYSLTR2	HRH4
PCOLCE2	CHIT1	PTGDS	KAL1	CLEC5A	GPC4
MS4A2	TPSAB1	GATA2	LOH11CR2A	SIGLEC6	ELA2
KCNJ15	FCAR	CEACAM3	FPRL1	HIST1H2BC	HPSE

ABCB4	BLNK	GLDC	MEF2C	MEF2C	IGHM
NCALD	GIMAP5	TRAL	CD3E	SKAP1	
DDX50	C13orf34	PPP2R5C	SLC25A12	ATF2	CD28
ATF7IP	REPS1	PSPC1	RPP38	HNRPH1	STX16
IL22	DGKI	CCL4	DPP4	GGT1	LRRN3
SNRPD1	CXCR6	MICAL2	DHFR	SMAD2	WDHD1
BLR1	SLC7A10	SMAD1	POMT1	PASK	MKL2
MYST3	ZEB1	ZNF609	C12orf47	THUMPD1	VAMP2
DUSP2	GZMA				
PSMD4	PRX	FZR1	ZNF205	AL080130	ZNF528
EDG8					
SLC7A8	GSTT1	NM_021941	FZD2	CSF1R	HS3ST2
RNASE2	CAT	LRP5L	SYNJ1	SYNJ1	THBS4
ME1	DNASE2B	CCL7	FN1	CD163	GM2A
LOH11CR2A	CMA1	SIGLEC6	PGDS	MLPH	ADCYAP1
FLJ11151	CREB5	S100A12	FCGR3B	TNFRSF10C	SLC22A4

FAM30A	SPIB	BCL11A	GNG7	IGKC	CD72
GOLGA8A					
CYLD	SNRPN	TRAF3IP3	NEFL	POLR2J2	AQP3
SYNGR3	ATP9A	BTG3	CMAH	HBEGF	SGCB
BIRC5	DHFR	SLC39A14	HELLS	LIMA1	CDC25C
PTPN13	PASK	KCNK5	C18orf1	ZNF764	MAF
ZNF91	ZNF22	TMC6	DNAJB1	FLT3LG	CDKN2AIP
MAPRE3	BCL2	NM_017616	ARL6IP2	SPN	FZR1
CH25H	SLC26A6	BLVRB	NUDT9	PREP	TM7SF4
GPR44	KBTBD11	HES1	ABHD2	TIPARP	SMPD3
SCARB2	BCAT1	BCAT1	RAI14	MSR1	COL8A2
SIGLEC6	SLC24A3	CALB2	SLC24A3	KIT	TAL1
KIAA0329	SLC25A37	BST1	FCAR	CEACAM3	CRISPLD2

MICAL3	BCL11A	BACH2	IGL@	CCR9	QRSL1
CG030	PDXDC2	CLUAP1	DOCK9	CYorf15B	CREBZF
CDC7<br>MYO6	GATA3<br>SIRPG	THADA	THADA	MAGEH1	B3GAT1
TSC22D3	TBCC	RBM3	ABT1	C19orf6	CAMLG
PDLIM4	NM_014274	LDB3	ADARB1	SMEK1	TCTN2
TACSTD2	CD1C				
MYO15B<br>CD163<br>ABCC4<br>TNFRSF10C	TGIF1<br>APOE<br>PPM1H<br>G0S2	ACACB<br>CHI3L1<br>MAOB<br>SIGLEC5	IGSF2<br>ATG7<br>HPGD<br>CD93	HES1<br>CD84<br>SCG2<br>MGAM	RCOR3<br>FDX1<br>PTGS1<br>ALPL

DTNB	HLA-DQA1	SCN3A	QRSL1	SLC15A2	
CEP68	TXK	SLC7A6	FYB	MAP3K1	
MAF	SH3TC1	HIST1H4K	STK39		
PPP1R2	AES	KLF9	PRF1		
TINAGL1	IGFBP5	ALDH1B1	NCR1	NCR1	NCR1
EPN2	C9orf156	SIAH1	ACACB		
MS4A4A	SGMS1	EMP1	CYBB	CD68	
CEACAM8	MPO	NR0B1	LOC339524		
FPR1	CD93	PDE4B	LILRB2		
