# Supplementary material for: Identification and Validation of a Proliferation-Associated Score Model Predicting Survival in Lung Adenocarcinomas
Source: Dis Markers. 2021 Oct 21;2021:3219594. doi: 10.1155/2021/3219594 (PMC8554523; doi:10.1155/2021/3219594)
Supplement: Supplementary 2 — Table S1: the table showed genes associated with microenvironment of the 24 immune cell subsets. Table S2: the table showed the sequences of all the siRNAs and primers used in this study. Table S3: the table showed 55 genes selected for LASSO Cox regression; all the 55 genes showed the same tendency in cell proliferation (the CERES dependency score) and survival (HR). Table S4: the table showed six genes used in the model and their LASSO coefficient after LASSO Cox regression. Table S5: the table showed the summary of genomic alterations in the two groups, including the somatic mutation numbers of each gene in high and low score groups. Table S6: the table showed the differentially expressed genes (DEGs) between high score group and low score group identified by limma. Table S7: the table showed the differentially expressed miRNAs between high score group and low score group identified by limma. Table S8: the table showed the comparison the abundance of 24 types of immune cells between the two groups by Wilcoxon test. [file 3219594.f2.zip › Table S2.docx]

**Table S2. The sequences of all the siRNAs and primers used in this study.**

| shRNA | Sequence |
| --- | --- |
| si-HSPA9_001 | 5’-GCGATATGATGATCCTGAA-3’ |
| si-HSPA9_002 | 5’-GAGTCAGATTGGAGCATTT-3’ |
| si-PSMB6_001 | 5’-GCAGTCCTTTGCCATTGGA-3’ |
| si-PSMB6_002 | 5’-CTGACAAGCTGACACCTAT-3’ |
| Primer | Sequence |
| GAPDH-F | 5’-AGAAGGCTGGGGCTCATTTG-3’ |
| GAPDH-R | 5’-AGGGGCCATCCACAGTCTTC-3’ |

**Supplementary Table 2. Results from shotgun liquid chromatography coupled with tandem mass spectrometry analyses.**

| Gene Name | Coverage | # Peptides | # PSMs | # Unique Peptides | # AAs | MW [kDa] | calc. pI | Score Mascot |
| --- | --- | --- | --- | --- | --- | --- | --- | --- |
| ZYX | 2.0979021 | 1 | 1 | 1 | 572 | 61.238 | 6.67 | 16.13 |
| VDAC1 | 2.82685512 | 1 | 1 | 1 | 283 | 30.754 | 8.54 | 20.79 |
| VIM | 10.7296137 | 6 | 6 | 5 | 466 | 53.619 | 5.12 | 180.31564 |
| MYO1C | 1.78739417 | 2 | 2 | 2 | 1063 | 121.606 | 9.41 | 49.13 |
| MYO1B | 1.84859155 | 2 | 2 | 2 | 1136 | 131.902 | 9.38 | 19.19 |
| UGDH | 4.65587045 | 2 | 2 | 2 | 494 | 54.989 | 7.12 | 70.11 |
| RPS27A | 8.33333333 | 1 | 1 | 1 | 156 | 17.953 | 9.64 | 31.99 |
| LSM4 | 5.03597122 | 1 | 1 | 1 | 139 | 15.34 | 9.99 | 33.32 |
| PRPF31 | 1.60320641 | 1 | 1 | 1 | 499 | 55.421 | 5.78 | 0 |
| TUBB4B | 2.69662921 | 1 | 1 | 1 | 445 | 49.799 | 4.89 | 0 |
| TUBA4A | 2.23214286 | 1 | 1 | 1 | 448 | 49.892 | 5.06 | 23.54 |
| TUBA1B | 2.2172949 | 1 | 1 | 1 | 451 | 50.12 | 5.06 | 30.23 |
| TPH2 | 2.04081633 | 1 | 1 | 1 | 490 | 56.021 | 6.42 | 0 |
| PRSS3 | 7.56578947 | 2 | 2 | 2 | 304 | 32.508 | 7.49 | 82.8683415 |
| TPM3 | 2.45614035 | 1 | 1 | 1 | 285 | 32.93 | 4.72 | 0 |
| HADHA | 1.57273919 | 1 | 1 | 1 | 763 | 82.947 | 9.04 | 33.41 |
| TMPRSS13 | 1.36518771 | 1 | 1 | 1 | 586 | 63.113 | 8.63 | 29.27 |
| TAF4 | 1.10599078 | 1 | 1 | 1 | 1085 | 110.047 | 9.94 | 41.27 |
| GTF2E2 | 29.209622 | 11 | 13 | 11 | 291 | 33.023 | 9.66 | 275.211529 |
| THRAP3 | 1.88481675 | 2 | 3 | 2 | 955 | 108.601 | 10.15 | 22.7 |
| TXN | 8.57142857 | 1 | 1 | 1 | 105 | 11.73 | 4.92 | 41.33 |
| CCT3 | 1.46788991 | 1 | 1 | 1 | 545 | 60.495 | 6.49 | 0 |
| CCT7 | 2.20994475 | 1 | 1 | 1 | 543 | 59.329 | 7.65 | 0 |
| TAF15 | 7.77027027 | 2 | 2 | 2 | 592 | 61.793 | 8.02 | 58.26 |
| SQOR | 3.11111111 | 1 | 1 | 1 | 450 | 49.929 | 9.11 | 24.71 |
| HSPA9 | 3.38733432 | 2 | 3 | 2 | 679 | 73.635 | 6.16 | 72.4266667 |
| SF3B2 | 1.34078212 | 1 | 1 | 1 | 895 | 100.165 | 5.67 | 31.4 |
| SPIN1 | 8.77862595 | 2 | 2 | 2 | 262 | 29.582 | 6.96 | 63.25 |
| SPTBN1 | 8.92554992 | 21 | 23 | 21 | 2364 | 274.439 | 5.57 | 411.715339 |
| SPTAN1 | 13.4708738 | 32 | 36 | 32 | 2472 | 284.364 | 5.35 | 584.305651 |
| SNRPB | 5.83333333 | 1 | 1 | 1 | 240 | 24.594 | 11.19 | 43.95 |
| SRP72 | 1.63934426 | 1 | 1 | 1 | 671 | 74.56 | 9.26 | 15.81 |
| SRP68 | 5.10366826 | 3 | 3 | 3 | 627 | 70.686 | 8.56 | 27.4666667 |
| ALB | 5.91133005 | 4 | 4 | 4 | 609 | 69.321 | 6.28 | 79.16 |
| SERPINH1 | 3.11004785 | 1 | 1 | 1 | 418 | 46.411 | 8.69 | 34.52 |
| PGAM5 | 10.7266436 | 3 | 3 | 3 | 289 | 31.985 | 8.68 | 61.48 |
| STK38 | 5.37634409 | 3 | 3 | 3 | 465 | 54.155 | 7.15 | 38.330818 |
| SRSF6 | 2.61627907 | 1 | 1 | 1 | 344 | 39.563 | 11.43 | 42.08 |
| SCYL2 | 7.42734123 | 6 | 6 | 6 | 929 | 103.642 | 8.22 | 72.5633333 |
| RALY | 5.22875817 | 2 | 2 | 2 | 306 | 32.444 | 9.17 | 0 |
| RBM10 | 1.1827957 | 1 | 1 | 1 | 930 | 103.469 | 5.97 | 16.51 |
| RBMX | 10.741688 | 4 | 4 | 4 | 391 | 42.306 | 10.05 | 88.4338405 |
| RRBP1 | 14.6808511 | 15 | 15 | 15 | 1410 | 152.365 | 8.6 | 307.169611 |
| RPF2 | 2.61437908 | 1 | 1 | 1 | 306 | 35.56 | 9.99 | 0 |
| RSL1D1 | 13.6734694 | 7 | 7 | 7 | 490 | 54.939 | 10.13 | 102.314987 |
| RACK1 | 3.15457413 | 1 | 1 | 1 | 317 | 35.055 | 7.69 | 27.49 |
| RAB12 | 4.50819672 | 1 | 1 | 1 | 244 | 27.231 | 8.41 | 13.19 |
| RAC2 | 4.16666667 | 1 | 1 | 1 | 192 | 21.415 | 7.61 | 25.01 |
| PKM | 14.6892655 | 10 | 11 | 10 | 531 | 57.9 | 7.84 | 223.813206 |
| PC | 0.84889643 | 1 | 1 | 1 | 1178 | 129.551 | 6.84 | 21.97 |
| RPS10P5 | 3.97727273 | 1 | 1 | 1 | 176 | 20.108 | 10.13 | 28.75 |
| TGM2 | 2.18340611 | 2 | 2 | 2 | 687 | 77.28 | 5.22 | 50.7559886 |
| SEC61B | 15.625 | 1 | 1 | 1 | 96 | 9.968 | 11.56 | 35.85 |
| SEC24C | 1.18829982 | 1 | 1 | 1 | 1094 | 118.249 | 7.06 | 0 |
| PPM1B | 16.0751566 | 7 | 8 | 7 | 479 | 52.609 | 5.05 | 188.12397 |
| NYNRIN | 0.63224447 | 1 | 1 | 1 | 1898 | 208.235 | 8.02 | 32.78 |
| P4HB | 15.9448819 | 9 | 11 | 9 | 508 | 57.081 | 4.87 | 213.890187 |
| PDIA4 | 3.56589147 | 2 | 2 | 2 | 645 | 72.887 | 5.07 | 61.3299148 |
| PDIA3 | 2.17821782 | 1 | 1 | 1 | 505 | 56.747 | 6.35 | 0 |
| PRMT5 | 12.244898 | 9 | 12 | 9 | 637 | 72.638 | 6.29 | 301.414369 |
| AHNAK2 | 0.1553063 | 1 | 1 | 1 | 5795 | 616.242 | 5.36 | 0 |
| PHB2 | 6.68896321 | 2 | 3 | 2 | 299 | 33.276 | 9.83 | 81.4766667 |
| PHB | 6.61764706 | 2 | 2 | 2 | 272 | 29.786 | 5.76 | 41.4895437 |
| PLOD2 | 2.71370421 | 2 | 2 | 2 | 737 | 84.632 | 6.71 | 57.8089035 |
| DDX17 | 5.21262003 | 3 | 3 | 3 | 729 | 80.222 | 8.27 | 106.946667 |
| NOP2 | 2.21674877 | 2 | 2 | 2 | 812 | 89.247 | 9.23 | 34.51 |
| DHX15 | 1.63522013 | 1 | 1 | 1 | 795 | 90.875 | 7.46 | 26.47 |
| PRPF8 | 0.47109208 | 1 | 1 | 1 | 2335 | 273.427 | 8.84 | 29.12 |
| PRPF6 | 1.27523911 | 1 | 1 | 1 | 941 | 106.858 | 8.25 | 13.96 |
| PTBP3 | 1.8115942 | 1 | 1 | 1 | 552 | 59.652 | 9.04 | 50.04 |
| GALNT5 | 1.27659574 | 1 | 1 | 1 | 940 | 106.199 | 9.47 | 17.1 |
| PABPC1 | 4.08805031 | 3 | 3 | 3 | 636 | 70.626 | 9.5 | 50.1564176 |
| PCBP3 | 2.96495957 | 1 | 1 | 1 | 371 | 39.44 | 8.07 | 58.5 |
| PARP1 | 0.98619329 | 1 | 1 | 1 | 1014 | 113.012 | 8.88 | 22.75 |
| PLEC | 20.2391119 | 110 | 119 | 110 | 4684 | 531.466 | 5.96 | 2103.31605 |
| PDGFA | 3.79146919 | 1 | 1 | 1 | 211 | 24.028 | 9.39 | 27.55 |
| SERBP1 | 5.39215686 | 2 | 2 | 2 | 408 | 44.938 | 8.65 | 68 |
| LPIN3 | 2.23266745 | 1 | 1 | 1 | 851 | 93.556 | 5.52 | 0 |
| FARSB | 1.86757216 | 1 | 1 | 1 | 589 | 66.074 | 6.84 | 33.84 |
| PRDX1 | 14.0703518 | 3 | 3 | 3 | 199 | 22.096 | 8.13 | 31.68 |
| ZDHHC2 | 1.90735695 | 1 | 1 | 1 | 367 | 41.994 | 8.35 | 29.24 |
| NPM1 | 7.4829932 | 2 | 2 | 2 | 294 | 32.555 | 4.78 | 77.62 |
| NCL | 7.18309859 | 5 | 7 | 5 | 710 | 76.568 | 4.7 | 128.042857 |
| DDX21 | 2.93742018 | 2 | 2 | 2 | 783 | 87.29 | 9.28 | 68.35 |
| YBX1 | 8.33333333 | 2 | 2 | 2 | 324 | 35.903 | 9.88 | 44.66 |
| GANAB | 1.48305085 | 2 | 2 | 2 | 944 | 106.807 | 6.14 | 48.5459109 |
| NAA25 | 0.92592593 | 1 | 1 | 1 | 972 | 112.221 | 6.64 | 18.82 |
| CMAS | 2.30414747 | 1 | 1 | 1 | 434 | 48.349 | 7.93 | 24.07 |
| MYH9 | 6.2755102 | 14 | 14 | 14 | 1960 | 226.392 | 5.6 | 147.370464 |
| MYL6 | 14.5695364 | 2 | 2 | 2 | 151 | 16.919 | 4.65 | 80.28 |
| MYBBP1A | 0.67771084 | 1 | 1 | 1 | 1328 | 148.762 | 9.28 | 33.02 |
| MBNL1 | 2.83505155 | 1 | 1 | 1 | 388 | 41.79 | 8.9 | 15.83 |
| MOB2 | 14.7679325 | 4 | 4 | 4 | 237 | 26.909 | 6.79 | 29.7451635 |
| SLC25A11 | 5.0955414 | 1 | 1 | 1 | 314 | 34.04 | 9.91 | 61.92 |
| MAP1B | 1.29659643 | 3 | 4 | 3 | 2468 | 270.468 | 4.81 | 44.07 |
| CLNS1A | 5.06329114 | 1 | 1 | 1 | 237 | 26.199 | 4.11 | 40.52 |
| WDR77 | 13.7426901 | 5 | 7 | 5 | 342 | 36.701 | 5.17 | 150.735714 |
| MARS | 1.11111111 | 1 | 1 | 1 | 900 | 101.052 | 6.16 | 0 |
| MTA2 | 1.49700599 | 1 | 1 | 1 | 668 | 74.976 | 9.66 | 26.22 |
| MATR3 | 4.95867769 | 4 | 5 | 4 | 847 | 94.565 | 6.25 | 73.1307621 |
| LDHB | 10.7784431 | 3 | 3 | 2 | 334 | 36.615 | 6.05 | 121.926397 |
| LDHA | 15.060241 | 5 | 5 | 4 | 332 | 36.665 | 8.27 | 195.446235 |
| LASP1 | 4.59770115 | 1 | 1 | 1 | 261 | 29.698 | 7.05 | 26.1 |
| LRRC59 | 5.86319218 | 2 | 2 | 2 | 307 | 34.909 | 9.57 | 35.15305 |
| LRRC43 | 1.52439024 | 1 | 2 | 1 | 656 | 72.976 | 5.24 | 24.89 |
| ILF3 | 4.58612975 | 4 | 4 | 4 | 894 | 95.279 | 8.76 | 41.7633333 |
| IRF8 | 1.87793427 | 1 | 1 | 1 | 426 | 48.325 | 6.77 | 0 |
| ITGB1 | 1.25313283 | 1 | 1 | 1 | 798 | 88.357 | 5.39 | 0 |
| IGF2BP3 | 1.72711572 | 1 | 1 | 1 | 579 | 63.666 | 8.87 | 28.76 |
| IGF2BP1 | 2.25303293 | 1 | 1 | 1 | 577 | 63.441 | 9.2 | 22.26 |
| IGKV2-40 | 10.7438017 | 1 | 2 | 1 | 121 | 13.302 | 4.61 | 74.42 |
| HIF1A | 6.17433414 | 1 | 1 | 1 | 826 | 92.612 | 5.33 | 0 |
| HYOU1 | 1.2012012 | 1 | 1 | 1 | 999 | 111.266 | 5.22 | 17.5 |
| HRNR | 1.92982456 | 1 | 1 | 1 | 2850 | 282.228 | 10.04 | 77.04 |
| ASH1L | 0.43785786 | 1 | 1 | 1 | 2969 | 332.582 | 9.39 | 0 |
| HIST1H4A | 38.8349515 | 4 | 6 | 4 | 103 | 11.36 | 11.36 | 115.963333 |
| HIST1H3A | 11.7647059 | 2 | 2 | 2 | 136 | 15.394 | 11.12 | 57.1506639 |
| HIST1H2BK | 41.2698413 | 7 | 8 | 7 | 126 | 13.882 | 10.32 | 184.367666 |
| HIST1H2AA | 10.6870229 | 2 | 2 | 2 | 131 | 14.225 | 10.86 | 50.7823055 |
| H1FX | 17.370892 | 3 | 3 | 3 | 213 | 22.474 | 10.76 | 101.081034 |
| HIST1H1B | 7.52212389 | 2 | 2 | 1 | 226 | 22.566 | 10.92 | 56.23 |
| HIST1H1E | 33.7899543 | 8 | 10 | 2 | 219 | 21.852 | 11.03 | 316.54549 |
| HIST1H1D | 25.3393665 | 7 | 9 | 1 | 221 | 22.336 | 11.02 | 299.431033 |
| HIST1H1C | 36.6197183 | 9 | 11 | 3 | 213 | 21.352 | 10.93 | 307.38 |
| H1F0 | 6.70103093 | 1 | 1 | 1 | 194 | 20.85 | 10.84 | 47.4 |
| HNRNPC | 14.0522876 | 5 | 6 | 5 | 306 | 33.65 | 5.08 | 167.91336 |
| HNRNPA2B1 | 2.83286119 | 1 | 1 | 1 | 353 | 37.407 | 8.95 | 17.81 |
| HNRNPUL1 | 1.1682243 | 1 | 1 | 1 | 856 | 95.679 | 6.92 | 13.49 |
| HNRNPU | 9.6969697 | 11 | 12 | 11 | 825 | 90.528 | 6 | 145.419925 |
| HNRNPR | 1.8957346 | 1 | 1 | 1 | 633 | 70.899 | 8.13 | 52.81 |
| HNRNPM | 7.26027397 | 5 | 6 | 5 | 730 | 77.464 | 8.7 | 117.392704 |
| HNRNPL | 3.39558574 | 2 | 2 | 2 | 589 | 64.092 | 8.22 | 25.3887875 |
| HNRNPK | 16.8466523 | 8 | 9 | 8 | 463 | 50.944 | 5.54 | 151.505252 |
| HNRNPF | 6.26506024 | 2 | 3 | 2 | 415 | 45.643 | 5.58 | 69.5033333 |
| HNRNPDL | 2.38095238 | 1 | 1 | 1 | 420 | 46.409 | 9.57 | 21.88 |
| HP1BP3 | 5.78661844 | 3 | 3 | 3 | 553 | 61.169 | 9.67 | 63.446477 |
| HBB | 8.84353741 | 1 | 1 | 1 | 147 | 15.988 | 7.28 | 14.32 |
| HSP90AB1 | 9.1160221 | 6 | 6 | 2 | 724 | 83.212 | 5.03 | 147.81 |
| HSP90AA1 | 7.65027322 | 5 | 5 | 2 | 732 | 84.607 | 5.02 | 99.27 |
| HSPB1 | 4.87804878 | 1 | 1 | 1 | 205 | 22.768 | 6.4 | 45.1 |
| HSPA8 | 9.44272446 | 5 | 5 | 2 | 646 | 70.854 | 5.52 | 109.451733 |
| HSPA6 | 10.1088647 | 6 | 6 | 1 | 643 | 70.984 | 6.14 | 117.83271 |
| HSPA1B | 10.1404056 | 6 | 6 | 1 | 641 | 70.009 | 5.66 | 118.13271 |
| GNB1 | 2.94117647 | 1 | 1 | 1 | 340 | 37.353 | 6 | 21.15 |
| DIRAS2 | 5.52763819 | 1 | 1 | 1 | 199 | 22.471 | 8.76 | 33.25 |
| RAN | 18.5185185 | 4 | 4 | 4 | 216 | 24.408 | 7.49 | 33.426368 |
| GAPDH | 14.9253731 | 6 | 6 | 6 | 335 | 36.03 | 8.46 | 149.754457 |
| GSTO1 | 9.95850622 | 2 | 2 | 2 | 241 | 27.548 | 6.6 | 34.4 |
| G6PD | 6.60194175 | 4 | 5 | 4 | 515 | 59.219 | 6.84 | 64.8096265 |
| GTF2E1 | 12.7562642 | 5 | 6 | 5 | 439 | 49.421 | 4.82 | 62.7482003 |
| PTK2 | 0.66539924 | 1 | 1 | 1 | 1052 | 119.157 | 6.62 | 23.27 |
| FLNB | 2.4980784 | 7 | 7 | 4 | 2602 | 277.99 | 5.73 | 90.7674556 |
| FLNA | 17.0003778 | 40 | 48 | 37 | 2647 | 280.564 | 6.06 | 1006.68578 |
| FTH1 | 3.82513661 | 1 | 1 | 1 | 183 | 21.212 | 5.55 | 43.47 |
| FABP3 | 6.76691729 | 1 | 1 | 1 | 133 | 14.849 | 6.8 | 31.66 |
| FASN | 0.35842294 | 1 | 1 | 1 | 2511 | 273.254 | 6.44 | 35.83 |
| FAN1 | 2.06489676 | 1 | 1 | 1 | 1017 | 114.152 | 7.34 | 0 |
| EIF4B | 33.7152209 | 21 | 26 | 21 | 611 | 69.11 | 5.73 | 516.982169 |
| EIF2S2 | 2.7027027 | 1 | 1 | 1 | 333 | 38.364 | 5.8 | 31.28 |
| EIF2S1 | 3.80952381 | 1 | 1 | 1 | 315 | 36.089 | 5.08 | 30.69 |
| EIF4A3 | 3.89294404 | 2 | 2 | 2 | 411 | 46.841 | 6.73 | 0 |
| MAP7 | 4.4058745 | 3 | 3 | 3 | 749 | 84.002 | 9.61 | 59.5995241 |
| ECE1 | 0.90909091 | 1 | 1 | 1 | 770 | 87.108 | 5.88 | 32.4 |
| HSP90B1 | 10.0871731 | 8 | 8 | 7 | 803 | 92.411 | 4.84 | 220.014286 |
| HSPA5 | 21.1009174 | 13 | 17 | 12 | 654 | 72.288 | 5.16 | 280.439588 |
| TUFM | 6.4159292 | 3 | 3 | 3 | 452 | 49.51 | 7.61 | 84.9703261 |
| EEF1G | 2.97482838 | 1 | 1 | 1 | 437 | 50.087 | 6.67 | 33.85 |
| EEF1A1 | 11.2554113 | 6 | 6 | 6 | 462 | 50.109 | 9.01 | 148.592609 |
| EFCAB5 | 0.66533599 | 1 | 1 | 1 | 1503 | 173.295 | 5.82 | 0 |
| RPN1 | 1.8121911 | 1 | 1 | 1 | 607 | 68.527 | 6.38 | 0 |
| PRKDC | 0.7751938 | 3 | 3 | 3 | 4128 | 468.788 | 7.12 | 35.2218645 |
| DAB2IP | 1.34566863 | 1 | 1 | 1 | 1189 | 131.543 | 8.72 | 0 |
| FMO3 | 3.57142857 | 1 | 1 | 1 | 532 | 59.994 | 7.78 | 0 |
| DSTN | 7.27272727 | 1 | 1 | 1 | 165 | 18.493 | 7.85 | 52.8 |
| DCD | 10 | 1 | 1 | 1 | 110 | 11.277 | 6.54 | 50.85 |
| ACO1 | 0.89988751 | 1 | 1 | 1 | 889 | 98.337 | 6.68 | 0 |
| UQCRC1 | 2.5 | 1 | 1 | 1 | 480 | 52.612 | 6.37 | 22.35 |
| COPRS | 6.52173913 | 1 | 1 | 1 | 184 | 20.054 | 4.18 | 18.38 |
| FAM120A | 0.80500894 | 1 | 1 | 1 | 1118 | 121.811 | 8.88 | 18.32 |
| C9 | 1.61001789 | 1 | 1 | 1 | 559 | 63.133 | 5.59 | 29.54 |
| C3 | 2.46542393 | 4 | 4 | 4 | 1663 | 187.03 | 6.4 | 61.7450499 |
| CCDC86 | 2.22222222 | 1 | 1 | 1 | 360 | 40.211 | 10.33 | 0 |
| CFL1 | 6.62650602 | 1 | 1 | 1 | 166 | 18.491 | 8.09 | 27.07 |
| COPA | 0.81699346 | 1 | 1 | 1 | 1224 | 138.258 | 7.66 | 23.35 |
| CLTC | 2.80597015 | 5 | 5 | 5 | 1675 | 191.493 | 5.69 | 94.560111 |
| CLIC1 | 4.97925311 | 1 | 1 | 1 | 241 | 26.906 | 5.17 | 21.23 |
| CAV1 | 4.49438202 | 1 | 1 | 1 | 178 | 20.458 | 6.02 | 17.07 |
| CAVIN1 | 2.82051282 | 1 | 1 | 1 | 390 | 43.45 | 5.6 | 0 |
| CMBL | 6.93877551 | 2 | 2 | 2 | 245 | 28.03 | 7.18 | 26.8 |
| CALR | 2.15827338 | 1 | 1 | 1 | 417 | 48.112 | 4.44 | 32.48 |
| CANX | 4.39189189 | 3 | 3 | 3 | 592 | 67.526 | 4.6 | 65.9266667 |
| SLC25A12 | 1.179941 | 1 | 1 | 1 | 678 | 74.715 | 8.38 | 34.7 |
| EPRS | 0.5952381 | 1 | 1 | 1 | 1512 | 170.483 | 7.33 | 29.22 |
| DDX24 | 2.67753201 | 2 | 2 | 2 | 859 | 96.271 | 9.06 | 19.22 |
| DHX9 | 2.28346457 | 3 | 4 | 3 | 1270 | 140.869 | 6.84 | 76.2039081 |
| CLPX | 2.05371248 | 1 | 1 | 1 | 633 | 69.181 | 7.58 | 36.62 |
| ACLY | 4.81380563 | 6 | 6 | 6 | 1101 | 120.762 | 7.33 | 100.783141 |
| ATP5O | 5.16431925 | 1 | 1 | 1 | 213 | 23.263 | 9.96 | 48.99 |
| ATP5F1C | 3.3557047 | 1 | 1 | 1 | 298 | 32.975 | 9.22 | 45.31 |
| ATP5F1D | 4.16666667 | 1 | 1 | 1 | 168 | 17.479 | 5.49 | 31.65 |
| ATP5F1B | 2.07939509 | 1 | 1 | 1 | 529 | 56.525 | 5.4 | 21.64 |
| ASPH | 3.03430079 | 2 | 2 | 2 | 758 | 85.809 | 5.01 | 56.3689035 |
| ANXA4 | 3.44827586 | 1 | 1 | 1 | 319 | 35.86 | 6.13 | 28.46 |
| ANXA2 | 13.8643068 | 5 | 5 | 5 | 339 | 38.58 | 7.75 | 147.027366 |
| ANXA1 | 3.17919075 | 1 | 1 | 1 | 346 | 38.69 | 7.02 | 60.51 |
| ANKHD1 | 0.27537372 | 1 | 1 | 1 | 2542 | 269.291 | 5.73 | 54.43 |
| ENO1 | 3.91705069 | 2 | 2 | 2 | 434 | 47.139 | 7.39 | 24.56 |
| AKR1B1 | 5.06329114 | 2 | 2 | 2 | 316 | 35.83 | 6.98 | 38.1340687 |
| AKR1C3 | 2.47678019 | 1 | 1 | 1 | 323 | 36.83 | 7.94 | 38.96 |
| ALDH3A1 | 2.64900662 | 1 | 1 | 1 | 453 | 50.363 | 6.54 | 47.67 |
| ARF4 | 5.55555556 | 1 | 1 | 1 | 180 | 20.498 | 7.14 | 0 |
| SLC25A6 | 8.38926174 | 3 | 3 | 1 | 298 | 32.845 | 9.74 | 53.1140666 |
| SLC25A5 | 8.38926174 | 3 | 3 | 1 | 298 | 32.831 | 9.69 | 86.0774 |
| SUB1 | 8.66141732 | 1 | 1 | 1 | 127 | 14.386 | 9.6 | 25.01 |
| ARPC1B | 3.49462366 | 1 | 1 | 1 | 372 | 40.923 | 8.35 | 32.15 |
| ABLIM1 | 1.79948586 | 1 | 1 | 1 | 778 | 87.631 | 8.59 | 26.98 |
| ACTB | 23.4666667 | 11 | 14 | 11 | 375 | 41.71 | 5.48 | 320.714624 |
| PGD | 2.27743271 | 1 | 1 | 1 | 483 | 53.106 | 7.23 | 47.2 |
| PFKFB3 | 3.84615385 | 2 | 2 | 2 | 520 | 59.571 | 8.21 | 72.7232204 |
| RPL8 | 20.233463 | 7 | 8 | 7 | 257 | 28.007 | 11.03 | 145.159378 |
| RPL7A | 21.8045113 | 6 | 6 | 6 | 266 | 29.977 | 10.61 | 70.2530877 |
| RPL7 | 19.7580645 | 4 | 5 | 4 | 248 | 29.207 | 10.65 | 55.7333333 |
| RPL6 | 21.1805556 | 8 | 9 | 8 | 288 | 32.708 | 10.58 | 111.187814 |
| RPL5 | 6.3973064 | 2 | 2 | 2 | 297 | 34.341 | 9.72 | 23.59 |
| RPL4 | 6.32318501 | 3 | 3 | 3 | 427 | 47.667 | 11.06 | 52.2078473 |
| RPL37A | 18.4782609 | 2 | 2 | 2 | 92 | 10.268 | 10.43 | 32.14 |
| RPL36A | 17.9245283 | 2 | 2 | 2 | 106 | 12.433 | 10.58 | 36.99 |
| RPL36 | 9.52380952 | 1 | 1 | 1 | 105 | 12.246 | 11.59 | 16.85 |
| RPL35A | 26.3636364 | 3 | 3 | 3 | 110 | 12.53 | 11.06 | 36.73 |
| RPL35 | 22.7642276 | 3 | 4 | 3 | 123 | 14.543 | 11.05 | 38.88 |
| RPL34 | 16.2393162 | 2 | 2 | 2 | 117 | 13.284 | 11.47 | 36.89 |
| RPL31 | 14.4 | 2 | 2 | 2 | 125 | 14.454 | 10.54 | 59.74 |
| RPL30 | 7.82608696 | 1 | 1 | 1 | 115 | 12.776 | 9.63 | 21.36 |
| RPL3 | 7.44416873 | 4 | 4 | 4 | 403 | 46.08 | 10.18 | 69.2683918 |
| RPL29 | 9.43396226 | 1 | 1 | 1 | 159 | 17.741 | 11.66 | 29.3 |
| RPL28 | 16.7883212 | 3 | 4 | 3 | 137 | 15.738 | 12.02 | 88.2665961 |
| RPL27A | 13.5135135 | 2 | 2 | 2 | 148 | 16.551 | 11 | 38.92 |
| RPL27 | 12.5 | 2 | 2 | 2 | 136 | 15.788 | 10.56 | 23.89 |
| RPL26 | 11.0344828 | 2 | 2 | 2 | 145 | 17.248 | 10.55 | 41.48 |
| RPL24 | 10.1910828 | 2 | 2 | 2 | 157 | 17.768 | 11.25 | 27.55 |
| RPL23A | 21.7948718 | 5 | 6 | 5 | 156 | 17.684 | 10.45 | 101.88704 |
| RPL23 | 12.8571429 | 2 | 2 | 2 | 140 | 14.856 | 10.51 | 38.95 |
| RPL18A | 17.6136364 | 3 | 4 | 3 | 176 | 20.749 | 10.71 | 71.3337416 |
| RPL18 | 5.85106383 | 1 | 1 | 1 | 188 | 21.621 | 11.72 | 27.35 |
| RPL17 | 21.7391304 | 4 | 4 | 4 | 184 | 21.383 | 10.17 | 61.33 |
| RPL15 | 7.84313725 | 2 | 2 | 2 | 204 | 24.131 | 11.62 | 59.3119804 |
| RPL14 | 3.25581395 | 1 | 1 | 1 | 215 | 23.417 | 10.93 | 27.5 |
| RPL13 | 13.7440758 | 4 | 5 | 4 | 211 | 24.247 | 11.65 | 92.3066898 |
| RPL12 | 5.45454545 | 1 | 1 | 1 | 165 | 17.808 | 9.42 | 30.75 |
| RPL10A | 11.5207373 | 3 | 3 | 3 | 217 | 24.816 | 9.94 | 46.6389438 |
| HSPD1 | 1.57068063 | 1 | 1 | 1 | 573 | 61.016 | 5.87 | 52.57 |
| RPSA | 4.40677966 | 1 | 1 | 1 | 295 | 32.833 | 4.87 | 44.85 |
| RPS9 | 22.6804124 | 6 | 6 | 6 | 194 | 22.578 | 10.65 | 59.8604923 |
| RPS8 | 5.76923077 | 2 | 2 | 2 | 208 | 24.19 | 10.32 | 23.94 |
| RPS7 | 4.63917526 | 1 | 1 | 1 | 194 | 22.113 | 10.1 | 14.45 |
| RPS6 | 12.8514056 | 3 | 3 | 3 | 249 | 28.663 | 10.84 | 62.66 |
| RPS4X | 10.2661597 | 3 | 3 | 3 | 263 | 29.579 | 10.15 | 78.3877416 |
| RPS3A | 28.030303 | 8 | 11 | 8 | 264 | 29.926 | 9.73 | 203.695897 |
| RPS3 | 5.34979424 | 1 | 1 | 1 | 243 | 26.671 | 9.66 | 33.02 |
| RPS27 | 9.52380952 | 1 | 1 | 1 | 84 | 9.455 | 9.45 | 30.49 |
| RPS26 | 7.82608696 | 1 | 1 | 1 | 115 | 13.007 | 11 | 34.08 |
| RPS25 | 28.8 | 4 | 4 | 4 | 125 | 13.734 | 10.11 | 123.683725 |
| RPS24 | 5.26315789 | 1 | 1 | 1 | 133 | 15.413 | 10.78 | 27.47 |
| RPS23 | 13.2867133 | 2 | 3 | 2 | 143 | 15.798 | 10.49 | 40.9516714 |
| RPS20 | 9.24369748 | 1 | 1 | 1 | 119 | 13.364 | 9.94 | 27.37 |
| RPS2 | 12.2866894 | 4 | 4 | 4 | 293 | 31.305 | 10.24 | 81.86 |
| RPS19 | 11.7241379 | 2 | 2 | 2 | 145 | 16.051 | 10.32 | 44.5 |
| RPS18 | 26.9736842 | 4 | 4 | 4 | 152 | 17.708 | 10.99 | 119.06 |
| RPS17 | 5.92592593 | 1 | 1 | 1 | 135 | 15.54 | 9.85 | 0 |
| RPS16 | 11.6438356 | 2 | 2 | 2 | 146 | 16.435 | 10.21 | 49.94 |
| RPS15A | 6.92307692 | 1 | 1 | 1 | 130 | 14.83 | 10.13 | 0 |
| RPS14 | 17.8807947 | 4 | 5 | 4 | 151 | 16.263 | 10.05 | 124.510976 |
| RPS13 | 12.5827815 | 2 | 2 | 2 | 151 | 17.212 | 10.54 | 58.5846078 |
| RPS11 | 17.0886076 | 3 | 4 | 3 | 158 | 18.419 | 10.3 | 50.9396001 |
| PSMC1 | 2.72727273 | 1 | 1 | 1 | 440 | 49.154 | 6.21 | 30.27 |
| PSMD3 | 2.05992509 | 1 | 1 | 1 | 534 | 60.939 | 8.44 | 40.3 |
| YWHAZ | 5.71428571 | 1 | 1 | 1 | 245 | 27.728 | 4.79 | 37.02 |
| YWHAQ | 5.71428571 | 1 | 1 | 1 | 245 | 27.747 | 4.78 | 33.85 |
| YWHAG | 3.6437247 | 1 | 1 | 1 | 247 | 28.285 | 4.89 | 0 |
