# Supplementary material for: Identification and Validation of a Proliferation-Associated Score Model Predicting Survival in Lung Adenocarcinomas
Source: Dis Markers. 2021 Oct 21;2021:3219594. doi: 10.1155/2021/3219594 (PMC8554523; doi:10.1155/2021/3219594)
Supplement: Supplementary 2 — Table S1: the table showed genes associated with microenvironment of the 24 immune cell subsets. Table S2: the table showed the sequences of all the siRNAs and primers used in this study. Table S3: the table showed 55 genes selected for LASSO Cox regression; all the 55 genes showed the same tendency in cell proliferation (the CERES dependency score) and survival (HR). Table S4: the table showed six genes used in the model and their LASSO coefficient after LASSO Cox regression. Table S5: the table showed the summary of genomic alterations in the two groups, including the somatic mutation numbers of each gene in high and low score groups. Table S6: the table showed the differentially expressed genes (DEGs) between high score group and low score group identified by limma. Table S7: the table showed the differentially expressed miRNAs between high score group and low score group identified by limma. Table S8: the table showed the comparison the abundance of 24 types of immune cells between the two groups by Wilcoxon test. [file 3219594.f2.zip › Table S3.pdf]

Table S3. Genes selected for Lasso Cox regression

Gene	Score	HR	P value
CDK1	-1.75992	1.851996	7.04E-05
DUT	-1.55221	1.747823	0.00027
RRM2	-1.77004	1.746326	0.000315
PLK1	-1.61773	1.705978	0.000478
ESPL1	-1.7687	1.689922	0.000599
FOLR2	0.264757	0.598964	0.000801
DONSON	-1.81252	1.663926	0.000884
DTYMK	-1.51194	1.659575	0.000999
NEDD1	-1.70409	1.66088	0.001001
MASTL	-1.66445	1.642753	0.001123
CENPW	-1.54079	1.633064	0.001497
SMC2	-1.56372	1.624149	0.001497
PHF5A	-1.78741	1.613414	0.001949
KIF11	-1.87955	1.601161	0.001976
CDC45	-1.79696	1.577992	0.002835
CHEK1	-1.50449	1.573666	0.003015
BUB3	-2.0575	1.556191	0.003846
SPC24	-1.7106	1.549357	0.004058
NCAPG	-1.62548	1.545276	0.004342
PSMA2	-1.5668	1.544468	0.004684
RFC2	-1.58293	1.539193	0.004908
FARSB	-1.86151	1.52013	0.006077
RRM1	-1.69195	1.511627	0.006343
SNRPA1	-1.6456	1.506129	0.007157
CDK7	-1.71719	1.511777	0.00733
DHX37	-1.68836	1.488164	0.008683
GIMAP4	0.367085	0.674173	0.009634
CHAF1B	-1.9005	1.484469	0.009639
PSMA5	-1.63432	1.482026	0.010311
TOP2A	-1.71317	1.471089	0.010702
DTL	-1.53441	1.472592	0.01073
LRRC27	0.29717	0.680345	0.011666
GINS1	-1.5261	1.465595	0.011853
POLE2	-1.57886	1.453735	0.013513
CCT4	-1.54658	1.455769	0.013626
KPNB1	-1.88112	1.425256	0.018898
ARHGAP4[illegible]	0.305112	0.70053	0.019091
PSMB6	-1.82164	1.43403	0.019692
RPL4	-1.85984	1.421648	0.020244
RUVBL1	-1.75478	1.42323	0.020551
SCGB3A1	0.297507	0.705024	0.021
POLR3B	-1.5324	1.409124	0.024133
NIP7	-1.75072	1.408685	0.024488
RPS11	-1.80472	1.398179	0.027578
GINS2	-1.89549	1.391948	0.029597
CDC23	-1.55075	1.390428	0.029787
CDCA8	-1.78417	1.386688	0.031099
HSPA9	-1.52471	1.382462	0.032156
POLD3	-1.60333	1.379951	0.03361
SNRPD1	-1.93936	1.380578	0.033722
CDC20	-1.548	1.377215	0.035111
SNRPB	-1.77452	1.371921	0.036829
NCBP2	-1.71304	1.360571	0.041734
TCP1	-1.50407	1.357444	0.04355
PCNA	-1.82392	1.35264	0.04639
