# Supplementary material for: Identification and Validation of a Proliferation-Associated Score Model Predicting Survival in Lung Adenocarcinomas
Source: Dis Markers. 2021 Oct 21;2021:3219594. doi: 10.1155/2021/3219594 (PMC8554523; doi:10.1155/2021/3219594)
Supplement: Supplementary 2 — Table S1: the table showed genes associated with microenvironment of the 24 immune cell subsets. Table S2: the table showed the sequences of all the siRNAs and primers used in this study. Table S3: the table showed 55 genes selected for LASSO Cox regression; all the 55 genes showed the same tendency in cell proliferation (the CERES dependency score) and survival (HR). Table S4: the table showed six genes used in the model and their LASSO coefficient after LASSO Cox regression. Table S5: the table showed the summary of genomic alterations in the two groups, including the somatic mutation numbers of each gene in high and low score groups. Table S6: the table showed the differentially expressed genes (DEGs) between high score group and low score group identified by limma. Table S7: the table showed the differentially expressed miRNAs between high score group and low score group identified by limma. Table S8: the table showed the comparison the abundance of 24 types of immune cells between the two groups by Wilcoxon test. [file 3219594.f2.zip › Table S4.pdf]

Table S4. LASSO coefficient

Gene	LASSO coefficient
PSMB6	0.002818802
HSPA9	0.131352781
FOLR2	-0.000254612
DUT	0.074883636
CDK7	0.016910962
PLK1	0.187494011
