# Supplementary material for: Identification and Validation of a Proliferation-Associated Score Model Predicting Survival in Lung Adenocarcinomas
Source: Dis Markers. 2021 Oct 21;2021:3219594. doi: 10.1155/2021/3219594 (PMC8554523; doi:10.1155/2021/3219594)
Supplement: Supplementary 2 — Table S1: the table showed genes associated with microenvironment of the 24 immune cell subsets. Table S2: the table showed the sequences of all the siRNAs and primers used in this study. Table S3: the table showed 55 genes selected for LASSO Cox regression; all the 55 genes showed the same tendency in cell proliferation (the CERES dependency score) and survival (HR). Table S4: the table showed six genes used in the model and their LASSO coefficient after LASSO Cox regression. Table S5: the table showed the summary of genomic alterations in the two groups, including the somatic mutation numbers of each gene in high and low score groups. Table S6: the table showed the differentially expressed genes (DEGs) between high score group and low score group identified by limma. Table S7: the table showed the differentially expressed miRNAs between high score group and low score group identified by limma. Table S8: the table showed the comparison the abundance of 24 types of immune cells between the two groups by Wilcoxon test. [file 3219594.f2.zip › Table S5.pdf]

Table S5. Summary of genomic alterations in the two groups.

Genes	Low	High	Results.pval	Results.or	Results.ci.up	Results.ci.low	Results.adjPval
SMARCA4	11	31	7.16E-08	0.159263723	0.336792119	0.070095454	0.000233991
TP53	126	110	8.85E-08	0.351426495	0.524924907	0.233638279	0.000233991
KEAP1	38	50	3.28E-06	0.321966796	0.529486016	0.194376509	0.005773055
SCN1A	6	20	7.16E-06	0.143008039	0.378901343	0.046024321	0.009457843
WDR78	2	14	1.40E-05	0.06999147	0.310572949	0.007617469	0.0147494
TTN	124	99	3.96E-05	0.449890428	0.667416928	0.302016289	0.034895276
UBTFL1	1	11	5.64E-05	0.045274852	0.316479275	0.001047936	0.042575716
MTCL1	5	16	8.43E-05	0.152432039	0.445709652	0.042868776	0.055706849
DIDO1	11	22	0.00010946	0.238692043	0.529498827	0.101672428	0.064289697
MCM10	1	10	0.000155971	0.050109924	0.357676374	0.001150027	0.080177489
GRIA4	4	14	0.000172091	0.140747431	0.457849978	0.033178469	0.080177489
SUV420H1	0	8	0.000182015	0	0.300090926	0	0.080177489
CABS1	2	11	0.000255561	0.090746138	0.423223775	0.009661043	0.103915164
HERC2	24	32	0.000280802	0.348135808	0.635625635	0.188430045	0.106022747
DYNC2H1	9	19	0.000303297	0.229265659	0.54677793	0.089136083	0.106028854
ROBO4	3	12	0.000342415	0.124362707	0.469811753	0.022184001	0.106028854
PPARGC1E	1	9	0.000426456	0.0560184	0.410299008	0.001272569	0.106028854
UHRF1BP1	1	9	0.000426456	0.0560184	0.410299008	0.001272569	0.106028854
PCDHB7	12	21	0.000491552	0.275447187	0.604356021	0.12009704	0.106028854
ACSM2B	7	16	0.000534741	0.214616178	0.565316561	0.073069676	0.106028854
NOS1	7	16	0.000534741	0.214616178	0.565316561	0.073069676	0.106028854
RBP3	7	16	0.000534741	0.214616178	0.565316561	0.073069676	0.106028854
CHN1	0	7	0.000541578	0	0.357352537	0	0.106028854
GMPPA	0	7	0.000541578	0	0.357352537	0	0.106028854
KRT7	0	7	0.000541578	0	0.357352537	0	0.106028854
MLLT6	0	7	0.000541578	0	0.357352537	0	0.106028854
STAT1	0	7	0.000541578	0	0.357352537	0	0.106028854
COL6A6	11	20	0.000630822	0.266062304	0.599909307	0.112071326	0.115221046
CENPF	13	22	0.000649486	0.283844687	0.607738338	0.127544058	0.115221046
MRC2	2	10	0.000653922	0.100457934	0.479473132	0.010579978	0.115221046
SNTG2	5	14	0.000717725	0.17642176	0.530041318	0.048835159	0.120915821
PIK3CA	8	17	0.000759597	0.230044983	0.577348158	0.083971495	0.120915821
ST18	8	17	0.000759597	0.230044983	0.577348158	0.083971495	0.120915821
RYR2	98	78	0.000777741	0.516743441	0.772261522	0.345404081	0.120915821
DNAH8	25	31	0.000881217	0.378247162	0.68972724	0.205607063	0.133088914
OR6N1	4	12	0.000954612	0.166260168	0.559853327	0.038461708	0.13638048
RPE65	4	12	0.000954612	0.166260168	0.559853327	0.038461708	0.13638048
BPIFB2	1	8	0.001150754	0.063402269	0.479676741	0.001422388	0.140605696
LATS2	1	8	0.001150754	0.063402269	0.479676741	0.001422388	0.140605696
ROR1	1	8	0.001150754	0.063402269	0.479676741	0.001422388	0.140605696
SLAMF8	1	8	0.001150754	0.063402269	0.479676741	0.001422388	0.140605696
ZFP14	1	8	0.001150754	0.063402269	0.479676741	0.001422388	0.140605696
DZIP3	9	17	0.001158715	0.25956638	0.63236282	0.099513229	0.140605696
SORCS1	27	32	0.001170384	0.395515889	0.710917368	0.21858004	0.140605696
PCNXL2	6	14	0.001223541	0.212294558	0.601855091	0.065556534	0.141393356
ARHGAP6	5	13	0.001435769	0.191181238	0.584032318	0.052413956	0.141393356
COL22A1	22	28	0.001452821	0.372722063	0.70199264	0.195702709	0.141393356
MYH6	14	21	0.001540975	0.323323622	0.688029182	0.147565875	0.141393356
C7orf34	0	6	0.001604919	0	0.439777501	0	0.141393356
DET1	0	6	0.001604919	0	0.439777501	0	0.141393356
EMILIN1	0	6	0.001604919	0	0.439777501	0	0.141393356
ERLIN2	0	6	0.001604919	0	0.439777501	0	0.141393356
IDE	0	6	0.001604919	0	0.439777501	0	0.141393356
KNSTRN	0	6	0.001604919	0	0.439777501	0	0.141393356
NMNAT2	0	6	0.001604919	0	0.439777501	0	0.141393356

OR51A2	0	6	0.001604919	0	0.439777501	0	0.141393356
PIP5K1B	0	6	0.001604919	0	0.439777501	0	0.141393356
THNSL1	0	6	0.001604919	0	0.439777501	0	0.141393356
USO1	0	6	0.001604919	0	0.439777501	0	0.141393356
ZNF721	0	6	0.001604919	0	0.439777501	0	0.141393356
TSKS	2	9	0.001643638	0.112303281	0.551535316	0.011657249	0.142430644
CSMD3	104	80	0.001681076	0.537392718	0.800680465	0.360273978	0.143033951
RGPD4	20	26	0.001704718	0.367602992	0.710221138	0.187689316	0.143033951
CRB1	23	28	0.001759717	0.390923161	0.731638958	0.206987342	0.145341642
ERICH3	35	37	0.001866951	0.439157395	0.751620423	0.255985888	0.147647955
GTDC1	3	10	0.001981791	0.151073927	0.597477938	0.026353782	0.147647955
KDM6B	3	10	0.001981791	0.151073927	0.597477938	0.026353782	0.147647955
MAGEB10	3	10	0.001981791	0.151073927	0.597477938	0.026353782	0.147647955
PRAMEF11	3	10	0.001981791	0.151073927	0.597477938	0.026353782	0.147647955
SLC35F1	3	10	0.001981791	0.151073927	0.597477938	0.026353782	0.147647955
MYCBP2	13	20	0.001983164	0.316344311	0.68896825	0.140529043	0.147647955
USH2A	85	68	0.002168124	0.538674331	0.814885312	0.356086575	0.156427829
ARAP2	9	16	0.002249057	0.277548993	0.684744038	0.10557612	0.156427829
DLC1	9	16	0.002249057	0.277548993	0.684744038	0.10557612	0.156427829
GUCY2F	9	16	0.002249057	0.277548993	0.684744038	0.10557612	0.156427829
OR2T33	9	16	0.002249057	0.277548993	0.684744038	0.10557612	0.156427829
NTRK3	16	22	0.002354464	0.352569683	0.726292581	0.167662835	0.156778204
IFT172	6	13	0.002526169	0.230051933	0.66357432	0.070317391	0.156778204
RYR3	44	42	0.002713397	0.483116291	0.796674448	0.293025347	0.156778204
FAT2	17	23	0.002715734	0.357053848	0.722392853	0.173302705	0.156778204
CD163L1	8	15	0.002719505	0.264048014	0.680302456	0.09476813	0.156778204
ATP10B	10	17	0.002781741	0.289277218	0.68746231	0.11541979	0.156778204
CNTN1	10	17	0.002781741	0.289277218	0.68746231	0.11541979	0.156778204
HECTD4	10	17	0.002781741	0.289277218	0.68746231	0.11541979	0.156778204
GLDC	5	12	0.00290151	0.208397558	0.649131269	0.056499741	0.156778204
KNTC1	5	12	0.00290151	0.208397558	0.649131269	0.056499741	0.156778204
WDR17	5	12	0.00290151	0.208397558	0.649131269	0.056499741	0.156778204
CCDC93	1	7	0.003058517	0.072892763	0.575131654	0.00160973	0.156778204
ELN	1	7	0.003058517	0.072892763	0.575131654	0.00160973	0.156778204
GPBP1L1	1	7	0.003058517	0.072892763	0.575131654	0.00160973	0.156778204
GRHL2	1	7	0.003058517	0.072892763	0.575131654	0.00160973	0.156778204
HRG	1	7	0.003058517	0.072892763	0.575131654	0.00160973	0.156778204
IPMK	1	7	0.003058517	0.072892763	0.575131654	0.00160973	0.156778204
LRMP	1	7	0.003058517	0.072892763	0.575131654	0.00160973	0.156778204
MAPK8IP3	1	7	0.003058517	0.072892763	0.575131654	0.00160973	0.156778204
MYH7B	1	7	0.003058517	0.072892763	0.575131654	0.00160973	0.156778204
SIX3	1	7	0.003058517	0.072892763	0.575131654	0.00160973	0.156778204
SLC25A14	1	7	0.003058517	0.072892763	0.575131654	0.00160973	0.156778204
SLC39A11	1	7	0.003058517	0.072892763	0.575131654	0.00160973	0.156778204
SPOCK1	1	7	0.003058517	0.072892763	0.575131654	0.00160973	0.156778204
STRA8	1	7	0.003058517	0.072892763	0.575131654	0.00160973	0.156778204
TMEM260	1	7	0.003058517	0.072892763	0.575131654	0.00160973	0.156778204
ZNF215	1	7	0.003058517	0.072892763	0.575131654	0.00160973	0.156778204
SPTA1	67	57	0.00308455	0.521600903	0.80912567	0.336475656	0.156778204
TENM3	21	26	0.003258414	0.387218139	0.742670531	0.199751757	0.164037861
TLL2	7	14	0.003584389	0.248388518	0.673534209	0.083074748	0.164733407
C7orf60	4	11	0.003721338	0.18249119	0.628143864	0.041713517	0.164733407
DCLK1	4	11	0.003721338	0.18249119	0.628143864	0.041713517	0.164733407
FCRLA	4	11	0.003721338	0.18249119	0.628143864	0.041713517	0.164733407
LRRK1	4	11	0.003721338	0.18249119	0.628143864	0.041713517	0.164733407
ZWINT	4	11	0.003721338	0.18249119	0.628143864	0.041713517	0.164733407
ZNF521	22	26	0.003764875	0.406961394	0.775294402	0.21183576	0.164733407
HEPH	19	24	0.003807576	0.382277642	0.754198851	0.191291448	0.164733407

ANKRD30A	26	29	0.003948203	0.427871991	0.783659562	0.232515427	0.164733407
FANCI	2	8	0.004047947	0.12710134	0.646970003	0.013021395	0.164733407
HELZ2	2	8	0.004047947	0.12710134	0.646970003	0.013021395	0.164733407
KRT31	2	8	0.004047947	0.12710134	0.646970003	0.013021395	0.164733407
KRTAP10-4	2	8	0.004047947	0.12710134	0.646970003	0.013021395	0.164733407
OR5M8	2	8	0.004047947	0.12710134	0.646970003	0.013021395	0.164733407
PCM1	2	8	0.004047947	0.12710134	0.646970003	0.013021395	0.164733407
POLR2B	2	8	0.004047947	0.12710134	0.646970003	0.013021395	0.164733407
SPAST	2	8	0.004047947	0.12710134	0.646970003	0.013021395	0.164733407
FAT1	23	27	0.004273583	0.408214629	0.768256841	0.215285951	0.164733407
DMBT1	14	20	0.004288223	0.341724467	0.733755112	0.15502342	0.164733407
FAT4	36	36	0.00455889	0.469302595	0.803580163	0.273888617	0.164733407
AGXT2	3	9	0.004612785	0.168875755	0.688649398	0.029029697	0.164733407
AK5	3	9	0.004612785	0.168875755	0.688649398	0.029029697	0.164733407
DNAJC13	3	9	0.004612785	0.168875755	0.688649398	0.029029697	0.164733407
GPRIN3	3	9	0.004612785	0.168875755	0.688649398	0.029029697	0.164733407
MYO9B	3	9	0.004612785	0.168875755	0.688649398	0.029029697	0.164733407
OPLAH	3	9	0.004612785	0.168875755	0.688649398	0.029029697	0.164733407
OR8G5	3	9	0.004612785	0.168875755	0.688649398	0.029029697	0.164733407
PTK7	3	9	0.004612785	0.168875755	0.688649398	0.029029697	0.164733407
UNC5A	3	9	0.004612785	0.168875755	0.688649398	0.029029697	0.164733407
ASB4	0	5	0.004736942	0	0.567937629	0	0.164733407
ASCC2	0	5	0.004736942	0	0.567937629	0	0.164733407
CATSPERE	0	5	0.004736942	0	0.567937629	0	0.164733407
CC2D2A	0	5	0.004736942	0	0.567937629	0	0.164733407
CDKN1B	0	5	0.004736942	0	0.567937629	0	0.164733407
COPB2	0	5	0.004736942	0	0.567937629	0	0.164733407
CXCR1	0	5	0.004736942	0	0.567937629	0	0.164733407
EGR1	0	5	0.004736942	0	0.567937629	0	0.164733407
IMPAD1	0	5	0.004736942	0	0.567937629	0	0.164733407
KIAA1468	0	5	0.004736942	0	0.567937629	0	0.164733407
LTB	0	5	0.004736942	0	0.567937629	0	0.164733407
MSRA	0	5	0.004736942	0	0.567937629	0	0.164733407
PCCA	0	5	0.004736942	0	0.567937629	0	0.164733407
PYGL	0	5	0.004736942	0	0.567937629	0	0.164733407
SEC31B	0	5	0.004736942	0	0.567937629	0	0.164733407
SLC24A3	0	5	0.004736942	0	0.567937629	0	0.164733407
TMC1	0	5	0.004736942	0	0.567937629	0	0.164733407
ZDHHC19	0	5	0.004736942	0	0.567937629	0	0.164733407
PIK3CG	10	16	0.004950961	0.309298876	0.744613667	0.122385342	0.16901531
SLCO5A1	10	16	0.004950961	0.309298876	0.744613667	0.122385342	0.16901531
A2ML1	8	14	0.005143287	0.284707855	0.74520685	0.101198958	0.16901531
CLCA4	8	14	0.005143287	0.284707855	0.74520685	0.101198958	0.16901531
OR51V1	8	14	0.005143287	0.284707855	0.74520685	0.101198958	0.16901531
PLXNB2	8	14	0.005143287	0.284707855	0.74520685	0.101198958	0.16901531
AUTS2	6	12	0.005147837	0.250765453	0.738004727	0.075751341	0.16901531
PGBD1	6	12	0.005147837	0.250765453	0.738004727	0.075751341	0.16901531
PIK3AP1	6	12	0.005147837	0.250765453	0.738004727	0.075751341	0.16901531
MYO3A	15	20	0.005200422	0.367264994	0.778722445	0.169741043	0.169687837
COL11A1	49	44	0.00524296	0.514715201	0.836662082	0.317037736	0.170026305
CACNA1E	34	34	0.005703523	0.47299045	0.820835869	0.27237322	0.183834296
ABLIM3	5	11	0.005865326	0.22872874	0.728950594	0.061220077	0.184548286
DDX60L	5	11	0.005865326	0.22872874	0.728950594	0.061220077	0.184548286
GCC2	5	11	0.005865326	0.22872874	0.728950594	0.061220077	0.184548286
MYO9A	5	11	0.005865326	0.22872874	0.728950594	0.061220077	0.184548286
USP29	13	18	0.006065493	0.356083057	0.791651217	0.155953599	0.18932797
NAV3	52	46	0.006103918	0.519886275	0.83713845	0.323260169	0.18932797
TG	26	28	0.006124685	0.446250841	0.821633467	0.241519327	0.18932797

ZDBF2	16	21	0.00618507	0.371808191	0.772395432	0.175753303	0.190083023
NRXN1	36	35	0.006681047	0.486271454	0.835634743	0.282973826	0.200040451
OR2G6	9	15	0.006788207	0.297922685	0.745567832	0.112279947	0.200040451
MUC17	48	43	0.006969228	0.518125162	0.845730544	0.317838252	0.200040451
ACSL6	4	10	0.007249456	0.201964335	0.713744962	0.045518651	0.200040451
C10orf12	4	10	0.007249456	0.201964335	0.713744962	0.045518651	0.200040451
CCDC40	4	10	0.007249456	0.201964335	0.713744962	0.045518651	0.200040451
LRIT2	4	10	0.007249456	0.201964335	0.713744962	0.045518651	0.200040451
MGAT4C	4	10	0.007249456	0.201964335	0.713744962	0.045518651	0.200040451
POP1	4	10	0.007249456	0.201964335	0.713744962	0.045518651	0.200040451
SLC17A3	4	10	0.007249456	0.201964335	0.713744962	0.045518651	0.200040451
KCNB2	17	21	0.007282664	0.39628242	0.81491689	0.190129223	0.200040451
DCC	12	17	0.007889	0.349201601	0.798172006	0.148109075	0.200040451
NDST4	12	17	0.007889	0.349201601	0.798172006	0.148109075	0.200040451
OGDHL	12	17	0.007889	0.349201601	0.798172006	0.148109075	0.200040451
ACTR3B	1	6	0.007984967	0.085540792	0.714112163	0.001850705	0.200040451
ALCAM	1	6	0.007984967	0.085540792	0.714112163	0.001850705	0.200040451
ANAPC4	1	6	0.007984967	0.085540792	0.714112163	0.001850705	0.200040451
ATP9B	1	6	0.007984967	0.085540792	0.714112163	0.001850705	0.200040451
BMP15	1	6	0.007984967	0.085540792	0.714112163	0.001850705	0.200040451
CTR9	1	6	0.007984967	0.085540792	0.714112163	0.001850705	0.200040451
CYP2B6	1	6	0.007984967	0.085540792	0.714112163	0.001850705	0.200040451
FSTL4	1	6	0.007984967	0.085540792	0.714112163	0.001850705	0.200040451
GCM1	1	6	0.007984967	0.085540792	0.714112163	0.001850705	0.200040451
ITPRIP	1	6	0.007984967	0.085540792	0.714112163	0.001850705	0.200040451
KRT4	1	6	0.007984967	0.085540792	0.714112163	0.001850705	0.200040451
MACC1	1	6	0.007984967	0.085540792	0.714112163	0.001850705	0.200040451
MYLIP	1	6	0.007984967	0.085540792	0.714112163	0.001850705	0.200040451
NAE1	1	6	0.007984967	0.085540792	0.714112163	0.001850705	0.200040451
OR11H4	1	6	0.007984967	0.085540792	0.714112163	0.001850705	0.200040451
OVCH2	1	6	0.007984967	0.085540792	0.714112163	0.001850705	0.200040451
RASGRP3	1	6	0.007984967	0.085540792	0.714112163	0.001850705	0.200040451
RNF43	1	6	0.007984967	0.085540792	0.714112163	0.001850705	0.200040451
RSAD2	1	6	0.007984967	0.085540792	0.714112163	0.001850705	0.200040451
SCAPER	1	6	0.007984967	0.085540792	0.714112163	0.001850705	0.200040451
SEC24B	1	6	0.007984967	0.085540792	0.714112163	0.001850705	0.200040451
TMPRSS6	1	6	0.007984967	0.085540792	0.714112163	0.001850705	0.200040451
TNFRSF8	1	6	0.007984967	0.085540792	0.714112163	0.001850705	0.200040451
TOPBP1	1	6	0.007984967	0.085540792	0.714112163	0.001850705	0.200040451
ZNF626	1	6	0.007984967	0.085540792	0.714112163	0.001850705	0.200040451
CTNNA2	25	27	0.008202142	0.446603406	0.831132698	0.23906649	0.204511892
SLIT2	18	22	0.008407617	0.399134856	0.806308427	0.195210843	0.208650991
ADGRL2	15	19	0.00872997	0.38911941	0.833396266	0.178557717	0.213469496
BRWD3	15	19	0.00872997	0.38911941	0.833396266	0.178557717	0.213469496
SRCAP	15	19	0.00872997	0.38911941	0.833396266	0.178557717	0.213469496
GTF3C1	10	15	0.008786764	0.332007804	0.810977778	0.130164017	0.213469496
CAD	8	13	0.009631677	0.308495108	0.82239514	0.108452236	0.213469496
CHRM3	8	13	0.009631677	0.308495108	0.82239514	0.108452236	0.213469496
SLC6A15	8	13	0.009631677	0.308495108	0.82239514	0.108452236	0.213469496
ZFHX4	84	64	0.009675319	0.585204461	0.888880133	0.385629361	0.213469496
CCDC74B	2	7	0.009735689	0.146122175	0.778957381	0.014630761	0.213469496
CD44	2	7	0.009735689	0.146122175	0.778957381	0.014630761	0.213469496
DOPEY1	2	7	0.009735689	0.146122175	0.778957381	0.014630761	0.213469496
FAM73A	2	7	0.009735689	0.146122175	0.778957381	0.014630761	0.213469496
FBXO43	2	7	0.009735689	0.146122175	0.778957381	0.014630761	0.213469496
FCHO2	2	7	0.009735689	0.146122175	0.778957381	0.014630761	0.213469496
FNDC3A	2	7	0.009735689	0.146122175	0.778957381	0.014630761	0.213469496
GAS7	2	7	0.009735689	0.146122175	0.778957381	0.014630761	0.213469496

IVL	2	7	0.009735689	0.146122175	0.778957381	0.014630761	0.213469496
KLHL10	2	7	0.009735689	0.146122175	0.778957381	0.014630761	0.213469496
PAH	2	7	0.009735689	0.146122175	0.778957381	0.014630761	0.213469496
PRR27	2	7	0.009735689	0.146122175	0.778957381	0.014630761	0.213469496
RNF10	2	7	0.009735689	0.146122175	0.778957381	0.014630761	0.213469496
TCOF1	2	7	0.009735689	0.146122175	0.778957381	0.014630761	0.213469496
TOR1AIP2	2	7	0.009735689	0.146122175	0.778957381	0.014630761	0.213469496
TTC27	2	7	0.009735689	0.146122175	0.778957381	0.014630761	0.213469496
ZNF816	2	7	0.009735689	0.146122175	0.778957381	0.014630761	0.213469496
ZYG11B	2	7	0.009735689	0.146122175	0.778957381	0.014630761	0.213469496
ASTN1	36	34	0.009768484	0.504236759	0.869822692	0.292608219	0.213469496
PTPRB	16	20	0.009822275	0.392967672	0.823933234	0.184600095	0.213469496
HCN1	23	25	0.010068649	0.446940673	0.85163586	0.233544597	0.213469496
PTPRT	23	25	0.010068649	0.446940673	0.85163586	0.233544597	0.213469496
PGK2	20	2	0.010168796	5.548726678	49.54571398	1.321618871	0.213469496
ARHGAP33	3	8	0.01045944	0.191122356	0.809774075	0.032223221	0.213469496
ASIC2	3	8	0.01045944	0.191122356	0.809774075	0.032223221	0.213469496
CD6	3	8	0.01045944	0.191122356	0.809774075	0.032223221	0.213469496
CYP2C19	3	8	0.01045944	0.191122356	0.809774075	0.032223221	0.213469496
HSPA8	3	8	0.01045944	0.191122356	0.809774075	0.032223221	0.213469496
IGSF8	3	8	0.01045944	0.191122356	0.809774075	0.032223221	0.213469496
MICAL2	3	8	0.01045944	0.191122356	0.809774075	0.032223221	0.213469496
NUP153	3	8	0.01045944	0.191122356	0.809774075	0.032223221	0.213469496
OR5M11	3	8	0.01045944	0.191122356	0.809774075	0.032223221	0.213469496
SLC24A5	3	8	0.01045944	0.191122356	0.809774075	0.032223221	0.213469496
SMYD3	3	8	0.01045944	0.191122356	0.809774075	0.032223221	0.213469496
SPATA16	3	8	0.01045944	0.191122356	0.809774075	0.032223221	0.213469496
TGFBRAP1	3	8	0.01045944	0.191122356	0.809774075	0.032223221	0.213469496
TTC14	3	8	0.01045944	0.191122356	0.809774075	0.032223221	0.213469496
ZNF366	3	8	0.01045944	0.191122356	0.809774075	0.032223221	0.213469496
FAM47B	20	23	0.010923045	0.424064739	0.836214447	0.213394058	0.221365449
COL24A1	11	16	0.010930076	0.341247362	0.804650664	0.139539094	0.221365449
COL12A1	28	28	0.011409297	0.483752368	0.882526673	0.265025325	0.221857766
UNC5D	14	18	0.01146145	0.384650577	0.8433571	0.172011999	0.221857766
CHD7	9	14	0.011664379	0.321209578	0.816982386	0.11981955	0.221857766
CTTNBP2	9	14	0.011664379	0.321209578	0.816982386	0.11981955	0.221857766
IGF2R	9	14	0.011664379	0.321209578	0.816982386	0.11981955	0.221857766
PRKCB	9	14	0.011664379	0.321209578	0.816982386	0.11981955	0.221857766
RAD51AP2	9	14	0.011664379	0.321209578	0.816982386	0.11981955	0.221857766
ABCB4	5	10	0.01175183	0.253129362	0.829196137	0.06671931	0.221857766
ANKFN1	5	10	0.01175183	0.253129362	0.829196137	0.06671931	0.221857766
AOX1	5	10	0.01175183	0.253129362	0.829196137	0.06671931	0.221857766
ARHGEF17	5	10	0.01175183	0.253129362	0.829196137	0.06671931	0.221857766
CHM	5	10	0.01175183	0.253129362	0.829196137	0.06671931	0.221857766
HK3	5	10	0.01175183	0.253129362	0.829196137	0.06671931	0.221857766
KIAA1429	5	10	0.01175183	0.253129362	0.829196137	0.06671931	0.221857766
NR4A2	5	10	0.01175183	0.253129362	0.829196137	0.06671931	0.221857766
PCDHA8	5	10	0.01175183	0.253129362	0.829196137	0.06671931	0.221857766
PNLIP	5	10	0.01175183	0.253129362	0.829196137	0.06671931	0.221857766
PRAMEF4	5	10	0.01175183	0.253129362	0.829196137	0.06671931	0.221857766
SND1	5	10	0.01175183	0.253129362	0.829196137	0.06671931	0.221857766
TSHZ3	29	29	0.011936618	0.481989382	0.870950376	0.266620087	0.224011346
CEP350	7	12	0.012035418	0.293387442	0.826787066	0.095871876	0.224011346
DACH1	7	12	0.012035418	0.293387442	0.826787066	0.095871876	0.224011346
PRG4	7	12	0.012035418	0.293387442	0.826787066	0.095871876	0.224011346
ITGAX	18	21	0.013032705	0.420913488	0.857649438	0.204594636	0.240877206
SLC39A12	18	21	0.013032705	0.420913488	0.857649438	0.204594636	0.240877206
HFM1	12	16	0.013339374	0.373393948	0.864909308	0.156987184	0.243985914

LAMA4	12	16	0.013339374	0.373393948	0.864909308	0.156987184	0.243985914
PCDHB16	12	16	0.013339374	0.373393948	0.864909308	0.156987184	0.243985914
EGFR	47	12	0.013423273	2.246980507	4.797831422	1.131718567	0.244673866
PCDH15	49	42	0.014242547	0.54770148	0.894802475	0.336028977	0.246847882
ATG2A	4	9	0.01424302	0.225750156	0.823932091	0.050041663	0.246847882
CPA3	4	9	0.01424302	0.225750156	0.823932091	0.050041663	0.246847882
GPR156	4	9	0.01424302	0.225750156	0.823932091	0.050041663	0.246847882
IGSF11	4	9	0.01424302	0.225750156	0.823932091	0.050041663	0.246847882
KCNK13	4	9	0.01424302	0.225750156	0.823932091	0.050041663	0.246847882
KL	4	9	0.01424302	0.225750156	0.823932091	0.050041663	0.246847882
LRGUK	4	9	0.01424302	0.225750156	0.823932091	0.050041663	0.246847882
NFRKB	4	9	0.01424302	0.225750156	0.823932091	0.050041663	0.246847882
PRTG	4	9	0.01424302	0.225750156	0.823932091	0.050041663	0.246847882
PTPRO	4	9	0.01424302	0.225750156	0.823932091	0.050041663	0.246847882
SPTBN2	4	9	0.01424302	0.225750156	0.823932091	0.050041663	0.246847882
UBAP2L	4	9	0.01424302	0.225750156	0.823932091	0.050041663	0.246847882
WNK2	4	9	0.01424302	0.225750156	0.823932091	0.050041663	0.246847882
ZCCHC11	4	9	0.01424302	0.225750156	0.823932091	0.050041663	0.246847882
SYNE1	27	27	0.014865165	0.485503381	0.894752396	0.26330704	0.256788435
DSCAM	19	21	0.015347936	0.445703068	0.900601557	0.219232281	0.261438252
TMPRSS15	19	21	0.015347936	0.445703068	0.900601557	0.219232281	0.261438252
PCDHGA6	10	14	0.015450519	0.357956175	0.88894381	0.138841465	0.261438252
SLC44A5	10	14	0.015450519	0.357956175	0.88894381	0.138841465	0.261438252
ABCA12	16	19	0.015578869	0.416351012	0.881882395	0.194209182	0.261438252
LCT	16	19	0.015578869	0.416351012	0.881882395	0.194209182	0.261438252
GRIN3A	13	17	0.015853737	0.379453594	0.85384382	0.164869893	0.261438252
PEG3	33	31	0.016178326	0.512730903	0.904144078	0.291187143	0.261438252
ADGRB1	6	11	0.016321344	0.275250046	0.829448963	0.081992394	0.261438252
ASXL2	6	11	0.016321344	0.275250046	0.829448963	0.081992394	0.261438252
CYLC2	6	11	0.016321344	0.275250046	0.829448963	0.081992394	0.261438252
HOXB3	6	11	0.016321344	0.275250046	0.829448963	0.081992394	0.261438252
KIAA1462	6	11	0.016321344	0.275250046	0.829448963	0.081992394	0.261438252
MYBPC1	6	11	0.016321344	0.275250046	0.829448963	0.081992394	0.261438252
MYOF	6	11	0.016321344	0.275250046	0.829448963	0.081992394	0.261438252
PLA2G4A	6	11	0.016321344	0.275250046	0.829448963	0.081992394	0.261438252
PLD1	6	11	0.016321344	0.275250046	0.829448963	0.081992394	0.261438252
RTN1	6	11	0.016321344	0.275250046	0.829448963	0.081992394	0.261438252
SALL4	6	11	0.016321344	0.275250046	0.829448963	0.081992394	0.261438252
SIPA1L1	6	11	0.016321344	0.275250046	0.829448963	0.081992394	0.261438252
SLC6A18	6	11	0.016321344	0.275250046	0.829448963	0.081992394	0.261438252
SP140	6	11	0.016321344	0.275250046	0.829448963	0.081992394	0.261438252
TMEM2	6	11	0.016321344	0.275250046	0.829448963	0.081992394	0.261438252
ZNF761	6	11	0.016321344	0.275250046	0.829448963	0.081992394	0.261438252
FLNC	20	22	0.016584708	0.446301651	0.887165574	0.223275831	0.264056518
ITGA8	20	22	0.016584708	0.446301651	0.887165574	0.223275831	0.264056518
COL5A2	24	25	0.016693524	0.467886117	0.886502229	0.246380722	0.264233533
ADGRB3	29	28	0.017511292	0.502692051	0.913327213	0.276912168	0.264233533
PRSS1	10	0	0.017775362	Inf	Inf	1.202244709	0.264233533
TPO	11	15	0.017929497	0.366301205	0.87659734	0.148349503	0.264233533
ALMS1	14	17	0.018649083	0.409895379	0.909752728	0.18177771	0.264233533
MYO16	14	17	0.018649083	0.409895379	0.909752728	0.18177771	0.264233533
SIPA1L2	14	17	0.018649083	0.409895379	0.909752728	0.18177771	0.264233533
LPA	21	22	0.019189464	0.470114679	0.927917488	0.237527676	0.264233533
XIRP2	66	51	0.019817266	0.602186342	0.943824053	0.385203126	0.264233533
CDH9	26	26	0.019881102	0.487242541	0.907765884	0.261413505	0.264233533
CASR	9	13	0.020023634	0.348072143	0.901965133	0.128325492	0.264233533
CFAP54	9	13	0.020023634	0.348072143	0.901965133	0.128325492	0.264233533
CREBBP	9	13	0.020023634	0.348072143	0.901965133	0.128325492	0.264233533

MAGEL2	9	13	0.020023634	0.348072143	0.901965133	0.128325492	0.264233533
NRXN3	18	20	0.020149431	0.444866477	0.915028632	0.21482543	0.264233533
ABI3	1	5	0.02039706	0.103251226	0.933588001	0.002172187	0.264233533
ACKR2	1	5	0.02039706	0.103251226	0.933588001	0.002172187	0.264233533
ADGRG3	1	5	0.02039706	0.103251226	0.933588001	0.002172187	0.264233533
AGAP4	1	5	0.02039706	0.103251226	0.933588001	0.002172187	0.264233533
ARSB	1	5	0.02039706	0.103251226	0.933588001	0.002172187	0.264233533
C16orf62	1	5	0.02039706	0.103251226	0.933588001	0.002172187	0.264233533
C1QTNF2	1	5	0.02039706	0.103251226	0.933588001	0.002172187	0.264233533
CCDC66	1	5	0.02039706	0.103251226	0.933588001	0.002172187	0.264233533
CCNT2	1	5	0.02039706	0.103251226	0.933588001	0.002172187	0.264233533
CCP110	1	5	0.02039706	0.103251226	0.933588001	0.002172187	0.264233533
CCT6A	1	5	0.02039706	0.103251226	0.933588001	0.002172187	0.264233533
CHAMP1	1	5	0.02039706	0.103251226	0.933588001	0.002172187	0.264233533
COPG1	1	5	0.02039706	0.103251226	0.933588001	0.002172187	0.264233533
CXorf36	1	5	0.02039706	0.103251226	0.933588001	0.002172187	0.264233533
EDARADD	1	5	0.02039706	0.103251226	0.933588001	0.002172187	0.264233533
EMX2	1	5	0.02039706	0.103251226	0.933588001	0.002172187	0.264233533
EP300	1	5	0.02039706	0.103251226	0.933588001	0.002172187	0.264233533
FARP2	1	5	0.02039706	0.103251226	0.933588001	0.002172187	0.264233533
FTHL17	1	5	0.02039706	0.103251226	0.933588001	0.002172187	0.264233533
GAL3ST3	1	5	0.02039706	0.103251226	0.933588001	0.002172187	0.264233533
GGN	1	5	0.02039706	0.103251226	0.933588001	0.002172187	0.264233533
GJB5	1	5	0.02039706	0.103251226	0.933588001	0.002172187	0.264233533
GNAI1	1	5	0.02039706	0.103251226	0.933588001	0.002172187	0.264233533
GPHN	1	5	0.02039706	0.103251226	0.933588001	0.002172187	0.264233533
GRAMD1C	1	5	0.02039706	0.103251226	0.933588001	0.002172187	0.264233533
GYS2	1	5	0.02039706	0.103251226	0.933588001	0.002172187	0.264233533
ITPRIPL1	1	5	0.02039706	0.103251226	0.933588001	0.002172187	0.264233533
LRRFIP2	1	5	0.02039706	0.103251226	0.933588001	0.002172187	0.264233533
10-Mar	1	5	0.02039706	0.103251226	0.933588001	0.002172187	0.264233533
MICALCL	1	5	0.02039706	0.103251226	0.933588001	0.002172187	0.264233533
MRPS35	1	5	0.02039706	0.103251226	0.933588001	0.002172187	0.264233533
NCF4	1	5	0.02039706	0.103251226	0.933588001	0.002172187	0.264233533
NTHL1	1	5	0.02039706	0.103251226	0.933588001	0.002172187	0.264233533
PDPR	1	5	0.02039706	0.103251226	0.933588001	0.002172187	0.264233533
PGM2L1	1	5	0.02039706	0.103251226	0.933588001	0.002172187	0.264233533
PLBD2	1	5	0.02039706	0.103251226	0.933588001	0.002172187	0.264233533
PRKRIR	1	5	0.02039706	0.103251226	0.933588001	0.002172187	0.264233533
PRMT5	1	5	0.02039706	0.103251226	0.933588001	0.002172187	0.264233533
RAD51AP1	1	5	0.02039706	0.103251226	0.933588001	0.002172187	0.264233533
RARS2	1	5	0.02039706	0.103251226	0.933588001	0.002172187	0.264233533
SAFB2	1	5	0.02039706	0.103251226	0.933588001	0.002172187	0.264233533
SASH3	1	5	0.02039706	0.103251226	0.933588001	0.002172187	0.264233533
SFPQ	1	5	0.02039706	0.103251226	0.933588001	0.002172187	0.264233533
SLC43A2	1	5	0.02039706	0.103251226	0.933588001	0.002172187	0.264233533
SPEM1	1	5	0.02039706	0.103251226	0.933588001	0.002172187	0.264233533
STIP1	1	5	0.02039706	0.103251226	0.933588001	0.002172187	0.264233533
TJP2	1	5	0.02039706	0.103251226	0.933588001	0.002172187	0.264233533
TMPRSS3	1	5	0.02039706	0.103251226	0.933588001	0.002172187	0.264233533
VCX	1	5	0.02039706	0.103251226	0.933588001	0.002172187	0.264233533
VNN1	1	5	0.02039706	0.103251226	0.933588001	0.002172187	0.264233533
WDFY1	1	5	0.02039706	0.103251226	0.933588001	0.002172187	0.264233533
XXYLT1	1	5	0.02039706	0.103251226	0.933588001	0.002172187	0.264233533
ZNF222	1	5	0.02039706	0.103251226	0.933588001	0.002172187	0.264233533
ZNF714	1	5	0.02039706	0.103251226	0.933588001	0.002172187	0.264233533
DCDC1	22	23	0.020423963	0.469466308	0.913173987	0.240724788	0.264233533
NOTCH4	22	23	0.020423963	0.469466308	0.913173987	0.240724788	0.264233533

STAB2	15	18	0.021401681	0.413398347	0.895298955	0.188284027	0.264233533
ADCY5	7	11	0.021905811	0.322018271	0.929845206	0.103762942	0.264233533
FOXP2	7	11	0.021905811	0.322018271	0.929845206	0.103762942	0.264233533
ITIH2	7	11	0.021905811	0.322018271	0.929845206	0.103762942	0.264233533
OR5T1	7	11	0.021905811	0.322018271	0.929845206	0.103762942	0.264233533
PPARGC1A	7	11	0.021905811	0.322018271	0.929845206	0.103762942	0.264233533
SMARCA1	7	11	0.021905811	0.322018271	0.929845206	0.103762942	0.264233533
WDR72	7	11	0.021905811	0.322018271	0.929845206	0.103762942	0.264233533
CDH11	12	15	0.022341418	0.400807044	0.942450888	0.166829218	0.264233533
CMYA5	12	15	0.022341418	0.400807044	0.942450888	0.166829218	0.264233533
MYH3	12	15	0.022341418	0.400807044	0.942450888	0.166829218	0.264233533
CYP7A1	2	6	0.022764315	0.171475029	0.972459024	0.016741823	0.264233533
DGKG	2	6	0.022764315	0.171475029	0.972459024	0.016741823	0.264233533
ERC2	2	6	0.022764315	0.171475029	0.972459024	0.016741823	0.264233533
FASLG	2	6	0.022764315	0.171475029	0.972459024	0.016741823	0.264233533
FRG1	2	6	0.022764315	0.171475029	0.972459024	0.016741823	0.264233533
GTF2E1	2	6	0.022764315	0.171475029	0.972459024	0.016741823	0.264233533
HPS4	2	6	0.022764315	0.171475029	0.972459024	0.016741823	0.264233533
INMT	2	6	0.022764315	0.171475029	0.972459024	0.016741823	0.264233533
IPO8	2	6	0.022764315	0.171475029	0.972459024	0.016741823	0.264233533
KARS	2	6	0.022764315	0.171475029	0.972459024	0.016741823	0.264233533
KRT9	2	6	0.022764315	0.171475029	0.972459024	0.016741823	0.264233533
LPIN2	2	6	0.022764315	0.171475029	0.972459024	0.016741823	0.264233533
MAP7	2	6	0.022764315	0.171475029	0.972459024	0.016741823	0.264233533
NEURL1	2	6	0.022764315	0.171475029	0.972459024	0.016741823	0.264233533
PRMT8	2	6	0.022764315	0.171475029	0.972459024	0.016741823	0.264233533
PSD2	2	6	0.022764315	0.171475029	0.972459024	0.016741823	0.264233533
RIPK1	2	6	0.022764315	0.171475029	0.972459024	0.016741823	0.264233533
RXFP2	2	6	0.022764315	0.171475029	0.972459024	0.016741823	0.264233533
SARDH	2	6	0.022764315	0.171475029	0.972459024	0.016741823	0.264233533
SCRIB	2	6	0.022764315	0.171475029	0.972459024	0.016741823	0.264233533
SLC30A8	2	6	0.022764315	0.171475029	0.972459024	0.016741823	0.264233533
SMARCC2	2	6	0.022764315	0.171475029	0.972459024	0.016741823	0.264233533
SOGA1	2	6	0.022764315	0.171475029	0.972459024	0.016741823	0.264233533
ON1-GTF2A	2	6	0.022764315	0.171475029	0.972459024	0.016741823	0.264233533
TAOK3	2	6	0.022764315	0.171475029	0.972459024	0.016741823	0.264233533
TBCK	2	6	0.022764315	0.171475029	0.972459024	0.016741823	0.264233533
TDP1	2	6	0.022764315	0.171475029	0.972459024	0.016741823	0.264233533
TLR2	2	6	0.022764315	0.171475029	0.972459024	0.016741823	0.264233533
TRAK2	2	6	0.022764315	0.171475029	0.972459024	0.016741823	0.264233533
TRAT1	2	6	0.022764315	0.171475029	0.972459024	0.016741823	0.264233533
ZAP70	2	6	0.022764315	0.171475029	0.972459024	0.016741823	0.264233533
ZNF470	2	6	0.022764315	0.171475029	0.972459024	0.016741823	0.264233533
ZNF773	2	6	0.022764315	0.171475029	0.972459024	0.016741823	0.264233533
ABCC12	5	9	0.023144178	0.28292805	0.958354619	0.073216951	0.264233533
ACE	5	9	0.023144178	0.28292805	0.958354619	0.073216951	0.264233533
ASTL	5	9	0.023144178	0.28292805	0.958354619	0.073216951	0.264233533
ATP11C	5	9	0.023144178	0.28292805	0.958354619	0.073216951	0.264233533
CALN1	5	9	0.023144178	0.28292805	0.958354619	0.073216951	0.264233533
CHRNA4	5	9	0.023144178	0.28292805	0.958354619	0.073216951	0.264233533
DOCK5	5	9	0.023144178	0.28292805	0.958354619	0.073216951	0.264233533
EIF4G1	5	9	0.023144178	0.28292805	0.958354619	0.073216951	0.264233533
LILRB5	5	9	0.023144178	0.28292805	0.958354619	0.073216951	0.264233533
MAGEB18	5	9	0.023144178	0.28292805	0.958354619	0.073216951	0.264233533
PPIG	5	9	0.023144178	0.28292805	0.958354619	0.073216951	0.264233533
RASGRF2	5	9	0.023144178	0.28292805	0.958354619	0.073216951	0.264233533
14-Sep	5	9	0.023144178	0.28292805	0.958354619	0.073216951	0.264233533
SGOL2	5	9	0.023144178	0.28292805	0.958354619	0.073216951	0.264233533

SLC22A12	5	9	0.023144178	0.28292805	0.958354619	0.073216951	0.264233533
SLC5A1	5	9	0.023144178	0.28292805	0.958354619	0.073216951	0.264233533
THSD7A	23	23	0.023312203	0.492390105	0.951967464	0.254589296	0.265578246
ZNF804B	24	24	0.024500053	0.490685785	0.936227805	0.257101799	0.278510279
CACNA1B	13	16	0.025082146	0.405740988	0.925404733	0.174678371	0.280948798
CNTN3	13	16	0.025082146	0.405740988	0.925404733	0.174678371	0.280948798
OR4A16	13	16	0.025082146	0.405740988	0.925404733	0.174678371	0.280948798
OR4C6	13	16	0.025082146	0.405740988	0.925404733	0.174678371	0.280948798
BOD1L1	20	21	0.025086612	0.470652881	0.94377901	0.233989025	0.280948798
THSD7B	20	21	0.025086612	0.470652881	0.94377901	0.233989025	0.280948798
WDFY3	20	21	0.025086612	0.470652881	0.94377901	0.233989025	0.280948798
LRP1B	92	66	0.025355794	0.630882349	0.952358897	0.41843925	0.283363063
CDH23	17	19	0.027431134	0.443756138	0.93059596	0.210005473	0.292017845
CLSTN2	8	12	0.027724038	0.336266714	0.91563591	0.116685782	0.292017845
CNBD1	8	12	0.027724038	0.336266714	0.91563591	0.116685782	0.292017845
EYA4	8	12	0.027724038	0.336266714	0.91563591	0.116685782	0.292017845
PCDHGA8	8	12	0.027724038	0.336266714	0.91563591	0.116685782	0.292017845
SPAM1	8	12	0.027724038	0.336266714	0.91563591	0.116685782	0.292017845
VCAM1	8	12	0.027724038	0.336266714	0.91563591	0.116685782	0.292017845
ACSL1	4	8	0.027898035	0.255480356	0.970633199	0.055474626	0.292017845
ADGRG6	4	8	0.027898035	0.255480356	0.970633199	0.055474626	0.292017845
APBA2	4	8	0.027898035	0.255480356	0.970633199	0.055474626	0.292017845
C10orf90	4	8	0.027898035	0.255480356	0.970633199	0.055474626	0.292017845
CASC5	4	8	0.027898035	0.255480356	0.970633199	0.055474626	0.292017845
CD1B	4	8	0.027898035	0.255480356	0.970633199	0.055474626	0.292017845
CHRD	4	8	0.027898035	0.255480356	0.970633199	0.055474626	0.292017845
ENAM	4	8	0.027898035	0.255480356	0.970633199	0.055474626	0.292017845
GAK	4	8	0.027898035	0.255480356	0.970633199	0.055474626	0.292017845
HEPACAM2	4	8	0.027898035	0.255480356	0.970633199	0.055474626	0.292017845
HTR3A	4	8	0.027898035	0.255480356	0.970633199	0.055474626	0.292017845
IGKC	4	8	0.027898035	0.255480356	0.970633199	0.055474626	0.292017845
IGKV1-17	4	8	0.027898035	0.255480356	0.970633199	0.055474626	0.292017845
NFATC2	4	8	0.027898035	0.255480356	0.970633199	0.055474626	0.292017845
OR2J2	4	8	0.027898035	0.255480356	0.970633199	0.055474626	0.292017845
OR4D5	4	8	0.027898035	0.255480356	0.970633199	0.055474626	0.292017845
PAX4	4	8	0.027898035	0.255480356	0.970633199	0.055474626	0.292017845
SPATA5	4	8	0.027898035	0.255480356	0.970633199	0.055474626	0.292017845
TAB3	4	8	0.027898035	0.255480356	0.970633199	0.055474626	0.292017845
TDRD1	4	8	0.027898035	0.255480356	0.970633199	0.055474626	0.292017845
UBQLN3	4	8	0.027898035	0.255480356	0.970633199	0.055474626	0.292017845
USP28	4	8	0.027898035	0.255480356	0.970633199	0.055474626	0.292017845
WDR36	4	8	0.027898035	0.255480356	0.970633199	0.055474626	0.292017845
WISP1	4	8	0.027898035	0.255480356	0.970633199	0.055474626	0.292017845
XPNPEP2	4	8	0.027898035	0.255480356	0.970633199	0.055474626	0.292017845
ENTHD1	6	10	0.028891712	0.304596284	0.944317873	0.089325754	0.297702902
IRS1	6	10	0.028891712	0.304596284	0.944317873	0.089325754	0.297702902
LHX8	6	10	0.028891712	0.304596284	0.944317873	0.089325754	0.297702902
MYOC	6	10	0.028891712	0.304596284	0.944317873	0.089325754	0.297702902
PEX5L	6	10	0.028891712	0.304596284	0.944317873	0.089325754	0.297702902
PRKCG	6	10	0.028891712	0.304596284	0.944317873	0.089325754	0.297702902
RIMS1	6	10	0.028891712	0.304596284	0.944317873	0.089325754	0.297702902
TXLNB	6	10	0.028891712	0.304596284	0.944317873	0.089325754	0.297702902
RP1L1	44	37	0.029237481	0.569594959	0.953411157	0.341477034	0.298037033
ABCC9	11	14	0.029318815	0.394928767	0.961131615	0.158221748	0.298037033
ADCY10	11	14	0.029318815	0.394928767	0.961131615	0.158221748	0.298037033
FRY	11	14	0.029318815	0.394928767	0.961131615	0.158221748	0.298037033
NES	11	14	0.029318815	0.394928767	0.961131615	0.158221748	0.298037033
NLRP2	11	14	0.029318815	0.394928767	0.961131615	0.158221748	0.298037033

PARD3B	11	14	0.029318815	0.394928767	0.961131615	0.158221748	0.298037033
ABCA10	14	16	0.030041782	0.438290761	0.986153988	0.19259082	0.304216207
COL5A1	14	16	0.030041782	0.438290761	0.986153988	0.19259082	0.304216207
PKHD1	32	29	0.030295083	0.537209501	0.959979472	0.301507676	0.305204876
FAM47A	22	22	0.03031263	0.494083046	0.968896872	0.251859887	0.305204876
UNC13C	22	22	0.03031263	0.494083046	0.968896872	0.251859887	0.305204876
HAUS7	9	0	0.030918888	Inf	Inf	1.055718223	0.309160946
MEX3B	9	0	0.030918888	Inf	Inf	1.055718223	0.309160946
SERPINB1(	9	0	0.030918888	Inf	Inf	1.055718223	0.309160946
KCNU1	18	19	0.030939489	0.471336703	0.979563137	0.225947924	0.309160946
ACAN	15	17	0.0328783	0.44052907	0.965916315	0.198916888	0.326068843
OR5L1	15	17	0.0328783	0.44052907	0.965916315	0.198916888	0.326068843
PCDHA2	15	17	0.0328783	0.44052907	0.965916315	0.198916888	0.326068843
ZEB2	15	17	0.0328783	0.44052907	0.965916315	0.198916888	0.326068843
TLR4	28	26	0.03318566	0.528187931	0.975180159	0.286807074	0.328500744
ADAM2	9	12	0.034092802	0.37940513	1.004662781	0.138018003	0.331276748
COL20A1	9	12	0.034092802	0.37940513	1.004662781	0.138018003	0.331276748
CPXM2	9	12	0.034092802	0.37940513	1.004662781	0.138018003	0.331276748
GRIK4	9	12	0.034092802	0.37940513	1.004662781	0.138018003	0.331276748
OR5D16	9	12	0.034092802	0.37940513	1.004662781	0.138018003	0.331276748
PCSK5	9	12	0.034092802	0.37940513	1.004662781	0.138018003	0.331276748
?11-407N17	9	12	0.034092802	0.37940513	1.004662781	0.138018003	0.331276748
SLC6A5	9	12	0.034092802	0.37940513	1.004662781	0.138018003	0.331276748
UGT2B11	9	12	0.034092802	0.37940513	1.004662781	0.138018003	0.331276748
YLPM1	9	12	0.034092802	0.37940513	1.004662781	0.138018003	0.331276748
GPR158	24	23	0.035759522	0.515464812	0.990998592	0.268578461	0.335949072
ACIN1	3	7	0.036734499	0.219716731	0.977895824	0.036190983	0.335949072
ACVRL1	3	7	0.036734499	0.219716731	0.977895824	0.036190983	0.335949072
ANO1	3	7	0.036734499	0.219716731	0.977895824	0.036190983	0.335949072
BTBD11	3	7	0.036734499	0.219716731	0.977895824	0.036190983	0.335949072
C1orf141	3	7	0.036734499	0.219716731	0.977895824	0.036190983	0.335949072
CAND1	3	7	0.036734499	0.219716731	0.977895824	0.036190983	0.335949072
CLVS2	3	7	0.036734499	0.219716731	0.977895824	0.036190983	0.335949072
DOCK1	3	7	0.036734499	0.219716731	0.977895824	0.036190983	0.335949072
GDF5	3	7	0.036734499	0.219716731	0.977895824	0.036190983	0.335949072
GIMAP8	3	7	0.036734499	0.219716731	0.977895824	0.036190983	0.335949072
HPX	3	7	0.036734499	0.219716731	0.977895824	0.036190983	0.335949072
KDM6A	3	7	0.036734499	0.219716731	0.977895824	0.036190983	0.335949072
KIAA0319L	3	7	0.036734499	0.219716731	0.977895824	0.036190983	0.335949072
KIAA1217	3	7	0.036734499	0.219716731	0.977895824	0.036190983	0.335949072
KRT72	3	7	0.036734499	0.219716731	0.977895824	0.036190983	0.335949072
KRT74	3	7	0.036734499	0.219716731	0.977895824	0.036190983	0.335949072
LPP	3	7	0.036734499	0.219716731	0.977895824	0.036190983	0.335949072
MED16	3	7	0.036734499	0.219716731	0.977895824	0.036190983	0.335949072
MYLK3	3	7	0.036734499	0.219716731	0.977895824	0.036190983	0.335949072
NLGN3	3	7	0.036734499	0.219716731	0.977895824	0.036190983	0.335949072
NOX5	3	7	0.036734499	0.219716731	0.977895824	0.036190983	0.335949072
OR13A1	3	7	0.036734499	0.219716731	0.977895824	0.036190983	0.335949072
OR6A2	3	7	0.036734499	0.219716731	0.977895824	0.036190983	0.335949072
PDIA4	3	7	0.036734499	0.219716731	0.977895824	0.036190983	0.335949072
PNLIPRP2	3	7	0.036734499	0.219716731	0.977895824	0.036190983	0.335949072
RGS9	3	7	0.036734499	0.219716731	0.977895824	0.036190983	0.335949072
RNF150	3	7	0.036734499	0.219716731	0.977895824	0.036190983	0.335949072
SHOX2	3	7	0.036734499	0.219716731	0.977895824	0.036190983	0.335949072
SLC27A3	3	7	0.036734499	0.219716731	0.977895824	0.036190983	0.335949072
STOX1	3	7	0.036734499	0.219716731	0.977895824	0.036190983	0.335949072
SULT1B1	3	7	0.036734499	0.219716731	0.977895824	0.036190983	0.335949072
TRIM3	3	7	0.036734499	0.219716731	0.977895824	0.036190983	0.335949072

ZNF606	3	7	0.036734499	0.219716731	0.977895824	0.036190983	0.335949072
PKHD1L1	30	27	0.037719349	0.54483656	0.99175055	0.300382005	0.344360066
SEMA6D	16	17	0.038321554	0.47135684	1.02236365	0.21627461	0.348653591
ZNF423	16	17	0.038321554	0.47135684	1.02236365	0.21627461	0.348653591
DNAH11	25	24	0.038487761	0.512796749	0.973266626	0.270584844	0.348841057
LAMA1	25	24	0.038487761	0.512796749	0.973266626	0.270584844	0.348841057
ADAD1	7	10	0.039266067	0.356366814	1.059394762	0.112877302	0.348841057
BNC1	7	10	0.039266067	0.356366814	1.059394762	0.112877302	0.348841057
DNMT3A	7	10	0.039266067	0.356366814	1.059394762	0.112877302	0.348841057
MAATS1	7	10	0.039266067	0.356366814	1.059394762	0.112877302	0.348841057
MC5R	7	10	0.039266067	0.356366814	1.059394762	0.112877302	0.348841057
NOS2	7	10	0.039266067	0.356366814	1.059394762	0.112877302	0.348841057
PTPN13	7	10	0.039266067	0.356366814	1.059394762	0.112877302	0.348841057
SERPINA3	7	10	0.039266067	0.356366814	1.059394762	0.112877302	0.348841057
SGCZ	7	10	0.039266067	0.356366814	1.059394762	0.112877302	0.348841057
SLC5A12	7	10	0.039266067	0.356366814	1.059394762	0.112877302	0.348841057
TMTC3	7	10	0.039266067	0.356366814	1.059394762	0.112877302	0.348841057
VWA3B	7	10	0.039266067	0.356366814	1.059394762	0.112877302	0.348841057
ANO4	13	15	0.039486271	0.435527809	1.00859299	0.185603033	0.349037503
NAV2	13	15	0.039486271	0.435527809	1.00859299	0.185603033	0.349037503
OR4A5	13	15	0.039486271	0.435527809	1.00859299	0.185603033	0.349037503
LRRTM4	21	21	0.040463343	0.49576472	0.987203896	0.248888161	0.354714122
SLITRK1	21	21	0.040463343	0.49576472	0.987203896	0.248888161	0.354714122
TRPA1	21	21	0.040463343	0.49576472	0.987203896	0.248888161	0.354714122
CYP11B1	10	13	0.040531088	0.387890616	0.981730561	0.148656203	0.354714122
OR2T3	10	13	0.040531088	0.387890616	0.981730561	0.148656203	0.354714122
TECRL	10	13	0.040531088	0.387890616	0.981730561	0.148656203	0.354714122
LARGE	12	1	0.040695272	6.52633054	280.9523272	0.950338876	0.35556233
ANK3	17	18	0.04111711	0.471442601	0.999938965	0.221350501	0.356474232
CFAP47	17	18	0.04111711	0.471442601	0.999938965	0.221350501	0.356474232
IGSF1	17	18	0.04111711	0.471442601	0.999938965	0.221350501	0.356474232
GRIN2B	26	24	0.041368996	0.535054596	1.010549006	0.284200625	0.356474232
COL3A1	27	25	0.043966222	0.53155913	0.992243748	0.285595617	0.356474232
DOCK2	27	25	0.043966222	0.53155913	0.992243748	0.285595617	0.356474232
APOB	48	38	0.045689287	0.60915071	1.008714246	0.369504003	0.356474232
C9	8	11	0.045920795	0.369080219	1.030350187	0.126173567	0.356474232
CDKN2A	8	11	0.045920795	0.369080219	1.030350187	0.126173567	0.356474232
CFHR4	8	11	0.045920795	0.369080219	1.030350187	0.126173567	0.356474232
MROH7	8	11	0.045920795	0.369080219	1.030350187	0.126173567	0.356474232
UMOD	8	11	0.045920795	0.369080219	1.030350187	0.126173567	0.356474232
ZNF33A	8	11	0.045920795	0.369080219	1.030350187	0.126173567	0.356474232
MYT1L	18	18	0.046991229	0.500743266	1.052681448	0.238136588	0.356474232
SCN3A	18	18	0.046991229	0.500743266	1.052681448	0.238136588	0.356474232
HYDIN	28	25	0.047091682	0.553067694	1.027931748	0.298856133	0.356474232
ADAM23	11	13	0.047494606	0.427953636	1.061829022	0.1693303	0.356474232
CHD8	11	13	0.047494606	0.427953636	1.061829022	0.1693303	0.356474232
KCNC2	11	13	0.047494606	0.427953636	1.061829022	0.1693303	0.356474232
NIPBL	11	13	0.047494606	0.427953636	1.061829022	0.1693303	0.356474232
OR6K2	11	13	0.047494606	0.427953636	1.061829022	0.1693303	0.356474232
PCDH7	11	13	0.047494606	0.427953636	1.061829022	0.1693303	0.356474232
PCDHB9	11	13	0.047494606	0.427953636	1.061829022	0.1693303	0.356474232
TLN2	11	13	0.047494606	0.427953636	1.061829022	0.1693303	0.356474232
TPR	11	13	0.047494606	0.427953636	1.061829022	0.1693303	0.356474232
AHNAK	29	26	0.049518467	0.548867146	1.009209633	0.299672187	0.356474232
CFH	15	16	0.050187871	0.471045602	1.047198226	0.21072551	0.356474232
DNAH10	15	16	0.050187871	0.471045602	1.047198226	0.21072551	0.356474232
GRIK2	15	16	0.050187871	0.471045602	1.047198226	0.21072551	0.356474232
MYO7B	15	16	0.050187871	0.471045602	1.047198226	0.21072551	0.356474232

ATP10A	6	9	0.050873789	0.340471577	1.092571596	0.097916305	0.356474232
EPHA4	6	9	0.050873789	0.340471577	1.092571596	0.097916305	0.356474232
FER	6	9	0.050873789	0.340471577	1.092571596	0.097916305	0.356474232
FRMD7	6	9	0.050873789	0.340471577	1.092571596	0.097916305	0.356474232
GLP2R	6	9	0.050873789	0.340471577	1.092571596	0.097916305	0.356474232
GRIA2	6	9	0.050873789	0.340471577	1.092571596	0.097916305	0.356474232
KANSL1	6	9	0.050873789	0.340471577	1.092571596	0.097916305	0.356474232
KDM5C	6	9	0.050873789	0.340471577	1.092571596	0.097916305	0.356474232
MNDA	6	9	0.050873789	0.340471577	1.092571596	0.097916305	0.356474232
NR5A2	6	9	0.050873789	0.340471577	1.092571596	0.097916305	0.356474232
NTM	6	9	0.050873789	0.340471577	1.092571596	0.097916305	0.356474232
OR5B3	6	9	0.050873789	0.340471577	1.092571596	0.097916305	0.356474232
PCDH9	6	9	0.050873789	0.340471577	1.092571596	0.097916305	0.356474232
PCDHA13	6	9	0.050873789	0.340471577	1.092571596	0.097916305	0.356474232
PLXND1	6	9	0.050873789	0.340471577	1.092571596	0.097916305	0.356474232
POLR3A	6	9	0.050873789	0.340471577	1.092571596	0.097916305	0.356474232
PRAMEF18	6	9	0.050873789	0.340471577	1.092571596	0.097916305	0.356474232
RAB11FIP1	6	9	0.050873789	0.340471577	1.092571596	0.097916305	0.356474232
SIM1	6	9	0.050873789	0.340471577	1.092571596	0.097916305	0.356474232
SLC9A9	6	9	0.050873789	0.340471577	1.092571596	0.097916305	0.356474232
TCF20	6	9	0.050873789	0.340471577	1.092571596	0.097916305	0.356474232
TIMD4	6	9	0.050873789	0.340471577	1.092571596	0.097916305	0.356474232
TMPRSS11	6	9	0.050873789	0.340471577	1.092571596	0.097916305	0.356474232
TNS3	6	9	0.050873789	0.340471577	1.092571596	0.097916305	0.356474232
TTC17	6	9	0.050873789	0.340471577	1.092571596	0.097916305	0.356474232
ACAP3	2	5	0.051427294	0.20695515	1.280531209	0.019514653	0.356474232
ADGRA2	2	5	0.051427294	0.20695515	1.280531209	0.019514653	0.356474232
AGAP2	2	5	0.051427294	0.20695515	1.280531209	0.019514653	0.356474232
AGAP3	2	5	0.051427294	0.20695515	1.280531209	0.019514653	0.356474232
ALG10B	2	5	0.051427294	0.20695515	1.280531209	0.019514653	0.356474232
ALS2CL	2	5	0.051427294	0.20695515	1.280531209	0.019514653	0.356474232
ANKRD1	2	5	0.051427294	0.20695515	1.280531209	0.019514653	0.356474232
ATAD3B	2	5	0.051427294	0.20695515	1.280531209	0.019514653	0.356474232
ATRIP	2	5	0.051427294	0.20695515	1.280531209	0.019514653	0.356474232
C1GALT1	2	5	0.051427294	0.20695515	1.280531209	0.019514653	0.356474232
CAPSL	2	5	0.051427294	0.20695515	1.280531209	0.019514653	0.356474232
CDH16	2	5	0.051427294	0.20695515	1.280531209	0.019514653	0.356474232
CLEC18B	2	5	0.051427294	0.20695515	1.280531209	0.019514653	0.356474232
CNOT2	2	5	0.051427294	0.20695515	1.280531209	0.019514653	0.356474232
CORO2B	2	5	0.051427294	0.20695515	1.280531209	0.019514653	0.356474232
CTPS1	2	5	0.051427294	0.20695515	1.280531209	0.019514653	0.356474232
DDC	2	5	0.051427294	0.20695515	1.280531209	0.019514653	0.356474232
DOK5	2	5	0.051427294	0.20695515	1.280531209	0.019514653	0.356474232
EIF3A	2	5	0.051427294	0.20695515	1.280531209	0.019514653	0.356474232
EIF3B	2	5	0.051427294	0.20695515	1.280531209	0.019514653	0.356474232
ELMOD1	2	5	0.051427294	0.20695515	1.280531209	0.019514653	0.356474232
ELP4	2	5	0.051427294	0.20695515	1.280531209	0.019514653	0.356474232
ERBB2IP	2	5	0.051427294	0.20695515	1.280531209	0.019514653	0.356474232
EXTL1	2	5	0.051427294	0.20695515	1.280531209	0.019514653	0.356474232
FGF6	2	5	0.051427294	0.20695515	1.280531209	0.019514653	0.356474232
GMPS	2	5	0.051427294	0.20695515	1.280531209	0.019514653	0.356474232
GPR17	2	5	0.051427294	0.20695515	1.280531209	0.019514653	0.356474232
GRIA1	2	5	0.051427294	0.20695515	1.280531209	0.019514653	0.356474232
GRM2	2	5	0.051427294	0.20695515	1.280531209	0.019514653	0.356474232
ICAM5	2	5	0.051427294	0.20695515	1.280531209	0.019514653	0.356474232
IL5RA	2	5	0.051427294	0.20695515	1.280531209	0.019514653	0.356474232
KPNB1	2	5	0.051427294	0.20695515	1.280531209	0.019514653	0.356474232
KRT24	2	5	0.051427294	0.20695515	1.280531209	0.019514653	0.356474232

KRT33B	2	5	0.051427294	0.20695515	1.280531209	0.019514653	0.356474232
KRT83	2	5	0.051427294	0.20695515	1.280531209	0.019514653	0.356474232
KY	2	5	0.051427294	0.20695515	1.280531209	0.019514653	0.356474232
MAP2K7	2	5	0.051427294	0.20695515	1.280531209	0.019514653	0.356474232
MAP3K1	2	5	0.051427294	0.20695515	1.280531209	0.019514653	0.356474232
MCTP1	2	5	0.051427294	0.20695515	1.280531209	0.019514653	0.356474232
MCTP2	2	5	0.051427294	0.20695515	1.280531209	0.019514653	0.356474232
MFN2	2	5	0.051427294	0.20695515	1.280531209	0.019514653	0.356474232
MFSD6	2	5	0.051427294	0.20695515	1.280531209	0.019514653	0.356474232
MICU3	2	5	0.051427294	0.20695515	1.280531209	0.019514653	0.356474232
MYH10	2	5	0.051427294	0.20695515	1.280531209	0.019514653	0.356474232
NMUR1	2	5	0.051427294	0.20695515	1.280531209	0.019514653	0.356474232
OLAH	2	5	0.051427294	0.20695515	1.280531209	0.019514653	0.356474232
OR2AG1	2	5	0.051427294	0.20695515	1.280531209	0.019514653	0.356474232
OSBPL5	2	5	0.051427294	0.20695515	1.280531209	0.019514653	0.356474232
PAX7	2	5	0.051427294	0.20695515	1.280531209	0.019514653	0.356474232
PPEF1	2	5	0.051427294	0.20695515	1.280531209	0.019514653	0.356474232
PROL1	2	5	0.051427294	0.20695515	1.280531209	0.019514653	0.356474232
PSD4	2	5	0.051427294	0.20695515	1.280531209	0.019514653	0.356474232
PTCRA	2	5	0.051427294	0.20695515	1.280531209	0.019514653	0.356474232
RAP1GDS1	2	5	0.051427294	0.20695515	1.280531209	0.019514653	0.356474232
RFPL3	2	5	0.051427294	0.20695515	1.280531209	0.019514653	0.356474232
RGS3	2	5	0.051427294	0.20695515	1.280531209	0.019514653	0.356474232
RNASE11	2	5	0.051427294	0.20695515	1.280531209	0.019514653	0.356474232
SH3RF1	2	5	0.051427294	0.20695515	1.280531209	0.019514653	0.356474232
SLC15A1	2	5	0.051427294	0.20695515	1.280531209	0.019514653	0.356474232
SLC26A11	2	5	0.051427294	0.20695515	1.280531209	0.019514653	0.356474232
SLC38A8	2	5	0.051427294	0.20695515	1.280531209	0.019514653	0.356474232
SLC6A7	2	5	0.051427294	0.20695515	1.280531209	0.019514653	0.356474232
SOBP	2	5	0.051427294	0.20695515	1.280531209	0.019514653	0.356474232
SPOCD1	2	5	0.051427294	0.20695515	1.280531209	0.019514653	0.356474232
STIM2	2	5	0.051427294	0.20695515	1.280531209	0.019514653	0.356474232
SUN5	2	5	0.051427294	0.20695515	1.280531209	0.019514653	0.356474232
TBC1D8	2	5	0.051427294	0.20695515	1.280531209	0.019514653	0.356474232
TLR5	2	5	0.051427294	0.20695515	1.280531209	0.019514653	0.356474232
TMCC3	2	5	0.051427294	0.20695515	1.280531209	0.019514653	0.356474232
TNKS	2	5	0.051427294	0.20695515	1.280531209	0.019514653	0.356474232
TRAM1	2	5	0.051427294	0.20695515	1.280531209	0.019514653	0.356474232
TRIM60	2	5	0.051427294	0.20695515	1.280531209	0.019514653	0.356474232
TRIM69	2	5	0.051427294	0.20695515	1.280531209	0.019514653	0.356474232
TRIM71	2	5	0.051427294	0.20695515	1.280531209	0.019514653	0.356474232
TRIM8	2	5	0.051427294	0.20695515	1.280531209	0.019514653	0.356474232
UBE4A	2	5	0.051427294	0.20695515	1.280531209	0.019514653	0.356474232
VAX1	2	5	0.051427294	0.20695515	1.280531209	0.019514653	0.356474232
VGF	2	5	0.051427294	0.20695515	1.280531209	0.019514653	0.356474232
WDR3	2	5	0.051427294	0.20695515	1.280531209	0.019514653	0.356474232
WDR60	2	5	0.051427294	0.20695515	1.280531209	0.019514653	0.356474232
WDR70	2	5	0.051427294	0.20695515	1.280531209	0.019514653	0.356474232
WNK4	2	5	0.051427294	0.20695515	1.280531209	0.019514653	0.356474232
XRN2	2	5	0.051427294	0.20695515	1.280531209	0.019514653	0.356474232
YARS2	2	5	0.051427294	0.20695515	1.280531209	0.019514653	0.356474232
ZNF133	2	5	0.051427294	0.20695515	1.280531209	0.019514653	0.356474232
ZNF141	2	5	0.051427294	0.20695515	1.280531209	0.019514653	0.356474232
ZNF264	2	5	0.051427294	0.20695515	1.280531209	0.019514653	0.356474232
ZNF488	2	5	0.051427294	0.20695515	1.280531209	0.019514653	0.356474232
KIF2B	24	22	0.051740309	0.542492999	1.051620874	0.280949527	0.356474232
SI	44	35	0.052973536	0.611104108	1.030319661	0.364311346	0.356474232
ACSS3	4	7	0.053851258	0.293692769	1.174841551	0.062141452	0.356474232

ANLN	4	7	0.053851258	0.293692769	1.174841551	0.062141452	0.356474232
BUB1B	4	7	0.053851258	0.293692769	1.174841551	0.062141452	0.356474232
C11orf30	4	7	0.053851258	0.293692769	1.174841551	0.062141452	0.356474232
CAPN13	4	7	0.053851258	0.293692769	1.174841551	0.062141452	0.356474232
CCDC144A	4	7	0.053851258	0.293692769	1.174841551	0.062141452	0.356474232
CD93	4	7	0.053851258	0.293692769	1.174841551	0.062141452	0.356474232
CRISP2	4	7	0.053851258	0.293692769	1.174841551	0.062141452	0.356474232
CSRP2BP	4	7	0.053851258	0.293692769	1.174841551	0.062141452	0.356474232
CYP4F8	4	7	0.053851258	0.293692769	1.174841551	0.062141452	0.356474232
DHX57	4	7	0.053851258	0.293692769	1.174841551	0.062141452	0.356474232
DSG2	4	7	0.053851258	0.293692769	1.174841551	0.062141452	0.356474232
ELF1	4	7	0.053851258	0.293692769	1.174841551	0.062141452	0.356474232
ERC1	4	7	0.053851258	0.293692769	1.174841551	0.062141452	0.356474232
FSCN3	4	7	0.053851258	0.293692769	1.174841551	0.062141452	0.356474232
HAVCR1	4	7	0.053851258	0.293692769	1.174841551	0.062141452	0.356474232
IQUB	4	7	0.053851258	0.293692769	1.174841551	0.062141452	0.356474232
KAT6B	4	7	0.053851258	0.293692769	1.174841551	0.062141452	0.356474232
KIAA0430	4	7	0.053851258	0.293692769	1.174841551	0.062141452	0.356474232
KRT3	4	7	0.053851258	0.293692769	1.174841551	0.062141452	0.356474232
MGAT5	4	7	0.053851258	0.293692769	1.174841551	0.062141452	0.356474232
MYEF2	4	7	0.053851258	0.293692769	1.174841551	0.062141452	0.356474232
NKX2-2	4	7	0.053851258	0.293692769	1.174841551	0.062141452	0.356474232
OR51F1	4	7	0.053851258	0.293692769	1.174841551	0.062141452	0.356474232
OSBPL1A	4	7	0.053851258	0.293692769	1.174841551	0.062141452	0.356474232
PCOLCE2	4	7	0.053851258	0.293692769	1.174841551	0.062141452	0.356474232
PNLIPRP3	4	7	0.053851258	0.293692769	1.174841551	0.062141452	0.356474232
PTGIS	4	7	0.053851258	0.293692769	1.174841551	0.062141452	0.356474232
SEC24D	4	7	0.053851258	0.293692769	1.174841551	0.062141452	0.356474232
SERPINB2	4	7	0.053851258	0.293692769	1.174841551	0.062141452	0.356474232
SPAG9	4	7	0.053851258	0.293692769	1.174841551	0.062141452	0.356474232
SPG11	4	7	0.053851258	0.293692769	1.174841551	0.062141452	0.356474232
SYMPK	4	7	0.053851258	0.293692769	1.174841551	0.062141452	0.356474232
TET2	4	7	0.053851258	0.293692769	1.174841551	0.062141452	0.356474232
THADA	4	7	0.053851258	0.293692769	1.174841551	0.062141452	0.356474232
TIE1	4	7	0.053851258	0.293692769	1.174841551	0.062141452	0.356474232
TMEM168	4	7	0.053851258	0.293692769	1.174841551	0.062141452	0.356474232
TRIP11	4	7	0.053851258	0.293692769	1.174841551	0.062141452	0.356474232
TSC2	4	7	0.053851258	0.293692769	1.174841551	0.062141452	0.356474232
USP8	4	7	0.053851258	0.293692769	1.174841551	0.062141452	0.356474232
WASF3	4	7	0.053851258	0.293692769	1.174841551	0.062141452	0.356474232
ZC3H3	4	7	0.053851258	0.293692769	1.174841551	0.062141452	0.356474232
ZNF287	4	7	0.053851258	0.293692769	1.174841551	0.062141452	0.356474232
ZNF33B	4	7	0.053851258	0.293692769	1.174841551	0.062141452	0.356474232
ZNF41	4	7	0.053851258	0.293692769	1.174841551	0.062141452	0.356474232
ZNF862	4	7	0.053851258	0.293692769	1.174841551	0.062141452	0.356474232
ATP2B3	12	14	0.054084815	0.432130108	1.033598679	0.177906047	0.356474232
C7	12	14	0.054084815	0.432130108	1.033598679	0.177906047	0.356474232
FRYL	12	14	0.054084815	0.432130108	1.033598679	0.177906047	0.356474232
NLGN1	12	14	0.054084815	0.432130108	1.033598679	0.177906047	0.356474232
OR5L2	12	14	0.054084815	0.432130108	1.033598679	0.177906047	0.356474232
TMEM200A	12	14	0.054084815	0.432130108	1.033598679	0.177906047	0.356474232
OR1M1	8	0	0.055416125	Inf	Inf	0.910520371	0.363436275
RBMX	8	0	0.055416125	Inf	Inf	0.910520371	0.363436275
SYT13	8	0	0.055416125	Inf	Inf	0.910520371	0.363436275
VANGL1	8	0	0.055416125	Inf	Inf	0.910520371	0.363436275
ATP1A4	9	11	0.057171022	0.416426135	1.131068691	0.149174287	0.368993924
BCL11B	9	11	0.057171022	0.416426135	1.131068691	0.149174287	0.368993924
CNKSR2	9	11	0.057171022	0.416426135	1.131068691	0.149174287	0.368993924

EPG5	9	11	0.057171022	0.416426135	1.131068691	0.149174287	0.368993924
GALNT8	9	11	0.057171022	0.416426135	1.131068691	0.149174287	0.368993924
KCND2	9	11	0.057171022	0.416426135	1.131068691	0.149174287	0.368993924
LRRTM1	9	11	0.057171022	0.416426135	1.131068691	0.149174287	0.368993924
MED13L	9	11	0.057171022	0.416426135	1.131068691	0.149174287	0.368993924
MICAL3	9	11	0.057171022	0.416426135	1.131068691	0.149174287	0.368993924
RNF213	9	11	0.057171022	0.416426135	1.131068691	0.149174287	0.368993924
SEMA5B	9	11	0.057171022	0.416426135	1.131068691	0.149174287	0.368993924
SPTBN5	9	11	0.057171022	0.416426135	1.131068691	0.149174287	0.368993924
USP26	9	11	0.057171022	0.416426135	1.131068691	0.149174287	0.368993924
PRKDC	21	20	0.058318533	0.523975668	1.053516671	0.261247265	0.375941175
DNAH3	26	23	0.058950151	0.562074075	1.069788694	0.296872136	0.379549934
ASPM	33	28	0.060715435	0.579743712	1.038271469	0.32540344	0.389912481
ADAMTS2(	27	24	0.061165431	0.557461065	1.048065775	0.297926788	0.389912481
MUC5B	27	24	0.061165431	0.557461065	1.048065775	0.297926788	0.389912481
CCDC178	17	17	0.061171216	0.502380988	1.079130332	0.233832513	0.389912481
DMD	40	32	0.06118241	0.612322884	1.054309403	0.357670634	0.389912481
ADAMTS1(	13	14	0.061518541	0.469562955	1.106377802	0.197862873	0.389912481
CAMTA1	13	14	0.061518541	0.469562955	1.106377802	0.197862873	0.389912481
EGFLAM	13	14	0.061518541	0.469562955	1.106377802	0.197862873	0.389912481
GLI2	13	14	0.061518541	0.469562955	1.106377802	0.197862873	0.389912481
LAMB1	13	14	0.061518541	0.469562955	1.106377802	0.197862873	0.389912481
MAGI2	13	14	0.061518541	0.469562955	1.106377802	0.197862873	0.389912481
OR2T4	13	14	0.061518541	0.469562955	1.106377802	0.197862873	0.389912481
UBA6	13	14	0.061518541	0.469562955	1.106377802	0.197862873	0.389912481
ABCA5	10	12	0.063979098	0.422806309	1.09395937	0.159826448	0.394131387
AMPH	10	12	0.063979098	0.422806309	1.09395937	0.159826448	0.394131387
CACNA2D4	10	12	0.063979098	0.422806309	1.09395937	0.159826448	0.394131387
COBL	10	12	0.063979098	0.422806309	1.09395937	0.159826448	0.394131387
GUCY1A2	10	12	0.063979098	0.422806309	1.09395937	0.159826448	0.394131387
ICE1	10	12	0.063979098	0.422806309	1.09395937	0.159826448	0.394131387
LEPR	10	12	0.063979098	0.422806309	1.09395937	0.159826448	0.394131387
LVRN	10	12	0.063979098	0.422806309	1.09395937	0.159826448	0.394131387
PLXNA2	10	12	0.063979098	0.422806309	1.09395937	0.159826448	0.394131387
WBSCR17	10	12	0.063979098	0.422806309	1.09395937	0.159826448	0.394131387
FLG	73	52	0.064080846	0.665818603	1.035256732	0.429642416	0.394131387
LRP2	34	28	0.064558254	0.599347213	1.070031856	0.337769395	0.394131387
ELMO1	14	2	0.0650848	3.810307389	34.94211485	0.859225407	0.394131387
ADAMTS12	51	39	0.065455562	0.632724671	1.039320369	0.387079227	0.394131387
DAB2	11	1	0.065584152	5.963746567	258.5106212	0.85337188	0.394131387
JAK3	11	1	0.065584152	5.963746567	258.5106212	0.85337188	0.394131387
PPL	11	1	0.065584152	5.963746567	258.5106212	0.85337188	0.394131387
FERD3L	15	2	0.065807665	4.095463151	37.29742951	0.934847139	0.394131387
UNC79	28	24	0.066195907	0.580023486	1.085824311	0.311748484	0.394131387
PTPRZ1	23	21	0.067060393	0.5464821	1.074830935	0.27907719	0.394131387
TEX15	23	21	0.067060393	0.5464821	1.074830935	0.27907719	0.394131387
ADGRL4	14	15	0.067940246	0.470466022	1.075019023	0.204614267	0.394131387
CHD6	14	15	0.067940246	0.470466022	1.075019023	0.204614267	0.394131387
ABRA	3	6	0.070004767	0.257820618	1.225412571	0.041200164	0.394131387
ACVR1B	3	6	0.070004767	0.257820618	1.225412571	0.041200164	0.394131387
ANGEL1	3	6	0.070004767	0.257820618	1.225412571	0.041200164	0.394131387
C3AR1	3	6	0.070004767	0.257820618	1.225412571	0.041200164	0.394131387
C8orf34	3	6	0.070004767	0.257820618	1.225412571	0.041200164	0.394131387
CCDC102B	3	6	0.070004767	0.257820618	1.225412571	0.041200164	0.394131387
CEP85L	3	6	0.070004767	0.257820618	1.225412571	0.041200164	0.394131387
CFTR	3	6	0.070004767	0.257820618	1.225412571	0.041200164	0.394131387
CLIP1	3	6	0.070004767	0.257820618	1.225412571	0.041200164	0.394131387
CNTNAP1	3	6	0.070004767	0.257820618	1.225412571	0.041200164	0.394131387

CRHBP	3	6	0.070004767	0.257820618	1.225412571	0.041200164	0.394131387
CXorf67	3	6	0.070004767	0.257820618	1.225412571	0.041200164	0.394131387
CYP4B1	3	6	0.070004767	0.257820618	1.225412571	0.041200164	0.394131387
DCAF8	3	6	0.070004767	0.257820618	1.225412571	0.041200164	0.394131387
DENND1B	3	6	0.070004767	0.257820618	1.225412571	0.041200164	0.394131387
DYRK4	3	6	0.070004767	0.257820618	1.225412571	0.041200164	0.394131387
ERG	3	6	0.070004767	0.257820618	1.225412571	0.041200164	0.394131387
ESF1	3	6	0.070004767	0.257820618	1.225412571	0.041200164	0.394131387
FCGR3B	3	6	0.070004767	0.257820618	1.225412571	0.041200164	0.394131387
FNDC3B	3	6	0.070004767	0.257820618	1.225412571	0.041200164	0.394131387
GFPT1	3	6	0.070004767	0.257820618	1.225412571	0.041200164	0.394131387
GLIS1	3	6	0.070004767	0.257820618	1.225412571	0.041200164	0.394131387
HAO1	3	6	0.070004767	0.257820618	1.225412571	0.041200164	0.394131387
HIPK3	3	6	0.070004767	0.257820618	1.225412571	0.041200164	0.394131387
HP1BP3	3	6	0.070004767	0.257820618	1.225412571	0.041200164	0.394131387
ITGB4	3	6	0.070004767	0.257820618	1.225412571	0.041200164	0.394131387
KDM4D	3	6	0.070004767	0.257820618	1.225412571	0.041200164	0.394131387
KLK6	3	6	0.070004767	0.257820618	1.225412571	0.041200164	0.394131387
LGR4	3	6	0.070004767	0.257820618	1.225412571	0.041200164	0.394131387
MAGEA11	3	6	0.070004767	0.257820618	1.225412571	0.041200164	0.394131387
MAGI3	3	6	0.070004767	0.257820618	1.225412571	0.041200164	0.394131387
MAML3	3	6	0.070004767	0.257820618	1.225412571	0.041200164	0.394131387
7-Mar	3	6	0.070004767	0.257820618	1.225412571	0.041200164	0.394131387
MOV10L1	3	6	0.070004767	0.257820618	1.225412571	0.041200164	0.394131387
MTRR	3	6	0.070004767	0.257820618	1.225412571	0.041200164	0.394131387
NBEAL2	3	6	0.070004767	0.257820618	1.225412571	0.041200164	0.394131387
NDN	3	6	0.070004767	0.257820618	1.225412571	0.041200164	0.394131387
NEK11	3	6	0.070004767	0.257820618	1.225412571	0.041200164	0.394131387
NT5C1A	3	6	0.070004767	0.257820618	1.225412571	0.041200164	0.394131387
OR1A1	3	6	0.070004767	0.257820618	1.225412571	0.041200164	0.394131387
OR4D2	3	6	0.070004767	0.257820618	1.225412571	0.041200164	0.394131387
OR4X2	3	6	0.070004767	0.257820618	1.225412571	0.041200164	0.394131387
OR8D2	3	6	0.070004767	0.257820618	1.225412571	0.041200164	0.394131387
PARP14	3	6	0.070004767	0.257820618	1.225412571	0.041200164	0.394131387
PHKB	3	6	0.070004767	0.257820618	1.225412571	0.041200164	0.394131387
PLK4	3	6	0.070004767	0.257820618	1.225412571	0.041200164	0.394131387
PNLIPRP1	3	6	0.070004767	0.257820618	1.225412571	0.041200164	0.394131387
PRDM15	3	6	0.070004767	0.257820618	1.225412571	0.041200164	0.394131387
PRKCA	3	6	0.070004767	0.257820618	1.225412571	0.041200164	0.394131387
SGCD	3	6	0.070004767	0.257820618	1.225412571	0.041200164	0.394131387
SLC2A9	3	6	0.070004767	0.257820618	1.225412571	0.041200164	0.394131387
SLX4	3	6	0.070004767	0.257820618	1.225412571	0.041200164	0.394131387
SORBS2	3	6	0.070004767	0.257820618	1.225412571	0.041200164	0.394131387
SOX30	3	6	0.070004767	0.257820618	1.225412571	0.041200164	0.394131387
SULT6B1	3	6	0.070004767	0.257820618	1.225412571	0.041200164	0.394131387
T	3	6	0.070004767	0.257820618	1.225412571	0.041200164	0.394131387
TAAR9	3	6	0.070004767	0.257820618	1.225412571	0.041200164	0.394131387
TBATA	3	6	0.070004767	0.257820618	1.225412571	0.041200164	0.394131387
TDRD9	3	6	0.070004767	0.257820618	1.225412571	0.041200164	0.394131387
TTC7B	3	6	0.070004767	0.257820618	1.225412571	0.041200164	0.394131387
U2SURP	3	6	0.070004767	0.257820618	1.225412571	0.041200164	0.394131387
YTHDC1	3	6	0.070004767	0.257820618	1.225412571	0.041200164	0.394131387
ZBTB16	3	6	0.070004767	0.257820618	1.225412571	0.041200164	0.394131387
ZMAT4	3	6	0.070004767	0.257820618	1.225412571	0.041200164	0.394131387
ZNF202	3	6	0.070004767	0.257820618	1.225412571	0.041200164	0.394131387
ZNF256	3	6	0.070004767	0.257820618	1.225412571	0.041200164	0.394131387
ZNF300	3	6	0.070004767	0.257820618	1.225412571	0.041200164	0.394131387
ACKR1	5	8	0.07090786	0.320204077	1.130718999	0.081084903	0.394131387

ADAM22	5	8	0.07090786	0.320204077	1.130718999	0.081084903	0.394131387
AKAP4	5	8	0.07090786	0.320204077	1.130718999	0.081084903	0.394131387
COPA	5	8	0.07090786	0.320204077	1.130718999	0.081084903	0.394131387
CYP4F2	5	8	0.07090786	0.320204077	1.130718999	0.081084903	0.394131387
DEPDC5	5	8	0.07090786	0.320204077	1.130718999	0.081084903	0.394131387
FGD6	5	8	0.07090786	0.320204077	1.130718999	0.081084903	0.394131387
GLI1	5	8	0.07090786	0.320204077	1.130718999	0.081084903	0.394131387
GPR37	5	8	0.07090786	0.320204077	1.130718999	0.081084903	0.394131387
IL21R	5	8	0.07090786	0.320204077	1.130718999	0.081084903	0.394131387
KIR3DL3	5	8	0.07090786	0.320204077	1.130718999	0.081084903	0.394131387
KYNU	5	8	0.07090786	0.320204077	1.130718999	0.081084903	0.394131387
LARP4B	5	8	0.07090786	0.320204077	1.130718999	0.081084903	0.394131387
LRRC16A	5	8	0.07090786	0.320204077	1.130718999	0.081084903	0.394131387
MCC	5	8	0.07090786	0.320204077	1.130718999	0.081084903	0.394131387
NNT	5	8	0.07090786	0.320204077	1.130718999	0.081084903	0.394131387
PAK3	5	8	0.07090786	0.320204077	1.130718999	0.081084903	0.394131387
PCDHGA7	5	8	0.07090786	0.320204077	1.130718999	0.081084903	0.394131387
PHF14	5	8	0.07090786	0.320204077	1.130718999	0.081084903	0.394131387
PRR16	5	8	0.07090786	0.320204077	1.130718999	0.081084903	0.394131387
RALGAPA1	5	8	0.07090786	0.320204077	1.130718999	0.081084903	0.394131387
RFWD2	5	8	0.07090786	0.320204077	1.130718999	0.081084903	0.394131387
SELE	5	8	0.07090786	0.320204077	1.130718999	0.081084903	0.394131387
SF1	5	8	0.07090786	0.320204077	1.130718999	0.081084903	0.394131387
TCERG1L	5	8	0.07090786	0.320204077	1.130718999	0.081084903	0.394131387
TEX13A	5	8	0.07090786	0.320204077	1.130718999	0.081084903	0.394131387
UGT2B7	5	8	0.07090786	0.320204077	1.130718999	0.081084903	0.394131387
FAT3	54	40	0.071961696	0.655413105	1.068621053	0.404073467	0.39956883
A2M	8	10	0.075440288	0.40844624	1.174672514	0.137185619	0.410063218
CCDC73	8	10	0.075440288	0.40844624	1.174672514	0.137185619	0.410063218
CDH19	8	10	0.075440288	0.40844624	1.174672514	0.137185619	0.410063218
COL27A1	8	10	0.075440288	0.40844624	1.174672514	0.137185619	0.410063218
COL28A1	8	10	0.075440288	0.40844624	1.174672514	0.137185619	0.410063218
GOLGA3	8	10	0.075440288	0.40844624	1.174672514	0.137185619	0.410063218
HDX	8	10	0.075440288	0.40844624	1.174672514	0.137185619	0.410063218
HEPHL1	8	10	0.075440288	0.40844624	1.174672514	0.137185619	0.410063218
HTR1A	8	10	0.075440288	0.40844624	1.174672514	0.137185619	0.410063218
KANK4	8	10	0.075440288	0.40844624	1.174672514	0.137185619	0.410063218
KIAA0100	8	10	0.075440288	0.40844624	1.174672514	0.137185619	0.410063218
LRRTM3	8	10	0.075440288	0.40844624	1.174672514	0.137185619	0.410063218
OR2A25	8	10	0.075440288	0.40844624	1.174672514	0.137185619	0.410063218
POTEC	8	10	0.075440288	0.40844624	1.174672514	0.137185619	0.410063218
PROX1	8	10	0.075440288	0.40844624	1.174672514	0.137185619	0.410063218
SIGLEC1	8	10	0.075440288	0.40844624	1.174672514	0.137185619	0.410063218
SOX5	8	10	0.075440288	0.40844624	1.174672514	0.137185619	0.410063218
TRPC7	8	10	0.075440288	0.40844624	1.174672514	0.137185619	0.410063218
GABRB3	11	12	0.075757791	0.466473678	1.183572304	0.18200842	0.410063218
HHIPL2	11	12	0.075757791	0.466473678	1.183572304	0.18200842	0.410063218
PMFBP1	11	12	0.075757791	0.466473678	1.183572304	0.18200842	0.410063218
SORL1	11	12	0.075757791	0.466473678	1.183572304	0.18200842	0.410063218
ZNF318	11	12	0.075757791	0.466473678	1.183572304	0.18200842	0.410063218
SEZ6L	15	15	0.075758266	0.505624384	1.141806387	0.22387424	0.410063218
ZFPM2	25	22	0.075791102	0.566938388	1.093368205	0.295723868	0.410063218
FCGBP	20	19	0.077818416	0.527032318	1.078308384	0.25830564	0.420601375
MUC16	117	76	0.08057105	0.706432691	1.049896339	0.475457102	0.43503429
MUC5AC	16	16	0.081803187	0.504007992	1.108589419	0.22909679	0.440521478
DNAH17	12	13	0.08225401	0.468264253	1.142183345	0.190398466	0.440521478
GABRG2	12	13	0.08225401	0.468264253	1.142183345	0.190398466	0.440521478
GABRG3	12	13	0.08225401	0.468264253	1.142183345	0.190398466	0.440521478

HIVEP2	12	13	0.08225401	0.468264253	1.142183345	0.190398466	0.440521478
PDHA2	12	13	0.08225401	0.468264253	1.142183345	0.190398466	0.440521478
USP34	12	13	0.08225401	0.468264253	1.142183345	0.190398466	0.440521478
ZIC4	12	13	0.08225401	0.468264253	1.142183345	0.190398466	0.440521478
GRM5	21	19	0.083298469	0.555151754	1.128137203	0.27472392	0.444763342
MYH4	21	19	0.083298469	0.555151754	1.128137203	0.27472392	0.444763342
ROBO2	21	19	0.083298469	0.555151754	1.128137203	0.27472392	0.444763342
AASS	6	8	0.088234422	0.385301908	1.2905867	0.108250477	0.452136233
ARFGEF1	6	8	0.088234422	0.385301908	1.2905867	0.108250477	0.452136233
CALCRL	6	8	0.088234422	0.385301908	1.2905867	0.108250477	0.452136233
CHSY3	6	8	0.088234422	0.385301908	1.2905867	0.108250477	0.452136233
DKK2	6	8	0.088234422	0.385301908	1.2905867	0.108250477	0.452136233
DLK1	6	8	0.088234422	0.385301908	1.2905867	0.108250477	0.452136233
DLX5	6	8	0.088234422	0.385301908	1.2905867	0.108250477	0.452136233
DSC1	6	8	0.088234422	0.385301908	1.2905867	0.108250477	0.452136233
GPR32	6	8	0.088234422	0.385301908	1.2905867	0.108250477	0.452136233
GUCY1A3	6	8	0.088234422	0.385301908	1.2905867	0.108250477	0.452136233
HECTD1	6	8	0.088234422	0.385301908	1.2905867	0.108250477	0.452136233
HERC4	6	8	0.088234422	0.385301908	1.2905867	0.108250477	0.452136233
HS3ST5	6	8	0.088234422	0.385301908	1.2905867	0.108250477	0.452136233
JAKMIP2	6	8	0.088234422	0.385301908	1.2905867	0.108250477	0.452136233
KNDC1	6	8	0.088234422	0.385301908	1.2905867	0.108250477	0.452136233
LARP1	6	8	0.088234422	0.385301908	1.2905867	0.108250477	0.452136233
11-Mar	6	8	0.088234422	0.385301908	1.2905867	0.108250477	0.452136233
MYO10	6	8	0.088234422	0.385301908	1.2905867	0.108250477	0.452136233
OFD1	6	8	0.088234422	0.385301908	1.2905867	0.108250477	0.452136233
OR10K2	6	8	0.088234422	0.385301908	1.2905867	0.108250477	0.452136233
OR5I1	6	8	0.088234422	0.385301908	1.2905867	0.108250477	0.452136233
OR5R1	6	8	0.088234422	0.385301908	1.2905867	0.108250477	0.452136233
PNPLA6	6	8	0.088234422	0.385301908	1.2905867	0.108250477	0.452136233
POU6F2	6	8	0.088234422	0.385301908	1.2905867	0.108250477	0.452136233
PSG5	6	8	0.088234422	0.385301908	1.2905867	0.108250477	0.452136233
SLC2A10	6	8	0.088234422	0.385301908	1.2905867	0.108250477	0.452136233
TAS2R16	6	8	0.088234422	0.385301908	1.2905867	0.108250477	0.452136233
ZFP64	6	8	0.088234422	0.385301908	1.2905867	0.108250477	0.452136233
STK11	43	33	0.088246811	0.640424571	1.090707232	0.378427399	0.452136233
CDH7	22	20	0.08844723	0.550689561	1.100225221	0.277023276	0.452136233
COL19A1	22	20	0.08844723	0.550689561	1.100225221	0.277023276	0.452136233
MROH2B	22	20	0.08844723	0.550689561	1.100225221	0.277023276	0.452136233
SALL1	22	20	0.08844723	0.550689561	1.100225221	0.277023276	0.452136233
DCHS1	17	16	0.089943302	0.537180618	1.17029242	0.2476156	0.452136233
KIAA1211	17	16	0.089943302	0.537180618	1.17029242	0.2476156	0.452136233
MED12	17	16	0.089943302	0.537180618	1.17029242	0.2476156	0.452136233
PCDHGA3	17	16	0.089943302	0.537180618	1.17029242	0.2476156	0.452136233
ALK	13	13	0.094353945	0.508825745	1.222920557	0.211669279	0.452136233
BSN	13	13	0.094353945	0.508825745	1.222920557	0.211669279	0.452136233
CMTR2	13	13	0.094353945	0.508825745	1.222920557	0.211669279	0.452136233
DPP6	13	13	0.094353945	0.508825745	1.222920557	0.211669279	0.452136233
KCNA4	13	13	0.094353945	0.508825745	1.222920557	0.211669279	0.452136233
OR4C46	13	13	0.094353945	0.508825745	1.222920557	0.211669279	0.452136233
PLG	13	13	0.094353945	0.508825745	1.222920557	0.211669279	0.452136233
PYHIN1	13	13	0.094353945	0.508825745	1.222920557	0.211669279	0.452136233
TRIO	13	13	0.094353945	0.508825745	1.222920557	0.211669279	0.452136233
DYNC1H1	22	5	0.094638584	2.416282347	8.320626532	0.871179573	0.452136233
SNTG1	30	25	0.097014447	0.596531242	1.10000443	0.325699309	0.452136233
ABCG1	6	0	0.09771631	Inf	Inf	0.624916671	0.452136233
ADAM17	6	0	0.09771631	Inf	Inf	0.624916671	0.452136233
AURKC	6	0	0.09771631	Inf	Inf	0.624916671	0.452136233

C4orf50	6	0	0.09771631	Inf	Inf	0.624916671	0.452136233
CARD8	6	0	0.09771631	Inf	Inf	0.624916671	0.452136233
CHRM4	6	0	0.09771631	Inf	Inf	0.624916671	0.452136233
CLEC1B	6	0	0.09771631	Inf	Inf	0.624916671	0.452136233
CP	6	0	0.09771631	Inf	Inf	0.624916671	0.452136233
CXorf57	6	0	0.09771631	Inf	Inf	0.624916671	0.452136233
DDX53	6	0	0.09771631	Inf	Inf	0.624916671	0.452136233
DHX38	6	0	0.09771631	Inf	Inf	0.624916671	0.452136233
ELF3	6	0	0.09771631	Inf	Inf	0.624916671	0.452136233
FOXJ3	6	0	0.09771631	Inf	Inf	0.624916671	0.452136233
GARS	6	0	0.09771631	Inf	Inf	0.624916671	0.452136233
HECTD3	6	0	0.09771631	Inf	Inf	0.624916671	0.452136233
HTR2C	6	0	0.09771631	Inf	Inf	0.624916671	0.452136233
IPO13	6	0	0.09771631	Inf	Inf	0.624916671	0.452136233
IRX4	6	0	0.09771631	Inf	Inf	0.624916671	0.452136233
KCNK16	6	0	0.09771631	Inf	Inf	0.624916671	0.452136233
KIRREL3	6	0	0.09771631	Inf	Inf	0.624916671	0.452136233
(RTAP10-1	6	0	0.09771631	Inf	Inf	0.624916671	0.452136233
MRAP2	6	0	0.09771631	Inf	Inf	0.624916671	0.452136233
NEDD4L	6	0	0.09771631	Inf	Inf	0.624916671	0.452136233
OR6C68	6	0	0.09771631	Inf	Inf	0.624916671	0.452136233
ORC1	6	0	0.09771631	Inf	Inf	0.624916671	0.452136233
QRICH2	6	0	0.09771631	Inf	Inf	0.624916671	0.452136233
RNASE12	6	0	0.09771631	Inf	Inf	0.624916671	0.452136233
RUFY3	6	0	0.09771631	Inf	Inf	0.624916671	0.452136233
SH3KBP1	6	0	0.09771631	Inf	Inf	0.624916671	0.452136233
SLC28A1	6	0	0.09771631	Inf	Inf	0.624916671	0.452136233
SPAG1	6	0	0.09771631	Inf	Inf	0.624916671	0.452136233
SPP2	6	0	0.09771631	Inf	Inf	0.624916671	0.452136233
SYNDIG1	6	0	0.09771631	Inf	Inf	0.624916671	0.452136233
TCF7L2	6	0	0.09771631	Inf	Inf	0.624916671	0.452136233
TNFAIP3	6	0	0.09771631	Inf	Inf	0.624916671	0.452136233
ZNF154	6	0	0.09771631	Inf	Inf	0.624916671	0.452136233
FMN2	24	21	0.098899285	0.572091677	1.119056353	0.294386829	0.452136233
JMJD1C	10	11	0.099956361	0.464059958	1.232157431	0.172630433	0.452136233
MYT1	10	11	0.099956361	0.464059958	1.232157431	0.172630433	0.452136233
OR2T11	10	11	0.099956361	0.464059958	1.232157431	0.172630433	0.452136233
PCDHGB3	10	11	0.099956361	0.464059958	1.232157431	0.172630433	0.452136233
PCK1	10	11	0.099956361	0.464059958	1.232157431	0.172630433	0.452136233
TNRC6A	10	11	0.099956361	0.464059958	1.232157431	0.172630433	0.452136233
TRPV6	10	11	0.099956361	0.464059958	1.232157431	0.172630433	0.452136233
ZNF99	10	11	0.099956361	0.464059958	1.232157431	0.172630433	0.452136233
FREM2	14	14	0.100273414	0.507230267	1.179533876	0.218081293	0.452136233
HIVEP3	14	14	0.100273414	0.507230267	1.179533876	0.218081293	0.452136233
OR2L13	14	14	0.100273414	0.507230267	1.179533876	0.218081293	0.452136233
OR4C16	14	14	0.100273414	0.507230267	1.179533876	0.218081293	0.452136233
PCDHB2	14	14	0.100273414	0.507230267	1.179533876	0.218081293	0.452136233
SPAG17	14	14	0.100273414	0.507230267	1.179533876	0.218081293	0.452136233
ABCC1	4	6	0.101270709	0.344623417	1.476374926	0.070523959	0.452136233
ABCC3	4	6	0.101270709	0.344623417	1.476374926	0.070523959	0.452136233
ACTL9	4	6	0.101270709	0.344623417	1.476374926	0.070523959	0.452136233
ACTRT2	4	6	0.101270709	0.344623417	1.476374926	0.070523959	0.452136233
AGO3	4	6	0.101270709	0.344623417	1.476374926	0.070523959	0.452136233
APOBR	4	6	0.101270709	0.344623417	1.476374926	0.070523959	0.452136233
ARHGAP3(	4	6	0.101270709	0.344623417	1.476374926	0.070523959	0.452136233
ATP2C1	4	6	0.101270709	0.344623417	1.476374926	0.070523959	0.452136233
BAHCC1	4	6	0.101270709	0.344623417	1.476374926	0.070523959	0.452136233
CAPRIN2	4	6	0.101270709	0.344623417	1.476374926	0.070523959	0.452136233

CCDC85A	4	6	0.101270709	0.344623417	1.476374926	0.070523959	0.452136233
CD96	4	6	0.101270709	0.344623417	1.476374926	0.070523959	0.452136233
CDH15	4	6	0.101270709	0.344623417	1.476374926	0.070523959	0.452136233
CFAP44	4	6	0.101270709	0.344623417	1.476374926	0.070523959	0.452136233
CHST2	4	6	0.101270709	0.344623417	1.476374926	0.070523959	0.452136233
COL6A1	4	6	0.101270709	0.344623417	1.476374926	0.070523959	0.452136233
CPT1C	4	6	0.101270709	0.344623417	1.476374926	0.070523959	0.452136233
DAPK1	4	6	0.101270709	0.344623417	1.476374926	0.070523959	0.452136233
DHX37	4	6	0.101270709	0.344623417	1.476374926	0.070523959	0.452136233
EHMT1	4	6	0.101270709	0.344623417	1.476374926	0.070523959	0.452136233
ELSPBP1	4	6	0.101270709	0.344623417	1.476374926	0.070523959	0.452136233
EXTL3	4	6	0.101270709	0.344623417	1.476374926	0.070523959	0.452136233
FAM91A1	4	6	0.101270709	0.344623417	1.476374926	0.070523959	0.452136233
FBXO24	4	6	0.101270709	0.344623417	1.476374926	0.070523959	0.452136233
FGF23	4	6	0.101270709	0.344623417	1.476374926	0.070523959	0.452136233
GANAB	4	6	0.101270709	0.344623417	1.476374926	0.070523959	0.452136233
GC	4	6	0.101270709	0.344623417	1.476374926	0.070523959	0.452136233
GRIP1	4	6	0.101270709	0.344623417	1.476374926	0.070523959	0.452136233
HDAC6	4	6	0.101270709	0.344623417	1.476374926	0.070523959	0.452136233
HTR6	4	6	0.101270709	0.344623417	1.476374926	0.070523959	0.452136233
GHV1OR15-	4	6	0.101270709	0.344623417	1.476374926	0.070523959	0.452136233
INTS2	4	6	0.101270709	0.344623417	1.476374926	0.070523959	0.452136233
IRF6	4	6	0.101270709	0.344623417	1.476374926	0.070523959	0.452136233
KDELC1	4	6	0.101270709	0.344623417	1.476374926	0.070523959	0.452136233
KIAA0226L	4	6	0.101270709	0.344623417	1.476374926	0.070523959	0.452136233
KIF5C	4	6	0.101270709	0.344623417	1.476374926	0.070523959	0.452136233
KIN	4	6	0.101270709	0.344623417	1.476374926	0.070523959	0.452136233
KLK2	4	6	0.101270709	0.344623417	1.476374926	0.070523959	0.452136233
KRT1	4	6	0.101270709	0.344623417	1.476374926	0.070523959	0.452136233
MAGEB3	4	6	0.101270709	0.344623417	1.476374926	0.070523959	0.452136233
MAGED1	4	6	0.101270709	0.344623417	1.476374926	0.070523959	0.452136233
4-Mar	4	6	0.101270709	0.344623417	1.476374926	0.070523959	0.452136233
MDM1	4	6	0.101270709	0.344623417	1.476374926	0.070523959	0.452136233
MEIS1	4	6	0.101270709	0.344623417	1.476374926	0.070523959	0.452136233
MROH5	4	6	0.101270709	0.344623417	1.476374926	0.070523959	0.452136233
MYF5	4	6	0.101270709	0.344623417	1.476374926	0.070523959	0.452136233
NXPE4	4	6	0.101270709	0.344623417	1.476374926	0.070523959	0.452136233
OR10V1	4	6	0.101270709	0.344623417	1.476374926	0.070523959	0.452136233
OR2T8	4	6	0.101270709	0.344623417	1.476374926	0.070523959	0.452136233
OR51G1	4	6	0.101270709	0.344623417	1.476374926	0.070523959	0.452136233
OR5B17	4	6	0.101270709	0.344623417	1.476374926	0.070523959	0.452136233
OR5H15	4	6	0.101270709	0.344623417	1.476374926	0.070523959	0.452136233
PCDH12	4	6	0.101270709	0.344623417	1.476374926	0.070523959	0.452136233
PLEKHM1	4	6	0.101270709	0.344623417	1.476374926	0.070523959	0.452136233
PPP1R21	4	6	0.101270709	0.344623417	1.476374926	0.070523959	0.452136233
PRAMEF20	4	6	0.101270709	0.344623417	1.476374926	0.070523959	0.452136233
PRDM11	4	6	0.101270709	0.344623417	1.476374926	0.070523959	0.452136233
PTPN5	4	6	0.101270709	0.344623417	1.476374926	0.070523959	0.452136233
RPH3A	4	6	0.101270709	0.344623417	1.476374926	0.070523959	0.452136233
RUFY2	4	6	0.101270709	0.344623417	1.476374926	0.070523959	0.452136233
SAMD7	4	6	0.101270709	0.344623417	1.476374926	0.070523959	0.452136233
SAXO2	4	6	0.101270709	0.344623417	1.476374926	0.070523959	0.452136233
SERPINA12	4	6	0.101270709	0.344623417	1.476374926	0.070523959	0.452136233
SETDB2	4	6	0.101270709	0.344623417	1.476374926	0.070523959	0.452136233
SLC24A4	4	6	0.101270709	0.344623417	1.476374926	0.070523959	0.452136233
SLC4A11	4	6	0.101270709	0.344623417	1.476374926	0.070523959	0.452136233
SMPD4	4	6	0.101270709	0.344623417	1.476374926	0.070523959	0.452136233
ST8SIA5	4	6	0.101270709	0.344623417	1.476374926	0.070523959	0.452136233

SVOP	4	6	0.101270709	0.344623417	1.476374926	0.070523959	0.452136233
SYDE2	4	6	0.101270709	0.344623417	1.476374926	0.070523959	0.452136233
TBC1D19	4	6	0.101270709	0.344623417	1.476374926	0.070523959	0.452136233
TDRD7	4	6	0.101270709	0.344623417	1.476374926	0.070523959	0.452136233
TMEFF1	4	6	0.101270709	0.344623417	1.476374926	0.070523959	0.452136233
TPX2	4	6	0.101270709	0.344623417	1.476374926	0.070523959	0.452136233
TTLL4	4	6	0.101270709	0.344623417	1.476374926	0.070523959	0.452136233
TWISTNB	4	6	0.101270709	0.344623417	1.476374926	0.070523959	0.452136233
UBASH3A	4	6	0.101270709	0.344623417	1.476374926	0.070523959	0.452136233
USP48	4	6	0.101270709	0.344623417	1.476374926	0.070523959	0.452136233
VNN3	4	6	0.101270709	0.344623417	1.476374926	0.070523959	0.452136233
ZNF217	4	6	0.101270709	0.344623417	1.476374926	0.070523959	0.452136233
ZNF568	4	6	0.101270709	0.344623417	1.476374926	0.070523959	0.452136233
ZNF675	4	6	0.101270709	0.344623417	1.476374926	0.070523959	0.452136233
ZNF808	4	6	0.101270709	0.344623417	1.476374926	0.070523959	0.452136233
ARHGEF9	7	0	0.101700715	Inf	Inf	0.766852531	0.452136233
ASB11	7	0	0.101700715	Inf	Inf	0.766852531	0.452136233
CCDC67	7	0	0.101700715	Inf	Inf	0.766852531	0.452136233
CKAP2	7	0	0.101700715	Inf	Inf	0.766852531	0.452136233
CMPK2	7	0	0.101700715	Inf	Inf	0.766852531	0.452136233
FAN1	7	0	0.101700715	Inf	Inf	0.766852531	0.452136233
GAL3ST1	7	0	0.101700715	Inf	Inf	0.766852531	0.452136233
KRT71	7	0	0.101700715	Inf	Inf	0.766852531	0.452136233
KRTAP21-1	7	0	0.101700715	Inf	Inf	0.766852531	0.452136233
MECOM	7	0	0.101700715	Inf	Inf	0.766852531	0.452136233
NAA15	7	0	0.101700715	Inf	Inf	0.766852531	0.452136233
NKD2	7	0	0.101700715	Inf	Inf	0.766852531	0.452136233
PDE5A	7	0	0.101700715	Inf	Inf	0.766852531	0.452136233
SMURF1	7	0	0.101700715	Inf	Inf	0.766852531	0.452136233
TRIM56	7	0	0.101700715	Inf	Inf	0.766852531	0.452136233
ZACN	7	0	0.101700715	Inf	Inf	0.766852531	0.452136233
ZNF493	7	0	0.101700715	Inf	Inf	0.766852531	0.452136233
NLRP5	19	17	0.103897862	0.56502847	1.193522408	0.269430008	0.459547771
ST6GAL2	19	17	0.103897862	0.56502847	1.193522408	0.269430008	0.459547771
ADGRV1	25	21	0.104941512	0.597882517	1.163530373	0.309786383	0.459547771
CELSR1	7	9	0.104988666	0.398336582	1.226789625	0.123642654	0.459547771
CT47B1	7	9	0.104988666	0.398336582	1.226789625	0.123642654	0.459547771
GFRA1	7	9	0.104988666	0.398336582	1.226789625	0.123642654	0.459547771
IMPG2	7	9	0.104988666	0.398336582	1.226789625	0.123642654	0.459547771
LAMB3	7	9	0.104988666	0.398336582	1.226789625	0.123642654	0.459547771
LMX1A	7	9	0.104988666	0.398336582	1.226789625	0.123642654	0.459547771
LRBA	7	9	0.104988666	0.398336582	1.226789625	0.123642654	0.459547771
MAN2A1	7	9	0.104988666	0.398336582	1.226789625	0.123642654	0.459547771
MYO7A	7	9	0.104988666	0.398336582	1.226789625	0.123642654	0.459547771
OR10K1	7	9	0.104988666	0.398336582	1.226789625	0.123642654	0.459547771
OR8K1	7	9	0.104988666	0.398336582	1.226789625	0.123642654	0.459547771
RET	7	9	0.104988666	0.398336582	1.226789625	0.123642654	0.459547771
SCN9A	7	9	0.104988666	0.398336582	1.226789625	0.123642654	0.459547771
SPAG16	7	9	0.104988666	0.398336582	1.226789625	0.123642654	0.459547771
TMPRSS11A	7	9	0.104988666	0.398336582	1.226789625	0.123642654	0.459547771
COL6A3	32	26	0.105019619	0.611763861	1.112670775	0.338810356	0.459547771
CAMK4	10	1	0.10710907	5.404757822	236.208281	0.757276516	0.467144015
KCNF1	10	1	0.10710907	5.404757822	236.208281	0.757276516	0.467144015
NT5C1B	10	1	0.10710907	5.404757822	236.208281	0.757276516	0.467144015
PRAME	10	1	0.10710907	5.404757822	236.208281	0.757276516	0.467144015
FBN2	41	31	0.108545181	0.654796817	1.130142446	0.382193631	0.473017168
ERBB4	20	18	0.1088572	0.559913106	1.159044281	0.272203939	0.473986128
SLC8A1	26	22	0.109129526	0.591555999	1.135379208	0.31053764	0.474390357

SPHKAP	26	22	0.109129526	0.591555999	1.135379208	0.31053764	0.474390357
SSPO	33	26	0.109688201	0.633016609	1.147605428	0.352072717	0.476427139
CHRM2	15	14	0.112567144	0.54513532	1.252987971	0.238549707	0.486931199
PLCB4	15	14	0.112567144	0.54513532	1.252987971	0.238549707	0.486931199
SDK1	15	14	0.112567144	0.54513532	1.252987971	0.238549707	0.486931199
TMEM132D	15	14	0.112567144	0.54513532	1.252987971	0.238549707	0.486931199
UBR5	15	14	0.112567144	0.54513532	1.252987971	0.238549707	0.486931199
LRRC7	44	33	0.116954876	0.657636258	1.117779451	0.389594111	0.492553345
DNAH5	35	27	0.117770582	0.646468918	1.157610069	0.364091384	0.492553345
AMER3	16	15	0.117772247	0.541005844	1.208871934	0.243320367	0.492553345
CUX2	16	15	0.117772247	0.541005844	1.208871934	0.243320367	0.492553345
FLNA	16	15	0.117772247	0.541005844	1.208871934	0.243320367	0.492553345
KALRN	16	15	0.117772247	0.541005844	1.208871934	0.243320367	0.492553345
TRHDE	16	15	0.117772247	0.541005844	1.208871934	0.243320367	0.492553345
BLK	5	7	0.120334666	0.368092204	1.371032904	0.090655975	0.492553345
CADM2	5	7	0.120334666	0.368092204	1.371032904	0.090655975	0.492553345
CARD6	5	7	0.120334666	0.368092204	1.371032904	0.090655975	0.492553345
CCKAR	5	7	0.120334666	0.368092204	1.371032904	0.090655975	0.492553345
CFAP52	5	7	0.120334666	0.368092204	1.371032904	0.090655975	0.492553345
COL6A5	5	7	0.120334666	0.368092204	1.371032904	0.090655975	0.492553345
DZIP1L	5	7	0.120334666	0.368092204	1.371032904	0.090655975	0.492553345
EFCAB6	5	7	0.120334666	0.368092204	1.371032904	0.090655975	0.492553345
FLRT3	5	7	0.120334666	0.368092204	1.371032904	0.090655975	0.492553345
FOCAD	5	7	0.120334666	0.368092204	1.371032904	0.090655975	0.492553345
GRM4	5	7	0.120334666	0.368092204	1.371032904	0.090655975	0.492553345
IGKV1D-17	5	7	0.120334666	0.368092204	1.371032904	0.090655975	0.492553345
ITK	5	7	0.120334666	0.368092204	1.371032904	0.090655975	0.492553345
JAG1	5	7	0.120334666	0.368092204	1.371032904	0.090655975	0.492553345
KIAA1522	5	7	0.120334666	0.368092204	1.371032904	0.090655975	0.492553345
KRT79	5	7	0.120334666	0.368092204	1.371032904	0.090655975	0.492553345
LRRC16B	5	7	0.120334666	0.368092204	1.371032904	0.090655975	0.492553345
LRRC4B	5	7	0.120334666	0.368092204	1.371032904	0.090655975	0.492553345
LRRCC1	5	7	0.120334666	0.368092204	1.371032904	0.090655975	0.492553345
LRTM2	5	7	0.120334666	0.368092204	1.371032904	0.090655975	0.492553345
LZTR1	5	7	0.120334666	0.368092204	1.371032904	0.090655975	0.492553345
MC3R	5	7	0.120334666	0.368092204	1.371032904	0.090655975	0.492553345
MUC6	5	7	0.120334666	0.368092204	1.371032904	0.090655975	0.492553345
NIPAL4	5	7	0.120334666	0.368092204	1.371032904	0.090655975	0.492553345
NR1H4	5	7	0.120334666	0.368092204	1.371032904	0.090655975	0.492553345
OR2A5	5	7	0.120334666	0.368092204	1.371032904	0.090655975	0.492553345
PIGG	5	7	0.120334666	0.368092204	1.371032904	0.090655975	0.492553345
PKD2L1	5	7	0.120334666	0.368092204	1.371032904	0.090655975	0.492553345
PRSS55	5	7	0.120334666	0.368092204	1.371032904	0.090655975	0.492553345
PTPN4	5	7	0.120334666	0.368092204	1.371032904	0.090655975	0.492553345
PTPRF	5	7	0.120334666	0.368092204	1.371032904	0.090655975	0.492553345
SERPINA5	5	7	0.120334666	0.368092204	1.371032904	0.090655975	0.492553345
SFSWAP	5	7	0.120334666	0.368092204	1.371032904	0.090655975	0.492553345
SLC22A25	5	7	0.120334666	0.368092204	1.371032904	0.090655975	0.492553345
SLC9A2	5	7	0.120334666	0.368092204	1.371032904	0.090655975	0.492553345
TAAR6	5	7	0.120334666	0.368092204	1.371032904	0.090655975	0.492553345
TBXAS1	5	7	0.120334666	0.368092204	1.371032904	0.090655975	0.492553345
THSD1	5	7	0.120334666	0.368092204	1.371032904	0.090655975	0.492553345
TRAF3IP3	5	7	0.120334666	0.368092204	1.371032904	0.090655975	0.492553345
ZC3H12B	5	7	0.120334666	0.368092204	1.371032904	0.090655975	0.492553345
ZFAT	5	7	0.120334666	0.368092204	1.371032904	0.090655975	0.492553345
ZNF658	5	7	0.120334666	0.368092204	1.371032904	0.090655975	0.492553345
ARHGAP35	8	9	0.121922476	0.456545977	1.361236357	0.150136001	0.492553345
ATP13A5	8	9	0.121922476	0.456545977	1.361236357	0.150136001	0.492553345

BCL9L	8	9	0.121922476	0.456545977	1.361236357	0.150136001	0.492553345
CDH4	8	9	0.121922476	0.456545977	1.361236357	0.150136001	0.492553345
CRISPLD1	8	9	0.121922476	0.456545977	1.361236357	0.150136001	0.492553345
DYTN	8	9	0.121922476	0.456545977	1.361236357	0.150136001	0.492553345
FASN	8	9	0.121922476	0.456545977	1.361236357	0.150136001	0.492553345
GABRA1	8	9	0.121922476	0.456545977	1.361236357	0.150136001	0.492553345
GRIK3	8	9	0.121922476	0.456545977	1.361236357	0.150136001	0.492553345
HDAC4	8	9	0.121922476	0.456545977	1.361236357	0.150136001	0.492553345
IQGAP3	8	9	0.121922476	0.456545977	1.361236357	0.150136001	0.492553345
MBD5	8	9	0.121922476	0.456545977	1.361236357	0.150136001	0.492553345
MCF2L2	8	9	0.121922476	0.456545977	1.361236357	0.150136001	0.492553345
NPAS3	8	9	0.121922476	0.456545977	1.361236357	0.150136001	0.492553345
OR5D18	8	9	0.121922476	0.456545977	1.361236357	0.150136001	0.492553345
PTPRK	8	9	0.121922476	0.456545977	1.361236357	0.150136001	0.492553345
REG3G	8	9	0.121922476	0.456545977	1.361236357	0.150136001	0.492553345
ROBO3	8	9	0.121922476	0.456545977	1.361236357	0.150136001	0.492553345
SERPINA9	8	9	0.121922476	0.456545977	1.361236357	0.150136001	0.492553345
SOS2	8	9	0.121922476	0.456545977	1.361236357	0.150136001	0.492553345
STAG3	8	9	0.121922476	0.456545977	1.361236357	0.150136001	0.492553345
SYCP2	8	9	0.121922476	0.456545977	1.361236357	0.150136001	0.492553345
SYT4	8	9	0.121922476	0.456545977	1.361236357	0.150136001	0.492553345
PTPRD	46	34	0.12282472	0.667214632	1.125074495	0.398422364	0.492553345
KIAA1755	12	12	0.123636578	0.510410918	1.273556174	0.204530491	0.492553345
PDE3A	12	12	0.123636578	0.510410918	1.273556174	0.204530491	0.492553345
PKD1	12	12	0.123636578	0.510410918	1.273556174	0.204530491	0.492553345
SPATA31A3	12	12	0.123636578	0.510410918	1.273556174	0.204530491	0.492553345
LPPR4	29	23	0.125650508	0.633184088	1.189850011	0.340180903	0.492553345
NLRP3	30	24	0.128816702	0.625607014	1.162017158	0.33974717	0.492553345
MAGEC1	23	19	0.130715672	0.611960624	1.228626146	0.307963334	0.492553345
NLGN4X	23	19	0.130715672	0.611960624	1.228626146	0.307963334	0.492553345
OR2T27	15	3	0.131764353	2.715298078	14.84017934	0.752796303	0.492553345
A4GNT	3	5	0.131850735	0.311142529	1.621581688	0.047735492	0.492553345
ADCK3	3	5	0.131850735	0.311142529	1.621581688	0.047735492	0.492553345
ALPP	3	5	0.131850735	0.311142529	1.621581688	0.047735492	0.492553345
ANKRD33	3	5	0.131850735	0.311142529	1.621581688	0.047735492	0.492553345
ARHGEF35	3	5	0.131850735	0.311142529	1.621581688	0.047735492	0.492553345
ASAP2	3	5	0.131850735	0.311142529	1.621581688	0.047735492	0.492553345
BTN2A2	3	5	0.131850735	0.311142529	1.621581688	0.047735492	0.492553345
CABIN1	3	5	0.131850735	0.311142529	1.621581688	0.047735492	0.492553345
CALCA	3	5	0.131850735	0.311142529	1.621581688	0.047735492	0.492553345
CASP1	3	5	0.131850735	0.311142529	1.621581688	0.047735492	0.492553345
CBL	3	5	0.131850735	0.311142529	1.621581688	0.047735492	0.492553345
CCAR1	3	5	0.131850735	0.311142529	1.621581688	0.047735492	0.492553345
CCDC70	3	5	0.131850735	0.311142529	1.621581688	0.047735492	0.492553345
CDH24	3	5	0.131850735	0.311142529	1.621581688	0.047735492	0.492553345
CSE1L	3	5	0.131850735	0.311142529	1.621581688	0.047735492	0.492553345
DAGLA	3	5	0.131850735	0.311142529	1.621581688	0.047735492	0.492553345
DCAF4L1	3	5	0.131850735	0.311142529	1.621581688	0.047735492	0.492553345
DHX40	3	5	0.131850735	0.311142529	1.621581688	0.047735492	0.492553345
DISP2	3	5	0.131850735	0.311142529	1.621581688	0.047735492	0.492553345
DRC7	3	5	0.131850735	0.311142529	1.621581688	0.047735492	0.492553345
DSE	3	5	0.131850735	0.311142529	1.621581688	0.047735492	0.492553345
EXOC8	3	5	0.131850735	0.311142529	1.621581688	0.047735492	0.492553345
FAM227B	3	5	0.131850735	0.311142529	1.621581688	0.047735492	0.492553345
FARP1	3	5	0.131850735	0.311142529	1.621581688	0.047735492	0.492553345
FCGR2B	3	5	0.131850735	0.311142529	1.621581688	0.047735492	0.492553345
GABRB2	3	5	0.131850735	0.311142529	1.621581688	0.047735492	0.492553345
GAPVD1	3	5	0.131850735	0.311142529	1.621581688	0.047735492	0.492553345

GDA	3	5	0.131850735	0.311142529	1.621581688	0.047735492	0.492553345
GPNMB	3	5	0.131850735	0.311142529	1.621581688	0.047735492	0.492553345
GSDMC	3	5	0.131850735	0.311142529	1.621581688	0.047735492	0.492553345
HIST1H1B	3	5	0.131850735	0.311142529	1.621581688	0.047735492	0.492553345
HMGCS2	3	5	0.131850735	0.311142529	1.621581688	0.047735492	0.492553345
HR	3	5	0.131850735	0.311142529	1.621581688	0.047735492	0.492553345
HSPA2	3	5	0.131850735	0.311142529	1.621581688	0.047735492	0.492553345
IBTK	3	5	0.131850735	0.311142529	1.621581688	0.047735492	0.492553345
IPO9	3	5	0.131850735	0.311142529	1.621581688	0.047735492	0.492553345
ITGB3	3	5	0.131850735	0.311142529	1.621581688	0.047735492	0.492553345
ITM2A	3	5	0.131850735	0.311142529	1.621581688	0.047735492	0.492553345
KANK1	3	5	0.131850735	0.311142529	1.621581688	0.047735492	0.492553345
KCNH4	3	5	0.131850735	0.311142529	1.621581688	0.047735492	0.492553345
KRTAP13-2	3	5	0.131850735	0.311142529	1.621581688	0.047735492	0.492553345
LCE2A	3	5	0.131850735	0.311142529	1.621581688	0.047735492	0.492553345
LRRC49	3	5	0.131850735	0.311142529	1.621581688	0.047735492	0.492553345
LSG1	3	5	0.131850735	0.311142529	1.621581688	0.047735492	0.492553345
LTF	3	5	0.131850735	0.311142529	1.621581688	0.047735492	0.492553345
MCF2L	3	5	0.131850735	0.311142529	1.621581688	0.047735492	0.492553345
MEIS2	3	5	0.131850735	0.311142529	1.621581688	0.047735492	0.492553345
MMP1	3	5	0.131850735	0.311142529	1.621581688	0.047735492	0.492553345
MMRN2	3	5	0.131850735	0.311142529	1.621581688	0.047735492	0.492553345
MS4A12	3	5	0.131850735	0.311142529	1.621581688	0.047735492	0.492553345
MSH2	3	5	0.131850735	0.311142529	1.621581688	0.047735492	0.492553345
MTBP	3	5	0.131850735	0.311142529	1.621581688	0.047735492	0.492553345
MYO19	3	5	0.131850735	0.311142529	1.621581688	0.047735492	0.492553345
NAP1L2	3	5	0.131850735	0.311142529	1.621581688	0.047735492	0.492553345
NFATC4	3	5	0.131850735	0.311142529	1.621581688	0.047735492	0.492553345
NLRC4	3	5	0.131850735	0.311142529	1.621581688	0.047735492	0.492553345
NPHS2	3	5	0.131850735	0.311142529	1.621581688	0.047735492	0.492553345
NRIP1	3	5	0.131850735	0.311142529	1.621581688	0.047735492	0.492553345
NTNG2	3	5	0.131850735	0.311142529	1.621581688	0.047735492	0.492553345
OLR1	3	5	0.131850735	0.311142529	1.621581688	0.047735492	0.492553345
OR10A5	3	5	0.131850735	0.311142529	1.621581688	0.047735492	0.492553345
OR1J4	3	5	0.131850735	0.311142529	1.621581688	0.047735492	0.492553345
OR3A2	3	5	0.131850735	0.311142529	1.621581688	0.047735492	0.492553345
OR52A1	3	5	0.131850735	0.311142529	1.621581688	0.047735492	0.492553345
OSBPL8	3	5	0.131850735	0.311142529	1.621581688	0.047735492	0.492553345
PHIP	3	5	0.131850735	0.311142529	1.621581688	0.047735492	0.492553345
PINX1	3	5	0.131850735	0.311142529	1.621581688	0.047735492	0.492553345
PIWIL4	3	5	0.131850735	0.311142529	1.621581688	0.047735492	0.492553345
PLCL2	3	5	0.131850735	0.311142529	1.621581688	0.047735492	0.492553345
PLEKHG2	3	5	0.131850735	0.311142529	1.621581688	0.047735492	0.492553345
PLOD3	3	5	0.131850735	0.311142529	1.621581688	0.047735492	0.492553345
PNN	3	5	0.131850735	0.311142529	1.621581688	0.047735492	0.492553345
PNPT1	3	5	0.131850735	0.311142529	1.621581688	0.047735492	0.492553345
PRF1	3	5	0.131850735	0.311142529	1.621581688	0.047735492	0.492553345
RBL2	3	5	0.131850735	0.311142529	1.621581688	0.047735492	0.492553345
RGL1	3	5	0.131850735	0.311142529	1.621581688	0.047735492	0.492553345
RNF112	3	5	0.131850735	0.311142529	1.621581688	0.047735492	0.492553345
ROPN1L	3	5	0.131850735	0.311142529	1.621581688	0.047735492	0.492553345
P11-385D13	3	5	0.131850735	0.311142529	1.621581688	0.047735492	0.492553345
S100A7	3	5	0.131850735	0.311142529	1.621581688	0.047735492	0.492553345
SEMA4F	3	5	0.131850735	0.311142529	1.621581688	0.047735492	0.492553345
SH3GL3	3	5	0.131850735	0.311142529	1.621581688	0.047735492	0.492553345
SKIL	3	5	0.131850735	0.311142529	1.621581688	0.047735492	0.492553345
SLC12A6	3	5	0.131850735	0.311142529	1.621581688	0.047735492	0.492553345
SLC28A2	3	5	0.131850735	0.311142529	1.621581688	0.047735492	0.492553345

SLC45A1	3	5	0.131850735	0.311142529	1.621581688	0.047735492	0.492553345
SLC6A1	3	5	0.131850735	0.311142529	1.621581688	0.047735492	0.492553345
SLC9B1	3	5	0.131850735	0.311142529	1.621581688	0.047735492	0.492553345
SPACA1	3	5	0.131850735	0.311142529	1.621581688	0.047735492	0.492553345
SPICE1	3	5	0.131850735	0.311142529	1.621581688	0.047735492	0.492553345
SRPX	3	5	0.131850735	0.311142529	1.621581688	0.047735492	0.492553345
SUPT5H	3	5	0.131850735	0.311142529	1.621581688	0.047735492	0.492553345
SYT3	3	5	0.131850735	0.311142529	1.621581688	0.047735492	0.492553345
TCERG1	3	5	0.131850735	0.311142529	1.621581688	0.047735492	0.492553345
TCN1	3	5	0.131850735	0.311142529	1.621581688	0.047735492	0.492553345
TEX26	3	5	0.131850735	0.311142529	1.621581688	0.047735492	0.492553345
TEX37	3	5	0.131850735	0.311142529	1.621581688	0.047735492	0.492553345
TTC21A	3	5	0.131850735	0.311142529	1.621581688	0.047735492	0.492553345
UBAP1	3	5	0.131850735	0.311142529	1.621581688	0.047735492	0.492553345
UBC	3	5	0.131850735	0.311142529	1.621581688	0.047735492	0.492553345
USP17L2	3	5	0.131850735	0.311142529	1.621581688	0.047735492	0.492553345
USP19	3	5	0.131850735	0.311142529	1.621581688	0.047735492	0.492553345
ZBED1	3	5	0.131850735	0.311142529	1.621581688	0.047735492	0.492553345
ZBTB33	3	5	0.131850735	0.311142529	1.621581688	0.047735492	0.492553345
ZC3H14	3	5	0.131850735	0.311142529	1.621581688	0.047735492	0.492553345
ZNF37A	3	5	0.131850735	0.311142529	1.621581688	0.047735492	0.492553345
ZNF415	3	5	0.131850735	0.311142529	1.621581688	0.047735492	0.492553345
ZNF43	3	5	0.131850735	0.311142529	1.621581688	0.047735492	0.492553345
ZNF624	3	5	0.131850735	0.311142529	1.621581688	0.047735492	0.492553345
ZNF645	3	5	0.131850735	0.311142529	1.621581688	0.047735492	0.492553345
ZNF724P	3	5	0.131850735	0.311142529	1.621581688	0.047735492	0.492553345
PAPPA2	49	35	0.133162239	0.692817034	1.157659861	0.417646026	0.496750596
TNR	49	35	0.133162239	0.692817034	1.157659861	0.417646026	0.496750596
AHNAK2	39	29	0.133370441	0.670582424	1.174693892	0.386116836	0.496826039
FAM135B	39	29	0.133370441	0.670582424	1.174693892	0.386116836	0.496826039
DCHS2	24	20	0.134426621	0.604659703	1.194450855	0.308930355	0.499704021
PCDH17	24	20	0.134426621	0.604659703	1.194450855	0.308930355	0.499704021
SPEF2	24	20	0.134426621	0.604659703	1.194450855	0.308930355	0.499704021
CNTN5	18	16	0.134610283	0.57057039	1.23230137	0.266380505	0.500035105
PRDM9	31	24	0.135588136	0.648629659	1.200531394	0.353915923	0.502994159
ABCA2	9	10	0.137119671	0.460839257	1.290238963	0.162106125	0.502994159
ADAMTS1	9	10	0.137119671	0.460839257	1.290238963	0.162106125	0.502994159
CD1E	9	10	0.137119671	0.460839257	1.290238963	0.162106125	0.502994159
CD5L	9	10	0.137119671	0.460839257	1.290238963	0.162106125	0.502994159
KIAA1551	9	10	0.137119671	0.460839257	1.290238963	0.162106125	0.502994159
LAMC1	9	10	0.137119671	0.460839257	1.290238963	0.162106125	0.502994159
MDC1	9	10	0.137119671	0.460839257	1.290238963	0.162106125	0.502994159
NCAM1	9	10	0.137119671	0.460839257	1.290238963	0.162106125	0.502994159
NSD1	9	10	0.137119671	0.460839257	1.290238963	0.162106125	0.502994159
OR11H12	9	10	0.137119671	0.460839257	1.290238963	0.162106125	0.502994159
OR8K5	9	10	0.137119671	0.460839257	1.290238963	0.162106125	0.502994159
PIK3R4	9	10	0.137119671	0.460839257	1.290238963	0.162106125	0.502994159
TGM6	9	10	0.137119671	0.460839257	1.290238963	0.162106125	0.502994159
UNC13A	9	10	0.137119671	0.460839257	1.290238963	0.162106125	0.502994159
VEGFC	9	10	0.137119671	0.460839257	1.290238963	0.162106125	0.502994159
ZNF107	9	10	0.137119671	0.460839257	1.290238963	0.162106125	0.502994159
ZNF648	9	10	0.137119671	0.460839257	1.290238963	0.162106125	0.502994159
PCDH10	32	25	0.138244583	0.640587514	1.173024779	0.35299135	0.506768978
BRCA2	14	13	0.146135419	0.549641796	1.304012584	0.233258197	0.52490902
CCNB3	14	13	0.146135419	0.549641796	1.304012584	0.233258197	0.52490902
CR1	14	13	0.146135419	0.549641796	1.304012584	0.233258197	0.52490902
FANCM	14	13	0.146135419	0.549641796	1.304012584	0.233258197	0.52490902
ITIH6	14	13	0.146135419	0.549641796	1.304012584	0.233258197	0.52490902

KCNH7	14	13	0.146135419	0.549641796	1.304012584	0.233258197	0.52490902
KIF4B	14	13	0.146135419	0.549641796	1.304012584	0.233258197	0.52490902
LRRIQ3	14	13	0.146135419	0.549641796	1.304012584	0.233258197	0.52490902
MTUS2	14	13	0.146135419	0.549641796	1.304012584	0.233258197	0.52490902
OR2M3	14	13	0.146135419	0.549641796	1.304012584	0.233258197	0.52490902
OR2M4	14	13	0.146135419	0.549641796	1.304012584	0.233258197	0.52490902
PCDHB3	14	13	0.146135419	0.549641796	1.304012584	0.233258197	0.52490902
WDR49	14	13	0.146135419	0.549641796	1.304012584	0.233258197	0.52490902
KIAA1109	26	21	0.146557614	0.623840248	1.208245256	0.325325544	0.52490902
TPTE	26	21	0.146557614	0.623840248	1.208245256	0.325325544	0.52490902
ABCC5	6	7	0.149250522	0.442919955	1.567095139	0.12084709	0.52490902
AKAP3	6	7	0.149250522	0.442919955	1.567095139	0.12084709	0.52490902
ANO2	6	7	0.149250522	0.442919955	1.567095139	0.12084709	0.52490902
ARMC4	6	7	0.149250522	0.442919955	1.567095139	0.12084709	0.52490902
CLEC14A	6	7	0.149250522	0.442919955	1.567095139	0.12084709	0.52490902
CTDP1	6	7	0.149250522	0.442919955	1.567095139	0.12084709	0.52490902
DMBX1	6	7	0.149250522	0.442919955	1.567095139	0.12084709	0.52490902
DNAJC10	6	7	0.149250522	0.442919955	1.567095139	0.12084709	0.52490902
EML5	6	7	0.149250522	0.442919955	1.567095139	0.12084709	0.52490902
ENPP1	6	7	0.149250522	0.442919955	1.567095139	0.12084709	0.52490902
GATA3	6	7	0.149250522	0.442919955	1.567095139	0.12084709	0.52490902
GDF2	6	7	0.149250522	0.442919955	1.567095139	0.12084709	0.52490902
IKZF1	6	7	0.149250522	0.442919955	1.567095139	0.12084709	0.52490902
IL9R	6	7	0.149250522	0.442919955	1.567095139	0.12084709	0.52490902
KCNK9	6	7	0.149250522	0.442919955	1.567095139	0.12084709	0.52490902
KLHL14	6	7	0.149250522	0.442919955	1.567095139	0.12084709	0.52490902
KRBA1	6	7	0.149250522	0.442919955	1.567095139	0.12084709	0.52490902
LAMB2	6	7	0.149250522	0.442919955	1.567095139	0.12084709	0.52490902
LPAR4	6	7	0.149250522	0.442919955	1.567095139	0.12084709	0.52490902
MEGF6	6	7	0.149250522	0.442919955	1.567095139	0.12084709	0.52490902
MMS22L	6	7	0.149250522	0.442919955	1.567095139	0.12084709	0.52490902
MUT	6	7	0.149250522	0.442919955	1.567095139	0.12084709	0.52490902
NAALADL[illegible]	6	7	0.149250522	0.442919955	1.567095139	0.12084709	0.52490902
NCKAP5L	6	7	0.149250522	0.442919955	1.567095139	0.12084709	0.52490902
NEK10	6	7	0.149250522	0.442919955	1.567095139	0.12084709	0.52490902
NLRP9	6	7	0.149250522	0.442919955	1.567095139	0.12084709	0.52490902
OPN5	6	7	0.149250522	0.442919955	1.567095139	0.12084709	0.52490902
OR10A2	6	7	0.149250522	0.442919955	1.567095139	0.12084709	0.52490902
OR2D3	6	7	0.149250522	0.442919955	1.567095139	0.12084709	0.52490902
PALB2	6	7	0.149250522	0.442919955	1.567095139	0.12084709	0.52490902
PCDHB15	6	7	0.149250522	0.442919955	1.567095139	0.12084709	0.52490902
PCDHGB5	6	7	0.149250522	0.442919955	1.567095139	0.12084709	0.52490902
PPP1R12B	6	7	0.149250522	0.442919955	1.567095139	0.12084709	0.52490902
PREPL	6	7	0.149250522	0.442919955	1.567095139	0.12084709	0.52490902
RPGRIP1L	6	7	0.149250522	0.442919955	1.567095139	0.12084709	0.52490902
SCG2	6	7	0.149250522	0.442919955	1.567095139	0.12084709	0.52490902
SEMA6A	6	7	0.149250522	0.442919955	1.567095139	0.12084709	0.52490902
SUN1	6	7	0.149250522	0.442919955	1.567095139	0.12084709	0.52490902
SYNRG	6	7	0.149250522	0.442919955	1.567095139	0.12084709	0.52490902
SYT16	6	7	0.149250522	0.442919955	1.567095139	0.12084709	0.52490902
TTC29	6	7	0.149250522	0.442919955	1.567095139	0.12084709	0.52490902
UBN2	6	7	0.149250522	0.442919955	1.567095139	0.12084709	0.52490902
VN1R4	6	7	0.149250522	0.442919955	1.567095139	0.12084709	0.52490902
ZBTB14	6	7	0.149250522	0.442919955	1.567095139	0.12084709	0.52490902
ZNF304	6	7	0.149250522	0.442919955	1.567095139	0.12084709	0.52490902
ZNF827	6	7	0.149250522	0.442919955	1.567095139	0.12084709	0.52490902
ANK2	56	39	0.150209949	0.707637198	1.154260845	0.436650234	0.527932041
LRRC4C	20	17	0.150722452	0.596668751	1.251214356	0.287466652	0.529381316

CTCFL	10	10	0.153508016	0.51355075	1.406238715	0.187530611	0.534040081
FGD5	10	10	0.153508016	0.51355075	1.406238715	0.187530611	0.534040081
IGSF9B	10	10	0.153508016	0.51355075	1.406238715	0.187530611	0.534040081
KIF19	10	10	0.153508016	0.51355075	1.406238715	0.187530611	0.534040081
LILRB2	10	10	0.153508016	0.51355075	1.406238715	0.187530611	0.534040081
OR10X1	10	10	0.153508016	0.51355075	1.406238715	0.187530611	0.534040081
OR2A2	10	10	0.153508016	0.51355075	1.406238715	0.187530611	0.534040081
PCDH8	10	10	0.153508016	0.51355075	1.406238715	0.187530611	0.534040081
PCDHGB2	10	10	0.153508016	0.51355075	1.406238715	0.187530611	0.534040081
POTEH	10	10	0.153508016	0.51355075	1.406238715	0.187530611	0.534040081
ROS1	10	10	0.153508016	0.51355075	1.406238715	0.187530611	0.534040081
SNRNP200	10	10	0.153508016	0.51355075	1.406238715	0.187530611	0.534040081
CSF1R	12	2	0.153564306	3.245483036	30.2010021	0.709905732	0.534040081
LMO7	12	2	0.153564306	3.245483036	30.2010021	0.709905732	0.534040081
STON2	12	2	0.153564306	3.245483036	30.2010021	0.709905732	0.534040081
DOCK11	17	4	0.161200252	2.309730097	9.590730378	0.736978494	0.550603566
SETX	17	4	0.161200252	2.309730097	9.590730378	0.736978494	0.550603566
TET1	17	4	0.161200252	2.309730097	9.590730378	0.736978494	0.550603566
ZNF257	17	4	0.161200252	2.309730097	9.590730378	0.736978494	0.550603566
BEND2	7	8	0.165851591	0.450781641	1.450670795	0.136542276	0.550603566
CEP152	7	8	0.165851591	0.450781641	1.450670795	0.136542276	0.550603566
CFHR5	7	8	0.165851591	0.450781641	1.450670795	0.136542276	0.550603566
CHD4	7	8	0.165851591	0.450781641	1.450670795	0.136542276	0.550603566
DROSHA	7	8	0.165851591	0.450781641	1.450670795	0.136542276	0.550603566
EDNRB	7	8	0.165851591	0.450781641	1.450670795	0.136542276	0.550603566
GPC6	7	8	0.165851591	0.450781641	1.450670795	0.136542276	0.550603566
IGHM	7	8	0.165851591	0.450781641	1.450670795	0.136542276	0.550603566
KIRREL2	7	8	0.165851591	0.450781641	1.450670795	0.136542276	0.550603566
MN1	7	8	0.165851591	0.450781641	1.450670795	0.136542276	0.550603566
MUC4	7	8	0.165851591	0.450781641	1.450670795	0.136542276	0.550603566
MYO15A	7	8	0.165851591	0.450781641	1.450670795	0.136542276	0.550603566
NEDD4	7	8	0.165851591	0.450781641	1.450670795	0.136542276	0.550603566
NUTM1	7	8	0.165851591	0.450781641	1.450670795	0.136542276	0.550603566
OR4D11	7	8	0.165851591	0.450781641	1.450670795	0.136542276	0.550603566
OR4K1	7	8	0.165851591	0.450781641	1.450670795	0.136542276	0.550603566
OR52R1	7	8	0.165851591	0.450781641	1.450670795	0.136542276	0.550603566
PCDHB1	7	8	0.165851591	0.450781641	1.450670795	0.136542276	0.550603566
POLR3B	7	8	0.165851591	0.450781641	1.450670795	0.136542276	0.550603566
RPGRIP1	7	8	0.165851591	0.450781641	1.450670795	0.136542276	0.550603566
SIRPG	7	8	0.165851591	0.450781641	1.450670795	0.136542276	0.550603566
SLC17A4	7	8	0.165851591	0.450781641	1.450670795	0.136542276	0.550603566
SLC45A2	7	8	0.165851591	0.450781641	1.450670795	0.136542276	0.550603566
SMYD1	7	8	0.165851591	0.450781641	1.450670795	0.136542276	0.550603566
TOX2	7	8	0.165851591	0.450781641	1.450670795	0.136542276	0.550603566
TTBK1	7	8	0.165851591	0.450781641	1.450670795	0.136542276	0.550603566
UBBP4	7	8	0.165851591	0.450781641	1.450670795	0.136542276	0.550603566
ZNF496	7	8	0.165851591	0.450781641	1.450670795	0.136542276	0.550603566
FER1L6	22	18	0.166088766	0.619874548	1.266634767	0.306878636	0.550603566
LAMB4	22	18	0.166088766	0.619874548	1.266634767	0.306878636	0.550603566
SPATA31A	22	18	0.166088766	0.619874548	1.266634767	0.306878636	0.550603566
ABCA6	11	11	0.166779508	0.511985887	1.333570086	0.1965505	0.550603566
ARID1B	11	11	0.166779508	0.511985887	1.333570086	0.1965505	0.550603566
BMS1	11	11	0.166779508	0.511985887	1.333570086	0.1965505	0.550603566
DTNA	11	11	0.166779508	0.511985887	1.333570086	0.1965505	0.550603566
FRMPD2	11	11	0.166779508	0.511985887	1.333570086	0.1965505	0.550603566
ITIH5	11	11	0.166779508	0.511985887	1.333570086	0.1965505	0.550603566
MUC3A	11	11	0.166779508	0.511985887	1.333570086	0.1965505	0.550603566
MYH11	11	11	0.166779508	0.511985887	1.333570086	0.1965505	0.550603566

NCAM2	11	11	0.166779508	0.511985887	1.333570086	0.1965505	0.550603566
NCOR2	11	11	0.166779508	0.511985887	1.333570086	0.1965505	0.550603566
NELL2	11	11	0.166779508	0.511985887	1.333570086	0.1965505	0.550603566
OR5T2	11	11	0.166779508	0.511985887	1.333570086	0.1965505	0.550603566
RGPD3	11	11	0.166779508	0.511985887	1.333570086	0.1965505	0.550603566
TKTL2	11	11	0.166779508	0.511985887	1.333570086	0.1965505	0.550603566
TRPM2	11	11	0.166779508	0.511985887	1.333570086	0.1965505	0.550603566
ZNF236	11	11	0.166779508	0.511985887	1.333570086	0.1965505	0.550603566
KCNH1	16	14	0.167331964	0.583289802	1.326890805	0.259225903	0.550603566
MDGA2	16	14	0.167331964	0.583289802	1.326890805	0.259225903	0.550603566
UTRN	16	14	0.167331964	0.583289802	1.326890805	0.259225903	0.550603566
ACOT7	5	0	0.169463409	Inf	Inf	0.485283434	0.550603566
AMBP	5	0	0.169463409	Inf	Inf	0.485283434	0.550603566
AP5Z1	5	0	0.169463409	Inf	Inf	0.485283434	0.550603566
ARMC9	5	0	0.169463409	Inf	Inf	0.485283434	0.550603566
ASB9	5	0	0.169463409	Inf	Inf	0.485283434	0.550603566
ASS1	5	0	0.169463409	Inf	Inf	0.485283434	0.550603566
ATP6V1A	5	0	0.169463409	Inf	Inf	0.485283434	0.550603566
AXIN1	5	0	0.169463409	Inf	Inf	0.485283434	0.550603566
CEP55	5	0	0.169463409	Inf	Inf	0.485283434	0.550603566
CYP3A7	5	0	0.169463409	Inf	Inf	0.485283434	0.550603566
DDX39B	5	0	0.169463409	Inf	Inf	0.485283434	0.550603566
DDX46	5	0	0.169463409	Inf	Inf	0.485283434	0.550603566
DEFB116	5	0	0.169463409	Inf	Inf	0.485283434	0.550603566
DNASE2	5	0	0.169463409	Inf	Inf	0.485283434	0.550603566
GPR22	5	0	0.169463409	Inf	Inf	0.485283434	0.550603566
HAL	5	0	0.169463409	Inf	Inf	0.485283434	0.550603566
HKDC1	5	0	0.169463409	Inf	Inf	0.485283434	0.550603566
HOXB6	5	0	0.169463409	Inf	Inf	0.485283434	0.550603566
IL27	5	0	0.169463409	Inf	Inf	0.485283434	0.550603566
IL3	5	0	0.169463409	Inf	Inf	0.485283434	0.550603566
JMY	5	0	0.169463409	Inf	Inf	0.485283434	0.550603566
KBTBD7	5	0	0.169463409	Inf	Inf	0.485283434	0.550603566
KIAA1033	5	0	0.169463409	Inf	Inf	0.485283434	0.550603566
KRTAP19-(	5	0	0.169463409	Inf	Inf	0.485283434	0.550603566
LPCAT2	5	0	0.169463409	Inf	Inf	0.485283434	0.550603566
LRRC37A2	5	0	0.169463409	Inf	Inf	0.485283434	0.550603566
LRRC47	5	0	0.169463409	Inf	Inf	0.485283434	0.550603566
MAP3K7	5	0	0.169463409	Inf	Inf	0.485283434	0.550603566
MRPS5	5	0	0.169463409	Inf	Inf	0.485283434	0.550603566
MTA2	5	0	0.169463409	Inf	Inf	0.485283434	0.550603566
MUTYH	5	0	0.169463409	Inf	Inf	0.485283434	0.550603566
MYCT1	5	0	0.169463409	Inf	Inf	0.485283434	0.550603566
NAGPA	5	0	0.169463409	Inf	Inf	0.485283434	0.550603566
NLGN4Y	5	0	0.169463409	Inf	Inf	0.485283434	0.550603566
NR2F1	5	0	0.169463409	Inf	Inf	0.485283434	0.550603566
NRF1	5	0	0.169463409	Inf	Inf	0.485283434	0.550603566
OR1L4	5	0	0.169463409	Inf	Inf	0.485283434	0.550603566
OR2T5	5	0	0.169463409	Inf	Inf	0.485283434	0.550603566
PIGR	5	0	0.169463409	Inf	Inf	0.485283434	0.550603566
RPS6KA3	5	0	0.169463409	Inf	Inf	0.485283434	0.550603566
SENP5	5	0	0.169463409	Inf	Inf	0.485283434	0.550603566
SIAH3	5	0	0.169463409	Inf	Inf	0.485283434	0.550603566
SLC10A5	5	0	0.169463409	Inf	Inf	0.485283434	0.550603566
SSX7	5	0	0.169463409	Inf	Inf	0.485283434	0.550603566
STK32C	5	0	0.169463409	Inf	Inf	0.485283434	0.550603566
SUSD5	5	0	0.169463409	Inf	Inf	0.485283434	0.550603566
TEK	5	0	0.169463409	Inf	Inf	0.485283434	0.550603566

ZCRB1	5	0	0.169463409	Inf	Inf	0.485283434	0.550603566
ZNF317	5	0	0.169463409	Inf	Inf	0.485283434	0.550603566
ZNF408	5	0	0.169463409	Inf	Inf	0.485283434	0.550603566
ZNF610	5	0	0.169463409	Inf	Inf	0.485283434	0.550603566
ZNF829	5	0	0.169463409	Inf	Inf	0.485283434	0.550603566
PLEC	30	23	0.169472569	0.657206206	1.230365865	0.354851946	0.550603566
APP	8	1	0.173222266	4.296290858	192.016587	0.568026912	0.555951971
DPP3	8	1	0.173222266	4.296290858	192.016587	0.568026912	0.555951971
iPR75-ASB	8	1	0.173222266	4.296290858	192.016587	0.568026912	0.555951971
MYO1G	8	1	0.173222266	4.296290858	192.016587	0.568026912	0.555951971
OR13C4	8	1	0.173222266	4.296290858	192.016587	0.568026912	0.555951971
OR52E8	8	1	0.173222266	4.296290858	192.016587	0.568026912	0.555951971
PER3	8	1	0.173222266	4.296290858	192.016587	0.568026912	0.555951971
PKN2	8	1	0.173222266	4.296290858	192.016587	0.568026912	0.555951971
PLXNC1	8	1	0.173222266	4.296290858	192.016587	0.568026912	0.555951971
PPFIA4	8	1	0.173222266	4.296290858	192.016587	0.568026912	0.555951971
PRICKLE2	8	1	0.173222266	4.296290858	192.016587	0.568026912	0.555951971
PRKCQ	8	1	0.173222266	4.296290858	192.016587	0.568026912	0.555951971
PUS7	8	1	0.173222266	4.296290858	192.016587	0.568026912	0.555951971
RBM41	8	1	0.173222266	4.296290858	192.016587	0.568026912	0.555951971
SIGLEC11	8	1	0.173222266	4.296290858	192.016587	0.568026912	0.555951971
SON	8	1	0.173222266	4.296290858	192.016587	0.568026912	0.555951971
SPARC	8	1	0.173222266	4.296290858	192.016587	0.568026912	0.555951971
TLR6	8	1	0.173222266	4.296290858	192.016587	0.568026912	0.555951971
TTC7A	8	1	0.173222266	4.296290858	192.016587	0.568026912	0.555951971
WSCD1	8	1	0.173222266	4.296290858	192.016587	0.568026912	0.555951971
ANKEF1	9	1	0.175828552	4.847892843	214.0440235	0.662111156	0.561249834
KCNV1	9	1	0.175828552	4.847892843	214.0440235	0.662111156	0.561249834
MTNR1B	9	1	0.175828552	4.847892843	214.0440235	0.662111156	0.561249834
NCAPG2	9	1	0.175828552	4.847892843	214.0440235	0.662111156	0.561249834
NRSN1	9	1	0.175828552	4.847892843	214.0440235	0.662111156	0.561249834
SLC12A7	9	1	0.175828552	4.847892843	214.0440235	0.662111156	0.561249834
SLC34A2	9	1	0.175828552	4.847892843	214.0440235	0.662111156	0.561249834
SMTN	9	1	0.175828552	4.847892843	214.0440235	0.662111156	0.561249834
WNT7A	9	1	0.175828552	4.847892843	214.0440235	0.662111156	0.561249834
HDAC9	17	15	0.1771246	0.576619616	1.27631702	0.262970367	0.564364459
SCN2A	17	15	0.1771246	0.576619616	1.27631702	0.262970367	0.564364459
SETD2	17	15	0.1771246	0.576619616	1.27631702	0.262970367	0.564364459
EPHA5	32	24	0.180267828	0.671808529	1.239251443	0.368184302	0.574033578
GABRA5	12	11	0.182612964	0.560208814	1.435527661	0.220778036	0.578712308
GFRAL	12	11	0.182612964	0.560208814	1.435527661	0.220778036	0.578712308
LRFN2	12	11	0.182612964	0.560208814	1.435527661	0.220778036	0.578712308
OR8U1	12	11	0.182612964	0.560208814	1.435527661	0.220778036	0.578712308
PRKD1	12	11	0.182612964	0.560208814	1.435527661	0.220778036	0.578712308
RIF1	12	11	0.182612964	0.560208814	1.435527661	0.220778036	0.578712308
ROBO1	12	11	0.182612964	0.560208814	1.435527661	0.220778036	0.578712308
SYT10	12	11	0.182612964	0.560208814	1.435527661	0.220778036	0.578712308
HMCN1	43	31	0.184384066	0.691575053	1.188623875	0.405804623	0.583625252
PCDH11X	43	31	0.184384066	0.691575053	1.188623875	0.405804623	0.583625252
CNTNAP5	33	25	0.185234067	0.66284064	1.209910063	0.366785937	0.585964859
KIAA1549L	18	15	0.18721909	0.612466686	1.344167375	0.28280736	0.591536228
TCHH	18	15	0.18721909	0.612466686	1.344167375	0.28280736	0.591536228
ARMC5	8	8	0.192278373	0.516650546	1.611075498	0.165674872	0.591596801
ASAP1	8	8	0.192278373	0.516650546	1.611075498	0.165674872	0.591596801
BRIP1	8	8	0.192278373	0.516650546	1.611075498	0.165674872	0.591596801
C10orf71	8	8	0.192278373	0.516650546	1.611075498	0.165674872	0.591596801
C8B	8	8	0.192278373	0.516650546	1.611075498	0.165674872	0.591596801
CNTN6	8	8	0.192278373	0.516650546	1.611075498	0.165674872	0.591596801

CORIN	8	8	0.192278373	0.516650546	1.611075498	0.165674872	0.591596801
CR2	8	8	0.192278373	0.516650546	1.611075498	0.165674872	0.591596801
CYP26B1	8	8	0.192278373	0.516650546	1.611075498	0.165674872	0.591596801
DSC3	8	8	0.192278373	0.516650546	1.611075498	0.165674872	0.591596801
FLT4	8	8	0.192278373	0.516650546	1.611075498	0.165674872	0.591596801
FN1	8	8	0.192278373	0.516650546	1.611075498	0.165674872	0.591596801
FYB	8	8	0.192278373	0.516650546	1.611075498	0.165674872	0.591596801
HTR5A	8	8	0.192278373	0.516650546	1.611075498	0.165674872	0.591596801
ITGBL1	8	8	0.192278373	0.516650546	1.611075498	0.165674872	0.591596801
KCNA1	8	8	0.192278373	0.516650546	1.611075498	0.165674872	0.591596801
KCTD8	8	8	0.192278373	0.516650546	1.611075498	0.165674872	0.591596801
KIF17	8	8	0.192278373	0.516650546	1.611075498	0.165674872	0.591596801
LINGO2	8	8	0.192278373	0.516650546	1.611075498	0.165674872	0.591596801
LRRN2	8	8	0.192278373	0.516650546	1.611075498	0.165674872	0.591596801
MED13	8	8	0.192278373	0.516650546	1.611075498	0.165674872	0.591596801
MTR	8	8	0.192278373	0.516650546	1.611075498	0.165674872	0.591596801
MYOM3	8	8	0.192278373	0.516650546	1.611075498	0.165674872	0.591596801
NOX3	8	8	0.192278373	0.516650546	1.611075498	0.165674872	0.591596801
OR10J1	8	8	0.192278373	0.516650546	1.611075498	0.165674872	0.591596801
OR51D1	8	8	0.192278373	0.516650546	1.611075498	0.165674872	0.591596801
OR6K6	8	8	0.192278373	0.516650546	1.611075498	0.165674872	0.591596801
OR6N2	8	8	0.192278373	0.516650546	1.611075498	0.165674872	0.591596801
PEAK1	8	8	0.192278373	0.516650546	1.611075498	0.165674872	0.591596801
PLCG2	8	8	0.192278373	0.516650546	1.611075498	0.165674872	0.591596801
PLEKHG4E	8	8	0.192278373	0.516650546	1.611075498	0.165674872	0.591596801
PRDM2	8	8	0.192278373	0.516650546	1.611075498	0.165674872	0.591596801
RFX4	8	8	0.192278373	0.516650546	1.611075498	0.165674872	0.591596801
RPTN	8	8	0.192278373	0.516650546	1.611075498	0.165674872	0.591596801
SHANK2	8	8	0.192278373	0.516650546	1.611075498	0.165674872	0.591596801
SLC4A3	8	8	0.192278373	0.516650546	1.611075498	0.165674872	0.591596801
SP100	8	8	0.192278373	0.516650546	1.611075498	0.165674872	0.591596801
TRIM49	8	8	0.192278373	0.516650546	1.611075498	0.165674872	0.591596801
TRPM8	8	8	0.192278373	0.516650546	1.611075498	0.165674872	0.591596801
WDR66	8	8	0.192278373	0.516650546	1.611075498	0.165674872	0.591596801
WNK3	8	8	0.192278373	0.516650546	1.611075498	0.165674872	0.591596801
CHD5	13	12	0.193948196	0.554622234	1.363980229	0.227368486	0.591596801
DLGAP3	13	12	0.193948196	0.554622234	1.363980229	0.227368486	0.591596801
DPP10	13	12	0.193948196	0.554622234	1.363980229	0.227368486	0.591596801
MEGF10	13	12	0.193948196	0.554622234	1.363980229	0.227368486	0.591596801
NFASC	13	12	0.193948196	0.554622234	1.363980229	0.227368486	0.591596801
FAM179B	14	3	0.194308652	2.526302062	13.90333271	0.691310581	0.591596801
OR13F1	14	3	0.194308652	2.526302062	13.90333271	0.691310581	0.591596801
PSG11	14	3	0.194308652	2.526302062	13.90333271	0.691310581	0.591596801
SLC5A7	14	3	0.194308652	2.526302062	13.90333271	0.691310581	0.591596801
ZMYM3	14	3	0.194308652	2.526302062	13.90333271	0.691310581	0.591596801
HECW1	26	20	0.194626367	0.659346751	1.289822118	0.341380073	0.591596801
GRM7	19	16	0.195722061	0.604181884	1.294643226	0.285253448	0.591596801
PCDHB8	19	16	0.195722061	0.604181884	1.294643226	0.285253448	0.591596801
SPEG	19	16	0.195722061	0.604181884	1.294643226	0.285253448	0.591596801
ADARB2	5	6	0.200780299	0.431916902	1.726755769	0.102626896	0.591596801
ADCY9	5	6	0.200780299	0.431916902	1.726755769	0.102626896	0.591596801
AGO2	5	6	0.200780299	0.431916902	1.726755769	0.102626896	0.591596801
APOA5	5	6	0.200780299	0.431916902	1.726755769	0.102626896	0.591596801
ATP8B3	5	6	0.200780299	0.431916902	1.726755769	0.102626896	0.591596801
ATXN2	5	6	0.200780299	0.431916902	1.726755769	0.102626896	0.591596801
BCKDHB	5	6	0.200780299	0.431916902	1.726755769	0.102626896	0.591596801
BEND6	5	6	0.200780299	0.431916902	1.726755769	0.102626896	0.591596801
BTNL8	5	6	0.200780299	0.431916902	1.726755769	0.102626896	0.591596801

C10orf120	5	6	0.200780299	0.431916902	1.726755769	0.102626896	0.591596801
C1QB	5	6	0.200780299	0.431916902	1.726755769	0.102626896	0.591596801
C9orf3	5	6	0.200780299	0.431916902	1.726755769	0.102626896	0.591596801
CADM3	5	6	0.200780299	0.431916902	1.726755769	0.102626896	0.591596801
CALB2	5	6	0.200780299	0.431916902	1.726755769	0.102626896	0.591596801
CALD1	5	6	0.200780299	0.431916902	1.726755769	0.102626896	0.591596801
CHD3	5	6	0.200780299	0.431916902	1.726755769	0.102626896	0.591596801
COL4A3	5	6	0.200780299	0.431916902	1.726755769	0.102626896	0.591596801
DDX50	5	6	0.200780299	0.431916902	1.726755769	0.102626896	0.591596801
EBF2	5	6	0.200780299	0.431916902	1.726755769	0.102626896	0.591596801
EPHB3	5	6	0.200780299	0.431916902	1.726755769	0.102626896	0.591596801
ERBB3	5	6	0.200780299	0.431916902	1.726755769	0.102626896	0.591596801
ESR1	5	6	0.200780299	0.431916902	1.726755769	0.102626896	0.591596801
FMO3	5	6	0.200780299	0.431916902	1.726755769	0.102626896	0.591596801
GABBR2	5	6	0.200780299	0.431916902	1.726755769	0.102626896	0.591596801
GLRA4	5	6	0.200780299	0.431916902	1.726755769	0.102626896	0.591596801
GOLGA4	5	6	0.200780299	0.431916902	1.726755769	0.102626896	0.591596801
GPRC5B	5	6	0.200780299	0.431916902	1.726755769	0.102626896	0.591596801
GUCY2C	5	6	0.200780299	0.431916902	1.726755769	0.102626896	0.591596801
HCN4	5	6	0.200780299	0.431916902	1.726755769	0.102626896	0.591596801
HEG1	5	6	0.200780299	0.431916902	1.726755769	0.102626896	0.591596801
HELB	5	6	0.200780299	0.431916902	1.726755769	0.102626896	0.591596801
HOXD3	5	6	0.200780299	0.431916902	1.726755769	0.102626896	0.591596801
KDM5B	5	6	0.200780299	0.431916902	1.726755769	0.102626896	0.591596801
KSR2	5	6	0.200780299	0.431916902	1.726755769	0.102626896	0.591596801
MAGED2	5	6	0.200780299	0.431916902	1.726755769	0.102626896	0.591596801
MCM6	5	6	0.200780299	0.431916902	1.726755769	0.102626896	0.591596801
MTMR4	5	6	0.200780299	0.431916902	1.726755769	0.102626896	0.591596801
NAV1	5	6	0.200780299	0.431916902	1.726755769	0.102626896	0.591596801
NUP133	5	6	0.200780299	0.431916902	1.726755769	0.102626896	0.591596801
OR10G9	5	6	0.200780299	0.431916902	1.726755769	0.102626896	0.591596801
OR8B12	5	6	0.200780299	0.431916902	1.726755769	0.102626896	0.591596801
P2RX5	5	6	0.200780299	0.431916902	1.726755769	0.102626896	0.591596801
PLCH2	5	6	0.200780299	0.431916902	1.726755769	0.102626896	0.591596801
PPAPDC1A	5	6	0.200780299	0.431916902	1.726755769	0.102626896	0.591596801
PRSS36	5	6	0.200780299	0.431916902	1.726755769	0.102626896	0.591596801
PRSS58	5	6	0.200780299	0.431916902	1.726755769	0.102626896	0.591596801
QSER1	5	6	0.200780299	0.431916902	1.726755769	0.102626896	0.591596801
RAD54L2	5	6	0.200780299	0.431916902	1.726755769	0.102626896	0.591596801
RASA3	5	6	0.200780299	0.431916902	1.726755769	0.102626896	0.591596801
RNF133	5	6	0.200780299	0.431916902	1.726755769	0.102626896	0.591596801
RPS6KC1	5	6	0.200780299	0.431916902	1.726755769	0.102626896	0.591596801
RTP2	5	6	0.200780299	0.431916902	1.726755769	0.102626896	0.591596801
SERPINI2	5	6	0.200780299	0.431916902	1.726755769	0.102626896	0.591596801
SF3B2	5	6	0.200780299	0.431916902	1.726755769	0.102626896	0.591596801
SV2B	5	6	0.200780299	0.431916902	1.726755769	0.102626896	0.591596801
SYCP2L	5	6	0.200780299	0.431916902	1.726755769	0.102626896	0.591596801
TANC2	5	6	0.200780299	0.431916902	1.726755769	0.102626896	0.591596801
TBC1D31	5	6	0.200780299	0.431916902	1.726755769	0.102626896	0.591596801
TRAPPC9	5	6	0.200780299	0.431916902	1.726755769	0.102626896	0.591596801
TRHR	5	6	0.200780299	0.431916902	1.726755769	0.102626896	0.591596801
JHRF1BP1l	5	6	0.200780299	0.431916902	1.726755769	0.102626896	0.591596801
UNC80	5	6	0.200780299	0.431916902	1.726755769	0.102626896	0.591596801
ZBTB20	5	6	0.200780299	0.431916902	1.726755769	0.102626896	0.591596801
ZNF454	5	6	0.200780299	0.431916902	1.726755769	0.102626896	0.591596801
ZNF678	5	6	0.200780299	0.431916902	1.726755769	0.102626896	0.591596801
ZSCAN10	5	6	0.200780299	0.431916902	1.726755769	0.102626896	0.591596801
NALCN	37	27	0.204299563	0.688108288	1.22552586	0.390256682	0.599819868

NELL1	20	16	0.205845754	0.638009902	1.357416905	0.304354514	0.599819868
ATR	9	9	0.205954041	0.515105604	1.496183133	0.177311621	0.599819868
BAZ2B	9	9	0.205954041	0.515105604	1.496183133	0.177311621	0.599819868
C1orf94	9	9	0.205954041	0.515105604	1.496183133	0.177311621	0.599819868
CACNB2	9	9	0.205954041	0.515105604	1.496183133	0.177311621	0.599819868
CPAMD8	9	9	0.205954041	0.515105604	1.496183133	0.177311621	0.599819868
EPPK1	9	9	0.205954041	0.515105604	1.496183133	0.177311621	0.599819868
HEATR5B	9	9	0.205954041	0.515105604	1.496183133	0.177311621	0.599819868
MAGEB4	9	9	0.205954041	0.515105604	1.496183133	0.177311621	0.599819868
NCOA6	9	9	0.205954041	0.515105604	1.496183133	0.177311621	0.599819868
OR4L1	9	9	0.205954041	0.515105604	1.496183133	0.177311621	0.599819868
OR4N2	9	9	0.205954041	0.515105604	1.496183133	0.177311621	0.599819868
OR4N5	9	9	0.205954041	0.515105604	1.496183133	0.177311621	0.599819868
OR6Y1	9	9	0.205954041	0.515105604	1.496183133	0.177311621	0.599819868
POTEG	9	9	0.205954041	0.515105604	1.496183133	0.177311621	0.599819868
TBX15	9	9	0.205954041	0.515105604	1.496183133	0.177311621	0.599819868
TDRD5	9	9	0.205954041	0.515105604	1.496183133	0.177311621	0.599819868
TRIM42	9	9	0.205954041	0.515105604	1.496183133	0.177311621	0.599819868
USP6	9	9	0.205954041	0.515105604	1.496183133	0.177311621	0.599819868
XIRP1	9	9	0.205954041	0.515105604	1.496183133	0.177311621	0.599819868
ADAM19	14	12	0.209262907	0.599124942	1.454842341	0.250508753	0.606782078
FSHR	14	12	0.209262907	0.599124942	1.454842341	0.250508753	0.606782078
NLRP7	14	12	0.209262907	0.599124942	1.454842341	0.250508753	0.606782078
OR2T12	14	12	0.209262907	0.599124942	1.454842341	0.250508753	0.606782078
OR5F1	14	12	0.209262907	0.599124942	1.454842341	0.250508753	0.606782078
SLC9A4	14	12	0.209262907	0.599124942	1.454842341	0.250508753	0.606782078
THOC2	14	12	0.209262907	0.599124942	1.454842341	0.250508753	0.606782078
ZFYVE26	14	12	0.209262907	0.599124942	1.454842341	0.250508753	0.606782078
VCAN	38	27	0.210160434	0.709145169	1.259818755	0.403496799	0.609050467
TRIML1	21	6	0.212705606	1.904730766	5.88415411	0.72497518	0.616088675
GRM8	21	17	0.213141275	0.628511719	1.309289535	0.30566933	0.616337406
OVCH1	21	17	0.213141275	0.628511719	1.309289535	0.30566933	0.616337406
ZNF479	21	17	0.213141275	0.628511719	1.309289535	0.30566933	0.616337406
NLRP13	15	13	0.218788856	0.590726779	1.385594963	0.255074334	0.631631835
PREX1	15	13	0.218788856	0.590726779	1.385594963	0.255074334	0.631631835
ΓMEM132E	15	13	0.218788856	0.590726779	1.385594963	0.255074334	0.631631835
NCKAP5	30	22	0.22042388	0.691672772	1.3060342	0.371128323	0.636004712
CDH8	22	17	0.223286563	0.660561779	1.367635762	0.324028758	0.643562036
DUSP27	22	17	0.223286563	0.660561779	1.367635762	0.324028758	0.643562036
CUBN	31	23	0.224732176	0.68139073	1.271150368	0.369628195	0.64737563
MMP16	30	10	0.225634508	1.642293057	3.866839192	0.757308755	0.64962092
COL14A1	23	18	0.229464558	0.650148769	1.320927143	0.32443856	0.654216736
MGAM	23	18	0.229464558	0.650148769	1.320927143	0.32443856	0.654216736
OR2M7	16	4	0.229981869	2.166963375	9.052834716	0.684032597	0.654216736
NPAP1	43	30	0.230195383	0.719764223	1.243158893	0.420712424	0.654216736
ARHGEF6	10	9	0.230201122	0.574028037	1.631638929	0.205025806	0.654216736
ATP7B	10	9	0.230201122	0.574028037	1.631638929	0.205025806	0.654216736
BMPER	10	9	0.230201122	0.574028037	1.631638929	0.205025806	0.654216736
C6orf118	10	9	0.230201122	0.574028037	1.631638929	0.205025806	0.654216736
CDH22	10	9	0.230201122	0.574028037	1.631638929	0.205025806	0.654216736
CNTLN	10	9	0.230201122	0.574028037	1.631638929	0.205025806	0.654216736
COL4A4	10	9	0.230201122	0.574028037	1.631638929	0.205025806	0.654216736
DENND2A	10	9	0.230201122	0.574028037	1.631638929	0.205025806	0.654216736
DNAI2	10	9	0.230201122	0.574028037	1.631638929	0.205025806	0.654216736
GPR139	10	9	0.230201122	0.574028037	1.631638929	0.205025806	0.654216736
INADL	10	9	0.230201122	0.574028037	1.631638929	0.205025806	0.654216736
LY75	10	9	0.230201122	0.574028037	1.631638929	0.205025806	0.654216736
MGAT5B	10	9	0.230201122	0.574028037	1.631638929	0.205025806	0.654216736

OR4K2	10	9	0.230201122	0.574028037	1.631638929	0.205025806	0.654216736
OTOA	10	9	0.230201122	0.574028037	1.631638929	0.205025806	0.654216736
PCDHGB1	10	9	0.230201122	0.574028037	1.631638929	0.205025806	0.654216736
PPFIA2	10	9	0.230201122	0.574028037	1.631638929	0.205025806	0.654216736
PTPRN	10	9	0.230201122	0.574028037	1.631638929	0.205025806	0.654216736
UNC45B	10	9	0.230201122	0.574028037	1.631638929	0.205025806	0.654216736
ZFC3H1	10	9	0.230201122	0.574028037	1.631638929	0.205025806	0.654216736
ADAM11	10	2	0.232607891	2.687850248	25.51083151	0.563332714	0.659992116
RALGAPA2	10	2	0.232607891	2.687850248	25.51083151	0.563332714	0.659992116
ZNF148	10	2	0.232607891	2.687850248	25.51083151	0.563332714	0.659992116
ASH1L	16	13	0.233632935	0.632073834	1.467579114	0.277146162	0.660066112
CD163	16	13	0.233632935	0.632073834	1.467579114	0.277146162	0.660066112
COL4A2	16	13	0.233632935	0.632073834	1.467579114	0.277146162	0.660066112
FHOD3	16	13	0.233632935	0.632073834	1.467579114	0.277146162	0.660066112
ITGA4	16	13	0.233632935	0.632073834	1.467579114	0.277146162	0.660066112
NLRP10	16	13	0.233632935	0.632073834	1.467579114	0.277146162	0.660066112
PCDHA3	16	13	0.233632935	0.632073834	1.467579114	0.277146162	0.660066112
POLQ	16	13	0.233632935	0.632073834	1.467579114	0.277146162	0.660066112
CACNG3	11	2	0.235496772	2.965777096	27.85675378	0.636258422	0.663558602
DOK6	11	2	0.235496772	2.965777096	27.85675378	0.636258422	0.663558602
PROKR2	11	2	0.235496772	2.965777096	27.85675378	0.636258422	0.663558602
SF3B1	11	2	0.235496772	2.965777096	27.85675378	0.636258422	0.663558602
ZNF235	11	2	0.235496772	2.965777096	27.85675378	0.636258422	0.663558602
ABCB5	24	18	0.239625654	0.680621765	1.375554417	0.342146818	0.670524292
CDH18	24	18	0.239625654	0.680621765	1.375554417	0.342146818	0.670524292
F8	24	18	0.239625654	0.680621765	1.375554417	0.342146818	0.670524292
ATG2B	11	10	0.241164724	0.566589628	1.522703304	0.213366039	0.670524292
C6	11	10	0.241164724	0.566589628	1.522703304	0.213366039	0.670524292
CDHR2	11	10	0.241164724	0.566589628	1.522703304	0.213366039	0.670524292
CSPG4	11	10	0.241164724	0.566589628	1.522703304	0.213366039	0.670524292
DNER	11	10	0.241164724	0.566589628	1.522703304	0.213366039	0.670524292
GCN1L1	11	10	0.241164724	0.566589628	1.522703304	0.213366039	0.670524292
HEATR1	11	10	0.241164724	0.566589628	1.522703304	0.213366039	0.670524292
IL16	11	10	0.241164724	0.566589628	1.522703304	0.213366039	0.670524292
IL7R	11	10	0.241164724	0.566589628	1.522703304	0.213366039	0.670524292
INSC	11	10	0.241164724	0.566589628	1.522703304	0.213366039	0.670524292
ITPR1	11	10	0.241164724	0.566589628	1.522703304	0.213366039	0.670524292
MAGEC2	11	10	0.241164724	0.566589628	1.522703304	0.213366039	0.670524292
NOL4	11	10	0.241164724	0.566589628	1.522703304	0.213366039	0.670524292
NPAS4	11	10	0.241164724	0.566589628	1.522703304	0.213366039	0.670524292
NUP205	11	10	0.241164724	0.566589628	1.522703304	0.213366039	0.670524292
OR8J3	11	10	0.241164724	0.566589628	1.522703304	0.213366039	0.670524292
PREX2	11	10	0.241164724	0.566589628	1.522703304	0.213366039	0.670524292
SIGLEC12	11	10	0.241164724	0.566589628	1.522703304	0.213366039	0.670524292
SLC12A1	11	10	0.241164724	0.566589628	1.522703304	0.213366039	0.670524292
SZT2	11	10	0.241164724	0.566589628	1.522703304	0.213366039	0.670524292
ZNF676	11	10	0.241164724	0.566589628	1.522703304	0.213366039	0.670524292
DOCK10	17	14	0.241520668	0.621690901	1.401153349	0.280122786	0.670524292
SAGE1	17	14	0.241520668	0.621690901	1.401153349	0.280122786	0.670524292
SAMD9L	17	14	0.241520668	0.621690901	1.401153349	0.280122786	0.670524292
SATB2	17	14	0.241520668	0.621690901	1.401153349	0.280122786	0.670524292
HRNR	34	24	0.243763035	0.718642297	1.317492016	0.397019667	0.676394437
TRPM6	25	8	0.255838483	1.702845901	4.470914598	0.723728668	0.697553331
DYSF	18	14	0.25594234	0.660336969	1.475830022	0.301213109	0.697553331
RIMS2	37	26	0.256854857	0.719497483	1.289680764	0.406092388	0.697553331
ADGRB2	7	7	0.256930824	0.518185671	1.763649402	0.152235127	0.697553331
AIM1L	7	7	0.256930824	0.518185671	1.763649402	0.152235127	0.697553331
C1orf168	7	7	0.256930824	0.518185671	1.763649402	0.152235127	0.697553331

CAPN11	7	7	0.256930824	0.518185671	1.763649402	0.152235127	0.697553331
CDX4	7	7	0.256930824	0.518185671	1.763649402	0.152235127	0.697553331
DUSP22	7	7	0.256930824	0.518185671	1.763649402	0.152235127	0.697553331
ESRRG	7	7	0.256930824	0.518185671	1.763649402	0.152235127	0.697553331
GON4L	7	7	0.256930824	0.518185671	1.763649402	0.152235127	0.697553331
IFI44L	7	7	0.256930824	0.518185671	1.763649402	0.152235127	0.697553331
IGF2BP1	7	7	0.256930824	0.518185671	1.763649402	0.152235127	0.697553331
IL1RAPL2	7	7	0.256930824	0.518185671	1.763649402	0.152235127	0.697553331
ITSN2	7	7	0.256930824	0.518185671	1.763649402	0.152235127	0.697553331
KCNK18	7	7	0.256930824	0.518185671	1.763649402	0.152235127	0.697553331
MAGEB16	7	7	0.256930824	0.518185671	1.763649402	0.152235127	0.697553331
MUC7	7	7	0.256930824	0.518185671	1.763649402	0.152235127	0.697553331
NEBL	7	7	0.256930824	0.518185671	1.763649402	0.152235127	0.697553331
NFE2L2	7	7	0.256930824	0.518185671	1.763649402	0.152235127	0.697553331
NOD2	7	7	0.256930824	0.518185671	1.763649402	0.152235127	0.697553331
NUP214	7	7	0.256930824	0.518185671	1.763649402	0.152235127	0.697553331
OR10G7	7	7	0.256930824	0.518185671	1.763649402	0.152235127	0.697553331
OR4P4	7	7	0.256930824	0.518185671	1.763649402	0.152235127	0.697553331
PCDH1	7	7	0.256930824	0.518185671	1.763649402	0.152235127	0.697553331
PDE6A	7	7	0.256930824	0.518185671	1.763649402	0.152235127	0.697553331
PPEF2	7	7	0.256930824	0.518185671	1.763649402	0.152235127	0.697553331
RLF	7	7	0.256930824	0.518185671	1.763649402	0.152235127	0.697553331
RNF17	7	7	0.256930824	0.518185671	1.763649402	0.152235127	0.697553331
RREB1	7	7	0.256930824	0.518185671	1.763649402	0.152235127	0.697553331
SMC3	7	7	0.256930824	0.518185671	1.763649402	0.152235127	0.697553331
TCF4	7	7	0.256930824	0.518185671	1.763649402	0.152235127	0.697553331
TINAG	7	7	0.256930824	0.518185671	1.763649402	0.152235127	0.697553331
TRERF1	7	7	0.256930824	0.518185671	1.763649402	0.152235127	0.697553331
TRIM29	7	7	0.256930824	0.518185671	1.763649402	0.152235127	0.697553331
URGCP	7	7	0.256930824	0.518185671	1.763649402	0.152235127	0.697553331
USP24	7	7	0.256930824	0.518185671	1.763649402	0.152235127	0.697553331
VN1R2	7	7	0.256930824	0.518185671	1.763649402	0.152235127	0.697553331
WRN	7	7	0.256930824	0.518185671	1.763649402	0.152235127	0.697553331
WT1	7	7	0.256930824	0.518185671	1.763649402	0.152235127	0.697553331
ZNF267	7	7	0.256930824	0.518185671	1.763649402	0.152235127	0.697553331
ZNF365	7	7	0.256930824	0.518185671	1.763649402	0.152235127	0.697553331
ATP7A	17	5	0.26175305	1.837291414	6.485582001	0.635773193	0.709188426
FREM1	17	5	0.26175305	1.837291414	6.485582001	0.635773193	0.709188426
PCDHA10	17	5	0.26175305	1.837291414	6.485582001	0.635773193	0.709188426
VWF	17	5	0.26175305	1.837291414	6.485582001	0.635773193	0.709188426
ADCY2	19	15	0.262367527	0.648543563	1.412417364	0.302854278	0.709515683
AKAP6	19	15	0.262367527	0.648543563	1.412417364	0.302854278	0.709515683
ATP8A2	13	11	0.272195448	0.608749323	1.537932224	0.245352823	0.709515683
CYP11B2	13	11	0.272195448	0.608749323	1.537932224	0.245352823	0.709515683
DNAH2	13	11	0.272195448	0.608749323	1.537932224	0.245352823	0.709515683
DSG4	13	11	0.272195448	0.608749323	1.537932224	0.245352823	0.709515683
FAM171A1	13	11	0.272195448	0.608749323	1.537932224	0.245352823	0.709515683
FRG2C	13	11	0.272195448	0.608749323	1.537932224	0.245352823	0.709515683
KCNK2	13	11	0.272195448	0.608749323	1.537932224	0.245352823	0.709515683
PCDHA6	13	11	0.272195448	0.608749323	1.537932224	0.245352823	0.709515683
PCDHB11	13	11	0.272195448	0.608749323	1.537932224	0.245352823	0.709515683
PCDHB5	13	11	0.272195448	0.608749323	1.537932224	0.245352823	0.709515683
POLE	13	11	0.272195448	0.608749323	1.537932224	0.245352823	0.709515683
POTEF	13	11	0.272195448	0.608749323	1.537932224	0.245352823	0.709515683
PTPRN2	13	11	0.272195448	0.608749323	1.537932224	0.245352823	0.709515683
ZNF735	13	11	0.272195448	0.608749323	1.537932224	0.245352823	0.709515683
C7orf33	7	1	0.272823628	3.74796884	170.1247361	0.475162048	0.709515683
CATSPERD	7	1	0.272823628	3.74796884	170.1247361	0.475162048	0.709515683

CIC	7	1	0.272823628	3.74796884	170.1247361	0.475162048	0.709515683
CNOT4	7	1	0.272823628	3.74796884	170.1247361	0.475162048	0.709515683
DDO	7	1	0.272823628	3.74796884	170.1247361	0.475162048	0.709515683
EFR3A	7	1	0.272823628	3.74796884	170.1247361	0.475162048	0.709515683
FRS2	7	1	0.272823628	3.74796884	170.1247361	0.475162048	0.709515683
FRZB	7	1	0.272823628	3.74796884	170.1247361	0.475162048	0.709515683
HK2	7	1	0.272823628	3.74796884	170.1247361	0.475162048	0.709515683
HSPA1L	7	1	0.272823628	3.74796884	170.1247361	0.475162048	0.709515683
IFIH1	7	1	0.272823628	3.74796884	170.1247361	0.475162048	0.709515683
INTS4	7	1	0.272823628	3.74796884	170.1247361	0.475162048	0.709515683
INTS5	7	1	0.272823628	3.74796884	170.1247361	0.475162048	0.709515683
MOGAT3	7	1	0.272823628	3.74796884	170.1247361	0.475162048	0.709515683
MORC3	7	1	0.272823628	3.74796884	170.1247361	0.475162048	0.709515683
NFATC1	7	1	0.272823628	3.74796884	170.1247361	0.475162048	0.709515683
OR3A1	7	1	0.272823628	3.74796884	170.1247361	0.475162048	0.709515683
OR51B5	7	1	0.272823628	3.74796884	170.1247361	0.475162048	0.709515683
OR5H6	7	1	0.272823628	3.74796884	170.1247361	0.475162048	0.709515683
PAQR9	7	1	0.272823628	3.74796884	170.1247361	0.475162048	0.709515683
PLA2G4C	7	1	0.272823628	3.74796884	170.1247361	0.475162048	0.709515683
PPP1R13L	7	1	0.272823628	3.74796884	170.1247361	0.475162048	0.709515683
PTGDR	7	1	0.272823628	3.74796884	170.1247361	0.475162048	0.709515683
PVRL1	7	1	0.272823628	3.74796884	170.1247361	0.475162048	0.709515683
SMG5	7	1	0.272823628	3.74796884	170.1247361	0.475162048	0.709515683
SPECC1	7	1	0.272823628	3.74796884	170.1247361	0.475162048	0.709515683
SYNM	7	1	0.272823628	3.74796884	170.1247361	0.475162048	0.709515683
TACR3	7	1	0.272823628	3.74796884	170.1247361	0.475162048	0.709515683
TBC1D8B	7	1	0.272823628	3.74796884	170.1247361	0.475162048	0.709515683
[illegible]MPRSS11I	7	1	0.272823628	3.74796884	170.1247361	0.475162048	0.709515683
TNRC6B	7	1	0.272823628	3.74796884	170.1247361	0.475162048	0.709515683
TRBC2	7	1	0.272823628	3.74796884	170.1247361	0.475162048	0.709515683
UGT1A10	7	1	0.272823628	3.74796884	170.1247361	0.475162048	0.709515683
WDR63	7	1	0.272823628	3.74796884	170.1247361	0.475162048	0.709515683
ZNF16	7	1	0.272823628	3.74796884	170.1247361	0.475162048	0.709515683
ZNF354A	7	1	0.272823628	3.74796884	170.1247361	0.475162048	0.709515683
ADAMTS6	12	3	0.280657245	2.15192273	12.04730579	0.569820725	0.709515683
CHST1	12	3	0.280657245	2.15192273	12.04730579	0.569820725	0.709515683
CKAP5	12	3	0.280657245	2.15192273	12.04730579	0.569820725	0.709515683
CROT	12	3	0.280657245	2.15192273	12.04730579	0.569820725	0.709515683
FAM46D	12	3	0.280657245	2.15192273	12.04730579	0.569820725	0.709515683
HTATSF1	12	3	0.280657245	2.15192273	12.04730579	0.569820725	0.709515683
LDB2	12	3	0.280657245	2.15192273	12.04730579	0.569820725	0.709515683
PCF11	12	3	0.280657245	2.15192273	12.04730579	0.569820725	0.709515683
STK31	12	3	0.280657245	2.15192273	12.04730579	0.569820725	0.709515683
WWC3	12	3	0.280657245	2.15192273	12.04730579	0.569820725	0.709515683
ABCB1	21	16	0.281538133	0.672056975	1.420578794	0.323565847	0.709515683
NHS	13	3	0.284108085	2.338511876	12.97239801	0.630271425	0.709515683
PCDH11Y	13	3	0.284108085	2.338511876	12.97239801	0.630271425	0.709515683
SLC24A2	13	3	0.284108085	2.338511876	12.97239801	0.630271425	0.709515683
ZNF407	13	3	0.284108085	2.338511876	12.97239801	0.630271425	0.709515683
CPS1	31	22	0.285419305	0.717124964	1.34936002	0.386566004	0.709515683
ABCG2	4	5	0.286571317	0.415892857	1.960710922	0.081380916	0.709515683
ACTG2	4	5	0.286571317	0.415892857	1.960710922	0.081380916	0.709515683
ADGRA1	4	5	0.286571317	0.415892857	1.960710922	0.081380916	0.709515683
AFM	4	5	0.286571317	0.415892857	1.960710922	0.081380916	0.709515683
AFP	4	5	0.286571317	0.415892857	1.960710922	0.081380916	0.709515683
AGAP1	4	5	0.286571317	0.415892857	1.960710922	0.081380916	0.709515683
AGTR1	4	5	0.286571317	0.415892857	1.960710922	0.081380916	0.709515683
ATXN1	4	5	0.286571317	0.415892857	1.960710922	0.081380916	0.709515683

B4GALNT1	4	5	0.286571317	0.415892857	1.960710922	0.081380916	0.709515683
BBOX1	4	5	0.286571317	0.415892857	1.960710922	0.081380916	0.709515683
BBS9	4	5	0.286571317	0.415892857	1.960710922	0.081380916	0.709515683
BTBD3	4	5	0.286571317	0.415892857	1.960710922	0.081380916	0.709515683
C9orf131	4	5	0.286571317	0.415892857	1.960710922	0.081380916	0.709515683
CBLN4	4	5	0.286571317	0.415892857	1.960710922	0.081380916	0.709515683
CCDC13	4	5	0.286571317	0.415892857	1.960710922	0.081380916	0.709515683
CD1C	4	5	0.286571317	0.415892857	1.960710922	0.081380916	0.709515683
CD34	4	5	0.286571317	0.415892857	1.960710922	0.081380916	0.709515683
CDH3	4	5	0.286571317	0.415892857	1.960710922	0.081380916	0.709515683
CEP135	4	5	0.286571317	0.415892857	1.960710922	0.081380916	0.709515683
CHD2	4	5	0.286571317	0.415892857	1.960710922	0.081380916	0.709515683
CPD	4	5	0.286571317	0.415892857	1.960710922	0.081380916	0.709515683
CUL3	4	5	0.286571317	0.415892857	1.960710922	0.081380916	0.709515683
DDX1	4	5	0.286571317	0.415892857	1.960710922	0.081380916	0.709515683
DDX11	4	5	0.286571317	0.415892857	1.960710922	0.081380916	0.709515683
DDX27	4	5	0.286571317	0.415892857	1.960710922	0.081380916	0.709515683
DGCR8	4	5	0.286571317	0.415892857	1.960710922	0.081380916	0.709515683
DIS3	4	5	0.286571317	0.415892857	1.960710922	0.081380916	0.709515683
EDRF1	4	5	0.286571317	0.415892857	1.960710922	0.081380916	0.709515683
EPX	4	5	0.286571317	0.415892857	1.960710922	0.081380916	0.709515683
ERCC4	4	5	0.286571317	0.415892857	1.960710922	0.081380916	0.709515683
FAM114A2	4	5	0.286571317	0.415892857	1.960710922	0.081380916	0.709515683
FAM184A	4	5	0.286571317	0.415892857	1.960710922	0.081380916	0.709515683
FOXC2	4	5	0.286571317	0.415892857	1.960710922	0.081380916	0.709515683
FOXO4	4	5	0.286571317	0.415892857	1.960710922	0.081380916	0.709515683
FOXP1	4	5	0.286571317	0.415892857	1.960710922	0.081380916	0.709515683
GALNT5	4	5	0.286571317	0.415892857	1.960710922	0.081380916	0.709515683
GCC1	4	5	0.286571317	0.415892857	1.960710922	0.081380916	0.709515683
GGNBP2	4	5	0.286571317	0.415892857	1.960710922	0.081380916	0.709515683
GIMAP4	4	5	0.286571317	0.415892857	1.960710922	0.081380916	0.709515683
GPD2	4	5	0.286571317	0.415892857	1.960710922	0.081380916	0.709515683
GPLD1	4	5	0.286571317	0.415892857	1.960710922	0.081380916	0.709515683
HPS5	4	5	0.286571317	0.415892857	1.960710922	0.081380916	0.709515683
HSPH1	4	5	0.286571317	0.415892857	1.960710922	0.081380916	0.709515683
IGKV2-30	4	5	0.286571317	0.415892857	1.960710922	0.081380916	0.709515683
IKBKB	4	5	0.286571317	0.415892857	1.960710922	0.081380916	0.709515683
INPP5D	4	5	0.286571317	0.415892857	1.960710922	0.081380916	0.709515683
IQSEC1	4	5	0.286571317	0.415892857	1.960710922	0.081380916	0.709515683
ITGA11	4	5	0.286571317	0.415892857	1.960710922	0.081380916	0.709515683
JPH2	4	5	0.286571317	0.415892857	1.960710922	0.081380916	0.709515683
KCNJ1	4	5	0.286571317	0.415892857	1.960710922	0.081380916	0.709515683
KCNN2	4	5	0.286571317	0.415892857	1.960710922	0.081380916	0.709515683
KCNQ4	4	5	0.286571317	0.415892857	1.960710922	0.081380916	0.709515683
KIAA0196	4	5	0.286571317	0.415892857	1.960710922	0.081380916	0.709515683
KLHL5	4	5	0.286571317	0.415892857	1.960710922	0.081380916	0.709515683
KRT75	4	5	0.286571317	0.415892857	1.960710922	0.081380916	0.709515683
KRTAP27-1	4	5	0.286571317	0.415892857	1.960710922	0.081380916	0.709515683
LARP4	4	5	0.286571317	0.415892857	1.960710922	0.081380916	0.709515683
LILRB3	4	5	0.286571317	0.415892857	1.960710922	0.081380916	0.709515683
LPCAT1	4	5	0.286571317	0.415892857	1.960710922	0.081380916	0.709515683
MAST2	4	5	0.286571317	0.415892857	1.960710922	0.081380916	0.709515683
MAT1A	4	5	0.286571317	0.415892857	1.960710922	0.081380916	0.709515683
MEOX2	4	5	0.286571317	0.415892857	1.960710922	0.081380916	0.709515683
METTL13	4	5	0.286571317	0.415892857	1.960710922	0.081380916	0.709515683
MURC	4	5	0.286571317	0.415892857	1.960710922	0.081380916	0.709515683
MYO1F	4	5	0.286571317	0.415892857	1.960710922	0.081380916	0.709515683
NPHP1	4	5	0.286571317	0.415892857	1.960710922	0.081380916	0.709515683

NPTX2	4	5	0.286571317	0.415892857	1.960710922	0.081380916	0.709515683
NSUN2	4	5	0.286571317	0.415892857	1.960710922	0.081380916	0.709515683
NUDCD1	4	5	0.286571317	0.415892857	1.960710922	0.081380916	0.709515683
OR10S1	4	5	0.286571317	0.415892857	1.960710922	0.081380916	0.709515683
OR1A2	4	5	0.286571317	0.415892857	1.960710922	0.081380916	0.709515683
OR2F1	4	5	0.286571317	0.415892857	1.960710922	0.081380916	0.709515683
OR52E2	4	5	0.286571317	0.415892857	1.960710922	0.081380916	0.709515683
OR8G1	4	5	0.286571317	0.415892857	1.960710922	0.081380916	0.709515683
OTC	4	5	0.286571317	0.415892857	1.960710922	0.081380916	0.709515683
PACRG	4	5	0.286571317	0.415892857	1.960710922	0.081380916	0.709515683
PAX2	4	5	0.286571317	0.415892857	1.960710922	0.081380916	0.709515683
PC	4	5	0.286571317	0.415892857	1.960710922	0.081380916	0.709515683
PCDHAC1	4	5	0.286571317	0.415892857	1.960710922	0.081380916	0.709515683
PCDHGB6	4	5	0.286571317	0.415892857	1.960710922	0.081380916	0.709515683
PGAP1	4	5	0.286571317	0.415892857	1.960710922	0.081380916	0.709515683
PHKA2	4	5	0.286571317	0.415892857	1.960710922	0.081380916	0.709515683
PIGO	4	5	0.286571317	0.415892857	1.960710922	0.081380916	0.709515683
PLCZ1	4	5	0.286571317	0.415892857	1.960710922	0.081380916	0.709515683
PRPF4B	4	5	0.286571317	0.415892857	1.960710922	0.081380916	0.709515683
PTBP2	4	5	0.286571317	0.415892857	1.960710922	0.081380916	0.709515683
RANBP6	4	5	0.286571317	0.415892857	1.960710922	0.081380916	0.709515683
RIT1	4	5	0.286571317	0.415892857	1.960710922	0.081380916	0.709515683
RNF219	4	5	0.286571317	0.415892857	1.960710922	0.081380916	0.709515683
SCAI	4	5	0.286571317	0.415892857	1.960710922	0.081380916	0.709515683
SEC16A	4	5	0.286571317	0.415892857	1.960710922	0.081380916	0.709515683
SH3D19	4	5	0.286571317	0.415892857	1.960710922	0.081380916	0.709515683
SLC4A4	4	5	0.286571317	0.415892857	1.960710922	0.081380916	0.709515683
SLCO1A2	4	5	0.286571317	0.415892857	1.960710922	0.081380916	0.709515683
SMG6	4	5	0.286571317	0.415892857	1.960710922	0.081380916	0.709515683
SSTR1	4	5	0.286571317	0.415892857	1.960710922	0.081380916	0.709515683
STAG1	4	5	0.286571317	0.415892857	1.960710922	0.081380916	0.709515683
STAM	4	5	0.286571317	0.415892857	1.960710922	0.081380916	0.709515683
STAP1	4	5	0.286571317	0.415892857	1.960710922	0.081380916	0.709515683
STT3A	4	5	0.286571317	0.415892857	1.960710922	0.081380916	0.709515683
TAF5L	4	5	0.286571317	0.415892857	1.960710922	0.081380916	0.709515683
TCEAL5	4	5	0.286571317	0.415892857	1.960710922	0.081380916	0.709515683
TMC5	4	5	0.286571317	0.415892857	1.960710922	0.081380916	0.709515683
TMEM67	4	5	0.286571317	0.415892857	1.960710922	0.081380916	0.709515683
TNNI3K	4	5	0.286571317	0.415892857	1.960710922	0.081380916	0.709515683
TNRC6C	4	5	0.286571317	0.415892857	1.960710922	0.081380916	0.709515683
TRIM11	4	5	0.286571317	0.415892857	1.960710922	0.081380916	0.709515683
UBQLN2	4	5	0.286571317	0.415892857	1.960710922	0.081380916	0.709515683
UNC13D	4	5	0.286571317	0.415892857	1.960710922	0.081380916	0.709515683
VAV2	4	5	0.286571317	0.415892857	1.960710922	0.081380916	0.709515683
XRCC6	4	5	0.286571317	0.415892857	1.960710922	0.081380916	0.709515683
ZC3H6	4	5	0.286571317	0.415892857	1.960710922	0.081380916	0.709515683
ZFP82	4	5	0.286571317	0.415892857	1.960710922	0.081380916	0.709515683
ZNF615	4	5	0.286571317	0.415892857	1.960710922	0.081380916	0.709515683
ZNF770	4	5	0.286571317	0.415892857	1.960710922	0.081380916	0.709515683
ZXDB	4	5	0.286571317	0.415892857	1.960710922	0.081380916	0.709515683
PROS1	19	6	0.288668956	1.712253911	5.343522671	0.641354396	0.71437458
LAMA2	33	23	0.297436641	0.730315792	1.353561543	0.399542302	0.734548955
ADAMTSL[illegible]	23	17	0.299219485	0.692821462	1.426361165	0.342545249	0.734548955
ATM	23	17	0.299219485	0.692821462	1.426361165	0.342545249	0.734548955
EYS	23	17	0.299219485	0.692821462	1.426361165	0.342545249	0.734548955
TMTC1	23	17	0.299219485	0.692821462	1.426361165	0.342545249	0.734548955
UBR4	23	17	0.299219485	0.692821462	1.426361165	0.342545249	0.734548955
FAM71B	15	12	0.299707009	0.643907843	1.546167915	0.273860417	0.734548955

GABRA2	15	12	0.299707009	0.643907843	1.546167915	0.273860417	0.734548955
IL1RAPL1	15	12	0.299707009	0.643907843	1.546167915	0.273860417	0.734548955
ITPR2	15	12	0.299707009	0.643907843	1.546167915	0.273860417	0.734548955
MED12L	15	12	0.299707009	0.643907843	1.546167915	0.273860417	0.734548955
NLRP4	15	12	0.299707009	0.643907843	1.546167915	0.273860417	0.734548955
NRG3	15	12	0.299707009	0.643907843	1.546167915	0.273860417	0.734548955
OR5D13	15	12	0.299707009	0.643907843	1.546167915	0.273860417	0.734548955
PCDHGA5	15	12	0.299707009	0.643907843	1.546167915	0.273860417	0.734548955
SCN11A	15	12	0.299707009	0.643907843	1.546167915	0.273860417	0.734548955
SCN7A	15	12	0.299707009	0.643907843	1.546167915	0.273860417	0.734548955
SLC17A6	15	12	0.299707009	0.643907843	1.546167915	0.273860417	0.734548955
ZSWIM2	15	12	0.299707009	0.643907843	1.546167915	0.273860417	0.734548955
ABCA8	9	8	0.303074399	0.582925788	1.772079832	0.195474664	0.734548955
ATF7IP	9	8	0.303074399	0.582925788	1.772079832	0.195474664	0.734548955
C2orf16	9	8	0.303074399	0.582925788	1.772079832	0.195474664	0.734548955
C2orf78	9	8	0.303074399	0.582925788	1.772079832	0.195474664	0.734548955
CDH20	9	8	0.303074399	0.582925788	1.772079832	0.195474664	0.734548955
CFAP58	9	8	0.303074399	0.582925788	1.772079832	0.195474664	0.734548955
CPZ	9	8	0.303074399	0.582925788	1.772079832	0.195474664	0.734548955
EBF3	9	8	0.303074399	0.582925788	1.772079832	0.195474664	0.734548955
GAB4	9	8	0.303074399	0.582925788	1.772079832	0.195474664	0.734548955
HIVEP1	9	8	0.303074399	0.582925788	1.772079832	0.195474664	0.734548955
LTN1	9	8	0.303074399	0.582925788	1.772079832	0.195474664	0.734548955
MYH15	9	8	0.303074399	0.582925788	1.772079832	0.195474664	0.734548955
NCOA2	9	8	0.303074399	0.582925788	1.772079832	0.195474664	0.734548955
NOVA1	9	8	0.303074399	0.582925788	1.772079832	0.195474664	0.734548955
OR4C13	9	8	0.303074399	0.582925788	1.772079832	0.195474664	0.734548955
OR8K3	9	8	0.303074399	0.582925788	1.772079832	0.195474664	0.734548955
PDE4DIP	9	8	0.303074399	0.582925788	1.772079832	0.195474664	0.734548955
PHLDB2	9	8	0.303074399	0.582925788	1.772079832	0.195474664	0.734548955
PLCH1	9	8	0.303074399	0.582925788	1.772079832	0.195474664	0.734548955
POU3F4	9	8	0.303074399	0.582925788	1.772079832	0.195474664	0.734548955
PSME4	9	8	0.303074399	0.582925788	1.772079832	0.195474664	0.734548955
RB1CC1	9	8	0.303074399	0.582925788	1.772079832	0.195474664	0.734548955
SLIT1	9	8	0.303074399	0.582925788	1.772079832	0.195474664	0.734548955
TGFBR3	9	8	0.303074399	0.582925788	1.772079832	0.195474664	0.734548955
ZNF462	9	8	0.303074399	0.582925788	1.772079832	0.195474664	0.734548955
ZZEF1	9	8	0.303074399	0.582925788	1.772079832	0.195474664	0.734548955
ADGRL3	35	24	0.308790232	0.742362054	1.357021684	0.41164497	0.740685265
TNN	36	25	0.313809348	0.730581936	1.321986066	0.408803059	0.740685265
ITGAD	22	7	0.313940136	1.705914888	4.8316296	0.685362222	0.740685265
CTNND2	25	18	0.315576077	0.711296022	1.430497523	0.360000594	0.740685265
EPHA6	25	18	0.315576077	0.711296022	1.430497523	0.360000594	0.740685265
KCNT2	25	18	0.315576077	0.711296022	1.430497523	0.360000594	0.740685265
NBAS	25	18	0.315576077	0.711296022	1.430497523	0.360000594	0.740685265
NLRP14	25	18	0.315576077	0.711296022	1.430497523	0.360000594	0.740685265
DPY19L2	14	4	0.320476591	1.884187881	7.987590013	0.579475505	0.740685265
EYA1	14	4	0.320476591	1.884187881	7.987590013	0.579475505	0.740685265
NAALAD2	14	4	0.320476591	1.884187881	7.987590013	0.579475505	0.740685265
OR8I2	14	4	0.320476591	1.884187881	7.987590013	0.579475505	0.740685265
PSKH2	14	4	0.320476591	1.884187881	7.987590013	0.579475505	0.740685265
STAG2	14	4	0.320476591	1.884187881	7.987590013	0.579475505	0.740685265
UNC5C	14	4	0.320476591	1.884187881	7.987590013	0.579475505	0.740685265
WNK1	14	4	0.320476591	1.884187881	7.987590013	0.579475505	0.740685265
MGA	33	12	0.323585034	1.502283732	3.288461542	0.731082891	0.740685265
ABCC11	17	13	0.324258156	0.673683344	1.549993805	0.299458976	0.740685265
BCORL1	17	13	0.324258156	0.673683344	1.549993805	0.299458976	0.740685265
DMXL1	17	13	0.324258156	0.673683344	1.549993805	0.299458976	0.740685265

FSCB	17	13	0.324258156	0.673683344	1.549993805	0.299458976	0.740685265
NID2	17	13	0.324258156	0.673683344	1.549993805	0.299458976	0.740685265
OR6F1	17	13	0.324258156	0.673683344	1.549993805	0.299458976	0.740685265
PCDHB12	17	13	0.324258156	0.673683344	1.549993805	0.299458976	0.740685265
PKD1L2	17	13	0.324258156	0.673683344	1.549993805	0.299458976	0.740685265
TDRD6	15	4	0.324460751	2.025119224	8.51848884	0.631494768	0.740685265
ANKK1	5	5	0.325083204	0.521226843	2.299548017	0.118141423	0.740685265
ARHGAP2(	5	5	0.325083204	0.521226843	2.299548017	0.118141423	0.740685265
ARHGAP2<	5	5	0.325083204	0.521226843	2.299548017	0.118141423	0.740685265
B4GALNT4	5	5	0.325083204	0.521226843	2.299548017	0.118141423	0.740685265
BOC	5	5	0.325083204	0.521226843	2.299548017	0.118141423	0.740685265
CACHD1	5	5	0.325083204	0.521226843	2.299548017	0.118141423	0.740685265
CAPRIN1	5	5	0.325083204	0.521226843	2.299548017	0.118141423	0.740685265
CATSPER1	5	5	0.325083204	0.521226843	2.299548017	0.118141423	0.740685265
CCDC7	5	5	0.325083204	0.521226843	2.299548017	0.118141423	0.740685265
CD86	5	5	0.325083204	0.521226843	2.299548017	0.118141423	0.740685265
CHAF1A	5	5	0.325083204	0.521226843	2.299548017	0.118141423	0.740685265
CLEC9A	5	5	0.325083204	0.521226843	2.299548017	0.118141423	0.740685265
CPA1	5	5	0.325083204	0.521226843	2.299548017	0.118141423	0.740685265
CPNE8	5	5	0.325083204	0.521226843	2.299548017	0.118141423	0.740685265
SGALNAC	5	5	0.325083204	0.521226843	2.299548017	0.118141423	0.740685265
CYFIP1	5	5	0.325083204	0.521226843	2.299548017	0.118141423	0.740685265
CYFIP2	5	5	0.325083204	0.521226843	2.299548017	0.118141423	0.740685265
CYSLTR2	5	5	0.325083204	0.521226843	2.299548017	0.118141423	0.740685265
DDB1	5	5	0.325083204	0.521226843	2.299548017	0.118141423	0.740685265
DDX4	5	5	0.325083204	0.521226843	2.299548017	0.118141423	0.740685265
EPHA10	5	5	0.325083204	0.521226843	2.299548017	0.118141423	0.740685265
F11	5	5	0.325083204	0.521226843	2.299548017	0.118141423	0.740685265
FBXW7	5	5	0.325083204	0.521226843	2.299548017	0.118141423	0.740685265
?GT-TNNI3	5	5	0.325083204	0.521226843	2.299548017	0.118141423	0.740685265
FTMT	5	5	0.325083204	0.521226843	2.299548017	0.118141423	0.740685265
GALNT3	5	5	0.325083204	0.521226843	2.299548017	0.118141423	0.740685265
GLTSCR1L	5	5	0.325083204	0.521226843	2.299548017	0.118141423	0.740685265
GLUD2	5	5	0.325083204	0.521226843	2.299548017	0.118141423	0.740685265
GOLGA2	5	5	0.325083204	0.521226843	2.299548017	0.118141423	0.740685265
HERC5	5	5	0.325083204	0.521226843	2.299548017	0.118141423	0.740685265
HOXA13	5	5	0.325083204	0.521226843	2.299548017	0.118141423	0.740685265
HOXB1	5	5	0.325083204	0.521226843	2.299548017	0.118141423	0.740685265
HOXD10	5	5	0.325083204	0.521226843	2.299548017	0.118141423	0.740685265
IGHMBP2	5	5	0.325083204	0.521226843	2.299548017	0.118141423	0.740685265
IGKV3D-2(	5	5	0.325083204	0.521226843	2.299548017	0.118141423	0.740685265
ITGA10	5	5	0.325083204	0.521226843	2.299548017	0.118141423	0.740685265
KCNJ16	5	5	0.325083204	0.521226843	2.299548017	0.118141423	0.740685265
KCNMB1	5	5	0.325083204	0.521226843	2.299548017	0.118141423	0.740685265
KIAA0319	5	5	0.325083204	0.521226843	2.299548017	0.118141423	0.740685265
KIAA0586	5	5	0.325083204	0.521226843	2.299548017	0.118141423	0.740685265
KIAA1715	5	5	0.325083204	0.521226843	2.299548017	0.118141423	0.740685265
KIR3DX1	5	5	0.325083204	0.521226843	2.299548017	0.118141423	0.740685265
KMT2E	5	5	0.325083204	0.521226843	2.299548017	0.118141423	0.740685265
KTN1	5	5	0.325083204	0.521226843	2.299548017	0.118141423	0.740685265
LAD1	5	5	0.325083204	0.521226843	2.299548017	0.118141423	0.740685265
LAMC2	5	5	0.325083204	0.521226843	2.299548017	0.118141423	0.740685265
LCE1F	5	5	0.325083204	0.521226843	2.299548017	0.118141423	0.740685265
LRP5	5	5	0.325083204	0.521226843	2.299548017	0.118141423	0.740685265
LY9	5	5	0.325083204	0.521226843	2.299548017	0.118141423	0.740685265
MAGEA10	5	5	0.325083204	0.521226843	2.299548017	0.118141423	0.740685265
MAP3K13	5	5	0.325083204	0.521226843	2.299548017	0.118141423	0.740685265
MAP3K5	5	5	0.325083204	0.521226843	2.299548017	0.118141423	0.740685265

MAPKBP1	5	5	0.325083204	0.521226843	2.299548017	0.118141423	0.740685265
MB21D2	5	5	0.325083204	0.521226843	2.299548017	0.118141423	0.740685265
MCM3AP	5	5	0.325083204	0.521226843	2.299548017	0.118141423	0.740685265
MERTK	5	5	0.325083204	0.521226843	2.299548017	0.118141423	0.740685265
MROH8	5	5	0.325083204	0.521226843	2.299548017	0.118141423	0.740685265
MYF6	5	5	0.325083204	0.521226843	2.299548017	0.118141423	0.740685265
MYLK2	5	5	0.325083204	0.521226843	2.299548017	0.118141423	0.740685265
MYO1B	5	5	0.325083204	0.521226843	2.299548017	0.118141423	0.740685265
NPNT	5	5	0.325083204	0.521226843	2.299548017	0.118141423	0.740685265
NR3C1	5	5	0.325083204	0.521226843	2.299548017	0.118141423	0.740685265
NWD1	5	5	0.325083204	0.521226843	2.299548017	0.118141423	0.740685265
OPRM1	5	5	0.325083204	0.521226843	2.299548017	0.118141423	0.740685265
OR4K14	5	5	0.325083204	0.521226843	2.299548017	0.118141423	0.740685265
OR51A4	5	5	0.325083204	0.521226843	2.299548017	0.118141423	0.740685265
OR52A5	5	5	0.325083204	0.521226843	2.299548017	0.118141423	0.740685265
OR5H1	5	5	0.325083204	0.521226843	2.299548017	0.118141423	0.740685265
OR5M1	5	5	0.325083204	0.521226843	2.299548017	0.118141423	0.740685265
OTUD7B	5	5	0.325083204	0.521226843	2.299548017	0.118141423	0.740685265
PADI4	5	5	0.325083204	0.521226843	2.299548017	0.118141423	0.740685265
PBXIP1	5	5	0.325083204	0.521226843	2.299548017	0.118141423	0.740685265
PDYN	5	5	0.325083204	0.521226843	2.299548017	0.118141423	0.740685265
PIK3CB	5	5	0.325083204	0.521226843	2.299548017	0.118141423	0.740685265
PIWIL3	5	5	0.325083204	0.521226843	2.299548017	0.118141423	0.740685265
PKDREJ	5	5	0.325083204	0.521226843	2.299548017	0.118141423	0.740685265
PNPLA7	5	5	0.325083204	0.521226843	2.299548017	0.118141423	0.740685265
PPP1R26	5	5	0.325083204	0.521226843	2.299548017	0.118141423	0.740685265
PPP3CA	5	5	0.325083204	0.521226843	2.299548017	0.118141423	0.740685265
PRDM5	5	5	0.325083204	0.521226843	2.299548017	0.118141423	0.740685265
PTK2B	5	5	0.325083204	0.521226843	2.299548017	0.118141423	0.740685265
RFTN2	5	5	0.325083204	0.521226843	2.299548017	0.118141423	0.740685265
RHAG	5	5	0.325083204	0.521226843	2.299548017	0.118141423	0.740685265
RHBDF2	5	5	0.325083204	0.521226843	2.299548017	0.118141423	0.740685265
SBSN	5	5	0.325083204	0.521226843	2.299548017	0.118141423	0.740685265
SCFD2	5	5	0.325083204	0.521226843	2.299548017	0.118141423	0.740685265
SEL1L	5	5	0.325083204	0.521226843	2.299548017	0.118141423	0.740685265
SLFN11	5	5	0.325083204	0.521226843	2.299548017	0.118141423	0.740685265
SNAI2	5	5	0.325083204	0.521226843	2.299548017	0.118141423	0.740685265
SNX19	5	5	0.325083204	0.521226843	2.299548017	0.118141423	0.740685265
SPERT	5	5	0.325083204	0.521226843	2.299548017	0.118141423	0.740685265
SPOCK3	5	5	0.325083204	0.521226843	2.299548017	0.118141423	0.740685265
SYTL5	5	5	0.325083204	0.521226843	2.299548017	0.118141423	0.740685265
TAAR5	5	5	0.325083204	0.521226843	2.299548017	0.118141423	0.740685265
TAT	5	5	0.325083204	0.521226843	2.299548017	0.118141423	0.740685265
TEC	5	5	0.325083204	0.521226843	2.299548017	0.118141423	0.740685265
THSD4	5	5	0.325083204	0.521226843	2.299548017	0.118141423	0.740685265
TMEM132A	5	5	0.325083204	0.521226843	2.299548017	0.118141423	0.740685265
TNIK	5	5	0.325083204	0.521226843	2.299548017	0.118141423	0.740685265
TOP3A	5	5	0.325083204	0.521226843	2.299548017	0.118141423	0.740685265
TPTE2	5	5	0.325083204	0.521226843	2.299548017	0.118141423	0.740685265
TRPM7	5	5	0.325083204	0.521226843	2.299548017	0.118141423	0.740685265
TSPEAR	5	5	0.325083204	0.521226843	2.299548017	0.118141423	0.740685265
UBE2O	5	5	0.325083204	0.521226843	2.299548017	0.118141423	0.740685265
USP53	5	5	0.325083204	0.521226843	2.299548017	0.118141423	0.740685265
VIT	5	5	0.325083204	0.521226843	2.299548017	0.118141423	0.740685265
VRTN	5	5	0.325083204	0.521226843	2.299548017	0.118141423	0.740685265
VSTM2A	5	5	0.325083204	0.521226843	2.299548017	0.118141423	0.740685265
WDPCP	5	5	0.325083204	0.521226843	2.299548017	0.118141423	0.740685265
ZNF225	5	5	0.325083204	0.521226843	2.299548017	0.118141423	0.740685265

ZNF80	5	5	0.325083204	0.521226843	2.299548017	0.118141423	0.740685265
ZNF813	5	5	0.325083204	0.521226843	2.299548017	0.118141423	0.740685265
ZNFX1	5	5	0.325083204	0.521226843	2.299548017	0.118141423	0.740685265
CSMD2	40	27	0.333275456	0.751722991	1.329132032	0.430236804	0.759023722
MXRA5	41	27	0.339368566	0.773206452	1.364180306	0.443778616	0.769797491
ACACB	11	9	0.341615206	0.633325429	1.767631609	0.233170507	0.769797491
AGBL1	11	9	0.341615206	0.633325429	1.767631609	0.233170507	0.769797491
ANO5	11	9	0.341615206	0.633325429	1.767631609	0.233170507	0.769797491
BRCA1	11	9	0.341615206	0.633325429	1.767631609	0.233170507	0.769797491
BRINP1	11	9	0.341615206	0.633325429	1.767631609	0.233170507	0.769797491
CAPN6	11	9	0.341615206	0.633325429	1.767631609	0.233170507	0.769797491
GHV1OR21-	11	9	0.341615206	0.633325429	1.767631609	0.233170507	0.769797491
LILRB4	11	9	0.341615206	0.633325429	1.767631609	0.233170507	0.769797491
MADD	11	9	0.341615206	0.633325429	1.767631609	0.233170507	0.769797491
MARCO	11	9	0.341615206	0.633325429	1.767631609	0.233170507	0.769797491
MORC1	11	9	0.341615206	0.633325429	1.767631609	0.233170507	0.769797491
OR8H2	11	9	0.341615206	0.633325429	1.767631609	0.233170507	0.769797491
P2RY10	11	9	0.341615206	0.633325429	1.767631609	0.233170507	0.769797491
ROCK1	11	9	0.341615206	0.633325429	1.767631609	0.233170507	0.769797491
RTTN	11	9	0.341615206	0.633325429	1.767631609	0.233170507	0.769797491
SCN8A	11	9	0.341615206	0.633325429	1.767631609	0.233170507	0.769797491
SLC9C2	11	9	0.341615206	0.633325429	1.767631609	0.233170507	0.769797491
TMEM132E	11	9	0.341615206	0.633325429	1.767631609	0.233170507	0.769797491
TNXB	11	9	0.341615206	0.633325429	1.767631609	0.233170507	0.769797491
TUBA3C	11	9	0.341615206	0.633325429	1.767631609	0.233170507	0.769797491
USP9X	11	9	0.341615206	0.633325429	1.767631609	0.233170507	0.769797491
WDR7	11	9	0.341615206	0.633325429	1.767631609	0.233170507	0.769797491
ZBED9	11	9	0.341615206	0.633325429	1.767631609	0.233170507	0.769797491
CCIN	9	2	0.344559376	2.41169082	23.1897417	0.49118074	0.769797491
CD1A	9	2	0.344559376	2.41169082	23.1897417	0.49118074	0.769797491
DBX2	9	2	0.344559376	2.41169082	23.1897417	0.49118074	0.769797491
DENND1A	9	2	0.344559376	2.41169082	23.1897417	0.49118074	0.769797491
DGKH	9	2	0.344559376	2.41169082	23.1897417	0.49118074	0.769797491
ESRRB	9	2	0.344559376	2.41169082	23.1897417	0.49118074	0.769797491
GPC5	9	2	0.344559376	2.41169082	23.1897417	0.49118074	0.769797491
GPRIN1	9	2	0.344559376	2.41169082	23.1897417	0.49118074	0.769797491
KCNA6	9	2	0.344559376	2.41169082	23.1897417	0.49118074	0.769797491
KIAA1614	9	2	0.344559376	2.41169082	23.1897417	0.49118074	0.769797491
KIF16B	9	2	0.344559376	2.41169082	23.1897417	0.49118074	0.769797491
6-Mar	9	2	0.344559376	2.41169082	23.1897417	0.49118074	0.769797491
NCOA3	9	2	0.344559376	2.41169082	23.1897417	0.49118074	0.769797491
PGBD2	9	2	0.344559376	2.41169082	23.1897417	0.49118074	0.769797491
PLEKHA6	9	2	0.344559376	2.41169082	23.1897417	0.49118074	0.769797491
PRAMEF2	9	2	0.344559376	2.41169082	23.1897417	0.49118074	0.769797491
PTPRS	9	2	0.344559376	2.41169082	23.1897417	0.49118074	0.769797491
SLC22A6	9	2	0.344559376	2.41169082	23.1897417	0.49118074	0.769797491
SMCHD1	9	2	0.344559376	2.41169082	23.1897417	0.49118074	0.769797491
SOS1	9	2	0.344559376	2.41169082	23.1897417	0.49118074	0.769797491
XKR6	9	2	0.344559376	2.41169082	23.1897417	0.49118074	0.769797491
MRC1	29	20	0.344870225	0.742812932	1.435225826	0.391026923	0.770166459
CPED1	19	14	0.34630754	0.699231191	1.550953971	0.322504063	0.773049687
FLT3	16	5	0.354125679	1.723745705	6.125410295	0.589816791	0.775496942
ABCC2	6	6	0.356074749	0.519711072	1.977241141	0.136601962	0.775496942
ACCSL	6	6	0.356074749	0.519711072	1.977241141	0.136601962	0.775496942
ADCYAP1R	6	6	0.356074749	0.519711072	1.977241141	0.136601962	0.775496942
ADGRF2	6	6	0.356074749	0.519711072	1.977241141	0.136601962	0.775496942
AKAP1	6	6	0.356074749	0.519711072	1.977241141	0.136601962	0.775496942
ALDH8A1	6	6	0.356074749	0.519711072	1.977241141	0.136601962	0.775496942

ANAPC1	6	6	0.356074749	0.519711072	1.977241141	0.136601962	0.775496942
APBB1IP	6	6	0.356074749	0.519711072	1.977241141	0.136601962	0.775496942
AQR	6	6	0.356074749	0.519711072	1.977241141	0.136601962	0.775496942
C12orf40	6	6	0.356074749	0.519711072	1.977241141	0.136601962	0.775496942
CELSR2	6	6	0.356074749	0.519711072	1.977241141	0.136601962	0.775496942
CNR1	6	6	0.356074749	0.519711072	1.977241141	0.136601962	0.775496942
CRMP1	6	6	0.356074749	0.519711072	1.977241141	0.136601962	0.775496942
CTAGE1	6	6	0.356074749	0.519711072	1.977241141	0.136601962	0.775496942
CYP2A13	6	6	0.356074749	0.519711072	1.977241141	0.136601962	0.775496942
DDX10	6	6	0.356074749	0.519711072	1.977241141	0.136601962	0.775496942
DICER1	6	6	0.356074749	0.519711072	1.977241141	0.136601962	0.775496942
DIP2B	6	6	0.356074749	0.519711072	1.977241141	0.136601962	0.775496942
EBF1	6	6	0.356074749	0.519711072	1.977241141	0.136601962	0.775496942
EXOC4	6	6	0.356074749	0.519711072	1.977241141	0.136601962	0.775496942
GRIK5	6	6	0.356074749	0.519711072	1.977241141	0.136601962	0.775496942
HRH2	6	6	0.356074749	0.519711072	1.977241141	0.136601962	0.775496942
HTT	6	6	0.356074749	0.519711072	1.977241141	0.136601962	0.775496942
IRX2	6	6	0.356074749	0.519711072	1.977241141	0.136601962	0.775496942
ITGA7	6	6	0.356074749	0.519711072	1.977241141	0.136601962	0.775496942
JPH1	6	6	0.356074749	0.519711072	1.977241141	0.136601962	0.775496942
KDM2B	6	6	0.356074749	0.519711072	1.977241141	0.136601962	0.775496942
KIRREL	6	6	0.356074749	0.519711072	1.977241141	0.136601962	0.775496942
LIFR	6	6	0.356074749	0.519711072	1.977241141	0.136601962	0.775496942
LRIT1	6	6	0.356074749	0.519711072	1.977241141	0.136601962	0.775496942
MAGEB2	6	6	0.356074749	0.519711072	1.977241141	0.136601962	0.775496942
MEPE	6	6	0.356074749	0.519711072	1.977241141	0.136601962	0.775496942
NUF2	6	6	0.356074749	0.519711072	1.977241141	0.136601962	0.775496942
OR10G4	6	6	0.356074749	0.519711072	1.977241141	0.136601962	0.775496942
OR10T2	6	6	0.356074749	0.519711072	1.977241141	0.136601962	0.775496942
OR13C5	6	6	0.356074749	0.519711072	1.977241141	0.136601962	0.775496942
OR2A14	6	6	0.356074749	0.519711072	1.977241141	0.136601962	0.775496942
OR51Q1	6	6	0.356074749	0.519711072	1.977241141	0.136601962	0.775496942
OR56A3	6	6	0.356074749	0.519711072	1.977241141	0.136601962	0.775496942
OR9G4	6	6	0.356074749	0.519711072	1.977241141	0.136601962	0.775496942
PCDHGA1(	6	6	0.356074749	0.519711072	1.977241141	0.136601962	0.775496942
PIKFYVE	6	6	0.356074749	0.519711072	1.977241141	0.136601962	0.775496942
PLXDC2	6	6	0.356074749	0.519711072	1.977241141	0.136601962	0.775496942
PRAMEF12	6	6	0.356074749	0.519711072	1.977241141	0.136601962	0.775496942
PRKG2	6	6	0.356074749	0.519711072	1.977241141	0.136601962	0.775496942
PTPRM	6	6	0.356074749	0.519711072	1.977241141	0.136601962	0.775496942
SASH1	6	6	0.356074749	0.519711072	1.977241141	0.136601962	0.775496942
SLC8A2	6	6	0.356074749	0.519711072	1.977241141	0.136601962	0.775496942
STK11IP	6	6	0.356074749	0.519711072	1.977241141	0.136601962	0.775496942
TLN1	6	6	0.356074749	0.519711072	1.977241141	0.136601962	0.775496942
TTC12	6	6	0.356074749	0.519711072	1.977241141	0.136601962	0.775496942
UBR1	6	6	0.356074749	0.519711072	1.977241141	0.136601962	0.775496942
UGT3A2	6	6	0.356074749	0.519711072	1.977241141	0.136601962	0.775496942
ULK4	6	6	0.356074749	0.519711072	1.977241141	0.136601962	0.775496942
VANGL2	6	6	0.356074749	0.519711072	1.977241141	0.136601962	0.775496942
ZNF585B	6	6	0.356074749	0.519711072	1.977241141	0.136601962	0.775496942
INSRR	20	15	0.356278872	0.684853062	1.481005861	0.323054927	0.775496942
TRPC4	20	15	0.356278872	0.684853062	1.481005861	0.323054927	0.775496942
CDH12	31	21	0.358041046	0.756318491	1.436358418	0.404897124	0.775496942
ADNP2	12	10	0.358553637	0.619970897	1.639620661	0.23961698	0.775496942
ATP10D	12	10	0.358553637	0.619970897	1.639620661	0.23961698	0.775496942
CACNA1A	12	10	0.358553637	0.619970897	1.639620661	0.23961698	0.775496942
CALCR	12	10	0.358553637	0.619970897	1.639620661	0.23961698	0.775496942
CASS4	12	10	0.358553637	0.619970897	1.639620661	0.23961698	0.775496942

CELSR3	12	10	0.358553637	0.619970897	1.639620661	0.23961698	0.775496942
CLCA2	12	10	0.358553637	0.619970897	1.639620661	0.23961698	0.775496942
EP400	12	10	0.358553637	0.619970897	1.639620661	0.23961698	0.775496942
FCRL2	12	10	0.358553637	0.619970897	1.639620661	0.23961698	0.775496942
GABRA6	12	10	0.358553637	0.619970897	1.639620661	0.23961698	0.775496942
KCNA5	12	10	0.358553637	0.619970897	1.639620661	0.23961698	0.775496942
KIF5A	12	10	0.358553637	0.619970897	1.639620661	0.23961698	0.775496942
LILRB1	12	10	0.358553637	0.619970897	1.639620661	0.23961698	0.775496942
NRAP	12	10	0.358553637	0.619970897	1.639620661	0.23961698	0.775496942
OR5W2	12	10	0.358553637	0.619970897	1.639620661	0.23961698	0.775496942
PTPRH	12	10	0.358553637	0.619970897	1.639620661	0.23961698	0.775496942
ADAMTS16	21	15	0.366228735	0.721398314	1.550036947	0.343442644	0.791773045
PLCB1	27	10	0.371643006	1.463333488	3.477874997	0.665786347	0.803150013
ANO3	13	10	0.37432509	0.673682857	1.757191295	0.266218889	0.804376986
DNAH1	13	10	0.37432509	0.673682857	1.757191295	0.266218889	0.804376986
KIF21A	13	10	0.37432509	0.673682857	1.757191295	0.266218889	0.804376986
NLRP8	13	10	0.37432509	0.673682857	1.757191295	0.266218889	0.804376986
NUP210	13	10	0.37432509	0.673682857	1.757191295	0.266218889	0.804376986
OR5J2	13	10	0.37432509	0.673682857	1.757191295	0.266218889	0.804376986
PASD1	13	10	0.37432509	0.673682857	1.757191295	0.266218889	0.804376986
PLCE1	13	10	0.37432509	0.673682857	1.757191295	0.266218889	0.804376986
RGS22	13	10	0.37432509	0.673682857	1.757191295	0.266218889	0.804376986
SLITRK5	13	10	0.37432509	0.673682857	1.757191295	0.266218889	0.804376986
TRIM58	13	10	0.37432509	0.673682857	1.757191295	0.266218889	0.804376986
TYR	13	10	0.37432509	0.673682857	1.757191295	0.266218889	0.804376986
WSCD2	13	10	0.37432509	0.673682857	1.757191295	0.266218889	0.804376986
MYH8	34	23	0.374341163	0.75499933	1.395158112	0.414637261	0.804376986
NETO1	18	6	0.383018738	1.616957169	5.075375252	0.600019105	0.80998142
SYNE2	36	24	0.385112885	0.766184628	1.396781001	0.426367744	0.80998142
ABCG4	7	6	0.386449705	0.608037685	2.228417334	0.171821051	0.80998142
ACTC1	7	6	0.386449705	0.608037685	2.228417334	0.171821051	0.80998142
AHSG	7	6	0.386449705	0.608037685	2.228417334	0.171821051	0.80998142
ANKRD44	7	6	0.386449705	0.608037685	2.228417334	0.171821051	0.80998142
ASB17	7	6	0.386449705	0.608037685	2.228417334	0.171821051	0.80998142
BAIAP3	7	6	0.386449705	0.608037685	2.228417334	0.171821051	0.80998142
BBX	7	6	0.386449705	0.608037685	2.228417334	0.171821051	0.80998142
BHLHB9	7	6	0.386449705	0.608037685	2.228417334	0.171821051	0.80998142
CEP192	7	6	0.386449705	0.608037685	2.228417334	0.171821051	0.80998142
CILP	7	6	0.386449705	0.608037685	2.228417334	0.171821051	0.80998142
DPYSL4	7	6	0.386449705	0.608037685	2.228417334	0.171821051	0.80998142
ENPP3	7	6	0.386449705	0.608037685	2.228417334	0.171821051	0.80998142
ERCC6	7	6	0.386449705	0.608037685	2.228417334	0.171821051	0.80998142
F9	7	6	0.386449705	0.608037685	2.228417334	0.171821051	0.80998142
FAM65B	7	6	0.386449705	0.608037685	2.228417334	0.171821051	0.80998142
HEATR5A	7	6	0.386449705	0.608037685	2.228417334	0.171821051	0.80998142
HELQ	7	6	0.386449705	0.608037685	2.228417334	0.171821051	0.80998142
HOXA3	7	6	0.386449705	0.608037685	2.228417334	0.171821051	0.80998142
IRGC	7	6	0.386449705	0.608037685	2.228417334	0.171821051	0.80998142
JAK2	7	6	0.386449705	0.608037685	2.228417334	0.171821051	0.80998142
KCNH6	7	6	0.386449705	0.608037685	2.228417334	0.171821051	0.80998142
KCNJ12	7	6	0.386449705	0.608037685	2.228417334	0.171821051	0.80998142
KCNK10	7	6	0.386449705	0.608037685	2.228417334	0.171821051	0.80998142
KIAA1324L	7	6	0.386449705	0.608037685	2.228417334	0.171821051	0.80998142
KIF4A	7	6	0.386449705	0.608037685	2.228417334	0.171821051	0.80998142
LONRF3	7	6	0.386449705	0.608037685	2.228417334	0.171821051	0.80998142
LRRC15	7	6	0.386449705	0.608037685	2.228417334	0.171821051	0.80998142
MAGEB1	7	6	0.386449705	0.608037685	2.228417334	0.171821051	0.80998142
MAMLD1	7	6	0.386449705	0.608037685	2.228417334	0.171821051	0.80998142

MGAT3	7	6	0.386449705	0.608037685	2.228417334	0.171821051	0.80998142
MIOS	7	6	0.386449705	0.608037685	2.228417334	0.171821051	0.80998142
MON2	7	6	0.386449705	0.608037685	2.228417334	0.171821051	0.80998142
MPDZ	7	6	0.386449705	0.608037685	2.228417334	0.171821051	0.80998142
NIN	7	6	0.386449705	0.608037685	2.228417334	0.171821051	0.80998142
NLRP1	7	6	0.386449705	0.608037685	2.228417334	0.171821051	0.80998142
NPHP4	7	6	0.386449705	0.608037685	2.228417334	0.171821051	0.80998142
NPR1	7	6	0.386449705	0.608037685	2.228417334	0.171821051	0.80998142
NUMA1	7	6	0.386449705	0.608037685	2.228417334	0.171821051	0.80998142
OR5A1	7	6	0.386449705	0.608037685	2.228417334	0.171821051	0.80998142
OR6Q1	7	6	0.386449705	0.608037685	2.228417334	0.171821051	0.80998142
OR6T1	7	6	0.386449705	0.608037685	2.228417334	0.171821051	0.80998142
OSBPL3	7	6	0.386449705	0.608037685	2.228417334	0.171821051	0.80998142
OTUD7A	7	6	0.386449705	0.608037685	2.228417334	0.171821051	0.80998142
PSG1	7	6	0.386449705	0.608037685	2.228417334	0.171821051	0.80998142
PSG4	7	6	0.386449705	0.608037685	2.228417334	0.171821051	0.80998142
PTPRR	7	6	0.386449705	0.608037685	2.228417334	0.171821051	0.80998142
RAB3GAP2	7	6	0.386449705	0.608037685	2.228417334	0.171821051	0.80998142
RAPGEF6	7	6	0.386449705	0.608037685	2.228417334	0.171821051	0.80998142
SERPINA4	7	6	0.386449705	0.608037685	2.228417334	0.171821051	0.80998142
SERPINA6	7	6	0.386449705	0.608037685	2.228417334	0.171821051	0.80998142
SLC22A16	7	6	0.386449705	0.608037685	2.228417334	0.171821051	0.80998142
SLC7A14	7	6	0.386449705	0.608037685	2.228417334	0.171821051	0.80998142
SLC9C1	7	6	0.386449705	0.608037685	2.228417334	0.171821051	0.80998142
SYNE3	7	6	0.386449705	0.608037685	2.228417334	0.171821051	0.80998142
SYTL4	7	6	0.386449705	0.608037685	2.228417334	0.171821051	0.80998142
TECPR2	7	6	0.386449705	0.608037685	2.228417334	0.171821051	0.80998142
TUBB4A	7	6	0.386449705	0.608037685	2.228417334	0.171821051	0.80998142
TUBB8	7	6	0.386449705	0.608037685	2.228417334	0.171821051	0.80998142
XRN1	7	6	0.386449705	0.608037685	2.228417334	0.171821051	0.80998142
ZFP30	7	6	0.386449705	0.608037685	2.228417334	0.171821051	0.80998142
CDH2	14	11	0.387801582	0.657591529	1.640830427	0.270230463	0.811413971
IRS4	14	11	0.387801582	0.657591529	1.640830427	0.270230463	0.811413971
KIF1A	14	11	0.387801582	0.657591529	1.640830427	0.270230463	0.811413971
SLC12A5	14	11	0.387801582	0.657591529	1.640830427	0.270230463	0.811413971
CACNA1C	29	11	0.387900701	1.429665599	3.2601097	0.671560985	0.811413971
ASTN2	24	17	0.391184708	0.725354524	1.48546174	0.361215565	0.817636365
ZAN	24	17	0.391184708	0.725354524	1.48546174	0.361215565	0.817636365
BRINP3	37	24	0.392751823	0.790173062	1.436838023	0.441143387	0.820263191
PRUNE2	37	24	0.392751823	0.790173062	1.436838023	0.441143387	0.820263191
CSMD1	57	36	0.396772445	0.801013953	1.318349829	0.491036869	0.828332996
ANTXR1	11	3	0.398093789	1.966521557	11.12797193	0.509966494	0.828800224
ATP4A	11	3	0.398093789	1.966521557	11.12797193	0.509966494	0.828800224
CNOT1	11	3	0.398093789	1.966521557	11.12797193	0.509966494	0.828800224
CPB1	11	3	0.398093789	1.966521557	11.12797193	0.509966494	0.828800224
HKR1	11	3	0.398093789	1.966521557	11.12797193	0.509966494	0.828800224
SOX6	11	3	0.398093789	1.966521557	11.12797193	0.509966494	0.828800224
TGM4	11	3	0.398093789	1.966521557	11.12797193	0.509966494	0.828800224
BIRC6	25	17	0.400850111	0.758041754	1.544934349	0.380030458	0.832827832
SLITRK3	25	17	0.400850111	0.758041754	1.544934349	0.380030458	0.832827832
AMER1	15	11	0.402483704	0.706740735	1.744297997	0.295365463	0.832827832
BPTF	15	11	0.402483704	0.706740735	1.744297997	0.295365463	0.832827832
F13A1	15	11	0.402483704	0.706740735	1.744297997	0.295365463	0.832827832
FOLH1	15	11	0.402483704	0.706740735	1.744297997	0.295365463	0.832827832
GRID1	15	11	0.402483704	0.706740735	1.744297997	0.295365463	0.832827832
PLCL1	15	11	0.402483704	0.706740735	1.744297997	0.295365463	0.832827832
TRPC5	15	11	0.402483704	0.706740735	1.744297997	0.295365463	0.832827832
DNAH7	26	18	0.40646075	0.742235193	1.485795819	0.377972235	0.832827832

LRRIQ1	26	18	0.40646075	0.742235193	1.485795819	0.377972235	0.832827832
ARID1A	20	7	0.408159101	1.540832756	4.406500962	0.609795919	0.832827832
ADAM12	8	7	0.408378687	0.593912566	1.960690214	0.184558126	0.832827832
AGRN	8	7	0.408378687	0.593912566	1.960690214	0.184558126	0.832827832
ATOH1	8	7	0.408378687	0.593912566	1.960690214	0.184558126	0.832827832
BRDT	8	7	0.408378687	0.593912566	1.960690214	0.184558126	0.832827832
CDHR1	8	7	0.408378687	0.593912566	1.960690214	0.184558126	0.832827832
CIT	8	7	0.408378687	0.593912566	1.960690214	0.184558126	0.832827832
CNTN2	8	7	0.408378687	0.593912566	1.960690214	0.184558126	0.832827832
COL16A1	8	7	0.408378687	0.593912566	1.960690214	0.184558126	0.832827832
DIAPH2	8	7	0.408378687	0.593912566	1.960690214	0.184558126	0.832827832
EPB41L2	8	7	0.408378687	0.593912566	1.960690214	0.184558126	0.832827832
FAM83C	8	7	0.408378687	0.593912566	1.960690214	0.184558126	0.832827832
GALNT15	8	7	0.408378687	0.593912566	1.960690214	0.184558126	0.832827832
GP2	8	7	0.408378687	0.593912566	1.960690214	0.184558126	0.832827832
GRM3	8	7	0.408378687	0.593912566	1.960690214	0.184558126	0.832827832
HPSE2	8	7	0.408378687	0.593912566	1.960690214	0.184558126	0.832827832
KCTD3	8	7	0.408378687	0.593912566	1.960690214	0.184558126	0.832827832
KRT6B	8	7	0.408378687	0.593912566	1.960690214	0.184558126	0.832827832
KRT82	8	7	0.408378687	0.593912566	1.960690214	0.184558126	0.832827832
LZTS1	8	7	0.408378687	0.593912566	1.960690214	0.184558126	0.832827832
MAGI1	8	7	0.408378687	0.593912566	1.960690214	0.184558126	0.832827832
MMP13	8	7	0.408378687	0.593912566	1.960690214	0.184558126	0.832827832
MTNR1A	8	7	0.408378687	0.593912566	1.960690214	0.184558126	0.832827832
MYRF	8	7	0.408378687	0.593912566	1.960690214	0.184558126	0.832827832
NCOA1	8	7	0.408378687	0.593912566	1.960690214	0.184558126	0.832827832
OR1S1	8	7	0.408378687	0.593912566	1.960690214	0.184558126	0.832827832
PCDHGB4	8	7	0.408378687	0.593912566	1.960690214	0.184558126	0.832827832
PGM5	8	7	0.408378687	0.593912566	1.960690214	0.184558126	0.832827832
PHF20L1	8	7	0.408378687	0.593912566	1.960690214	0.184558126	0.832827832
SKIV2L2	8	7	0.408378687	0.593912566	1.960690214	0.184558126	0.832827832
SLC18A3	8	7	0.408378687	0.593912566	1.960690214	0.184558126	0.832827832
SLC1A3	8	7	0.408378687	0.593912566	1.960690214	0.184558126	0.832827832
SLC7A13	8	7	0.408378687	0.593912566	1.960690214	0.184558126	0.832827832
SLCO6A1	8	7	0.408378687	0.593912566	1.960690214	0.184558126	0.832827832
STARD13	8	7	0.408378687	0.593912566	1.960690214	0.184558126	0.832827832
TNS1	8	7	0.408378687	0.593912566	1.960690214	0.184558126	0.832827832
TRIM48	8	7	0.408378687	0.593912566	1.960690214	0.184558126	0.832827832
URB2	8	7	0.408378687	0.593912566	1.960690214	0.184558126	0.832827832
XDH	8	7	0.408378687	0.593912566	1.960690214	0.184558126	0.832827832
ZCCHC12	8	7	0.408378687	0.593912566	1.960690214	0.184558126	0.832827832
ZIM2	8	7	0.408378687	0.593912566	1.960690214	0.184558126	0.832827832
ZNF221	8	7	0.408378687	0.593912566	1.960690214	0.184558126	0.832827832
LTBP2	16	12	0.413222289	0.6889736	1.637995126	0.297485121	0.84108318
MYPN	16	12	0.413222289	0.6889736	1.637995126	0.297485121	0.84108318
PCDHA1	16	12	0.413222289	0.6889736	1.637995126	0.297485121	0.84108318
RGS7	16	12	0.413222289	0.6889736	1.637995126	0.297485121	0.84108318
SGIP1	16	12	0.413222289	0.6889736	1.637995126	0.297485121	0.84108318
ATRX	17	12	0.427024421	0.734387568	1.730277206	0.321321124	0.84478108
FCRL5	17	12	0.427024421	0.734387568	1.730277206	0.321321124	0.84478108
KIAA2022	17	12	0.427024421	0.734387568	1.730277206	0.321321124	0.84478108
OR4Q3	17	12	0.427024421	0.734387568	1.730277206	0.321321124	0.84478108
POSTN	17	12	0.427024421	0.734387568	1.730277206	0.321321124	0.84478108
RIMBP2	17	12	0.427024421	0.734387568	1.730277206	0.321321124	0.84478108
SETBP1	35	14	0.42933846	1.3580719	2.819468811	0.687477324	0.84478108
SLITRK6	22	8	0.430279272	1.483877138	3.944602162	0.61900167	0.84478108
ADGRG4	46	29	0.430548147	0.8107131	1.399967549	0.475283718	0.84478108
ZNF804A	46	29	0.430548147	0.8107131	1.399967549	0.475283718	0.84478108

ACTN1	6	1	0.430595253	3.202917452	148.367269	0.383757797	0.84478108
ADAM20	6	1	0.430595253	3.202917452	148.367269	0.383757797	0.84478108
AFAP1L1	6	1	0.430595253	3.202917452	148.367269	0.383757797	0.84478108
ALOX12B	6	1	0.430595253	3.202917452	148.367269	0.383757797	0.84478108
AMPD2	6	1	0.430595253	3.202917452	148.367269	0.383757797	0.84478108
APLNR	6	1	0.430595253	3.202917452	148.367269	0.383757797	0.84478108
BPIFB4	6	1	0.430595253	3.202917452	148.367269	0.383757797	0.84478108
CADPS2	6	1	0.430595253	3.202917452	148.367269	0.383757797	0.84478108
CCDC181	6	1	0.430595253	3.202917452	148.367269	0.383757797	0.84478108
CDC20	6	1	0.430595253	3.202917452	148.367269	0.383757797	0.84478108
CEACAM1[illegible]	6	1	0.430595253	3.202917452	148.367269	0.383757797	0.84478108
CRTAM	6	1	0.430595253	3.202917452	148.367269	0.383757797	0.84478108
CUL4B	6	1	0.430595253	3.202917452	148.367269	0.383757797	0.84478108
CYC1	6	1	0.430595253	3.202917452	148.367269	0.383757797	0.84478108
DCBLD1	6	1	0.430595253	3.202917452	148.367269	0.383757797	0.84478108
DDN	6	1	0.430595253	3.202917452	148.367269	0.383757797	0.84478108
DIS3L2	6	1	0.430595253	3.202917452	148.367269	0.383757797	0.84478108
DKC1	6	1	0.430595253	3.202917452	148.367269	0.383757797	0.84478108
DLD	6	1	0.430595253	3.202917452	148.367269	0.383757797	0.84478108
DPP8	6	1	0.430595253	3.202917452	148.367269	0.383757797	0.84478108
EDN3	6	1	0.430595253	3.202917452	148.367269	0.383757797	0.84478108
EML4	6	1	0.430595253	3.202917452	148.367269	0.383757797	0.84478108
EPC2	6	1	0.430595253	3.202917452	148.367269	0.383757797	0.84478108
ERMN	6	1	0.430595253	3.202917452	148.367269	0.383757797	0.84478108
EVI2B	6	1	0.430595253	3.202917452	148.367269	0.383757797	0.84478108
FOXB2	6	1	0.430595253	3.202917452	148.367269	0.383757797	0.84478108
FUT11	6	1	0.430595253	3.202917452	148.367269	0.383757797	0.84478108
GATAD2A	6	1	0.430595253	3.202917452	148.367269	0.383757797	0.84478108
GCLC	6	1	0.430595253	3.202917452	148.367269	0.383757797	0.84478108
GIGYF2	6	1	0.430595253	3.202917452	148.367269	0.383757797	0.84478108
GJA5	6	1	0.430595253	3.202917452	148.367269	0.383757797	0.84478108
GUCY1B3	6	1	0.430595253	3.202917452	148.367269	0.383757797	0.84478108
HIST1H3E	6	1	0.430595253	3.202917452	148.367269	0.383757797	0.84478108
HSP90AB1	6	1	0.430595253	3.202917452	148.367269	0.383757797	0.84478108
IFNB1	6	1	0.430595253	3.202917452	148.367269	0.383757797	0.84478108
IQGAP1	6	1	0.430595253	3.202917452	148.367269	0.383757797	0.84478108
JAM2	6	1	0.430595253	3.202917452	148.367269	0.383757797	0.84478108
KCNJ6	6	1	0.430595253	3.202917452	148.367269	0.383757797	0.84478108
KCNK6	6	1	0.430595253	3.202917452	148.367269	0.383757797	0.84478108
KIFC1	6	1	0.430595253	3.202917452	148.367269	0.383757797	0.84478108
LACC1	6	1	0.430595253	3.202917452	148.367269	0.383757797	0.84478108
MAN2A2	6	1	0.430595253	3.202917452	148.367269	0.383757797	0.84478108
MASP2	6	1	0.430595253	3.202917452	148.367269	0.383757797	0.84478108
NEUROD6	6	1	0.430595253	3.202917452	148.367269	0.383757797	0.84478108
NUP43	6	1	0.430595253	3.202917452	148.367269	0.383757797	0.84478108
OR2J3	6	1	0.430595253	3.202917452	148.367269	0.383757797	0.84478108
OR52M1	6	1	0.430595253	3.202917452	148.367269	0.383757797	0.84478108
PCSK4	6	1	0.430595253	3.202917452	148.367269	0.383757797	0.84478108
PHLDB1	6	1	0.430595253	3.202917452	148.367269	0.383757797	0.84478108
PPP2R5B	6	1	0.430595253	3.202917452	148.367269	0.383757797	0.84478108
PPP3R2	6	1	0.430595253	3.202917452	148.367269	0.383757797	0.84478108
PREB	6	1	0.430595253	3.202917452	148.367269	0.383757797	0.84478108
RAB11FIP4	6	1	0.430595253	3.202917452	148.367269	0.383757797	0.84478108
RALGPS2	6	1	0.430595253	3.202917452	148.367269	0.383757797	0.84478108
RAPGEF1	6	1	0.430595253	3.202917452	148.367269	0.383757797	0.84478108
RPL3L	6	1	0.430595253	3.202917452	148.367269	0.383757797	0.84478108
SLC5A6	6	1	0.430595253	3.202917452	148.367269	0.383757797	0.84478108
SPATA7	6	1	0.430595253	3.202917452	148.367269	0.383757797	0.84478108

SPDL1	6	1	0.430595253	3.202917452	148.367269	0.383757797	0.84478108
SYBU	6	1	0.430595253	3.202917452	148.367269	0.383757797	0.84478108
TBC1D23	6	1	0.430595253	3.202917452	148.367269	0.383757797	0.84478108
TFDP1	6	1	0.430595253	3.202917452	148.367269	0.383757797	0.84478108
TMEM130	6	1	0.430595253	3.202917452	148.367269	0.383757797	0.84478108
TNS2	6	1	0.430595253	3.202917452	148.367269	0.383757797	0.84478108
TRIM22	6	1	0.430595253	3.202917452	148.367269	0.383757797	0.84478108
TSGA10	6	1	0.430595253	3.202917452	148.367269	0.383757797	0.84478108
TUBB2B	6	1	0.430595253	3.202917452	148.367269	0.383757797	0.84478108
YY2	6	1	0.430595253	3.202917452	148.367269	0.383757797	0.84478108
ZBTB3	6	1	0.430595253	3.202917452	148.367269	0.383757797	0.84478108
ZFP36	6	1	0.430595253	3.202917452	148.367269	0.383757797	0.84478108
ZNF683	6	1	0.430595253	3.202917452	148.367269	0.383757797	0.84478108
ZNF74	6	1	0.430595253	3.202917452	148.367269	0.383757797	0.84478108
ADGRG7	9	7	0.433577577	0.670106455	2.158590448	0.217582969	0.84478108
CCDC80	9	7	0.433577577	0.670106455	2.158590448	0.217582969	0.84478108
CCSER1	9	7	0.433577577	0.670106455	2.158590448	0.217582969	0.84478108
CHGB	9	7	0.433577577	0.670106455	2.158590448	0.217582969	0.84478108
DLGAP4	9	7	0.433577577	0.670106455	2.158590448	0.217582969	0.84478108
E2F7	9	7	0.433577577	0.670106455	2.158590448	0.217582969	0.84478108
FGB	9	7	0.433577577	0.670106455	2.158590448	0.217582969	0.84478108
FHL5	9	7	0.433577577	0.670106455	2.158590448	0.217582969	0.84478108
GBP6	9	7	0.433577577	0.670106455	2.158590448	0.217582969	0.84478108
GJA8	9	7	0.433577577	0.670106455	2.158590448	0.217582969	0.84478108
GNAS	9	7	0.433577577	0.670106455	2.158590448	0.217582969	0.84478108
GPRASP2	9	7	0.433577577	0.670106455	2.158590448	0.217582969	0.84478108
HTR1E	9	7	0.433577577	0.670106455	2.158590448	0.217582969	0.84478108
LRP12	9	7	0.433577577	0.670106455	2.158590448	0.217582969	0.84478108
MEFV	9	7	0.433577577	0.670106455	2.158590448	0.217582969	0.84478108
MYH9	9	7	0.433577577	0.670106455	2.158590448	0.217582969	0.84478108
NCOR1	9	7	0.433577577	0.670106455	2.158590448	0.217582969	0.84478108
NME8	9	7	0.433577577	0.670106455	2.158590448	0.217582969	0.84478108
OLFM3	9	7	0.433577577	0.670106455	2.158590448	0.217582969	0.84478108
OR10Q1	9	7	0.433577577	0.670106455	2.158590448	0.217582969	0.84478108
OR11L1	9	7	0.433577577	0.670106455	2.158590448	0.217582969	0.84478108
OR51G2	9	7	0.433577577	0.670106455	2.158590448	0.217582969	0.84478108
PLXNA3	9	7	0.433577577	0.670106455	2.158590448	0.217582969	0.84478108
POF1B	9	7	0.433577577	0.670106455	2.158590448	0.217582969	0.84478108
PSG7	9	7	0.433577577	0.670106455	2.158590448	0.217582969	0.84478108
SLC5A11	9	7	0.433577577	0.670106455	2.158590448	0.217582969	0.84478108
SMARCA2	9	7	0.433577577	0.670106455	2.158590448	0.217582969	0.84478108
TBR1	9	7	0.433577577	0.670106455	2.158590448	0.217582969	0.84478108
TBX4	9	7	0.433577577	0.670106455	2.158590448	0.217582969	0.84478108
TEX14	9	7	0.433577577	0.670106455	2.158590448	0.217582969	0.84478108
TSHR	9	7	0.433577577	0.670106455	2.158590448	0.217582969	0.84478108
ZNF492	9	7	0.433577577	0.670106455	2.158590448	0.217582969	0.84478108
ZNF560	9	7	0.433577577	0.670106455	2.158590448	0.217582969	0.84478108
ZPLD1	9	7	0.433577577	0.670106455	2.158590448	0.217582969	0.84478108
CCDC129	18	13	0.435563523	0.715558977	1.632891682	0.321922339	0.847713101
FRAS1	18	13	0.435563523	0.715558977	1.632891682	0.321922339	0.847713101
GLI3	18	13	0.435563523	0.715558977	1.632891682	0.321922339	0.847713101
CWH43	13	4	0.439999059	1.744159524	7.46128596	0.527875137	0.853830772
DCLK3	13	4	0.439999059	1.744159524	7.46128596	0.527875137	0.853830772
HOXA1	13	4	0.439999059	1.744159524	7.46128596	0.527875137	0.853830772
KIF13A	13	4	0.439999059	1.744159524	7.46128596	0.527875137	0.853830772
PDCD11	13	4	0.439999059	1.744159524	7.46128596	0.527875137	0.853830772
RALGAPB	13	4	0.439999059	1.744159524	7.46128596	0.527875137	0.853830772
SRGAP3	13	4	0.439999059	1.744159524	7.46128596	0.527875137	0.853830772

TNC	13	4	0.439999059	1.744159524	7.46128596	0.527875137	0.853830772
DNAH9	51	32	0.447743952	0.811943016	1.369751024	0.48646637	0.863990385
DCAF12L1	19	13	0.448640033	0.75776586	1.716267618	0.344622641	0.863990385
NLRP12	19	13	0.448640033	0.75776586	1.716267618	0.344622641	0.863990385
ACACA	10	8	0.449484215	0.649616445	1.933714018	0.225906653	0.863990385
C5orf42	10	8	0.449484215	0.649616445	1.933714018	0.225906653	0.863990385
CACNA1S	10	8	0.449484215	0.649616445	1.933714018	0.225906653	0.863990385
CHAT	10	8	0.449484215	0.649616445	1.933714018	0.225906653	0.863990385
CYLC1	10	8	0.449484215	0.649616445	1.933714018	0.225906653	0.863990385
DCAF8L2	10	8	0.449484215	0.649616445	1.933714018	0.225906653	0.863990385
DLG2	10	8	0.449484215	0.649616445	1.933714018	0.225906653	0.863990385
DPYS	10	8	0.449484215	0.649616445	1.933714018	0.225906653	0.863990385
GABRB1	10	8	0.449484215	0.649616445	1.933714018	0.225906653	0.863990385
GBA3	10	8	0.449484215	0.649616445	1.933714018	0.225906653	0.863990385
HCFC1	10	8	0.449484215	0.649616445	1.933714018	0.225906653	0.863990385
HECW2	10	8	0.449484215	0.649616445	1.933714018	0.225906653	0.863990385
IL18RAP	10	8	0.449484215	0.649616445	1.933714018	0.225906653	0.863990385
ITPR3	10	8	0.449484215	0.649616445	1.933714018	0.225906653	0.863990385
MYO3B	10	8	0.449484215	0.649616445	1.933714018	0.225906653	0.863990385
NRK	10	8	0.449484215	0.649616445	1.933714018	0.225906653	0.863990385
OR10R2	10	8	0.449484215	0.649616445	1.933714018	0.225906653	0.863990385
OR4S2	10	8	0.449484215	0.649616445	1.933714018	0.225906653	0.863990385
PIEZO2	10	8	0.449484215	0.649616445	1.933714018	0.225906653	0.863990385
PRRC2B	10	8	0.449484215	0.649616445	1.933714018	0.225906653	0.863990385
RBBP6	10	8	0.449484215	0.649616445	1.933714018	0.225906653	0.863990385
VAV3	10	8	0.449484215	0.649616445	1.933714018	0.225906653	0.863990385
VPS13A	10	8	0.449484215	0.649616445	1.933714018	0.225906653	0.863990385
NID1	20	14	0.455386438	0.738438154	1.626520256	0.343984345	0.874699386
SEMA5A	20	14	0.455386438	0.738438154	1.626520256	0.343984345	0.874699386
PCLO	54	33	0.459216148	0.836818126	1.399495996	0.50575546	0.881735038
NEB	40	17	0.462205033	1.275973342	2.485260951	0.680147503	0.884487385
NBEA	26	10	0.467522673	1.404480641	3.349709658	0.63572416	0.884487385
ANK1	21	14	0.46785445	0.777839573	1.702535482	0.365679033	0.884487385
EPHB6	21	14	0.46785445	0.777839573	1.702535482	0.365679033	0.884487385
TRRAP	21	14	0.46785445	0.777839573	1.702535482	0.365679033	0.884487385
VPS13C	21	14	0.46785445	0.777839573	1.702535482	0.365679033	0.884487385
BACH2	11	8	0.471134551	0.716714632	2.096133574	0.256744525	0.884487385
BTAF1	11	8	0.471134551	0.716714632	2.096133574	0.256744525	0.884487385
BZRAP1	11	8	0.471134551	0.716714632	2.096133574	0.256744525	0.884487385
CACNA1G	11	8	0.471134551	0.716714632	2.096133574	0.256744525	0.884487385
COL2A1	11	8	0.471134551	0.716714632	2.096133574	0.256744525	0.884487385
DACT1	11	8	0.471134551	0.716714632	2.096133574	0.256744525	0.884487385
ENPP2	11	8	0.471134551	0.716714632	2.096133574	0.256744525	0.884487385
FAM171B	11	8	0.471134551	0.716714632	2.096133574	0.256744525	0.884487385
FCRL1	11	8	0.471134551	0.716714632	2.096133574	0.256744525	0.884487385
FCRL3	11	8	0.471134551	0.716714632	2.096133574	0.256744525	0.884487385
GBP4	11	8	0.471134551	0.716714632	2.096133574	0.256744525	0.884487385
GPATCH8	11	8	0.471134551	0.716714632	2.096133574	0.256744525	0.884487385
GRIA3	11	8	0.471134551	0.716714632	2.096133574	0.256744525	0.884487385
MYLK	11	8	0.471134551	0.716714632	2.096133574	0.256744525	0.884487385
OR10AG1	11	8	0.471134551	0.716714632	2.096133574	0.256744525	0.884487385
OR4K13	11	8	0.471134551	0.716714632	2.096133574	0.256744525	0.884487385
OR4K17	11	8	0.471134551	0.716714632	2.096133574	0.256744525	0.884487385
PCDH19	11	8	0.471134551	0.716714632	2.096133574	0.256744525	0.884487385
PCDHA11	11	8	0.471134551	0.716714632	2.096133574	0.256744525	0.884487385
RBFOX1	11	8	0.471134551	0.716714632	2.096133574	0.256744525	0.884487385
SLC27A6	11	8	0.471134551	0.716714632	2.096133574	0.256744525	0.884487385
SPTBN1	11	8	0.471134551	0.716714632	2.096133574	0.256744525	0.884487385

TRPM3	11	8	0.471134551	0.716714632	2.096133574	0.256744525	0.884487385
UGT2B28	11	8	0.471134551	0.716714632	2.096133574	0.256744525	0.884487385
CACNA2D1	22	15	0.473120441	0.758243712	1.619486476	0.36397648	0.884487385
NRCAM	22	15	0.473120441	0.758243712	1.619486476	0.36397648	0.884487385
OTOF	22	15	0.473120441	0.758243712	1.619486476	0.36397648	0.884487385
GALNT13	15	5	0.473937391	1.610932433	5.766798801	0.544238007	0.884487385
SCN4A	15	5	0.473937391	1.610932433	5.766798801	0.544238007	0.884487385
TAF3	15	5	0.473937391	1.610932433	5.766798801	0.544238007	0.884487385
ARHGAP32	12	9	0.482846957	0.692987674	1.90430244	0.261721273	0.884487385
CNGB3	12	9	0.482846957	0.692987674	1.90430244	0.261721273	0.884487385
DCSTAMP	12	9	0.482846957	0.692987674	1.90430244	0.261721273	0.884487385
DOCK4	12	9	0.482846957	0.692987674	1.90430244	0.261721273	0.884487385
DSEL	12	9	0.482846957	0.692987674	1.90430244	0.261721273	0.884487385
MKRN3	12	9	0.482846957	0.692987674	1.90430244	0.261721273	0.884487385
NPHS1	12	9	0.482846957	0.692987674	1.90430244	0.261721273	0.884487385
OR1C1	12	9	0.482846957	0.692987674	1.90430244	0.261721273	0.884487385
PCDHGA1	12	9	0.482846957	0.692987674	1.90430244	0.261721273	0.884487385
RAG1	12	9	0.482846957	0.692987674	1.90430244	0.261721273	0.884487385
SERPINB3	12	9	0.482846957	0.692987674	1.90430244	0.261721273	0.884487385
SLC26A7	12	9	0.482846957	0.692987674	1.90430244	0.261721273	0.884487385
SPEN	12	9	0.482846957	0.692987674	1.90430244	0.261721273	0.884487385
TRPC6	12	9	0.482846957	0.692987674	1.90430244	0.261721273	0.884487385
UGT3A1	12	9	0.482846957	0.692987674	1.90430244	0.261721273	0.884487385
ZC3H13	12	9	0.482846957	0.692987674	1.90430244	0.261721273	0.884487385
ZNF292	12	9	0.482846957	0.692987674	1.90430244	0.261721273	0.884487385
FLG2	40	25	0.486294406	0.823040805	1.475030065	0.466309546	0.884487385
MYO18B	24	16	0.489099953	0.775602506	1.612163844	0.382263474	0.884487385
RELN	43	27	0.497703308	0.816632579	1.434917943	0.471140362	0.884487385
KMT2C	29	12	0.497768935	1.302490866	2.883801501	0.623896286	0.884487385
ACSM2A	13	9	0.501927656	0.753082772	2.041723627	0.290617913	0.884487385
ARMC3	13	9	0.501927656	0.753082772	2.041723627	0.290617913	0.884487385
CENPE	13	9	0.501927656	0.753082772	2.041723627	0.290617913	0.884487385
CEP128	13	9	0.501927656	0.753082772	2.041723627	0.290617913	0.884487385
CTNNA3	13	9	0.501927656	0.753082772	2.041723627	0.290617913	0.884487385
KCNB1	13	9	0.501927656	0.753082772	2.041723627	0.290617913	0.884487385
KPRP	13	9	0.501927656	0.753082772	2.041723627	0.290617913	0.884487385
LGR5	13	9	0.501927656	0.753082772	2.041723627	0.290617913	0.884487385
MAP1B	13	9	0.501927656	0.753082772	2.041723627	0.290617913	0.884487385
OR14K1	13	9	0.501927656	0.753082772	2.041723627	0.290617913	0.884487385
OR2L2	13	9	0.501927656	0.753082772	2.041723627	0.290617913	0.884487385
PCDHA9	13	9	0.501927656	0.753082772	2.041723627	0.290617913	0.884487385
PCNX	13	9	0.501927656	0.753082772	2.041723627	0.290617913	0.884487385
SDK2	13	9	0.501927656	0.753082772	2.041723627	0.290617913	0.884487385
C2CD3	17	6	0.502135228	1.522279419	4.808952059	0.559059934	0.884487385
OR5AS1	17	6	0.502135228	1.522279419	4.808952059	0.559059934	0.884487385
TAS1R2	17	6	0.502135228	1.522279419	4.808952059	0.559059934	0.884487385
ACOT12	5	4	0.503709862	0.655144689	3.347721434	0.138946877	0.884487385
ADAM10	5	4	0.503709862	0.655144689	3.347721434	0.138946877	0.884487385
ADCY7	5	4	0.503709862	0.655144689	3.347721434	0.138946877	0.884487385
ADD3	5	4	0.503709862	0.655144689	3.347721434	0.138946877	0.884487385
ADH1B	5	4	0.503709862	0.655144689	3.347721434	0.138946877	0.884487385
AK9	5	4	0.503709862	0.655144689	3.347721434	0.138946877	0.884487385
AKNAD1	5	4	0.503709862	0.655144689	3.347721434	0.138946877	0.884487385
ALDH1B1	5	4	0.503709862	0.655144689	3.347721434	0.138946877	0.884487385
AMER2	5	4	0.503709862	0.655144689	3.347721434	0.138946877	0.884487385
AMY2A	5	4	0.503709862	0.655144689	3.347721434	0.138946877	0.884487385
ANKLE2	5	4	0.503709862	0.655144689	3.347721434	0.138946877	0.884487385
APBA1	5	4	0.503709862	0.655144689	3.347721434	0.138946877	0.884487385

APLP2	5	4	0.503709862	0.655144689	3.347721434	0.138946877	0.884487385
ARAP1	5	4	0.503709862	0.655144689	3.347721434	0.138946877	0.884487385
ARSH	5	4	0.503709862	0.655144689	3.347721434	0.138946877	0.884487385
ASIC5	5	4	0.503709862	0.655144689	3.347721434	0.138946877	0.884487385
ATG9B	5	4	0.503709862	0.655144689	3.347721434	0.138946877	0.884487385
ATP1A3	5	4	0.503709862	0.655144689	3.347721434	0.138946877	0.884487385
BACH1	5	4	0.503709862	0.655144689	3.347721434	0.138946877	0.884487385
BAZ1B	5	4	0.503709862	0.655144689	3.347721434	0.138946877	0.884487385
BCL6	5	4	0.503709862	0.655144689	3.347721434	0.138946877	0.884487385
BCL9	5	4	0.503709862	0.655144689	3.347721434	0.138946877	0.884487385
C10orf2	5	4	0.503709862	0.655144689	3.347721434	0.138946877	0.884487385
C1orf116	5	4	0.503709862	0.655144689	3.347721434	0.138946877	0.884487385
C20orf194	5	4	0.503709862	0.655144689	3.347721434	0.138946877	0.884487385
CCDC54	5	4	0.503709862	0.655144689	3.347721434	0.138946877	0.884487385
CCDC63	5	4	0.503709862	0.655144689	3.347721434	0.138946877	0.884487385
CDH26	5	4	0.503709862	0.655144689	3.347721434	0.138946877	0.884487385
CEACAM5	5	4	0.503709862	0.655144689	3.347721434	0.138946877	0.884487385
CEP120	5	4	0.503709862	0.655144689	3.347721434	0.138946877	0.884487385
CFHR3	5	4	0.503709862	0.655144689	3.347721434	0.138946877	0.884487385
CGN	5	4	0.503709862	0.655144689	3.347721434	0.138946877	0.884487385
CGNL1	5	4	0.503709862	0.655144689	3.347721434	0.138946877	0.884487385
CLCN3	5	4	0.503709862	0.655144689	3.347721434	0.138946877	0.884487385
COLEC12	5	4	0.503709862	0.655144689	3.347721434	0.138946877	0.884487385
CRYBG3	5	4	0.503709862	0.655144689	3.347721434	0.138946877	0.884487385
CSF3R	5	4	0.503709862	0.655144689	3.347721434	0.138946877	0.884487385
CYP2C9	5	4	0.503709862	0.655144689	3.347721434	0.138946877	0.884487385
DENND5A	5	4	0.503709862	0.655144689	3.347721434	0.138946877	0.884487385
DMGDH	5	4	0.503709862	0.655144689	3.347721434	0.138946877	0.884487385
DNMT3B	5	4	0.503709862	0.655144689	3.347721434	0.138946877	0.884487385
EMILIN3	5	4	0.503709862	0.655144689	3.347721434	0.138946877	0.884487385
EPHA2	5	4	0.503709862	0.655144689	3.347721434	0.138946877	0.884487385
EPHB4	5	4	0.503709862	0.655144689	3.347721434	0.138946877	0.884487385
EPS8L3	5	4	0.503709862	0.655144689	3.347721434	0.138946877	0.884487385
F2	5	4	0.503709862	0.655144689	3.347721434	0.138946877	0.884487385
FAM131B	5	4	0.503709862	0.655144689	3.347721434	0.138946877	0.884487385
FAM153B	5	4	0.503709862	0.655144689	3.347721434	0.138946877	0.884487385
FGF13	5	4	0.503709862	0.655144689	3.347721434	0.138946877	0.884487385
FGF14	5	4	0.503709862	0.655144689	3.347721434	0.138946877	0.884487385
FGFR4	5	4	0.503709862	0.655144689	3.347721434	0.138946877	0.884487385
FMO4	5	4	0.503709862	0.655144689	3.347721434	0.138946877	0.884487385
GDAP2	5	4	0.503709862	0.655144689	3.347721434	0.138946877	0.884487385
GLRA3	5	4	0.503709862	0.655144689	3.347721434	0.138946877	0.884487385
GPR15	5	4	0.503709862	0.655144689	3.347721434	0.138946877	0.884487385
GPR26	5	4	0.503709862	0.655144689	3.347721434	0.138946877	0.884487385
GRAP2	5	4	0.503709862	0.655144689	3.347721434	0.138946877	0.884487385
GRK7	5	4	0.503709862	0.655144689	3.347721434	0.138946877	0.884487385
HCN3	5	4	0.503709862	0.655144689	3.347721434	0.138946877	0.884487385
HDLBP	5	4	0.503709862	0.655144689	3.347721434	0.138946877	0.884487385
HJURP	5	4	0.503709862	0.655144689	3.347721434	0.138946877	0.884487385
HK1	5	4	0.503709862	0.655144689	3.347721434	0.138946877	0.884487385
HNRNPA2B	5	4	0.503709862	0.655144689	3.347721434	0.138946877	0.884487385
HNRNPU	5	4	0.503709862	0.655144689	3.347721434	0.138946877	0.884487385
HSF5	5	4	0.503709862	0.655144689	3.347721434	0.138946877	0.884487385
HSPA13	5	4	0.503709862	0.655144689	3.347721434	0.138946877	0.884487385
HSPA6	5	4	0.503709862	0.655144689	3.347721434	0.138946877	0.884487385
IFT122	5	4	0.503709862	0.655144689	3.347721434	0.138946877	0.884487385
IGKV1D-16	5	4	0.503709862	0.655144689	3.347721434	0.138946877	0.884487385
IGKV2-24	5	4	0.503709862	0.655144689	3.347721434	0.138946877	0.884487385

IGSF21	5	4	0.503709862	0.655144689	3.347721434	0.138946877	0.884487385
IL17A	5	4	0.503709862	0.655144689	3.347721434	0.138946877	0.884487385
INO80	5	4	0.503709862	0.655144689	3.347721434	0.138946877	0.884487385
JAG2	5	4	0.503709862	0.655144689	3.347721434	0.138946877	0.884487385
KANSL1L	5	4	0.503709862	0.655144689	3.347721434	0.138946877	0.884487385
KCNV2	5	4	0.503709862	0.655144689	3.347721434	0.138946877	0.884487385
KIF18A	5	4	0.503709862	0.655144689	3.347721434	0.138946877	0.884487385
KIR2DL3	5	4	0.503709862	0.655144689	3.347721434	0.138946877	0.884487385
KIR2DS4	5	4	0.503709862	0.655144689	3.347721434	0.138946877	0.884487385
KRT2	5	4	0.503709862	0.655144689	3.347721434	0.138946877	0.884487385
KRTAP10-9	5	4	0.503709862	0.655144689	3.347721434	0.138946877	0.884487385
KSR1	5	4	0.503709862	0.655144689	3.347721434	0.138946877	0.884487385
LCP1	5	4	0.503709862	0.655144689	3.347721434	0.138946877	0.884487385
LGI1	5	4	0.503709862	0.655144689	3.347721434	0.138946877	0.884487385
LIPF	5	4	0.503709862	0.655144689	3.347721434	0.138946877	0.884487385
LIPG	5	4	0.503709862	0.655144689	3.347721434	0.138946877	0.884487385
LRFN1	5	4	0.503709862	0.655144689	3.347721434	0.138946877	0.884487385
LUC7L	5	4	0.503709862	0.655144689	3.347721434	0.138946877	0.884487385
MAML1	5	4	0.503709862	0.655144689	3.347721434	0.138946877	0.884487385
MAP4K1	5	4	0.503709862	0.655144689	3.347721434	0.138946877	0.884487385
MAP7D2	5	4	0.503709862	0.655144689	3.347721434	0.138946877	0.884487385
MARK2	5	4	0.503709862	0.655144689	3.347721434	0.138946877	0.884487385
MCMDC2	5	4	0.503709862	0.655144689	3.347721434	0.138946877	0.884487385
MLLT4	5	4	0.503709862	0.655144689	3.347721434	0.138946877	0.884487385
MOS	5	4	0.503709862	0.655144689	3.347721434	0.138946877	0.884487385
MS4A3	5	4	0.503709862	0.655144689	3.347721434	0.138946877	0.884487385
MTMR12	5	4	0.503709862	0.655144689	3.347721434	0.138946877	0.884487385
MYH14	5	4	0.503709862	0.655144689	3.347721434	0.138946877	0.884487385
MYO5A	5	4	0.503709862	0.655144689	3.347721434	0.138946877	0.884487385
OPRK1	5	4	0.503709862	0.655144689	3.347721434	0.138946877	0.884487385
OR10C1	5	4	0.503709862	0.655144689	3.347721434	0.138946877	0.884487385
OR10J5	5	4	0.503709862	0.655144689	3.347721434	0.138946877	0.884487385
OR4X1	5	4	0.503709862	0.655144689	3.347721434	0.138946877	0.884487385
OR51I2	5	4	0.503709862	0.655144689	3.347721434	0.138946877	0.884487385
OR52I2	5	4	0.503709862	0.655144689	3.347721434	0.138946877	0.884487385
OR52L1	5	4	0.503709862	0.655144689	3.347721434	0.138946877	0.884487385
OR8B8	5	4	0.503709862	0.655144689	3.347721434	0.138946877	0.884487385
OR9A4	5	4	0.503709862	0.655144689	3.347721434	0.138946877	0.884487385
OTOP1	5	4	0.503709862	0.655144689	3.347721434	0.138946877	0.884487385
PBX1	5	4	0.503709862	0.655144689	3.347721434	0.138946877	0.884487385
PCSK6	5	4	0.503709862	0.655144689	3.347721434	0.138946877	0.884487385
PDE6C	5	4	0.503709862	0.655144689	3.347721434	0.138946877	0.884487385
PFKL	5	4	0.503709862	0.655144689	3.347721434	0.138946877	0.884487385
PIK3C3	5	4	0.503709862	0.655144689	3.347721434	0.138946877	0.884487385
PIK3R5	5	4	0.503709862	0.655144689	3.347721434	0.138946877	0.884487385
PLEKHM3	5	4	0.503709862	0.655144689	3.347721434	0.138946877	0.884487385
POU4F2	5	4	0.503709862	0.655144689	3.347721434	0.138946877	0.884487385
PRDM4	5	4	0.503709862	0.655144689	3.347721434	0.138946877	0.884487385
RASSF2	5	4	0.503709862	0.655144689	3.347721434	0.138946877	0.884487385
RNF216	5	4	0.503709862	0.655144689	3.347721434	0.138946877	0.884487385
RTEL1	5	4	0.503709862	0.655144689	3.347721434	0.138946877	0.884487385
SDCCAG8	5	4	0.503709862	0.655144689	3.347721434	0.138946877	0.884487385
SEC23IP	5	4	0.503709862	0.655144689	3.347721434	0.138946877	0.884487385
SEL1L2	5	4	0.503709862	0.655144689	3.347721434	0.138946877	0.884487385
SHOX	5	4	0.503709862	0.655144689	3.347721434	0.138946877	0.884487385
SLC18A2	5	4	0.503709862	0.655144689	3.347721434	0.138946877	0.884487385
SLC22A11	5	4	0.503709862	0.655144689	3.347721434	0.138946877	0.884487385
SLC35F3	5	4	0.503709862	0.655144689	3.347721434	0.138946877	0.884487385

SSH2	5	4	0.503709862	0.655144689	3.347721434	0.138946877	0.884487385
ST8SIA2	5	4	0.503709862	0.655144689	3.347721434	0.138946877	0.884487385
TBC1D9	5	4	0.503709862	0.655144689	3.347721434	0.138946877	0.884487385
TEKT3	5	4	0.503709862	0.655144689	3.347721434	0.138946877	0.884487385
TKTL1	5	4	0.503709862	0.655144689	3.347721434	0.138946877	0.884487385
TLE4	5	4	0.503709862	0.655144689	3.347721434	0.138946877	0.884487385
TMC2	5	4	0.503709862	0.655144689	3.347721434	0.138946877	0.884487385
TMEM196	5	4	0.503709862	0.655144689	3.347721434	0.138946877	0.884487385
TNK2	5	4	0.503709862	0.655144689	3.347721434	0.138946877	0.884487385
TOP2B	5	4	0.503709862	0.655144689	3.347721434	0.138946877	0.884487385
TSGA10IP	5	4	0.503709862	0.655144689	3.347721434	0.138946877	0.884487385
TTLL5	5	4	0.503709862	0.655144689	3.347721434	0.138946877	0.884487385
TYRO3	5	4	0.503709862	0.655144689	3.347721434	0.138946877	0.884487385
VAV1	5	4	0.503709862	0.655144689	3.347721434	0.138946877	0.884487385
VIL1	5	4	0.503709862	0.655144689	3.347721434	0.138946877	0.884487385
WDR59	5	4	0.503709862	0.655144689	3.347721434	0.138946877	0.884487385
WDR90	5	4	0.503709862	0.655144689	3.347721434	0.138946877	0.884487385
WNT16	5	4	0.503709862	0.655144689	3.347721434	0.138946877	0.884487385
WNT8A	5	4	0.503709862	0.655144689	3.347721434	0.138946877	0.884487385
ZBTB21	5	4	0.503709862	0.655144689	3.347721434	0.138946877	0.884487385
ZMIZ2	5	4	0.503709862	0.655144689	3.347721434	0.138946877	0.884487385
ZNF282	5	4	0.503709862	0.655144689	3.347721434	0.138946877	0.884487385
ZNF425	5	4	0.503709862	0.655144689	3.347721434	0.138946877	0.884487385
ZNF438	5	4	0.503709862	0.655144689	3.347721434	0.138946877	0.884487385
ZNF516	5	4	0.503709862	0.655144689	3.347721434	0.138946877	0.884487385
ZNF518A	5	4	0.503709862	0.655144689	3.347721434	0.138946877	0.884487385
ZNF595	5	4	0.503709862	0.655144689	3.347721434	0.138946877	0.884487385
ZNF649	5	4	0.503709862	0.655144689	3.347721434	0.138946877	0.884487385
ZNF777	5	4	0.503709862	0.655144689	3.347721434	0.138946877	0.884487385
ZNF841	5	4	0.503709862	0.655144689	3.347721434	0.138946877	0.884487385
ZP1	5	4	0.503709862	0.655144689	3.347721434	0.138946877	0.884487385
ADGRE5	8	2	0.5056604	2.137264794	20.88303344	0.419969214	0.884487385
ANKMY1	8	2	0.5056604	2.137264794	20.88303344	0.419969214	0.884487385
APOA4	8	2	0.5056604	2.137264794	20.88303344	0.419969214	0.884487385
ATP1B4	8	2	0.5056604	2.137264794	20.88303344	0.419969214	0.884487385
ATP2A2	8	2	0.5056604	2.137264794	20.88303344	0.419969214	0.884487385
ATP2B4	8	2	0.5056604	2.137264794	20.88303344	0.419969214	0.884487385
BBS7	8	2	0.5056604	2.137264794	20.88303344	0.419969214	0.884487385
BTBD9	8	2	0.5056604	2.137264794	20.88303344	0.419969214	0.884487385
CHRNB2	8	2	0.5056604	2.137264794	20.88303344	0.419969214	0.884487385
CLCN4	8	2	0.5056604	2.137264794	20.88303344	0.419969214	0.884487385
CLSPN	8	2	0.5056604	2.137264794	20.88303344	0.419969214	0.884487385
DENND2C	8	2	0.5056604	2.137264794	20.88303344	0.419969214	0.884487385
F2RL2	8	2	0.5056604	2.137264794	20.88303344	0.419969214	0.884487385
HSP90B1	8	2	0.5056604	2.137264794	20.88303344	0.419969214	0.884487385
ITGB6	8	2	0.5056604	2.137264794	20.88303344	0.419969214	0.884487385
MAGEA8	8	2	0.5056604	2.137264794	20.88303344	0.419969214	0.884487385
MBL2	8	2	0.5056604	2.137264794	20.88303344	0.419969214	0.884487385
NUP155	8	2	0.5056604	2.137264794	20.88303344	0.419969214	0.884487385
OR4C11	8	2	0.5056604	2.137264794	20.88303344	0.419969214	0.884487385
OR4E2	8	2	0.5056604	2.137264794	20.88303344	0.419969214	0.884487385
PDE1B	8	2	0.5056604	2.137264794	20.88303344	0.419969214	0.884487385
PKLR	8	2	0.5056604	2.137264794	20.88303344	0.419969214	0.884487385
PRELP	8	2	0.5056604	2.137264794	20.88303344	0.419969214	0.884487385
PRODH2	8	2	0.5056604	2.137264794	20.88303344	0.419969214	0.884487385
PTGFR	8	2	0.5056604	2.137264794	20.88303344	0.419969214	0.884487385
SAMD3	8	2	0.5056604	2.137264794	20.88303344	0.419969214	0.884487385
SERPINE1	8	2	0.5056604	2.137264794	20.88303344	0.419969214	0.884487385

SLAMF9	8	2	0.5056604	2.137264794	20.88303344	0.419969214	0.884487385
TEX13B	8	2	0.5056604	2.137264794	20.88303344	0.419969214	0.884487385
TRIP13	8	2	0.5056604	2.137264794	20.88303344	0.419969214	0.884487385
TTC37	8	2	0.5056604	2.137264794	20.88303344	0.419969214	0.884487385
USP32	8	2	0.5056604	2.137264794	20.88303344	0.419969214	0.884487385
WDR62	8	2	0.5056604	2.137264794	20.88303344	0.419969214	0.884487385
ZIK1	8	2	0.5056604	2.137264794	20.88303344	0.419969214	0.884487385
ZNF184	8	2	0.5056604	2.137264794	20.88303344	0.419969214	0.884487385
ZNF354C	8	2	0.5056604	2.137264794	20.88303344	0.419969214	0.884487385
DCAF4L2	31	13	0.510383164	1.285889311	2.758462713	0.631922401	0.886104691
CARD11	14	10	0.510602427	0.72779192	1.875562363	0.293086829	0.886104691
OR2T1	14	10	0.510602427	0.72779192	1.875562363	0.293086829	0.886104691
PAPPA	14	10	0.510602427	0.72779192	1.875562363	0.293086829	0.886104691
PCDHGA4	14	10	0.510602427	0.72779192	1.875562363	0.293086829	0.886104691
PHF3	14	10	0.510602427	0.72779192	1.875562363	0.293086829	0.886104691
RUNX1T1	14	10	0.510602427	0.72779192	1.875562363	0.293086829	0.886104691
SLC8A3	14	10	0.510602427	0.72779192	1.875562363	0.293086829	0.886104691
SPATA31E1	14	10	0.510602427	0.72779192	1.875562363	0.293086829	0.886104691
TEP1	14	10	0.510602427	0.72779192	1.875562363	0.293086829	0.886104691
LYST	28	18	0.516801491	0.80461396	1.59749518	0.414406051	0.886104691
ABCB7	6	5	0.524521676	0.627189622	2.639008396	0.156881322	0.886104691
ACSM1	6	5	0.524521676	0.627189622	2.639008396	0.156881322	0.886104691
AIFM1	6	5	0.524521676	0.627189622	2.639008396	0.156881322	0.886104691
AKNA	6	5	0.524521676	0.627189622	2.639008396	0.156881322	0.886104691
AKR1B15	6	5	0.524521676	0.627189622	2.639008396	0.156881322	0.886104691
ANGPT4	6	5	0.524521676	0.627189622	2.639008396	0.156881322	0.886104691
ANKRD27	6	5	0.524521676	0.627189622	2.639008396	0.156881322	0.886104691
APAF1	6	5	0.524521676	0.627189622	2.639008396	0.156881322	0.886104691
ARHGAP11A	6	5	0.524521676	0.627189622	2.639008396	0.156881322	0.886104691
ARHGAP4	6	5	0.524521676	0.627189622	2.639008396	0.156881322	0.886104691
ARSG	6	5	0.524521676	0.627189622	2.639008396	0.156881322	0.886104691
ATP13A4	6	5	0.524521676	0.627189622	2.639008396	0.156881322	0.886104691
BCAS3	6	5	0.524521676	0.627189622	2.639008396	0.156881322	0.886104691
BCR	6	5	0.524521676	0.627189622	2.639008396	0.156881322	0.886104691
BEST3	6	5	0.524521676	0.627189622	2.639008396	0.156881322	0.886104691
BTRC	6	5	0.524521676	0.627189622	2.639008396	0.156881322	0.886104691
C3	6	5	0.524521676	0.627189622	2.639008396	0.156881322	0.886104691
CCDC170	6	5	0.524521676	0.627189622	2.639008396	0.156881322	0.886104691
CD226	6	5	0.524521676	0.627189622	2.639008396	0.156881322	0.886104691
CDHR3	6	5	0.524521676	0.627189622	2.639008396	0.156881322	0.886104691
CELF4	6	5	0.524521676	0.627189622	2.639008396	0.156881322	0.886104691
CNGA3	6	5	0.524521676	0.627189622	2.639008396	0.156881322	0.886104691
CRNKL1	6	5	0.524521676	0.627189622	2.639008396	0.156881322	0.886104691
CSNK1A1L	6	5	0.524521676	0.627189622	2.639008396	0.156881322	0.886104691
CSRNP3	6	5	0.524521676	0.627189622	2.639008396	0.156881322	0.886104691
DDIAS	6	5	0.524521676	0.627189622	2.639008396	0.156881322	0.886104691
DHX34	6	5	0.524521676	0.627189622	2.639008396	0.156881322	0.886104691
DIP2A	6	5	0.524521676	0.627189622	2.639008396	0.156881322	0.886104691
DMP1	6	5	0.524521676	0.627189622	2.639008396	0.156881322	0.886104691
DNHD1	6	5	0.524521676	0.627189622	2.639008396	0.156881322	0.886104691
DOCK8	6	5	0.524521676	0.627189622	2.639008396	0.156881322	0.886104691
DOT1L	6	5	0.524521676	0.627189622	2.639008396	0.156881322	0.886104691
ECE2	6	5	0.524521676	0.627189622	2.639008396	0.156881322	0.886104691
EIF4ENIF1	6	5	0.524521676	0.627189622	2.639008396	0.156881322	0.886104691
ENOX2	6	5	0.524521676	0.627189622	2.639008396	0.156881322	0.886104691
EVI5	6	5	0.524521676	0.627189622	2.639008396	0.156881322	0.886104691
FAM65C	6	5	0.524521676	0.627189622	2.639008396	0.156881322	0.886104691
FBXO38	6	5	0.524521676	0.627189622	2.639008396	0.156881322	0.886104691

FKBP6	6	5	0.524521676	0.627189622	2.639008396	0.156881322	0.886104691
FMR1	6	5	0.524521676	0.627189622	2.639008396	0.156881322	0.886104691
GPR83	6	5	0.524521676	0.627189622	2.639008396	0.156881322	0.886104691
HTR3B	6	5	0.524521676	0.627189622	2.639008396	0.156881322	0.886104691
IL12RB2	6	5	0.524521676	0.627189622	2.639008396	0.156881322	0.886104691
IL18R1	6	5	0.524521676	0.627189622	2.639008396	0.156881322	0.886104691
JPH3	6	5	0.524521676	0.627189622	2.639008396	0.156881322	0.886104691
KDM5A	6	5	0.524521676	0.627189622	2.639008396	0.156881322	0.886104691
LMBRD2	6	5	0.524521676	0.627189622	2.639008396	0.156881322	0.886104691
MAPT	6	5	0.524521676	0.627189622	2.639008396	0.156881322	0.886104691
MDGA1	6	5	0.524521676	0.627189622	2.639008396	0.156881322	0.886104691
MLIP	6	5	0.524521676	0.627189622	2.639008396	0.156881322	0.886104691
MME	6	5	0.524521676	0.627189622	2.639008396	0.156881322	0.886104691
MYO1H	6	5	0.524521676	0.627189622	2.639008396	0.156881322	0.886104691
NTRK1	6	5	0.524521676	0.627189622	2.639008396	0.156881322	0.886104691
OAS2	6	5	0.524521676	0.627189622	2.639008396	0.156881322	0.886104691
OPN4	6	5	0.524521676	0.627189622	2.639008396	0.156881322	0.886104691
OR5B2	6	5	0.524521676	0.627189622	2.639008396	0.156881322	0.886104691
OR5M10	6	5	0.524521676	0.627189622	2.639008396	0.156881322	0.886104691
OR6B1	6	5	0.524521676	0.627189622	2.639008396	0.156881322	0.886104691
PCDHGB7	6	5	0.524521676	0.627189622	2.639008396	0.156881322	0.886104691
PCSK2	6	5	0.524521676	0.627189622	2.639008396	0.156881322	0.886104691
PDE6B	6	5	0.524521676	0.627189622	2.639008396	0.156881322	0.886104691
PI4KA	6	5	0.524521676	0.627189622	2.639008396	0.156881322	0.886104691
PKD2	6	5	0.524521676	0.627189622	2.639008396	0.156881322	0.886104691
PRDM10	6	5	0.524521676	0.627189622	2.639008396	0.156881322	0.886104691
PTGER3	6	5	0.524521676	0.627189622	2.639008396	0.156881322	0.886104691
RABEP2	6	5	0.524521676	0.627189622	2.639008396	0.156881322	0.886104691
RBM15	6	5	0.524521676	0.627189622	2.639008396	0.156881322	0.886104691
RNF165	6	5	0.524521676	0.627189622	2.639008396	0.156881322	0.886104691
RXFP3	6	5	0.524521676	0.627189622	2.639008396	0.156881322	0.886104691
SECISBP2L	6	5	0.524521676	0.627189622	2.639008396	0.156881322	0.886104691
SERPINB7	6	5	0.524521676	0.627189622	2.639008396	0.156881322	0.886104691
SETD1A	6	5	0.524521676	0.627189622	2.639008396	0.156881322	0.886104691
SEZ6	6	5	0.524521676	0.627189622	2.639008396	0.156881322	0.886104691
SIGLEC6	6	5	0.524521676	0.627189622	2.639008396	0.156881322	0.886104691
SLC22A7	6	5	0.524521676	0.627189622	2.639008396	0.156881322	0.886104691
SLC4A5	6	5	0.524521676	0.627189622	2.639008396	0.156881322	0.886104691
SLCO1B3	6	5	0.524521676	0.627189622	2.639008396	0.156881322	0.886104691
SMG8	6	5	0.524521676	0.627189622	2.639008396	0.156881322	0.886104691
SPAG6	6	5	0.524521676	0.627189622	2.639008396	0.156881322	0.886104691
SULF1	6	5	0.524521676	0.627189622	2.639008396	0.156881322	0.886104691
TLR10	6	5	0.524521676	0.627189622	2.639008396	0.156881322	0.886104691
TP53BP1	6	5	0.524521676	0.627189622	2.639008396	0.156881322	0.886104691
TRPM5	6	5	0.524521676	0.627189622	2.639008396	0.156881322	0.886104691
TTC3	6	5	0.524521676	0.627189622	2.639008396	0.156881322	0.886104691
UBE3A	6	5	0.524521676	0.627189622	2.639008396	0.156881322	0.886104691
UBR2	6	5	0.524521676	0.627189622	2.639008396	0.156881322	0.886104691
UCK2	6	5	0.524521676	0.627189622	2.639008396	0.156881322	0.886104691
UMODL1	6	5	0.524521676	0.627189622	2.639008396	0.156881322	0.886104691
UROC1	6	5	0.524521676	0.627189622	2.639008396	0.156881322	0.886104691
VPS16	6	5	0.524521676	0.627189622	2.639008396	0.156881322	0.886104691
VWA8	6	5	0.524521676	0.627189622	2.639008396	0.156881322	0.886104691
ZC3H12C	6	5	0.524521676	0.627189622	2.639008396	0.156881322	0.886104691
ZNF445	6	5	0.524521676	0.627189622	2.639008396	0.156881322	0.886104691
ZNF534	6	5	0.524521676	0.627189622	2.639008396	0.156881322	0.886104691
ZNF585A	6	5	0.524521676	0.627189622	2.639008396	0.156881322	0.886104691
ZNF680	6	5	0.524521676	0.627189622	2.639008396	0.156881322	0.886104691

LAMA3	18	7	0.526499838	1.377903192	3.986697916	0.535406405	0.889162347
MYH7	31	20	0.534076266	0.799359721	1.533746407	0.424758064	0.899204124
SVEP1	31	20	0.534076266	0.799359721	1.533746407	0.424758064	0.899204124
ALPK2	16	11	0.534146983	0.756263188	1.848328709	0.320792301	0.899204124
BRINP2	16	11	0.534146983	0.756263188	1.848328709	0.320792301	0.899204124
CACNA1F	16	11	0.534146983	0.756263188	1.848328709	0.320792301	0.899204124
CDH6	16	11	0.534146983	0.756263188	1.848328709	0.320792301	0.899204124
DGKI	16	11	0.534146983	0.756263188	1.848328709	0.320792301	0.899204124
FZD10	16	11	0.534146983	0.756263188	1.848328709	0.320792301	0.899204124
TBX22	16	11	0.534146983	0.756263188	1.848328709	0.320792301	0.899204124
ZIC1	16	11	0.534146983	0.756263188	1.848328709	0.320792301	0.899204124
TENM1	37	16	0.54259819	1.24899137	2.48667488	0.6529244	0.913140411
ANKRD30B	20	8	0.546280177	1.340294563	3.599100775	0.550529466	0.918459611
GABRG1	20	8	0.546280177	1.340294563	3.599100775	0.550529466	0.918459611
KLHL4	20	8	0.546280177	1.340294563	3.599100775	0.550529466	0.918459611
COL4A1	18	12	0.554435884	0.78003246	1.823180307	0.345405126	0.922601599
FBXL7	18	12	0.554435884	0.78003246	1.823180307	0.345405126	0.922601599
MYOM2	18	12	0.554435884	0.78003246	1.823180307	0.345405126	0.922601599
OR2G2	18	12	0.554435884	0.78003246	1.823180307	0.345405126	0.922601599
ADAMTS4	10	3	0.556730763	1.782294989	10.21520349	0.450730458	0.922601599
ARSF	10	3	0.556730763	1.782294989	10.21520349	0.450730458	0.922601599
BICD1	10	3	0.556730763	1.782294989	10.21520349	0.450730458	0.922601599
CAMK2B	10	3	0.556730763	1.782294989	10.21520349	0.450730458	0.922601599
CHRNA1	10	3	0.556730763	1.782294989	10.21520349	0.450730458	0.922601599
ERBB2	10	3	0.556730763	1.782294989	10.21520349	0.450730458	0.922601599
GLG1	10	3	0.556730763	1.782294989	10.21520349	0.450730458	0.922601599
IL31RA	10	3	0.556730763	1.782294989	10.21520349	0.450730458	0.922601599
KAT6A	10	3	0.556730763	1.782294989	10.21520349	0.450730458	0.922601599
MORC4	10	3	0.556730763	1.782294989	10.21520349	0.450730458	0.922601599
NEGR1	10	3	0.556730763	1.782294989	10.21520349	0.450730458	0.922601599
NUP98	10	3	0.556730763	1.782294989	10.21520349	0.450730458	0.922601599
PHRF1	10	3	0.556730763	1.782294989	10.21520349	0.450730458	0.922601599
SESTD1	10	3	0.556730763	1.782294989	10.21520349	0.450730458	0.922601599
TET3	10	3	0.556730763	1.782294989	10.21520349	0.450730458	0.922601599
ZIC3	10	3	0.556730763	1.782294989	10.21520349	0.450730458	0.922601599
AASDH	9	3	0.556946974	1.59922918	9.308465473	0.392224964	0.922601599
ABCA1	9	3	0.556946974	1.59922918	9.308465473	0.392224964	0.922601599
ANKRD55	9	3	0.556946974	1.59922918	9.308465473	0.392224964	0.922601599
APCS	9	3	0.556946974	1.59922918	9.308465473	0.392224964	0.922601599
CCDC88A	9	3	0.556946974	1.59922918	9.308465473	0.392224964	0.922601599
CLASP1	9	3	0.556946974	1.59922918	9.308465473	0.392224964	0.922601599
GAA	9	3	0.556946974	1.59922918	9.308465473	0.392224964	0.922601599
HDC	9	3	0.556946974	1.59922918	9.308465473	0.392224964	0.922601599
IGKV3D-11	9	3	0.556946974	1.59922918	9.308465473	0.392224964	0.922601599
KIAA1549	9	3	0.556946974	1.59922918	9.308465473	0.392224964	0.922601599
LAX1	9	3	0.556946974	1.59922918	9.308465473	0.392224964	0.922601599
MBD1	9	3	0.556946974	1.59922918	9.308465473	0.392224964	0.922601599
MIB1	9	3	0.556946974	1.59922918	9.308465473	0.392224964	0.922601599
NYAP2	9	3	0.556946974	1.59922918	9.308465473	0.392224964	0.922601599
OR4D10	9	3	0.556946974	1.59922918	9.308465473	0.392224964	0.922601599
OR51M1	9	3	0.556946974	1.59922918	9.308465473	0.392224964	0.922601599
OR5B21	9	3	0.556946974	1.59922918	9.308465473	0.392224964	0.922601599
PAMR1	9	3	0.556946974	1.59922918	9.308465473	0.392224964	0.922601599
PKNOX2	9	3	0.556946974	1.59922918	9.308465473	0.392224964	0.922601599
SAMSN1	9	3	0.556946974	1.59922918	9.308465473	0.392224964	0.922601599
SFMBT2	9	3	0.556946974	1.59922918	9.308465473	0.392224964	0.922601599
SLC6A2	9	3	0.556946974	1.59922918	9.308465473	0.392224964	0.922601599
SNCAIP	9	3	0.556946974	1.59922918	9.308465473	0.392224964	0.922601599

TF	9	3	0.556946974	1.59922918	9.308465473	0.392224964	0.922601599
TRBV3-1	9	3	0.556946974	1.59922918	9.308465473	0.392224964	0.922601599
ZFP36L1	9	3	0.556946974	1.59922918	9.308465473	0.392224964	0.922601599
ZNF695	9	3	0.556946974	1.59922918	9.308465473	0.392224964	0.922601599
AKAP9	22	9	0.563615001	1.311156669	3.314549872	0.56406426	0.933062604
COL7A1	22	9	0.563615001	1.311156669	3.314549872	0.56406426	0.933062604
ABCD2	8	6	0.571815719	0.696899359	2.480477135	0.208064633	0.933194779
ALS2	8	6	0.571815719	0.696899359	2.480477135	0.208064633	0.933194779
ATP12A	8	6	0.571815719	0.696899359	2.480477135	0.208064633	0.933194779
BPIFC	8	6	0.571815719	0.696899359	2.480477135	0.208064633	0.933194779
CACNA1H	8	6	0.571815719	0.696899359	2.480477135	0.208064633	0.933194779
CD22	8	6	0.571815719	0.696899359	2.480477135	0.208064633	0.933194779
CLTCL1	8	6	0.571815719	0.696899359	2.480477135	0.208064633	0.933194779
DDI1	8	6	0.571815719	0.696899359	2.480477135	0.208064633	0.933194779
ETNPPL	8	6	0.571815719	0.696899359	2.480477135	0.208064633	0.933194779
GPR148	8	6	0.571815719	0.696899359	2.480477135	0.208064633	0.933194779
GPR149	8	6	0.571815719	0.696899359	2.480477135	0.208064633	0.933194779
HIP1	8	6	0.571815719	0.696899359	2.480477135	0.208064633	0.933194779
IGSF3	8	6	0.571815719	0.696899359	2.480477135	0.208064633	0.933194779
ITGB8	8	6	0.571815719	0.696899359	2.480477135	0.208064633	0.933194779
KIF1B	8	6	0.571815719	0.696899359	2.480477135	0.208064633	0.933194779
LATS1	8	6	0.571815719	0.696899359	2.480477135	0.208064633	0.933194779
LILRA1	8	6	0.571815719	0.696899359	2.480477135	0.208064633	0.933194779
MAP3K9	8	6	0.571815719	0.696899359	2.480477135	0.208064633	0.933194779
MED1	8	6	0.571815719	0.696899359	2.480477135	0.208064633	0.933194779
NRG1	8	6	0.571815719	0.696899359	2.480477135	0.208064633	0.933194779
NRP2	8	6	0.571815719	0.696899359	2.480477135	0.208064633	0.933194779
OR11H6	8	6	0.571815719	0.696899359	2.480477135	0.208064633	0.933194779
OR4D6	8	6	0.571815719	0.696899359	2.480477135	0.208064633	0.933194779
OR5K3	8	6	0.571815719	0.696899359	2.480477135	0.208064633	0.933194779
PABPC5	8	6	0.571815719	0.696899359	2.480477135	0.208064633	0.933194779
LM2-AKA	8	6	0.571815719	0.696899359	2.480477135	0.208064633	0.933194779
PARK2	8	6	0.571815719	0.696899359	2.480477135	0.208064633	0.933194779
PLEKHA7	8	6	0.571815719	0.696899359	2.480477135	0.208064633	0.933194779
PLEKHH2	8	6	0.571815719	0.696899359	2.480477135	0.208064633	0.933194779
PSG9	8	6	0.571815719	0.696899359	2.480477135	0.208064633	0.933194779
RABGAP1I	8	6	0.571815719	0.696899359	2.480477135	0.208064633	0.933194779
REG1A	8	6	0.571815719	0.696899359	2.480477135	0.208064633	0.933194779
RFX6	8	6	0.571815719	0.696899359	2.480477135	0.208064633	0.933194779
P11-240B13	8	6	0.571815719	0.696899359	2.480477135	0.208064633	0.933194779
RPL10L	8	6	0.571815719	0.696899359	2.480477135	0.208064633	0.933194779
SEMA3C	8	6	0.571815719	0.696899359	2.480477135	0.208064633	0.933194779
SMO	8	6	0.571815719	0.696899359	2.480477135	0.208064633	0.933194779
TARS	8	6	0.571815719	0.696899359	2.480477135	0.208064633	0.933194779
TAS2R41	8	6	0.571815719	0.696899359	2.480477135	0.208064633	0.933194779
TRIM49C	8	6	0.571815719	0.696899359	2.480477135	0.208064633	0.933194779
TRPC3	8	6	0.571815719	0.696899359	2.480477135	0.208064633	0.933194779
UBE4B	8	6	0.571815719	0.696899359	2.480477135	0.208064633	0.933194779
USP51	8	6	0.571815719	0.696899359	2.480477135	0.208064633	0.933194779
ZKSCAN2	8	6	0.571815719	0.696899359	2.480477135	0.208064633	0.933194779
ZNF677	8	6	0.571815719	0.696899359	2.480477135	0.208064633	0.933194779
ZNF730	8	6	0.571815719	0.696899359	2.480477135	0.208064633	0.933194779
F5	20	13	0.572147092	0.800186224	1.800143941	0.367528584	0.933447385
ADCY8	24	10	0.57896275	1.287921867	3.096281512	0.576279365	0.944275562
ADAMTS2	22	14	0.587776597	0.817500338	1.778947034	0.387498194	0.958059542
RBM10	22	14	0.587776597	0.817500338	1.778947034	0.387498194	0.958059542
ADGRF4	11	4	0.593322844	1.466771853	6.41469774	0.426210416	0.959011478
CFAP46	11	4	0.593322844	1.466771853	6.41469774	0.426210416	0.959011478

CFAP74	11	4	0.593322844	1.466771853	6.41469774	0.426210416	0.959011478
CHD9	11	4	0.593322844	1.466771853	6.41469774	0.426210416	0.959011478
CLEC16A	11	4	0.593322844	1.466771853	6.41469774	0.426210416	0.959011478
CLTC	11	4	0.593322844	1.466771853	6.41469774	0.426210416	0.959011478
DRP2	11	4	0.593322844	1.466771853	6.41469774	0.426210416	0.959011478
FCRL4	11	4	0.593322844	1.466771853	6.41469774	0.426210416	0.959011478
ILDR2	11	4	0.593322844	1.466771853	6.41469774	0.426210416	0.959011478
KLHL6	11	4	0.593322844	1.466771853	6.41469774	0.426210416	0.959011478
MPO	11	4	0.593322844	1.466771853	6.41469774	0.426210416	0.959011478
OR5B12	11	4	0.593322844	1.466771853	6.41469774	0.426210416	0.959011478
SH3TC2	11	4	0.593322844	1.466771853	6.41469774	0.426210416	0.959011478
SHROOM3	11	4	0.593322844	1.466771853	6.41469774	0.426210416	0.959011478
SIGLEC8	11	4	0.593322844	1.466771853	6.41469774	0.426210416	0.959011478
STAB1	11	4	0.593322844	1.466771853	6.41469774	0.426210416	0.959011478
TBX3	11	4	0.593322844	1.466771853	6.41469774	0.426210416	0.959011478
UTP20	11	4	0.593322844	1.466771853	6.41469774	0.426210416	0.959011478
ZNF334	11	4	0.593322844	1.466771853	6.41469774	0.426210416	0.959011478
ADAM29	12	4	0.594353629	1.605024219	6.9360098	0.476775482	0.959011478
BICC1	12	4	0.594353629	1.605024219	6.9360098	0.476775482	0.959011478
CDYL	12	4	0.594353629	1.605024219	6.9360098	0.476775482	0.959011478
CFAP43	12	4	0.594353629	1.605024219	6.9360098	0.476775482	0.959011478
DRD2	12	4	0.594353629	1.605024219	6.9360098	0.476775482	0.959011478
IL1RL1	12	4	0.594353629	1.605024219	6.9360098	0.476775482	0.959011478
KRTAP24-1	12	4	0.594353629	1.605024219	6.9360098	0.476775482	0.959011478
LUM	12	4	0.594353629	1.605024219	6.9360098	0.476775482	0.959011478
MAGEE1	12	4	0.594353629	1.605024219	6.9360098	0.476775482	0.959011478
MYO5B	12	4	0.594353629	1.605024219	6.9360098	0.476775482	0.959011478
NPR3	12	4	0.594353629	1.605024219	6.9360098	0.476775482	0.959011478
NUP210L	12	4	0.594353629	1.605024219	6.9360098	0.476775482	0.959011478
TLR7	12	4	0.594353629	1.605024219	6.9360098	0.476775482	0.959011478
ZSCAN1	12	4	0.594353629	1.605024219	6.9360098	0.476775482	0.959011478
MAP2	23	15	0.594529061	0.795270661	1.689357829	0.38473336	0.959011478
ADAMTS17	10	7	0.607084597	0.746823946	2.357148643	0.251205177	0.971429702
AKAP13	10	7	0.607084597	0.746823946	2.357148643	0.251205177	0.971429702
ALDH1L1	10	7	0.607084597	0.746823946	2.357148643	0.251205177	0.971429702
ARHGEF12	10	7	0.607084597	0.746823946	2.357148643	0.251205177	0.971429702
CDH17	10	7	0.607084597	0.746823946	2.357148643	0.251205177	0.971429702
CHRDL1	10	7	0.607084597	0.746823946	2.357148643	0.251205177	0.971429702
CRB2	10	7	0.607084597	0.746823946	2.357148643	0.251205177	0.971429702
FAM71A	10	7	0.607084597	0.746823946	2.357148643	0.251205177	0.971429702
GRIK1	10	7	0.607084597	0.746823946	2.357148643	0.251205177	0.971429702
HAS2	10	7	0.607084597	0.746823946	2.357148643	0.251205177	0.971429702
HNF4A	10	7	0.607084597	0.746823946	2.357148643	0.251205177	0.971429702
KIAA2026	10	7	0.607084597	0.746823946	2.357148643	0.251205177	0.971429702
KRTAP11-1	10	7	0.607084597	0.746823946	2.357148643	0.251205177	0.971429702
NPY2R	10	7	0.607084597	0.746823946	2.357148643	0.251205177	0.971429702
OLFML2B	10	7	0.607084597	0.746823946	2.357148643	0.251205177	0.971429702
PCDHB13	10	7	0.607084597	0.746823946	2.357148643	0.251205177	0.971429702
PIK3C2G	10	7	0.607084597	0.746823946	2.357148643	0.251205177	0.971429702
REV3L	10	7	0.607084597	0.746823946	2.357148643	0.251205177	0.971429702
SRGAP1	10	7	0.607084597	0.746823946	2.357148643	0.251205177	0.971429702
SSTR4	10	7	0.607084597	0.746823946	2.357148643	0.251205177	0.971429702
TAF1	10	7	0.607084597	0.746823946	2.357148643	0.251205177	0.971429702
TRIML2	10	7	0.607084597	0.746823946	2.357148643	0.251205177	0.971429702
TTK	10	7	0.607084597	0.746823946	2.357148643	0.251205177	0.971429702
ZNF638	10	7	0.607084597	0.746823946	2.357148643	0.251205177	0.971429702
KDR	25	16	0.607189508	0.810555489	1.676838382	0.402167148	0.971429702
KLHL1	25	16	0.607189508	0.810555489	1.676838382	0.402167148	0.971429702

MYH13	25	16	0.607189508	0.810555489	1.676838382	0.402167148	0.971429702
MMRN1	26	16	0.614192625	0.845742814	1.741914203	0.422192283	0.982336525
ABCC8	13	5	0.62174985	1.387471529	5.058467685	0.454312821	0.991124761
CCDC141	13	5	0.62174985	1.387471529	5.058467685	0.454312821	0.991124761
CNTN4	13	5	0.62174985	1.387471529	5.058467685	0.454312821	0.991124761
DCAF8L1	13	5	0.62174985	1.387471529	5.058467685	0.454312821	0.991124761
INPP4B	13	5	0.62174985	1.387471529	5.058467685	0.454312821	0.991124761
LAMC3	13	5	0.62174985	1.387471529	5.058467685	0.454312821	0.991124761
MMP2	13	5	0.62174985	1.387471529	5.058467685	0.454312821	0.991124761
OR13G1	13	5	0.62174985	1.387471529	5.058467685	0.454312821	0.991124761
OR8H1	13	5	0.62174985	1.387471529	5.058467685	0.454312821	0.991124761
SMC4	13	5	0.62174985	1.387471529	5.058467685	0.454312821	0.991124761
SPATA18	13	5	0.62174985	1.387471529	5.058467685	0.454312821	0.991124761
ARHGAP36	14	5	0.623541354	1.498843696	5.411753962	0.499088712	0.992185309
DLG5	14	5	0.623541354	1.498843696	5.411753962	0.499088712	0.992185309
IGSF22	14	5	0.623541354	1.498843696	5.411753962	0.499088712	0.992185309
PDZRN4	14	5	0.623541354	1.498843696	5.411753962	0.499088712	0.992185309
RXFP1	14	5	0.623541354	1.498843696	5.411753962	0.499088712	0.992185309
SULF2	14	5	0.623541354	1.498843696	5.411753962	0.499088712	0.992185309
AXDND1	12	8	0.634668723	0.784290836	2.259345472	0.288051667	1
CES5A	12	8	0.634668723	0.784290836	2.259345472	0.288051667	1
COL5A3	12	8	0.634668723	0.784290836	2.259345472	0.288051667	1
EPRS	12	8	0.634668723	0.784290836	2.259345472	0.288051667	1
HCRTR2	12	8	0.634668723	0.784290836	2.259345472	0.288051667	1
KEL	12	8	0.634668723	0.784290836	2.259345472	0.288051667	1
MAP3K19	12	8	0.634668723	0.784290836	2.259345472	0.288051667	1
NCAPD3	12	8	0.634668723	0.784290836	2.259345472	0.288051667	1
OLFM4	12	8	0.634668723	0.784290836	2.259345472	0.288051667	1
OR8H3	12	8	0.634668723	0.784290836	2.259345472	0.288051667	1
PCDHB10	12	8	0.634668723	0.784290836	2.259345472	0.288051667	1
PXDN	12	8	0.634668723	0.784290836	2.259345472	0.288051667	1
TAS2R1	12	8	0.634668723	0.784290836	2.259345472	0.288051667	1
TIAM1	34	15	0.635666715	1.219395667	2.488210323	0.62366059	1
NLRP11	15	6	0.644745394	1.334754551	4.281123898	0.478116788	1
PCNT	15	6	0.644745394	1.334754551	4.281123898	0.478116788	1
PRRC2C	15	6	0.644745394	1.334754551	4.281123898	0.478116788	1
ALPK1	16	6	0.647026122	1.428214078	4.544167487	0.518410471	1
OR2T6	16	6	0.647026122	1.428214078	4.544167487	0.518410471	1
ZCCHC5	16	6	0.647026122	1.428214078	4.544167487	0.518410471	1
MYH1	38	17	0.652146027	1.20374935	2.354357026	0.638155051	1
ACSM5	14	9	0.656992242	0.813496834	2.179774642	0.319883572	1
ACTRT1	14	9	0.656992242	0.813496834	2.179774642	0.319883572	1
ANKRD17	14	9	0.656992242	0.813496834	2.179774642	0.319883572	1
APC	14	9	0.656992242	0.813496834	2.179774642	0.319883572	1
COL9A1	14	9	0.656992242	0.813496834	2.179774642	0.319883572	1
DAB1	14	9	0.656992242	0.813496834	2.179774642	0.319883572	1
KCNQ2	14	9	0.656992242	0.813496834	2.179774642	0.319883572	1
OR5D14	14	9	0.656992242	0.813496834	2.179774642	0.319883572	1
PTCHD3	14	9	0.656992242	0.813496834	2.179774642	0.319883572	1
SEMA3D	14	9	0.656992242	0.813496834	2.179774642	0.319883572	1
TACC2	14	9	0.656992242	0.813496834	2.179774642	0.319883572	1
ZFHX3	14	9	0.656992242	0.813496834	2.179774642	0.319883572	1
TRPV5	17	7	0.663834442	1.297237396	3.778545851	0.498699172	1
ABCB11	15	10	0.666442061	0.782182466	1.994252055	0.320255053	1
CFAP61	15	10	0.666442061	0.782182466	1.994252055	0.320255053	1
DACH2	15	10	0.666442061	0.782182466	1.994252055	0.320255053	1
GREB1	15	10	0.666442061	0.782182466	1.994252055	0.320255053	1
MDN1	15	10	0.666442061	0.782182466	1.994252055	0.320255053	1

OCA2	15	10	0.666442061	0.782182466	1.994252055	0.320255053	1
OR2T34	15	10	0.666442061	0.782182466	1.994252055	0.320255053	1
TECTA	15	10	0.666442061	0.782182466	1.994252055	0.320255053	1
ZBBX	15	10	0.666442061	0.782182466	1.994252055	0.320255053	1
AADACL4	5	1	0.669456871	2.661266568	126.7430256	0.294373938	1
ABHD2	5	1	0.669456871	2.661266568	126.7430256	0.294373938	1
AGTPBP1	5	1	0.669456871	2.661266568	126.7430256	0.294373938	1
AMFR	5	1	0.669456871	2.661266568	126.7430256	0.294373938	1
ARSI	5	1	0.669456871	2.661266568	126.7430256	0.294373938	1
BBS10	5	1	0.669456871	2.661266568	126.7430256	0.294373938	1
BTBD7	5	1	0.669456871	2.661266568	126.7430256	0.294373938	1
BTD	5	1	0.669456871	2.661266568	126.7430256	0.294373938	1
C11orf87	5	1	0.669456871	2.661266568	126.7430256	0.294373938	1
C2CD2L	5	1	0.669456871	2.661266568	126.7430256	0.294373938	1
C3orf67	5	1	0.669456871	2.661266568	126.7430256	0.294373938	1
CALB1	5	1	0.669456871	2.661266568	126.7430256	0.294373938	1
CBX2	5	1	0.669456871	2.661266568	126.7430256	0.294373938	1
CCR2	5	1	0.669456871	2.661266568	126.7430256	0.294373938	1
CD101	5	1	0.669456871	2.661266568	126.7430256	0.294373938	1
CD300LB	5	1	0.669456871	2.661266568	126.7430256	0.294373938	1
CD40LG	5	1	0.669456871	2.661266568	126.7430256	0.294373938	1
CDKAL1	5	1	0.669456871	2.661266568	126.7430256	0.294373938	1
CES1	5	1	0.669456871	2.661266568	126.7430256	0.294373938	1
CFC1	5	1	0.669456871	2.661266568	126.7430256	0.294373938	1
CLCN2	5	1	0.669456871	2.661266568	126.7430256	0.294373938	1
CLDN17	5	1	0.669456871	2.661266568	126.7430256	0.294373938	1
CYP2C18	5	1	0.669456871	2.661266568	126.7430256	0.294373938	1
CYP46A1	5	1	0.669456871	2.661266568	126.7430256	0.294373938	1
CYP4F11	5	1	0.669456871	2.661266568	126.7430256	0.294373938	1
CYTIP	5	1	0.669456871	2.661266568	126.7430256	0.294373938	1
DCAF13	5	1	0.669456871	2.661266568	126.7430256	0.294373938	1
DPH2	5	1	0.669456871	2.661266568	126.7430256	0.294373938	1
DPYSL3	5	1	0.669456871	2.661266568	126.7430256	0.294373938	1
EFCAB12	5	1	0.669456871	2.661266568	126.7430256	0.294373938	1
EPB41L4B	5	1	0.669456871	2.661266568	126.7430256	0.294373938	1
ERN1	5	1	0.669456871	2.661266568	126.7430256	0.294373938	1
EXOC2	5	1	0.669456871	2.661266568	126.7430256	0.294373938	1
FAM196A	5	1	0.669456871	2.661266568	126.7430256	0.294373938	1
FHOD1	5	1	0.669456871	2.661266568	126.7430256	0.294373938	1
FURIN	5	1	0.669456871	2.661266568	126.7430256	0.294373938	1
FUT8	5	1	0.669456871	2.661266568	126.7430256	0.294373938	1
FZD2	5	1	0.669456871	2.661266568	126.7430256	0.294373938	1
GCM2	5	1	0.669456871	2.661266568	126.7430256	0.294373938	1
GDF9	5	1	0.669456871	2.661266568	126.7430256	0.294373938	1
GNL2	5	1	0.669456871	2.661266568	126.7430256	0.294373938	1
GPR132	5	1	0.669456871	2.661266568	126.7430256	0.294373938	1
GRHL1	5	1	0.669456871	2.661266568	126.7430256	0.294373938	1
HAPLN3	5	1	0.669456871	2.661266568	126.7430256	0.294373938	1
HCFC2	5	1	0.669456871	2.661266568	126.7430256	0.294373938	1
HEXA	5	1	0.669456871	2.661266568	126.7430256	0.294373938	1
HMGCS1	5	1	0.669456871	2.661266568	126.7430256	0.294373938	1
HSP90AA1	5	1	0.669456871	2.661266568	126.7430256	0.294373938	1
HSPA12B	5	1	0.669456871	2.661266568	126.7430256	0.294373938	1
IGHV5-51	5	1	0.669456871	2.661266568	126.7430256	0.294373938	1
IGSF5	5	1	0.669456871	2.661266568	126.7430256	0.294373938	1
IMPDH1	5	1	0.669456871	2.661266568	126.7430256	0.294373938	1
IPO7	5	1	0.669456871	2.661266568	126.7430256	0.294373938	1
KBTBD6	5	1	0.669456871	2.661266568	126.7430256	0.294373938	1

KCNC4	5	1	0.669456871	2.661266568	126.7430256	0.294373938	1
KCNS3	5	1	0.669456871	2.661266568	126.7430256	0.294373938	1
KIAA0391	5	1	0.669456871	2.661266568	126.7430256	0.294373938	1
KIAA0895	5	1	0.669456871	2.661266568	126.7430256	0.294373938	1
KIAA1644	5	1	0.669456871	2.661266568	126.7430256	0.294373938	1
KIAA1683	5	1	0.669456871	2.661266568	126.7430256	0.294373938	1
KIF1C	5	1	0.669456871	2.661266568	126.7430256	0.294373938	1
KRTAP19-7	5	1	0.669456871	2.661266568	126.7430256	0.294373938	1
LAS1L	5	1	0.669456871	2.661266568	126.7430256	0.294373938	1
LIN9	5	1	0.669456871	2.661266568	126.7430256	0.294373938	1
LRP10	5	1	0.669456871	2.661266568	126.7430256	0.294373938	1
MAPK7	5	1	0.669456871	2.661266568	126.7430256	0.294373938	1
MKLN1	5	1	0.669456871	2.661266568	126.7430256	0.294373938	1
MOXD1	5	1	0.669456871	2.661266568	126.7430256	0.294373938	1
MRGPRX1	5	1	0.669456871	2.661266568	126.7430256	0.294373938	1
NBPF11	5	1	0.669456871	2.661266568	126.7430256	0.294373938	1
NCOA5	5	1	0.669456871	2.661266568	126.7430256	0.294373938	1
NFXL1	5	1	0.669456871	2.661266568	126.7430256	0.294373938	1
NGF	5	1	0.669456871	2.661266568	126.7430256	0.294373938	1
NKRF	5	1	0.669456871	2.661266568	126.7430256	0.294373938	1
NKX2-1	5	1	0.669456871	2.661266568	126.7430256	0.294373938	1
NKX6-1	5	1	0.669456871	2.661266568	126.7430256	0.294373938	1
NLGN2	5	1	0.669456871	2.661266568	126.7430256	0.294373938	1
OIT3	5	1	0.669456871	2.661266568	126.7430256	0.294373938	1
OPN1SW	5	1	0.669456871	2.661266568	126.7430256	0.294373938	1
OR10H2	5	1	0.669456871	2.661266568	126.7430256	0.294373938	1
OR13C9	5	1	0.669456871	2.661266568	126.7430256	0.294373938	1
OR14J1	5	1	0.669456871	2.661266568	126.7430256	0.294373938	1
OR51B2	5	1	0.669456871	2.661266568	126.7430256	0.294373938	1
OR52N5	5	1	0.669456871	2.661266568	126.7430256	0.294373938	1
OR56A5	5	1	0.669456871	2.661266568	126.7430256	0.294373938	1
OR6V1	5	1	0.669456871	2.661266568	126.7430256	0.294373938	1
PAPLN	5	1	0.669456871	2.661266568	126.7430256	0.294373938	1
PARP3	5	1	0.669456871	2.661266568	126.7430256	0.294373938	1
PDE9A	5	1	0.669456871	2.661266568	126.7430256	0.294373938	1
PFKFB1	5	1	0.669456871	2.661266568	126.7430256	0.294373938	1
PHF2	5	1	0.669456871	2.661266568	126.7430256	0.294373938	1
PLA2G3	5	1	0.669456871	2.661266568	126.7430256	0.294373938	1
PLEKHH1	5	1	0.669456871	2.661266568	126.7430256	0.294373938	1
PLK2	5	1	0.669456871	2.661266568	126.7430256	0.294373938	1
PMS2	5	1	0.669456871	2.661266568	126.7430256	0.294373938	1
PNPLA8	5	1	0.669456871	2.661266568	126.7430256	0.294373938	1
PODN	5	1	0.669456871	2.661266568	126.7430256	0.294373938	1
PPP2R5D	5	1	0.669456871	2.661266568	126.7430256	0.294373938	1
PRDM7	5	1	0.669456871	2.661266568	126.7430256	0.294373938	1
PRR23B	5	1	0.669456871	2.661266568	126.7430256	0.294373938	1
PSEN1	5	1	0.669456871	2.661266568	126.7430256	0.294373938	1
PTGIR	5	1	0.669456871	2.661266568	126.7430256	0.294373938	1
PTX4	5	1	0.669456871	2.661266568	126.7430256	0.294373938	1
RABEPK	5	1	0.669456871	2.661266568	126.7430256	0.294373938	1
RABGGTB	5	1	0.669456871	2.661266568	126.7430256	0.294373938	1
RASAL3	5	1	0.669456871	2.661266568	126.7430256	0.294373938	1
RCOR1	5	1	0.669456871	2.661266568	126.7430256	0.294373938	1
RECQL5	5	1	0.669456871	2.661266568	126.7430256	0.294373938	1
RERGL	5	1	0.669456871	2.661266568	126.7430256	0.294373938	1
RMND5A	5	1	0.669456871	2.661266568	126.7430256	0.294373938	1
RORA	5	1	0.669456871	2.661266568	126.7430256	0.294373938	1
RUSC2	5	1	0.669456871	2.661266568	126.7430256	0.294373938	1

SCNN1B	5	1	0.669456871	2.661266568	126.7430256	0.294373938	1
SCNN1D	5	1	0.669456871	2.661266568	126.7430256	0.294373938	1
SEL1L3	5	1	0.669456871	2.661266568	126.7430256	0.294373938	1
SKAP2	5	1	0.669456871	2.661266568	126.7430256	0.294373938	1
SKIV2L	5	1	0.669456871	2.661266568	126.7430256	0.294373938	1
SLC16A7	5	1	0.669456871	2.661266568	126.7430256	0.294373938	1
SLC17A7	5	1	0.669456871	2.661266568	126.7430256	0.294373938	1
SLC44A3	5	1	0.669456871	2.661266568	126.7430256	0.294373938	1
SNX29	5	1	0.669456871	2.661266568	126.7430256	0.294373938	1
SOCS5	5	1	0.669456871	2.661266568	126.7430256	0.294373938	1
SRL	5	1	0.669456871	2.661266568	126.7430256	0.294373938	1
SRP54	5	1	0.669456871	2.661266568	126.7430256	0.294373938	1
SRP72	5	1	0.669456871	2.661266568	126.7430256	0.294373938	1
STAU2	5	1	0.669456871	2.661266568	126.7430256	0.294373938	1
SYT1	5	1	0.669456871	2.661266568	126.7430256	0.294373938	1
TAAR8	5	1	0.669456871	2.661266568	126.7430256	0.294373938	1
TAL1	5	1	0.669456871	2.661266568	126.7430256	0.294373938	1
TAS2R38	5	1	0.669456871	2.661266568	126.7430256	0.294373938	1
TCL1B	5	1	0.669456871	2.661266568	126.7430256	0.294373938	1
TLK1	5	1	0.669456871	2.661266568	126.7430256	0.294373938	1
TMEM255A	5	1	0.669456871	2.661266568	126.7430256	0.294373938	1
TNFRSF11A	5	1	0.669456871	2.661266568	126.7430256	0.294373938	1
TPP1	5	1	0.669456871	2.661266568	126.7430256	0.294373938	1
TRBV24-1	5	1	0.669456871	2.661266568	126.7430256	0.294373938	1
TRIM27	5	1	0.669456871	2.661266568	126.7430256	0.294373938	1
USP49	5	1	0.669456871	2.661266568	126.7430256	0.294373938	1
VSIG1	5	1	0.669456871	2.661266568	126.7430256	0.294373938	1
WBSCR28	5	1	0.669456871	2.661266568	126.7430256	0.294373938	1
ac-BPG116M	5	1	0.669456871	2.661266568	126.7430256	0.294373938	1
ZFYVE16	5	1	0.669456871	2.661266568	126.7430256	0.294373938	1
ZMYM4	5	1	0.669456871	2.661266568	126.7430256	0.294373938	1
ZNF169	5	1	0.669456871	2.661266568	126.7430256	0.294373938	1
ZNF180	5	1	0.669456871	2.661266568	126.7430256	0.294373938	1
ZNF274	5	1	0.669456871	2.661266568	126.7430256	0.294373938	1
ZNF28	5	1	0.669456871	2.661266568	126.7430256	0.294373938	1
ZNF480	5	1	0.669456871	2.661266568	126.7430256	0.294373938	1
ZNF546	5	1	0.669456871	2.661266568	126.7430256	0.294373938	1
ZNF558	5	1	0.669456871	2.661266568	126.7430256	0.294373938	1
ZNF559	5	1	0.669456871	2.661266568	126.7430256	0.294373938	1
ZNF571	5	1	0.669456871	2.661266568	126.7430256	0.294373938	1
ABCA9	16	10	0.675528996	0.836922313	2.113845576	0.347750737	1
ADAMTSL1	16	10	0.675528996	0.836922313	2.113845576	0.347750737	1
CCDC108	16	10	0.675528996	0.836922313	2.113845576	0.347750737	1
COL21A1	16	10	0.675528996	0.836922313	2.113845576	0.347750737	1
EPB41L3	16	10	0.675528996	0.836922313	2.113845576	0.347750737	1
FSIP2	16	10	0.675528996	0.836922313	2.113845576	0.347750737	1
EPHA7	19	8	0.680002519	1.269169368	3.428014395	0.516670067	1
PDE10A	19	8	0.680002519	1.269169368	3.428014395	0.516670067	1
BDP1	17	11	0.682834785	0.806042488	1.95293964	0.346398367	1
OR11H1	17	11	0.682834785	0.806042488	1.95293964	0.346398367	1
PCDHA5	17	11	0.682834785	0.806042488	1.95293964	0.346398367	1
SMG1	17	11	0.682834785	0.806042488	1.95293964	0.346398367	1
TRIM51	17	11	0.682834785	0.806042488	1.95293964	0.346398367	1
ZNF716	17	11	0.682834785	0.806042488	1.95293964	0.346398367	1
ABCA13	44	26	0.684175242	0.876834102	1.547904273	0.504582286	1
OBSCN	44	26	0.684175242	0.876834102	1.547904273	0.504582286	1
ADAMTS5	18	11	0.691233241	0.856145279	2.058189282	0.372315204	1
IGSF10	18	11	0.691233241	0.856145279	2.058189282	0.372315204	1

KIAA1210	18	11	0.691233241	0.856145279	2.058189282	0.372315204	1
SHANK1	18	11	0.691233241	0.856145279	2.058189282	0.372315204	1
TRO	18	11	0.691233241	0.856145279	2.058189282	0.372315204	1
DCAF12L2	21	9	0.693918814	1.247478784	3.169331871	0.532604307	1
KMT2A	21	9	0.693918814	1.247478784	3.169331871	0.532604307	1
XYLT1	21	9	0.693918814	1.247478784	3.169331871	0.532604307	1
GABRA4	19	12	0.696908679	0.825974229	1.916625477	0.36972043	1
CDH10	52	30	0.703402222	0.898965459	1.529530982	0.535463431	1
FAM83B	20	12	0.704753587	0.872217575	2.010508264	0.394206684	1
GRM1	20	12	0.704753587	0.872217575	2.010508264	0.394206684	1
PCDH18	20	12	0.704753587	0.872217575	2.010508264	0.394206684	1
PTPRC	20	12	0.704753587	0.872217575	2.010508264	0.394206684	1
TSHZ2	20	12	0.704753587	0.872217575	2.010508264	0.394206684	1
HGF	21	13	0.709157946	0.842885381	1.884521091	0.390616766	1
PDGFRA	21	13	0.709157946	0.842885381	1.884521091	0.390616766	1
TLL1	21	13	0.709157946	0.842885381	1.884521091	0.390616766	1
FSTL5	22	13	0.716548298	0.885867348	1.969410133	0.413930147	1
OR4C15	22	13	0.716548298	0.885867348	1.969410133	0.413930147	1
ABCB8	6	2	0.720305907	1.593544095	16.31619247	0.281002957	1
ACSBG1	6	2	0.720305907	1.593544095	16.31619247	0.281002957	1
ADAM30	6	2	0.720305907	1.593544095	16.31619247	0.281002957	1
ALB	6	2	0.720305907	1.593544095	16.31619247	0.281002957	1
ALG13	6	2	0.720305907	1.593544095	16.31619247	0.281002957	1
AP1G1	6	2	0.720305907	1.593544095	16.31619247	0.281002957	1
AR	6	2	0.720305907	1.593544095	16.31619247	0.281002957	1
ARNTL2	6	2	0.720305907	1.593544095	16.31619247	0.281002957	1
ATP13A2	6	2	0.720305907	1.593544095	16.31619247	0.281002957	1
ATP13A3	6	2	0.720305907	1.593544095	16.31619247	0.281002957	1
ATP2A1	6	2	0.720305907	1.593544095	16.31619247	0.281002957	1
ATP2A3	6	2	0.720305907	1.593544095	16.31619247	0.281002957	1
C3orf30	6	2	0.720305907	1.593544095	16.31619247	0.281002957	1
C7orf62	6	2	0.720305907	1.593544095	16.31619247	0.281002957	1
C8orf74	6	2	0.720305907	1.593544095	16.31619247	0.281002957	1
CC2D1A	6	2	0.720305907	1.593544095	16.31619247	0.281002957	1
CCDC81	6	2	0.720305907	1.593544095	16.31619247	0.281002957	1
CCDC89	6	2	0.720305907	1.593544095	16.31619247	0.281002957	1
CD4	6	2	0.720305907	1.593544095	16.31619247	0.281002957	1
CHML	6	2	0.720305907	1.593544095	16.31619247	0.281002957	1
CHRNB3	6	2	0.720305907	1.593544095	16.31619247	0.281002957	1
CPA6	6	2	0.720305907	1.593544095	16.31619247	0.281002957	1
CPN2	6	2	0.720305907	1.593544095	16.31619247	0.281002957	1
CXorf21	6	2	0.720305907	1.593544095	16.31619247	0.281002957	1
DEPDC1	6	2	0.720305907	1.593544095	16.31619247	0.281002957	1
DMRT1	6	2	0.720305907	1.593544095	16.31619247	0.281002957	1
DNAJB8	6	2	0.720305907	1.593544095	16.31619247	0.281002957	1
DPYSL5	6	2	0.720305907	1.593544095	16.31619247	0.281002957	1
EPN2	6	2	0.720305907	1.593544095	16.31619247	0.281002957	1
EXT2	6	2	0.720305907	1.593544095	16.31619247	0.281002957	1
FANCB	6	2	0.720305907	1.593544095	16.31619247	0.281002957	1
FBXO18	6	2	0.720305907	1.593544095	16.31619247	0.281002957	1
FCN1	6	2	0.720305907	1.593544095	16.31619247	0.281002957	1
GABBR1	6	2	0.720305907	1.593544095	16.31619247	0.281002957	1
GDPD4	6	2	0.720305907	1.593544095	16.31619247	0.281002957	1
GJA9	6	2	0.720305907	1.593544095	16.31619247	0.281002957	1
GK2	6	2	0.720305907	1.593544095	16.31619247	0.281002957	1
GLA	6	2	0.720305907	1.593544095	16.31619247	0.281002957	1
GPR35	6	2	0.720305907	1.593544095	16.31619247	0.281002957	1
GPR61	6	2	0.720305907	1.593544095	16.31619247	0.281002957	1

GRIN1	6	2	0.720305907	1.593544095	16.31619247	0.281002957	1
HOMEZ	6	2	0.720305907	1.593544095	16.31619247	0.281002957	1
HOXA2	6	2	0.720305907	1.593544095	16.31619247	0.281002957	1
HTR7	6	2	0.720305907	1.593544095	16.31619247	0.281002957	1
HTRA1	6	2	0.720305907	1.593544095	16.31619247	0.281002957	1
IFT88	6	2	0.720305907	1.593544095	16.31619247	0.281002957	1
IGHD	6	2	0.720305907	1.593544095	16.31619247	0.281002957	1
IL10RA	6	2	0.720305907	1.593544095	16.31619247	0.281002957	1
IL13RA2	6	2	0.720305907	1.593544095	16.31619247	0.281002957	1
IRF2BP2	6	2	0.720305907	1.593544095	16.31619247	0.281002957	1
ITIH1	6	2	0.720305907	1.593544095	16.31619247	0.281002957	1
KDM4A	6	2	0.720305907	1.593544095	16.31619247	0.281002957	1
KIF26A	6	2	0.720305907	1.593544095	16.31619247	0.281002957	1
KRT36	6	2	0.720305907	1.593544095	16.31619247	0.281002957	1
LINGO1	6	2	0.720305907	1.593544095	16.31619247	0.281002957	1
LTK	6	2	0.720305907	1.593544095	16.31619247	0.281002957	1
MAP10	6	2	0.720305907	1.593544095	16.31619247	0.281002957	1
MAP2K4	6	2	0.720305907	1.593544095	16.31619247	0.281002957	1
MATN4	6	2	0.720305907	1.593544095	16.31619247	0.281002957	1
MIB2	6	2	0.720305907	1.593544095	16.31619247	0.281002957	1
MSH6	6	2	0.720305907	1.593544095	16.31619247	0.281002957	1
MYO1C	6	2	0.720305907	1.593544095	16.31619247	0.281002957	1
MYO6	6	2	0.720305907	1.593544095	16.31619247	0.281002957	1
NCAPH	6	2	0.720305907	1.593544095	16.31619247	0.281002957	1
OR52E4	6	2	0.720305907	1.593544095	16.31619247	0.281002957	1
OR8B4	6	2	0.720305907	1.593544095	16.31619247	0.281002957	1
PADI2	6	2	0.720305907	1.593544095	16.31619247	0.281002957	1
PARP4	6	2	0.720305907	1.593544095	16.31619247	0.281002957	1
PDS5B	6	2	0.720305907	1.593544095	16.31619247	0.281002957	1
PFAS	6	2	0.720305907	1.593544095	16.31619247	0.281002957	1
PGLYRP4	6	2	0.720305907	1.593544095	16.31619247	0.281002957	1
PGM2	6	2	0.720305907	1.593544095	16.31619247	0.281002957	1
PIP4K2C	6	2	0.720305907	1.593544095	16.31619247	0.281002957	1
PLOD2	6	2	0.720305907	1.593544095	16.31619247	0.281002957	1
PPM1B	6	2	0.720305907	1.593544095	16.31619247	0.281002957	1
PRAMEF17	6	2	0.720305907	1.593544095	16.31619247	0.281002957	1
PRLHR	6	2	0.720305907	1.593544095	16.31619247	0.281002957	1
PRPF8	6	2	0.720305907	1.593544095	16.31619247	0.281002957	1
RAD54L	6	2	0.720305907	1.593544095	16.31619247	0.281002957	1
RCBTB2	6	2	0.720305907	1.593544095	16.31619247	0.281002957	1
RIMS4	6	2	0.720305907	1.593544095	16.31619247	0.281002957	1
RIT2	6	2	0.720305907	1.593544095	16.31619247	0.281002957	1
RSPO2	6	2	0.720305907	1.593544095	16.31619247	0.281002957	1
SAP130	6	2	0.720305907	1.593544095	16.31619247	0.281002957	1
SCML4	6	2	0.720305907	1.593544095	16.31619247	0.281002957	1
SECISBP2	6	2	0.720305907	1.593544095	16.31619247	0.281002957	1
SERPINB13	6	2	0.720305907	1.593544095	16.31619247	0.281002957	1
SKOR1	6	2	0.720305907	1.593544095	16.31619247	0.281002957	1
SLC17A9	6	2	0.720305907	1.593544095	16.31619247	0.281002957	1
SLC22A2	6	2	0.720305907	1.593544095	16.31619247	0.281002957	1
SLC4A7	6	2	0.720305907	1.593544095	16.31619247	0.281002957	1
SLC6A9	6	2	0.720305907	1.593544095	16.31619247	0.281002957	1
SLCO2A1	6	2	0.720305907	1.593544095	16.31619247	0.281002957	1
SMG7	6	2	0.720305907	1.593544095	16.31619247	0.281002957	1
SNX14	6	2	0.720305907	1.593544095	16.31619247	0.281002957	1
SPOPL	6	2	0.720305907	1.593544095	16.31619247	0.281002957	1
ST14	6	2	0.720305907	1.593544095	16.31619247	0.281002957	1
T6GALNAC	6	2	0.720305907	1.593544095	16.31619247	0.281002957	1

SYN1	6	2	0.720305907	1.593544095	16.31619247	0.281002957	1
SYT11	6	2	0.720305907	1.593544095	16.31619247	0.281002957	1
TAS2R60	6	2	0.720305907	1.593544095	16.31619247	0.281002957	1
TBC1D14	6	2	0.720305907	1.593544095	16.31619247	0.281002957	1
TIMELESS	6	2	0.720305907	1.593544095	16.31619247	0.281002957	1
TLR1	6	2	0.720305907	1.593544095	16.31619247	0.281002957	1
TMEM55A	6	2	0.720305907	1.593544095	16.31619247	0.281002957	1
TPP2	6	2	0.720305907	1.593544095	16.31619247	0.281002957	1
TRIM24	6	2	0.720305907	1.593544095	16.31619247	0.281002957	1
TRIM36	6	2	0.720305907	1.593544095	16.31619247	0.281002957	1
TRPC1	6	2	0.720305907	1.593544095	16.31619247	0.281002957	1
VCP	6	2	0.720305907	1.593544095	16.31619247	0.281002957	1
XPO5	6	2	0.720305907	1.593544095	16.31619247	0.281002957	1
ZBED4	6	2	0.720305907	1.593544095	16.31619247	0.281002957	1
ZBTB18	6	2	0.720305907	1.593544095	16.31619247	0.281002957	1
ZKSCAN1	6	2	0.720305907	1.593544095	16.31619247	0.281002957	1
ZMAT1	6	2	0.720305907	1.593544095	16.31619247	0.281002957	1
ZNF157	6	2	0.720305907	1.593544095	16.31619247	0.281002957	1
ZNF320	6	2	0.720305907	1.593544095	16.31619247	0.281002957	1
ZNF737	6	2	0.720305907	1.593544095	16.31619247	0.281002957	1
AARS2	7	2	0.72521789	1.864557063	18.59041975	0.349802733	1
ACAD11	7	2	0.72521789	1.864557063	18.59041975	0.349802733	1
ACAP2	7	2	0.72521789	1.864557063	18.59041975	0.349802733	1
ACE2	7	2	0.72521789	1.864557063	18.59041975	0.349802733	1
ADCY4	7	2	0.72521789	1.864557063	18.59041975	0.349802733	1
ADGRF5	7	2	0.72521789	1.864557063	18.59041975	0.349802733	1
ARHGEF28	7	2	0.72521789	1.864557063	18.59041975	0.349802733	1
ASB12	7	2	0.72521789	1.864557063	18.59041975	0.349802733	1
BTN1A1	7	2	0.72521789	1.864557063	18.59041975	0.349802733	1
CD33	7	2	0.72521789	1.864557063	18.59041975	0.349802733	1
CENPI	7	2	0.72521789	1.864557063	18.59041975	0.349802733	1
CPA2	7	2	0.72521789	1.864557063	18.59041975	0.349802733	1
CUL7	7	2	0.72521789	1.864557063	18.59041975	0.349802733	1
CYLD	7	2	0.72521789	1.864557063	18.59041975	0.349802733	1
DENND5B	7	2	0.72521789	1.864557063	18.59041975	0.349802733	1
DPY19L3	7	2	0.72521789	1.864557063	18.59041975	0.349802733	1
ELAVL4	7	2	0.72521789	1.864557063	18.59041975	0.349802733	1
FNDC7	7	2	0.72521789	1.864557063	18.59041975	0.349802733	1
FRMD3	7	2	0.72521789	1.864557063	18.59041975	0.349802733	1
GALNT2	7	2	0.72521789	1.864557063	18.59041975	0.349802733	1
HHIPL1	7	2	0.72521789	1.864557063	18.59041975	0.349802733	1
HZGJ	7	2	0.72521789	1.864557063	18.59041975	0.349802733	1
ITGB2	7	2	0.72521789	1.864557063	18.59041975	0.349802733	1
KMO	7	2	0.72521789	1.864557063	18.59041975	0.349802733	1
KRT28	7	2	0.72521789	1.864557063	18.59041975	0.349802733	1
KRTAP15-1	7	2	0.72521789	1.864557063	18.59041975	0.349802733	1
LIG1	7	2	0.72521789	1.864557063	18.59041975	0.349802733	1
MS4A6A	7	2	0.72521789	1.864557063	18.59041975	0.349802733	1
MTUS1	7	2	0.72521789	1.864557063	18.59041975	0.349802733	1
NUAK1	7	2	0.72521789	1.864557063	18.59041975	0.349802733	1
OGDH	7	2	0.72521789	1.864557063	18.59041975	0.349802733	1
OR12D2	7	2	0.72521789	1.864557063	18.59041975	0.349802733	1
OR51F2	7	2	0.72521789	1.864557063	18.59041975	0.349802733	1
PARVG	7	2	0.72521789	1.864557063	18.59041975	0.349802733	1
PI15	7	2	0.72521789	1.864557063	18.59041975	0.349802733	1
PLXNB1	7	2	0.72521789	1.864557063	18.59041975	0.349802733	1
PML	7	2	0.72521789	1.864557063	18.59041975	0.349802733	1
PPP2R2B	7	2	0.72521789	1.864557063	18.59041975	0.349802733	1

PRICKLE1	7	2	0.72521789	1.864557063	18.59041975	0.349802733	1
PRRX1	7	2	0.72521789	1.864557063	18.59041975	0.349802733	1
RASA1	7	2	0.72521789	1.864557063	18.59041975	0.349802733	1
RBMXL2	7	2	0.72521789	1.864557063	18.59041975	0.349802733	1
RGAG4	7	2	0.72521789	1.864557063	18.59041975	0.349802733	1
RRP8	7	2	0.72521789	1.864557063	18.59041975	0.349802733	1
SEMG2	7	2	0.72521789	1.864557063	18.59041975	0.349802733	1
SH3BP5L	7	2	0.72521789	1.864557063	18.59041975	0.349802733	1
SH3PXD2A	7	2	0.72521789	1.864557063	18.59041975	0.349802733	1
SLC17A1	7	2	0.72521789	1.864557063	18.59041975	0.349802733	1
SLC22A9	7	2	0.72521789	1.864557063	18.59041975	0.349802733	1
SLFN13	7	2	0.72521789	1.864557063	18.59041975	0.349802733	1
SOX7	7	2	0.72521789	1.864557063	18.59041975	0.349802733	1
SPPL2A	7	2	0.72521789	1.864557063	18.59041975	0.349802733	1
SYNJ2	7	2	0.72521789	1.864557063	18.59041975	0.349802733	1
TBC1D32	7	2	0.72521789	1.864557063	18.59041975	0.349802733	1
TCTEX1D1	7	2	0.72521789	1.864557063	18.59041975	0.349802733	1
TMTC4	7	2	0.72521789	1.864557063	18.59041975	0.349802733	1
TRBV19	7	2	0.72521789	1.864557063	18.59041975	0.349802733	1
TUB	7	2	0.72521789	1.864557063	18.59041975	0.349802733	1
XRCC5	7	2	0.72521789	1.864557063	18.59041975	0.349802733	1
ZBTB24	7	2	0.72521789	1.864557063	18.59041975	0.349802733	1
ZNF473	7	2	0.72521789	1.864557063	18.59041975	0.349802733	1
ZNF567	7	2	0.72521789	1.864557063	18.59041975	0.349802733	1
ZNF574	7	2	0.72521789	1.864557063	18.59041975	0.349802733	1
ATRNL1	25	15	0.729524673	0.870066079	1.830404013	0.426698937	1
PLXNA4	29	13	0.734829318	1.194861973	2.579200054	0.582188906	1
PPP1R3A	29	13	0.734829318	1.194861973	2.579200054	0.582188906	1
ZP4	28	13	0.734967877	1.149891583	2.490176986	0.557606058	1
TENM2	27	16	0.738112759	0.881167502	1.807325003	0.442372106	1
A1CF	6	4	0.743179098	0.788387413	3.852265482	0.184039016	1
ACTB	6	4	0.743179098	0.788387413	3.852265482	0.184039016	1
ACTN3	6	4	0.743179098	0.788387413	3.852265482	0.184039016	1
ADAM28	6	4	0.743179098	0.788387413	3.852265482	0.184039016	1
ADAMTS13	6	4	0.743179098	0.788387413	3.852265482	0.184039016	1
AHR	6	4	0.743179098	0.788387413	3.852265482	0.184039016	1
AJAP1	6	4	0.743179098	0.788387413	3.852265482	0.184039016	1
AKAP12	6	4	0.743179098	0.788387413	3.852265482	0.184039016	1
ANKRD7	6	4	0.743179098	0.788387413	3.852265482	0.184039016	1
ARHGAP19	6	4	0.743179098	0.788387413	3.852265482	0.184039016	1
ASB2	6	4	0.743179098	0.788387413	3.852265482	0.184039016	1
ASMT	6	4	0.743179098	0.788387413	3.852265482	0.184039016	1
ATAD5	6	4	0.743179098	0.788387413	3.852265482	0.184039016	1
C1orf101	6	4	0.743179098	0.788387413	3.852265482	0.184039016	1
C2CD5	6	4	0.743179098	0.788387413	3.852265482	0.184039016	1
CCDC148	6	4	0.743179098	0.788387413	3.852265482	0.184039016	1
CD1D	6	4	0.743179098	0.788387413	3.852265482	0.184039016	1
CECR2	6	4	0.743179098	0.788387413	3.852265482	0.184039016	1
CEP162	6	4	0.743179098	0.788387413	3.852265482	0.184039016	1
CNTRL	6	4	0.743179098	0.788387413	3.852265482	0.184039016	1
COL17A1	6	4	0.743179098	0.788387413	3.852265482	0.184039016	1
COLGALT2	6	4	0.743179098	0.788387413	3.852265482	0.184039016	1
CPSF1	6	4	0.743179098	0.788387413	3.852265482	0.184039016	1
CRNN	6	4	0.743179098	0.788387413	3.852265482	0.184039016	1
CRX	6	4	0.743179098	0.788387413	3.852265482	0.184039016	1
CUEDC1	6	4	0.743179098	0.788387413	3.852265482	0.184039016	1
CWF19L2	6	4	0.743179098	0.788387413	3.852265482	0.184039016	1
DCAF6	6	4	0.743179098	0.788387413	3.852265482	0.184039016	1

DHTKD1	6	4	0.743179098	0.788387413	3.852265482	0.184039016	1
DMRT3	6	4	0.743179098	0.788387413	3.852265482	0.184039016	1
DMRTB1	6	4	0.743179098	0.788387413	3.852265482	0.184039016	1
DRD1	6	4	0.743179098	0.788387413	3.852265482	0.184039016	1
EOMES	6	4	0.743179098	0.788387413	3.852265482	0.184039016	1
FILIP1	6	4	0.743179098	0.788387413	3.852265482	0.184039016	1
FLCN	6	4	0.743179098	0.788387413	3.852265482	0.184039016	1
FNBP4	6	4	0.743179098	0.788387413	3.852265482	0.184039016	1
FSD1	6	4	0.743179098	0.788387413	3.852265482	0.184039016	1
GAN	6	4	0.743179098	0.788387413	3.852265482	0.184039016	1
GOLGA6B	6	4	0.743179098	0.788387413	3.852265482	0.184039016	1
GRB10	6	4	0.743179098	0.788387413	3.852265482	0.184039016	1
HLX	6	4	0.743179098	0.788387413	3.852265482	0.184039016	1
HOXA5	6	4	0.743179098	0.788387413	3.852265482	0.184039016	1
HTR1F	6	4	0.743179098	0.788387413	3.852265482	0.184039016	1
IFI44	6	4	0.743179098	0.788387413	3.852265482	0.184039016	1
GHV3OR16	6	4	0.743179098	0.788387413	3.852265482	0.184039016	1
IRF4	6	4	0.743179098	0.788387413	3.852265482	0.184039016	1
ITGA2B	6	4	0.743179098	0.788387413	3.852265482	0.184039016	1
KCNQ5	6	4	0.743179098	0.788387413	3.852265482	0.184039016	1
KCTD19	6	4	0.743179098	0.788387413	3.852265482	0.184039016	1
KIAA1958	6	4	0.743179098	0.788387413	3.852265482	0.184039016	1
KIAA2018	6	4	0.743179098	0.788387413	3.852265482	0.184039016	1
KIR3DL2	6	4	0.743179098	0.788387413	3.852265482	0.184039016	1
L3MBTL1	6	4	0.743179098	0.788387413	3.852265482	0.184039016	1
LRRC6	6	4	0.743179098	0.788387413	3.852265482	0.184039016	1
MALT1	6	4	0.743179098	0.788387413	3.852265482	0.184039016	1
MMP10	6	4	0.743179098	0.788387413	3.852265482	0.184039016	1
MOCOS	6	4	0.743179098	0.788387413	3.852265482	0.184039016	1
MTHFD1L	6	4	0.743179098	0.788387413	3.852265482	0.184039016	1
MYBL2	6	4	0.743179098	0.788387413	3.852265482	0.184039016	1
NOS3	6	4	0.743179098	0.788387413	3.852265482	0.184039016	1
NOTCH3	6	4	0.743179098	0.788387413	3.852265482	0.184039016	1
NR0B1	6	4	0.743179098	0.788387413	3.852265482	0.184039016	1
NXF1	6	4	0.743179098	0.788387413	3.852265482	0.184039016	1
OR13C8	6	4	0.743179098	0.788387413	3.852265482	0.184039016	1
OR1S2	6	4	0.743179098	0.788387413	3.852265482	0.184039016	1
OR2V2	6	4	0.743179098	0.788387413	3.852265482	0.184039016	1
OR4B1	6	4	0.743179098	0.788387413	3.852265482	0.184039016	1
OR52N4	6	4	0.743179098	0.788387413	3.852265482	0.184039016	1
OR5K1	6	4	0.743179098	0.788387413	3.852265482	0.184039016	1
OR5M9	6	4	0.743179098	0.788387413	3.852265482	0.184039016	1
OR8D1	6	4	0.743179098	0.788387413	3.852265482	0.184039016	1
OXR1	6	4	0.743179098	0.788387413	3.852265482	0.184039016	1
PADI1	6	4	0.743179098	0.788387413	3.852265482	0.184039016	1
PAPD7	6	4	0.743179098	0.788387413	3.852265482	0.184039016	1
PCDHGC4	6	4	0.743179098	0.788387413	3.852265482	0.184039016	1
PGLYRP2	6	4	0.743179098	0.788387413	3.852265482	0.184039016	1
PLCB2	6	4	0.743179098	0.788387413	3.852265482	0.184039016	1
PROSER1	6	4	0.743179098	0.788387413	3.852265482	0.184039016	1
PTDSS1	6	4	0.743179098	0.788387413	3.852265482	0.184039016	1
PTH2R	6	4	0.743179098	0.788387413	3.852265482	0.184039016	1
RBM12B	6	4	0.743179098	0.788387413	3.852265482	0.184039016	1
RHCG	6	4	0.743179098	0.788387413	3.852265482	0.184039016	1
S1PR1	6	4	0.743179098	0.788387413	3.852265482	0.184039016	1
SATL1	6	4	0.743179098	0.788387413	3.852265482	0.184039016	1
SCUBE1	6	4	0.743179098	0.788387413	3.852265482	0.184039016	1
SERPINA11	6	4	0.743179098	0.788387413	3.852265482	0.184039016	1

SERPINB12	6	4	0.743179098	0.788387413	3.852265482	0.184039016	1
SHROOM4	6	4	0.743179098	0.788387413	3.852265482	0.184039016	1
SLC12A3	6	4	0.743179098	0.788387413	3.852265482	0.184039016	1
SLC12A9	6	4	0.743179098	0.788387413	3.852265482	0.184039016	1
SLC6A13	6	4	0.743179098	0.788387413	3.852265482	0.184039016	1
SLC6A14	6	4	0.743179098	0.788387413	3.852265482	0.184039016	1
SLCO2B1	6	4	0.743179098	0.788387413	3.852265482	0.184039016	1
SLK	6	4	0.743179098	0.788387413	3.852265482	0.184039016	1
SORBS1	6	4	0.743179098	0.788387413	3.852265482	0.184039016	1
SPATA31A1	6	4	0.743179098	0.788387413	3.852265482	0.184039016	1
SRBD1	6	4	0.743179098	0.788387413	3.852265482	0.184039016	1
SRMS	6	4	0.743179098	0.788387413	3.852265482	0.184039016	1
STXBP5	6	4	0.743179098	0.788387413	3.852265482	0.184039016	1
TBC1D5	6	4	0.743179098	0.788387413	3.852265482	0.184039016	1
TBX21	6	4	0.743179098	0.788387413	3.852265482	0.184039016	1
TMC3	6	4	0.743179098	0.788387413	3.852265482	0.184039016	1
TNKS2	6	4	0.743179098	0.788387413	3.852265482	0.184039016	1
TRIM21	6	4	0.743179098	0.788387413	3.852265482	0.184039016	1
UBAP2	6	4	0.743179098	0.788387413	3.852265482	0.184039016	1
UTP14A	6	4	0.743179098	0.788387413	3.852265482	0.184039016	1
WDR35	6	4	0.743179098	0.788387413	3.852265482	0.184039016	1
ZC3HAV1	6	4	0.743179098	0.788387413	3.852265482	0.184039016	1
ZNF347	6	4	0.743179098	0.788387413	3.852265482	0.184039016	1
ZNF543	6	4	0.743179098	0.788387413	3.852265482	0.184039016	1
ZNF607	6	4	0.743179098	0.788387413	3.852265482	0.184039016	1
ZNF622	6	4	0.743179098	0.788387413	3.852265482	0.184039016	1
ZNF75D	6	4	0.743179098	0.788387413	3.852265482	0.184039016	1
ZSWIM3	6	4	0.743179098	0.788387413	3.852265482	0.184039016	1
LTBP1	29	17	0.745863506	0.891001114	1.786603737	0.456792482	1
KRAS	89	44	0.748967224	1.093671824	1.711909127	0.704784631	1
ANKAR	8	3	0.755300832	1.417309545	8.403041311	0.334546604	1
ARHGAP15	8	3	0.755300832	1.417309545	8.403041311	0.334546604	1
ASXL1	8	3	0.755300832	1.417309545	8.403041311	0.334546604	1
BIRC8	8	3	0.755300832	1.417309545	8.403041311	0.334546604	1
CASP2	8	3	0.755300832	1.417309545	8.403041311	0.334546604	1
CASP8AP2	8	3	0.755300832	1.417309545	8.403041311	0.334546604	1
CHRNB4	8	3	0.755300832	1.417309545	8.403041311	0.334546604	1
CUL9	8	3	0.755300832	1.417309545	8.403041311	0.334546604	1
CYBB	8	3	0.755300832	1.417309545	8.403041311	0.334546604	1
EHD3	8	3	0.755300832	1.417309545	8.403041311	0.334546604	1
ELAVL2	8	3	0.755300832	1.417309545	8.403041311	0.334546604	1
FAM13C	8	3	0.755300832	1.417309545	8.403041311	0.334546604	1
FOXD4L5	8	3	0.755300832	1.417309545	8.403041311	0.334546604	1
GABRR1	8	3	0.755300832	1.417309545	8.403041311	0.334546604	1
GLYAT	8	3	0.755300832	1.417309545	8.403041311	0.334546604	1
HADHB	8	3	0.755300832	1.417309545	8.403041311	0.334546604	1
HS3ST4	8	3	0.755300832	1.417309545	8.403041311	0.334546604	1
ISL1	8	3	0.755300832	1.417309545	8.403041311	0.334546604	1
KIAA1024	8	3	0.755300832	1.417309545	8.403041311	0.334546604	1
LPPR5	8	3	0.755300832	1.417309545	8.403041311	0.334546604	1
LRRC37B	8	3	0.755300832	1.417309545	8.403041311	0.334546604	1
MRE11A	8	3	0.755300832	1.417309545	8.403041311	0.334546604	1
MRGPRX4	8	3	0.755300832	1.417309545	8.403041311	0.334546604	1
OR10A4	8	3	0.755300832	1.417309545	8.403041311	0.334546604	1
OR10G2	8	3	0.755300832	1.417309545	8.403041311	0.334546604	1
PBRM1	8	3	0.755300832	1.417309545	8.403041311	0.334546604	1
PHF8	8	3	0.755300832	1.417309545	8.403041311	0.334546604	1
PHLPP2	8	3	0.755300832	1.417309545	8.403041311	0.334546604	1

RDH8	8	3	0.755300832	1.417309545	8.403041311	0.334546604	1
RGS1	8	3	0.755300832	1.417309545	8.403041311	0.334546604	1
RSPO1	8	3	0.755300832	1.417309545	8.403041311	0.334546604	1
SBNO1	8	3	0.755300832	1.417309545	8.403041311	0.334546604	1
SCYL2	8	3	0.755300832	1.417309545	8.403041311	0.334546604	1
SERPINB11	8	3	0.755300832	1.417309545	8.403041311	0.334546604	1
SLC25A13	8	3	0.755300832	1.417309545	8.403041311	0.334546604	1
SLC6A17	8	3	0.755300832	1.417309545	8.403041311	0.334546604	1
SMARCAD	8	3	0.755300832	1.417309545	8.403041311	0.334546604	1
SPATA17	8	3	0.755300832	1.417309545	8.403041311	0.334546604	1
SPPL2C	8	3	0.755300832	1.417309545	8.403041311	0.334546604	1
TSHZ1	8	3	0.755300832	1.417309545	8.403041311	0.334546604	1
UGT2B17	8	3	0.755300832	1.417309545	8.403041311	0.334546604	1
WAC	8	3	0.755300832	1.417309545	8.403041311	0.334546604	1
WWP1	8	3	0.755300832	1.417309545	8.403041311	0.334546604	1
YEATS2	8	3	0.755300832	1.417309545	8.403041311	0.334546604	1
ZNF484	8	3	0.755300832	1.417309545	8.403041311	0.334546604	1
ZNF831	32	19	0.75597742	0.877193824	1.696788535	0.464313952	1
SLITRK2	33	19	0.759329075	0.907668516	1.750500912	0.482367259	1
SPATA31D	33	19	0.759329075	0.907668516	1.750500912	0.482367259	1
ADAM7	7	5	0.759625037	0.733842795	2.97955242	0.197015677	1
ADARB1	7	5	0.759625037	0.733842795	2.97955242	0.197015677	1
AKAP11	7	5	0.759625037	0.733842795	2.97955242	0.197015677	1
ALPK3	7	5	0.759625037	0.733842795	2.97955242	0.197015677	1
ANKRD50	7	5	0.759625037	0.733842795	2.97955242	0.197015677	1
ARHGAP29	7	5	0.759625037	0.733842795	2.97955242	0.197015677	1
ARID4A	7	5	0.759625037	0.733842795	2.97955242	0.197015677	1
BMP7	7	5	0.759625037	0.733842795	2.97955242	0.197015677	1
BRSK1	7	5	0.759625037	0.733842795	2.97955242	0.197015677	1
BUB1	7	5	0.759625037	0.733842795	2.97955242	0.197015677	1
CERKL	7	5	0.759625037	0.733842795	2.97955242	0.197015677	1
CHRND	7	5	0.759625037	0.733842795	2.97955242	0.197015677	1
CNKSR1	7	5	0.759625037	0.733842795	2.97955242	0.197015677	1
CNST	7	5	0.759625037	0.733842795	2.97955242	0.197015677	1
COL9A3	7	5	0.759625037	0.733842795	2.97955242	0.197015677	1
CTSG	7	5	0.759625037	0.733842795	2.97955242	0.197015677	1
DSTYK	7	5	0.759625037	0.733842795	2.97955242	0.197015677	1
DUOX2	7	5	0.759625037	0.733842795	2.97955242	0.197015677	1
EHBP1	7	5	0.759625037	0.733842795	2.97955242	0.197015677	1
EIF4G3	7	5	0.759625037	0.733842795	2.97955242	0.197015677	1
ERICH6	7	5	0.759625037	0.733842795	2.97955242	0.197015677	1
ERN2	7	5	0.759625037	0.733842795	2.97955242	0.197015677	1
EVPL	7	5	0.759625037	0.733842795	2.97955242	0.197015677	1
FAM188B	7	5	0.759625037	0.733842795	2.97955242	0.197015677	1
FRMPD3	7	5	0.759625037	0.733842795	2.97955242	0.197015677	1
FUT9	7	5	0.759625037	0.733842795	2.97955242	0.197015677	1
GAL3ST4	7	5	0.759625037	0.733842795	2.97955242	0.197015677	1
GIMAP6	7	5	0.759625037	0.733842795	2.97955242	0.197015677	1
JAKMIP1	7	5	0.759625037	0.733842795	2.97955242	0.197015677	1
KCNG1	7	5	0.759625037	0.733842795	2.97955242	0.197015677	1
KIF14	7	5	0.759625037	0.733842795	2.97955242	0.197015677	1
KLHL34	7	5	0.759625037	0.733842795	2.97955242	0.197015677	1
KRT34	7	5	0.759625037	0.733842795	2.97955242	0.197015677	1
KRTAP5-1(	7	5	0.759625037	0.733842795	2.97955242	0.197015677	1
LSAMP	7	5	0.759625037	0.733842795	2.97955242	0.197015677	1
MAGEA4	7	5	0.759625037	0.733842795	2.97955242	0.197015677	1
MCM4	7	5	0.759625037	0.733842795	2.97955242	0.197015677	1
MPPED1	7	5	0.759625037	0.733842795	2.97955242	0.197015677	1

MS4A4A	7	5	0.759625037	0.733842795	2.97955242	0.197015677	1
NEO1	7	5	0.759625037	0.733842795	2.97955242	0.197015677	1
NEU4	7	5	0.759625037	0.733842795	2.97955242	0.197015677	1
NFAT5	7	5	0.759625037	0.733842795	2.97955242	0.197015677	1
NOS1AP	7	5	0.759625037	0.733842795	2.97955242	0.197015677	1
NXPE1	7	5	0.759625037	0.733842795	2.97955242	0.197015677	1
OR14I1	7	5	0.759625037	0.733842795	2.97955242	0.197015677	1
OR4K15	7	5	0.759625037	0.733842795	2.97955242	0.197015677	1
OR52K1	7	5	0.759625037	0.733842795	2.97955242	0.197015677	1
PADI3	7	5	0.759625037	0.733842795	2.97955242	0.197015677	1
PKP1	7	5	0.759625037	0.733842795	2.97955242	0.197015677	1
PLXNA1	7	5	0.759625037	0.733842795	2.97955242	0.197015677	1
POLR2A	7	5	0.759625037	0.733842795	2.97955242	0.197015677	1
PRSS38	7	5	0.759625037	0.733842795	2.97955242	0.197015677	1
PTPRJ	7	5	0.759625037	0.733842795	2.97955242	0.197015677	1
RAPGEF4	7	5	0.759625037	0.733842795	2.97955242	0.197015677	1
RPS6KA6	7	5	0.759625037	0.733842795	2.97955242	0.197015677	1
SEC16B	7	5	0.759625037	0.733842795	2.97955242	0.197015677	1
SEMA6B	7	5	0.759625037	0.733842795	2.97955242	0.197015677	1
SHROOM2	7	5	0.759625037	0.733842795	2.97955242	0.197015677	1
SLC18A1	7	5	0.759625037	0.733842795	2.97955242	0.197015677	1
SLC5A4	7	5	0.759625037	0.733842795	2.97955242	0.197015677	1
SLC5A8	7	5	0.759625037	0.733842795	2.97955242	0.197015677	1
SOWAHB	7	5	0.759625037	0.733842795	2.97955242	0.197015677	1
STARD8	7	5	0.759625037	0.733842795	2.97955242	0.197015677	1
SUCO	7	5	0.759625037	0.733842795	2.97955242	0.197015677	1
TAF4	7	5	0.759625037	0.733842795	2.97955242	0.197015677	1
TECPR1	7	5	0.759625037	0.733842795	2.97955242	0.197015677	1
THEMIS	7	5	0.759625037	0.733842795	2.97955242	0.197015677	1
TMPRSS11I	7	5	0.759625037	0.733842795	2.97955242	0.197015677	1
TMTC2	7	5	0.759625037	0.733842795	2.97955242	0.197015677	1
TOPORS	7	5	0.759625037	0.733842795	2.97955242	0.197015677	1
TULP4	7	5	0.759625037	0.733842795	2.97955242	0.197015677	1
UACA	7	5	0.759625037	0.733842795	2.97955242	0.197015677	1
WAPAL	7	5	0.759625037	0.733842795	2.97955242	0.197015677	1
ZFP57	7	5	0.759625037	0.733842795	2.97955242	0.197015677	1
ZNF285	7	5	0.759625037	0.733842795	2.97955242	0.197015677	1
ZNF667	7	5	0.759625037	0.733842795	2.97955242	0.197015677	1
ZPBP	7	5	0.759625037	0.733842795	2.97955242	0.197015677	1
ZSCAN18	7	5	0.759625037	0.733842795	2.97955242	0.197015677	1
ZSCAN5B	7	5	0.759625037	0.733842795	2.97955242	0.197015677	1
DST	36	17	0.760950862	1.132611868	2.225298199	0.596848962	1
ARAP3	8	5	0.772867917	0.841077773	3.321727448	0.238165323	1
ARFGEF2	8	5	0.772867917	0.841077773	3.321727448	0.238165323	1
ASAH2	8	5	0.772867917	0.841077773	3.321727448	0.238165323	1
ATP8B2	8	5	0.772867917	0.841077773	3.321727448	0.238165323	1
ATP9A	8	5	0.772867917	0.841077773	3.321727448	0.238165323	1
BCLAF1	8	5	0.772867917	0.841077773	3.321727448	0.238165323	1
C3orf20	8	5	0.772867917	0.841077773	3.321727448	0.238165323	1
CCDC132	8	5	0.772867917	0.841077773	3.321727448	0.238165323	1
CCDC171	8	5	0.772867917	0.841077773	3.321727448	0.238165323	1
CCDC87	8	5	0.772867917	0.841077773	3.321727448	0.238165323	1
CEP126	8	5	0.772867917	0.841077773	3.321727448	0.238165323	1
CMKLR1	8	5	0.772867917	0.841077773	3.321727448	0.238165323	1
CNGA4	8	5	0.772867917	0.841077773	3.321727448	0.238165323	1
CXorf66	8	5	0.772867917	0.841077773	3.321727448	0.238165323	1
CYYR1	8	5	0.772867917	0.841077773	3.321727448	0.238165323	1
DCTN1	8	5	0.772867917	0.841077773	3.321727448	0.238165323	1

DDX60	8	5	0.772867917	0.841077773	3.321727448	0.238165323	1
EFCAB5	8	5	0.772867917	0.841077773	3.321727448	0.238165323	1
FAP	8	5	0.772867917	0.841077773	3.321727448	0.238165323	1
FHDC1	8	5	0.772867917	0.841077773	3.321727448	0.238165323	1
FIGN	8	5	0.772867917	0.841077773	3.321727448	0.238165323	1
FLRT2	8	5	0.772867917	0.841077773	3.321727448	0.238165323	1
GPC4	8	5	0.772867917	0.841077773	3.321727448	0.238165323	1
INTS1	8	5	0.772867917	0.841077773	3.321727448	0.238165323	1
KIR2DL1	8	5	0.772867917	0.841077773	3.321727448	0.238165323	1
KMT2B	8	5	0.772867917	0.841077773	3.321727448	0.238165323	1
LIPI	8	5	0.772867917	0.841077773	3.321727448	0.238165323	1
1-Mar	8	5	0.772867917	0.841077773	3.321727448	0.238165323	1
NBPF12	8	5	0.772867917	0.841077773	3.321727448	0.238165323	1
NOX4	8	5	0.772867917	0.841077773	3.321727448	0.238165323	1
NRDE2	8	5	0.772867917	0.841077773	3.321727448	0.238165323	1
OR52N2	8	5	0.772867917	0.841077773	3.321727448	0.238165323	1
OR5AR1	8	5	0.772867917	0.841077773	3.321727448	0.238165323	1
PJA1	8	5	0.772867917	0.841077773	3.321727448	0.238165323	1
POT1	8	5	0.772867917	0.841077773	3.321727448	0.238165323	1
PRRC2A	8	5	0.772867917	0.841077773	3.321727448	0.238165323	1
PTCHD1	8	5	0.772867917	0.841077773	3.321727448	0.238165323	1
PUM2	8	5	0.772867917	0.841077773	3.321727448	0.238165323	1
REM1	8	5	0.772867917	0.841077773	3.321727448	0.238165323	1
RFC1	8	5	0.772867917	0.841077773	3.321727448	0.238165323	1
RNF20	8	5	0.772867917	0.841077773	3.321727448	0.238165323	1
RXRG	8	5	0.772867917	0.841077773	3.321727448	0.238165323	1
SCAF11	8	5	0.772867917	0.841077773	3.321727448	0.238165323	1
SENP7	8	5	0.772867917	0.841077773	3.321727448	0.238165323	1
SERPINA7	8	5	0.772867917	0.841077773	3.321727448	0.238165323	1
SLC17A8	8	5	0.772867917	0.841077773	3.321727448	0.238165323	1
SLC39A6	8	5	0.772867917	0.841077773	3.321727448	0.238165323	1
SMC2	8	5	0.772867917	0.841077773	3.321727448	0.238165323	1
SNRPN	8	5	0.772867917	0.841077773	3.321727448	0.238165323	1
T6GALNA(	8	5	0.772867917	0.841077773	3.321727448	0.238165323	1
TEX11	8	5	0.772867917	0.841077773	3.321727448	0.238165323	1
TPH2	8	5	0.772867917	0.841077773	3.321727448	0.238165323	1
UGGT2	8	5	0.772867917	0.841077773	3.321727448	0.238165323	1
USH1C	8	5	0.772867917	0.841077773	3.321727448	0.238165323	1
USP7	8	5	0.772867917	0.841077773	3.321727448	0.238165323	1
ZNF226	8	5	0.772867917	0.841077773	3.321727448	0.238165323	1
ZNF248	8	5	0.772867917	0.841077773	3.321727448	0.238165323	1
ZNF280A	8	5	0.772867917	0.841077773	3.321727448	0.238165323	1
ZNF382	8	5	0.772867917	0.841077773	3.321727448	0.238165323	1
ZNF556	8	5	0.772867917	0.841077773	3.321727448	0.238165323	1
BTK	10	4	0.779601269	1.32939256	5.895774709	0.376231966	1
C11orf63	10	4	0.779601269	1.32939256	5.895774709	0.376231966	1
CCDC136	10	4	0.779601269	1.32939256	5.895774709	0.376231966	1
EFHB	10	4	0.779601269	1.32939256	5.895774709	0.376231966	1
KRT37	10	4	0.779601269	1.32939256	5.895774709	0.376231966	1
LUZP2	10	4	0.779601269	1.32939256	5.895774709	0.376231966	1
OR2F2	10	4	0.779601269	1.32939256	5.895774709	0.376231966	1
OR51S1	10	4	0.779601269	1.32939256	5.895774709	0.376231966	1
PEAR1	10	4	0.779601269	1.32939256	5.895774709	0.376231966	1
PHACTR3	10	4	0.779601269	1.32939256	5.895774709	0.376231966	1
PHEX	10	4	0.779601269	1.32939256	5.895774709	0.376231966	1
PSG6	10	4	0.779601269	1.32939256	5.895774709	0.376231966	1
SIPA1L3	10	4	0.779601269	1.32939256	5.895774709	0.376231966	1
SLC28A3	10	4	0.779601269	1.32939256	5.895774709	0.376231966	1

SLC30A10	10	4	0.779601269	1.32939256	5.895774709	0.376231966	1
SV2C	10	4	0.779601269	1.32939256	5.895774709	0.376231966	1
TFAP2B	10	4	0.779601269	1.32939256	5.895774709	0.376231966	1
TIAM2	10	4	0.779601269	1.32939256	5.895774709	0.376231966	1
[MPRSS11I	10	4	0.779601269	1.32939256	5.895774709	0.376231966	1
TRAPPC8	10	4	0.779601269	1.32939256	5.895774709	0.376231966	1
ADAM33	9	6	0.783520493	0.786358767	2.733594015	0.245053856	1
ADAMTS7	9	6	0.783520493	0.786358767	2.733594015	0.245053856	1
C2orf71	9	6	0.783520493	0.786358767	2.733594015	0.245053856	1
CDK12	9	6	0.783520493	0.786358767	2.733594015	0.245053856	1
DOCK7	9	6	0.783520493	0.786358767	2.733594015	0.245053856	1
EMILIN2	9	6	0.783520493	0.786358767	2.733594015	0.245053856	1
FMO1	9	6	0.783520493	0.786358767	2.733594015	0.245053856	1
FMO2	9	6	0.783520493	0.786358767	2.733594015	0.245053856	1
GALNTL6	9	6	0.783520493	0.786358767	2.733594015	0.245053856	1
GBP5	9	6	0.783520493	0.786358767	2.733594015	0.245053856	1
HCLS1	9	6	0.783520493	0.786358767	2.733594015	0.245053856	1
IRX1	9	6	0.783520493	0.786358767	2.733594015	0.245053856	1
KCNJ2	9	6	0.783520493	0.786358767	2.733594015	0.245053856	1
KCNS2	9	6	0.783520493	0.786358767	2.733594015	0.245053856	1
KLB	9	6	0.783520493	0.786358767	2.733594015	0.245053856	1
KLHL38	9	6	0.783520493	0.786358767	2.733594015	0.245053856	1
NPC1L1	9	6	0.783520493	0.786358767	2.733594015	0.245053856	1
OBSL1	9	6	0.783520493	0.786358767	2.733594015	0.245053856	1
OPHN1	9	6	0.783520493	0.786358767	2.733594015	0.245053856	1
PDE11A	9	6	0.783520493	0.786358767	2.733594015	0.245053856	1
PKP4	9	6	0.783520493	0.786358767	2.733594015	0.245053856	1
PSG2	9	6	0.783520493	0.786358767	2.733594015	0.245053856	1
PTPN14	9	6	0.783520493	0.786358767	2.733594015	0.245053856	1
R3HCC1L	9	6	0.783520493	0.786358767	2.733594015	0.245053856	1
RGS12	9	6	0.783520493	0.786358767	2.733594015	0.245053856	1
SLC10A2	9	6	0.783520493	0.786358767	2.733594015	0.245053856	1
SLC1A6	9	6	0.783520493	0.786358767	2.733594015	0.245053856	1
SLC4A8	9	6	0.783520493	0.786358767	2.733594015	0.245053856	1
SLCO1C1	9	6	0.783520493	0.786358767	2.733594015	0.245053856	1
TTI1	9	6	0.783520493	0.786358767	2.733594015	0.245053856	1
XKR7	9	6	0.783520493	0.786358767	2.733594015	0.245053856	1
ZNF135	9	6	0.783520493	0.786358767	2.733594015	0.245053856	1
ZNF578	9	6	0.783520493	0.786358767	2.733594015	0.245053856	1
ZNF592	9	6	0.783520493	0.786358767	2.733594015	0.245053856	1
ZNF91	9	6	0.783520493	0.786358767	2.733594015	0.245053856	1
RYR1	48	27	0.792756322	0.927961913	1.616310798	0.541366052	1
ABCA7	10	6	0.794041224	0.876313651	2.987949325	0.282690478	1
ADGRA3	10	6	0.794041224	0.876313651	2.987949325	0.282690478	1
AMOT	10	6	0.794041224	0.876313651	2.987949325	0.282690478	1
AMY2B	10	6	0.794041224	0.876313651	2.987949325	0.282690478	1
ANKRD12	10	6	0.794041224	0.876313651	2.987949325	0.282690478	1
ANKRD26	10	6	0.794041224	0.876313651	2.987949325	0.282690478	1
ASB5	10	6	0.794041224	0.876313651	2.987949325	0.282690478	1
ATP1A2	10	6	0.794041224	0.876313651	2.987949325	0.282690478	1
C8A	10	6	0.794041224	0.876313651	2.987949325	0.282690478	1
CACNA2D3	10	6	0.794041224	0.876313651	2.987949325	0.282690478	1
CEP164	10	6	0.794041224	0.876313651	2.987949325	0.282690478	1
COL11A2	10	6	0.794041224	0.876313651	2.987949325	0.282690478	1
CPXCR1	10	6	0.794041224	0.876313651	2.987949325	0.282690478	1
DIAPH3	10	6	0.794041224	0.876313651	2.987949325	0.282690478	1
DOCK9	10	6	0.794041224	0.876313651	2.987949325	0.282690478	1
DSC2	10	6	0.794041224	0.876313651	2.987949325	0.282690478	1

FAM135A	10	6	0.794041224	0.876313651	2.987949325	0.282690478	1
FOXG1	10	6	0.794041224	0.876313651	2.987949325	0.282690478	1
GPR174	10	6	0.794041224	0.876313651	2.987949325	0.282690478	1
INTU	10	6	0.794041224	0.876313651	2.987949325	0.282690478	1
MTMR8	10	6	0.794041224	0.876313651	2.987949325	0.282690478	1
NINL	10	6	0.794041224	0.876313651	2.987949325	0.282690478	1
OGT	10	6	0.794041224	0.876313651	2.987949325	0.282690478	1
OR14C36	10	6	0.794041224	0.876313651	2.987949325	0.282690478	1
PALLD	10	6	0.794041224	0.876313651	2.987949325	0.282690478	1
PGR	10	6	0.794041224	0.876313651	2.987949325	0.282690478	1
PMS1	10	6	0.794041224	0.876313651	2.987949325	0.282690478	1
PTCH1	10	6	0.794041224	0.876313651	2.987949325	0.282690478	1
SCAF8	10	6	0.794041224	0.876313651	2.987949325	0.282690478	1
SV2A	10	6	0.794041224	0.876313651	2.987949325	0.282690478	1
UGT2A3	10	6	0.794041224	0.876313651	2.987949325	0.282690478	1
UGT2B4	10	6	0.794041224	0.876313651	2.987949325	0.282690478	1
VGLL3	10	6	0.794041224	0.876313651	2.987949325	0.282690478	1
WDR64	10	6	0.794041224	0.876313651	2.987949325	0.282690478	1
ZNF800	10	6	0.794041224	0.876313651	2.987949325	0.282690478	1
CAMSAP2	12	5	0.797719059	1.276834119	4.707168399	0.41000401	1
HELZ	12	5	0.797719059	1.276834119	4.707168399	0.41000401	1
NMUR2	12	5	0.797719059	1.276834119	4.707168399	0.41000401	1
NPFFR2	12	5	0.797719059	1.276834119	4.707168399	0.41000401	1
OR51T1	12	5	0.797719059	1.276834119	4.707168399	0.41000401	1
OR6K3	12	5	0.797719059	1.276834119	4.707168399	0.41000401	1
PRAMEF15	12	5	0.797719059	1.276834119	4.707168399	0.41000401	1
AHCTF1	11	7	0.801338824	0.823956415	2.556935321	0.285371241	1
ANO7	11	7	0.801338824	0.823956415	2.556935321	0.285371241	1
ARHGAP21	11	7	0.801338824	0.823956415	2.556935321	0.285371241	1
CTNNB1	11	7	0.801338824	0.823956415	2.556935321	0.285371241	1
DHX9	11	7	0.801338824	0.823956415	2.556935321	0.285371241	1
DRD5	11	7	0.801338824	0.823956415	2.556935321	0.285371241	1
GABRA3	11	7	0.801338824	0.823956415	2.556935321	0.285371241	1
GABRQ	11	7	0.801338824	0.823956415	2.556935321	0.285371241	1
GDF10	11	7	0.801338824	0.823956415	2.556935321	0.285371241	1
GHR	11	7	0.801338824	0.823956415	2.556935321	0.285371241	1
GRM6	11	7	0.801338824	0.823956415	2.556935321	0.285371241	1
MAGEC3	11	7	0.801338824	0.823956415	2.556935321	0.285371241	1
MAP1A	11	7	0.801338824	0.823956415	2.556935321	0.285371241	1
NPY5R	11	7	0.801338824	0.823956415	2.556935321	0.285371241	1
NTRK2	11	7	0.801338824	0.823956415	2.556935321	0.285371241	1
PRDM14	11	7	0.801338824	0.823956415	2.556935321	0.285371241	1
RICTOR	11	7	0.801338824	0.823956415	2.556935321	0.285371241	1
SLC4A1	11	7	0.801338824	0.823956415	2.556935321	0.285371241	1
TRANK1	11	7	0.801338824	0.823956415	2.556935321	0.285371241	1
ZIM3	11	7	0.801338824	0.823956415	2.556935321	0.285371241	1
ZNF385D	11	7	0.801338824	0.823956415	2.556935321	0.285371241	1
ZNF711	11	7	0.801338824	0.823956415	2.556935321	0.285371241	1
ABI3BP	12	7	0.810108028	0.901578381	2.757614428	0.319982296	1
ADGRE1	12	7	0.810108028	0.901578381	2.757614428	0.319982296	1
AGL	12	7	0.810108028	0.901578381	2.757614428	0.319982296	1
DSP	12	7	0.810108028	0.901578381	2.757614428	0.319982296	1
ITGA2	12	7	0.810108028	0.901578381	2.757614428	0.319982296	1
NOBOX	12	7	0.810108028	0.901578381	2.757614428	0.319982296	1
OR10Z1	12	7	0.810108028	0.901578381	2.757614428	0.319982296	1
OR2B11	12	7	0.810108028	0.901578381	2.757614428	0.319982296	1
PCDH20	12	7	0.810108028	0.901578381	2.757614428	0.319982296	1
SHPRH	12	7	0.810108028	0.901578381	2.757614428	0.319982296	1

TTC21B	12	7	0.810108028	0.901578381	2.757614428	0.319982296	1
ARHGEF11	14	6	0.811885936	1.241933994	4.019295513	0.438204163	1
ARPP21	14	6	0.811885936	1.241933994	4.019295513	0.438204163	1
BCL11A	14	6	0.811885936	1.241933994	4.019295513	0.438204163	1
ENOX1	14	6	0.811885936	1.241933994	4.019295513	0.438204163	1
MTOR	14	6	0.811885936	1.241933994	4.019295513	0.438204163	1
RASAL2	14	6	0.811885936	1.241933994	4.019295513	0.438204163	1
SLC14A2	14	6	0.811885936	1.241933994	4.019295513	0.438204163	1
SMAD4	14	6	0.811885936	1.241933994	4.019295513	0.438204163	1
ZSCAN4	14	6	0.811885936	1.241933994	4.019295513	0.438204163	1
ADAMTS14	13	8	0.815273583	0.852233533	2.423439567	0.31970857	1
BCAN	13	8	0.815273583	0.852233533	2.423439567	0.31970857	1
CCT8L2	13	8	0.815273583	0.852233533	2.423439567	0.31970857	1
GOLGB1	13	8	0.815273583	0.852233533	2.423439567	0.31970857	1
HERC1	13	8	0.815273583	0.852233533	2.423439567	0.31970857	1
KCNJ3	13	8	0.815273583	0.852233533	2.423439567	0.31970857	1
LRRC32	13	8	0.815273583	0.852233533	2.423439567	0.31970857	1
MROH9	13	8	0.815273583	0.852233533	2.423439567	0.31970857	1
NRXN2	13	8	0.815273583	0.852233533	2.423439567	0.31970857	1
OR2G3	13	8	0.815273583	0.852233533	2.423439567	0.31970857	1
ZNF98	13	8	0.815273583	0.852233533	2.423439567	0.31970857	1
ADCY1	14	8	0.822829979	0.920613025	2.588440847	0.351799008	1
DOPEY2	14	8	0.822829979	0.920613025	2.588440847	0.351799008	1
FLT1	14	8	0.822829979	0.920613025	2.588440847	0.351799008	1
LRRC66	14	8	0.822829979	0.920613025	2.588440847	0.351799008	1
NOTCH1	14	8	0.822829979	0.920613025	2.588440847	0.351799008	1
OR14A16	14	8	0.822829979	0.920613025	2.588440847	0.351799008	1
PCDHA7	14	8	0.822829979	0.920613025	2.588440847	0.351799008	1
INHBA	16	7	0.823348295	1.217123092	3.572029469	0.46225748	1
NTNG1	16	7	0.823348295	1.217123092	3.572029469	0.46225748	1
OR10J3	16	7	0.823348295	1.217123092	3.572029469	0.46225748	1
PIK3C2B	16	7	0.823348295	1.217123092	3.572029469	0.46225748	1
PZP	16	7	0.823348295	1.217123092	3.572029469	0.46225748	1
RAPGEF2	16	7	0.823348295	1.217123092	3.572029469	0.46225748	1
CACNA1I	15	9	0.826549872	0.874298976	2.31864485	0.34944558	1
LAMA5	15	9	0.826549872	0.874298976	2.31864485	0.34944558	1
NCAN	15	9	0.826549872	0.874298976	2.31864485	0.34944558	1
PCDHB6	15	9	0.826549872	0.874298976	2.31864485	0.34944558	1
AGMO	18	8	0.832863902	1.198620959	3.258136443	0.483100443	1
ARID2	18	8	0.832863902	1.198620959	3.258136443	0.483100443	1
FNDC1	18	8	0.832863902	1.198620959	3.258136443	0.483100443	1
IQGAP2	18	8	0.832863902	1.198620959	3.258136443	0.483100443	1
COL15A1	17	10	0.835912522	0.892017751	2.23400155	0.375445979	1
COL4A6	17	10	0.835912522	0.892017751	2.23400155	0.375445979	1
PTCHD4	17	10	0.835912522	0.892017751	2.23400155	0.375445979	1
SELP	17	10	0.835912522	0.892017751	2.23400155	0.375445979	1
TRPM1	17	10	0.835912522	0.892017751	2.23400155	0.375445979	1
BCHE	20	9	0.840923366	1.184331954	3.025470248	0.501457921	1
PCDHB4	19	9	0.840923366	1.121505627	2.882447191	0.47051599	1
VPS13D	20	9	0.840923366	1.184331954	3.025470248	0.501457921	1
ROR2	19	11	0.843843779	0.906576598	2.164188732	0.398461061	1
MKI67	21	10	0.847592511	1.116050357	2.721964744	0.488804805	1
DLGAP2	22	10	0.847860142	1.17299404	2.84587873	0.517737852	1
VPS13B	22	10	0.847860142	1.17299404	2.84587873	0.517737852	1
COL1A2	21	12	0.850671422	0.91876666	2.105236881	0.418960169	1
DSG3	21	12	0.850671422	0.91876666	2.105236881	0.418960169	1
KCNH5	21	12	0.850671422	0.91876666	2.105236881	0.418960169	1
PCDHGA2	21	12	0.850671422	0.91876666	2.105236881	0.418960169	1

KCNH8	23	11	0.853436766	1.111668419	2.594021986	0.505152232	1
L1CAM	24	11	0.85390982	1.163804123	2.703274704	0.532357871	1
ACTN2	23	13	0.856627033	0.929135666	2.054862311	0.437434094	1
COL4A5	23	13	0.856627033	0.929135666	2.054862311	0.437434094	1
BRAF	24	14	0.859244186	0.897614687	1.933394215	0.431731442	1
POM121L1	24	14	0.859244186	0.897614687	1.933394215	0.431731442	1
HUWE1	25	14	0.861879482	0.93807487	2.011208625	0.454084444	1
RP1	25	14	0.861879482	0.93807487	2.011208625	0.454084444	1
SLITRK4	25	14	0.861879482	0.93807487	2.011208625	0.454084444	1
CNTNAP4	27	13	0.863229761	1.105139399	2.402032503	0.533204348	1
ZNF208	27	13	0.863229761	1.105139399	2.402032503	0.533204348	1
PKD1L1	26	15	0.863991837	0.907841003	1.901601925	0.447905185	1
OTOGL	27	15	0.866555184	0.945871214	1.973248186	0.469323559	1
SPATA31A	29	16	0.870750902	0.952742145	1.939675855	0.483276596	1
NF1	34	19	0.878791986	0.938354295	1.804560485	0.500569761	1
MYH2	36	20	0.881725959	0.944167752	1.785734326	0.51136036	1
CNTNAP2	40	22	0.886934973	0.9543217	1.752843119	0.530869592	1
ASXL3	42	23	0.889260427	0.958795235	1.738377679	0.53970293	1
PXDNL	45	25	0.892157308	0.942353587	1.672439894	0.540630641	1
HD1-EIF4I	12	6	1	1.057948388	3.500713283	0.359546024	1
ARHGAP5	8	4	1	1.057209929	4.869245447	0.278277123	1
ARHGEF40	8	4	1	1.057209929	4.869245447	0.278277123	1
ASIC4	10	5	1	1.057576789	4.010941832	0.322889375	1
ATXN2L	8	4	1	1.057209929	4.869245447	0.278277123	1
AVPR1A	8	4	1	1.057209929	4.869245447	0.278277123	1
BNC2	13	7	1	0.97968724	2.959500974	0.354975521	1
BPI	8	4	1	1.057209929	4.869245447	0.278277123	1
CA10	8	4	1	1.057209929	4.869245447	0.278277123	1
CADPS	13	7	1	0.97968724	2.959500974	0.354975521	1
CASP5	8	4	1	1.057209929	4.869245447	0.278277123	1
CCDC150	8	4	1	1.057209929	4.869245447	0.278277123	1
CCDC39	10	5	1	1.057576789	4.010941832	0.322889375	1
CCKBR	13	7	1	0.97968724	2.959500974	0.354975521	1
CD109	13	7	1	0.97968724	2.959500974	0.354975521	1
CILP2	10	5	1	1.057576789	4.010941832	0.322889375	1
COL6A2	12	6	1	1.057948388	3.500713283	0.359546024	1
CREB5	8	4	1	1.057209929	4.869245447	0.278277123	1
CRIM1	8	4	1	1.057209929	4.869245447	0.278277123	1
CRISP3	8	4	1	1.057209929	4.869245447	0.278277123	1
DGKK	16	8	1	1.058706176	2.921477257	0.416840098	1
DIO2	10	5	1	1.057576789	4.010941832	0.322889375	1
DLGAP1	10	5	1	1.057576789	4.010941832	0.322889375	1
DPYD	12	6	1	1.057948388	3.500713283	0.359546024	1
DRC1	8	4	1	1.057209929	4.869245447	0.278277123	1
DRD3	8	4	1	1.057209929	4.869245447	0.278277123	1
DSG1	13	7	1	0.97968724	2.959500974	0.354975521	1
DVL3	8	4	1	1.057209929	4.869245447	0.278277123	1
EDIL3	13	7	1	0.97968724	2.959500974	0.354975521	1
EGF	8	4	1	1.057209929	4.869245447	0.278277123	1
EPAS1	8	4	1	1.057209929	4.869245447	0.278277123	1
EPHB1	21	11	1	1.008439538	2.377701719	0.45135381	1
ERCC5	8	4	1	1.057209929	4.869245447	0.278277123	1
ESPNL	8	4	1	1.057209929	4.869245447	0.278277123	1
EVC2	13	7	1	0.97968724	2.959500974	0.354975521	1
F13B	13	7	1	0.97968724	2.959500974	0.354975521	1
FAM208B	10	5	1	1.057576789	4.010941832	0.322889375	1
FAM21A	8	4	1	1.057209929	4.869245447	0.278277123	1
FAM21C	8	4	1	1.057209929	4.869245447	0.278277123	1

FBLN1	8	4	1	1.057209929	4.869245447	0.278277123	1
FGD1	8	4	1	1.057209929	4.869245447	0.278277123	1
FIG4	8	4	1	1.057209929	4.869245447	0.278277123	1
FYCO1	8	4	1	1.057209929	4.869245447	0.278277123	1
GAD2	13	7	1	0.97968724	2.959500974	0.354975521	1
GALNT14	12	6	1	1.057948388	3.500713283	0.359546024	1
GPR141	10	5	1	1.057576789	4.010941832	0.322889375	1
GPR45	8	4	1	1.057209929	4.869245447	0.278277123	1
GPRASP1	12	6	1	1.057948388	3.500713283	0.359546024	1
GPRC6A	13	7	1	0.97968724	2.959500974	0.354975521	1
GRAMD1B	8	4	1	1.057209929	4.869245447	0.278277123	1
IFI16	10	5	1	1.057576789	4.010941832	0.322889375	1
ITGA1	8	4	1	1.057209929	4.869245447	0.278277123	1
ITSN1	12	6	1	1.057948388	3.500713283	0.359546024	1
KCNH2	8	4	1	1.057209929	4.869245447	0.278277123	1
KCNT1	10	5	1	1.057576789	4.010941832	0.322889375	1
KIAA0556	12	6	1	1.057948388	3.500713283	0.359546024	1
KIF27	8	4	1	1.057209929	4.869245447	0.278277123	1
KLHL13	8	4	1	1.057209929	4.869245447	0.278277123	1
KMT2D	21	11	1	1.008439538	2.377701719	0.45135381	1
KRT38	8	4	1	1.057209929	4.869245447	0.278277123	1
LILRA2	13	7	1	0.97968724	2.959500974	0.354975521	1
LRPPRC	8	4	1	1.057209929	4.869245447	0.278277123	1
MAGEA12	8	4	1	1.057209929	4.869245447	0.278277123	1
MARK1	13	7	1	0.97968724	2.959500974	0.354975521	1
MASP1	8	4	1	1.057209929	4.869245447	0.278277123	1
MCF2	12	6	1	1.057948388	3.500713283	0.359546024	1
MPEG1	8	4	1	1.057209929	4.869245447	0.278277123	1
MYO18A	10	5	1	1.057576789	4.010941832	0.322889375	1
NPSR1	8	4	1	1.057209929	4.869245447	0.278277123	1
NPY1R	8	4	1	1.057209929	4.869245447	0.278277123	1
NXF3	8	4	1	1.057209929	4.869245447	0.278277123	1
OR10G8	13	7	1	0.97968724	2.959500974	0.354975521	1
OR10W1	10	5	1	1.057576789	4.010941832	0.322889375	1
OR2C3	10	5	1	1.057576789	4.010941832	0.322889375	1
OR2D2	8	4	1	1.057209929	4.869245447	0.278277123	1
OR2H1	8	4	1	1.057209929	4.869245447	0.278277123	1
OR2T10	8	4	1	1.057209929	4.869245447	0.278277123	1
OR4A47	10	5	1	1.057576789	4.010941832	0.322889375	1
OR51A7	10	5	1	1.057576789	4.010941832	0.322889375	1
OR56A1	8	4	1	1.057209929	4.869245447	0.278277123	1
OR56A4	13	7	1	0.97968724	2.959500974	0.354975521	1
OR7D4	8	4	1	1.057209929	4.869245447	0.278277123	1
OR8J1	10	5	1	1.057576789	4.010941832	0.322889375	1
PALD1	13	7	1	0.97968724	2.959500974	0.354975521	1
PARD3	8	4	1	1.057209929	4.869245447	0.278277123	1
PCDHGA12	8	4	1	1.057209929	4.869245447	0.278277123	1
PCDHGA9	12	6	1	1.057948388	3.500713283	0.359546024	1
PDE4B	8	4	1	1.057209929	4.869245447	0.278277123	1
PDZD2	13	7	1	0.97968724	2.959500974	0.354975521	1
PLA2R1	8	4	1	1.057209929	4.869245447	0.278277123	1
POLA1	10	5	1	1.057576789	4.010941832	0.322889375	1
PPP1R16B	12	6	1	1.057948388	3.500713283	0.359546024	1
PPP2R1A	8	4	1	1.057209929	4.869245447	0.278277123	1
PRAMEF19	8	4	1	1.057209929	4.869245447	0.278277123	1
PRKCI	8	4	1	1.057209929	4.869245447	0.278277123	1
PROKR1	8	4	1	1.057209929	4.869245447	0.278277123	1
PSG3	8	4	1	1.057209929	4.869245447	0.278277123	1

RC3H2	10	5	1	1.057576789	4.010941832	0.322889375	1
RGAG1	16	8	1	1.058706176	2.921477257	0.416840098	1
RTP5	10	5	1	1.057576789	4.010941832	0.322889375	1
SEMA3A	16	8	1	1.058706176	2.921477257	0.416840098	1
SERPINB4	12	6	1	1.057948388	3.500713283	0.359546024	1
SIGLEC10	12	6	1	1.057948388	3.500713283	0.359546024	1
SIGLEC9	8	4	1	1.057209929	4.869245447	0.278277123	1
SLC26A3	12	6	1	1.057948388	3.500713283	0.359546024	1
SLC32A1	13	7	1	0.97968724	2.959500974	0.354975521	1
SLC9A6	8	4	1	1.057209929	4.869245447	0.278277123	1
SLCO1B1	10	5	1	1.057576789	4.010941832	0.322889375	1
SPATC1	8	4	1	1.057209929	4.869245447	0.278277123	1
SRRM2	16	8	1	1.058706176	2.921477257	0.416840098	1
SSFA2	10	5	1	1.057576789	4.010941832	0.322889375	1
SYCP1	10	5	1	1.057576789	4.010941832	0.322889375	1
SYT9	12	6	1	1.057948388	3.500713283	0.359546024	1
TANC1	10	5	1	1.057576789	4.010941832	0.322889375	1
TBX20	8	4	1	1.057209929	4.869245447	0.278277123	1
TCHHL1	13	7	1	0.97968724	2.959500974	0.354975521	1
UGT2A1	12	6	1	1.057948388	3.500713283	0.359546024	1
UGT2B15	8	4	1	1.057209929	4.869245447	0.278277123	1
UNC5B	10	5	1	1.057576789	4.010941832	0.322889375	1
USP31	8	4	1	1.057209929	4.869245447	0.278277123	1
VWA3A	8	4	1	1.057209929	4.869245447	0.278277123	1
XKR3	8	4	1	1.057209929	4.869245447	0.278277123	1
ZEB1	21	11	1	1.008439538	2.377701719	0.45135381	1
ZFP42	8	4	1	1.057209929	4.869245447	0.278277123	1
ZNF229	10	5	1	1.057576789	4.010941832	0.322889375	1
ZNF536	64	34	1	0.992262186	1.633802294	0.610087665	1
AACS	7	4	1	0.922377319	4.359730392	0.230573749	1
ABCA4	9	5	1	0.948984229	3.665287923	0.280199847	1
ABCC4	11	5	1	1.166870309	4.357892975	0.366182928	1
ABCG5	11	6	1	0.966810152	3.243613275	0.320874982	1
ABCG8	9	5	1	0.948984229	3.665287923	0.280199847	1
ABL2	9	5	1	0.948984229	3.665287923	0.280199847	1
ACBD5	6	3	1	1.056847718	6.613295974	0.222355569	1
ACOXL	5	2	1	1.32421267	14.04843817	0.214017959	1
ACPT	5	2	1	1.32421267	14.04843817	0.214017959	1
ADAM18	9	4	1	1.19284652	5.381296202	0.326878945	1
ADAMTS1(	5	2	1	1.32421267	14.04843817	0.214017959	1
ADAMTS1:	9	5	1	0.948984229	3.665287923	0.280199847	1
ADAMTS1ℓ	18	9	1	1.059092557	2.740321176	0.439843457	1
ADAMTS3	13	6	1	1.149648897	3.759608513	0.398659974	1
ADAMTS9	15	7	1	1.137466096	3.36666144	0.426154782	1
ADAMTSL:	7	3	1	1.236475779	7.503909445	0.277828088	1
ADGRD1	9	4	1	1.19284652	5.381296202	0.326878945	1
ADGRE2	9	5	1	0.948984229	3.665287923	0.280199847	1
ADGRE3	7	3	1	1.236475779	7.503909445	0.277828088	1
ADGRG2	9	4	1	1.19284652	5.381296202	0.326878945	1
ADGRL1	5	2	1	1.32421267	14.04843817	0.214017959	1
ADH7	6	3	1	1.056847718	6.613295974	0.222355569	1
ADORA1	5	2	1	1.32421267	14.04843817	0.214017959	1
ADRA1B	5	2	1	1.32421267	14.04843817	0.214017959	1
AEBP1	15	7	1	1.137466096	3.36666144	0.426154782	1
AFF2	35	18	1	1.029750795	1.999957428	0.546276181	1
AFF3	6	3	1	1.056847718	6.613295974	0.222355569	1
AGPAT4	5	2	1	1.32421267	14.04843817	0.214017959	1
AIM1	7	3	1	1.236475779	7.503909445	0.277828088	1

AKR1D1	6	3	1	1.056847718	6.613295974	0.222355569	1
ALDH1A2	5	2	1	1.32421267	14.04843817	0.214017959	1
ALDOB	5	2	1	1.32421267	14.04843817	0.214017959	1
ALG10	9	4	1	1.19284652	5.381296202	0.326878945	1
ALOX5	7	4	1	0.922377319	4.359730392	0.230573749	1
ALX1	5	2	1	1.32421267	14.04843817	0.214017959	1
AMBRA1	6	3	1	1.056847718	6.613295974	0.222355569	1
AMELX	5	2	1	1.32421267	14.04843817	0.214017959	1
AMOTL1	6	3	1	1.056847718	6.613295974	0.222355569	1
AMPD1	11	5	1	1.166870309	4.357892975	0.366182928	1
ANGPT1	7	4	1	0.922377319	4.359730392	0.230573749	1
ANKRD34A	6	3	1	1.056847718	6.613295974	0.222355569	1
ANKRD35	6	3	1	1.056847718	6.613295974	0.222355569	1
ANKRD36	5	2	1	1.32421267	14.04843817	0.214017959	1
ANKS1B	15	8	1	0.989431896	2.754371144	0.384167072	1
ANO6	7	4	1	0.922377319	4.359730392	0.230573749	1
AOAH	6	3	1	1.056847718	6.613295974	0.222355569	1
AOC1	9	4	1	1.19284652	5.381296202	0.326878945	1
AP2A2	5	2	1	1.32421267	14.04843817	0.214017959	1
AP3B2	9	5	1	0.948984229	3.665287923	0.280199847	1
APPBP2	5	2	1	1.32421267	14.04843817	0.214017959	1
ARFGAP2	5	2	1	1.32421267	14.04843817	0.214017959	1
ARFGEF3	11	6	1	0.966810152	3.243613275	0.320874982	1
ARHGAP12	6	3	1	1.056847718	6.613295974	0.222355569	1
ARHGAP17	5	2	1	1.32421267	14.04843817	0.214017959	1
ARHGAP25	9	5	1	0.948984229	3.665287923	0.280199847	1
ARHGAP31	11	5	1	1.166870309	4.357892975	0.366182928	1
ARHGEF10	6	3	1	1.056847718	6.613295974	0.222355569	1
ARHGEF10L	6	3	1	1.056847718	6.613295974	0.222355569	1
ARHGEF15	5	2	1	1.32421267	14.04843817	0.214017959	1
ARHGEF3	5	2	1	1.32421267	14.04843817	0.214017959	1
ARMCX2	9	5	1	0.948984229	3.665287923	0.280199847	1
ASB15	7	3	1	1.236475779	7.503909445	0.277828088	1
ASCC3	13	6	1	1.149648897	3.759608513	0.398659974	1
ATAD2	7	3	1	1.236475779	7.503909445	0.277828088	1
ATG16L1	5	2	1	1.32421267	14.04843817	0.214017959	1
ATP11B	6	3	1	1.056847718	6.613295974	0.222355569	1
ATP2B2	15	8	1	0.989431896	2.754371144	0.384167072	1
ATP6V1C1	5	2	1	1.32421267	14.04843817	0.214017959	1
ATP8B4	14	7	1	1.058324818	3.162286269	0.390371972	1
ATRN	6	3	1	1.056847718	6.613295974	0.222355569	1
B3GAT2	6	3	1	1.056847718	6.613295974	0.222355569	1
BAAT	5	2	1	1.32421267	14.04843817	0.214017959	1
BAP1	5	2	1	1.32421267	14.04843817	0.214017959	1
BARD1	7	3	1	1.236475779	7.503909445	0.277828088	1
BCAS1	7	3	1	1.236475779	7.503909445	0.277828088	1
BCO2	6	3	1	1.056847718	6.613295974	0.222355569	1
BCOR	15	7	1	1.137466096	3.36666144	0.426154782	1
BMP1	7	3	1	1.236475779	7.503909445	0.277828088	1
BMP10	7	3	1	1.236475779	7.503909445	0.277828088	1
BMP6	6	3	1	1.056847718	6.613295974	0.222355569	1
BTNL9	5	2	1	1.32421267	14.04843817	0.214017959	1
C12orf42	5	2	1	1.32421267	14.04843817	0.214017959	1
C12orf66	6	3	1	1.056847718	6.613295974	0.222355569	1
C17orf47	5	2	1	1.32421267	14.04843817	0.214017959	1
C1orf127	6	3	1	1.056847718	6.613295974	0.222355569	1
C6orf222	7	4	1	0.922377319	4.359730392	0.230573749	1
CACNA1D	9	5	1	0.948984229	3.665287923	0.280199847	1

CAMSAP1	7	4	1	0.922377319	4.359730392	0.230573749	1
CAPN5	5	2	1	1.32421267	14.04843817	0.214017959	1
CASD1	5	2	1	1.32421267	14.04843817	0.214017959	1
CASKIN1	7	3	1	1.236475779	7.503909445	0.277828088	1
CCDC116	6	3	1	1.056847718	6.613295974	0.222355569	1
CCDC120	5	2	1	1.32421267	14.04843817	0.214017959	1
CCDC146	5	2	1	1.32421267	14.04843817	0.214017959	1
CCDC180	6	3	1	1.056847718	6.613295974	0.222355569	1
CCDC83	5	2	1	1.32421267	14.04843817	0.214017959	1
CCDC88C	6	3	1	1.056847718	6.613295974	0.222355569	1
CCDC91	5	2	1	1.32421267	14.04843817	0.214017959	1
CCER1	11	6	1	0.966810152	3.243613275	0.320874982	1
CD200R1	7	3	1	1.236475779	7.503909445	0.277828088	1
CD244	7	3	1	1.236475779	7.503909445	0.277828088	1
CDC20B	6	3	1	1.056847718	6.613295974	0.222355569	1
CDC42BPC	6	3	1	1.056847718	6.613295974	0.222355569	1
CDC73	5	2	1	1.32421267	14.04843817	0.214017959	1
CDH13	7	3	1	1.236475779	7.503909445	0.277828088	1
CDK5RAP2	7	4	1	0.922377319	4.359730392	0.230573749	1
CEL	5	2	1	1.32421267	14.04843817	0.214017959	1
CEP170	11	5	1	1.166870309	4.357892975	0.366182928	1
CEP250	7	4	1	0.922377319	4.359730392	0.230573749	1
CEP57	5	2	1	1.32421267	14.04843817	0.214017959	1
CHIT1	6	3	1	1.056847718	6.613295974	0.222355569	1
CHL1	26	13	1	1.060690368	2.314409385	0.508976052	1
CHPF	7	3	1	1.236475779	7.503909445	0.277828088	1
CKAP2L	7	4	1	0.922377319	4.359730392	0.230573749	1
CLCA1	6	3	1	1.056847718	6.613295974	0.222355569	1
CLCN1	22	12	1	0.965653579	2.200189712	0.443867588	1
CLEC4F	9	5	1	0.948984229	3.665287923	0.280199847	1
CLIP2	9	4	1	1.19284652	5.381296202	0.326878945	1
CMTM5	6	3	1	1.056847718	6.613295974	0.222355569	1
CNGA2	11	5	1	1.166870309	4.357892975	0.366182928	1
COL13A1	6	3	1	1.056847718	6.613295974	0.222355569	1
COL1A1	11	5	1	1.166870309	4.357892975	0.366182928	1
COL25A1	17	9	1	0.99709128	2.598933372	0.409443831	1
COLEC11	6	3	1	1.056847718	6.613295974	0.222355569	1
CPA4	9	5	1	0.948984229	3.665287923	0.280199847	1
CPE	6	3	1	1.056847718	6.613295974	0.222355569	1
CPN1	5	2	1	1.32421267	14.04843817	0.214017959	1
CPNE4	9	4	1	1.19284652	5.381296202	0.326878945	1
CPNE7	5	2	1	1.32421267	14.04843817	0.214017959	1
CPQ	9	4	1	1.19284652	5.381296202	0.326878945	1
CPVL	5	2	1	1.32421267	14.04843817	0.214017959	1
CPXM1	9	4	1	1.19284652	5.381296202	0.326878945	1
CREB3L2	5	2	1	1.32421267	14.04843817	0.214017959	1
CROCC	11	5	1	1.166870309	4.357892975	0.366182928	1
CRYGC	5	2	1	1.32421267	14.04843817	0.214017959	1
CSF2RB	9	4	1	1.19284652	5.381296202	0.326878945	1
CSPP1	11	6	1	0.966810152	3.243613275	0.320874982	1
CTC1	7	3	1	1.236475779	7.503909445	0.277828088	1
CTGLF11P	7	3	1	1.236475779	7.503909445	0.277828088	1
CTTN	5	2	1	1.32421267	14.04843817	0.214017959	1
CUL1	6	3	1	1.056847718	6.613295974	0.222355569	1
CUX1	9	4	1	1.19284652	5.381296202	0.326878945	1
CYP1A1	5	2	1	1.32421267	14.04843817	0.214017959	1
CYP4F3	7	4	1	0.922377319	4.359730392	0.230573749	1
DBH	7	3	1	1.236475779	7.503909445	0.277828088	1

DCLRE1A	9	4	1	1.19284652	5.381296202	0.326878945	1
DCT	9	4	1	1.19284652	5.381296202	0.326878945	1
DDHD1	7	3	1	1.236475779	7.503909445	0.277828088	1
DDR2	9	5	1	0.948984229	3.665287923	0.280199847	1
DDX26B	11	6	1	0.966810152	3.243613275	0.320874982	1
DENND4B	5	2	1	1.32421267	14.04843817	0.214017959	1
DGKB	23	12	1	1.012794614	2.29604161	0.469006413	1
DHX8	6	3	1	1.056847718	6.613295974	0.222355569	1
DIP2C	9	5	1	0.948984229	3.665287923	0.280199847	1
DISC1	6	3	1	1.056847718	6.613295974	0.222355569	1
DISP1	9	5	1	0.948984229	3.665287923	0.280199847	1
DMXL2	7	4	1	0.922377319	4.359730392	0.230573749	1
DNAJC6	11	5	1	1.166870309	4.357892975	0.366182928	1
DOCK3	11	6	1	0.966810152	3.243613275	0.320874982	1
DPP4	7	3	1	1.236475779	7.503909445	0.277828088	1
DPY19L4	6	3	1	1.056847718	6.613295974	0.222355569	1
DSCAML1	19	10	1	1.003293632	2.476501108	0.431647155	1
DSPP	6	3	1	1.056847718	6.613295974	0.222355569	1
DUSP10	6	3	1	1.056847718	6.613295974	0.222355569	1
DYNC1I1	9	4	1	1.19284652	5.381296202	0.326878945	1
DZIP1	5	2	1	1.32421267	14.04843817	0.214017959	1
EEF1A2	6	3	1	1.056847718	6.613295974	0.222355569	1
EEF2	5	2	1	1.32421267	14.04843817	0.214017959	1
EEPD1	5	2	1	1.32421267	14.04843817	0.214017959	1
EFS	6	3	1	1.056847718	6.613295974	0.222355569	1
EHHADH	7	3	1	1.236475779	7.503909445	0.277828088	1
EIF2AK1	6	3	1	1.056847718	6.613295974	0.222355569	1
EIF2AK3	6	3	1	1.056847718	6.613295974	0.222355569	1
EIF5B	7	4	1	0.922377319	4.359730392	0.230573749	1
ENGASE	6	3	1	1.056847718	6.613295974	0.222355569	1
ENPEP	9	5	1	0.948984229	3.665287923	0.280199847	1
ENPP7	5	2	1	1.32421267	14.04843817	0.214017959	1
EPC1	5	2	1	1.32421267	14.04843817	0.214017959	1
EPHA3	26	13	1	1.060690368	2.314409385	0.508976052	1
EPHA8	9	5	1	0.948984229	3.665287923	0.280199847	1
EPHB2	5	2	1	1.32421267	14.04843817	0.214017959	1
EPYC	5	2	1	1.32421267	14.04843817	0.214017959	1
EXO1	7	4	1	0.922377319	4.359730392	0.230573749	1
EXPH5	17	8	1	1.128434513	3.089144624	0.449825508	1
EYA2	9	4	1	1.19284652	5.381296202	0.326878945	1
EZH2	7	3	1	1.236475779	7.503909445	0.277828088	1
FAM120A	9	4	1	1.19284652	5.381296202	0.326878945	1
FAM129A	6	3	1	1.056847718	6.613295974	0.222355569	1
FAM129C	5	2	1	1.32421267	14.04843817	0.214017959	1
FAM169A	6	3	1	1.056847718	6.613295974	0.222355569	1
FAM47C	31	16	1	1.025292856	2.073772636	0.524735683	1
FAR2	7	3	1	1.236475779	7.503909445	0.277828088	1
FBN1	15	8	1	0.989431896	2.754371144	0.384167072	1
FBN3	15	8	1	0.989431896	2.754371144	0.384167072	1
FBXO30	7	3	1	1.236475779	7.503909445	0.277828088	1
FBXO40	7	3	1	1.236475779	7.503909445	0.277828088	1
FCAR	5	2	1	1.32421267	14.04843817	0.214017959	1
FCN2	7	3	1	1.236475779	7.503909445	0.277828088	1
FGA	9	5	1	0.948984229	3.665287923	0.280199847	1
FGF10	7	3	1	1.236475779	7.503909445	0.277828088	1
FGFR2	6	3	1	1.056847718	6.613295974	0.222355569	1
FH	7	3	1	1.236475779	7.503909445	0.277828088	1
FIGNL1	7	3	1	1.236475779	7.503909445	0.277828088	1

FLI1	9	4	1	1.19284652	5.381296202	0.326878945	1
FOXF2	5	2	1	1.32421267	14.04843817	0.214017959	1
FOXI1	9	4	1	1.19284652	5.381296202	0.326878945	1
FPR2	6	3	1	1.056847718	6.613295974	0.222355569	1
FRMD4A	5	2	1	1.32421267	14.04843817	0.214017959	1
FRMD6	9	5	1	0.948984229	3.665287923	0.280199847	1
FRMPD1	20	11	1	0.957352453	2.270551158	0.424790484	1
FRMPD4	22	11	1	1.059880796	2.485482144	0.478173409	1
G2E3	5	2	1	1.32421267	14.04843817	0.214017959	1
GAS2	6	3	1	1.056847718	6.613295974	0.222355569	1
GBF1	9	5	1	0.948984229	3.665287923	0.280199847	1
GDPD2	6	3	1	1.056847718	6.613295974	0.222355569	1
GFAP	5	2	1	1.32421267	14.04843817	0.214017959	1
GIMAP1	6	3	1	1.056847718	6.613295974	0.222355569	1
GJA10	9	4	1	1.19284652	5.381296202	0.326878945	1
GLCCI1	6	3	1	1.056847718	6.613295974	0.222355569	1
GLIPR1	5	2	1	1.32421267	14.04843817	0.214017959	1
GLRA2	11	6	1	0.966810152	3.243613275	0.320874982	1
GLYATL2	5	2	1	1.32421267	14.04843817	0.214017959	1
GMDS	5	2	1	1.32421267	14.04843817	0.214017959	1
GMEB2	6	3	1	1.056847718	6.613295974	0.222355569	1
GNL3L	5	2	1	1.32421267	14.04843817	0.214017959	1
GPA33	9	4	1	1.19284652	5.381296202	0.326878945	1
GPATCH2	5	2	1	1.32421267	14.04843817	0.214017959	1
GPR101	9	5	1	0.948984229	3.665287923	0.280199847	1
GPR142	7	3	1	1.236475779	7.503909445	0.277828088	1
GPR179	11	6	1	0.966810152	3.243613275	0.320874982	1
GPR31	5	2	1	1.32421267	14.04843817	0.214017959	1
GPR39	5	2	1	1.32421267	14.04843817	0.214017959	1
GPR50	11	5	1	1.166870309	4.357892975	0.366182928	1
GPR63	6	3	1	1.056847718	6.613295974	0.222355569	1
GPR85	9	4	1	1.19284652	5.381296202	0.326878945	1
GRID2	28	14	1	1.061103427	2.248246178	0.522296747	1
GRIN2A	31	16	1	1.025292856	2.073772636	0.524735683	1
GTF2IRD1	6	3	1	1.056847718	6.613295974	0.222355569	1
GTPBP1	5	2	1	1.32421267	14.04843817	0.214017959	1
HAND2	6	3	1	1.056847718	6.613295974	0.222355569	1
HAPLN1	9	5	1	0.948984229	3.665287923	0.280199847	1
HAVCR2	7	3	1	1.236475779	7.503909445	0.277828088	1
HCK	9	5	1	0.948984229	3.665287923	0.280199847	1
HDAC2	5	2	1	1.32421267	14.04843817	0.214017959	1
HEPACAM	5	2	1	1.32421267	14.04843817	0.214017959	1
HERC6	5	2	1	1.32421267	14.04843817	0.214017959	1
HFE2	5	2	1	1.32421267	14.04843817	0.214017959	1
HIPK1	7	4	1	0.922377319	4.359730392	0.230573749	1
HIST1H2AC	5	2	1	1.32421267	14.04843817	0.214017959	1
HNRNPA1	5	2	1	1.32421267	14.04843817	0.214017959	1
HNRNPA3	5	2	1	1.32421267	14.04843817	0.214017959	1
HNRNPM	5	2	1	1.32421267	14.04843817	0.214017959	1
HOOK3	5	2	1	1.32421267	14.04843817	0.214017959	1
HSD3B2	9	4	1	1.19284652	5.381296202	0.326878945	1
HSPA12A	9	4	1	1.19284652	5.381296202	0.326878945	1
HSPG2	14	7	1	1.058324818	3.162286269	0.390371972	1
HYOU1	6	3	1	1.056847718	6.613295974	0.222355569	1
IARS2	9	5	1	0.948984229	3.665287923	0.280199847	1
IFNA16	5	2	1	1.32421267	14.04843817	0.214017959	1
IGDCC4	13	6	1	1.149648897	3.759608513	0.398659974	1
IGF2BP3	6	3	1	1.056847718	6.613295974	0.222355569	1

IGFN1	9	4	1	1.19284652	5.381296202	0.326878945	1
IGHG3	5	2	1	1.32421267	14.04843817	0.214017959	1
IGKV1-27	6	3	1	1.056847718	6.613295974	0.222355569	1
IGKV1-5	9	5	1	0.948984229	3.665287923	0.280199847	1
IGKV1D-42	5	2	1	1.32421267	14.04843817	0.214017959	1
IGKV2D-24	6	3	1	1.056847718	6.613295974	0.222355569	1
IKBKE	6	3	1	1.056847718	6.613295974	0.222355569	1
IKZF2	6	3	1	1.056847718	6.613295974	0.222355569	1
IL4R	6	3	1	1.056847718	6.613295974	0.222355569	1
IMPG1	11	6	1	0.966810152	3.243613275	0.320874982	1
INPPL1	7	4	1	0.922377319	4.359730392	0.230573749	1
INSR	9	4	1	1.19284652	5.381296202	0.326878945	1
IQSEC2	7	4	1	0.922377319	4.359730392	0.230573749	1
IQSEC3	7	3	1	1.236475779	7.503909445	0.277828088	1
IRF8	7	3	1	1.236475779	7.503909445	0.277828088	1
IRX3	5	2	1	1.32421267	14.04843817	0.214017959	1
IRX6	5	2	1	1.32421267	14.04843817	0.214017959	1
ITGA3	5	2	1	1.32421267	14.04843817	0.214017959	1
ITGA5	5	2	1	1.32421267	14.04843817	0.214017959	1
ITGAE	11	5	1	1.166870309	4.357892975	0.366182928	1
ITGAL	15	7	1	1.137466096	3.36666144	0.426154782	1
ITGAM	15	7	1	1.137466096	3.36666144	0.426154782	1
ITGAV	9	4	1	1.19284652	5.381296202	0.326878945	1
ITLN1	6	3	1	1.056847718	6.613295974	0.222355569	1
JADE3	7	3	1	1.236475779	7.503909445	0.277828088	1
JARID2	7	4	1	0.922377319	4.359730392	0.230573749	1
KBTBD3	6	3	1	1.056847718	6.613295974	0.222355569	1
KCNA2	6	3	1	1.056847718	6.613295974	0.222355569	1
KCND3	7	4	1	0.922377319	4.359730392	0.230573749	1
KCNG4	6	3	1	1.056847718	6.613295974	0.222355569	1
KCNH3	9	5	1	0.948984229	3.665287923	0.280199847	1
KCNJ5	7	3	1	1.236475779	7.503909445	0.277828088	1
KCNMA1	11	5	1	1.166870309	4.357892975	0.366182928	1
KCNN1	5	2	1	1.32421267	14.04843817	0.214017959	1
KCTD16	7	4	1	0.922377319	4.359730392	0.230573749	1
KDM3B	9	4	1	1.19284652	5.381296202	0.326878945	1
KDM4C	9	4	1	1.19284652	5.381296202	0.326878945	1
KHDC3L	7	3	1	1.236475779	7.503909445	0.277828088	1
KHDRBS2	7	3	1	1.236475779	7.503909445	0.277828088	1
KIAA0368	6	3	1	1.056847718	6.613295974	0.222355569	1
KIDINS220	9	5	1	0.948984229	3.665287923	0.280199847	1
KIF20B	7	4	1	0.922377319	4.359730392	0.230573749	1
KIF21B	20	11	1	0.957352453	2.270551158	0.424790484	1
KIF26B	11	6	1	0.966810152	3.243613275	0.320874982	1
KIF6	9	5	1	0.948984229	3.665287923	0.280199847	1
KIR3DL1	11	5	1	1.166870309	4.357892975	0.366182928	1
KIT	5	2	1	1.32421267	14.04843817	0.214017959	1
KLF17	6	3	1	1.056847718	6.613295974	0.222355569	1
KLF4	5	2	1	1.32421267	14.04843817	0.214017959	1
KLHL18	6	3	1	1.056847718	6.613295974	0.222355569	1
KLK8	6	3	1	1.056847718	6.613295974	0.222355569	1
KRT14	5	2	1	1.32421267	14.04843817	0.214017959	1
KRT25	7	4	1	0.922377319	4.359730392	0.230573749	1
KRT6A	7	3	1	1.236475779	7.503909445	0.277828088	1
LAMP5	9	5	1	0.948984229	3.665287923	0.280199847	1
LAYN	5	2	1	1.32421267	14.04843817	0.214017959	1
LCE3E	5	2	1	1.32421267	14.04843817	0.214017959	1
LCP2	5	2	1	1.32421267	14.04843817	0.214017959	1

LDLRAD4	6	3	1	1.056847718	6.613295974	0.222355569	1
LEF1	9	5	1	0.948984229	3.665287923	0.280199847	1
LGI2	9	4	1	1.19284652	5.381296202	0.326878945	1
LGR6	9	4	1	1.19284652	5.381296202	0.326878945	1
LHCGR	7	3	1	1.236475779	7.503909445	0.277828088	1
LHX9	6	3	1	1.056847718	6.613295974	0.222355569	1
LILRA4	6	3	1	1.056847718	6.613295974	0.222355569	1
LILRA6	7	3	1	1.236475779	7.503909445	0.277828088	1
LINS	7	3	1	1.236475779	7.503909445	0.277828088	1
LLGL2	7	3	1	1.236475779	7.503909445	0.277828088	1
LMAN1L	5	2	1	1.32421267	14.04843817	0.214017959	1
LMTK2	7	3	1	1.236475779	7.503909445	0.277828088	1
LRFN5	28	14	1	1.061103427	2.248246178	0.522296747	1
LRP1	23	12	1	1.012794614	2.29604161	0.469006413	1
LRP4	14	7	1	1.058324818	3.162286269	0.390371972	1
LRP6	9	5	1	0.948984229	3.665287923	0.280199847	1
LRRC30	6	3	1	1.056847718	6.613295974	0.222355569	1
LRRC8C	6	3	1	1.056847718	6.613295974	0.222355569	1
LRRK2	13	6	1	1.149648897	3.759608513	0.398659974	1
LRRN3	11	6	1	0.966810152	3.243613275	0.320874982	1
LYZL2	5	2	1	1.32421267	14.04843817	0.214017959	1
MACF1	30	16	1	0.988888918	2.006373414	0.503923774	1
MAEL	5	2	1	1.32421267	14.04843817	0.214017959	1
MAGEB6	11	5	1	1.166870309	4.357892975	0.366182928	1
MAN2B2	5	2	1	1.32421267	14.04843817	0.214017959	1
MANBA	7	3	1	1.236475779	7.503909445	0.277828088	1
MANEA	6	3	1	1.056847718	6.613295974	0.222355569	1
MAP3K11	5	2	1	1.32421267	14.04843817	0.214017959	1
MAP3K15	6	3	1	1.056847718	6.613295974	0.222355569	1
MAP3K4	11	5	1	1.166870309	4.357892975	0.366182928	1
MARS2	5	2	1	1.32421267	14.04843817	0.214017959	1
MAST1	7	4	1	0.922377319	4.359730392	0.230573749	1
MAST4	6	3	1	1.056847718	6.613295974	0.222355569	1
MATN2	6	3	1	1.056847718	6.613295974	0.222355569	1
MAX	5	2	1	1.32421267	14.04843817	0.214017959	1
MCHR2	7	4	1	0.922377319	4.359730392	0.230573749	1
MED23	7	4	1	0.922377319	4.359730392	0.230573749	1
MEGF8	9	5	1	0.948984229	3.665287923	0.280199847	1
MEI1	6	3	1	1.056847718	6.613295974	0.222355569	1
MEMO1	5	2	1	1.32421267	14.04843817	0.214017959	1
MET	11	5	1	1.166870309	4.357892975	0.366182928	1
MFRP	5	2	1	1.32421267	14.04843817	0.214017959	1
MIA3	9	5	1	0.948984229	3.665287923	0.280199847	1
MLH1	5	2	1	1.32421267	14.04843817	0.214017959	1
MLLT10	9	4	1	1.19284652	5.381296202	0.326878945	1
MLLT3	5	2	1	1.32421267	14.04843817	0.214017959	1
MPP2	6	3	1	1.056847718	6.613295974	0.222355569	1
MPPED2	13	6	1	1.149648897	3.759608513	0.398659974	1
MS4A14	15	7	1	1.137466096	3.36666144	0.426154782	1
MSC	5	2	1	1.32421267	14.04843817	0.214017959	1
MSH3	5	2	1	1.32421267	14.04843817	0.214017959	1
MSH4	11	5	1	1.166870309	4.357892975	0.366182928	1
MTM1	6	3	1	1.056847718	6.613295974	0.222355569	1
MTTP	7	3	1	1.236475779	7.503909445	0.277828088	1
MUSK	5	2	1	1.32421267	14.04843817	0.214017959	1
MYBPC3	9	4	1	1.19284652	5.381296202	0.326878945	1
MYL2	5	2	1	1.32421267	14.04843817	0.214017959	1
MYO1E	5	2	1	1.32421267	14.04843817	0.214017959	1

MYOCD	11	6	1	0.966810152	3.243613275	0.320874982	1
MYOM1	9	4	1	1.19284652	5.381296202	0.326878945	1
NAA16	6	3	1	1.056847718	6.613295974	0.222355569	1
NAP1L3	9	5	1	0.948984229	3.665287923	0.280199847	1
NAT10	6	3	1	1.056847718	6.613295974	0.222355569	1
NCKAP1	7	4	1	0.922377319	4.359730392	0.230573749	1
NCKAP1L	11	6	1	0.966810152	3.243613275	0.320874982	1
NCSTN	7	4	1	0.922377319	4.359730392	0.230573749	1
NDST3	7	4	1	0.922377319	4.359730392	0.230573749	1
NFATC3	7	4	1	0.922377319	4.359730392	0.230573749	1
NFKBIZ	5	2	1	1.32421267	14.04843817	0.214017959	1
NGEF	5	2	1	1.32421267	14.04843817	0.214017959	1
NISCH	5	2	1	1.32421267	14.04843817	0.214017959	1
NKX2-5	5	2	1	1.32421267	14.04843817	0.214017959	1
NLRC5	11	6	1	0.966810152	3.243613275	0.320874982	1
NOTCH2	11	6	1	0.966810152	3.243613275	0.320874982	1
NPHP3	5	2	1	1.32421267	14.04843817	0.214017959	1
NR4A3	5	2	1	1.32421267	14.04843817	0.214017959	1
NRD1	6	3	1	1.056847718	6.613295974	0.222355569	1
NTSR1	5	2	1	1.32421267	14.04843817	0.214017959	1
NUP188	6	3	1	1.056847718	6.613295974	0.222355569	1
NXF5	9	5	1	0.948984229	3.665287923	0.280199847	1
NXPH2	7	3	1	1.236475779	7.503909445	0.277828088	1
NYNRIN	9	5	1	0.948984229	3.665287923	0.280199847	1
OCRL	7	3	1	1.236475779	7.503909445	0.277828088	1
OPRL1	7	3	1	1.236475779	7.503909445	0.277828088	1
OR10H4	5	2	1	1.32421267	14.04843817	0.214017959	1
OR10P1	5	2	1	1.32421267	14.04843817	0.214017959	1
OR11G2	9	5	1	0.948984229	3.665287923	0.280199847	1
OR13C2	7	3	1	1.236475779	7.503909445	0.277828088	1
OR13H1	5	2	1	1.32421267	14.04843817	0.214017959	1
OR1L8	7	3	1	1.236475779	7.503909445	0.277828088	1
OR1N2	7	4	1	0.922377319	4.359730392	0.230573749	1
OR1Q1	7	4	1	0.922377319	4.359730392	0.230573749	1
OR2A12	9	4	1	1.19284652	5.381296202	0.326878945	1
OR2AK2	15	8	1	0.989431896	2.754371144	0.384167072	1
OR2H2	5	2	1	1.32421267	14.04843817	0.214017959	1
OR2L3	11	5	1	1.166870309	4.357892975	0.366182928	1
OR2L8	20	11	1	0.957352453	2.270551158	0.424790484	1
OR2M2	13	6	1	1.149648897	3.759608513	0.398659974	1
OR2M5	19	10	1	1.003293632	2.476501108	0.431647155	1
OR2T2	11	5	1	1.166870309	4.357892975	0.366182928	1
OR2W3	23	12	1	1.012794614	2.29604161	0.469006413	1
OR2Z1	5	2	1	1.32421267	14.04843817	0.214017959	1
OR4A15	24	13	1	0.972672636	2.140822536	0.461051195	1
OR4C12	6	3	1	1.056847718	6.613295974	0.222355569	1
OR4D9	7	3	1	1.236475779	7.503909445	0.277828088	1
OR4K5	9	5	1	0.948984229	3.665287923	0.280199847	1
OR4M1	15	8	1	0.989431896	2.754371144	0.384167072	1
OR51B6	6	3	1	1.056847718	6.613295974	0.222355569	1
OR51E1	5	2	1	1.32421267	14.04843817	0.214017959	1
OR51E2	9	4	1	1.19284652	5.381296202	0.326878945	1
OR51I1	5	2	1	1.32421267	14.04843817	0.214017959	1
OR52B2	5	2	1	1.32421267	14.04843817	0.214017959	1
OR52J3	7	3	1	1.236475779	7.503909445	0.277828088	1
OR52K2	6	3	1	1.056847718	6.613295974	0.222355569	1
OR56B1	6	3	1	1.056847718	6.613295974	0.222355569	1
OR5H14	5	2	1	1.32421267	14.04843817	0.214017959	1

OR5K2	5	2	1	1.32421267	14.04843817	0.214017959	1
OR5M3	9	5	1	0.948984229	3.665287923	0.280199847	1
OR5T3	13	6	1	1.149648897	3.759608513	0.398659974	1
OR5V1	5	2	1	1.32421267	14.04843817	0.214017959	1
OR7C2	5	2	1	1.32421267	14.04843817	0.214017959	1
OR8D4	6	3	1	1.056847718	6.613295974	0.222355569	1
OR9A2	7	3	1	1.236475779	7.503909445	0.277828088	1
OR9G1	9	4	1	1.19284652	5.381296202	0.326878945	1
OR9K2	5	2	1	1.32421267	14.04843817	0.214017959	1
OSBPL6	9	4	1	1.19284652	5.381296202	0.326878945	1
OTUD6A	9	5	1	0.948984229	3.665287923	0.280199847	1
OTX2	5	2	1	1.32421267	14.04843817	0.214017959	1
P2RY8	6	3	1	1.056847718	6.613295974	0.222355569	1
PABPC3	5	2	1	1.32421267	14.04843817	0.214017959	1
PAK7	18	10	1	0.947473805	2.354901545	0.403436631	1
PASK	6	3	1	1.056847718	6.613295974	0.222355569	1
PAX1	7	3	1	1.236475779	7.503909445	0.277828088	1
PAX6	6	3	1	1.056847718	6.613295974	0.222355569	1
PCDHA12	11	6	1	0.966810152	3.243613275	0.320874982	1
PCDHA4	17	8	1	1.128434513	3.089144624	0.449825508	1
PCDHB14	18	9	1	1.059092557	2.740321176	0.439843457	1
PCDHGA11	9	4	1	1.19284652	5.381296202	0.326878945	1
PCDHGC5	6	3	1	1.056847718	6.613295974	0.222355569	1
PCNXL3	7	4	1	0.922377319	4.359730392	0.230573749	1
PDE1A	9	5	1	0.948984229	3.665287923	0.280199847	1
PDE1C	15	8	1	0.989431896	2.754371144	0.384167072	1
PDE2A	6	3	1	1.056847718	6.613295974	0.222355569	1
PDE3B	7	4	1	0.922377319	4.359730392	0.230573749	1
PDGFRB	9	5	1	0.948984229	3.665287923	0.280199847	1
PDILT	15	7	1	1.137466096	3.36666144	0.426154782	1
PDZRN3	20	11	1	0.957352453	2.270551158	0.424790484	1
PFKP	5	2	1	1.32421267	14.04843817	0.214017959	1
PHF21B	5	2	1	1.32421267	14.04843817	0.214017959	1
PHKA1	9	5	1	0.948984229	3.665287923	0.280199847	1
PI4KB	7	4	1	0.922377319	4.359730392	0.230573749	1
PIWIL1	9	4	1	1.19284652	5.381296202	0.326878945	1
PIWIL2	7	3	1	1.236475779	7.503909445	0.277828088	1
PKP2	16	9	1	0.935495847	2.458375385	0.379300029	1
PLAT	7	3	1	1.236475779	7.503909445	0.277828088	1
PLB1	11	6	1	0.966810152	3.243613275	0.320874982	1
PLCXD3	9	4	1	1.19284652	5.381296202	0.326878945	1
PLD5	7	4	1	0.922377319	4.359730392	0.230573749	1
PLEKHG4	7	3	1	1.236475779	7.503909445	0.277828088	1
PLEKHS1	5	2	1	1.32421267	14.04843817	0.214017959	1
PLK1	5	2	1	1.32421267	14.04843817	0.214017959	1
PLSCR1	6	3	1	1.056847718	6.613295974	0.222355569	1
PLVAP	5	2	1	1.32421267	14.04843817	0.214017959	1
PLXNB3	9	5	1	0.948984229	3.665287923	0.280199847	1
POTEB3	9	4	1	1.19284652	5.381296202	0.326878945	1
POTEE	14	7	1	1.058324818	3.162286269	0.390371972	1
PPP2R2C	5	2	1	1.32421267	14.04843817	0.214017959	1
PPRC1	11	6	1	0.966810152	3.243613275	0.320874982	1
PRCP	5	2	1	1.32421267	14.04843817	0.214017959	1
PRDM16	11	5	1	1.166870309	4.357892975	0.366182928	1
PRKCH	5	2	1	1.32421267	14.04843817	0.214017959	1
PRKG1	11	6	1	0.966810152	3.243613275	0.320874982	1
PRLR	11	5	1	1.166870309	4.357892975	0.366182928	1
PRPF40B	6	3	1	1.056847718	6.613295974	0.222355569	1

PRR23A	6	3	1	1.056847718	6.613295974	0.222355569	1
PRR30	7	3	1	1.236475779	7.503909445	0.277828088	1
PSG8	15	8	1	0.989431896	2.754371144	0.384167072	1
PTCHD2	25	13	1	1.016542601	2.227192272	0.484920248	1
PTGS1	11	6	1	0.966810152	3.243613275	0.320874982	1
PTPRG	6	3	1	1.056847718	6.613295974	0.222355569	1
PTPRU	11	5	1	1.166870309	4.357892975	0.366182928	1
PUS7L	5	2	1	1.32421267	14.04843817	0.214017959	1
PYGM	6	3	1	1.056847718	6.613295974	0.222355569	1
R3HDM1	7	3	1	1.236475779	7.503909445	0.277828088	1
R3HDML	6	3	1	1.056847718	6.613295974	0.222355569	1
RAB3IP	6	3	1	1.056847718	6.613295974	0.222355569	1
RABGEF1	5	2	1	1.32421267	14.04843817	0.214017959	1
RAD50	7	3	1	1.236475779	7.503909445	0.277828088	1
RAD54B	7	3	1	1.236475779	7.503909445	0.277828088	1
RAF1	5	2	1	1.32421267	14.04843817	0.214017959	1
RAI2	6	3	1	1.056847718	6.613295974	0.222355569	1
RALYL	7	4	1	0.922377319	4.359730392	0.230573749	1
RANBP2	17	9	1	0.99709128	2.598933372	0.409443831	1
RANBP3L	6	3	1	1.056847718	6.613295974	0.222355569	1
RASA2	6	3	1	1.056847718	6.613295974	0.222355569	1
RASGRP4	7	3	1	1.236475779	7.503909445	0.277828088	1
RB1	20	10	1	1.059484063	2.598745485	0.460108802	1
RBM19	11	5	1	1.166870309	4.357892975	0.366182928	1
RC3H1	9	4	1	1.19284652	5.381296202	0.326878945	1
REG1B	14	7	1	1.058324818	3.162286269	0.390371972	1
REG3A	18	10	1	0.947473805	2.354901545	0.403436631	1
RFX1	5	2	1	1.32421267	14.04843817	0.214017959	1
RGS18	7	3	1	1.236475779	7.503909445	0.277828088	1
RGS6	9	4	1	1.19284652	5.381296202	0.326878945	1
RLIM	7	3	1	1.236475779	7.503909445	0.277828088	1
RLTPR	9	4	1	1.19284652	5.381296202	0.326878945	1
RNF144A	5	2	1	1.32421267	14.04843817	0.214017959	1
ROCK2	5	2	1	1.32421267	14.04843817	0.214017959	1
RORB	7	3	1	1.236475779	7.503909445	0.277828088	1
P11-812E19	7	3	1	1.236475779	7.503909445	0.277828088	1
RPAP1	9	5	1	0.948984229	3.665287923	0.280199847	1
RPL5	5	2	1	1.32421267	14.04843817	0.214017959	1
RPS6KL1	5	2	1	1.32421267	14.04843817	0.214017959	1
RPTOR	9	4	1	1.19284652	5.381296202	0.326878945	1
RSBN1	6	3	1	1.056847718	6.613295974	0.222355569	1
RSF1	6	3	1	1.056847718	6.613295974	0.222355569	1
RSPH10B2	5	2	1	1.32421267	14.04843817	0.214017959	1
RTCB	5	2	1	1.32421267	14.04843817	0.214017959	1
RTL1	5	2	1	1.32421267	14.04843817	0.214017959	1
SACS	23	12	1	1.012794614	2.29604161	0.469006413	1
SALL3	15	8	1	0.989431896	2.754371144	0.384167072	1
SAMD9	17	9	1	0.99709128	2.598933372	0.409443831	1
SAMHD1	5	2	1	1.32421267	14.04843817	0.214017959	1
SAXO1	7	3	1	1.236475779	7.503909445	0.277828088	1
SBF2	9	4	1	1.19284652	5.381296202	0.326878945	1
SCG3	7	3	1	1.236475779	7.503909445	0.277828088	1
SCML2	7	4	1	0.922377319	4.359730392	0.230573749	1
SCN10A	25	13	1	1.016542601	2.227192272	0.484920248	1
SCN5A	20	10	1	1.059484063	2.598745485	0.460108802	1
SEC24A	6	3	1	1.056847718	6.613295974	0.222355569	1
SEC31A	5	2	1	1.32421267	14.04843817	0.214017959	1
SEMA3E	7	4	1	0.922377319	4.359730392	0.230573749	1

SEMA4C	5	2	1	1.32421267	14.04843817	0.214017959	1
SEMG1	5	2	1	1.32421267	14.04843817	0.214017959	1
SERGEF	6	3	1	1.056847718	6.613295974	0.222355569	1
SERPINA1(	6	3	1	1.056847718	6.613295974	0.222355569	1
SERPING1	5	2	1	1.32421267	14.04843817	0.214017959	1
SETDB1	5	2	1	1.32421267	14.04843817	0.214017959	1
SF3A1	6	3	1	1.056847718	6.613295974	0.222355569	1
SH2D3C	7	3	1	1.236475779	7.503909445	0.277828088	1
SH3BP4	9	5	1	0.948984229	3.665287923	0.280199847	1
SH3GL2	5	2	1	1.32421267	14.04843817	0.214017959	1
SIGLEC14	6	3	1	1.056847718	6.613295974	0.222355569	1
SIGLEC7	5	2	1	1.32421267	14.04843817	0.214017959	1
SIN3A	7	4	1	0.922377319	4.359730392	0.230573749	1
SKIDA1	7	3	1	1.236475779	7.503909445	0.277828088	1
SLAMF1	5	2	1	1.32421267	14.04843817	0.214017959	1
SLC12A2	11	5	1	1.166870309	4.357892975	0.366182928	1
SLC26A10	9	5	1	0.948984229	3.665287923	0.280199847	1
SLC26A4	7	3	1	1.236475779	7.503909445	0.277828088	1
SLC26A5	6	3	1	1.056847718	6.613295974	0.222355569	1
SLC34A1	7	3	1	1.236475779	7.503909445	0.277828088	1
SLC35G3	7	3	1	1.236475779	7.503909445	0.277828088	1
SLC36A2	5	2	1	1.32421267	14.04843817	0.214017959	1
SLC3A1	7	4	1	0.922377319	4.359730392	0.230573749	1
SLC4A10	11	6	1	0.966810152	3.243613275	0.320874982	1
SLC4A2	7	3	1	1.236475779	7.503909445	0.277828088	1
SLC6A19	7	4	1	0.922377319	4.359730392	0.230573749	1
SLC9B2	6	3	1	1.056847718	6.613295974	0.222355569	1
SLIT3	17	8	1	1.128434513	3.089144624	0.449825508	1
SMOC1	7	4	1	0.922377319	4.359730392	0.230573749	1
SMPD3	5	2	1	1.32421267	14.04843817	0.214017959	1
SNAP91	9	4	1	1.19284652	5.381296202	0.326878945	1
SOGA3	7	4	1	0.922377319	4.359730392	0.230573749	1
SOHLH2	5	2	1	1.32421267	14.04843817	0.214017959	1
SORCS3	28	15	1	0.984152034	2.045395189	0.490879792	1
SP110	5	2	1	1.32421267	14.04843817	0.214017959	1
SPAG5	5	2	1	1.32421267	14.04843817	0.214017959	1
SPATA8	5	2	1	1.32421267	14.04843817	0.214017959	1
SPECC1L	5	2	1	1.32421267	14.04843817	0.214017959	1
SPRY3	5	2	1	1.32421267	14.04843817	0.214017959	1
SPTAN1	11	6	1	0.966810152	3.243613275	0.320874982	1
SPTB	13	6	1	1.149648897	3.759608513	0.398659974	1
SPTBN4	11	6	1	0.966810152	3.243613275	0.320874982	1
SPZ1	6	3	1	1.056847718	6.613295974	0.222355569	1
ST8SIA3	7	4	1	0.922377319	4.359730392	0.230573749	1
ST8SIA6	11	6	1	0.966810152	3.243613275	0.320874982	1
STAC	5	2	1	1.32421267	14.04843817	0.214017959	1
STIM1	6	3	1	1.056847718	6.613295974	0.222355569	1
STPG2	6	3	1	1.056847718	6.613295974	0.222355569	1
STRN	6	3	1	1.056847718	6.613295974	0.222355569	1
STRN3	5	2	1	1.32421267	14.04843817	0.214017959	1
STX2	5	2	1	1.32421267	14.04843817	0.214017959	1
STXBP5L	16	9	1	0.935495847	2.458375385	0.379300029	1
SUGCT	5	2	1	1.32421267	14.04843817	0.214017959	1
SUGP2	5	2	1	1.32421267	14.04843817	0.214017959	1
SULT1E1	9	4	1	1.19284652	5.381296202	0.326878945	1
SUPT6H	11	5	1	1.166870309	4.357892975	0.366182928	1
SVIL	11	5	1	1.166870309	4.357892975	0.366182928	1
SYN3	5	2	1	1.32421267	14.04843817	0.214017959	1

SYNCRIP	6	3	1	1.056847718	6.613295974	0.222355569	1
SYNJ1	6	3	1	1.056847718	6.613295974	0.222355569	1
SYNPO2	15	8	1	0.989431896	2.754371144	0.384167072	1
SYP	5	2	1	1.32421267	14.04843817	0.214017959	1
SYT14	7	4	1	0.922377319	4.359730392	0.230573749	1
TAF1A	5	2	1	1.32421267	14.04843817	0.214017959	1
TAF1L	32	17	1	0.993116089	1.972030725	0.515945248	1
TARBP1	7	3	1	1.236475779	7.503909445	0.277828088	1
TBC1D2	5	2	1	1.32421267	14.04843817	0.214017959	1
TBC1D21	5	2	1	1.32421267	14.04843817	0.214017959	1
TBC1D9B	5	2	1	1.32421267	14.04843817	0.214017959	1
TBX18	9	5	1	0.948984229	3.665287923	0.280199847	1
TBX5	11	6	1	0.966810152	3.243613275	0.320874982	1
TCAF1	6	3	1	1.056847718	6.613295974	0.222355569	1
TCEB3B	9	5	1	0.948984229	3.665287923	0.280199847	1
TENM4	17	9	1	0.99709128	2.598933372	0.409443831	1
TFAP2D	14	7	1	1.058324818	3.162286269	0.390371972	1
TGIF2LX	11	6	1	0.966810152	3.243613275	0.320874982	1
TGM2	5	2	1	1.32421267	14.04843817	0.214017959	1
TH	5	2	1	1.32421267	14.04843817	0.214017959	1
THBS2	22	11	1	1.059880796	2.485482144	0.478173409	1
THNSL2	7	3	1	1.236475779	7.503909445	0.277828088	1
TIGD3	7	4	1	0.922377319	4.359730392	0.230573749	1
TJP1	7	4	1	0.922377319	4.359730392	0.230573749	1
TLR8	9	5	1	0.948984229	3.665287923	0.280199847	1
TMEM145	7	4	1	0.922377319	4.359730392	0.230573749	1
TMEM169	7	3	1	1.236475779	7.503909445	0.277828088	1
TMX3	5	2	1	1.32421267	14.04843817	0.214017959	1
TNKS1BP1	7	4	1	0.922377319	4.359730392	0.230573749	1
TOP2A	6	3	1	1.056847718	6.613295974	0.222355569	1
TP53I11	5	2	1	1.32421267	14.04843817	0.214017959	1
TP63	7	3	1	1.236475779	7.503909445	0.277828088	1
TPK1	7	4	1	0.922377319	4.359730392	0.230573749	1
TRBV4-2	7	4	1	0.922377319	4.359730392	0.230573749	1
TRIM68	5	2	1	1.32421267	14.04843817	0.214017959	1
TRIM72	6	3	1	1.056847718	6.613295974	0.222355569	1
TRIM9	9	5	1	0.948984229	3.665287923	0.280199847	1
TRIOBP	9	5	1	0.948984229	3.665287923	0.280199847	1
TRIP12	9	4	1	1.19284652	5.381296202	0.326878945	1
TRPM4	5	2	1	1.32421267	14.04843817	0.214017959	1
TRPS1	34	17	1	1.062377075	2.097748304	0.556084648	1
TRPV3	7	4	1	0.922377319	4.359730392	0.230573749	1
TRPV4	9	4	1	1.19284652	5.381296202	0.326878945	1
TSLP	5	2	1	1.32421267	14.04843817	0.214017959	1
TTF1	6	3	1	1.056847718	6.613295974	0.222355569	1
TTLL7	11	6	1	0.966810152	3.243613275	0.320874982	1
TXNDC2	5	2	1	1.32421267	14.04843817	0.214017959	1
UBE3D	5	2	1	1.32421267	14.04843817	0.214017959	1
UGGT1	7	3	1	1.236475779	7.503909445	0.277828088	1
UGT1A1	5	2	1	1.32421267	14.04843817	0.214017959	1
UGT1A3	5	2	1	1.32421267	14.04843817	0.214017959	1
UGT1A5	5	2	1	1.32421267	14.04843817	0.214017959	1
UPF3B	5	2	1	1.32421267	14.04843817	0.214017959	1
USP11	6	3	1	1.056847718	6.613295974	0.222355569	1
USP33	7	3	1	1.236475779	7.503909445	0.277828088	1
VARS	5	2	1	1.32421267	14.04843817	0.214017959	1
VNN2	7	4	1	0.922377319	4.359730392	0.230573749	1
VPS41	5	2	1	1.32421267	14.04843817	0.214017959	1

VSNL1	5	2	1	1.32421267	14.04843817	0.214017959	1
VSTM4	6	3	1	1.056847718	6.613295974	0.222355569	1
VWA5A	7	3	1	1.236475779	7.503909445	0.277828088	1
VWC2	7	4	1	0.922377319	4.359730392	0.230573749	1
VWC2L	6	3	1	1.056847718	6.613295974	0.222355569	1
WDR47	5	2	1	1.32421267	14.04843817	0.214017959	1
WIPF1	6	3	1	1.056847718	6.613295974	0.222355569	1
WWC1	7	3	1	1.236475779	7.503909445	0.277828088	1
YTHDC2	6	3	1	1.056847718	6.613295974	0.222355569	1
ZBTB41	11	5	1	1.166870309	4.357892975	0.366182928	1
ZBTB49	5	2	1	1.32421267	14.04843817	0.214017959	1
ZBTB7B	5	2	1	1.32421267	14.04843817	0.214017959	1
ZCCHC16	9	4	1	1.19284652	5.381296202	0.326878945	1
ZFYVE28	9	4	1	1.19284652	5.381296202	0.326878945	1
ZGRF1	11	5	1	1.166870309	4.357892975	0.366182928	1
ZMYND8	9	4	1	1.19284652	5.381296202	0.326878945	1
ZNF142	9	4	1	1.19284652	5.381296202	0.326878945	1
ZNF160	7	4	1	0.922377319	4.359730392	0.230573749	1
ZNF239	5	2	1	1.32421267	14.04843817	0.214017959	1
ZNF251	5	2	1	1.32421267	14.04843817	0.214017959	1
ZNF284	5	2	1	1.32421267	14.04843817	0.214017959	1
ZNF331	7	3	1	1.236475779	7.503909445	0.277828088	1
ZNF335	7	4	1	0.922377319	4.359730392	0.230573749	1
ZNF418	5	2	1	1.32421267	14.04843817	0.214017959	1
ZNF507	5	2	1	1.32421267	14.04843817	0.214017959	1
ZNF518B	11	6	1	0.966810152	3.243613275	0.320874982	1
ZNF532	6	3	1	1.056847718	6.613295974	0.222355569	1
ZNF547	5	2	1	1.32421267	14.04843817	0.214017959	1
ZNF549	5	2	1	1.32421267	14.04843817	0.214017959	1
ZNF611	7	4	1	0.922377319	4.359730392	0.230573749	1
ZNF616	7	4	1	0.922377319	4.359730392	0.230573749	1
ZNF630	5	2	1	1.32421267	14.04843817	0.214017959	1
ZNF644	7	3	1	1.236475779	7.503909445	0.277828088	1
ZNF646	6	3	1	1.056847718	6.613295974	0.222355569	1
ZNF665	7	4	1	0.922377319	4.359730392	0.230573749	1
ZNF713	7	4	1	0.922377319	4.359730392	0.230573749	1
ZNF728	7	4	1	0.922377319	4.359730392	0.230573749	1
ZNF746	6	3	1	1.056847718	6.613295974	0.222355569	1
ZNF780B	7	4	1	0.922377319	4.359730392	0.230573749	1
ZNF786	5	2	1	1.32421267	14.04843817	0.214017959	1
ZNF79	5	2	1	1.32421267	14.04843817	0.214017959	1
ZNF835	11	6	1	0.966810152	3.243613275	0.320874982	1
ZSCAN20	9	5	1	0.948984229	3.665287923	0.280199847	1
ZXDA	5	2	1	1.32421267	14.04843817	0.214017959	1
ZZZ3	7	4	1	0.922377319	4.359730392	0.230573749	1
AADACL2	5	3	1	0.878264758	5.724376621	0.168518419	1
AAK1	5	3	1	0.878264758	5.724376621	0.168518419	1
ABAT	5	3	1	0.878264758	5.724376621	0.168518419	1
ABCC6	5	3	1	0.878264758	5.724376621	0.168518419	1
ACVR1C	5	3	1	0.878264758	5.724376621	0.168518419	1
ADAR	5	3	1	0.878264758	5.724376621	0.168518419	1
AGO1	5	3	1	0.878264758	5.724376621	0.168518419	1
AHI1	5	3	1	0.878264758	5.724376621	0.168518419	1
ALG11	5	3	1	0.878264758	5.724376621	0.168518419	1
ALOX12	5	3	1	0.878264758	5.724376621	0.168518419	1
ALPI	5	3	1	0.878264758	5.724376621	0.168518419	1
ANKRD11	5	3	1	0.878264758	5.724376621	0.168518419	1
AQP10	5	3	1	0.878264758	5.724376621	0.168518419	1

ARHGAP27	5	3	1	0.878264758	5.724376621	0.168518419	1
BAZ1A	5	3	1	0.878264758	5.724376621	0.168518419	1
BEST2	5	3	1	0.878264758	5.724376621	0.168518419	1
BMPR2	5	3	1	0.878264758	5.724376621	0.168518419	1
BRD8	5	3	1	0.878264758	5.724376621	0.168518419	1
BRWD1	5	3	1	0.878264758	5.724376621	0.168518419	1
BUD13	5	3	1	0.878264758	5.724376621	0.168518419	1
C15orf59	5	3	1	0.878264758	5.724376621	0.168518419	1
CA3	5	3	1	0.878264758	5.724376621	0.168518419	1
CASZ1	5	3	1	0.878264758	5.724376621	0.168518419	1
CATSPERG	5	3	1	0.878264758	5.724376621	0.168518419	1
CBFA2T3	5	3	1	0.878264758	5.724376621	0.168518419	1
CCDC74A	5	3	1	0.878264758	5.724376621	0.168518419	1
CDC42BPA	5	3	1	0.878264758	5.724376621	0.168518419	1
CDH5	5	3	1	0.878264758	5.724376621	0.168518419	1
CENPJ	5	3	1	0.878264758	5.724376621	0.168518419	1
CERS3	5	3	1	0.878264758	5.724376621	0.168518419	1
CFHR2	5	3	1	0.878264758	5.724376621	0.168518419	1
CLIP4	5	3	1	0.878264758	5.724376621	0.168518419	1
CLNK	5	3	1	0.878264758	5.724376621	0.168518419	1
CLVS1	5	3	1	0.878264758	5.724376621	0.168518419	1
COL10A1	5	3	1	0.878264758	5.724376621	0.168518419	1
CORO1C	5	3	1	0.878264758	5.724376621	0.168518419	1
CSN2	5	3	1	0.878264758	5.724376621	0.168518419	1
CYP4F12	5	3	1	0.878264758	5.724376621	0.168518419	1
CYP4V2	5	3	1	0.878264758	5.724376621	0.168518419	1
DCX	5	3	1	0.878264758	5.724376621	0.168518419	1
EFCAB3	5	3	1	0.878264758	5.724376621	0.168518419	1
EGFL6	5	3	1	0.878264758	5.724376621	0.168518419	1
EIF3E	5	3	1	0.878264758	5.724376621	0.168518419	1
EPHA1	5	3	1	0.878264758	5.724376621	0.168518419	1
ESYT2	5	3	1	0.878264758	5.724376621	0.168518419	1
FAM155B	5	3	1	0.878264758	5.724376621	0.168518419	1
FBXO34	5	3	1	0.878264758	5.724376621	0.168518419	1
FCER1A	5	3	1	0.878264758	5.724376621	0.168518419	1
FLNB	5	3	1	0.878264758	5.724376621	0.168518419	1
FUBP1	5	3	1	0.878264758	5.724376621	0.168518419	1
GABRD	5	3	1	0.878264758	5.724376621	0.168518419	1
GALNT18	5	3	1	0.878264758	5.724376621	0.168518419	1
GALNT7	5	3	1	0.878264758	5.724376621	0.168518419	1
GAS2L3	5	3	1	0.878264758	5.724376621	0.168518419	1
GPR4	5	3	1	0.878264758	5.724376621	0.168518419	1
GPR52	5	3	1	0.878264758	5.724376621	0.168518419	1
GPR65	5	3	1	0.878264758	5.724376621	0.168518419	1
GRIN2D	5	3	1	0.878264758	5.724376621	0.168518419	1
GTPBP10	5	3	1	0.878264758	5.724376621	0.168518419	1
HACE1	5	3	1	0.878264758	5.724376621	0.168518419	1
HAUS6	5	3	1	0.878264758	5.724376621	0.168518419	1
HBD	5	3	1	0.878264758	5.724376621	0.168518419	1
HBG2	5	3	1	0.878264758	5.724376621	0.168518419	1
HERC3	5	3	1	0.878264758	5.724376621	0.168518419	1
HGFAC	5	3	1	0.878264758	5.724376621	0.168518419	1
HHAT	5	3	1	0.878264758	5.724376621	0.168518419	1
HIF3A	5	3	1	0.878264758	5.724376621	0.168518419	1
HIRA	5	3	1	0.878264758	5.724376621	0.168518419	1
HMGCLL1	5	3	1	0.878264758	5.724376621	0.168518419	1
HSD11B1	5	3	1	0.878264758	5.724376621	0.168518419	1
HYAL4	5	3	1	0.878264758	5.724376621	0.168518419	1

IGF1R	5	3	1	0.878264758	5.724376621	0.168518419	1
IGHA1	5	3	1	0.878264758	5.724376621	0.168518419	1
IGHG1	5	3	1	0.878264758	5.724376621	0.168518419	1
IGHV1-58	5	3	1	0.878264758	5.724376621	0.168518419	1
INPP5B	5	3	1	0.878264758	5.724376621	0.168518419	1
JAK1	5	3	1	0.878264758	5.724376621	0.168518419	1
KCNJ8	5	3	1	0.878264758	5.724376621	0.168518419	1
KCNQ3	5	3	1	0.878264758	5.724376621	0.168518419	1
KHNYN	5	3	1	0.878264758	5.724376621	0.168518419	1
KIAA0195	5	3	1	0.878264758	5.724376621	0.168518419	1
KIAA0922	5	3	1	0.878264758	5.724376621	0.168518419	1
KLHL32	5	3	1	0.878264758	5.724376621	0.168518419	1
KLK15	5	3	1	0.878264758	5.724376621	0.168518419	1
KRT17	5	3	1	0.878264758	5.724376621	0.168518419	1
KRT35	5	3	1	0.878264758	5.724376621	0.168518419	1
KRTAP10-(	5	3	1	0.878264758	5.724376621	0.168518419	1
KRTAP26-1	5	3	1	0.878264758	5.724376621	0.168518419	1
KRTAP9-3	5	3	1	0.878264758	5.724376621	0.168518419	1
LANCL2	5	3	1	0.878264758	5.724376621	0.168518419	1
LCE2D	5	3	1	0.878264758	5.724376621	0.168518419	1
LIN7A	5	3	1	0.878264758	5.724376621	0.168518419	1
LIPC	5	3	1	0.878264758	5.724376621	0.168518419	1
LIPK	5	3	1	0.878264758	5.724376621	0.168518419	1
MAG	5	3	1	0.878264758	5.724376621	0.168518419	1
MAGEE2	5	3	1	0.878264758	5.724376621	0.168518419	1
MAML2	5	3	1	0.878264758	5.724376621	0.168518419	1
MAP2K1	5	3	1	0.878264758	5.724376621	0.168518419	1
MAP3K12	5	3	1	0.878264758	5.724376621	0.168518419	1
MAS1L	5	3	1	0.878264758	5.724376621	0.168518419	1
MC2R	5	3	1	0.878264758	5.724376621	0.168518419	1
MCAM	5	3	1	0.878264758	5.724376621	0.168518419	1
MKS1	5	3	1	0.878264758	5.724376621	0.168518419	1
MLK4	5	3	1	0.878264758	5.724376621	0.168518419	1
MOSPD2	5	3	1	0.878264758	5.724376621	0.168518419	1
MRGPRX3	5	3	1	0.878264758	5.724376621	0.168518419	1
MYBPC2	5	3	1	0.878264758	5.724376621	0.168518419	1
MYRIP	5	3	1	0.878264758	5.724376621	0.168518419	1
N4BP1	5	3	1	0.878264758	5.724376621	0.168518419	1
N4BP2	5	3	1	0.878264758	5.724376621	0.168518419	1
NARS2	5	3	1	0.878264758	5.724376621	0.168518419	1
NCR1	5	3	1	0.878264758	5.724376621	0.168518419	1
NETO2	5	3	1	0.878264758	5.724376621	0.168518419	1
NLRP6	5	3	1	0.878264758	5.724376621	0.168518419	1
NTF3	5	3	1	0.878264758	5.724376621	0.168518419	1
OR11A1	5	3	1	0.878264758	5.724376621	0.168518419	1
OR52B4	5	3	1	0.878264758	5.724376621	0.168518419	1
OR56B4	5	3	1	0.878264758	5.724376621	0.168518419	1
OR6C74	5	3	1	0.878264758	5.724376621	0.168518419	1
OR6M1	5	3	1	0.878264758	5.724376621	0.168518419	1
OR6X1	5	3	1	0.878264758	5.724376621	0.168518419	1
OR7G1	5	3	1	0.878264758	5.724376621	0.168518419	1
OR9Q2	5	3	1	0.878264758	5.724376621	0.168518419	1
OXGR1	5	3	1	0.878264758	5.724376621	0.168518419	1
PAPL	5	3	1	0.878264758	5.724376621	0.168518419	1
PAPOLG	5	3	1	0.878264758	5.724376621	0.168518419	1
PAPSS2	5	3	1	0.878264758	5.724376621	0.168518419	1
PELI2	5	3	1	0.878264758	5.724376621	0.168518419	1
PGLYRP3	5	3	1	0.878264758	5.724376621	0.168518419	1

PITPNM3	5	3	1	0.878264758	5.724376621	0.168518419	1
PLCB3	5	3	1	0.878264758	5.724376621	0.168518419	1
PPFIA3	5	3	1	0.878264758	5.724376621	0.168518419	1
PPIAL4A	5	3	1	0.878264758	5.724376621	0.168518419	1
PPIP5K2	5	3	1	0.878264758	5.724376621	0.168518419	1
PPP1R15B	5	3	1	0.878264758	5.724376621	0.168518419	1
PPP2R2A	5	3	1	0.878264758	5.724376621	0.168518419	1
PPP6R1	5	3	1	0.878264758	5.724376621	0.168518419	1
PRKAA2	5	3	1	0.878264758	5.724376621	0.168518419	1
PRR5L	5	3	1	0.878264758	5.724376621	0.168518419	1
QSOX2	5	3	1	0.878264758	5.724376621	0.168518419	1
RAG2	5	3	1	0.878264758	5.724376621	0.168518419	1
RASIP1	5	3	1	0.878264758	5.724376621	0.168518419	1
RBM27	5	3	1	0.878264758	5.724376621	0.168518419	1
SBF1	5	3	1	0.878264758	5.724376621	0.168518419	1
SEZ6L2	5	3	1	0.878264758	5.724376621	0.168518419	1
SH3PXD2B	5	3	1	0.878264758	5.724376621	0.168518419	1
SHC3	5	3	1	0.878264758	5.724376621	0.168518419	1
SIRPB1	5	3	1	0.878264758	5.724376621	0.168518419	1
SLC25A12	5	3	1	0.878264758	5.724376621	0.168518419	1
SLC5A2	5	3	1	0.878264758	5.724376621	0.168518419	1
SLC9A3	5	3	1	0.878264758	5.724376621	0.168518419	1
SLCO4A1	5	3	1	0.878264758	5.724376621	0.168518419	1
SMAD2	5	3	1	0.878264758	5.724376621	0.168518419	1
SNX31	5	3	1	0.878264758	5.724376621	0.168518419	1
SRPK3	5	3	1	0.878264758	5.724376621	0.168518419	1
SSMEM1	5	3	1	0.878264758	5.724376621	0.168518419	1
SSTR5	5	3	1	0.878264758	5.724376621	0.168518419	1
STIL	5	3	1	0.878264758	5.724376621	0.168518419	1
STK10	5	3	1	0.878264758	5.724376621	0.168518419	1
STON1	5	3	1	0.878264758	5.724376621	0.168518419	1
SULT1C3	5	3	1	0.878264758	5.724376621	0.168518419	1
SUPT20H	5	3	1	0.878264758	5.724376621	0.168518419	1
SWT1	5	3	1	0.878264758	5.724376621	0.168518419	1
SYTL2	5	3	1	0.878264758	5.724376621	0.168518419	1
TAAR2	5	3	1	0.878264758	5.724376621	0.168518419	1
TAOK2	5	3	1	0.878264758	5.724376621	0.168518419	1
TCAF2	5	3	1	0.878264758	5.724376621	0.168518419	1
TDRD3	5	3	1	0.878264758	5.724376621	0.168518419	1
TGM3	5	3	1	0.878264758	5.724376621	0.168518419	1
THBS1	5	3	1	0.878264758	5.724376621	0.168518419	1
THRAP3	5	3	1	0.878264758	5.724376621	0.168518419	1
TMEM131	5	3	1	0.878264758	5.724376621	0.168518419	1
TMEM135	5	3	1	0.878264758	5.724376621	0.168518419	1
TMEM71	5	3	1	0.878264758	5.724376621	0.168518419	1
TRIP6	5	3	1	0.878264758	5.724376621	0.168518419	1
TUBA3E	5	3	1	0.878264758	5.724376621	0.168518419	1
UGT2B10	5	3	1	0.878264758	5.724376621	0.168518419	1
URI1	5	3	1	0.878264758	5.724376621	0.168518419	1
USP15	5	3	1	0.878264758	5.724376621	0.168518419	1
USP47	5	3	1	0.878264758	5.724376621	0.168518419	1
VPS8	5	3	1	0.878264758	5.724376621	0.168518419	1
WFIKKN2	5	3	1	0.878264758	5.724376621	0.168518419	1
WHSC1	5	3	1	0.878264758	5.724376621	0.168518419	1
ZDHHC15	5	3	1	0.878264758	5.724376621	0.168518419	1
ZFP91	5	3	1	0.878264758	5.724376621	0.168518419	1
ZHX1	5	3	1	0.878264758	5.724376621	0.168518419	1
ZMYM1	5	3	1	0.878264758	5.724376621	0.168518419	1

ZMYM2	5	3	1	0.878264758	5.724376621	0.168518419	1
ZNF106	5	3	1	0.878264758	5.724376621	0.168518419	1
ZNF195	5	3	1	0.878264758	5.724376621	0.168518419	1
ZNF223	5	3	1	0.878264758	5.724376621	0.168518419	1
ZNF234	5	3	1	0.878264758	5.724376621	0.168518419	1
ZNF25	5	3	1	0.878264758	5.724376621	0.168518419	1
ZNF260	5	3	1	0.878264758	5.724376621	0.168518419	1
ZNF431	5	3	1	0.878264758	5.724376621	0.168518419	1
ZNF71	5	3	1	0.878264758	5.724376621	0.168518419	1
ZNF768	5	3	1	0.878264758	5.724376621	0.168518419	1
ZNF845	5	3	1	0.878264758	5.724376621	0.168518419	1
