# Supplementary material for: Identification and Validation of a Proliferation-Associated Score Model Predicting Survival in Lung Adenocarcinomas
Source: Dis Markers. 2021 Oct 21;2021:3219594. doi: 10.1155/2021/3219594 (PMC8554523; doi:10.1155/2021/3219594)
Supplement: Supplementary 2 — Table S1: the table showed genes associated with microenvironment of the 24 immune cell subsets. Table S2: the table showed the sequences of all the siRNAs and primers used in this study. Table S3: the table showed 55 genes selected for LASSO Cox regression; all the 55 genes showed the same tendency in cell proliferation (the CERES dependency score) and survival (HR). Table S4: the table showed six genes used in the model and their LASSO coefficient after LASSO Cox regression. Table S5: the table showed the summary of genomic alterations in the two groups, including the somatic mutation numbers of each gene in high and low score groups. Table S6: the table showed the differentially expressed genes (DEGs) between high score group and low score group identified by limma. Table S7: the table showed the differentially expressed miRNAs between high score group and low score group identified by limma. Table S8: the table showed the comparison the abundance of 24 types of immune cells between the two groups by Wilcoxon test. [file 3219594.f2.zip › Table S6.pdf]

Table S6. Differentially expressed genes (DEGs) between high-score group and low-score group identified by limma.

Genes	logFC	AveExpr	t	P.Value	adj.P.Val	B	logP	Group
SAMD11	-0.2095764	1.17574747	-3.0235761	0.00262754	0.01107124	-3.6545068	2.580450741	up-regulated in High
NOC2L	0.38973607	4.18495976	7.94027163	1.36E-14	4.46E-13	21.5204601	13.86596004	up-regulated in Low
PLEKHN1	-0.146214	1.77766196	-2.0272872	0.04316777	0.11613846	-6.1404964	1.364840341	up-regulated in High
HES4	0.33877789	2.62079235	3.99002498	7.60E-05	0.00048786	-0.3447953	4.118928335	up-regulated in Low
ISG15	0.40640271	6.10760343	3.13662036	0.00181069	0.00801581	-3.312436	2.742157045	up-regulated in Low
AGRN	-0.173331	5.26918854	-1.9842523	0.04777889	0.1259443	-6.2262269	1.320763931	up-regulated in High
C1orf159	0.15360087	1.63061611	3.43259712	0.00064788	0.0032462	-2.3598376	3.188503787	up-regulated in Low
TTLL10	-0.2362071	0.35311406	-4.8680525	1.52E-06	1.39E-05	3.39922344	5.818828347	up-regulated in High
TNFRSF18	0.2227996	2.46407188	2.07438096	0.03855963	0.10610213	-6.0446077	1.41386715	up-regulated in Low
UBE2J2	0.10571816	3.51973442	2.83533587	0.00476487	0.01853473	-4.1972288	2.321949201	up-regulated in Low
SCNN1D	-0.2090325	0.95176039	-3.4074943	0.00070899	0.00351617	-2.4438149	3.149359721	up-regulated in High
PUSL1	0.225054	2.79870403	4.15928862	3.76E-05	0.00025862	0.32335401	4.424496486	up-regulated in Low
DVL1	0.18166611	4.58148115	2.73958115	0.00637355	0.02380277	-4.4603479	2.195618562	up-regulated in Low
MXRA8	-0.584735	4.54988884	-5.8340049	9.78E-09	1.34E-07	8.28964951	8.009705072	up-regulated in High
AURKAIP1	0.29088	5.44399176	5.13125598	4.14E-07	4.24E-06	4.65345346	6.383098283	up-regulated in Low
CCNL2	-0.2662123	4.11295518	-3.0414042	0.00247953	0.01053809	-3.6013622	2.605630908	up-regulated in High
ANKRD65	-0.8637943	3.33985888	-7.3069877	1.10E-12	2.76E-11	17.1949337	11.95844348	up-regulated in High
VWA1	-0.4498658	4.94544923	-6.0847505	2.34E-09	3.59E-08	9.68565598	8.631399907	up-regulated in High
ATAD3C	-0.5783439	1.83400903	-7.3312483	9.35E-13	2.37E-11	17.355367	12.02928666	up-regulated in High
ATAD3B	0.27283106	2.14946235	4.14513099	3.99E-05	0.00027269	0.26647482	4.398529362	up-regulated in Low
ATAD3A	0.40713418	3.26416138	7.29551628	1.19E-12	2.97E-11	17.1192236	11.92500909	up-regulated in Low
C1orf233	-0.6335026	2.6635951	-6.4038485	3.52E-10	6.14E-09	11.5349992	9.453162023	up-regulated in High
MIB2	-0.1816705	2.11491112	-3.2001777	0.00146149	0.00665824	-3.1148139	2.835204229	up-regulated in High
SLC35E2B	-0.2258865	2.80452737	-3.923549	9.96E-05	0.00062168	-0.6000862	4.001843513	up-regulated in High
SLC35E2	-0.1152707	0.79239823	-3.5255101	0.00046189	0.00241523	-2.0439045	3.335460718	up-regulated in High
GNB1	0.12621839	6.02144232	3.41790243	0.00068303	0.00340186	-2.4090676	3.165560666	up-regulated in Low
TMEM52	0.15664216	1.26735304	2.2902287	0.0224273	0.06821541	-5.5774715	1.649222923	up-regulated in Low
CFAP74	-0.116564	0.27196648	-2.7747962	0.00573227	0.02173846	-4.3646008	2.241673521	up-regulated in High
GABRD	-0.1401541	0.93867298	-2.7361369	0.00643963	0.02400055	-4.469649	2.191139352	up-regulated in High
PRKCZ	-0.4398328	2.55906426	-6.054084	2.79E-09	4.22E-08	9.51219828	8.554221608	up-regulated in High
SKI	-0.3627301	4.11951274	-6.7509641	4.12E-11	8.33E-10	13.6372109	10.38518305	up-regulated in High
PEX10	0.11633497	2.53462559	3.08232256	0.0021683	0.00937644	-3.4782486	2.663880115	up-regulated in Low
PLCH2	-0.2925901	0.63160356	-5.2648027	2.09E-07	2.27E-06	5.31253935	6.678865749	up-regulated in High
TNFRSF14	-0.4881323	3.40846273	-6.8067881	2.89E-11	5.98E-10	13.9839334	10.53871593	up-regulated in High
FAM213B	-0.2878519	3.52110965	-4.4745746	9.51E-06	7.40E-05	1.63664966	5.021999832	up-regulated in High
MMEL1	-0.1709489	0.48999362	-4.063401	5.62E-05	0.00037198	-0.0583392	4.250082078	up-regulated in High
TTC34	-0.1339282	0.21125024	-6.5611994	1.35E-10	2.52E-09	12.4764099	9.870791384	up-regulated in High
PRDM16	-0.7722168	1.14431621	-10.137462	4.50E-22	3.75E-20	38.5601511	21.34649134	up-regulated in High
MEGF6	-0.9618214	2.6352606	-11.583263	1.27E-27	1.75E-25	51.2490215	26.89709507	up-regulated in High
TPRG1L	-0.3745796	4.838907	-8.4637617	2.94E-16	1.21E-14	25.3049203	15.53127573	up-regulated in High
SMIM1	-0.5078314	2.07440127	-6.4413929	2.80E-10	4.99E-09	11.7578697	9.552071238	up-regulated in High
C1orf174	0.15063746	2.67710575	4.23090962	2.77E-05	0.00019582	0.61386443	4.556999919	up-regulated in Low
KCNAB2	-0.290311	2.17779416	-4.6144293	5.03E-06	4.14E-05	2.24753685	5.298753581	up-regulated in High
ACOT7	0.44556595	3.03386725	7.64456286	1.10E-13	3.17E-12	19.4653729	12.96030662	up-regulated in Low
ZBTB48	-0.161816	2.56791767	-3.4686592	0.00056865	0.00289441	-2.2381695	3.245154342	up-regulated in High
DNAJC11	0.24200277	2.8427447	6.32146843	5.78E-10	9.74E-09	11.0498489	9.2377658	up-regulated in Low
CAMTA1	-0.1336604	2.01015372	-3.7819246	0.00017462	0.00102383	-1.1305106	3.757916123	up-regulated in High
VAMP3	-0.1955464	5.35738589	-5.2063437	2.83E-07	2.99E-06	5.02216196	6.54861648	up-regulated in High
PER3	-0.7386303	1.94594613	-10.572921	1.07E-23	1.04E-21	42.2711089	22.97106471	up-regulated in High
PARK7	0.12994108	6.18926363	3.41812743	0.00068248	0.00339941	-2.4083153	3.165911354	up-regulated in Low
RERE	-0.2074363	3.55848781	-3.7444917	0.00020198	0.00116644	-1.2676342	3.69470095	up-regulated in High
ENO1	0.35790264	8.86530222	6.17495689	1.38E-09	2.19E-08	10.2002532	8.860257403	up-regulated in Low
GPR157	0.21210159	2.46311687	3.28473285	0.00109303	0.00514474	-2.8460099	2.961367363	up-regulated in Low
H6PD	-0.2454554	3.84655464	-4.0444487	6.08E-05	0.00039836	-0.1327961	4.216014072	up-regulated in High
SPSB1	-0.2800999	4.19221997	-4.3730041	1.49E-05	0.00011186	1.2038632	4.825505499	up-regulated in High
SLC25A33	0.21959476	1.83100365	4.8540248	1.62E-06	1.48E-05	3.33405789	5.789454194	up-regulated in Low
TMEM201	0.24781348	1.83032476	5.94331559	5.27E-09	7.57E-08	8.89197075	8.278100839	up-regulated in Low
PIK3CD	-0.3611344	2.53480102	-5.6511538	2.69E-08	3.42E-07	7.30395044	7.569904317	up-regulated in High
CTNNBIP1	-0.4265943	4.03793418	-7.4251013	4.95E-13	1.30E-11	17.9800247	12.30504454	up-regulated in High
LZIC	0.14364767	3.1874142	3.49197321	0.00052235	0.00268684	-2.1588668	3.28204043	up-regulated in Low
PGD	0.71236945	6.20631229	8.19501665	2.15E-15	7.93E-14	23.3391254	14.66661092	up-regulated in Low
APITD1	0.15854833	1.72131546	3.47493616	0.00055582	0.00283752	-2.2168681	3.255065189	up-regulated in Low
DFFA	0.13437224	3.22525906	3.74273337	0.00020336	0.00117318	-1.2740436	3.691744483	up-regulated in Low
CASZ1	-0.3016543	1.33531489	-6.3423642	5.10E-10	8.68E-09	11.1724025	9.292188548	up-regulated in High
C1orf127	-0.1341192	0.433177	-3.2352543	0.00129655	0.00598809	-3.0041194	2.88721236	up-regulated in High
TARDBP	0.17125227	4.18737087	5.55869713	4.45E-08	5.47E-07	6.81604103	7.351923338	up-regulated in Low
TARDBP	0.17125227	4.18737087	5.55869713	4.45E-08	5.47E-07	6.81604103	7.351923338	up-regulated in Low
MASP2	-0.1036808	0.32881911	-4.9849427	8.58E-07	8.28E-06	3.94886477	6.066355181	up-regulated in High
SRM	0.47321914	5.10472654	9.05447046	3.15E-18	1.66E-16	29.7899129	17.50151351	up-regulated in Low

EXOSC10	0.19209612	3.6572408	5.65849234	2.59E-08	3.29E-07	7.34298034	7.58733306	up-regulated in Low
MTOR	0.11318288	2.79697391	2.59608277	0.00970922	0.03400872	-4.8382252	2.012815883	up-regulated in Low
FBXO2	-0.5332436	2.84518578	-4.4901761	8.86E-06	6.93E-05	1.70393944	5.052517964	up-regulated in High
FBXO44	-0.2795035	2.69000818	-4.8414668	1.72E-06	1.57E-05	3.27586508	5.763218007	up-regulated in High
MAD2L2	0.38688965	3.26117271	6.61336298	9.75E-11	1.86E-09	12.7927376	10.01102662	up-regulated in Low
MTHFR	-0.215378	2.35439204	-3.9599645	8.59E-05	0.00054373	-0.4607366	4.065777945	up-regulated in High
CLCN6	-0.2252948	2.00862841	-4.654312	4.18E-06	3.50E-05	2.42490771	5.378986539	up-regulated in High
KIAA2013	0.11038425	4.52130853	3.00834153	0.00276039	0.01154934	-3.6996818	2.559029181	up-regulated in Low
PLOD1	0.21714653	5.08590975	3.80694957	0.00015832	0.00093845	-1.0381214	3.800470927	up-regulated in Low
TNFRSF1B	-0.3683402	4.07141041	-5.0984676	4.88E-07	4.94E-06	4.4939606	6.311451698	up-regulated in High
VPS13D	-0.3123774	2.14526006	-6.7552569	4.01E-11	8.11E-10	13.6637895	10.39695412	up-regulated in High
DHRS3	-0.4827534	4.76364656	-5.6481208	2.74E-08	3.47E-07	7.28783215	7.562706361	up-regulated in High
C1orf158	-0.2064171	0.2340794	-4.9067529	1.26E-06	1.18E-05	3.57989075	5.900235398	up-regulated in High
PRDM2	-0.2921362	2.52730945	-7.2317826	1.82E-12	4.44E-11	16.7003362	11.73999117	up-regulated in High
KAZN	-0.2126314	0.78098315	-4.9836902	8.64E-07	8.33E-06	3.94291289	6.063676937	up-regulated in High
FHAD1	-0.192302	0.50113839	-4.607548	5.19E-06	4.26E-05	2.21707515	5.284969009	up-regulated in High
EFHD2	0.20619016	5.43339015	2.88162775	0.00412789	0.01638557	-4.0668882	2.384271577	up-regulated in Low
AGMAT	0.5539921	1.18550866	10.425842	3.82E-23	3.56E-21	41.0065009	22.41758074	up-regulated in Low
PLEKHM2	-0.1050383	4.36832436	-2.4637324	0.01408846	0.0464631	-5.1692009	1.851136561	up-regulated in High
SLC25A34	-0.1097132	0.41783165	-4.0993537	4.84E-05	0.00032514	0.08380067	4.315077197	up-regulated in High
UQCRHL	0.22464641	1.02181791	6.375811	4.17E-10	7.20E-09	11.3692833	9.379601476	up-regulated in Low
HSPB7	-0.5305897	1.04985646	-9.9586595	2.03E-21	1.58E-19	37.06598	20.69202409	up-regulated in High
CLCNKA	-0.1009547	0.32143511	-2.3401145	0.01967377	0.06125378	-5.4630702	1.706112497	up-regulated in High
CLCNKB	-0.1826607	0.49059693	-3.7089285	0.00023167	0.00131709	-1.3967148	3.635130997	up-regulated in High
FAM131C	0.15700193	0.23664221	5.03762643	6.61E-07	6.51E-06	4.20045126	6.179523848	up-regulated in Low
EPHA2	-0.2848852	3.89585609	-2.6008484	0.0095772	0.03359472	-4.8259931	2.018761366	up-regulated in High
ARHGEF19	-0.3828456	3.36784601	-4.6752395	3.79E-06	3.19E-05	2.5185385	5.421318971	up-regulated in High
RSG1	-0.2199847	1.81518105	-4.8350479	1.78E-06	1.61E-05	3.2461733	5.749829628	up-regulated in High
SZRD1	0.10166022	4.87822641	3.01902764	0.00266658	0.01121549	-3.6680174	2.57404582	up-regulated in Low
NECAP2	-0.1642084	4.01460595	-4.053912	5.85E-05	0.00038509	-0.0956588	4.233008278	up-regulated in High
CROCC	-0.3210792	1.90591921	-5.5394082	4.93E-08	6.01E-07	6.71514481	7.306821321	up-regulated in High
SDHB	0.21604523	4.7677917	5.68959165	2.18E-08	2.81E-07	7.50887532	7.661399618	up-regulated in Low
PADI2	-0.3460333	1.5384334	-4.058289	5.74E-05	0.00037926	-0.0784545	4.240879747	up-regulated in High
RCC2	0.40777309	5.38647415	7.60481648	1.44E-13	4.09E-12	19.1938207	12.84055663	up-regulated in Low
ARHGEF10L	-0.2178928	2.64960458	-3.7244318	0.00021825	0.00124957	-1.3405869	3.661041291	up-regulated in High
ACTL8	0.22039223	0.19389454	3.90176659	0.00010868	0.00067143	-0.6828616	3.96383745	up-regulated in Low
IGSF21	-0.1975568	0.63417892	-4.2412492	2.65E-05	0.00018802	0.65618531	4.576285925	up-regulated in High
KLHDC7A	-0.681554	1.50188556	-8.1412805	3.19E-15	1.14E-13	22.9518347	14.49616902	up-regulated in High
PAX7	-0.3134823	0.57704306	-2.7279995	0.0065982	0.02448512	-4.4915783	2.180574744	up-regulated in High
ALDH4A1	-0.1582328	2.18067615	-2.9017355	0.00387618	0.0155204	-4.0096365	2.411596056	up-regulated in High
IFFO2	-0.4018948	2.33992013	-6.0839198	2.35E-09	3.61E-08	9.68094765	8.629305227	up-regulated in High
EMC1	0.28066642	3.15060689	6.23916205	9.44E-10	1.54E-08	10.5704738	9.024810324	up-regulated in Low
MRTO4	0.43885511	4.11521762	9.87936988	3.94E-21	2.99E-19	36.4090568	20.40421313	up-regulated in Low
AKR7A3	0.436337	1.73404226	2.9005136	0.00389107	0.01557254	-4.0131265	2.409931235	up-regulated in Low
NBL1	-0.4026708	4.12099002	-4.4162755	1.23E-05	9.39E-05	1.38711754	4.908752478	up-regulated in High
TMCO4	-0.1455519	3.27902012	-3.0531417	0.0023863	0.01019232	-3.5662089	2.622274741	up-regulated in High
RNF186	0.37278883	0.57600927	5.05697656	6.01E-07	5.97E-06	4.29345549	6.221339079	up-regulated in Low
OTUD3	-0.2212313	1.66024438	-3.9233924	9.96E-05	0.00062201	-0.6006826	4.001569747	up-regulated in High
PLA2G2A	-0.4207926	1.35060199	-3.2417757	0.00126785	0.00586733	-2.9834116	2.896933106	up-regulated in High
PLA2G5	-0.2679776	0.81833443	-7.0713134	5.25E-12	1.21E-10	15.6588727	11.27973763	up-regulated in High
UBXN10	-0.5800926	2.12698409	-6.0363725	3.09E-09	4.64E-08	9.41236176	8.509791809	up-regulated in High
CDA	0.63316612	3.06252396	3.7546952	0.00019414	0.00112588	-1.2303846	3.711880028	up-regulated in Low
PINK1	-0.2826714	2.62735908	-6.1189304	1.92E-09	2.98E-08	9.87987542	8.717794018	up-regulated in High
DDOST	0.20538252	6.82197039	5.12494039	4.27E-07	4.37E-06	4.62266097	6.36926814	up-regulated in Low
SH2D5	0.21975744	0.1481138	8.33514041	7.68E-16	2.99E-14	24.3581241	15.11491537	up-regulated in Low
NBPF3	-0.1982524	1.08569274	-5.2154408	2.70E-07	2.87E-06	5.06715839	6.568805642	up-regulated in High
ALPL	-1.5244342	4.26824913	-9.3158199	3.96E-19	2.32E-17	31.8434925	18.40264695	up-regulated in High
RAP1GAP	-1.0246675	3.61058422	-8.3653903	6.13E-16	2.42E-14	24.5798177	15.21242174	up-regulated in High
LDLRAD2	-0.2504274	0.59999383	-8.7096967	4.57E-17	2.07E-15	27.1452602	16.340121	up-regulated in High
HSPG2	-0.5093946	4.00756264	-5.2206084	2.63E-07	2.80E-06	5.09274987	6.580287142	up-regulated in High
WNT4	-0.3620996	1.13100161	-4.3998471	1.33E-05	0.00010021	1.31734676	4.87706575	up-regulated in High
C1QA	-0.2734656	6.92206032	-2.3605218	0.01863564	0.05858078	-5.4155759	1.729655596	up-regulated in High
C1QC	-0.2628672	6.85899155	-2.2530798	0.02469062	0.07397051	-5.661096	1.607468004	up-regulated in High
C1QB	-0.3000326	6.81640338	-2.4724931	0.01375202	0.04555424	-5.1478143	1.861633613	up-regulated in High
KDM1A	0.37889064	4.11602899	9.06187535	2.97E-18	1.57E-16	29.847525	17.52680215	up-regulated in Low
HTR1D	0.45130659	0.83842266	5.75219729	1.54E-08	2.05E-07	7.84525307	7.81151494	up-regulated in Low
HNRNPR	0.31758645	4.05981676	8.8536174	1.51E-17	7.24E-16	28.2401714	16.82107782	up-regulated in Low
ZNF436	-0.1042722	2.39427217	-2.4928404	0.01299793	0.04351549	-5.0978569	1.886125765	up-regulated in High
TCEA3	-0.8227552	4.02025475	-8.1545999	2.90E-15	1.04E-13	23.0476495	14.53833886	up-regulated in High
ASAP3	-0.5478268	2.74371375	-7.8752503	2.16E-14	6.87E-13	21.0633676	13.66461327	up-regulated in High
E2F2	0.64006087	1.14473422	12.987	2.13E-33	4.25E-31	64.4748382	32.67254041	up-regulated in Low

RPL11	-0.1050975	8.42731057	-2.1952013	0.02861218	0.0833373	-5.7887132	1.543448986	up-regulated in High
TCEB3	-0.1262858	3.88399152	-3.0047031	0.00279302	0.01167026	-3.7104384	2.55392613	up-regulated in High
HMGCL	-0.1673551	3.23133652	-3.977771	7.99E-05	0.00051079	-0.3921576	4.097221053	up-regulated in High
FUCA1	-0.7468419	5.74322228	-11.652304	6.72E-28	9.56E-26	51.8800855	27.17286531	up-regulated in High
CNR2	-0.164046	0.26115853	-5.7019912	2.04E-08	2.64E-07	7.57524163	7.691023858	up-regulated in High
PNRC2	-0.1579024	4.65280359	-4.1138812	4.56E-05	0.00030784	0.14156749	4.341476482	up-regulated in High
IL22RA1	0.25744552	0.97284601	3.20019704	0.00146139	0.00665824	-3.1147531	2.835232811	up-regulated in Low
IFNLR1	-0.2511959	1.44538844	-4.3790746	1.45E-05	0.0001091	1.22947081	4.837142407	up-regulated in High
NIPAL3	-0.4895302	2.55735246	-9.8299019	5.95E-21	4.39E-19	36.0009936	20.22541033	up-regulated in High
NCMAP	-0.52296	1.88685403	-5.7974294	1.20E-08	1.62E-07	8.09028634	7.920811871	up-regulated in High
RUNX3	-0.2797425	2.31042852	-3.6426338	0.00029829	0.00164481	-1.6342257	3.525355033	up-regulated in High
SYF2	-0.1275432	4.03271246	-4.3044973	2.02E-05	0.00014686	0.91715441	4.695121449	up-regulated in High
RSRP1	-0.3920734	2.91803618	-4.8414604	1.73E-06	1.57E-05	3.27583527	5.763204565	up-regulated in High
TMEM50A	-0.2074181	5.45790798	-5.9525493	5.00E-09	7.23E-08	8.94329448	8.300959334	up-regulated in High
RHCE	-0.1644495	0.66158063	-3.2662545	0.00116529	0.00544871	-2.9053267	2.933564718	up-regulated in High
TMEM57	-0.253682	3.64629491	-6.082282	2.37E-09	3.64E-08	9.67166568	8.625175748	up-regulated in High
LDLRAP1	-0.3951015	3.14030213	-7.7517643	5.18E-14	1.56E-12	20.2033395	13.28564038	up-regulated in High
MAN1C1	-0.4633812	1.36872024	-8.8162586	2.02E-17	9.55E-16	27.9546972	16.69569703	up-regulated in High
SEPN1	-0.2888214	5.27492801	-5.4569157	7.67E-08	9.02E-07	6.28714687	7.115400455	up-regulated in High
MTFR1L	-0.1460531	3.05072849	-4.2821774	2.22E-05	0.00016038	0.82465026	4.653016085	up-regulated in High
AUNIP	0.82198676	1.02944639	18.6227913	4.53E-59	4.64E-56	123.39887	58.3442316	up-regulated in Low
STMN1	0.90574117	4.41586816	10.7052755	3.36E-24	3.46E-22	43.4186519	23.47320134	up-regulated in Low
PAFAH2	-0.1435219	3.07872045	-3.2586464	0.00119631	0.00557678	-2.9296561	2.922155111	up-regulated in High
PDIK1L	-0.2471411	2.45737463	-5.1859415	3.14E-07	3.30E-06	4.92150413	6.503444916	up-regulated in High
FAM110D	-0.2487988	0.84263911	-5.9964681	3.89E-09	5.74E-08	9.18835299	8.410079144	up-regulated in High
CNKSR1	-0.2841941	3.34306654	-4.7082283	3.25E-06	2.77E-05	2.66691222	5.488372756	up-regulated in High
CEP85	0.32840667	2.3445477	6.6609708	7.25E-11	1.41E-09	13.0832692	10.13978605	up-regulated in Low
UBXN11	-0.1693142	2.61097229	-2.4744195	0.013679	0.04536638	-5.1431016	1.863945718	up-regulated in High
CD52	-0.7974098	5.08052076	-7.4803736	3.40E-13	9.12E-12	18.3508731	12.46870254	up-regulated in High
AIM1L	0.28554421	0.99771856	3.65612635	0.00028342	0.00157109	-1.5862153	3.54756298	up-regulated in Low
LIN28A	0.1605511	0.07296205	4.07046498	5.46E-05	0.00036238	-0.030504	4.262814333	up-regulated in Low
HMGN2	0.23821293	6.13503795	4.73350848	2.88E-06	2.49E-05	2.78126063	5.540025828	up-regulated in Low
RPS6KA1	-0.4243307	4.03995049	-9.4585	1.25E-19	7.88E-18	32.9819208	18.90196665	up-regulated in High
PIGV	-0.1186764	2.76265858	-2.8444874	0.00463228	0.01808525	-4.1716235	2.334205252	up-regulated in High
NUDC	0.17543133	5.37738902	4.12242083	4.40E-05	0.00029787	0.17561329	4.357031263	up-regulated in Low
NR0B2	-0.5988252	1.4298542	-4.2594209	2.45E-05	0.00017521	0.73079634	4.610276747	up-regulated in High
SLC9A1	-0.290237	3.59911312	-4.6247752	4.79E-06	3.96E-05	2.29341398	5.319510978	up-regulated in High
WDTC1	-0.371717	4.02269178	-8.3972822	4.84E-16	1.95E-14	24.814198	15.31549796	up-regulated in High
TMEM222	-0.1030418	3.35544855	-2.746499	0.00624268	0.02338733	-4.4416323	2.204628739	up-regulated in High
SYTL1	-0.5614996	3.09673678	-6.9805169	9.49E-12	2.10E-10	15.0780466	11.02288942	up-regulated in High
MAP3K6	-0.4592933	3.45120335	-7.4302599	4.78E-13	1.26E-11	18.014543	12.3202794	up-regulated in High
FCN3	-0.6152725	1.93752669	-5.4992902	6.12E-08	7.34E-07	6.50628993	7.213432018	up-regulated in High
CD164L2	-0.5772636	1.3210734	-7.126977	3.64E-12	8.57E-11	16.0179825	11.43848045	up-regulated in High
GPR3	0.12561432	0.80285484	2.92547917	0.003597	0.01455529	-3.9415369	2.44405919	up-regulated in Low
AHDC1	-0.3651323	2.80335289	-5.7523281	1.54E-08	2.05E-07	7.84595907	7.811829917	up-regulated in High
FGR	-0.4809278	2.65877949	-6.1101931	2.02E-09	3.13E-08	9.83013889	8.695672033	up-regulated in High
FAM76A	-0.2448314	2.17939515	-7.262176	1.49E-12	3.67E-11	16.8997255	11.82806622	up-regulated in High
STX12	-0.2954516	4.14066483	-8.7884909	2.50E-17	1.17E-15	27.7430827	16.60274734	up-regulated in High
PPP1R8	0.16207852	4.04949688	5.14337465	3.89E-07	4.00E-06	4.71263508	6.409676055	up-regulated in Low
THEMIS2	-0.2732973	3.37731488	-3.7454369	0.00020124	0.00116241	-1.2641877	3.696290627	up-regulated in High
SMPDL3B	-0.5112584	4.39822695	-5.0831748	5.27E-07	5.30E-06	4.41988672	6.278166424	up-regulated in High
XKR8	-0.1953997	2.63034229	-4.8628125	1.56E-06	1.43E-05	3.37486103	5.807847395	up-regulated in High
PTAFR	-0.262785	2.87837563	-2.9124774	0.00374752	0.0150939	-3.9788938	2.42625625	up-regulated in High
SESN2	-0.1281248	2.93801504	-2.3192133	0.02078918	0.0641244	-5.5112958	1.682162569	up-regulated in High
RCC1	0.39310423	4.12242145	6.94187307	1.22E-11	2.65E-10	14.8327104	10.91436175	up-regulated in Low
TRNAU1AP	-0.2043806	2.78406609	-3.8913622	0.00011331	0.0006966	-0.7222463	3.945746559	up-regulated in High
EPB41	-0.1537634	3.11102423	-2.7687496	0.00583805	0.02208112	-4.3811254	2.233732178	up-regulated in High
PTPRU	-0.7101915	3.47731717	-7.1663182	2.81E-12	6.70E-11	16.2731717	11.55125875	up-regulated in High
LAPTM5	-0.4184137	6.33097728	-4.4925326	8.77E-06	6.86E-05	1.71412189	5.057135296	up-regulated in High
SDC3	-0.3551899	4.06821116	-4.9585037	9.77E-07	9.34E-06	3.82350926	6.009937629	up-regulated in High
NKAIN1	0.3574018	0.30641559	7.0149155	7.59E-12	1.71E-10	15.2973715	11.1198918	up-regulated in Low
SNRNP40	0.23137782	3.4857582	5.74355608	1.62E-08	2.14E-07	7.79863248	7.790714752	up-regulated in Low
FABP3	-0.9585694	3.42965607	-7.1792648	2.58E-12	6.17E-11	16.3574005	11.58847801	up-regulated in High
SERINC2	-0.2834639	5.65116492	-3.2490883	0.00123637	0.00574058	-2.9601442	2.907852307	up-regulated in High
AC114494.1	-0.1042346	0.51802948	-3.0213101	0.00264692	0.01114406	-3.6612401	2.577258882	up-regulated in High
PEF1	-0.1154061	5.10393946	-3.1620871	0.00166243	0.00743903	-3.2337079	2.779256069	up-regulated in High
COL16A1	-0.3760302	2.59750492	-4.6349382	4.57E-06	3.79E-05	2.33857143	5.339939279	up-regulated in High
BAI2	0.11384354	0.87483451	1.99148686	0.04697587	0.12423135	-6.2119414	1.328125216	up-regulated in Low
SPOCD1	0.16327208	1.0183368	2.37377095	0.01798768	0.05689618	-5.3845249	1.745024889	up-regulated in Low
KHDRBS1	0.25305556	5.59894974	8.66920775	6.23E-17	2.77E-15	26.8396029	16.20582183	up-regulated in Low
DCDC2B	-0.2929846	0.43942176	-5.8221466	1.05E-08	1.43E-07	8.22489378	7.980834572	up-regulated in High

EIF3I	0.23724829	6.86626156	6.10104049	2.13E-09	3.29E-08	9.77810339	8.672525901	up-regulated in Low
FAM167B	-0.1443518	2.39849573	-2.4054597	0.01651759	0.05301299	-5.3095699	1.782053394	up-regulated in High
HDAC1	0.24086693	5.10798567	5.50086032	6.07E-08	7.28E-07	6.51443865	7.217076437	up-regulated in Low
MARCKSL1	0.39918039	6.79466828	4.83807798	1.75E-06	1.59E-05	3.26018489	5.756147779	up-regulated in Low
FAM229A	-0.1803918	0.8935281	-4.0692942	5.49E-05	0.00036378	-0.0351203	4.260702901	up-regulated in High
BSDC1	-0.1636431	4.01461067	-4.7145053	3.15E-06	2.70E-05	2.69525264	5.501176507	up-regulated in High
RBBP4	0.13845979	4.31505196	3.45538185	0.00059671	0.00301984	-2.2831062	3.22423953	up-regulated in Low
SYNC	-0.231127	0.6965294	-6.9412344	1.22E-11	2.66E-10	14.8286649	10.91257195	up-regulated in High
YARS	0.23860129	3.99471227	6.5070091	1.88E-10	3.43E-09	12.1500212	9.726047133	up-regulated in Low
FNDC5	-0.3590937	0.81323195	-5.4734923	7.02E-08	8.33E-07	6.37269522	7.153674879	up-regulated in High
RNF19B	-0.191978	4.54907132	-3.7203341	0.00022173	0.00126734	-1.3554438	3.654184065	up-regulated in High
FKSG48	-0.1077227	0.52577228	-2.0975522	0.03645069	0.10137886	-5.9966339	1.438294261	up-regulated in High
AZIN2	-0.299307	1.18842182	-6.4047554	3.50E-10	6.11E-09	11.54037	9.455545848	up-regulated in High
TRIM62	-0.1050008	1.84693493	-2.6748045	0.00772446	0.02795389	-4.6333709	2.112131931	up-regulated in High
ZNF362	-0.2961043	3.65440116	-5.5686356	4.21E-08	5.20E-07	6.86814758	7.375212267	up-regulated in High
PHC2	-0.1118597	5.06139851	-2.5404852	0.01137393	0.03883333	-4.9793161	1.94408941	up-regulated in High
SMIM12	0.13199688	2.3364006	4.00677426	7.10E-05	0.00045918	-0.2798378	4.148689393	up-regulated in Low
GJB3	0.6133643	1.77764266	4.36470662	1.55E-05	0.00011568	1.16891404	4.809621305	up-regulated in Low
GJA4	-0.3198832	2.48187696	-4.7141265	3.16E-06	2.71E-05	2.69354129	5.500403385	up-regulated in High
ZMYM6NB	-0.2644706	2.91735347	-4.9467822	1.04E-06	9.84E-06	3.76812696	5.985005723	up-regulated in High
ZMYM6	-0.1439538	1.34415482	-6.0526396	2.81E-09	4.25E-08	9.50404696	8.550594297	up-regulated in High
SFPQ	0.17271947	5.2217239	4.35222516	1.64E-05	0.00012157	1.11645779	4.785775438	up-regulated in Low
KIAA0319L	-0.5290272	3.94434688	-10.457454	2.91E-23	2.74E-21	41.2773561	22.53613772	up-regulated in High
NCDN	-0.190116	3.15724041	-4.0485687	5.98E-05	0.00039261	-0.1166377	4.223408691	up-regulated in High
TFAP2E	-0.2387771	1.02055727	-3.9460566	9.09E-05	0.00057222	-0.5141	4.041301432	up-regulated in High
PSMB2	0.35037896	4.78637357	9.00449811	4.66E-18	2.40E-16	29.4019975	17.33122681	up-regulated in Low
CLSPN	0.76423583	1.15023124	13.6303475	3.74E-36	8.92E-34	70.7881012	35.42688131	up-regulated in Low
AGO4	-0.2806905	2.52775564	-6.3732475	4.24E-10	7.30E-09	11.3541622	9.372888602	up-regulated in High
AGO3	-0.1067732	1.13038222	-3.6603868	0.00027888	0.00154896	-1.5710207	3.554589583	up-regulated in High
TEKT2	-0.4593936	1.12930483	-4.7520245	2.64E-06	2.30E-05	2.86536789	5.578005493	up-regulated in High
COL8A2	-0.7091067	2.54786791	-7.2834068	1.29E-12	3.21E-11	17.0394059	11.8897588	up-regulated in High
STK40	-0.1382782	3.86842958	-2.7828886	0.00559341	0.02129376	-4.3424306	2.252323475	up-regulated in High
OSCP1	-0.3040927	2.09815193	-5.571048	4.16E-08	5.14E-07	6.8808079	7.380870421	up-regulated in High
MRPS15	0.37851119	4.5757408	7.08806434	4.71E-12	1.09E-10	15.7666986	11.32740626	up-regulated in Low
CSF3R	-0.5244521	2.5248504	-4.5897237	5.63E-06	4.59E-05	2.13836571	5.249343924	up-regulated in High
MEAF6	-0.210967	4.3922175	-4.7743194	2.38E-06	2.09E-05	2.96703794	5.623901419	up-regulated in High
DNALI1	-0.7659333	2.61514544	-7.7697434	4.57E-14	1.39E-12	20.3278953	13.34053735	up-regulated in High
GNL2	0.18027264	3.68649105	4.59990052	5.37E-06	4.40E-05	2.18327077	5.269669942	up-regulated in Low
RSPO1	-0.2305798	0.26272951	-6.9953873	8.61E-12	1.92E-10	15.1727517	11.0647775	up-regulated in High
C1orf109	0.19072896	2.27297388	5.25992209	2.15E-07	2.32E-06	5.28818518	6.667945093	up-regulated in Low
CDCA8	1.259281	2.93515807	16.7532583	3.04E-50	1.61E-47	103.122962	49.5175125	up-regulated in Low
EPHA10	-0.3278664	1.22422893	-5.1668324	3.46E-07	3.61E-06	4.8275473	6.461270377	up-regulated in High
MANEAL	-0.2034938	2.68798604	-2.263157	0.02405784	0.07236439	-5.6385438	1.618743333	up-regulated in High
YRDC	0.25773433	3.68272082	6.02676061	3.27E-09	4.88E-08	9.35828657	8.485724344	up-regulated in Low
C1orf122	0.17772934	4.08074795	3.42200156	0.00067305	0.00335789	-2.395355	3.171952509	up-regulated in Low
INPP5B	-0.3840393	1.97251126	-7.8276214	3.03E-14	9.43E-13	20.7303956	13.5179099	up-regulated in High
SF3A3	0.17294736	4.17974001	5.08897267	5.12E-07	5.16E-06	4.44794628	6.29077582	up-regulated in Low
UTP11L	0.20821277	3.41323034	6.21067675	1.12E-09	1.80E-08	10.4058171	8.9516346	up-regulated in Low
RHBDL2	-0.1698571	2.09628782	-2.1688523	0.03056878	0.08801751	-5.8457317	1.514721951	up-regulated in High
AKIRIN1	0.13603553	4.73603323	2.95946231	0.00322928	0.01324351	-3.843137	2.490894673	up-regulated in Low
NDUFS5	0.14945642	7.63514781	2.75941125	0.00600489	0.02261547	-4.4065773	2.221494828	up-regulated in Low
MACF1	-0.3758486	2.86522431	-6.0344064	3.13E-09	4.69E-08	9.40129435	8.504866143	up-regulated in High
HEYL	-0.4069607	2.6142748	-6.3573203	4.66E-10	7.97E-09	11.2603315	9.33123058	up-regulated in High
NT5C1A	-0.2362359	0.25128022	-5.9039161	6.59E-09	9.31E-08	8.67375317	8.180891545	up-regulated in High
HPCAL4	-0.2939343	0.45389606	-4.8335388	1.79E-06	1.62E-05	3.23919795	5.746684178	up-regulated in High
TRIT1	0.21057539	3.25376317	4.29471649	2.11E-05	0.00015256	0.87656335	4.676647845	up-regulated in Low
MFSD2A	-0.9009783	3.23992357	-9.0944651	2.30E-18	1.24E-16	30.1014821	17.63827039	up-regulated in High
PPT1	-0.1547125	6.00830079	-2.7553215	0.00607931	0.02286034	-4.4176976	2.216145949	up-regulated in High
ZMPSTE24	0.1190883	4.6367123	2.67198827	0.00778864	0.0281408	-4.640802	2.108538449	up-regulated in Low
COL9A2	-0.6847248	2.55312139	-5.7387469	1.66E-08	2.19E-07	7.77271281	7.779149749	up-regulated in High
SMAP2	-0.4004751	4.36431273	-7.4408165	4.45E-13	1.18E-11	18.0852421	12.35148178	up-regulated in High
ZFP69B	0.19741041	1.00227294	5.08037996	5.34E-07	5.36E-06	4.40637082	6.27209232	up-regulated in Low
RIMS3	-0.332889	1.10471142	-6.2952472	6.77E-10	1.12E-08	10.8965482	9.16967781	up-regulated in High
CITED4	0.22592408	2.35008815	2.30737937	0.02144492	0.06581018	-5.5384126	1.668675585	up-regulated in Low
CTPS1	0.62397402	2.85628253	11.3510115	1.06E-26	1.33E-24	49.1420541	25.97619718	up-regulated in Low
SCMH1	-0.1295506	3.28061361	-2.8628244	0.00437665	0.01722679	-4.1200776	2.358858641	up-regulated in High
HIVEP3	-0.1078784	1.05821076	-2.5839028	0.01005404	0.03497449	-4.8693887	1.997659551	up-regulated in High
GUCA2B	0.2978157	0.21194362	4.23593985	2.71E-05	0.00019194	0.63444166	4.566377668	up-regulated in Low
ZMYND12	-0.3594988	1.30579159	-6.3033071	6.45E-10	1.08E-08	10.9436119	9.190582342	up-regulated in High
CCDC30	-0.1237046	0.38895273	-5.4457971	8.13E-08	9.53E-07	6.22989451	7.089781935	up-regulated in High
PPIH	0.39511238	4.09997263	8.09379686	4.51E-15	1.57E-13	22.6112276	14.34624607	up-regulated in Low

YBX1	0.37166394	7.49377052	8.42396119	3.96E-16	1.61E-14	25.0107824	15.40194432	up-regulated in Low
P3H1	0.10921827	2.85815814	2.13876473	0.03294292	0.09339798	-5.910014	1.482237958	up-regulated in Low
ERMAP	-0.3380929	2.09263846	-8.0537112	6.02E-15	2.06E-13	22.3248762	14.22018537	up-regulated in High
ZNF691	-0.1156899	2.75826204	-2.9322357	0.00352098	0.01429224	-3.9220604	2.453336125	up-regulated in High
SLC2A1	1.2949022	4.63428333	10.8032344	1.42E-24	1.50E-22	44.2737111	23.84728839	up-regulated in Low
FAM183A	-0.5093629	1.63906495	-4.3813185	1.44E-05	0.00010814	1.23894499	4.841447428	up-regulated in High
EBNA1BP2	0.35937587	4.15939622	8.97480231	5.88E-18	3.00E-16	29.1722116	17.23034542	up-regulated in Low
CFAP57	-0.4803562	0.70329874	-7.7781294	4.30E-14	1.31E-12	20.3860692	13.36617569	up-regulated in High
TMEM125	-1.0579477	5.21934468	-12.039786	1.83E-29	2.85E-27	55.4608477	28.73722065	up-regulated in High
C1orf210	-0.4149327	3.92833081	-5.5071196	5.87E-08	7.06E-07	6.54694456	7.231613729	up-regulated in High
TIE1	-0.3291457	2.166414	-5.2218364	2.61E-07	2.78E-06	5.09883432	6.583016798	up-regulated in High
CDC20	1.71008518	3.80055842	18.9212282	1.70E-60	2.25E-57	126.675281	59.77012596	up-regulated in Low
ELOVL1	-0.1435184	5.39962245	-3.522994	0.00046619	0.00243508	-2.0525657	3.331438175	up-regulated in High
SZT2	-0.2107648	1.91102491	-4.4369449	1.13E-05	8.63E-05	1.47524136	4.948760417	up-regulated in High
HYI	-0.2324664	2.29724689	-4.1532756	3.86E-05	0.00026459	0.29917434	4.413458703	up-regulated in High
ST3GAL3	-0.185103	1.97813789	-3.6659725	0.00027302	0.00152018	-1.5510739	3.56381237	up-regulated in High
ARTN	0.50430367	0.78424367	7.83266398	2.93E-14	9.13E-13	20.7655739	13.53341027	up-regulated in Low
DPH2	0.30708962	3.38713077	6.91295417	1.47E-11	3.16E-10	14.6498475	10.83345518	up-regulated in Low
ATP6V0B	0.15102839	5.24746139	2.60504347	0.00946232	0.03324768	-4.8152073	2.024002237	up-regulated in Low
B4GALT2	0.16605784	4.13568358	3.24862098	0.00123836	0.00574799	-2.9616327	2.907153871	up-regulated in Low
CCDC24	-0.1645049	2.41250621	-2.6895057	0.00739708	0.02691427	-4.5944555	2.130939754	up-regulated in High
ERI3	0.15537	4.31948874	3.81686178	0.00015227	0.00090701	-1.0013678	3.817391541	up-regulated in Low
TMEM53	-0.1365961	2.60726715	-3.2180269	0.00137527	0.00631423	-3.0586302	2.861610931	up-regulated in High
C1orf228	-0.120427	0.99674274	-2.2018679	0.02813464	0.08220061	-5.7741797	1.55075871	up-regulated in High
KIF2C	1.4593759	2.60767707	18.6593737	3.03E-59	3.16E-56	123.800017	58.51881642	up-regulated in Low
PLK3	-0.3013509	2.78170984	-5.6779689	2.32E-08	2.97E-07	7.44678219	7.633679677	up-regulated in High
TCTEX1D4	-0.3137963	0.56574969	-5.9359869	5.50E-09	7.87E-08	8.85128503	8.259979032	up-regulated in High
BTBD19	-0.1978616	1.28535179	-3.6273226	0.00031606	0.00172802	-1.6885033	3.500236923	up-regulated in High
EIF2B3	0.15811257	2.90401701	4.35565693	1.61E-05	0.00011994	1.1308667	4.792326101	up-regulated in Low
HECTD3	-0.1183342	3.66650682	-3.2287073	0.00132596	0.00610944	-3.0248683	2.87746966	up-regulated in High
ZSWIM5	-0.2141495	1.47079273	-3.6043548	0.00034457	0.00186473	-1.7695167	3.462723714	up-regulated in High
HPDL	0.6780972	0.93125528	9.47715824	1.08E-19	6.82E-18	33.1316829	18.96764114	up-regulated in Low
MUTYH	0.14245636	2.09409274	3.11866535	0.00192243	0.00844392	-3.3675745	2.716148844	up-regulated in Low
TOE1	0.13727785	2.31590601	3.80731348	0.00015809	0.0009373	-1.0367736	3.801091476	up-regulated in Low
TESK2	-0.1488941	1.4129309	-3.7928224	0.00016733	0.00098608	-1.0903478	3.776418921	up-regulated in High
CCDC163P	0.10648252	1.08336181	2.5043896	0.01258643	0.04229792	-5.0693232	1.900097335	up-regulated in Low
MMACHC	0.12565471	1.58380067	3.69753598	0.00024202	0.00136821	-1.4378186	3.616148711	up-regulated in Low
PRDX1	0.45825835	7.78891282	7.051105	5.99E-12	1.37E-10	15.5290679	11.22234691	up-regulated in Low
NASP	0.36626082	3.71671248	6.390596	3.82E-10	6.63E-09	11.4565934	9.418359783	up-regulated in Low
CCDC17	-0.4306214	1.04522633	-4.7917949	2.19E-06	1.94E-05	3.04703356	5.660002123	up-regulated in High
GPBP1L1	-0.1696456	4.27777871	-5.1960141	2.98E-07	3.14E-06	4.97115488	6.525727754	up-regulated in High
TMEM69	0.26920772	3.6123838	6.97147618	1.01E-11	2.22E-10	15.0205501	10.99745707	up-regulated in Low
MAST2	0.18271557	3.09784324	3.30611647	0.00101461	0.00482422	-2.7769671	2.993702865	up-regulated in Low
TSPAN1	-0.512664	4.91242151	-3.1422536	0.00177688	0.00788588	-3.2950742	2.750342082	up-regulated in High
LURAP1	-0.201025	0.47531035	-6.9454476	1.19E-11	2.59E-10	14.8553572	10.92438076	up-regulated in High
RAD54L	0.94017774	1.44506073	15.9720753	1.26E-46	5.52E-44	94.8180923	45.90051344	up-regulated in Low
UQCRH	0.42788974	6.13238747	7.92310714	1.54E-14	4.99E-13	21.3995125	13.81268801	up-regulated in Low
FAAH	-0.5214103	3.16803683	-7.386221	6.45E-13	1.67E-11	17.7204763	12.19048002	up-regulated in High
MKNK1	-0.1424049	2.42043244	-3.9146923	0.00010319	0.00064169	-0.6337947	3.986368929	up-regulated in High
MOB3C	-0.3085961	2.33696192	-7.7798823	4.25E-14	1.30E-12	20.3982357	13.37153763	up-regulated in High
EFCAB14	-0.4735817	4.75227734	-10.722596	2.89E-24	2.98E-22	43.5694817	23.53919317	up-regulated in High
CYP4B1	-1.9736856	3.48101384	-10.99617	2.57E-25	2.96E-23	45.9718042	24.59004227	up-regulated in High
CYP4X1	-0.8763596	2.23070428	-7.7698339	4.56E-14	1.39E-12	20.3285231	13.34081402	up-regulated in High
CYP4Z1	-0.1801456	0.28413941	-4.9867373	8.51E-07	8.22E-06	3.95739536	6.070193724	up-regulated in High
PDZK1IP1	-0.6891067	5.13098085	-4.5052849	8.28E-06	6.51E-05	1.76930987	5.082157442	up-regulated in High
TAL1	-0.1853227	0.5105628	-5.98929	4.05E-09	5.96E-08	9.148194	8.392199816	up-regulated in High
STIL	0.69814294	1.41256295	14.5803867	2.52E-40	7.61E-38	80.3570534	39.59924545	up-regulated in Low
CMPK1	-0.1126366	5.72359004	-2.6655457	0.00793727	0.02859111	-4.6577733	2.100328979	up-regulated in High
TRABD2B	-0.1683655	0.46193314	-5.1420717	3.92E-07	4.03E-06	4.70626603	6.40681598	up-regulated in High
SLC5A9	-0.2457445	0.44891205	-5.14019	3.96E-07	4.06E-06	4.69707058	6.402686609	up-regulated in High
SPATA6	-0.4184723	1.50619837	-8.7971556	2.33E-17	1.10E-15	27.8090633	16.63172944	up-regulated in High
BEND5	-0.2758356	1.21100519	-4.1222811	4.40E-05	0.00029801	0.17505558	4.356776481	up-regulated in High
FAF1	0.17022751	2.94879417	4.95632911	9.88E-07	9.43E-06	3.81322583	6.005308556	up-regulated in Low
CDKN2C	0.20333306	2.32000603	3.0968287	0.00206689	0.00899519	-3.4342229	2.684682921	up-regulated in Low
RNF11	-0.138916	4.90617048	-3.2549312	0.00121174	0.00563563	-2.941517	2.916591514	up-regulated in High
EPS15	-0.1614307	3.47563498	-4.8647934	1.54E-06	1.41E-05	3.3840681	5.811997426	up-regulated in High
NRD1	0.26203192	4.45891803	6.64435323	8.04E-11	1.55E-09	12.9816607	10.09475891	up-regulated in Low
RAB3B	0.52120883	0.51321934	8.06967723	5.37E-15	1.85E-13	22.4387987	14.27033971	up-regulated in Low
TXNDC12	0.13873515	4.75538542	3.40078462	0.00072621	0.00359211	-2.4661615	3.138937301	up-regulated in Low
KTI12	0.11947773	2.81179524	3.01166965	0.00273086	0.01143887	-3.6898317	2.56370136	up-regulated in Low
BTF3L4	0.16427083	3.3619897	3.8200413	0.00015037	0.00089708	-0.9895594	3.822826936	up-regulated in Low

CC2D1B	-0.1076917	3.15816126	-2.6696491	0.00784231	0.02829969	-4.6469685	2.105555944	up-regulated in High
ORC1	1.06172485	1.59289339	18.944721	1.31E-60	1.78E-57	126.933562	59.88252582	up-regulated in Low
FAM159A	-0.1505046	0.46731149	-5.2704237	2.03E-07	2.21E-06	5.3406127	6.691453349	up-regulated in High
COA7	0.40193873	2.7376949	9.30780643	4.22E-19	2.46E-17	31.7799133	18.37475606	up-regulated in Low
ZYG11B	-0.1619603	2.5713557	-4.4819615	9.20E-06	7.17E-05	1.6684828	5.036438281	up-regulated in High
ZYG11A	0.29614862	0.69976624	5.1926829	3.03E-07	3.19E-06	4.95472469	6.518354325	up-regulated in Low
ECHDC2	-0.52073	2.51478366	-8.1095688	4.02E-15	1.41E-13	22.7241928	14.395972	up-regulated in High
SCP2	-0.3617462	4.19498582	-8.7567984	3.18E-17	1.47E-15	27.5021539	16.49691301	up-regulated in High
PODN	-0.9649531	3.07186312	-11.49861	2.75E-27	3.69E-25	50.4781875	26.56021521	up-regulated in High
SLC1A7	-1.0593491	1.49203081	-6.7785282	3.46E-11	7.06E-10	13.8081155	10.46086785	up-regulated in High
MAGOH	0.33508474	4.2912903	7.73947214	5.65E-14	1.70E-12	20.1183114	13.24816273	up-regulated in Low
LRP8	0.46096474	1.41580743	7.60666894	1.43E-13	4.04E-12	19.2064521	12.8461273	up-regulated in Low
NDC1	0.66358395	2.93858444	13.4880233	1.54E-35	3.57E-33	69.3791403	34.81230016	up-regulated in Low
DIO1	-0.2760826	0.53783734	-3.5684677	0.000394	0.00209931	-1.8951224	3.404507549	up-regulated in High
HSPB11	0.14012301	2.80915464	3.00294758	0.00280889	0.01173206	-3.7156237	2.551465803	up-regulated in Low
LRRC42	0.39854344	3.85447327	8.15795676	2.83E-15	1.02E-13	23.0718167	14.54897496	up-regulated in Low
TMEM59	-0.3593558	5.26926227	-7.6491787	1.06E-13	3.09E-12	19.4969816	12.97424427	up-regulated in High
MRPL37	0.45450895	4.73458503	12.2518529	2.48E-30	4.11E-28	57.4477446	29.60497	up-regulated in Low
SSBP3	-0.1194576	3.46268086	-2.0916605	0.03697736	0.10255056	-6.0088818	1.432064148	up-regulated in High
TTC4	0.1114633	0.89546411	5.55573956	4.52E-08	5.55E-07	6.80055055	7.344999451	up-regulated in Low
PARS2	0.15257511	1.99634353	4.07861326	5.28E-05	0.00035147	0.00166001	4.277524012	up-regulated in Low
DHCR24	-0.422578	7.08140088	-5.622816	3.14E-08	3.95E-07	7.15365378	7.502778031	up-regulated in High
PCSK9	0.29167742	1.30488093	2.55159701	0.01102221	0.03780755	-4.9513553	1.957731532	up-regulated in Low
PPAP2B	-0.3390503	3.66168001	-5.9882003	4.08E-09	5.99E-08	9.14210121	8.389487133	up-regulated in High
PRKAA2	0.41251471	1.15772576	6.38443668	3.96E-10	6.85E-09	11.4201998	9.402204599	up-regulated in Low
C1orf168	-0.2059839	0.57762052	-3.4717973	0.0005622	0.00286559	-2.2275246	3.250107329	up-regulated in High
OMA1	-0.1430037	2.47156781	-3.3944836	0.00074274	0.00366733	-2.4871087	3.129165245	up-regulated in High
TACSTD2	-0.4406747	6.83658784	-3.9005324	0.00010922	0.00067412	-0.6875388	3.961689319	up-regulated in High
JUN	-0.4599069	5.76703652	-5.5113561	5.74E-08	6.92E-07	6.56896407	7.241460757	up-regulated in High
FGGY	-0.2472017	1.43785551	-4.9049039	1.27E-06	1.18E-05	3.5712294	5.896333696	up-regulated in High
HOOK1	0.14447525	2.75769937	2.34382872	0.01948113	0.06076095	-5.454456	1.710385772	up-regulated in Low
C1orf87	-0.2869976	0.32026624	-5.3629667	1.26E-07	1.42E-06	5.80664953	6.900298222	up-regulated in High
NFIA	-0.3086132	1.94184516	-4.9557242	9.91E-07	9.45E-06	3.81036604	6.004021201	up-regulated in High
INADL	-0.148981	2.80525911	-2.831072	0.00482781	0.01873589	-4.2091316	2.31624967	up-regulated in High
USP1	0.39225479	3.64457061	7.88858548	1.97E-14	6.28E-13	21.1568743	13.70580644	up-regulated in Low
DOCK7	0.13952434	1.93077277	3.40574414	0.00071345	0.00353556	-2.4496479	3.146639439	up-regulated in Low
FOXD3	0.14908428	0.18788232	5.05872223	5.96E-07	5.92E-06	4.30186168	6.225118031	up-regulated in Low
ALG6	0.1552511	2.07270277	4.22018632	2.90E-05	0.00020417	0.5700745	4.537040039	up-regulated in Low
ITGB3BP	0.25323846	1.6943852	6.03589319	3.10E-09	4.65E-08	9.4096632	8.508590795	up-regulated in Low
PGM1	0.10884019	4.45985561	1.97390922	0.04894704	0.12828219	-6.2465616	1.310273609	up-regulated in Low
ROR1	-0.266634	1.14200221	-4.6652363	3.97E-06	3.33E-05	2.47373549	5.401064388	up-regulated in High
CACHD1	-0.805387	1.86686745	-8.7952403	2.37E-17	1.11E-15	27.7944748	16.6253215	up-regulated in High
JAK1	-0.2514816	4.78957521	-4.9570238	9.84E-07	9.40E-06	3.81651063	6.00678722	up-regulated in High
AK4	0.59165653	1.89910617	6.28471655	7.20E-10	1.19E-08	10.8351341	9.142397353	up-regulated in Low
DNAJC6	0.20202964	0.91580684	3.65928521	0.00028005	0.00155442	-1.5749509	3.552772172	up-regulated in Low
LEPROT	-0.2247418	4.68723179	-5.6242726	3.12E-08	3.92E-07	7.16136349	7.506221832	up-regulated in High
PDE4B	-0.1284778	1.70645134	-2.0561802	0.04028832	0.10999318	-6.0819235	1.394820853	up-regulated in High
TCTEX1D1	-0.2412022	0.44402524	-5.2348114	2.44E-07	2.62E-06	5.16320392	6.611892368	up-regulated in High
WDR78	-0.2594647	0.77727757	-4.787256	2.24E-06	1.98E-05	3.02623062	5.65061502	up-regulated in High
IL12RB2	0.20017676	0.35537416	5.24791725	2.28E-07	2.46E-06	5.22836779	6.641119721	up-regulated in Low
IL12RB2	0.20017676	0.35537416	5.24791725	2.28E-07	2.46E-06	5.22836779	6.641119721	up-regulated in Low
SERBP1	0.29018281	5.07659145	7.86472244	2.33E-14	7.35E-13	20.9896322	13.63212863	up-regulated in Low
GNG12	-0.2588783	4.50492628	-3.5173523	0.00047596	0.00248037	-2.071965	3.322427262	up-regulated in High
DIRAS3	-0.354014	1.09575414	-3.8355565	0.00014145	0.00084966	-0.9318044	3.849404618	up-regulated in High
WLS	-0.6694658	3.99304777	-6.592462	1.11E-10	2.10E-09	12.6657387	9.954730627	up-regulated in High
DEPDC1	1.10560585	1.3449465	18.8122836	5.63E-60	6.38E-57	125.478217	59.24917556	up-regulated in Low
LRRC40	0.2096199	2.83306983	4.51776285	7.82E-06	6.19E-05	1.82345	5.106698988	up-regulated in Low
ANKRD13C	-0.132349	2.80766201	-3.174201	0.00159592	0.00718323	-3.1960444	2.796989502	up-regulated in High
HHLA3	-0.1690253	2.61746026	-2.4176839	0.01597942	0.05162345	-5.2803959	1.796438887	up-regulated in High
NEGR1	-0.179259	0.42107723	-5.22325	2.59E-07	2.77E-06	5.10584059	6.586159958	up-regulated in High
ERICH3	-0.3358544	0.41103633	-5.1176562	4.43E-07	4.52E-06	4.5871884	6.353334627	up-regulated in High
CRYZ	-0.2063834	3.71105922	-2.698562	0.00720167	0.02632633	-4.5703797	2.142566569	up-regulated in High
LHX8	0.10700057	0.07686928	3.75001649	0.0001977	0.00114446	-1.2474769	3.703997869	up-regulated in Low
SLC44A5	0.22206853	1.32821277	2.32856048	0.0202837	0.06281116	-5.4897812	1.692852805	up-regulated in Low
RABGGTB	0.15647076	3.26018552	3.5054856	0.00049715	0.00257469	-2.112673	3.303512953	up-regulated in Low
ST6GALNAC	-0.1852027	1.27055973	-2.6635599	0.00798359	0.02872961	-4.6629964	2.097801721	up-regulated in High
ZZZ3	0.11408899	2.37608946	2.93132953	0.00353109	0.01432631	-3.9246751	2.452090879	up-regulated in Low
NEXN	-0.2825577	1.75916872	-4.5104685	8.08E-06	6.38E-05	1.79178384	5.092345481	up-regulated in High
FUBP1	0.22130546	4.18608869	5.28972045	1.84E-07	2.02E-06	5.43719158	6.734751366	up-regulated in Low
DNAJB4	0.29982712	2.37327546	4.40496583	1.30E-05	9.82E-05	1.33906005	4.886927998	up-regulated in Low
PTGFR	-0.3236503	0.53940867	-5.2063934	2.83E-07	2.99E-06	5.02240727	6.548726549	up-regulated in High

ELTD1	-0.1706982	2.41828653	-2.7760035	0.00571136	0.02167213	-4.3612974	2.2432607	up-regulated in High
TTLL7	0.23554508	0.59853026	4.73721322	2.83E-06	2.45E-05	2.79806502	5.547614932	up-regulated in Low
DNASE2B	-0.3199807	0.42937437	-7.561435	1.95E-13	5.42E-12	18.8987115	12.71039584	up-regulated in High
RPF1	0.25612249	4.07107944	6.32614661	5.62E-10	9.49E-09	11.0772566	9.249937494	up-regulated in Low
GNG5	0.15420435	6.38441881	3.48765865	0.00053064	0.00272446	-2.173581	3.275198685	up-regulated in Low
SSX2IP	0.51618793	1.60034652	10.5546573	1.25E-23	1.22E-21	42.1134611	22.90207377	up-regulated in Low
WDR63	-0.2475961	0.43123434	-4.9757062	8.98E-07	8.64E-06	3.90500344	6.046617246	up-regulated in High
SYDE2	-0.1064266	0.91592891	-2.2601536	0.02424494	0.07285198	-5.6452756	1.615378838	up-regulated in High
DDAH1	-0.6506644	4.20376902	-9.2548788	6.44E-19	3.70E-17	31.360948	18.19095152	up-regulated in High
CYR61	-0.6791645	5.86470918	-6.3251765	5.66E-10	9.54E-09	11.0715716	9.247412844	up-regulated in High
COL24A1	-0.1040209	0.46611638	-3.3069272	0.00101174	0.00481241	-2.7743408	2.994932299	up-regulated in High
HS2ST1	0.19333847	2.87617652	3.66868266	0.00027021	0.00150702	-1.5413854	3.568291478	up-regulated in Low
PKN2	0.26814313	3.24162523	5.89081815	7.10E-09	9.96E-08	8.60148797	8.148692426	up-regulated in Low
GBP1	0.39401391	3.74187014	3.61754969	0.00032791	0.00178596	-1.7230347	3.484250511	up-regulated in Low
GBP5	0.265855	2.07942634	2.5987149	0.0096361	0.03378103	-4.8314719	2.016098623	up-regulated in Low
LRRC8C	-0.1830737	1.36375046	-4.1770879	3.49E-05	0.00024139	0.39512048	4.457248631	up-regulated in High
LRRC8D	0.14666639	2.85050695	3.30368097	0.00102327	0.00485894	-2.7848524	2.990011254	up-regulated in Low
CDC7	0.72716581	1.88797265	12.3507836	9.72E-31	1.67E-28	58.3809975	30.01249042	up-regulated in Low
TGFBR3	-0.4487368	1.31829418	-7.0896218	4.66E-12	1.08E-10	15.7767348	11.33184295	up-regulated in High
GLMN	0.24676126	1.80487722	5.58830854	3.79E-08	4.70E-07	6.9715336	7.421413615	up-regulated in Low
EVI5	-0.1861667	1.9747269	-5.0381728	6.60E-07	6.50E-06	4.20307271	6.180702613	up-regulated in High
MTF2	0.15198037	2.44145227	3.56199226	0.00040359	0.00214439	-1.9176594	3.394054836	up-regulated in Low
CCDC18	0.22600202	0.83485917	6.96103057	1.08E-11	2.36E-10	14.9541949	10.96810479	up-regulated in Low
BCAR3	0.33054922	2.43174384	4.5636299	6.35E-06	5.12E-05	2.02364506	5.197400288	up-regulated in Low
DNTTIP2	0.35533441	3.30476847	9.07045143	2.78E-18	1.47E-16	29.9142913	17.55610846	up-regulated in Low
GCLM	0.65298159	3.14937178	7.49275639	3.12E-13	8.43E-12	18.4342561	12.5054946	up-regulated in Low
ABCA4	-0.5166596	1.22515593	-4.2738969	2.30E-05	0.00016566	0.79044579	4.637442311	up-regulated in High
ARHGAP29	-0.1838781	2.00671764	-2.8684559	0.00430075	0.01698168	-4.1041832	2.366455508	up-regulated in High
ABCD3	-0.2129699	3.33999996	-4.0886546	5.06E-05	0.00033848	0.04137916	4.295685105	up-regulated in High
SLC44A3	-0.3996906	3.14668576	-5.6243662	3.12E-08	3.92E-07	7.16185876	7.506443057	up-regulated in High
CNN3	-0.2959357	5.79653451	-4.2256534	2.84E-05	0.0001998	0.59238713	4.547210917	up-regulated in High
TMEM56	-0.1323506	1.58491368	-2.1208659	0.03442907	0.09679828	-5.9478367	1.463074746	up-regulated in High
PTBP2	0.11327511	1.10367681	2.85318408	0.0045094	0.01767403	-4.1472168	2.345881668	up-regulated in Low
DPYD	-0.6223965	3.1405954	-7.10426	4.23E-12	9.86E-11	15.8711482	11.37357843	up-regulated in High
SNX7	0.19193551	3.34996094	2.90244494	0.00386756	0.01549243	-4.0076095	2.412562927	up-regulated in Low
LPPR4	-0.3289255	0.64391572	-8.2003046	2.07E-15	7.65E-14	23.3773422	14.68342802	up-regulated in High
PALMD	-0.3719035	1.19134838	-6.9909554	8.87E-12	1.97E-10	15.144509	11.05228607	up-regulated in High
FRRS1	0.2230137	1.21009603	4.02742573	6.52E-05	0.00042443	-0.1993956	4.185528235	up-regulated in Low
SASS6	0.51443803	1.50910335	12.1375787	7.30E-30	1.18E-27	56.3747572	29.13638157	up-regulated in Low
LRRC39	-0.1295146	0.58847162	-4.1859874	3.36E-05	0.00023321	0.43110988	4.473668384	up-regulated in High
RTCA	0.27720447	3.56776592	7.02067948	7.31E-12	1.65E-10	15.3342089	11.13618246	up-regulated in Low
CDC14A	-0.1651648	1.24193831	-3.710559	0.00023022	0.00130976	-1.3908221	3.637851813	up-regulated in High
VCAM1	-0.2886697	2.65226073	-2.9961275	0.00287132	0.01195264	-3.735741	2.541918588	up-regulated in High
DPH5	0.17480557	2.80129839	3.91726397	0.00010213	0.00063625	-0.6240144	3.990859139	up-regulated in Low
S1PR1	-0.5266836	2.91252313	-7.3556716	7.93E-13	2.03E-11	17.517307	12.10078712	up-regulated in High
RNPC3	-0.3499531	1.62481435	-6.4458871	2.73E-10	4.86E-09	11.7846222	9.563942236	up-regulated in High
AMY2B	-0.3848923	0.83643289	-7.0885971	4.69E-12	1.08E-10	15.7701312	11.32892371	up-regulated in High
HENMT1	0.35584691	2.11821432	4.32409973	1.85E-05	0.00013586	0.99876433	4.732252428	up-regulated in Low
GPSM2	0.30254328	1.77344446	5.70935806	1.96E-08	2.55E-07	7.61473106	7.708649309	up-regulated in Low
WDR47	-0.1681999	2.07107899	-4.2980686	2.08E-05	0.00015062	0.89046541	4.682975291	up-regulated in High
TAF13	0.2188034	4.47232761	4.20886898	3.05E-05	0.00021322	0.52397084	4.516020639	up-regulated in Low
TMEM167B	-0.1927362	3.76687073	-5.542949	4.84E-08	5.91E-07	6.73364293	7.315090901	up-regulated in High
C1orf194	-0.5191303	1.06525214	-4.9934976	8.23E-07	7.97E-06	3.98955566	6.084664186	up-regulated in High
KIAA1324	-0.530213	2.7692821	-3.336869	0.00091094	0.00438079	-2.6769229	3.040509433	up-regulated in High
PSRC1	0.81264388	1.79869884	15.0648587	1.70E-42	5.84E-40	85.3367192	41.76959533	up-regulated in Low
MYBPHL	-0.8313447	1.16520775	-7.7868163	4.05E-14	1.24E-12	20.4463823	13.39275595	up-regulated in High
SORT1	-0.418177	3.98359411	-7.0130994	7.68E-12	1.72E-10	15.2857697	11.11476105	up-regulated in High
PSMA5	0.37379079	4.19195729	8.37389487	5.76E-16	2.29E-14	24.6422538	15.23988103	up-regulated in Low
CYB561D1	-0.1632818	1.71717828	-3.4906127	0.00052495	0.00269824	-2.1635084	3.27988232	up-regulated in High
AMIGO1	-0.3848512	1.5856224	-7.4072104	5.59E-13	1.46E-11	17.8604571	12.25226994	up-regulated in High
GNAI3	0.16107649	2.20537074	4.76249045	2.51E-06	2.20E-05	2.91304096	5.599528037	up-regulated in Low
GSTM2	-0.3015067	1.17887449	-5.6020246	3.52E-08	4.39E-07	7.04380457	7.453704859	up-regulated in High
GSTM5	-0.3187784	0.56024331	-9.8636646	4.49E-21	3.38E-19	36.2793541	20.3473826	up-regulated in High
EPS8L3	0.32396474	0.59079919	2.76915671	0.00583087	0.02205969	-4.380014	2.234266377	up-regulated in Low
CSF1	-0.5058916	3.54527758	-5.7794808	1.33E-08	1.78E-07	7.99285383	7.877357517	up-regulated in High
AHCYL1	-0.1480052	5.05975735	-3.5741725	0.00038572	0.00205794	-1.8752352	3.413729439	up-regulated in High
SLC6A17	0.18569089	0.1971259	5.55491002	4.54E-08	5.57E-07	6.7962071	7.34305799	up-regulated in Low
KCNC4	-0.1248391	0.48516727	-5.1481065	3.80E-07	3.92E-06	4.73577709	6.420067766	up-regulated in High
RBM15	0.16673532	1.9068652	5.51201549	5.71E-08	6.90E-07	6.57239261	7.242993947	up-regulated in Low
SLC16A4	-0.3616152	3.06360471	-2.7523449	0.00613399	0.02303333	-4.4257812	2.212256886	up-regulated in High
LAMTOR5	0.13520289	4.89679474	2.50665016	0.01250726	0.04205362	-5.0637232	1.902837945	up-regulated in Low

KCNA3	-0.5911249	1.26233985	-8.1235202	3.63E-15	1.28E-13	22.8242588	14.44001757	up-regulated in High
CD53	-0.4732574	4.85823652	-5.0564866	6.02E-07	5.98E-06	4.29109669	6.220278682	up-regulated in High
LRIF1	0.22767844	2.63624268	4.2094845	3.04E-05	0.00021273	0.52647536	4.51716262	up-regulated in Low
CEPT1	-0.1328154	1.97712188	-3.7226935	0.00021972	0.00125735	-1.3468914	3.658131534	up-regulated in High
DENND2D	-0.2349388	3.16419313	-5.0287613	6.91E-07	6.78E-06	4.15794967	6.160411347	up-regulated in High
CHI3L2	-0.7112584	2.13253825	-6.2884032	7.05E-10	1.17E-08	10.8566242	9.151943601	up-regulated in High
CHIA	-0.8236341	1.12377846	-6.9610319	1.08E-11	2.36E-10	14.954203	10.96810838	up-regulated in High
PIFO	-0.8342642	1.89206213	-8.577098	1.25E-16	5.42E-15	26.1481677	15.90196261	up-regulated in High
OVGP1	-0.3176088	1.36406935	-4.6713689	3.86E-06	3.25E-05	2.50119235	5.413477497	up-regulated in High
WDR77	0.18451	3.48772303	4.45010487	1.06E-05	8.18E-05	1.53154638	4.974314702	up-regulated in Low
ATP5F1	0.16642997	5.12949945	3.80204825	0.00016139	0.00095406	-1.0562617	3.792117757	up-regulated in Low
C1orf162	-0.5334042	2.86888783	-7.1447542	3.24E-12	7.66E-11	16.1331542	11.48938213	up-regulated in High
ADORA3	-0.4488814	1.4116368	-7.4723067	3.59E-13	9.62E-12	18.2966117	12.44475912	up-regulated in High
RAP1A	-0.2773283	3.8912444	-6.9348807	1.27E-11	2.76E-10	14.7884378	10.89477476	up-regulated in High
FAM212B	-0.1944231	0.69033927	-6.6456788	7.97E-11	1.54E-09	12.9897581	10.09834738	up-regulated in High
KCND3	-0.4391923	0.84464469	-8.1664246	2.66E-15	9.64E-14	23.132813	14.5758192	up-regulated in High
CTTNBP2NL	-0.1909514	2.69680922	-4.2826234	2.22E-05	0.00016016	0.82649445	4.653855696	up-regulated in High
WNT2B	-0.146057	0.38343327	-6.4587806	2.52E-10	4.52E-09	11.8614598	9.598035737	up-regulated in High
CAPZA1	0.22052375	5.45330027	5.05885716	5.95E-07	5.92E-06	4.3025115	6.225410149	up-regulated in Low
MOV10	0.13533383	3.46638276	2.62709671	0.00887845	0.03147648	-4.7582292	2.051662734	up-regulated in Low
SLC16A1	0.73234908	2.05084776	7.05565317	5.82E-12	1.33E-10	15.5582557	11.23525224	up-regulated in Low
LRIG2	-0.1256748	1.36697158	-3.6623299	0.00027682	0.00153889	-1.564085	3.557796594	up-regulated in High
MAGI3	-0.5874179	2.53794725	-8.4658242	2.90E-16	1.19E-14	25.3201912	15.53798988	up-regulated in High
RSBN1	-0.2169659	2.19244147	-5.8327188	9.85E-09	1.35E-07	8.28262096	8.006571631	up-regulated in High
PTPN22	-0.3591186	1.68281541	-5.0079042	7.66E-07	7.47E-06	4.05822184	6.11555599	up-regulated in High
BCL2L15	-0.5121954	1.79749991	-5.4502197	7.94E-08	9.33E-07	6.25265537	7.099967019	up-regulated in High
AP4B1	-0.2077271	2.10474268	-4.8547883	1.62E-06	1.48E-05	3.3376001	5.791051039	up-regulated in High
DCLRE1B	0.17527836	2.04412021	4.37065032	1.51E-05	0.00011295	1.19394269	4.820996944	up-regulated in Low
HIPK1	-0.2350787	3.2803392	-4.6095145	5.14E-06	4.22E-05	2.22577623	5.288906595	up-regulated in High
OLFML3	-0.6739639	3.97578286	-6.3228516	5.74E-10	9.66E-09	11.0579505	9.241363734	up-regulated in High
BCAS2	0.17125986	4.74400258	3.80349426	0.00016048	0.00094914	-1.0509122	3.794581201	up-regulated in Low
AMPD1	-0.2977215	0.47853139	-6.7293267	4.72E-11	9.44E-10	13.5034592	10.32594284	up-regulated in High
NRAS	0.37090078	4.19258413	6.92747357	1.34E-11	2.89E-10	14.7415795	10.87404301	up-regulated in Low
SIKE1	0.11021666	2.82710005	2.64664275	0.00838817	0.02996391	-4.7073384	2.076332761	up-regulated in Low
TSPAN2	-0.284586	1.29999709	-4.9180239	1.19E-06	1.12E-05	3.63275219	5.924045832	up-regulated in High
NGF	-0.358889	1.16502885	-3.5377516	0.0004415	0.00232047	-2.0016808	3.355065886	up-regulated in High
VANGL1	0.29178459	2.15755281	6.078717	2.42E-09	3.70E-08	9.65146935	8.616190376	up-regulated in Low
CASQ2	-0.304583	0.38246433	-8.278694	1.17E-15	4.43E-14	23.946065	14.93365631	up-regulated in High
SLC22A15	-0.3270253	1.22718356	-6.4762903	2.27E-10	4.09E-09	11.9660144	9.644422836	up-regulated in High
ATP1A1	-0.4913575	6.87679247	-7.4349213	4.63E-13	1.22E-11	18.0457513	12.33405308	up-regulated in High
CD58	-0.1389751	3.23744988	-2.5641568	0.01063634	0.0366386	-4.9196079	1.973207633	up-regulated in High
IGSF3	-0.3166693	3.14039539	-4.4142342	1.25E-05	9.47E-05	1.378435	4.904809798	up-regulated in High
CD2	-0.3486058	3.46103478	-3.434893	0.00064255	0.00322112	-2.3521276	3.192095821	up-regulated in High
CD101	-0.2545594	0.96765538	-5.686066	2.22E-08	2.86E-07	7.4900281	7.652986063	up-regulated in High
TTF2	0.32492092	1.59775086	9.21008388	9.21E-19	5.19E-17	31.0076772	18.03595004	up-regulated in Low
VTCN1	-0.5296048	1.40955344	-4.0720417	5.42E-05	0.00036035	-0.024285	4.265658699	up-regulated in High
FAM46C	-0.7215367	3.35823707	-8.3476961	6.99E-16	2.74E-14	24.4500683	15.15535586	up-regulated in High
WDR3	0.26717233	2.32526441	5.97566085	4.38E-09	6.40E-08	9.0720581	8.358300202	up-regulated in Low
SPAG17	-0.2753226	0.64924313	-5.0444114	6.39E-07	6.32E-06	4.23302594	6.194170819	up-regulated in High
ZNF697	0.24441505	1.34258913	5.3342443	1.46E-07	1.63E-06	5.66123249	6.835156044	up-regulated in Low
PHGDH	0.73432142	2.26798727	6.48178071	2.19E-10	3.96E-09	11.9988484	9.658989017	up-regulated in Low
NOTCH2	-0.2913305	3.38611964	-4.1087243	4.65E-05	0.00031391	0.12103981	4.332096385	up-regulated in High
FAM72B	0.44825213	0.4302017	17.7566329	5.90E-55	4.47E-52	113.94527	54.22940799	up-regulated in Low
FAM72C	0.27834948	0.21903081	15.415444	4.40E-44	1.67E-41	88.9778503	43.35620401	up-regulated in Low
FAM72D	0.38095026	0.33705498	17.4646503	1.41E-53	9.90E-51	110.780125	52.85150236	up-regulated in Low
GPR89A	0.13507869	0.88164097	5.70829111	1.97E-08	2.56E-07	7.60900896	7.70609542	up-regulated in Low
POLR3C	0.18218816	3.66776	4.63430723	4.58E-06	3.80E-05	2.33576528	5.338669935	up-regulated in Low
NUDT17	-0.1264718	1.78280967	-2.4977343	0.01282213	0.04299842	-5.0857817	1.892039932	up-regulated in High
ANKRD35	-0.2476963	1.02988302	-4.5639195	6.34E-06	5.12E-05	2.02491491	5.197975383	up-regulated in High
ITGA10	-0.2859741	0.72098692	-5.5975533	3.60E-08	4.49E-07	7.02022813	7.443171201	up-regulated in High
PEX11B	-0.248649	4.71581832	-5.5448344	4.79E-08	5.86E-07	6.74349708	7.319496069	up-regulated in High
RBM8A	0.25867315	4.43442932	6.61888182	9.42E-11	1.80E-09	12.8263275	10.02591513	up-regulated in Low
LIX1L	-0.3297961	3.10223583	-5.0441197	6.40E-07	6.33E-06	4.23162466	6.19354077	up-regulated in High
ANKRD34A	-0.1577129	0.57932958	-3.550675	0.0004209	0.00222438	-1.9569544	3.375824453	up-regulated in High
POLR3GL	-0.4513506	3.99973385	-9.1698782	1.27E-18	7.03E-17	30.6916321	17.89726771	up-regulated in High
TXNIP	-0.9405951	7.39386704	-10.894107	6.37E-25	7.05E-23	45.0712175	24.19614722	up-regulated in High
NBPF12	-0.1312274	1.13271932	-3.2933129	0.00106091	0.00501586	-2.8183585	2.974320924	up-regulated in High
PRKAB2	0.13511243	2.4936302	2.55858238	0.01080609	0.03717725	-4.9337171	1.966331496	up-regulated in Low
FMO5	-1.0614492	2.90464086	-8.9614166	6.53E-18	3.30E-16	29.0688119	17.18494802	up-regulated in High
CHD1L	0.26008786	4.30227402	4.88158935	1.42E-06	1.31E-05	3.46227054	5.84724192	up-regulated in Low
GJA5	-0.5253613	2.97979675	-6.408081	3.43E-10	6.01E-09	11.5600691	9.464289231	up-regulated in High

AC245100.1	0.14745553	1.17702883	2.6691219	0.00785445	0.02833825	-4.6483576	2.104884032	up-regulated in Low
PDE4DIP	-0.1519195	1.8686486	-3.7948227	0.00016603	0.00097918	-1.0829642	3.779819897	up-regulated in High
HIST2H2BF	0.14007856	0.70507108	2.2053751	0.02788619	0.08161357	-5.7665166	1.55461088	up-regulated in Low
HIST2H3D	0.15969647	0.36944337	3.05449521	0.00237576	0.01015101	-3.5621468	2.624197429	up-regulated in Low
HIST2H2BE	0.24841834	3.55634528	2.29922524	0.0219072	0.06694733	-5.5570184	1.65941316	up-regulated in Low
HIST2H2AC	0.4943733	1.31754621	5.28542532	1.88E-07	2.06E-06	5.41566747	6.725102564	up-regulated in Low
HIST2H2AB	0.16303913	0.27589579	2.31288445	0.02113766	0.0650142	-5.5258149	1.674943087	up-regulated in Low
BOLA1	0.1896955	3.54149396	3.02503958	0.00261509	0.01102533	-3.6501556	2.582513146	up-regulated in Low
SV2A	0.42576222	1.21582586	4.9795761	8.81E-07	8.49E-06	3.92337141	6.054883275	up-regulated in Low
SF3B4	0.37064011	5.61003499	7.54176179	2.23E-13	6.16E-12	18.7653229	12.65155569	up-regulated in Low
VPS45	0.14696511	3.09602616	3.66423094	0.00027483	0.00152882	-1.557296	3.560935604	up-regulated in Low
ANP32E	0.58045002	4.94146507	7.1412573	3.32E-12	7.81E-11	16.1104808	11.47936167	up-regulated in Low
C1orf54	-0.1611446	2.63119902	-2.8273996	0.00488263	0.01892221	-4.2193691	2.311346439	up-regulated in High
MRPS21	0.15185804	5.31028021	2.46524026	0.01403004	0.04630443	-5.1655252	1.852941215	up-regulated in Low
PRPF3	0.17342834	3.52273652	3.23161104	0.00131284	0.00605663	-3.0156707	2.881788743	up-regulated in Low
TARS2	0.21660858	3.48260827	4.79433303	2.16E-06	1.92E-05	3.05867431	5.665254609	up-regulated in Low
ECM1	-0.7088529	3.81161266	-5.342653	1.40E-07	1.57E-06	5.70373236	6.854196792	up-regulated in High
ADAMTSL4	-0.2328981	2.07650493	-2.8135058	0.00509516	0.01964676	-4.2579849	2.292842073	up-regulated in High
MCL1	-0.1132642	6.92105989	-2.2124444	0.02739116	0.0804997	-5.7510341	1.562389614	up-regulated in High
ENSA	0.10707146	5.35888471	2.36870374	0.01823312	0.05753873	-5.3964206	1.739139011	up-regulated in Low
GOLPH3L	-0.1654702	4.20133009	-2.9316923	0.00352704	0.01431286	-3.9236284	2.452589392	up-regulated in High
CTSS	-0.4714601	5.83317008	-5.7750759	1.36E-08	1.82E-07	7.96898245	7.866709943	up-regulated in High
CTSK	-0.4610794	4.98613231	-4.2503769	2.55E-05	0.00018149	0.69362571	4.593344389	up-regulated in High
SETDB1	0.16164429	3.02476164	3.54107933	0.00043611	0.00229501	-1.9901789	3.36040506	up-regulated in Low
ANXA9	-0.1446972	2.45151939	-2.1675441	0.03066885	0.08824472	-5.8485448	1.513302573	up-regulated in High
FAM63A	-0.2017212	3.14657968	-3.3674096	0.00081781	0.00398457	-2.576692	3.087347957	up-regulated in High
PRUNE	0.10604133	3.88811906	2.15790605	0.03141489	0.08996811	-5.8692204	1.502864527	up-regulated in Low
BNIPL	-0.5918388	1.94477361	-5.1115254	4.57E-07	4.65E-06	4.55736746	6.339938566	up-regulated in High
C1orf56	0.14827693	2.45710803	2.41516516	0.01608903	0.0518828	-5.2864188	1.793470249	up-regulated in Low
MLLT11	0.6899786	1.79369548	7.53778799	2.29E-13	6.30E-12	18.7384131	12.6396847	up-regulated in Low
TNFAIP8L2	-0.5059185	2.56336843	-6.6692685	6.88E-11	1.34E-09	13.134085	10.16230303	up-regulated in High
SCNM1	0.31972704	3.38286147	6.8099622	2.83E-11	5.87E-10	14.0037192	10.54747583	up-regulated in Low
LYSMD1	0.28827248	2.53394687	5.52836617	5.24E-08	6.35E-07	6.65752616	7.281061016	up-regulated in Low
VPS72	0.29341872	3.82891577	6.23236382	9.83E-10	1.60E-08	10.5311187	9.007321779	up-regulated in Low
PIP5K1A	0.24005512	4.01955533	5.05759818	5.99E-07	5.95E-06	4.29644855	6.222684601	up-regulated in Low
PSMD4	0.3796897	5.81355444	8.35043348	6.85E-16	2.69E-14	24.4701273	15.16417832	up-regulated in Low
ZNF687	0.12286712	3.19798931	2.46424278	0.01406866	0.04640567	-5.167957	1.85174731	up-regulated in Low
SELENBP1	-1.3254809	5.31210858	-11.033149	1.85E-25	2.15E-23	46.2993586	24.73329264	up-regulated in High
PSMB4	0.40469439	6.82255545	8.50550448	2.15E-16	9.03E-15	25.6145242	15.66739053	up-regulated in Low
CGN	-0.2507101	4.55793112	-2.6059971	0.00943638	0.0331685	-4.8127531	2.02519455	up-regulated in High
TUFT1	0.34417482	3.59976617	5.47279006	7.05E-08	8.35E-07	6.3690663	7.152051432	up-regulated in Low
RIIAD1	-0.1118416	0.35970395	-2.3727732	0.01803578	0.05701606	-5.3868691	1.743865202	up-regulated in High
MRPL9	0.44811334	4.88932844	10.3212077	9.40E-23	8.42E-21	40.1137483	22.0267667	up-regulated in Low
RORC	-0.5035284	3.14647652	-5.2144162	2.71E-07	2.88E-06	5.06208652	6.566530087	up-regulated in High
C2CD4D	0.3186748	0.98588936	5.54174886	4.87E-08	5.94E-07	6.72737199	7.312287513	up-regulated in Low
THEM5	0.25227813	2.66625211	2.59528348	0.00973152	0.0340746	-4.8402746	2.011819549	up-regulated in Low
TCHH	0.10663115	0.19931909	2.79169501	0.00544576	0.02081154	-4.3182334	2.263941288	up-regulated in Low
IVL	-0.6954899	1.25516407	-4.6986314	3.40E-06	2.89E-05	2.62364986	5.468825059	up-regulated in High
PGLYRP4	-0.1963741	0.72702628	-2.5407882	0.01136421	0.03881608	-4.9785552	1.944460841	up-regulated in High
S100A12	0.24360318	0.80568731	3.20400916	0.00144257	0.00658623	-3.1027789	2.840862465	up-regulated in Low
S100A8	0.61780402	3.88442111	3.78322825	0.00017373	0.00101914	-1.1257118	3.760127236	up-regulated in Low
S100A7	0.32202554	0.6123164	2.48110474	0.01342826	0.04468503	-5.1267194	1.871980356	up-regulated in Low
S100A6	-0.3199116	10.2640067	-2.8794051	0.00415661	0.0164794	-4.0731931	2.3812606	up-regulated in High
S100A4	-0.4977727	6.92330945	-4.1788257	3.46E-05	0.00023973	0.40214251	4.460452602	up-regulated in High
S100A13	-0.2648978	4.59452949	-3.6066196	0.00034165	0.00185082	-1.7615499	3.466413935	up-regulated in High
CHTOP	0.21708117	4.1786152	5.90219357	6.66E-09	9.39E-08	8.66424139	8.176653597	up-regulated in Low
ILF2	0.49940395	6.67496428	10.233198	2.00E-22	1.71E-20	39.3673463	21.69996606	up-regulated in Low
NPR1	-0.5915544	1.63417586	-7.8271871	3.04E-14	9.45E-13	20.7273668	13.51657531	up-regulated in High
SLC27A3	-0.486145	2.75502635	-7.6112367	1.38E-13	3.92E-12	19.2376084	12.85986761	up-regulated in High
DENND4B	0.11766915	3.21345146	2.19769233	0.02843293	0.08291033	-5.7832877	1.546178383	up-regulated in Low
SLC39A1	0.18161455	5.86140181	4.16485298	3.68E-05	0.00025296	0.34575869	4.434722707	up-regulated in Low
CREB3L4	0.14973942	3.23919835	2.19373354	0.02871826	0.0835689	-5.7919071	1.541841935	up-regulated in Low
NUP210L	-0.1811977	0.40252153	-4.4953861	8.66E-06	6.79E-05	1.72645854	5.06272921	up-regulated in High
TPM3	0.35083967	5.75365834	9.12129849	1.86E-18	1.01E-16	30.3110707	17.73025739	up-regulated in Low
C1orf189	-0.4491892	0.83717738	-4.9946859	8.18E-07	7.92E-06	3.99521237	6.087209274	up-regulated in High
C1orf43	0.25202296	6.75465612	4.81459391	1.96E-06	1.76E-05	3.15179859	5.707266511	up-regulated in Low
UBAP2L	0.21288827	4.76366697	4.62352421	4.82E-06	3.98E-05	2.28786155	5.316998935	up-regulated in Low
HAX1	0.33363835	5.47072425	6.48156705	2.20E-10	3.97E-09	11.9975703	9.65842199	up-regulated in Low
ATP8B2	-0.2911115	2.66210205	-4.1665802	3.65E-05	0.00025126	0.35271907	4.437899394	up-regulated in High
IL6R	-0.6678251	3.14436521	-8.9377308	7.86E-18	3.93E-16	28.8861198	17.10473363	up-regulated in High
SHE	-0.9194162	1.81236861	-9.6836759	1.99E-20	1.36E-18	34.8028699	19.70032155	up-regulated in High

TDRD10	-0.7641085	1.28268771	-8.4185275	4.13E-16	1.68E-14	24.9707064	15.38432182	up-regulated in High
UBE2Q1	0.1421645	4.85990929	3.57229127	0.00038843	0.00207136	-1.8817967	3.410687022	up-regulated in Low
CHRNB2	0.16120348	0.14689649	3.3896944	0.00075553	0.00371981	-2.5030052	3.121747858	up-regulated in Low
ADAR	0.21108133	5.80960862	4.20974384	3.04E-05	0.00021257	0.52753068	4.517643807	up-regulated in Low
KCNN3	-0.2074818	0.66148235	-5.278184	1.96E-07	2.13E-06	5.37941462	6.708850098	up-regulated in High
PMVK	-0.1260827	5.32443373	-2.2556913	0.02452526	0.07357015	-5.655261	1.610386379	up-regulated in High
PBXIP1	-0.6727137	5.79981797	-10.301552	1.11E-22	9.87E-21	39.946693	21.95362841	up-regulated in High
PYGO2	0.11863412	4.55694903	2.69107178	0.00736295	0.02682352	-4.5902978	2.132948093	up-regulated in Low
SHC1	0.27800227	5.35959345	4.71179042	3.19E-06	2.73E-05	2.68299079	5.495636956	up-regulated in Low
CKS1B	1.08732398	3.57815029	16.9194436	5.09E-51	2.86E-48	104.90429	50.29318976	up-regulated in Low
FLAD1	0.50577331	3.846453	10.2524526	1.69E-22	1.46E-20	39.5302883	21.77131194	up-regulated in Low
ADAM15	-0.1368539	5.07752539	-2.0319791	0.04268865	0.11514654	-6.1310403	1.369687601	up-regulated in High
EFNA3	0.24051029	1.88737256	3.29573982	0.00105199	0.0049779	-2.8105244	2.977990049	up-regulated in Low
EFNA1	-0.2818046	6.13701573	-3.1205551	0.00191038	0.00839793	-3.3617854	2.718880478	up-regulated in High
SLC50A1	0.20646459	5.47578499	2.81814614	0.00502326	0.01939899	-4.2451083	2.299014077	up-regulated in Low
KRTCAP2	0.11108798	3.11222585	2.07670847	0.03834319	0.10560113	-6.0398125	1.416311728	up-regulated in Low
MUC1	-0.9525925	7.34526558	-7.4678041	3.70E-13	9.90E-12	18.2663456	12.43140349	up-regulated in High
AC234582.1	-0.2417662	1.25896022	-3.2190574	0.00137044	0.00629502	-3.0553774	2.863139159	up-regulated in High
THBS3	-0.2296412	2.99816482	-3.8063638	0.00015868	0.00094013	-1.0402907	3.799472063	up-regulated in High
MTX1	0.35951494	2.91341495	8.84032047	1.67E-17	7.97E-16	28.1384634	16.77640892	up-regulated in Low
GBA	0.1240117	4.22889483	2.525531	0.01186304	0.04021876	-5.0167578	1.925803883	up-regulated in Low
FAM189B	0.36744458	3.28440948	7.55529681	2.03E-13	5.64E-12	18.8570638	12.69202481	up-regulated in Low
SCAMP3	0.13759826	5.32125313	3.06902107	0.0022653	0.00974104	-3.5184433	2.644875093	up-regulated in Low
HCN3	0.1899412	1.37526285	3.30793842	0.00100817	0.0047974	-2.7710646	2.996465963	up-regulated in Low
FDPS	0.32690844	4.84994743	6.27957767	7.43E-10	1.23E-08	10.8051963	9.129098119	up-regulated in Low
RUSC1	0.15659173	3.46907968	3.01992747	0.00265881	0.01118686	-3.6653462	2.575312276	up-regulated in Low
ASH1L	-0.1317005	2.93360406	-2.3210258	0.02069031	0.06385018	-5.5071305	1.684232912	up-regulated in High
MSTO1	0.15067234	2.25457269	3.25200825	0.001224	0.00568814	-2.9508394	2.912218054	up-regulated in Low
DAP3	0.28621544	4.75575265	7.03161794	6.81E-12	1.54E-10	15.4041845	11.16712641	up-regulated in Low
ARHGEF2	-0.379644	3.68208582	-6.310934	6.16E-10	1.03E-08	10.9881951	9.210383999	up-regulated in High
SSR2	0.12585173	5.64057525	2.46895286	0.01388711	0.04590277	-5.1564656	1.857388209	up-regulated in Low
UBQLN4	0.19601217	4.17008726	3.85793577	0.00012945	0.00078486	-0.8481095	3.887899583	up-regulated in Low
LAMTOR2	0.1043372	5.19015377	2.01538069	0.04440415	0.11888806	-6.1643966	1.352576429	up-regulated in Low
RAB25	-0.1849341	6.19249887	-2.3169151	0.02091514	0.06445541	-5.5165725	1.679539248	up-regulated in High
MEX3A	0.47762489	2.22209822	4.59519639	5.49E-06	4.49E-05	2.16250253	5.260269752	up-regulated in Low
LMNA	-0.1707656	5.99242232	-2.6551629	0.00818216	0.02933571	-4.6850403	2.087131826	up-regulated in High
SEMA4A	-0.4892793	2.95669696	-6.9284869	1.33E-11	2.88E-10	14.7479876	10.87687825	up-regulated in High
BGLAP	-0.112813	0.77250872	-2.2222924	0.02671423	0.0788556	-5.7293848	1.573257355	up-regulated in High
PAQR6	0.21501868	1.11294664	3.04619056	0.00244112	0.01039374	-3.5870429	2.612411714	up-regulated in Low
SMG5	0.29327191	4.82623884	5.7609369	1.47E-08	1.96E-07	7.89246695	7.832578152	up-regulated in Low
TMEM79	0.233892	2.1701283	4.5125832	8.01E-06	6.32E-05	1.80095943	5.096504746	up-regulated in Low
GLMP	0.18675586	4.30883071	3.53992591	0.00043797	0.00230398	-1.9941668	3.358553965	up-regulated in Low
CCT3	0.63502768	6.35090758	13.124822	5.53E-34	1.13E-31	65.815036	33.25735773	up-regulated in Low
TSACC	0.42989458	0.74939886	10.2027145	2.59E-22	2.19E-20	39.1097883	21.58718665	up-regulated in Low
RHBG	0.14649304	0.36853992	3.71015988	0.00023058	0.00131151	-1.3922647	3.637185723	up-regulated in Low
C1orf61	0.27552586	0.26583585	5.85111938	8.88E-09	1.22E-07	8.38331134	8.051457654	up-regulated in Low
IQGAP3	1.02889958	2.29428215	13.6474651	3.16E-36	7.55E-34	70.9580136	35.50099188	up-regulated in Low
APOA1BP	0.33202825	5.96753282	5.28103051	1.93E-07	2.10E-06	5.39365997	6.715236573	up-regulated in Low
GPATCH4	0.29176105	3.22705088	5.60886111	3.39E-08	4.24E-07	7.07988527	7.46982437	up-regulated in Low
BCAN	0.24234859	0.41452107	5.54899061	4.69E-08	5.73E-07	6.76522978	7.329211101	up-regulated in Low
ISG20L2	0.30090297	3.45467015	8.1442704	3.12E-15	1.12E-13	22.9733323	14.50563066	up-regulated in Low
MRPL24	0.30009358	5.21310214	5.20447628	2.85E-07	3.02E-06	5.01293388	6.544475715	up-regulated in Low
HDGF	0.55163053	6.73005201	11.4662771	3.70E-27	4.85E-25	50.1846375	26.43191502	up-regulated in Low
PRCC	0.25830175	4.34720564	6.74096756	4.39E-11	8.82E-10	13.575373	10.35779527	up-regulated in Low
SH2D2A	0.31044969	1.85822356	4.75028428	2.66E-06	2.32E-05	2.85745026	5.574430658	up-regulated in Low
PEAR1	-0.2133441	1.10548684	-4.3617211	1.57E-05	0.00011705	1.15635409	4.803912253	up-regulated in High
LRRC71	-0.3007317	0.40192441	-4.9875533	8.47E-07	8.19E-06	3.96127513	6.071939491	up-regulated in High
FCRL5	-0.1843228	0.9724457	-2.584751	0.01002968	0.03490925	-4.8672233	1.99871315	up-regulated in High
FCRL3	-0.2880138	0.61237148	-5.4930724	6.32E-08	7.57E-07	6.47404007	7.19900807	up-regulated in High
FCRL2	-0.2221992	0.74098534	-3.5972627	0.00035385	0.00190681	-1.7944339	3.45118036	up-regulated in High
FCRL1	-0.29295	0.46707844	-6.1573729	1.53E-09	2.42E-08	10.0994324	8.815431632	up-regulated in High
KIRREL	-0.3532386	2.65519444	-4.7114716	3.20E-06	2.74E-05	2.6815511	5.49498653	up-regulated in High
CD1D	-0.2853965	1.27963772	-5.5519539	4.61E-08	5.65E-07	6.78073369	7.336141466	up-regulated in High
CD1A	-1.2225162	1.65919149	-9.7909456	8.22E-21	5.92E-19	35.6806126	20.08501546	up-regulated in High
CD1C	-1.1997845	1.87327733	-12.719766	2.84E-32	5.38E-30	61.896233	31.54712937	up-regulated in High
CD1B	-0.5355086	0.7177619	-8.7863583	2.54E-17	1.19E-15	27.7268507	16.59561731	up-regulated in High
CD1E	-0.9387239	1.1822999	-11.557785	1.60E-27	2.19E-25	51.0166813	26.79555838	up-regulated in High
MNDA	-0.6890056	3.04321418	-7.4987974	3.00E-13	8.09E-12	18.4749744	12.52346051	up-regulated in High
PYHIN1	-0.1469668	0.8078048	-3.0104338	0.00274179	0.01147973	-3.6934907	2.561965922	up-regulated in High
CADM3	-0.3758944	0.74625022	-6.1795315	1.34E-09	2.14E-08	10.2265227	8.8719361	up-regulated in High
ACKR1	-1.1590103	2.33864835	-10.834074	1.08E-24	1.16E-22	44.5438967	23.96548277	up-regulated in High

FCER1A	-1.4202985	1.89550063	-12.218377	3.41E-30	5.59E-28	57.132859	29.4674608	up-regulated in High
CRP	0.10882947	0.08318259	2.62667035	0.00888943	0.03150583	-4.7593352	2.051126216	up-regulated in Low
DUSP23	0.17434259	5.81147217	2.02978883	0.04291175	0.11563081	-6.1354573	1.367423758	up-regulated in Low
FCRL6	-0.1341481	0.79441586	-2.564858	0.01061516	0.03657857	-4.9178308	1.974073492	up-regulated in High
CFAP45	-0.3872049	1.50485684	-3.9620843	8.52E-05	0.00053966	-0.4525875	4.069514994	up-regulated in High
TAGLN2	-0.1700724	8.77335434	-2.7423714	0.00632047	0.02364538	-4.4528047	2.199250533	up-regulated in High
IGSF9	0.1931415	2.22033254	2.12899874	0.03374682	0.09524243	-5.9306896	1.471767176	up-regulated in Low
SLAMF9	0.31893574	0.83266111	4.97054837	9.21E-07	8.85E-06	3.88054212	6.03560839	up-regulated in Low
KCNJ10	0.11367873	0.35542812	2.92800987	0.00356836	0.01445339	-3.934247	2.447531868	up-regulated in Low
ATP1A2	-0.3426202	0.40321744	-10.273659	1.41E-22	1.23E-20	39.709974	21.84998649	up-regulated in High
PEA15	-0.2143667	6.09139733	-4.26848	2.36E-05	0.00016919	0.768103	4.6272679	up-regulated in High
DCAF8	-0.1591646	3.36015365	-3.3076963	0.00100902	0.00480028	-2.7718491	2.996098738	up-regulated in High
COPA	0.18506985	5.83589429	4.07815381	5.29E-05	0.00035211	-0.0001552	4.276693939	up-regulated in Low
NCSTN	0.16419922	5.44298377	3.7075062	0.00023294	0.00132353	-1.4018528	3.632758537	up-regulated in Low
SLAMF6	-0.3457542	1.90841308	-4.271051	2.33E-05	0.00016752	0.77870424	4.632095608	up-regulated in High
CD84	-0.3912577	1.83436004	-5.322215	1.56E-07	1.73E-06	5.60053677	6.807960073	up-regulated in High
SLAMF1	-0.241381	1.07178965	-4.5008639	8.44E-06	6.63E-05	1.75016088	5.073475964	up-regulated in High
CD48	-0.4771738	2.69855955	-4.9689139	9.29E-07	8.92E-06	3.8727954	6.032121803	up-regulated in High
LY9	-0.2281875	0.67010166	-5.8696369	8.00E-09	1.11E-07	8.48492005	8.096745757	up-regulated in High
ITLN1	-0.1808177	0.55482604	-1.9848276	0.04771461	0.1258005	-6.2250927	1.321348605	up-regulated in High
ITLN2	-0.3134169	0.5025774	-4.0552928	5.81E-05	0.00038347	-0.0902332	4.235490745	up-regulated in High
F11R	-0.1283195	5.44837075	-2.3602239	0.01865044	0.05861783	-5.416272	1.729310826	up-regulated in High
TSTD1	-0.3493127	5.03387808	-4.4004585	1.32E-05	1.00E-04	1.31993908	4.87824324	up-regulated in High
ARHGAP30	-0.4800315	2.99748894	-6.3970863	3.67E-10	6.39E-09	11.4949748	9.435396669	up-regulated in High
PVRL4	-0.4297339	4.16659734	-4.595609	5.48E-06	4.48E-05	2.16432327	5.261093893	up-regulated in High
KLHDC9	-0.1616409	2.04741128	-2.1346365	0.03328071	0.09420471	-5.9187651	1.477807486	up-regulated in High
PFDN2	0.57759203	6.24936377	10.9417302	4.17E-25	4.71E-23	45.4907979	24.37966878	up-regulated in Low
DEDD	0.16574172	3.98544694	5.04804686	6.28E-07	6.22E-06	4.25049591	6.202025527	up-regulated in Low
USP21	0.11279376	3.37049706	2.57865989	0.01020581	0.03540831	-4.8827595	1.991152683	up-regulated in Low
PPOX	-0.1329046	2.26516837	-2.5974099	0.00967229	0.03389514	-4.8348211	2.014470668	up-regulated in High
B4GALT3	0.2472674	4.24873701	5.4104424	9.80E-08	1.13E-06	6.04853503	7.008609474	up-regulated in Low
ADAMTS4	0.29402807	1.47770971	3.67184179	0.00026698	0.00149135	-1.5300834	3.573516064	up-regulated in Low
NDUFS2	0.27171792	4.91154388	6.73630941	4.52E-11	9.06E-10	13.5465841	10.34504418	up-regulated in Low
FCER1G	-0.2620196	5.55072484	-2.6929543	0.00732211	0.02670347	-4.5852968	2.135363513	up-regulated in High
APOA2	0.13317426	0.15104726	2.22713709	0.02638657	0.07802647	-5.7186999	1.578617079	up-regulated in Low
TOMM40L	0.15202832	2.80838215	3.16107058	0.00166813	0.00746223	-3.2368623	2.777770464	up-regulated in Low
PCP4L1	-1.1767766	2.97545375	-6.8810509	1.80E-11	3.83E-10	14.4488434	10.74450739	up-regulated in High
SDHC	0.14005012	2.98966757	3.45216931	0.00060369	0.00305199	-2.2939543	3.219189069	up-regulated in Low
FCGR2A	-0.4770416	3.54177176	-5.8018252	1.17E-08	1.59E-07	8.11418909	7.931471294	up-regulated in High
HSPA6	0.22413185	2.36456991	2.16280962	0.03103339	0.0891136	-5.8587126	1.508170731	up-regulated in Low
FCGR2B	-0.2665147	1.5821404	-3.904651	0.00010743	0.0006647	-0.6719256	3.968859919	up-regulated in High
FCRLA	-0.4058601	1.03245295	-5.2561536	2.19E-07	2.37E-06	5.26939467	6.659518815	up-regulated in High
DUSP12	0.24300535	3.4458384	6.1762241	1.37E-09	2.18E-08	10.2075285	8.863491825	up-regulated in Low
C1orf226	-0.1696693	2.48573851	-2.32186	0.02064495	0.06373485	-5.5052124	1.685186193	up-regulated in High
UHMK1	0.1090877	4.31088153	2.2235218	0.02663075	0.07864099	-5.7266755	1.574616613	up-regulated in Low
UAP1	0.28391534	4.53897302	5.39423203	1.07E-07	1.23E-06	5.96573123	6.971538005	up-regulated in Low
DDR2	-0.3869506	1.77740227	-5.8438265	9.25E-09	1.27E-07	8.34337049	8.033653584	up-regulated in High
C1orf110	-0.1305388	0.25783903	-2.3831972	0.01753881	0.05572089	-5.36233	1.755999896	up-regulated in High
RGS5	-0.7194139	3.627886	-8.7247605	4.07E-17	1.85E-15	27.2592464	16.39020011	up-regulated in High
NUF2	1.33820592	2.01839194	18.9826508	8.63E-61	1.20E-57	127.350672	60.06404359	up-regulated in Low
RXRG	-0.3101191	0.44460482	-5.6313208	3.00E-08	3.78E-07	7.19869169	7.522895035	up-regulated in High
LRRC52	-0.1693625	0.26386945	-4.005806	7.13E-05	0.0004607	-0.2835997	4.14696613	up-regulated in High
MGST3	0.18484346	3.3816464	3.84237905	0.00013768	0.00082961	-0.9063377	3.861120324	up-regulated in Low
ALDH9A1	-0.1148875	4.79974984	-2.4003461	0.01674741	0.0536209	-5.321731	1.77605248	up-regulated in High
TMCO1	0.11389633	4.60009046	2.41131028	0.01625806	0.05232673	-5.295625	1.788931376	up-regulated in Low
UCK2	1.0695293	2.2038383	14.4749757	7.40E-40	2.17E-37	79.2820676	39.13064234	up-regulated in Low
TADA1	0.1065889	2.74703394	2.68253032	0.00755082	0.02740764	-4.6129458	2.122005656	up-regulated in Low
GPA33	-0.1596557	0.5488671	-2.3947206	0.01700348	0.0542949	-5.3350805	1.769462156	up-regulated in High
POU2F1	0.12060413	1.63381272	3.26502369	0.00117026	0.00546799	-2.9092663	2.931717417	up-regulated in Low
CD247	-0.1780939	1.52534381	-2.6865982	0.00746082	0.02711577	-4.6021684	2.127213552	up-regulated in High
RCSD1	-0.4835115	2.01232504	-7.664316	9.55E-14	2.79E-12	19.6007441	13.01999582	up-regulated in High
DCAF6	-0.1879808	3.68194538	-4.429681	1.16E-05	8.89E-05	1.44422815	4.934682271	up-regulated in High
TIPRL	0.16990076	4.23960146	4.17462928	3.53E-05	0.00024361	0.38519021	4.4527175	up-regulated in Low
SFT2D2	-0.1845926	3.29255129	-3.6849716	0.00025394	0.00142675	-1.4830118	3.595270472	up-regulated in High
TBX19	-0.1783886	1.11141129	-4.3040481	2.02E-05	0.00014711	0.91528832	4.694272246	up-regulated in High
XCL1	0.16623761	0.76220535	2.95521098	0.00327332	0.01339872	-3.855507	2.485011485	up-regulated in Low
DPT	-0.6781642	3.35052694	-5.6257241	3.09E-08	3.89E-07	7.16904714	7.509653943	up-regulated in High
ATP1B1	-0.3499708	7.41723862	-3.3855125	0.00076687	0.00376783	-2.5168684	3.115278165	up-regulated in High
SELP	-0.6298202	1.50942532	-9.5741697	4.89E-20	3.23E-18	33.9136378	19.31050514	up-regulated in High
C1orf112	0.48666227	1.35250994	13.3616757	5.38E-35	1.19E-32	68.1340844	34.26915888	up-regulated in Low
SELL	-0.4996523	2.80388755	-4.8741732	1.47E-06	1.36E-05	3.42771034	5.831667283	up-regulated in High

SCYL3	-0.1597716	1.94163093	-4.8094691	2.01E-06	1.80E-05	3.12820979	5.69662591	up-regulated in High
KIFAP3	-0.1210706	3.14465426	-2.7174818	0.00680837	0.02514676	-4.5198284	2.166957123	up-regulated in High
GORAB	-0.1553817	2.45008579	-3.6043217	0.00034461	0.00186477	-1.7696332	3.462669739	up-regulated in High
PRRX1	-0.2068296	2.45072826	-2.4420045	0.01495452	0.04884751	-5.2219231	1.825227661	up-regulated in High
MROH9	-0.1285294	0.13405045	-5.1964899	2.97E-07	3.13E-06	4.97350236	6.526781218	up-regulated in High
FMO3	-0.6157613	1.84013494	-7.9995968	8.90E-15	2.98E-13	21.9400461	14.05074315	up-regulated in High
FMO2	-0.9444629	2.27770667	-10.833542	1.09E-24	1.17E-22	44.5392378	23.96344476	up-regulated in High
FMO4	-0.4432907	1.48408551	-8.7336965	3.80E-17	1.73E-15	27.3269323	16.41993652	up-regulated in High
PRRC2C	0.15765504	4.15013891	2.76549223	0.00589577	0.02226336	-4.3900129	2.229459808	up-regulated in Low
MYOC	-0.1346416	0.12774037	-4.9995637	7.99E-07	7.75E-06	4.01844662	6.097662489	up-regulated in High
VAMP4	-0.1040289	2.45229752	-3.1571294	0.00169038	0.00754272	-3.2490822	2.772014531	up-regulated in High
METTL13	0.15883601	3.74281385	4.04081138	6.17E-05	0.00040349	-0.1470486	4.209491028	up-regulated in Low
DNM3	-0.1426579	0.61329269	-3.1697231	0.00162021	0.00727632	-3.2099831	2.790427733	up-regulated in High
PIGC	0.15311742	3.06663083	3.92616777	9.85E-05	0.00061575	-0.5901054	4.00642474	up-regulated in Low
PRDX6	0.24763364	6.71006016	5.2793418	1.94E-07	2.12E-06	5.3852079	6.711447366	up-regulated in Low
ANKRD45	-0.299216	0.7664853	-4.2590837	2.46E-05	0.00017544	0.72940946	4.60964504	up-regulated in High
KLHL20	-0.217958	2.6565739	-6.0352664	3.11E-09	4.67E-08	9.40613471	8.507020406	up-regulated in High
CENPL	0.528674	1.48666505	13.4455679	2.35E-35	5.33E-33	68.9601672	34.62953379	up-regulated in Low
DARS2	0.62712151	3.37778421	11.9887173	2.95E-29	4.54E-27	54.9852046	28.52946137	up-regulated in Low
RC3H1	-0.1314227	1.98596396	-3.8952099	0.00011158	0.00068758	-0.707693	3.95243202	up-regulated in High
RABGAP1L	-0.362434	2.37317808	-7.7246391	6.27E-14	1.88E-12	20.0158487	13.202998	up-regulated in High
CACYBP	0.53809514	4.13345004	11.6235471	8.75E-28	1.24E-25	51.6169736	27.05789012	up-regulated in Low
TNN	-0.1326445	0.21473042	-6.2899388	6.98E-10	1.16E-08	10.8655788	9.155921288	up-regulated in High
KIAA0040	-0.3504947	3.40103869	-6.6149171	9.66E-11	1.84E-09	12.8021941	10.01521822	up-regulated in High
RALGPS2	0.18099829	2.17728905	3.1318263	0.00183992	0.00812982	-3.327188	2.735200775	up-regulated in Low
ANGPTL1	-0.1889421	0.63948866	-4.3416657	1.72E-05	0.00012686	1.07218764	4.765646337	up-regulated in High
FAM20B	0.21495808	3.61441374	5.38715236	1.11E-07	1.27E-06	5.92963701	6.955376482	up-regulated in Low
TDRD5	0.21366067	0.69646363	2.86007111	0.00441419	0.01735916	-4.1278377	2.355148769	up-regulated in Low
TOR1AIP1	-0.2117336	3.19726982	-5.801254	1.17E-08	1.59E-07	8.1110821	7.930085756	up-regulated in High
QSOX1	-0.3954139	5.85531799	-5.1768611	3.29E-07	3.44E-06	4.87681807	6.483387846	up-regulated in High
LHX4-AS1	0.17189432	2.61876493	5.05714096	6.00E-07	5.96E-06	4.29424703	6.221694914	up-regulated in Low
XPR1	-0.2514342	4.09079285	-2.9488994	0.00333972	0.01364175	-3.8738398	2.476289966	up-regulated in High
STX6	-0.1133492	3.56262803	-3.0186418	0.00266991	0.0112271	-3.6691625	2.573502888	up-regulated in High
MR1	-0.5931599	2.8447918	-11.373972	8.58E-27	1.09E-24	49.3492407	26.06676466	up-regulated in High
IER5	0.23102953	3.77305228	3.3799526	0.00078219	0.00383104	-2.5352745	3.106686809	up-regulated in Low
GLUL	-0.2875509	6.37249424	-4.4577734	1.03E-05	7.92E-05	1.56442722	4.989234957	up-regulated in High
RNASEL	-0.3319883	2.01385741	-7.6460638	1.08E-13	3.14E-12	19.475649	12.96483785	up-regulated in High
RGS16	-0.4228437	3.60796879	-4.4952317	8.66E-06	6.79E-05	1.72579055	5.062426325	up-regulated in High
NPL	-0.2339964	2.56025884	-3.1766547	0.00158275	0.00713495	-3.188399	2.80058812	up-regulated in High
DHX9	0.24109277	5.13167547	6.45637375	2.56E-10	4.58E-09	11.8471064	9.591667233	up-regulated in Low
LAMC1	0.20255909	4.85672616	3.04424423	0.00245667	0.01045158	-3.5928683	2.609653326	up-regulated in Low
LAMC2	0.33388103	4.79616863	2.33059933	0.02017488	0.06252725	-5.4850771	1.695188984	up-regulated in Low
NMNAT2	-0.3786741	1.33334597	-3.2796742	0.00111239	0.00522351	-2.8622803	2.953743238	up-regulated in High
SMG7	0.16649905	3.91637689	3.91695845	0.00010225	0.00063691	-0.6251767	3.990325556	up-regulated in Low
NCF2	-0.3774566	3.72741963	-4.2083728	3.05E-05	0.00021364	0.52195203	4.515100117	up-regulated in High
RGL1	-0.7159516	3.11471363	-10.294157	1.19E-22	1.05E-20	39.8838908	21.92613239	up-regulated in High
APOBEC4	-0.2542734	0.32285662	-4.8549552	1.62E-06	1.48E-05	3.33837476	5.791400256	up-regulated in High
COLGALT2	-0.17846	0.41146722	-3.2049327	0.00143805	0.00656681	-3.0998757	2.842227215	up-regulated in High
TSEN15	0.24022964	4.01246475	4.74173772	2.77E-06	2.40E-05	2.81860413	5.55689009	up-regulated in Low
C1orf21	-0.5821414	2.07944659	-8.4970967	2.29E-16	9.58E-15	25.5520727	15.63993564	up-regulated in High
FAM129A	-0.6250542	3.11923299	-7.0311016	6.83E-12	1.55E-10	15.4008795	11.16566496	up-regulated in High
RNF2	0.20780329	2.90027722	4.75664905	2.59E-06	2.25E-05	2.88642115	5.587510654	up-regulated in Low
HMCN1	-0.618047	1.38389094	-9.3782586	2.40E-19	1.44E-17	32.3401979	18.62052391	up-regulated in High
PRG4	-0.7149585	0.9955503	-7.4014536	5.82E-13	1.51E-11	17.8220325	12.23530924	up-regulated in High
RGS18	-0.2812457	0.92982297	-5.8569681	8.60E-09	1.19E-07	8.41537424	8.065749224	up-regulated in High
RGS1	-0.489403	4.1777441	-4.2097196	3.04E-05	0.00021257	0.52743194	4.517598783	up-regulated in High
RGS13	-0.1645406	0.25318178	-6.6961982	5.81E-11	1.14E-09	13.2993687	10.23553414	up-regulated in High
UCHL5	0.31691298	2.95059747	8.67193417	6.10E-17	2.72E-15	26.8601522	16.21485123	up-regulated in Low
GLRX2	0.38564412	2.70177973	9.19729643	1.02E-18	5.73E-17	30.9070525	17.99179682	up-regulated in Low
CDC73	0.21090601	3.4821019	5.56544901	4.29E-08	5.29E-07	6.85143174	7.367741405	up-regulated in Low
B3GALT2	-0.1852605	0.63100394	-2.7040159	0.00708625	0.02599226	-4.5558426	2.149583606	up-regulated in High
CFH	-0.2868643	3.53864551	-2.8226634	0.00495416	0.01916888	-4.2325535	2.305030275	up-regulated in High
ASPM	1.03334652	1.41376951	17.9839534	4.95E-56	4.25E-53	116.417508	55.30558192	up-regulated in Low
DENND1B	-0.1791101	1.71276521	-4.7277828	2.96E-06	2.55E-05	2.75531284	5.528306576	up-regulated in High
LHX9	-0.1164197	0.26285184	-2.4125702	0.01620264	0.05218	-5.2926176	1.79041427	up-regulated in High
NEK7	-0.2471938	3.82730848	-4.8880238	1.38E-06	1.28E-05	3.49229426	5.86077084	up-regulated in High
PTPRC	-0.5336029	3.15839472	-5.5636136	4.33E-08	5.33E-07	6.84180765	7.363439975	up-regulated in High
KIF14	0.92259056	1.13565375	18.9140594	1.84E-60	2.33E-57	126.596477	59.73583166	up-regulated in Low
ASCL5	-0.1817501	0.53916929	-4.1456694	3.99E-05	0.00027217	0.26863456	4.399515499	up-regulated in High
PHLDA3	-0.7111136	4.31285416	-8.6130534	9.54E-17	4.18E-15	26.4174224	16.02029937	up-regulated in High
CSRP1	-0.2954672	4.62755735	-6.5411799	1.52E-10	2.82E-09	12.3555667	9.817206666	up-regulated in High

NAV1	0.19838102	1.78941438	3.28984177	0.0010738	0.00506797	-2.8295533	2.96907713	up-regulated in Low
IPO9	0.26982597	3.33176729	6.43871462	2.85E-10	5.06E-09	11.7419346	9.54500009	up-regulated in Low
SHISA4	-0.3185199	3.47824004	-4.6962534	3.44E-06	2.92E-05	2.61294244	5.463986547	up-regulated in High
LMOD1	-0.7498466	2.22456777	-9.9992996	1.45E-21	1.15E-19	37.4040438	20.8401196	up-regulated in High
TIMM17A	0.31444393	4.2290654	7.48310026	3.34E-13	8.96E-12	18.3692246	12.47680015	up-regulated in Low
PTPN7	-0.2030845	1.84961457	-2.9374507	0.00346331	0.01408459	-3.906998	2.460508238	up-regulated in High
LGR6	-0.3918039	0.97922201	-4.956558	9.87E-07	9.42E-06	3.81430805	6.005795722	up-regulated in High
UBE2T	1.38322266	3.97989672	17.8111651	3.26E-55	2.57E-52	114.537718	54.48730906	up-regulated in Low
PPP1R12B	-0.5586238	1.90888233	-9.0062242	4.60E-18	2.37E-16	29.415371	17.33709785	up-regulated in High
SYT2	-0.1572077	0.50206251	-2.7122717	0.0069147	0.02548308	-4.5337835	2.160226857	up-regulated in High
CYB5R1	-0.4026613	4.25564851	-7.6335356	1.18E-13	3.40E-12	19.3899212	12.92703565	up-regulated in High
TMEM183A	0.22892972	3.41234053	5.26807726	2.06E-07	2.24E-06	5.32889027	6.686197317	up-regulated in Low
PPFIA4	0.1194588	0.59657367	2.52197475	0.01198208	0.0405607	-5.0256301	1.921467882	up-regulated in Low
ADORA1	-0.3523576	1.37646956	-3.5608663	0.00040529	0.00215239	-1.9215742	3.392238954	up-regulated in High
MYBPH	-0.3421583	0.87658473	-3.2428797	0.00126305	0.00584732	-2.9799022	2.898580281	up-regulated in High
CHI3L1	-0.5846013	4.74614648	-3.7033585	0.00023668	0.00134138	-1.4168261	3.62584416	up-regulated in High
CHIT1	-1.1938731	2.47055105	-7.9477475	1.29E-14	4.24E-13	21.5732011	13.88918905	up-regulated in High
BTG2	-0.8837458	5.4916704	-8.8929143	1.11E-17	5.43E-16	28.5414012	16.95336493	up-regulated in High
FMOD	-0.5223361	4.28101587	-5.7511241	1.55E-08	2.06E-07	7.83945993	7.808930369	up-regulated in High
PRELP	-1.0636931	3.54737214	-10.59114	9.12E-24	9.00E-22	42.4285316	23.03995519	up-regulated in High
ATP2B4	-0.3171596	4.17525949	-4.1807368	3.44E-05	0.00023796	0.40986799	4.463977397	up-regulated in High
LAX1	-0.2796138	1.37135632	-3.5123441	0.0004848	0.00251967	-2.0891613	3.314438186	up-regulated in High
SNRPE	0.46351386	5.13397362	9.50172244	8.83E-20	5.63E-18	33.3291609	19.0542362	up-regulated in Low
SOX13	-0.3256705	2.33918715	-4.6417787	4.43E-06	3.69E-05	2.36901733	5.353710443	up-regulated in High
ETNK2	-0.1593294	1.82229779	-2.0297383	0.04291691	0.11563939	-6.1355592	1.367371513	up-regulated in High
REN	-0.2328155	0.46670366	-4.9611441	9.65E-07	9.23E-06	3.83600122	6.015560683	up-regulated in High
GOLT1A	-0.4123133	3.54125563	-3.6295006	0.00031347	0.00171501	-1.6807958	3.503804463	up-regulated in High
PPP1R15B	0.17785439	4.32101521	4.45720435	1.03E-05	7.94E-05	1.56198561	4.988127104	up-regulated in Low
PIK3C2B	-0.2476771	2.31161034	-3.7792175	0.00017647	0.00103367	-1.1404703	3.753326907	up-regulated in High
MDM4	-0.1495922	2.31985055	-2.8065261	0.00520507	0.02001503	-4.2773146	2.283573807	up-regulated in High
NFASC	-0.1514552	0.47532569	-4.0027423	7.22E-05	0.0004658	-0.2954979	4.14151563	up-regulated in High
RBBP5	0.12266953	3.16278957	3.39302152	0.00074662	0.00368308	-2.491964	3.126899879	up-regulated in Low
DSTYK	-0.1955202	2.14761026	-4.6454525	4.35E-06	3.63E-05	2.38538586	5.361113554	up-regulated in High
TMCC2	-0.1188582	0.84972996	-2.7370599	0.00642186	0.02395073	-4.4671575	2.192339302	up-regulated in High
NUAK2	-0.1932704	2.95580064	-2.7155317	0.00684799	0.02528033	-4.5250548	2.164436781	up-regulated in High
KLHDC8A	-0.2276566	0.60013132	-3.9064465	0.00010666	0.00066048	-0.665114	3.971987999	up-regulated in High
LEMD1	-0.4032423	1.21526243	-4.6355403	4.56E-06	3.78E-05	2.34124989	5.341150851	up-regulated in High
CDK18	-0.5066133	2.54891056	-7.9408683	1.36E-14	4.44E-13	21.5246678	13.86781329	up-regulated in High
MFSD4	-1.247152	2.63393512	-9.3290586	3.56E-19	2.09E-17	31.9486139	18.44876046	up-regulated in High
ELK4	-0.1537099	2.59315488	-3.3561303	0.00085112	0.0041271	-2.6138111	3.070008318	up-regulated in High
SLC45A3	-0.1495755	2.13703967	-2.1293911	0.0337142	0.09516417	-5.9298607	1.472187162	up-regulated in High
NUCKS1	0.1366273	5.71347032	2.77897301	0.00566021	0.02150697	-4.3531658	2.247167242	up-regulated in Low
RAB29	-0.2051611	3.00811334	-3.5150912	0.00047993	0.00249803	-2.0797319	3.318819103	up-regulated in High
SLC41A1	-0.5347825	3.10360105	-8.8914843	1.13E-17	5.48E-16	28.5304225	16.9485438	up-regulated in High
SLC26A9	-1.3054744	2.47129811	-8.8742998	1.29E-17	6.24E-16	28.3985914	16.89065076	up-regulated in High
RAB7B	-0.8557724	2.57669229	-8.4957579	2.31E-16	9.67E-15	25.5421329	15.63556582	up-regulated in High
CTSE	-1.8710316	5.04648901	-8.4401978	3.51E-16	1.44E-14	25.1306504	15.45465178	up-regulated in High
C1orf186	-0.4118591	0.87658726	-6.9832525	9.32E-12	2.06E-10	15.0954564	11.03059	up-regulated in High
FAM72A	0.35317968	0.37149505	19.2170978	6.50E-62	9.98E-59	129.931687	61.18721628	up-regulated in Low
RASSF5	-0.6225086	2.92251899	-10.476224	2.47E-23	2.35E-21	41.4384263	22.60663739	up-regulated in High
DYRK3	-0.2342952	1.57852206	-4.5825124	5.82E-06	4.73E-05	2.1066015	5.234963919	up-regulated in High
MAPKAPK2	-0.1451556	5.48064208	-3.9325384	9.60E-05	0.00060155	-0.5657993	4.017580018	up-regulated in High
IL24	-0.3584234	1.11951256	-6.467321	2.40E-10	4.30E-09	11.9124265	9.620648472	up-regulated in High
FAIM3	-0.7266699	2.42864264	-8.8532797	1.51E-17	7.26E-16	28.2375872	16.81994286	up-regulated in High
PIGR	-2.3900312	5.83460249	-10.35489	7.04E-23	6.39E-21	40.4004955	22.15230152	up-regulated in High
FCAMR	-0.1291735	0.17232696	-5.757412	1.50E-08	1.99E-07	7.87341667	7.824079582	up-regulated in High
C1orf116	-1.8723289	4.81790652	-14.881604	1.13E-41	3.68E-39	83.4457882	40.94551544	up-regulated in High
PFKFB2	-0.2156525	2.40144825	-3.5085224	0.00049165	0.00255139	-2.1022678	3.308348264	up-regulated in High
YOD1	-0.1269934	2.1314325	-2.5495578	0.01108602	0.03799256	-4.9564954	1.955224515	up-regulated in High
C4BPA	-1.769864	5.2043454	-8.9440603	7.48E-18	3.75E-16	28.9349059	17.12615455	up-regulated in High
CD55	-0.7216997	6.31842098	-6.6959029	5.82E-11	1.14E-09	13.2975532	10.2347298	up-regulated in High
CR2	-0.577011	1.35163997	-5.5842516	3.87E-08	4.80E-07	6.95018725	7.411875058	up-regulated in High
CR1	-0.1836071	0.60021811	-4.7277034	2.96E-06	2.55E-05	2.75495356	5.528144302	up-regulated in High
CD46	-0.1452327	5.80911271	-2.4461298	0.01478655	0.04836923	-5.2119482	1.830133064	up-regulated in High
CD34	-0.4672581	2.53486828	-7.6026177	1.47E-13	4.14E-12	19.1788311	12.83394587	up-regulated in High
PLXNA2	-0.7660142	2.43065871	-9.8233699	6.28E-21	4.62E-19	35.9472133	20.2018439	up-regulated in High
CAMK1G	-0.1015431	0.47922589	-4.2257576	2.84E-05	0.00019976	0.59281281	4.547404945	up-regulated in High
TRAF3IP3	-0.3078019	1.14413709	-5.5016425	6.04E-08	7.26E-07	6.5184991	7.218892403	up-regulated in High
C1orf74	0.16286772	1.22993028	5.49914979	6.12E-08	7.35E-07	6.50556119	7.213106097	up-regulated in Low
DIEXF	0.14708627	2.0899613	3.76689543	0.00018515	0.00107921	-1.1857199	3.732472243	up-regulated in Low
SERTAD4	-0.5147186	2.00742353	-5.9967519	3.88E-09	5.73E-08	9.18994145	8.410786325	up-regulated in High

HHAT	-0.2830951	1.59068141	-5.4345114	8.63E-08	1.01E-06	6.17188834	7.063823015	up-regulated in High
RCOR3	-0.1713801	2.45688679	-4.1581849	3.78E-05	0.00025958	0.31891342	4.42246951	up-regulated in High
NEK2	1.44532021	2.32656496	20.0668148	5.34E-66	1.63E-62	139.321412	65.27284988	up-regulated in Low
LPGAT1	0.20425171	3.64507464	3.46669233	0.00057273	0.00291161	-2.2448366	3.242051887	up-regulated in Low
INTS7	0.33036521	2.87524631	7.80246676	3.62E-14	1.12E-12	20.5551766	13.44069981	up-regulated in Low
DTL	0.98710038	1.97921419	17.3556061	4.59E-53	3.08E-50	109.601224	52.33825118	up-regulated in Low
PPP2R5A	-0.3908181	4.58846438	-7.1007489	4.33E-12	1.01E-10	15.8484881	11.36356178	up-regulated in High
TMEM206	0.44644307	2.37421542	9.04451721	3.41E-18	1.78E-16	29.7125271	17.46754445	up-regulated in Low
NENF	-0.1585439	5.56447706	-2.5950515	0.009738	0.03409116	-4.8408694	2.011530386	up-regulated in High
NSL1	0.13594364	3.10448864	2.98046787	0.00301951	0.01248849	-3.7817658	2.520063868	up-regulated in Low
FLVCR1	0.28758379	2.19524767	5.21546899	2.70E-07	2.87E-06	5.06729772	6.568868153	up-regulated in Low
VASH2	0.11415987	0.56349942	3.11518083	0.00194484	0.00853144	-3.3782399	2.711115619	up-regulated in Low
ANGEL2	-0.1117852	2.39800117	-3.2821064	0.00110304	0.00518462	-2.8544605	2.957407672	up-regulated in High
RPS6KC1	0.15814717	2.54414683	3.90511042	0.00010724	0.00066361	-0.6701829	3.969660235	up-regulated in Low
SMYD2	0.15146231	3.33964352	3.50608102	0.00049607	0.00257045	-2.1106335	3.304460735	up-regulated in Low
PTPN14	-0.1713034	1.59335212	-3.4040966	0.00071766	0.00355495	-2.4551362	3.144079785	up-regulated in High
CENPF	1.24400612	2.21815102	16.5212172	3.65E-49	1.82E-46	100.643923	48.43793879	up-regulated in Low
KCTD3	0.51245186	3.30788465	7.36375284	7.51E-13	1.93E-11	17.5709858	12.12448585	up-regulated in Low
RRP15	0.21238133	2.14349833	5.71427611	1.90E-08	2.49E-07	7.64111887	7.720426391	up-regulated in Low
TGFB2	-0.3346662	1.62455347	-3.636266	0.00030556	0.00167726	-1.6568256	3.514897921	up-regulated in High
SLC30A10	0.11300763	0.05936933	4.14201178	4.05E-05	0.00027601	0.2539675	4.392818291	up-regulated in Low
EPRS	0.28842014	5.03013812	5.88003571	7.55E-09	1.05E-07	8.54210286	8.122229563	up-regulated in Low
IARS2	0.25005104	5.23381995	5.26025434	2.14E-07	2.32E-06	5.28984247	6.668688257	up-regulated in Low
MARK1	0.30153232	1.22027653	3.94375893	9.18E-05	0.00057706	-0.522899	4.037264702	up-regulated in Low
2-Mar	-0.6042363	2.74940947	-8.5835591	1.19E-16	5.18E-15	26.1964909	15.92320144	up-regulated in High
1-Mar	-0.226099	1.35759709	-4.6458374	4.35E-06	3.63E-05	2.38710126	5.361889361	up-regulated in High
HLX	-0.19832	1.31889147	-4.2458953	2.60E-05	0.00018468	0.67523335	4.584964931	up-regulated in High
DUSP10	-0.2976643	2.94496694	-4.083697	5.17E-05	0.00034486	0.02175777	4.28671399	up-regulated in High
HHIPL2	0.62518991	1.15129373	4.94561625	1.04E-06	9.89E-06	3.76262473	5.982528519	up-regulated in Low
TAF1A	0.28691954	1.67669639	7.60861624	1.41E-13	3.99E-12	19.2197328	12.85198429	up-regulated in Low
MIA3	-0.1280839	3.44586114	-2.6024266	0.00953384	0.03346673	-4.8219373	2.020732277	up-regulated in High
DISP1	-0.4058576	1.80440996	-7.6410797	1.12E-13	3.24E-12	19.4415306	12.94979338	up-regulated in High
TLR5	-0.6286368	1.78048587	-12.134614	7.51E-30	1.21E-27	56.3469937	29.12425614	up-regulated in High
SUSD4	-0.8230229	1.85355112	-8.741864	3.57E-17	1.63E-15	27.3888421	16.44713465	up-regulated in High
CAPN8	-1.262135	3.653482	-9.2583538	6.27E-19	3.60E-17	31.3884038	18.20299737	up-regulated in High
CAPN2	-0.3431511	5.75223185	-5.3966573	1.05E-07	1.21E-06	5.97810561	6.977078462	up-regulated in High
CNIH3	-0.1863822	0.75681791	-4.04506	6.07E-05	0.00039767	-0.1303996	4.217110813	up-regulated in High
DNAH14	0.13894602	0.61458591	5.05767924	5.99E-07	5.95E-06	4.29683891	6.222860085	up-regulated in Low
LBR	0.4224515	3.88319638	8.43122788	3.75E-16	1.53E-14	25.064408	15.42552448	up-regulated in Low
ENAH	0.12670533	3.49652777	2.20880944	0.02764474	0.08105726	-5.7590011	1.558387545	up-regulated in Low
SRP9	0.21804944	6.61117888	4.44463493	1.09E-05	8.36E-05	1.50812443	4.963685298	up-regulated in Low
EPHX1	-0.6142271	6.73605982	-5.1815106	3.21E-07	3.36E-06	4.89969006	6.493654079	up-regulated in High
TMEM63A	-0.521887	3.51365515	-8.1318071	3.42E-15	1.21E-13	22.8837586	14.46620635	up-regulated in High
LEFTY1	0.10767192	0.26764795	2.30040844	0.02183959	0.06677915	-5.5543226	1.660755621	up-regulated in Low
LEFTY2	-0.3299934	0.44023511	-7.3939282	6.12E-13	1.58E-11	17.7718395	12.21315329	up-regulated in High
LIN9	0.46887144	1.62240348	12.2378693	2.83E-30	4.66E-28	57.3161543	29.54750564	up-regulated in Low
PARP1	0.32426931	4.78862295	6.24308032	9.23E-10	1.50E-08	10.5931734	9.034897153	up-regulated in Low
ITPKB	-0.3398405	2.96037899	-5.3849481	1.12E-07	1.28E-06	5.91840769	6.950348194	up-regulated in High
PSEN2	-0.1289143	3.18094976	-3.1869278	0.00152869	0.00693033	-3.1563265	2.815680059	up-regulated in High
ADCK3	-0.2297164	2.77369244	-3.6572368	0.00028223	0.00156522	-1.5822566	3.549393745	up-regulated in High
CDC42BPA	-0.4844246	2.84364536	-6.3002194	6.57E-10	1.09E-08	10.9255759	9.182571332	up-regulated in High
JMJD4	0.16676078	2.36969177	4.34088085	1.72E-05	0.00012726	1.06890137	4.764151945	up-regulated in Low
WNT3A	-0.1523109	0.32727536	-3.555458	0.0004135	0.00219052	-1.9403617	3.383523267	up-regulated in High
ARF1	0.10763782	7.64987296	2.74615523	0.00624913	0.02340847	-4.4425635	2.204180531	up-regulated in Low
C1orf35	0.19141276	2.66333915	4.08996158	5.03E-05	0.00033678	0.04655581	4.298051755	up-regulated in Low
MRPL55	0.12520661	4.25284053	2.19329983	0.02874967	0.08364177	-5.7928504	1.541367212	up-regulated in Low
GJC2	-0.1552885	0.78169243	-3.3195959	0.00096789	0.00462531	-2.7332249	3.014174962	up-regulated in High
TRIM11	0.12978834	2.38880238	3.04770122	0.00242911	0.01034789	-3.582519	2.614553651	up-regulated in Low
HIST3H2A	0.43009565	2.68932612	2.95472232	0.00327842	0.01341394	-3.8569277	2.484335703	up-regulated in Low
HIST3H2BB	0.1041738	0.45585098	2.14516295	0.0324252	0.09220778	-5.8964179	1.489117334	up-regulated in Low
RNF187	0.12463728	5.29613011	3.03081149	0.00256652	0.01085172	-3.6329748	2.59065534	up-regulated in Low
RHOU	-0.316885	3.72075105	-3.6130717	0.00033347	0.00181122	-1.7388276	3.476937477	up-regulated in High
RAB4A	-0.1591062	3.63968645	-3.8848358	0.0001163	0.00071313	-0.746901	3.934419253	up-regulated in High
CCSAP	0.22440994	1.69376125	5.89357851	6.99E-09	9.82E-08	8.61670604	8.155473422	up-regulated in Low
NUP133	0.11134111	3.37339626	2.78124881	0.0056213	0.02138326	-4.3469282	2.250163381	up-regulated in Low
TAF5L	0.12211703	2.7450632	3.70600896	0.00023428	0.00133	-1.4072597	3.630261846	up-regulated in Low
URB2	0.40561614	2.01601793	10.0894798	6.76E-22	5.51E-20	38.1574622	21.17012927	up-regulated in Low
GALNT2	0.21759469	4.25134048	3.99594078	7.42E-05	0.00047752	-0.3218816	4.129427904	up-regulated in Low
CAPN9	-0.426907	1.59846682	-3.4681391	0.00056973	0.00289889	-2.2399329	3.244333795	up-regulated in High
C1orf198	-0.4921373	4.44981692	-8.7502692	3.35E-17	1.54E-15	27.4525976	16.47514298	up-regulated in High
ARV1	-0.268493	3.48697851	-5.0643082	5.79E-07	5.78E-06	4.32877839	6.237217689	up-regulated in High

C1orf131	0.16484772	1.83924576	5.32540616	1.53E-07	1.70E-06	5.61662637	6.815169713	up-regulated in Low
EXOC8	-0.1783308	2.5219796	-4.536825	7.17E-06	5.72E-05	1.90642442	5.144300567	up-regulated in High
SPRTN	0.12861343	1.76427379	4.25763657	2.47E-05	0.00017643	0.72345709	4.606933748	up-regulated in Low
EGLN1	0.24345374	3.26462723	4.49642367	8.61E-06	6.76E-05	1.73094592	5.064763893	up-regulated in Low
TSNAX	0.11893635	3.14209864	2.58706172	0.00996357	0.03471435	-4.8613202	2.001584949	up-regulated in Low
SIPA1L2	-0.3165317	2.71249448	-3.4394475	0.00063208	0.00317793	-2.3368186	3.199227359	up-regulated in High
MLK4	0.12982751	1.65657254	2.59095782	0.009853	0.03441534	-4.8513553	2.006431694	up-regulated in Low
COA6	0.52216396	4.26830553	8.39351813	4.97E-16	2.00E-14	24.7865004	15.30331759	up-regulated in Low
IRF2BP2	-0.1522158	5.29350623	-3.3920828	0.00074913	0.00369391	-2.4950804	3.125445797	up-regulated in High
TOMM20	0.14594758	6.12217564	2.89204609	0.00399567	0.01593875	-4.0372727	2.398409914	up-regulated in Low
ARID4B	-0.1557654	2.62045777	-3.9126926	0.00010402	0.00064618	-0.6413957	3.982879031	up-regulated in High
TBCE	0.31737102	3.71072115	6.96032162	1.08E-11	2.37E-10	14.9496943	10.96611388	up-regulated in Low
B3GALNT2	0.19443659	1.62220905	4.36497561	1.55E-05	0.00011556	1.17004607	4.810135843	up-regulated in Low
GNG4	0.864592	0.92695491	8.05277295	6.06E-15	2.07E-13	22.3181872	14.21724047	up-regulated in Low
LYST	-0.3255061	1.46604965	-7.3845325	6.52E-13	1.68E-11	17.709229	12.185515	up-regulated in High
ERO1LB	-0.2039988	2.21538778	-1.9901377	0.04712475	0.12453484	-6.2146093	1.326750946	up-regulated in High
EDARADD	0.31001149	0.78420267	4.15777573	3.79E-05	0.00025997	0.31726723	4.421718068	up-regulated in Low
LGALS8	-0.1518749	3.18657714	-3.1009033	0.0020392	0.00888726	-3.4218209	2.690540535	up-regulated in High
HEATR1	0.31251162	2.6757761	6.66497027	7.07E-11	1.37E-09	13.1077557	10.15063641	up-regulated in Low
MTR	-0.2418234	2.46307498	-5.5130034	5.68E-08	6.86E-07	6.57753007	7.245291322	up-regulated in High
RYR2	-0.1263226	0.28394689	-4.121303	4.42E-05	0.0002992	0.17115314	4.354993689	up-regulated in High
CHRM3	0.12461169	0.30528794	3.19121001	0.00150667	0.00683845	-3.1429281	2.821982788	up-regulated in Low
FH	0.32163627	4.93184447	7.58388222	1.67E-13	4.68E-12	19.0512454	12.77767535	up-regulated in Low
OPN3	0.49796947	2.52697282	5.1912895	3.05E-07	3.21E-06	4.94785501	6.515271309	up-regulated in Low
CHML	0.52228716	2.5775387	6.29235003	6.88E-10	1.14E-08	10.8796433	9.16216877	up-regulated in Low
EXO1	1.19265085	1.54826944	21.0175004	1.37E-70	8.87E-67	149.874508	69.86393634	up-regulated in Low
MAP1LC3C	-0.3240333	1.23341466	-3.7036139	0.00023645	0.00134032	-1.4159044	3.626269788	up-regulated in High
PLD5	-0.3033647	0.54224285	-4.9050947	1.27E-06	1.18E-05	3.57212292	5.896736209	up-regulated in High
SDCCAG8	-0.2464255	2.69819887	-6.7132296	5.22E-11	1.03E-09	13.4041869	10.28196907	up-regulated in High
ZBTB18	-0.5824587	3.58513144	-7.1260265	3.67E-12	8.61E-11	16.0118316	11.43576182	up-regulated in High
C1orf101	-0.1254767	0.29882769	-6.7938795	3.14E-11	6.45E-10	13.9035481	10.50312474	up-regulated in High
RP11-11N7.5	-0.2527938	1.40169195	-4.0827993	5.19E-05	0.00034599	0.01820708	4.285090464	up-regulated in High
HNRNPU	0.19718913	5.7507419	4.92835375	1.13E-06	1.07E-05	3.68129586	5.945908087	up-regulated in Low
KIF26B	-0.2376683	1.94825945	-3.0642015	0.00230141	0.00987525	-3.5329658	2.638005553	up-regulated in High
TFB2M	0.21470039	3.79419475	4.30816393	1.99E-05	0.00014466	0.93239354	4.70205607	up-regulated in Low
SCCPDH	0.18639707	4.70787046	2.89239711	0.00399129	0.01592451	-4.036273	2.398886991	up-regulated in Low
AHCTF1	0.23833315	2.67642937	5.87755875	7.65E-09	1.07E-07	8.52847419	8.116156086	up-regulated in Low
ZNF670	0.12180479	1.52547354	2.74995443	0.00617823	0.0231756	-4.432267	2.209136018	up-regulated in Low
ZNF695	0.34421383	0.3536555	10.0975841	6.31E-22	5.17E-20	38.2253891	21.19987966	up-regulated in Low
ZNF670	0.12180479	1.52547354	2.74995443	0.00617823	0.0231756	-4.432267	2.209136018	up-regulated in Low
NLRP3	-0.3158152	1.01121263	-6.4676332	2.39E-10	4.29E-09	11.9142908	9.621475597	up-regulated in High
GCSAML	-0.1387026	0.21915545	-5.3452422	1.38E-07	1.55E-06	5.71683112	6.860064921	up-regulated in High
TRIM58	0.12072864	0.14734294	3.38448887	0.00076967	0.00377954	-2.5202594	3.11369551	up-regulated in Low
LYPD8	0.13677185	0.27171548	3.10169982	0.00203383	0.00886583	-3.4193946	2.691686366	up-regulated in Low
ZNF672	-0.1459286	3.89914435	-3.5574777	0.00041042	0.00217647	-1.933349	3.386776699	up-regulated in High
PGBD2	-0.1182069	1.76763026	-3.051627	0.00239815	0.01023543	-3.5707525	2.620124055	up-regulated in High
SH3YL1	-0.1688947	2.66565157	-3.1083885	0.00198922	0.00869866	-3.3989969	2.701317698	up-regulated in High
ACP1	0.33935755	4.45022423	9.370436	2.55E-19	1.53E-17	32.2778412	18.59317321	up-regulated in Low
FAM150B	-0.2290551	0.37594251	-6.2059942	1.15E-09	1.85E-08	10.3788116	8.939631488	up-regulated in High
TMEM18	0.1136516	2.59879957	3.17327785	0.0016009	0.00720121	-3.1989196	2.795636095	up-regulated in Low
TSSC1	0.40545176	2.62731204	11.5437013	1.82E-27	2.48E-25	50.8883749	26.73948496	up-regulated in Low
RNASEH1	0.4082779	2.81627575	10.7253615	2.82E-24	2.91E-22	43.5935825	23.5497377	up-regulated in Low
RPS7	0.33104973	6.37318344	4.91408331	1.21E-06	1.14E-05	3.61425813	5.915715965	up-regulated in Low
COLEC11	0.19878313	0.7010234	3.10776127	0.00199336	0.00871345	-3.4009115	2.700413774	up-regulated in Low
RSAD2	-0.2236947	2.23067034	-2.5406074	0.01137001	0.03882908	-4.9790093	1.944239203	up-regulated in High
KIDINS220	-0.2354634	3.21585619	-4.8076913	2.03E-06	1.81E-05	3.12003242	5.692937016	up-regulated in High
MBOAT2	-0.1831996	2.91302662	-2.304239	0.02162194	0.06624895	-5.5455858	1.665105426	up-regulated in High
ASAP2	0.3143222	1.55434906	4.13702955	4.13E-05	0.00028144	0.23400817	4.383703669	up-regulated in Low
ITGB1BP1	0.20709256	2.84183405	5.36126222	1.27E-07	1.43E-06	5.7980007	6.896424405	up-regulated in Low
CPSF3	0.41777211	3.7780579	11.5938099	1.15E-27	1.60E-25	51.3452832	26.93916216	up-regulated in Low
IAH1	0.11409315	2.74279137	2.77995402	0.00564341	0.02145752	-4.3504776	2.248458514	up-regulated in Low
ADAM17	0.29204992	3.01382978	5.33128454	1.48E-07	1.66E-06	5.64628725	6.828459861	up-regulated in Low
YWHAQ	0.39301787	6.60549977	8.18449044	2.33E-15	8.52E-14	23.2631072	14.63315868	up-regulated in Low
TAF1B	0.11942781	2.17796522	2.93427436	0.00349834	0.0142092	-3.9161753	2.456138623	up-regulated in Low
GRHL1	-0.1969498	1.84767771	-2.4926856	0.01300353	0.04352425	-5.0982385	1.8859388	up-regulated in High
KLF11	-0.1958466	2.67399773	-3.4705125	0.00056483	0.00287649	-2.2318838	3.248079102	up-regulated in High
CYS1	-0.5893525	1.31294407	-6.440255	2.82E-10	5.02E-09	11.751099	9.549066794	up-regulated in High
RRM2	1.55705751	3.23099326	18.847813	3.81E-60	4.45E-57	125.868484	59.41901737	up-regulated in Low
HPCAL1	-0.1598321	3.71852968	-2.6952787	0.00727197	0.02654343	-4.5791172	2.138347784	up-regulated in High
ODC1	0.45374731	5.71139516	3.28818181	0.00108001	0.00509442	-2.834903	2.966571029	up-regulated in Low
NOL10	0.39732052	3.34070016	11.6488669	6.93E-28	9.85E-26	51.8486181	27.15911483	up-regulated in Low

PDIA6	0.34142619	6.15248088	5.75684448	1.50E-08	2.00E-07	7.87035075	7.822711808	up-regulated in Low
KCNF1	0.39267233	0.46153587	6.48655372	2.13E-10	3.85E-09	12.0274111	9.671659878	up-regulated in Low
PQLC3	-0.2989286	3.97135097	-5.382634	1.13E-07	1.29E-06	5.90662335	6.945071244	up-regulated in High
E2F6	0.28462597	2.1438986	7.94734584	1.29E-14	4.25E-13	21.5703667	13.88794069	up-regulated in Low
GREB1	-0.1704429	0.71153179	-2.6279551	0.00885639	0.03141353	-4.7560019	2.052743143	up-regulated in High
TRIB2	-0.1979224	3.37567094	-2.3911428	0.01716813	0.05471293	-5.3435546	1.765277055	up-regulated in High
DDX1	0.31107629	4.82429893	8.53075928	1.78E-16	7.52E-15	25.8023887	15.74997527	up-regulated in Low
MYCN	0.33933329	0.80621806	3.02069612	0.0026522	0.01116385	-3.6630637	2.576394364	up-regulated in Low
FAM49A	-0.4624204	1.95577578	-7.5091352	2.79E-13	7.59E-12	18.5447158	12.55423098	up-regulated in High
SMC6	0.26826599	2.14683011	6.33582733	5.31E-10	9.00E-09	11.134027	9.275147869	up-regulated in Low
GEN1	0.37945809	1.13019076	11.3813106	8.02E-27	1.03E-24	49.4155142	26.09573421	up-regulated in Low
KCNS3	-0.5417515	4.07231216	-5.9599567	4.79E-09	6.96E-08	8.9845173	8.319317828	up-regulated in High
OSR1	-0.3396911	1.00333084	-6.4298713	3.01E-10	5.31E-09	11.6893583	9.521668742	up-regulated in High
TTC32	0.22331754	1.9666997	3.96996457	8.25E-05	0.00052515	-0.4222583	4.083421728	up-regulated in Low
MATN3	-0.5945337	1.75859605	-6.8891084	1.71E-11	3.64E-10	14.4995366	10.76694151	up-regulated in High
LAPTM4A	-0.2227394	7.45463607	-5.5122915	5.71E-08	6.89E-07	6.5738281	7.243635874	up-regulated in High
SDC1	-0.6459016	7.34793276	-8.0631616	5.63E-15	1.93E-13	22.3922867	14.24986313	up-regulated in High
RHOB	-0.2798427	6.92197764	-2.9223752	0.00363243	0.01467726	-3.9504699	2.43980321	up-regulated in High
GDF7	-0.1544875	0.2339075	-5.9908388	4.02E-09	5.91E-08	9.15685534	8.396056053	up-regulated in High
C2orf43	0.18784514	2.43832599	4.82525226	1.86E-06	1.68E-05	3.2009307	5.729426764	up-regulated in Low
KLHL29	-0.1412019	0.75789447	-3.1791996	0.0015692	0.00708589	-3.1804631	2.80432304	up-regulated in High
UBXN2A	0.16753542	2.10260911	5.14140619	3.93E-07	4.04E-06	4.70301355	6.405355412	up-regulated in Low
MFSD2B	0.11169096	0.1956591	9.17132893	1.25E-18	6.96E-17	30.7030185	17.90226438	up-regulated in Low
C2orf44	0.20691393	1.89487243	6.05733679	2.74E-09	4.15E-08	9.53056103	8.562392816	up-regulated in Low
SF3B6	0.37322227	5.77096714	9.11760715	1.92E-18	1.04E-16	30.2822125	17.71759209	up-regulated in Low
ITSN2	-0.2293995	2.75973505	-5.4792024	6.81E-08	8.10E-07	6.40221692	7.166881372	up-regulated in High
CENPO	0.65974555	1.7234229	16.5816293	1.91E-49	9.71E-47	101.288398	48.71860453	up-regulated in Low
ADCY3	0.25978925	2.54299538	4.416581	1.23E-05	9.38E-05	1.38841736	4.909342706	up-regulated in Low
DNAJC27	-0.1093173	1.05594344	-3.9793092	7.94E-05	0.00050782	-0.3862198	4.099942822	up-regulated in High
DNMT3A	0.35653787	2.41204988	6.93660909	1.26E-11	2.73E-10	14.7993779	10.89961492	up-regulated in Low
KIF3C	0.48313452	1.87904221	6.23447257	9.71E-10	1.58E-08	10.5433224	9.012744916	up-regulated in Low
RAB10	0.48850588	5.65314555	11.4047467	6.48E-27	8.35E-25	49.6273267	26.18832022	up-regulated in Low
GAREML	0.37390837	1.23509524	6.04551833	2.93E-09	4.42E-08	9.46388346	8.532721064	up-regulated in Low
HADHA	0.14664183	5.7193373	4.81587567	1.95E-06	1.75E-05	3.15770194	5.70992931	up-regulated in Low
EPT1	0.71309999	2.85341304	13.5228626	1.09E-35	2.55E-33	69.7234098	34.96247464	up-regulated in Low
DRC1	-0.341543	0.76791383	-4.0955485	4.92E-05	0.00032974	0.06870145	4.308175455	up-regulated in High
KCNK3	-0.2626458	1.24436638	-2.7706028	0.00580544	0.02198058	-4.3760647	2.236164541	up-regulated in High
SLC35F6	0.16597163	4.15485461	4.19561742	3.23E-05	0.00022475	0.47013426	4.491469253	up-regulated in Low
CENPA	1.36789873	1.84561366	20.0914474	4.06E-66	1.32E-62	139.59431	65.39158275	up-regulated in Low
DPYSL5	0.19570915	0.09682932	4.32723055	1.83E-05	0.00013435	1.01183056	4.738195973	up-regulated in Low
MAPRE3	-0.3248766	2.67451615	-5.3192814	1.58E-07	1.75E-06	5.58575337	6.801335504	up-regulated in High
AGBL5	0.2422924	2.89912594	5.39620508	1.06E-07	1.21E-06	5.97579789	6.976045228	up-regulated in Low
OST4	0.12539798	7.55419244	2.53041343	0.01170133	0.03976446	-5.004557	1.931764667	up-regulated in Low
EMILIN1	-0.2563187	4.33627047	-2.6219856	0.00901082	0.03187609	-4.7714763	2.045235601	up-regulated in High
KHK	0.18226489	1.46746228	3.79923675	0.00016318	0.00096366	-1.0666574	3.787330286	up-regulated in Low
CGREF1	0.4154136	1.14917127	4.50753359	8.19E-06	6.45E-05	1.77905642	5.086575922	up-regulated in Low
PREB	0.33720814	4.54577883	9.1537463	1.44E-18	7.94E-17	30.5650992	17.84174063	up-regulated in Low
SLC5A6	0.41873908	2.89455859	9.23666754	7.45E-19	4.25E-17	31.2171809	18.12787408	up-regulated in Low
CAD	0.53070055	3.16242624	9.01864316	4.17E-18	2.17E-16	29.5116436	17.3793613	up-regulated in Low
TRIM54	-0.1935405	0.64677198	-2.5208683	0.01201933	0.04066596	-5.028388	1.92011982	up-regulated in High
GTF3C2	0.28421346	3.7227056	8.22622594	1.71E-15	6.38E-14	23.5649493	14.76597908	up-regulated in Low
EIF2B4	0.100572	3.09871771	3.22132629	0.00135986	0.00625276	-3.0482119	2.866505402	up-regulated in Low
SNX17	0.11347106	5.10632063	3.28712735	0.00108398	0.00511023	-2.8382999	2.964979628	up-regulated in Low
PPM1G	0.50492943	5.20290364	13.9496755	1.53E-37	3.93E-35	73.9734648	36.81609015	up-regulated in Low
NRBP1	0.21402771	4.5188085	5.95050952	5.06E-09	7.31E-08	8.93195087	8.29590728	up-regulated in Low
IFT172	-0.2577523	2.13530051	-4.8421379	1.72E-06	1.56E-05	3.27897114	5.764618493	up-regulated in High
ZNF512	-0.1755135	2.77595952	-3.7569825	0.00019243	0.00111654	-1.2220214	3.715736345	up-regulated in High
GPN1	0.37665383	3.79514605	8.48315604	2.54E-16	1.06E-14	25.4486257	15.59445676	up-regulated in Low
SUPT7L	0.14954698	3.41335943	4.33631787	1.76E-05	0.00012951	1.04980538	4.755467811	up-regulated in Low
SLC4A1AP	0.20506907	2.94608608	6.16887573	1.43E-09	2.27E-08	10.1653579	8.844743354	up-regulated in Low
MRPL33	0.14994202	4.65087288	2.70284807	0.00711082	0.02605785	-4.5589579	2.148080077	up-regulated in Low
RBKS	-0.1376914	1.51930545	-3.058776	0.0023427	0.01002076	-3.5492882	2.63028277	up-regulated in High
PLB1	-0.1688374	0.69247513	-5.1427254	3.91E-07	4.02E-06	4.70946131	6.408250851	up-regulated in High
PPP1CB	0.14702346	5.74477688	3.36397493	0.00082782	0.00402549	-2.5880077	3.08206275	up-regulated in Low
TRMT61B	0.23558305	2.61864542	6.34583861	5.00E-10	8.51E-09	11.1928135	9.301251753	up-regulated in Low
WDR43	0.55290289	3.69634206	12.5278424	1.79E-31	3.22E-29	60.0611147	30.74604229	up-regulated in Low
FAM179A	-0.1484254	0.22855459	-4.7994625	2.11E-06	1.88E-05	3.08221663	5.675876648	up-regulated in High
YPEL5	-0.3159324	5.14074602	-7.3999607	5.88E-13	1.52E-11	17.8120719	12.23091255	up-regulated in High
LBH	-0.5419727	4.88488185	-7.1060591	4.18E-12	9.75E-11	15.8827632	11.37871264	up-regulated in High
LCLAT1	0.30218202	1.96074977	8.19206084	2.20E-15	8.10E-14	23.3177716	14.65721417	up-regulated in Low
CAPN13	-0.4595721	2.59078202	-3.3981579	0.00073306	0.00362168	-2.4748982	3.134861821	up-regulated in High

GALNT14	0.45015644	2.03233359	3.88287055	0.00011722	0.00071807	-0.7543175	3.931011403	up-regulated in Low
SRD5A2	-0.2056337	0.5013229	-3.0974012	0.00206298	0.00898017	-3.4324812	2.685505607	up-regulated in High
DPY30	0.26851026	4.17478735	5.72830401	1.76E-08	2.31E-07	7.71649493	7.754064336	up-regulated in Low
SPAST	0.17329537	2.75614082	4.21765212	2.94E-05	0.00020625	0.55974088	4.532329212	up-regulated in Low
SLC30A6	0.19489819	3.2004935	5.16055293	3.57E-07	3.71E-06	4.79674019	6.447439789	up-regulated in Low
NLRC4	-0.2496306	1.06585297	-5.7777874	1.34E-08	1.80E-07	7.98367509	7.873263488	up-regulated in High
TTC27	0.28095879	2.91250861	7.69383506	7.77E-14	2.30E-12	19.8035554	13.10941233	up-regulated in Low
RASGRP3	-0.1780286	1.29059883	-4.5585004	6.50E-06	5.23E-05	2.00116402	5.187218518	up-regulated in High
FAM98A	0.2155654	3.33932041	6.30169419	6.51E-10	1.09E-08	10.9341898	9.186397357	up-regulated in Low
CRIM1	-0.2759925	3.49947634	-3.6412137	0.0002999	0.00165239	-1.6392688	3.523021732	up-regulated in High
FEZ2	-0.1147404	2.65846852	-3.0596328	0.00233614	0.01	-3.5467123	2.631501667	up-regulated in High
STRN	0.13481342	2.60223876	3.20587213	0.00143346	0.00655085	-3.0969221	2.843615663	up-regulated in Low
EIF2AK2	0.18926881	3.16287234	3.84185101	0.00013797	0.00083117	-0.9083103	3.860212945	up-regulated in Low
CEBPZOS	0.24791218	3.5599685	5.90065395	6.72E-09	9.47E-08	8.65574182	8.172866585	up-regulated in Low
CEBPZ	0.26003721	4.00480756	7.00322907	8.19E-12	1.83E-10	15.2227599	11.08689477	up-regulated in Low
QPCT	0.41134431	3.30196634	2.58050518	0.01015216	0.03525989	-4.8780566	1.99344162	up-regulated in Low
CDC42EP3	-0.4905733	3.06703454	-6.3246955	5.67E-10	9.56E-09	11.0687532	9.24616122	up-regulated in High
RMDN2	-0.5160647	1.32546306	-10.369862	6.19E-23	5.68E-21	40.5281556	22.20818742	up-regulated in High
CYP1B1	-0.4637363	3.39365977	-4.5227623	7.65E-06	6.07E-05	1.84518065	5.116547899	up-regulated in High
HNRNPLL	0.1308852	2.95527966	3.8522919	0.00013238	0.00080064	-0.8692599	3.878173769	up-regulated in Low
SRSF7	0.21525316	4.8795168	6.21654081	1.08E-09	1.74E-08	10.4396612	8.966676617	up-regulated in Low
GEMIN6	0.26727331	2.14297948	7.26698334	1.44E-12	3.56E-11	16.931325	11.84202329	up-regulated in Low
DHX57	0.20442558	2.19503859	5.82205587	1.05E-08	1.43E-07	8.2243985	7.980613745	up-regulated in Low
MORN2	0.12634079	3.41028951	2.25568781	0.02452548	0.07357015	-5.6552688	1.610382444	up-regulated in Low
THUMPD2	0.13235233	2.05012837	4.06158132	5.66E-05	0.00037459	-0.0655022	4.246805291	up-regulated in Low
COX7A2L	0.27398927	4.21627029	6.09844146	2.16E-09	3.34E-08	9.76333936	8.665958348	up-regulated in Low
KCNG3	0.10184858	0.31109266	2.96754161	0.00314707	0.01294725	-3.8195815	2.502094088	up-regulated in Low
MTA3	0.2511038	2.39725818	6.50712917	1.88E-10	3.43E-09	12.1507419	9.726366785	up-regulated in Low
OXER1	-0.1626319	0.89544711	-3.1723362	0.00160599	0.00722135	-3.2018513	2.794256001	up-regulated in High
HAAO	-0.2445881	1.41140381	-5.1513375	3.74E-07	3.87E-06	4.75159021	6.427168155	up-regulated in High
ZFP36L2	-0.3268863	5.47713775	-4.9396752	1.07E-06	1.02E-05	3.73460577	5.969913248	up-regulated in High
PLEKHH2	-0.5459589	1.48104382	-8.4613974	3.00E-16	1.23E-14	25.2874185	15.52358071	up-regulated in High
DYNC2LI1	-0.1584829	2.22607582	-3.9987945	7.34E-05	0.00047261	-0.3108169	4.134497434	up-regulated in High
LRPPRC	0.47147858	4.05541699	11.1339573	7.49E-26	8.93E-24	47.1956801	25.12524625	up-regulated in Low
PREPL	-0.2123016	2.90916846	-4.8485759	1.67E-06	1.52E-05	3.30879117	5.7780633	up-regulated in High
SIX3	0.17512571	0.26659342	3.34325221	0.0008907	0.00429724	-2.6560461	3.050269825	up-regulated in Low
PRKCE	-0.3116412	1.42667967	-8.4889947	2.44E-16	1.01E-14	25.491936	15.61349769	up-regulated in High
EPAS1	-0.3012393	5.55443908	-4.0172128	6.80E-05	0.00044174	-0.2392256	4.167290209	up-regulated in High
ATP6V1E2	0.13990197	1.24970682	3.69247266	0.00024676	0.00139149	-1.4560486	3.607727828	up-regulated in Low
PIGF	0.14970392	1.89290008	4.24172209	2.65E-05	0.00018771	0.65812318	4.577168934	up-regulated in Low
CRIPT	0.23012679	3.21619247	5.03119879	6.83E-07	6.71E-06	4.16962873	6.165663513	up-regulated in Low
SOCS5	-0.1453735	2.66668693	-4.0030951	7.21E-05	0.00046523	-0.294128	4.142143165	up-regulated in High
MCFD2	0.12294511	4.56427651	2.76235901	0.00595177	0.02244725	-4.3985521	2.2253541	up-regulated in Low
MSH2	0.57265588	3.24673022	10.9980382	2.53E-25	2.92E-23	45.9883362	24.59727246	up-regulated in Low
MSH6	0.4412503	2.84516244	9.40746628	1.89E-19	1.16E-17	32.5733429	18.72278101	up-regulated in Low
FOXN2	0.13434587	2.44371808	2.8693673	0.00428858	0.01694279	-4.1016078	2.367686182	up-regulated in Low
PPP1R21	-0.1584945	3.03393576	-4.2158365	2.96E-05	0.00020761	0.55234097	4.528955634	up-regulated in High
STON1	-0.1120459	0.99374338	-2.7490678	0.00619471	0.02323295	-4.4346711	2.207979064	up-regulated in High
CHAC2	0.50947564	1.89962845	9.4034142	1.96E-19	1.19E-17	32.5409677	18.70858166	up-regulated in Low
PSME4	0.33026937	3.3588504	7.93418339	1.42E-14	4.64E-13	21.4775368	13.84705462	up-regulated in Low
ACYP2	-0.11609	1.3983418	-3.5198554	0.0004716	0.00246028	-2.0633618	3.326423632	up-regulated in High
C2orf73	-0.1300874	0.17734402	-4.7418004	2.77E-06	2.40E-05	2.81888859	5.557018546	up-regulated in High
RPS27A	0.20524507	7.12134029	4.1557235	3.82E-05	0.00026196	0.30901387	4.417950528	up-regulated in Low
MTIF2	0.39186821	3.58399136	11.4477974	4.38E-27	5.72E-25	50.0170748	26.35867706	up-regulated in Low
CCDC88A	0.13723372	1.74366526	2.51991319	0.01205157	0.04075851	-5.0307678	1.918956477	up-regulated in Low
SMEK2	0.11397022	3.8526619	3.38469851	0.0007691	0.00377767	-2.519565	3.114019608	up-regulated in Low
PNPT1	0.50207371	3.10558291	11.0454358	1.66E-25	1.93E-23	46.4083436	24.78095376	up-regulated in Low
EFEMP1	-0.6905461	4.51806602	-6.8202292	2.66E-11	5.52E-10	14.06777	10.57583241	up-regulated in High
CCDC85A	-0.1432867	0.35891767	-5.0594653	5.93E-07	5.90E-06	4.30544064	6.226726902	up-regulated in High
VRK2	0.32077624	2.67751625	7.71994764	6.48E-14	1.93E-12	19.9834733	13.18872662	up-regulated in Low
FANCL	0.28731753	2.94405367	6.82397291	2.59E-11	5.40E-10	14.091145	10.58618059	up-regulated in Low
REL	-0.2603251	1.20587675	-5.835014	9.72E-09	1.33E-07	8.29516545	8.012164139	up-regulated in High
PUS10	-0.2780533	1.30931667	-9.1676464	1.29E-18	7.15E-17	30.6741169	17.88958158	up-regulated in High
KIAA1841	0.11365724	1.58996922	2.42174153	0.01580424	0.05114522	-5.27068	1.801226477	up-regulated in Low
C2orf74	-0.19988	1.28301508	-4.4530371	1.05E-05	8.08E-05	1.54411305	4.980017294	up-regulated in High
AHSA2	-0.1805493	2.34257229	-2.2221025	0.02672714	0.07888573	-5.729803	1.573047496	up-regulated in High
XPO1	0.35160709	4.07829062	8.86310682	1.40E-17	6.75E-16	28.3128238	16.85298486	up-regulated in Low
FAM161A	0.17767525	1.14174932	4.23594705	2.71E-05	0.00019194	0.63447112	4.566391095	up-regulated in Low
CCT4	0.53422966	5.87777425	11.8573498	1.01E-28	1.49E-26	53.7667966	27.99721209	up-regulated in Low
TMEM17	0.13703838	1.18493581	3.36429272	0.00082689	0.00402197	-2.5869612	3.082551584	up-regulated in Low
EHBP1	0.43842522	2.43449879	9.34768607	3.06E-19	1.82E-17	32.0967005	18.51371909	up-regulated in Low

OTX1	0.39398741	1.10351514	6.88013808	1.81E-11	3.85E-10	14.4431036	10.74196723	up-regulated in Low
MDH1	0.29538985	4.533254	8.30039249	9.93E-16	3.80E-14	24.1042138	15.00322765	up-regulated in Low
PELI1	-0.4165821	3.93638235	-5.9201534	6.01E-09	8.57E-08	8.76353238	8.220889365	up-regulated in High
LGALSL	-0.2965855	2.44883102	-4.1576576	3.79E-05	0.00026007	0.31679198	4.421501128	up-regulated in High
CEP68	-0.2247855	2.00614125	-6.42293	3.14E-10	5.53E-09	11.6481325	9.503373346	up-regulated in High
RAB1A	0.11521521	6.15323191	3.69647819	0.000243	0.00137323	-1.4416291	3.61438868	up-regulated in Low
ACTR2	0.13211892	6.22406194	3.24011677	0.00127509	0.00589693	-2.9886833	2.894458729	up-regulated in Low
SPRED2	-0.34337	3.71537008	-6.0136833	3.52E-09	5.24E-08	9.28483524	8.453030077	up-regulated in High
MEIS1	-0.2373111	1.14714769	-5.4957679	6.23E-08	7.47E-07	6.48801685	7.205259385	up-regulated in High
C1D	0.10804042	2.47494116	2.85326107	0.00450832	0.01767403	-4.1470004	2.34598517	up-regulated in Low
PNO1	0.43484612	3.5546176	9.78801809	8.42E-21	6.04E-19	35.6565709	20.07447968	up-regulated in Low
PPP3R1	0.20772638	4.16142183	6.11181328	2.00E-09	3.10E-08	9.8393568	8.699772116	up-regulated in Low
CNRIP1	-0.3867294	1.53921389	-8.1635603	2.71E-15	9.83E-14	23.1121751	14.56673664	up-regulated in High
PLEK	-0.3902373	3.44707302	-4.0517738	5.90E-05	0.00038796	-0.1040571	4.22916548	up-regulated in High
ARHGAP25	-0.3880873	2.12743884	-6.7003662	5.66E-11	1.11E-09	13.325	10.24688932	up-regulated in High
GKN2	-1.0090778	1.45264	-6.3941746	3.73E-10	6.49E-09	11.4777519	9.427751815	up-regulated in High
ANTXR1	-0.4140174	4.08697712	-4.1627694	3.71E-05	0.00025502	0.33736609	4.430892198	up-regulated in High
NFU1	0.17256699	3.71145817	3.92111655	0.00010056	0.00062746	-0.6093512	3.997590573	up-regulated in Low
ANXA4	-0.2400228	3.88270614	-3.4912842	0.00052366	0.00269219	-2.1612178	3.28094735	up-regulated in High
SNRNP27	0.19900708	3.76952422	6.06471975	2.62E-09	3.99E-08	9.57227129	8.580952614	up-regulated in Low
MXD1	0.25134507	2.42934223	4.00588681	7.13E-05	0.0004606	-0.2832859	4.147109905	up-regulated in Low
PCBP1	0.17696329	6.63861775	4.93558335	1.09E-06	1.03E-05	3.71532534	5.961231812	up-regulated in Low
PCYOX1	-0.2727123	4.44595439	-5.082174	5.30E-07	5.32E-06	4.4150459	6.275990964	up-regulated in High
SNRPG	0.56514732	4.61107453	9.8038429	7.39E-21	5.36E-19	35.7865862	20.13145559	up-regulated in Low
FAM136A	0.50392897	4.06182051	12.6666441	4.73E-32	8.86E-30	61.3868667	31.32478903	up-regulated in Low
ADD2	0.13520138	0.36383749	2.76939481	0.00582668	0.02204668	-4.3793638	2.234578867	up-regulated in Low
CLEC4F	-0.3066802	0.33517209	-9.4139325	1.80E-19	1.10E-17	32.6250263	18.74544837	up-regulated in High
CD207	-1.5036133	1.82972795	-10.666676	4.72E-24	4.81E-22	43.083063	23.32636655	up-regulated in High
VAX2	0.2547068	0.873301	4.19253949	3.27E-05	0.00022737	0.45765231	4.485776045	up-regulated in Low
MCEE	-0.1447986	2.80035208	-3.147158	0.00174792	0.00776918	-3.2799342	2.757477998	up-regulated in High
MPHOSPH1(	0.21070477	3.31146931	6.55006172	1.44E-10	2.68E-09	12.4091415	9.840963835	up-regulated in Low
SFXN5	0.1891591	1.28682489	5.57863299	3.99E-08	4.94E-07	6.92064615	7.39867406	up-regulated in Low
RAB11FIP5	-0.2023947	3.06908504	-4.0663296	5.55E-05	0.00036786	-0.0468045	4.255358481	up-regulated in High
SMYD5	0.17571856	3.59214761	4.92408873	1.16E-06	1.09E-05	3.66124177	5.93687686	up-regulated in Low
PRADC1	0.13887868	3.46692395	2.42797903	0.01553824	0.05041854	-5.2557136	1.808598146	up-regulated in Low
CCT7	0.47954652	5.98884694	14.2852076	5.12E-39	1.43E-36	77.3548272	38.29045367	up-regulated in Low
FBXO41	0.12120669	1.45651638	2.03247412	0.04263836	0.1150428	-6.1300414	1.370199476	up-regulated in Low
TPRKB	0.4033595	3.17344454	10.141312	4.36E-22	3.64E-20	38.592516	21.36066524	up-regulated in Low
DUSP11	0.12377529	3.00190556	3.52255801	0.00046694	0.00243834	-2.054066	3.330741342	up-regulated in Low
STAMBP	0.23085936	2.8624546	7.95394398	1.23E-14	4.07E-13	21.6169457	13.90845524	up-regulated in Low
ACTG2	-0.4876627	2.11612972	-4.9854299	8.56E-07	8.26E-06	3.95118076	6.067397326	up-regulated in High
DGUOK	0.28695589	4.88300772	7.11937701	3.83E-12	8.98E-11	15.9688166	11.41674953	up-regulated in Low
TET3	0.20082892	1.96724307	3.99015165	7.60E-05	0.00048767	-0.344305	4.119153013	up-regulated in Low
BOLA3	0.4956669	2.40563691	10.6384935	6.03E-24	6.09E-22	42.8385151	23.21936065	up-regulated in Low
MOB1A	0.14227815	4.7601596	3.85160767	0.00013274	0.00080223	-0.8718221	3.876995472	up-regulated in Low
MTHFD2	1.05594351	4.0277729	15.4112547	4.60E-44	1.73E-41	88.9341647	43.33716981	up-regulated in Low
SLC4A5	-0.257065	0.43314633	-6.5895544	1.13E-10	2.14E-09	12.6480982	9.94691037	up-regulated in High
C2orf81	-0.2712273	1.73449448	-4.3479292	1.67E-05	0.0001237	1.09843504	4.777581216	up-regulated in High
RTKN	0.2580834	3.17457209	5.22394798	2.58E-07	2.76E-06	5.10930026	6.587712018	up-regulated in Low
WBP1	-0.1490757	2.62612722	-2.9731118	0.00309151	0.01274564	-3.8033053	2.509829902	up-regulated in High
MOGS	0.11345425	4.82180462	2.74170466	0.00633312	0.02367934	-4.4546079	2.198382394	up-regulated in Low
MRPL53	0.1303267	1.67314477	3.44842064	0.00061193	0.00308964	-2.3066006	3.213300681	up-regulated in Low
CCDC142	0.10141466	1.26810871	2.9845751	0.00297998	0.01234686	-3.7697168	2.525786938	up-regulated in Low
AUP1	0.18558646	5.90451257	4.41236309	1.26E-05	9.53E-05	1.37047995	4.901197331	up-regulated in Low
HTRA2	0.1333554	3.12114709	3.70627282	0.00023404	0.00132878	-1.406307	3.630701776	up-regulated in Low
DOK1	-0.3078388	2.81671313	-6.4492106	2.67E-10	4.77E-09	11.8044159	9.572725122	up-regulated in High
SEMA4F	-0.1179964	1.86167178	-2.4432801	0.0149024	0.04869091	-5.2188406	1.826743761	up-regulated in High
HK2	0.39183991	3.58397526	4.03364224	6.36E-05	0.00041471	-0.1751051	4.196648628	up-regulated in Low
POLE4	0.30060237	3.9277605	5.49347015	6.31E-08	7.55E-07	6.47610204	7.19993033	up-regulated in Low
EVA1A	-0.6957271	3.14074453	-7.0507008	6.01E-12	1.37E-10	15.5264745	11.22120023	up-regulated in High
MRPL19	0.36519031	3.29914131	10.2779707	1.36E-22	1.19E-20	39.7465417	21.86599715	up-regulated in Low
GCFC2	0.23809039	2.16624285	6.65537715	7.51E-11	1.45E-09	13.0490431	10.12461946	up-regulated in Low
SUCLG1	0.14254507	4.01947997	3.58263825	0.00037373	0.0020003	-1.8456678	3.427436982	up-regulated in Low
DNAH6	-0.2133574	0.31979139	-5.686378	2.22E-08	2.86E-07	7.49169571	7.65373051	up-regulated in High
TRABD2A	-0.2229474	0.84509232	-3.8447458	0.0001364	0.00082238	-0.8974934	3.865188567	up-regulated in High
TMSB10	0.34999975	11.3423784	4.87369357	1.48E-06	1.36E-05	3.42547699	5.830660763	up-regulated in Low
KCMF1	0.35194474	3.18723406	11.4094177	6.21E-27	8.02E-25	49.6695729	26.20678625	up-regulated in Low
TCF7L1	-0.3175644	2.58296935	-3.3673999	0.00081784	0.00398457	-2.576724	3.087333015	up-regulated in High
RETSAT	-0.1870819	4.2171434	-3.6497918	0.00029032	0.00160473	-1.6087763	3.537128212	up-regulated in High
ELMOD3	-0.1358364	1.8082864	-3.9750421	8.08E-05	0.00051588	-0.402686	4.092394707	up-regulated in High
CAPG	-0.2131131	6.29893484	-2.9022515	0.00386991	0.01549848	-4.0081621	2.41229933	up-regulated in High

GGCX	-0.3048386	3.56035078	-4.9334684	1.10E-06	1.04E-05	3.7053656	5.95674703	up-regulated in High
VAMP8	-0.3833041	7.55446452	-6.0453131	2.94E-09	4.43E-08	9.46272679	8.532206315	up-regulated in High
TMEM150A	-0.4930488	3.54456743	-7.7113516	6.88E-14	2.05E-12	19.9241928	13.16259454	up-regulated in High
USP39	0.25771215	4.13747923	7.2361033	1.77E-12	4.32E-11	16.72864	11.75249444	up-regulated in Low
SFTPB	-2.6007562	9.55566825	-10.850981	9.32E-25	1.02E-22	44.6922244	24.03036737	up-regulated in High
GNLY	0.27335742	2.07802154	2.34483887	0.01942903	0.06064704	-5.4521109	1.711548865	up-regulated in Low
ATOH8	-0.779825	2.0285157	-6.4727549	2.32E-10	4.17E-09	11.9448848	9.635048817	up-regulated in High
ST3GAL5	-1.2607927	3.66836407	-13.647714	3.15E-36	7.55E-34	70.9604837	35.50206924	up-regulated in High
POLR1A	0.2614235	2.47241316	5.90256087	6.64E-09	9.38E-08	8.66626938	8.177557169	up-regulated in Low
PTCD3	0.29312363	2.74778248	8.45033177	3.25E-16	1.34E-14	25.205553	15.48758604	up-regulated in Low
IMMT	0.3852113	4.49179408	12.0687006	1.40E-29	2.20E-27	55.7306496	28.85506414	up-regulated in Low
MRPL35	0.2975397	3.18856771	9.21247691	9.03E-19	5.10E-17	31.026519	18.04421751	up-regulated in Low
RNF103	-0.1008616	2.59702385	-2.5068484	0.01250034	0.0420352	-5.0632319	1.903078331	up-regulated in High
RMND5A	0.14387943	3.09891855	3.0339443	0.00254051	0.01075808	-3.6236363	2.595079965	up-regulated in Low
KRCC1	-0.2433272	4.43666258	-3.6567397	0.00028277	0.00156788	-1.5840288	3.548574149	up-regulated in High
FOXI3	0.23837211	0.60464719	2.57674451	0.01026176	0.03557707	-4.8876375	1.988778163	up-regulated in Low
RPIA	0.20398828	3.92426893	4.379334	1.45E-05	0.000109	1.230566	4.837640063	up-regulated in Low
TEKT4	-0.2105131	0.26957332	-5.244326	2.33E-07	2.50E-06	5.21049739	6.633104923	up-regulated in High
MAL	-0.7133457	1.93610586	-7.5808409	1.70E-13	4.78E-12	19.0305582	12.76855106	up-regulated in High
MRPS5	0.25514404	3.22103668	7.40872867	5.54E-13	1.44E-11	17.8705949	12.25674473	up-regulated in Low
ANKRD36C	-0.2010079	0.57298828	-3.5862155	0.00036878	0.00197742	-1.8331539	3.43323736	up-regulated in High
STARD7	0.1452063	5.29577072	4.018259	6.77E-05	0.0004399	-0.2351499	4.169156648	up-regulated in Low
CIAO1	0.14248033	4.43803471	5.26272494	2.12E-07	2.29E-06	5.30216874	6.674215541	up-regulated in Low
SNRNP200	0.26284031	4.78896208	5.26157234	2.13E-07	2.30E-06	5.29641756	6.671636644	up-regulated in Low
NCAPH	1.29732231	2.18829764	18.9158163	1.80E-60	2.33E-57	126.61579	59.74423649	up-regulated in Low
CNNM3	-0.2068091	3.16613046	-4.6381232	4.50E-06	3.74E-05	2.35274223	5.346349152	up-regulated in High
COX5B	0.33428878	5.90759278	5.66464547	2.50E-08	3.19E-07	7.37573998	7.60196088	up-regulated in Low
ACTR1B	-0.1532393	4.71873501	-3.7948484	0.00016601	0.00097918	-1.0828693	3.779863567	up-regulated in High
ZAP70	-0.2196758	1.43168147	-2.9102697	0.00377364	0.01518166	-3.9852212	2.423239645	up-regulated in High
VWA3B	-0.2766582	0.46922966	-4.8441077	1.70E-06	1.55E-05	3.28809117	5.768730529	up-regulated in High
INPP4A	-0.2048098	2.0593738	-5.4398661	8.39E-08	9.81E-07	6.1993971	7.076134125	up-regulated in High
KIAA1211L	-0.4572642	2.05918773	-6.6249178	9.07E-11	1.74E-09	12.863092	10.04221016	up-regulated in High
TSGA10	-0.1259426	0.77698203	-3.5833522	0.00037274	0.00199589	-1.8431712	3.428594244	up-regulated in High
MITD1	0.16553302	2.32137346	4.29128613	2.14E-05	0.00015472	0.86234736	4.670177072	up-regulated in Low
MRPL30	0.32551569	3.50523998	8.74436635	3.50E-17	1.61E-15	27.4078182	16.45547108	up-regulated in Low
TXNDC9	0.27785313	3.70973307	6.06907719	2.56E-09	3.90E-08	9.5969093	8.591915274	up-regulated in Low
EIF5B	0.34956365	4.35964839	6.27317963	7.72E-10	1.27E-08	10.7679522	9.112552482	up-regulated in Low
REV1	-0.2147411	2.46931876	-5.451428	7.89E-08	9.27E-07	6.2588766	7.102750831	up-regulated in High
AFF3	-0.3707965	0.56503891	-7.4539626	4.07E-13	1.08E-11	18.1733956	12.39038544	up-regulated in High
LONRF2	-0.1886475	0.93691724	-3.0486996	0.0024212	0.01031947	-3.579528	2.615969731	up-regulated in High
CHST10	-0.1526376	1.89727466	-2.3504408	0.0191423	0.05991215	-5.4390878	1.718005984	up-regulated in High
PDCL3	0.37036896	3.7658345	9.31758435	3.90E-19	2.29E-17	31.8574972	18.40879045	up-regulated in Low
CNOT11	0.14778623	4.87257092	3.34767375	0.00087692	0.00423953	-2.6415626	3.057039744	up-regulated in Low
CREG2	0.24737561	0.23260429	6.55141504	1.43E-10	2.66E-09	12.41731	9.844585936	up-regulated in Low
MAP4K4	0.36542434	3.51029003	5.71405793	1.91E-08	2.49E-07	7.63994779	7.71990374	up-regulated in Low
IL1R2	0.53462138	1.11899805	6.8195123	2.67E-11	5.54E-10	14.063295	10.5738513	up-regulated in Low
IL1R1	-0.6827912	4.28612694	-9.1948365	1.04E-18	5.83E-17	30.8877067	17.98330789	up-regulated in High
SLC9A2	0.14828618	0.42120418	2.49980718	0.0127483	0.04278525	-5.08066	1.894547728	up-regulated in Low
MFSD9	0.1405381	2.38055425	3.1922526	0.00150135	0.0068159	-3.1396634	2.823518372	up-regulated in Low
TMEM182	0.17431988	1.44766005	4.40710416	1.29E-05	9.74E-05	1.34813762	4.891050774	up-regulated in Low
MRPS9	0.25701471	3.74971976	7.18477831	2.49E-12	5.97E-11	16.3933079	11.60434414	up-regulated in Low
C2orf49	0.13450661	2.46985543	3.94978286	8.96E-05	0.00056438	-0.49982	4.047852166	up-regulated in Low
FHL2	0.22021719	3.12405082	2.09688176	0.03651029	0.10152045	-5.9980293	1.437584688	up-regulated in Low
C2orf40	-0.6958587	0.83202934	-8.5480274	1.56E-16	6.67E-15	25.9310806	15.8065443	up-regulated in High
UXS1	-0.2117622	3.48227681	-4.729032	2.95E-06	2.54E-05	2.76097183	5.530862535	up-regulated in High
SULT1C2	-0.7278593	1.37317766	-7.8093693	3.45E-14	1.07E-12	20.6032135	13.46186798	up-regulated in High
SULT1C4	-0.2540184	0.71870714	-5.3732286	1.19E-07	1.35E-06	5.85877282	6.923642719	up-regulated in High
GCC2	-0.2134656	2.2714939	-4.720137	3.07E-06	2.64E-05	2.72070833	5.512675906	up-regulated in High
CCDC138	0.31452889	0.99304331	8.97027857	6.10E-18	3.10E-16	29.1372549	17.21499791	up-regulated in Low
SH3RF3	-0.1616351	1.42104155	-3.0705301	0.0022541	0.00970216	-3.5138917	2.647027823	up-regulated in High
SOWAHC	0.3030296	2.5498455	5.09748242	4.91E-07	4.96E-06	4.48918271	6.309304937	up-regulated in Low
MALL	-1.0283392	2.85918869	-10.781704	1.72E-24	1.80E-22	44.085362	23.76489067	up-regulated in High
NPHP1	-0.1193186	0.74263501	-3.5939903	0.00035821	0.00192675	-1.8059153	3.445860498	up-regulated in High
LINC00116	0.14392043	2.90439138	2.2319963	0.02606143	0.07728421	-5.7079601	1.584001733	up-regulated in Low
BUB1	1.22186519	2.13154944	18.3284199	1.14E-57	1.08E-54	120.176108	56.94158254	up-regulated in Low
ACOXL	-0.5373413	0.78864728	-9.662314	2.38E-20	1.61E-18	34.6288609	19.6240475	up-regulated in High
BCL2L11	0.16650605	2.82418775	3.55241225	0.0004182	0.00221211	-1.9509303	3.378619708	up-regulated in Low
ANAPC1	0.24344802	1.34032222	7.30907116	1.09E-12	2.72E-11	17.2086947	11.96452029	up-regulated in Low
MERTK	-0.2975276	2.3658522	-4.8643774	1.54E-06	1.42E-05	3.38213402	5.811125663	up-regulated in High
ZC3H8	0.18583946	1.33157536	5.80949582	1.12E-08	1.53E-07	8.15593648	7.950087524	up-regulated in Low
ZC3H6	-0.2521793	1.20118756	-7.7914088	3.92E-14	1.20E-12	20.4782898	13.40681736	up-regulated in High

TTL	0.29952091	1.69672955	7.82086734	3.18E-14	9.85E-13	20.683306	13.49716065	up-regulated in Low
POLR1B	0.24904692	2.53976506	6.7242612	4.87E-11	9.71E-10	13.4721987	10.31209613	up-regulated in Low
CHCHD5	0.12449759	2.46534613	2.5613363	0.01072193	0.03690511	-4.9267504	1.969727037	up-regulated in Low
SLC20A1	0.39104851	3.82854768	6.15050405	1.59E-09	2.51E-08	10.0601156	8.797949455	up-regulated in Low
CKAP2L	1.00314949	1.46874092	17.4004941	2.82E-53	1.96E-50	110.086303	52.54943949	up-regulated in Low
IL1A	0.2120707	0.59489825	3.01379748	0.00271212	0.01137264	-3.6835286	2.566690728	up-regulated in Low
IL37	-1.2115172	1.66682946	-6.6691895	6.89E-11	1.34E-09	13.1336008	10.16208848	up-regulated in High
IL36G	-0.189654	0.41190581	-3.3637044	0.00082862	0.00402901	-2.5888985	3.081646668	up-regulated in High
PSD4	-0.2475087	2.74556954	-5.2931576	1.81E-07	1.99E-06	5.45442717	6.74247737	up-regulated in High
PAX8	0.15081768	0.73233438	2.69158692	0.00735176	0.02679277	-4.5889296	2.133608931	up-regulated in Low
RABL2A	-0.2434413	1.21222883	-5.891599	7.07E-09	9.92E-08	8.60579249	8.150610484	up-regulated in High
ACTR3	0.30522164	4.50213917	7.25309969	1.58E-12	3.88E-11	16.8401117	11.80173469	up-regulated in Low
DDX18	0.28041048	3.71470535	7.69073405	7.94E-14	2.34E-12	19.782221	13.10000681	up-regulated in Low
INSIG2	-0.2262494	3.40030628	-4.7945499	2.16E-06	1.91E-05	3.05966918	5.665703499	up-regulated in High
MARCO	-0.8416226	3.97823992	-5.2370959	2.42E-07	2.59E-06	5.17455206	6.616982573	up-regulated in High
C1QL2	-0.4110589	0.51296947	-4.1423172	4.04E-05	0.00027569	0.25519177	4.393377332	up-regulated in High
STEAP3	-0.5893752	4.06216744	-7.0678606	5.37E-12	1.23E-10	15.6366729	11.26992283	up-regulated in High
C2orf76	0.20392025	2.2676191	4.66893328	3.90E-06	3.28E-05	2.49028357	5.408545839	up-regulated in Low
TMEM37	-0.7889034	2.99480714	-8.5098182	2.08E-16	8.77E-15	25.6465835	15.68148418	up-regulated in High
SCTR	-1.5605425	2.09398704	-12.411238	5.47E-31	9.61E-29	58.9532421	30.26235085	up-regulated in High
CFAP221	-0.943254	1.20398915	-13.91622	2.14E-37	5.38E-35	73.6382148	36.66989453	up-regulated in High
TMEM177	0.24141419	2.45110571	6.01633086	3.47E-09	5.17E-08	9.2996947	8.45964452	up-regulated in Low
PTPN4	-0.1620764	1.4919586	-4.4068135	1.29E-05	9.75E-05	1.34690338	4.890490227	up-regulated in High
EPB41L5	-0.1280179	2.61348875	-2.0831394	0.03775055	0.10430843	-6.0265357	1.423076687	up-regulated in High
TMEM185B	0.25415628	3.73736908	5.69146538	2.16E-08	2.79E-07	7.51889591	7.665872777	up-regulated in Low
INHBB	-0.5548401	2.82519329	-4.5627193	6.37E-06	5.14E-05	2.01965251	5.195592108	up-regulated in High
TFCP2L1	-0.3287485	2.65800667	-2.7666682	0.00587487	0.02219307	-4.3868055	2.231001737	up-regulated in High
NIFK	0.41430865	4.12679346	10.4158587	4.17E-23	3.87E-21	40.9210728	22.38018642	up-regulated in Low
TSN	0.32105368	4.41639308	8.28467469	1.11E-15	4.25E-14	23.9896238	14.95281877	up-regulated in Low
GYPC	-0.473137	2.6507291	-7.1920255	2.37E-12	5.71E-11	16.4405403	11.62521374	up-regulated in High
ERCC3	0.14484463	3.13109775	5.12617829	4.25E-07	4.34E-06	4.62869382	6.371977823	up-regulated in Low
IWS1	0.15462231	4.07165524	4.61125152	5.10E-06	4.19E-05	2.23346444	5.29238571	up-regulated in Low
LIMS2	-0.5029937	1.47575326	-9.4582399	1.26E-19	7.88E-18	32.9798347	18.90105183	up-regulated in High
GPR17	-0.1353194	0.25686145	-4.6166856	4.97E-06	4.10E-05	2.25753378	5.30327706	up-regulated in High
POLR2D	0.22885752	3.3111673	6.13603543	1.73E-09	2.72E-08	9.97742165	8.761176653	up-regulated in Low
SAP130	0.19183865	3.17050573	4.98783023	8.46E-07	8.18E-06	3.96259216	6.072532105	up-regulated in Low
UGGT1	0.29614634	3.54197728	5.89406082	6.97E-09	9.79E-08	8.61936567	8.156658506	up-regulated in Low
SMPD4	0.3618077	3.33616761	8.74200769	3.57E-17	1.63E-15	27.3899315	16.44761324	up-regulated in Low
MZT2B	0.33983629	4.75821925	5.46631318	7.29E-08	8.62E-07	6.3356169	7.13708682	up-regulated in Low
CCDC115	-0.2012193	3.70450604	-5.785465	1.28E-08	1.73E-07	8.02530942	7.89183331	up-regulated in High
IMP4	0.28832361	3.96458819	8.16868091	2.61E-15	9.49E-14	23.1490738	14.58297543	up-regulated in Low
PTPN18	-0.2949033	3.86017911	-6.6700475	6.85E-11	1.33E-09	13.1388586	10.16441819	up-regulated in High
ARHGEF4	0.41373226	0.84882539	6.45646593	2.56E-10	4.58E-09	11.8476561	9.591911109	up-regulated in Low
FAM168B	0.122732	4.38804411	2.36239827	0.01854264	0.05834497	-5.4111884	1.731828349	up-regulated in Low
MZT2A	0.42554235	2.84654689	8.17854395	2.43E-15	8.87E-14	23.2201957	14.61427472	up-regulated in Low
GPR39	-0.3485171	2.32395764	-4.7028905	3.33E-06	2.83E-05	2.6428397	5.477496176	up-regulated in High
TMEM163	-1.1881222	2.28514674	-12.295392	1.64E-30	2.77E-28	57.8579728	29.78410792	up-regulated in High
MAP3K19	-0.2280904	0.3117138	-4.8135158	1.97E-06	1.77E-05	3.14683452	5.70502735	up-regulated in High
ZRANB3	0.14557332	0.60170976	7.45121853	4.15E-13	1.10E-11	18.1549843	12.3822604	up-regulated in Low
R3HDM1	0.41715239	2.35982062	11.5920603	1.17E-27	1.62E-25	51.3293108	26.93218214	up-regulated in Low
UBXN4	0.14801966	5.09397415	4.21670242	2.95E-05	0.00020696	0.55586979	4.530564421	up-regulated in Low
LCT	-0.1249794	0.21711287	-3.102389	0.00202919	0.00885024	-3.4172947	2.692678027	up-regulated in High
MCM6	0.89431332	3.7473989	14.3895169	1.77E-39	5.09E-37	78.4128764	38.75172679	up-regulated in Low
DARS	0.3445285	4.66348902	8.93630347	7.95E-18	3.97E-16	28.8751222	17.09990475	up-regulated in Low
CXCR4	-0.4180667	5.12255202	-4.6880135	3.57E-06	3.02E-05	2.57587879	5.447236672	up-regulated in High
THSD7B	-0.1169851	0.22233753	-5.0281104	6.93E-07	6.80E-06	4.15483162	6.159009104	up-regulated in High
HNMT	-0.6856909	3.44880735	-9.8339679	5.75E-21	4.25E-19	36.0344818	20.24008467	up-regulated in High
KYNU	0.79566036	1.18379187	8.17995923	2.41E-15	8.80E-14	23.2304066	14.61876825	up-regulated in Low
ARHGAP15	-0.3445166	1.1083201	-7.671867	9.06E-14	2.66E-12	19.6525648	13.0428438	up-regulated in High
ZEB2	-0.3401931	1.51458418	-6.6681337	6.93E-11	1.35E-09	13.1271322	10.15922223	up-regulated in High
ACVR2A	-0.1907752	1.58692986	-5.3698243	1.21E-07	1.37E-06	5.84147134	6.915894185	up-regulated in High
ORC4	0.10589358	2.23265557	3.09076285	0.00210875	0.00915078	-3.4526568	2.675974355	up-regulated in Low
EPC2	-0.1271837	2.75835705	-3.3930313	0.0007466	0.00368308	-2.4919316	3.126915	up-regulated in High
KIF5C	0.20514793	0.38148098	3.93608292	9.47E-05	0.00059371	-0.5522596	4.023793265	up-regulated in Low
LYPD6B	0.49170356	1.08384345	5.20822239	2.80E-07	2.97E-06	5.03144849	6.552783372	up-regulated in Low
LYPD6	0.30729625	0.47899858	6.32005997	5.83E-10	9.81E-09	11.0416007	9.234102695	up-regulated in Low
MMADHC	0.30668844	5.17089616	7.04158401	6.38E-12	1.45E-10	15.4680171	11.19535237	up-regulated in Low
RND3	0.36430743	3.14091564	3.75554532	0.0001935	0.00112229	-1.2272768	3.713313083	up-regulated in Low
RBM43	-0.4053683	1.94688562	-9.1076904	2.07E-18	1.12E-16	30.2047265	17.68358438	up-regulated in High
NMI	0.18033166	3.24155186	3.19881339	0.00146828	0.00668553	-3.1190959	2.833190828	up-regulated in Low
TNFAIP6	0.17844179	1.9556173	2.08009727	0.03802992	0.10492642	-6.0328213	1.419874593	up-regulated in Low

RIF1	0.24703322	2.07296469	6.30640268	6.33E-10	1.06E-08	10.9617016	9.19861702	up-regulated in Low
NEB	0.16093666	0.25047701	4.94689861	1.03E-06	9.83E-06	3.76867663	5.985253195	up-regulated in Low
CACNB4	-0.112618	0.27498539	-3.8550604	0.00013094	0.00079273	-0.8588888	3.882943027	up-regulated in High
FMNL2	-0.1698903	3.45264004	-2.6046314	0.00947355	0.03327912	-4.8162675	2.023487176	up-regulated in High
PRPF40A	0.31994223	3.6966118	8.92241886	8.85E-18	4.38E-16	28.7682031	17.05295746	up-regulated in Low
ARL6IP6	0.35844495	2.82456374	6.75576603	4.00E-11	8.09E-10	13.6669426	10.39835054	up-regulated in Low
GALNT13	0.2550135	0.46373111	4.4493271	1.06E-05	8.20E-05	1.52821441	4.972802643	up-regulated in Low
GPD2	0.26052418	3.40563028	5.65953883	2.57E-08	3.27E-07	7.34854969	7.589819946	up-regulated in Low
GALNT5	-0.7445787	2.23416101	-7.503507	2.90E-13	7.85E-12	18.5067371	12.53747466	up-regulated in High
CYTIP	-0.4226347	2.6667207	-5.2550898	2.20E-07	2.38E-06	5.26409227	6.657140977	up-regulated in High
ACVR1	-0.2551554	3.78962907	-5.6993144	2.07E-08	2.68E-07	7.56090365	7.68462404	up-regulated in High
CCDC148	-0.1010121	0.42631209	-3.5033062	0.00050114	0.00259166	-2.1201349	3.300045068	up-regulated in High
TANC1	-0.2330457	2.22184022	-5.3254919	1.53E-07	1.70E-06	5.6170586	6.815363386	up-regulated in High
BAZ2B	-0.1301571	1.57421198	-3.5051646	0.00049774	0.00257681	-2.1137725	3.303001979	up-regulated in High
CD302	-0.7703992	2.09024945	-11.689564	4.76E-28	6.85E-26	52.2215487	27.3220728	up-regulated in High
LY75	-0.1863923	1.42688832	-3.3533999	0.00085937	0.004164	-2.6227786	3.065818181	up-regulated in High
PLA2R1	-0.1779239	0.91303978	-4.6009576	5.35E-06	4.38E-05	2.18794039	5.271783419	up-regulated in High
ITGB6	-0.8868538	4.43350648	-7.8527573	2.54E-14	7.97E-13	20.905924	13.59524887	up-regulated in High
RBMS1	-0.166464	3.00028567	-3.921906	0.00010024	0.00062561	-0.6063449	3.998970585	up-regulated in High
PSMD14	0.53454204	3.43116605	12.5453638	1.52E-31	2.73E-29	60.2280536	30.81892229	up-regulated in Low
DPP4	-1.322776	4.04552592	-8.823378	1.91E-17	9.05E-16	28.0090322	16.71956201	up-regulated in High
FIGN	0.23631609	0.53479141	6.67223022	6.76E-11	1.32E-09	13.1522357	10.17034553	up-regulated in Low
COBLL1	-0.4021603	1.75616262	-7.7401437	5.62E-14	1.69E-12	20.1229541	13.25020909	up-regulated in High
SCN3A	0.19711367	0.30958106	2.67607659	0.00769562	0.02786853	-4.6300117	2.113756177	up-regulated in Low
SCN1A	-0.2302411	0.34889907	-6.3436138	5.06E-10	8.62E-09	11.1797424	9.295447756	up-regulated in High
SCN7A	-0.9734855	1.32807806	-14.293051	4.73E-39	1.33E-36	77.4342721	38.32508995	up-regulated in High
STK39	-0.2764981	3.87272487	-3.6288698	0.00031422	0.00171861	-1.6830283	3.502771149	up-regulated in High
CERS6	0.12624053	2.45370859	2.0936536	0.03679846	0.10216537	-6.0047422	1.434170307	up-regulated in Low
NOSTRIN	-0.3580954	1.63608865	-5.1612677	3.56E-07	3.69E-06	4.80024542	6.449013486	up-regulated in High
SPC25	1.08679932	1.72610155	19.5291235	2.07E-63	4.02E-60	133.373774	62.68500992	up-regulated in Low
DHRS9	-0.2902812	1.48715462	-2.7108232	0.00694452	0.02557524	-4.5376585	2.158357597	up-regulated in High
LRP2	-0.195513	0.95501046	-2.1676311	0.03066218	0.08824472	-5.8483578	1.513396914	up-regulated in High
BBS5	-0.1551281	1.13311778	-4.698971	3.39E-06	2.88E-05	2.62517924	5.469516143	up-regulated in High
FASTKD1	0.26126961	2.37616862	6.56694003	1.30E-10	2.44E-09	12.5111187	9.886180848	up-regulated in Low
CCDC173	-0.2479859	0.57994209	-4.7149162	3.15E-06	2.70E-05	2.69710865	5.502014982	up-regulated in High
KLHL23	0.19208522	2.4032887	2.94921775	0.00333634	0.01362993	-3.8729162	2.476729444	up-regulated in Low
SSB	0.35444636	4.33106687	9.57249939	4.96E-20	3.27E-18	33.9001278	19.30458203	up-regulated in Low
METTL5	0.36284615	3.28669588	9.19621461	1.03E-18	5.77E-17	30.8985442	17.98806337	up-regulated in Low
ERICH2	-0.5855756	2.15237558	-6.565183	1.31E-10	2.46E-09	12.5004925	9.881469376	up-regulated in High
GAD1	0.10561961	0.47050374	2.10788727	0.03554231	0.09931128	-5.9750673	1.44925441	up-regulated in Low
GORASP2	0.10757513	4.96393697	2.78152109	0.00561666	0.02136973	-4.3461816	2.250521984	up-regulated in Low
TLK1	0.21987319	3.00493338	5.94974798	5.08E-09	7.33E-08	8.92771663	8.294021477	up-regulated in Low
METTL8	0.22850548	1.29003026	7.84569047	2.67E-14	8.36E-13	20.8565307	13.5734866	up-regulated in Low
DCAF17	0.12260397	1.78949364	4.13943891	4.09E-05	0.00027889	0.24365754	4.388110273	up-regulated in Low
CYBRD1	-1.1214661	4.40357454	-12.487497	2.64E-31	4.68E-29	59.6771801	30.57842455	up-regulated in High
DYNC1I2	0.12116027	4.1702564	3.39582452	0.00073919	0.00365075	-2.4826541	3.131243563	up-regulated in Low
HAT1	0.33527084	2.84820053	9.08163216	2.54E-18	1.36E-16	30.0014031	17.59434423	up-regulated in Low
METAP1D	0.18687522	1.2442583	4.84418765	1.70E-06	1.55E-05	3.28846156	5.768897529	up-regulated in Low
ITGA6	0.28135569	3.37413302	2.65394066	0.00821143	0.02942259	-4.6882433	2.085580974	up-regulated in Low
PDK1	0.33390035	1.60015353	6.44352703	2.77E-10	4.93E-09	11.7705715	9.557707493	up-regulated in Low
pk	0.1844927	2.32376114	3.35711407	0.00084817	0.00411346	-2.6105783	3.071518774	up-regulated in Low
CDCA7	0.59480696	2.63171484	5.92578022	5.82E-09	8.31E-08	8.79469401	8.234770998	up-regulated in Low
OLA1	0.45618156	3.87714855	10.4276413	3.76E-23	3.51E-21	41.0219033	22.42432275	up-regulated in Low
SP9	0.11766685	0.09660463	3.87382337	0.00012152	0.00074188	-0.7884142	3.915342048	up-regulated in Low
GPR155	-0.1053333	1.18993926	-2.5919452	0.00982515	0.03433718	-4.8488277	2.007660853	up-regulated in High
WIPF1	-0.2005877	3.03881912	-2.924483	0.00360834	0.01459609	-3.9444049	2.442692872	up-regulated in High
ATP5G3	0.47508159	4.46716259	10.653226	5.31E-24	5.37E-22	42.9663015	23.27527626	up-regulated in Low
KIAA1715	0.13584107	2.59336269	3.53334419	0.00044875	0.00235365	-2.0168991	3.348000714	up-regulated in Low
HOXD13	0.39271924	0.21058678	6.4299169	3.01E-10	5.31E-09	11.6896292	9.521788949	up-regulated in Low
HOXD11	0.23003014	0.14361729	6.12807117	1.82E-09	2.84E-08	9.93197405	8.740965028	up-regulated in Low
HOXD10	0.26832087	0.29579708	5.78537759	1.28E-08	1.73E-07	8.02483509	7.891621754	up-regulated in Low
HOXD9	0.32371237	0.80163843	4.56088254	6.43E-06	5.18E-05	2.01160137	5.191945756	up-regulated in Low
HOXD8	0.23315985	0.71369532	3.36254693	0.00083202	0.0040432	-2.5927091	3.079866695	up-regulated in Low
HOXD1	-0.8289838	1.20831216	-8.333464	7.77E-16	3.02E-14	24.3458556	15.10951907	up-regulated in High
MTX2	0.17333584	4.58367135	4.31213095	1.95E-05	0.00014249	0.94889448	4.709564311	up-regulated in Low
HNRNPA3	0.18426462	5.49195099	4.49807172	8.55E-06	6.71E-05	1.73807571	5.067996622	up-regulated in Low
NFE2L2	-0.1677461	4.42507534	-3.8954289	0.00011148	0.00068709	-0.7068641	3.952812786	up-regulated in High
AGPS	0.21063753	3.05103232	5.146001	3.84E-07	3.96E-06	4.72547739	6.415442853	up-regulated in Low
OSBPL6	-0.1303076	0.61758505	-2.7777652	0.00568096	0.02157362	-4.3564742	2.245577936	up-regulated in High
PRKRA	0.15177235	3.63522341	4.42006657	1.21E-05	9.24E-05	1.40325229	4.916078759	up-regulated in Low
FKBP7	-0.1903637	2.33774337	-3.9121086	0.00010427	0.00064749	-0.643615	3.98186005	up-regulated in High

SESTD1	-0.128357	2.43191535	-2.228163	0.02631763	0.07788968	-5.7164344	1.579753179	up-regulated in High
ZNF385B	-0.7502913	1.33270453	-6.8100584	2.83E-11	5.86E-10	14.0043192	10.54774146	up-regulated in High
CWC22	0.15846551	3.36471748	4.92748528	1.14E-06	1.07E-05	3.67721102	5.94406855	up-regulated in Low
UBE2E3	0.34743426	4.05525129	6.57193918	1.26E-10	2.37E-09	12.5413654	9.899591398	up-regulated in Low
ITGA4	-0.3780696	1.63482146	-6.0620874	2.66E-09	4.05E-08	9.55739472	8.574333119	up-regulated in High
CERKL	-0.5257936	2.00136519	-8.9354179	8.00E-18	3.99E-16	28.8682994	17.09690896	up-regulated in High
NEUROD1	0.27036816	0.23652513	3.22444829	0.00134543	0.0061918	-3.0383443	2.871140512	up-regulated in Low
PDE1A	-0.2828702	0.94000084	-6.0348074	3.12E-09	4.68E-08	9.40355131	8.505870632	up-regulated in High
DNAJC10	0.33526634	3.00663168	6.56646029	1.30E-10	2.44E-09	12.5082171	9.884894324	up-regulated in Low
FRZB	-0.7910615	2.38984798	-9.5203516	7.59E-20	4.87E-18	33.4791614	19.12000901	up-regulated in High
NCKAP1	0.1894786	3.30933862	4.52493825	7.57E-06	6.02E-05	1.8546455	5.120837355	up-regulated in Low
NUP35	0.27174757	2.0539772	7.56171856	1.94E-13	5.41E-12	18.9006363	12.71124487	up-regulated in Low
ZC3H15	0.40802055	4.52334714	11.5221496	2.22E-27	2.99E-25	50.6922083	26.65375284	up-regulated in Low
FAM171B	0.19108663	1.18551498	2.83342583	0.00479297	0.01862792	-4.2025628	2.319395223	up-regulated in Low
CALCRL	-0.5163315	2.4083751	-7.151445	3.10E-12	7.35E-11	16.1765615	11.50856542	up-regulated in High
TFPI	-0.2938915	3.56854999	-2.4890032	0.01313727	0.0438964	-5.1073086	1.881494952	up-regulated in High
GULP1	0.20405698	1.77180694	2.56219452	0.01069582	0.03682176	-4.9245779	1.970785802	up-regulated in Low
WDR75	0.37851415	3.20043767	10.8902517	6.59E-25	7.28E-23	45.0372989	24.1813109	up-regulated in Low
SLC40A1	-0.9787188	5.1855029	-9.9927536	1.53E-21	1.20E-19	37.3495295	20.8162393	up-regulated in High
PMS1	0.18051642	1.92839374	5.28585283	1.88E-07	2.06E-06	5.41780913	6.726062648	up-regulated in Low
INPP1	-0.1161079	2.46641428	-2.160533	0.03121002	0.08952831	-5.8635941	1.505706029	up-regulated in High
MFSD6	-0.2269071	3.77404291	-3.9333958	9.57E-05	0.00059954	-0.562525	4.019082605	up-regulated in High
TMEM194B	0.15631331	1.28986869	5.00091941	7.93E-07	7.70E-06	4.02490777	6.100569276	up-regulated in Low
NAB1	0.23943013	3.2075072	4.76729499	2.46E-06	2.15E-05	2.93495799	5.609421572	up-regulated in Low
GLS	-0.3690366	3.95101176	-3.4316776	0.00065003	0.00325612	-2.3629241	3.187065685	up-regulated in High
STAT1	0.336576	5.7959699	3.91185413	0.00010437	0.00064802	-0.6445818	3.981416165	up-regulated in Low
STAT4	-0.2010768	1.45813452	-3.6512239	0.00028875	0.00159694	-1.6036791	3.539485914	up-regulated in High
MYO1B	-0.2065812	4.04618393	-3.7485976	0.00019879	0.00115006	-1.2526563	3.701609189	up-regulated in High
SDPR	-1.0101409	3.32831463	-9.8827697	3.83E-21	2.92E-19	36.4371527	20.41652336	up-regulated in High
DNAH7	-0.1136205	0.22175382	-4.0467254	6.02E-05	0.00039539	-0.123869	4.220099486	up-regulated in High
STK17B	-0.4656276	3.79863219	-7.9698534	1.10E-14	3.65E-13	21.7293798	13.95797196	up-regulated in High
HECW2	-0.2915876	1.39444551	-5.8250934	1.03E-08	1.40E-07	8.24097505	7.988004481	up-regulated in High
CCDC150	0.2099122	0.3303388	8.86550308	1.38E-17	6.64E-16	28.3311788	16.86104578	up-regulated in Low
GTF3C3	0.25238866	2.63514981	7.46960236	3.66E-13	9.78E-12	18.2784317	12.43673677	up-regulated in Low
ANKRD44	-0.5486123	1.49368106	-10.936998	4.35E-25	4.90E-23	45.4490533	24.36141052	up-regulated in High
HSPD1	0.83675731	6.25025488	17.1480649	4.33E-52	2.75E-49	107.362464	51.36352469	up-regulated in Low
HSPE1	0.68104116	5.44506931	12.9364741	3.48E-33	6.81E-31	63.9852579	32.45888709	up-regulated in Low
MOB4	0.1773755	3.04790699	4.46547071	9.90E-06	7.68E-05	1.59748424	5.004233051	up-regulated in Low
RFTN2	-0.1590272	0.67098743	-5.5978927	3.60E-08	4.48E-07	7.02201682	7.443970379	up-regulated in High
MARS2	0.31423485	2.10491572	7.79874388	3.72E-14	1.14E-12	20.5292815	13.42928856	up-regulated in Low
PLCL1	-0.2010397	0.59811286	-7.0835816	4.85E-12	1.12E-10	15.7378225	11.31464086	up-regulated in High
C2orf69	0.24233313	2.40648347	6.49817331	1.98E-10	3.60E-09	12.0970194	9.70253751	up-regulated in Low
C2orf47	0.3326288	3.38600669	9.27041093	5.69E-19	3.28E-17	31.4837234	18.24481675	up-regulated in Low
KCTD18	-0.1252261	2.32612838	-4.1833202	3.40E-05	0.0002357	0.42031632	4.468744278	up-regulated in High
SGOL2	0.73011586	1.3868169	15.6724505	2.97E-45	1.20E-42	91.6657486	44.52727121	up-regulated in Low
AC007163.2	0.11913477	0.10483097	3.24632617	0.00124817	0.00578941	-2.9689388	2.903725461	up-regulated in Low
BZW1	0.43346155	4.3069306	8.53363659	1.74E-16	7.37E-15	25.8238186	15.7593954	up-regulated in Low
CLK1	-0.2621343	3.76642737	-4.334638	1.77E-05	0.00013038	1.04277993	4.752272704	up-regulated in High
NIF3L1	0.39278164	3.78803909	10.9669149	3.33E-25	3.80E-23	45.7131384	24.47691392	up-regulated in Low
ORC2	0.22198132	2.56969953	6.8278095	2.53E-11	5.27E-10	14.115111	10.59679018	up-regulated in Low
FAM126B	-0.1133035	1.60158091	-3.0909983	0.00210711	0.00914571	-3.4519419	2.676312126	up-regulated in High
NDUFB3	0.29322982	4.92640223	6.71723028	5.09E-11	1.01E-09	13.4288412	10.29289037	up-regulated in Low
CFLAR	-0.3634338	3.11119876	-6.8360236	2.40E-11	5.02E-10	14.1664597	10.61952111	up-regulated in High
CASP10	-0.4343468	2.53758952	-8.1866219	2.29E-15	8.40E-14	23.2784939	14.63992977	up-regulated in High
ALS2CR12	-0.1867572	0.3182733	-5.2992542	1.75E-07	1.93E-06	5.48502377	6.756191811	up-regulated in High
TRAK2	-0.2993138	3.16694698	-6.7372927	4.49E-11	9.01E-10	13.5526595	10.34773509	up-regulated in High
TMEM237	0.27213249	1.92545708	6.34693466	4.96E-10	8.46E-09	11.1992543	9.304111664	up-regulated in Low
FZD7	-0.2635029	2.3542045	-3.8520695	0.0001325	0.00080118	-0.8700929	3.877790683	up-regulated in High
KIAA2012	-0.1168725	0.14658487	-3.8619593	0.0001274	0.00077378	-0.8330136	3.894840398	up-regulated in High
SUMO1	0.21220272	5.66769161	6.03703935	3.08E-09	4.63E-08	9.41611583	8.511462581	up-regulated in Low
NOP58	0.43092066	4.5394805	11.1343412	7.47E-26	8.92E-24	47.1991033	25.12674309	up-regulated in Low
BMPR2	-0.2640734	3.23003616	-5.9057848	6.52E-09	9.23E-08	8.68407466	8.185490186	up-regulated in High
WDR12	0.41065419	2.06942722	11.2801038	2.01E-26	2.48E-24	48.5037612	25.69716345	up-regulated in Low
CARF	-0.2296153	0.95031793	-7.9298474	1.47E-14	4.77E-13	21.4469831	13.83359707	up-regulated in High
NBEAL1	-0.177516	0.99251887	-4.356294	1.61E-05	0.00011966	1.13354275	4.793542655	up-regulated in High
CYP20A1	-0.1875261	1.99357335	-6.6420791	8.15E-11	1.57E-09	12.9677721	10.08860387	up-regulated in High
CD28	-0.3395202	1.02478935	-6.9963888	8.56E-12	1.91E-10	15.1791355	11.06760091	up-regulated in High
ICOS	-0.1670447	0.98446857	-3.0851569	0.00214814	0.00930094	-3.4696622	2.667938429	up-regulated in High
PARD3B	-0.4539452	1.29383718	-10.53838	1.44E-23	1.39E-21	41.9731073	22.84064945	up-regulated in High
NDUFS1	0.28506901	3.1010424	7.64753508	1.07E-13	3.11E-12	19.4857243	12.96928047	up-regulated in Low
ZDBF2	0.17920332	0.84686231	4.40182691	1.32E-05	9.94E-05	1.32574219	4.880879098	up-regulated in Low

ADAM23	0.11610758	0.41015541	2.68939904	0.00739941	0.02691986	-4.5947386	2.130802982	up-regulated in Low
MDH1B	-0.1350331	0.54741701	-2.5792886	0.0101875	0.0353574	-4.8811575	1.991932428	up-regulated in High
FASTKD2	0.2572954	2.77549549	7.74629586	5.38E-14	1.62E-12	20.1654997	13.26896203	up-regulated in Low
KLF7	-0.1443969	2.37382364	-2.7671817	0.00586577	0.02216872	-4.3854047	2.231675161	up-regulated in High
METTL21A	0.26664988	1.79956513	7.909454	1.70E-14	5.46E-13	21.3034521	13.77037525	up-regulated in Low
IDH1	0.29859565	5.1621788	4.26797805	2.36E-05	0.00016951	0.76603397	4.626325649	up-regulated in Low
PTH2R	0.16218838	0.18582523	4.0545723	5.83E-05	0.00038432	-0.0930646	4.234195248	up-regulated in Low
MAP2	0.22054963	1.7428338	2.26895323	0.02370031	0.07147676	-5.6255278	1.625245935	up-regulated in Low
RPE	0.36692547	3.62399554	9.17170709	1.25E-18	6.94E-17	30.7059869	17.90356696	up-regulated in Low
KANSL1L	-0.1828241	1.67914819	-4.5786439	5.93E-06	4.81E-05	2.08958036	5.227257539	up-regulated in High
ACADL	-0.3111167	0.67510733	-5.2332039	2.46E-07	2.64E-06	5.15522124	6.608311658	up-regulated in High
LANCL1	0.1674465	3.34199916	3.18003575	0.00156477	0.00706917	-3.1778542	2.80555077	up-regulated in Low
CPS1	1.71104523	1.56328729	7.77224415	4.49E-14	1.36E-12	20.3452382	13.34818077	up-regulated in Low
ERBB4	-0.2006591	0.29032601	-6.0831377	2.36E-09	3.62E-08	9.67651467	8.627333033	up-regulated in High
IKZF2	-0.1820787	1.34141214	-4.2253913	2.84E-05	0.0002	0.5913167	4.546723005	up-regulated in High
BARD1	0.39869228	1.68898693	8.78567014	2.55E-17	1.19E-15	27.7216135	16.59331678	up-regulated in Low
ATIC	0.42212559	4.72550841	10.1327335	4.69E-22	3.89E-20	38.5204114	21.32908759	up-regulated in Low
XRCC5	0.31944795	5.85822407	8.97243213	5.99E-18	3.05E-16	29.1538947	17.22230353	up-regulated in Low
4-Mar	0.17806454	0.22948471	3.81887844	0.00015106	0.00090075	-0.9938792	3.820838585	up-regulated in Low
SMARCAL1	0.16117181	2.67446674	5.92874916	5.73E-09	8.18E-08	8.81114653	8.242099871	up-regulated in Low
RPL37A	0.12825755	6.6726619	2.35218066	0.019054	0.059687	-5.435037	1.720013789	up-regulated in Low
TNS1	-0.9502788	3.85749486	-12.252706	2.46E-30	4.09E-28	57.4557753	29.60847688	up-regulated in High
CXCR2	-0.1711862	0.54168364	-4.1530193	3.86E-05	0.00026484	0.29814431	4.412988471	up-regulated in High
GPBAR1	-0.1097367	0.44706103	-3.9084808	0.0001058	0.00065574	-0.6573929	3.975533593	up-regulated in High
AAMP	0.16077948	5.28750553	4.79161744	2.19E-06	1.94E-05	3.04621994	5.659634999	up-regulated in Low
PNKD	-0.1634536	4.70892589	-2.447425	0.01473417	0.04822219	-5.2088132	1.831674425	up-regulated in High
TMBIM1	-0.3234554	5.54125861	-5.4379452	8.48E-08	9.91E-07	6.18952628	7.071716675	up-regulated in High
CATIP	-0.1511282	0.42583955	-3.5545837	0.00041484	0.00219696	-1.9433964	3.38211528	up-regulated in High
SLC11A1	-0.2491405	2.05681449	-3.1630325	0.00165715	0.00741824	-3.2307735	2.780638029	up-regulated in High
CTDSP1	-0.1782876	4.97120874	-4.8690118	1.51E-06	1.39E-05	3.40368586	5.820839614	up-regulated in High
VIL1	0.42773079	1.20911867	2.87036283	0.00427533	0.01689842	-4.098794	2.36903076	up-regulated in Low
USP37	0.17152435	1.01674241	4.57406909	6.05E-06	4.90E-05	2.06946856	5.218151196	up-regulated in Low
RQCD1	0.32028007	3.6045798	10.4797407	2.40E-23	2.29E-21	41.4686205	22.61985301	up-regulated in Low
ZNF142	0.1115352	2.23050624	2.98347427	0.00299053	0.01238354	-3.7729478	2.524252391	up-regulated in Low
BCS1L	0.19966329	2.83437868	5.4408737	8.35E-08	9.77E-07	6.20457626	7.078451894	up-regulated in Low
RNF25	0.10239709	3.32947766	3.095504	0.00207597	0.00902597	-3.4382516	2.682779904	up-regulated in Low
TTLL4	0.24804642	2.23741491	5.90910846	6.40E-09	9.07E-08	8.70243934	8.193672208	up-regulated in Low
CYP27A1	-0.8686395	4.2355693	-9.3171191	3.92E-19	2.30E-17	31.8538045	18.40717057	up-regulated in High
WNT10A	-0.3906286	1.17664054	-4.3998582	1.33E-05	0.00010021	1.31739399	4.877087207	up-regulated in High
CDK5R2	0.26835414	0.35020153	3.96825107	8.31E-05	0.00052802	-0.428858	4.080395837	up-regulated in Low
CRYBA2	0.18149695	0.17322201	3.99083393	7.58E-05	0.00048663	-0.3416639	4.120363315	up-regulated in Low
CCDC108	-0.2406338	0.33217185	-4.9114516	1.23E-06	1.15E-05	3.60191451	5.910156046	up-regulated in High
CNPPD1	-0.182877	4.95419795	-5.0007229	7.94E-07	7.71E-06	4.02397104	6.100147857	up-regulated in High
ABCB6	0.36828444	1.42007095	5.98333069	4.19E-09	6.15E-08	9.11488524	8.377369514	up-regulated in Low
ATG9A	0.13078015	3.45421972	3.52284663	0.00046644	0.00243597	-2.0530729	3.33120261	up-regulated in Low
GLB1L	-0.1257232	1.99790858	-2.4770683	0.01357916	0.04507883	-5.136616	1.867127106	up-regulated in High
TUBA4A	0.29100442	4.45105686	3.55272043	0.00041772	0.00220978	-1.9498613	3.379115703	up-regulated in Low
DNAJB2	-0.1607601	4.14669265	-3.4319624	0.00064937	0.00325335	-2.3619683	3.187511028	up-regulated in High
PTPRN	0.33918744	0.33416978	5.2751209	1.99E-07	2.16E-06	5.36409268	6.70198073	up-regulated in Low
DNPEP	0.14919453	3.8700613	4.30947476	1.97E-05	0.00014401	0.93784442	4.70453639	up-regulated in Low
DES	-0.9945816	1.92738107	-8.9979792	4.91E-18	2.52E-16	29.3515073	17.30906107	up-regulated in High
GMPPA	0.15399733	3.52520509	3.90632439	0.00010672	0.00066074	-0.6655773	3.97177523	up-regulated in Low
TMEM198	0.15472007	1.22556667	2.58968132	0.0098891	0.03451416	-4.8546217	2.004843095	up-regulated in Low
INHA	0.85046852	1.1625657	5.54357611	4.82E-08	5.89E-07	6.73692021	7.316555975	up-regulated in Low
EPHA4	-0.7992537	1.97258428	-10.26363	1.54E-22	1.34E-20	39.6249717	21.81276901	up-regulated in High
SGPP2	-0.7775323	4.35432255	-7.1925391	2.36E-12	5.69E-11	16.4438888	11.62669326	up-regulated in High
FARSB	0.48886086	3.87534031	12.6409241	6.06E-32	1.12E-29	61.1406387	31.21730575	up-regulated in Low
ACSL3	0.32581203	3.95793398	5.99586591	3.90E-09	5.75E-08	9.18498219	8.408578456	up-regulated in Low
SCG2	0.31518959	1.04050976	2.66753471	0.00789111	0.02844766	-4.652538	2.102861809	up-regulated in Low
AP1S3	0.22747491	1.87224288	3.90155362	0.00010878	0.00067179	-0.6836688	3.963466738	up-regulated in Low
MRPL44	0.3023258	4.17231959	8.68118522	5.68E-17	2.55E-15	26.9299136	16.245504	up-regulated in Low
SERPINE2	0.1944543	1.8965913	2.15112058	0.03194944	0.09117903	-5.8837222	1.495536804	up-regulated in Low
FAM124B	-0.1445156	0.51989943	-5.0517829	6.16E-07	6.11E-06	4.26846122	6.210102557	up-regulated in High
DOCK10	-0.3556057	1.63574445	-5.9083236	6.43E-09	9.11E-08	8.69810159	8.191739631	up-regulated in High
COL4A4	-0.7151786	1.82215541	-8.8740849	1.29E-17	6.24E-16	28.3969437	16.88992715	up-regulated in High
COL4A3	-0.8037389	1.54136354	-9.0310011	3.79E-18	1.98E-16	29.6075376	17.42145725	up-regulated in High
MFF	0.33827311	3.40693913	7.88300676	2.05E-14	6.52E-13	21.117741	13.68856703	up-regulated in Low
TM4SF20	0.33937043	0.15613269	4.73394565	2.88E-06	2.49E-05	2.78324297	5.540921104	up-regulated in Low
AGFG1	0.37630802	3.36714297	8.74523577	3.48E-17	1.60E-15	27.4144123	16.45836791	up-regulated in Low
SLC19A3	-0.1840254	0.65796711	-3.3256278	0.00094764	0.00453819	-2.7135954	3.023358379	up-regulated in High
PID1	-0.7284896	2.20724135	-8.4946835	2.33E-16	9.74E-15	25.5341566	15.63205926	up-regulated in High

DNER	0.41622698	0.95189312	4.176163	3.50E-05	0.00024223	0.3913841	4.45554377	up-regulated in Low
TRIP12	0.14533921	3.9713516	3.35342638	0.00085929	0.004164	-2.6226917	3.065858791	up-regulated in Low
SLC16A14	0.82629748	2.18179791	5.94653206	5.18E-09	7.45E-08	8.90984107	8.286060097	up-regulated in Low
SP140	-0.1483459	1.18640398	-2.4643047	0.01406626	0.04640038	-5.1678061	1.851821387	up-regulated in High
SP140L	0.11009913	2.42558621	2.0713024	0.03884751	0.10672325	-6.0509422	1.410636863	up-regulated in Low
SP100	-0.1256898	2.85224458	-2.9459596	0.00337107	0.01375543	-3.8823662	2.472232711	up-regulated in High
C2orf72	0.29102299	0.89191463	3.17612728	0.00158557	0.00714519	-3.1900427	2.79981445	up-regulated in Low
PSMD1	0.4291041	4.51371337	10.4852351	2.29E-23	2.19E-21	41.515814	22.64050885	up-regulated in Low
ARMC9	-0.3522025	1.80338495	-5.4604686	7.52E-08	8.87E-07	6.30546329	7.123595806	up-regulated in High
B3GNT7	-1.0116102	4.07460212	-7.8426663	2.73E-14	8.53E-13	20.8354044	13.56417836	up-regulated in High
NCL	0.42157546	6.34424512	9.53440706	6.77E-20	4.37E-18	33.5924689	19.1696908	up-regulated in Low
PTMA	0.31900463	7.85005282	8.40000474	4.74E-16	1.91E-14	24.8342379	15.32431067	up-regulated in Low
PDE6D	0.14384866	2.85789306	3.89300123	0.00011257	0.00069278	-0.7160487	3.948593714	up-regulated in Low
COPS7B	0.23561676	2.93629868	6.7006058	5.66E-11	1.11E-09	13.3264736	10.24754218	up-regulated in Low
ECEL1	0.29503074	0.76657462	2.91213939	0.00375151	0.01510199	-3.979863	2.425794237	up-regulated in Low
PRSS56	0.100675	0.0595608	3.01550757	0.00269715	0.01131719	-3.6784598	2.569094446	up-regulated in Low
TIGD1	0.14911205	1.61965921	2.98079133	0.00301638	0.01247643	-3.7808174	2.520514351	up-regulated in Low
C2orf82	0.20954467	0.53838823	5.81059635	1.11E-08	1.52E-07	8.16193007	7.952760115	up-regulated in Low
NGEF	0.37445502	1.44810623	4.17777645	3.48E-05	0.00024072	0.39790223	4.458517889	up-regulated in Low
INPP5D	-0.4650218	2.35526783	-6.5073234	1.88E-10	3.42E-09	12.1519075	9.726883796	up-regulated in High
ATG16L1	0.15844425	2.93599612	4.48646785	9.01E-06	7.04E-05	1.68792612	5.045256174	up-regulated in Low
DGKD	-0.1982524	2.82150431	-2.964579	0.00317699	0.01305566	-3.8282263	2.497984496	up-regulated in High
UGT1A6	0.40619038	0.66054392	3.49475321	0.00051707	0.00266367	-2.1493769	3.286452487	up-regulated in Low
MROH2A	-0.1444654	0.21834928	-3.8255677	0.00014714	0.0008802	-0.9690129	3.832283329	up-regulated in High
HJURP	1.42517245	2.10901326	19.9300424	2.43E-65	6.18E-62	137.806779	64.61385279	up-regulated in Low
AGAP1	0.11712513	2.03260696	2.2794948	0.02306191	0.0698799	-5.6017718	1.637104722	up-regulated in Low
GBX2	0.15416152	0.11319863	6.0908201	2.26E-09	3.47E-08	9.72007679	8.646712899	up-regulated in Low
IQCA1	-0.3026235	0.91429584	-4.4263436	1.18E-05	9.01E-05	1.42999535	4.928220794	up-regulated in High
ACKR3	0.33215438	3.15440356	3.03691105	0.00251609	0.01067018	-3.6147844	2.599273479	up-regulated in Low
COPS8	0.21936125	3.52749574	6.374271	4.21E-10	7.26E-09	11.3601989	9.375568571	up-regulated in Low
COL6A3	-0.3302684	4.91411271	-3.0310437	0.00256458	0.01084589	-3.6322829	2.590983204	up-regulated in High
MLPH	-0.710355	4.42419232	-6.5077227	1.87E-10	3.41E-09	12.1543045	9.72794699	up-regulated in High
RAB17	-0.3154811	2.92588638	-4.5182878	7.80E-06	6.18E-05	1.82573066	5.107732687	up-regulated in High
LRRFIP1	-0.1010387	3.71650057	-2.2453489	0.02518581	0.07521478	-5.6783302	1.598844006	up-regulated in High
UBE2F	0.24011657	3.12161276	7.01519673	7.57E-12	1.70E-10	15.2991683	11.12068641	up-regulated in Low
ILKAP	0.13467144	2.66085607	4.16202133	3.72E-05	0.00025576	0.33435367	4.429517243	up-regulated in Low
HES6	0.52973346	2.89196876	4.137807	4.12E-05	0.00028058	0.23712123	4.385125346	up-regulated in Low
ASB1	0.16775387	2.14016298	4.17384713	3.54E-05	0.00024433	0.3820323	4.451276513	up-regulated in Low
HDAC4	0.1341295	1.26267328	3.27156389	0.00114409	0.00535771	-2.888316	2.941540005	up-regulated in Low
NDUFA10	0.21338361	3.24595062	5.84193062	9.35E-09	1.29E-07	8.33299472	8.029028283	up-regulated in Low
MYEOV2	0.21187263	4.02714028	4.04234468	6.13E-05	0.00040143	-0.141042	4.212240176	up-regulated in Low
OTOS	0.1250521	0.10892701	2.87864045	0.00416653	0.01651312	-4.075361	2.380225224	up-regulated in Low
ANKMY1	-0.145651	1.21295238	-3.6744363	0.00026436	0.00147902	-1.5207946	3.577809601	up-regulated in High
GPR35	0.4229794	1.20867265	4.71288858	3.18E-06	2.72E-05	2.68794983	5.497877338	up-regulated in Low
KIF1A	0.80998971	0.89235068	5.96127444	4.76E-09	6.91E-08	8.99185499	8.322585532	up-regulated in Low
C2orf54	-0.5954371	2.42194215	-4.9619683	9.61E-07	9.19E-06	3.83990174	6.017316395	up-regulated in High
SNED1	-0.6745332	1.74399992	-12.154227	6.24E-30	1.01E-27	56.5307374	29.20450368	up-regulated in High
PASK	0.12639211	1.43656755	2.82870674	0.00486305	0.01885063	-4.2157267	2.31309109	up-regulated in Low
HDLBP	0.16598329	5.90963895	3.56751694	0.00039539	0.00210541	-1.8984339	3.402971836	up-regulated in Low
2-Sep	0.12702878	5.91307573	3.60989557	0.00033748	0.0018304	-1.7500178	3.471755156	up-regulated in Low
FARP2	0.12509517	1.82369311	3.18042121	0.00156273	0.0070616	-3.1766514	2.806116802	up-regulated in Low
STK25	0.19018697	3.71248424	4.81911512	1.92E-06	1.73E-05	3.17262809	5.71666175	up-regulated in Low
BOK	-0.6188336	4.11638189	-8.2171937	1.83E-15	6.80E-14	23.499527	14.73719271	up-regulated in High
THAP4	0.23797516	4.08430119	6.36201162	4.53E-10	7.78E-09	11.2879484	9.343492118	up-regulated in Low
ATG4B	0.10025637	3.15028624	2.17907227	0.0297966	0.08618517	-5.8236961	1.525833352	up-regulated in Low
DTYMK	0.81362034	3.76066665	15.9844816	1.10E-46	4.88E-44	94.9490351	45.9575516	up-regulated in Low
GAL3ST2	0.20143542	0.30527315	4.26994775	2.34E-05	0.00016822	0.7741543	4.630023637	up-regulated in Low
CNTN6	-0.2381395	0.29935461	-6.2152724	1.09E-09	1.75E-08	10.432338	8.963421893	up-regulated in High
CNTN4	-0.1191373	0.43643878	-2.8352096	0.00476672	0.0185407	-4.1975814	2.321780342	up-regulated in High
IL5RA	-0.2587645	0.33828236	-6.3071222	6.30E-10	1.06E-08	10.9659076	9.200485093	up-regulated in High
AC024060.1	-0.1633018	1.0569384	-2.6318072	0.00875801	0.03111938	-4.7459984	2.057594824	up-regulated in High
CRBN	-0.2004901	2.29876079	-5.4480096	8.04E-08	9.43E-07	6.24127914	7.094876418	up-regulated in High
SUMF1	-0.1528819	2.97851576	-3.4625677	0.00058137	0.00295039	-2.2588064	3.235550542	up-regulated in High
ITPR1	-0.2654959	1.97863598	-3.9017172	0.00010871	0.00067149	-0.6830487	3.963751519	up-regulated in High
BHLHE40	-0.274042	6.1581232	-3.3061402	0.00101452	0.00482422	-2.7768902	2.99373885	up-regulated in High
ARL8B	-0.1154763	4.86578931	-3.044831	0.00245197	0.01043464	-3.5911124	2.610484767	up-regulated in High
EDEM1	-0.3919962	4.18568917	-6.3055037	6.36E-10	1.06E-08	10.9564478	9.19628351	up-regulated in High
LMCD1	-0.3211171	2.04181613	-6.1172845	1.93E-09	3.01E-08	9.87050166	8.713624839	up-regulated in High
RAD18	0.25768738	1.67556778	7.9006194	1.81E-14	5.79E-13	21.2413623	13.74302478	up-regulated in Low
SRGAP3	-0.1416629	0.45019351	-5.2381696	2.40E-07	2.58E-06	5.17988732	6.619375651	up-regulated in High
THUMPD3	0.12596882	2.92686656	3.88992073	0.00011396	0.00069982	-0.7276953	3.943243268	up-regulated in Low

CAMK1	-0.2806698	1.58986274	-6.6925113	5.95E-11	1.17E-09	13.276707	10.22549427	up-regulated in High
TTLL3	-0.3034647	1.3359565	-5.2073358	2.81E-07	2.98E-06	5.02706562	6.550816777	up-regulated in High
RPUSD3	0.11206367	2.41712328	3.14727748	0.00174722	0.00776666	-3.279565	2.757651973	up-regulated in Low
CIDEC	0.12701708	0.22492706	2.23431447	0.02590755	0.07692555	-5.7028285	1.586573708	up-regulated in Low
JAGN1	0.17858647	4.38869853	4.1360093	4.15E-05	0.00028258	0.22992368	4.381838324	up-regulated in Low
IL17RE	-0.5256448	2.42718347	-6.5559034	1.39E-10	2.60E-09	12.4444114	9.856603172	up-regulated in High
IL17RC	-0.2017882	3.0187151	-4.279368	2.25E-05	0.00016217	0.81303849	4.647729379	up-regulated in High
CRELD1	-0.2808285	3.47769002	-5.3673028	1.23E-07	1.39E-06	5.82866322	6.910157832	up-regulated in High
PRRT3	-0.3262345	1.30664214	-5.7909468	1.24E-08	1.68E-07	8.05506601	7.905104611	up-regulated in High
EMC3	-0.1526008	3.9531274	-3.7500497	0.00019767	0.00114442	-1.2473557	3.704053768	up-regulated in High
FANCD2	0.5869161	1.50757753	14.8384042	1.77E-41	5.65E-39	83.0013045	40.75179475	up-regulated in Low
GHRL	-0.2227943	0.49134352	-7.6331368	1.19E-13	3.41E-12	19.3871944	12.92583321	up-regulated in High
SEC13	0.17274108	4.49612537	4.60892512	5.16E-06	4.23E-05	2.22316796	5.287726263	up-regulated in Low
SLC6A1	-0.1957464	0.52917163	-5.3857652	1.12E-07	1.27E-06	5.92256974	6.952211901	up-regulated in High
HRH1	-0.1337476	1.76906135	-2.5792123	0.01018972	0.035363	-4.881352	1.991837758	up-regulated in High
TSEN2	0.13753968	1.27772596	4.43234492	1.15E-05	8.79E-05	1.45559636	4.939842981	up-regulated in Low
TMEM40	0.13090797	0.5652098	2.43657996	0.01517794	0.0494473	-5.2350146	1.818787132	up-regulated in Low
IQSEC1	-0.3347637	3.19765306	-6.6267436	8.97E-11	1.72E-09	12.8742178	10.04714128	up-regulated in High
NUP210	0.50182823	3.56867688	6.46851864	2.38E-10	4.27E-09	11.9195785	9.623821569	up-regulated in Low
HDAC11	-0.2925901	2.44409195	-5.8567314	8.61E-09	1.19E-07	8.41407594	8.065170542	up-regulated in High
FBLN2	-0.3749746	3.68012058	-3.6678712	0.00027105	0.00151082	-1.5442869	3.566950126	up-regulated in High
CHCHD4	0.14434901	2.99000916	3.91426751	0.00010337	0.00064259	-0.6354097	3.985627424	up-regulated in Low
TMEM43	-0.4611988	5.13942122	-9.2336849	7.63E-19	4.33E-17	31.1936539	18.11755142	up-regulated in High
.P11-434D12.	-0.1814444	0.44098709	-6.5423603	1.51E-10	2.80E-09	12.3626838	9.820362722	up-regulated in High
XPC	-0.3487518	2.97592581	-7.709065	6.99E-14	2.08E-12	19.9084331	13.1556472	up-regulated in High
LSM3	0.23026855	3.29143828	6.59504198	1.09E-10	2.07E-09	12.6813969	9.96167197	up-regulated in Low
CCDC174	-0.104996	2.59869648	-3.8376921	0.00014026	0.00084321	-0.9238375	3.853069933	up-regulated in High
FGD5	-0.4419429	1.75568596	-8.3335439	7.77E-16	3.02E-14	24.3464402	15.10977624	up-regulated in High
NR2C2	-0.1706913	2.34706673	-3.4941531	0.0005182	0.00266858	-2.151426	3.285499864	up-regulated in High
MRPS25	-0.2943988	3.42059436	-4.4889964	8.91E-06	6.96E-05	1.69884399	5.050207313	up-regulated in High
RBSN	-0.3010001	2.50559545	-7.470978	3.62E-13	9.70E-12	18.287679	12.44081738	up-regulated in High
SH3BP5	-0.3378667	1.89129947	-5.4206696	9.29E-08	1.08E-06	6.10089002	7.032045572	up-regulated in High
METTL6	0.11740711	1.45231055	4.64980237	4.27E-06	3.57E-05	2.40478188	5.369885346	up-regulated in Low
COLQ	-0.1223837	0.50550271	-4.4108244	1.26E-05	9.59E-05	1.36394029	4.898227509	up-regulated in High
BTD	-0.3770424	2.52057078	-9.0826592	2.52E-18	1.35E-16	30.0094085	17.59785799	up-regulated in High
GALNT15	-0.2450899	0.743559	-4.7113215	3.20E-06	2.74E-05	2.68087356	5.494680427	up-regulated in High
RFTN1	-0.7804008	3.77067507	-9.8609601	4.60E-21	3.44E-19	36.257033	20.33760221	up-regulated in High
PLCL2	-0.2010061	1.38970593	-3.7272873	0.00021586	0.00123673	-1.330225	3.665823382	up-regulated in High
TBC1D5	-0.1516716	2.55105185	-3.5384441	0.00044038	0.00231475	-1.9992882	3.356176601	up-regulated in High
SATB1	-0.3555856	1.46757436	-6.4363527	2.89E-10	5.12E-09	11.7278863	9.538766147	up-regulated in High
EFHB	-0.2784177	0.44094326	-5.987775	4.09E-09	6.00E-08	9.13972358	8.38842854	up-regulated in High
KAT2B	-0.4952866	2.2309512	-9.7939794	8.02E-21	5.79E-19	35.7055321	20.09593587	up-regulated in High
SGOL1	0.85899378	1.05689611	20.3233371	3.09E-67	1.29E-63	142.164954	66.50999475	up-regulated in Low
UBE2E1	0.13251374	4.56152002	3.0793131	0.00218991	0.00945903	-3.4873573	2.65957435	up-regulated in Low
NKIRAS1	-0.1738527	1.83089033	-5.6114427	3.34E-08	4.19E-07	7.09351985	7.475915511	up-regulated in High
RPL15	-0.1245002	6.56507806	-2.4270585	0.01557725	0.05051984	-5.2579246	1.807509344	up-regulated in High
NR1D2	-0.2590379	3.08184082	-4.4068371	1.29E-05	9.75E-05	1.34700369	4.890535783	up-regulated in High
THRB	-0.1802148	1.01074806	-3.547967	0.00042514	0.00224334	-1.9663394	3.371469392	up-regulated in High
TOP2B	-0.1007876	4.42582597	-2.053932	0.04050635	0.1105006	-6.0865102	1.392476887	up-regulated in High
OXSM	0.11436083	2.18663537	3.23357762	0.00130402	0.00601928	-3.0094371	2.884715675	up-regulated in Low
NEK10	-0.1213336	0.24878437	-3.6441952	0.00029654	0.0016357	-1.6286784	3.527921477	up-regulated in High
CMC1	0.10887219	1.32985082	3.69114724	0.00024801	0.00139762	-1.4608167	3.60552509	up-regulated in Low
ZCWPW2	-0.1690805	0.42736381	-11.725242	3.43E-28	4.97E-26	52.5490895	27.46519059	up-regulated in High
RBMS3	-0.5494522	1.26931153	-10.587268	9.43E-24	9.27E-22	42.3950608	23.02530802	up-regulated in High
TGFBR2	-0.7667243	5.11371221	-11.006727	2.34E-25	2.71E-23	46.0652468	24.63090859	up-regulated in High
GPD1L	-0.8364845	3.72393463	-11.540222	1.88E-27	2.55E-25	50.8566914	26.7256382	up-regulated in High
CMTM8	-0.2535832	3.80235374	-4.4561074	1.03E-05	7.98E-05	1.55727955	4.985991757	up-regulated in High
CMTM7	-0.4719621	3.07883262	-7.7506041	5.22E-14	1.58E-12	20.1953094	13.28210107	up-regulated in High
CMTM6	-0.111851	4.95064573	-2.4699936	0.01384727	0.04578925	-5.1539237	1.858635726	up-regulated in High
CNOT10	0.14347915	2.68309803	4.95177311	1.01E-06	9.62E-06	3.79169392	5.995615549	up-regulated in Low
TRIM71	-0.1043395	0.16470271	-3.1051487	0.00201071	0.00877621	-3.4088823	2.696650413	up-regulated in High
CCR4	-0.5403645	1.23550351	-7.913246	1.65E-14	5.34E-13	21.3301187	13.78212158	up-regulated in High
CRTAP	-0.3077585	4.49073703	-6.762927	3.82E-11	7.75E-10	13.7113126	10.41800036	up-regulated in High
STAC	-0.3952994	1.19223033	-5.3367711	1.44E-07	1.61E-06	5.67399749	6.840875196	up-regulated in High
TRANK1	-0.4360196	2.1766318	-7.5127415	2.72E-13	7.42E-12	18.5690629	12.56497283	up-regulated in High
EPM2AIP1	-0.2250018	2.37284124	-5.7308622	1.74E-08	2.28E-07	7.73025806	7.760205912	up-regulated in High
ITGA9	-0.9932804	2.22808543	-13.896341	2.61E-37	6.54E-35	73.4391739	36.5830953	up-regulated in High
CTDSPL	-0.5239515	3.27540816	-9.7940316	8.01E-21	5.79E-19	35.7059607	20.09612371	up-regulated in High
VILL	-0.4812808	1.65049093	-4.8410796	1.73E-06	1.57E-05	3.27407284	5.7624099	up-regulated in High
PLCD1	-0.5289026	2.25825883	-9.6910603	1.88E-20	1.29E-18	34.8630828	19.72671411	up-regulated in High
DLEC1	-0.4003493	0.60839308	-6.4353882	2.91E-10	5.15E-09	11.7221508	9.536220961	up-regulated in High

ACAA1	-0.3067858	2.88485736	-6.9536091	1.13E-11	2.47E-10	14.9071003	10.94727146	up-regulated in High
MYD88	-0.1923111	4.25408605	-4.146445	3.97E-05	0.0002714	0.27174634	4.400936316	up-regulated in High
OXSR1	0.12897515	3.22419152	3.40030142	0.00072747	0.00359649	-2.4677692	3.138187383	up-regulated in Low
XYLB	0.20596908	1.05883401	5.27652674	1.97E-07	2.15E-06	5.37112379	6.705133058	up-regulated in Low
WDR48	-0.1178595	2.59101348	-3.6734064	0.0002654	0.00148384	-1.5244824	3.576105036	up-regulated in High
GORASP1	-0.1405297	3.0313435	-4.5651265	6.30E-06	5.09E-05	2.03020862	5.200372787	up-regulated in High
TTC21A	-0.2678269	0.8251041	-6.3412147	5.14E-10	8.73E-09	11.1656519	9.289191011	up-regulated in High
CSRNP1	-0.5084859	3.85549999	-7.0930903	4.55E-12	1.06E-10	15.7990917	11.3417261	up-regulated in High
CX3CR1	-0.7781776	1.06077708	-12.313354	1.39E-30	2.35E-28	58.0274396	29.85810821	up-regulated in High
CCR8	-0.136839	0.66162051	-3.3379457	0.0009075	0.00436602	-2.673404	3.042154774	up-regulated in High
RPSA	0.17894108	6.87582657	2.71505978	0.00685761	0.02531264	-4.5263189	2.163827147	up-regulated in Low
ENTPD3	-0.9495834	2.18723438	-11.532998	2.01E-27	2.72E-25	50.790922	26.69689468	up-regulated in High
CTNNB1	-0.1690081	6.32978667	-2.8084512	0.00517454	0.01991732	-4.2719879	2.28612823	up-regulated in High
VIPR1	-0.7877742	1.45966598	-11.813753	1.51E-28	2.21E-26	53.3640999	27.82128101	up-regulated in High
NKTR	-0.223643	2.2483498	-3.607433	0.00034061	0.00184603	-1.7586876	3.467739725	up-regulated in High
ZBTB47	-0.3316869	2.18285023	-6.9898712	8.93E-12	1.98E-10	15.1376019	11.04923112	up-regulated in High
CCDC13	-0.1631078	0.27877081	-5.914301	6.22E-09	8.83E-08	8.73114862	8.20646261	up-regulated in High
HHATL	-0.2294285	0.25808824	-3.4141643	0.00069225	0.00344255	-2.421559	3.159737273	up-regulated in High
HIGD1A	0.17816609	4.61633353	2.96328923	0.0031901	0.01310214	-3.8319872	2.496196407	up-regulated in Low
ZNF662	-0.250964	0.85923884	-4.7976535	2.13E-06	1.89E-05	3.07391155	5.672129578	up-regulated in High
FAM198A	-0.2326276	0.52843242	-7.4281234	4.85E-13	1.27E-11	18.0002446	12.31396876	up-regulated in High
SNRK	-0.2264025	2.62489273	-5.7134371	1.91E-08	2.50E-07	7.63661566	7.718416608	up-regulated in High
ZKSCAN7	-0.1856701	0.79098587	-5.8779729	7.64E-09	1.07E-07	8.53075269	8.117171488	up-regulated in High
ZNF197	-0.1051214	1.68024227	-3.3782482	0.00078695	0.00385206	-2.5409113	3.104055402	up-regulated in High
ZNF502	-0.1905674	1.29233739	-3.3656397	0.00082296	0.00400516	-2.5825244	3.084623932	up-regulated in High
KIF15	0.8227621	1.2616878	16.1747433	1.47E-47	6.69E-45	96.9611787	46.83399541	up-regulated in Low
TMEM42	-0.1309257	2.411042	-2.9736643	0.00308604	0.01272672	-3.8016893	2.510597823	up-regulated in High
ZDHHC3	-0.2891412	3.56802085	-5.7789803	1.33E-08	1.78E-07	7.9901409	7.876147464	up-regulated in High
CLEC3B	-0.9381389	2.68354993	-9.1156162	1.95E-18	1.06E-16	30.2666507	17.71076226	up-regulated in High
CDCP1	0.42337726	3.41562195	4.40435122	1.30E-05	9.84E-05	1.3364517	4.885743328	up-regulated in Low
TMEM158	0.3309943	1.44271776	3.84129946	0.00013827	0.00083264	-0.9103704	3.85926529	up-regulated in Low
LARS2	0.20707942	2.29547732	5.95717154	4.87E-09	7.06E-08	8.96901227	8.312412828	up-regulated in Low
LIMD1	-0.3975451	2.4944222	-7.0787681	5.00E-12	1.15E-10	15.7068326	11.30094066	up-regulated in High
SACM1L	-0.2281305	2.88895152	-6.0992127	2.15E-09	3.32E-08	9.76771963	8.667906856	up-regulated in High
SLC6A20	-0.458486	0.8413023	-5.1617206	3.55E-07	3.69E-06	4.80246651	6.450010654	up-regulated in High
LZTFL1	-0.219293	2.03395721	-5.4444143	8.19E-08	9.59E-07	6.22278156	7.086598915	up-regulated in High
FYCO1	-0.5107471	2.9746563	-9.7237242	1.43E-20	1.00E-18	35.1298004	19.84361724	up-regulated in High
XCR1	-0.2421861	0.42566583	-7.2901858	1.23E-12	3.07E-11	17.0840758	11.90948687	up-regulated in High
CCR1	-0.2285715	2.8439076	-2.6618462	0.00802376	0.02884927	-4.6675006	2.095622023	up-regulated in High
CCR2	-0.4599521	1.48447981	-6.8383289	2.37E-11	4.95E-10	14.1808794	10.62590419	up-regulated in High
CCR5	-0.2370974	2.06479101	-3.002355	0.00281426	0.01174983	-3.7173736	2.550635498	up-regulated in High
CCRL2	-0.3720158	1.67305896	-5.7455757	1.60E-08	2.12E-07	7.80952317	7.795573878	up-regulated in High
LTF	-1.0764038	3.49460621	-5.7113224	1.93E-08	2.53E-07	7.62526813	7.713352147	up-regulated in High
ALS2CL	-0.3534596	2.22139181	-3.9723166	8.17E-05	0.00052095	-0.413195	4.087576984	up-regulated in High
TMIE	0.14720981	0.50282133	3.91322672	0.0001038	0.00064494	-0.6393659	3.983811018	up-regulated in Low
MYL3	-0.1830289	0.36538832	-7.4895472	3.19E-13	8.60E-12	18.4126359	12.49595505	up-regulated in High
PTH1R	-0.3756894	0.84343699	-7.9144901	1.64E-14	5.29E-13	21.3388703	13.78597654	up-regulated in High
NBEAL2	-0.1837862	3.3665528	-2.4687585	0.01389456	0.0459248	-5.1569403	1.857155237	up-regulated in High
ELP6	0.11617132	2.70990748	3.65292979	0.00028688	0.001588	-1.5976048	3.542295449	up-regulated in Low
CSPG5	-0.2470516	1.0769035	-3.2418899	0.00126735	0.00586578	-2.9830489	2.897103387	up-regulated in High
SMARCC1	0.17735526	4.11488387	3.51004031	0.00048892	0.00253948	-2.0970638	3.310766399	up-regulated in Low
DHX30	0.16138078	3.47219938	4.63747303	4.52E-06	3.75E-05	2.34984885	5.345040416	up-regulated in Low
CDC25A	0.81412653	1.13614116	18.0441681	2.56E-56	2.23E-53	117.073491	55.59112197	up-regulated in Low
CAMP	-0.1680037	0.58292732	-2.7507577	0.00616333	0.02312715	-4.4300883	2.210184434	up-regulated in High
ZNF589	-0.2307868	1.67952427	-4.7964994	2.14E-06	1.90E-05	3.06861446	5.669739594	up-regulated in High
NME6	0.1074277	2.15941111	3.93862541	9.37E-05	0.00058821	-0.5425406	4.028252908	up-regulated in Low
PLXNB1	-0.3003918	3.11038191	-3.9631135	8.49E-05	0.00053787	-0.4486299	4.071329816	up-regulated in High
CCDC51	0.34280378	2.71214059	9.25257534	6.56E-19	3.76E-17	31.3427529	18.18296859	up-regulated in Low
PFKFB4	0.38173181	1.6264126	5.91976423	6.03E-09	8.58E-08	8.76137798	8.219929617	up-regulated in Low
UCN2	0.23854101	0.31306861	5.07509602	5.49E-07	5.49E-06	4.38083611	6.260616309	up-regulated in Low
COL7A1	0.46194002	1.35674002	4.59296871	5.55E-06	4.53E-05	2.15267441	5.255821056	up-regulated in Low
UQCRC1	0.33081194	5.44891177	8.57425514	1.28E-16	5.53E-15	26.1269141	15.8926212	up-regulated in Low
CELSR3	0.2982802	1.07727117	4.46467899	9.94E-06	7.70E-05	1.59408171	5.002689407	up-regulated in Low
SLC25A20	-0.1167748	3.28407576	-2.3720404	0.01807117	0.05710769	-5.3885903	1.743013646	up-regulated in High
P4HTM	-0.2053819	2.64970292	-4.2127028	3.00E-05	0.00021015	0.53957602	4.523135857	up-regulated in High
WDR6	-0.1535094	4.60960436	-2.9129917	0.00374146	0.015074	-3.9774192	2.426959262	up-regulated in High
IMPDH2	0.18667212	5.59069148	3.5054396	0.00049723	0.0025749	-2.1128305	3.303439742	up-regulated in Low
LAMB2	-0.4528567	4.67819864	-7.4850591	3.29E-13	8.85E-12	18.3824117	12.48261896	up-regulated in High
KLHDC8B	-0.4429621	3.86130237	-6.8008908	3.00E-11	6.18E-10	13.947194	10.52244961	up-regulated in High
C3orf62	-0.2096256	1.50689229	-5.9199024	6.02E-09	8.58E-08	8.7621429	8.220270377	up-regulated in High
USP4	-0.2644744	3.33299792	-7.9481775	1.29E-14	4.23E-13	21.5762361	13.89052576	up-regulated in High

GPX1	-0.1451398	7.49677083	-2.1468994	0.0322859	0.09192359	-5.892721	1.49098706	up-regulated in High
TCTA	-0.3301982	3.95149426	-5.6038962	3.48E-08	4.35E-07	7.05367846	7.458116252	up-regulated in High
AMT	-0.4450862	1.66958554	-6.0411884	3.01E-09	4.53E-08	9.43948275	8.521861963	up-regulated in High
NICN1	-0.416463	2.71510614	-8.7427488	3.55E-17	1.63E-15	27.3955515	16.45008217	up-regulated in High
DAG1	0.11098091	4.54914553	2.16650114	0.03074884	0.08842695	-5.8507866	1.512171314	up-regulated in Low
IP6K1	-0.1054582	3.46435937	-3.3027352	0.00102665	0.00487222	-2.7879129	2.988578327	up-regulated in High
CDHR4	-0.4308917	0.63882244	-5.1612931	3.56E-07	3.69E-06	4.80036988	6.449069363	up-regulated in High
FAM212A	-0.2251752	1.32595058	-4.6084905	5.17E-06	4.24E-05	2.22124471	5.286855919	up-regulated in High
UBA7	-0.6166264	3.71567172	-9.0334268	3.72E-18	1.94E-16	29.626372	17.4297251	up-regulated in High
TRAIP	0.58769788	1.36179412	14.7603591	3.96E-41	1.25E-38	82.1995616	40.40235693	up-regulated in Low
RBM6	-0.2097811	3.35596305	-3.3994829	0.0007296	0.0036058	-2.4704921	3.136917238	up-regulated in High
RBM5	-0.3182783	3.06078398	-6.3309422	5.46E-10	9.25E-09	11.1053699	9.262422187	up-regulated in High
SEMA3F	0.30023033	2.99129261	3.96849391	8.30E-05	0.00052767	-0.4279228	4.080824597	up-regulated in Low
SLC38A3	0.1385556	0.32821878	2.29191659	0.02232891	0.06797279	-5.5736402	1.65113244	up-regulated in Low
GNAI2	-0.2847725	5.36258226	-5.9541951	4.95E-09	7.17E-08	8.95244951	8.305036604	up-regulated in High
SEMA3B	-0.4892524	2.44370753	-4.2730453	2.31E-05	0.00016623	0.78693154	4.635842072	up-regulated in High
IFRD2	0.15630301	3.79880935	3.55198826	0.00041885	0.00221518	-1.9524008	3.377937385	up-regulated in Low
NAT6	-0.1704749	2.13300803	-3.9087455	0.00010568	0.00065511	-0.6563881	3.975995014	up-regulated in High
RASSF1	-0.1044467	3.04342954	-2.4384651	0.01509997	0.04924831	-5.2304683	1.821024054	up-regulated in High
ZMYND10	-0.5666605	1.22616657	-5.811039	1.11E-08	1.51E-07	8.1643409	7.953835119	up-regulated in High
TMEM115	-0.1072621	4.39613938	-2.9862903	0.00296361	0.01228777	-3.7646805	2.52817879	up-regulated in High
CACNA2D2	-1.45072	2.52790028	-10.188781	2.91E-22	2.45E-20	38.9922316	21.53570895	up-regulated in High
C3orf18	-0.2710459	1.64109512	-6.2305811	9.94E-10	1.61E-08	10.5208047	9.002738316	up-regulated in High
HEMK1	-0.1896175	1.28471621	-5.3332975	1.47E-07	1.64E-06	5.65645091	6.83301369	up-regulated in High
CISH	-0.8800217	3.21609468	-11.151873	6.38E-26	7.68E-24	47.3554909	25.19512446	up-regulated in High
MAPKAPK3	-0.3404357	4.10952802	-5.990283	4.03E-09	5.93E-08	9.15374702	8.39467216	up-regulated in High
MANF	0.2241964	5.36304151	4.01235116	6.94E-05	0.00044985	-0.2581526	4.158621989	up-regulated in Low
RBM15B	0.12149907	3.62553172	3.27280323	0.00113919	0.00533606	-2.8843414	2.943403168	up-regulated in Low
RRP9	0.3647795	3.84830044	8.26372782	1.30E-15	4.90E-14	23.8371669	14.885748	up-regulated in Low
PARP3	-0.4136191	3.35948147	-7.821286	3.17E-14	9.82E-13	20.6862238	13.49844636	up-regulated in High
ABHD14B	-0.3044748	4.33802962	-5.3299798	1.49E-07	1.67E-06	5.63970139	6.825509012	up-regulated in High
ABHD14A	-0.3551168	2.81062993	-6.0162144	3.47E-09	5.17E-08	9.29904082	8.459353458	up-regulated in High
DUSP7	-0.1673321	2.60499334	-2.9385945	0.00345078	0.01403949	-3.903691	2.462082673	up-regulated in High
POC1A	0.84414047	2.33226447	16.4183433	1.09E-48	5.24E-46	99.5480355	47.96067064	up-regulated in Low
PPM1M	-0.5153036	3.10671153	-10.842862	1.00E-24	1.09E-22	44.6209819	23.99920318	up-regulated in High
GLYCTK	0.13125514	1.37163738	2.30766441	0.02142892	0.0657749	-5.5377611	1.668999817	up-regulated in Low
DNAH1	-0.2020624	0.87905073	-4.2652976	2.39E-05	0.00017134	0.75498887	4.621295487	up-regulated in High
SEMA3G	-0.2987207	1.33947375	-5.4719204	7.08E-08	8.39E-07	6.36457309	7.150041314	up-regulated in High
TNNC1	-0.9039849	2.28388682	-8.3775476	5.60E-16	2.24E-14	24.6690851	15.25168115	up-regulated in High
NISCH	-0.3219064	3.26939391	-6.0550266	2.78E-09	4.20E-08	9.51751866	8.556589137	up-regulated in High
STAB1	-0.2691681	2.7971188	-3.4189102	0.00068056	0.00339132	-2.4056979	3.167131491	up-regulated in High
GNL3	0.3878585	4.71419241	8.46996642	2.81E-16	1.16E-14	25.3508683	15.55147749	up-regulated in Low
NEK4	0.1009089	2.10461414	2.82505743	0.00491788	0.0190424	-4.2258918	2.308221866	up-regulated in Low
ITIH3	-0.1671316	0.33709031	-8.0231041	7.51E-15	2.54E-13	22.106971	14.12424487	up-regulated in High
SFMBT1	0.13473949	1.83976506	2.76122581	0.00597214	0.02250809	-4.4016382	2.223870098	up-regulated in Low
RFT1	0.1604616	2.46235846	5.24491043	2.32E-07	2.50E-06	5.21340465	6.634408842	up-regulated in Low
PRKCD	-0.3444056	4.65682219	-6.7114755	5.28E-11	1.05E-09	13.3933813	10.27718237	up-regulated in High
TKT	0.16422886	5.81848767	2.39810857	0.01684885	0.0538925	-5.3270444	1.773429763	up-regulated in Low
CACNA1D	-0.2780568	1.02239895	-4.3311662	1.80E-05	0.00013219	1.02826818	4.745672568	up-regulated in High
CHDH	-0.139584	1.59186359	-2.2402526	0.02551695	0.07598216	-5.6896595	1.593171203	up-regulated in High
CACNA2D3	-0.1420074	0.43740981	-4.1580702	3.78E-05	0.00025968	0.31845195	4.422258864	up-regulated in High
WNT5A	-0.4380189	2.10965726	-5.457847	7.63E-08	8.98E-07	6.29194687	7.117548159	up-regulated in High
CCDC66	-0.1008702	1.18695633	-3.0857432	0.00214399	0.00928467	-3.4678851	2.668778302	up-regulated in High
ARHGEF3	-0.2250073	2.73164716	-5.2970749	1.77E-07	1.95E-06	5.47408328	6.751288021	up-regulated in High
IL17RD	0.11707081	0.79497188	2.29949037	0.02189203	0.066915	-5.5564144	1.659713928	up-regulated in Low
DNAH12	-0.2075659	0.27249969	-4.723181	3.03E-06	2.61E-05	2.73447926	5.518896385	up-regulated in High
DNASE1L3	-0.2374732	0.68039956	-3.8977617	0.00011044	0.00068099	-0.6980333	3.956869098	up-regulated in High
PXK	-0.2201154	1.98142709	-5.6471266	2.75E-08	3.49E-07	7.28255069	7.560347769	up-regulated in High
KCTD6	0.14661069	1.83293074	3.66972045	0.00026915	0.00150151	-1.5376736	3.570007374	up-regulated in Low
ACOX2	-0.4620963	2.09409125	-6.3572454	4.67E-10	7.98E-09	11.2598906	9.331034845	up-regulated in High
FAM107A	-0.6708084	1.33439228	-8.1714144	2.56E-15	9.31E-14	23.1687786	14.59164721	up-regulated in High
FAM3D	-0.5912585	1.26776771	-4.6696271	3.89E-06	3.27E-05	2.49339051	5.409950449	up-regulated in High
C3orf67	0.17363542	0.47270816	6.06167482	2.67E-09	4.06E-08	9.55506356	8.573295829	up-regulated in Low
FHIT	-0.11075	0.97703345	-3.3263394	0.00094527	0.00452762	-2.7112776	3.024442582	up-regulated in High
PTPRG	-0.3705185	1.92727856	-5.8483246	9.02E-09	1.24E-07	8.36799995	8.044632569	up-regulated in High
SNTN	-0.5604816	0.70562346	-7.3433961	8.61E-13	2.19E-11	17.4358597	12.06482714	up-regulated in High
ATXN7	-0.2701249	2.28737047	-6.1912365	1.25E-09	2.00E-08	10.2938145	8.901850279	up-regulated in High
PRICKLE2	-0.4477258	1.36241472	-10.544573	1.37E-23	1.32E-21	42.0264936	22.86401359	up-regulated in High
MAGI1	-0.3217705	1.67774874	-6.4414954	2.80E-10	4.98E-09	11.7584797	9.552341898	up-regulated in High
LRIG1	-0.5230889	3.55499241	-7.3423153	8.68E-13	2.21E-11	17.4286939	12.06166325	up-regulated in High
KBTBD8	-0.1701699	0.7501842	-4.747471	2.70E-06	2.34E-05	2.84465611	5.568653847	up-regulated in High

EOGT	-0.1450146	2.39834783	-3.3035432	0.00102376	0.00486088	-2.7852983	2.989802483	up-regulated in High
TMF1	-0.1120968	3.18368697	-2.6277943	0.00886052	0.03142627	-4.7564193	2.05254069	up-regulated in High
ARL6IP5	-0.414613	6.20842801	-6.9390334	1.24E-11	2.70E-10	14.8147267	10.90640552	up-regulated in High
FRMD4B	-0.3104909	1.97841848	-6.592399	1.11E-10	2.10E-09	12.6653561	9.954561038	up-regulated in High
MITF	-0.198647	1.50853031	-3.9007144	0.00010914	0.00067382	-0.6868491	3.962006055	up-regulated in High
FOXP1	-0.2797694	2.57229385	-6.0750262	2.47E-09	3.78E-08	9.63057099	8.606892401	up-regulated in High
EIF4E3	-0.3704521	1.80808733	-8.2551633	1.39E-15	5.20E-14	23.7749162	14.85836056	up-regulated in High
GXYLT2	-0.3437218	1.71677512	-4.7374237	2.83E-06	2.45E-05	2.79902028	5.548046327	up-regulated in High
PPP4R2	0.17395148	3.45330673	4.78241615	2.29E-06	2.02E-05	3.00406836	5.640613825	up-regulated in Low
PDZRN3	-0.3108304	1.32421986	-5.4760611	6.92E-08	8.22E-07	6.38597262	7.159614603	up-regulated in High
CNTN3	-0.2960146	0.60405255	-6.0025646	3.76E-09	5.56E-08	9.22249327	8.425278033	up-regulated in High
ROBO2	-0.3714075	0.47519744	-9.4354754	1.51E-19	9.35E-18	32.7973948	18.82104356	up-regulated in High
GBE1	0.18347651	2.92514724	3.26578308	0.00116719	0.00545628	-2.9068358	2.93285709	up-regulated in Low
VGLL3	-0.1507492	1.34370843	-2.1117299	0.03520954	0.09854648	-5.9670222	1.45333961	up-regulated in High
ZNF654	-0.1283234	1.94512119	-3.6200301	0.00032486	0.00177085	-1.7142789	3.488304467	up-regulated in High
EPHA3	-0.3042686	0.86263424	-4.2759194	2.28E-05	0.00016438	0.79879424	4.641243719	up-regulated in High
PROS1	-0.4503119	3.25304334	-5.3535562	1.32E-07	1.49E-06	5.75892902	6.878923266	up-regulated in High
STX19	-0.246116	1.70311057	-3.5022484	0.00050308	0.00260056	-2.1237552	3.298362431	up-regulated in High
MINA	0.29572881	2.21881654	8.13455497	3.35E-15	1.19E-13	22.9034986	14.47489471	up-regulated in Low
CLDND1	0.18773961	3.68454849	4.01473273	6.87E-05	0.00044582	-0.2488836	4.162867162	up-regulated in Low
CPOX	0.20849283	2.97243852	4.3767253	1.47E-05	0.00011013	1.21955675	4.832637304	up-regulated in Low
ST3GAL6	-0.3765104	1.25548491	-7.4835809	3.32E-13	8.94E-12	18.3724596	12.47822763	up-regulated in High
COL8A1	-0.743826	3.6906242	-7.2959434	1.19E-12	2.97E-11	17.1220405	11.9262531	up-regulated in High
CMSS1	0.58917613	2.37863523	12.8052135	1.24E-32	2.42E-30	62.7178156	31.90573091	up-regulated in Low
FILIP1L	-0.652508	3.03663871	-7.992399	9.37E-15	3.13E-13	21.8890101	14.02826934	up-regulated in High
NIT2	0.23964251	2.48747642	5.99124179	4.01E-09	5.90E-08	9.15910937	8.397059592	up-regulated in Low
TOMM70A	0.38860954	4.00990913	10.2571036	1.63E-22	1.41E-20	39.5696776	21.78855861	up-regulated in Low
TMEM45A	0.27050895	2.20559238	2.47200123	0.01377072	0.04560066	-5.149017	1.861043481	up-regulated in Low
TFG	0.44322333	4.66369391	10.131952	4.72E-22	3.91E-20	38.5138446	21.32621166	up-regulated in Low
ABI3BP	-0.7285151	1.59036509	-9.7663077	1.01E-20	7.14E-19	35.4784313	19.99641153	up-regulated in High
SENP7	-0.1693916	1.94452103	-4.2048868	3.10E-05	0.00021666	0.50777612	4.508636004	up-regulated in High
TRMT10C	0.43196645	3.69989643	9.71283505	1.57E-20	1.09E-18	35.0408173	19.80461653	up-regulated in Low
PCNP	0.1424373	5.28873238	3.44412251	0.0006215	0.003133	-2.3210844	3.20655576	up-regulated in Low
CEP97	0.17436804	1.21985559	4.90077621	1.30E-06	1.21E-05	3.55190473	5.887628089	up-regulated in Low
NFKBIZ	-0.3207122	3.09876792	-3.5724725	0.00038817	0.00207034	-1.8811648	3.410980017	up-regulated in High
ALCAM	-0.3164961	4.89990564	-3.7040678	0.00023603	0.00133877	-1.4142665	3.627026158	up-regulated in High
BBX	0.17203432	2.52951106	3.6460698	0.00029444	0.00162505	-1.6220153	3.531004014	up-regulated in Low
CD47	-0.5342427	4.87572323	-7.8975238	1.85E-14	5.91E-13	21.2196194	13.73344681	up-regulated in High
IFT57	-0.820012	4.32718922	-8.7975052	2.33E-17	1.10E-15	27.8117266	16.63289928	up-regulated in High
HHLA2	-0.9182667	1.56192625	-5.1558055	3.66E-07	3.78E-06	4.77347162	6.436992853	up-regulated in High
KIAA1524	0.93631076	1.48686551	16.8997698	6.29E-51	3.43E-48	104.69316	50.20125588	up-regulated in Low
RETNLB	0.1269512	0.11691218	4.85155969	1.64E-06	1.50E-05	3.32262391	5.784299572	up-regulated in Low
TRAT1	-0.2174402	0.80506328	-4.1025455	4.78E-05	0.00032131	0.09647607	4.320870568	up-regulated in High
DPPA2	0.11689927	0.08556271	2.77265008	0.00576961	0.02185909	-4.37047	2.238853272	up-regulated in Low
PVRL3	-0.4488888	1.52062348	-5.5724435	4.13E-08	5.10E-07	6.88813386	7.384144483	up-regulated in High
CD96	-0.2870658	1.53303031	-3.7798596	0.00017603	0.0010315	-1.1381085	3.754415191	up-regulated in High
ABHD10	0.2486153	3.25146968	6.78768567	3.27E-11	6.69E-10	13.8650219	10.48606605	up-regulated in Low
TAGLN3	0.24773584	0.24668124	4.3075086	1.99E-05	0.00014502	0.92966904	4.700816314	up-regulated in Low
C3orf52	-0.148814	1.70013113	-2.8397095	0.00470108	0.018322	-4.1850018	2.327802509	up-regulated in High
GCSAM	-0.2233382	0.73523613	-7.05299	5.92E-12	1.35E-10	15.541163	11.22769478	up-regulated in High
CD200	-0.1742047	1.97322611	-2.9099045	0.00377798	0.01519338	-3.9862674	2.422740825	up-regulated in High
BTLA	-0.2004918	0.54512294	-5.4971297	6.19E-08	7.42E-07	6.49508015	7.208418483	up-regulated in High
ATG3	0.13328924	3.06745831	4.10871963	4.65E-05	0.00031391	0.1210213	4.332087925	up-regulated in Low
SLC35A5	-0.1069463	2.90064396	-2.5847698	0.01002914	0.03490925	-4.8671753	1.998736467	up-regulated in High
CCDC80	-0.3758335	3.1976836	-3.6630999	0.00027602	0.00153468	-1.5613354	3.559067944	up-regulated in High
CD200R1	-0.231347	0.69984725	-6.3585907	4.63E-10	7.92E-09	11.2678082	9.334550181	up-regulated in High
GTPBP8	0.20890003	1.79579057	6.37510544	4.19E-10	7.23E-09	11.365121	9.377753683	up-regulated in Low
C3orf17	0.14914687	2.81700816	4.71740051	3.11E-06	2.67E-05	2.70833575	5.507086821	up-regulated in Low
BOC	-0.1921194	0.70287377	-4.7994956	2.11E-06	1.88E-05	3.08236897	5.675945379	up-regulated in High
CFAP44	-0.1896049	0.96363262	-3.8900439	0.00011391	0.00069955	-0.7272297	3.943457183	up-regulated in High
SPICE1	0.1996944	1.75576211	5.04335526	6.43E-07	6.35E-06	4.22795269	6.191889752	up-regulated in Low
SIDT1	-0.3477043	1.31879635	-5.576915	4.03E-08	4.98E-07	6.91161872	7.394639837	up-regulated in High
NAA50	0.50155107	4.32615677	11.4151221	5.90E-27	7.65E-25	49.7211796	26.22934367	up-regulated in Low
ZDHHC23	0.23832708	1.3704983	5.12239893	4.33E-07	4.42E-06	4.61027938	6.363706765	up-regulated in Low
KIAA1407	-0.2114135	1.39830382	-4.2598785	2.45E-05	0.0001749	0.73267926	4.611134391	up-regulated in High
QTRTD1	0.25905252	2.00785952	7.47990203	3.41E-13	9.15E-12	18.3477	12.46730241	up-regulated in Low
GAP43	0.14245195	0.34770388	2.8691514	0.00429146	0.01695187	-4.1022179	2.36739464	up-regulated in Low
LSAMP	-0.3995071	1.03343797	-7.7818735	4.19E-14	1.28E-12	20.4120585	13.3776294	up-regulated in High
UPK1B	0.53855782	0.58022858	3.84910641	0.00013406	0.0008093	-0.8811847	3.872689636	up-regulated in Low
B4GALT4	0.33264878	2.43540638	5.76761848	1.42E-08	1.89E-07	7.928605	7.848699043	up-regulated in Low
ARHGAP31	-0.672362	2.19195687	-10.337256	8.19E-23	7.38E-21	40.2502942	22.08654591	up-regulated in High

TMEM39A	0.13945923	3.16126878	3.50504553	0.00049795	0.00257725	-2.1141801	3.302812556	up-regulated in Low
TIMMDC1	0.27987022	4.42366107	6.84306683	2.30E-11	4.81E-10	14.2105293	10.63902887	up-regulated in Low
CD80	-0.1423629	0.71856258	-3.7520306	0.00019616	0.00113645	-1.2401215	3.707389961	up-regulated in High
ADPRH	-0.3676023	2.06467133	-7.5956335	1.54E-13	4.34E-12	19.1312408	12.81295713	up-regulated in High
PLA1A	-0.3727462	1.90251827	-3.7591559	0.00019081	0.00110842	-1.2140701	3.719402476	up-regulated in High
POPDC2	-0.1806494	0.77865996	-6.4042437	3.51E-10	6.13E-09	11.5373392	9.454200617	up-regulated in High
COX17	0.39668343	3.68711939	7.91207498	1.67E-14	5.38E-13	21.3218827	13.77849376	up-regulated in Low
MAATS1	-0.2604861	0.59865292	-4.5864045	5.72E-06	4.65E-05	2.12373944	5.242722668	up-regulated in High
GSK3B	0.17654224	3.11686038	4.11753823	4.49E-05	0.00030354	0.15613927	4.348134377	up-regulated in Low
LRRC58	0.23456827	2.75792443	4.97677471	8.94E-07	8.60E-06	3.91007358	6.048898978	up-regulated in Low
FSTL1	-0.2049028	4.55261786	-2.3137988	0.021087	0.06488915	-5.5237196	1.675985198	up-regulated in High
NDUFB4	0.40983815	5.40909217	8.50142986	2.22E-16	9.29E-15	25.584253	15.65408279	up-regulated in Low
HGD	0.70686742	2.0887616	4.47185661	9.62E-06	7.48E-05	1.62494911	5.016692368	up-regulated in Low
RABL3	0.12535182	2.7005878	3.38877352	0.00075801	0.0037314	-2.5060594	3.120322607	up-regulated in Low
GTF2E1	0.32448935	2.51335676	8.77629059	2.74E-17	1.27E-15	27.6502598	16.56197353	up-regulated in Low
STXBP5L	0.14267063	0.07489804	5.407407	9.96E-08	1.15E-06	6.03301319	7.001660804	up-regulated in Low
POLQ	0.61595889	0.7016318	16.8290088	1.35E-50	7.21E-48	103.934334	49.87082921	up-regulated in Low
HCLS1	-0.4402481	3.66120298	-5.3292316	1.50E-07	1.67E-06	5.63592551	6.823817179	up-regulated in High
IQCB1	0.11027881	3.07868364	2.08004234	0.03803498	0.10493542	-6.0329347	1.419816812	up-regulated in Low
SLC15A2	-0.9179494	2.0952177	-10.83868	1.04E-24	1.12E-22	44.5842979	23.98315605	up-regulated in High
ILDR1	-0.3720579	2.69312187	-6.0793785	2.41E-09	3.69E-08	9.65521602	8.617857292	up-regulated in High
CD86	-0.2707969	2.58390279	-3.5533758	0.00041671	0.00220542	-1.9475877	3.380170624	up-regulated in High
CASR	-0.2183641	0.20972624	-3.8822549	0.00011751	0.00071961	-0.75664	3.929944198	up-regulated in High
CCDC58	0.70187554	3.08959292	15.2260739	3.18E-43	1.16E-40	87.0073186	42.49758739	up-regulated in Low
FAM162A	0.45391207	3.59211313	8.09150773	4.58E-15	1.60E-13	22.5948458	14.3390348	up-regulated in Low
KPNA1	0.29118414	2.70531142	8.36477822	6.16E-16	2.43E-14	24.5753256	15.2104461	up-regulated in Low
DTX3L	0.14498696	4.39247656	2.56059196	0.01074462	0.03697884	-4.9286341	1.968808998	up-regulated in Low
PARP15	-0.4112359	1.17920469	-6.1720706	1.40E-09	2.23E-08	10.1836874	8.852892539	up-regulated in High
PDIA5	0.123947	3.47160071	2.25335151	0.02467337	0.07392709	-5.6604891	1.607771558	up-regulated in Low
SEC22A	0.11944429	2.15593721	3.43596141	0.00064008	0.00321122	-2.3485382	3.193768007	up-regulated in Low
PTPLB	0.22219382	3.89564799	4.44083414	1.11E-05	8.49E-05	1.49186532	4.956305917	up-regulated in Low
MYLK	-0.4741283	2.31887891	-6.5355966	1.58E-10	2.91E-09	12.3219199	9.802285637	up-regulated in High
UMPS	0.390348	2.33440349	11.3603603	9.70E-27	1.23E-24	49.2263847	26.01306093	up-regulated in Low
ITGB5	-0.2336335	4.58528222	-3.6595725	0.00027974	0.00155288	-1.573926	3.553246124	up-regulated in High
MUC13	1.04957654	2.13147148	4.92521471	1.15E-06	1.08E-05	3.6665346	5.939260504	up-regulated in Low
HEG1	-0.3339705	2.92099404	-4.3120477	1.95E-05	0.00014252	0.94854805	4.709406686	up-regulated in High
SLC12A8	0.35625147	2.34187869	5.38531041	1.12E-07	1.28E-06	5.9202531	6.951174545	up-regulated in Low
SNX4	0.13779933	4.05833135	3.56867932	0.00039369	0.00209785	-1.8943852	3.404849419	up-regulated in Low
ALG1L	0.34772565	2.75887697	2.80352408	0.00525299	0.02017275	-4.2856141	2.279593155	up-regulated in Low
KLF15	-0.5418614	1.59153415	-5.9317103	5.63E-09	8.05E-08	8.82756287	8.24941244	up-regulated in High
CCDC37	-0.2745809	0.41011166	-4.5752167	6.02E-06	4.88E-05	2.07451173	5.220434741	up-regulated in High
PLXNA1	0.14369671	3.33516991	2.05979653	0.03993969	0.10924057	-6.0745348	1.398595352	up-regulated in Low
MCM2	1.12146266	3.4193259	15.5100125	1.64E-44	6.32E-42	89.9651132	43.78635264	up-regulated in Low
ABTB1	-0.4279104	2.58241845	-8.4896349	2.42E-16	1.01E-14	25.4966861	15.61558603	up-regulated in High
MGLL	-0.9657483	4.16987742	-11.293945	1.77E-26	2.20E-24	48.6281696	25.75155137	up-regulated in High
SEC61A1	0.25534063	6.67958435	6.27362633	7.70E-10	1.27E-08	10.7705515	9.113707228	up-regulated in Low
RUVBL1	0.62413951	3.18731619	14.5690848	2.83E-40	8.46E-38	80.2416463	39.54893908	up-regulated in Low
GATA2	-0.139752	1.81161097	-2.0029751	0.0457241	0.12162612	-6.1891514	1.339854793	up-regulated in High
RPN1	0.29760068	6.64556758	7.85861877	2.44E-14	7.66E-13	20.9469184	13.61331018	up-regulated in Low
RAB7A	0.19070376	6.60717384	5.17059893	3.39E-07	3.55E-06	4.84604229	6.469573029	up-regulated in Low
ACAD9	0.27151359	3.37090151	7.21449991	2.04E-12	4.95E-11	16.5872586	11.69003637	up-regulated in Low
EFCC1	-0.5593882	1.18200401	-10.462619	2.78E-23	2.64E-21	41.3216567	22.55552811	up-regulated in High
ISY1	0.20202677	2.59850244	5.98594074	4.13E-09	6.06E-08	9.12947022	8.383863378	up-regulated in Low
CNBP	0.2280113	7.29320223	6.08240821	2.37E-09	3.63E-08	9.67238079	8.625493897	up-regulated in Low
COPG1	0.26840994	6.02591214	6.48646811	2.13E-10	3.85E-09	12.0268987	9.67143254	up-regulated in Low
HMCES	0.3034194	4.33494524	7.83792555	2.82E-14	8.80E-13	20.8022984	13.54959162	up-regulated in Low
H1FX	0.24344962	6.02968734	4.2285814	2.80E-05	0.00019755	0.60434807	4.552662654	up-regulated in Low
EFCAB12	-0.1441478	0.32424088	-3.8905113	0.00011369	0.00069853	-0.725463	3.944268825	up-regulated in High
MBD4	0.12361618	3.74725665	2.98330887	0.00299211	0.01238835	-3.7734331	2.524021875	up-regulated in Low
PLXND1	-0.4232152	4.31667402	-5.4105141	9.80E-08	1.13E-06	6.04890184	7.00877368	up-regulated in High
COL6A5	-0.3538556	0.53349248	-8.3485725	6.95E-16	2.73E-14	24.4564893	15.15817999	up-regulated in High
COL6A6	-0.4624037	0.70717755	-8.2362813	1.59E-15	5.95E-14	23.6378469	14.79805365	up-regulated in High
PIK3R4	0.1720722	2.8459673	4.21292277	3.00E-05	0.00020998	0.54047162	4.523544189	up-regulated in Low
ATP2C1	0.19473734	3.92265403	3.67393453	0.00026486	0.00148156	-1.5225914	3.576979108	up-regulated in Low
NEK11	-0.2045206	1.4880165	-3.8670626	0.00012484	0.00075958	-0.8138451	3.903652643	up-regulated in High
NUDT16	-0.2982072	3.09461095	-5.5926375	3.70E-08	4.60E-07	6.99432655	7.431598161	up-regulated in High
MRPL3	0.51717978	5.11442572	12.3152087	1.36E-30	2.32E-28	58.044946	29.86575257	up-regulated in Low
CPNE4	-0.1126342	0.59024721	-2.2098117	0.02757462	0.08092482	-5.7568057	1.559490504	up-regulated in High
DNAJC13	0.16495909	2.77424096	3.69046299	0.00024866	0.00140101	-1.4632776	3.604388187	up-regulated in Low
ACKR4	-0.1107777	0.48864438	-2.5562761	0.01087702	0.03738823	-4.9395456	1.963490135	up-regulated in High
UBA5	0.17690529	2.62724004	5.14133074	3.93E-07	4.04E-06	4.70264484	6.405189836	up-regulated in Low

NPHP3	-0.1045815	0.93699829	-3.4024479	0.00072191	0.00357294	-2.460626	3.141519299	up-regulated in High
TMEM108	-0.3961148	1.11779076	-5.7310416	1.74E-08	2.28E-07	7.73122383	7.760636868	up-regulated in High
CDV3	0.28589949	5.21925441	5.82005672	1.06E-08	1.44E-07	8.21349299	7.975751365	up-regulated in Low
TOPBP1	0.65246274	2.87449321	12.5688245	1.21E-31	2.20E-29	60.4517689	30.91658711	up-regulated in Low
TF	0.66521581	0.83754842	5.10449438	4.74E-07	4.81E-06	4.52320781	6.324592213	up-regulated in Low
SRPRB	0.3972281	4.35243707	8.27294745	1.22E-15	4.60E-14	23.9042339	14.9152535	up-regulated in Low
RAB6B	0.15516325	1.70674222	1.99721891	0.04634773	0.12297123	-6.2005864	1.333971542	up-regulated in Low
C3orf36	-0.2656775	0.64918277	-5.6810687	2.29E-08	2.93E-07	7.46333124	7.641067887	up-regulated in High
SLCO2A1	-0.7872113	3.40559325	-7.7236889	6.31E-14	1.89E-12	20.0092901	13.20010691	up-regulated in High
RYK	0.1009849	3.09067708	2.39784903	0.01686065	0.05392405	-5.3276604	1.773125668	up-regulated in Low
EPHB1	0.20328327	0.50621842	3.77001049	0.00018292	0.00106705	-1.1742938	3.737738982	up-regulated in Low
PPP2R3A	-0.1537966	2.03310093	-2.5905442	0.00986468	0.03444998	-4.8524139	2.005916855	up-regulated in High
PCCB	0.36511901	2.72126291	7.85477666	2.50E-14	7.87E-13	20.9200444	13.60147007	up-regulated in Low
STAG1	0.22857435	2.07105758	4.89981304	1.30E-06	1.21E-05	3.54739751	5.885597552	up-regulated in Low
SLC35G2	0.42990126	1.25587874	6.53752054	1.56E-10	2.88E-09	12.3335117	9.80742619	up-regulated in Low
NCK1	0.23014462	3.22497777	4.64406823	4.38E-06	3.65E-05	2.37921692	5.358323537	up-regulated in Low
IL20RB	0.7104692	1.330914	5.65783141	2.60E-08	3.30E-07	7.33946332	7.585762589	up-regulated in Low
CLDN18	-1.430587	2.39058412	-7.3889607	6.33E-13	1.64E-11	17.7387291	12.19853744	up-regulated in High
DZIP1L	-0.1332074	0.93533856	-2.4722843	0.01375995	0.04557793	-5.1483248	1.861383119	up-regulated in High
DBR1	0.1540013	2.67583114	4.34718051	1.67E-05	0.00012403	1.09529592	4.776153911	up-regulated in Low
NME9	-0.1765463	0.36187707	-4.7767496	2.35E-06	2.07E-05	2.97814644	5.628915075	up-regulated in High
MRAS	-0.1656327	2.0836418	-3.1822339	0.00155318	0.00702823	-3.170993	2.808779461	up-regulated in High
ESYT3	-0.7212297	1.35136286	-10.573387	1.06E-23	1.04E-21	42.2751277	22.97282343	up-regulated in High
CEP70	-0.1208866	2.8847354	-2.0195084	0.04397218	0.11792769	-6.1561268	1.356821999	up-regulated in High
MRPS22	0.27804883	2.53581108	8.36630439	6.09E-16	2.41E-14	24.5865259	15.21537203	up-regulated in Low
COPB2	0.27196049	4.66620119	6.52978947	1.63E-10	3.01E-09	12.28695	9.786777356	up-regulated in Low
RBP2	-0.1617682	0.3739413	-2.9718684	0.00310383	0.01278923	-3.806941	2.508102091	up-regulated in High
RASA2	-0.1624719	2.32898701	-3.6070406	0.00034111	0.00184824	-1.7600686	3.467100073	up-regulated in High
RNF7	0.28150339	4.30280229	6.91015453	1.49E-11	3.22E-10	14.6321779	10.8256367	up-regulated in Low
ATP1B3	0.40756843	4.93679724	6.97399901	9.90E-12	2.18E-10	15.0365884	11.00455142	up-regulated in Low
ATR	0.18574812	1.7888444	4.85671908	1.60E-06	1.47E-05	3.34656086	5.795090518	up-regulated in Low
U2SURP	0.36948314	3.30048597	8.5959369	1.09E-16	4.73E-15	26.2891402	15.9639212	up-regulated in Low
CHST2	-0.238086	2.23951409	-2.5997017	0.00960882	0.03369551	-4.8289385	2.017329958	up-regulated in High
SLC9A9	-0.3515274	1.52883546	-6.5766667	1.22E-10	2.30E-09	12.5699862	9.912280657	up-regulated in High
PLOD2	1.07987171	3.59195872	9.88475706	3.77E-21	2.88E-19	36.4535789	20.42372051	up-regulated in Low
PLSCR4	-0.4817827	2.56699709	-7.5288347	2.44E-13	6.68E-12	18.6778242	12.61295572	up-regulated in High
PLSCR1	0.18685075	4.09981543	2.7716417	0.00578724	0.02192017	-4.3732261	2.237528761	up-regulated in Low
ZIC1	0.19840278	0.19119874	4.04228425	6.14E-05	0.00040149	-0.1412787	4.212131818	up-regulated in Low
AGTR1	-0.1741621	0.55572818	-3.1719883	0.00160788	0.00722816	-3.2029344	2.793746113	up-regulated in High
CPA3	-1.3524059	3.23382513	-11.064225	1.40E-25	1.64E-23	46.5751397	24.85389512	up-regulated in High
GYG1	0.20513675	3.72759184	4.36583179	1.54E-05	0.0001152	1.17364966	4.811773765	up-regulated in Low
HLTF	0.50382646	3.09815891	8.1137967	3.90E-15	1.37E-13	22.7545034	14.4093139	up-regulated in Low
HPS3	0.22301579	2.94724789	4.48624507	9.02E-06	7.04E-05	1.68696447	5.044820064	up-regulated in Low
TM4SF18	-0.1905861	1.44124457	-2.5156072	0.01219787	0.0411865	-5.0414858	1.913716069	up-regulated in High
TM4SF1	-0.3088172	6.37690869	-2.9382697	0.00345433	0.01405199	-3.90463	2.461635645	up-regulated in High
WWTR1	-0.1483302	3.40977792	-2.4384261	0.01510158	0.04925072	-5.2305625	1.82097774	up-regulated in High
COMMD2	0.23658472	2.97954208	5.09518181	4.96E-07	5.01E-06	4.47802835	6.304293054	up-regulated in Low
PFN2	1.31123853	4.39655882	11.6170865	9.29E-28	1.31E-25	51.5579133	27.0320813	up-regulated in Low
TSC22D2	0.20583726	2.08706878	3.5939559	0.00035826	0.00192682	-1.806036	3.445804545	up-regulated in Low
EIF2A	0.24064835	4.52153111	6.5090251	1.86E-10	3.39E-09	12.1621228	9.731414746	up-regulated in Low
SELT	0.15444783	4.70166734	2.73684061	0.00642608	0.02396214	-4.4677495	2.192054191	up-regulated in Low
SIAH2	0.2994757	3.6623545	6.93811257	1.25E-11	2.71E-10	14.8088961	10.90382597	up-regulated in Low
GPR171	-0.3725755	1.55882796	-4.0416479	6.15E-05	0.00040228	-0.1437718	4.210990792	up-regulated in High
P2RY14	-0.3245806	1.12775133	-6.6643548	7.10E-11	1.38E-09	13.1039869	10.14896642	up-regulated in High
P2RY13	-0.4863112	1.64072452	-6.7715901	3.62E-11	7.36E-10	13.765043	10.44179442	up-regulated in High
P2RY12	-0.472751	0.75962314	-9.5318738	6.91E-20	4.46E-18	33.5720386	19.16073286	up-regulated in High
IGSF10	-0.1884405	0.55474086	-3.5863181	0.00036864	0.00197699	-1.8327949	3.433403727	up-regulated in High
AADAC	-0.4164261	1.08835399	-3.5876727	0.00036677	0.00196828	-1.8280531	3.435601427	up-regulated in High
MBNL1	-0.2592397	4.19635159	-4.9668824	9.38E-07	9.00E-06	3.86317023	6.027789668	up-regulated in High
ARHGEF26	-0.2659667	1.77786708	-3.8384374	0.00013985	0.00084091	-0.921056	3.854349588	up-regulated in High
DHX36	0.283506	2.64825564	7.39805723	5.95E-13	1.54E-11	17.7993742	12.22530762	up-regulated in Low
PLCH1	-0.4517786	2.16204721	-5.9719903	4.48E-09	6.52E-08	9.05157901	8.349181207	up-regulated in High
SLC33A1	0.17138541	2.69305085	4.416883	1.23E-05	9.37E-05	1.38970226	4.909926155	up-regulated in Low
GMPS	0.5976465	3.11340923	13.018284	1.57E-33	3.18E-31	64.7784414	32.80502851	up-regulated in Low
KCNAB1	-0.1232273	0.48257154	-5.3783374	1.16E-07	1.32E-06	5.88475455	6.93527819	up-regulated in High
SSR3	0.29723666	5.69822221	5.66297354	2.52E-08	3.22E-07	7.36683541	7.597984902	up-regulated in Low
TIPARP	-0.2692194	3.58853933	-2.7931545	0.00542164	0.02073311	-4.3142159	2.265869608	up-regulated in High
VEPH1	-0.6622681	1.3630793	-7.7228362	6.35E-14	1.90E-12	20.003405	13.19751272	up-regulated in High
SHOX2	0.28554292	0.41516305	5.5479671	4.71E-08	5.76E-07	6.75987648	7.326818086	up-regulated in Low
RSRC1	0.41139694	2.40835262	10.3085437	1.05E-22	9.31E-21	40.0060911	21.97963369	up-regulated in Low
GFM1	0.34277929	2.76156368	8.90857375	9.86E-18	4.85E-16	28.6617079	17.00619457	up-regulated in Low

MFSD1	-0.138071	4.0230021	-2.9069463	0.00381327	0.01531052	-3.9947373	2.418702061	up-regulated in High
IQCJ-SCHIP1	0.11287649	0.56275766	3.82002137	0.00015039	0.00089708	-0.9896334	3.822792851	up-regulated in Low
IFT80	-0.1927966	1.86093862	-3.7840321	0.00017319	0.00101615	-1.1227522	3.76149085	up-regulated in High
SMC4	0.56179532	2.76795681	8.40405549	4.60E-16	1.85E-14	24.864063	15.33742636	up-regulated in Low
TRIM59	0.2984746	1.19640778	7.11836001	3.86E-12	9.03E-11	15.9622406	11.41384293	up-regulated in Low
KPNA4	0.34033424	3.47237068	8.65943798	6.71E-17	2.98E-15	26.7660063	16.17348278	up-regulated in Low
ARL14	0.33621154	0.71429526	2.9987371	0.00284728	0.01186696	-3.7280487	2.545569577	up-regulated in Low
B3GALNT1	0.36902064	1.97029899	4.92511062	1.15E-06	1.08E-05	3.66604523	5.939040116	up-regulated in Low
NMD3	0.42025972	3.60504376	8.6279141	8.52E-17	3.75E-15	26.5289514	16.06931241	up-regulated in Low
SPTSSB	0.20521655	0.75328841	2.02934181	0.04295741	0.11572179	-6.1363582	1.366961946	up-regulated in Low
BCHE	-0.2750442	0.753047	-4.5385639	7.12E-06	5.69E-05	1.91400927	5.147737171	up-regulated in High
ZBBX	-0.2541431	0.43419956	-4.1974679	3.20E-05	0.00022307	0.47764257	4.494893725	up-regulated in High
WDR49	-0.1473371	0.4314071	-3.0195147	0.00266237	0.01119861	-3.6665717	2.574731253	up-regulated in High
PDCD10	0.39038927	3.6824292	7.57522062	1.77E-13	4.95E-12	18.9923455	12.75169659	up-regulated in Low
MECOM	-0.5005669	2.79812317	-5.2222325	2.61E-07	2.78E-06	5.10079737	6.58389747	up-regulated in High
ACTRT3	0.11762791	1.03021607	3.11140245	0.00196941	0.00862497	-3.3897918	2.705663138	up-regulated in Low
MYNN	0.1553512	2.10497	4.05193518	5.90E-05	0.00038779	-0.1034233	4.229455505	up-regulated in Low
LRRC31	-0.6687271	1.14126754	-6.2136275	1.10E-09	1.77E-08	10.4228437	8.959202146	up-regulated in High
SEC62	-0.1066892	4.61682664	-2.4007684	0.01672832	0.05356861	-5.3207278	1.776547624	up-regulated in High
GPR160	-0.6474365	3.18521093	-7.1875131	2.44E-12	5.87E-11	16.4111268	11.61221748	up-regulated in High
PHC3	-0.1287928	1.83613095	-3.025564	0.00261064	0.01101197	-3.6485961	2.583252348	up-regulated in High
RPL22L1	0.45938761	3.55902387	5.93777026	5.44E-09	7.80E-08	8.86118131	8.264387029	up-regulated in Low
EIF5A2	0.14828688	1.07746441	3.15172436	0.00172135	0.00766742	-3.2658173	2.764130264	up-regulated in Low
TNIK	-0.5699644	1.78161476	-7.1207125	3.80E-12	8.91E-11	15.9774534	11.42056697	up-regulated in High
PLD1	0.14274024	1.28059121	3.12273611	0.00189655	0.00834281	-3.3551002	2.722034692	up-regulated in Low
TMEM212	-0.2313059	0.25435612	-5.1033669	4.76E-07	4.83E-06	4.51773412	6.322133008	up-regulated in High
FNDC3B	0.13310488	3.44516916	2.34107907	0.01962358	0.06113665	-5.4608343	1.707221765	up-regulated in Low
TNFSF10	-0.4780102	4.99819884	-4.5396196	7.08E-06	5.66E-05	1.91861588	5.149824324	up-regulated in High
ECT2	1.14201737	3.18871692	16.4633989	6.77E-49	3.29E-46	100.027754	48.1695946	up-regulated in Low
NAALADL2	-0.2162599	1.09710765	-3.9979686	7.36E-05	0.00047394	-0.31402	4.133029923	up-regulated in High
TBL1XR1	0.16663154	3.92219004	3.74188758	0.00020402	0.00117617	-1.2771257	3.69032279	up-regulated in Low
ZMAT3	-0.231489	2.0555588	-5.0471098	6.31E-07	6.25E-06	4.245992	6.200000551	up-regulated in High
ZNF639	0.36631763	2.38581622	9.84061162	5.44E-21	4.04E-19	36.0892214	20.26407098	up-regulated in Low
MFN1	0.23748304	3.05804194	5.77727027	1.34E-08	1.80E-07	7.98087242	7.872013391	up-regulated in Low
GNB4	0.19739837	2.42017127	3.02028151	0.00265576	0.01117725	-3.6642949	2.575810658	up-regulated in Low
ACTL6A	0.59592845	4.04084704	11.7891366	1.90E-28	2.77E-26	53.1370836	27.72209776	up-regulated in Low
MRPL47	0.56053695	4.51913941	11.5751794	1.37E-27	1.88E-25	51.1752704	26.86486489	up-regulated in Low
NDUFB5	0.28147861	3.63044714	5.63506113	2.94E-08	3.71E-07	7.21851752	7.531750076	up-regulated in Low
CCDC39	-0.2030659	0.54717016	-5.4870432	6.53E-08	7.80E-07	6.44279941	7.185034605	up-regulated in High
FXR1	0.36310068	3.27603252	9.19265201	1.06E-18	5.92E-17	30.8705299	17.97577072	up-regulated in Low
DNAJC19	0.18822923	2.82793302	4.70842332	3.25E-06	2.77E-05	2.66779231	5.488770387	up-regulated in Low
SOX2	0.328754	2.33922575	2.03056523	0.04283255	0.11546003	-6.1338921	1.368226032	up-regulated in Low
ATP11B	0.14017302	2.90013427	2.70274827	0.00711293	0.02606204	-4.559224	2.147951619	up-regulated in Low
DCUN1D1	0.29149964	2.41535177	7.09396475	4.53E-12	1.05E-10	15.8047292	11.34421821	up-regulated in Low
MCCC1	-0.1454948	3.12792997	-2.3108855	0.02124878	0.06529723	-5.5303925	1.672666023	up-regulated in High
LAMP3	-0.9562395	4.21732628	-7.5384863	2.28E-13	6.27E-12	18.7431412	12.64177047	up-regulated in High
B3GNT5	0.69604873	2.29380397	10.3448714	7.67E-23	6.95E-21	40.3151423	22.11493557	up-regulated in Low
KLHL6	-0.394651	1.33963937	-6.411851	3.36E-10	5.88E-09	11.5824111	9.474205385	up-regulated in High
KLHL24	-0.1146588	3.07231328	-2.0748651	0.03851452	0.10600299	-6.0436107	1.414375488	up-regulated in High
YEATS2	0.32723305	2.46894802	6.30126154	6.53E-10	1.09E-08	10.9316627	9.185274886	up-regulated in Low
MAP6D1	0.33749936	0.94156412	7.44669571	4.28E-13	1.13E-11	18.1246507	12.36887378	up-regulated in Low
PARL	0.36637068	4.54108348	9.08188218	2.54E-18	1.36E-16	30.0033519	17.59519963	up-regulated in Low
EIF2B5	0.19407296	3.42424704	5.28743841	1.86E-07	2.04E-06	5.42575367	6.72962405	up-regulated in Low
DVL3	0.28808958	4.12406509	5.73928697	1.66E-08	2.19E-07	7.77562259	7.780448082	up-regulated in Low
AP2M1	0.27963386	5.91407799	6.40597617	3.48E-10	6.08E-09	11.5476	9.458754884	up-regulated in Low
ABCF3	0.2866632	3.59261412	8.24728813	1.47E-15	5.50E-14	23.7177194	14.8331959	up-regulated in Low
ALG3	0.49289975	4.0819073	10.2105413	2.42E-22	2.05E-20	39.1758694	21.61612279	up-regulated in Low
ECE2	0.63888739	1.51380844	13.2937174	1.05E-34	2.30E-32	67.4666841	33.97799143	up-regulated in Low
CAMK2N2	0.21470519	0.49197521	3.59457097	0.00035743	0.00192292	-1.8038788	3.446804141	up-regulated in Low
PSMD2	0.55019544	5.4163773	11.6324043	8.07E-28	1.14E-25	51.6979736	27.09328609	up-regulated in Low
EIF4G1	0.39406028	5.82707047	7.25817094	1.53E-12	3.76E-11	16.8734126	11.8164439	up-regulated in Low
CLCN2	0.31118939	1.56268279	7.03412343	6.70E-12	1.52E-10	15.4202251	11.17421953	up-regulated in Low
POLR2H	0.44597686	4.1337554	9.51025828	8.23E-20	5.27E-18	33.3978656	19.08436244	up-regulated in Low
THPO	0.33220366	0.85161815	3.76275954	0.00018816	0.00109464	-1.2008766	3.725485197	up-regulated in Low
MAGEF1	0.29283351	4.87905836	5.32378948	1.54E-07	1.72E-06	5.6084741	6.811516767	up-regulated in Low
MAP3K13	-0.1197834	2.59102452	-2.523698	0.01192427	0.04039629	-5.0213325	1.923568324	up-regulated in High
TMEM41A	0.1739977	2.60588328	3.91731341	0.00010211	0.00063619	-0.6238263	3.990945503	up-regulated in Low
LIPH	-0.5235151	3.76823588	-4.3473419	1.67E-05	0.00012396	1.09597256	4.776461568	up-regulated in High
SENP2	0.23996764	2.83192239	5.42946503	8.87E-08	1.03E-06	6.14598549	7.05222996	up-regulated in Low
IGF2BP2	0.50414149	2.35065376	4.65916813	4.09E-06	3.42E-05	2.44660004	5.388795374	up-regulated in Low
TRA2B	0.29477801	3.4043936	10.5064591	1.90E-23	1.83E-21	41.6982634	22.72036236	up-regulated in Low

ETV5	-0.3862483	3.11859373	-5.3164844	1.60E-07	1.78E-06	5.57166528	6.795022309	up-regulated in High
DGKG	0.17552548	0.51243749	5.40171044	1.03E-07	1.18E-06	6.00390404	6.988628882	up-regulated in Low
CRYGS	-0.122525	0.79170628	-3.160661	0.00167043	0.00746966	-3.2381328	2.777172066	up-regulated in High
TBCCD1	0.21369369	2.11579835	5.10790288	4.66E-07	4.73E-06	4.53976255	6.332029663	up-regulated in Low
DNAJB11	0.42837794	3.93298502	9.60621409	3.76E-20	2.49E-18	34.1731334	19.4242706	up-regulated in Low
KNG1	0.18465603	0.13677142	3.89093777	0.0001135	0.00069749	-0.7238511	3.945009345	up-regulated in Low
RFC4	1.02142994	2.731753	17.1377986	4.84E-52	3.04E-49	107.251896	51.31538327	up-regulated in Low
ST6GAL1	-0.3693916	3.83511605	-4.5851968	5.75E-06	4.68E-05	2.11842019	5.240314573	up-regulated in High
RPL39L	0.89545884	3.52599001	8.10790133	4.07E-15	1.43E-13	22.7122417	14.39071142	up-regulated in Low
SST	0.39060564	0.64609625	2.28272199	0.02286949	0.06936826	-5.5944775	1.640743543	up-regulated in Low
BCL6	-0.2796812	3.7380271	-4.8655988	1.54E-06	1.41E-05	3.38781216	5.813685006	up-regulated in High
LPP	-0.1283145	2.76117385	-2.2596092	0.02427899	0.07293192	-5.6464949	1.614769304	up-regulated in High
TPRG1	-0.129524	0.50373175	-3.600709	0.00034931	0.00188548	-1.7823318	3.456787202	up-regulated in High
TP63	-0.2047509	1.02215682	-2.0287128	0.04302172	0.11584148	-6.1376256	1.366312229	up-regulated in High
P3H2	-0.6040795	2.15337151	-5.1378387	4.00E-07	4.11E-06	4.68558449	6.39752845	up-regulated in High
CLDN1	-0.4337389	4.05990485	-3.2556114	0.0012089	0.00562378	-2.9393463	2.917609798	up-regulated in High
CLDN16	-0.1835114	0.34996356	-5.2846356	1.89E-07	2.07E-06	5.41171149	6.723329132	up-regulated in High
IL1RAP	-0.1320457	1.71507178	-1.9954729	0.04653831	0.12333677	-6.2040487	1.332189381	up-regulated in High
FGF12	0.43523244	0.4287771	7.65129599	1.05E-13	3.04E-12	19.5114851	12.98063941	up-regulated in Low
MB21D2	0.16443326	1.49320964	2.67878012	0.00763467	0.02767181	-4.6228675	2.117210038	up-regulated in Low
ATP13A4	-1.4639956	2.21533675	-12.477427	2.91E-31	5.14E-29	59.581445	30.53662762	up-regulated in High
OPA1	0.43377207	3.09898806	9.72166761	1.46E-20	1.02E-18	35.1129894	19.83624917	up-regulated in Low
LRRC15	-0.2767404	1.96961834	-2.2168458	0.0270868	0.07974974	-5.7413699	1.567242264	up-regulated in High
ATP13A3	0.4450378	3.3548189	7.44931417	4.20E-13	1.11E-11	18.1422104	12.37662313	up-regulated in Low
TMEM44	0.17592477	2.02151184	3.34011677	0.00090059	0.00433743	-2.6663055	3.04547359	up-regulated in Low
LSG1	0.44874506	3.40978503	9.54993513	5.96E-20	3.89E-18	33.7177819	19.22463487	up-regulated in Low
XXYLT1	0.27616177	2.04284018	5.64124306	2.84E-08	3.59E-07	7.25131082	7.546396228	up-regulated in Low
ACAP2	0.15245834	2.47897174	3.18283036	0.00155004	0.00701624	-3.1691305	2.809655869	up-regulated in Low
APOD	-1.305822	3.67059475	-7.954891	1.23E-14	4.05E-13	21.6236337	13.91140077	up-regulated in High
TFRC	0.69851964	4.07224416	7.95532937	1.22E-14	4.03E-13	21.6267295	13.9127642	up-regulated in Low
PCYT1A	0.17991753	3.1721595	4.40183131	1.32E-05	9.94E-05	1.32576089	4.880887591	up-regulated in Low
TCTEX1D2	0.3326389	1.74333492	5.23183711	2.48E-07	2.66E-06	5.14843569	6.605267881	up-regulated in Low
TM4SF19	0.34458935	0.61387617	5.8188273	1.06E-08	1.45E-07	8.206788	7.972761804	up-regulated in Low
UBXN7	0.17862221	2.30690558	3.64006419	0.00030121	0.00165802	-1.6433499	3.521133439	up-regulated in Low
RNF168	0.21856752	2.28123492	4.42573085	1.18E-05	9.03E-05	1.4273831	4.927034831	up-regulated in Low
WDR53	0.43738533	2.56374493	9.80863505	7.10E-21	5.16E-19	35.825986	20.14872122	up-regulated in Low
FBXO45	0.6296245	2.28670991	13.9568684	1.42E-37	3.67E-35	74.0455903	36.84754217	up-regulated in Low
NRROS	-0.2363674	1.91613481	-3.8140561	0.00015396	0.00091642	-1.01178	3.812598447	up-regulated in High
PIGX	0.33451497	2.62380267	7.6967418	7.62E-14	2.26E-12	19.8235594	13.11823121	up-regulated in Low
PAK2	0.31184838	4.23017346	6.87545542	1.87E-11	3.96E-10	14.4136687	10.72894038	up-regulated in Low
SENP5	0.27135078	2.59218451	6.64890637	7.81E-11	1.51E-09	13.0094799	10.10708726	up-regulated in Low
NCBP2	0.2752973	4.13214568	6.78987307	3.22E-11	6.60E-10	13.8786243	10.49208902	up-regulated in Low
MFI2	0.58621816	1.60429996	6.03433161	3.13E-09	4.69E-08	9.4008736	8.504678882	up-regulated in Low
DLG1	0.10836727	2.60807482	2.61034615	0.00931888	0.03280376	-4.8015497	2.030636327	up-regulated in Low
BDH1	0.27641089	1.76627855	4.90338395	1.28E-06	1.19E-05	3.5641118	5.893127335	up-regulated in Low
FYTTD1	0.25217347	3.16785708	5.81799436	1.07E-08	1.46E-07	8.20224602	7.97073665	up-regulated in Low
IQCG	-0.1261588	1.42378123	-2.4871124	0.01320641	0.04408455	-5.1119608	1.879215131	up-regulated in High
RPL35A	0.2830637	7.12894723	4.85991517	1.58E-06	1.45E-05	3.36140067	5.801780004	up-regulated in Low
ZNF718	0.13646458	1.47154622	2.83612143	0.00475335	0.01849733	-4.195034	2.323000001	up-regulated in Low
PDE6B	-0.3551902	1.20010048	-6.0110336	3.58E-09	5.32E-08	9.26996955	8.446412721	up-regulated in High
ATP5I	0.23999022	5.98627783	4.18523837	3.37E-05	0.0002339	0.42807815	4.472285316	up-regulated in Low
MYL5	-0.1174732	1.51128881	-2.1911946	0.02890255	0.08401957	-5.797427	1.539063894	up-regulated in High
MFSD7	-0.4887197	2.68080665	-7.1483596	3.16E-12	7.49E-11	16.1565405	11.49971749	up-regulated in High
CPLX1	0.28746987	1.42763062	3.75242504	0.00019586	0.00113483	-1.2386805	3.708054489	up-regulated in Low
GAK	0.11235966	3.08880741	2.0989683	0.03632506	0.10108024	-5.993685	1.439793667	up-regulated in Low
TMEM175	-0.2276147	2.96462903	-4.3342053	1.77E-05	0.00013059	1.04097066	4.751449846	up-regulated in High
IDUA	-0.3838307	2.48973758	-5.5118804	5.72E-08	6.90E-07	6.57169036	7.242679914	up-regulated in High
SPON2	-0.2044097	2.81501466	-2.0856162	0.0375244	0.10379654	-6.0214116	1.425686209	up-regulated in High
MAEA	0.18711527	2.88506096	5.4854742	6.59E-08	7.86E-07	6.43467434	7.181400244	up-regulated in Low
SLBP	0.44707125	4.83909745	10.0667472	8.19E-22	6.61E-20	37.9671197	21.08676126	up-regulated in Low
TMEM129	-0.1702497	4.03243154	-3.1705266	0.00161583	0.00725886	-3.2074834	2.791604582	up-regulated in High
TACC3	1.0105592	3.03375502	16.9552975	3.46E-51	1.96E-48	105.289225	50.46080322	up-regulated in Low
FGFR3	-0.9695444	2.51362181	-7.4677371	3.71E-13	9.90E-12	18.2658952	12.43120473	up-regulated in High
LETM1	0.3551241	2.97323064	8.60173691	1.04E-16	4.53E-15	26.3325881	15.98301624	up-regulated in Low
WHSC1	0.57592742	2.40626355	11.5634267	1.52E-27	2.08E-25	51.068102	26.81803038	up-regulated in Low
NELFA	0.24387727	2.91489786	5.94792774	5.13E-09	7.40E-08	8.91759785	8.28951482	up-regulated in Low
C4orf48	0.24450529	2.80508555	2.6522962	0.00825096	0.02955334	-4.6925505	2.083495286	up-regulated in Low
NAT8L	0.31468516	0.57354661	4.73790918	2.82E-06	2.44E-05	2.80122322	5.549041171	up-regulated in Low
HAUS3	0.11567239	1.68054792	3.90825835	0.00010589	0.00065619	-0.6582375	3.975145742	up-regulated in Low
MXD4	-0.3300453	3.66854475	-6.8146326	2.75E-11	5.70E-10	14.0328453	10.56037075	up-regulated in High
ZFYVE28	-0.1459993	1.12566874	-3.584643	0.00037095	0.00198812	-1.8386563	3.430687005	up-regulated in High

CFAP99	-0.1070924	0.18206838	-3.6392508	0.00030214	0.00166141	-1.646237	3.519797594	up-regulated in High
RNF4	0.18697449	3.39883327	5.62780037	3.06E-08	3.85E-07	7.18004168	7.514564868	up-regulated in Low
TNIP2	0.15665786	4.02443042	3.77797253	0.00017733	0.00103808	-1.1450485	3.751217223	up-regulated in Low
ADD1	-0.1547374	4.06514918	-4.2638315	2.41E-05	0.00017228	0.74895081	4.618545516	up-regulated in High
NOP14	0.3319696	3.88650541	8.5615782	1.41E-16	6.06E-15	26.0322041	15.8509931	up-regulated in Low
RGS12	-0.1011309	1.79036738	-2.3119662	0.02118864	0.06514443	-5.5279183	1.673896833	up-regulated in High
DOK7	-0.1742304	0.73379018	-3.3176841	0.00097439	0.00465372	-2.7394392	3.011267225	up-regulated in High
TMEM128	-0.1001939	3.85335294	-2.1503184	0.03201314	0.0913162	-5.8854336	1.494671695	up-regulated in High
LYAR	0.6213217	3.07544635	14.8946419	9.91E-42	3.27E-39	83.5800299	41.0040215	up-regulated in Low
D4S234E	-0.1271053	0.38326503	-2.4876287	0.0131875	0.04403652	-5.1106908	1.879837506	up-regulated in High
STX18	0.14306292	2.79392552	4.57369309	6.06E-06	4.91E-05	2.06781645	5.217403109	up-regulated in Low
CYTL1	-0.1620569	0.97608452	-2.3580728	0.01875763	0.05890716	-5.4212968	1.72682199	up-regulated in High
EVC	-0.2592126	1.92141938	-4.7317121	2.91E-06	2.51E-05	2.77311678	5.536347793	up-regulated in High
WFS1	-0.2774736	3.7615866	-4.398338	1.34E-05	0.00010083	1.31094987	4.874160083	up-regulated in High
PPP2R2C	0.76279746	0.79491062	8.312978	9.04E-16	3.49E-14	24.1960863	15.04364111	up-regulated in Low
MAN2B2	-0.2250135	3.3492701	-4.6057523	5.23E-06	4.29E-05	2.20913274	5.281374638	up-regulated in High
MRFAP1	0.11117859	6.54413018	3.13395657	0.00182688	0.00807769	-3.3206356	2.738290763	up-regulated in Low
S100P	1.17082041	5.36207726	4.36828644	1.53E-05	0.00011405	1.18398473	4.816471159	up-regulated in Low
BLOC1S4	0.31376392	3.58893786	7.02093688	7.30E-12	1.65E-10	15.3358545	11.13691018	up-regulated in Low
KIAA0232	-0.1165776	2.88392155	-2.451048	0.01458849	0.04780973	-5.2000347	1.835989666	up-regulated in High
CCDC96	-0.2407825	1.34255738	-3.8557549	0.00013058	0.000791	-0.8562856	3.884140053	up-regulated in High
GRPEL1	0.3020943	3.25073358	8.11802749	3.78E-15	1.33E-13	22.7848468	14.42267006	up-regulated in Low
SORCS2	-0.9372872	1.80399975	-9.3926214	2.13E-19	1.30E-17	32.4547832	18.67078172	up-regulated in High
SH3TC1	-0.1440082	1.90298551	-3.0774843	0.00220313	0.00950734	-3.4928883	2.656959482	up-regulated in High
CPZ	-0.107605	0.38240588	-3.7998675	0.00016278	0.00096166	-1.0643257	3.788404145	up-regulated in High
SLC2A9	-0.1350792	0.72561734	-4.3700343	1.51E-05	0.00011323	1.19134731	4.81981739	up-regulated in High
WDR1	0.13077968	5.40830643	3.37700867	0.00079042	0.00386572	-2.5450087	3.102142499	up-regulated in Low
ZNF518B	0.17033185	1.58040466	2.94346882	0.00339783	0.0138482	-3.8895837	2.468797837	up-regulated in Low
CLNK	-0.1107907	0.30597648	-4.4486765	1.07E-05	8.22E-05	1.52542776	4.971538038	up-regulated in High
HS3ST1	-0.2771525	1.8159973	-3.3382297	0.00090659	0.00436202	-2.6724759	3.042588703	up-regulated in High
CPEB2	-0.1552662	2.08570357	-3.228985	0.0013247	0.00610412	-3.0239891	2.877882539	up-regulated in High
C1QTNF7	-0.5746669	0.62456666	-13.210933	2.38E-34	5.06E-32	66.6558609	33.62423181	up-regulated in High
CC2D2A	-0.1900831	1.58116272	-3.9630465	8.49E-05	0.00053791	-0.4488876	4.071211651	up-regulated in High
FBXL5	-0.1534711	4.4599591	-3.5469554	0.00042673	0.00225073	-1.9698437	3.369843148	up-regulated in High
FGFBP2	-0.1308092	0.45363448	-2.6023557	0.00953578	0.03347154	-4.8221196	2.020643721	up-regulated in High
PROM1	-0.3971583	1.64004945	-2.7058254	0.00704832	0.02588242	-4.5510134	2.151914119	up-regulated in High
TAPT1	-0.515867	2.46319102	-10.215367	2.32E-22	1.98E-20	39.216633	21.63397249	up-regulated in High
LDB2	-0.4762618	1.82729729	-8.6084304	9.88E-17	4.32E-15	26.3827559	16.00506422	up-regulated in High
DCAF16	0.24403265	3.11828256	5.15804706	3.61E-07	3.75E-06	4.78445572	6.441924483	up-regulated in Low
NCAPG	1.34037262	1.81875293	21.8382773	1.46E-74	2.12E-70	159.008114	73.83695128	up-regulated in Low
SLIT2	-0.508055	1.6130392	-7.7066243	7.11E-14	2.11E-12	19.8916147	13.1482331	up-regulated in High
PACRGL	0.14166604	1.89856659	3.90757416	0.00010618	0.00065786	-0.6608346	3.973953164	up-regulated in Low
PPARGC1A	0.35556978	0.69280871	4.96989781	9.24E-07	8.88E-06	3.87745847	6.034220529	up-regulated in Low
DHX15	0.26018314	4.11235544	6.81490197	2.75E-11	5.69E-10	14.034526	10.56111483	up-regulated in Low
SOD3	-0.7966358	3.98735867	-8.296502	1.02E-15	3.90E-14	24.0758349	14.99074384	up-regulated in High
ZCCHC4	0.11986525	1.79676339	3.77265417	0.00018105	0.00105697	-1.1645898	3.742211593	up-regulated in Low
SLC34A2	-2.1172783	7.90085828	-13.143063	4.62E-34	9.57E-32	65.9929271	33.33497842	up-regulated in High
SEL1L3	-0.3635732	4.79638795	-5.7703531	1.40E-08	1.86E-07	7.94340616	7.855301439	up-regulated in High
SMIM20	0.18163577	4.00846427	3.70632023	0.000234	0.00132867	-1.4061358	3.630780818	up-regulated in Low
ARAP2	-0.1538043	1.65857639	-3.0021369	0.00281624	0.01175606	-3.7180174	2.550329985	up-regulated in High
DTHD1	-0.2896007	0.43734796	-5.407786	9.94E-08	1.15E-06	6.03495057	7.002528126	up-regulated in High
C4orf19	-0.2523384	1.54063961	-2.5785658	0.01020855	0.03541571	-4.8829991	1.991036038	up-regulated in High
PGM2	0.3844977	2.95474949	6.37030305	4.31E-10	7.43E-09	11.3368009	9.365181058	up-regulated in Low
TBC1D1	0.21412498	2.4012437	3.53886965	0.00043968	0.00231153	-1.9978177	3.356859231	up-regulated in Low
TLR10	-0.3961602	0.86633462	-6.2244574	1.03E-09	1.67E-08	10.4853947	8.987001902	up-regulated in High
TLR1	-0.1490095	1.7042452	-2.5693575	0.01048013	0.0361816	-4.9064177	1.979633381	up-regulated in High
TLR6	0.17148431	1.06155679	3.65060819	0.00028942	0.00160037	-1.6058708	3.538472147	up-regulated in Low
FAM114A1	0.16947391	4.18437101	3.05914576	0.00233987	0.01001303	-3.5481765	2.630808799	up-regulated in Low
KLHL5	0.3095615	3.21193468	4.31521513	1.93E-05	0.00014076	0.96173295	4.715405645	up-regulated in Low
WDR19	-0.1322625	1.70614919	-3.185044	0.00153847	0.00697143	-3.162215	2.812909718	up-regulated in High
RFC1	0.18398005	3.25611259	4.22945162	2.79E-05	0.00019689	0.60790442	4.554283555	up-regulated in Low
UGDH	0.72814064	4.89417498	6.8200652	2.66E-11	5.52E-10	14.0667462	10.57537917	up-regulated in Low
SMIM14	-0.364783	4.57594972	-5.841588	9.37E-09	1.29E-07	8.33111992	8.028192528	up-regulated in High
UBE2K	0.35852131	4.29447835	9.28994796	4.87E-19	2.82E-17	31.6383613	18.3126582	up-regulated in Low
N4BP2	0.24121127	1.58603306	5.42738375	8.96E-08	1.04E-06	6.13530858	7.047451227	up-regulated in Low
RHOH	-0.1347352	1.35806016	-2.2163158	0.0271233	0.07983706	-5.7425347	1.566657541	up-regulated in High
CHRNA9	0.4621612	0.53025219	4.4983876	8.54E-06	6.70E-05	1.73944259	5.06861637	up-regulated in Low
NSUN7	-0.1605596	1.7438552	-3.0289676	0.00258195	0.01090825	-3.6384668	2.588052887	up-regulated in High
APBB2	-0.1423582	1.92759315	-2.8629797	0.00437454	0.01722061	-4.1196397	2.35906798	up-regulated in High
UCHL1	1.4085638	3.41460205	7.34178255	8.71E-13	2.21E-11	17.4251618	12.06010375	up-regulated in Low
LIMCH1	-0.5020751	3.64547908	-5.9240132	5.88E-09	8.39E-08	8.78490515	8.230410408	up-regulated in High

TMEM33	0.21785577	3.60968798	5.02422083	7.07E-07	6.92E-06	4.13620742	6.150633227	up-regulated in Low
SHISA3	-1.2464444	1.68585	-7.2475593	1.64E-12	4.02E-11	16.8037518	11.78567393	up-regulated in High
ATP8A1	-0.7987334	2.27726814	-9.6738081	2.16E-20	1.47E-18	34.7224567	19.6650741	up-regulated in High
GUF1	0.21583305	2.92102745	5.23144181	2.49E-07	2.66E-06	5.14647349	6.604387692	up-regulated in Low
GABRA2	0.11577889	0.06487071	4.34773442	1.67E-05	0.00012378	1.09761842	4.777209911	up-regulated in Low
COX7B2	0.38993079	0.37079224	4.309465	1.97E-05	0.00014401	0.93780384	4.704517928	up-regulated in Low
COMMD8	0.2588695	3.37374533	4.58809689	5.67E-06	4.62E-05	2.13119601	5.246098275	up-regulated in Low
CORIN	-0.1105808	0.40619119	-3.4206084	0.00067643	0.00337272	-2.4000173	3.169779393	up-regulated in High
NFXL1	0.38000915	1.99541248	9.5482594	6.04E-20	3.93E-18	33.7042518	19.21870262	up-regulated in Low
CNGA1	-0.1632799	0.52926369	-2.9776546	0.00304686	0.01258646	-3.7900095	2.51614752	up-regulated in High
TXK	-0.1136796	0.55673621	-3.3783981	0.00078653	0.00385033	-2.5404156	3.104286788	up-regulated in High
SLC10A4	0.17881481	0.54124128	2.88285383	0.00411213	0.01633076	-4.0634083	2.385933299	up-regulated in Low
OCIAD1	0.11769522	4.82896062	2.96834652	0.00313898	0.01291857	-3.8172314	2.503211203	up-regulated in Low
SPATA18	-0.8085596	1.41148307	-9.1228759	1.84E-18	1.00E-16	30.3234049	17.73567057	up-regulated in High
ERVMER34-1	0.43887663	0.94261894	6.11148702	2.00E-09	3.11E-08	9.83750034	8.698946375	up-regulated in Low
RASL11B	-0.2825307	1.30328367	-3.7064591	0.00023388	0.00132822	-1.4056345	3.631012331	up-regulated in High
FIP1L1	0.27859149	2.60526618	7.77745856	4.32E-14	1.32E-12	20.381414	13.36412411	up-regulated in Low
PDGFRA	-0.3848166	2.23666979	-4.8838721	1.41E-06	1.30E-05	3.47291795	5.85203987	up-regulated in High
KDR	-0.5444855	3.19192345	-5.3108942	1.65E-07	1.83E-06	5.5435277	6.782412603	up-regulated in High
SRD5A3	-0.3373773	4.0124065	-3.6472125	0.00029317	0.00161895	-1.6179523	3.532883587	up-regulated in High
NMU	0.49886537	1.94621224	3.70378513	0.00023629	0.00133975	-1.4152866	3.626555074	up-regulated in Low
CEP135	0.12781019	1.14912326	4.13572836	4.16E-05	0.00028288	0.22879913	4.381324748	up-regulated in Low
KIAA1211	0.13181254	0.81836395	2.34778868	0.01927758	0.06025805	-5.4452572	1.714947485	up-regulated in Low
PPAT	0.60417199	2.22502017	11.9885364	2.96E-29	4.54E-27	54.9835216	28.52872622	up-regulated in Low
PAICS	0.81364692	4.13981956	15.8998615	2.70E-46	1.14E-43	94.0565519	45.56878347	up-regulated in Low
SRP72	0.34899327	4.7338292	8.21174954	1.91E-15	7.06E-14	23.46012	14.71985285	up-regulated in Low
ARL9	0.22880865	0.79577794	3.69078194	0.00024836	0.00139944	-1.4621306	3.604918111	up-regulated in Low
HOPX	-1.6704167	4.64118842	-11.543308	1.83E-27	2.48E-25	50.8847897	26.73791808	up-regulated in High
SPINK2	-0.2919876	0.96301889	-3.3940246	0.00074395	0.0036721	-2.4886331	3.128454036	up-regulated in High
NOA1	0.15515554	3.29448358	4.23533993	2.72E-05	0.00019237	0.63198637	4.565258762	up-regulated in Low
POLR2B	0.21509272	4.17877599	5.26254668	2.12E-07	2.29E-06	5.30127921	6.673816664	up-regulated in Low
IGFBP7	-0.5496829	6.92539655	-7.1785638	2.59E-12	6.20E-11	16.3528364	11.58646128	up-regulated in High
CENPC	-0.1377322	1.65777072	-4.4926148	8.76E-06	6.86E-05	1.71447715	5.057296388	up-regulated in High
STAP1	-0.3816308	0.93568478	-6.8275931	2.53E-11	5.28E-10	14.1137592	10.59619174	up-regulated in High
UBA6	0.31676738	2.45069702	7.62110941	1.29E-13	3.68E-12	19.3050008	12.88958757	up-regulated in Low
TMPRSS11E	0.44676349	1.74462643	2.76884283	0.00583641	0.02207633	-4.3808709	2.233854476	up-regulated in Low
UGT2B15	-0.2128347	0.48818745	-2.0872114	0.03737936	0.10345414	-6.0181082	1.427368155	up-regulated in High
UGT2B4	0.290583	0.4226193	3.02809574	0.00258927	0.01093366	-3.6410625	2.586822777	up-regulated in Low
SULT1B1	-0.119464	0.31522175	-2.5808963	0.01014082	0.03522471	-4.8770595	1.99392689	up-regulated in High
SULT1E1	-0.1271493	0.307071	-2.7585111	0.0060212	0.02267104	-4.4090262	2.220316979	up-regulated in High
ODAM	-0.132527	0.22598071	-3.8458336	0.00013581	0.0008191	-0.8934265	3.867059178	up-regulated in High
FDCSP	-1.0453747	2.1542529	-6.9813134	9.44E-12	2.09E-10	15.0831153	11.02513138	up-regulated in High
IGJ	-1.0938123	7.58960221	-6.6150684	9.65E-11	1.84E-09	12.803115	10.01562641	up-regulated in High
RUFY3	-0.1877802	2.3210341	-4.012695	6.93E-05	0.00044931	-0.2568146	4.159234814	up-regulated in High
GRSF1	0.26301414	3.88230703	7.53872926	2.28E-13	6.27E-12	18.7447862	12.64249612	up-regulated in Low
DCK	0.35087319	3.32541013	6.22597218	1.02E-09	1.65E-08	10.4941509	8.990893263	up-regulated in Low
SLC4A4	-0.6753758	2.02996335	-6.8363408	2.40E-11	5.01E-10	14.1684433	10.62039918	up-regulated in High
NPFFR2	0.19905982	0.26710095	3.47875882	0.00054814	0.00280174	-2.2038777	3.261108162	up-regulated in Low
ADAMTS3	0.12519622	0.51734109	2.83271205	0.00480351	0.01866019	-4.2045553	2.318441148	up-regulated in Low
ANKRD17	0.10303251	3.2518279	2.2173402	0.0270528	0.07966971	-5.7402832	1.567787829	up-regulated in Low
AFP	0.135197	0.09633881	2.81123151	0.00513074	0.01977089	-4.2642886	2.289819992	up-regulated in Low
CXCL8	0.52625469	3.83410639	3.56244437	0.00040292	0.00214118	-1.9160872	3.394784112	up-regulated in Low
CXCL5	0.52790265	1.95152329	3.41277537	0.00069571	0.00345762	-2.4261969	3.157574905	up-regulated in Low
CXCL2	-0.5068803	4.01719843	-3.86145	0.00012766	0.00077527	-0.8349252	3.893961519	up-regulated in High
BTC	-0.4351882	2.04280491	-5.4583896	7.61E-08	8.96E-07	6.29474407	7.11879972	up-regulated in High
PARM1	-1.1260801	4.78793031	-9.3550588	2.89E-19	1.72E-17	32.1553705	18.53945408	up-regulated in High
C4orf26	0.12133258	0.14088021	3.95431157	8.79E-05	0.00055538	-0.4824477	4.055820609	up-regulated in Low
CDKL2	-0.9124504	1.66065191	-10.540481	1.42E-23	1.37E-21	41.9912192	22.84857602	up-regulated in High
NAAA	-0.1542652	2.47376442	-2.7375448	0.00641254	0.02392849	-4.4658482	2.192969841	up-regulated in High
SDAD1	0.24803742	3.2660842	6.03250585	3.16E-09	4.73E-08	9.3905994	8.500106173	up-regulated in Low
CXCL10	0.46892077	4.24309098	3.05193863	0.00239571	0.0102265	-3.569818	2.620566417	up-regulated in Low
CXCL11	0.28760345	1.85222983	2.39361368	0.01705427	0.05442434	-5.3377036	1.768166846	up-regulated in Low
NUP54	0.2586329	3.25638559	6.68622639	6.19E-11	1.21E-09	13.2381009	10.20839002	up-regulated in Low
FAM47E	-0.1000527	0.34649335	-4.9846875	8.59E-07	8.29E-06	3.9476523	6.065809593	up-regulated in High
AM47E-STBI	-0.1055032	1.09253284	-2.2509765	0.0248245	0.07428393	-5.6657905	1.605119534	up-regulated in High
AM47E-STBI	-0.1055032	1.09253284	-2.2509765	0.0248245	0.07428393	-5.6657905	1.605119534	up-regulated in High
SHROOM3	-0.3727209	2.83069611	-5.2582115	2.17E-07	2.34E-06	5.27965449	6.664119697	up-regulated in High
SOWAHB	-0.1978626	2.12089905	-2.5829334	0.01008195	0.03505983	-4.871863	1.996455611	up-regulated in High
CNOT6L	-0.1564411	2.48788296	-3.9699398	8.25E-05	0.00052515	-0.4223536	4.083378045	up-regulated in High
MRPL1	0.3165857	3.44103715	7.47875687	3.44E-13	9.22E-12	18.3399948	12.46390243	up-regulated in Low
PAQR3	0.13914918	1.27693284	3.07418305	0.00222719	0.00960265	-3.5028646	2.652242498	up-regulated in Low

ANTXR2	-0.2400121	2.796868	-3.6399695	0.00030132	0.00165846	-1.643686	3.520977943	up-regulated in High
FGF5	0.15765494	0.08300451	4.67638947	3.77E-06	3.18E-05	2.52369452	5.423649703	up-regulated in Low
BMP3	-0.9785864	1.95912235	-7.8477806	2.63E-14	8.25E-13	20.8711362	13.5799217	up-regulated in High
RASGEF1B	-0.3063467	1.59248769	-6.5933257	1.10E-10	2.09E-09	12.6709801	9.957054178	up-regulated in High
HNRNPD	0.31114489	4.89382924	7.91104618	1.68E-14	5.41E-13	21.3146477	13.77530682	up-regulated in Low
ENOPH1	0.41542455	4.60477249	10.8320589	1.10E-24	1.18E-22	44.5262317	23.95775529	up-regulated in Low
SCD5	-0.3967634	2.06603497	-4.2245347	2.85E-05	0.00020062	0.58781922	4.545128801	up-regulated in High
LIN54	0.27189873	1.80296771	9.38287921	2.31E-19	1.39E-17	32.3770473	18.63668646	up-regulated in Low
PLAC8	-0.5822565	2.00240497	-5.2051535	2.84E-07	3.01E-06	5.01627989	6.545977127	up-regulated in High
COQ2	0.2527764	2.56270856	6.74972844	4.15E-11	8.39E-10	13.6295632	10.38179598	up-regulated in Low
HELQ	-0.1150415	1.9198898	-4.0713505	5.44E-05	0.0003613	-0.0270116	4.264411632	up-regulated in High
MRPS18C	0.16944033	1.72163295	6.55144846	1.43E-10	2.66E-09	12.4175117	9.844675397	up-regulated in Low
AGPAT9	0.32772041	1.73379946	4.0787013	5.28E-05	0.00035138	0.00200787	4.277683083	up-regulated in Low
CDS1	-0.3211707	3.49543816	-5.5223502	5.41E-08	6.56E-07	6.62617664	7.267043967	up-regulated in High
WDFY3	-0.1690246	2.04031152	-4.1885776	3.32E-05	0.00023085	0.44159816	4.478452952	up-regulated in High
ARHGAP24	-0.4162581	1.44650905	-9.4578259	1.26E-19	7.90E-18	32.9765142	18.89959567	up-regulated in High
MAPK10	-0.4169699	0.66190114	-9.02037	4.12E-18	2.14E-16	29.525038	17.38524133	up-regulated in High
PTPN13	-1.1337683	2.62603578	-10.371488	6.11E-23	5.61E-21	40.5420223	22.21425777	up-regulated in High
AFF1	-0.1533786	3.11614138	-2.9158727	0.00370767	0.01495025	-3.9691539	2.430899135	up-regulated in High
HSD17B13	-0.409763	0.64204203	-4.9329908	1.11E-06	1.04E-05	3.7031169	5.955734442	up-regulated in High
HSD17B11	-0.4939522	5.26824192	-6.7384691	4.46E-11	8.95E-10	13.5599294	10.35095509	up-regulated in High
SPARCL1	-0.9570399	5.3139958	-11.172328	5.31E-26	6.42E-24	47.5381286	25.27498181	up-regulated in High
PKD2	-0.3700296	2.72194268	-6.6670937	6.98E-11	1.36E-09	13.1207616	10.15639941	up-regulated in High
PPM1K	-0.3202705	1.65936163	-6.4222588	3.15E-10	5.55E-09	11.6441483	9.501605161	up-regulated in High
HERC6	-0.2885537	2.36993388	-4.1151119	4.53E-05	0.00030636	0.14647015	4.343716583	up-regulated in High
PYURF	-0.1007219	1.44791901	-3.0350203	0.00253163	0.01072826	-3.6204267	2.596600541	up-regulated in High
HERC3	-0.2049715	1.93624327	-4.5165752	7.86E-06	6.22E-05	1.81829094	5.104360639	up-regulated in High
NAP1L5	-0.1934283	1.33701892	-4.4717501	9.63E-06	7.49E-05	1.62449076	5.01648445	up-regulated in High
FAM13A	-0.3599713	2.40391205	-5.3865824	1.11E-07	1.27E-06	5.92673301	6.954076137	up-regulated in High
SNCA	-0.1055856	0.70920475	-2.4648605	0.01404473	0.04634768	-5.166451	1.852486685	up-regulated in High
MMRN1	-0.5146046	1.20274825	-7.6265007	1.24E-13	3.56E-12	19.3418313	12.90582928	up-regulated in High
SMARCAD1	0.10793869	2.756805	2.80391301	0.00524676	0.02015015	-4.2845393	2.280108675	up-regulated in Low
HPGDS	-0.9319098	1.38805157	-13.392002	3.99E-35	8.89E-33	68.4324311	34.39931398	up-regulated in High
BMPR1B	0.18867118	0.87179576	2.71996118	0.00675829	0.02499342	-4.5131785	2.17016345	up-regulated in Low
UNC5C	-0.2284105	0.71174879	-6.1570139	1.53E-09	2.42E-08	10.0973766	8.814517563	up-regulated in High
EIF4E	0.20099215	1.74638558	6.85440181	2.14E-11	4.49E-10	14.2815314	10.67045699	up-regulated in Low
METAP1	0.22016584	3.89837594	5.72522081	1.79E-08	2.35E-07	7.6999141	7.746665212	up-regulated in Low
ADH4	0.12381756	0.1406669	2.77482414	0.00573178	0.02173833	-4.3645244	2.241710214	up-regulated in Low
ADH1B	-1.6916993	2.17718099	-13.485374	1.58E-35	3.64E-33	69.3529802	34.80088868	up-regulated in High
TRMT10A	0.21564895	1.48856867	5.99136413	4.01E-09	5.90E-08	9.15979366	8.397364251	up-regulated in Low
DAPP1	-0.3446267	2.19815342	-4.6101693	5.13E-06	4.21E-05	2.22867413	5.290217981	up-regulated in High
DNAJB14	-0.1061303	1.97038833	-3.123479	0.00189187	0.00832784	-3.352822	2.723109483	up-regulated in High
H2AFZ	0.87205956	6.02998264	16.9583609	3.35E-51	1.92E-48	105.322124	50.47512873	up-regulated in Low
EMCN	-0.6544508	1.58082377	-10.289808	1.23E-22	1.08E-20	39.8469723	21.90996864	up-regulated in High
PPP3CA	-0.218344	3.86163172	-4.4021113	1.31E-05	9.93E-05	1.32694826	4.881426901	up-regulated in High
BANK1	-0.5704667	1.54493371	-9.6913489	1.87E-20	1.28E-18	34.8654372	19.7277461	up-regulated in High
SLC39A8	-0.6794726	3.97505033	-7.6979636	7.55E-14	2.24E-12	19.8319696	13.12193888	up-regulated in High
NFKB1	-0.1650401	3.59276568	-3.4924523	0.00052143	0.00268285	-2.1572318	3.282800623	up-regulated in High
MANBA	-0.1874647	2.80471972	-3.7068222	0.00023355	0.00132649	-1.4043231	3.631617851	up-regulated in High
CISD2	0.30204771	3.98850481	7.8076355	3.50E-14	1.08E-12	20.5911444	13.45654962	up-regulated in Low
BDH2	-0.3705573	2.98905883	-6.635994	8.47E-11	1.63E-09	12.9306286	10.07214262	up-regulated in High
CENPE	0.82879815	1.12092663	17.3679087	4.02E-53	2.76E-50	109.734142	52.39611953	up-regulated in Low
TET2	-0.1546286	1.44400475	-4.5837778	5.79E-06	4.71E-05	2.11217184	5.2374858	up-regulated in High
ARHGEF38	-0.487598	1.69886998	-7.8641585	2.34E-14	7.38E-13	20.9856846	13.63038946	up-regulated in High
GSTCD	0.26177964	1.60803426	6.82269583	2.61E-11	5.43E-10	14.08317	10.58265005	up-regulated in Low
NPNT	-0.5719116	3.27479922	-4.8806879	1.43E-06	1.32E-05	3.45806696	5.845347658	up-regulated in High
TBCK	-0.1498131	1.63256909	-4.8374793	1.76E-06	1.59E-05	3.25741599	5.754899243	up-regulated in High
AIMP1	0.23166636	3.99113035	7.66762034	9.33E-14	2.73E-12	19.6234162	13.02999211	up-regulated in Low
DKK2	-0.2889404	0.86635533	-6.0671918	2.59E-09	3.94E-08	9.58624726	8.587171257	up-regulated in High
PAPSS1	-0.13084	4.21237847	-3.2036976	0.0014441	0.00659034	-3.103758	2.840402156	up-regulated in High
SGMS2	-0.6229472	3.17940187	-8.77986	2.67E-17	1.24E-15	27.6774073	16.57389859	up-regulated in High
CYP2U1	-0.3363448	1.35467014	-8.1774063	2.45E-15	8.94E-14	23.2119889	14.6106631	up-regulated in High
HADH	-0.1765435	4.095499	-3.3901775	0.00075423	0.00371403	-2.5014026	3.122495714	up-regulated in High
LEF1	-0.140805	1.5163428	-2.4838866	0.01332513	0.0443996	-5.1198898	1.875328693	up-regulated in High
RPL34	-0.1465233	6.69919501	-2.5937655	0.00977399	0.03419872	-4.8441652	2.009928004	up-regulated in High
OSTC	0.25375073	5.94293985	4.91520953	1.21E-06	1.13E-05	3.61954232	5.918096054	up-regulated in Low
ETNPPL	0.17223013	0.32034169	2.54628296	0.01118918	0.03828961	-4.9647419	1.95120159	up-regulated in Low
CASP6	0.16563312	3.81937659	3.30820155	0.00100724	0.00479377	-2.7702119	2.996865119	up-regulated in Low
CFI	-0.7220687	4.13419082	-7.4856682	3.28E-13	8.82E-12	18.3865125	12.48442839	up-regulated in High
GAR1	0.32547194	3.36145548	8.16350659	2.71E-15	9.83E-14	23.1117882	14.56656638	up-regulated in Low
ELOVL6	0.50056412	1.57798332	6.06111727	2.68E-09	4.07E-08	9.55191348	8.571894143	up-regulated in Low

PITX2	0.33068966	0.80572881	3.57527972	0.00038413	0.00205049	-1.871372	3.415520656	up-regulated in Low
AP1AR	0.31314376	2.85390815	8.15688104	2.85E-15	1.03E-13	23.0640715	14.54556625	up-regulated in Low
TIFA	0.11386973	2.31792093	2.57329512	0.01036322	0.03586067	-4.8964134	1.984505431	up-regulated in Low
ALPK1	-0.1751301	1.66899189	-4.5316823	7.34E-06	5.85E-05	1.8840071	5.134142931	up-regulated in High
ZGRF1	0.22505014	0.61558645	8.54123435	1.64E-16	6.98E-15	25.8804317	15.78428097	up-regulated in Low
CAMK2D	-0.493191	3.29020948	-8.0129413	8.08E-15	2.72E-13	22.0347585	14.09244843	up-regulated in High
UGT8	0.29454663	1.66936352	2.79459841	0.00539786	0.02065844	-4.3102395	2.267778063	up-regulated in Low
PRSS12	-0.6325392	1.41117779	-6.1341933	1.75E-09	2.74E-08	9.96690519	8.756499844	up-regulated in High
SYNPO2	-0.3635204	1.03572383	-7.1709513	2.73E-12	6.51E-11	16.3032997	11.56457208	up-regulated in High
USP53	-0.2667129	2.10894886	-5.9398684	5.38E-09	7.71E-08	8.87282792	8.269574579	up-regulated in High
PDE5A	-0.2793484	1.4991435	-5.5421169	4.86E-08	5.93E-07	6.72929486	7.313147124	up-regulated in High
MAD2L1	1.17214045	1.9582806	18.8772485	2.76E-60	3.35E-57	126.191904	59.55976671	up-regulated in Low
PRDM5	-0.1143371	0.61544317	-4.3168028	1.91E-05	0.00013986	0.96834525	4.718414014	up-regulated in High
NDNF	-1.5854687	3.72233951	-12.293316	1.68E-30	2.82E-28	57.8383982	29.77556029	up-regulated in High
EXOSC9	0.27646462	2.58394725	8.3239317	8.34E-16	3.23E-14	24.2761323	15.07885093	up-regulated in Low
CCNA2	1.46998091	2.77528337	20.2168343	1.01E-66	3.93E-63	140.983955	65.99618228	up-regulated in Low
KIAA1109	-0.2680854	2.0262918	-5.3362924	1.45E-07	1.62E-06	5.67157871	6.839791512	up-regulated in High
BBS12	-0.1089292	1.30259855	-3.1627231	0.00165888	0.00742483	-3.2317341	2.780185675	up-regulated in High
SPATA5	0.23733778	1.12936257	7.13678844	3.42E-12	8.04E-11	16.0815183	11.46656153	up-regulated in Low
SPRY1	-0.5040495	3.57978408	-7.6483219	1.07E-13	3.10E-12	19.491113	12.97165657	up-regulated in High
ANKRD50	-0.2919125	2.66491127	-4.8900639	1.36E-06	1.27E-05	3.50182104	5.865063418	up-regulated in High
FAT4	-0.4908429	0.89639578	-10.61938	7.13E-24	7.13E-22	42.672898	23.14688952	up-regulated in High
HSPA4L	0.31022246	0.90898108	6.43537959	2.91E-10	5.15E-09	11.7220996	9.53619823	up-regulated in Low
PLK4	0.85689425	1.49407286	17.1176799	6.01E-52	3.69E-49	107.035268	51.22106213	up-regulated in Low
LARP1B	0.21006353	1.85417214	6.14897679	1.61E-09	2.53E-08	10.0513788	8.79406452	up-regulated in Low
SCLT1	0.1969161	1.04467982	7.2678982	1.43E-12	3.54E-11	16.9373404	11.84468019	up-regulated in Low
PCDH18	-0.1147011	1.10862751	-2.4565642	0.01436914	0.04724201	-5.1866447	1.842569296	up-regulated in High
SLC7A11	0.85823027	1.82773881	7.25009266	1.61E-12	3.96E-11	16.8203745	11.7930165	up-regulated in Low
CCRN4L	0.41861172	1.6030989	9.77774479	9.17E-21	6.54E-19	35.5722424	20.03752384	up-regulated in Low
NDUFC1	0.17339096	4.22539291	4.05799237	5.75E-05	0.00037964	-0.079621	4.240346063	up-regulated in Low
NAA15	0.41953803	2.68542116	10.8734428	7.64E-25	8.39E-23	44.8895051	24.11666354	up-regulated in Low
RAB33B	-0.1453523	1.98901769	-3.7400001	0.00020552	0.00118397	-1.2840012	3.687151073	up-regulated in High
MGST2	-0.1376611	3.54248043	-2.7923415	0.00543506	0.02077493	-4.3164542	2.264795308	up-regulated in High
MAML3	-0.1542172	1.72875713	-3.037611	0.00251036	0.01064976	-3.6126948	2.600263295	up-regulated in High
SCOC	0.17613527	3.81641425	3.13499665	0.00182054	0.00805271	-3.3174348	2.739800042	up-regulated in Low
CLGN	0.8977256	1.12358263	9.75030939	1.15E-20	8.12E-19	35.3473321	19.9389563	up-regulated in Low
TBC1D9	-0.3162728	3.14666689	-5.7851585	1.29E-08	1.73E-07	8.0236464	7.89109159	up-regulated in High
GAB1	-0.3083492	1.6637607	-7.0434662	6.30E-12	1.43E-10	15.4800806	11.20068653	up-regulated in High
GYPE	-0.1231732	0.25706438	-5.3044946	1.71E-07	1.88E-06	5.51134841	6.767990648	up-regulated in High
HHIP	-0.6118689	0.81003919	-7.2463644	1.65E-12	4.05E-11	16.7959126	11.78221115	up-regulated in High
ANAPC10	0.10842958	1.95511067	3.78706578	0.00017114	0.00100579	-1.1115768	3.766639529	up-regulated in Low
ABCE1	0.4099517	3.64085251	10.0736022	7.73E-22	6.26E-20	38.0244872	21.111888	up-regulated in Low
LSM6	0.16522202	2.60151934	4.50061523	8.45E-06	6.64E-05	1.74908425	5.072987837	up-regulated in Low
TTC29	-0.2645989	0.33535916	-5.0667571	5.72E-07	5.71E-06	4.34058697	6.242525633	up-regulated in High
EDNRA	-0.4512854	2.68472637	-5.6661709	2.48E-08	3.16E-07	7.38386647	7.605589384	up-regulated in High
NR3C2	-0.8778775	1.66404316	-14.194894	1.28E-38	3.46E-36	76.4413351	37.89217833	up-regulated in High
DCLK2	-0.1330667	0.96437546	-2.909384	0.00378417	0.01521169	-3.9877582	2.422029973	up-regulated in High
SH3D19	-0.3096377	3.26148793	-6.6514547	7.69E-11	1.49E-09	13.0250567	10.11399009	up-regulated in High
GATB	0.16762082	1.31842846	6.05639326	2.75E-09	4.17E-08	9.52523367	8.560022226	up-regulated in Low
TMEM154	-0.1701502	1.20574114	-3.8369858	0.00014065	0.00084531	-0.9264727	3.851857584	up-regulated in High
FHDC1	-0.4002502	2.12531843	-6.4752859	2.28E-10	4.11E-09	11.9600106	9.641759323	up-regulated in High
TRIM2	-0.6499394	3.51111775	-7.1521691	3.09E-12	7.32E-11	16.1812615	11.51064248	up-regulated in High
MND1	0.83684108	1.55707194	15.7161579	1.87E-45	7.60E-43	92.1243635	44.7270672	up-regulated in Low
TLR2	-1.1373552	3.31222945	-13.028961	1.41E-33	2.87E-31	64.8821402	32.8502804	up-regulated in High
RNF175	-0.311448	0.53155665	-9.4698657	1.14E-19	7.21E-18	33.0731243	18.94196202	up-regulated in High
PLRG1	0.17206356	3.47151464	4.58983974	5.63E-06	4.59E-05	2.1388773	5.249575512	up-regulated in Low
FGB	1.80253785	2.20359047	6.54091792	1.53E-10	2.83E-09	12.3539876	9.816506391	up-regulated in Low
FGA	1.68175374	2.82126586	6.22845091	1.01E-09	1.63E-08	10.5084835	8.997262772	up-regulated in Low
FGG	1.04797575	3.97590762	3.66153994	0.00027766	0.00154278	-1.566905	3.556492689	up-regulated in Low
MAP9	-0.1724965	1.53575715	-3.5337554	0.00044806	0.0023503	-2.01548	3.348659592	up-regulated in High
GUCY1A3	-0.5599246	2.43346118	-7.9828704	1.00E-14	3.34E-13	21.8215019	13.99854103	up-regulated in High
GUCY1B3	-0.3703445	2.4722003	-5.9360294	5.49E-09	7.87E-08	8.85152066	8.260083987	up-regulated in High
CTSO	-0.7067056	4.11825937	-11.155295	6.19E-26	7.46E-24	47.386028	25.20847683	up-regulated in High
PDGFC	-0.3373456	2.81402689	-4.7439408	2.75E-06	2.38E-05	2.82861158	5.561409074	up-regulated in High
RXFP1	-0.1099441	0.26302741	-4.7221973	3.04E-06	2.62E-05	2.73002845	5.516885937	up-regulated in High
C4orf46	0.46880387	1.98730569	9.81805865	6.57E-21	4.82E-19	35.9035024	20.18268966	up-regulated in Low
PPID	0.26319186	3.51060679	7.4635632	3.81E-13	1.02E-11	18.2378519	12.41882974	up-regulated in Low
FNIP2	-0.7223831	2.76305369	-9.8710915	4.22E-21	3.19E-19	36.3406724	20.37425002	up-regulated in High
RAPGEF2	-0.2655762	2.19367495	-5.9929002	3.97E-09	5.85E-08	9.16838655	8.401189943	up-regulated in High
FSTL5	0.17025019	0.15229193	3.70489551	0.00023529	0.0013349	-1.4112793	3.628405666	up-regulated in Low
NPY1R	0.11447854	0.36613923	2.04074304	0.04180577	0.11323134	-6.1133196	1.378763808	up-regulated in Low

TMA16	0.29573919	2.57440876	8.56207423	1.40E-16	6.05E-15	26.035908	15.85262111	up-regulated in Low
1-Mar	-0.226099	1.35759709	-4.6458374	4.35E-06	3.63E-05	2.38710126	5.361889361	up-regulated in High
TRIM61	-0.1211897	0.31649089	-4.2890289	2.16E-05	0.00015612	0.85299864	4.665921515	up-regulated in High
TMEM192	-0.1091744	2.02912344	-2.985885	0.00296747	0.01230151	-3.7658708	2.527613489	up-regulated in High
KLHL2	-0.1223014	3.08763648	-2.4706939	0.01382052	0.04572756	-5.1522125	1.859475489	up-regulated in High
CPE	0.60570311	4.91378035	4.14424597	4.01E-05	0.00027355	0.26292519	4.396908576	up-regulated in Low
CBR4	-0.12548	2.18935055	-3.2518946	0.00122448	0.00568991	-2.9512016	2.912048111	up-regulated in High
SH3RF1	-0.4216621	3.21836396	-6.9968467	8.53E-12	1.90E-10	15.182055	11.06889213	up-regulated in High
NEK1	-0.1291392	1.40081611	-3.5672319	0.00039581	0.00210745	-1.8994265	3.402511495	up-regulated in High
C4orf27	0.14134288	3.56752771	3.33537722	0.00091573	0.0044013	-2.6817964	3.038230624	up-regulated in Low
AADAT	-0.1878199	1.49333306	-2.8208773	0.00498138	0.01926144	-4.23752	2.302650536	up-regulated in High
HMGB2	0.84475235	4.86815362	12.7218237	2.78E-32	5.29E-30	61.9159837	31.55575037	up-regulated in Low
SAP30	0.32881389	2.56477295	5.87449305	7.79E-09	1.09E-07	8.51161307	8.108641922	up-regulated in Low
FBXO8	-0.1282555	2.98195797	-3.563652	0.00040111	0.00213276	-1.9118867	3.396732452	up-regulated in High
HPGD	-1.2557654	3.40539042	-7.8563173	2.48E-14	7.79E-13	20.9308192	13.60621727	up-regulated in High
SPATA4	-0.1221739	0.1920621	-4.358109	1.60E-05	0.00011879	1.14116855	4.797009317	up-regulated in High
VEGFC	0.46822882	1.97270984	5.56438521	4.31E-08	5.31E-07	6.84585321	7.365248123	up-regulated in Low
NEIL3	1.07374998	1.12581034	16.0607665	4.91E-47	2.22E-44	95.7549009	46.30857678	up-regulated in Low
WWC2	-0.2193715	1.97934824	-3.9616036	8.54E-05	0.00054059	-0.454436	4.068667303	up-regulated in High
ING2	0.12981054	3.03816953	3.00237921	0.00281404	0.01174983	-3.717302	2.550669481	up-regulated in Low
RWDD4	0.17582144	2.33842897	5.73156208	1.73E-08	2.28E-07	7.73402468	7.76188668	up-regulated in Low
IRF2	-0.2021322	3.91281489	-5.6644271	2.50E-08	3.19E-07	7.37457695	7.60144158	up-regulated in High
CASP3	0.22315469	3.86692472	5.18504328	3.15E-07	3.31E-06	4.91708061	6.501459544	up-regulated in Low
PRIMPOL	0.11869153	2.15001447	3.2163754	0.00138305	0.00634444	-3.0638411	2.859162591	up-regulated in Low
CENPU	1.05613959	2.50419157	15.3873731	5.91E-44	2.21E-41	88.6852076	43.22869637	up-regulated in Low
ACSL1	-0.3258787	4.26390703	-4.3422154	1.71E-05	0.0001266	1.07449015	4.766693363	up-regulated in High
SLC25A4	-0.2606231	3.11371089	-4.81169	1.99E-06	1.78E-05	3.13842932	5.701235905	up-regulated in High
SNX25	-0.7117481	2.92610601	-10.188631	2.92E-22	2.45E-20	38.9909675	21.53515539	up-regulated in High
LRP2BP	-0.263668	0.6204587	-7.9201912	1.57E-14	5.09E-13	21.3789862	13.80364672	up-regulated in High
C4orf47	-0.1276661	1.08616583	-2.0444871	0.04143334	0.11248284	-6.1057262	1.382650049	up-regulated in High
PDLIM3	-0.2031082	2.03452947	-2.6357802	0.00865756	0.03080182	-4.7356658	2.062604733	up-regulated in High
SORBS2	-0.3421239	1.5911068	-4.0699267	5.47E-05	0.00036307	-0.0326266	4.261843492	up-regulated in High
TLR3	-0.4143495	1.67479499	-6.4603533	2.50E-10	4.48E-09	11.8708407	9.602197915	up-regulated in High
FAM149A	-0.4347462	1.41802812	-6.5259711	1.67E-10	3.08E-09	12.2639702	9.77658604	up-regulated in High
CYP4V2	-0.3929475	1.91426	-7.6456717	1.09E-13	3.15E-12	19.4729645	12.96365411	up-regulated in High
F11	-0.360191	0.35566889	-7.3266646	9.64E-13	2.43E-11	17.325022	12.01588772	up-regulated in High
FAT1	0.25236968	3.2472494	2.68563335	0.00748208	0.02718796	-4.6047261	2.125977747	up-regulated in Low
ZFP42	0.2972961	0.41058485	3.42856507	0.00065736	0.00328886	-2.3733658	3.182200326	up-regulated in Low
PLEKHG4B	-0.2210857	0.97302302	-2.5593233	0.01078339	0.03710571	-4.9318434	1.967244766	up-regulated in High
SDHA	0.22520215	3.9657945	4.50249421	8.38E-06	6.59E-05	1.75722026	5.076676519	up-regulated in Low
PDCD6	0.2096185	3.80380007	3.96681236	8.36E-05	0.00053077	-0.4343972	4.07785605	up-regulated in Low
CEP72	0.28869503	1.68320569	5.49979469	6.10E-08	7.32E-07	6.50890791	7.214602884	up-regulated in Low
TPPP	-0.8485983	1.81669945	-9.594746	4.13E-20	2.74E-18	34.0801964	19.38352698	up-regulated in High
ZDHHC11B	-0.6954213	1.23773688	-7.646702	1.08E-13	3.13E-12	19.4800196	12.96676504	up-regulated in High
ZDHHC11	-0.2685786	0.89274482	-4.1700362	3.60E-05	0.0002479	0.36665385	4.444258807	up-regulated in High
BRD9	0.11250009	2.6614788	2.21788607	0.0270153	0.0795954	-5.739083	1.568390269	up-regulated in Low
TRIP13	1.25606865	2.5390836	15.2317068	3.00E-43	1.11E-40	87.0658068	42.52307355	up-regulated in Low
NKD2	-0.539489	2.04490185	-6.6262652	9.00E-11	1.72E-09	12.8713023	10.04584909	up-regulated in High
SLC12A7	0.1985609	4.6970503	2.41216866	0.01622028	0.05221667	-5.2935763	1.789941577	up-regulated in Low
TERT	0.23388546	0.24540759	6.76372166	3.80E-11	7.72E-10	13.7162392	10.42018209	up-regulated in Low
CLPTM1L	-0.2923778	5.3605181	-4.4598935	1.02E-05	7.85E-05	1.57352706	4.993363798	up-regulated in High
LPCAT1	-0.7116355	6.48827519	-5.9495585	5.09E-09	7.34E-08	8.92666296	8.2935522	up-regulated in High
MRPL36	0.33555878	3.85908549	5.68906882	2.19E-08	2.82E-07	7.50607974	7.660151665	up-regulated in Low
NDUFS6	0.43852402	5.37639876	7.28066176	1.31E-12	3.27E-11	17.0213276	11.88177455	up-regulated in Low
IRX4	0.14722829	0.11196563	3.52473215	0.00046322	0.00242129	-2.0465831	3.334216736	up-regulated in Low
IRX2	-1.4285945	2.57247239	-9.7075785	1.64E-20	1.13E-18	34.9978866	19.78579999	up-regulated in High
C5orf38	-1.1369944	1.88285483	-9.989848	1.56E-21	1.23E-19	37.3253394	20.80564262	up-regulated in High
IRX1	-0.4828337	0.71435631	-5.383275	1.13E-07	1.29E-06	5.90988718	6.946532781	up-regulated in High
ADAMTS16	-0.2740264	0.92986404	-4.2100237	3.03E-05	0.00021234	0.52866944	4.518163037	up-regulated in High
ICE1	0.15376398	2.88466604	2.94858546	0.00334306	0.01365354	-3.8747509	2.475856483	up-regulated in Low
MED10	0.15709572	4.01622368	2.71239323	0.0069122	0.02547709	-4.5334581	2.160383787	up-regulated in Low
NSUN2	0.43196452	4.26156498	7.91093108	1.68E-14	5.41E-13	21.3138383	13.77495027	up-regulated in Low
SRD5A1	0.1794226	2.12536148	2.75241424	0.00613271	0.0230315	-4.4255931	2.212347394	up-regulated in Low
PAPD7	0.12063632	3.03510202	2.25909746	0.02431104	0.0730169	-5.6476407	1.61419652	up-regulated in Low
C5orf49	-0.6216765	1.29865018	-6.4090495	3.41E-10	5.98E-09	11.5658073	9.466836025	up-regulated in High
MTRR	-0.2129757	3.00843533	-3.5997522	0.00035057	0.0018912	-1.7856927	3.455230154	up-regulated in High
SEMA5A	-0.5209415	1.31897691	-7.5503194	2.10E-13	5.83E-12	18.8233118	12.67713627	up-regulated in High
CCT5	0.74143206	5.30313512	12.9601237	2.76E-33	5.45E-31	64.2142979	32.55884152	up-regulated in Low
CMBL	0.51423155	3.65103603	3.99152299	7.56E-05	0.00048542	-0.338996	4.12158582	up-regulated in Low
6-Mar	0.1649646	4.13399286	3.02635287	0.00260397	0.01098857	-3.6462492	2.584364641	up-regulated in Low
ROPN1L	-0.3001271	0.87107707	-3.4039327	0.00071808	0.00355613	-2.4556821	3.143825179	up-regulated in High

CTNND2	0.24992545	0.58684565	2.47816157	0.01353814	0.04497589	-5.133937	1.868441024	up-regulated in Low
DNAH5	-0.4617101	1.40331623	-6.3287958	5.54E-10	9.36E-09	11.0927846	9.256833281	up-regulated in High
FAM105A	-0.4538868	2.91535705	-4.4155688	1.24E-05	9.41E-05	1.38411154	4.907387494	up-regulated in High
OTULIN	0.10303448	2.26827681	2.19420145	0.0286844	0.08350183	-5.7908891	1.542354181	up-regulated in Low
ANKH	-0.1828301	2.7451358	-2.7012451	0.00714468	0.0261495	-4.5632317	2.146017218	up-regulated in High
FBXL7	-0.2970868	1.41840845	-5.2649407	2.09E-07	2.27E-06	5.31322816	6.679174607	up-regulated in High
ZNF622	0.170298	4.52212376	3.78834252	0.00017029	0.00100175	-1.1068712	3.768807379	up-regulated in Low
FAM134B	-0.2989611	1.65078971	-3.3831331	0.00077339	0.00379431	-2.524749	3.111599964	up-regulated in High
BASP1	0.73702507	4.80151381	5.94428668	5.24E-09	7.54E-08	8.89736513	8.28050346	up-regulated in Low
CDH18	0.24365512	0.14430801	5.64581411	2.77E-08	3.51E-07	7.27557926	7.557234438	up-regulated in Low
CDH12	0.10821496	0.09025824	3.82511576	0.0001474	0.00088159	-0.9706941	3.831509609	up-regulated in Low
DROSHA	0.29359489	3.26822843	5.43456271	8.63E-08	1.01E-06	6.17215184	7.06394094	up-regulated in Low
C5orf22	0.29591496	3.25790599	6.09614623	2.19E-09	3.38E-08	9.7503056	8.660160372	up-regulated in Low
PDZD2	-0.5086893	1.21089093	-7.5389158	2.27E-13	6.26E-12	18.7460495	12.64305344	up-regulated in High
MTMR12	-0.1305447	3.38519033	-2.5998183	0.0096056	0.03368623	-4.8286389	2.017475552	up-regulated in High
ZFR	0.18234279	3.89961985	4.90247564	1.28E-06	1.20E-05	3.55985922	5.891211585	up-regulated in Low
SUB1	0.20904774	4.08068613	4.30825682	1.99E-05	0.00014462	0.93277974	4.702231806	up-regulated in Low
NPR3	-0.3286651	1.41638884	-4.726007	2.99E-06	2.57E-05	2.74727105	5.524674311	up-regulated in High
TARS	0.63417579	4.20415321	11.3145747	1.47E-26	1.84E-24	48.8137649	25.83268668	up-regulated in Low
C1QTNF3	-0.3874784	1.47519859	-4.681244	3.69E-06	3.11E-05	2.54547364	5.433494265	up-regulated in High
RAI14	-0.2474887	4.13540938	-3.1681068	0.00162907	0.00730933	-3.2150092	2.788061311	up-regulated in High
BRIX1	0.60346511	3.24629474	11.4771692	3.35E-27	4.41E-25	50.2834741	26.47511352	up-regulated in Low
SPEF2	-0.1574938	0.59470268	-4.0521141	5.89E-05	0.00038755	-0.1027205	4.229777081	up-regulated in High
IL7R	-0.5858889	2.99329515	-5.8445446	9.22E-09	1.27E-07	8.34730155	8.035405946	up-regulated in High
CAPSL	-0.5840774	0.94229571	-5.4844737	6.62E-08	7.89E-07	6.42949444	7.179083231	up-regulated in High
UGT3A1	0.13622228	0.15007867	2.36365414	0.01848063	0.0581812	-5.4082501	1.733283232	up-regulated in Low
UGT3A2	0.11178692	0.16939602	3.49523546	0.00051616	0.00265945	-2.14773	3.287218147	up-regulated in Low
SKP2	0.74543204	2.5065565	11.0677936	1.36E-25	1.59E-23	46.6068426	24.86775885	up-regulated in Low
NADK2	0.24882408	2.71728618	4.89320618	1.34E-06	1.25E-05	3.51650212	5.871678177	up-regulated in Low
RANBP3L	-0.228889	0.32918107	-8.367277	6.05E-16	2.40E-14	24.5936641	15.21851144	up-regulated in High
SLC1A3	-0.286095	1.6042681	-4.0623879	5.65E-05	0.00037346	-0.0623276	4.248257565	up-regulated in High
C5orf42	-0.1009711	1.51551593	-2.0551198	0.04039104	0.11022207	-6.0840875	1.393715015	up-regulated in High
NUP155	0.55406087	2.73089537	9.63492983	2.98E-20	2.00E-18	34.4061787	19.52643339	up-regulated in Low
EGFLAM	0.11441732	0.90972638	2.38496973	0.01745551	0.05548645	-5.3581469	1.758067389	up-regulated in Low
LIFR	-0.4270764	2.47812228	-5.0951947	4.96E-07	5.01E-06	4.47809104	6.304321223	up-regulated in High
OSMR	-0.269484	4.76259532	-2.9866462	0.00296023	0.01227722	-3.7636349	2.528675319	up-regulated in High
FYB	-0.1947814	2.50586671	-2.2564595	0.02447681	0.07345432	-5.6535435	1.611245244	up-regulated in High
DAB2	-0.5140664	3.1464436	-7.2751967	1.36E-12	3.38E-11	16.9853519	11.86588551	up-regulated in High
PTGER4	-0.4946738	2.03083441	-6.9384873	1.24E-11	2.71E-10	14.8112688	10.90487567	up-regulated in High
RPL37	0.18135131	6.40438558	2.9072225	0.00380997	0.0153025	-3.9939468	2.419079045	up-regulated in Low
CARD6	-0.1961618	2.28697884	-3.2175483	0.00137752	0.00632243	-3.0601405	2.860901308	up-regulated in High
C7	-1.8274639	3.33776908	-13.755057	1.08E-36	2.64E-34	72.0281878	35.96774797	up-regulated in High
C6	-0.4400295	0.57722595	-6.2714148	7.80E-10	1.28E-08	10.7576842	9.10799082	up-regulated in High
PLCXD3	-0.1275583	0.40096655	-2.6209277	0.00903844	0.03195442	-4.7742148	2.043906647	up-regulated in High
C5orf51	0.11004931	3.22669122	2.24240053	0.02537693	0.07564238	-5.6848877	1.5955609	up-regulated in Low
FBXO4	-0.1409564	2.39731831	-2.6509451	0.00828357	0.02964467	-4.6960874	2.081782474	up-regulated in High
GHR	-0.1980178	0.39513788	-6.1056777	2.07E-09	3.21E-08	9.80445914	8.684249519	up-regulated in High
CCDC152	-0.2370063	0.60141997	-8.4836009	2.54E-16	1.05E-14	25.4519248	15.59590719	up-regulated in High
SEPP1	-0.8423801	3.84306345	-8.5435047	1.62E-16	6.88E-15	25.897356	15.79172027	up-regulated in High
ANXA2R	-0.1484924	1.35065758	-2.5715608	0.01041457	0.03599996	-4.9008216	1.982358828	up-regulated in High
ZNF131	0.20376023	2.2487955	4.76365537	2.50E-06	2.19E-05	2.91835316	5.601926079	up-regulated in Low
HMGCS1	0.30675861	3.68847845	4.00316951	7.21E-05	0.00046514	-0.2938393	4.142275437	up-regulated in Low
CCL28	-0.3244232	1.70760134	-3.4876148	0.00053073	0.00272466	-2.1737304	3.275129196	up-regulated in High
C5orf28	0.20228398	2.69740362	3.88167523	0.00011778	0.00072105	-0.7588267	3.928939385	up-regulated in Low
C5orf34	0.55051891	1.16721185	12.0075752	2.48E-29	3.83E-27	55.1607157	28.60612533	up-regulated in Low
PAIP1	0.28677356	4.35912541	5.61110196	3.35E-08	4.19E-07	7.09172003	7.475111463	up-regulated in Low
FGF10	-0.1083341	0.15121276	-4.2707891	2.34E-05	0.00016769	0.77762375	4.631603575	up-regulated in High
MRPS30	0.3369192	2.85275654	8.15553731	2.88E-15	1.04E-13	23.0543976	14.54130874	up-regulated in Low
HCN1	-0.1174058	0.16400495	-2.7594796	0.00600366	0.02261227	-4.4063914	2.221584211	up-regulated in High
EMB	-0.7914239	4.00933706	-8.1321589	3.41E-15	1.21E-13	22.8862854	14.46731847	up-regulated in High
PARP8	-0.2619325	2.24175635	-5.801288	1.17E-08	1.59E-07	8.11126695	7.930168189	up-regulated in High
ITGA1	-0.3127225	2.26639477	-4.862011	1.56E-06	1.43E-05	3.37113683	5.806168701	up-regulated in High
PELO	0.13088691	3.07697258	3.19517378	0.00148654	0.0067555	-3.1305108	2.827823	up-regulated in Low
ITGA2	-0.3168071	3.3929948	-2.5269714	0.01181513	0.04010056	-5.0131607	1.927561509	up-regulated in High
FST	0.46476345	1.3754342	3.97768814	8.00E-05	0.00051091	-0.3924772	4.097074532	up-regulated in Low
NDUFS4	0.18302648	4.67078038	3.7188362	0.00022301	0.0012733	-1.3608706	3.651679091	up-regulated in Low
ARL15	-0.2838422	2.44226141	-6.2353498	9.66E-10	1.57E-08	10.5484004	9.015001477	up-regulated in High
HSPB3	-0.1117953	0.2701643	-3.5465802	0.00042733	0.00225285	-1.971143	3.369240176	up-regulated in High
SNX18	-0.3961974	3.14277901	-6.9390063	1.24E-11	2.70E-10	14.8145551	10.90632958	up-regulated in High
ESM1	0.34099112	2.45298411	3.32374411	0.00095392	0.00456416	-2.7197292	3.020489008	up-regulated in Low
GZMK	-0.343341	1.87345809	-3.5244943	0.00046362	0.00242297	-2.047402	3.333836393	up-regulated in High

GPX8	0.31072895	2.91826589	3.74590745	0.00020087	0.00116064	-1.2624715	3.697082223	up-regulated in Low
MCIDAS	0.24090341	0.93090096	3.45482852	0.0005979	0.00302537	-2.2849754	3.223369361	up-regulated in Low
SKIV2L2	0.11211373	3.34684095	2.67649048	0.00768626	0.02783809	-4.6289184	2.114284753	up-regulated in Low
PPAP2A	-0.2046475	3.79370923	-3.014547	0.00270555	0.0113508	-3.6813074	2.567744111	up-regulated in High
SLC38A9	0.11006144	1.47772363	2.92286795	0.00362678	0.01465852	-3.9490525	2.440478556	up-regulated in Low
IL6ST	-0.5979847	4.5116062	-9.4012075	1.99E-19	1.21E-17	32.5233408	18.70085069	up-regulated in High
ANKRD55	-0.1045687	0.19581685	-5.0271755	6.97E-07	6.83E-06	4.15035398	6.156995409	up-regulated in High
MAP3K1	-0.3099198	2.52517908	-6.4363614	2.89E-10	5.12E-09	11.7279379	9.538789021	up-regulated in High
GAPT	-0.4666591	0.83025283	-10.697034	3.62E-24	3.70E-22	43.346933	23.44182195	up-regulated in High
DEPDC1B	1.02163867	1.44206859	17.0908489	8.03E-52	4.78E-49	106.746465	51.09531527	up-regulated in Low
ELOVL7	0.27188432	1.76999861	3.66612493	0.00027286	0.00151944	-1.5505291	3.56406426	up-regulated in Low
ERCC8	0.13305788	1.38672259	4.71456306	3.15E-06	2.70E-05	2.69551342	5.501294321	up-regulated in Low
NDUFAF2	0.24171704	3.71081684	4.47998404	9.28E-06	7.23E-05	1.65995635	5.032571155	up-regulated in Low
SMIM15	0.20422575	4.58961067	5.17523215	3.31E-07	3.47E-06	4.8688094	6.479792968	up-regulated in Low
KIF2A	0.25923054	2.78467306	4.52572729	7.54E-06	6.00E-05	1.85807872	5.122393242	up-regulated in Low
DIMT1	0.15280188	3.16771148	4.01588445	6.84E-05	0.00044391	-0.2443992	4.164920876	up-regulated in Low
IPO11	0.13892414	2.0067618	3.6662048	0.00027278	0.00151912	-1.5502436	3.564196226	up-regulated in Low
RNF180	-0.2648165	0.96481838	-5.2030849	2.87E-07	3.04E-06	5.00606041	6.541391436	up-regulated in High
RGS7BP	-0.1800544	0.39187016	-4.3127404	1.95E-05	0.00014218	0.95143085	4.710718352	up-regulated in High
CWC27	0.21012546	2.71168674	6.00805858	3.64E-09	5.40E-08	9.25328528	8.438985663	up-regulated in Low
ADAMTS6	0.13838838	0.32974459	5.18693457	3.12E-07	3.28E-06	4.92639524	6.505640126	up-regulated in Low
CENPK	0.76530444	1.28789775	15.6462077	3.91E-45	1.57E-42	91.3905931	44.4073976	up-regulated in Low
TRIM23	-0.2080965	1.95851322	-5.2779169	1.96E-07	2.13E-06	5.37807828	6.70825098	up-regulated in High
CTC-534A2.2	0.10904138	1.24160639	2.92133603	0.00364436	0.01472038	-3.9534586	2.438379126	up-regulated in Low
NLN	0.39636401	2.18377829	9.40781379	1.89E-19	1.15E-17	32.5761199	18.72399894	up-regulated in Low
MAST4	-0.3613059	2.05785866	-5.9717886	4.48E-09	6.53E-08	9.05045394	8.348680224	up-regulated in High
CD180	-0.351288	1.36934671	-5.9602266	4.79E-09	6.95E-08	8.98601992	8.319986993	up-regulated in High
PIK3R1	-0.4648013	2.38538017	-9.1005808	2.19E-18	1.18E-16	30.1492118	17.65921917	up-regulated in High
CCNB1	1.635143	3.81684391	23.4874502	1.51E-82	8.81E-78	177.366189	81.82136517	up-regulated in Low
CENPH	0.84773934	2.18734153	17.1000091	7.27E-52	4.38E-49	106.84505	51.13823988	up-regulated in Low
CDK7	0.24361893	3.46249438	5.70148569	2.04E-08	2.65E-07	7.57253329	7.689814994	up-regulated in Low
TAF9	0.3159306	3.99138592	7.23825462	1.74E-12	4.27E-11	16.7427379	11.75872208	up-regulated in Low
TAF9	0.3159306	3.99138592	7.23825462	1.74E-12	4.27E-11	16.7427379	11.75872208	up-regulated in Low
MARVELD2	-0.1574588	3.01593232	-2.8427966	0.00465652	0.01816653	-4.1763602	2.331938519	up-regulated in High
OCLN	-0.4712125	2.59015749	-6.4247215	3.10E-10	5.48E-09	11.6587694	9.508093932	up-regulated in High
SMN2	0.17431081	0.93303471	3.73963286	0.00020581	0.0011853	-1.2853386	3.686534124	up-regulated in Low
SMN1	0.24481672	1.29845019	8.87493443	1.28E-17	6.21E-16	28.4034567	16.89278738	up-regulated in Low
MCCC2	0.2416927	3.78873015	5.04862535	6.26E-07	6.20E-06	4.25327688	6.203275849	up-regulated in Low
MAP1B	0.26278146	2.05157071	2.60557962	0.00944773	0.0332044	-4.8138276	2.024672519	up-regulated in Low
MRPS27	0.18959745	3.29229399	4.59143883	5.59E-06	4.56E-05	2.14592735	5.252766901	up-regulated in Low
ZNF366	-0.2433526	0.55292694	-7.8991393	1.83E-14	5.85E-13	21.2309655	13.7384449	up-regulated in High
TNPO1	0.28525902	3.4092368	6.53084868	1.62E-10	2.99E-09	12.2933266	9.789605235	up-regulated in Low
FCHO2	-0.4056446	3.37449034	-6.7589582	3.92E-11	7.93E-10	13.6867171	10.40710804	up-regulated in High
TMEM171	0.41317313	0.84024476	5.98747285	4.10E-09	6.01E-08	9.13803421	8.387676374	up-regulated in Low
FOXD1	0.31269457	0.40371643	4.57577552	6.00E-06	4.87E-05	2.0769682	5.221547013	up-regulated in Low
BTF3	0.12906901	7.11626227	2.63951741	0.00856401	0.0305155	-4.7259327	2.067322729	up-regulated in Low
ANKRA2	-0.1707779	2.84840124	-4.044678	6.08E-05	0.00039816	-0.1318972	4.216425422	up-regulated in High
UTP15	0.22911709	1.7758671	7.72745709	6.14E-14	1.84E-12	20.0353027	13.21157336	up-regulated in Low
ARHGEF28	-0.1592632	1.11744714	-2.8971959	0.00393175	0.01571921	-4.0225954	2.405413769	up-regulated in High
ENC1	-0.6621895	3.9691881	-8.3599721	6.38E-16	2.52E-14	24.5400644	15.19493795	up-regulated in High
HEXB	-0.1687383	4.81466191	-3.3339672	0.00092029	0.00441953	-2.6864008	3.036077533	up-regulated in High
GFM2	0.24644857	2.90169923	6.04030425	3.02E-09	4.55E-08	9.43450232	8.519645468	up-regulated in Low
FAM169A	0.1681419	0.41117298	5.16182493	3.55E-07	3.69E-06	4.80297795	6.450240266	up-regulated in Low
GCNT4	-0.2942247	0.63864233	-6.2644588	8.13E-10	1.34E-08	10.7172387	9.090021908	up-regulated in High
HMGCR	0.25378421	3.20447271	4.96528948	9.45E-07	9.06E-06	3.85562531	6.024393737	up-regulated in Low
COL4A3BP	-0.3016437	2.66524243	-6.7418361	4.36E-11	8.78E-10	13.580743	10.36017367	up-regulated in High
POLK	-0.1708624	1.75724303	-4.5811393	5.86E-06	4.76E-05	2.10055816	5.232227832	up-regulated in High
ANKDD1B	-0.516719	0.84245185	-9.1338527	1.69E-18	9.26E-17	30.4092795	17.77335845	up-regulated in High
IQGAP2	-0.4268075	1.7761406	-5.6558051	2.62E-08	3.34E-07	7.32868312	7.5809488	up-regulated in High
F2RL2	0.15494256	0.63339887	2.34448118	0.01944747	0.06068512	-5.4529414	1.71113697	up-regulated in Low
ZBED3	-0.150776	1.77755153	-2.581739	0.01011643	0.03514834	-4.8749101	1.994972858	up-regulated in High
PDE8B	-0.1353718	0.79044028	-3.601853	0.00034782	0.00187898	-1.7783118	3.458649515	up-regulated in High
TBCA	0.35062198	4.46872066	8.32997688	7.97E-16	3.10E-14	24.3203425	15.09829714	up-regulated in Low
SCAMP1	0.11980446	3.50151953	2.47415956	0.01368883	0.04539129	-5.1437378	1.863633641	up-regulated in Low
BHMT2	0.40304344	1.72855502	2.63263093	0.00873709	0.03104954	-4.7438574	2.058633033	up-regulated in Low
JMY	-0.1267491	1.96491563	-2.7305823	0.00654749	0.02433098	-4.4846249	2.183925187	up-regulated in High
HOMER1	0.36195258	1.58452013	7.28740013	1.25E-12	3.13E-11	17.0657157	11.90137836	up-regulated in Low
CMYA5	-0.159116	0.43967893	-5.2303764	2.50E-07	2.67E-06	5.14118578	6.602015751	up-regulated in High
THBS4	-0.5200592	1.2198134	-4.3517955	1.64E-05	0.00012179	1.11465432	4.784955501	up-regulated in High
SERINC5	-0.2503526	3.00488993	-4.1812662	3.43E-05	0.00023751	0.41200868	4.464954076	up-regulated in High
ANKRD34B	-0.2314571	0.50327301	-3.3865726	0.00076398	0.00375634	-2.5133557	3.116917556	up-regulated in High

DHFR	0.46375366	1.88110421	10.6362641	6.15E-24	6.19E-22	42.8191872	23.21090322	up-regulated in Low
RASGRF2	-0.2036401	0.91956844	-4.6847185	3.63E-06	3.07E-05	2.5610746	5.440545714	up-regulated in High
CKMT2	-0.1731054	0.62546221	-3.6359197	0.00030596	0.00167929	-1.6580536	3.514329626	up-regulated in High
ZCCHC9	0.14070311	2.28300888	4.42332213	1.20E-05	9.12E-05	1.41711807	4.922374359	up-regulated in Low
SSBP2	-0.3248712	1.30806618	-7.3616921	7.61E-13	1.95E-11	17.557293	12.11844064	up-regulated in High
XRCC4	0.13500345	2.22213054	3.04369696	0.00246106	0.01046721	-3.5945056	2.608877978	up-regulated in Low
COX7C	0.13560632	6.5275742	2.64142316	0.00851666	0.0303616	-4.7209643	2.069730665	up-regulated in Low
MEF2C	-0.6327909	1.98825958	-10.887922	6.72E-25	7.42E-23	45.0168046	24.17234646	up-regulated in High
POLR3G	0.48036628	1.00096743	10.9263603	4.78E-25	5.37E-23	45.35526	24.32038681	up-regulated in Low
LYSMD3	-0.1398611	3.0278782	-3.1396272	0.00179257	0.00794524	-3.3031725	2.746524483	up-regulated in High
ARRDC3	-0.5183421	5.05573679	-6.3656166	4.44E-10	7.62E-09	11.3091818	9.352919211	up-regulated in High
NR2F1	-0.5613693	2.87117908	-7.0045999	8.11E-12	1.82E-10	15.2315065	11.09076305	up-regulated in High
FAM172A	-0.1736396	2.40970544	-4.2603661	2.44E-05	0.00017458	0.7346853	4.612048104	up-regulated in High
ANKRD32	0.39728137	1.28766763	8.33216853	7.85E-16	3.05E-14	24.3363767	15.10534979	up-regulated in Low
FAM81B	-0.4982837	1.02543146	-5.885324	7.32E-09	1.03E-07	8.57121669	8.13520341	up-regulated in High
RHOBTB3	0.24389947	3.29067333	2.77815721	0.00567422	0.02155222	-4.3554006	2.246093702	up-regulated in Low
PCSK1	0.82565235	0.77943824	5.33582507	1.45E-07	1.62E-06	5.66921763	6.838733674	up-regulated in Low
CAST	-0.1726703	4.40163188	-3.4664779	0.00057317	0.00291362	-2.2455632	3.241713793	up-regulated in High
LNPEP	-0.1605009	2.2306912	-3.7722874	0.00018131	0.00105837	-1.1659364	3.741590984	up-regulated in High
RGMB	-0.1997151	1.37613176	-5.1634353	3.52E-07	3.66E-06	4.81087688	6.453786465	up-regulated in High
FAM174A	-0.1020893	3.92760326	-2.1746932	0.03012538	0.08698533	-5.8331503	1.521067533	up-regulated in High
ST8SIA4	-0.284837	2.33719604	-3.509306	0.00049023	0.00254475	-2.0995817	3.309596444	up-regulated in High
SLCO4C1	-0.3541505	1.68376545	-3.4328586	0.00064727	0.0032437	-2.3589596	3.188912845	up-regulated in High
PAM	-0.5483467	4.7150014	-7.8184622	3.24E-14	1.00E-12	20.6665451	13.48977511	up-regulated in High
C5orf30	0.36037697	2.01767602	6.1285776	1.81E-09	2.83E-08	9.93486248	8.742249618	up-regulated in Low
NUDT12	-0.1659827	2.0873588	-2.7725209	0.00577187	0.02186479	-4.370823	2.238683616	up-regulated in High
FBXL17	-0.1281643	1.95919766	-3.2850677	0.00109176	0.00514111	-2.844932	2.961872402	up-regulated in High
PJA2	-0.1936507	4.4931745	-3.9401533	9.31E-05	0.00058484	-0.5366971	4.030934077	up-regulated in High
TMEM232	-0.161072	0.20788817	-5.498094	6.16E-08	7.38E-07	6.50008287	7.210655947	up-regulated in High
SLC25A46	-0.1520765	2.63700826	-3.4402779	0.00063019	0.00316952	-2.3340253	3.200528475	up-regulated in High
WDR36	0.17099231	2.48189105	4.69524245	3.45E-06	2.93E-05	2.60839187	5.461930165	up-regulated in Low
STARD4	0.1997833	2.06454194	3.43522306	0.00064178	0.00321784	-2.351019	3.192612309	up-regulated in Low
EPB41L4A	-0.3828997	1.05186502	-8.6688915	6.24E-17	2.78E-15	26.8372194	16.20477452	up-regulated in High
APC	-0.1114042	1.68391721	-2.827793	0.00487673	0.01890112	-4.2182731	2.311871418	up-regulated in High
SRP19	0.14905838	2.78759189	4.47853318	9.34E-06	7.27E-05	1.65370278	5.029734798	up-regulated in Low
REEP5	-0.2541582	5.25470073	-6.0933965	2.22E-09	3.43E-08	9.73469629	8.653216542	up-regulated in High
MCC	-0.2235353	1.09514735	-4.643767	4.39E-06	3.66E-05	2.37787488	5.357716565	up-regulated in High
YTHDC2	-0.1110196	1.88354762	-3.0295983	0.00257666	0.01088907	-3.6365885	2.588942949	up-regulated in High
KCNN2	-0.1046205	0.51518589	-3.0643088	0.0023006	0.00987251	-3.5326429	2.638158321	up-regulated in High
FEM1C	-0.248124	2.79599359	-5.6390722	2.88E-08	3.63E-07	7.2397913	7.541251476	up-regulated in High
TMED7	0.12559499	4.81817418	2.57075536	0.01043849	0.03606131	-4.9028678	1.981362323	up-regulated in Low
CDO1	-0.3651777	0.77186422	-6.9756824	9.79E-12	2.16E-10	15.0472927	11.00928626	up-regulated in High
AP3S1	0.17414817	3.96948568	4.32520756	1.84E-05	0.00013534	1.00338676	4.734355111	up-regulated in Low
DMXL1	-0.1653197	1.66184942	-4.5485213	6.80E-06	5.45E-05	1.95749519	5.167438121	up-regulated in High
TNFAIP8	-0.210197	1.93133007	-3.6089731	0.00033865	0.00183617	-1.753266	3.470250783	up-regulated in High
HSD17B4	-0.3512825	3.834579	-7.0218464	7.25E-12	1.64E-10	15.34167	11.1394819	up-regulated in High
SRFBP1	0.16870697	2.15045914	4.26227499	2.42E-05	0.00017336	0.74254205	4.615626618	up-regulated in Low
ZNF474	-0.2320867	0.4399644	-4.7204106	3.07E-06	2.64E-05	2.72194598	5.51323498	up-regulated in High
PRDM6	-0.3764178	0.7345064	-10.478631	2.42E-23	2.30E-21	41.4590885	22.61568099	up-regulated in High
CEP120	-0.1349755	2.16780319	-3.3713671	0.00080641	0.00393547	-2.5636402	3.093443148	up-regulated in High
ZNF608	-0.5318452	1.87152629	-8.7715483	2.84E-17	1.32E-15	27.6142046	16.54613538	up-regulated in High
GRAMD3	-0.3263179	2.84234896	-5.6313876	3.00E-08	3.78E-07	7.19904539	7.523053015	up-regulated in High
LMNB1	1.11006474	3.61516381	16.5320009	3.25E-49	1.64E-46	100.758914	48.4880172	up-regulated in Low
SLC12A2	-0.1881819	2.74093464	-2.0334839	0.04253594	0.11481951	-6.128003	1.371243939	up-regulated in High
FBN2	0.1671037	0.29412915	3.00254744	0.00281252	0.01174553	-3.7168052	2.550905172	up-regulated in Low
ISOC1	0.14775477	3.75263126	3.05500299	0.00237182	0.01013638	-3.5606224	2.624918917	up-regulated in Low
CHSY3	-0.2112356	1.00018038	-4.0501903	5.94E-05	0.00039018	-0.1102738	4.226320806	up-regulated in High
HINT1	0.25609339	6.35915658	4.54772075	6.83E-06	5.47E-05	1.95399598	5.165852959	up-regulated in Low
CDC42SE2	-0.1544804	4.07433095	-3.2443525	0.00125667	0.00582189	-2.9752185	2.900778454	up-regulated in High
CSF2	-0.3409915	1.34798611	-3.4355925	0.00064093	0.00321494	-2.3497778	3.193190527	up-regulated in High
PDLIM4	-0.2403758	2.53860751	-2.1940716	0.02869379	0.083525	-5.7911716	1.542212032	up-regulated in High
SLC22A4	-0.1915601	1.1070435	-3.1923779	0.00150071	0.00681406	-3.1392711	2.823702893	up-regulated in High
C5orf56	-0.1768493	0.97240641	-4.623732	4.81E-06	3.98E-05	2.28878365	5.317416116	up-regulated in High
IRF1	0.18092567	3.51462235	2.33665991	0.01985443	0.06169782	-5.4710701	1.702142659	up-regulated in Low
RAD50	0.10472387	2.80483543	2.45752222	0.01433134	0.04713633	-5.1843163	1.843713148	up-regulated in Low
CCNI2	0.14541985	0.58159123	3.37642671	0.00079206	0.00387253	-2.546932	3.101244558	up-regulated in Low
UQCRQ	0.29658679	5.87931698	4.85625505	1.61E-06	1.47E-05	3.34440702	5.794119585	up-regulated in Low
HSPA4	0.35788317	4.62891891	9.23573104	7.51E-19	4.28E-17	31.2097931	18.12463265	up-regulated in Low
C5orf15	0.14812105	5.26679256	3.0065211	0.00277667	0.01160742	-3.7050652	2.556475344	up-regulated in Low
VDAC1	0.57464189	5.78032627	13.134143	5.05E-34	1.04E-31	65.9059221	33.29701495	up-regulated in Low
PPP2CA	0.24239268	4.58632794	7.40272118	5.77E-13	1.50E-11	17.8304911	12.23904292	up-regulated in Low

CDKN2AIPNL	0.34925262	3.63464311	6.98508548	9.21E-12	2.04E-10	15.1071249	11.03575111	up-regulated in Low
JADE2	-0.2756171	2.75527459	-4.8541876	1.62E-06	1.48E-05	3.33481295	5.78979458	up-regulated in High
SAR1B	-0.1159119	2.87851862	-3.0285039	0.00258584	0.01092075	-3.6398473	2.587398652	up-regulated in High
SEC24A	0.1237824	3.07598338	2.69204358	0.00734184	0.02676333	-4.5877166	2.134194825	up-regulated in Low
DDX46	0.16205305	2.99462499	4.25148842	2.54E-05	0.00018075	0.69819004	4.595423751	up-regulated in Low
TXNDC15	-0.276519	3.50365348	-6.4690914	2.37E-10	4.26E-09	11.9229992	9.625339188	up-regulated in High
PITX1	0.83288938	1.86420038	7.09403688	4.52E-12	1.05E-10	15.8051943	11.3444238	up-regulated in Low
H2AFY	0.24933475	3.62592863	7.36303746	7.54E-13	1.93E-11	17.5662322	12.12238717	up-regulated in Low
CXCL14	-1.4626127	4.22868956	-6.5216461	1.72E-10	3.16E-09	12.2379555	9.765048493	up-regulated in High
SPOCK1	0.4167795	1.26397493	3.8914234	0.00011328	0.0006965	-0.7220151	3.945852768	up-regulated in Low
HNRNPA0	0.18876704	4.25219274	4.88625403	1.39E-06	1.29E-05	3.48403291	5.857048334	up-regulated in Low
FAM13B	-0.2928752	2.1466466	-6.5539979	1.41E-10	2.63E-09	12.4329038	9.85150056	up-regulated in High
NME5	-0.374735	1.12106053	-5.5611712	4.39E-08	5.40E-07	6.82900493	7.357717748	up-regulated in High
KIF20A	1.25857995	2.3922623	19.853807	5.67E-65	1.32E-61	136.963027	64.2467398	up-regulated in Low
CDC23	0.19164472	3.0146819	6.05336817	2.80E-09	4.23E-08	9.50815829	8.55242383	up-regulated in Low
GFRA3	-0.5697031	2.18810772	-3.1274131	0.00186722	0.00823237	-3.340749	2.728804792	up-regulated in High
CDC25C	0.95270881	1.21601341	20.6135406	1.23E-68	5.97E-65	145.385705	67.91118618	up-regulated in Low
FAM53C	0.13496735	2.64402646	3.67803749	0.00026075	0.00146052	-1.5078911	3.583773432	up-regulated in Low
REEP2	0.15596468	0.68017881	3.33906696	0.00090392	0.00435062	-2.6697386	3.043868528	up-regulated in Low
EGR1	-0.7593327	5.68443322	-6.4455111	2.74E-10	4.87E-09	11.7823834	9.562948801	up-regulated in High
ETF1	0.19141673	4.70059858	5.12446178	4.28E-07	4.38E-06	4.62032887	6.368220655	up-regulated in Low
HSPA9	0.52974611	5.67977715	12.6270297	6.93E-32	1.27E-29	61.0077278	31.15928647	up-regulated in Low
MZB1	-0.341986	3.71363681	-2.3242051	0.02051788	0.0634186	-5.4998165	1.687867573	up-regulated in High
PROB1	0.1461066	0.82606684	5.0020922	7.89E-07	7.66E-06	4.03049842	6.10308439	up-regulated in Low
DNAJC18	-0.1109495	1.17441565	-3.4133009	0.0006944	0.0034517	-2.4244422	3.158392992	up-regulated in High
ECSCR	-0.3428642	1.82226205	-6.4629049	2.46E-10	4.41E-09	11.8860654	9.608952842	up-regulated in High
ECSCR	-0.3428642	1.82226205	-6.4629049	2.46E-10	4.41E-09	11.8860654	9.608952842	up-regulated in High
TMEM173	-0.9385383	4.73898545	-13.165226	3.72E-34	7.73E-32	66.2092326	33.42935929	up-regulated in High
UBE2D2	0.16049355	4.58477198	5.01524195	7.39E-07	7.22E-06	4.09326427	6.131318715	up-regulated in Low
CXXC5	-0.2577218	3.89424137	-4.283663	2.21E-05	0.00015952	0.83079373	4.655813016	up-regulated in High
PURA	-0.2342781	1.85579174	-5.8059999	1.14E-08	1.55E-07	8.13690383	7.941600536	up-regulated in High
IGIP	-0.490714	2.01110696	-10.222214	2.19E-22	1.87E-20	39.274484	21.65930427	up-regulated in High
CYSTM1	-0.2131	4.77882315	-2.8071741	0.00519477	0.01998466	-4.2755219	2.284433539	up-regulated in High
PFDN1	0.12372311	4.72780637	3.23926046	0.00127885	0.00591242	-2.9914033	2.89318194	up-regulated in Low
HBEGF	-0.3005039	2.94302676	-3.3385225	0.00090566	0.00435789	-2.6715187	3.043036233	up-regulated in High
KHD1-EIF4E	-0.1120833	0.50342747	-5.5578549	4.47E-08	5.49E-07	6.81162915	7.349951351	up-regulated in High
SRA1	0.11199262	3.52125675	2.70575394	0.00704982	0.02588465	-4.5512041	2.151822082	up-regulated in Low
EIF4EBP3	-0.2815791	2.37043278	-3.5533934	0.00041668	0.00220542	-1.9475266	3.380198958	up-regulated in High
CD14	-0.2503133	5.46463771	-2.5140807	0.01225011	0.04133884	-5.0452811	1.911859998	up-regulated in High
TMCO6	0.11700106	2.00304734	3.10152931	0.00203497	0.00887018	-3.4199141	2.691441058	up-regulated in Low
HARS	0.16674147	2.94062567	5.53044727	5.18E-08	6.29E-07	6.66837788	7.285912857	up-regulated in Low
HARS2	0.17570355	3.48161083	4.77848542	2.33E-06	2.05E-05	2.986084	5.632497461	up-regulated in Low
PCDHA1	0.15490548	0.16774687	5.04529746	6.37E-07	6.29E-06	4.23728271	6.196084747	up-regulated in Low
PCDHA11	0.11805652	0.41143232	3.5387211	0.00043993	0.00231259	-1.998331	3.356620928	up-regulated in Low
PCDHAC2	-0.104413	0.52329435	-2.2530523	0.02469236	0.07397194	-5.6611573	1.607437322	up-regulated in High
PCDHB2	0.33471111	1.40546374	4.14587571	3.98E-05	0.00027199	0.26946226	4.399893424	up-regulated in Low
PCDHB4	-0.1790976	0.68095719	-4.8851233	1.40E-06	1.29E-05	3.47875576	5.854670444	up-regulated in High
PCDHB5	0.20947899	1.15783208	2.20259916	0.02808268	0.08206934	-5.7725829	1.551561488	up-regulated in Low
PCDHB6	0.19265829	0.49583674	3.97246227	8.17E-05	0.0005207	-0.4126334	4.087834436	up-regulated in Low
PCDHB8	0.383229	0.69691107	5.8481171	9.03E-09	1.24E-07	8.36686361	8.044126037	up-regulated in Low
PCDHGA1	0.10150324	0.25784907	4.62684443	4.75E-06	3.92E-05	2.30260078	5.323667176	up-regulated in Low
PCDHGB1	0.15565074	0.39108798	4.1718091	3.57E-05	0.00024628	0.37380648	4.447522864	up-regulated in Low
PCDHGB5	0.17292609	0.90464325	2.47226015	0.01376087	0.04557839	-5.1483839	1.861354112	up-regulated in Low
PCDHGA9	-0.1070108	0.37604871	-4.3841614	1.42E-05	0.00010688	1.25095445	4.846904203	up-regulated in High
PCDHGA10	-0.1406733	0.53844847	-3.2863082	0.00108707	0.00512231	-2.840938	2.963743654	up-regulated in High
PCDHGB7	-0.2080698	0.7342452	-5.5753493	4.06E-08	5.02E-07	6.90339353	7.390964062	up-regulated in High
PCDHGA12	-0.1121802	0.45366802	-3.6364333	0.00030537	0.00167651	-1.6562324	3.515172421	up-regulated in High
PCDHGC3	-0.3177051	1.76630841	-4.7727054	2.40E-06	2.10E-05	2.95966303	5.62057276	up-regulated in High
RELL2	0.1042427	0.79975638	2.59190641	0.00982624	0.03433718	-4.8489269	2.007612609	up-regulated in Low
FCHSD1	-0.1767257	2.04021903	-3.4395744	0.00063179	0.00317702	-2.3363917	3.199426212	up-regulated in High
ARAP3	-0.1937865	2.01308529	-3.8002851	0.00016251	0.00096019	-1.0627821	3.789115005	up-regulated in High
PCDH1	-0.2077105	3.58646575	-2.7359704	0.00644284	0.02401084	-4.4700983	2.190922923	up-regulated in High
KIAA0141	-0.2147915	3.31630278	-5.1907921	3.06E-07	3.22E-06	4.94540323	6.514170968	up-regulated in High
PCDH12	-0.2716279	1.8309	-4.9330774	1.11E-06	1.04E-05	3.70352473	5.95591809	up-regulated in High
GNPDA1	0.19226587	3.74633125	4.23838336	2.69E-05	0.00019013	0.64444555	4.570936429	up-regulated in Low
NDFIP1	-0.1222192	4.55800686	-2.9900629	0.0029279	0.01216043	-3.7535929	2.53344373	up-regulated in High
SPRY4	-0.6254157	3.29608998	-8.0946404	4.48E-15	1.56E-13	22.617265	14.34890375	up-regulated in High
FGF1	-0.2018706	0.83707902	-3.7935492	0.00016686	0.00098348	-1.0876655	3.777654436	up-regulated in High
NR3C1	-0.1265865	3.34485178	-2.2473519	0.02505669	0.07488279	-5.6738705	1.601076243	up-regulated in High
SH3RF2	-0.190298	1.2432164	-2.1802327	0.02970999	0.0859943	-5.8211875	1.527097515	up-regulated in High
LARS	0.12321479	3.70406612	3.05830097	0.00234635	0.01003489	-3.5507158	2.629607214	up-regulated in Low

RBM27	0.1381103	2.2965506	4.64178705	4.43E-06	3.69E-05	2.36905464	5.35372732	up-regulated in Low
TCERG1	0.31804663	2.73395764	7.96447806	1.14E-14	3.79E-13	21.6913721	13.94123342	up-regulated in Low
STK32A	-0.7019484	1.7743019	-8.7367683	3.71E-17	1.69E-15	27.350212	16.43016378	up-regulated in High
DPYSL3	-0.2047708	4.55645374	-1.9692007	0.04948673	0.12942939	-6.2557841	1.305511258	up-regulated in High
SPINK1	-0.62691	4.28488001	-2.229701	0.02621458	0.07765553	-5.713036	1.581457152	up-regulated in High
SCGB3A2	-2.5848906	5.72383581	-9.762425	1.04E-20	7.36E-19	35.4466008	19.98246177	up-regulated in High
SPINK5	-0.7628678	1.75729658	-5.4847263	6.61E-08	7.88E-07	6.43080216	7.17966819	up-regulated in High
SPINK14	-0.1094715	0.33876066	-2.4213102	0.01582278	0.05119671	-5.2717137	1.800717209	up-regulated in High
SPINK13	-0.3349808	0.71272041	-3.604265	0.00034469	0.00186482	-1.7698326	3.462577401	up-regulated in High
FBXO38	-0.2706789	2.56383038	-6.5529287	1.42E-10	2.64E-09	12.426448	9.848637933	up-regulated in High
ADRB2	-0.5919533	1.22178285	-9.8035099	7.41E-21	5.37E-19	35.7838485	20.13025591	up-regulated in High
GRPEL2	0.32197735	2.33603826	9.00785411	4.54E-18	2.35E-16	29.4280005	17.34264224	up-regulated in Low
ARHGEF37	-0.198762	1.26891868	-3.2931695	0.00106144	0.00501776	-2.818821	2.974104291	up-regulated in High
SLC26A2	0.17660628	2.19560479	2.11493148	0.03493433	0.09794058	-5.9603082	1.456747606	up-regulated in Low
HMGXB3	0.11697813	3.34647852	3.06978149	0.00225965	0.00972337	-3.51615	2.645959753	up-regulated in Low
CSF1R	-0.5734014	3.84451483	-6.148674	1.61E-09	2.53E-08	10.0496472	8.793294523	up-regulated in High
PDGFRB	-0.3904984	4.0650091	-4.984789	8.59E-07	8.29E-06	3.94813428	6.066026479	up-regulated in High
TCOF1	0.43771077	2.684032	9.63378155	3.00E-20	2.02E-18	34.3968506	19.52234421	up-regulated in Low
CD74	-1.0220247	9.89337299	-9.6179715	3.42E-20	2.28E-18	34.2684941	19.4660757	up-regulated in High
NDST1	-0.1599496	3.46033025	-2.7130181	0.00689937	0.02543799	-4.5317857	2.161190496	up-regulated in High
SYNPO	-0.6771217	3.51615474	-9.5428343	6.32E-20	4.10E-18	33.6604599	19.19950203	up-regulated in High
DCTN4	-0.1266295	3.57167418	-3.2178632	0.00137604	0.00631627	-3.0591466	2.861368281	up-regulated in High
SMIM3	-0.4065556	3.46752924	-5.1693374	3.41E-07	3.57E-06	4.83984646	6.466791677	up-regulated in High
ZNF300	0.26435437	1.08392597	4.10995143	4.63E-05	0.00031255	0.12592244	4.334327593	up-regulated in Low
GPX3	-0.5092318	5.59213065	-4.8234954	1.88E-06	1.69E-05	3.19282524	5.725771166	up-regulated in High
ANXA6	-0.3440909	4.4720316	-5.3593301	1.28E-07	1.45E-06	5.78819977	6.892034474	up-regulated in High
CCDC69	-0.7074433	3.18344554	-9.5190121	7.67E-20	4.92E-18	33.4683693	19.11527693	up-regulated in High
SPARC	-0.281946	7.7632538	-3.0183077	0.00267281	0.01123683	-3.6701542	2.573032704	up-regulated in High
ATOX1	0.1510171	2.94391739	3.67296355	0.00026585	0.00148592	-1.526068	3.575372128	up-regulated in Low
G3BP1	0.21355158	3.8252048	5.60278403	3.50E-08	4.37E-07	7.04781086	7.455494775	up-regulated in Low
GRIA1	-0.2138395	0.24525363	-6.5173252	1.76E-10	3.23E-09	12.2119799	9.753527939	up-regulated in High
FAM114A2	-0.1180981	2.06516212	-3.5528893	0.00041746	0.0022086	-1.9492754	3.379387558	up-regulated in High
GALNT10	-0.6837909	3.6080624	-9.1309652	1.72E-18	9.46E-17	30.3866827	17.76344148	up-regulated in High
SAP30L	-0.2355259	2.43897908	-6.0869716	2.31E-09	3.55E-08	9.69824874	8.637002204	up-regulated in High
HAND1	-0.1372378	0.14833717	-3.231531	0.0013132	0.00605781	-3.0159242	2.881669703	up-regulated in High
LARP1	0.37671536	4.71321571	7.09168328	4.59E-12	1.06E-10	15.7900211	11.33771635	up-regulated in Low
FAXDC2	-0.5451111	2.43161019	-4.9223637	1.17E-06	1.10E-05	3.65313493	5.933225841	up-regulated in High
CNOT8	-0.1567776	4.45404451	-3.1350544	0.00182019	0.00805238	-3.3172572	2.739883825	up-regulated in High
GEMIN5	0.17065637	2.57612027	4.16236269	3.71E-05	0.00025543	0.33572818	4.43014461	up-regulated in Low
MRPL22	0.28673431	2.33890726	7.4599033	3.91E-13	1.04E-11	18.2132722	12.40798297	up-regulated in Low
SGCD	-0.3284238	0.81389953	-7.180121	2.56E-12	6.14E-11	16.3629749	11.59094113	up-regulated in High
TIMD4	-0.1366486	0.54461914	-3.0384389	0.0025036	0.01062571	-3.6102224	2.601434386	up-regulated in High
HAVCR1	0.21958622	0.82051711	2.37426442	0.01796393	0.05684368	-5.3833651	1.74559861	up-regulated in Low
HAVCR2	-0.2889783	2.74744314	-3.5103625	0.00048834	0.00253671	-2.095959	3.311279748	up-regulated in High
ITK	-0.2505827	0.99442782	-4.5168327	7.85E-06	6.21E-05	1.81940931	5.104867542	up-regulated in High
CYFIP2	-0.5728063	2.35601072	-7.9519133	1.25E-14	4.13E-13	21.6026072	13.90214025	up-regulated in High
NIPAL4	0.12663456	0.25273442	3.06498111	0.00229554	0.00985365	-3.5306183	2.639116134	up-regulated in Low
LSM11	0.11018021	1.00768869	4.44952798	1.06E-05	8.20E-05	1.52907492	4.973193146	up-regulated in Low
EBF1	-0.1331758	0.80167194	-3.5273908	0.0004587	0.00240049	-2.0374265	3.338469056	up-regulated in High
RNF145	-0.391856	5.34155982	-4.8906516	1.36E-06	1.26E-05	3.50456652	5.866300456	up-regulated in High
IL12B	-0.1409331	0.27515664	-5.5511338	4.63E-08	5.68E-07	6.77644197	7.334223053	up-regulated in High
ADRA1B	-0.1924283	0.8306017	-3.0560384	0.00236379	0.01010506	-3.5575133	2.626390361	up-regulated in High
CCNJL	-0.5422791	3.06971485	-5.9752595	4.39E-09	6.41E-08	9.06981836	8.357302895	up-regulated in High
C1QTNF2	-0.1769194	0.62524714	-4.7541687	2.62E-06	2.28E-05	2.87512694	5.582411609	up-regulated in High
SLU7	-0.1153674	3.44435752	-3.1724278	0.0016055	0.00722023	-3.2015662	2.794390226	up-regulated in High
PTTG1	1.10826791	3.45583266	13.5719143	6.70E-36	1.59E-33	70.2088135	35.17420695	up-regulated in Low
ATP10B	-0.328934	0.88376786	-3.7197618	0.00022222	0.00126952	-1.3575174	3.653226906	up-regulated in High
GABRG2	0.10036746	0.10093408	3.36162463	0.00083474	0.00405407	-2.5957446	3.078448734	up-regulated in Low
CCNG1	-0.1532919	4.41676703	-2.3522219	0.01905191	0.05968366	-5.4349409	1.72006143	up-regulated in High
NUDCD2	0.11851959	1.98613844	3.77377601	0.00018026	0.00105309	-1.16047	3.744110332	up-regulated in Low
HMMR	1.1948375	2.06321253	17.9691299	5.82E-56	4.92E-53	116.25609	55.23531831	up-regulated in Low
MAT2B	-0.1673965	4.02608827	-3.430474	0.00065285	0.0032683	-2.366963	3.185183829	up-regulated in High
WWC1	-0.1943795	2.72016321	-3.1969977	0.00147736	0.00671955	-3.124792	2.830512379	up-regulated in High
RARS	0.33834562	4.30593264	9.44392578	1.41E-19	8.76E-18	32.8650826	18.85072823	up-regulated in Low
PANK3	0.12936635	2.74455717	2.88761836	0.00405139	0.01613402	-4.0498717	2.392396175	up-regulated in Low
SLIT3	-0.7835268	1.43805903	-10.635312	6.20E-24	6.24E-22	42.8109345	23.20729202	up-regulated in High
SPDL1	0.61623106	1.79001104	14.7454346	4.62E-41	1.44E-38	82.0464327	40.33561433	up-regulated in Low
DOCK2	-0.4356326	1.78586913	-6.6008165	1.05E-10	2.00E-09	12.7164617	9.977215983	up-regulated in High
LCP2	-0.295854	2.58502253	-4.1472517	3.96E-05	0.00027058	0.27498353	4.402414371	up-regulated in High
KCNMB1	-0.2080399	0.83944576	-5.1417092	3.93E-07	4.04E-06	4.70449436	6.406020388	up-regulated in High
RANBP17	-0.1583376	0.74116496	-4.8201757	1.91E-06	1.72E-05	3.17751695	5.718866805	up-regulated in High

NPM1	0.38814136	6.87300411	7.12845898	3.61E-12	8.49E-11	16.027575	11.44272015	up-regulated in Low
FGF18	-0.2532906	0.89797278	-3.2194953	0.00136839	0.00628809	-3.0539948	2.863788707	up-regulated in High
FBXW11	-0.1660707	3.48903864	-3.6211614	0.00032348	0.00176415	-1.7102834	3.490154315	up-regulated in High
STK10	-0.1999593	2.42369197	-3.510461	0.00048816	0.00253601	-2.095621	3.311436791	up-regulated in High
SH3PXD2B	0.18684598	3.15523491	2.45865399	0.0142868	0.04701632	-5.1815644	1.845064937	up-regulated in Low
DUSP1	-0.6204923	6.83048862	-5.2724188	2.01E-07	2.19E-06	5.35058319	6.695923743	up-regulated in High
RPL26L1	0.38735755	3.83163752	7.59376831	1.56E-13	4.39E-12	19.1185371	12.80735429	up-regulated in Low
ATP6V0E1	-0.1653174	6.82494647	-3.2037995	0.0014436	0.00658857	-3.1034379	2.840552661	up-regulated in High
CREBRF	-0.4541454	1.68822379	-12.594507	9.47E-32	1.73E-29	60.6969186	31.02360685	up-regulated in High
BNIP1	0.21084675	2.49717843	4.94858072	1.03E-06	9.76E-06	3.77661721	5.988828113	up-regulated in Low
NKX2-5	0.12018231	0.08920286	3.61068041	0.00033648	0.00182553	-1.7472535	3.473035395	up-regulated in Low
STC2	1.00138953	1.70286921	11.6115664	9.77E-28	1.37E-25	51.5074656	27.01003593	up-regulated in Low
BOD1	0.24591319	4.21139897	4.96664163	9.39E-07	9.01E-06	3.86202957	6.027276267	up-regulated in Low
CPEB4	-0.2868423	2.46068032	-5.8723923	7.88E-09	1.10E-07	8.50006339	8.103494689	up-regulated in High
DRD1	-0.1432382	0.32938871	-3.5036119	0.00050057	0.00258967	-2.1190887	3.300531289	up-regulated in High
SFXN1	0.45643407	2.86129771	10.7317481	2.67E-24	2.77E-22	43.6492473	23.57409191	up-regulated in Low
CPLX2	0.96789118	0.58117507	7.21105801	2.09E-12	5.06E-11	16.5647649	11.68009874	up-regulated in Low
THOC3	0.4435413	1.80957244	11.1840027	4.78E-26	5.79E-24	47.6424661	25.32060187	up-regulated in Low
KIAA1191	-0.1399189	4.81657017	-3.238687	0.00128137	0.00592282	-2.9932243	2.892327103	up-regulated in High
NOP16	0.45831921	2.83626272	9.83915287	5.51E-21	4.08E-19	36.0772003	20.25880345	up-regulated in Low
HIGD2A	0.11749668	6.11284548	2.31254746	0.02115636	0.06506485	-5.5265869	1.674559101	up-regulated in Low
FAF2	0.1050208	3.90854623	2.60069292	0.00958148	0.03360772	-4.8263925	2.018567291	up-regulated in Low
CDHR2	0.18879914	0.39943947	2.50184393	0.01267613	0.0425675	-5.0756236	1.897013388	up-regulated in Low
GPRIN1	0.44358321	1.6848964	6.34588482	5.00E-10	8.51E-09	11.193085	9.301372324	up-regulated in Low
UIMC1	0.10489748	2.43166384	3.31936731	0.00096866	0.00462864	-2.733968	3.013827236	up-regulated in Low
MXD3	0.3004882	1.29812898	6.08200119	2.37E-09	3.64E-08	9.67007442	8.624467807	up-regulated in Low
PRELID1	0.29469552	4.59944215	6.65719097	7.42E-11	1.44E-09	13.0601387	10.12953633	up-regulated in Low
F12	0.56128126	1.38698595	8.15146667	2.96E-15	1.06E-13	23.0250996	14.52841439	up-regulated in Low
PRR7	0.38600235	1.32693433	6.67629727	6.59E-11	1.29E-09	13.1771712	10.18139407	up-regulated in Low
DBN1	0.40711977	4.38469833	5.58529901	3.85E-08	4.78E-07	6.95569698	7.4143371	up-regulated in Low
DOK3	-0.2157968	2.0093501	-3.4670583	0.00057197	0.00290825	-2.2435964	3.242629025	up-regulated in High
DDX41	0.18594274	4.07627744	5.21571485	2.70E-07	2.86E-06	5.06851478	6.569414198	up-regulated in Low
FAM193B	-0.2249297	2.76346899	-3.2226934	0.00135352	0.00622563	-3.043892	2.868534663	up-regulated in High
NHP2	0.28926025	4.83592243	5.78014716	1.32E-08	1.77E-07	7.99646649	7.878968867	up-regulated in Low
HNRNPAB	0.38671649	6.02516782	9.18875357	1.09E-18	6.11E-17	30.8398837	17.96232299	up-regulated in Low
PHYKPL	-0.3573744	2.35121052	-6.985925	9.16E-12	2.03E-10	15.11247	11.03811524	up-regulated in High
CLK4	-0.3625729	1.63443033	-8.1456719	3.09E-15	1.11E-13	22.9834111	14.51006657	up-regulated in High
ZNF354B	-0.1495461	1.47768768	-3.5500831	0.00042182	0.00222785	-1.9590063	3.374872315	up-regulated in High
ZFP2	-0.2653895	0.52623629	-10.268944	1.47E-22	1.28E-20	39.6700036	21.8324859	up-regulated in High
ZNF454	-0.1458031	0.45049943	-3.6354837	0.00030647	0.00168159	-1.6595993	3.513614337	up-regulated in High
ZNF879	-0.1059732	0.8109321	-2.7197995	0.00676154	0.02500229	-4.5136123	2.169954282	up-regulated in High
ZNF354C	-0.1519767	0.9888417	-3.5537006	0.0004162	0.00220317	-1.9464607	3.380693535	up-regulated in High
RUFY1	-0.1009974	3.39123416	-2.534948	0.01155291	0.03935659	-4.9932051	1.937308795	up-regulated in High
CANX	0.11482686	7.04859161	2.28073622	0.02298772	0.06968404	-5.598967	1.638504023	up-regulated in Low
C5orf45	-0.2042988	1.63184959	-3.6778996	0.00026089	0.00146115	-1.5083854	3.583544978	up-regulated in High
TBC1D9B	-0.1044406	3.83512765	-2.2345876	0.02588947	0.07687969	-5.7022236	1.586876828	up-regulated in High
RNF130	-0.2951509	2.99585795	-6.9132385	1.46E-11	3.16E-10	14.6516423	10.83424936	up-regulated in High
GFPT2	0.20833655	1.9634072	2.5761382	0.01027953	0.03562564	-4.8891809	1.988026803	up-regulated in Low
SCGB3A1	-2.8229521	5.42655536	-10.741178	2.45E-24	2.55E-22	43.7314776	23.61006848	up-regulated in High
FLT4	-0.1426294	1.61809239	-2.483193	0.01335078	0.04446476	-5.1215934	1.874493535	up-regulated in High
MGAT1	-0.138603	5.04424088	-3.1627897	0.00165851	0.00742373	-3.2315274	2.780283007	up-regulated in High
BTNL9	-0.5432029	0.87787177	-8.3711926	5.87E-16	2.33E-14	24.6224099	15.23115382	up-regulated in High
TRIM52	-0.2169425	1.45332984	-4.9288967	1.13E-06	1.06E-05	3.68384977	5.947058185	up-regulated in High
DUSP22	-0.2440024	3.16179462	-5.9055957	6.53E-09	9.23E-08	8.68303003	8.185024762	up-regulated in High
IRF4	-0.3115983	1.51502264	-3.595317	0.00035644	0.00191845	-1.8012619	3.448016696	up-regulated in High
EXOC2	0.1423772	3.12985734	3.68672483	0.00025224	0.00141845	-1.4767144	3.598180207	up-regulated in Low
FOXQ1	-0.9378885	3.0431491	-6.1753152	1.38E-09	2.19E-08	10.2023104	8.861172008	up-regulated in High
FOXF2	-0.7216843	1.89966608	-9.6629543	2.36E-20	1.60E-18	34.6340722	19.62633182	up-regulated in High
GMDS	0.32005079	2.92829084	4.65870334	4.09E-06	3.43E-05	2.44452292	5.387856174	up-regulated in Low
MYLK4	-0.1224347	0.39529196	-4.7666715	2.47E-06	2.16E-05	2.93211279	5.608137265	up-regulated in High
WRNIP1	0.30076567	4.07759871	7.5711859	1.82E-13	5.09E-12	18.9649271	12.73960297	up-regulated in Low
SERPINB1	-0.1589124	6.0062824	-1.9955471	0.04653019	0.12332437	-6.2039015	1.332265161	up-regulated in High
SERPINB6	-0.1529653	4.65433843	-2.9368982	0.00346938	0.01410436	-3.9085949	2.459747949	up-regulated in High
NQO2	0.19585615	2.04050568	4.30350014	2.03E-05	0.00014745	0.91301237	4.693236511	up-regulated in Low
TUBB2B	0.38094009	1.70349579	2.47587317	0.01362413	0.04520494	-5.1395431	1.865691358	up-regulated in Low
PSMG4	0.12566173	1.23333367	4.17349775	3.54E-05	0.00024466	0.38062188	4.450632915	up-regulated in Low
SLC22A23	-0.3975461	2.57773465	-4.584579	5.77E-06	4.69E-05	2.11569967	5.239082941	up-regulated in High
PXDC1	-0.247526	3.81060962	-3.3459639	0.00088223	0.00426199	-2.6471657	3.054420875	up-regulated in High
CDYL	0.20666251	3.08217137	4.93282021	1.11E-06	1.05E-05	3.70231398	5.955372887	up-regulated in Low
RPP40	0.47372542	2.02339859	9.44459703	1.40E-19	8.72E-18	32.8704611	18.85308697	up-regulated in Low
PPP1R3G	0.22999544	0.62648017	5.48519815	6.60E-08	7.87E-07	6.43324505	7.180760914	up-regulated in Low

LYRM4	0.20271162	1.8814067	4.60213877	5.32E-06	4.36E-05	2.19315921	5.27414542	up-regulated in Low
F13A1	-0.8689192	3.13130345	-7.2849297	1.28E-12	3.18E-11	17.049438	11.89418946	up-regulated in High
LY86	-0.847187	2.88480964	-10.09748	6.32E-22	5.17E-20	38.2245123	21.19949567	up-regulated in High
SSR1	0.28914661	4.38106405	7.0616347	5.59E-12	1.28E-10	15.5966655	11.25223461	up-regulated in Low
RIOK1	0.43816814	3.11322467	10.5256718	1.61E-23	1.55E-21	41.8636241	22.79273421	up-regulated in Low
DSP	1.09741164	4.77242897	8.46536832	2.91E-16	1.20E-14	25.3168153	15.53650561	up-regulated in Low
SNRNP48	0.24908985	1.85623965	6.08264271	2.37E-09	3.63E-08	9.67370967	8.62608511	up-regulated in Low
EEF1E1	0.55591257	2.60846655	12.591353	9.76E-32	1.78E-29	60.6667974	31.01045763	up-regulated in Low
TFAP2A	0.48515216	1.35064687	5.88301814	7.42E-09	1.04E-07	8.55851935	8.129545233	up-regulated in Low
GCNT2	0.17506771	2.14885236	3.26487126	0.00117088	0.00547	-2.9097541	2.931488667	up-regulated in Low
C6orf52	0.28049701	1.08023804	4.41289704	1.25E-05	9.52E-05	1.37274978	4.902228096	up-regulated in Low
PAK1IP1	0.54790662	3.69067779	10.4621405	2.79E-23	2.64E-21	41.3175519	22.55373142	up-regulated in Low
TMEM14C	0.17834266	6.27205675	3.47879996	0.00054806	0.00280157	-2.2037378	3.261173229	up-regulated in Low
TMEM14B	0.32570224	3.83017989	6.19505307	1.23E-09	1.96E-08	10.3157796	8.911614167	up-regulated in Low
MAK	-0.1148219	0.57033827	-2.7375723	0.00641201	0.02392805	-4.465774	2.193005586	up-regulated in High
SYCP2L	0.13976213	0.35855021	3.34181239	0.00089523	0.00431552	-2.6607584	3.048066894	up-regulated in Low
ELOVL2	0.21812207	0.51298013	4.19021119	3.30E-05	0.00022944	0.44821601	4.481471757	up-regulated in Low
SMIM13	0.35529997	2.15508228	8.21328014	1.88E-15	6.99E-14	23.471197	14.72472699	up-regulated in Low
NEDD9	-0.9030153	3.5960451	-10.310059	1.03E-22	9.21E-21	40.018968	21.98527134	up-regulated in High
TMEM170B	-0.1669797	1.18966171	-3.7859534	0.00017189	0.00100987	-1.1156754	3.764751271	up-regulated in High
ADTRP	-0.1273704	0.9137544	-2.0007144	0.04596817	0.12217514	-6.1936462	1.337542781	up-regulated in High
EDN1	-0.4533244	3.32685301	-4.1023415	4.78E-05	0.00032151	0.09566578	4.320500235	up-regulated in High
PHACTR1	-0.4916793	1.11372293	-9.4503288	1.34E-19	8.37E-18	32.9163987	18.87323267	up-regulated in High
TBC1D7	0.31964064	1.60308758	7.77587811	4.37E-14	1.33E-12	20.3704474	13.35929094	up-regulated in Low
NOL7	0.33014153	4.79102539	9.80903896	7.08E-21	5.15E-19	35.8293074	20.15017672	up-regulated in Low
RNF182	0.14248357	0.33571637	3.10272911	0.0020269	0.00884159	-3.4162585	2.693167383	up-regulated in Low
CD83	-0.6756124	3.38965434	-8.763527	3.02E-17	1.40E-15	27.553252	16.51935998	up-regulated in High
JARID2	0.22762044	2.4532063	4.52190775	7.68E-06	6.09E-05	1.84146466	5.114863774	up-regulated in Low
MYLIP	-0.4287068	3.71079359	-6.8762655	1.86E-11	3.94E-10	14.4187597	10.7311935	up-regulated in High
GMPR	-0.4818801	2.22815573	-6.3334912	5.38E-10	9.12E-09	11.1203203	9.269061216	up-regulated in High
ATXN1	-0.2331692	2.00225344	-5.7307593	1.74E-08	2.28E-07	7.7297047	7.759958988	up-regulated in High
STMND1	-0.2799751	0.57060523	-4.2897572	2.15E-05	0.00015569	0.85601453	4.667294378	up-regulated in High
CAP2	0.15566379	1.71148795	2.73725017	0.0064182	0.02394349	-4.4666438	2.192586688	up-regulated in Low
FAM8A1	-0.2402379	3.71164985	-4.8023739	2.08E-06	1.85E-05	3.0955894	5.681909935	up-regulated in High
NUP153	0.40537468	3.51501461	8.30213436	9.80E-16	3.76E-14	24.1169229	15.00881833	up-regulated in Low
KIF13A	-0.3657366	2.65051171	-6.6408699	8.22E-11	1.58E-09	12.9603886	10.08533171	up-regulated in High
KDM1B	0.10634469	2.91747543	2.16383899	0.03095382	0.08892446	-5.8565038	1.509285761	up-regulated in Low
DEK	0.42905307	5.01230219	8.10602475	4.12E-15	1.44E-13	22.6987941	14.38479206	up-regulated in Low
RNF144B	-0.5205391	2.7683585	-7.5420528	2.23E-13	6.15E-12	18.7672939	12.65242515	up-regulated in High
ID4	-0.4900028	2.66318621	-4.6114714	5.10E-06	4.19E-05	2.23443804	5.29282628	up-regulated in High
MBOAT1	-0.1680725	3.09716226	-2.2867021	0.0226341	0.06872276	-5.5854677	1.645236705	up-regulated in High
E2F3	0.40723992	2.73241544	8.08331624	4.86E-15	1.69E-13	22.5362542	14.3132422	up-regulated in Low
CDKAL1	0.15461735	2.49663169	3.59392753	0.0003583	0.00192685	-1.8061355	3.445758438	up-regulated in Low
SOX4	0.1673414	5.29319659	2.18295206	0.02950789	0.08550684	-5.8153041	1.530061816	up-regulated in Low
MRS2	0.14448624	3.22086456	3.01662936	0.00268737	0.01128351	-3.6751332	2.570671868	up-regulated in Low
GPLD1	-0.1313084	0.44776555	-4.8094411	2.01E-06	1.80E-05	3.12808082	5.696567729	up-regulated in High
ALDH5A1	-0.330884	2.74300725	-4.9436757	1.05E-06	9.97E-06	3.75346931	5.978406492	up-regulated in High
KIAA0319	0.42018238	0.75490404	5.205367	2.84E-07	3.01E-06	5.01733503	6.546450584	up-regulated in Low
TDP2	0.33521623	4.09864941	5.99901859	3.83E-09	5.66E-08	9.20263196	8.41643608	up-regulated in Low
ACOT13	0.21800112	3.0678675	4.37330323	1.49E-05	0.00011174	1.20512417	4.826078555	up-regulated in Low
GMNN	0.77181654	3.00121677	13.3118578	8.80E-35	1.93E-32	67.6446782	34.05564664	up-regulated in Low
FAM65B	-0.5480619	1.48730852	-7.9693871	1.11E-14	3.66E-13	21.7260821	13.9565197	up-regulated in High
LRRC16A	0.12245739	2.47912541	2.4224417	0.01577418	0.05105643	-5.2690019	1.802053223	up-regulated in Low
SCGN	0.13710812	0.22375175	1.9816827	0.04806688	0.1265663	-6.2312885	1.318154031	up-regulated in Low
TRIM38	-0.2541766	2.74781307	-6.4020018	3.56E-10	6.21E-09	11.5240655	9.448309045	up-regulated in High
HIST1H3B	0.42071738	0.44135231	4.84740904	1.68E-06	1.53E-05	3.30338382	5.775625409	up-regulated in Low
HIST1H3C	0.23683635	0.3392414	3.13093599	0.0018454	0.00815032	-3.3299252	2.73390988	up-regulated in Low
HFE	-0.1075726	2.19441431	-2.0530066	0.04059639	0.11068934	-6.0883968	1.391512578	up-regulated in High
HIST1H2BC	0.24555653	2.73469927	2.06447555	0.03949242	0.10822003	-6.0649561	1.403486219	up-regulated in Low
HIST1H1E	0.35056082	0.77578442	3.44488223	0.0006198	0.00312534	-2.3185255	3.207747456	up-regulated in Low
HIST1H2BD	0.3251509	4.33990156	3.15684943	0.00169198	0.00754924	-3.2499498	2.771605862	up-regulated in Low
HIST1H3D	0.42357285	1.22840441	4.28260835	2.22E-05	0.00016016	0.82643223	4.653827368	up-regulated in Low
HIST1H2AD	0.41675348	0.91389659	4.27714177	2.27E-05	0.00016363	0.80384208	4.643542142	up-regulated in Low
HIST1H2BF	0.32301543	0.60993851	3.86996322	0.00012341	0.00075188	-0.8029394	3.908665753	up-regulated in Low
HIST1H4E	0.24545091	0.85818221	2.21535984	0.02718923	0.07999079	-5.7446347	1.565603171	up-regulated in Low
HIST1H2BG	0.48545541	1.49686539	3.94525626	9.12E-05	0.00057383	-0.5171655	4.039895091	up-regulated in Low
HIST1H2AE	0.61247758	1.60655203	4.69787574	3.41E-06	2.90E-05	2.62024678	5.467287282	up-regulated in Low
HIST1H1D	0.245144	0.55960998	2.65970095	0.0080743	0.02900242	-4.6731353	2.092894926	up-regulated in Low
HIST1H3F	0.1798797	0.22818121	2.58674879	0.0099725	0.03474131	-4.8621199	2.001195914	up-regulated in Low
HIST1H2BH	0.48742173	1.00695194	4.51419734	7.95E-06	6.28E-05	1.80796562	5.099680522	up-regulated in Low
HIST1H3G	0.15800497	0.30537418	3.30612982	0.00101456	0.00482422	-2.7769238	2.993723117	up-regulated in Low

BTN3A2	-0.2279555	3.23228546	-3.1567772	0.00169239	0.0075505	-3.2501736	2.771500446	up-regulated in High
BTN2A2	-0.3040476	2.1284344	-6.3681795	4.37E-10	7.51E-09	11.3242841	9.359624118	up-regulated in High
BTN3A1	-0.2853343	3.41692891	-4.3787761	1.46E-05	0.00010921	1.22821089	4.83656989	up-regulated in High
BTN3A3	-0.3548073	2.79995023	-5.6105184	3.36E-08	4.21E-07	7.08863774	7.473734484	up-regulated in High
HMGN4	0.18134258	5.29190114	3.15911132	0.00167916	0.00750008	-3.242939	2.774908302	up-regulated in Low
ABT1	0.20023324	3.77860237	5.00533644	7.76E-07	7.55E-06	4.04596989	6.110044472	up-regulated in Low
ZNF322	-0.2295462	1.47216853	-3.3342478	0.00091938	0.00441554	-2.6854846	3.036505966	up-regulated in High
HIST1H2BJ	0.59117499	1.41495898	6.16169081	1.49E-09	2.36E-08	10.124167	8.826429413	up-regulated in Low
HIST1H2AG	0.30328433	0.70434677	4.42286142	1.20E-05	9.14E-05	1.41515527	4.921483192	up-regulated in Low
HIST1H4I	0.32028168	3.25957087	3.84986745	0.00013366	0.00080737	-0.8783366	3.873999498	up-regulated in Low
HIST1H2BK	0.38419916	6.00661126	3.80032613	0.00016249	0.00096013	-1.0626302	3.789184952	up-regulated in Low
HIST1H2AH	0.19255877	0.21807357	2.93590384	0.00348033	0.01414394	-3.9114685	2.45837979	up-regulated in Low
PRSS16	-0.2208197	2.40831034	-3.8358679	0.00014127	0.00084879	-0.9306428	3.849939067	up-regulated in High
HIST1H2BL	0.20391801	0.40403085	3.24529327	0.00125261	0.00580585	-2.9722257	2.902182978	up-regulated in Low
HIST1H2AI	0.28524146	1.01838184	3.02343771	0.00262872	0.01107541	-3.6549182	2.580255711	up-regulated in Low
HIST1H3H	0.37939918	1.81355715	3.69811928	0.00024148	0.00136555	-1.435717	3.617119419	up-regulated in Low
HIST1H2AJ	0.18944143	0.25482142	2.90325967	0.00385768	0.01546117	-4.0052811	2.413673526	up-regulated in Low
HIST1H4K	0.13328398	0.74555858	2.22334931	0.02664245	0.07866866	-5.7270557	1.574425875	up-regulated in Low
HIST1H2AL	0.25063168	0.28517435	4.10526297	4.72E-05	0.00031816	0.10727518	4.325806039	up-regulated in Low
HIST1H1B	0.27861756	0.36011022	3.07593456	0.0022144	0.00955171	-3.4975729	2.654744626	up-regulated in Low
HIST1H3I	0.12985654	0.17941684	2.10418204	0.03586572	0.10004259	-5.9828111	1.445320495	up-regulated in Low
HIST1H3J	0.14883648	0.25114766	3.09761446	0.00206152	0.00897534	-3.4318325	2.685812041	up-regulated in Low
HIST1H2AM	0.29754511	0.77574806	3.90432648	0.00010757	0.00066549	-0.6731563	3.968294742	up-regulated in Low
HIST1H2BO	0.45931504	0.60209355	5.92926242	5.71E-09	8.16E-08	8.8139915	8.243367158	up-regulated in Low
OR2B6	0.18262298	0.26293861	5.50164441	6.04E-08	7.26E-07	6.51850881	7.218896745	up-regulated in Low
ZNF165	0.15927018	1.69883234	3.25227575	0.00122287	0.00568381	-2.9499865	2.912618166	up-regulated in Low
ZSCAN26	-0.1238475	2.33822101	-2.937022	0.00346802	0.01410079	-3.9082372	2.459918246	up-regulated in High
PGBD1	0.16784025	1.30744919	3.07258232	0.00223894	0.00964619	-3.5076983	2.649956781	up-regulated in Low
ZSCAN31	-0.2525045	2.20696158	-2.9282692	0.00356543	0.01444355	-3.9334995	2.447887927	up-regulated in High
UBD	0.2829295	2.98712535	1.98238916	0.04798756	0.12639163	-6.2298975	1.318871331	up-regulated in Low
GABBR1	-0.3829575	1.74171048	-4.981474	8.73E-07	8.42E-06	3.93238419	6.058939087	up-regulated in High
HLA-F	-0.3458318	4.52584341	-3.5312698	0.00045219	0.00237024	-2.0240555	3.344677941	up-regulated in High
HLA-A	-0.2401095	9.06059026	-2.8162134	0.0050531	0.01950646	-4.250474	2.296442414	up-regulated in High
ZNRD1	0.15399387	2.59757137	3.72988103	0.00021371	0.00122658	-1.3208061	3.670169881	up-regulated in Low
RNF39	-0.5496117	2.06419178	-5.9967631	3.88E-09	5.73E-08	9.19000394	8.410814147	up-regulated in High
TRIM31	0.38364241	1.23980359	3.39768739	0.00073429	0.00362747	-2.4764627	3.134132013	up-regulated in Low
TRIM15	0.28363601	0.45488596	3.9472726	9.05E-05	0.00056959	-0.5094414	4.043438563	up-regulated in Low
RPP21	0.18105488	2.49262685	3.90063943	0.00010918	0.0006739	-0.6871332	3.961875606	up-regulated in Low
HLA-E	-0.5297843	8.10896417	-7.8837221	2.04E-14	6.49E-13	21.122758	13.69077719	up-regulated in High
PRR3	0.11836658	2.28831133	2.7892896	0.00548574	0.02093573	-4.3248501	2.260765021	up-regulated in Low
ABCF1	0.26896625	4.56041957	5.62002687	3.19E-08	4.01E-07	7.13889717	7.496186358	up-regulated in Low
MRPS18B	0.15720803	5.31049779	3.23865627	0.0012815	0.00592282	-2.9933221	2.892281238	up-regulated in Low
C6orf136	0.28669017	2.99109552	5.74640429	1.59E-08	2.11E-07	7.81399219	7.797567807	up-regulated in Low
DHX16	0.17542571	3.83130408	4.26493961	2.40E-05	0.00017152	0.75351446	4.62062399	up-regulated in Low
NRM	0.41748761	3.07482549	6.75264881	4.08E-11	8.24E-10	13.64764	10.38980193	up-regulated in Low
MDC1	0.32402577	2.76390863	5.97504519	4.40E-09	6.42E-08	9.0686224	8.356770361	up-regulated in Low
TUBB	0.61383703	7.5773755	10.609981	7.74E-24	7.70E-22	42.59152	23.11127918	up-regulated in Low
DDR1	-0.2189778	5.06149437	-3.2986245	0.00104147	0.00493373	-2.8012058	2.982354024	up-regulated in High
VARS2	0.11616793	2.60457118	2.45546481	0.01441262	0.04735298	-5.1893158	1.841257015	up-regulated in Low
SFTA2	-1.7619243	7.00960702	-10.640899	5.91E-24	5.97E-22	42.8593693	23.2284859	up-regulated in High
DPCR1	-0.6746733	0.95675208	-5.2231908	2.59E-07	2.77E-06	5.10554687	6.586028191	up-regulated in High
MUC21	-1.435617	3.13175217	-7.2954183	1.19E-12	2.97E-11	17.1185773	11.92472367	up-regulated in High
PSORS1C1	-0.1276202	0.68917004	-3.2833851	0.00109816	0.00516665	-2.8503472	2.959335087	up-regulated in High
CCHCR1	0.2366518	2.76364784	4.04484973	6.07E-05	0.00039795	-0.131224	4.216733541	up-regulated in Low
TCF19	0.64913691	2.92001628	9.93751122	2.43E-21	1.87E-19	36.8904212	20.6151126	up-regulated in Low
POU5F1	-0.2248753	0.90532553	-2.8369463	0.00474129	0.01845777	-4.1927286	2.324103682	up-regulated in High
HCG27	-0.1677324	0.78632486	-3.9426636	9.22E-05	0.0005795	-0.527092	4.035340993	up-regulated in High
HLA-C	-0.2575997	8.96263082	-3.1154392	0.00194317	0.0085254	-3.3774494	2.711488721	up-regulated in High
HLA-B	-0.3988145	9.33020601	-4.1982958	3.19E-05	0.00022242	0.48100268	4.496426199	up-regulated in High
MICA	-0.2899021	3.13824245	-4.8571863	1.60E-06	1.46E-05	3.34872966	5.796068192	up-regulated in High
MICB	0.23882973	2.20002205	3.25600949	0.00120724	0.00561741	-2.9380758	2.918205763	up-regulated in Low
NFKBIL1	0.15360357	3.65200656	3.1806521	0.00156151	0.00705718	-3.1759308	2.806455895	up-regulated in Low
LTA	-0.1502786	0.72423388	-3.383709	0.00077181	0.00378813	-2.522842	3.112490103	up-regulated in High
TNF	-0.1613016	0.99519669	-2.4211675	0.01582892	0.05121147	-5.2720556	1.800548756	up-regulated in High
LTB	-0.5773028	3.34176919	-5.2528257	2.23E-07	2.40E-06	5.25281062	6.652081673	up-regulated in High
LST1	-0.6347129	2.86058998	-7.2930396	1.21E-12	3.02E-11	17.1028904	11.91779596	up-regulated in High
NCR3	-0.248988	0.82752287	-4.6670701	3.94E-06	3.31E-05	2.48194242	5.40477483	up-regulated in High
AIF1	-0.4909812	4.4375711	-5.369347	1.22E-07	1.38E-06	5.83904656	6.914808218	up-regulated in High
PRRC2A	0.2602273	5.15011243	4.67079084	3.87E-06	3.26E-05	2.49860269	5.412306778	up-regulated in Low
BAG6	0.14771134	5.73744809	3.33680042	0.00091116	0.00438112	-2.677147	3.040404653	up-regulated in Low
C6orf47	-0.1035874	3.78855793	-2.6267018	0.00888862	0.03150487	-4.7592536	2.051165792	up-regulated in High

CSNK2B	0.25810125	4.23570827	6.2375958	9.53E-10	1.55E-08	10.5614035	9.020779753	up-regulated in Low
LY6G5C	-0.140651	0.95629538	-4.620598	4.89E-06	4.03E-05	2.27487953	5.311125378	up-regulated in High
CLIC1	0.2372077	7.72767464	5.07285149	5.55E-07	5.55E-06	4.36999664	6.255744515	up-regulated in Low
MSH5	0.10009756	0.73613587	2.35381207	0.01897154	0.05947654	-5.431236	1.72189753	up-regulated in Low
VARS	0.43809215	4.49170591	8.55375274	1.50E-16	6.41E-15	25.9737917	15.82531816	up-regulated in Low
LSM2	0.4537815	5.170483	7.98664897	9.77E-15	3.25E-13	21.8482647	14.01032659	up-regulated in Low
HSPA1A	0.55072345	4.88336727	6.21090966	1.12E-09	1.80E-08	10.4071609	8.952231843	up-regulated in Low
HSPA1B	0.50588052	5.12215388	6.85851651	2.08E-11	4.38E-10	14.3073299	10.68187582	up-regulated in Low
SLC44A4	-1.0237712	4.9935587	-8.0252216	7.40E-15	2.51E-13	22.1220261	14.13087373	up-regulated in High
EHMT2	0.2555366	3.5136883	4.86937581	1.51E-06	1.39E-05	3.40537959	5.821602995	up-regulated in Low
C2	-0.3977902	3.62486368	-3.642378	0.00029858	0.0016459	-1.6351342	3.524934687	up-regulated in High
ZBTB12	0.2877066	2.13623043	4.54588506	6.88E-06	5.51E-05	1.94597382	5.162218788	up-regulated in Low
NELFE	0.36723025	4.87465386	8.33858629	7.48E-16	2.92E-14	24.3833475	15.12600973	up-regulated in Low
SKIV2L	0.12441837	3.73327384	2.78050011	0.00563407	0.02142761	-4.3489808	2.24917748	up-regulated in Low
C4A	-0.7247691	1.57767735	-9.0771615	2.64E-18	1.40E-16	29.9665617	17.57905151	up-regulated in High
C4B	-0.7527324	1.71482583	-8.801193	2.26E-17	1.07E-15	27.839824	16.64524091	up-regulated in High
TNXB	-0.6874562	1.09119496	-9.352165	2.96E-19	1.76E-17	32.1323382	18.52935123	up-regulated in High
FKBPL	0.26980227	2.77928913	6.0208344	3.38E-09	5.05E-08	9.32498374	8.470901192	up-regulated in Low
PRRT1	0.10336088	0.39202333	3.79143804	0.00016824	0.00099104	-1.0954558	3.774066004	up-regulated in Low
PPT2	0.38616197	1.50554635	7.04458447	6.25E-12	1.42E-10	15.4872494	11.20385639	up-regulated in Low
AGER	-1.4618528	3.74232002	-8.542306	1.63E-16	6.93E-15	25.8884203	15.78779247	up-regulated in High
GPSM3	-0.5875337	4.42271936	-7.8427086	2.73E-14	8.53E-13	20.8356997	13.56430846	up-regulated in High
NOTCH4	-0.2745362	1.70681969	-5.0385701	6.58E-07	6.49E-06	4.20497943	6.181559988	up-regulated in High
HLA-DRA	-1.0079944	9.83340778	-8.9501809	7.13E-18	3.59E-16	28.9821062	17.14687884	up-regulated in High
HLA-DRB5	-1.1510368	7.30937753	-8.1193624	3.74E-15	1.32E-13	22.7944233	14.42688527	up-regulated in High
HLA-DRB1	-1.1224332	8.96075481	-9.7360015	1.30E-20	9.09E-19	35.2302086	19.88762443	up-regulated in High
HLA-DQA1	-0.934543	4.89832046	-7.7606921	4.87E-14	1.47E-12	20.2651616	13.31288848	up-regulated in High
HLA-DQB1	-1.1159316	5.61026904	-8.9203198	9.00E-18	4.45E-16	28.7520499	17.04586457	up-regulated in High
HLA-DQA2	-0.7660002	4.43260394	-4.4361832	1.13E-05	8.65E-05	1.47198671	4.947283087	up-regulated in High
HLA-DQB2	-1.463516	4.4076963	-10.241354	1.86E-22	1.60E-20	39.4363388	21.73017544	up-regulated in High
HLA-DOB	-0.6298187	2.26956602	-7.3988426	5.92E-13	1.54E-11	17.8046132	12.22762017	up-regulated in High
TAP2	0.22431193	3.01131609	3.21451303	0.00139187	0.00637687	-3.0697144	2.856402856	up-regulated in Low
PSMB9	0.23208692	4.21081712	2.27350834	0.02342259	0.07075614	-5.6152758	1.630365094	up-regulated in Low
TAP1	0.32202457	5.09275727	3.47310739	0.00055953	0.00285322	-2.223078	3.252176171	up-regulated in Low
HLA-DMB	-0.7702174	4.16891639	-7.9630719	1.16E-14	3.83E-13	21.6814326	13.93685605	up-regulated in High
HLA-DMA	-0.9818214	5.81825729	-10.588806	9.31E-24	9.17E-22	42.4083542	23.03112534	up-regulated in High
BRD2	0.10705588	5.09803659	2.36525866	0.01840167	0.05796073	-5.4044939	1.735142873	up-regulated in Low
HLA-DOA	-0.9681344	3.78625587	-8.9447382	7.44E-18	3.74E-16	28.9401325	17.12844941	up-regulated in High
HLA-DPA1	-1.0217651	6.16892406	-9.0739141	2.70E-18	1.44E-16	29.9412616	17.56794662	up-regulated in High
HLA-DPB1	-1.0853857	6.92539649	-10.23845	1.91E-22	1.64E-20	39.4117727	21.71941888	up-regulated in High
RXRB	-0.1225077	3.9084744	-3.1309846	0.0018451	0.00814962	-3.3297758	2.733980326	up-regulated in High
SLC39A7	0.12786507	6.3815824	2.34934659	0.01919801	0.06006078	-5.441634	1.716743759	up-regulated in Low
HSD17B8	-0.286945	3.77925453	-3.794205	0.00016643	0.00098125	-1.0852445	3.778769585	up-regulated in High
B3GALT4	-0.2237377	2.83515542	-4.1290658	4.27E-05	0.00029018	0.20215113	4.369153748	up-regulated in High
WDR46	0.12908541	4.25438866	3.23822766	0.00128339	0.00593013	-2.994683	2.891642369	up-regulated in Low
PFDN6	0.40165986	3.77778253	8.25183974	1.42E-15	5.32E-14	23.7507724	14.84773818	up-regulated in Low
RGL2	-0.2029311	4.32651682	-3.504464	0.00049901	0.00258229	-2.1161715	3.301887056	up-regulated in High
ZBTB22	-0.2284373	3.45534204	-5.5342688	5.07E-08	6.17E-07	6.68831411	7.29482614	up-regulated in High
DAXX	0.13806734	4.25152763	3.95813362	8.66E-05	0.00054725	-0.4677716	4.062551623	up-regulated in Low
KIFC1	1.37399034	2.85541048	17.8165137	3.07E-55	2.49E-52	114.595847	54.51261346	up-regulated in Low
PHF1	-0.2080084	4.09390887	-4.8168492	1.94E-06	1.74E-05	3.16218678	5.711952229	up-regulated in High
BAK1	0.16882304	3.98373609	3.19792758	0.00147271	0.00670202	-3.1218752	2.831883946	up-regulated in Low
ITPR3	-0.3332611	4.48851897	-3.702754	0.00023723	0.0013441	-1.419007	3.624836976	up-regulated in High
UQCC2	0.39095387	3.2801498	6.55457877	1.40E-10	2.62E-09	12.4364115	9.853055901	up-regulated in Low
LEMD2	0.12723256	3.10015464	3.19028618	0.00151139	0.00685777	-3.1458201	2.820622488	up-regulated in Low
HMGA1	1.23640469	6.52824819	14.9138471	8.12E-42	2.70E-39	83.7778559	41.09023854	up-regulated in Low
C6orf1	-0.2413909	3.40683867	-4.092375	4.98E-05	0.00033383	0.05611872	4.302423517	up-regulated in High
NUDT3	0.13122749	1.93051972	4.61024083	5.12E-06	4.21E-05	2.22899061	5.290361197	up-regulated in Low
RPS10	0.21117317	6.59534595	3.14156916	0.00178096	0.00789917	-3.2971852	2.749346994	up-regulated in Low
PACSIN1	0.17705348	0.56598207	3.57597955	0.00038313	0.00204609	-1.8689296	3.416653077	up-regulated in Low
SPDEF	-0.4370941	3.87644971	-3.0225287	0.00263648	0.01110411	-3.6576198	2.578975086	up-regulated in High
C6orf106	-0.1067871	5.20662728	-2.3248984	0.02048044	0.06332247	-5.4982202	1.68866066	up-regulated in High
SNRPC	0.44491752	5.89977591	10.0969496	6.35E-22	5.19E-20	38.2200696	21.19754985	up-regulated in Low
UHRF1BP1	0.14759773	2.32754233	3.11939656	0.00191776	0.0084253	-3.3653349	2.717205622	up-regulated in Low
TAF11	0.27223666	4.35519719	6.20223586	1.17E-09	1.89E-08	10.3571492	8.93000293	up-regulated in Low
TCP11	-0.1526557	0.27087371	-3.0647822	0.00229703	0.00985936	-3.5312174	2.638832717	up-regulated in High
SCUBE3	-0.614316	1.40529308	-6.1477746	1.62E-09	2.55E-08	10.0445029	8.791007018	up-regulated in High
DEF6	-0.2955939	3.491667	-4.8758751	1.46E-06	1.35E-05	3.43563744	5.835239799	up-regulated in High
FANCE	0.22924791	2.21699088	4.52884071	7.44E-06	5.92E-05	1.87163087	5.128534679	up-regulated in Low
CLPSL1	0.14695261	0.38512854	2.56627141	0.01057258	0.03645335	-4.9142478	1.975819146	up-regulated in Low
SRPK1	0.45618781	3.81979527	9.30791407	4.22E-19	2.46E-17	31.780767	18.37513058	up-regulated in Low

MAPK13	-0.145944	4.41312417	-2.623907	0.00896086	0.03171819	-4.7664993	2.047650566	up-regulated in High
C6orf222	-0.1967908	0.44062152	-2.9955178	0.00287696	0.01197245	-3.7375374	2.541065917	up-regulated in High
SRSF3	0.22498544	5.1132244	7.16467267	2.84E-12	6.77E-11	16.2624752	11.54653199	up-regulated in Low
CDKN1A	-0.1610192	5.20886813	-2.1475851	0.03223104	0.0918032	-5.8912604	1.491725689	up-regulated in High
RAB44	-0.126767	0.16768658	-8.4403969	3.51E-16	1.44E-14	25.1321212	15.45529849	up-regulated in High
CPNE5	-0.2377533	1.6136745	-3.1645348	0.00164879	0.00738761	-3.226109	2.782834699	up-regulated in High
PPIL1	0.53700499	4.41753312	9.7937776	8.03E-21	5.79E-19	35.7038741	20.09520931	up-regulated in Low
C6orf89	-0.1793442	4.1355429	-4.7591562	2.55E-06	2.23E-05	2.89784266	5.592666971	up-regulated in High
PI16	-0.3227634	0.43533028	-6.2071068	1.14E-09	1.84E-08	10.385227	8.942482985	up-regulated in High
FGD2	-0.361973	1.26840708	-6.8241827	2.59E-11	5.39E-10	14.0924554	10.58676069	up-regulated in High
TMEM217	-0.1037166	0.6583058	-3.0008509	0.00282795	0.01179816	-3.721813	2.548528864	up-regulated in High
CCDC167	0.55042313	4.71495326	8.62238612	8.89E-17	3.90E-15	26.4874475	16.05107317	up-regulated in Low
MDGA1	-0.1319445	0.50377875	-4.2181171	2.93E-05	0.00020586	0.56163648	4.533193387	up-regulated in High
BTBD9	-0.7459276	2.72634292	-11.575718	1.36E-27	1.87E-25	51.1801852	26.86701274	up-regulated in High
GLP1R	-0.1288738	0.17385233	-3.6850437	0.00025387	0.00142649	-1.482753	3.595390042	up-regulated in High
SAYSD1	-0.1341041	3.03529566	-3.5615482	0.00040426	0.00214734	-1.9192035	3.393338633	up-regulated in High
KCNK5	-1.0396682	3.81632598	-9.7739677	9.46E-21	6.73E-19	35.5412531	20.02394302	up-regulated in High
KCNK17	-0.3486617	0.82087076	-4.9335951	1.10E-06	1.04E-05	3.70596238	5.95701576	up-regulated in High
KIF6	-0.1020161	0.20687549	-4.5375377	7.15E-06	5.71E-05	1.90953257	5.145708846	up-regulated in High
DAAM2	-0.7639868	1.94767175	-8.8950604	1.09E-17	5.36E-16	28.5578802	16.96060136	up-regulated in High
MOCS1	-0.425496	1.72406452	-7.8092186	3.46E-14	1.07E-12	20.6021644	13.46140567	up-regulated in High
UNC5CL	-0.4440261	2.37539029	-4.5861922	5.72E-06	4.66E-05	2.12280451	5.24229942	up-regulated in High
OARD1	0.11392217	2.41602482	3.12676503	0.00187126	0.00824831	-3.3427387	2.727866249	up-regulated in Low
APOBEC2	-0.2831639	0.53367483	-4.1109899	4.61E-05	0.00031134	0.13005554	4.336216244	up-regulated in High
NFYA	0.17119413	3.6864851	3.44216761	0.00062591	0.00315177	-2.3276663	3.203490304	up-regulated in Low
TREML1	-0.2809707	0.5137769	-8.8408542	1.67E-17	7.94E-16	28.142544	16.77820109	up-regulated in High
TREM2	-0.8637081	4.1361593	-8.1880981	2.27E-15	8.32E-14	23.2891528	14.64462034	up-regulated in High
TREML2	-0.1090122	0.33653034	-2.8320599	0.00481316	0.01869021	-4.2063752	2.31756966	up-regulated in High
TREM1	-0.5424577	3.30938747	-4.0430005	6.12E-05	0.00040044	-0.1384721	4.213416339	up-regulated in High
FOXP4	-0.1994167	4.18609795	-2.871044	0.00426628	0.01687065	-4.0968683	2.369950909	up-regulated in High
MDFI	0.53301927	3.07289639	5.09913152	4.87E-07	4.93E-06	4.49718108	6.312898681	up-regulated in Low
TFEB	-0.4924974	2.33772528	-9.9666873	1.90E-21	1.48E-19	37.1326868	20.7212472	up-regulated in High
PGC	-2.2952655	4.50643163	-7.0926089	4.57E-12	1.06E-10	15.7959881	11.34035413	up-regulated in High
PRICKLE4	-0.5754325	1.84147646	-8.2710764	1.23E-15	4.65E-14	23.8906189	14.90926379	up-regulated in High
PRICKLE4	-0.5754325	1.84147646	-8.2710764	1.23E-15	4.65E-14	23.8906189	14.90926379	up-regulated in High
MED20	0.25548294	3.08690856	5.51761945	5.55E-08	6.71E-07	6.60154583	7.256030395	up-regulated in Low
BYSL	0.61870789	3.62317041	11.766836	2.33E-28	3.40E-26	52.9316565	27.63234442	up-regulated in Low
CCND3	-0.3464536	4.41300316	-4.7027728	3.33E-06	2.83E-05	2.64230942	5.477256572	up-regulated in High
MRPS10	0.42297497	4.65424359	8.98645186	5.37E-18	2.75E-16	29.2622906	17.26989312	up-regulated in Low
TRERF1	0.29647435	1.62084639	5.41288607	9.68E-08	1.12E-06	6.06103663	7.014205905	up-regulated in Low
GLTSCR1L	-0.1868768	2.41484559	-4.4068234	1.29E-05	9.75E-05	1.34694544	4.890509332	up-regulated in High
RPL7L1	0.28889468	3.96688439	7.44639915	4.29E-13	1.13E-11	18.1226623	12.36799624	up-regulated in Low
PTCRA	-0.1942868	0.45942091	-5.7500467	1.56E-08	2.07E-07	7.8336444	7.806335783	up-regulated in High
GNMT	-0.2514922	0.71476286	-5.1568268	3.64E-07	3.77E-06	4.77847554	6.439239528	up-regulated in High
PPP2R5D	0.24291605	4.13022228	6.12020115	1.90E-09	2.96E-08	9.88711407	8.721013523	up-regulated in Low
MEA1	0.24960696	5.87454721	5.52723115	5.27E-08	6.39E-07	6.65160922	7.27841549	up-regulated in Low
KLHDC3	0.11155515	5.51937364	2.30266221	0.0217113	0.0664774	-5.5491839	1.66331422	up-regulated in Low
RRP36	0.33684045	4.36279561	8.83794062	1.70E-17	8.10E-16	28.1202717	16.76841919	up-regulated in Low
KLC4	-0.2522449	2.62541707	-5.0201122	7.21E-07	7.06E-06	4.11654873	6.141791636	up-regulated in High
MRPL2	0.37324622	3.64085349	8.50719902	2.12E-16	8.93E-15	25.6271165	15.67292627	up-regulated in Low
PTK7	-0.5633758	4.34257954	-7.3377694	8.95E-13	2.27E-11	17.3985633	12.04835965	up-regulated in High
DNPH1	0.23501249	4.53299034	3.64756174	0.00029278	0.00161723	-1.6167101	3.533458226	up-regulated in Low
CRIP3	0.10798163	0.20988403	3.36350535	0.0008292	0.00403118	-2.5895539	3.081340525	up-regulated in Low
DLK2	0.16593259	1.0200935	2.68128457	0.00757858	0.02749585	-4.6162431	2.120412049	up-regulated in Low
POLR1C	0.26665217	3.47815479	6.05716608	2.74E-09	4.15E-08	9.52959713	8.561963899	up-regulated in Low
XPO5	0.46202909	3.02292185	9.61221833	3.58E-20	2.38E-18	34.221822	19.44561528	up-regulated in Low
MAD2L1BP	0.17186008	3.82353981	4.34962413	1.66E-05	0.00012289	1.10554384	4.780813381	up-regulated in Low
RSPH9	-0.2451863	0.91963917	-4.4488746	1.07E-05	8.22E-05	1.52627597	4.971922963	up-regulated in High
MRPS18A	0.29425181	4.11508298	6.31216903	6.12E-10	1.03E-08	10.9954185	9.213592147	up-regulated in Low
TMEM63B	-0.4035993	4.59668331	-6.4513748	2.64E-10	4.71E-09	11.8173095	9.578446218	up-regulated in High
SLC29A1	-0.2555358	4.65102682	-4.0970608	4.89E-05	0.00032793	0.07470088	4.310917822	up-regulated in High
HSP90AB1	0.33854443	8.84892107	6.94843634	1.17E-11	2.55E-10	14.8742994	10.93276076	up-regulated in Low
SLC35B2	0.23945942	5.35751508	4.92043429	1.18E-06	1.11E-05	3.64407112	5.929143738	up-regulated in Low
TCTE1	-0.2521797	0.30868641	-5.2062448	2.83E-07	2.99E-06	5.0216732	6.548397169	up-regulated in High
AARS2	0.13324333	2.60404451	2.9377427	0.00346011	0.0140745	-3.9061538	2.460910164	up-regulated in Low
CDC5L	0.24896207	3.39154445	6.18339593	1.31E-09	2.09E-08	10.2487273	8.881807312	up-regulated in Low
SUPT3H	0.12242394	1.47554454	3.38610348	0.00076526	0.00376199	-2.5149103	3.116192028	up-regulated in Low
CLIC5	-0.9554981	1.78628037	-9.482333	1.03E-19	6.54E-18	33.1732545	18.98587086	up-regulated in High
ENPP4	-0.4931099	3.36951556	-7.490379	3.17E-13	8.56E-12	18.4182389	12.4984273	up-regulated in High
ENPP5	-0.7129332	2.79237705	-8.8412357	1.66E-17	7.93E-16	28.1454608	16.77948215	up-regulated in High
RCAN2	-0.7448308	2.44038903	-8.1869279	2.29E-15	8.38E-14	23.2807037	14.64090222	up-regulated in High

CYP39A1	-0.241809	1.25187759	-3.5898363	0.00036382	0.00195404	-1.8204756	3.439113217	up-regulated in High
SLC25A27	-0.2691073	1.02164695	-4.3495522	1.66E-05	0.00012292	1.10524197	4.780676131	up-regulated in High
ANKRD66	-0.2427125	0.27547859	-5.3257036	1.53E-07	1.70E-06	5.6181265	6.815841894	up-regulated in High
GPR116	-1.8823864	4.60149995	-16.722415	4.23E-50	2.22E-47	102.792886	49.37377609	up-regulated in High
GPR110	-0.9520054	2.11566834	-6.5119326	1.82E-10	3.33E-09	12.1795815	9.739158354	up-regulated in High
CD2AP	0.14383141	4.2883784	2.41385668	0.01614623	0.05204139	-5.2895453	1.791928965	up-regulated in Low
GPR115	0.46058871	1.12570845	4.81311935	1.98E-06	1.77E-05	3.14500905	5.704203919	up-regulated in Low
PTCHD4	-0.2383097	0.38996781	-5.2878855	1.86E-07	2.04E-06	5.42799417	6.73062842	up-regulated in High
CENPQ	0.39905246	2.29210688	7.5116537	2.74E-13	7.47E-12	18.5617177	12.56173218	up-regulated in Low
C6orf141	0.17288106	1.21423733	2.07463572	0.03853589	0.1060518	-6.0440831	1.414134623	up-regulated in Low
CRISP3	0.26396128	0.35751146	3.24396929	0.00125833	0.00582817	-2.9764374	2.9002064	up-regulated in Low
MCM3	0.68115777	4.77946004	12.5109837	2.11E-31	3.75E-29	59.9006045	30.67596768	up-regulated in Low
PAQR8	-0.3842234	2.01010066	-5.5954235	3.65E-08	4.54E-07	7.00900358	7.438156049	up-regulated in High
EFHC1	-0.2822682	1.74867201	-4.6174293	4.96E-06	4.09E-05	2.26083025	5.304768628	up-regulated in High
TMEM14A	0.40396219	4.77822273	6.51742309	1.76E-10	3.23E-09	12.2125679	9.753788753	up-regulated in Low
GSTA3	-0.1178604	0.2593547	-2.667854	0.00788372	0.02842694	-4.6516971	2.103268595	up-regulated in High
ELOVL5	0.20374826	5.10627988	3.44831044	0.00061217	0.0030906	-2.3069721	3.213127665	up-regulated in Low
GCLC	1.08782698	3.05004182	8.39400257	4.96E-16	1.99E-14	24.7900646	15.30488501	up-regulated in Low
KLHL31	0.15197216	0.28476109	5.79756121	1.20E-08	1.62E-07	8.09100306	7.921131498	up-regulated in Low
LRRC1	0.34525643	2.6342188	7.07540215	5.11E-12	1.18E-10	15.6851726	11.2913649	up-regulated in Low
TINAG	0.25120596	0.35882223	3.49944253	0.00050828	0.00262347	-2.133353	3.293901394	up-regulated in Low
FAM83B	0.42607904	0.57869428	6.29543415	6.76E-10	1.12E-08	10.8976392	9.170162414	up-regulated in Low
HMGCLL1	-0.160045	0.28913358	-6.489468	2.09E-10	3.79E-09	12.0448597	9.679400124	up-regulated in High
BMP5	-0.5621072	1.91501255	-5.4284468	8.91E-08	1.04E-06	6.14076162	7.049891889	up-regulated in High
COL21A1	-0.6566965	1.19285507	-8.6378203	7.91E-17	3.49E-15	26.6033757	16.10201814	up-regulated in High
BEND6	0.14335987	0.51984724	3.63056854	0.00031221	0.00170907	-1.6770147	3.505554518	up-regulated in Low
KIAA1586	0.13290671	1.73108343	3.06734346	0.00227781	0.00978835	-3.5235009	2.642482907	up-regulated in Low
BAG2	0.60154414	1.7583923	9.30151762	4.44E-19	2.58E-17	31.7300443	18.3528792	up-regulated in Low
RAB23	0.117849	1.57544876	2.28780268	0.02256939	0.06856542	-5.5829735	1.646480232	up-regulated in Low
PRIM2	0.44538324	2.00652759	10.6561684	5.17E-24	5.25E-22	42.9918363	23.28644935	up-regulated in Low
KHDRBS2	-0.3169498	0.74507356	-4.340692	1.72E-05	0.00012735	1.06811062	4.763792359	up-regulated in High
PTP4A1	0.37542816	5.72540304	4.69934837	3.39E-06	2.88E-05	2.62687912	5.470284272	up-regulated in Low
LMBRD1	-0.3895328	4.61669039	-8.4104971	4.38E-16	1.77E-14	24.911514	15.35829276	up-regulated in High
SMAP1	0.11272129	2.04594144	2.79588643	0.00537674	0.02058703	-4.3066906	2.269481171	up-regulated in Low
OGFRL1	-0.4482403	2.50153173	-7.1500835	3.13E-12	7.41E-11	16.1677262	11.50466083	up-regulated in High
KCNQ5	-0.1371278	0.61930006	-2.3748336	0.01793658	0.05678069	-5.3820272	1.746260418	up-regulated in High
KHDC1L	0.29870882	0.28827511	4.34330987	1.70E-05	0.00012605	1.07907437	4.768777924	up-regulated in Low
KHDC1	0.109542	0.88203964	2.82601974	0.00490337	0.01899377	-4.2232125	2.309505371	up-regulated in Low
MB21D1	0.46175381	1.99514223	8.37156823	5.86E-16	2.33E-14	24.625168	15.2323668	up-regulated in Low
EEF1A1	-0.2033155	9.38967161	-3.445142	0.00061922	0.00312295	-2.3176504	3.208155005	up-regulated in High
SLC17A5	-0.1868985	3.32360349	-3.5984118	0.00035233	0.0019002	-1.7903998	3.453049377	up-regulated in High
CD109	0.37164947	1.93229039	3.69997483	0.00023977	0.00135743	-1.4290294	3.620208249	up-regulated in Low
COX7A2	0.37048442	5.49298335	5.95465105	4.94E-09	7.16E-08	8.95498636	8.306166402	up-regulated in Low
TMEM30A	-0.1993144	5.62530273	-3.5669429	0.00039624	0.00210914	-1.9004327	3.402044849	up-regulated in High
FILIP1	-0.3214374	1.23707408	-4.4334542	1.14E-05	8.75E-05	1.4603318	4.941992605	up-regulated in High
MYO6	-0.3847813	4.12895346	-6.247215	9.00E-10	1.47E-08	10.6171399	9.045546642	up-regulated in High
HMGN3	-0.4861991	6.18062076	-6.746341	4.24E-11	8.55E-10	13.6086031	10.37251295	up-regulated in High
LCA5	-0.4319594	1.27379376	-8.5410438	1.65E-16	6.98E-15	25.8790115	15.78365669	up-regulated in High
SH3BGRL2	-0.6138201	3.62988232	-6.7315658	4.66E-11	9.31E-10	13.5172838	10.33206628	up-regulated in High
ELOVL4	0.39237778	0.64094356	6.14619196	1.63E-09	2.57E-08	10.0354528	8.78698269	up-regulated in Low
TTK	1.08396197	1.54338626	16.9746384	2.81E-51	1.62E-48	105.496962	50.55125809	up-regulated in Low
FAM46A	-0.2248331	3.19371044	-2.5786824	0.01020515	0.03540813	-4.8827021	1.991180614	up-regulated in High
TPBG	0.15085031	2.72120965	2.04704607	0.04118042	0.11194105	-6.1005285	1.38530919	up-regulated in Low
DOPEY1	-0.2209852	1.41718357	-5.6489361	2.72E-08	3.45E-07	7.29216404	7.56464088	up-regulated in High
PGM3	0.28137351	2.89905702	6.02170672	3.36E-09	5.02E-08	9.32988405	8.473082372	up-regulated in Low
ME1	0.46822564	3.279574	4.82717453	1.85E-06	1.66E-05	3.20980237	5.733427816	up-regulated in Low
PRSS35	-0.1027825	0.31306355	-3.0690323	0.00226521	0.00974104	-3.5184094	2.644891136	up-regulated in High
RIPPLY2	0.25760874	0.14810072	6.35229056	4.81E-10	8.21E-09	11.2307413	9.31809246	up-regulated in Low
TBX18	0.20634339	0.3254447	3.84972851	0.00013373	0.00080764	-0.8788566	3.873760346	up-regulated in Low
NT5E	-0.5042923	3.47837287	-3.6709387	0.0002679	0.00149542	-1.5333152	3.572022144	up-regulated in High
SYNCRIP	0.22596305	4.28244831	5.72527573	1.79E-08	2.35E-07	7.70020941	7.746796993	up-regulated in Low
CGA	0.38749011	0.3942116	3.15542521	0.00170009	0.00758024	-3.2543617	2.769527451	up-regulated in Low
SLC35A1	-0.333669	3.14271588	-5.9078093	6.45E-09	9.13E-08	8.69525991	8.190473582	up-regulated in High
ORC3	0.11512776	2.80700815	2.63882286	0.00858133	0.03056974	-4.7277426	2.066445495	up-regulated in Low
AKIRIN2	0.16360336	4.27811643	4.19636858	3.21E-05	0.00022409	0.47318176	4.492859208	up-regulated in Low
CNR1	-0.4008659	0.77321672	-6.8397435	2.35E-11	4.91E-10	14.1897303	10.62982214	up-regulated in High
RNGTT	0.14862504	2.26975567	3.90754287	0.00010619	0.00065786	-0.6609534	3.973898626	up-regulated in Low
PNRC1	-0.2069399	4.99638958	-4.6149938	5.01E-06	4.13E-05	2.25003769	5.299885192	up-regulated in High
SRSF12	0.25956484	0.52471908	6.7438956	4.31E-11	8.68E-10	13.5934778	10.36581399	up-regulated in Low
PM20D2	0.26220047	2.58777833	4.53744518	7.15E-06	5.71E-05	1.9091292	5.145526082	up-regulated in Low
ANKRD6	-0.2142943	1.39044117	-4.6675872	3.93E-06	3.30E-05	2.48425707	5.405821286	up-regulated in High

CASP8AP2	0.17756725	1.62821733	4.470837	9.67E-06	7.51E-05	1.62056145	5.014702023	up-regulated in Low
EPHA7	0.20872415	0.27302925	4.52311055	7.63E-06	6.06E-05	1.84669514	5.117234277	up-regulated in Low
FHL5	-0.463872	0.91063471	-8.8943915	1.10E-17	5.38E-16	28.5527436	16.95834574	up-regulated in High
GPR63	0.1287325	0.25121269	5.9570062	4.88E-09	7.06E-08	8.96809205	8.312003013	up-regulated in Low
NDUFAF4	0.37117536	2.35300843	8.99121681	5.18E-18	2.65E-16	29.2991594	17.2860794	up-regulated in Low
MMS22L	0.24320636	0.64062652	10.1141999	5.48E-22	4.53E-20	38.3647683	21.26092318	up-regulated in Low
FAXC	-0.2424839	0.93765964	-4.8101731	2.00E-06	1.79E-05	3.13144899	5.698087121	up-regulated in High
COQ3	0.43192163	2.24841794	9.92349661	2.73E-21	2.10E-19	36.7742186	20.56420305	up-regulated in Low
PNISR	-0.1806641	2.89537219	-3.0197676	0.00266019	0.01119185	-3.6658207	2.575087281	up-regulated in High
CCNC	0.15228799	3.50203105	2.92557158	0.00359595	0.01455205	-3.9412709	2.444185955	up-regulated in Low
ASCC3	0.14465909	2.49084901	3.02529558	0.00261292	0.01101918	-3.6493943	2.582874013	up-regulated in Low
LIN28B	0.2491908	0.12712561	6.53649805	1.57E-10	2.90E-09	12.3273509	9.804694116	up-regulated in Low
BVES	0.17465125	0.57902491	4.59699459	5.45E-06	4.45E-05	2.17043911	5.263862121	up-regulated in Low
POPDC3	0.86697995	0.69076833	9.99074108	1.55E-21	1.22E-19	37.3327741	20.80889943	up-regulated in Low
ATG5	0.13762043	3.40450445	3.23059843	0.0013174	0.00607336	-3.018879	2.880282206	up-regulated in Low
AIM1	-0.3024893	2.80302064	-3.1602094	0.00167297	0.00747872	-3.2395335	2.776512305	up-regulated in High
RTN4IP1	0.23972095	2.01601988	6.09536347	2.20E-09	3.39E-08	9.74586155	8.658183442	up-regulated in Low
QRSL1	0.16098841	2.05243256	4.398696	1.33E-05	0.00010068	1.31246709	4.874849258	up-regulated in Low
BEND3	0.19725378	1.24956429	5.30980493	1.66E-07	1.84E-06	5.53804785	6.779956746	up-regulated in Low
SOBP	-0.1212945	0.65518434	-2.6286721	0.00883801	0.03135403	-4.7541411	2.053645702	up-regulated in High
SCML4	-0.1384016	0.36351805	-4.8262271	1.86E-06	1.67E-05	3.20542954	5.731455722	up-regulated in High
OSTM1	-0.1131087	2.83516701	-2.3665329	0.01833916	0.05780869	-5.4015091	1.736620457	up-regulated in High
FOXO3	-0.2666511	2.89131823	-4.8471042	1.68E-06	1.53E-05	3.30197136	5.774988598	up-regulated in High
ARMC2	-0.1159854	0.84471697	-3.1646384	0.00164822	0.0073856	-3.2257873	2.782986166	up-regulated in High
SESN1	-0.5086767	2.1768986	-10.039175	1.03E-21	8.25E-20	37.7366364	20.98580741	up-regulated in High
CEP57L1	0.20132078	1.07333325	6.49904596	1.97E-10	3.59E-09	12.1022513	9.704858248	up-regulated in Low
PPIL6	-0.4397965	1.01068176	-7.1843024	2.49E-12	5.99E-11	16.3902073	11.6029741	up-regulated in High
MICAL1	-0.3300816	2.9166436	-4.967368	9.36E-07	8.98E-06	3.86547056	6.028825017	up-regulated in High
AK9	-0.1246939	0.7468102	-4.0405113	6.18E-05	0.0004039	-0.1482238	4.208953165	up-regulated in High
FIG4	-0.1650238	2.71234536	-4.1648842	3.67E-05	0.00025295	0.34588451	4.434780131	up-regulated in High
WASF1	0.46290123	1.76458545	7.03172776	6.80E-12	1.54E-10	15.4048874	11.16743727	up-regulated in Low
DDO	-0.2751017	1.09550512	-4.4912826	8.82E-06	6.90E-05	1.70872014	5.054685844	up-regulated in High
AMD1	0.20266923	3.64363741	4.95944961	9.73E-07	9.30E-06	3.82798384	6.011951813	up-regulated in Low
GTF3C6	0.27656078	5.20943212	6.2725652	7.75E-10	1.28E-08	10.7643772	9.110964245	up-regulated in Low
RPF2	0.37201002	2.87996106	9.09082308	2.37E-18	1.27E-16	30.0730691	17.62579966	up-regulated in Low
SLC16A10	-0.1258307	0.64411024	-3.2811341	0.00110677	0.00520089	-2.8575873	2.955942466	up-regulated in High
REV3L	-0.2057681	1.54543361	-4.6215744	4.86E-06	4.01E-05	2.27921033	5.313084829	up-regulated in High
FYN	-0.3579445	2.71213683	-5.4525566	7.85E-08	9.22E-07	6.26468828	7.105351345	up-regulated in High
FAM229B	-0.309666	1.74831649	-4.7240811	3.01E-06	2.60E-05	2.73855289	5.520736432	up-regulated in High
LAMA4	-0.1623484	2.57713986	-2.1675741	0.03066655	0.08824472	-5.8484804	1.513335058	up-regulated in High
MARCKS	0.29977564	5.16409101	4.09168138	5.00E-05	0.00033461	0.05336979	4.30116685	up-regulated in Low
HDAC2	0.43146223	2.83175453	9.77895486	9.08E-21	6.48E-19	35.5821721	20.04187545	up-regulated in Low
HS3ST5	-0.1756428	0.39761996	-2.8787697	0.00416486	0.01650872	-4.0749947	2.380400157	up-regulated in High
FRK	-0.1092465	1.83970376	-2.085148	0.03756706	0.10388993	-6.0223807	1.425192756	up-regulated in High
NT5DC1	-0.1905926	2.50181664	-4.8804483	1.43E-06	1.32E-05	3.45694981	5.844844232	up-regulated in High
COL10A1	-0.6975462	3.46024433	-5.023885	7.08E-07	6.93E-06	4.1346002	6.14991039	up-regulated in High
TSPYL4	-0.1426446	2.64823834	-2.7532065	0.00611812	0.02298555	-4.4234422	2.213382247	up-regulated in High
TSPYL1	-0.2776071	4.40055071	-5.9214119	5.97E-09	8.51E-08	8.77049979	8.223993209	up-regulated in High
RSPH4A	-0.5345877	0.93323142	-6.0026651	3.75E-09	5.56E-08	9.22305609	8.425528587	up-regulated in High
ZUFSP	0.16118666	1.96801029	4.89614384	1.32E-06	1.23E-05	3.53023472	5.877865322	up-regulated in Low
KPNA5	-0.2105819	1.53355702	-5.5762315	4.05E-08	5.00E-07	6.90802778	7.393035083	up-regulated in High
FAM162B	-0.4713812	1.48529776	-7.2448462	1.67E-12	4.09E-11	16.7859545	11.7778124	up-regulated in High
RFX6	0.12791665	0.1180883	3.17014582	0.00161791	0.00726707	-3.2086679	2.791046908	up-regulated in Low
VGLL2	0.10150451	0.05350055	2.38458166	0.01747372	0.05553435	-5.359063	1.757614633	up-regulated in Low
ROS1	-1.4910067	2.80900087	-12.113174	9.19E-30	1.47E-27	56.1463066	29.03660687	up-regulated in High
DCBLD1	-0.1292284	2.38840544	-2.0125221	0.04470541	0.11953143	-6.1701141	1.349639948	up-regulated in High
NUS1	0.21012446	3.13208105	5.39757283	1.05E-07	1.21E-06	5.98277821	6.979170517	up-regulated in Low
PLN	-0.3483789	1.96660517	-3.758335	0.00019142	0.00111174	-1.2170737	3.718017648	up-regulated in High
ASF1A	0.17821279	3.28582985	3.37745105	0.00078918	0.00386105	-2.5435464	3.102825154	up-regulated in Low
FAM184A	-0.4870288	1.17116185	-7.6471504	1.08E-13	3.12E-12	19.48309	12.9681189	up-regulated in High
MAN1A1	-0.2915264	3.7423991	-3.6169048	0.0003287	0.00178963	-1.7253103	3.483196847	up-regulated in High
HSF2	0.13798026	2.5855078	3.28427265	0.00109478	0.005152	-2.847491	2.960673382	up-regulated in Low
SERINC1	-0.2864102	5.88566468	-6.0269616	3.26E-09	4.88E-08	9.3594163	8.486227174	up-regulated in High
PKIB	0.51917357	1.95850672	4.96502487	9.47E-07	9.07E-06	3.85437224	6.023829729	up-regulated in Low
SMPDL3A	-0.3003175	3.43591415	-3.2018654	0.00145313	0.00662739	-3.1095143	2.837695949	up-regulated in High
NKAIN2	0.24199348	0.21748684	5.71589119	1.89E-08	2.47E-07	7.64978891	7.724295776	up-regulated in Low
TPD52L1	0.40742705	1.89088172	4.83657732	1.77E-06	1.60E-05	3.25324448	5.753018223	up-regulated in Low
HDDC2	0.24969638	2.79048376	5.50531113	5.92E-08	7.13E-07	6.5375493	7.227412078	up-regulated in Low
NCOA7	-0.3564358	4.0976487	-3.8097912	0.00015656	0.00092945	-1.0275941	3.805317953	up-regulated in High
HINT3	-0.1255332	3.78554956	-2.4668683	0.0139672	0.04612441	-5.1615541	1.854890648	up-regulated in High
TRMT11	0.14445493	2.35122411	3.68387282	0.00025501	0.00143206	-1.4869572	3.593447406	up-regulated in Low

CENPW	1.22444209	3.07850224	15.9435225	1.70E-46	7.36E-44	94.5168516	45.76929306	up-regulated in Low
RSPO3	0.63879805	1.10695727	5.94655206	5.17E-09	7.45E-08	8.90995218	8.286109583	up-regulated in Low
RNF146	-0.2693957	3.29257352	-6.991815	8.82E-12	1.96E-10	15.1499854	11.05470822	up-regulated in High
ECHDC1	-0.296351	3.46735912	-5.2309782	2.49E-07	2.67E-06	5.14417236	6.603355463	up-regulated in High
THEMIS	-0.1400952	0.74513773	-2.8322846	0.00480983	0.01868226	-4.2057482	2.317869898	up-regulated in High
PTPRK	-0.1252959	3.32646758	-2.1963435	0.02852987	0.08314317	-5.7862262	1.544700217	up-regulated in High
LAMA2	-0.6826426	1.82064609	-10.549829	1.31E-23	1.27E-21	42.0718125	22.88384686	up-regulated in High
ARHGAP18	-0.4005083	3.49838132	-6.6036442	1.04E-10	1.97E-09	12.733642	9.984831673	up-regulated in High
TMEM200A	-0.250517	1.2804578	-4.2974666	2.08E-05	0.00015092	0.88796804	4.681838662	up-regulated in High
EPB41L2	-0.2995373	2.269455	-4.6068248	5.21E-06	4.27E-05	2.21387611	5.283521283	up-regulated in High
AKAP7	-0.2080993	1.11335397	-5.4855128	6.58E-08	7.86E-07	6.4348741	7.1814896	up-regulated in High
ENPP3	-0.5375125	1.3265466	-4.6920305	3.50E-06	2.97E-05	2.59394016	5.455399275	up-regulated in High
ENPP1	0.24828664	0.78435349	4.43832297	1.12E-05	8.58E-05	1.48113005	4.951433315	up-regulated in Low
CTGF	-0.498839	5.979819	-4.6482169	4.30E-06	3.59E-05	2.39771038	5.366687355	up-regulated in High
MOXD1	-0.7121317	2.95051086	-7.9894445	9.57E-15	3.19E-13	21.8680715	14.01904877	up-regulated in High
STX7	-0.1097501	2.25710998	-3.1496372	0.00173345	0.00771307	-3.2722722	2.761088717	up-regulated in High
VNN2	-0.2934615	1.50354441	-3.6777964	0.00026099	0.00146159	-1.5087553	3.583374039	up-regulated in High
TCF21	-0.6792605	1.04140276	-10.457053	2.92E-23	2.75E-21	41.2739118	22.53463014	up-regulated in High
TBPL1	0.14089614	1.83914194	3.67337128	0.00026543	0.00148389	-1.5246082	3.576046887	up-regulated in Low
SGK1	-0.4981844	3.34890031	-6.2523198	8.74E-10	1.43E-08	10.6467481	9.058702526	up-regulated in High
HBS1L	0.25942163	2.17063528	7.06648563	5.42E-12	1.24E-10	15.6278347	11.26601529	up-regulated in Low
MYB	0.29086317	1.0457059	4.75723191	2.58E-06	2.25E-05	2.88907596	5.588709204	up-regulated in Low
PDE7B	-0.1714458	0.667218	-3.8649176	0.00012591	0.00076553	-0.8219048	3.899947557	up-regulated in High
MTFR2	0.85775978	1.28674827	17.6625158	1.64E-54	1.21E-51	112.923716	53.78470094	up-regulated in Low
MAP7	0.23351952	3.20201669	3.84033561	0.0001388	0.00083565	-0.9139698	3.857609505	up-regulated in Low
MAP3K5	-0.3612884	2.99868713	-5.9851785	4.15E-09	6.09E-08	9.12521022	8.381966657	up-regulated in High
IL22RA2	-0.2549931	0.51525511	-4.8837366	1.41E-06	1.30E-05	3.47228567	5.851754952	up-regulated in High
IFNGR1	-0.3961923	5.45316535	-6.3802823	4.06E-10	7.02E-09	11.3956695	9.391315136	up-regulated in High
PERP	0.33212832	5.8045772	3.97314434	8.15E-05	0.00051948	-0.4100041	4.089039844	up-regulated in Low
KIAA1244	-0.37051	2.32805335	-4.7889728	2.22E-06	1.96E-05	3.0340971	5.654164761	up-regulated in High
HEBP2	0.25112288	2.74590757	5.35039798	1.34E-07	1.51E-06	5.74293053	6.871756722	up-regulated in Low
CCDC28A	-0.3663778	4.00712026	-7.3491398	8.29E-13	2.11E-11	17.4739551	12.08164696	up-regulated in High
ECT2L	-0.270556	0.37751218	-5.0180074	7.29E-07	7.13E-06	4.10648316	6.13726441	up-regulated in High
REPS1	0.2101746	2.17041687	5.69446551	2.12E-08	2.74E-07	7.53494652	7.673037555	up-regulated in Low
ABRACL	0.38427185	4.86176668	5.8536278	8.76E-09	1.21E-07	8.39705913	8.05758562	up-regulated in Low
HECA	-0.1918281	2.89546755	-3.7958311	0.00016537	0.00097551	-1.0792403	3.781535069	up-regulated in High
CITED2	-0.8251994	5.33067687	-9.4563464	1.28E-19	7.99E-18	32.9646482	18.894392	up-regulated in High
VTA1	0.29301072	2.71975935	7.44967762	4.19E-13	1.11E-11	18.144648	12.3776989	up-regulated in Low
FUCA2	0.43715119	4.76077394	8.66735584	6.31E-17	2.81E-15	26.8256476	16.19968981	up-regulated in Low
PHACTR2	-0.1783623	2.00511708	-3.2308554	0.00131624	0.00606897	-3.0180648	2.880664535	up-regulated in High
LTV1	0.35304892	3.59944527	8.93044503	8.32E-18	4.13E-16	28.8299944	17.08008969	up-regulated in Low
SF3B5	0.2092435	6.44628231	4.03353304	6.36E-05	0.00041485	-0.175532	4.196453168	up-regulated in Low
STX11	-0.2862008	2.04012177	-4.1457689	3.98E-05	0.00027208	0.26903358	4.399697691	up-regulated in High
UTRN	-0.4611666	2.96218234	-7.1031054	4.26E-12	9.93E-11	15.8636958	11.37028417	up-regulated in High
FBXO30	0.19414842	1.66549358	5.9324254	5.61E-09	8.02E-08	8.83152852	8.251178892	up-regulated in Low
ADGB	-0.1206033	0.12866386	-4.5267211	7.51E-06	5.97E-05	1.86240348	5.124353134	up-regulated in High
SASH1	-0.1463157	2.0871464	-2.6652548	0.00794404	0.02860667	-4.6585386	2.09995868	up-regulated in High
UST	-0.4006309	1.46763238	-5.6816234	2.28E-08	2.92E-07	7.46629375	7.642390456	up-regulated in High
ZC3H12D	-0.1759288	0.71700525	-4.4992337	8.51E-06	6.68E-05	1.74310401	5.070276458	up-regulated in High
GINM1	-0.1440196	4.40575921	-3.3292288	0.00093574	0.00448561	-2.7018606	3.028847326	up-regulated in High
KATNA1	0.11935653	2.87276514	3.20593651	0.00143314	0.00655056	-3.0967196	2.84371083	up-regulated in Low
NUP43	0.23898501	3.09118771	6.45557575	2.57E-10	4.60E-09	11.8423485	9.589556173	up-regulated in Low
PCMT1	0.20893712	4.27688557	4.89176235	1.35E-06	1.26E-05	3.5097554	5.868638387	up-regulated in Low
LRP11	0.21693714	4.16762579	3.41756346	0.00068386	0.00340571	-2.4102009	3.16503239	up-regulated in Low
RAET1E	0.23176646	0.47340844	4.86196241	1.56E-06	1.43E-05	3.37091094	5.806066881	up-regulated in Low
RAET1G	0.23972378	0.56582627	4.50698865	8.21E-06	6.47E-05	1.77669402	5.085504976	up-regulated in Low
PPP1R14C	-0.7008105	2.7695817	-5.5939727	3.68E-08	4.57E-07	7.00135991	7.434740777	up-regulated in High
IYD	-0.2572554	0.85281625	-3.0485508	0.00242238	0.01032298	-3.5799739	2.615758646	up-regulated in High
PLEKHG1	-0.3335617	2.14263756	-5.5453035	4.78E-08	5.84E-07	6.74594943	7.320592349	up-regulated in High
MTHFD1L	0.4396074	2.89716059	9.69149603	1.87E-20	1.28E-18	34.866637	19.72827199	up-regulated in Low
AKAP12	0.58705636	2.19019121	5.14992639	3.77E-07	3.89E-06	4.74468277	6.42406662	up-regulated in Low
ZBTB2	0.16242054	2.89259116	4.68881595	3.56E-06	3.01E-05	2.57948568	5.4488668	up-regulated in Low
CCDC170	-0.5862769	1.04494953	-8.0150933	7.96E-15	2.68E-13	22.050044	14.09917899	up-regulated in High
SYNE1	-0.6263998	1.51263207	-10.951548	3.82E-25	4.33E-23	45.5774321	24.41756043	up-regulated in High
MYCT1	-0.3337309	1.75511672	-5.9936194	3.95E-09	5.83E-08	9.17241057	8.402981489	up-regulated in High
FBXO5	0.69908279	1.71655303	14.8935039	1.00E-41	3.29E-39	83.5683109	40.99891408	up-regulated in Low
MTRF1L	0.17300877	1.53700727	6.2969069	6.70E-10	1.11E-08	10.9062354	9.17398071	up-regulated in Low
IPCEF1	-0.4141612	1.0395515	-5.8228043	1.04E-08	1.42E-07	8.22848231	7.98243455	up-regulated in High
SYNJ2	0.22073307	1.94729043	4.24077771	2.66E-05	0.00018834	0.65425337	4.575405616	up-regulated in Low
SYTL3	-0.267168	2.93946819	-3.8900827	0.00011389	0.00069951	-0.7270831	3.943524519	up-regulated in High
EZR	-0.2951764	7.44762008	-4.4717923	9.63E-06	7.49E-05	1.62467235	5.016566825	up-regulated in High

RSPH3	-0.1315151	2.06654763	-3.1982602	0.00147104	0.00669602	-3.1208318	2.832374592	up-regulated in High
TAGAP	-0.4381203	1.99658274	-6.3043588	6.41E-10	1.07E-08	10.9497573	9.19331186	up-regulated in High
WTAP	0.11821761	3.81704072	3.25855664	0.00119668	0.00557798	-2.9299428	2.922020629	up-regulated in Low
ACAT2	0.4516254	2.68286077	8.21482793	1.86E-15	6.91E-14	23.4824001	14.72965656	up-regulated in Low
TCP1	0.52944599	4.85724721	12.213534	3.57E-30	5.84E-28	57.0873439	29.44758413	up-regulated in Low
MRPL18	0.26280239	5.06637946	6.12509581	1.85E-09	2.88E-08	9.91500838	8.733419681	up-regulated in Low
SLC22A3	-1.2948463	2.87513374	-10.834899	1.07E-24	1.16E-22	44.5511355	23.96864936	up-regulated in High
PACRG	-0.2601703	0.47187832	-4.8074064	2.03E-06	1.81E-05	3.11872204	5.692345879	up-regulated in High
QKI	-0.1560454	2.68366349	-3.1004634	0.00204217	0.00889756	-3.4231606	2.689907846	up-regulated in High
C6orf118	-0.3323582	0.45077233	-5.0012331	7.92E-07	7.69E-06	4.02640304	6.101241969	up-regulated in High
PDE10A	0.32776344	0.58274933	5.69573471	2.11E-08	2.73E-07	7.54173899	7.676069559	up-regulated in Low
MPC1	-0.2263995	4.28842192	-3.8918576	0.00011308	0.00069551	-0.7203734	3.946606988	up-regulated in High
RPS6KA2	-0.8931143	3.81408252	-12.107198	9.72E-30	1.55E-27	56.090405	29.01219172	up-regulated in High
MLLT4	-0.2481046	3.33456797	-4.3364317	1.76E-05	0.00012946	1.05028153	4.755684353	up-regulated in High
SMOC2	-0.7575588	2.57039003	-7.6169075	1.33E-13	3.78E-12	19.27631	12.87693511	up-regulated in High
C6orf120	0.17664755	3.15806479	3.48601881	0.00053383	0.00273937	-2.1791688	3.27260019	up-regulated in Low
PSMB1	0.2750574	5.84160882	5.81279517	1.10E-08	1.50E-07	8.17390799	7.958101093	up-regulated in Low
TBP	0.20573537	2.91577965	5.46293503	7.43E-08	8.77E-07	6.3181846	7.129287539	up-regulated in Low
PDCD2	0.17863537	2.80164508	5.09542887	4.96E-07	5.01E-06	4.47922599	6.304831185	up-regulated in Low
PDGFA	-0.3574362	2.88799273	-4.2526945	2.53E-05	0.00017988	0.70314388	4.597680507	up-regulated in High
PRKAR1B	0.17512915	2.52551272	2.65757891	0.00812458	0.02915611	-4.6787047	2.090199075	up-regulated in Low
DNAAF5	0.40200613	2.92876182	9.12052912	1.87E-18	1.02E-16	30.3050552	17.72761732	up-regulated in Low
C7orf50	0.13144527	4.04350912	2.12482149	0.03409578	0.0960555	-5.9395049	1.467299385	up-regulated in Low
GPR146	-0.1883325	0.50997985	-7.2596113	1.51E-12	3.73E-11	16.8828744	11.82062316	up-regulated in High
GPER1	-0.1820162	0.88145337	-3.1212773	0.00190579	0.00838092	-3.3595722	2.719924731	up-regulated in High
ZFAND2A	0.42162402	3.00815269	6.62628868	9.00E-11	1.72E-09	12.8714455	10.04591257	up-regulated in Low
MICALL2	-0.2293387	2.671097	-2.9161202	0.00370478	0.0149417	-3.9684437	2.431237668	up-regulated in High
INTS1	0.32609184	4.17865094	4.83215626	1.80E-06	1.63E-05	3.23280906	5.743803121	up-regulated in Low
MAFK	0.38340985	3.57356068	4.05912213	5.72E-05	0.00037799	-0.0751778	4.242378846	up-regulated in Low
TMEM184A	0.23117999	2.61238869	2.78124786	0.00562131	0.02138326	-4.3469308	2.250162134	up-regulated in Low
PSMG3	0.43231224	4.40158355	6.81542009	2.74E-11	5.68E-10	14.0377583	10.56254585	up-regulated in Low
FTSJ2	0.13936671	3.77279592	3.31334881	0.00098929	0.00471868	-2.7535185	3.004678554	up-regulated in Low
NUDT1	0.58710497	2.97004619	11.1429952	6.91E-26	8.27E-24	47.2762786	25.16048862	up-regulated in Low
SNX8	0.15598789	3.15866595	2.47393718	0.01369725	0.04540888	-5.1442819	1.863366684	up-regulated in Low
EIF3B	0.47758402	4.76751366	9.50041889	8.92E-20	5.69E-18	33.3186725	19.0496371	up-regulated in Low
CHST12	-0.1281363	1.63107581	-3.2938206	0.00105904	0.00500843	-2.8167199	2.975088396	up-regulated in High
LFNG	-0.3090646	3.95672481	-2.8456455	0.00461574	0.01802671	-4.1683774	2.335758556	up-regulated in High
BRAT1	0.12784571	3.32856959	2.31232489	0.02116872	0.06509257	-5.5270967	1.674305505	up-regulated in Low
CARD11	-0.2343143	2.72165931	-2.2845118	0.02276338	0.06907569	-5.5904279	1.642763306	up-regulated in High
SDK1	-0.1966082	1.87799602	-1.9729699	0.0490543	0.12850564	-6.2484032	1.309322885	up-regulated in High
FOXK1	0.18424471	2.44844613	3.08680716	0.00213647	0.00925662	-3.4646592	2.670302821	up-regulated in Low
RADIL	-0.1097055	0.47089366	-2.704289	0.00708051	0.02597775	-4.555114	2.149935254	up-regulated in High
SLC29A4	0.25385922	2.08255233	2.16758428	0.03066577	0.08824472	-5.8484585	1.513346112	up-regulated in Low
FBXL18	0.23788607	1.32082696	5.41326022	9.66E-08	1.12E-06	6.06295118	7.015062951	up-regulated in Low
FSCN1	0.5918709	4.51776193	4.98668615	8.51E-07	8.22E-06	3.95715236	6.070084385	up-regulated in Low
PMS2	0.19515796	2.01860558	4.63891221	4.49E-06	3.73E-05	2.35625417	5.347937651	up-regulated in Low
AIMP2	0.56271149	3.69170486	12.0536932	1.61E-29	2.52E-27	55.5905734	28.7938824	up-regulated in Low
EIF2AK1	0.2317152	5.51378011	5.33607957	1.45E-07	1.62E-06	5.67050337	6.839309727	up-regulated in Low
USP42	0.18081991	1.91705327	4.8046001	2.06E-06	1.83E-05	3.10581971	5.686525283	up-regulated in Low
CYTH3	-0.6331187	3.25543662	-8.0665292	5.49E-15	1.88E-13	22.4163227	14.26044489	up-regulated in High
FAM220A	0.20074287	3.47177651	4.11187025	4.59E-05	0.00031023	0.13355982	4.337817517	up-regulated in Low
RAC1	0.12632703	6.82917112	2.86306584	0.00437337	0.01721717	-4.1193968	2.359184066	up-regulated in Low
DAGLB	-0.1724261	2.86888958	-3.7215014	0.00022073	0.00126203	-1.3512131	3.656136818	up-regulated in High
GRID2IP	-0.1805346	0.61078504	-6.2646596	8.12E-10	1.33E-08	10.7184058	9.090540409	up-regulated in High
ZNF853	-0.1763108	1.67798071	-2.3524632	0.0190397	0.05965499	-5.4343789	1.720339965	up-regulated in High
COL28A1	-0.2224723	0.94710931	-2.7912526	0.0054531	0.02083703	-4.3194508	2.263356922	up-regulated in High
RPA3	0.39072654	3.10863913	7.19296741	2.36E-12	5.68E-11	16.4466817	11.62792726	up-regulated in Low
GLCCI1	-0.5148334	2.3570749	-6.5770677	1.22E-10	2.30E-09	12.5724152	9.91335756	up-regulated in High
PHF14	0.15539233	2.61305488	3.66634002	0.00027263	0.00151862	-1.5497604	3.564419638	up-regulated in Low
VWDE	0.28539783	0.59319243	5.20956552	2.78E-07	2.95E-06	5.03808964	6.555763219	up-regulated in Low
ARL4A	0.16811529	2.87273617	2.66978728	0.00783913	0.02829029	-4.6466044	2.105732062	up-regulated in Low
ETV1	-0.7347011	2.12799543	-9.1067492	2.09E-18	1.13E-16	30.1973752	17.68035794	up-regulated in High
AGMO	0.15847197	0.23126431	3.44298175	0.00062407	0.00314415	-2.3249256	3.204766768	up-regulated in Low
MEOX2	-0.5821942	1.39522509	-8.6780515	5.82E-17	2.60E-15	26.9062763	16.23511799	up-regulated in High
SOSTDC1	-0.442838	0.68941016	-5.280476	1.93E-07	2.11E-06	5.39088444	6.713992264	up-regulated in High
ANKMY2	-0.1321756	3.03144651	-2.7192421	0.00677278	0.02503432	-4.5151077	2.169233315	up-regulated in High
BZW2	0.46513174	4.72658634	8.42718877	3.87E-16	1.58E-14	25.0345965	15.41241589	up-regulated in Low
TSPAN13	-0.1696413	6.61478473	-2.1905761	0.0289476	0.08413796	-5.7987708	1.53838751	up-regulated in High
AGR2	-0.398392	6.92059531	-2.3381969	0.01977387	0.06150968	-5.4675122	1.703908391	up-regulated in High
AGR3	-1.4338516	4.76776083	-7.5771098	1.75E-13	4.89E-12	19.0051874	12.75736084	up-regulated in High
AHR	-0.5866164	4.85944041	-6.6919659	5.97E-11	1.17E-09	13.2733554	10.22400938	up-regulated in High

SNX13	-0.1080908	2.03149172	-2.6570041	0.00813825	0.02919439	-4.6802126	2.089469084	up-regulated in High
TWIST1	0.23520751	0.97496089	2.8339036	0.00478593	0.01860393	-4.2012289	2.32003393	up-regulated in Low
TWISTNB	0.23559936	2.93462884	4.93811776	1.08E-06	1.02E-05	3.72726543	5.966608158	up-regulated in Low
MACC1	-0.6928862	2.73770237	-7.3458186	8.47E-13	2.16E-11	17.4519241	12.07191995	up-regulated in High
ITGB8	-0.2291217	1.46313506	-2.2548017	0.02458148	0.07371163	-5.6572495	1.609391906	up-regulated in High
SP4	-0.1250396	1.70885831	-2.5683589	0.01050996	0.0362696	-4.9089524	1.978398765	up-regulated in High
DNAH11	-0.2096191	0.80631315	-3.0852334	0.00214759	0.00929928	-3.4694304	2.668047996	up-regulated in High
RAPGEF5	-0.6081189	2.39834855	-9.6959201	1.80E-20	1.24E-18	34.9027274	19.74409095	up-regulated in High
STEAP1B	0.35373556	0.57963183	5.41949082	9.35E-08	1.08E-06	6.09485101	7.029342412	up-regulated in Low
IL6	0.30416998	1.78964738	2.81693346	0.00504196	0.01946605	-4.2484754	2.297400315	up-regulated in Low
KLHL7	0.12405201	2.48703071	2.53394975	0.01158543	0.03944019	-4.9957057	1.936087677	up-regulated in Low
NUPL2	0.15876908	2.80164005	3.64008219	0.00030119	0.00165802	-1.643286	3.521163007	up-regulated in Low
GPNMB	-0.5439936	5.23838582	-4.7731449	2.39E-06	2.10E-05	2.96167077	5.621478962	up-regulated in High
MALSU1	0.20389314	2.78401954	5.22341104	2.59E-07	2.76E-06	5.10663859	6.586517955	up-regulated in Low
IGF2BP3	0.82248987	1.01139188	9.46505844	1.19E-19	7.48E-18	33.0345396	18.92504164	up-regulated in Low
MPP6	0.3345354	1.26260533	6.79699548	3.08E-11	6.33E-10	13.9229404	10.51171108	up-regulated in Low
CYCS	0.68428659	4.76615944	12.4065594	5.71E-31	1.00E-28	58.9089024	30.24299124	up-regulated in Low
HNRNPA2B1	0.27952932	6.49587277	7.28257757	1.30E-12	3.23E-11	17.0339444	11.88734674	up-regulated in Low
CBX3	0.45348421	5.50264353	9.54496507	6.21E-20	4.03E-18	33.6776577	19.20704245	up-regulated in Low
HOXA1	0.35758037	0.60698288	5.46099001	7.50E-08	8.85E-07	6.30815202	7.1247988	up-regulated in Low
HOXA3	0.27951329	1.21238393	3.42071868	0.00067616	0.00337167	-2.3996482	3.169951413	up-regulated in Low
HOXA5	-0.1808182	1.8803326	-2.714949	0.00685987	0.02531938	-4.5266156	2.163684038	up-regulated in High
HOXA9	0.21701842	0.22369648	4.79375969	2.17E-06	1.92E-05	3.0560443	5.664067922	up-regulated in Low
HOXA10	0.43415891	0.69632834	5.46191405	7.47E-08	8.81E-07	6.31291793	7.126931161	up-regulated in Low
HOXA11	0.33239178	0.21560156	7.31867629	1.02E-12	2.56E-11	17.2721758	11.9925526	up-regulated in Low
HOXA13	0.13632794	0.13610178	3.62176135	0.00032275	0.00176099	-1.7081641	3.491135463	up-regulated in Low
HIBADH	0.17098336	4.56162693	3.20913588	0.00141761	0.00648568	-3.0866537	2.848442187	up-regulated in Low
JAZF1	-0.2967069	2.02920193	-6.0379495	3.06E-09	4.61E-08	9.42124052	8.513743339	up-regulated in High
TRIL	-0.2468202	1.43416921	-2.9641564	0.00318128	0.01307053	-3.8294589	2.497398496	up-regulated in High
CPVL	-0.5309012	3.26237037	-5.3034035	1.72E-07	1.89E-06	5.50586529	6.765533142	up-regulated in High
PRR15	0.46693106	1.9058827	3.42914603	0.00065598	0.00328283	-2.3714175	3.183108191	up-regulated in Low
WIPF3	0.16614725	0.70216585	3.06929652	0.00226325	0.00973438	-3.5176127	2.645267965	up-regulated in Low
PLEKHA8	0.11467722	1.5717292	2.78585091	0.00554334	0.02113624	-4.3342995	2.25622817	up-regulated in Low
MTURN	-0.5936035	2.60016566	-7.9506283	1.26E-14	4.16E-13	21.5935355	13.89814485	up-regulated in High
NOD1	-0.5640511	2.15182768	-10.174517	3.29E-22	2.75E-20	38.8719893	21.48305394	up-regulated in High
GGCT	0.30553884	5.00743487	4.51405718	7.95E-06	6.29E-05	1.8073572	5.09940474	up-regulated in Low
GARS	0.52037189	5.01680657	10.1265043	4.94E-22	4.09E-20	38.4680779	21.30616822	up-regulated in Low
INMT	-1.3662319	2.565962	-12.778437	1.61E-32	3.11E-30	62.460059	31.79322915	up-regulated in High
AQP1	-1.6677055	5.94242389	-10.917966	5.15E-25	5.76E-23	45.2812883	24.28803231	up-regulated in High
CCDC129	-0.1555981	0.38743088	-2.2606174	0.02421597	0.07278003	-5.6442366	1.615898196	up-regulated in High
LSM5	0.44334651	3.08488787	10.0722835	7.82E-22	6.32E-20	38.0134497	21.10705362	up-regulated in Low
AVL9	0.20705924	2.67515563	4.40962827	1.27E-05	9.64E-05	1.3588581	4.895919503	up-regulated in Low
KBTBD2	0.11560492	3.66751949	3.10252069	0.0020283	0.00884704	-3.4168936	2.692867451	up-regulated in Low
FKBP9	0.17092882	4.61280286	2.9612667	0.00321075	0.01317585	-3.8378816	2.493393735	up-regulated in Low
NT5C3A	0.44463274	3.32452296	8.87739191	1.26E-17	6.11E-16	28.422299	16.901062	up-regulated in Low
RP9	0.12539854	2.42624779	3.15400416	0.00170823	0.00761535	-3.2587619	2.767454429	up-regulated in Low
BBS9	-0.2038585	1.84125975	-4.9342834	1.10E-06	1.04E-05	3.7092032	5.958475085	up-regulated in High
BMPER	-0.2227475	0.44347693	-4.2772817	2.27E-05	0.00016355	0.80441984	4.64380521	up-regulated in High
KIAA0895	0.12158444	1.58642526	2.34108177	0.01962344	0.06113665	-5.4608281	1.707224879	up-regulated in Low
ANLN	1.4091756	2.76457198	15.5853675	7.42E-45	2.89E-42	90.7532859	44.12974411	up-regulated in Low
AOAH	-0.2119688	1.97855869	-2.7098952	0.00696369	0.02562967	-4.54014	2.157160511	up-regulated in High
ELMO1	-0.3553802	1.72867381	-5.8820661	7.46E-09	1.04E-07	8.55327818	8.127209635	up-regulated in High
EPDR1	-1.0642452	4.02951908	-10.297106	1.16E-22	1.02E-20	39.9089324	21.93709612	up-regulated in High
SFRP4	-0.7772134	3.71016852	-5.7241234	1.80E-08	2.36E-07	7.69401461	7.744032542	up-regulated in High
VPS41	-0.1079299	3.40097159	-2.4780374	0.01354279	0.04498623	-5.1342412	1.868291822	up-regulated in High
POU6F2	0.15524471	0.43370511	2.40783146	0.01641194	0.05273189	-5.3039208	1.784840108	up-regulated in Low
YAE1D1	0.14322447	2.44494194	3.03002329	0.0025731	0.01087641	-3.6353228	2.589542715	up-regulated in Low
RALA	0.3037755	4.45456203	6.96720512	1.03E-11	2.27E-10	14.9934085	10.98545117	up-regulated in Low
MPLKIP	0.27482553	2.11295933	7.66963848	9.20E-14	2.70E-12	19.6372669	13.03609896	up-regulated in Low
SUGCT	-0.1693602	1.18458438	-2.4207563	0.01584662	0.05125963	-5.2730405	1.800063489	up-regulated in High
PSMA2	0.23371258	2.15492437	6.1904429	1.26E-09	2.01E-08	10.2892488	8.899820688	up-regulated in Low
PSMA2	0.23371258	2.15492437	6.1904429	1.26E-09	2.01E-08	10.2892488	8.899820688	up-regulated in Low
MRPL32	0.20291984	3.54123457	4.45445636	1.04E-05	8.03E-05	1.55019821	4.982778553	up-regulated in Low
STK17A	-0.2006524	3.52432357	-2.9101462	0.00377511	0.01518521	-3.985575	2.423070915	up-regulated in High
COA1	0.20517618	2.3604744	5.17885606	3.25E-07	3.41E-06	4.88662965	6.487791906	up-regulated in Low
BLVRA	-0.3242274	5.29494764	-5.3849323	1.12E-07	1.28E-06	5.91832719	6.950312145	up-regulated in High
MRPS24	0.35626914	2.23532453	6.64094677	8.21E-11	1.58E-09	12.9608581	10.08553978	up-regulated in Low
DBNL	-0.1308281	3.69847379	-3.1454046	0.00175822	0.00781081	-3.2853494	2.754925836	up-regulated in High
AEBP1	-0.5260394	5.92886695	-4.7864343	2.24E-06	1.98E-05	3.02246651	5.648916436	up-regulated in High
POLD2	0.52613425	4.68050553	10.5754094	1.05E-23	1.02E-21	42.292598	22.98046876	up-regulated in Low
YKT6	0.40040051	5.08374287	8.15299089	2.93E-15	1.05E-13	23.0360687	14.53324202	up-regulated in Low

CAMK2B	0.11641972	0.28932443	2.18463186	0.02938365	0.08522293	-5.8116662	1.531894333	up-regulated in Low
DDX56	0.33549415	4.40925178	8.13678626	3.30E-15	1.18E-13	22.9195313	14.48195129	up-regulated in Low
PPIA	0.39877365	6.35176014	8.59525814	1.09E-16	4.76E-15	26.284057	15.96168715	up-regulated in Low
H2AFV	0.2531499	4.97406398	6.26513213	8.10E-10	1.33E-08	10.7211522	9.091760613	up-regulated in Low
MYO1G	-0.4861466	2.26103648	-4.5954421	5.49E-06	4.48E-05	2.16358693	5.260760594	up-regulated in High
TBRG4	0.55181971	3.58066109	12.1821346	4.80E-30	7.81E-28	56.7924746	29.31881108	up-regulated in Low
RAMP3	-0.601774	3.13153206	-7.5902147	1.60E-13	4.49E-12	19.0943409	12.79668273	up-regulated in High
IGFBP1	0.46489297	0.44296243	4.85393276	1.62E-06	1.48E-05	3.33363087	5.789261688	up-regulated in Low
IGFBP3	0.29069487	5.73748731	2.23960701	0.02555917	0.0760652	-5.6910929	1.592453277	up-regulated in Low
TNS3	-0.153416	3.73211216	-2.295751	0.02210679	0.06740556	-5.5649261	1.655474352	up-regulated in High
SUN3	0.14472372	0.35937276	1.9855871	0.04762987	0.12564222	-6.223595	1.322120647	up-regulated in Low
C7orf57	-0.3879452	0.91282465	-5.0769213	5.44E-07	5.45E-06	4.38965389	6.264579355	up-regulated in High
ABCA13	-0.2266034	0.59235918	-3.4440298	0.00062171	0.00313363	-2.3213967	3.206410323	up-regulated in High
IKZF1	-0.4357186	1.67914263	-6.7318381	4.65E-11	9.31E-10	13.5189654	10.33281109	up-regulated in High
FIGNL1	0.43088403	2.10348108	7.55107012	2.09E-13	5.80E-12	18.8284011	12.67938127	up-regulated in Low
DDC	0.59946996	0.95842295	4.60384093	5.28E-06	4.32E-05	2.20068222	5.277550203	up-regulated in Low
GRB10	0.28468669	2.64397394	3.77005677	0.00018289	0.00106697	-1.174124	3.737817264	up-regulated in Low
COBL	-0.1514566	1.59425771	-2.0698139	0.03898735	0.10702687	-6.0540016	1.409076273	up-regulated in High
SEC61G	0.49028536	5.15535579	8.43620089	3.62E-16	1.48E-14	25.101127	15.44167023	up-regulated in Low
LANCL2	0.17999107	2.3463124	3.69012256	0.00024899	0.00140267	-1.4645018	3.603822621	up-regulated in Low
VOPP1	0.18808048	3.4599799	3.00789924	0.00276434	0.01156254	-3.7009901	2.558408581	up-regulated in Low
MRPS17	0.26998073	2.59245666	5.86090963	8.41E-09	1.17E-07	8.43699739	8.075387017	up-regulated in Low
GBAS	0.14297657	4.00457838	2.26076488	0.02420676	0.07275986	-5.6439062	1.616063309	up-regulated in Low
MRPS17	0.26998073	2.59245666	5.86090963	8.41E-09	1.17E-07	8.43699739	8.075387017	up-regulated in Low
PSPH	0.56934637	2.81419339	7.86928646	2.26E-14	7.14E-13	21.0215882	13.64620725	up-regulated in Low
CCT6A	0.65641976	5.78251288	11.4829259	3.18E-27	4.22E-25	50.3357339	26.49795448	up-regulated in Low
CHCHD2	0.55170413	7.50595071	8.7227476	4.14E-17	1.87E-15	27.2440069	16.38350486	up-regulated in Low
ZNF680	0.1129623	1.88769879	2.18116459	0.0296406	0.0858298	-5.8191722	1.528113002	up-regulated in Low
ZNF138	0.13963635	2.14813713	2.89684466	0.00393608	0.01573328	-4.0235973	2.404935752	up-regulated in Low
ZNF117	-0.4783951	2.32753383	-5.3220192	1.56E-07	1.73E-06	5.59954987	6.807517841	up-regulated in High
ERV3-1	-0.3904962	2.82081734	-3.7162769	0.00022522	0.0012849	-1.3701382	3.647401035	up-regulated in High
ZNF92	0.12044037	2.16302893	2.45173838	0.01456088	0.04773263	-5.1983605	1.836812496	up-regulated in Low
VKORC1L1	-0.2270965	4.22143964	-4.3247495	1.85E-05	0.00013556	1.00147517	4.733485561	up-regulated in High
GUSB	-0.1815083	4.97285316	-3.339779	0.00090166	0.00434187	-2.6674101	3.044957195	up-regulated in High
CRCP	0.11372071	3.920694	2.79431768	0.00540248	0.02067339	-4.3110128	2.267406939	up-regulated in Low
KCTD7	-0.2426837	1.56767978	-6.2311632	9.90E-10	1.61E-08	10.5241721	9.004234744	up-regulated in High
AUTS2	-0.8706215	2.55575114	-9.1249189	1.81E-18	9.88E-17	30.3393828	17.74268289	up-regulated in High
WBSCR17	-0.3939167	0.89765528	-5.8314	9.92E-09	1.36E-07	8.2754147	8.003358927	up-regulated in High
POM121	0.13935791	2.29991233	3.00743864	0.00276846	0.0115781	-3.7023522	2.557762381	up-regulated in Low
NSUN5	0.27662803	3.35832592	5.62731304	3.07E-08	3.86E-07	7.17746082	7.513412088	up-regulated in Low
BAZ1B	0.20463216	4.11691312	4.11670732	4.50E-05	0.00030453	0.15282738	4.346621208	up-regulated in Low
TBL2	0.18154573	3.72814737	4.28984103	2.15E-05	0.00015567	0.85636177	4.667452443	up-regulated in Low
VPS37D	0.17902715	1.60098673	2.56338401	0.01065973	0.0367105	-4.9215656	1.972253704	up-regulated in Low
WBSCR22	0.20036194	3.75549059	4.68223555	3.67E-06	3.10E-05	2.5499248	5.435506176	up-regulated in Low
STX1A	0.33765997	2.28101411	3.85816801	0.00012933	0.00078446	-0.8472386	3.888300047	up-regulated in Low
CLDN4	-0.2305861	6.44466971	-2.6760341	0.00769659	0.02787029	-4.6301239	2.113701902	up-regulated in High
WBSCR28	0.2320361	0.4843622	4.83658577	1.77E-06	1.60E-05	3.25328358	5.753035853	up-regulated in Low
ELN	-1.431324	3.61787552	-12.966291	2.60E-33	5.16E-31	64.2740592	32.58492135	up-regulated in High
EIF4H	0.2200728	6.03531299	5.64915339	2.72E-08	3.45E-07	7.2933189	7.565156607	up-regulated in Low
LAT2	-0.3098052	2.29805051	-4.5682989	6.21E-06	5.02E-05	2.04412819	5.206676414	up-regulated in High
RFC2	0.62375392	3.95190905	12.7788723	1.60E-32	3.11E-30	62.4642479	31.79505746	up-regulated in Low
GTF2IRD1	0.30525824	2.63732774	6.41218903	3.35E-10	5.87E-09	11.5844149	9.475094697	up-regulated in Low
NCF1	-0.1287915	1.2203642	-2.1752594	0.03008269	0.08687927	-5.831929	1.521683309	up-regulated in High
GTF2IRD2	-0.1530229	0.48366882	-7.4558052	4.02E-13	1.07E-11	18.1857608	12.39584225	up-regulated in High
GATSL2	0.2876053	1.04723814	6.54491278	1.49E-10	2.76E-09	12.378076	9.827188327	up-regulated in Low
WBSCR16	0.19251877	3.95202096	5.08348867	5.26E-07	5.29E-06	4.42140487	6.278848675	up-regulated in Low
GTF2IRD2B	-0.2118531	0.87197032	-7.5658926	1.89E-13	5.26E-12	18.9289737	12.72374438	up-regulated in High
HIP1	-0.6077211	3.35332222	-8.0713798	5.30E-15	1.83E-13	22.4509573	14.27569233	up-regulated in High
CCL26	0.33628382	0.70509336	5.64908166	2.72E-08	3.45E-07	7.29293775	7.564986395	up-regulated in Low
RHBDD2	-0.1526097	5.62739753	-3.0630455	0.00231015	0.00990333	-3.5364459	2.636359151	up-regulated in High
TMEM120A	-0.1158046	4.31590556	-2.2321282	0.02605266	0.07726604	-5.7076684	1.584147967	up-regulated in High
STYXL1	0.15833836	4.27539429	2.9265094	0.00358532	0.01451805	-3.93857	2.445472605	up-regulated in Low
MDH2	0.45010769	5.83886715	10.2079539	2.47E-22	2.10E-20	39.1540204	21.60655543	up-regulated in Low
YWHAG	0.55660143	6.00615762	11.3085166	1.55E-26	1.94E-24	48.7592431	25.808852	up-regulated in Low
ZP3	0.57951066	1.63784014	8.43477353	3.66E-16	1.49E-14	25.0905862	15.43703534	up-regulated in Low
CCDC146	-0.4236751	1.2721623	-6.1540197	1.56E-09	2.46E-08	10.0802343	8.806895319	up-regulated in High
FGL2	-0.5594567	3.17235059	-5.6975537	2.09E-08	2.70E-07	7.5514762	7.680415968	up-regulated in High
GSAP	-0.4462118	2.17370421	-7.7928321	3.88E-14	1.19E-12	20.4881809	13.41117627	up-regulated in High
RSBN1L	-0.10546	2.76072415	-2.7266188	0.00662545	0.02457219	-4.4952927	2.178784783	up-regulated in High
PHTF2	0.13641269	2.31914163	2.73325813	0.00649533	0.02417252	-4.4774141	2.187399066	up-regulated in Low
MAGI2	-0.1807511	0.76223447	-5.4004051	1.03E-07	1.19E-06	5.99723765	6.985644282	up-regulated in High

CD36	-0.4384377	1.49247348	-4.938829	1.08E-06	1.02E-05	3.73061735	5.968117418	up-regulated in High
HGF	-0.3209342	1.16500355	-5.0728152	5.55E-07	5.55E-06	4.36982135	6.255665728	up-regulated in High
SEMA3A	0.35064575	1.42922811	3.43157713	0.00065027	0.00325702	-2.3632613	3.186908613	up-regulated in Low
KIAA1324L	-0.2486376	1.59394551	-3.6663168	0.00027266	0.00151862	-1.5498434	3.56438127	up-regulated in High
TMEM243	-0.7091903	3.21454526	-10.862382	8.43E-25	9.22E-23	44.792331	24.07415719	up-regulated in High
CROT	-0.300265	1.95997511	-6.1856157	1.30E-09	2.07E-08	10.2614873	8.887479702	up-regulated in High
SLC25A40	0.18409452	2.34626023	4.5701954	6.16E-06	4.98E-05	2.05245354	5.210446477	up-regulated in Low
DBF4	0.72402268	1.95811582	15.2201165	3.38E-43	1.23E-40	86.9454692	42.47063655	up-regulated in Low
STEAP4	-0.9023079	3.70055503	-6.973619	9.92E-12	2.19E-10	15.034172	11.00348257	up-regulated in High
STEAP1	0.69277576	3.44100675	5.46378023	7.39E-08	8.73E-07	6.32254522	7.131238525	up-regulated in Low
STEAP2	0.29591238	2.14174649	3.16327178	0.00165582	0.00741451	-3.2300308	2.780987801	up-regulated in Low
CFAP69	-0.1056441	0.55845497	-3.4485089	0.00061173	0.00308892	-2.3063029	3.213439288	up-regulated in High
GTPBP10	0.10452793	1.66908053	3.19751725	0.00147476	0.00671032	-3.1231624	2.831278664	up-regulated in Low
CLDN12	0.13505253	3.49344692	2.24835411	0.0249923	0.07471228	-5.6716377	1.602193715	up-regulated in Low
CDK14	-0.1853575	1.93219292	-2.7933144	0.005419	0.02072574	-4.3137758	2.266080838	up-regulated in High
FZD1	-0.3766672	2.95726796	-4.83413	1.79E-06	1.62E-05	3.24192993	5.74791614	up-regulated in High
MTERF1	0.20842592	1.64286699	6.19126472	1.25E-09	2.00E-08	10.293977	8.901922506	up-regulated in Low
CYP51A1	0.11174625	1.28336934	2.46685813	0.01396759	0.04612441	-5.1615789	1.854878475	up-regulated in Low
KRIT1	0.11205724	2.65209346	2.83330155	0.00479481	0.0186338	-4.2029098	2.319229094	up-regulated in Low
ANKIB1	0.28996942	3.37262362	6.0350392	3.12E-09	4.67E-08	9.40485613	8.50645136	up-regulated in Low
PEX1	0.10963482	2.16125934	2.53194434	0.01165103	0.03963116	-5.0007267	1.933635556	up-regulated in Low
CDK6	0.4303945	1.64224711	5.86656492	8.14E-09	1.13E-07	8.46804444	8.089224622	up-regulated in Low
HEPACAM2	0.48154426	0.45008618	4.84587965	1.69E-06	1.54E-05	3.29629817	5.772430798	up-regulated in Low
CCDC132	0.11257842	1.85131598	3.28392877	0.00109609	0.00515733	-2.8485977	2.960154846	up-regulated in Low
GNGT1	0.2119941	0.39462765	4.88771047	1.38E-06	1.28E-05	3.49083154	5.860111757	up-regulated in Low
GNG11	-0.2423109	3.28990548	-2.4155239	0.01607337	0.05185524	-5.2855613	1.793892942	up-regulated in High
CASD1	-0.2833367	3.00332301	-5.490524	6.41E-08	7.67E-07	6.46083177	7.193100309	up-regulated in High
PPP1R9A	-0.1773099	1.7206867	-3.2868077	0.00108518	0.00511467	-2.8393293	2.964497322	up-regulated in High
PON1	-0.2070464	0.59073304	-3.3289594	0.00093662	0.00448949	-2.7027391	3.028436458	up-regulated in High
PON3	-0.7211188	3.19441288	-6.0730649	2.50E-09	3.82E-08	9.61947025	8.601953413	up-regulated in High
PON2	-0.313086	5.6020794	-3.7796216	0.00017619	0.00103225	-1.1389839	3.754011825	up-regulated in High
PDK4	-0.4401315	2.93271523	-3.7870817	0.00017113	0.00100579	-1.1115181	3.766666566	up-regulated in High
DYNC1I1	0.20536574	0.57914028	4.0731665	5.40E-05	0.00035879	-0.0198468	4.267688519	up-regulated in Low
SLC25A13	0.43155117	3.40479294	8.24752647	1.47E-15	5.49E-14	23.7194498	14.83395724	up-regulated in Low
SHFM1	0.42914153	3.11071591	8.67227821	6.08E-17	2.71E-15	26.8627455	16.21599074	up-regulated in Low
DLX6	0.27043336	0.28639516	5.78514695	1.29E-08	1.73E-07	8.02358367	7.891063612	up-regulated in Low
DLX5	0.145303	0.57322637	2.18972365	0.02900978	0.08429772	-5.8006222	1.537455528	up-regulated in Low
ACN9	0.37561874	2.9054477	6.59907548	1.07E-10	2.02E-09	12.7058871	9.972528395	up-regulated in Low
TAC1	0.15417873	0.1588866	2.20275334	0.02807173	0.08204487	-5.7722462	1.551730788	up-regulated in Low
ASNS	0.72428372	3.14025768	9.93962447	2.38E-21	1.84E-19	36.9079528	20.62279322	up-regulated in Low
LMTK2	0.15933782	2.88321572	2.85813629	0.00444075	0.01744598	-4.1332866	2.35254349	up-regulated in Low
BHLHA15	0.41510048	1.87274567	3.98090701	7.89E-05	0.00050469	-0.3800496	4.102771036	up-regulated in Low
BAIAP2L1	0.24038712	4.26413421	3.76462824	0.00018679	0.00108756	-1.1940304	3.728641327	up-regulated in Low
NPTX2	0.26277303	1.06711095	2.54708366	0.01116388	0.03821455	-4.9627266	1.952184814	up-regulated in Low
TMEM130	-1.04479	1.62521744	-9.6844767	1.98E-20	1.35E-18	34.8093987	19.70318324	up-regulated in High
TRRAP	0.23951467	3.05171147	4.0776442	5.30E-05	0.00035281	-0.0021683	4.275773327	up-regulated in Low
ARPC1A	0.45303067	5.81869159	8.91287085	9.53E-18	4.70E-16	28.694748	17.02070291	up-regulated in Low
ARPC1B	0.15172208	6.10995592	2.4879092	0.01317724	0.04401232	-5.1100008	1.880175652	up-regulated in Low
PDAP1	0.38627936	5.32642311	8.80729584	2.16E-17	1.02E-15	27.88634	16.66567253	up-regulated in Low
BUD31	0.35720778	4.38517015	7.26936952	1.42E-12	3.51E-11	16.947016	11.84895368	up-regulated in Low
PTCD1	0.246103	1.25568016	7.73081837	6.00E-14	1.80E-12	20.0585147	13.22180512	up-regulated in Low
CPSF4	0.37827006	2.98247587	7.85421727	2.51E-14	7.89E-13	20.9161325	13.59974656	up-regulated in Low
ATP5J2	0.47745007	5.3570729	7.87455368	2.18E-14	6.89E-13	21.0584859	13.66246265	up-regulated in Low
ZKSCAN5	0.16477039	1.8902621	5.05104546	6.19E-07	6.14E-06	4.26491415	6.20850786	up-regulated in Low
ZNF655	-0.1372848	3.12069148	-2.7791151	0.00565778	0.02150095	-4.3527766	2.24735421	up-regulated in High
TRIM4	-0.1773192	3.10838438	-4.077469	5.30E-05	0.00035303	-0.0028603	4.275456871	up-regulated in High
GJC3	0.12289444	0.39109515	2.40106806	0.01671479	0.05352821	-5.3200156	1.7768991	up-regulated in Low
ZKSCAN1	0.29488281	3.00721382	4.82027594	1.91E-06	1.72E-05	3.17797893	5.71907517	up-regulated in Low
ZSCAN21	0.21457232	1.77666552	5.26298553	2.11E-07	2.29E-06	5.3034692	6.674798675	up-regulated in Low
ZNF3	0.16307571	2.89747216	3.91600984	0.00010264	0.00063905	-0.6287848	3.988669069	up-regulated in Low
COPS6	0.43432882	5.14080776	9.74274059	1.23E-20	8.61E-19	35.2853599	19.91179586	up-regulated in Low
MCM7	1.00241716	4.72252761	16.216701	9.38E-48	4.35E-45	97.405904	47.0276982	up-regulated in Low
AP4M1	0.20477806	2.57191078	5.07992376	5.36E-07	5.37E-06	4.40416526	6.27110111	up-regulated in Low
TAF6	0.39808631	3.48917874	8.99078444	5.19E-18	2.66E-16	29.2958133	17.28461042	up-regulated in Low
MBLAC1	0.14311196	0.9649134	3.80671632	0.00015846	0.00093892	-1.0389852	3.800073194	up-regulated in Low
LAMTOR4	0.13656086	4.92054664	2.19670554	0.02850382	0.08307141	-5.7854376	1.545096909	up-regulated in Low
GAL3ST4	-0.2125551	2.09898753	-3.0980809	0.00205834	0.00896334	-3.4304131	2.686482461	up-regulated in High
GPC2	0.31896365	0.63067064	5.14110792	3.94E-07	4.05E-06	4.70155596	6.404700857	up-regulated in Low
SPDYE3	0.18113206	1.39507947	5.38688102	1.11E-07	1.27E-06	5.92825446	6.954757409	up-regulated in Low
PILRA	-0.2745412	2.55327333	-3.5644129	0.00039998	0.00212732	-1.9092394	3.39796033	up-regulated in High
ZCWPW1	-0.2652169	1.42033722	-5.3756603	1.18E-07	1.34E-06	5.87113697	6.929179874	up-regulated in High

MEPCE	0.2123189	4.08941212	4.87778289	1.45E-06	1.34E-05	3.44452607	5.839245542	up-regulated in Low
PPP1R35	0.36201423	3.96570123	5.43925487	8.42E-08	9.84E-07	6.1962559	7.074728363	up-regulated in Low
AGFG2	-0.1189251	2.63566687	-2.0966268	0.03653299	0.10157388	-5.9985599	1.43731483	up-regulated in High
LRCH4	-0.2627985	2.12235005	-5.5473778	4.73E-08	5.78E-07	6.75679458	7.325440414	up-regulated in High
PCOLCE	-0.2327529	4.23581251	-2.4890311	0.01313625	0.04389551	-5.10724	1.881528546	up-regulated in High
TFR2	0.22546034	0.70452444	4.81893393	1.92E-06	1.73E-05	3.17179299	5.716285091	up-regulated in Low
ACTL6B	0.11485671	0.07068919	3.10766608	0.00199399	0.00871496	-3.401202	2.700276621	up-regulated in Low
GNB2	0.1862832	6.26948075	3.88025611	0.00011845	0.00072484	-0.7641784	3.92648015	up-regulated in Low
POP7	0.60999942	4.76688657	11.2716603	2.17E-26	2.66E-24	48.4279125	25.664004	up-regulated in Low
SLC12A9	-0.1478349	2.85643391	-2.6662602	0.00792066	0.02854185	-4.6558932	2.101238608	up-regulated in High
TRIP6	-0.3365661	4.66154427	-3.8356777	0.00014138	0.00084934	-0.9313524	3.849612606	up-regulated in High
SRRT	0.33296535	4.51449521	7.49418846	3.09E-13	8.35E-12	18.4439064	12.50975261	up-regulated in Low
UFSP1	0.12782936	1.45889924	2.43272039	0.0153387	0.0498764	-5.2443119	1.814211479	up-regulated in Low
ACHE	-0.5970911	1.89988057	-4.8012291	2.09E-06	1.86E-05	3.09033018	5.679537207	up-regulated in High
MUC3A	-0.608549	1.77155574	-4.2372588	2.70E-05	0.00019095	0.63984085	4.568838101	up-regulated in High
TRIM56	-0.1676829	3.10270995	-2.8147349	0.00507603	0.01958242	-4.2545763	2.294476053	up-regulated in High
AP1S1	0.25337992	5.25080517	4.47298854	9.57E-06	7.45E-05	1.62982113	5.018902387	up-regulated in Low
VGF	0.43777082	0.54832763	4.63818686	4.50E-06	3.74E-05	2.35302566	5.34647735	up-regulated in Low
PLOD3	0.36062055	4.03726796	6.88286687	1.78E-11	3.78E-10	14.4602643	10.74956176	up-regulated in Low
ZNHIT1	0.16309242	3.93736537	2.81392679	0.0050886	0.01962405	-4.2568176	2.293401632	up-regulated in Low
COL26A1	0.12903845	0.42391598	2.92209487	0.00363564	0.01468822	-3.9512763	2.43941897	up-regulated in Low
CUX1	-0.1146893	3.19430788	-2.6161044	0.00916532	0.03234619	-4.786688	2.037852447	up-regulated in High
SH2B2	0.13946642	1.78761165	2.61169767	0.00928263	0.03270289	-4.7980643	2.032328895	up-regulated in Low
PRKRIP1	0.14407263	3.18051755	3.06699999	0.00228038	0.00979795	-3.524536	2.641993269	up-regulated in Low
ALKBH4	0.1670153	2.67173031	4.25594132	2.49E-05	0.0001776	0.71648674	4.603758664	up-regulated in Low
LRWD1	0.31761413	2.29885911	6.89407615	1.66E-11	3.54E-10	14.5308154	10.78078332	up-regulated in Low
POLR2J	0.36131071	4.73175809	6.82275391	2.61E-11	5.43E-10	14.0835326	10.58281059	up-regulated in Low
FBXL13	0.11990764	0.46009691	2.6851295	0.0074932	0.02721905	-4.6060614	2.125332528	up-regulated in Low
LRRC17	-0.3498595	1.30618128	-5.2053764	2.84E-07	3.01E-06	5.01738136	6.546471371	up-regulated in High
ARMC10	0.13947039	3.14528756	3.3347024	0.00091791	0.00441103	-2.6840003	3.037200051	up-regulated in Low
PMPCB	0.17451572	3.75834135	4.37388539	1.49E-05	0.00011147	1.20757868	4.827194015	up-regulated in Low
DNAJC2	0.38951182	2.54843824	9.40322561	1.96E-19	1.19E-17	32.5394612	18.70792093	up-regulated in Low
PSMC2	0.26802178	4.7823632	6.39013165	3.83E-10	6.64E-09	11.4538486	9.417141396	up-regulated in Low
SLC26A5	-0.1590099	0.33135757	-5.2319773	2.48E-07	2.66E-06	5.14913154	6.605580019	up-regulated in High
ORC5	0.38187985	2.57800091	9.06431789	2.92E-18	1.54E-16	29.8665359	17.53514686	up-regulated in Low
LHFPL3	-0.2799484	0.42499989	-4.9946746	8.18E-07	7.92E-06	3.99515861	6.087185087	up-regulated in High
KMT2E	-0.1810149	3.36301199	-4.0647039	5.59E-05	0.0003701	-0.0532086	4.252429059	up-regulated in High
SRPK2	0.22818164	3.05644507	5.42816306	8.93E-08	1.04E-06	6.139306	7.049240387	up-regulated in Low
PUS7	0.49778221	2.58516012	9.81611535	6.67E-21	4.89E-19	35.8875132	20.17568308	up-regulated in Low
RINT1	0.26603958	2.84107744	6.43853552	2.85E-10	5.06E-09	11.7408692	9.544527316	up-regulated in Low
EFCAB10	-0.1708819	0.69690326	-3.5565054	0.0004119	0.00218314	-1.9367253	3.385210318	up-regulated in High
CDHR3	-0.4644537	1.00335603	-4.5695467	6.18E-06	5.00E-05	2.0496053	5.20915669	up-regulated in High
NAMPT	0.64465215	4.4641056	6.36791645	4.38E-10	7.52E-09	11.3227337	9.358935807	up-regulated in Low
PIK3CG	-0.3667509	1.19435862	-6.399877	3.61E-10	6.28E-09	11.5114883	9.442726495	up-regulated in High
PRKAR2B	-0.3399772	2.02516132	-4.0790477	5.27E-05	0.00035092	0.00337648	4.278308933	up-regulated in High
HBP1	-0.1762489	3.32044162	-3.6149294	0.00033115	0.00180045	-1.7322781	3.479970395	up-regulated in High
COG5	0.17445925	2.53773542	4.00665697	7.10E-05	0.00045935	-0.2802935	4.148480624	up-regulated in Low
DUS4L	0.34687474	1.47277843	9.60684827	3.75E-20	2.48E-18	34.178275	19.42652462	up-regulated in Low
CBLL1	0.21868644	2.59263821	5.94191212	5.31E-09	7.63E-08	8.88417592	8.274629031	up-regulated in Low
DLD	0.39247142	3.81744868	8.98581925	5.40E-18	2.76E-16	29.2573969	17.26774463	up-regulated in Low
DNAJB9	-0.1850413	4.36076994	-2.9180242	0.00368262	0.01486258	-3.9629764	2.433843436	up-regulated in High
IMMP2L	0.10895373	1.75897978	2.40748074	0.01642752	0.05277325	-5.3047565	1.784427889	up-regulated in Low
LRRN3	-0.2688762	0.50803802	-6.3837255	3.98E-10	6.88E-09	11.4159996	9.400340094	up-regulated in High
DOCK4	-0.2789037	1.72744568	-5.4620501	7.46E-08	8.81E-07	6.3136196	7.127245099	up-regulated in High
IFRD1	0.57878614	2.64355806	9.01741606	4.22E-18	2.19E-16	29.5021268	17.3751835	up-regulated in Low
C7orf60	-0.1169918	2.2517012	-2.1675507	0.03066834	0.08824472	-5.8485307	1.513309702	up-regulated in High
GPR85	-0.1393224	0.38113928	-4.7257441	2.99E-06	2.58E-05	2.74608075	5.524136676	up-regulated in High
FOXP2	0.10931038	0.56278066	2.65371023	0.00821696	0.02944059	-4.688847	2.085288655	up-regulated in Low
MDFIC	-0.1874675	3.43366032	-2.4993608	0.01276417	0.04283087	-5.0817632	1.894007568	up-regulated in High
TFEC	-0.1923639	1.07371133	-3.456033	0.0005953	0.00301377	-2.2809063	3.225263649	up-regulated in High
TES	0.14361369	4.77290075	2.43910422	0.01507361	0.04918149	-5.2289262	1.821782775	up-regulated in Low
CAV2	-0.3922579	3.36102844	-3.910713	0.00010485	0.00065072	-0.6489166	3.979425777	up-regulated in High
CAV1	-0.4039515	4.46401456	-3.3184218	0.00097188	0.00464323	-2.7370417	3.012389034	up-regulated in High
ST7	-0.1353228	2.79376927	-3.1087632	0.00198675	0.00869045	-3.3978531	2.701857699	up-regulated in High
WNT2	-0.3296018	1.79266674	-3.970713	8.23E-05	0.00052396	-0.4193747	4.08474379	up-regulated in High
CFTR	-0.6760962	1.29452636	-7.0664202	5.42E-12	1.24E-10	15.6274143	11.26582944	up-regulated in High
CTTNBP2	-0.1179635	0.40979497	-3.4389315	0.00063326	0.00318221	-2.3385541	3.198418939	up-regulated in High
LSM8	0.26523387	2.47888128	6.94858967	1.17E-11	2.55E-10	14.8752714	10.93319076	up-regulated in Low
KCND2	0.20724906	0.56363919	4.33748516	1.75E-05	0.00012893	1.05468872	4.757688643	up-regulated in Low
TSPAN12	-0.3225089	3.58302104	-3.9010215	0.00010901	0.00067308	-0.6856855	3.962540517	up-regulated in High
CPED1	-0.4258967	1.12296253	-8.0777547	5.06E-15	1.75E-13	22.4964995	14.29574138	up-regulated in High

WNT16	0.18940452	0.27952997	2.60682726	0.00941385	0.03310326	-4.810616	2.026232708	up-regulated in Low
PTPRZ1	-0.2914375	0.67836008	-3.3667904	0.00081961	0.00399119	-2.5787327	3.086394839	up-regulated in High
AASS	-0.4484948	1.7642805	-6.1442967	1.65E-09	2.59E-08	10.0246175	8.782164462	up-regulated in High
FEZF1	-0.3284264	0.89065589	-3.5283532	0.00045708	0.00239328	-2.0341103	3.340009034	up-regulated in High
CADPS2	-0.4120679	3.41678245	-5.8949375	6.94E-09	9.76E-08	8.62420074	8.158812913	up-regulated in High
GPR37	0.32840677	1.39791547	3.24899272	0.00123678	0.00574201	-2.9604487	2.907709427	up-regulated in Low
POT1	0.1754574	2.30467443	3.67811397	0.00026068	0.00146024	-1.5076169	3.583900136	up-regulated in Low
ARF5	0.18440304	6.08572515	3.5641212	0.00040042	0.00212924	-1.9102542	3.397489645	up-regulated in Low
SND1	0.22193476	5.35751033	5.69701483	2.09E-08	2.71E-07	7.54859119	7.679128193	up-regulated in Low
LRRC4	-0.5515398	1.41784605	-6.443564	2.77E-10	4.93E-09	11.7707918	9.557805243	up-regulated in High
LEP	0.11260825	0.17577783	2.79614429	0.00537252	0.02057357	-4.3059799	2.269822212	up-regulated in Low
RBM28	0.32684482	1.68143739	9.43140785	1.56E-19	9.64E-18	32.7648288	18.80676144	up-regulated in Low
HILPDA	0.96794625	3.41151298	9.98847199	1.58E-21	1.24E-19	37.3138853	20.80062502	up-regulated in Low
METTL2B	0.18621869	2.3092338	5.04641571	6.33E-07	6.26E-06	4.24265606	6.198500681	up-regulated in Low
CALU	0.45932214	5.87627936	6.86363619	2.01E-11	4.25E-10	14.3394473	10.69609115	up-regulated in Low
FLNC	0.58505026	1.47933312	5.67969604	2.30E-08	2.95E-07	7.45600193	7.637795794	up-regulated in Low
KCP	0.23366042	0.52789889	4.25101108	2.54E-05	0.00018108	0.69622976	4.594530718	up-regulated in Low
ATP6V1F	0.21037733	6.9110344	4.09696847	4.89E-05	0.00032802	0.07433442	4.310750315	up-regulated in Low
IRF5	-0.1647001	2.4492158	-2.3742173	0.0179662	0.05684672	-5.383476	1.74554378	up-regulated in High
TNPO3	0.28770469	3.91571157	6.53090499	1.62E-10	2.99E-09	12.2936656	9.789755588	up-regulated in Low
SMO	0.24811328	1.93423327	2.92893475	0.00355794	0.01441519	-3.9315813	2.448801618	up-regulated in Low
AHCYL2	-0.7296491	4.20187038	-6.0860091	2.32E-09	3.57E-08	9.69279107	8.634574195	up-regulated in High
STRIP2	0.68025162	1.25817492	8.19740873	2.12E-15	7.80E-14	23.3564109	14.67421734	up-regulated in Low
SMKR1	0.57836418	1.68647583	5.09553476	4.95E-07	5.01E-06	4.4797393	6.305061828	up-regulated in Low
ZC3HC1	0.33141642	2.7850079	9.22742842	8.02E-19	4.55E-17	31.1443197	18.09590542	up-regulated in Low
KLHDC10	-0.1157584	3.41942931	-2.3441104	0.01946659	0.06072533	-5.4538022	1.710710022	up-regulated in High
TMEM209	0.25961522	2.94465079	5.69065361	2.17E-08	2.79E-07	7.51455426	7.663934687	up-regulated in Low
CPA4	0.10022453	0.16870168	2.39599508	0.01694517	0.05414237	-5.3320588	1.770954198	up-regulated in Low
CEP41	0.1537651	1.16007164	5.06392151	5.80E-07	5.78E-06	4.32691402	6.236379641	up-regulated in Low
MEST	0.6367619	3.91098707	9.12589754	1.79E-18	9.81E-17	30.3470369	17.74604207	up-regulated in Low
COPG2	0.27917236	3.39058864	5.32239861	1.55E-07	1.73E-06	5.60146228	6.808374798	up-regulated in Low
PODXL	-0.1878938	3.53323348	-2.8364807	0.00474809	0.01848057	-4.1940299	2.323480712	up-regulated in High
CHCHD3	0.46261319	3.725326	12.2794256	1.91E-30	3.19E-28	57.7074473	29.71837755	up-regulated in Low
SLC35B4	0.12119095	1.85962076	3.21477922	0.0013906	0.00637231	-3.0688751	2.856797225	up-regulated in Low
AKR1B1	0.42779169	4.31992979	4.05563953	5.81E-05	0.00038306	-0.0888707	4.236114143	up-regulated in Low
AKR1B10	1.42033255	2.1723878	5.74724042	1.59E-08	2.10E-07	7.81850254	7.799580156	up-regulated in Low
AKR1B15	0.54124541	0.6691518	5.89722975	6.85E-09	9.64E-08	8.63684513	8.164446923	up-regulated in Low
BPGM	-0.1712102	3.55251257	-2.789672	0.00547937	0.02091278	-4.3237987	2.261269781	up-regulated in High
CALD1	-0.1717106	4.11403159	-2.1577625	0.03142611	0.08999345	-5.8695276	1.502709348	up-regulated in High
C7orf49	0.28770367	2.63053814	8.09058195	4.61E-15	1.61E-13	22.5882217	14.33611882	up-regulated in Low
WDR91	-0.4376862	2.61863417	-7.0373302	6.56E-12	1.49E-10	15.4407624	11.18330089	up-regulated in High
NUP205	0.53793111	3.28708665	10.8107182	1.33E-24	1.40E-22	44.3392344	23.87595245	up-regulated in Low
C7orf73	0.34314258	3.8292229	7.84138127	2.75E-14	8.60E-13	20.8264289	13.56022373	up-regulated in Low
FAM180A	-0.2281025	0.50497726	-5.6880372	2.20E-08	2.83E-07	7.50056411	7.657689459	up-regulated in High
MTPN	0.25891743	5.50056945	5.27178218	2.02E-07	2.20E-06	5.34740133	6.694497128	up-regulated in Low
PTN	-0.6096159	2.10621744	-5.1481725	3.80E-07	3.92E-06	4.73610008	6.420212797	up-regulated in High
RPL41	0.20776908	7.53217683	3.74312967	0.00020304	0.00117203	-1.2725993	3.692410733	up-regulated in Low
ATP6V0A4	0.26512788	1.15031993	2.43124173	0.01540068	0.05003894	-5.24787	1.812459978	up-regulated in Low
TMEM213	-0.2645778	0.71630487	-2.9150543	0.00371724	0.01498367	-3.9715028	2.42977955	up-regulated in High
KIAA1549	0.39296117	1.36198044	6.64346939	8.08E-11	1.56E-09	12.9762625	10.09236657	up-regulated in Low
ZC3HAV1L	0.36165934	1.94613241	6.59390225	1.10E-10	2.08E-09	12.674479	9.95860528	up-regulated in Low
CLEC2L	0.12948998	0.20344265	2.76443028	0.00591469	0.02232328	-4.3929082	2.228067835	up-regulated in Low
TBXAS1	-0.3237755	2.40553563	-4.9170839	1.20E-06	1.12E-05	3.62833948	5.922058357	up-regulated in High
KDM7A	-0.1703395	2.34177792	-3.6415753	0.00029949	0.00165044	-1.6379848	3.523615838	up-regulated in High
SLC37A3	0.14882262	2.54832304	3.67364862	0.00026515	0.00148261	-1.5236152	3.576505885	up-regulated in Low
DENND2A	-0.2525156	1.34950099	-4.3189044	1.89E-05	0.0001387	0.97710141	4.72239762	up-regulated in High
NDUFB2	0.12067276	4.02548877	2.55497967	0.01091707	0.03749722	-4.9428198	1.961893755	up-regulated in Low
BRAF	-0.1183326	2.07950633	-2.9243347	0.00361003	0.0145999	-3.9448318	2.442489462	up-regulated in High
MRPS33	0.28858331	2.90950473	5.95252815	5.00E-09	7.23E-08	8.94317688	8.300906959	up-regulated in Low
TMEM178B	0.17797897	0.39021813	3.29108266	0.00106918	0.0050486	-2.8255525	2.970951217	up-regulated in Low
AGK	0.11841304	2.53124856	3.34279066	0.00089215	0.00430245	-2.6575568	3.049563575	up-regulated in Low
SSBP1	0.37365391	3.73426465	8.93299208	8.15E-18	4.06E-16	28.8496118	17.08870349	up-regulated in Low
CLEC5A	-0.4528802	1.66359745	-5.4233934	9.16E-08	1.06E-06	6.11484844	7.038293455	up-regulated in High
EPHB6	-0.3020281	0.9749868	-4.2863765	2.18E-05	0.00015783	0.84201949	4.660923536	up-regulated in High
TRPV6	-0.1183543	0.15907054	-4.8546839	1.62E-06	1.48E-05	3.33711576	5.790832697	up-regulated in High
PIP	-0.5533933	1.25403105	-4.2116363	3.01E-05	0.00021104	0.53523347	4.521155919	up-regulated in High
CASP2	0.19140033	2.80919407	4.26148313	2.43E-05	0.00017385	0.73928255	4.614142032	up-regulated in Low
TPK1	-0.1854113	0.69440242	-5.9146605	6.20E-09	8.82E-08	8.73313722	8.207348536	up-regulated in High
CNTNAP2	0.42533569	0.47981867	5.97586073	4.38E-09	6.39E-08	9.0731736	8.358796904	up-regulated in Low
CUL1	0.14223923	4.39453688	4.33706854	1.75E-05	0.00012915	1.05294565	4.756895937	up-regulated in Low
EZH2	0.86244396	2.37195909	13.2928722	1.06E-34	2.31E-32	67.4583929	33.97437416	up-regulated in Low

PDIA4	0.39502142	7.12619364	5.95922185	4.81E-09	6.98E-08	8.98042557	8.317495635	up-regulated in Low
ZNF425	-0.1295571	0.80790622	-4.1742455	3.53E-05	0.00024395	0.38364074	4.452010466	up-regulated in High
ZNF282	0.10910531	3.40387664	2.44111279	0.01499104	0.04894492	-5.2240772	1.824168154	up-regulated in Low
ZNF777	0.19715552	2.88714727	4.65346375	4.19E-06	3.51E-05	2.42112076	5.377274078	up-regulated in Low
ZNF746	0.1568902	2.07340471	4.84916816	1.66E-06	1.51E-05	3.31153627	5.779300909	up-regulated in Low
ZNF862	-0.2257593	1.45472456	-4.7820383	2.29E-06	2.02E-05	3.00233886	5.639833321	up-regulated in High
LRRC61	0.39814792	2.97382436	7.09250725	4.57E-12	1.06E-10	15.7953326	11.34006436	up-regulated in Low
ZBED6CL	0.24014617	2.55422448	3.72774147	0.00021548	0.00123517	-1.328576	3.66658434	up-regulated in Low
RARRES2	-0.4605334	5.0525935	-4.4714212	9.64E-06	7.50E-05	1.62307515	5.0158423	up-regulated in High
REPIN1	0.19696141	4.55538225	3.83816654	0.00014	0.00084172	-0.9220669	3.853884549	up-regulated in Low
GIMAP8	-0.4318264	2.13580939	-6.4620654	2.47E-10	4.43E-09	11.881056	9.606730269	up-regulated in High
GIMAP7	-0.4803297	3.31093029	-5.9122112	6.29E-09	8.92E-08	8.71959152	8.20131382	up-regulated in High
GIMAP4	-0.3881003	3.72125678	-4.9527091	1.01E-06	9.58E-06	3.79611588	5.997606228	up-regulated in High
GIMAP6	-0.4298062	2.58874028	-5.9965869	3.89E-09	5.74E-08	9.18901805	8.410375229	up-regulated in High
GIMAP2	-0.3963485	2.60214008	-5.331803	1.48E-07	1.65E-06	5.64890441	6.829632489	up-regulated in High
GIMAP1	-0.3595701	1.30399513	-7.227225	1.88E-12	4.57E-11	16.6704953	11.72680863	up-regulated in High
GIMAP5	-0.1071824	0.46901648	-3.3878639	0.00076048	0.00374225	-2.5090754	3.118915167	up-regulated in High
ABCB8	0.10130366	2.44289938	2.34448678	0.01944718	0.06068512	-5.4529284	1.711143416	up-regulated in Low
CDK5	0.1849048	2.80825408	3.99942094	7.32E-05	0.00047155	-0.308387	4.135610714	up-regulated in Low
FASTK	0.13752372	4.39788852	2.38298461	0.01754882	0.05574057	-5.3628315	1.755751989	up-regulated in Low
TMUB1	0.22181956	4.52888188	3.65582459	0.00028375	0.00157244	-1.5872909	3.547065548	up-regulated in Low
AGAP3	-0.1029826	3.12890554	-2.1000086	0.03623301	0.10088878	-5.9915175	1.44089558	up-regulated in High
ABCF2	0.32944404	4.02292472	8.93072527	8.30E-18	4.13E-16	28.8321526	17.08103733	up-regulated in Low
SMARCD3	-0.5707683	2.43533824	-8.2028156	2.04E-15	7.51E-14	23.3954957	14.69141622	up-regulated in High
WDR86	-0.2654991	1.35470381	-2.8844069	0.00409224	0.01626615	-4.0589981	2.388039073	up-regulated in High
CRYGN	-0.1421795	0.42100934	-3.0143492	0.00270728	0.01135636	-3.6818936	2.567466121	up-regulated in High
RHEB	0.24231353	4.76038856	4.46293101	1.00E-05	7.76E-05	1.58657145	4.999282108	up-regulated in Low
GALNT11	-0.3800171	3.09702976	-6.9899212	8.93E-12	1.98E-10	15.1379207	11.04937212	up-regulated in High
KMT2C	-0.1383231	2.47314167	-2.6493624	0.00832191	0.02976	-4.7002285	2.079776794	up-regulated in High
XRCC2	0.73011798	1.18380329	14.3576217	2.45E-39	7.02E-37	78.0890142	38.61053715	up-regulated in Low
ACTR3B	0.20326945	1.13942176	6.02143348	3.37E-09	5.03E-08	9.32834907	8.472399138	up-regulated in Low
PAXIP1	0.26547684	1.63412712	7.47008157	3.65E-13	9.76E-12	18.2816529	12.43815819	up-regulated in Low
EN2	-0.1089787	0.25729134	-3.0223492	0.00263802	0.01110978	-3.6581533	2.578722206	up-regulated in High
SHH	-0.6088835	1.06630948	-8.3174084	8.75E-16	3.38E-14	24.2284524	15.05787813	up-regulated in High
LMBR1	0.15602247	2.42421421	3.868018	0.00012437	0.00075686	-0.8102538	3.905303544	up-regulated in Low
NOM1	0.22318556	2.04617008	6.02043267	3.39E-09	5.06E-08	9.32272726	8.469896801	up-regulated in Low
UBE3C	0.22631982	3.57362455	5.37671042	1.17E-07	1.33E-06	5.87647794	6.931571722	up-regulated in Low
DNAJB6	0.13644205	2.74923757	3.68982952	0.00024927	0.00140374	-1.4655555	3.603335811	up-regulated in Low
PTPRN2	-0.2396127	1.90437617	-2.0443224	0.04144966	0.11251145	-6.1060605	1.382479018	up-regulated in High
NCAPG2	0.91821832	2.31636529	15.5060384	1.71E-44	6.55E-42	89.9235831	43.76825842	up-regulated in Low
VIPR2	0.11840255	0.23927558	3.48404772	0.00053768	0.00275574	-2.1858821	3.269478135	up-regulated in Low
ZNF596	-0.1270646	0.5945454	-4.9695198	9.26E-07	8.89E-06	3.87566708	6.033414273	up-regulated in High
FBXO25	-0.1938713	1.85330583	-4.5034622	8.34E-06	6.57E-05	1.76141284	5.078577292	up-regulated in High
TDRP	-0.5549011	2.88043426	-7.4306907	4.77E-13	1.25E-11	18.0174266	12.32155206	up-regulated in High
KBTBD11	-0.1880225	1.41733875	-2.7622684	0.00595339	0.02245193	-4.3987989	2.225235407	up-regulated in High
ANGPT2	0.28088504	1.39462783	4.17131078	3.58E-05	0.00024677	0.37179578	4.446605306	up-regulated in Low
AGPAT5	0.4284254	2.57141108	7.57834688	1.73E-13	4.85E-12	19.0135983	12.76107061	up-regulated in Low
DEFB1	1.0144979	2.38298616	5.58556092	3.85E-08	4.77E-07	6.95707488	7.414952819	up-regulated in Low
SGK223	-0.1554779	2.6139439	-2.4657403	0.01401071	0.04625895	-5.1643056	1.853539919	up-regulated in High
CLDN23	-0.4816136	2.93024158	-6.639334	8.30E-11	1.60E-09	12.9510122	10.08117631	up-regulated in High
ERI1	0.24853496	2.02121896	5.91123468	6.33E-09	8.97E-08	8.71419243	8.198908449	up-regulated in Low
MSRA	-0.2562738	1.85215882	-5.5187974	5.51E-08	6.67E-07	6.6076769	7.258771928	up-regulated in High
SOX7	-0.2439672	1.14582059	-4.0083032	7.06E-05	0.00045647	-0.2738953	4.151411385	up-regulated in High
PINX1	0.22660806	1.688693	6.07180945	2.52E-09	3.84E-08	9.61236597	8.598792513	up-regulated in Low
FAM167A	-0.3576459	1.51365478	-3.8734213	0.00012172	0.00074292	-0.7899277	3.914646402	up-regulated in High
BLK	-0.3634774	0.67442839	-6.0592668	2.71E-09	4.11E-08	9.54146019	8.567242716	up-regulated in High
LONRF1	-0.1225068	1.92205304	-2.7471836	0.00622987	0.0233468	-4.4397779	2.205521289	up-regulated in High
DLC1	-1.1104913	2.68650592	-12.64023	6.10E-32	1.13E-29	61.1339956	31.21440585	up-regulated in High
C8orf48	-0.2069096	0.68308812	-5.0642648	5.79E-07	5.78E-06	4.32856912	6.237123623	up-regulated in High
TUSC3	0.39636862	3.05141946	3.50755422	0.00049339	0.00255955	-2.1055862	3.306806283	up-regulated in Low
MSR1	-0.5127592	2.78370714	-5.1818615	3.20E-07	3.36E-06	4.9014172	6.494429291	up-regulated in High
MICU3	-0.1897799	0.70817613	-5.8586086	8.52E-09	1.18E-07	8.42437223	8.069759818	up-regulated in High
ZDHHC2	-0.2973739	2.69625108	-4.3920507	1.37E-05	0.00010352	1.28431995	4.862063034	up-regulated in High
CNOT7	0.14946574	2.97110251	3.60578619	0.00034272	0.00185645	-1.7644823	3.465055715	up-regulated in Low
VPS37A	0.20524492	2.69976155	3.57505066	0.00038446	0.00205187	-1.8721713	3.415150042	up-regulated in Low
MTMR7	0.21138089	0.70845231	2.45060877	0.01460608	0.04785664	-5.2010996	1.835466241	up-regulated in Low
SLC7A2	0.45078372	2.83941674	2.65177474	0.00826354	0.02958929	-4.6939158	2.082834122	up-regulated in Low
MTUS1	-0.4580579	3.77873967	-5.5081821	5.83E-08	7.02E-07	6.55246533	7.234082632	up-regulated in High
FGL1	1.12922521	1.85491952	5.51460476	5.64E-08	6.81E-07	6.58585938	7.249015975	up-regulated in Low
ASAH1	-0.527647	6.08921689	-7.7367687	5.76E-14	1.73E-12	20.0996251	13.23992612	up-regulated in High
NAT1	-0.1407135	1.82631284	-2.5954122	0.00972792	0.0340661	-4.8399447	2.011979977	up-regulated in High

SH2D4A	-0.2419871	3.40898537	-3.3833107	0.0007729	0.00379255	-2.524161	3.111874424	up-regulated in High
CSGALNACT	0.21680153	1.95875235	3.21939361	0.00136887	0.00628928	-3.0543158	2.863637888	up-regulated in Low
LPL	-0.9689005	2.92067371	-7.0969452	4.44E-12	1.03E-10	15.8239494	11.35271458	up-regulated in High
ATP6V1B2	-0.2176402	3.94877776	-4.6509587	4.24E-06	3.55E-05	2.4099407	5.372218304	up-regulated in High
GFRA2	-0.1303214	0.27772737	-5.738442	1.67E-08	2.20E-07	7.7710701	7.77841678	up-regulated in High
DOK2	-0.4887858	2.86484718	-5.7885823	1.26E-08	1.70E-07	8.04222781	7.89937891	up-regulated in High
XPO7	0.17051055	3.58204547	3.93796583	9.40E-05	0.00058967	-0.5450625	4.027095748	up-regulated in Low
DMTN	-0.2797491	3.00686675	-3.4828456	0.00054004	0.00276517	-2.1899747	3.267574782	up-regulated in High
FAM160B2	-0.1536712	2.90231498	-2.7044628	0.00707687	0.025966	-4.5546503	2.15015905	up-regulated in High
NUDT18	-0.1784465	2.51408551	-3.3218221	0.00096037	0.004592	-2.7259843	3.017562637	up-regulated in High
HR	-0.1362322	0.379997	-3.4938773	0.00051873	0.00267103	-2.1523676	3.285062094	up-regulated in High
REEP4	0.29282491	3.77644489	6.09223731	2.24E-09	3.45E-08	9.72811801	8.65029014	up-regulated in Low
LGI3	-0.4718601	0.63918211	-5.61842	3.22E-08	4.04E-07	7.13039849	7.49238998	up-regulated in High
SFTPC	-2.5684841	5.15465589	-8.1347973	3.34E-15	1.19E-13	22.9052398	14.47566106	up-regulated in High
POLR3D	0.20765442	2.4938959	5.47252607	7.06E-08	8.36E-07	6.36770226	7.151441204	up-regulated in Low
SLC39A14	0.48175558	2.98591332	6.60583701	1.02E-10	1.95E-09	12.7469693	9.990739329	up-regulated in Low
PPP3CC	-0.2031917	2.50282198	-5.1625708	3.53E-07	3.67E-06	4.80663607	6.451882575	up-regulated in High
SORBS3	-0.3182043	3.57787725	-5.2663034	2.08E-07	2.26E-06	5.32003146	6.682225162	up-regulated in High
PDLIM2	-0.3957911	1.4607704	-9.3741902	2.48E-19	1.49E-17	32.3077631	18.60629751	up-regulated in High
CCAR2	0.10832942	3.60188309	2.43147951	0.0153907	0.05001486	-5.247298	1.812741572	up-regulated in Low
EGR3	-0.4506929	1.29293169	-6.4784116	2.24E-10	4.04E-09	11.9786977	9.650049622	up-regulated in High
PEBP4	-1.6897239	2.76597646	-10.288648	1.24E-22	1.09E-20	39.8371262	21.90565775	up-regulated in High
RHOBTB2	-0.9712259	3.84033413	-9.7318135	1.34E-20	9.39E-19	35.1959483	19.87260888	up-regulated in High
TNFRSF10C	-0.4350297	1.98299009	-6.0933548	2.22E-09	3.43E-08	9.73445967	8.653111282	up-regulated in High
LOXL2	0.49928827	3.04953419	4.64482921	4.37E-06	3.64E-05	2.38260801	5.359857225	up-regulated in Low
NKX3-1	0.17678041	1.05820867	3.11660698	0.00193564	0.00849427	-3.3738761	2.713175064	up-regulated in Low
STC1	0.88053908	2.59638611	8.06343645	5.61E-15	1.92E-13	22.3942484	14.25072676	up-regulated in Low
ADAM28	-0.3028757	2.01281535	-3.5475338	0.00042582	0.00224654	-1.9678402	3.370772916	up-regulated in High
ADAMDEC1	0.34853358	2.03575185	2.86279892	0.00437699	0.01722679	-4.1201495	2.358824256	up-regulated in Low
NEFM	0.15672219	0.12927278	3.69876093	0.00024089	0.00136233	-1.4334048	3.618187395	up-regulated in Low
NEFL	0.18691794	0.28974362	2.71206985	0.00691885	0.02549193	-4.5343235	2.159966379	up-regulated in Low
GNRH1	-0.1219323	0.65503719	-3.3404786	0.00089944	0.00433298	-2.6651221	3.046026867	up-regulated in High
KCTD9	0.16164122	2.70519323	3.41347634	0.00069396	0.00345012	-2.4238564	3.158666127	up-regulated in Low
CDCA2	0.93603429	1.24451297	18.6872014	2.23E-59	2.37E-56	124.105254	58.65165879	up-regulated in Low
BNIP3L	-0.2614362	4.98829622	-4.8178343	1.93E-06	1.73E-05	3.16672557	5.713999456	up-regulated in High
PNMA2	-0.8384888	2.32406162	-8.5470527	1.57E-16	6.71E-15	25.9238111	15.80334895	up-regulated in High
DPYSL2	-0.9641084	4.32361739	-13.351719	5.94E-35	1.31E-32	68.0362027	34.2264569	up-regulated in High
TRIM35	-0.1390021	2.23004406	-3.9536357	8.82E-05	0.00055672	-0.4850415	4.05463092	up-regulated in High
PTK2B	-0.4912809	2.9006119	-8.1621478	2.74E-15	9.92E-14	23.1019996	14.56225846	up-regulated in High
EPHX2	-0.4243235	1.89869688	-6.1965685	1.21E-09	1.95E-08	10.3245042	8.915492305	up-regulated in High
CLU	-0.9434693	5.20039922	-6.5980098	1.07E-10	2.04E-09	12.6994152	9.969659422	up-regulated in High
SCARA3	-0.5082491	2.29288122	-5.5153673	5.61E-08	6.78E-07	6.58982626	7.250789842	up-regulated in High
ESCO2	0.56906917	0.70684823	17.2217939	1.95E-52	1.25E-49	108.157014	51.709469	up-regulated in Low
PBK	1.53889224	2.12595464	20.4699973	6.05E-68	2.72E-64	143.792155	67.21791894	up-regulated in Low
SCARA5	-0.2598248	0.83524999	-2.9409807	0.00342477	0.01394532	-3.8967876	2.465368931	up-regulated in High
NUGGC	-0.175451	0.64825132	-3.6925766	0.00024666	0.00139107	-1.4556747	3.607900552	up-regulated in High
PNOC	-0.1647878	0.78626212	-2.8321744	0.00481147	0.01868736	-4.2060558	2.317722597	up-regulated in High
ZNF395	-0.1024403	3.04772423	-2.0043322	0.04557811	0.12129303	-6.1864507	1.341243683	up-regulated in High
DUSP4	0.83432914	3.13789946	5.89268669	7.03E-09	9.86E-08	8.61178867	8.153282313	up-regulated in Low
SARAF	-0.3258078	6.33981306	-6.8504563	2.19E-11	4.60E-10	14.2568058	10.65951277	up-regulated in High
DCTN6	0.1171946	3.56981585	2.61210987	0.0092716	0.03266995	-4.797001	2.03284525	up-regulated in Low
RBPMS	-0.8138863	3.23796977	-11.376765	8.36E-27	1.07E-24	49.3744637	26.07779021	up-regulated in High
GTF2E2	0.322186	3.68394457	7.16358981	2.86E-12	6.81E-11	16.2554373	11.54342189	up-regulated in Low
GSR	0.51638653	5.06445617	5.75819703	1.49E-08	1.99E-07	7.87765867	7.825972013	up-regulated in Low
PPP2CB	-0.2089352	4.31489838	-4.4985362	8.53E-06	6.70E-05	1.7400855	5.068907869	up-regulated in High
TEX15	0.13462045	0.09045523	5.98331783	4.19E-09	6.15E-08	9.11481343	8.377337538	up-regulated in Low
WRN	0.1381133	1.78936351	3.40827794	0.000707	0.00350751	-2.4412023	3.150578031	up-regulated in Low
TTI2	0.12616815	1.682392	4.17122548	3.58E-05	0.00024683	0.37145159	4.446448241	up-regulated in Low
MAK16	0.230823	2.25064145	6.00033893	3.80E-09	5.62E-08	9.21002604	8.419727842	up-regulated in Low
RNF122	-0.1473977	2.61728517	-2.7856863	0.00554612	0.02114404	-4.3347515	2.256011102	up-regulated in High
DUSP26	-0.1489851	0.38733218	-4.2770339	2.27E-05	0.00016365	0.80339665	4.64333933	up-regulated in High
UNC5D	0.11029422	0.08591916	3.04254382	0.00247033	0.01050282	-3.5979547	2.607244636	up-regulated in Low
ERLIN2	0.24213716	3.10239134	4.14244585	4.04E-05	0.00027557	0.2557075	4.39361283	up-regulated in Low
GPR124	-0.2807655	2.21653526	-4.2373471	2.70E-05	0.0001909	0.64020239	4.569002857	up-regulated in High
BRF2	0.2193979	1.97330227	4.39508758	1.36E-05	0.00010221	1.29717816	4.867904255	up-regulated in Low
RAB11FIP1	-0.4290678	3.49569831	-5.0071973	7.69E-07	7.49E-06	4.05484844	6.114038489	up-regulated in High
EIF4EBP1	1.1277944	5.01987423	12.3616505	8.76E-31	1.51E-28	58.4837518	30.05735737	up-regulated in Low
ASH2L	0.19344074	3.1205613	4.03052902	6.44E-05	0.0004196	-0.1872741	4.191077747	up-regulated in Low
LSM1	0.36688545	3.65617096	6.9792024	9.57E-12	2.11E-10	15.069683	11.01919002	up-regulated in Low
BAG4	0.25862363	2.66868469	4.90075997	1.30E-06	1.21E-05	3.55182871	5.887593844	up-regulated in Low
DDHD2	0.23762367	2.21189192	4.28976679	2.15E-05	0.00015569	0.85605432	4.66731249	up-regulated in Low

WHSC1L1	0.17274718	2.63411034	3.32515905	0.0009492	0.00454417	-2.7151221	3.022644194	up-regulated in Low
LETM2	0.13319056	0.71226368	3.44156776	0.00062726	0.00315779	-2.3296852	3.202549978	up-regulated in Low
TACC1	-0.4704131	3.01923208	-6.7402311	4.41E-11	8.86E-10	13.5708203	10.35577881	up-regulated in High
PLEKHA2	-0.1538541	2.79346917	-2.5340058	0.01158361	0.03943843	-4.9955653	1.936156268	up-regulated in High
HTRA4	-0.2005325	0.85967608	-3.3252277	0.00094897	0.00454351	-2.7148987	3.022748734	up-regulated in High
TM2D2	0.13604089	3.48465706	2.57082172	0.01043652	0.03605925	-4.9026992	1.981444418	up-regulated in Low
ADAM9	0.18348195	4.8819187	2.23244492	0.02603159	0.07721925	-5.7069674	1.584499312	up-regulated in Low
ZMAT4	0.36716797	0.27420238	5.74733439	1.59E-08	2.10E-07	7.81900945	7.799806322	up-regulated in Low
SFRP1	0.24175316	0.83107307	2.39825953	0.01684199	0.05387646	-5.3266861	1.773606644	up-regulated in Low
GOLGA7	0.28741651	4.46946729	5.39627302	1.06E-07	1.21E-06	5.9761446	6.976200458	up-regulated in Low
GINS4	0.73600295	1.12068399	14.3903574	1.76E-39	5.08E-37	78.4214148	38.7554491	up-regulated in Low
AGPAT6	0.21066668	3.61088411	3.42538512	0.00066492	0.00332156	-2.3840243	3.177233341	up-regulated in Low
KAT6A	0.15334766	2.65373021	2.74597102	0.00625259	0.02341842	-4.4430624	2.203940391	up-regulated in Low
AP3M2	0.1280352	2.16795543	2.6264786	0.00889437	0.03151951	-4.7598326	2.050884944	up-regulated in Low
PLAT	-0.7196559	3.54112099	-4.7918127	2.19E-06	1.94E-05	3.04711506	5.660038899	up-regulated in High
IKBKB	-0.3211393	2.95075488	-5.1598057	3.58E-07	3.72E-06	4.79307653	6.445794949	up-regulated in High
POLB	0.18019685	2.91420374	2.86206579	0.00438696	0.01726138	-4.1222165	2.357836148	up-regulated in Low
VDAC3	0.54459242	4.8269242	9.37821236	2.40E-19	1.44E-17	32.3398291	18.62036216	up-regulated in Low
THAP1	0.15328275	2.39637195	4.05187957	5.90E-05	0.00038783	-0.1036417	4.229355577	up-regulated in Low
HOOK3	-0.1334477	2.52383243	-2.2927313	0.02228155	0.06784278	-5.5717898	1.652054531	up-regulated in High
FNTA	0.21729284	2.92596035	4.61111717	5.10E-06	4.20E-05	2.23286968	5.29211657	up-regulated in Low
POMK	0.16946609	1.20139909	2.67465749	0.0077278	0.0279625	-4.6337589	2.111944329	up-regulated in Low
HGSNAT	-0.1753413	4.00655422	-2.5831639	0.0100753	0.03504091	-4.8712748	1.996741857	up-regulated in High
CEBPD	-0.3298579	5.37429107	-3.4110995	0.0006999	0.00347608	-2.4317905	3.154966805	up-regulated in High
PRKDC	0.63441546	3.85479878	9.31244793	4.07E-19	2.38E-17	31.8167346	18.39090889	up-regulated in Low
MCM4	1.18747865	3.64684133	17.2237786	1.91E-52	1.24E-49	108.178414	51.71878637	up-regulated in Low
UBE2V2	0.45030921	3.18862996	10.5761688	1.04E-23	1.02E-21	42.2991567	22.98333899	up-regulated in Low
EFCAB1	-0.3323876	0.54541751	-4.6280219	4.72E-06	3.90E-05	2.30783007	5.326032892	up-regulated in High
PCMTD1	-0.1840953	3.39548867	-3.9365431	9.45E-05	0.00059273	-0.5505009	4.024600295	up-regulated in High
ST18	0.10122071	0.09801404	3.26091903	0.00118697	0.00553764	-2.9223943	2.925561	up-regulated in Low
RB1CC1	0.14220347	3.3122598	2.94035682	0.00343155	0.01397099	-3.898593	2.464509537	up-regulated in Low
ATP6V1H	0.12955891	3.56890223	3.23959976	0.00127736	0.00590601	-2.9903256	2.893687811	up-regulated in Low
RGS20	0.45390829	0.54663763	8.21107079	1.92E-15	7.09E-14	23.4552083	14.7176916	up-regulated in Low
TCEA1	0.24750985	4.31588519	4.75253606	2.64E-06	2.29E-05	2.8676958	5.579056537	up-regulated in Low
LYPLA1	0.37346371	4.41541259	5.16649271	3.46E-07	3.61E-06	4.82588007	6.460521914	up-regulated in Low
MRPL15	0.69582949	5.39567738	12.000195	2.65E-29	4.09E-27	55.0920098	28.57611448	up-regulated in Low
SOX17	-0.1750817	0.89950632	-3.5969652	0.00035425	0.00190858	-1.7954782	3.450696481	up-regulated in High
TMEM68	0.16945094	2.01297644	3.7849082	0.00017259	0.00101339	-1.1195256	3.762977422	up-regulated in Low
TGS1	0.26158577	3.06762352	5.84141783	9.38E-09	1.29E-07	8.33018877	8.027777436	up-regulated in Low
RPS20	0.13103178	8.02292303	2.20958809	0.02759025	0.08094342	-5.7572955	1.559244426	up-regulated in Low
SDR16C5	-0.7138522	3.77963359	-5.8252696	1.03E-08	1.40E-07	8.24193649	7.988433138	up-regulated in High
PENK	-0.228296	0.50305989	-2.9567371	0.00325745	0.01333936	-3.8510685	2.487122594	up-regulated in High
IMPAD1	0.29479784	4.05826035	6.10798045	2.04E-09	3.17E-08	9.81755292	8.690073764	up-regulated in Low
FAM110B	-0.160818	0.85308368	-3.1974386	0.00147515	0.00671159	-3.1234091	2.831162679	up-regulated in High
NSMAF	0.12870568	2.67910215	3.29180706	0.00106648	0.00503752	-2.8232163	2.972045536	up-regulated in Low
TOX	-0.4409484	1.89268381	-5.1987377	2.94E-07	3.10E-06	4.98459503	6.531759115	up-regulated in High
RAB2A	0.1589272	4.96307487	3.56187988	0.00040376	0.00214489	-1.9180503	3.393873558	up-regulated in Low
CHD7	0.40439715	1.60343383	7.11135408	4.04E-12	9.43E-11	15.9169608	11.39382878	up-regulated in Low
ASPH	0.43055652	4.03900837	3.9701998	8.24E-05	0.00052483	-0.4213521	4.083837208	up-regulated in Low
GGH	1.14061678	2.87356621	11.4213748	5.57E-27	7.24E-25	49.7777634	26.25407638	up-regulated in Low
YTHDF3	0.12714623	3.88771541	2.94887909	0.00333994	0.01364175	-3.8738989	2.476261858	up-regulated in Low
BHLHE22	-0.326096	0.78622693	-8.1893042	2.25E-15	8.25E-14	23.2978619	14.64845283	up-regulated in High
CYP7B1	-0.300286	1.76080344	-4.3396783	1.73E-05	0.00012784	1.06386706	4.761862605	up-regulated in High
ARMC1	0.42459475	3.86282213	8.51726384	1.97E-16	8.30E-15	25.7019477	15.70582246	up-regulated in Low
MTFR1	0.54613472	3.13696458	9.01708688	4.23E-18	2.19E-16	29.499574	17.37406285	up-regulated in Low
DNAJC5B	-0.2046302	0.84237693	-4.1373515	4.13E-05	0.00028109	0.23529737	4.384292427	up-regulated in High
RRS1	0.59332598	3.98974833	9.86026471	4.62E-21	3.46E-19	36.2512943	20.33508771	up-regulated in Low
ADHFE1	-0.4226761	1.03576176	-7.1627594	2.88E-12	6.85E-11	16.2500409	11.54103722	up-regulated in High
MYBL1	0.41118809	0.96667259	8.10457917	4.17E-15	1.46E-13	22.6884368	14.38023292	up-regulated in Low
VCPIP1	0.11145046	2.23043887	2.85185894	0.00452793	0.01773833	-4.1509404	2.344100646	up-regulated in Low
TCF24	0.12459886	0.20678656	5.00920959	7.61E-07	7.42E-06	4.06445237	6.118358706	up-regulated in Low
PPP1R42	-0.1350132	0.19688406	-4.1934585	3.25E-05	0.00022661	0.46137843	4.487475623	up-regulated in High
COPS5	0.33143822	3.07683409	7.45906455	3.93E-13	1.05E-11	18.2076406	12.40549776	up-regulated in Low
CSPP1	0.12085999	1.71274486	2.59220627	0.0098178	0.0343232	-4.8481591	2.007985976	up-regulated in Low
CPA6	-0.1860997	0.43818337	-3.0218746	0.00264208	0.01112448	-3.6595631	2.578053888	up-regulated in High
PREX2	-0.2237353	0.66507234	-5.4444871	8.19E-08	9.59E-07	6.22315604	7.086766496	up-regulated in High
C8orf34	-0.2375805	0.35334916	-7.4290147	4.82E-13	1.27E-11	18.0062097	12.31660146	up-regulated in High
SULF1	0.28836194	3.77864423	2.25599019	0.0245064	0.07352045	-5.6545928	1.610720513	up-regulated in Low
NCOA2	-0.1924221	2.86309224	-3.4542394	0.00059918	0.00303078	-2.2869653	3.222442967	up-regulated in High
TRAM1	-0.3867018	6.84679569	-5.3009031	1.74E-07	1.91E-06	5.49330419	6.759903214	up-regulated in High
TRPA1	0.15020011	0.163024	5.1575239	3.62E-07	3.75E-06	4.78189172	6.440773312	up-regulated in Low

TERF1	0.19588654	1.88009448	5.97424183	4.42E-09	6.44E-08	9.06413977	8.354774336	up-regulated in Low
RPL7	0.28102009	7.47527429	4.50889254	8.14E-06	6.42E-05	1.78494873	5.089247046	up-regulated in Low
RDH10	0.44888724	4.00977699	3.69163617	0.00024755	0.00139541	-1.459058	3.606337574	up-regulated in Low
STAU2	0.13500885	2.84161242	2.54567954	0.01120829	0.03834407	-4.9662603	1.95046077	up-regulated in Low
TCEB1	0.46284705	3.59617798	8.64783579	7.33E-17	3.24E-15	26.6786854	16.13511196	up-regulated in Low
TMEM70	0.2612165	3.52292942	4.66887988	3.90E-06	3.28E-05	2.49004446	5.408437739	up-regulated in Low
LY96	-0.2589191	4.30187343	-3.1601297	0.00167342	0.00748016	-3.2397809	2.776395815	up-regulated in High
GDAP1	0.25649419	1.7010272	3.9404309	9.30E-05	0.00058425	-0.5356353	4.031421292	up-regulated in Low
PI15	0.33692398	0.77446672	4.96559936	9.44E-07	9.05E-06	3.85709289	6.025054291	up-regulated in Low
HNF4G	0.28791647	1.27827201	3.73354474	0.00021071	0.0012115	-1.3074914	3.676313653	up-regulated in Low
ZC2HC1A	-0.1587414	2.22872628	-2.6513427	0.00827396	0.02961574	-4.6950468	2.082286411	up-regulated in High
IL7	-0.2206493	1.14002065	-4.3642199	1.55E-05	0.00011589	1.16686572	4.808690276	up-regulated in High
MRPS28	0.37314848	3.14068499	6.15402621	1.56E-09	2.46E-08	10.0802715	8.806911873	up-regulated in Low
TPD52	0.40556242	3.79460613	5.75367551	1.53E-08	2.03E-07	7.85323445	7.815075736	up-regulated in Low
ZBTB10	0.27563892	2.28939272	4.08739221	5.09E-05	0.00033998	0.03638075	4.293399871	up-regulated in Low
ZNF704	-0.367869	1.66144486	-5.591445	3.73E-08	4.63E-07	6.98804602	7.428791884	up-regulated in High
PAG1	-0.5947248	2.24584738	-8.2395795	1.55E-15	5.81E-14	23.6617722	14.80858043	up-regulated in High
FABP4	-0.6082628	1.4993686	-5.2867474	1.87E-07	2.05E-06	5.42229097	6.72807179	up-regulated in High
IMPA1	0.22724486	2.77681702	4.52676946	7.51E-06	5.97E-05	1.86261412	5.12444859	up-regulated in Low
ZFAND1	0.28884382	3.25918405	5.03264697	6.78E-07	6.67E-06	4.17657011	6.16878503	up-regulated in Low
CHMP4C	0.29818981	3.84840124	4.36258442	1.56E-05	0.00011665	1.15998518	4.805562779	up-regulated in Low
SNX16	0.12299066	1.72338577	2.60839405	0.00937146	0.0329661	-4.8065807	2.028192847	up-regulated in Low
LRRCC1	0.11936617	2.34391867	2.25347596	0.02466547	0.07391034	-5.6602112	1.607910576	up-regulated in Low
E2F5	0.24108257	1.85513829	4.37984368	1.45E-05	0.0001088	1.23271765	4.838617773	up-regulated in Low
C8orf59	0.33292488	3.67694872	5.88429406	7.37E-09	1.03E-07	8.56554475	8.1326759	up-regulated in Low
CA13	-0.4905854	1.66288252	-7.2233177	1.93E-12	4.68E-11	16.6449245	11.71551221	up-regulated in High
CA3	-0.6450163	0.96826866	-9.6780369	2.09E-20	1.42E-18	34.7569104	19.68017625	up-regulated in High
CA2	-0.3539621	2.88180027	-2.864483	0.00435417	0.01716009	-4.1153994	2.361094865	up-regulated in High
ATP6V0D2	-0.3707295	0.9335271	-5.6618876	2.54E-08	3.24E-07	7.36105318	7.595403046	up-regulated in High
RIPK2	0.47886793	3.30629252	7.95975043	1.18E-14	3.92E-13	21.6579606	13.9265188	up-regulated in Low
OSGIN2	0.33504228	3.11531589	5.74219316	1.63E-08	2.15E-07	7.7912849	7.787436414	up-regulated in Low
NBN	0.37168336	3.39548708	7.29134696	1.22E-12	3.05E-11	17.0917302	11.91286728	up-regulated in Low
DECR1	0.14732251	3.96931488	2.98531096	0.00297295	0.01232035	-3.7675564	2.52681297	up-regulated in Low
CALB1	0.18059237	0.18914352	2.54967293	0.01108241	0.03798687	-4.9562054	1.955365973	up-regulated in Low
NECAB1	-0.1887242	0.46101805	-4.9864975	8.52E-07	8.22E-06	3.95625555	6.069680848	up-regulated in High
TMEM55A	0.12691872	1.73553267	2.42600101	0.01562216	0.05063176	-5.2604638	1.80625887	up-regulated in Low
OTUD6B	0.40709652	2.07279129	8.84807796	1.58E-17	7.54E-16	28.1977869	16.80246324	up-regulated in Low
RUNX1T1	-0.1574448	0.44743764	-5.8059503	1.14E-08	1.55E-07	8.13663389	7.941480162	up-regulated in High
TRIQK	-0.122557	2.9632326	-2.1894831	0.02902735	0.08434039	-5.8011445	1.537192569	up-regulated in High
FAM92A1	0.15353075	1.6044125	3.4936275	0.0005192	0.00267324	-2.1532206	3.28466555	up-regulated in Low
RBM12B	0.11962801	1.83647485	3.06957284	0.00226119	0.00972842	-3.5167793	2.64566212	up-regulated in Low
PDP1	-0.1602755	3.13936114	-2.2633193	0.02404777	0.07233782	-5.6381798	1.618925218	up-regulated in High
CDH17	0.52107739	0.95096612	3.83532655	0.00014158	0.00085035	-0.932662	3.849010079	up-regulated in Low
GEM	-0.3102056	3.86890996	-2.7217939	0.00672148	0.02486991	-4.5082592	2.172535003	up-regulated in High
RAD54B	0.34947054	0.60041299	14.7547081	4.20E-41	1.32E-38	82.1415742	40.37708268	up-regulated in Low
KIAA1429	0.26455305	2.89862909	6.62353542	9.15E-11	1.75E-09	12.8546694	10.03847707	up-regulated in Low
ESRP1	0.2828095	4.6088817	4.52358676	7.62E-06	6.05E-05	1.84876633	5.118172942	up-regulated in Low
DPY19L4	0.12485231	2.65126965	2.89018561	0.004019	0.01602084	-4.0425689	2.3958821	up-regulated in Low
INTS8	0.34360403	2.7472845	7.69490779	7.71E-14	2.28E-12	19.8109372	13.11266665	up-regulated in Low
CCNE2	0.47963	1.07360931	9.29712304	4.60E-19	2.67E-17	31.6952103	18.3375978	up-regulated in Low
TP53INP1	-0.6512656	3.77110654	-9.2047705	9.60E-19	5.41E-17	30.9658539	18.01759849	up-regulated in High
UQCRB	0.2321149	4.01446413	4.93347546	1.10E-06	1.04E-05	3.7053989	5.956762022	up-regulated in Low
MTERF3	0.41443312	3.45886568	8.58103364	1.22E-16	5.27E-15	26.1775992	15.91489829	up-regulated in Low
PTDSS1	0.34372516	3.77953276	7.90994361	1.69E-14	5.45E-13	21.3068947	13.77189167	up-regulated in Low
SDC2	-0.4162625	3.34702194	-4.9114697	1.23E-06	1.15E-05	3.60199944	5.9101943	up-regulated in High
CPQ	-0.815454	3.24420792	-12.524225	1.86E-31	3.33E-29	60.0266656	30.73100281	up-regulated in High
MTDH	0.28238881	4.64184143	5.44438098	8.19E-08	9.59E-07	6.22261031	7.086522279	up-regulated in Low
LAPTM4B	0.28424354	6.5185047	3.17875612	0.00157155	0.00709433	-3.1818463	2.803672072	up-regulated in Low
RPL30	0.18167485	7.27101326	3.22595305	0.00133852	0.00616292	-3.033585	2.873375899	up-regulated in Low
ERICH5	0.2534859	1.60473278	2.42045144	0.01585975	0.05128791	-5.2737708	1.799703643	up-regulated in Low
HRSP12	0.23160544	3.4874928	4.12035044	4.43E-05	0.00030026	0.16735301	4.353257595	up-regulated in Low
POP1	0.47015063	1.52065603	13.2702082	1.33E-34	2.88E-32	67.2361812	33.87742611	up-regulated in Low
NIPAL2	-0.2314709	2.76153354	-3.9134561	0.0001037	0.00064462	-0.638494	3.98421133	up-regulated in High
STK3	0.1076079	1.95704646	2.80428469	0.00524081	0.02013392	-4.2835121	2.280601393	up-regulated in Low
COX6C	0.31373532	4.97952043	5.51128968	5.74E-08	6.92E-07	6.5686187	7.241306315	up-regulated in Low
RGS22	-0.2576812	0.33610495	-6.3800707	4.07E-10	7.03E-09	11.3944203	9.390760615	up-regulated in High
FBXO43	0.19264243	0.26779349	8.99833621	4.89E-18	2.52E-16	29.3542719	17.31027476	up-regulated in Low
POLR2K	0.34655546	4.90627934	6.69095175	6.01E-11	1.18E-09	13.2671245	10.22124883	up-regulated in Low
RNF19A	-0.2895769	4.04356694	-3.9904828	7.59E-05	0.0004871	-0.343023	4.11974047	up-regulated in High
PABPC1	0.26710653	8.26191825	4.17862503	3.47E-05	0.00023991	0.40133138	4.460082512	up-regulated in Low
YWHAZ	0.45265884	6.80287056	8.9682281	6.19E-18	3.14E-16	29.1214143	17.20804316	up-regulated in Low

ZNF706	0.24531653	3.29477659	5.54956648	4.67E-08	5.72E-07	6.7682421	7.330557648	up-regulated in Low
GRHL2	0.12978414	3.330022	2.14533219	0.0324116	0.09217809	-5.8960577	1.489299513	up-regulated in Low
NCALD	-0.5861373	1.75796847	-8.7298736	3.92E-17	1.78E-15	27.2979694	16.40721237	up-regulated in High
RRM2B	-0.4258251	3.40265092	-7.3114075	1.07E-12	2.68E-11	17.2241295	11.97133622	up-regulated in High
UBR5	0.22571702	3.24875758	4.00705241	7.09E-05	0.00045871	-0.2787569	4.149184508	up-regulated in Low
AZIN1	0.26017857	4.91242347	4.82955212	1.83E-06	1.65E-05	3.22077991	5.73837844	up-regulated in Low
ATP6V1C1	0.18752149	3.84627871	4.33698117	1.75E-05	0.00012918	1.05258013	4.75672971	up-regulated in Low
CTHRC1	0.35211813	4.89757979	2.7015	0.00713929	0.0261359	-4.5625522	2.146345201	up-regulated in Low
SLC25A32	0.29340664	3.15245418	7.16823638	2.77E-12	6.62E-11	16.2856435	11.55676998	up-regulated in Low
DCAF13	0.45739518	2.79358771	9.25170054	6.61E-19	3.79E-17	31.3358434	18.17993713	up-regulated in Low
RIMS2	0.27935111	0.21131608	7.52145273	2.56E-13	7.01E-12	18.6279123	12.59093621	up-regulated in Low
DCSTAMP	-0.284328	0.64898176	-5.5637748	4.33E-08	5.33E-07	6.84265258	7.363817613	up-regulated in High
LRP12	0.36890608	1.46853961	6.67703535	6.56E-11	1.28E-09	13.1816978	10.18339973	up-regulated in Low
ZFPM2	-0.1634752	0.76576702	-4.1546536	3.84E-05	0.00026311	0.30471262	4.415987003	up-regulated in High
OXR1	-0.1709846	2.90708319	-3.2714372	0.00114459	0.00535963	-2.8887221	2.941349608	up-regulated in High
ANGPT1	-0.4899866	1.20383682	-7.5220119	2.56E-13	6.98E-12	18.6316915	12.59260349	up-regulated in High
RSPO2	-0.3224919	0.35051749	-7.4062694	5.63E-13	1.46E-11	17.8541748	12.24949694	up-regulated in High
EIF3E	0.12917121	5.69123713	2.19950033	0.02830344	0.08261112	-5.7793461	1.548160822	up-regulated in Low
TMEM74	0.12907136	0.21978435	3.74305462	0.0002031	0.00117205	-1.2728728	3.69228455	up-regulated in Low
NUDCD1	0.50129736	2.78594025	9.58040274	4.65E-20	3.07E-18	33.9640662	19.33261408	up-regulated in Low
ENY2	0.19732039	3.14653766	4.89901637	1.31E-06	1.22E-05	3.54367006	5.883918285	up-regulated in Low
SYBU	-0.5780406	1.82344082	-5.6074366	3.42E-08	4.27E-07	7.07236393	7.466464205	up-regulated in High
KCNV1	0.10158093	0.09899072	4.62264688	4.84E-06	4.00E-05	2.28396848	5.315237593	up-regulated in Low
TRPS1	0.15297767	1.251883	2.75314382	0.00611927	0.02298841	-4.4236125	2.21330033	up-regulated in Low
EIF3H	0.26785107	5.28024093	5.90726925	6.47E-09	9.15E-08	8.69227575	8.189144049	up-regulated in Low
UTP23	0.16874949	2.46565489	3.85800835	0.00012941	0.00078479	-0.8478373	3.888024746	up-regulated in Low
RAD21	0.50948028	5.24450785	9.51785915	7.74E-20	4.96E-18	33.4590805	19.11120404	up-regulated in Low
AARD	-0.2688079	0.63901325	-3.9309691	9.66E-05	0.00060511	-0.5717902	4.014830666	up-regulated in High
MED30	0.19993881	2.98168957	3.84173389	0.00013804	0.00083138	-0.9087478	3.860011703	up-regulated in Low
SAMD12	-0.2419394	1.752048	-4.6319648	4.63E-06	3.84E-05	2.32535041	5.333958717	up-regulated in High
MAL2	-0.2915935	6.76878899	-3.383072	0.00077356	0.0037945	-2.5249514	3.111505527	up-regulated in High
ENPP2	-0.3955865	3.05568688	-4.9026714	1.28E-06	1.20E-05	3.56077582	5.891624508	up-regulated in High
TAF2	0.36084034	2.7193903	8.08252945	4.89E-15	1.70E-13	22.5306289	14.31076582	up-regulated in Low
DSCC1	1.02030624	1.82459917	17.8119322	3.23E-55	2.57E-52	114.546055	54.49093821	up-regulated in Low
DEPTOR	-0.6347023	3.34212306	-7.0774813	5.04E-12	1.16E-10	15.6985511	11.29727949	up-regulated in High
COL14A1	-1.0008636	2.40694413	-10.103532	6.00E-22	4.94E-20	38.2752621	21.22172262	up-regulated in High
MRPL13	0.53687899	3.22308108	9.66321481	2.36E-20	1.60E-18	34.6361932	19.62726153	up-regulated in Low
MTBP	0.44250632	0.78363354	14.615809	1.75E-40	5.38E-38	80.7189917	39.75701348	up-regulated in Low
SNTB1	-0.4815902	3.49035733	-5.7604907	1.47E-08	1.96E-07	7.89005466	7.831502016	up-regulated in High
HAS2	-0.2139423	1.26038831	-2.965428	0.00316839	0.01302581	-3.8257498	2.499161853	up-regulated in High
ZHX2	-0.1639171	2.95695096	-3.3815378	0.00077779	0.00381302	-2.5300296	3.109135145	up-regulated in High
DERL1	0.23974242	4.01915505	6.18119667	1.33E-09	2.12E-08	10.2360892	8.876188983	up-regulated in Low
TBC1D31	0.52497093	1.01381997	15.6108987	5.67E-45	2.25E-42	91.0206259	44.24621617	up-regulated in Low
FAM83A	0.91295414	3.72684568	5.98478804	4.16E-09	6.10E-08	9.12302827	8.380995161	up-regulated in Low
C8orf76	0.45840519	2.30139668	9.12889154	1.75E-18	9.61E-17	30.370458	17.75632092	up-regulated in Low
ZHX1-C8orf7(	0.20238643	0.6885697	6.98624424	9.14E-12	2.03E-10	15.1145027	11.03901434	up-regulated in Low
ATAD2	1.00141096	2.80067421	15.0856707	1.37E-42	4.79E-40	85.5520166	41.86341822	up-regulated in Low
WDYHV1	0.40739752	2.77513638	8.10508484	4.15E-15	1.45E-13	22.6920596	14.38182765	up-regulated in Low
FBXO32	0.30032046	2.97398923	3.3321281	0.00092625	0.004446	-2.6924037	3.033270247	up-regulated in Low
ANXA13	0.13265992	0.26755675	2.18756457	0.02916781	0.08467685	-5.8053083	1.535096216	up-regulated in Low
FAM91A1	0.36406799	3.45991105	8.09023292	4.62E-15	1.61E-13	22.5857245	14.33501953	up-regulated in Low
TMEM65	0.37912184	2.21972035	6.24212664	9.28E-10	1.51E-08	10.5876473	9.032441605	up-regulated in Low
TRMT12	0.26913708	2.73493765	6.23703703	9.56E-10	1.56E-08	10.5581681	9.019342037	up-regulated in Low
TATDN1	0.35185529	2.24751878	7.70089002	7.40E-14	2.19E-12	19.8521175	13.13082106	up-regulated in Low
NDUFB9	0.42400516	5.57659524	6.55018201	1.44E-10	2.68E-09	12.4098675	9.841285749	up-regulated in Low
MTSS1	-0.3600938	2.62266948	-4.0890796	5.05E-05	0.00033795	0.04306235	4.296454627	up-regulated in High
ZNF572	0.10977075	0.8938948	2.49319694	0.01298505	0.04347985	-5.0969779	1.886556324	up-regulated in Low
SQLE	0.47876719	3.88369085	6.10258297	2.11E-09	3.26E-08	9.78686813	8.676424696	up-regulated in Low
KIAA0196	0.2519779	3.79721385	5.25450265	2.21E-07	2.38E-06	5.26116622	6.65582879	up-regulated in Low
NSMCE2	0.32887493	2.96735869	6.0728123	2.50E-09	3.82E-08	9.61804068	8.601317359	up-regulated in Low
TRIB1	-0.2202669	4.70382829	-3.0447053	0.00245298	0.01043816	-3.5914886	2.610306652	up-regulated in High
MYC	0.46017446	4.32096286	4.844411	1.70E-06	1.55E-05	3.28949589	5.769363878	up-regulated in Low
GSDMC	0.21282847	1.46730993	2.07301916	0.03868675	0.10636669	-6.0474109	1.412437792	up-regulated in Low
FAM49B	0.28257309	3.47288138	5.78384298	1.29E-08	1.74E-07	8.01650939	7.887908411	up-regulated in Low
ASAP1	0.34890044	2.77019682	5.76838379	1.41E-08	1.88E-07	7.93274662	7.850546524	up-regulated in Low
KCNQ3	-0.3956573	1.6972116	-4.7796989	2.32E-06	2.04E-05	2.99163465	5.635002529	up-regulated in High
TMEM71	-0.1221771	1.0185534	-2.0948092	0.03669508	0.10192263	-6.0023402	1.435392163	up-regulated in High
PHF20L1	0.18980718	1.73645849	5.39662487	1.05E-07	1.21E-06	5.97794014	6.977004379	up-regulated in Low
SLA	-0.3950271	2.25570828	-5.6596654	2.57E-08	3.27E-07	7.34922339	7.590120773	up-regulated in High
NDRG1	0.33235348	5.62690287	3.44102896	0.00062848	0.0031623	-2.3314983	3.201705469	up-regulated in Low
ST3GAL1	-0.3387129	3.3726138	-3.7169681	0.00022462	0.00128186	-1.3676361	3.648556062	up-regulated in High

ZFAT	0.14337227	1.37276603	3.09105658	0.00210671	0.00914462	-3.4517649	2.676395736	up-regulated in Low
COL22A1	0.17601409	0.60995868	2.71250318	0.00690994	0.02547037	-4.5331639	2.160525706	up-regulated in Low
KCNK9	0.10292082	0.15610464	2.60630467	0.00942803	0.03314712	-4.8119614	2.02557913	up-regulated in Low
TRAPPC9	0.16909597	2.86202284	3.21627271	0.00138353	0.00634617	-3.064165	2.859010391	up-regulated in Low
CHRAC1	0.32404051	3.82816195	7.62727088	1.24E-13	3.54E-12	19.3470945	12.90815025	up-regulated in Low
AGO2	0.35368908	2.262075	6.69012409	6.04E-11	1.18E-09	13.2620397	10.21899607	up-regulated in Low
DENND3	-0.267211	1.94615804	-5.152062	3.73E-07	3.85E-06	4.75513713	6.428760751	up-regulated in High
SLC45A4	0.24095296	2.5574127	3.30782044	0.00100859	0.00479859	-2.7714469	2.996286997	up-regulated in Low
ARC	-0.1831997	0.43045921	-4.3248851	1.85E-05	0.0001355	1.002041	4.733742947	up-regulated in High
JRK	0.14997299	1.36838492	3.48354788	0.00053866	0.00275955	-2.187584	3.268686653	up-regulated in Low
LY6K	0.51884406	1.28338817	4.20013043	3.16E-05	0.00022087	0.48845133	4.499823268	up-regulated in Low
LYNX1	-0.5118314	1.49331536	-5.8943473	6.96E-09	9.78E-08	8.6209453	8.157362359	up-regulated in High
LY6E	-0.5273853	6.93446035	-4.6276453	4.73E-06	3.91E-05	2.30615763	5.32527629	up-regulated in High
LY6H	-0.1010242	0.51179501	-2.159953	0.03125515	0.08962255	-5.8648369	1.505078415	up-regulated in High
GPIHBP1	-0.6021431	1.1211512	-7.540306	2.25E-13	6.21E-12	18.755463	12.64720612	up-regulated in High
ZNF696	0.13625978	1.60863183	3.08741525	0.00213219	0.00924149	-3.4628151	2.671174287	up-regulated in Low
TOP1MT	0.16889405	2.56841224	3.1292436	0.00185585	0.00818968	-3.3351263	2.731456839	up-regulated in Low
ZC3H3	0.27003235	3.32257089	5.1828766	3.19E-07	3.34E-06	4.9064134	6.496671777	up-regulated in Low
MROH6	-0.326177	3.15788334	-3.0402367	0.00248898	0.01057211	-3.6048516	2.603978259	up-regulated in High
EEF1D	0.10112405	4.02646185	1.99685938	0.04638692	0.12304166	-6.2012996	1.333604489	up-regulated in Low
TIGD5	0.22643491	1.74730817	5.1315723	4.13E-07	4.23E-06	4.65499664	6.383791359	up-regulated in Low
PYCRL	0.36618201	2.9799909	5.91452843	6.21E-09	8.82E-08	8.73240649	8.207022993	up-regulated in Low
ZNF623	0.13491707	2.6773708	2.91687383	0.00369599	0.01491038	-3.96628	2.432268944	up-regulated in Low
FAM83H	0.33135737	4.52644467	4.48064635	9.25E-06	7.21E-05	1.66281173	5.033866213	up-regulated in Low
SCRIB	0.33709387	4.26240596	4.50422752	8.32E-06	6.54E-05	1.76472835	5.080080407	up-regulated in Low
PUF60	0.41815391	4.88879718	8.64969669	7.22E-17	3.20E-15	26.6926851	16.14126385	up-regulated in Low
NRBP2	-0.2148741	3.05665256	-2.6383308	0.00859361	0.0306023	-4.7290245	2.065824132	up-regulated in High
EXOSC4	0.40147582	4.03263931	6.46078102	2.49E-10	4.47E-09	11.8733926	9.60333014	up-regulated in Low
GPAA1	0.13466607	5.39160616	2.29368595	0.02222617	0.06770947	-5.5696209	1.653135281	up-regulated in Low
CYC1	0.65738686	5.76362093	10.3590873	6.79E-23	6.20E-21	40.4362724	22.16796376	up-regulated in Low
SHARPIN	0.1462906	4.86664579	2.56553102	0.01059487	0.03651942	-4.916125	1.974904585	up-regulated in Low
MAF1	0.14243294	5.76614623	3.1971947	0.00147638	0.00671558	-3.1241741	2.830802907	up-regulated in Low
HGH1	0.24582513	3.74401971	4.37941206	1.45E-05	0.00010897	1.2308955	4.837789787	up-regulated in Low
BOP1	0.7467481	4.019357	10.8387638	1.04E-24	1.12E-22	44.5850307	23.98347664	up-regulated in Low
HSF1	0.35670758	4.32111131	7.43967858	4.49E-13	1.18E-11	18.0776175	12.3481168	up-regulated in Low
SLC52A2	0.37649665	4.8263657	5.9748925	4.40E-09	6.42E-08	9.06777038	8.356390975	up-regulated in Low
FBXL6	0.1401774	2.98439518	2.2204978	0.02683649	0.07916047	-5.7333369	1.571274215	up-regulated in Low
CPSF1	0.19349603	4.19699865	2.92587294	0.00359253	0.01454071	-3.9404031	2.444599365	up-regulated in Low
TONSL	0.68540765	2.00302714	11.4800343	3.26E-27	4.31E-25	50.3094818	26.48648062	up-regulated in Low
FOXH1	0.11207065	0.18828721	2.66764378	0.00788859	0.02844032	-4.6522508	2.103000749	up-regulated in Low
PPP1R16A	0.14177857	2.69878761	2.18698294	0.0292105	0.08478394	-5.8065699	1.534460943	up-regulated in Low
MFSD3	0.33698274	3.47234433	4.361103	1.57E-05	0.00011735	1.15375469	4.80273067	up-regulated in Low
RECQL4	1.01340619	2.56634861	13.1324509	5.13E-34	1.05E-31	65.889421	33.28981489	up-regulated in Low
LRRC14	0.1235824	2.64511728	2.47095359	0.01381062	0.04571205	-5.1515779	1.859786866	up-regulated in Low
C8orf82	0.15012972	3.09179735	2.48268651	0.01336953	0.04451795	-5.122837	1.873883875	up-regulated in Low
ARHGAP39	0.29933022	2.16739025	4.8119069	1.99E-06	1.78E-05	3.13942783	5.701686325	up-regulated in Low
ZNF34	-0.1037027	1.91696316	-2.4763358	0.0136067	0.04515486	-5.1384102	1.86624707	up-regulated in High
RPL8	0.28501524	9.50719674	4.10965109	4.64E-05	0.00031291	0.12472732	4.333781466	up-regulated in Low
COMMD5	0.19014651	3.53121339	3.66211251	0.00027705	0.00153986	-1.564861	3.557437783	up-regulated in Low
ZNF16	0.12455714	1.58831202	3.91001511	0.00010515	0.00065213	-0.6515673	3.978208627	up-regulated in Low
C8orf33	0.3402186	4.13868867	6.54504545	1.49E-10	2.76E-09	12.3788761	9.827543144	up-regulated in Low
DOCK8	-0.4851747	2.05548319	-7.4192985	5.15E-13	1.35E-11	17.9412186	12.28791681	up-regulated in High
KANK1	-0.2916685	1.90819867	-5.8583441	8.53E-09	1.18E-07	8.42292144	8.069113172	up-regulated in High
SMARCA2	-0.5578259	3.43955875	-9.3139377	4.02E-19	2.35E-17	31.8285558	18.39609457	up-regulated in High
KIAA0020	0.28033853	3.52890532	5.32219563	1.56E-07	1.73E-06	5.60043914	6.807916326	up-regulated in Low
GLIS3	-0.2445355	1.51813976	-4.0885284	5.06E-05	0.00033857	0.04087955	4.295456689	up-regulated in High
SLC1A1	-0.7669276	2.17289427	-8.3211409	8.51E-16	3.29E-14	24.2557303	15.06987683	up-regulated in High
SPATA6L	-0.2423005	0.67663468	-6.9407155	1.23E-11	2.67E-10	14.8253785	10.91111802	up-regulated in High
CDC37L1	-0.1854338	2.55035629	-5.0177783	7.30E-07	7.14E-06	4.10538776	6.136771722	up-regulated in High
AK3	-0.1934682	3.88507635	-4.1710717	3.58E-05	0.00024696	0.37083129	4.44616517	up-regulated in High
JAK2	-0.2366037	1.98089378	-4.4291598	1.17E-05	8.91E-05	1.44200488	4.933672969	up-regulated in High
INSL4	0.75590652	0.57900449	6.06460662	2.63E-09	3.99E-08	9.57163181	8.580668073	up-regulated in Low
ERMP1	0.38554156	2.91983532	5.87396573	7.81E-09	1.09E-07	8.50871364	8.107349772	up-regulated in Low
KIAA2026	-0.1415243	2.02826851	-3.3415367	0.0008961	0.00431865	-2.6616604	3.047645218	up-regulated in High
RANBP6	-0.1210611	2.66587678	-2.5149238	0.01222123	0.04125584	-5.0431852	1.912885008	up-regulated in High
IL33	-0.9702202	2.69259537	-9.7343231	1.31E-20	9.21E-19	35.2164773	19.88160632	up-regulated in High
UHRF2	0.13008804	2.11927242	2.76477012	0.00590863	0.02230472	-4.3919818	2.228513241	up-regulated in Low
GLDC	0.61993738	0.79182157	7.2991724	1.16E-12	2.90E-11	17.1433431	11.93566075	up-regulated in Low
KDM4C	-0.1639584	1.22023921	-5.8737894	7.82E-09	1.09E-07	8.50774404	8.106917657	up-regulated in High
KDM4C	-0.1639584	1.22023921	-5.8737894	7.82E-09	1.09E-07	8.50774404	8.106917657	up-regulated in High
PTPRD	-0.2485838	0.66150783	-5.932062	5.62E-09	8.04E-08	8.82951308	8.25028114	up-regulated in High

TYRP1	-0.2962249	0.62887926	-5.0602993	5.91E-07	5.88E-06	4.30945836	6.228532994	up-regulated in High
LURAP1L	-0.375968	3.1197428	-5.4711356	7.11E-08	8.42E-07	6.36051883	7.148227558	up-regulated in High
MPDZ	-0.113494	1.46778088	-2.2152083	0.02719969	0.08001755	-5.7449675	1.565436069	up-regulated in High
NFIB	-0.2885745	2.97029301	-4.0946778	4.94E-05	0.00033087	0.06524808	4.306596858	up-regulated in High
ZDHHC21	-0.4165943	2.36757348	-6.5995732	1.06E-10	2.02E-09	12.7089097	9.973868283	up-regulated in High
FREM1	-0.1103172	0.40654383	-2.8091725	0.00516315	0.0198787	-4.2699912	2.287085687	up-regulated in High
TTC39B	-0.3155525	1.69091161	-6.433508	2.94E-10	5.20E-09	11.7109724	9.531260444	up-regulated in High
PSIP1	0.17721392	3.02997293	3.03987418	0.00249192	0.01058306	-3.6059348	2.603465222	up-regulated in Low
BNC2	-0.1170491	0.57233005	-3.2750782	0.00113025	0.00529716	-2.8770418	2.94682483	up-regulated in High
SH3GL2	0.13885219	0.24591329	2.76178641	0.00596205	0.02247733	-4.4001116	2.224604186	up-regulated in Low
HAUS6	0.39987709	1.70144075	9.41918753	1.72E-19	1.06E-17	32.6670477	18.76387792	up-regulated in Low
PLIN2	0.37743058	3.85300368	4.09901876	4.85E-05	0.00032548	0.08247117	4.314469515	up-regulated in Low
DENND4C	-0.1366073	2.57820192	-2.5839335	0.01005316	0.03497449	-4.8693105	1.997697596	up-regulated in High
ACER2	-0.1487197	1.13620756	-2.5741775	0.01033718	0.03579254	-4.8941695	1.985598024	up-regulated in High
MLLT3	-0.2460325	2.06994122	-4.3635065	1.56E-05	0.00011624	1.16386411	4.807325927	up-regulated in High
PTPLAD2	-0.3096569	1.59293309	-6.0527918	2.81E-09	4.24E-08	9.50490555	8.550976368	up-regulated in High
IFNE	0.12404101	0.18340023	3.58118235	0.00037577	0.00201027	-1.8507574	3.4250777	up-regulated in Low
MTAP	0.139951	1.61301244	2.63933425	0.00856858	0.0305299	-4.72641	2.067091376	up-regulated in Low
CDKN2A	0.6368605	1.96134142	4.54875432	6.79E-06	5.45E-05	1.95851408	5.167899682	up-regulated in Low
CDKN2B	-0.2700149	2.56238859	-2.8604188	0.00440944	0.01734162	-4.1268582	2.355617059	up-regulated in High
ELAVL2	0.12797365	0.21443822	3.59684225	0.00035441	0.00190928	-1.7959097	3.450496577	up-regulated in Low
PLAA	0.21093752	2.99541279	4.65925544	4.08E-06	3.42E-05	2.44699026	5.388971815	up-regulated in Low
TEK	-0.4870396	1.54867939	-7.7505592	5.22E-14	1.58E-12	20.1949988	13.28196416	up-regulated in High
MOB3B	-0.1594592	1.58535162	-2.7411392	0.00634386	0.02370896	-4.4561368	2.197646235	up-regulated in High
LINGO2	0.16931272	0.11696609	6.11319064	1.98E-09	3.08E-08	9.84719509	8.703258515	up-regulated in Low
ACO1	-0.1436279	3.43920349	-2.277181	0.02320073	0.0702313	-5.6069951	1.634498274	up-regulated in High
NDUFB6	0.26885964	4.44205149	5.24175179	2.36E-07	2.53E-06	5.19769424	6.627362557	up-regulated in Low
APTX	0.21289328	2.95742095	5.82367411	1.04E-08	1.42E-07	8.23322855	7.984550692	up-regulated in Low
DNAJA1	0.31432706	6.0900027	6.50280925	1.93E-10	3.51E-09	12.1248207	9.714869303	up-regulated in Low
SMU1	0.14920084	3.33782331	4.12272944	4.39E-05	0.00029752	0.17684489	4.357593897	up-regulated in Low
B4GALT1	0.3025747	5.9533078	4.86890357	1.51E-06	1.39E-05	3.40318244	5.820612721	up-regulated in Low
CHMP5	0.17257743	5.32008245	3.48682367	0.00053226	0.00273181	-2.1764265	3.273875449	up-regulated in Low
AQP7	-0.3393286	0.80700184	-4.7341106	2.88E-06	2.48E-05	2.783991	5.541258934	up-regulated in High
AQP3	-1.388794	6.63758484	-8.5477995	1.56E-16	6.68E-15	25.9293808	15.80579715	up-regulated in High
NOL6	0.16566349	3.75825639	3.37521223	0.00079548	0.00388829	-2.5509447	3.09937109	up-regulated in Low
ANKRD18B	0.25335531	0.25279945	8.07836901	5.04E-15	1.75E-13	22.50089	14.29767415	up-regulated in Low
PRSS3	0.45058254	0.7881388	3.91452824	0.00010326	0.00064198	-0.6344186	3.986082504	up-regulated in Low
UBE2R2	0.22981354	4.5173411	5.80023459	1.18E-08	1.60E-07	8.10553814	7.927613454	up-regulated in Low
UBAP2	0.26614792	2.33023853	6.14481032	1.65E-09	2.59E-08	10.0275537	8.783470132	up-regulated in Low
KIF24	0.36142599	0.90282044	9.20620796	9.49E-19	5.35E-17	30.9771671	18.02256261	up-regulated in Low
NUDT2	0.30062442	3.29296699	4.82758051	1.84E-06	1.66E-05	3.21167648	5.734273009	up-regulated in Low
C9orf24	-0.6878088	1.15679805	-6.0283941	3.24E-09	4.84E-08	9.36747124	8.489812332	up-regulated in High
DNAI1	-0.3342599	0.55047701	-4.7980071	2.12E-06	1.89E-05	3.07553473	5.672861931	up-regulated in High
CNTFR	-0.1328984	0.25696406	-3.4438372	0.00062215	0.00313527	-2.3220453	3.206108258	up-regulated in High
RPP25L	0.23578055	3.98834439	4.55451758	6.62E-06	5.32E-05	1.98372463	5.179319519	up-regulated in Low
SIGMAR1	0.35422261	4.78637652	7.26071959	1.50E-12	3.71E-11	16.8901557	11.82383928	up-regulated in Low
GALT	-0.1276111	2.47702317	-3.205894	0.00143335	0.00655085	-3.0968533	2.843647992	up-regulated in High
IL11RA	-0.3930896	1.68579518	-7.8682744	2.27E-14	7.18E-13	21.0145008	13.64308482	up-regulated in High
CCL19	-1.064103	4.16961139	-6.808983	2.85E-11	5.90E-10	13.9976145	10.54477307	up-regulated in High
VCP	0.23086318	5.99511784	6.14579644	1.64E-09	2.57E-08	10.0331914	8.785977087	up-regulated in Low
FANCG	0.62308287	2.54201875	13.0072846	1.74E-33	3.51E-31	64.6716542	32.75842851	up-regulated in Low
PIGO	0.16559763	2.71251873	3.97499958	8.09E-05	0.00051591	-0.4028501	4.092319497	up-regulated in Low
STOML2	0.54313479	5.46860044	10.7410625	2.46E-24	2.55E-22	43.7304664	23.60962608	up-regulated in Low
UNC13B	-0.6440002	4.0707409	-7.317818	1.02E-12	2.58E-11	17.2665005	11.99004652	up-regulated in High
FAM166B	-0.4342229	1.02666493	-4.8833977	1.41E-06	1.30E-05	3.47070504	5.8510427	up-regulated in High
SIT1	-0.2809694	1.86326127	-3.410535	0.00070131	0.00348223	-2.4336742	3.154088461	up-regulated in High
CCDC107	-0.1702599	2.33285359	-3.5029362	0.00050182	0.00259494	-2.1214015	3.299456414	up-regulated in High
ARHGEF39	0.52332414	1.04568017	13.0664955	9.78E-34	2.00E-31	65.247021	33.00950322	up-regulated in Low
TLN1	-0.1489279	5.20478693	-2.5760215	0.01028295	0.03563361	-4.889478	1.987882162	up-regulated in High
RGP1	0.10834145	2.7519645	2.14065098	0.03278956	0.09303547	-5.9060099	1.484264458	up-regulated in Low
NPR2	-0.2983258	1.64000509	-6.4861262	2.14E-10	3.86E-09	12.0248522	9.67052469	up-regulated in High
SPAG8	-0.3122078	0.67237103	-5.643573	2.81E-08	3.55E-07	7.26367868	7.551919738	up-regulated in High
TMEM8B	-0.4416262	2.06025849	-8.8644541	1.39E-17	6.68E-16	28.3231435	16.85751695	up-regulated in High
HRCT1	-0.1767109	1.22412168	-2.613515	0.00923409	0.03254955	-4.7933749	2.034605957	up-regulated in High
RECK	-0.2856783	1.41284976	-6.1015524	2.12E-09	3.28E-08	9.7810118	8.673819645	up-regulated in High
GLIPR2	-0.353965	3.44252593	-4.2929559	2.12E-05	0.00015364	0.86926574	4.673326206	up-regulated in High
CLTA	0.10155376	5.59744435	2.29330483	0.02224827	0.0677591	-5.570487	1.652703767	up-regulated in Low
RNF38	-0.2388623	2.89228028	-5.2599058	2.15E-07	2.32E-06	5.2881038	6.667908599	up-regulated in High
MELK	1.36273559	2.32351528	17.1260309	5.49E-52	3.41E-49	107.12518	51.26021033	up-regulated in Low
PAX5	-0.1866244	0.59044286	-3.3041499	0.00102159	0.00485297	-2.7833347	2.990721808	up-regulated in High
ZCCHC7	0.11210706	2.70606343	2.75510354	0.0060833	0.02286798	-4.4182899	2.215861014	up-regulated in Low

POLR1E	0.16781421	3.00103477	3.7607851	0.0001896	0.0011023	-1.2081067	3.722151913	up-regulated in Low
TOMM5	0.43886408	2.50523505	10.2891918	1.24E-22	1.09E-20	39.8417449	21.90767994	up-regulated in Low
TRMT10B	-0.1778413	1.93182461	-4.2840052	2.21E-05	0.00015931	0.8322089	4.656457289	up-regulated in High
EXOSC3	0.33166406	2.58908755	8.47904573	2.62E-16	1.09E-14	25.4181492	15.58105787	up-regulated in Low
SHB	-0.1274877	2.96023717	-2.2678991	0.02376499	0.07163851	-5.6278973	1.624062436	up-regulated in High
ALDH1B1	0.24440422	3.73676937	3.59765509	0.00035333	0.00190436	-1.7930566	3.451818487	up-regulated in Low
ZNF658	-0.1280543	0.42151342	-7.4457258	4.31E-13	1.14E-11	18.1181479	12.36600396	up-regulated in High
PGM5	-0.5402314	1.0855988	-8.3939957	4.96E-16	1.99E-14	24.790014	15.30486277	up-regulated in High
TMEM252	-0.1317069	0.16415897	-5.0850777	5.22E-07	5.25E-06	4.42909296	6.282303626	up-regulated in High
PIP5K1B	-0.4354541	1.22358204	-6.3443726	5.04E-10	8.59E-09	11.1842001	9.297427153	up-regulated in High
FAM122A	-0.2058622	2.41711103	-6.7344489	4.57E-11	9.16E-10	13.5350902	10.33995327	up-regulated in High
FXN	0.15919467	1.81299782	5.4689146	7.19E-08	8.51E-07	6.34904757	7.143095562	up-regulated in Low
FAM189A2	-0.9307324	1.71403503	-11.850476	1.07E-28	1.59E-26	53.7032532	27.96945167	up-regulated in High
APBA1	-0.1854137	0.75574579	-5.4626592	7.44E-08	8.78E-07	6.31676143	7.128650798	up-regulated in High
PTAR1	-0.2094569	2.15832428	-4.7844578	2.27E-06	2.00E-05	3.01341471	5.644831655	up-regulated in High
C9orf135	-0.6162202	0.95898303	-5.7680413	1.41E-08	1.89E-07	7.9308931	7.849719715	up-regulated in High
MAMDC2	-0.9733495	1.517411	-11.738394	3.03E-28	4.42E-26	52.6699719	27.51800825	up-regulated in High
KLF9	-0.4642237	3.35342006	-6.9094806	1.50E-11	3.23E-10	14.6279254	10.82375505	up-regulated in High
ABHD17B	0.10028692	2.58851508	2.61675209	0.00914819	0.03229745	-4.7850144	2.038664914	up-regulated in Low
GDA	0.19068748	0.31944952	3.09626074	0.00207078	0.00901009	-3.4359504	2.683866934	up-regulated in Low
ZFAND5	-0.2182509	4.93731002	-4.3788726	1.46E-05	0.00010918	1.2286183	4.836755017	up-regulated in High
ALDH1A1	-0.3319837	4.52569585	-2.2091383	0.02762171	0.08100601	-5.7582807	1.558749477	up-regulated in High
ANXA1	-0.2810348	5.8038117	-2.1051612	0.03578001	0.099856	-5.9807661	1.446359541	up-regulated in High
C9orf40	0.52216948	1.61384872	11.8310045	1.29E-28	1.90E-26	53.5233486	27.89085499	up-regulated in Low
C9orf41	0.1403535	2.46755444	3.75620751	0.00019301	0.00111963	-1.2248555	3.714429542	up-regulated in Low
NMRK1	-0.214951	2.76885922	-4.4911477	8.82E-06	6.90E-05	1.70813699	5.054421408	up-regulated in High
PCSK5	-0.3122749	0.91242525	-6.9224367	1.38E-11	2.98E-10	14.7097389	10.85995515	up-regulated in High
RFK	0.29590582	3.34826142	4.61665107	4.97E-06	4.10E-05	2.2573809	5.303207882	up-regulated in Low
VPS13A	-0.1255361	1.48868322	-2.8796345	0.00415364	0.01646985	-4.0725425	2.381571316	up-regulated in High
GNA14	-0.5987308	2.42333384	-7.3529812	8.07E-13	2.06E-11	17.4994471	12.09290189	up-regulated in High
GNAQ	-0.3330873	3.76594504	-6.9854283	9.19E-12	2.04E-10	15.1093073	11.0367164	up-regulated in High
CEP78	0.27708481	1.1222712	9.07135815	2.76E-18	1.46E-16	29.9213529	17.55920803	up-regulated in Low
PSAT1	1.09975983	3.36703585	10.0763655	7.55E-22	6.13E-20	38.0476204	21.12202015	up-regulated in Low
TLE4	-0.3901874	1.59646064	-7.6378938	1.15E-13	3.31E-12	19.4197313	12.9401808	up-regulated in High
TLE1	0.17626502	2.95713342	3.1317452	0.00184042	0.00813079	-3.3274374	2.735083168	up-regulated in Low
RASEF	-0.2564858	2.83934644	-3.5800794	0.00037732	0.00201763	-1.8546118	3.423290913	up-regulated in High
IDNK	-0.2019637	1.9003472	-4.2658215	2.39E-05	0.00017098	0.75714751	4.622278598	up-regulated in High
UBQLN1	0.23795316	4.22064946	6.36268675	4.52E-10	7.75E-09	11.2919241	9.34525728	up-regulated in Low
HNRNPK	0.17969905	6.70699753	7.06573067	5.45E-12	1.25E-10	15.6229826	11.2638701	up-regulated in Low
RMI1	0.38385158	2.38205671	8.65297588	7.05E-17	3.13E-15	26.7173603	16.15210676	up-regulated in Low
SLC28A3	-0.2229865	0.93480777	-3.1092318	0.00198366	0.00868019	-3.3964221	2.702533246	up-regulated in High
NTRK2	-0.1170101	0.30849926	-2.3242098	0.02051763	0.0634186	-5.4998058	1.687872866	up-regulated in High
NAA35	0.27172476	2.71635338	7.3045179	1.12E-12	2.80E-11	17.1786252	11.95124165	up-regulated in Low
GOLM1	0.34055797	5.97784689	4.01043351	7.00E-05	0.000453	-0.2656124	4.155205297	up-regulated in Low
GAS1	-0.2445859	1.7460975	-2.7210554	0.00673629	0.02491838	-4.5102417	2.171579269	up-regulated in High
DAPK1	-0.5548197	3.27381578	-6.8600214	2.06E-11	4.34E-10	14.3167683	10.68605336	up-regulated in High
CTSL	0.53590199	5.95709004	6.24931329	8.89E-10	1.45E-08	10.6293077	9.050953235	up-regulated in Low
CDK20	-0.1026224	1.26098436	-2.6937983	0.00730387	0.02664654	-4.5830536	2.136446858	up-regulated in High
SPIN1	0.12690394	4.1341478	3.36408471	0.0008275	0.00402426	-2.5876462	3.082231611	up-regulated in Low
SHC3	-0.4121828	0.84491849	-7.9099577	1.69E-14	5.45E-13	21.306994	13.77193542	up-regulated in High
CKS2	1.12725605	5.26533909	13.9403822	1.68E-37	4.29E-35	73.880303	36.77546456	up-regulated in Low
SECISBP2	-0.1052983	2.53877441	-2.7262503	0.00663274	0.02459611	-4.4962839	2.178307083	up-regulated in High
SEMA4D	-0.1247928	2.21154183	-2.4889344	0.01313978	0.04390227	-5.1074779	1.881411965	up-regulated in High
GADD45G	-0.254409	3.57277732	-2.3185847	0.02082357	0.06421727	-5.5127394	1.681444895	up-regulated in High
DIRAS2	0.10219937	0.15083834	3.67770392	0.00026108	0.0014619	-1.5090868	3.583220817	up-regulated in Low
SYK	-0.3085776	3.66395798	-4.6841884	3.64E-06	3.07E-05	2.55869336	5.439469446	up-regulated in High
NFIL3	0.21868976	4.00907685	3.72258967	0.00021981	0.00125761	-1.3472677	3.657957844	up-regulated in Low
SPTLC1	0.12310887	3.93411299	2.86759069	0.00431233	0.01701705	-4.106627	2.365287605	up-regulated in Low
IARS	0.40602778	4.12313144	9.08342499	2.51E-18	1.34E-16	30.0153786	17.60047836	up-regulated in Low
NOL8	0.10341513	2.22432028	2.98435588	0.00298208	0.0123538	-3.7703604	2.525481303	up-regulated in Low
CENPP	0.12373389	0.51849105	7.41043111	5.47E-13	1.43E-11	17.8819645	12.26176317	up-regulated in Low
OGN	-0.8035157	1.08184528	-9.7938579	8.03E-21	5.79E-19	35.704534	20.0954985	up-regulated in High
OMD	-0.5792726	1.12250926	-7.3639506	7.50E-13	1.93E-11	17.5723002	12.12506613	up-regulated in High
ASPN	-0.613628	3.32688054	-5.241635	2.36E-07	2.54E-06	5.19711329	6.627101993	up-regulated in High
ECM2	-0.5131824	1.60293574	-8.5889352	1.15E-16	4.98E-15	26.2367195	15.94088226	up-regulated in High
IPPK	0.17699613	0.84454315	6.86009242	2.06E-11	4.34E-10	14.317214	10.68625059	up-regulated in Low
ZNF484	-0.204127	1.41452946	-4.2234718	2.86E-05	0.00020151	0.58348015	4.543150944	up-regulated in High
FGD3	-0.388406	1.78850444	-6.588607	1.14E-10	2.15E-09	12.6423518	9.944362886	up-regulated in High
SUSD3	-0.3446429	2.25432774	-4.8304415	1.82E-06	1.64E-05	3.22488754	5.740230845	up-regulated in High
NINJ1	-0.2233783	4.6169024	-3.8061682	0.0001588	0.00094047	-1.0410147	3.799138684	up-regulated in High
WNK2	0.26799621	0.93230191	4.05450816	5.83E-05	0.00038435	-0.0933166	4.234079944	up-regulated in Low

PHF2	-0.1614167	3.29889757	-3.6032087	0.00034605	0.00187101	-1.7735466	3.460856975	up-regulated in High
BARX1	0.78416792	1.61039888	4.16597501	3.66E-05	0.00025185	0.35027989	4.436786174	up-regulated in Low
FBP1	-0.8994093	5.70123126	-10.030679	1.11E-21	8.85E-20	37.6657022	20.95473654	up-regulated in High
C9orf3	-0.1756881	2.25580228	-3.3855203	0.00076685	0.00376783	-2.5168425	3.115290248	up-regulated in High
FANCC	0.24452136	1.33520561	7.28326328	1.29E-12	3.21E-11	17.0384608	11.88934143	up-regulated in Low
PTCH1	-0.290739	0.92320975	-7.0061073	8.03E-12	1.80E-10	15.2411262	11.09501746	up-regulated in High
ZNF367	0.64796885	1.38002478	13.1822928	3.15E-34	6.66E-32	66.3759131	33.50208604	up-regulated in Low
HABP4	-0.1330853	1.95225304	-3.0589885	0.00234107	0.01001599	-3.5486492	2.630585117	up-regulated in High
CDC14B	-0.1011995	1.61148922	-2.5236939	0.0119244	0.04039629	-5.0213426	1.923563394	up-regulated in High
AAED1	-0.1259913	2.60783588	-2.8669896	0.0043204	0.01704541	-4.1083246	2.364476318	up-regulated in High
CTSV	1.01017919	1.21406045	12.7722552	1.71E-32	3.29E-30	62.4005904	31.76727279	up-regulated in Low
NCBP1	0.31640932	3.02014689	8.22987494	1.67E-15	6.22E-14	23.5913953	14.7776153	up-regulated in Low
XPA	-0.1372379	2.71381869	-4.3089091	1.98E-05	0.00014427	0.93549214	4.70346604	up-regulated in High
ANP32B	0.36232337	6.25015805	9.08443816	2.49E-18	1.33E-16	30.0232773	17.60394527	up-regulated in Low
NANS	0.18640471	3.6004141	3.78267439	0.00017411	0.00102114	-1.1277507	3.759187789	up-regulated in Low
TRIM14	-0.1684736	3.34740932	-2.7338047	0.00648472	0.02414073	-4.4759403	2.188109005	up-regulated in High
CORO2A	-0.2540805	3.22574099	-3.642437	0.00029852	0.00164569	-1.6349247	3.525031648	up-regulated in High
TBC1D2	-0.3330344	3.74852208	-4.1173228	4.49E-05	0.00030378	0.15528042	4.34774198	up-regulated in High
GABBR2	-0.372855	0.5756863	-4.777472	2.34E-06	2.06E-05	2.98144932	5.630405745	up-regulated in High
ANKS6	-0.1381784	2.23776091	-3.1388261	0.00179738	0.00796354	-3.3056414	2.74536054	up-regulated in High
GALNT12	-0.4742339	3.1057948	-6.0848365	2.34E-09	3.59E-08	9.68614331	8.631616713	up-regulated in High
SEC61B	0.2501876	6.00258094	5.72364777	1.81E-08	2.37E-07	7.69145766	7.742891482	up-regulated in Low
NR4A3	-0.3974745	1.28236942	-4.5386993	7.11E-06	5.68E-05	1.91459994	5.148004794	up-regulated in High
INVS	0.11610483	1.46330222	4.07159837	5.43E-05	0.00036097	-0.0260337	4.264858908	up-regulated in Low
TEX10	0.28215582	2.06115541	8.74032534	3.61E-17	1.65E-15	27.3771758	16.44200945	up-regulated in Low
MSANTD3	0.39115956	2.87900129	8.96084129	6.56E-18	3.31E-16	29.0643703	17.18299788	up-regulated in Low
MURC	0.12616806	0.30102769	5.28817092	1.86E-07	2.04E-06	5.42942467	6.731269678	up-regulated in Low
RP11-35N6.1	-0.4054209	1.04292807	-4.4930627	8.75E-06	6.85E-05	1.71641291	5.058174156	up-regulated in High
BAAT	-0.3933704	0.81668052	-4.1205648	4.43E-05	0.00030006	0.16820818	4.353648286	up-regulated in High
MRPL50	0.12219728	2.99492687	3.08573269	0.00214406	0.00928467	-3.4679168	2.668763305	up-regulated in Low
ZNF189	-0.2486788	2.82443802	-5.3183065	1.59E-07	1.76E-06	5.58084203	6.799134642	up-regulated in High
ALDOB	-0.2967953	0.49098392	-3.6887404	0.00025031	0.00140892	-1.4694709	3.601526873	up-regulated in High
SMC2	0.64661347	2.2894743	12.6412257	6.05E-32	1.12E-29	61.1435243	31.21856535	up-regulated in Low
NIPSNAP3B	-0.1603838	0.77219098	-4.9058692	1.26E-06	1.18E-05	3.57575091	5.898370525	up-regulated in High
ABCA1	-0.1502931	2.45727078	-2.1310305	0.0335782	0.09485825	-5.9263958	1.473942604	up-regulated in High
SLC44A1	0.23176101	3.52223643	4.49753957	8.57E-06	6.72E-05	1.73577326	5.066952674	up-regulated in Low
FKTN	0.14583255	1.73478049	4.23044802	2.78E-05	0.00019619	0.61197731	4.556139842	up-regulated in Low
TMEM38B	0.4692653	1.85733583	8.16416563	2.70E-15	9.79E-14	23.1165362	14.56865591	up-regulated in Low
ZNF462	-0.273596	1.34502495	-5.0399329	6.54E-07	6.45E-06	4.2115201	6.184501018	up-regulated in High
RAD23B	0.35041237	5.38627	8.6415194	7.69E-17	3.40E-15	26.6311829	16.11423775	up-regulated in Low
FAM206A	0.10043045	3.01189979	2.40749504	0.01642689	0.05277325	-5.3047224	1.784444702	up-regulated in Low
CTNNAL1	0.19406778	3.53947954	3.13875016	0.00179784	0.00796496	-3.3058754	2.745250219	up-regulated in Low
TMEM245	-0.2481804	3.53880921	-4.540113	7.07E-06	5.65E-05	1.92076875	5.150799724	up-regulated in High
PTPN3	-0.1387545	2.33238873	-2.4849391	0.01328629	0.04429547	-5.1173038	1.876596341	up-regulated in High
C9orf152	-0.6818532	2.77176817	-5.8855877	7.31E-09	1.02E-07	8.57266885	8.135850517	up-regulated in High
TXN	0.56883995	7.31000149	7.83293212	2.92E-14	9.12E-13	20.7674449	13.53423469	up-regulated in Low
SVEP1	-0.5817492	1.19313159	-8.760996	3.08E-17	1.42E-15	27.5340281	16.51091516	up-regulated in High
KIAA0368	0.11743935	3.86534926	2.53398633	0.01158424	0.03943843	-4.9956141	1.936132417	up-regulated in Low
PTGR1	0.54410661	3.68200806	5.416277	9.51E-08	1.10E-06	6.07839262	7.021975192	up-regulated in Low
DNAJC25	0.11602279	2.32566752	3.27140099	0.00114474	0.00535987	-2.8888383	2.941295146	up-regulated in Low
UGCG	-0.2103212	4.22682832	-3.7287842	0.00021462	0.00123082	-1.3247897	3.668331632	up-regulated in High
SUSD1	-0.1795136	2.62232749	-3.497348	0.00051219	0.00264143	-2.1405128	3.290573202	up-regulated in High
PTBP3	0.17695138	4.32410882	3.80879675	0.00015717	0.00093242	-1.0312791	3.803621344	up-regulated in Low
HSDL2	-0.2824568	3.85889263	-5.1739753	3.33E-07	3.49E-06	4.86263139	6.477019778	up-regulated in High
SNX30	-0.8043196	2.63520681	-10.361346	6.66E-23	6.09E-21	40.4555323	22.17639521	up-regulated in High
SLC46A2	-0.7306086	1.19886504	-7.4207309	5.10E-13	1.34E-11	17.9507951	12.2921436	up-regulated in High
SLC31A2	-0.1118169	0.90497403	-2.8156696	0.00506152	0.0195351	-4.2519831	2.295719083	up-regulated in High
FKBP15	-0.1520889	2.70132692	-3.9708668	8.22E-05	0.00052369	-0.4187822	4.085015456	up-regulated in High
PRPF4	0.43597988	3.48945008	11.6004234	1.08E-27	1.51E-25	51.4056719	26.96555223	up-regulated in Low
RNF183	0.30037057	0.70830688	3.71090784	0.00022991	0.00130826	-1.389561	3.638434039	up-regulated in Low
ALAD	-0.298757	3.53087469	-7.1673583	2.79E-12	6.66E-11	16.279934	11.55424699	up-regulated in High
POLE3	0.33915173	4.74932473	7.34755704	8.38E-13	2.13E-11	17.4634551	12.07701107	up-regulated in Low
RGS3	-0.3884	2.5336201	-9.11701	1.93E-18	1.05E-16	30.2775449	17.71554353	up-regulated in High
KIF12	-0.5460177	1.95497819	-4.6574694	4.12E-06	3.45E-05	2.43900927	5.385363067	up-regulated in High
COL27A1	-0.2512428	1.41571729	-3.5963345	0.00035508	0.00191256	-1.7976916	3.44967096	up-regulated in High
ORM1	-0.3614673	2.31288915	-2.1724056	0.03029837	0.08739407	-5.8380817	1.518580772	up-regulated in High
ORM2	-0.345939	1.85699104	-2.3004694	0.02183611	0.06677901	-5.5541837	1.660824791	up-regulated in High
AKNA	-0.3925057	2.55664256	-6.5361472	1.57E-10	2.90E-09	12.3252371	9.80375672	up-regulated in High
C9orf91	-0.1341063	2.6853518	-2.6819018	0.00756482	0.02745102	-4.6146094	2.121201611	up-regulated in High
TNFSF15	-0.5108929	1.79227105	-6.2965155	6.71E-10	1.12E-08	10.9039507	9.172965886	up-regulated in High
TNFSF8	-0.3393555	1.04242417	-6.9846148	9.24E-12	2.05E-10	15.1041285	11.03442576	up-regulated in High

TLR4	-0.3574058	2.02018855	-4.8759579	1.46E-06	1.35E-05	3.43602315	5.835413624	up-regulated in High
BRINP1	-0.3353639	0.86386927	-2.8664772	0.00432728	0.01707142	-4.1097714	2.363784849	up-regulated in High
CDK5RAP2	0.1443936	2.79970263	2.55574929	0.01089328	0.0374265	-4.9408763	1.962841337	up-regulated in Low
MEGF9	-0.524152	3.56047053	-6.688741	6.09E-11	1.19E-09	13.2535435	10.21523184	up-regulated in High
PHF19	0.41036875	2.43635391	8.03722796	6.78E-15	2.31E-13	22.2074457	14.16848372	up-regulated in Low
TRAF1	-0.2636572	2.10744414	-4.1152128	4.53E-05	0.00030627	0.14687189	4.343900145	up-regulated in High
C5	-0.3046137	2.12100502	-2.400254	0.01675157	0.05362835	-5.3219498	1.775944499	up-regulated in High
RAB14	-0.1120849	4.93739995	-2.9391209	0.00344503	0.01402008	-3.9021684	2.462807486	up-regulated in High
GSN	-0.6615723	5.26969343	-9.634901	2.98E-20	2.00E-18	34.4059445	19.52633071	up-regulated in High
STOM	-0.547371	6.24522814	-7.6815987	8.47E-14	2.49E-12	19.7194106	13.0723154	up-regulated in High
DAB2IP	-0.2782368	3.06357029	-4.8844279	1.40E-06	1.30E-05	3.47551122	5.853208427	up-regulated in High
TTLL11	-0.1791422	0.99175749	-5.4734329	7.02E-08	8.33E-07	6.37238833	7.153537587	up-regulated in High
NDUFA8	0.27459817	5.42444306	6.52755242	1.66E-10	3.05E-09	12.2734857	9.780806089	up-regulated in Low
MORN5	-0.4674539	0.81540266	-4.8769885	1.45E-06	1.34E-05	3.44082445	5.837577386	up-regulated in High
MRRF	0.12364981	1.75697912	3.89059456	0.00011366	0.00069837	-0.7251484	3.944413325	up-regulated in Low
PTGS1	-0.2923129	2.12182034	-4.0763299	5.33E-05	0.00035462	-0.0073591	4.273399554	up-regulated in High
RC3H2	0.11357463	2.38167087	2.61604532	0.00916688	0.03234976	-4.7868407	2.037778349	up-regulated in Low
STRBP	0.18534106	2.42584569	4.35378259	1.63E-05	0.00012085	1.12299562	4.788747757	up-regulated in Low
DENND1A	0.24526924	1.91859055	5.03186444	6.81E-07	6.69E-06	4.17281908	6.167098214	up-regulated in Low
LHX2	0.28373088	0.25775737	6.31038158	6.18E-10	1.04E-08	10.9849642	9.208949	up-regulated in Low
NEK6	-0.1706704	4.24469285	-3.2318537	0.00131175	0.00605207	-3.0149017	2.882149848	up-regulated in High
PSMB7	0.35744866	5.78706374	9.81304545	6.85E-21	5.00E-19	35.8622587	20.16461635	up-regulated in Low
OLFML2A	-0.1675215	2.07003535	-2.1617621	0.03111455	0.08932469	-5.8609593	1.507036422	up-regulated in High
WDR38	-0.5575293	0.98754048	-5.1602279	3.57E-07	3.71E-06	4.79514653	6.446724298	up-regulated in High
RPL35	0.2249575	7.90598932	3.16560592	0.00164285	0.00736439	-3.2227819	2.784401445	up-regulated in Low
ARPC5L	0.15619756	4.09069864	3.76433239	0.00018701	0.00108861	-1.1951145	3.728141563	up-regulated in Low
SCAI	-0.1761714	0.99492288	-5.4728659	7.04E-08	8.35E-07	6.36945808	7.1522267	up-regulated in High
RABEPK	0.22719029	2.77310849	6.37750088	4.13E-10	7.13E-09	11.3792538	9.384027766	up-regulated in Low
HSPA5	0.12075743	7.93682449	2.562736	0.01067938	0.03677166	-4.9232068	1.971453952	up-regulated in Low
MVB12B	-0.3693475	1.96577339	-6.1694904	1.42E-09	2.26E-08	10.1688838	8.846310957	up-regulated in High
ZBTB43	-0.2182952	1.93769459	-5.2767997	1.97E-07	2.15E-06	5.37248892	6.705745091	up-regulated in High
ZBTB34	-0.1149446	1.68589367	-3.1478092	0.00174411	0.00775578	-3.2779222	2.758426192	up-regulated in High
RALGPS1	-0.3144618	1.34866751	-7.3821082	6.63E-13	1.71E-11	17.6930846	12.17838814	up-regulated in High
GARNL3	-0.1696668	0.5288553	-7.4176957	5.21E-13	1.36E-11	17.9305043	12.28318779	up-regulated in High
LRSAM1	-0.1277618	2.73658077	-2.9015984	0.00387785	0.01552602	-4.0100281	2.411409263	up-regulated in High
FAM129B	-0.169515	6.27720087	-2.7639211	0.00592379	0.02235218	-4.3942961	2.227400506	up-regulated in High
STXBP1	-0.7323573	3.45952287	-8.5810376	1.22E-16	5.27E-15	26.1776288	15.91491129	up-regulated in High
C9orf117	-0.2411775	0.87271378	-3.5467021	0.00042713	0.00225211	-1.9707209	3.369436046	up-regulated in High
TTC16	-0.1735684	0.26142478	-5.5261436	5.30E-08	6.43E-07	6.64594093	7.275881112	up-regulated in High
SH2D3C	-0.4113155	2.17756319	-7.2573301	1.53E-12	3.78E-11	16.86789	11.81400455	up-regulated in High
ENG	-0.5761052	5.10976411	-8.3430961	7.24E-16	2.84E-14	24.4163707	15.14053459	up-regulated in High
AK1	-0.6610919	2.78635551	-8.8891849	1.15E-17	5.58E-16	28.512772	16.9407928	up-regulated in High
;T6GALNAC	-0.6385644	3.5048987	-13.531723	9.98E-36	2.36E-33	69.8110345	35.00069694	up-regulated in High
;T6GALNAC	-0.3406072	3.93215212	-4.2469483	2.59E-05	0.00018387	0.67955319	4.586933097	up-regulated in High
PIP5KL1	-0.463122	1.77176598	-6.1364573	1.73E-09	2.71E-08	9.97983039	8.76224784	up-regulated in High
NAIF1	0.17119452	1.98452505	5.95878771	4.83E-09	7.00E-08	8.97800858	8.316419258	up-regulated in Low
PTGES2	0.36310706	3.95806077	7.92298925	1.54E-14	4.99E-13	21.3986825	13.81232242	up-regulated in Low
CIZ1	0.11483664	4.16665234	2.75628752	0.00606166	0.02280131	-4.4150724	2.217408767	up-regulated in Low
DNM1	0.19042106	1.22798491	3.14023836	0.00178891	0.00793081	-3.3012886	2.747412606	up-regulated in Low
SWI5	0.15714759	3.50605232	3.41591447	0.00068792	0.00342271	-2.4157122	3.162463099	up-regulated in Low
TRUB2	0.23170109	2.90086328	5.99888045	3.84E-09	5.66E-08	9.20185842	8.416091708	up-regulated in Low
SLC27A4	0.29834156	3.74818291	6.06943714	2.55E-09	3.89E-08	9.59894525	8.592821152	up-regulated in Low
URM1	0.15184836	3.65593521	4.34215765	1.71E-05	0.00012662	1.07424812	4.766583305	up-regulated in Low
ODF2	0.20771085	2.98116178	4.46890931	9.75E-06	7.57E-05	1.61226868	5.010940124	up-regulated in Low
WDR34	0.35134605	4.94684623	5.64111307	2.84E-08	3.60E-07	7.25062096	7.546088131	up-regulated in Low
SET	0.40743795	6.01471877	9.455525	1.28E-19	8.03E-18	32.9580611	18.89150329	up-regulated in Low
PKN3	0.21469686	2.22617373	4.04549716	6.05E-05	0.000397	-0.1286856	4.217895213	up-regulated in Low
ZDHHC12	0.23132403	4.06299703	4.27473458	2.30E-05	0.0001651	0.79390306	4.639016574	up-regulated in Low
ZER1	-0.2208495	4.04648614	-5.2516681	2.24E-07	2.42E-06	5.24704425	6.649495669	up-regulated in High
PHYHD1	-0.8481306	2.44361872	-7.7695108	4.57E-14	1.39E-12	20.3262828	13.33982667	up-regulated in High
NUP188	0.22622913	3.30556977	4.55277666	6.67E-06	5.36E-05	1.97610611	5.175868612	up-regulated in Low
SH3GLB2	-0.1156575	3.4872723	-2.0797122	0.03806541	0.10499207	-6.0336163	1.419469532	up-regulated in High
DOLPP1	0.2095393	3.52832538	5.03909241	6.57E-07	6.47E-06	4.20748604	6.182687096	up-regulated in Low
CRAT	-0.1675544	3.58140924	-3.220818	0.00136223	0.0062622	-3.0498174	2.865751178	up-regulated in High
IER5L	0.46850118	2.54358062	5.4817584	6.72E-08	7.99E-07	6.41544061	7.172796715	up-regulated in Low
NTMT1	0.34140195	2.84175655	8.12718747	3.54E-15	1.25E-13	22.8505841	14.45160471	up-regulated in Low
PTGES	0.46623406	4.0376547	3.7176644	0.00022402	0.00127856	-1.3651147	3.649720005	up-regulated in Low
USP20	-0.1450062	2.78725622	-3.1839228	0.00154432	0.00699462	-3.1657182	2.811261443	up-regulated in High
FNBP1	-0.17794	3.1214109	-3.0043511	0.00279619	0.01168239	-3.7114781	2.553432814	up-regulated in High
NCS1	0.22097842	2.27560817	3.14991761	0.00173182	0.00770817	-3.2714052	2.761497257	up-regulated in Low
HMCN2	-0.1730939	0.25498601	-8.3056281	9.55E-16	3.67E-14	24.1424203	15.02003435	up-regulated in High

ASS1	-0.3520089	5.30553883	-3.0232267	0.00263052	0.0110822	-3.6555455	2.579958376	up-regulated in High
FUBP3	0.19835471	3.49927955	5.68599441	2.22E-08	2.86E-07	7.48964548	7.652815256	up-regulated in Low
EXOSC2	0.42140508	2.64048089	11.6807838	5.17E-28	7.41E-26	52.1410271	27.28688829	up-regulated in Low
FIBCD1	0.18286394	0.62844283	2.59205095	0.00982217	0.03433437	-4.8485568	2.007792583	up-regulated in Low
LAMC3	-0.1531038	1.53731056	-2.0240808	0.04349783	0.11691331	-6.1469464	1.361532411	up-regulated in High
FAM78A	-0.2892292	1.58270808	-5.2027776	2.88E-07	3.04E-06	5.00454206	6.540710114	up-regulated in High
MED27	0.4137612	3.12976809	8.86576933	1.37E-17	6.63E-16	28.3332185	16.86194155	up-regulated in Low
SETX	-0.1256079	2.93728985	-2.7637094	0.00592757	0.02236321	-4.3948728	2.227123221	up-regulated in High
C9orf171	-0.3686153	0.51189045	-5.380153	1.15E-07	1.31E-06	5.89399345	6.939415514	up-regulated in High
AK8	-0.2924151	0.99143808	-5.3898949	1.09E-07	1.25E-06	5.94361415	6.961635025	up-regulated in High
C9orf9	-0.309498	2.06920909	-4.7844422	2.27E-06	2.00E-05	3.01334347	5.644799507	up-regulated in High
TSC1	-0.1157059	2.20380773	-2.7161342	0.00683573	0.02523984	-4.5234404	2.165215318	up-regulated in High
GTF3C5	0.33813637	3.88143879	7.9390512	1.37E-14	4.49E-13	21.5118538	13.86216944	up-regulated in Low
RALGDS	-0.2041521	3.4845886	-4.0432738	6.11E-05	0.00040012	-0.1374013	4.213906423	up-regulated in High
GBGT1	-0.3274006	1.94905685	-6.1528086	1.57E-09	2.48E-08	10.0733024	8.803813067	up-regulated in High
ABO	-0.8112883	2.43864312	-7.8660584	2.31E-14	7.28E-13	20.9989844	13.63624889	up-regulated in High
SURF2	0.13989504	3.82182467	2.78322158	0.00558776	0.02127781	-4.3415172	2.252762174	up-regulated in Low
REXO4	0.21407733	3.4971407	6.13220885	1.77E-09	2.77E-08	9.95557914	8.751462928	up-regulated in Low
CACFD1	-0.3525127	3.78183592	-6.0004673	3.80E-09	5.62E-08	9.21074508	8.420047951	up-regulated in High
ADAMTSL2	-0.7584419	2.22496085	-10.405794	4.54E-23	4.21E-21	40.8350029	22.34251057	up-regulated in High
SARDH	-0.1998305	1.00207435	-4.2613607	2.43E-05	0.0001739	0.73877882	4.613912597	up-regulated in High
VAV2	0.14549686	3.5446405	2.41752638	0.01598626	0.05163696	-5.2807727	1.796253205	up-regulated in Low
WDR5	0.34205783	3.729743	8.41035823	4.39E-16	1.77E-14	24.9104905	15.35784272	up-regulated in Low
RXRA	-0.2773704	3.09670345	-5.39626	1.06E-07	1.21E-06	5.97607836	6.976170799	up-regulated in High
FCN1	-0.4816849	1.56448032	-5.9298182	5.69E-09	8.14E-08	8.81707221	8.244739452	up-regulated in High
OLFM1	-0.5807929	1.26259097	-7.5698224	1.84E-13	5.13E-12	18.9556636	12.735517	up-regulated in High
C9orf116	-0.1418237	1.65577752	-2.0442641	0.04145545	0.11252193	-6.106179	1.382418404	up-regulated in High
MRPS2	0.33786232	3.99811891	7.41617041	5.26E-13	1.37E-11	17.9203094	12.27868798	up-regulated in Low
PAEP	0.60913533	2.58318116	2.29607775	0.02208795	0.06735868	-5.564183	1.655844574	up-regulated in Low
UBAC1	0.22756461	3.64011537	5.91441196	6.21E-09	8.82E-08	8.73176225	8.206735983	up-regulated in Low
NACC2	-0.1225346	2.47021236	-2.3845674	0.01747439	0.05553435	-5.3590965	1.757598048	up-regulated in High
C9orf69	0.17540337	3.80064117	4.04184162	6.15E-05	0.00040205	-0.1430129	4.211338106	up-regulated in Low
QSOX2	0.36795199	2.73835002	7.68956678	8.01E-14	2.36E-12	19.7741922	13.09646718	up-regulated in Low
MGC50722	-0.124776	0.43793274	-2.9112451	0.00376208	0.01514037	-3.9824261	2.424572246	up-regulated in High
CARD9	-0.1265821	0.90603317	-2.7349886	0.00646179	0.02406766	-4.4727472	2.189647075	up-regulated in High
SDCCAG3	0.21859945	3.62488102	5.29970958	1.75E-07	1.92E-06	5.48731037	6.757216702	up-regulated in Low
PMPCA	0.17420688	3.15344297	4.51633587	7.87E-06	6.22E-05	1.81725152	5.10388951	up-regulated in Low
NOTCH1	-0.1983161	2.65043639	-3.1227981	0.00189616	0.00834172	-3.3549102	2.72212432	up-regulated in High
EGFL7	-0.2117081	4.02617059	-2.207642	0.02772661	0.08123205	-5.7615571	1.557103248	up-regulated in High
AGPAT2	-0.3423462	5.98389395	-4.0125935	6.93E-05	0.00044945	-0.2572099	4.159053788	up-regulated in High
TMEM141	0.29386178	5.07913886	3.9601116	8.59E-05	0.00054352	-0.4601712	4.066037205	up-regulated in Low
RABL6	0.15537349	3.42768609	3.32050117	0.00096482	0.0046118	-2.730281	3.015552356	up-regulated in Low
TRAF2	0.34422645	3.2319985	5.79933983	1.19E-08	1.61E-07	8.10067272	7.925443725	up-regulated in Low
FBXW5	0.15182537	5.20414322	2.55601293	0.01088514	0.03741174	-4.9402104	1.963165994	up-regulated in Low
C8G	0.25132357	0.7971588	3.79850877	0.00016365	0.00096631	-1.069348	3.786091134	up-regulated in Low
PTGDS	-1.208732	4.31212734	-9.7764555	9.27E-21	6.60E-19	35.5616633	20.03288767	up-regulated in High
C9orf142	0.1262808	4.30394582	2.1198749	0.03451301	0.09698515	-5.9499218	1.462017219	up-regulated in Low
CLIC3	-0.9325778	3.49095502	-7.6494529	1.06E-13	3.08E-12	19.4988595	12.97507231	up-regulated in High
ABCA2	-0.2058505	2.43107963	-3.3820759	0.00077631	0.00380637	-2.5282486	3.109966452	up-regulated in High
FUT7	-0.1548041	0.48345513	-4.7745426	2.37E-06	2.09E-05	2.96805808	5.624361852	up-regulated in High
NPDC1	-0.3627098	3.71894491	-3.6823914	0.00025645	0.00143866	-1.4922747	3.59099026	up-regulated in High
ENTPD2	0.16033179	1.14012938	1.98974291	0.04716839	0.12463325	-6.2153897	1.326348938	up-regulated in Low
SAPCD2	1.04885968	2.21164664	14.0767083	4.24E-38	1.11E-35	75.249617	37.37256412	up-regulated in Low
DPP7	-0.3764313	5.26738802	-5.5894309	3.77E-08	4.68E-07	6.97744143	7.424053445	up-regulated in High
SSNA1	0.19054648	5.34244181	3.6894693	0.00024961	0.0014054	-1.4668507	3.602737449	up-regulated in Low
NDOR1	0.15073164	2.61754903	2.77016266	0.00581317	0.02200481	-4.3772669	2.235586753	up-regulated in Low
CYSRT1	0.19581058	1.16305671	2.69120778	0.00735999	0.02681442	-4.5899366	2.133122554	up-regulated in Low
TUBB4B	0.27800763	7.17355359	4.6629879	4.01E-06	3.37E-05	2.46367746	5.396516896	up-regulated in Low
NELFB	0.11522563	4.84276979	2.72135121	0.00673035	0.02490116	-4.5094477	2.171962077	up-regulated in Low
TOR4A	-0.1650781	3.37078514	-2.2625976	0.0240926	0.07245405	-5.6397984	1.618116394	up-regulated in High
NRARP	0.50166953	1.8267938	7.60995733	1.39E-13	3.95E-12	19.2288807	12.85601859	up-regulated in Low
EXD3	-0.1423577	1.40607423	-3.0897148	0.00211606	0.0091791	-3.4558381	2.674471171	up-regulated in High
PNPLA7	-0.2553514	1.00383216	-3.9413267	9.27E-05	0.00058239	-0.5322082	4.032993698	up-regulated in High
MRPL41	0.17894176	4.4601918	2.7141278	0.00687665	0.02536366	-4.5288149	2.16262336	up-regulated in Low
DPH7	0.12862328	2.3979868	2.79754961	0.00534957	0.02049658	-4.3021057	2.271681272	up-regulated in Low
ZMYND19	0.35762446	3.16450996	7.77374161	4.44E-14	1.35E-12	20.3556251	13.35275851	up-regulated in Low
ARRDC1	-0.1222542	4.00853401	-2.161608	0.03112651	0.08935022	-5.8612897	1.50686959	up-regulated in High
EHMT1	0.11561316	2.16937022	2.98878606	0.00293994	0.0122061	-3.757347	2.531661193	up-regulated in Low
GTPBP4	0.60062312	3.36191309	12.6470526	5.72E-32	1.07E-29	61.1992855	31.2429064	up-regulated in Low
IDI1	0.19470278	4.12412685	2.97843235	0.00303928	0.01255779	-3.7877312	2.517229936	up-regulated in Low
WDR37	-0.1609521	2.27273544	-4.0558901	5.80E-05	0.00038274	-0.0878859	4.236564714	up-regulated in High

PFKP	0.93135845	4.84059982	9.52075895	7.56E-20	4.86E-18	33.4824435	19.12144816	up-regulated in Low
PITRM1	0.20098939	3.66297885	4.23464176	2.73E-05	0.0001929	0.62912938	4.563956776	up-regulated in Low
KLF6	-0.1550996	4.69600394	-2.1744997	0.03013997	0.08701887	-5.8335676	1.520857139	up-regulated in High
AKR1E2	0.3169464	1.14198914	4.92605935	1.15E-06	1.08E-05	3.67050565	5.941048855	up-regulated in Low
AKR1C2	1.53697247	2.64315493	5.85292459	8.79E-09	1.21E-07	8.39320454	8.055867481	up-regulated in Low
AKR1C1	0.9024908	2.58508642	3.95102751	8.91E-05	0.00056179	-0.4950473	4.050041414	up-regulated in Low
AKR1C3	0.81801268	3.65883813	4.17837598	3.47E-05	0.00024013	0.40032488	4.459623276	up-regulated in Low
AKR1C4	0.44541048	0.37681729	5.46173405	7.47E-08	8.82E-07	6.31198947	7.126515748	up-regulated in Low
UCN3	-0.4137512	1.08762327	-2.9263665	0.00358694	0.0145226	-3.9389816	2.445276531	up-regulated in High
TUBAL3	0.20902946	0.36356624	4.42074969	1.21E-05	9.22E-05	1.40616099	4.917399452	up-regulated in Low
CALML3	0.29567112	0.32625593	3.301164	0.00103229	0.00489621	-2.7929956	2.986198511	up-regulated in Low
FAM208B	0.25526325	2.70796874	5.48410313	6.63E-08	7.90E-07	6.42757606	7.178225116	up-regulated in Low
GDI2	0.20008048	6.06328475	4.65607861	4.14E-06	3.47E-05	2.43279681	5.382553919	up-regulated in Low
ANKRD16	0.16433891	1.63693933	4.50804537	8.17E-06	6.44E-05	1.78127524	5.087581775	up-regulated in Low
IL15RA	0.13583929	2.67476589	2.03526926	0.04235536	0.11441669	-6.1243964	1.373091617	up-regulated in Low
RBM17	0.30760282	3.5906118	8.13550794	3.33E-15	1.19E-13	22.9103457	14.47790836	up-regulated in Low
PFKFB3	-0.2600822	4.49556832	-2.948063	0.00334861	0.0136724	-3.8762665	2.475135307	up-regulated in High
PRKCQ	-0.1446804	1.2086441	-2.3961924	0.01693615	0.05412135	-5.3315909	1.771185207	up-regulated in High
ITIH5	-0.6260951	0.99950839	-9.8124564	6.88E-21	5.02E-19	35.8574131	20.16249296	up-regulated in High
KIN	0.10405464	1.57607532	3.89170554	0.00011315	0.00069586	-0.7209484	3.946342821	up-regulated in Low
ATP5C1	0.37748037	5.88182047	8.18947388	2.24E-15	8.25E-14	23.2990873	14.64899209	up-regulated in Low
CELF2	-0.8249995	2.91956559	-11.482033	3.20E-27	4.24E-25	50.3276314	26.49441317	up-regulated in High
ECHDC3	-0.2748718	3.07819866	-2.9441881	0.00339008	0.01382144	-3.8874999	2.469789572	up-regulated in High
PROSER2	0.20997501	2.47714833	3.35257802	0.00086187	0.00417472	-2.6254766	3.064557425	up-regulated in Low
UPF2	0.1629507	3.02039121	3.53960502	0.00043849	0.00230567	-1.995276	3.358039066	up-regulated in Low
DHTKD1	0.25715942	3.33111216	4.60694276	5.20E-06	4.27E-05	2.21439791	5.283757424	up-regulated in Low
SEC61A2	0.22501433	1.51664912	5.02524699	7.03E-07	6.89E-06	4.14111964	6.152842445	up-regulated in Low
NUDT5	0.24977025	3.81601197	5.38955325	1.09E-07	1.25E-06	5.94187272	6.960855276	up-regulated in Low
CDC123	0.40376594	4.68484094	9.91204278	3.00E-21	2.30E-19	36.6793301	20.52263045	up-regulated in Low
CAMK1D	-0.7838972	2.7340613	-8.4977032	2.28E-16	9.55E-15	25.5565759	15.64191535	up-regulated in High
CCDC3	-0.3908747	2.43137076	-4.7153785	3.14E-06	2.69E-05	2.69919756	5.50295866	up-regulated in High
MCM10	1.08950892	1.29249026	20.1870448	1.40E-66	4.82E-63	140.653727	65.8525095	up-regulated in Low
SEPHS1	0.38913338	3.82617445	10.9425633	4.14E-25	4.69E-23	45.4981479	24.38288349	up-regulated in Low
BEND7	-0.1156335	1.01475181	-2.2169938	0.02707662	0.07972781	-5.7410447	1.567405542	up-regulated in High
PRPF18	0.11679242	2.74093626	3.71435881	0.00022688	0.00129328	-1.3770801	3.644196302	up-regulated in Low
RP11-295P9.3	-0.1009472	0.72340936	-3.3173574	0.00097551	0.00465828	-2.7405009	3.010770419	up-regulated in High
HSPA14	0.41285931	2.69476992	9.88111317	3.89E-21	2.95E-19	36.4234624	20.41052494	up-regulated in Low
SUV39H2	0.55910655	1.93826163	13.3377058	6.82E-35	1.50E-32	67.8984988	34.16638146	up-regulated in Low
DCLRE1C	0.10655309	1.52540284	3.50275115	0.00050216	0.00259624	-2.1220348	3.299162074	up-regulated in Low
OLAH	0.12670282	0.13332905	4.5123565	8.02E-06	6.33E-05	1.79997561	5.09605879	up-regulated in Low
ACBD7	0.34828735	0.62678538	7.07739614	5.05E-12	1.16E-10	15.6980031	11.29703721	up-regulated in Low
RPP38	0.22120673	3.00624909	6.20504921	1.16E-09	1.86E-08	10.3733639	8.93721012	up-regulated in Low
ITGA8	-0.8468532	1.50993957	-13.439859	2.48E-35	5.61E-33	68.9038764	34.60497793	up-regulated in High
FAM188A	-0.1363769	2.519825	-3.0975864	0.00206171	0.00897534	-3.431918	2.685771645	up-regulated in High
PTER	0.20134871	2.80901559	3.80492834	0.00015958	0.00094448	-1.0456049	3.797025104	up-regulated in Low
VIM	-0.4449557	7.03577219	-5.5342346	5.07E-08	6.17E-07	6.68813587	7.29474645	up-regulated in High
PTPLA	0.28765109	1.34195855	3.91935985	0.00010127	0.00063117	-0.616039	3.994520477	up-regulated in Low
STAM	0.24719826	3.3167309	6.23343007	9.77E-10	1.59E-08	10.5372889	9.010063707	up-regulated in Low
TMEM236	-0.1475618	0.36967639	-5.1566656	3.64E-07	3.77E-06	4.77768553	6.438884829	up-regulated in High
MRC1	-0.8879202	3.57986145	-7.0409174	6.41E-12	1.46E-10	15.4637454	11.19346354	up-regulated in High
CACNB2	0.14821341	0.46022496	3.52244198	0.00046714	0.00243916	-2.0544652	3.33055592	up-regulated in Low
NSUN6	0.10875822	1.80537635	2.94311508	0.00340165	0.01386279	-3.8906082	2.468310194	up-regulated in Low
ARL5B	0.37276616	2.74412955	7.27965438	1.32E-12	3.29E-11	17.0146946	11.87884501	up-regulated in Low
MALRD1	-0.1873615	0.27227325	-7.4646806	3.78E-13	1.01E-11	18.2453581	12.4221421	up-regulated in High
PLXDC2	-0.5899859	2.76823736	-8.0738324	5.21E-15	1.80E-13	22.4684752	14.28340427	up-regulated in High
CASC10	0.11184473	0.51572627	2.98082663	0.00301604	0.0124759	-3.7807139	2.520563514	up-regulated in Low
DNAJC1	0.19808025	3.9147545	4.13492433	4.17E-05	0.00028381	0.22558116	4.37985509	up-regulated in Low
SPAG6	-0.4642902	0.98976008	-4.585185	5.75E-06	4.68E-05	2.11836853	5.240291187	up-regulated in High
ARMC3	-0.1800898	0.64054529	-2.8532041	0.00450912	0.01767403	-4.1471604	2.345908648	up-regulated in High
OTUD1	-0.2912153	2.77604304	-6.2066093	1.14E-09	1.84E-08	10.3823584	8.941207951	up-regulated in High
KIAA1217	-0.2561491	3.39448728	-4.2983691	2.07E-05	0.00015046	0.89171213	4.68354271	up-regulated in High
ARHGAP21	0.19155806	2.82834468	3.8419512	0.00013792	0.00083093	-0.907936	3.860385099	up-regulated in Low
PRTFDC1	0.76962459	2.29128898	11.3373025	1.20E-26	1.50E-24	49.018465	25.92217139	up-regulated in Low
ENKUR	-0.2890885	0.69630326	-4.1508571	3.90E-05	0.0002669	0.28945798	4.409022869	up-regulated in High
THNSL1	0.13996224	1.9433258	3.01004712	0.00274522	0.01149244	-3.6946352	2.561423051	up-regulated in Low
GPR158	0.17094315	0.30629644	4.16908604	3.61E-05	0.00024878	0.36282164	4.442509947	up-regulated in Low
APBB1IP	-0.3024232	2.4144047	-4.1392769	4.09E-05	0.00027904	0.24300871	4.387813977	up-regulated in High
PDSS1	0.64759754	1.896024	17.0629431	1.08E-51	6.40E-49	106.446218	50.96458382	up-regulated in Low
ANKRD26	0.11949229	1.06245013	3.57540259	0.00038396	0.00204974	-1.8709432	3.415719472	up-regulated in Low
YME1L1	0.31043604	4.5451573	8.32707343	8.15E-16	3.16E-14	24.2991057	15.08895601	up-regulated in Low
MASTL	0.70865925	2.39504681	15.9029702	2.61E-46	1.11E-43	94.0893116	45.58305395	up-regulated in Low

ACBD5	0.21653417	3.44933788	4.52250315	7.66E-06	6.08E-05	1.84405363	5.116037128	up-regulated in Low
RAB18	0.12624822	3.94374817	3.11052378	0.00197517	0.00864823	-3.3924763	2.704395934	up-regulated in Low
MKX	0.18137887	0.5327701	3.02511592	0.00261444	0.01102481	-3.6499286	2.582620765	up-regulated in Low
ARMC4	-0.2018265	0.51571437	-3.3128833	0.0009909	0.00472405	-2.7550291	3.003971565	up-regulated in High
MPP7	-0.2337023	3.09249992	-3.2507131	0.00122947	0.0057122	-2.9549676	2.910281184	up-regulated in High
WAC	0.14812638	3.65606524	4.24239845	2.64E-05	0.00018725	0.66089523	4.57843202	up-regulated in Low
SVIL	-0.1592526	3.30868111	-2.4804181	0.01345382	0.04474925	-5.128404	1.871154337	up-regulated in High
KIAA1462	-0.2565862	2.35481443	-3.5808908	0.00037618	0.00201191	-1.8517762	3.424605403	up-regulated in High
MTPAP	0.32903692	2.00075684	9.93322816	2.51E-21	1.94E-19	36.8548964	20.59954897	up-regulated in Low
MAP3K8	-0.150904	2.5926511	-2.4708439	0.0138148	0.04571813	-5.1518461	1.859655265	up-regulated in High
ZNF438	-0.1431375	1.8605265	-4.7209607	3.06E-06	2.63E-05	2.72443405	5.514358886	up-regulated in High
ZEB1	-0.3616303	1.76917363	-7.0204495	7.32E-12	1.65E-10	15.3327385	11.13553218	up-regulated in High
KIF5B	0.2400416	5.13406977	5.26388406	2.10E-07	2.28E-06	5.30795362	6.67680951	up-regulated in Low
EPC1	-0.1209962	1.95579672	-4.2892109	2.16E-05	0.00015602	0.85375249	4.666264677	up-regulated in High
ITGB1	0.37598828	5.89183924	5.32974677	1.50E-07	1.67E-06	5.63852518	6.824981999	up-regulated in Low
NRP1	-0.3209059	3.91155127	-4.288555	2.16E-05	0.00015643	0.85103643	4.665028288	up-regulated in High
PARD3	0.3513398	3.37848004	6.45040112	2.66E-10	4.74E-09	11.8115082	9.575872093	up-regulated in Low
CUL2	0.35328409	3.10165533	9.64500105	2.74E-20	1.85E-18	34.4880254	19.56231203	up-regulated in Low
CREM	0.22511094	2.24261273	4.82769763	1.84E-06	1.66E-05	3.21221715	5.734516842	up-regulated in Low
FZD8	-0.2046448	2.076314	-2.7071893	0.00701986	0.02580386	-4.5473712	2.153671647	up-regulated in High
ZNF25	-0.3181382	1.82930354	-9.0458334	3.37E-18	1.77E-16	29.722757	17.47203501	up-regulated in High
ZNF33A	-0.1342529	2.72186775	-2.6988028	0.00719654	0.02631087	-4.5697385	2.142876125	up-regulated in High
ZNF33B	-0.2018001	2.23664141	-4.2782995	2.26E-05	0.00016287	0.80862401	4.645719437	up-regulated in High
BMS1	0.30110198	3.11804488	7.61809923	1.32E-13	3.76E-12	19.2844457	12.88052293	up-regulated in Low
RET	0.22781964	0.77028298	2.09976676	0.03625439	0.10093868	-5.9920214	1.440639416	up-regulated in Low
CSGALNACT	-0.1906788	3.2887447	-4.0653525	5.58E-05	0.00036927	-0.0506539	4.25359765	up-regulated in High
FXYD4	0.33176147	1.03038786	2.45689983	0.01435589	0.04720376	-5.1858291	1.842969975	up-regulated in Low
HNRNPF	0.30482878	6.29734849	8.05073638	6.15E-15	2.10E-13	22.3036695	14.21084881	up-regulated in Low
ZNF239	0.3013966	1.74486624	5.38029556	1.15E-07	1.31E-06	5.89471926	6.939740537	up-regulated in Low
ZNF485	0.1034274	1.24035107	2.95003738	0.00332766	0.01359541	-3.8705373	2.477861293	up-regulated in Low
ZNF32	-0.136801	4.12274306	-2.4375325	0.01513849	0.04934357	-5.2327177	1.819917314	up-regulated in High
CXCL12	-0.5228058	2.58896223	-5.7839668	1.29E-08	1.74E-07	8.01718112	7.888208008	up-regulated in High
RASSF4	-0.3578669	2.83946116	-5.047916	6.28E-07	6.22E-06	4.2498671	6.201742812	up-regulated in High
C10orf10	-0.5378055	5.02241881	-6.0586599	2.72E-09	4.12E-08	9.5380326	8.56571752	up-regulated in High
ALOX5	-0.6313409	3.59109567	-6.8303091	2.49E-11	5.19E-10	14.1307311	10.60370496	up-regulated in High
8-Mar	-0.1465955	2.59002317	-3.4911537	0.00052391	0.00269323	-2.161663	3.280740368	up-regulated in High
TIMM23	0.45191867	5.35187849	11.9093618	6.20E-29	9.31E-27	54.2483086	28.20756495	up-regulated in Low
NCOA4	-0.1389458	5.6361108	-2.8613636	0.00439653	0.01729669	-4.1241959	2.356889892	up-regulated in High
GPRIN2	-0.5100942	2.13926911	-5.1300069	4.17E-07	4.26E-06	4.64736057	6.380361809	up-regulated in High
SYT15	-0.1416726	0.1895549	-6.7244212	4.87E-11	9.70E-10	13.4731855	10.31253323	up-regulated in High
PTPN20A	0.12996737	0.3896444	2.83574322	0.00475889	0.01851396	-4.1960907	2.322494069	up-regulated in Low
GDF10	-0.5228958	0.92576246	-6.7206083	4.99E-11	9.92E-10	13.4496675	10.30211574	up-regulated in High
ZNF488	0.21134692	0.24021424	5.33315237	1.47E-07	1.64E-06	5.65571797	6.832685302	up-regulated in Low
ANXA8	0.21391092	0.3925093	3.30610822	0.00101464	0.00482422	-2.7769938	2.993690362	up-regulated in Low
SYT15	-0.1416726	0.1895549	-6.7244212	4.87E-11	9.70E-10	13.4731855	10.31253323	up-regulated in High
MAPK8	0.13213222	2.09946386	3.92434071	9.93E-05	0.00061997	-0.5970694	4.003228259	up-regulated in Low
WDFY4	-0.3317713	1.41852274	-4.6210028	4.88E-06	4.02E-05	2.27667503	5.311937748	up-regulated in High
LRRC18	-0.2510972	0.33759591	-5.460089	7.54E-08	8.88E-07	6.30350568	7.122719921	up-regulated in High
VSTM4	-0.4598367	1.52847278	-7.6036388	1.46E-13	4.12E-12	19.185792	12.83701579	up-regulated in High
C10orf128	-0.4523254	1.78728329	-7.5671132	1.87E-13	5.22E-12	18.9372621	12.72740032	up-regulated in High
DRGX	-0.2089951	0.52726055	-2.6249855	0.00893292	0.03163692	-4.7637042	2.049006706	up-regulated in High
OGDHL	0.32695408	0.48867374	5.02553845	7.02E-07	6.88E-06	4.14251505	6.15347001	up-regulated in Low
PARG	0.23296954	2.33974122	6.80735748	2.88E-11	5.96E-10	13.9874823	10.54028718	up-regulated in Low
TIMM23B	0.14310179	1.59971364	4.94611172	1.04E-06	9.86E-06	3.76496287	5.983581197	up-regulated in Low
SGMS1	-0.1722439	2.94499685	-2.6379432	0.0086033	0.0306312	-4.7300341	2.065334777	up-regulated in High
ASAH2B	0.17189044	0.98841865	6.41780877	3.24E-10	5.69E-09	11.6177413	9.489885595	up-regulated in Low
A1CF	0.10158422	0.0743661	3.8706451	0.00012307	0.00075	-0.8003746	3.909844691	up-regulated in Low
PRKG1	-0.3835765	1.19237897	-8.033986	6.95E-15	2.36E-13	22.1843708	14.15832406	up-regulated in High
CSTF2T	0.13321442	3.24779782	3.4336227	0.00064549	0.00323534	-2.3563942	3.190108092	up-regulated in Low
DKK1	0.95190923	1.67740232	5.82724001	1.02E-08	1.39E-07	8.25269374	7.993229192	up-regulated in Low
ZWINT	1.32660713	3.63593677	19.1239191	1.82E-61	2.59E-58	128.905317	60.74058075	up-regulated in Low
IPMK	0.21129851	1.94567302	5.14390496	3.88E-07	4.00E-06	4.71522768	6.410840269	up-regulated in Low
CISD1	0.32353178	3.14875187	7.71831433	6.55E-14	1.96E-12	19.9722056	13.18375968	up-regulated in Low
UBE2D1	0.21462147	3.22482492	4.7505876	2.66E-06	2.31E-05	2.85883011	5.575053673	up-regulated in Low
TFAM	0.35109204	3.19280371	8.06757808	5.45E-15	1.87E-13	22.4238109	14.26374146	up-regulated in Low
BICC1	-0.3861223	1.74371046	-4.9214213	1.17E-06	1.10E-05	3.64870763	5.931231918	up-regulated in High
FAM13C	-0.1804311	0.45107129	-6.6804713	6.42E-11	1.25E-09	13.202776	10.19273893	up-regulated in High
SLC16A9	-0.5262823	1.96686132	-3.8458532	0.0001358	0.0008191	-0.8933532	3.867092909	up-regulated in High
ANK3	-0.3282651	1.31123223	-5.7097003	1.95E-08	2.54E-07	7.61656662	7.709468548	up-regulated in High
CDK1	1.39785096	3.0103954	18.3378468	1.03E-57	9.88E-55	120.279164	56.98643787	up-regulated in Low
C10orf107	-0.6717727	0.92709607	-8.5460302	1.58E-16	6.76E-15	25.9161861	15.79999727	up-regulated in High

ARID5B	-0.3224491	3.03798268	-5.9764679	4.36E-09	6.38E-08	9.07656256	8.360305924	up-regulated in High
ADO	0.12055062	3.02579303	3.68163359	0.0002572	0.00144228	-1.494994	3.589733631	up-regulated in Low
EGR2	-0.4094876	2.10078706	-5.2299532	2.51E-07	2.68E-06	5.13908544	6.601073579	up-regulated in High
NRBF2	0.14679521	3.68113135	4.42126363	1.21E-05	9.20E-05	1.4083496	4.918393179	up-regulated in Low
JMJD1C	-0.1009838	2.73796249	-2.230855	0.02613748	0.07746645	-5.7104846	1.582736274	up-regulated in High
PBLD	-0.1690371	1.60424394	-2.2562948	0.02448719	0.07347793	-5.6539117	1.611061082	up-regulated in High
HNRNPH3	0.16812983	4.45704908	4.10941998	4.64E-05	0.00031318	0.12380771	4.333361234	up-regulated in Low
DNA2	0.70427936	1.42242208	14.5191999	4.71E-40	1.39E-37	79.7326862	39.32707759	up-regulated in Low
SLC25A16	-0.2950044	2.33251705	-5.6671982	2.47E-08	3.15E-07	7.3893401	7.608033348	up-regulated in High
TET1	0.17886087	0.40050684	5.94638353	5.18E-09	7.46E-08	8.90901566	8.285692474	up-regulated in Low
CCAR1	0.26830734	3.7081508	7.35752345	7.83E-13	2.00E-11	17.5296038	12.10621611	up-regulated in Low
DDX50	0.16247078	3.93581614	4.41183268	1.26E-05	9.55E-05	1.36822538	4.900173484	up-regulated in Low
DDX21	0.5461911	4.51515987	9.3472968	3.07E-19	1.82E-17	32.0936038	18.51236071	up-regulated in Low
KIAA1279	0.13178588	3.3600498	3.34440301	0.00088709	0.00428197	-2.6522782	3.052031136	up-regulated in Low
VPS26A	0.20228113	4.41664451	5.06057584	5.90E-07	5.87E-06	4.31079049	6.229131828	up-regulated in Low
SUPV3L1	0.32026358	2.76048223	9.64427158	2.76E-20	1.86E-18	34.4820952	19.55971245	up-regulated in Low
C10orf35	0.11766599	3.31442874	1.96529078	0.04993868	0.13043031	-6.2634258	1.301562949	up-regulated in Low
COL13A1	-0.3431949	0.98430244	-6.6227145	9.20E-11	1.76E-09	12.8496688	10.0362607	up-regulated in High
H2AFY2	0.25572371	2.79117442	2.4597907	0.01424219	0.0468933	-5.1787992	1.846423112	up-regulated in Low
AIFM2	0.38520133	2.7950453	6.38668856	3.91E-10	6.77E-09	11.433502	9.408109561	up-regulated in Low
TYSND1	0.10809102	2.75915377	2.41725587	0.015998	0.05166075	-5.2814198	1.795934279	up-regulated in Low
NPFFR1	-0.1595318	0.28587116	-4.9321787	1.11E-06	1.05E-05	3.69929396	5.954012959	up-regulated in High
EIF4EBP2	-0.1663322	4.47535307	-3.9369639	9.43E-05	0.00059185	-0.5488926	4.02533828	up-regulated in High
PALD1	-0.3717487	2.12046554	-6.0138572	3.52E-09	5.24E-08	9.28581093	8.453464394	up-regulated in High
SGPL1	0.11553125	3.9932675	2.30405463	0.02163237	0.06627048	-5.5460066	1.664895926	up-regulated in Low
UNC5B	-0.2501879	2.77571643	-3.423233	0.00067008	0.00334507	-2.3912324	3.173873998	up-regulated in High
SLC29A3	-0.1907265	2.75351031	-3.6585381	0.00028084	0.00155869	-1.5776159	3.551539796	up-regulated in High
CDH23	-0.1602703	0.37290873	-6.1930619	1.24E-09	1.99E-08	10.3043185	8.906519519	up-regulated in High
C10orf54	-0.4204363	3.33421745	-6.7612428	3.86E-11	7.82E-10	13.7008739	10.4133775	up-regulated in High
PSAP	-0.1628934	9.19251605	-3.0536928	0.002382	0.01017545	-3.5645551	2.623057516	up-regulated in High
SPOCK2	-0.5144296	3.28415604	-5.2334456	2.46E-07	2.64E-06	5.15642158	6.608850088	up-regulated in High
ASCC1	0.14449115	2.45490207	4.13097011	4.24E-05	0.0002881	0.2097636	4.372630793	up-regulated in Low
ANAPC16	-0.1382662	4.70792416	-3.1994885	0.00146492	0.00667229	-3.1169772	2.834187086	up-regulated in High
DDIT4	0.27218074	5.57342067	2.4178279	0.01597318	0.05160898	-5.2800513	1.796608738	up-regulated in Low
OIT3	-0.1913303	0.50131687	-3.9014043	0.00010884	0.00067212	-0.6842347	3.963206832	up-regulated in High
PLA2G12B	-0.5534927	1.26849545	-4.6036211	5.28E-06	4.33E-05	2.1997104	5.27711038	up-regulated in High
P4HA1	0.39040209	5.11245692	5.65038765	2.70E-08	3.43E-07	7.29987815	7.568085774	up-regulated in Low
NUDT13	-0.1090389	1.20078871	-2.7371747	0.00641965	0.02394623	-4.4668475	2.1924886	up-regulated in High
ECD	0.1292694	3.28671679	4.18981689	3.31E-05	0.00022974	0.44661843	4.480743012	up-regulated in Low
DNAJC9	0.58230874	2.4009007	16.0057128	8.81E-47	3.96E-44	95.1731971	46.05519492	up-regulated in Low
MRPS16	0.39831519	4.82969178	10.69934	3.54E-24	3.64E-22	43.3669981	23.45060112	up-regulated in Low
CFAP70	-0.2529115	0.60364189	-4.9549771	9.94E-07	9.48E-06	3.80683444	6.002431413	up-regulated in High
ANXA7	0.14062141	5.72053007	3.88600764	0.00011576	0.00071017	-0.7424772	3.936451867	up-regulated in Low
USP54	-0.4891641	2.71115115	-8.5047899	2.16E-16	9.07E-15	25.6092147	15.6650564	up-regulated in High
MYOZ1	-0.5145551	1.00418078	-7.7728668	4.47E-14	1.36E-12	20.3495569	13.35008412	up-regulated in High
CHCHD1	0.26424213	4.46778159	5.85966908	8.47E-09	1.17E-07	8.43019036	8.072353047	up-regulated in Low
ZSWIM8	-0.1371737	3.03202029	-2.8472632	0.00459273	0.01795726	-4.1638412	2.337929014	up-regulated in High
CAMK2G	-0.1490588	3.14174239	-3.2891393	0.00107643	0.00507913	-2.8318177	2.968016386	up-regulated in High
AP3M1	0.11765517	3.41602345	3.07424011	0.00222677	0.00960155	-3.5026923	2.652323992	up-regulated in Low
DUSP13	0.51428408	0.65740685	5.94277408	5.29E-09	7.59E-08	8.888963	8.276761195	up-regulated in Low
SAMD8	0.12363574	2.36151667	3.24680088	0.00124614	0.00578088	-2.9674279	2.904434507	up-regulated in Low
VDAC2	0.52939225	4.40779986	12.3213189	1.29E-30	2.19E-28	58.1026285	29.89094017	up-regulated in Low
COMTD1	0.46021115	3.0248816	5.78033494	1.32E-08	1.77E-07	7.99748453	7.879422941	up-regulated in Low
ZNF503	-0.1827377	3.05320214	-2.4495401	0.01464897	0.04796836	-5.2036899	1.83419301	up-regulated in High
C10orf11	-0.2514999	1.19997813	-6.4107944	3.38E-10	5.91E-09	11.5761484	9.471425764	up-regulated in High
DLG5	0.24466778	2.44094939	3.88133844	0.00011794	0.00072194	-0.760097	3.928355682	up-regulated in Low
POLR3A	0.30055714	2.16019195	7.8007604	3.67E-14	1.13E-12	20.5433065	13.43546902	up-regulated in Low
RPS24	0.12364492	7.36981104	2.02271214	0.04363936	0.11723434	-6.1496965	1.360121658	up-regulated in Low
ZMIZ1	-0.1822724	3.27561032	-3.2118913	0.00140436	0.0064301	-3.0779768	2.852520169	up-regulated in High
PPIF	0.62046815	4.56695175	13.0009786	1.85E-33	3.72E-31	64.6104525	32.73172097	up-regulated in Low
ZCCHC24	-0.5936904	2.6902444	-11.238774	2.92E-26	3.57E-24	48.132814	25.53498973	up-regulated in High
SFTPA2	-2.5563488	8.1660208	-9.0306294	3.80E-18	1.98E-16	29.6046521	17.42019059	up-regulated in High
SFTPA1	-2.7175479	7.89009172	-9.5662236	5.22E-20	3.43E-18	33.8493824	19.28233375	up-regulated in High
SFTPD	-2.1113795	5.8332784	-10.326553	8.98E-23	8.07E-21	40.1592175	22.04667312	up-regulated in High
TMEM254	0.12961099	3.44128803	2.52959518	0.0117283	0.03984823	-5.0066034	1.930765069	up-regulated in Low
PLAC9	-0.7527446	1.91944921	-12.295433	1.64E-30	2.77E-28	57.8583634	29.7842785	up-regulated in High
ANXA11	-0.2503738	5.15208729	-5.7186726	1.86E-08	2.43E-07	7.66472497	7.730961513	up-regulated in High
MAT1A	0.24580167	0.6413886	3.52092967	0.00046974	0.00245146	-2.0596675	3.328139602	up-regulated in Low
DYDC2	-0.1299926	0.60694412	-2.0698684	0.03898222	0.10701782	-6.0538895	1.409133431	up-regulated in High
FAM213A	0.14979611	3.58696078	3.27032801	0.00114899	0.00537852	-2.892278	2.939682609	up-regulated in Low
GHITM	0.31033235	6.31825205	6.70937242	5.35E-11	1.06E-09	13.380429	10.27144463	up-regulated in Low

GRID1	-0.1694444	0.36395966	-7.4184636	5.18E-13	1.36E-11	17.9356368	12.28545315	up-regulated in High
WAPAL	0.14014346	3.22453196	3.70595919	0.00023433	0.00133012	-1.4074394	3.630178861	up-regulated in Low
MMRN2	-0.3551296	2.28881325	-5.6981126	2.08E-08	2.69E-07	7.55446863	7.681751691	up-regulated in High
SNCG	0.68315059	3.39146683	4.20333301	3.12E-05	0.00021805	0.50146088	4.505756148	up-regulated in Low
MINPP1	0.22863276	3.22423367	5.25834452	2.17E-07	2.34E-06	5.28031755	6.664417034	up-regulated in Low
PAPSS2	-0.4775899	4.18016163	-5.3586272	1.29E-07	1.45E-06	5.78463493	6.890437724	up-regulated in High
ATAD1	0.10158906	3.54578483	2.63475211	0.00868345	0.03088077	-4.738341	2.061307727	up-regulated in Low
PTEN	-0.1780176	2.99864508	-4.593709	5.53E-06	4.51E-05	2.15593975	5.257299127	up-regulated in High
LIPK	0.12492566	0.22193439	2.97141216	0.00310836	0.01280572	-3.8082749	2.507468182	up-regulated in Low
LIPM	-0.1664381	0.83033715	-2.9075794	0.00380569	0.0152885	-3.9929252	2.419566171	up-regulated in High
ACTA2	-0.4070593	5.68522349	-4.1695411	3.60E-05	0.00024839	0.36465676	4.443347424	up-regulated in High
FAS	-0.4646616	2.77293011	-5.7835691	1.30E-08	1.74E-07	8.01502358	7.88724572	up-regulated in High
CH25H	-0.9864448	2.46547573	-8.5291706	1.80E-16	7.61E-15	25.7905587	15.74477503	up-regulated in High
LIPA	-0.5255626	4.82052374	-6.7005903	5.66E-11	1.11E-09	13.326378	10.24749983	up-regulated in High
IFIT2	-0.1707726	2.7402243	-2.0574131	0.04016917	0.10973458	-6.0794058	1.396107158	up-regulated in High
IFIT1	-0.3204833	2.79258263	-2.7500521	0.00617642	0.0231707	-4.4320022	2.209263432	up-regulated in High
IFIT5	-0.2414733	3.09928291	-4.1938153	3.25E-05	0.0002263	0.46282497	4.488135423	up-regulated in High
SLC16A12	-0.3135368	0.58801985	-4.5998428	5.38E-06	4.40E-05	2.18301584	5.269554563	up-regulated in High
PANK1	0.1284328	1.0209943	3.08419566	0.00215496	0.00932355	-3.472575	2.666561777	up-regulated in Low
KIF20B	0.69060355	1.59121043	14.9327913	6.68E-42	2.24E-39	83.9730885	41.17532444	up-regulated in Low
RPP30	0.28975012	2.45745772	10.1239872	5.05E-22	4.17E-20	38.4469378	21.29690987	up-regulated in Low
PPP1R3C	-0.3668686	2.07200294	-4.176012	3.51E-05	0.00024233	0.39077409	4.455265428	up-regulated in High
CPEB3	-0.1519857	0.91432675	-5.718775	1.86E-08	2.43E-07	7.66527501	7.731206987	up-regulated in High
5-Mar	0.22217315	3.5325735	6.13751919	1.72E-09	2.69E-08	9.98589426	8.764944484	up-regulated in Low
KIF11	1.23647808	2.50188711	19.2540903	4.32E-62	6.82E-59	130.339364	61.36461826	up-regulated in Low
HHEX	-0.1985492	2.08045729	-3.3408395	0.0008983	0.00432783	-2.6639414	3.046578856	up-regulated in High
CYP26A1	-0.1109549	0.27079874	-2.3669368	0.0183194	0.05776207	-5.4005627	1.737088876	up-regulated in High
MYOF	-0.3651642	4.80036231	-4.2140549	2.98E-05	0.00020907	0.54508272	4.525646509	up-regulated in High
CEP55	1.3824698	2.74662962	17.7967751	3.81E-55	2.96E-52	114.381344	54.41923758	up-regulated in Low
FFAR4	-0.231149	0.65831257	-4.6436096	4.39E-06	3.66E-05	2.3771736	5.357399392	up-regulated in High
PLCE1	-0.2957651	1.14893484	-6.0422072	2.99E-09	4.50E-08	9.44522287	8.524416536	up-regulated in High
NOC3L	0.20820077	2.46316085	5.27037522	2.04E-07	2.21E-06	5.34037031	6.69134467	up-regulated in Low
HELLS	0.65511739	1.26771941	13.925126	1.96E-37	4.96E-35	73.7274232	36.70879679	up-regulated in Low
PDLIM1	-0.2583893	5.97491256	-4.6399475	4.47E-06	3.72E-05	2.36086289	5.350022212	up-regulated in High
SORBS1	-0.5382977	1.59578274	-9.7154707	1.53E-20	1.07E-18	35.0623491	19.81405388	up-regulated in High
ALDH18A1	0.33470436	4.9205853	6.76211838	3.84E-11	7.79E-10	13.7063006	10.41578078	up-regulated in Low
ENTPD1	-0.2204301	2.22293442	-4.9242237	1.16E-06	1.09E-05	3.66187609	5.937162528	up-regulated in High
CCNJ	0.26675873	1.98067544	5.97769286	4.33E-09	6.33E-08	9.0833999	8.363350394	up-regulated in Low
ZNF518A	0.10519632	1.8899557	2.49179277	0.01303584	0.04360991	-5.1004388	1.884860897	up-regulated in Low
BLNK	-0.4049406	1.96271746	-6.9462065	1.18E-11	2.58E-10	14.8601659	10.92650812	up-regulated in High
PIK3AP1	-0.2501078	2.57431396	-3.0577104	0.00235089	0.01005358	-3.5524906	2.628767344	up-regulated in High
C10orf12	0.17169429	1.06321395	4.73069654	2.92E-06	2.52E-05	2.76851389	5.53426893	up-regulated in Low
ARHGAP19	0.10759972	1.67023282	3.08431169	0.00215413	0.00932137	-3.4722234	2.666727941	up-regulated in Low
FRAT1	-0.2513439	1.82206782	-4.569371	6.18E-06	5.00E-05	2.04883426	5.208807531	up-regulated in High
FRAT2	0.25315364	3.00588808	4.4031393	1.31E-05	9.89E-05	1.33130937	4.883407723	up-regulated in Low
RRP12	0.37578461	2.80011766	7.89711839	1.85E-14	5.92E-13	21.216772	13.73219253	up-regulated in Low
PGAM1	0.46526876	4.01875366	9.47567166	1.09E-19	6.89E-18	33.1197433	18.9624054	up-regulated in Low
EXOSC1	0.18908248	3.08733194	5.75461873	1.52E-08	2.02E-07	7.85832813	7.817348198	up-regulated in Low
ZDHHC16	-0.1800254	4.30902813	-3.6272935	0.00031609	0.00172805	-1.6886062	3.500189256	up-regulated in High
UBTD1	-0.6493234	4.15725591	-10.091029	6.67E-22	5.45E-20	38.1704438	21.17581495	up-regulated in High
MORN4	-0.1002016	1.98313799	-2.2047865	0.02792775	0.08169836	-5.7678035	1.553964071	up-regulated in High
SFRP5	-0.2079387	0.53269633	-2.9822296	0.00300249	0.01242604	-3.7765995	2.522517923	up-regulated in High
GOLGA7B	-0.3372243	2.01411962	-3.3021647	0.00102869	0.00488113	-2.7897588	2.98771405	up-regulated in High
CRTAC1	-0.9387931	2.36747982	-6.8631092	2.02E-11	4.26E-10	14.3361406	10.6946276	up-regulated in High
LOXL4	-0.6611047	2.10594531	-6.1660175	1.45E-09	2.30E-08	10.1489668	8.837455775	up-regulated in High
PYROXD2	-0.5102122	2.04656465	-7.3082268	1.09E-12	2.74E-11	17.2031172	11.96205733	up-regulated in High
HPS1	-0.2445686	3.73379358	-6.0926182	2.23E-09	3.44E-08	9.73027963	8.651251756	up-regulated in High
HPSE2	-0.2285245	0.25822633	-7.5395681	2.26E-13	6.24E-12	18.7504664	12.64500192	up-regulated in High
GOT1	0.37323035	4.51533772	5.67033982	2.42E-08	3.10E-07	7.40608495	7.61550974	up-regulated in Low
NKX2-3	0.21927925	0.1573414	4.41248074	1.25E-05	9.53E-05	1.37098004	4.901424429	up-regulated in Low
ENTPD7	0.36034263	2.33582692	6.48652521	2.13E-10	3.85E-09	12.0272405	9.67158416	up-regulated in Low
CUTC	0.12588176	3.12773985	3.00159864	0.00282114	0.01177227	-3.7196062	2.549576059	up-regulated in Low
COX15	0.22069382	3.54371689	5.85879509	8.51E-09	1.18E-07	8.42539543	8.070215877	up-regulated in Low
ABCC2	1.1712589	0.88589472	8.9045951	1.02E-17	4.99E-16	28.6311267	16.99276579	up-regulated in Low
CPN1	0.14173655	0.11381547	2.96216072	0.0032016	0.01314294	-3.8352766	2.494632412	up-regulated in Low
ERLIN1	0.43385827	3.33035482	9.48705002	9.94E-20	6.32E-18	33.211163	19.00249408	up-regulated in Low
CHUK	0.21125597	2.50186927	5.62013653	3.19E-08	4.01E-07	7.1394772	7.496445458	up-regulated in Low
CWF19L1	0.28276765	2.92215932	7.04513563	6.23E-12	1.42E-10	15.490783	11.20541882	up-regulated in Low
BLOC1S2	0.11611622	3.7961829	2.83206117	0.00481314	0.01869021	-4.2063717	2.317571332	up-regulated in Low
SCD	0.55659792	6.0378426	5.69932547	2.07E-08	2.68E-07	7.56096296	7.684650512	up-regulated in Low
SEC31B	-0.1963007	0.84626943	-4.0651471	5.58E-05	0.00036951	-0.0514631	4.253227496	up-regulated in High

NDUFB8	0.14814524	3.92230436	3.48444079	0.00053691	0.002753	-2.1845437	3.2701006	up-regulated in Low
MRPL43	0.12658223	3.97405011	2.99210207	0.00290876	0.01209041	-3.7475943	2.536291735	up-regulated in Low
C10orf2	0.49438357	2.34419164	10.9603835	3.53E-25	4.01E-23	45.6554469	24.45168167	up-regulated in Low
SFXN3	-0.5653204	3.80585332	-8.8617009	1.42E-17	6.82E-16	28.3020564	16.84825614	up-regulated in High
KAZALD1	0.13688805	1.79175765	1.98581873	0.04760404	0.12560248	-6.2231381	1.322356149	up-regulated in Low
DPCD	0.1087246	3.34884088	2.23870342	0.02561836	0.07621027	-5.6930984	1.591448726	up-regulated in Low
FBXW4	-0.3885787	3.57408569	-7.5608883	1.96E-13	5.43E-12	18.8950014	12.70875932	up-regulated in High
NPM3	0.61322514	4.58422382	9.99599228	1.49E-21	1.18E-19	37.376498	20.82805303	up-regulated in Low
MGEA5	-0.1007356	4.28898471	-2.1668847	0.0307194	0.08837277	-5.8499624	1.512587228	up-regulated in High
C10orf76	-0.1978036	3.01926786	-6.5375337	1.56E-10	2.88E-09	12.3335911	9.807461436	up-regulated in High
LDB1	-0.268197	4.49318582	-5.8461936	9.13E-09	1.26E-07	8.35632957	8.039430361	up-regulated in High
PPRC1	0.16941503	3.41359704	3.74017207	0.00020538	0.0011833	-1.2833749	3.687440007	up-regulated in Low
NOLC1	0.49996963	4.90356421	10.9099849	5.53E-25	6.16E-23	45.2109834	24.25728127	up-regulated in Low
ELOVL3	0.27002164	0.6120896	4.26789053	2.37E-05	0.00016956	0.76567325	4.626161375	up-regulated in Low
NFKB2	0.15205228	3.96467986	2.5414783	0.01134209	0.03875013	-4.9768219	1.945306788	up-regulated in Low
PSD	-0.1040015	0.86028114	-2.7327957	0.00650431	0.02419979	-4.4786607	2.186798545	up-regulated in High
FBXL15	-0.1136438	2.55826931	-2.1194203	0.03455157	0.09706325	-5.950878	1.461532187	up-regulated in High
CUEDC2	0.12938556	4.93595681	3.50874819	0.00049124	0.00254966	-2.1014939	3.308707876	up-regulated in Low
SUFU	-0.1470078	2.16997857	-4.0851544	5.14E-05	0.00034289	0.02752353	4.289350263	up-regulated in High
TRIM8	-0.3248378	5.75993594	-5.18457	3.16E-07	3.32E-06	4.91475011	6.500413556	up-regulated in High
WBP1L	-0.239511	4.23250048	-6.5000068	1.96E-10	3.57E-09	12.1080127	9.707413868	up-regulated in High
C10orf32	-0.3296069	3.19841717	-7.0520987	5.95E-12	1.36E-10	15.5354437	11.22516598	up-regulated in High
INA	0.52339666	0.54944735	5.68489959	2.24E-08	2.87E-07	7.48379501	7.650203499	up-regulated in Low
PCGF6	0.33071537	1.97756855	9.34441696	3.15E-19	1.86E-17	32.0706964	18.50231252	up-regulated in Low
TAF5	0.29860161	1.43693948	9.82093086	6.41E-21	4.71E-19	35.9271385	20.19304709	up-regulated in Low
USMG5	0.46281009	5.78219821	9.07318444	2.72E-18	1.44E-16	29.9355777	17.56545176	up-regulated in Low
PDCD11	0.3658706	3.08758683	7.63817294	1.15E-13	3.30E-12	19.4216408	12.94102282	up-regulated in Low
CALHM2	-0.1562792	2.53819023	-2.7326815	0.00650653	0.02420651	-4.4789685	2.186650267	up-regulated in High
NEURL1	0.35827778	0.4916166	4.89047602	1.36E-06	1.26E-05	3.50374622	5.865930851	up-regulated in Low
SH3PXD2A	-0.2808636	3.4961722	-4.6364391	4.54E-06	3.77E-05	2.3452482	5.342959422	up-regulated in High
OBFC1	-0.1369511	4.19368815	-2.3992436	0.01679732	0.05376304	-5.3243498	1.774759896	up-regulated in High
COL17A1	-0.412101	2.1920296	-2.4674145	0.01394618	0.04607193	-5.1602213	1.855544876	up-regulated in High
SFR1	0.30106576	2.46753766	6.51594298	1.78E-10	3.26E-09	12.2036732	9.749843738	up-regulated in Low
CFAP43	-0.3190147	0.69672862	-4.4350476	1.13E-05	8.69E-05	1.46713613	4.945081308	up-regulated in High
GSTO1	0.22096173	5.60938711	3.49564163	0.00051539	0.00265597	-2.1463427	3.287863083	up-regulated in Low
GSTO2	0.15715098	1.19847136	2.53368036	0.01159423	0.03946553	-4.9963804	1.935758188	up-regulated in Low
ITPRIP	-0.1504872	2.44504666	-2.4204892	0.01585812	0.05128549	-5.2736803	1.799748221	up-regulated in High
CFAP58	-0.1923887	0.34633201	-4.6096135	5.14E-06	4.22E-05	2.22621402	5.289104712	up-regulated in High
XPNPEP1	0.20873777	3.12953639	5.57082317	4.17E-08	5.14E-07	6.87962801	7.380343111	up-regulated in Low
ADD3	-0.1879987	3.48000907	-2.1467462	0.03229817	0.09195404	-5.8930474	1.490822031	up-regulated in High
SMNDC1	0.27357162	2.89997804	9.33185956	3.48E-19	2.06E-17	31.9708677	18.45852235	up-regulated in Low
DUSP5	0.19634544	3.94483899	1.98172194	0.04806248	0.1265604	-6.2312113	1.318193867	up-regulated in Low
SMC3	0.39422516	3.92069947	7.6324911	1.19E-13	3.42E-12	19.3827789	12.92388613	up-regulated in Low
PDCD4	-0.2468612	3.81116901	-4.486087	9.03E-06	7.04E-05	1.68628223	5.044510669	up-regulated in High
ADRA2A	-0.70725	1.73047838	-6.0432928	2.97E-09	4.48E-08	9.45133996	8.527138849	up-regulated in High
GPAM	-0.1391804	1.6410182	-2.3825471	0.01756945	0.05579394	-5.3638636	1.755241855	up-regulated in High
ACSL5	-1.0155034	5.11342032	-9.3218744	3.77E-19	2.22E-17	31.8915552	18.42373077	up-regulated in High
VTI1A	0.16014884	1.76672069	6.15453461	1.56E-09	2.46E-08	10.0831816	8.808205864	up-regulated in Low
TCF7L2	-0.1334642	2.9926202	-2.749401	0.00618851	0.02321119	-4.4337678	2.208413768	up-regulated in High
HABP2	-1.0815762	2.12363058	-7.2453842	1.66E-12	4.08E-11	16.789483	11.77937101	up-regulated in High
PLEKHS1	-0.546258	1.90876729	-4.0569233	5.78E-05	0.00038124	-0.0838246	4.238422846	up-regulated in High
DCLRE1A	0.36320742	2.20942594	7.92883416	1.48E-14	4.81E-13	21.4398448	13.83045294	up-regulated in Low
ADRB1	-0.4735445	0.95411875	-6.1714856	1.41E-09	2.24E-08	10.1803306	8.851400146	up-regulated in High
CCDC186	-0.2438291	2.54813883	-5.2387335	2.40E-07	2.57E-06	5.18268973	6.620632633	up-regulated in High
VWA2	-0.7618866	2.43340677	-8.3630179	6.24E-16	2.46E-14	24.5624087	15.2047652	up-regulated in High
TRUB1	0.30722432	2.97771841	6.40800921	3.43E-10	6.01E-09	11.5596437	9.464100381	up-regulated in Low
GFRA1	-0.4141735	0.51307919	-8.3910155	5.07E-16	2.03E-14	24.7680899	15.29522128	up-regulated in High
C10orf82	0.10906914	0.11808836	3.60104729	0.00034887	0.00188379	-1.7811432	3.457337868	up-regulated in Low
KIAA1598	0.30666039	2.99106106	5.28951005	1.84E-07	2.02E-06	5.43613684	6.73427856	up-regulated in Low
VAX1	0.10994309	0.0441822	7.18680786	2.45E-12	5.90E-11	16.4065313	11.61018695	up-regulated in Low
SLC18A2	-0.2677742	0.53586699	-5.9582616	4.84E-09	7.02E-08	8.9750796	8.315114866	up-regulated in High
PDZD8	0.14037535	2.44764719	2.47734869	0.01356863	0.04505155	-5.135929	1.867464069	up-regulated in Low
RAB11FIP2	-0.2404589	2.02566681	-5.4304179	8.82E-08	1.03E-06	6.150875	7.054418348	up-regulated in High
CACUL1	0.15590806	2.64443123	4.51888792	7.78E-06	6.16E-05	1.82833827	5.10891456	up-regulated in Low
EIF3A	0.26347367	5.07230274	4.79915905	2.11E-06	1.88E-05	3.08082349	5.6752481	up-regulated in Low
FAM45A	0.11990571	1.89368149	3.83933969	0.00013935	0.00083834	-0.9176881	3.855898974	up-regulated in Low
SFXN4	0.48549849	3.87121473	9.87227329	4.18E-21	3.16E-19	36.3504323	20.37852641	up-regulated in Low
PRDX3	0.33594108	5.42596149	6.78981965	3.22E-11	6.60E-10	13.8782921	10.49194192	up-regulated in Low
GRK5	-0.2155222	1.88963441	-3.295608	0.00105247	0.00497938	-2.8109502	2.977790635	up-regulated in High
RGS10	-0.2076488	4.48525025	-2.5350604	0.01154925	0.03934682	-4.9929233	1.937446411	up-regulated in High
TIAL1	0.18722508	3.50324338	5.75937714	1.48E-08	1.97E-07	7.8840361	7.828817076	up-regulated in Low

INPP5F	-0.1057374	2.17559321	-2.0194012	0.04398336	0.11794682	-6.1563418	1.356711651	up-regulated in High
MCMBP	0.29131255	3.67614961	8.0687295	5.40E-15	1.86E-13	22.4320316	14.26736057	up-regulated in Low
SEC23IP	0.1084717	3.19835793	3.06571258	0.00229003	0.00983582	-3.528415	2.640158384	up-regulated in Low
FGFR2	-0.7193216	1.85229209	-8.6368074	7.97E-17	3.51E-15	26.5957633	16.09867289	up-regulated in High
ATE1	0.15439466	2.60809795	3.54796915	0.00042514	0.00224334	-1.966332	3.371472821	up-regulated in Low
NSMCE4A	0.1001879	3.03078282	2.19969709	0.02828938	0.08257833	-5.7789169	1.548376644	up-regulated in Low
BTBD16	0.20827798	0.41565857	4.32106823	1.88E-05	0.00013752	0.986121	4.726500898	up-regulated in Low
HTRA1	-0.4769246	5.2090723	-5.5114178	5.73E-08	6.91E-07	6.56928466	7.24160412	up-regulated in High
DMBT1	-1.5022207	2.99465893	-7.7162599	6.64E-14	1.98E-12	19.9580355	13.17751324	up-regulated in High
CUZD1	0.13801984	0.55348598	2.60594086	0.00943791	0.03317188	-4.8128979	2.025124191	up-regulated in Low
FAM24B	0.41683643	1.26243879	8.55252579	1.51E-16	6.46E-15	25.9646368	15.82129412	up-regulated in Low
C10orf88	0.20018738	2.0909099	6.16714755	1.44E-09	2.29E-08	10.1554465	8.840336731	up-regulated in Low
IKZF5	-0.1589981	2.45464002	-4.2225284	2.87E-05	0.00020228	0.57962996	4.541395895	up-regulated in High
ACADSB	-0.4707542	2.72290296	-6.4516695	2.63E-10	4.70E-09	11.8190656	9.579225419	up-regulated in High
HMX2	0.11690051	0.07255838	4.37456589	1.48E-05	0.00011115	1.21044823	4.828498073	up-regulated in Low
BUB3	0.496759	3.48670193	14.4497379	9.58E-40	2.80E-37	79.0251587	39.01864743	up-regulated in Low
CPXM2	-0.4472255	2.13424853	-4.1182014	4.47E-05	0.00030277	0.15878302	4.349342253	up-regulated in High
CHST15	-0.1802582	3.60198558	-2.365766	0.01837676	0.05790415	-5.4033058	1.735731032	up-regulated in High
NKX1-2	0.21224072	0.42941078	3.07083459	0.00225184	0.00969532	-3.5129731	2.647462266	up-regulated in Low
LHPP	-0.1477052	2.82743218	-2.4105838	0.01629009	0.05241248	-5.2973584	1.788076607	up-regulated in High
METTL10	0.21288687	1.74803905	6.55748631	1.38E-10	2.57E-09	12.453973	9.860842858	up-regulated in Low
FAM175B	0.16274983	3.10305577	5.3159143	1.61E-07	1.78E-06	5.56879437	6.79373576	up-regulated in Low
CTBP2	0.2611577	3.11186481	7.00388542	8.15E-12	1.82E-10	15.2269476	11.08874685	up-regulated in Low
BCCIP	0.50348106	3.60026123	13.8420461	4.51E-37	1.12E-34	72.8961845	36.34629847	up-regulated in Low
FANK1	-0.2965029	1.45794927	-4.7571331	2.58E-06	2.25E-05	2.88862609	5.588506103	up-regulated in High
ADAM12	0.22929296	1.64491632	2.40158494	0.01669147	0.05346821	-5.3187872	1.777505367	up-regulated in Low
DOCK1	-0.2387534	3.37861449	-3.8971781	0.0001107	0.00068237	-0.7002433	3.95585398	up-regulated in High
FAM196A	0.2389651	0.32251988	5.87277118	7.86E-09	1.09E-07	8.5021463	8.104422966	up-regulated in Low
PTPRE	-0.6300877	2.64271714	-9.4458359	1.39E-19	8.65E-18	32.8803886	18.85744066	up-regulated in High
MKI67	1.32553324	2.53530543	16.5927912	1.70E-49	8.76E-47	101.407547	48.7704923	up-regulated in Low
GLRX3	0.40032869	3.67490968	8.37717847	5.62E-16	2.24E-14	24.6663732	15.2504885	up-regulated in Low
BNIP3	0.37051678	4.01892592	4.81190893	1.99E-06	1.78E-05	3.13943717	5.701690538	up-regulated in Low
JAKMIP3	0.11807982	0.33673444	2.83310714	0.00479768	0.01864123	-4.2034525	2.318969218	up-regulated in Low
STK32C	0.12147966	2.30723198	2.1130499	0.03509585	0.09828958	-5.9642552	1.454744275	up-regulated in Low
LRRC27	-0.1615619	1.06368495	-5.1564879	3.64E-07	3.77E-06	4.77681485	6.438493907	up-regulated in High
INPP5A	-0.1816937	2.83008603	-4.3834396	1.43E-05	0.00010718	1.24790485	4.845518576	up-regulated in High
CFAP46	-0.1824803	0.24980758	-4.8244988	1.87E-06	1.68E-05	3.19745427	5.72785889	up-regulated in High
KNDC1	-0.5217537	1.11987775	-6.2534729	8.68E-10	1.42E-08	10.6534391	9.061675489	up-regulated in High
VENTX	-0.2126641	0.60559352	-5.6835292	2.25E-08	2.89E-07	7.47647314	7.646934844	up-regulated in High
ADAM8	-0.3326272	3.62740452	-3.2976826	0.00104489	0.00494794	-2.8042494	2.980928759	up-regulated in High
ZNF511	0.2243223	2.69753325	4.70192064	3.35E-06	2.85E-05	2.63846854	5.475521078	up-regulated in Low
PRAP1	0.32658412	0.59445896	3.09007869	0.00211352	0.00916904	-3.4547337	2.67499301	up-regulated in Low
CALY	0.15377006	0.26514567	2.38787537	0.01731972	0.05512983	-5.351283	1.761459139	up-regulated in Low
ECHS1	0.21932915	6.21060045	4.91810143	1.19E-06	1.12E-05	3.63311606	5.924209719	up-regulated in Low
PAOX	-0.1655422	1.74542489	-4.2175857	2.94E-05	0.00020626	0.55947025	4.532205838	up-regulated in High
BET1L	-0.1823235	4.47920737	-4.801894	2.08E-06	1.86E-05	3.09338444	5.680915157	up-regulated in High
SIRT3	-0.3252612	2.44708695	-8.8931409	1.11E-17	5.43E-16	28.5431411	16.95412895	up-regulated in High
PSMD13	0.15118986	4.80427986	3.7517954	0.00019634	0.00113727	-1.2409805	3.706993811	up-regulated in Low
ATHL1	-0.4023063	4.12049416	-2.6476784	0.00836288	0.0298937	-4.7046318	2.077643941	up-regulated in High
IFITM2	-0.4898909	6.20684775	-6.3718955	4.27E-10	7.36E-09	11.3461899	9.369349311	up-regulated in High
IFITM1	-0.3484188	6.58825082	-3.0405395	0.00248653	0.01056245	-3.6039466	2.604406909	up-regulated in High
IFITM3	-0.2193499	8.3003711	-2.981179	0.00301263	0.01246445	-3.7796806	2.521054366	up-regulated in High
B4GALNT4	0.37326097	1.55388558	3.12741298	0.00186722	0.00823237	-3.3407492	2.728804683	up-regulated in Low
PKP3	0.280764	4.43095256	3.91342287	0.00010372	0.00064463	-0.6386204	3.984153316	up-regulated in Low
SIGIRR	-0.2005924	3.73598457	-3.3296387	0.00093439	0.00448026	-2.7005242	3.02947242	up-regulated in High
RNH1	-0.2683837	4.58511177	-6.0679884	2.58E-09	3.92E-08	9.59075142	8.589175366	up-regulated in High
HRAS	0.22732102	3.8360715	4.28685032	2.18E-05	0.00015757	0.8439802	4.661816121	up-regulated in Low
LRRC56	-0.3446168	1.83470939	-4.7720316	2.40E-06	2.11E-05	2.95658506	5.619183499	up-regulated in High
LMNTD2	-0.2901018	1.32725849	-4.3086605	1.98E-05	0.0001444	0.9344581	4.702995519	up-regulated in High
RASSF7	-0.46903	4.64347224	-5.7721776	1.38E-08	1.85E-07	7.95328448	7.859707801	up-regulated in High
SCT	-0.227214	1.03304498	-3.6140084	0.0003323	0.00180552	-1.7355256	3.478466564	up-regulated in High
DEAF1	-0.2584112	2.98562135	-5.200034	2.92E-07	3.08E-06	4.99099411	6.534630676	up-regulated in High
EPS8L2	-0.2953653	4.42050903	-4.1021021	4.79E-05	0.00032172	0.09471464	4.32006552	up-regulated in High
TMEM80	-0.3382645	2.47610224	-5.0705977	5.61E-07	5.61E-06	4.35911683	6.250854442	up-regulated in High
TALDO1	0.28646073	6.24705488	3.93704697	9.43E-05	0.00059172	-0.5485751	4.02548397	up-regulated in Low
PDDC1	-0.1128878	3.3078757	-2.1722829	0.03030767	0.08741659	-5.8383461	1.518447451	up-regulated in High
SLC25A22	0.19305041	3.0224572	3.57755422	0.00038089	0.00203542	-1.8634323	3.419201778	up-regulated in Low
PNPLA2	-0.3199556	4.3675185	-5.0030549	7.85E-07	7.63E-06	4.0350886	6.105149388	up-regulated in High
CRACR2B	-0.1970955	3.09662764	-1.9917628	0.04694546	0.1241847	-6.2113955	1.328406379	up-regulated in High
CD151	-0.317094	6.48265474	-5.0977468	4.90E-07	4.96E-06	4.49046479	6.309880992	up-regulated in High
POLR2L	-0.1314219	6.20378597	-2.2537103	0.02465061	0.07387338	-5.6596878	1.60817237	up-regulated in High

TSPAN4	-0.6131572	3.41286943	-9.1087119	2.06E-18	1.11E-16	30.2127055	17.68708632	up-regulated in High
AP2A2	-0.2486313	3.49215997	-5.6108604	3.35E-08	4.20E-07	7.090444	7.474541413	up-regulated in High
TOLLIP	-0.2473479	3.48639404	-6.3955583	3.70E-10	6.44E-09	11.4859356	9.4313844	up-regulated in High
MOB2	-0.2334719	2.79694829	-6.1194404	1.91E-09	2.97E-08	9.88278059	8.719086144	up-regulated in High
DUSP8	-0.2934127	2.0016223	-4.798915	2.11E-06	1.88E-05	3.07970273	5.674742441	up-regulated in High
IFITM10	-0.2667594	2.04813963	-3.1124314	0.00196269	0.00859942	-3.3866474	2.707147397	up-regulated in High
CTSD	-0.6526592	8.81842869	-9.1137252	1.98E-18	1.07E-16	30.2518732	17.7042766	up-regulated in High
SYT8	-0.450049	1.21043592	-3.9500429	8.95E-05	0.00056391	-0.4988228	4.048309578	up-regulated in High
TNNI2	-0.3354484	1.26781793	-3.8910057	0.00011347	0.00069749	-0.7235941	3.945127372	up-regulated in High
LSP1	-0.560267	3.75094964	-6.4923137	2.06E-10	3.73E-09	12.0619039	9.686960872	up-regulated in High
PRR33	-0.1923023	0.55716787	-4.4651131	9.92E-06	7.69E-05	1.5959471	5.003535692	up-regulated in High
TNNT3	-0.1123652	0.24433308	-2.708916	0.00698397	0.02569135	-4.5427577	2.155897607	up-regulated in High
MRPL23	0.10587591	3.238808	2.04172558	0.04170776	0.11304139	-6.1113283	1.379783146	up-regulated in Low
IGF2	-0.4094608	2.15178512	-2.5116724	0.01233293	0.04158244	-5.0512642	1.908933588	up-regulated in High
ASCL2	0.20727959	1.15514009	2.47563287	0.01363318	0.04522988	-5.1401315	1.865402739	up-regulated in Low
C11orf21	-0.174018	0.55389947	-4.4017198	1.32E-05	9.95E-05	1.32528773	4.880672677	up-regulated in High
TSPAN32	-0.1978991	0.57884008	-5.0694551	5.64E-07	5.64E-06	4.35360251	6.248375906	up-regulated in High
CD81	-0.5522612	6.10722597	-10.626205	6.72E-24	6.74E-22	42.7320119	23.17275698	up-regulated in High
KCNQ1	-0.8728692	3.13324237	-8.0193134	7.72E-15	2.61E-13	22.0800279	14.11238147	up-regulated in High
CDKN1C	-0.2975728	2.670511	-3.8077684	0.00015781	0.00093576	-1.0350887	3.80186728	up-regulated in High
SLC22A18	-0.2706208	2.85000124	-3.6535412	0.00028622	0.00158477	-1.5954272	3.543302603	up-regulated in High
CARS	0.20717856	2.70598887	5.90144478	6.69E-09	9.43E-08	8.66010741	8.174811692	up-regulated in Low
OSBPL5	-0.312418	2.33619779	-4.4084518	1.28E-05	9.69E-05	1.35386073	4.893649967	up-regulated in High
MRGPRE	-0.1085979	0.17081264	-2.7789531	0.00566055	0.02150697	-4.3532202	2.247141093	up-regulated in High
NUP98	0.13420953	3.6154879	3.6098378	0.00033755	0.00183063	-1.7502212	3.471660941	up-regulated in Low
RHOG	-0.1198936	5.01647987	-2.7417004	0.0063332	0.02367934	-4.4546194	2.198376842	up-regulated in High
STIM1	-0.1929478	3.5579928	-4.3254995	1.84E-05	0.00013522	1.00460506	4.734909292	up-regulated in High
RRM1	0.53176755	3.9965016	10.8979013	6.16E-25	6.85E-23	45.1046053	24.21075127	up-regulated in Low
TRIM21	-0.1301752	3.91122204	-2.4375953	0.0151359	0.04933785	-5.2325663	1.819991843	up-regulated in High
TRIM68	-0.309165	2.53493939	-4.5506517	6.74E-06	5.40E-05	1.96681081	5.171658045	up-regulated in High
HBB	-0.8510718	3.95741932	-4.9530719	1.00E-06	9.57E-06	3.79783009	5.998377925	up-regulated in High
HBE1	0.21453257	0.19110155	4.05250105	5.88E-05	0.00038706	-0.1012011	4.230472342	up-regulated in Low
UBQLNL	-0.1354489	0.31866397	-6.0167225	3.46E-09	5.16E-08	9.30189313	8.460623101	up-regulated in High
TRIM34	-0.1063413	0.43084452	-4.8124442	1.98E-06	1.77E-05	3.14190121	5.702802031	up-regulated in High
TRIM5	-0.2925565	2.89071821	-6.5463216	1.48E-10	2.74E-09	12.3865738	9.830956584	up-regulated in High
TRIM22	-0.8123963	3.66401235	-10.36612	6.39E-23	5.86E-21	40.4962378	22.19421487	up-regulated in High
OR52N4	-0.1076124	0.16805475	-5.9543716	4.95E-09	7.17E-08	8.95343176	8.305474052	up-regulated in High
FAM160A2	-0.209525	3.15312821	-4.6260072	4.76E-06	3.94E-05	2.29888303	5.321985245	up-regulated in High
CNGA4	-0.2715882	0.38483728	-5.2651256	2.09E-07	2.27E-06	5.31415084	6.679588336	up-regulated in High
SMPD1	-0.4669609	3.98264339	-9.0864378	2.45E-18	1.31E-16	30.0388683	17.61078844	up-regulated in High
APBB1	-0.4908075	2.24469994	-7.5081694	2.81E-13	7.63E-12	18.5381968	12.55135481	up-regulated in High
TRIM3	-0.2101406	1.48450545	-5.207042	2.82E-07	2.98E-06	5.02561343	6.550165175	up-regulated in High
TIMM10B	-0.269469	3.5391325	-6.8898662	1.70E-11	3.63E-10	14.5043067	10.76905243	up-regulated in High
TIMM10B	-0.269469	3.5391325	-6.8898662	1.70E-11	3.63E-10	14.5043067	10.76905243	up-regulated in High
ILK	-0.1553353	2.09194387	-3.6396449	0.00030169	0.00165956	-1.6448383	3.520444772	up-regulated in High
TPP1	-0.3150833	5.62218769	-6.357642	4.66E-10	7.96E-09	11.2622244	9.332071013	up-regulated in High
DCHS1	-0.1851009	1.68337363	-3.0002787	0.00283317	0.01181623	-3.7235015	2.547727558	up-regulated in High
MRPL17	0.2891079	4.62557283	6.53703318	1.56E-10	2.89E-09	12.3305751	9.806123924	up-regulated in Low
ZNF214	-0.2250016	0.73821445	-5.9825528	4.21E-09	6.17E-08	9.11053924	8.375434457	up-regulated in High
OLFML1	-0.5110878	1.80428595	-8.3653586	6.13E-16	2.42E-14	24.5795844	15.21231914	up-regulated in High
PPFIBP2	-0.4921648	2.01078297	-9.1883379	1.09E-18	6.12E-17	30.8366165	17.96088933	up-regulated in High
OVCH2	-0.1103368	0.15612859	-4.4814485	9.22E-06	7.18E-05	1.66627062	5.035434971	up-regulated in High
TUB	-0.2996588	0.88698078	-5.7613435	1.47E-08	1.95E-07	7.89466485	7.833558643	up-regulated in High
RIC3	-0.3075491	0.44791389	-7.5861957	1.64E-13	4.61E-12	19.066986	12.78461786	up-regulated in High
LMO1	0.11252125	0.13978736	5.33556112	1.45E-07	1.62E-06	5.66788412	6.838136219	up-regulated in Low
STK33	-0.2600637	1.04104569	-4.7977601	2.13E-06	1.89E-05	3.07440071	5.672350279	up-regulated in High
TRIM66	-0.2251463	1.12659184	-5.0612596	5.88E-07	5.85E-06	4.31408499	6.230612787	up-regulated in High
ST5	-0.5156424	2.57610428	-10.459823	2.85E-23	2.69E-21	41.2976738	22.54503077	up-regulated in High
C11orf16	-0.3173209	0.71060798	-4.1751019	3.52E-05	0.00024321	0.38709879	4.453588397	up-regulated in High
TMEM9B	-0.3464527	4.50264417	-8.6202971	9.03E-17	3.96E-15	26.471768	16.04418262	up-regulated in High
SCUBE2	-0.8017869	1.47619404	-8.1062311	4.12E-15	1.44E-13	22.7002725	14.38544279	up-regulated in High
DENND5A	-0.1046886	3.11732665	-2.3822648	0.01758277	0.05581499	-5.3645294	1.754912742	up-regulated in High
TMEM41B	-0.152121	3.89122059	-3.0159424	0.00269336	0.01130289	-3.6771706	2.569705808	up-regulated in High
IPO7	0.16433879	4.75779027	3.30418791	0.00102146	0.00485272	-2.7832115	2.990779468	up-regulated in Low
ZNF143	0.12414642	2.32661832	4.39916531	1.33E-05	0.0001005	1.31445642	4.87575288	up-regulated in Low
SWAP70	-0.3571452	3.5927513	-7.1095863	4.09E-12	9.54E-11	15.905541	11.38878099	up-regulated in High
ADM	0.6475954	2.78223203	5.78166683	1.31E-08	1.76E-07	8.00470651	7.88264412	up-regulated in Low
AMPD3	-0.199409	2.11838295	-3.9974466	7.38E-05	0.0004749	-0.3160438	4.132102641	up-regulated in High
RNF141	-0.3442552	3.11789205	-6.889976	1.70E-11	3.63E-10	14.5049981	10.76935842	up-regulated in High
LYVE1	-0.1515991	1.38219113	-2.0615387	0.03977265	0.10886531	-6.0709708	1.400415445	up-regulated in High
MRVI1	-0.4579019	1.61498231	-7.9686879	1.11E-14	3.68E-13	21.7211373	13.95434203	up-regulated in High

ZBED5	-0.2445142	2.44677266	-6.1791142	1.35E-09	2.14E-08	10.224126	8.870870587	up-regulated in High
GALNT18	-0.6624374	3.01524726	-9.5376474	6.59E-20	4.27E-18	33.6186071	19.18115139	up-regulated in High
USP47	-0.1338791	2.91481595	-3.2368418	0.00128951	0.00595746	-2.9990825	2.889577073	up-regulated in High
DKK3	-0.6736585	3.78295252	-7.615788	1.34E-13	3.81E-12	19.268668	12.87356499	up-regulated in High
MICAL2	-0.5913178	3.13678156	-7.7565837	5.01E-14	1.51E-12	20.2367049	13.30034631	up-regulated in High
MICALCL	-0.528493	1.24986162	-8.1811105	2.39E-15	8.73E-14	23.2387138	14.62242399	up-regulated in High
PARVA	-0.3659812	3.23490617	-8.0766032	5.10E-15	1.77E-13	22.4882717	14.29211927	up-regulated in High
ARNTL	-0.2302685	1.69385883	-5.4235237	9.15E-08	1.06E-06	6.11551624	7.038592362	up-regulated in High
SPON1	-0.8192714	3.06992976	-8.4096295	4.41E-16	1.78E-14	24.9051209	15.35548144	up-regulated in High
RRAS2	-0.1694579	3.10742102	-2.9128409	0.00374323	0.01508012	-3.9778516	2.426753116	up-regulated in High
PSMA1	0.20193731	4.47401249	5.30896594	1.67E-07	1.84E-06	5.53382799	6.778065544	up-regulated in Low
PSMA1	0.20193731	4.47401249	5.30896594	1.67E-07	1.84E-06	5.53382799	6.778065544	up-regulated in Low
CYP2R1	-0.1605182	1.9719611	-4.010303	7.00E-05	0.00045319	-0.2661201	4.154972754	up-regulated in High
CALCB	0.20777296	0.21585891	3.78138832	0.00017498	0.00102577	-1.1324841	3.757006819	up-regulated in Low
CALCA	0.76963085	1.33614405	2.94033418	0.0034318	0.01397102	-3.8986585	2.464478354	up-regulated in Low
SOX6	-0.1023814	0.30085899	-3.5874866	0.00036703	0.00196928	-1.8287045	3.435299543	up-regulated in High
PLEKHA7	-0.282874	2.28924701	-4.8323404	1.80E-06	1.63E-05	3.23365963	5.744186689	up-regulated in High
NUCB2	-0.116808	3.37828825	-1.9988145	0.04617415	0.12259425	-6.19742	1.335601095	up-regulated in High
NCR3LG1	0.1280508	0.3783494	3.69409036	0.00024524	0.00138424	-1.4502268	3.610417193	up-regulated in Low
KCNJ11	-0.2071775	1.34253315	-2.6099119	0.00933055	0.03284002	-4.8026692	2.030092608	up-regulated in High
USH1C	0.20959819	0.5514562	2.15702227	0.03148407	0.09013731	-5.8711117	1.501909148	up-regulated in Low
SERGEF	-0.1873072	2.21389304	-4.6313483	4.65E-06	3.85E-05	2.32261011	5.332719096	up-regulated in High
SAAL1	0.34111379	2.11657024	8.69913679	4.95E-17	2.24E-15	27.0654412	16.30505175	up-regulated in Low
LDHA	0.53051438	6.98353163	8.30879318	9.33E-16	3.59E-14	24.165526	15.03019822	up-regulated in Low
TMEM86A	-0.2426225	1.79563655	-4.4851356	9.06E-06	7.07E-05	1.68217612	5.04264852	up-regulated in High
E2F8	0.77246045	1.44624932	13.7664074	9.61E-37	2.37E-34	72.1413104	36.01708429	up-regulated in Low
HTATIP2	0.33971093	4.56305214	5.43680738	8.53E-08	9.96E-07	6.18368063	7.069100552	up-regulated in Low
PRMT3	0.2456369	2.03733155	6.57016148	1.27E-10	2.39E-09	12.5306075	9.894821657	up-regulated in Low
NELL1	-0.3945027	0.70973766	-3.4204407	0.00067683	0.00337446	-2.4005784	3.16951785	up-regulated in High
ANO5	-0.1899398	0.65661851	-3.7194307	0.0002225	0.00127089	-1.3587168	3.652673282	up-regulated in High
FANCF	0.17678102	2.5570129	4.64566689	4.35E-06	3.63E-05	2.38634146	5.361545734	up-regulated in Low
GAS2	0.30867199	0.62526749	4.77290444	2.39E-06	2.10E-05	2.96057238	5.620983201	up-regulated in Low
LUZP2	-0.1091066	0.16602359	-4.4367498	1.13E-05	8.63E-05	1.4744076	4.948381965	up-regulated in High
ANO3	0.19580014	0.31033913	4.17090974	3.58E-05	0.00024707	0.37017772	4.44586692	up-regulated in Low
MUC15	-0.6306479	2.14350907	-4.7094078	3.23E-06	2.76E-05	2.67223499	5.490777599	up-regulated in High
FIBIN	-0.445955	2.1011096	-4.831275	1.81E-06	1.64E-05	3.22873769	5.741967114	up-regulated in High
CCDC34	0.69808399	2.04894485	12.0721215	1.35E-29	2.13E-27	55.7625922	28.86901571	up-regulated in Low
LIN7C	0.10410367	3.16371536	2.70791921	0.00700467	0.02575126	-4.5454213	2.15461247	up-regulated in Low
KIF18A	0.67798464	1.28973005	15.0709514	1.60E-42	5.51E-40	85.3997354	41.79705681	up-regulated in Low
ARL14EP	-0.146704	2.35390505	-4.4016558	1.32E-05	9.95E-05	1.32501665	4.880549551	up-regulated in High
RCN1	0.12790774	4.1850076	2.00829127	0.04515446	0.12043457	-6.1785616	1.345299388	up-regulated in Low
EIF3M	0.21621157	4.01965374	5.99621194	3.90E-09	5.74E-08	9.18691899	8.409440726	up-regulated in Low
PRRG4	-0.2143177	3.01920462	-3.3302315	0.00093245	0.00447168	-2.6985909	3.030376623	up-regulated in High
QSER1	0.20066243	2.19936315	4.74712458	2.70E-06	2.35E-05	2.84308126	5.567942754	up-regulated in Low
CSTF3	0.31088601	2.94031939	9.3885848	2.20E-19	1.34E-17	32.4225671	18.65665169	up-regulated in Low
HIPK3	-0.3392502	3.75573676	-7.3816729	6.65E-13	1.71E-11	17.6901862	12.17710867	up-regulated in High
CD59	-0.6582149	6.20286214	-9.4086521	1.88E-19	1.15E-17	32.5828192	18.72693713	up-regulated in High
FBXO3	-0.1613808	2.02708082	-5.0894015	5.11E-07	5.15E-06	4.45002286	6.291708957	up-regulated in High
LMO2	-0.3784693	2.14421639	-6.945174	1.19E-11	2.60E-10	14.853623	10.92361359	up-regulated in High
CAPRIN1	0.12005817	5.08990108	2.94245081	0.00340883	0.01388914	-3.8925319	2.467394619	up-regulated in Low
NAT10	0.24449171	3.70227944	6.07443827	2.48E-09	3.79E-08	9.6272431	8.60541175	up-regulated in Low
CAT	-0.5989206	4.74063389	-9.2163587	8.76E-19	4.96E-17	31.0570902	18.05763154	up-regulated in High
ELF5	-0.429127	1.38138411	-4.0694368	5.48E-05	0.0003636	-0.0345583	4.260959956	up-regulated in High
EHF	-0.4343681	3.82575461	-4.2233996	2.86E-05	0.00020155	0.58318563	4.543016693	up-regulated in High
PDHX	0.3230159	3.14853249	8.06872089	5.40E-15	1.86E-13	22.4319701	14.2673335	up-regulated in Low
CD44	-0.432946	4.89405853	-5.0040231	7.81E-07	7.60E-06	4.03970552	6.107226387	up-regulated in High
PAMR1	-0.365191	1.17966452	-6.2362626	9.61E-10	1.56E-08	10.5536846	9.017349687	up-regulated in High
FJX1	0.36443755	1.40516416	5.77614409	1.35E-08	1.81E-07	7.97476988	7.869291404	up-regulated in Low
LDLRAD3	0.28226608	1.44132617	4.46937372	9.73E-06	7.56E-05	1.61426622	5.011846295	up-regulated in Low
PRR5L	-0.1661189	1.59740302	-2.5228212	0.01195365	0.04047658	-5.0235194	1.922499499	up-regulated in High
TRAF6	-0.1513528	1.74221033	-5.1109036	4.59E-07	4.67E-06	4.55434512	6.338580826	up-regulated in High
TTC17	-0.1060932	3.05986673	-2.7146105	0.00686678	0.02534008	-4.5275224	2.163246743	up-regulated in High
ALKBH3	-0.1729327	2.85955431	-3.588115	0.00036617	0.00196539	-1.8265042	3.436319305	up-regulated in High
C11orf96	-0.7610638	3.39832818	-7.9753806	1.06E-14	3.51E-13	21.7684821	13.97519222	up-regulated in High
ACCS	-0.3906921	1.89000649	-5.820405	1.06E-08	1.44E-07	8.2153925	7.976598299	up-regulated in High
CD82	-0.6250902	4.1303538	-7.9234381	1.54E-14	4.98E-13	21.4018426	13.81371433	up-regulated in High
TSPAN18	-0.2383042	1.80591412	-3.6355771	0.00030636	0.00168115	-1.6592684	3.513767456	up-regulated in High
TP53I11	-0.2455454	4.22995095	-3.4685175	0.00056894	0.0028954	-2.2386499	3.244930808	up-regulated in High
PRDM11	-0.1201833	0.78145787	-4.2491345	2.56E-05	0.00018241	0.68852517	4.591020688	up-regulated in High
SYT13	0.42368492	1.0265432	3.13541247	0.00181801	0.00804458	-3.3161549	2.740403554	up-regulated in Low
CRY2	-0.7838012	3.02245848	-15.181437	5.06E-43	1.81E-40	86.5441108	42.29574274	up-regulated in High

MAPK8IP1	-0.2988495	2.07070247	-4.0545969	5.83E-05	0.00038432	-0.0929679	4.234239492	up-regulated in High
PHF21A	-0.1171418	2.15691592	-3.3087575	0.00100529	0.00478564	-2.76841	2.997708583	up-regulated in High
CREB3L1	-0.6995574	4.12462936	-5.1202052	4.38E-07	4.46E-06	4.59959626	6.358908136	up-regulated in High
DGKZ	0.13720404	2.71873444	3.26917912	0.00115357	0.00539777	-2.8959598	2.937956477	up-regulated in Low
AMBRA1	-0.1464221	2.69547303	-4.0300813	6.45E-05	0.00042028	-0.1890234	4.190276895	up-regulated in High
ARHGAP1	-0.2682708	4.66323315	-5.9785056	4.31E-09	6.31E-08	9.08793707	8.365370647	up-regulated in High
F2	0.38110978	0.23513861	5.72433742	1.80E-08	2.36E-07	7.69516486	7.744545845	up-regulated in Low
CKAP5	0.46656307	3.70951891	8.84351039	1.63E-17	7.81E-16	28.1628529	16.78712064	up-regulated in Low
LRP4	0.35066425	1.19084731	4.1933829	3.26E-05	0.00022662	0.46107174	4.487335736	up-regulated in Low
C11orf49	-0.1306323	2.70918557	-2.7488646	0.00619849	0.02324564	-4.4352219	2.207713986	up-regulated in High
ARFGAP2	-0.1591429	4.08243431	-4.9426608	1.06E-06	1.00E-05	3.7486825	5.976251291	up-regulated in High
DDB2	-0.1008991	2.5395724	-2.1231183	0.03423894	0.09636119	-5.9430943	1.465479636	up-regulated in High
MADD	-0.1963147	2.93659278	-4.3084504	1.98E-05	0.00014452	0.93358483	4.702598152	up-regulated in High
SPI1	-0.529881	4.13818084	-5.7514411	1.55E-08	2.05E-07	7.84117078	7.809693657	up-regulated in High
SLC39A13	-0.3186141	3.67365778	-6.3981257	3.65E-10	6.35E-09	11.5011245	9.438126355	up-regulated in High
PSMC3	0.23398545	5.66016322	6.2209019	1.05E-09	1.70E-08	10.4648486	8.977870772	up-regulated in Low
NDUFS3	0.13053228	4.07597367	3.32271321	0.00095737	0.00457956	-2.7230846	3.01891926	up-regulated in Low
KBTBD4	-0.1016117	1.86240335	-2.9457054	0.00337379	0.01376558	-3.883103	2.471882079	up-regulated in High
MTCH2	0.32779577	4.83824809	8.21630481	1.84E-15	6.84E-14	23.4930914	14.73436091	up-regulated in Low
AGBL2	-0.1778906	0.49368472	-4.279511	2.25E-05	0.00016214	0.81362928	4.647998364	up-regulated in High
FNBP4	-0.1268751	3.16899531	-2.3012346	0.02179248	0.06668052	-5.5524394	1.661693323	up-regulated in High
NUP160	0.13544247	3.0510237	3.00223552	0.00281535	0.01175316	-3.7177262	2.550468182	up-regulated in Low
PTPRJ	-0.3208943	2.71925428	-5.4046802	1.01E-07	1.17E-06	6.01907619	6.995421425	up-regulated in High
FOLH1	0.1506436	0.81656327	2.92296002	0.00362573	0.01465527	-3.9487876	2.440604766	up-regulated in Low
LRRC55	-0.1023763	0.26105082	-2.126589	0.03394775	0.09573414	-5.9357771	1.469189	up-regulated in High
APLNR	-0.2798102	2.03386241	-3.6155051	0.00033044	0.00179723	-1.7302476	3.480910634	up-regulated in High
TNKS1BP1	-0.3379107	4.86648389	-5.3938396	1.07E-07	1.23E-06	5.96372944	6.97064172	up-regulated in High
SSRP1	0.44507612	4.85481817	10.7510401	2.25E-24	2.35E-22	43.817518	23.6477115	up-regulated in Low
SLC43A3	-0.3758579	3.91432868	-4.2812483	2.23E-05	0.00016096	0.82080945	4.651267443	up-regulated in High
RTN4RL2	-0.3942309	2.34594616	-3.3250061	0.00094971	0.00454586	-2.7156203	3.022411138	up-regulated in High
SLC43A1	-0.1413953	2.19543214	-1.9757835	0.04873359	0.12786627	-6.2428845	1.312171585	up-regulated in High
TIMM10	0.39919708	4.83252591	7.65494427	1.02E-13	2.98E-12	19.5364839	12.99166216	up-regulated in Low
SMTNL1	0.17671969	0.56729485	3.28779708	0.00108146	0.00509959	-2.8361425	2.965990338	up-regulated in Low
SERPING1	-0.4425978	6.25858179	-5.3082958	1.67E-07	1.85E-06	5.53045792	6.77655518	up-regulated in High
MED19	0.14743883	3.06898049	3.91011102	0.00010511	0.00065209	-0.6512031	3.978375869	up-regulated in Low
TMX2	0.26404876	5.20588965	6.91921825	1.41E-11	3.04E-10	14.6894038	10.85095771	up-regulated in Low
C11orf31	0.1143266	4.85290592	2.2491933	0.0249385	0.0745729	-5.6697672	1.60312972	up-regulated in Low
RP11-691N7.(	-0.1936076	0.60844723	-4.8970667	1.32E-06	1.23E-05	3.53455039	5.879809668	up-regulated in High
LPXN	-0.3953364	3.32549128	-5.8658002	8.18E-09	1.14E-07	8.46384476	8.087352873	up-regulated in High
ZFP91	0.14506847	3.92591551	3.63781301	0.00030378	0.00166952	-1.6513385	3.517437004	up-regulated in Low
FAM111B	0.76174347	1.865335	10.4865717	2.26E-23	2.16E-21	41.5272973	22.6455349	up-regulated in Low
DTX4	-0.7720657	3.57938573	-9.1603562	1.37E-18	7.55E-17	30.6169254	17.86448403	up-regulated in High
MPEG1	-0.5445377	3.29817335	-6.1924734	1.24E-09	1.99E-08	10.3009322	8.905014226	up-regulated in High
PATL1	0.40285602	3.85042375	8.33214125	7.85E-16	3.05E-14	24.3361771	15.10526202	up-regulated in Low
MRPL16	0.23562885	3.9648373	6.71622289	5.13E-11	1.02E-09	13.4226321	10.29013989	up-regulated in Low
MS4A2	-0.6174653	0.87082957	-11.093797	1.07E-25	1.27E-23	46.8380157	24.9688492	up-regulated in High
MS4A6A	-0.5267604	3.12786065	-5.9092539	6.40E-09	9.07E-08	8.70324296	8.194030241	up-regulated in High
MS4A4E	-0.216452	0.37888305	-7.7236318	6.31E-14	1.89E-12	20.0088963	13.19993331	up-regulated in High
MS4A4A	-0.4304328	3.0694893	-4.7078537	3.25E-06	2.78E-05	2.66522235	5.487609263	up-regulated in High
MS4A7	-0.6409392	2.85074496	-7.6123017	1.37E-13	3.90E-12	19.2448753	12.86307237	up-regulated in High
MS4A14	-0.2249011	0.62936342	-5.5757399	4.06E-08	5.01E-07	6.90544544	7.391881053	up-regulated in High
MS4A1	-0.6756376	1.45765586	-6.1196573	1.91E-09	2.97E-08	9.88401601	8.719635616	up-regulated in High
MS4A8	-0.7494316	1.21677593	-5.7666412	1.42E-08	1.90E-07	7.923317	7.846340171	up-regulated in High
MS4A15	-1.3649649	1.78452951	-8.3760026	5.67E-16	2.26E-14	24.6577348	15.24668944	up-regulated in High
CCDC86	0.56786893	3.64005538	12.0267365	2.07E-29	3.21E-27	55.3392051	28.68408866	up-regulated in Low
PTGDR2	-0.1003771	0.22243749	-4.9436905	1.05E-06	9.97E-06	3.75353934	5.978438022	up-regulated in High
PRPF19	0.40293142	5.48180067	10.8714327	7.78E-25	8.52E-23	44.8718408	24.10893681	up-regulated in Low
TMEM109	-0.2104809	5.6012979	-4.7088255	3.24E-06	2.77E-05	2.66960729	5.489590404	up-regulated in High
TMEM132A	0.49693362	2.9587981	5.53021175	5.18E-08	6.30E-07	6.6671496	7.285363691	up-regulated in Low
SLC15A3	-0.397325	2.96312133	-5.1596667	3.59E-07	3.72E-06	4.79239511	6.445489018	up-regulated in High
CD6	-0.2458607	1.73335125	-3.4679688	0.00057008	0.00290042	-2.2405101	3.244065192	up-regulated in High
CD5	-0.4602956	1.97309029	-5.7896465	1.25E-08	1.69E-07	8.0480051	7.901955535	up-regulated in High
VPS37C	0.11688373	3.53597843	2.64431816	0.00844518	0.03014911	-4.7134101	2.073391181	up-regulated in Low
DDB1	0.1712964	4.33144716	4.84035296	1.73E-06	1.57E-05	3.27071012	5.760893665	up-regulated in Low
DAK	0.10715406	2.69904054	2.34869861	0.01923107	0.06014801	-5.4431413	1.715996519	up-regulated in Low
CYB561A3	-0.109712	2.22314573	-3.1036288	0.00202087	0.00881988	-3.4135164	2.694462236	up-regulated in High
CPSF7	0.13889426	4.01136772	3.70479733	0.00023537	0.00133516	-1.4116337	3.628242009	up-regulated in Low
SDHAF2	0.13228847	2.92613018	3.78381095	0.00017333	0.00101693	-1.1235663	3.761115721	up-regulated in Low
PPP1R32	-0.3454143	1.00118777	-5.8646285	8.23E-09	1.14E-07	8.45741094	8.08448537	up-regulated in High
LRRC10B	-0.2647048	1.26389606	-2.6240255	0.00895778	0.03171151	-4.7661923	2.047799546	up-regulated in High
SYT7	0.26188524	3.11723513	2.00892803	0.04508663	0.12028114	-6.1772913	1.345952231	up-regulated in Low

TMEM258	0.23391089	4.71106845	5.51216779	5.71E-08	6.89E-07	6.5731846	7.243348113	up-regulated in Low
FEN1	1.00521979	3.76392555	17.7868144	4.24E-55	3.26E-52	114.273118	54.37212556	up-regulated in Low
FADS2	0.50756898	3.0163965	4.65913881	4.09E-06	3.42E-05	2.44646902	5.388736131	up-regulated in Low
FADS1	0.52767142	2.82165318	7.11532209	3.93E-12	9.21E-11	15.9426019	11.4051625	up-regulated in Low
INCENP	0.80440669	2.60295008	14.1593921	1.84E-38	4.92E-36	76.0829077	37.73590058	up-regulated in Low
SCGB2A1	-0.4810598	1.42389985	-3.3002738	0.0010355	0.00490943	-2.7958742	2.984850638	up-regulated in High
SCGB1D2	0.12177552	0.12056331	2.57175764	0.01040873	0.0359819	-4.9003214	1.982602421	up-regulated in Low
SCGB1A1	-2.4534623	4.61983425	-8.1072302	4.09E-15	1.43E-13	22.7074322	14.38859436	up-regulated in High
AHNAK	-0.6133567	4.74752143	-6.0591741	2.71E-09	4.11E-08	9.54093664	8.567009751	up-regulated in High
MTA2	0.325645	4.62414804	9.00767245	4.55E-18	2.35E-16	29.4265928	17.34202428	up-regulated in Low
EML3	-0.1689091	3.36763097	-3.8710837	0.00012286	0.000749	-0.7987247	3.910603021	up-regulated in High
ROM1	-0.2392344	1.26220241	-5.5604354	4.41E-08	5.42E-07	6.82514861	7.355994122	up-regulated in High
B3GAT3	0.21518936	4.25358588	3.96834583	8.31E-05	0.00052793	-0.4284931	4.080563151	up-regulated in Low
GANAB	0.31154601	6.58301392	6.91952618	1.41E-11	3.04E-10	14.6913491	10.85181842	up-regulated in Low
INTS5	-0.1072796	3.74050933	-2.4871843	0.01320378	0.04408028	-5.1117841	1.879301723	up-regulated in High
C11orf98	0.32541918	2.494468	6.85043841	2.19E-11	4.60E-10	14.2566937	10.65946312	up-regulated in Low
METTL12	0.12401686	1.04911442	3.29070565	0.00107058	0.00505441	-2.8267682	2.970381763	up-regulated in Low
UQCC3	0.38347863	3.29487834	6.40584483	3.48E-10	6.08E-09	11.546822	9.458409576	up-regulated in Low
LRRN4CL	-0.2222114	0.53173322	-5.8884145	7.20E-09	1.01E-07	8.58824171	8.142789912	up-regulated in High
TTC9C	0.14218259	3.09507476	3.92004647	0.00010099	0.00062973	-0.6134254	3.995720312	up-regulated in Low
ZBTB3	-0.1382562	1.35728897	-4.4064932	1.29E-05	9.76E-05	1.34554372	4.889872715	up-regulated in High
POLR2G	0.26448205	4.83153979	6.11162119	2.00E-09	3.11E-08	9.83826379	8.699285956	up-regulated in Low
TMEM223	0.28954307	3.26985329	6.09226034	2.24E-09	3.45E-08	9.7282487	8.650348277	up-regulated in Low
NXF1	-0.1477578	3.72447502	-3.2111364	0.00140798	0.00644566	-3.0803547	2.851402645	up-regulated in High
WDR74	0.26902632	2.91801697	7.6648045	9.52E-14	2.78E-12	19.6040953	13.02147339	up-regulated in Low
SLC3A2	0.46417977	5.06781987	8.45598212	3.12E-16	1.28E-14	25.2473452	15.5059614	up-regulated in Low
HRASLS5	-0.2438657	0.59950162	-3.0282804	0.00258772	0.01092789	-3.6405128	2.587083261	up-regulated in High
RARRES3	-0.5691709	5.60112393	-4.4784024	9.34E-06	7.28E-05	1.65313924	5.029479194	up-regulated in High
PLA2G16	-0.499591	4.06486739	-4.5299375	7.40E-06	5.89E-05	1.87640699	5.130698998	up-regulated in High
RTN3	0.15353511	5.05600567	3.52172483	0.00046837	0.00244495	-2.0569324	3.32940998	up-regulated in Low
C11orf84	0.50628007	3.30954115	9.09765337	2.24E-18	1.21E-16	30.1263618	17.64919022	up-regulated in Low
MARK2	0.15700252	3.88658773	3.46091489	0.00058486	0.00296658	-2.2643998	3.232947216	up-regulated in Low
RCOR2	0.75373467	1.25237452	9.25006484	6.69E-19	3.83E-17	31.3229255	18.17426948	up-regulated in Low
NAA40	0.18036328	2.72995851	3.5141286	0.00048163	0.002505	-2.0830367	3.317283715	up-regulated in Low
COX8A	0.39524331	7.75753032	8.20775109	1.96E-15	7.25E-14	23.4311903	14.70712305	up-regulated in Low
OTUB1	0.16679728	4.28965564	4.52933975	7.42E-06	5.91E-05	1.87380389	5.129519396	up-regulated in Low
STIP1	0.5095787	5.35432796	11.6033225	1.05E-27	1.48E-25	51.4321504	26.97712339	up-regulated in Low
FERMT3	-0.3703403	3.61308806	-4.4538464	1.04E-05	8.05E-05	1.54758276	4.981591751	up-regulated in High
TRPT1	0.10591533	3.57330593	2.14634154	0.0323306	0.09201475	-5.8939091	1.490386238	up-regulated in Low
DNAJC4	-0.2609954	3.81782868	-5.5332208	5.10E-08	6.21E-07	6.68284596	7.29238142	up-regulated in High
VEGFB	-0.1735025	5.76776269	-3.8445231	0.00013652	0.00082294	-0.8983257	3.864805736	up-regulated in High
PPP1R14B	0.52426269	5.78796691	8.02418896	7.45E-15	2.52E-13	22.1146836	14.12764079	up-regulated in Low
PLCB3	0.162638	3.45656409	3.17213067	0.00160711	0.0072258	-3.2024912	2.793954767	up-regulated in Low
ESRRA	0.16548671	4.09101561	3.15960087	0.0016764	0.00749061	-3.2414209	2.775623326	up-regulated in Low
TRMT112	0.21269	6.2617114	4.44833397	1.07E-05	8.24E-05	1.52396059	4.970872216	up-regulated in Low
PRDX5	-0.1358015	8.14740505	-2.2764156	0.02324682	0.07033799	-5.6087221	1.63363642	up-regulated in High
CCDC88B	-0.2737088	2.00743196	-4.038715	6.23E-05	0.00040657	-0.1552578	4.205733642	up-regulated in High
RASGRP2	-0.433284	1.05699083	-7.5344254	2.35E-13	6.44E-12	18.7156509	12.62964321	up-regulated in High
PYGM	-0.1161512	0.31348584	-4.4675698	9.81E-06	7.61E-05	1.60650824	5.008326908	up-regulated in High
MEN1	0.15205847	3.58149082	3.28811778	0.00108025	0.00509514	-2.8351092	2.966474391	up-regulated in Low
CDC42BPG	-0.2401509	2.82123687	-3.4452223	0.00061904	0.00312232	-2.31738	3.208280938	up-regulated in High
PPP2R5B	-0.1656387	2.82554789	-3.3293406	0.00093537	0.00448422	-2.7014961	3.02901785	up-regulated in High
BATF2	0.17403925	2.51219634	2.06687446	0.03926477	0.107672	-6.0600369	1.405996923	up-regulated in Low
ARL2	0.14895734	4.16014551	2.80913369	0.00516376	0.01987975	-4.2700986	2.287034197	up-regulated in Low
SAC3D1	0.42754569	2.82842225	8.38126999	5.45E-16	2.18E-14	24.696437	15.26371013	up-regulated in Low
NAALADL1	-0.2232452	0.8494888	-6.6873959	6.14E-11	1.20E-09	13.2452824	10.2115718	up-regulated in High
CDCA5	1.42957476	2.53123584	19.8745377	4.50E-65	1.10E-61	137.192433	64.34655389	up-regulated in Low
ZFPL1	0.25849544	2.02271202	8.62437323	8.76E-17	3.85E-15	26.5023644	16.05762858	up-regulated in Low
TM7SF2	-0.2143113	3.41398224	-2.6689594	0.0078582	0.02835002	-4.6487857	2.104676927	up-regulated in High
ZNHIT2	0.19184396	3.38478758	3.44982504	0.00060883	0.00307558	-2.3018643	3.215506088	up-regulated in Low
SYVN1	-0.1027685	4.45160321	-2.0731173	0.03867757	0.10634647	-6.0472089	1.412540819	up-regulated in High
SPDYC	0.16686002	0.28554858	2.22701186	0.026395	0.07804348	-5.7189764	1.578478421	up-regulated in Low
POLA2	0.54948764	2.26933171	13.0097219	1.70E-33	3.44E-31	64.6953126	32.76875266	up-regulated in Low
CDC42EP2	0.14677404	2.66068628	2.12532616	0.03405346	0.09596405	-5.9384408	1.467838815	up-regulated in Low
TIGD3	0.13908412	0.62045143	3.76645997	0.00018547	0.00108083	-1.1873165	3.731736293	up-regulated in Low
SLC25A45	-0.1325369	1.3428008	-3.1595461	0.00167671	0.00749084	-3.2415909	2.77554326	up-regulated in High
MALAT1	-0.4170548	4.56012173	-3.5196383	0.00047198	0.00246159	-2.064108	3.326077002	up-regulated in High
SCYL1	0.10272376	4.72714193	2.39940716	0.01678991	0.05374225	-5.3239613	1.774951658	up-regulated in Low
LTBP3	-0.5844894	4.09654709	-8.2742448	1.20E-15	4.56E-14	23.913676	14.9194074	up-regulated in High
SSSCA1	0.2221373	3.49338905	4.79780702	2.13E-06	1.89E-05	3.07461625	5.672447528	up-regulated in Low
SIPA1	-0.123853	3.89713704	-2.4159684	0.016054	0.05180704	-5.2844986	1.794416765	up-regulated in High

PCNXL3	0.22672221	3.62913555	4.61072809	5.11E-06	4.20E-05	2.23114736	5.291337183	up-regulated in Low
OVOL1	0.2092989	1.17268465	3.29288295	0.0010625	0.00502114	-2.8197456	2.973671202	up-regulated in Low
CFL1	0.30988865	7.16470725	8.35367839	6.69E-16	2.63E-14	24.4939122	15.17463949	up-regulated in Low
MUS81	0.18668181	2.84899262	4.71885308	3.09E-06	2.65E-05	2.71490258	5.510053296	up-regulated in Low
EFEMP2	-0.3591054	3.04521559	-5.1310415	4.14E-07	4.24E-06	4.6524074	6.382628472	up-regulated in High
CTSW	-0.2255613	2.60384247	-2.2180447	0.02700441	0.07958253	-5.7387343	1.568565308	up-regulated in High
FIBP	0.17986475	4.09254061	4.93096332	1.12E-06	1.05E-05	3.69357379	5.951437108	up-regulated in Low
CCDC85B	0.20790413	3.25161511	2.77999807	0.00564266	0.02145605	-4.3503569	2.248516508	up-regulated in Low
FOSL1	0.81841858	2.2264984	6.54852962	1.46E-10	2.70E-09	12.3998956	9.836863925	up-regulated in Low
DRAP1	0.35529811	5.29344251	6.49101944	2.07E-10	3.75E-09	12.054151	9.683521735	up-regulated in Low
SART1	0.24710848	4.19848901	4.84260531	1.72E-06	1.56E-05	3.28113507	5.765594177	up-regulated in Low
EIF1AD	0.2056	3.38906706	6.48022936	2.21E-10	4.00E-09	11.9895686	9.654872273	up-regulated in Low
BANF1	0.45078547	6.07337839	9.99575725	1.49E-21	1.18E-19	37.3745407	20.82719563	up-regulated in Low
CST6	-0.6282695	3.27433156	-3.9006969	0.00010915	0.00067382	-0.6869156	3.961975542	up-regulated in High
SF3B2	0.25991567	4.7448112	7.61771511	1.32E-13	3.76E-12	19.2818232	12.87936642	up-regulated in Low
PACS1	0.27397296	3.95761711	5.11456668	4.50E-07	4.59E-06	4.57215665	6.346582244	up-regulated in Low
KLC2	0.34254545	2.95419057	6.90337113	1.56E-11	3.35E-10	14.5893898	10.80670324	up-regulated in Low
RAB1B	0.11375364	6.59569615	3.04322381	0.00246486	0.01048109	-3.595921	2.608207742	up-regulated in Low
CNIH2	0.26789202	0.7045439	5.21315589	2.73E-07	2.90E-06	5.05584987	6.5637319	up-regulated in Low
YIF1A	0.20529994	5.11071174	3.91256893	0.00010407	0.00064644	-0.6418657	3.982663243	up-regulated in Low
BRMS1	0.29254994	4.39174424	7.14381432	3.26E-12	7.70E-11	16.1270593	11.48668853	up-regulated in Low
B4GAT1	-0.3044667	3.52837626	-6.0674537	2.58E-09	3.93E-08	9.58772777	8.587830006	up-regulated in High
SLC29A2	0.16626981	3.35361937	2.20402872	0.02798134	0.08181816	-5.7694598	1.553131509	up-regulated in Low
MRPL11	0.56237558	4.05690784	11.8219841	1.40E-28	2.06E-26	53.4400635	27.85446882	up-regulated in Low
DPP3	0.34309925	4.15063093	7.36217224	7.59E-13	1.94E-11	17.5604832	12.1198491	up-regulated in Low
CTSF	-0.4635402	4.01275178	-5.7885084	1.26E-08	1.70E-07	8.04182651	7.899199935	up-regulated in High
RBM14	0.21338668	3.75656123	6.36496972	4.45E-10	7.65E-09	11.3053711	9.351227372	up-regulated in Low
RBM4	0.11460778	2.02127349	3.86564755	0.00012554	0.00076363	-0.8191625	3.901208244	up-regulated in Low
RBM4B	0.16893984	2.20170992	4.59100894	5.60E-06	4.56E-05	2.14403184	5.251908858	up-regulated in Low
SPTBN2	0.17577425	2.69742808	2.28884956	0.02250798	0.0684109	-5.5806	1.647663482	up-regulated in Low
C11orf80	0.10860276	2.8108313	1.9749119	0.04883275	0.12808353	-6.2445949	1.31128882	up-regulated in Low
RCE1	0.30155951	3.11505294	7.79556105	3.81E-14	1.17E-12	20.5071506	13.41953591	up-regulated in Low
LRFN4	0.72527477	2.55616886	8.53884108	1.67E-16	7.09E-15	25.8625948	15.77644039	up-regulated in Low
C11orf86	0.4150121	0.83835381	3.75306279	0.00019538	0.00113236	-1.2363504	3.709128999	up-regulated in Low
ADRBK1	0.13156199	4.40212443	2.6643781	0.00796448	0.02866965	-4.6608447	2.098842858	up-regulated in Low
RAD9A	0.20867303	2.44458517	3.83459261	0.00014199	0.00085246	-0.9353988	3.847750863	up-regulated in Low
PPP1CA	0.24290453	6.21285291	6.19524428	1.22E-09	1.96E-08	10.3168804	8.912103461	up-regulated in Low
TBC1D10C	-0.3398975	1.72649916	-4.650892	4.25E-06	3.55E-05	2.40964296	5.372083661	up-regulated in High
CARNS1	-0.1816226	0.65527241	-3.6954321	0.00024398	0.00137808	-1.4453965	3.612648471	up-regulated in High
RPS6KB2	0.24335996	3.35128019	5.86234803	8.34E-09	1.16E-07	8.44489161	8.078905519	up-regulated in Low
CORO1B	0.11825488	4.69656585	2.6271991	0.00887582	0.03147288	-4.7579636	2.051791583	up-regulated in Low
GSTP1	0.30238923	8.3726731	3.87977799	0.00011867	0.00072584	-0.7659811	3.92565175	up-regulated in Low
NDUFV1	0.24240889	4.92535704	5.50180568	6.04E-08	7.25E-07	6.51934603	7.219271175	up-regulated in Low
NUDT8	0.20165415	2.66644302	2.87610703	0.00419956	0.01663273	-4.0825397	2.376796316	up-regulated in Low
TBX10	0.10646145	0.1739996	2.32987881	0.02021328	0.06262631	-5.48674	1.694363202	up-regulated in Low
ALDH3B2	0.56011832	1.83708203	5.03305832	6.77E-07	6.65E-06	4.17854209	6.169671812	up-regulated in Low
ALDH3B1	-0.4044074	3.93245125	-3.7341007	0.00021026	0.00120914	-1.3054699	3.677246371	up-regulated in High
NDUFS8	0.27658485	4.17510139	5.34618239	1.37E-07	1.54E-06	5.72158874	6.862196252	up-regulated in Low
CHKA	-0.213605	3.2551116	-2.9579925	0.00324444	0.0132973	-3.8474156	2.488859901	up-regulated in High
C11orf24	0.30774668	4.00207322	6.09159761	2.25E-09	3.46E-08	9.72448819	8.648675373	up-regulated in Low
PPP6R3	0.24802564	3.8031056	7.15162906	3.10E-12	7.34E-11	16.1777564	11.50909347	up-regulated in Low
GAL	1.00620999	0.86446447	9.55071279	5.92E-20	3.87E-18	33.7240613	19.22738807	up-regulated in Low
MTL5	0.7730237	0.81538454	14.1473652	2.07E-38	5.51E-36	75.9615713	37.68299595	up-regulated in Low
CPT1A	0.12248079	3.43242013	1.97516184	0.0488043	0.12802303	-6.2441045	1.311541935	up-regulated in Low
MRPL21	0.50155676	3.52462487	11.0483589	1.61E-25	1.88E-23	46.4342822	24.79229705	up-regulated in Low
IGHMBP2	0.13882284	2.03698116	3.66555261	0.00027345	0.00152188	-1.5525742	3.56311871	up-regulated in Low
MRGPRF	-0.2428967	1.55000906	-3.6192995	0.00032575	0.00177556	-1.7168584	3.487110196	up-regulated in High
MYEOV	0.84162724	1.62698422	5.80322673	1.16E-08	1.57E-07	8.12181331	7.934871223	up-regulated in Low
CCND1	-0.372126	5.69292517	-3.6546793	0.00028499	0.00157869	-1.5913725	3.545177905	up-regulated in High
ORAOV1	0.11914842	1.57885816	2.98842696	0.00294334	0.01221585	-3.7584025	2.531159986	up-regulated in Low
ANO1	-0.2784203	2.15491348	-2.7808224	0.00562857	0.02140807	-4.3480972	2.2496019	up-regulated in High
FADD	0.22795384	2.89512077	5.74349035	1.62E-08	2.14E-07	7.79827809	7.790556634	up-regulated in Low
PPFIA1	0.13555196	2.86194924	3.54649737	0.00042746	0.00225334	-1.9714298	3.369107058	up-regulated in Low
SHANK2	-0.1552139	1.38709426	-2.8994796	0.00390371	0.0156167	-4.0160789	2.408522798	up-regulated in High
DHCR7	0.37481441	4.15378559	5.51522854	5.62E-08	6.79E-07	6.58910449	7.250467093	up-regulated in Low
FAM86C1	0.17977132	1.76023878	5.02636378	6.99E-07	6.86E-06	4.14646675	6.155247216	up-regulated in Low
RNF121	0.13064606	3.03872512	3.77370395	0.00018031	0.00105323	-1.1607346	3.743988368	up-regulated in Low
LAMTOR1	0.11953391	5.17731701	2.86343528	0.00436836	0.01720012	-4.118355	2.359682109	up-regulated in Low
ANAPC15	0.21138505	2.26522219	5.36005229	1.28E-07	1.44E-06	5.79186274	6.893675164	up-regulated in Low
FOLR1	-1.5681747	5.54885549	-8.6379616	7.90E-17	3.49E-15	26.6044379	16.10248491	up-regulated in High
FOLR2	-0.8717139	3.58495906	-8.2261981	1.71E-15	6.38E-14	23.5647476	14.76589033	up-regulated in High

CLPB	0.42247827	2.26802656	9.71126186	1.59E-20	1.10E-18	35.0279672	19.79898435	up-regulated in Low
PDE2A	-0.1650072	0.8460186	-3.9298253	9.71E-05	0.00060755	-0.576155	4.012827461	up-regulated in High
ARAP1	-0.2895886	4.00424333	-5.6060283	3.44E-08	4.30E-07	7.06493028	7.463143173	up-regulated in High
ATG16L2	-0.3298748	1.80167989	-4.6451481	4.36E-06	3.64E-05	2.38402915	5.360499961	up-regulated in High
FCHSD2	-0.3362373	3.22858169	-7.930503	1.46E-14	4.75E-13	21.4516017	13.83563137	up-regulated in High
P2RY2	-0.182755	1.84126194	-3.0143292	0.00270746	0.01135636	-3.6819529	2.567437965	up-regulated in High
ARHGEF17	-0.5359537	2.89243425	-9.7041769	1.68E-20	1.17E-18	34.9701137	19.77362703	up-regulated in High
RELT	0.11933887	1.54755167	2.47268137	0.01374487	0.04554088	-5.1473539	1.861859512	up-regulated in Low
PLEKHB1	-0.3809773	2.85455704	-4.0112315	6.97E-05	0.00045172	-0.2625085	4.156626943	up-regulated in High
RAB6A	0.22887723	4.94437698	5.60949567	3.38E-08	4.23E-07	7.08323616	7.471321368	up-regulated in Low
MRPL48	0.36846831	2.57672978	8.17194503	2.55E-15	9.28E-14	23.172604	14.5933307	up-regulated in Low
COA4	0.40570502	4.47704496	8.6104545	9.73E-17	4.25E-15	26.3979324	16.01173398	up-regulated in Low
DNAJB13	-0.3337362	0.97382329	-5.0813572	5.32E-07	5.34E-06	4.41109588	6.274215809	up-regulated in High
C2CD3	0.15071623	1.89345672	3.6600941	0.00027919	0.00155024	-1.5720649	3.554106696	up-regulated in Low
PPME1	0.31005838	3.2391087	7.64394591	1.10E-13	3.18E-12	19.4611492	12.95844422	up-regulated in Low
PGM2L1	0.165208	2.42931948	2.28103987	0.02296961	0.06963997	-5.5982808	1.638846379	up-regulated in Low
LIPT2	0.25141024	1.26714377	5.96427139	4.68E-09	6.80E-08	9.00854896	8.330019754	up-regulated in Low
POLD3	0.32811261	2.44996243	9.04921974	3.28E-18	1.73E-16	29.7490813	17.4835903	up-regulated in Low
SPCS2	0.14947565	4.19556933	3.69886178	0.00024079	0.00136193	-1.4330413	3.618355261	up-regulated in Low
SLCO2B1	-0.6388428	3.05687949	-7.1472581	3.19E-12	7.54E-11	16.1493949	11.49655958	up-regulated in High
ARRB1	-0.4658575	3.27313614	-6.7278924	4.76E-11	9.51E-10	13.4946061	10.32202143	up-regulated in High
RPS3	0.23565303	7.55907453	3.50190465	0.00050371	0.00260338	-2.1249314	3.297815754	up-regulated in Low
GDPD5	-0.2190003	1.31735152	-4.0455465	6.05E-05	0.000397	-0.1284921	4.217983767	up-regulated in High
MAP6	-0.5138148	1.00550324	-9.3315651	3.49E-19	2.06E-17	31.9685278	18.45749589	up-regulated in High
WNT11	-0.2475896	0.8464039	-3.6043905	0.00034452	0.00186473	-1.7693913	3.462781793	up-regulated in High
PRKRIR	0.17779742	2.6718307	4.00621382	7.12E-05	0.00046009	-0.2820154	4.147691877	up-regulated in Low
LRRC32	-0.3455808	2.9419182	-4.9375375	1.08E-06	1.02E-05	3.7245311	5.96537697	up-regulated in High
TSKU	0.75055174	3.93597274	7.95551064	1.22E-14	4.03E-13	21.6280098	13.91332805	up-regulated in Low
B3GNT6	-0.5961557	1.81439494	-3.7274895	0.00021569	0.00123601	-1.3294907	3.666162227	up-regulated in High
MYO7A	0.11390287	1.41766685	2.12838797	0.03379765	0.09534902	-5.9319796	1.471113514	up-regulated in Low
PAK1	0.1581408	3.85736917	2.97526446	0.00307028	0.01267064	-3.7970074	2.512822583	up-regulated in Low
CLNS1A	0.30373201	4.05218269	7.67893318	8.63E-14	2.54E-12	19.701095	13.06424038	up-regulated in Low
AQP11	0.11447003	0.83684755	3.51434937	0.00048124	0.00250386	-2.0822788	3.317635845	up-regulated in Low
AAMDC	-0.1152438	2.75443644	-2.4110181	0.01627093	0.0523624	-5.2963223	1.788587519	up-regulated in High
INTS4	0.15072796	2.38688506	4.29382594	2.11E-05	0.00015311	0.87287176	4.674967566	up-regulated in Low
KCTD14	-0.327406	1.96495778	-3.7783189	0.00017709	0.00103699	-1.1437748	3.751804168	up-regulated in High
NDUFC2	0.12146052	3.83059034	2.90276046	0.00386373	0.01548118	-4.0067078	2.412993005	up-regulated in Low
ALG8	0.3479524	3.67324106	7.82925526	3.00E-14	9.34E-13	20.741792	13.52293147	up-regulated in Low
USP35	0.10194874	1.51713024	2.41031148	0.01630211	0.05244538	-5.298008	1.787756263	up-regulated in Low
GAB2	-0.1602336	2.83186745	-2.3914665	0.01715318	0.05467423	-5.3427886	1.765655419	up-regulated in High
NARS2	0.23476329	2.96977619	4.87091097	1.50E-06	1.38E-05	3.41252327	5.824822668	up-regulated in Low
TENM4	-0.1626236	0.94261239	-3.0161256	0.00269176	0.0112981	-3.6766272	2.56996345	up-regulated in High
DDIAS	0.7426295	1.07913488	16.243274	7.07E-48	3.30E-45	97.6877429	47.15045315	up-regulated in Low
RAB30	-0.2017787	1.17380811	-4.9126145	1.22E-06	1.15E-05	3.60736805	5.912612502	up-regulated in High
ANKRD42	-0.1206122	1.56968234	-3.3421478	0.00089417	0.00431114	-2.6596608	3.048580038	up-regulated in High
CCDC90B	0.26546084	2.37843067	5.10671732	4.68E-07	4.76E-06	4.53400328	6.329442267	up-regulated in Low
DLG2	-0.1097736	0.34408644	-5.0782756	5.40E-07	5.41E-06	4.39619849	6.267520684	up-regulated in High
TMEM126A	0.22471235	4.04463626	4.80195355	2.08E-06	1.86E-05	3.09365822	5.681038674	up-regulated in Low
CCDC89	-0.1716317	0.55777365	-3.6857445	0.00025319	0.00142295	-1.480236	3.596553039	up-regulated in High
SYTL2	-0.5408456	2.16763959	-7.3282294	9.54E-13	2.41E-11	17.3353797	12.02046123	up-regulated in High
EED	0.27563694	1.99636602	8.2686337	1.25E-15	4.73E-14	23.872847	14.90144525	up-regulated in Low
C11orf73	0.16652555	2.94908426	4.54400771	6.94E-06	5.56E-05	1.93777275	5.15850345	up-regulated in Low
CCDC81	-0.1495325	0.27968048	-4.6393031	4.48E-06	3.73E-05	2.35799433	5.348724745	up-regulated in High
TMEM135	0.18153176	2.5335534	3.68639043	0.00025257	0.00141985	-1.4779158	3.597625126	up-regulated in Low
RAB38	-0.4634168	3.17687981	-5.1873736	3.11E-07	3.27E-06	4.92855803	6.506610813	up-regulated in High
CHORDC1	0.34723668	1.79020654	8.36665916	6.07E-16	2.41E-14	24.5891296	15.21651717	up-regulated in Low
SLC36A4	0.23342223	1.68936078	5.16609587	3.47E-07	3.62E-06	4.82393229	6.459647502	up-regulated in Low
CCDC67	0.10818924	0.27811874	3.40680548	0.00071074	0.00352425	-2.446111	3.148288917	up-regulated in Low
SMCO4	-0.1272374	4.55371984	-2.1251072	0.03407181	0.09600538	-5.9389025	1.467604775	up-regulated in High
CEP295	0.17552325	1.17819598	4.77496925	2.37E-06	2.08E-05	2.97000782	5.625241847	up-regulated in Low
TAF1D	0.18770221	3.14548946	3.79325166	0.00016705	0.00098452	-1.0887636	3.777148633	up-regulated in Low
C11orf54	-0.310493	2.76035457	-5.8295255	1.00E-08	1.37E-07	8.26517469	7.998793648	up-regulated in High
PANX1	0.16663413	3.31462259	3.22752317	0.00133135	0.0061328	-3.0286167	2.87570928	up-regulated in Low
MRE11A	0.1610437	2.19907944	4.24271755	2.64E-05	0.00018704	0.66220319	4.579027993	up-regulated in Low
FUT4	0.10998145	1.53263138	2.10803733	0.03552926	0.09928317	-5.9747534	1.449413845	up-regulated in Low
AMOTL1	-0.1573643	2.18375686	-2.299387	0.02189794	0.06692604	-5.5566498	1.659596712	up-regulated in High
CWC15	0.11681208	4.1755732	3.04951089	0.00241479	0.01029592	-3.5770969	2.617120692	up-regulated in Low
SRSF8	-0.2450536	3.91277133	-6.2337552	9.75E-10	1.58E-08	10.5391704	9.010899841	up-regulated in High
ENDOD1	-0.574032	4.00199057	-6.8190299	2.68E-11	5.55E-10	14.0602837	10.57251818	up-regulated in High
SESN3	-0.7602601	2.65823874	-9.9837989	1.65E-21	1.29E-19	37.2749937	20.78358801	up-regulated in High
MTMR2	0.36170199	2.90048502	8.28930198	1.08E-15	4.11E-14	24.0233418	14.96765181	up-regulated in Low

MAML2	-0.5968198	2.21296053	-8.9720658	6.01E-18	3.06E-16	29.1510641	17.22106077	up-regulated in High
ARHGAP42	-0.2955998	1.76107456	-5.0860265	5.20E-07	5.23E-06	4.43368404	6.284366777	up-regulated in High
TMEM133	-0.4794731	2.25430397	-7.8909692	1.94E-14	6.18E-13	21.1736023	13.71317551	up-regulated in High
PGR	-0.1596124	0.22002211	-8.438404	3.56E-16	1.46E-14	25.1173992	15.44882518	up-regulated in High
TRPC6	-0.6480813	1.85215019	-6.7295214	4.72E-11	9.43E-10	13.5046616	10.32647541	up-regulated in High
C11orf70	-0.2578629	1.25691993	-3.7681136	0.00018428	0.00107432	-1.1812526	3.734531464	up-regulated in High
YAP1	-0.3220798	4.46917033	-5.140224	3.96E-07	4.06E-06	4.69723672	6.402761217	up-regulated in High
MMP7	-0.9295113	4.29397297	-5.0979853	4.89E-07	4.95E-06	4.49162163	6.310400773	up-regulated in High
MMP12	0.92737778	3.22975975	5.40462494	1.01E-07	1.17E-06	6.01879357	6.995294899	up-regulated in Low
MMP13	-0.6829971	2.07774062	-3.9094329	0.00010539	0.00065352	-0.6537783	3.977193395	up-regulated in High
DCUN1D5	0.46357988	3.92290835	9.37211381	2.52E-19	1.51E-17	32.2912126	18.59903815	up-regulated in Low
DYNC2H1	-0.2916869	0.86783501	-6.343925	5.06E-10	8.61E-09	11.1815704	9.296259463	up-regulated in High
PDGFD	-0.4899067	1.74668823	-6.8846431	1.76E-11	3.74E-10	14.4714373	10.75450635	up-regulated in High
CASP4	-0.1727908	3.59926207	-2.8852318	0.00408171	0.01622873	-4.056655	2.389157792	up-regulated in High
CASP1	-0.4753734	2.83853103	-6.4005812	3.59E-10	6.26E-09	11.515656	9.444576398	up-regulated in High
CARD16	-0.3586321	2.34997318	-5.211814	2.75E-07	2.92E-06	5.04921096	6.560753188	up-regulated in High
MSANTD4	-0.2361371	2.35011769	-5.0842074	5.24E-07	5.27E-06	4.42488206	6.280411297	up-regulated in High
KBTBD3	-0.2107948	1.10674851	-6.0757527	2.46E-09	3.76E-08	9.63468368	8.608722215	up-regulated in High
GUCY1A2	-0.299376	0.69861253	-7.8008437	3.67E-14	1.13E-12	20.5438863	13.43572449	up-regulated in High
SLC35F2	0.16838662	3.53900892	2.30599945	0.02152255	0.06600331	-5.5415658	1.667106342	up-regulated in Low
RAB39A	0.13799465	0.48062984	3.37515767	0.00079563	0.00388872	-2.5511249	3.09928693	up-regulated in Low
ATM	-0.1190066	1.69088719	-2.6869323	0.00745347	0.02709589	-4.6012826	2.127641514	up-regulated in High
KDELC2	0.20285765	3.46905514	3.17822131	0.00157439	0.00710332	-3.1835143	2.802887061	up-regulated in Low
EXPH5	-0.3306564	1.82420918	-5.4205904	9.29E-08	1.08E-06	6.10048399	7.031863824	up-regulated in High
DDX10	0.33293414	1.99708311	8.78660137	2.53E-17	1.18E-15	27.7287007	16.59642992	up-regulated in Low
RDX	0.33104836	3.90697528	5.63060734	3.01E-08	3.80E-07	7.19491103	7.521206398	up-regulated in Low
ARHGAP20	-0.5842106	1.01706902	-9.3648714	2.67E-19	1.60E-17	32.2335058	18.57372664	up-regulated in High
C11orf53	0.26875864	0.43213598	3.14185433	0.00177926	0.00789343	-3.2963057	2.749761594	up-regulated in Low
COLCA2	-0.3912891	1.42143282	-4.8280304	1.84E-06	1.66E-05	3.21375351	5.735209715	up-regulated in High
POU2AF1	-0.3510772	1.84731726	-3.4679199	0.00057018	0.00290043	-2.2406758	3.243988074	up-regulated in High
C11orf88	-0.3964802	0.48500411	-5.8157188	1.08E-08	1.48E-07	8.18984054	7.96520528	up-regulated in High
LAYN	-0.2322292	1.85829987	-3.2194114	0.00136879	0.00628928	-3.0542596	2.863664301	up-regulated in High
PPP2R1B	0.40599523	2.75476594	7.25602706	1.55E-12	3.81E-11	16.8593322	11.81022457	up-regulated in Low
ALG9	0.18869577	1.12741998	4.43770968	1.12E-05	8.60E-05	1.47850908	4.950243654	up-regulated in Low
C11orf1	-0.1534131	2.27129848	-2.8423509	0.00466293	0.01818778	-4.1776082	2.331341214	up-regulated in High
CRYAB	-0.1644093	1.92964675	-2.0621863	0.03971072	0.10874676	-6.0696453	1.401092258	up-regulated in High
HSPB2	-0.1881352	0.5839524	-6.4696789	2.36E-10	4.24E-09	11.9265082	9.626896001	up-regulated in High
DIXDC1	-0.2437128	1.48494076	-5.9022524	6.66E-09	9.39E-08	8.66456595	8.176798202	up-regulated in High
DLAT	0.2584399	3.61413754	5.8923378	7.04E-09	9.88E-08	8.60986514	8.152425216	up-regulated in Low
PIH1D2	-0.1557313	0.88428917	-3.562269	0.00040318	0.00214238	-1.9166972	3.394501176	up-regulated in High
TIMM8B	0.33882256	4.58624535	5.32399192	1.54E-07	1.72E-06	5.60949481	6.81197414	up-regulated in Low
BCO2	-0.1003604	0.28433395	-4.0046415	7.16E-05	0.00046255	-0.2881232	4.144893991	up-regulated in High
PTS	0.31596299	2.63820565	6.78334817	3.36E-11	6.86E-10	13.8380597	10.47412735	up-regulated in Low
TTC12	-0.1981928	1.64958758	-4.339745	1.73E-05	0.00012782	1.06414615	4.761989522	up-regulated in High
ANKK1	-0.2125015	0.40202548	-5.4066612	1.00E-07	1.15E-06	6.02920061	6.999953989	up-regulated in High
ZW10	0.22627353	2.94682336	5.34649474	1.37E-07	1.54E-06	5.72316955	6.862904422	up-regulated in Low
ZBTB16	-0.4815567	0.6606954	-7.264566	1.46E-12	3.62E-11	16.9154334	11.83500421	up-regulated in High
CADM1	-0.7422203	2.97927982	-6.9623443	1.07E-11	2.34E-10	14.9625358	10.9717945	up-regulated in High
BUD13	0.15429068	3.43005646	4.29365614	2.12E-05	0.00015321	0.87216796	4.674647218	up-regulated in Low
ZPR1	0.26911975	2.94244796	7.68390951	8.33E-14	2.45E-12	19.7352932	13.07931774	up-regulated in Low
APOC3	0.10545502	0.08982321	2.1982727	0.02839131	0.08283032	-5.7820227	1.546814612	up-regulated in Low
PAFAH1B2	0.15912993	4.08052121	4.19976799	3.17E-05	0.00022116	0.48697962	4.499152082	up-regulated in Low
SIDT2	-0.3814164	2.8908429	-8.4812846	2.58E-16	1.07E-14	25.4347486	15.58835576	up-regulated in High
TAGLN	-0.4387046	5.35021328	-4.55877	6.49E-06	5.22E-05	2.00234513	5.187753473	up-regulated in High
PCSK7	-0.1238586	1.22534578	-3.9049983	0.00010728	0.00066384	-0.6706081	3.969464981	up-regulated in High
RNF214	0.13552375	2.24574396	3.82611322	0.00014682	0.00087849	-0.9669831	3.833217446	up-regulated in Low
BACE1	-0.2649815	2.51612985	-5.0029945	7.85E-07	7.63E-06	4.03480047	6.105019764	up-regulated in High
FXYD6	-0.3539297	1.95604003	-5.6914132	2.16E-08	2.79E-07	7.51861666	7.665748121	up-regulated in High
IL10RA	-0.422665	2.71744554	-5.4336084	8.67E-08	1.01E-06	6.16725179	7.06174793	up-regulated in High
TMPRSS4	-0.3498905	3.14850199	-2.5280231	0.01178026	0.03999383	-5.0105332	1.928845223	up-regulated in High
SCN4B	-0.6347661	0.93619154	-12.186675	4.60E-30	7.50E-28	56.8350921	29.33742291	up-regulated in High
SCN2B	-0.2067858	0.27952562	-7.7957258	3.80E-14	1.17E-12	20.5082961	13.42004073	up-regulated in High
AMICA1	-0.6470844	1.78316305	-9.6251445	3.22E-20	2.15E-18	34.3267112	19.49159698	up-regulated in High
MPZL3	-0.2970109	3.24045984	-4.7393526	2.81E-06	2.43E-05	2.80777441	5.55199963	up-regulated in High
MPZL2	-0.9260508	4.98292481	-9.6717101	2.20E-20	1.50E-18	34.705367	19.6575831	up-regulated in High
CD3E	-0.3430935	3.00391854	-3.5849609	0.00037051	0.00198631	-1.8375442	3.431202447	up-regulated in High
CD3D	-0.2377503	3.26613796	-2.3283025	0.0202975	0.06284724	-5.490376	1.692557356	up-regulated in High
CD3G	-0.1943084	1.34071033	-2.989408	0.00293407	0.01218519	-3.7555186	2.532529369	up-regulated in High
ATP5L	0.1751572	5.33979754	3.43762024	0.00063626	0.00319537	-2.342963	3.196365207	up-regulated in Low
TMEM25	-0.3116208	2.35332415	-5.4875297	6.51E-08	7.78E-07	6.44531879	7.186161517	up-regulated in High
IFT46	-0.1867511	2.68542418	-3.5578995	0.00040977	0.00217346	-1.9318838	3.387456412	up-regulated in High

ARCN1	0.12062328	5.7703761	2.72225005	0.00671235	0.02484399	-4.5070342	2.173125522	up-regulated in Low
PHLDB1	-0.3140655	1.94134072	-6.4543315	2.59E-10	4.63E-09	11.8349311	9.586265037	up-regulated in High
UPK2	0.15716116	0.51786525	2.2799686	0.02303357	0.06981577	-5.6007015	1.637638717	up-regulated in Low
CCDC84	-0.2652735	2.31169562	-3.7159668	0.00022549	0.00128602	-1.3712609	3.646882716	up-regulated in High
HYOU1	0.25299283	5.09019804	3.81277481	0.00015474	0.0009203	-1.0165329	3.810410414	up-regulated in Low
VPS11	-0.1497247	3.37561793	-4.5608231	6.43E-06	5.18E-05	2.01134086	5.191827771	up-regulated in High
HMBS	0.43053275	2.96273319	8.62242418	8.89E-17	3.90E-15	26.4877332	16.05119873	up-regulated in Low
H2AFX	0.81868473	4.72559703	13.2680275	1.35E-34	2.93E-32	67.2148093	33.86810176	up-regulated in Low
C2CD2L	-0.1565301	1.79420218	-3.9563266	8.72E-05	0.00055115	-0.474712	4.059368565	up-regulated in High
NLRX1	-0.171885	2.48062005	-4.0556333	5.81E-05	0.00038306	-0.0888952	4.236102897	up-regulated in High
CCDC153	-0.2127579	0.97405269	-3.8645728	0.00012608	0.0007665	-0.8232	3.899352096	up-regulated in High
RNF26	0.29731557	4.20852257	5.96165411	4.75E-09	6.90E-08	8.99396945	8.323527163	up-regulated in Low
USP2	-0.2578495	0.84662878	-4.7068327	3.27E-06	2.79E-05	2.6606162	5.485528145	up-regulated in High
POU2F3	-0.3742991	0.98803934	-6.1886894	1.27E-09	2.03E-08	10.2791622	8.895336873	up-regulated in High
ARHGEF12	-0.2942086	3.18379532	-5.7228067	1.82E-08	2.38E-07	7.68693676	7.740873983	up-regulated in High
TBCEL	-0.2748034	2.0696131	-6.7275403	4.77E-11	9.53E-10	13.492433	10.32105886	up-regulated in High
SORL1	-0.3336693	2.77897793	-3.6380329	0.00030353	0.00166829	-1.6505585	3.517797948	up-regulated in High
CRTAM	-0.1308725	0.80640926	-2.6654105	0.00794041	0.02859538	-4.658129	2.10015685	up-regulated in High
C11orf63	-0.1377035	1.0466307	-2.943796	0.00339431	0.01383673	-3.888636	2.469248854	up-regulated in High
HSPA8	0.23246682	7.71283657	3.79093725	0.00016857	0.00099258	-1.0973031	3.773215028	up-regulated in Low
GRAMD1B	0.42202815	0.91768961	5.15842318	3.61E-07	3.74E-06	4.78629921	6.442752159	up-regulated in Low
VWA5A	-0.6299211	2.88420128	-8.866087	1.37E-17	6.62E-16	28.3356522	16.86301035	up-regulated in High
TBRG1	-0.1768243	2.39344699	-5.1801475	3.23E-07	3.39E-06	4.89298275	6.490643519	up-regulated in High
SIAE	-0.5139799	2.82311691	-7.0045139	8.12E-12	1.82E-10	15.2309577	11.09052035	up-regulated in High
SPA17	-0.1616558	1.57033453	-3.0942732	0.00208443	0.00905938	-3.4419931	2.681012423	up-regulated in High
NRGN	-1.1339661	3.75190673	-8.4803568	2.60E-16	1.08E-14	25.4278694	15.58533134	up-regulated in High
VSIG2	-1.7796084	3.24087583	-12.089128	1.15E-29	1.83E-27	55.9214652	28.93840598	up-regulated in High
ESAM	-0.8165404	3.83084941	-10.441287	3.35E-23	3.13E-21	41.1387647	22.47547518	up-regulated in High
MSANTD2	-0.3251499	1.80463188	-5.96691	4.61E-09	6.70E-08	9.02325264	8.336567499	up-regulated in High
AP000866.1	-0.1154484	0.49918432	-3.6681485	0.00027076	0.00150966	-1.5432953	3.567408513	up-regulated in High
ROBO3	-0.1007443	0.74852205	-2.1563565	0.03153628	0.09024695	-5.8725361	1.501189589	up-regulated in High
ROBO4	-0.3638036	1.85940142	-5.7066815	1.98E-08	2.58E-07	7.6003783	7.702243333	up-regulated in High
CCDC15	0.12821351	0.63819774	4.27665847	2.28E-05	0.00016387	0.80184617	4.642633355	up-regulated in Low
SLC37A2	-0.3651517	1.73883618	-5.225645	2.56E-07	2.74E-06	5.1177143	6.591486632	up-regulated in High
PKNOX2	-0.275333	0.56623713	-6.8946157	1.65E-11	3.53E-10	14.5342139	10.78228724	up-regulated in High
FEZ1	-0.292824	1.08168604	-6.7236955	4.89E-11	9.74E-10	13.4687085	10.31055013	up-regulated in High
EI24	0.12002233	5.23145034	2.9292149	0.00355479	0.01440643	-3.9307736	2.4491863	up-regulated in Low
STT3A	0.18930316	4.74378156	3.94575952	9.10E-05	0.00057278	-0.515238	4.04077938	up-regulated in Low
CHEK1	0.8964283	1.72027413	16.1959759	1.17E-47	5.38E-45	97.1861869	46.9319995	up-regulated in Low
ACRV1	0.13781053	0.16953797	8.64538446	7.46E-17	3.30E-15	26.6602471	16.1270096	up-regulated in Low
HYLS1	0.47668175	1.68611147	11.1460528	6.72E-26	8.06E-24	47.3035545	25.17241517	up-regulated in Low
CDON	-0.1277576	0.96040834	-2.1973492	0.02845757	0.08294902	-5.7840354	1.545802253	up-regulated in High
RPUSD4	0.10793962	2.7132587	2.9307492	0.00353758	0.01434566	-3.9263492	2.451293593	up-regulated in Low
FAM118B	-0.3088238	2.75625052	-5.8803868	7.53E-09	1.05E-07	8.54403519	8.123090677	up-regulated in High
FOXRED1	0.17066814	2.49018047	3.67059622	0.00026825	0.00149694	-1.5345406	3.57145568	up-regulated in Low
DCPS	0.21261388	2.77889662	5.00164123	7.90E-07	7.68E-06	4.02834853	6.102117205	up-regulated in Low
ST3GAL4	0.35868095	3.02778903	5.00515171	7.77E-07	7.56E-06	4.04508866	6.109648046	up-regulated in Low
ETS1	-0.3019458	3.83012463	-3.9869675	7.70E-05	0.00049345	-0.3566255	4.11350685	up-regulated in High
FLI1	-0.4231562	1.77815622	-7.8825405	2.06E-14	6.54E-13	21.1144715	13.68712671	up-regulated in High
KCNJ5	-0.4858048	1.01811231	-7.2180632	1.99E-12	4.84E-11	16.6105546	11.70032831	up-regulated in High
C11orf45	-0.1048723	0.8238373	-2.6411709	0.00852292	0.03038204	-4.7216221	2.069411866	up-regulated in High
TMEM45B	-0.2225706	4.06041035	-2.0992333	0.03630159	0.10103638	-5.993133	1.440074324	up-regulated in High
APLP2	-0.6229446	7.12223545	-9.7233195	1.44E-20	1.00E-18	35.1264922	19.84216727	up-regulated in High
ZBTB44	-0.1281751	2.56784938	-2.9520042	0.00330691	0.01351724	-3.8648265	2.480578282	up-regulated in High
ADAMTS8	-0.7427309	0.86236436	-11.276314	2.08E-26	2.56E-24	48.4697141	25.68227883	up-regulated in High
SNX19	-0.156129	3.47323474	-3.4923934	0.00052155	0.00268319	-2.157433	3.282707054	up-regulated in High
NTM	-0.3252657	1.49838239	-4.3213224	1.88E-05	0.0001374	0.98718056	4.726982911	up-regulated in High
IGSF9B	-0.3904714	0.6425089	-7.2605283	1.50E-12	3.71E-11	16.888899	11.82328421	up-regulated in High
JAM3	-0.2454774	2.16261498	-3.5771091	0.00038152	0.00203824	-1.8649863	3.418481278	up-regulated in High
NCAPD3	0.496875	2.18290625	9.64361257	2.77E-20	1.87E-18	34.476738	19.5573641	up-regulated in Low
VPS26B	-0.2167489	3.40500754	-5.2089699	2.79E-07	2.96E-06	5.03514443	6.554441727	up-regulated in High
THYN1	-0.2679755	4.10569653	-5.4440981	8.21E-08	9.61E-07	6.22115537	7.085871195	up-regulated in High
ACAD8	-0.7634789	2.98640266	-10.311843	1.02E-22	9.08E-21	40.0341262	21.99190773	up-regulated in High
GLB1L3	-1.0479353	1.07801068	-8.9012619	1.04E-17	5.12E-16	28.6055141	16.98151877	up-regulated in High
GLB1L2	-0.743515	2.50179283	-8.7197862	4.23E-17	1.91E-15	27.2215909	16.37365663	up-regulated in High
B3GAT1	-0.3043042	0.51635688	-5.1279614	4.21E-07	4.30E-06	4.63738593	6.375881846	up-regulated in High
IQSEC3	-0.1041128	0.30874284	-3.6708071	0.00026804	0.00149602	-1.5337862	3.571804422	up-regulated in High
SLC6A12	-0.1223904	0.62731175	-3.0015054	0.00282198	0.01177497	-3.7198815	2.549445423	up-regulated in High
CCDC77	0.41423645	1.76488188	9.86266248	4.53E-21	3.41E-19	36.2710829	20.34375844	up-regulated in Low
B4GALNT3	-0.738853	3.33581655	-7.2598025	1.51E-12	3.73E-11	16.8841304	11.82117792	up-regulated in High
NINJ2	-0.7255162	3.04607318	-8.4141561	4.26E-16	1.73E-14	24.9384792	15.37015044	up-regulated in High

WNK1	-0.2515803	4.21178768	-3.6643869	0.00027467	0.00152806	-1.556739	3.561193131	up-regulated in High
WNT5B	-0.1691663	1.53361129	-2.0987409	0.03634521	0.10112884	-5.9941586	1.439552847	up-regulated in High
FBXL14	0.15055945	2.61376362	2.8555726	0.00447617	0.01756259	-4.140501	2.349093597	up-regulated in Low
ADIPOR2	0.3468877	3.83473332	7.25374911	1.57E-12	3.87E-11	16.8443751	11.80361788	up-regulated in Low
CACNA2D4	-0.1223079	0.57172001	-5.0338886	6.74E-07	6.63E-06	4.18252288	6.171461924	up-regulated in High
DCP1B	-0.2200107	1.8670299	-5.9434698	5.27E-09	7.57E-08	8.89282735	8.278482364	up-regulated in High
CACNA1C	-0.2450892	0.57948151	-8.2580736	1.36E-15	5.10E-14	23.7960638	14.86766462	up-regulated in High
FKBP4	0.82932395	4.44292293	12.7126312	3.04E-32	5.74E-30	61.8277559	31.51723944	up-regulated in Low
ITFG2	-0.1251287	2.16142265	-3.2065674	0.00143007	0.00653804	-3.0947355	2.844643521	up-regulated in High
NRIP2	-0.2677603	1.04490185	-8.2552026	1.39E-15	5.20E-14	23.7752021	14.85848633	up-regulated in High
FOXM1	1.5552077	2.70501732	19.3740728	1.15E-62	2.03E-59	131.662396	61.94033099	up-regulated in Low
RHNO1	0.38089343	3.83012944	8.85948056	1.44E-17	6.93E-16	28.285054	16.84078918	up-regulated in Low
TULP3	0.11494869	3.23524405	2.48239512	0.01338033	0.04454541	-5.1235524	1.873533147	up-regulated in Low
TEAD4	0.62074109	2.6137597	10.9295294	4.65E-25	5.23E-23	45.3831968	24.33260602	up-regulated in Low
TSPAN9	-0.385696	3.20651264	-6.2954861	6.76E-10	1.12E-08	10.8979423	9.170297033	up-regulated in High
PRMT8	-0.1919455	0.28638333	-5.0039417	7.82E-07	7.60E-06	4.03931724	6.10705171	up-regulated in High
PARP11	-0.1428942	1.31677588	-3.5265832	0.00046007	0.00240721	-2.0402085	3.337177144	up-regulated in High
CCND2	-0.4292467	2.55490432	-5.5443786	4.80E-08	5.87E-07	6.74111424	7.318430861	up-regulated in High
C12orf5	0.2321912	2.38879196	5.47334676	7.03E-08	8.33E-07	6.37194301	7.153338366	up-regulated in Low
C12orf4	0.13236351	2.22234663	3.4218614	0.00067339	0.00335929	-2.3958241	3.171733853	up-regulated in Low
RAD51AP1	1.0944089	2.09201126	16.5822324	1.90E-49	9.71E-47	101.294835	48.72140774	up-regulated in Low
AKAP3	-0.1009809	0.40825133	-3.1606953	0.00167024	0.00746937	-3.2380264	2.777222182	up-regulated in High
NDUFA9	0.38545034	2.14745348	9.78387503	8.72E-21	6.23E-19	35.6225553	20.05957289	up-regulated in Low
KCNA5	-0.20107	0.29206182	-8.3031909	9.72E-16	3.73E-14	24.124633	15.0122099	up-regulated in High
VWF	-0.6977441	4.03012795	-6.9602193	1.08E-11	2.37E-10	14.9490449	10.9658266	up-regulated in High
CD9	-0.4013243	6.59273945	-5.1002279	4.84E-07	4.90E-06	4.5025001	6.315288522	up-regulated in High
PLEKHG6	0.17511521	1.923325	2.22756954	0.02635749	0.0779646	-5.7177451	1.579095929	up-regulated in Low
SCNN1A	-0.3107521	5.58324835	-2.7226403	0.00670454	0.02481982	-4.5059861	2.173630784	up-regulated in High
LTBR	0.36370783	4.61229751	6.1926647	1.24E-09	1.99E-08	10.3020328	8.905503497	up-regulated in Low
CD27	-0.5280707	2.71386517	-5.2340769	2.45E-07	2.63E-06	5.15955618	6.610256147	up-regulated in High
VAMP1	-0.1870173	1.7153466	-3.1183001	0.00192477	0.00845356	-3.368693	2.715621023	up-regulated in High
MRPL51	0.44452458	5.59033383	9.86854588	4.31E-21	3.25E-19	36.3196516	20.36503954	up-regulated in Low
NCAPD2	0.89352711	3.75043728	15.0114276	2.96E-42	1.01E-39	84.7844878	41.52893763	up-regulated in Low
GAPDH	0.86733822	9.38706269	13.2227716	2.11E-34	4.52E-32	66.7716658	33.67475854	up-regulated in Low
IFFO1	-0.2836527	1.61902033	-4.8809134	1.43E-06	1.32E-05	3.45911856	5.845821541	up-regulated in High
NOP2	0.57765889	3.39342235	11.4729405	3.48E-27	4.58E-25	50.2450958	26.45833959	up-regulated in Low
LPAR5	-0.1407125	1.45274216	-2.3147771	0.02103292	0.06475685	-5.521477	1.6771005	up-regulated in High
ING4	-0.1583867	3.42463053	-3.180799	0.00156073	0.00705532	-3.1754722	2.806671706	up-regulated in High
ZNF384	0.24178353	2.46332606	5.64512354	2.78E-08	3.52E-07	7.27191179	7.555596591	up-regulated in Low
MLF2	0.37638641	5.64479354	8.14046427	3.21E-15	1.15E-13	22.9459666	14.49358629	up-regulated in Low
PTMS	0.16548148	6.76165351	2.29090854	0.02238763	0.06811957	-5.5759287	1.649991894	up-regulated in Low
LAG3	0.19833199	1.70986518	2.25473706	0.02458557	0.07371631	-5.6573939	1.609319696	up-regulated in Low
CD4	-0.6625379	4.46142935	-7.8035419	3.60E-14	1.11E-12	20.5626567	13.44399606	up-regulated in High
GPR162	-0.3602952	1.15668661	-5.7636197	1.45E-08	1.93E-07	7.90697284	7.83904923	up-regulated in High
P3H3	-0.1764623	2.51808414	-2.0269124	0.04320625	0.11623125	-6.1412509	1.36445348	up-regulated in High
CDCA3	1.06467231	1.47176664	20.2006184	1.21E-66	4.41E-63	140.804189	65.91797136	up-regulated in Low
USP5	0.36500275	4.69921559	8.76913694	2.90E-17	1.34E-15	27.5958768	16.53808439	up-regulated in Low
TPI1	0.62861388	7.62760659	11.8947074	7.11E-29	1.06E-26	54.1125239	28.14824743	up-regulated in Low
LRRC23	-0.2142558	2.3812838	-3.2257935	0.00133925	0.0061658	-3.0340898	2.873138798	up-regulated in High
C12orf57	0.16681379	4.7504564	2.54433607	0.01125092	0.03847189	-4.9696395	1.948811912	up-regulated in Low
PTPN6	-0.1470771	3.96387279	-3.1578245	0.00168644	0.00752799	-3.2469282	2.77302923	up-regulated in High
PHB2	0.42761487	6.09614403	8.48078003	2.59E-16	1.07E-14	25.431007	15.58671079	up-regulated in Low
EMG1	0.28276475	2.1328497	6.79323035	3.15E-11	6.47E-10	13.8995087	10.50133621	up-regulated in Low
LPCAT3	-0.2044085	2.33797898	-4.1332	4.20E-05	0.00028571	0.21868186	4.376704076	up-regulated in High
C1S	-0.3162915	5.63093028	-3.3725823	0.00080294	0.0039205	-2.5596292	3.0953161	up-regulated in High
C1R	-0.3933665	5.55142104	-4.9239564	1.16E-06	1.09E-05	3.66061968	5.936596699	up-regulated in High
C1RL	-0.1749969	3.98359812	-3.5035286	0.00050073	0.00259023	-2.1193737	3.300398822	up-regulated in High
RBP5	-0.3803324	2.13433332	-6.0620791	2.66E-09	4.05E-08	9.5573477	8.574312195	up-regulated in High
PEX5	0.16026849	3.13385	3.7454575	0.00020122	0.00116241	-1.2641125	3.69632533	up-regulated in Low
CD163	-0.2662282	3.71909036	-2.3082373	0.02139678	0.06569353	-5.5364513	1.66965158	up-regulated in High
APOBEC1	0.17599048	0.48635978	2.13026088	0.03364199	0.09500167	-5.9280228	1.473118383	up-regulated in Low
FOXJ2	-0.1010004	2.64881405	-2.4023324	0.0166578	0.05338561	-5.3170102	1.778382287	up-regulated in High
C3AR1	-0.4292068	3.30065326	-4.6526019	4.21E-06	3.52E-05	2.4172736	5.375534363	up-regulated in High
NECAP1	0.14317226	3.51615989	3.70306199	0.00023695	0.00134264	-1.4178958	3.625350117	up-regulated in Low
CLEC4A	-0.4028473	2.23587348	-5.1436528	3.89E-07	4.00E-06	4.71399501	6.410286734	up-regulated in High
RIMKLB	0.25108695	2.13730753	2.67420503	0.00773808	0.02798798	-4.6349533	2.111366806	up-regulated in Low
KLRG1	-0.1827103	0.67694431	-5.7077546	1.97E-08	2.57E-07	7.60613181	7.70481128	up-regulated in High
A2M	-1.0686015	7.08454567	-11.068115	1.35E-25	1.59E-23	46.6096978	24.86900743	up-regulated in High
PZP	-0.1410218	0.27489207	-3.2058543	0.00143354	0.00655085	-3.0969781	2.843589322	up-regulated in High
KLRB1	-0.461099	1.97649154	-5.9288014	5.72E-09	8.18E-08	8.81143591	8.242228774	up-regulated in High
CLEC2D	-0.2524357	1.53707106	-4.4022008	1.31E-05	9.93E-05	1.32732809	4.881599421	up-regulated in High

CLECL1	-0.2732427	0.64862283	-7.2624435	1.48E-12	3.67E-11	16.9014836	11.82884274	up-regulated in High
CD69	-0.4740403	2.04246927	-5.3716988	1.20E-07	1.36E-06	5.85099681	6.920160241	up-regulated in High
CLEC12A	-0.2616757	1.09623292	-3.8067303	0.00015845	0.00093892	-1.0389335	3.800096973	up-regulated in High
CLEC9A	-0.1241621	0.22398835	-6.7877328	3.26E-11	6.69E-10	13.8653148	10.48619574	up-regulated in High
CLEC1A	-0.2533031	0.88344718	-7.0186804	7.40E-12	1.67E-10	15.3214302	11.13053136	up-regulated in High
CLEC7A	-0.3999509	2.56242453	-4.5985285	5.41E-06	4.42E-05	2.17721125	5.266927341	up-regulated in High
OLR1	-0.7483453	3.14020275	-6.6457075	7.97E-11	1.54E-09	12.9899335	10.09842508	up-regulated in High
TMEM52B	-0.1117614	0.46245397	-3.7148406	0.00022646	0.00129101	-1.3753369	3.645001082	up-regulated in High
GABARAPL1	0.14699126	4.03865221	2.01157571	0.04480553	0.11971326	-6.1720053	1.348668416	up-regulated in Low
KLRC2	0.17566651	0.26082718	4.83652504	1.77E-06	1.60E-05	3.25300274	5.752909215	up-regulated in Low
KLRC1	0.10846692	0.3540535	3.17459231	0.00159381	0.00717541	-3.1948256	2.797563219	up-regulated in Low
MAGOHB	0.44858846	2.10133177	11.5467574	1.77E-27	2.42E-25	50.9162088	26.7516492	up-regulated in Low
STYK1	0.41614443	1.78808391	5.67548036	2.36E-08	3.01E-07	7.43350162	7.627750496	up-regulated in Low
YBX3	0.15536109	4.2787789	2.87301131	0.00424024	0.01678018	-4.0913035	2.372609675	up-regulated in Low
BCL2L14	-0.1498043	0.67407355	-3.1811149	0.00155907	0.00704833	-3.1744865	2.807135553	up-regulated in High
LRP6	-0.1628632	2.35938869	-2.9533205	0.00329308	0.01346639	-3.861002	2.482397627	up-regulated in High
LOH12CR1	0.19230014	1.62915137	4.67743927	3.75E-06	3.17E-05	2.52840258	5.425777895	up-regulated in Low
CREBL2	-0.369742	4.19574023	-8.1559448	2.87E-15	1.03E-13	23.0573313	14.54259988	up-regulated in High
GPR19	0.30057858	0.51319468	9.03124675	3.78E-18	1.97E-16	29.609445	17.42229456	up-regulated in Low
GPRC5A	-0.6262348	5.80570901	-5.4766445	6.90E-08	8.20E-07	6.38898918	7.160964057	up-regulated in High
HEBP1	-0.1586214	3.5529525	-3.7297888	0.00021379	0.00122678	-1.3211411	3.670015321	up-regulated in High
EMP1	-0.4531029	3.97000867	-5.2221386	2.61E-07	2.78E-06	5.10033209	6.583688732	up-regulated in High
GUCY2C	0.12976874	0.60013354	2.21771912	0.02702676	0.07961311	-5.7394501	1.568206004	up-regulated in Low
H2AFJ	0.20648631	3.56632145	2.83645868	0.00474842	0.01848059	-4.1940915	2.323451198	up-regulated in Low
WBP11	0.36152205	4.12918793	8.55013751	1.54E-16	6.57E-15	25.9468195	15.81346246	up-regulated in Low
SMCO3	-0.2157088	0.37307678	-8.6739956	6.00E-17	2.68E-15	26.8756924	16.22167956	up-regulated in High
ART4	-0.209229	0.29337669	-6.8170565	2.71E-11	5.62E-10	14.0479683	10.56706597	up-regulated in High
MGP	-1.3437999	5.95983905	-13.745761	1.18E-36	2.89E-34	71.9355827	35.92735972	up-regulated in High
ERP27	-0.5737067	2.48585678	-5.7201812	1.84E-08	2.41E-07	7.67282906	7.734578178	up-regulated in High
ARHGDIB	-0.8340474	6.70722036	-11.663553	6.06E-28	8.67E-26	51.9831101	27.21788416	up-regulated in High
RERG	-0.5576957	1.62433916	-8.1325195	3.40E-15	1.21E-13	22.8888757	14.46845861	up-regulated in High
PTPRO	-0.2580132	0.8694618	-5.7105597	1.94E-08	2.53E-07	7.62117681	7.711526145	up-regulated in High
EPS8	-0.3933644	4.24860206	-4.8059949	2.04E-06	1.82E-05	3.11223162	5.6894179	up-regulated in High
STRAP	0.6391302	5.61722868	12.3669008	8.34E-31	1.44E-28	58.5334136	30.07904165	up-regulated in Low
DERA	0.35684007	4.01598222	6.26024215	8.33E-10	1.37E-08	10.6927397	9.079137173	up-regulated in Low
LMO3	-1.2446309	3.07392498	-10.063949	8.38E-22	6.74E-20	37.9437085	21.07650718	up-regulated in High
AEBP2	0.18797331	2.71389147	4.1634602	3.70E-05	0.00025436	0.3401481	4.432161967	up-regulated in Low
PDE3A	0.27449946	1.31641734	2.74258497	0.00631643	0.02363339	-4.452227	2.199528647	up-regulated in Low
SLCO1B1	0.11290178	0.11231162	3.75039279	0.00019741	0.00114302	-1.246103	3.70463151	up-regulated in Low
SLCO1A2	0.14764166	0.14908611	4.7906037	2.20E-06	1.95E-05	3.04157231	5.657537856	up-regulated in Low
PYROXD1	0.10261419	1.80701352	2.54831782	0.01112498	0.03810596	-4.9596191	1.953700775	up-regulated in Low
RECQL	0.33197232	2.81775443	5.22778185	2.53E-07	2.71E-06	5.12831229	6.596240881	up-regulated in Low
GOLT1B	0.45971663	4.10107304	7.27421146	1.37E-12	3.40E-11	16.9788683	11.86302193	up-regulated in Low
SPX	0.4872519	0.42746042	6.30263869	6.47E-10	1.08E-08	10.9397071	9.18884795	up-regulated in Low
LDHB	0.41632685	7.07527746	4.15837183	3.78E-05	0.00025941	0.31966526	4.422812705	up-regulated in Low
KCNJ8	-0.2632982	2.39319927	-3.0440391	0.00245831	0.01045706	-3.5934821	2.609362662	up-regulated in High
ABCC9	-0.260946	1.09275628	-4.5720464	6.11E-06	4.94E-05	2.06058233	5.214127406	up-regulated in High
CMAS	0.53327639	4.25911176	9.24990858	6.70E-19	3.83E-17	31.3216915	18.1737281	up-regulated in Low
ST8SIA1	-0.1160898	0.38446221	-4.0626997	5.64E-05	0.00037302	-0.0611001	4.2488191	up-regulated in High
C2CD5	0.11717405	2.71212817	2.39198215	0.01712938	0.0546103	-5.3415677	1.766258427	up-regulated in Low
BCAT1	-0.3749981	2.64296002	-3.1761159	0.00158563	0.00714519	-3.1900783	2.799797729	up-regulated in High
LRMP	-0.3523436	1.27006447	-5.2849394	1.89E-07	2.07E-06	5.41323323	6.724011314	up-regulated in High
CASC1	-0.3260117	0.67981234	-5.6175326	3.23E-08	4.06E-07	7.12570601	7.490293813	up-regulated in High
KRAS	0.22566307	3.40232315	3.65971985	0.00027958	0.00155216	-1.5734003	3.553489217	up-regulated in Low
RASSF8	-0.1617715	1.85871059	-2.1807998	0.02966775	0.08589759	-5.8199613	1.527715414	up-regulated in High
BHLHE41	-0.4444822	2.45635107	-5.1094848	4.62E-07	4.70E-06	4.54744937	6.335482969	up-regulated in High
SSPN	-0.3598849	1.65478548	-5.4705295	7.13E-08	8.44E-07	6.35738789	7.146826853	up-regulated in High
ITPR2	-0.3979822	2.04991494	-6.2926754	6.87E-10	1.14E-08	10.8815415	9.16301192	up-regulated in High
ASUN	0.52614172	3.05213698	10.5979676	8.59E-24	8.49E-22	42.487577	23.06579379	up-regulated in Low
STK38L	0.23348295	2.76474998	4.11050246	4.62E-05	0.00031194	0.12811532	4.335329655	up-regulated in Low
ARNTL2	0.78698903	2.13475784	7.92532182	1.52E-14	4.92E-13	21.4151066	13.81955669	up-regulated in Low
SMCO2	0.12258192	0.23157889	5.80535977	1.15E-08	1.56E-07	8.13341996	7.940046992	up-regulated in Low
MRPS35	0.64755425	5.11085384	11.9612303	3.82E-29	5.82E-27	54.7296545	28.41783321	up-regulated in Low
KLHL42	0.25033109	2.15636243	5.36036139	1.28E-07	1.44E-06	5.7934307	6.894377469	up-regulated in Low
PTHLH	0.49158867	1.13249975	4.91881734	1.19E-06	1.11E-05	3.63647746	5.925723669	up-regulated in Low
CCDC91	0.11750121	2.12036481	3.01785035	0.00267677	0.01124851	-3.6715112	2.572389304	up-regulated in Low
ERGIC2	0.34398105	3.45162959	7.25971737	1.51E-12	3.73E-11	16.8835711	11.82093091	up-regulated in Low
IPO8	0.28063338	3.21307424	6.42126988	3.17E-10	5.58E-09	11.6382787	9.49900024	up-regulated in Low
TSPAN11	-0.3907595	1.76640923	-3.1827388	0.00155053	0.00701787	-3.1694165	2.809521303	up-regulated in High
DDX11	0.53562244	1.96821043	8.79222144	2.43E-17	1.14E-15	27.7714846	16.61522303	up-regulated in Low
FAM60A	0.54901785	3.69327162	8.00752955	8.40E-15	2.82E-13	21.996334	14.07552898	up-regulated in Low

DENND5B	0.16409533	1.14110746	3.16050322	0.00167132	0.00747191	-3.2386223	2.776941497	up-regulated in Low
AMN1	0.19653551	1.32735142	4.97361074	9.08E-07	8.72E-06	3.8950627	6.042143495	up-regulated in Low
BICD1	0.12137928	1.04767062	2.97341164	0.00308854	0.01273522	-3.8024284	2.510246602	up-regulated in Low
FGD4	-0.1994858	1.72793082	-4.0163218	6.83E-05	0.00044327	-0.2426959	4.165700939	up-regulated in High
DNM1L	0.41183789	3.26969847	8.92838615	8.45E-18	4.19E-16	28.8141398	17.07312807	up-regulated in Low
YARS2	0.52122802	3.14826819	11.9442639	4.48E-29	6.77E-27	54.5720769	28.34899928	up-regulated in Low
PKP2	0.71523573	1.47843585	7.245886	1.66E-12	4.07E-11	16.7927745	11.78082497	up-regulated in Low
ALG10	0.21814005	0.97065726	6.51684488	1.77E-10	3.24E-09	12.2090929	9.752247522	up-regulated in Low
KIF21A	0.37932358	2.00252895	6.52487644	1.68E-10	3.10E-09	12.2573846	9.77366535	up-regulated in Low
SLC2A13	-0.1090884	1.33948541	-2.0899923	0.03712765	0.10289895	-6.0123434	1.430302532	up-regulated in High
LRRK2	-1.1136562	2.3228404	-8.8768685	1.26E-17	6.13E-16	28.4182857	16.89929958	up-regulated in High
CNTN1	0.20256925	0.85158226	2.35393107	0.01896553	0.0594641	-5.4309586	1.722034975	up-regulated in Low
GXYLT1	0.11543966	2.10192718	2.66799238	0.00788053	0.02841981	-4.6513328	2.103444819	up-regulated in Low
PPHLN1	0.20668854	2.35412116	7.18307188	2.52E-12	6.03E-11	16.3821921	11.59943255	up-regulated in Low
ZCRB1	0.19010101	4.22663118	4.67893872	3.73E-06	3.14E-05	2.53512886	5.42881833	up-regulated in Low
PRICKLE1	-0.2732122	0.83955273	-6.4375258	2.87E-10	5.09E-09	11.734863	9.541862072	up-regulated in High
PUS7L	0.16956224	1.42807361	5.37534605	1.18E-07	1.34E-06	5.86953884	6.928464173	up-regulated in Low
TWF1	0.51819789	4.13210888	9.29591672	4.64E-19	2.69E-17	31.6856503	18.33340384	up-regulated in Low
TMEM117	0.21570013	1.17233044	4.78174479	2.29E-06	2.02E-05	3.00099571	5.639227171	up-regulated in Low
NELL2	-0.2047482	0.95850626	-3.1843618	0.00154203	0.00698593	-3.1643468	2.811906708	up-regulated in High
SLC38A1	0.70933682	3.38154169	8.11732686	3.80E-15	1.34E-13	22.779821	14.42045789	up-regulated in Low
SLC38A2	0.26813271	4.84821009	4.15005798	3.91E-05	0.00026761	0.28624878	4.407557711	up-regulated in Low
AMIGO2	-0.3137159	3.82884782	-2.6269371	0.00888256	0.03148531	-4.7586433	2.05146186	up-regulated in High
RPAP3	0.29866682	2.74579599	8.35333237	6.71E-16	2.64E-14	24.4913756	15.17352382	up-regulated in Low
RAPGEF3	-0.2974943	1.090088	-6.1394874	1.70E-09	2.67E-08	9.99713612	8.769943762	up-regulated in High
HDAC7	-0.1828829	3.47168107	-3.5969815	0.00035422	0.00190858	-1.7954209	3.450723048	up-regulated in High
VDR	-0.5404786	3.31617323	-7.1641482	2.85E-12	6.79E-11	16.2590667	11.54502575	up-regulated in High
TMEM106C	0.63458552	4.46317464	9.4842745	1.02E-19	6.45E-18	33.188856	18.99271231	up-regulated in Low
COL2A1	0.33328429	0.30471814	3.60418621	0.00034479	0.0018652	-1.7701098	3.46244901	up-regulated in Low
SENP1	0.38805658	2.16317045	10.8526544	9.19E-25	1.00E-22	44.7069156	24.03679383	up-regulated in Low
PFKM	0.29121387	2.76577827	5.51921944	5.50E-08	6.66E-07	6.60987418	7.259754442	up-regulated in Low
CCDC184	-0.161244	0.691194	-3.2578769	0.00119949	0.00558714	-2.9321139	2.921002309	up-regulated in High
ZNF641	-0.1884039	2.02732398	-4.9366732	1.09E-06	1.03E-05	3.72045902	5.963543413	up-regulated in High
KANSL2	0.24093795	3.24779263	6.05385304	2.79E-09	4.22E-08	9.51089471	8.553641523	up-regulated in Low
CCNT1	0.21409851	2.6150002	5.36576396	1.24E-07	1.40E-06	5.82084891	6.906657971	up-regulated in Low
ADCY6	-0.2142814	2.78158375	-3.701145	0.0002387	0.00135163	-1.4248102	3.622156861	up-regulated in High
CACNB3	-0.3295297	2.57259178	-4.9902546	8.36E-07	8.09E-06	3.97412308	6.077720494	up-regulated in High
DDX23	0.33610042	4.72821821	9.52549453	7.27E-20	4.68E-18	33.5206075	19.13818201	up-regulated in Low
CCDC65	-0.231648	1.1856512	-3.9306179	9.68E-05	0.00060584	-0.5731304	4.014215611	up-regulated in High
ARF3	0.12489381	5.61231804	2.94797052	0.0033496	0.01367547	-3.8765349	2.475007615	up-regulated in Low
DDN	0.11271916	0.22722951	4.52252485	7.65E-06	6.08E-05	1.84414802	5.116079904	up-regulated in Low
PRKAG1	0.20062116	3.68047658	5.50834083	5.83E-08	7.02E-07	6.55329038	7.234451595	up-regulated in Low
RHEBL1	0.26960506	1.30707956	5.16259378	3.53E-07	3.67E-06	4.80674898	6.451933265	up-regulated in Low
TUBA1B	0.74183604	5.99565407	12.6674693	4.70E-32	8.82E-30	61.3947708	31.32823928	up-regulated in Low
TUBA1A	-0.3376898	5.25061315	-3.9569061	8.70E-05	0.00054992	-0.4724867	4.0603892	up-regulated in High
TUBA1C	0.79231198	4.7748372	14.2582152	6.74E-39	1.87E-36	77.0815529	38.17131061	up-regulated in Low
TROAP	1.3831463	1.93612686	19.2668555	3.75E-62	6.08E-59	130.480069	61.42584624	up-regulated in Low
C1QL4	0.1137255	0.09892873	4.9917797	8.30E-07	8.03E-06	3.98137945	6.080985442	up-regulated in Low
DNAJC22	0.27011396	1.33551712	3.07846495	0.00219603	0.0094795	-3.4899228	2.658361487	up-regulated in Low
SPATS2	0.48398988	2.56401343	10.8230935	1.19E-24	1.27E-22	44.4476448	23.92337739	up-regulated in Low
MCRS1	0.35552087	4.22309432	10.2975925	1.15E-22	1.02E-20	39.9130631	21.93890465	up-regulated in Low
PRPF40B	0.1773923	1.07591267	4.88671871	1.39E-06	1.28E-05	3.48620184	5.858025648	up-regulated in Low
FMNL3	-0.23849	2.05744627	-4.9580619	9.79E-07	9.36E-06	3.82141976	6.008997055	up-regulated in High
NCKAP5L	-0.2005608	2.29899519	-4.2336699	2.74E-05	0.00019359	0.62515295	4.562144609	up-regulated in High
FAIM2	-0.2396262	0.64048037	-3.1864252	0.0015313	0.0069416	-3.1578978	2.814940844	up-regulated in High
AQP5	-1.5467933	3.22597354	-6.7346422	4.57E-11	9.15E-10	13.5362844	10.34048222	up-regulated in High
RACGAP1	1.1838188	2.99430093	19.5132898	2.46E-63	4.63E-60	133.198927	62.608929	up-regulated in Low
ASIC1	0.14816589	0.687682	3.22330555	0.00135069	0.00621408	-3.0419572	2.869443499	up-regulated in Low
SMARCD1	0.30250271	3.70257142	6.89955528	1.60E-11	3.42E-10	14.5653354	10.79605905	up-regulated in Low
GPD1	-0.4425336	0.90296789	-6.0450144	2.94E-09	4.43E-08	9.46104288	8.531456932	up-regulated in High
LIMA1	-0.1410903	3.92190836	-2.3420165	0.01957492	0.06099805	-5.4586606	1.708300125	up-regulated in High
LARP4	0.39119492	3.11873513	9.38739164	2.23E-19	1.35E-17	32.4130463	18.65247585	up-regulated in Low
DIP2B	0.33597633	2.64427548	7.11312281	3.99E-12	9.33E-11	15.9283888	11.39888014	up-regulated in Low
ATF1	0.28828974	3.32069044	6.25537126	8.58E-10	1.40E-08	10.664457	9.066570952	up-regulated in Low
METTL7A	-0.911417	4.01466094	-11.090603	1.11E-25	1.31E-23	46.8096016	24.95642411	up-regulated in High
SLC11A2	-0.1738018	3.29835239	-3.349099	0.00087252	0.00422071	-2.6368901	3.059223574	up-regulated in High
TFCP2	0.20889184	3.56903206	5.61955803	3.20E-08	4.02E-07	7.13641724	7.495078576	up-regulated in Low
POU6F1	-0.2298231	1.32182433	-6.3725123	4.26E-10	7.33E-09	11.3498269	9.370963945	up-regulated in High
SMAGP	0.24430017	2.62257915	3.49925171	0.00050863	0.00262507	-2.1340054	3.293598114	up-regulated in Low
BIN2	-0.2984504	2.09310273	-4.2951688	2.10E-05	0.0001523	0.87843847	4.677501323	up-regulated in High
ACVRL1	-0.3911527	2.76804493	-6.5084384	1.86E-10	3.40E-09	12.1586008	9.729852573	up-regulated in High

ACVR1B	-0.23852	3.66728373	-3.5962115	0.00035525	0.00191327	-1.7981233	3.449470914	up-regulated in High
GRASP	-0.4327761	1.90523834	-6.8043286	2.94E-11	6.06E-10	13.9686081	10.53193075	up-regulated in High
NR4A1	-0.3107131	3.4702894	-2.3579073	0.0187659	0.05892362	-5.4216831	1.726630591	up-regulated in High
ATG101	0.35716286	3.32890593	8.41320013	4.29E-16	1.74E-14	24.9314333	15.36705207	up-regulated in Low
KRT80	0.41944149	3.6109313	3.89508384	0.00011163	0.00068775	-0.7081699	3.95221295	up-regulated in Low
KRT86	0.27291632	1.19938153	3.62104885	0.00032362	0.00176457	-1.710681	3.489970242	up-regulated in Low
KRT81	0.66573943	1.19891075	4.18803255	3.33E-05	0.00023127	0.43939066	4.47744595	up-regulated in Low
KRT83	0.34645303	0.30743361	6.6305247	8.76E-11	1.68E-09	12.8972678	10.05735723	up-regulated in Low
KRT6B	0.4638078	0.65010725	4.23836772	2.69E-05	0.00019013	0.64438149	4.570907237	up-regulated in Low
KRT6C	0.1444337	0.18833081	3.26543493	0.0011686	0.00546154	-2.9079501	2.932334571	up-regulated in Low
KRT6A	1.1258995	1.99746888	5.21765738	2.67E-07	2.84E-06	5.07813251	6.573729217	up-regulated in Low
KRT1	-0.1294452	0.17270163	-5.2048671	2.85E-07	3.01E-06	5.01486466	6.545342089	up-regulated in High
KRT78	0.14563769	0.1742361	4.19823327	3.19E-05	0.00022245	0.48074906	4.496310529	up-regulated in Low
KRT8	0.3705609	7.64176173	4.48462151	9.09E-06	7.09E-05	1.67995758	5.041642386	up-regulated in Low
KRT18	0.44614011	7.75446028	5.84017196	9.45E-09	1.30E-07	8.32337236	8.024738769	up-regulated in Low
EIF4B	0.10762299	5.67452848	2.24380403	0.0252858	0.07542849	-5.6817672	1.597123329	up-regulated in Low
TNS2	-0.7471833	3.27195082	-11.277293	2.06E-26	2.54E-24	48.4785064	25.6861226	up-regulated in High
SPRYD3	-0.1603378	4.19879574	-3.6725392	0.00026628	0.00148804	-1.5275872	3.574669872	up-regulated in High
IGFBP6	-0.3488563	2.92037777	-2.7975474	0.0053496	0.02049658	-4.3021117	2.271678394	up-regulated in High
CSAD	-0.255828	2.04595112	-3.354672	0.00085552	0.0041474	-2.6186016	3.067769981	up-regulated in High
ITGB7	-0.1433149	1.28792988	-2.5464462	0.01118402	0.03827451	-4.9643311	1.951402037	up-regulated in High
MFSD5	0.11875347	3.95881103	3.5157477	0.00047878	0.0024937	-2.0774772	3.319866546	up-regulated in Low
ESPL1	1.06561583	1.30916276	19.9974069	1.15E-65	3.06E-62	138.552644	64.93837113	up-regulated in Low
C12orf10	0.29030069	3.84300167	7.60335649	1.46E-13	4.12E-12	19.1838672	12.83616691	up-regulated in Low
AAAS	0.26570576	3.3513814	7.17159516	2.71E-12	6.48E-11	16.3074881	11.56642286	up-regulated in Low
SP1	0.14622041	3.87993717	3.63462984	0.00030746	0.00168639	-1.6626264	3.512213478	up-regulated in Low
PRR13	0.19269631	5.05765182	4.49670379	8.60E-06	6.75E-05	1.7321576	5.065313289	up-regulated in Low
PCBP2	0.33330639	5.25516085	8.95438179	6.90E-18	3.47E-16	29.014515	17.16110843	up-regulated in Low
TARBP2	0.36651863	3.04663611	8.38048539	5.48E-16	2.19E-14	24.690671	15.26117434	up-regulated in Low
P11-793H13..	-0.1769985	0.64725816	-4.807668	2.03E-06	1.81E-05	3.1199251	5.692888598	up-regulated in High
ATP5G2	0.25704989	5.79080699	5.30888406	1.67E-07	1.84E-06	5.53341614	6.777880967	up-regulated in Low
CALCOCO1	-0.4693577	3.19096145	-9.0164853	4.25E-18	2.20E-16	29.4949088	17.37201486	up-regulated in High
HOXC13	0.20497944	0.67477945	2.47922106	0.01349849	0.04486971	-5.1313398	1.869714746	up-regulated in Low
HOXC12	0.16033665	0.30579085	2.09813869	0.03639861	0.10126779	-5.9954128	1.438915173	up-regulated in Low
HOXC11	0.25538052	0.61222045	3.14221127	0.00177713	0.0078864	-3.2952047	2.750280582	up-regulated in Low
HOXC10	0.45287905	1.11160437	3.2551653	0.00121076	0.00563198	-2.94077	2.916941946	up-regulated in Low
HOXC6	0.31208899	0.71401208	4.75522848	2.60E-06	2.27E-05	2.87995202	5.584590032	up-regulated in Low
HOXC9	0.3372675	0.76917926	4.32631576	1.83E-05	0.00013482	1.00801185	4.736458963	up-regulated in Low
HOXC8	0.28152363	0.52056239	5.11549889	4.48E-07	4.57E-06	4.57669136	6.348619303	up-regulated in Low
HOXC4	0.14135691	0.79260887	2.26972082	0.02365331	0.07136451	-5.6238016	1.626108023	up-regulated in Low
SMUG1	0.34012149	2.94008894	7.63262079	1.19E-13	3.42E-12	19.3836657	12.92427718	up-regulated in Low
CBX5	0.38044567	3.13502962	7.01772031	7.45E-12	1.68E-10	15.3152939	11.1278177	up-regulated in Low
HNRNPA1	0.23586846	6.63601882	6.21418807	1.09E-09	1.77E-08	10.4260792	8.960640163	up-regulated in Low
NFE2	-0.2964894	1.41478813	-3.8218844	0.00014929	0.00089189	-0.9827099	3.825979504	up-regulated in High
COPZ1	0.34888149	5.61393215	9.63356144	3.01E-20	2.02E-18	34.3950626	19.52156044	up-regulated in Low
ITGA5	0.49530534	3.63435123	5.7022102	2.03E-08	2.64E-07	7.5764147	7.691547459	up-regulated in Low
GTSF1	0.18604432	0.60131102	2.02791114	0.0431038	0.11599842	-6.1392402	1.365484431	up-regulated in Low
NCKAP1L	-0.4810255	2.3311187	-6.3877483	3.88E-10	6.73E-09	11.4397635	9.410889061	up-regulated in High
PDE1B	-0.2182061	0.63743424	-5.8059767	1.14E-08	1.55E-07	8.13677757	7.941544232	up-regulated in High
MUCL1	0.2406071	0.38218189	3.0296615	0.00257613	0.01088763	-3.6364004	2.58903209	up-regulated in Low
TESPA1	-0.3409586	0.83858182	-8.014311	8.00E-15	2.70E-13	22.0444871	14.09673216	up-regulated in High
METTL7B	-0.4333928	3.07712324	-3.4088373	0.00070559	0.00350138	-2.4393372	3.151447787	up-regulated in High
ITGA7	-0.2468851	1.38787311	-4.7250784	3.00E-06	2.58E-05	2.74306711	5.52277546	up-regulated in High
BLOC1S1	0.15447042	4.17557569	2.87062366	0.00427186	0.01688815	-4.0980566	2.3693831	up-regulated in Low
CD63	-0.2327088	8.41908491	-4.3621256	1.57E-05	0.00011687	1.15805525	4.804685526	up-regulated in High
GDF11	-0.1091844	1.35006208	-2.2385772	0.02562663	0.07622712	-5.6933785	1.591308449	up-regulated in High
ORMDL2	0.24503321	3.90324267	5.38765212	1.11E-07	1.26E-06	5.93218357	6.956516769	up-regulated in Low
DNAJC14	0.33091065	2.74628289	8.51840639	1.95E-16	8.23E-15	25.7104466	15.70955858	up-regulated in Low
MMP19	-0.4383977	2.06071824	-5.6492928	2.72E-08	3.45E-07	7.29405986	7.565487499	up-regulated in High
WIBG	0.24897288	3.91544895	6.56119258	1.35E-10	2.52E-09	12.4763684	9.870772995	up-regulated in Low
DGKA	-0.1225937	1.98868239	-2.0882746	0.03728295	0.10327053	-6.015905	1.428489734	up-regulated in High
PMEL	0.11849931	0.98343389	2.64151198	0.00851446	0.0303556	-4.7207326	2.069842933	up-regulated in Low
CDK2	0.66595876	3.24870523	14.2095342	1.10E-38	3.01E-36	76.5892483	37.95666885	up-regulated in Low
SUOX	-0.202955	2.96026079	-4.6308053	4.66E-06	3.86E-05	2.32019653	5.331627265	up-regulated in High
IKZF4	-0.1589778	1.7393873	-3.587729	0.0003667	0.00196804	-1.8278558	3.4356929	up-regulated in High
RPS26	0.43062619	5.97425774	5.32078104	1.57E-07	1.74E-06	5.59330964	6.804721565	up-regulated in Low
ERBB3	-0.3240205	4.83285339	-3.8569783	0.00012994	0.00078768	-0.8516997	3.886248754	up-regulated in High
PA2G4	0.61367708	5.11140393	16.8384997	1.22E-50	6.57E-48	104.036063	49.91512695	up-regulated in Low
RPL41	0.20776908	7.53217683	3.74312967	0.00020304	0.00117203	-1.2725993	3.692410733	up-regulated in Low
MYL6B	0.57047162	3.4296447	10.8793094	7.26E-25	7.98E-23	44.9410719	24.13921989	up-regulated in Low
MYL6	0.12239013	7.37497501	2.40915411	0.01635328	0.05257814	-5.3007681	1.786395066	up-regulated in Low

SMARCC2	0.17470331	3.51129892	3.48404681	0.00053768	0.00275574	-2.1858853	3.269476683	up-regulated in Low
NABP2	0.58730231	4.08199344	14.9145033	8.07E-42	2.69E-39	83.7846174	41.09318533	up-regulated in Low
SLC39A5	0.21339903	0.33548111	3.07074487	0.00225251	0.00969742	-3.5132438	2.647334248	up-regulated in Low
ANKRD52	0.32909449	2.99055449	5.91884646	6.06E-09	8.63E-08	8.75629799	8.217666552	up-regulated in Low
CS	0.35247987	4.71735462	8.17634858	2.47E-15	9.00E-14	23.2043594	14.60730556	up-regulated in Low
CNPY2	0.31425944	3.6051502	6.3753142	4.19E-10	7.22E-09	11.3663525	9.378300399	up-regulated in Low
IL23A	0.25885506	1.49303905	3.44805453	0.00061274	0.00309319	-2.307835	3.212725893	up-regulated in Low
APOF	0.10897374	0.08640861	3.7874799	0.00017087	0.00100437	-1.1100506	3.767342628	up-regulated in Low
TIMELESS	0.9523996	3.0785176	16.4794324	5.70E-49	2.80E-46	100.198561	48.24398214	up-regulated in Low
SPRYD4	0.11374799	1.48842612	3.46675445	0.0005726	0.00291121	-2.2446261	3.242149858	up-regulated in Low
RBMS2	-0.3441293	3.05249951	-6.6998385	5.68E-11	1.12E-09	13.3217541	10.24545134	up-regulated in High
BAZ2A	0.20384177	3.78732123	3.70978733	0.00023091	0.00131314	-1.3936112	3.636564044	up-regulated in Low
ATP5B	0.57824165	7.65836703	12.7487082	2.15E-32	4.12E-30	62.1742018	31.66845929	up-regulated in Low
PTGES3	0.4982233	6.46718308	11.8862015	7.69E-29	1.15E-26	54.0337526	28.11383576	up-regulated in Low
NACA	0.22450235	5.76682196	5.62998551	3.02E-08	3.81E-07	7.19161643	7.519734853	up-regulated in Low
PRIM1	0.82043016	2.1360313	14.8823872	1.12E-41	3.67E-39	83.4538492	40.94902864	up-regulated in Low
HSD17B6	-1.2808303	2.67415084	-10.489274	2.21E-23	2.12E-21	41.5505143	22.65569653	up-regulated in High
ZBTB39	0.17947037	1.55627063	4.26687404	2.38E-05	0.00017026	0.76148406	4.624253561	up-regulated in Low
TAC3	0.15349167	0.17981763	2.36101409	0.0186112	0.05852601	-5.4144251	1.730225523	up-regulated in Low
TMEM194A	0.58733608	2.57405602	10.8494283	9.45E-25	1.03E-22	44.6785993	24.02440726	up-regulated in Low
NAB2	-0.2582173	3.8695604	-3.8810291	0.00011808	0.00072276	-0.7612636	3.927819601	up-regulated in High
STAT6	-0.2827125	4.93084446	-5.9170376	6.12E-09	8.71E-08	8.74628784	8.21320712	up-regulated in High
LRP1	-0.4345425	4.11684068	-5.229334	2.51E-07	2.69E-06	5.13601289	6.599695284	up-regulated in High
NXPH4	0.45227155	1.14151191	3.91695211	0.00010226	0.00063691	-0.6252008	3.990314486	up-regulated in Low
SHMT2	0.7327139	4.48419594	12.5620174	1.29E-31	2.34E-29	60.3868356	30.88824016	up-regulated in Low
STAC3	-0.1385834	1.62577141	-2.7353202	0.00645538	0.02404839	-4.4718526	2.190077987	up-regulated in High
R3HDM2	0.1046816	3.0313312	2.36827481	0.01825403	0.05759078	-5.3974264	1.738641225	up-regulated in Low
INHBE	0.27725135	0.26081684	6.32832039	5.55E-10	9.38E-09	11.0899978	9.255595731	up-regulated in Low
GLI1	-0.1930767	0.56219266	-4.6665869	3.95E-06	3.31E-05	2.47977977	5.403797082	up-regulated in High
ARHGAP9	-0.3439251	1.94276573	-4.9579425	9.80E-07	9.36E-06	3.82085524	6.008742939	up-regulated in High
MARS	0.55485949	4.1227159	11.699174	4.36E-28	6.28E-26	52.3097163	27.36059793	up-regulated in Low
DDIT3	0.48516412	4.01581471	6.24015533	9.39E-10	1.53E-08	10.576227	9.027366849	up-regulated in Low
DCTN2	0.20820837	4.86786303	5.14393223	3.88E-07	4.00E-06	4.71536102	6.410900146	up-regulated in Low
PIP4K2C	0.26523884	4.63841571	4.7290341	2.95E-06	2.54E-05	2.76098119	5.530866762	up-regulated in Low
DTX3	-0.2414652	2.82783374	-3.3508681	0.00086709	0.00419722	-2.6310878	3.061935205	up-regulated in High
ARHGEF25	-0.1568328	1.85185263	-2.5820006	0.01010887	0.03513252	-4.8742429	1.995297537	up-regulated in High
B4GALNT1	0.41188793	0.62424225	6.38091018	4.05E-10	7.00E-09	11.3993762	9.392960633	up-regulated in Low
AGAP2	-0.1242201	0.97040459	-2.9150983	0.00371672	0.01498262	-3.9713763	2.429839818	up-regulated in High
TSPAN31	-0.2400115	3.30075782	-3.8114821	0.00015552	0.00092454	-1.0213263	3.808203626	up-regulated in High
CDK4	0.471786	4.65000324	7.42375711	5.00E-13	1.31E-11	17.9710333	12.30107608	up-regulated in Low
9-Mar	-0.3423078	3.09816154	-5.4531414	7.82E-08	9.19E-07	6.26770019	7.106699052	up-regulated in High
METTL1	0.35317123	3.30694795	6.03509217	3.11E-09	4.67E-08	9.40515431	8.506584067	up-regulated in Low
TSFM	0.34845088	3.37038889	6.77310694	3.58E-11	7.30E-10	13.7744564	10.44596295	up-regulated in Low
AVIL	-0.1577638	1.17019888	-2.3683787	0.01824896	0.05757915	-5.3971827	1.738761831	up-regulated in High
CTDSP2	-0.2503442	5.61049157	-4.6942229	3.47E-06	2.94E-05	2.60380372	5.459856763	up-regulated in High
XRCC6BP1	0.17089737	2.41437954	3.36088663	0.00083692	0.00406365	-2.5981729	3.077314348	up-regulated in Low
LRIG3	-0.7336864	3.19909847	-7.1438906	3.26E-12	7.70E-11	16.127554	11.48690714	up-regulated in High
SLC16A7	-0.4992721	1.2733744	-7.5044711	2.88E-13	7.81E-12	18.5132415	12.54034444	up-regulated in High
USP15	0.13897863	1.85147152	4.58830686	5.67E-06	4.62E-05	2.13212125	5.246517126	up-regulated in Low
PPM1H	0.28987097	2.15706354	3.40897734	0.00070524	0.00350022	-2.4388701	3.151665616	up-regulated in Low
AVPR1A	0.22480846	0.49700909	3.8654402	0.00012565	0.00076418	-0.8199415	3.900850108	up-regulated in Low
DPY19L2	0.11891424	0.31504304	3.79646917	0.00016496	0.00097343	-1.0768837	3.782620483	up-regulated in Low
TMEM5	0.27112469	1.75906792	7.94729969	1.29E-14	4.25E-13	21.570041	13.88779723	up-regulated in Low
SRGAP1	0.31201944	0.71899274	9.42661619	1.62E-19	1.00E-17	32.7264777	18.78994209	up-regulated in Low
C12orf66	0.16757289	1.53080735	4.96155444	9.63E-07	9.21E-06	3.83794309	6.016434765	up-regulated in Low
C12orf56	0.36109273	0.38492181	7.00687637	7.99E-12	1.79E-10	15.246035	11.09718843	up-regulated in Low
XPOT	0.72755451	3.89505377	13.9276015	1.91E-37	4.86E-35	73.7522247	36.71961229	up-regulated in Low
TBK1	0.220944	2.9788116	6.13788666	1.71E-09	2.69E-08	9.98799286	8.765877745	up-regulated in Low
WIF1	-1.1765799	2.02831183	-6.639483	8.29E-11	1.59E-09	12.9519217	10.08157939	up-regulated in High
LEMD3	0.1343472	2.807871	3.04895606	0.00241917	0.01031234	-3.5787596	2.616333522	up-regulated in Low
MSRB3	-0.5609329	2.162717	-8.1747282	2.50E-15	9.10E-14	23.1926725	14.60216242	up-regulated in High
HMGA2	0.39319456	0.58036203	4.28419497	2.20E-05	0.00015922	0.83299377	4.656814607	up-regulated in Low
LLPH	0.30517903	2.02085002	8.78660695	2.53E-17	1.18E-15	27.7287432	16.59644859	up-regulated in Low
TMBIM4	-0.2561074	3.83742347	-4.6202276	4.89E-06	4.04E-05	2.27323689	5.310382165	up-regulated in High
IRAK3	-0.3139179	1.77454924	-3.9337738	9.56E-05	0.00059869	-0.5610813	4.019745131	up-regulated in High
CAND1	0.32988245	3.38772055	6.41460534	3.30E-10	5.79E-09	11.5987411	9.481453039	up-regulated in Low
IFNG	0.29891495	0.65118791	4.78384044	2.27E-06	2.00E-05	3.01058828	5.643556154	up-regulated in Low
NUP107	0.54858952	2.66002702	10.7034597	3.42E-24	3.51E-22	43.4028479	23.4662866	up-regulated in Low
SLC35E3	0.13391353	1.0317077	3.55402429	0.00041571	0.00220092	-1.9453377	3.381214572	up-regulated in Low
CPM	-0.7894566	4.32165988	-6.5265243	1.67E-10	3.07E-09	12.2672988	9.77806226	up-regulated in High
CPSF6	0.20983164	3.62317488	5.68802716	2.20E-08	2.83E-07	7.50051065	7.657665596	up-regulated in Low

LYZ	-0.725755	7.35726	-4.7046476	3.30E-06	2.81E-05	2.65076133	5.481075472	up-regulated in High
YEATS4	0.47254103	3.35595072	6.86906369	1.94E-11	4.11E-10	14.3735173	10.71117028	up-regulated in Low
FRS2	-0.1167098	2.35750246	-2.1292239	0.0337281	0.0951942	-5.930214	1.472008179	up-regulated in High
CCT2	0.60169567	5.06888923	9.11107099	2.02E-18	1.09E-16	30.2311344	17.69517461	up-regulated in Low
RAB3IP	0.1523689	1.83675008	2.27117001	0.0235648	0.07113324	-5.6205411	1.627736237	up-regulated in Low
CNOT2	0.12027514	2.54217743	3.07056326	0.00225385	0.00970182	-3.5137917	2.647075103	up-regulated in Low
KCNMB4	0.41907522	1.70780305	3.96825197	8.31E-05	0.00052802	-0.4288545	4.080397425	up-regulated in Low
PTPRB	-0.3476551	1.71668575	-5.3521222	1.33E-07	1.50E-06	5.75166373	6.875668801	up-regulated in High
ATXN7L3B	0.22505085	4.11446734	5.13906388	3.98E-07	4.08E-06	4.69156901	6.40021599	up-regulated in Low
GLIPR1L2	-0.1059112	0.24061099	-6.3100512	6.19E-10	1.04E-08	10.9830322	9.208090945	up-regulated in High
GLIPR1	-0.1588472	2.12987464	-2.4024836	0.016651	0.05336784	-5.3166508	1.778559643	up-regulated in High
KRR1	0.26224674	2.09911018	7.87377697	2.19E-14	6.92E-13	21.0530438	13.66006511	up-regulated in Low
CSRP2	0.37596502	1.44968882	6.59022555	1.13E-10	2.13E-09	12.6521694	9.948715199	up-regulated in Low
E2F7	0.65679704	0.68803501	14.1908459	1.34E-38	3.59E-36	76.4004435	37.8743494	up-regulated in Low
SYT1	0.43837061	0.96779371	4.94742942	1.03E-06	9.81E-06	3.77118209	5.98638118	up-regulated in Low
PAWR	0.36601187	2.60985134	8.48884288	2.44E-16	1.02E-14	25.4908098	15.61300257	up-regulated in Low
PPP1R12A	0.20643499	2.57285291	4.99472906	8.18E-07	7.92E-06	3.99541803	6.087301807	up-regulated in Low
OTOGL	0.12590069	0.10242197	7.52961231	2.43E-13	6.65E-12	18.6830844	12.61527634	up-regulated in Low
CCDC59	0.45956796	2.78717418	11.4560917	4.06E-27	5.31E-25	50.0922634	26.39154053	up-regulated in Low
TMTC2	-0.1459507	1.9750929	-2.6022657	0.00953825	0.0334782	-4.8223509	2.020531314	up-regulated in High
SLC6A15	0.2527633	0.14635059	6.299554	6.59E-10	1.10E-08	10.9216904	9.180845468	up-regulated in Low
TSPAN19	-0.2882272	0.33209326	-4.956013	9.89E-07	9.44E-06	3.8117312	6.004635739	up-regulated in High
LRRIQ1	-0.1324203	0.43855286	-2.7909634	0.00545789	0.02084991	-4.3202466	2.262974941	up-regulated in High
ALX1	0.31775533	0.22726872	7.14220597	3.30E-12	7.77E-11	16.1166309	11.48207973	up-regulated in Low
NTS	1.18088486	0.91814236	6.54900078	1.45E-10	2.70E-09	12.4027388	9.838124679	up-regulated in Low
C12orf29	0.28326729	1.7700978	7.3791028	6.77E-13	1.74E-11	17.6730761	12.1695554	up-regulated in Low
TMTC3	0.36678436	2.39587326	7.62472469	1.26E-13	3.60E-12	19.3296963	12.90047795	up-regulated in Low
KITLG	-0.3747473	3.82302804	-4.1322281	4.22E-05	0.00028683	0.21479436	4.374928544	up-regulated in High
DUSP6	-0.6272007	5.03046336	-5.8077714	1.13E-08	1.54E-07	8.14654735	7.945900782	up-regulated in High
POC1B	0.33821481	1.88381396	7.16329398	2.87E-12	6.82E-11	16.2535147	11.5425723	up-regulated in Low
GALNT4	0.13956881	0.33337907	4.5522604	6.69E-06	5.37E-05	1.97384741	5.174845485	up-regulated in Low
ATP2B1	0.53755626	2.53958722	8.68057624	5.71E-17	2.55E-15	26.9253197	16.24348548	up-regulated in Low
EPYC	-0.1653057	0.49545308	-2.2435607	0.02530158	0.07545243	-5.6823082	1.59685244	up-regulated in High
LUM	-0.7232898	7.18182795	-6.804269	2.94E-11	6.06E-10	13.9682368	10.53176635	up-regulated in High
DCN	-0.9464624	4.63645829	-9.472709	1.12E-19	7.05E-18	33.0959519	18.95197244	up-regulated in High
PLEKHG7	-0.1627573	0.53189985	-3.6793932	0.00025941	0.00145383	-1.5030305	3.58601977	up-regulated in High
EEA1	-0.1276138	1.99308166	-3.0337556	0.00254207	0.01076313	-3.624199	2.594813389	up-regulated in High
UBE2N	0.37112642	3.85458988	9.18304943	1.14E-18	6.36E-17	30.7950592	17.94265355	up-regulated in Low
MRPL42	0.35129415	2.05746818	9.82815584	6.04E-21	4.45E-19	35.9866152	20.21910979	up-regulated in Low
SOCS2	-0.4413162	1.81498357	-5.3111932	1.65E-07	1.82E-06	5.5450317	6.783086636	up-regulated in High
PLXNC1	-0.450456	2.13366455	-7.0783966	5.01E-12	1.16E-10	15.7044419	11.29988377	up-regulated in High
NDUFA12	0.36253322	4.35818284	8.47053032	2.80E-16	1.15E-14	25.3550454	15.55331401	up-regulated in Low
METAP2	0.22311325	4.31644159	5.32404954	1.54E-07	1.71E-06	5.60978531	6.812104311	up-regulated in Low
USP44	-0.4171963	0.95311746	-7.5094892	2.78E-13	7.58E-12	18.5471052	12.5552852	up-regulated in High
NTN4	-0.7078354	3.50381652	-8.118982	3.75E-15	1.32E-13	22.7916944	14.4256841	up-regulated in High
SNRPF	0.67327702	3.77273965	13.7412871	1.24E-36	3.01E-34	71.8910169	35.90792295	up-regulated in Low
AMDHD1	0.19875404	0.49918346	3.64097361	0.00030017	0.00165358	-1.6401214	3.522627259	up-regulated in Low
HAL	0.53534406	1.14193767	5.39549902	1.06E-07	1.22E-06	5.97219515	6.974432157	up-regulated in Low
LTA4H	-0.3471905	4.88605053	-5.6170751	3.24E-08	4.06E-07	7.12328699	7.489213208	up-regulated in High
ELK3	-0.1961841	4.02525835	-2.8590643	0.004428	0.01740641	-4.1306736	2.353792891	up-regulated in High
NEDD1	0.3711765	2.58707343	8.93586421	7.97E-18	3.98E-16	28.8717378	17.09841872	up-regulated in Low
TMPO	0.88545429	3.67614646	16.9055146	5.91E-51	3.29E-48	104.754804	50.22809806	up-regulated in Low
SLC25A3	0.32647307	5.12545957	9.04450337	3.41E-18	1.78E-16	29.7124196	17.46749726	up-regulated in Low
IKBIP	0.19797137	2.60092438	3.80229102	0.00016124	0.00095324	-1.0553637	3.792531289	up-regulated in Low
UHRF1BP1L	0.19327971	1.95122659	5.1000839	4.84E-07	4.91E-06	4.50180133	6.314974566	up-regulated in Low
ACTR6	0.3246609	2.73974961	7.90020882	1.81E-14	5.80E-13	21.2384781	13.74175423	up-regulated in Low
SCYL2	0.16852952	3.33030261	3.96549461	8.40E-05	0.00053304	-0.4394691	4.075530452	up-regulated in Low
NR1H4	0.1369069	0.17448444	3.02799746	0.0025901	0.01093636	-3.6413551	2.586684128	up-regulated in Low
GAS2L3	0.4425565	1.15008605	10.0604891	8.63E-22	6.93E-20	37.9147692	21.0638317	up-regulated in Low
SLC5A8	-0.250615	0.79390494	-2.446771	0.0147606	0.04829481	-5.2103963	1.830896104	up-regulated in High
UTP20	0.39355913	2.19932439	7.06892879	5.33E-12	1.23E-10	15.6435396	11.2729587	up-regulated in Low
ARL1	0.14571186	4.41528769	3.30581034	0.00101569	0.00482846	-2.7779585	2.993238729	up-regulated in Low
GNPTAB	-0.2909655	3.15591498	-6.474089	2.30E-10	4.14E-09	11.9528571	9.638585733	up-regulated in High
DRAM1	-0.9448708	4.79531275	-9.6946084	1.82E-20	1.25E-18	34.8920255	19.73940018	up-regulated in High
NUP37	0.59600765	2.71431144	14.557839	3.17E-40	9.44E-38	80.1268478	39.49889769	up-regulated in Low
PARPBP	0.79720762	1.18361911	18.1270074	1.04E-56	9.46E-54	117.976685	55.98426163	up-regulated in Low
PAH	0.44529758	0.37183172	6.0912459	2.25E-09	3.47E-08	9.72249259	8.647787604	up-regulated in Low
ASCL1	0.63984219	1.02899257	3.1648009	0.00164731	0.00738212	-3.2252826	2.783223879	up-regulated in Low
NT5DC3	0.25711765	1.47847157	5.23464446	2.45E-07	2.62E-06	5.16237483	6.611520476	up-regulated in Low
HSP90B1	0.39525937	7.80904595	6.87598372	1.86E-11	3.94E-10	14.4169887	10.73040972	up-regulated in Low
C12orf73	0.36856647	1.7792143	10.1436617	4.27E-22	3.57E-20	38.612273	21.36931758	up-regulated in Low

TDG	0.55156494	2.57787166	11.9625618	3.77E-29	5.77E-27	54.7420263	28.42323749	up-regulated in Low
GLT8D2	-0.281268	1.75966894	-3.924466	9.92E-05	0.00061972	-0.5965919	4.003447418	up-regulated in High
HCFC2	-0.1284215	1.62797639	-3.6159274	0.00032991	0.00179471	-1.7287583	3.481600266	up-regulated in High
NFYB	0.13672995	2.80762386	3.91588169	0.0001027	0.00063931	-0.6292721	3.988445309	up-regulated in Low
TXNRD1	1.28538912	5.17358263	10.2299425	2.05E-22	1.75E-20	39.3398169	21.6879118	up-regulated in Low
EID3	0.33015914	1.09801301	5.46084084	7.51E-08	8.85E-07	6.30738272	7.124454599	up-regulated in Low
CHST11	0.26553094	2.75617923	3.56764295	0.00039521	0.00210461	-1.897995	3.403175355	up-regulated in Low
C12orf45	0.36122271	2.99620891	8.15042727	2.98E-15	1.07E-13	23.0176203	14.52512266	up-regulated in Low
ALDH1L2	0.22172469	1.20624599	3.32510362	0.00094938	0.00454468	-2.7153027	3.022559744	up-regulated in Low
KIAA1033	0.15699248	3.03495745	3.76260894	0.00018827	0.00109517	-1.2014282	3.725230897	up-regulated in Low
APPL2	0.13598328	3.00038841	2.8984035	0.0039169	0.01566411	-4.0191501	2.407057584	up-regulated in Low
C12orf75	0.39107979	2.40179514	3.37466716	0.00079702	0.00389452	-2.5527451	3.098530435	up-regulated in Low
CKAP4	0.48388914	5.36679497	7.90083191	1.80E-14	5.79E-13	21.2428552	13.74368239	up-regulated in Low
TCP11L2	-0.1292563	1.22675453	-3.0831076	0.0021627	0.00935429	-3.4758712	2.665003838	up-regulated in High
POLR3B	0.14936271	1.81996736	4.46907907	9.74E-06	7.57E-05	1.61299882	5.01127135	up-regulated in Low
RIC8B	0.12987632	1.3988447	4.56493614	6.31E-06	5.09E-05	2.02937363	5.199994641	up-regulated in Low
TMEM263	0.33637691	4.03746557	6.26306148	8.20E-10	1.35E-08	10.7091187	9.086414286	up-regulated in Low
CRY1	0.18754392	2.49394066	4.09602928	4.91E-05	0.00032916	0.07060845	4.309047164	up-regulated in Low
BTBD11	0.25739383	0.58756255	4.6522181	4.22E-06	3.53E-05	2.41556075	5.374759791	up-regulated in Low
PWP1	0.37129056	4.0602711	11.3418874	1.15E-26	1.44E-24	49.0597887	25.94023577	up-regulated in Low
PRDM4	0.26299303	3.04195429	6.93622174	1.26E-11	2.74E-10	14.796926	10.89853013	up-regulated in Low
CMKLR1	-0.2545285	2.05210042	-3.2808625	0.00110781	0.00520495	-2.8584605	2.955533302	up-regulated in High
SART3	0.28843929	3.09000922	7.87619089	2.15E-14	6.82E-13	21.0699587	13.66751696	up-regulated in Low
ISCU	-0.2137649	4.20341137	-5.7770124	1.34E-08	1.80E-07	7.97947509	7.871390125	up-regulated in High
TMEM119	-0.8674319	3.19532626	-10.10198	6.08E-22	5.00E-20	38.2622516	21.21602441	up-regulated in High
SELPLG	-0.6041764	3.8336443	-7.3839016	6.55E-13	1.69E-11	17.7050275	12.18366026	up-regulated in High
CORO1C	0.27118942	4.39676284	4.75854707	2.56E-06	2.24E-05	2.89506735	5.591414058	up-regulated in Low
ALKBH2	0.48335561	2.5587366	10.1049452	5.93E-22	4.89E-20	38.2871176	21.22691494	up-regulated in Low
UNG	0.73927204	4.0254746	15.8522055	4.47E-46	1.84E-43	93.554601	45.35012597	up-regulated in Low
ACACB	-0.1706482	0.94395938	-4.2989361	2.07E-05	0.0001501	0.8940645	4.684613331	up-regulated in High
FOXN4	0.18043651	0.15251075	4.60422392	5.27E-06	4.32E-05	2.20237528	5.278316438	up-regulated in Low
KCTD10	-0.1197816	3.07138396	-3.7089817	0.00023162	0.00131695	-1.3965225	3.63521981	up-regulated in High
FAM222A	0.49561227	0.91536938	8.96846427	6.18E-18	3.14E-16	29.1232387	17.20884414	up-regulated in Low
TRPV4	-0.192402	1.71246415	-2.4058988	0.01649798	0.05296463	-5.3085244	1.782569195	up-regulated in High
TCHP	0.135062	1.63439716	4.20088629	3.15E-05	0.00022021	0.49152095	4.501223175	up-regulated in Low
ANKRD13A	-0.1573678	3.39650371	-3.1146809	0.00194808	0.00854243	-3.3797693	2.710393802	up-regulated in High
C12orf76	-0.1420739	1.73839153	-2.9884673	0.00294296	0.01221585	-3.7582839	2.531216324	up-regulated in High
IFT81	0.25625462	1.84161612	5.16546391	3.48E-07	3.63E-06	4.82083072	6.458255116	up-regulated in Low
ATP2A2	0.41869577	4.92601546	7.88998674	1.95E-14	6.22E-13	21.1667072	13.71013806	up-regulated in Low
ANAPC7	0.43786773	3.14592513	12.0607461	1.50E-29	2.36E-27	55.6563925	28.82263062	up-regulated in Low
ARPC3	0.29008986	6.26911889	6.54519555	1.49E-10	2.76E-09	12.3797815	9.827944617	up-regulated in Low
GPN3	0.36494169	3.28014343	8.77358022	2.80E-17	1.30E-15	27.6296514	16.55292082	up-regulated in Low
FAM216A	0.33176864	1.31646215	6.99875385	8.43E-12	1.88E-10	15.194215	11.07427025	up-regulated in Low
VPS29	0.29920082	4.41822912	6.80293992	2.96E-11	6.11E-10	13.9599567	10.52810033	up-regulated in Low
PPTC7	0.23032848	3.42899261	4.99911072	8.00E-07	7.77E-06	4.01628804	6.096691362	up-regulated in Low
TCTN1	-0.1338459	1.98475084	-2.9550927	0.00327455	0.01340189	-3.8558509	2.484847918	up-regulated in High
HVCN1	-0.277431	1.74842972	-5.4677456	7.24E-08	8.56E-07	6.34301135	7.14039504	up-regulated in High
PPP1CC	0.43347723	4.34341112	11.4919723	2.93E-27	3.91E-25	50.4178872	26.53386054	up-regulated in Low
SH2B3	-0.2465327	2.8220003	-4.2437641	2.62E-05	0.00018627	0.6664936	4.580982877	up-regulated in High
ATXN2	0.12031628	2.15816454	3.13500564	0.00182048	0.00805271	-3.3174072	2.739813082	up-regulated in Low
BRAP	0.22343342	2.79034824	7.43584758	4.60E-13	1.21E-11	18.0519547	12.33679089	up-regulated in Low
ALDH2	-0.5654072	4.4104608	-6.9220407	1.38E-11	2.99E-10	14.7072369	10.85884812	up-regulated in High
MAPKAPK5	0.23430809	1.88473655	8.89724396	1.08E-17	5.28E-16	28.5746493	16.96796521	up-regulated in Low
ERP29	0.14715092	6.11707658	2.73008951	0.00655714	0.02435907	-4.485952	2.183285776	up-regulated in Low
NAA25	0.38946521	2.00702398	10.1746234	3.29E-22	2.75E-20	38.872887	21.48344705	up-regulated in Low
RPL6	0.23764975	6.84138131	5.12175088	4.34E-07	4.43E-06	4.60712309	6.362289038	up-regulated in Low
PTPN11	0.37639518	4.09866304	7.45397156	4.07E-13	1.08E-11	18.1734554	12.39041185	up-regulated in Low
OAS1	0.41988062	3.9947953	4.15166146	3.89E-05	0.00026606	0.29268892	4.410497925	up-regulated in Low
OAS3	0.45121442	3.82114878	5.05539003	6.05E-07	6.01E-06	4.2858179	6.217905573	up-regulated in Low
RASAL1	0.3314022	1.48450423	4.0744894	5.37E-05	0.00035706	-0.0146258	4.270076318	up-regulated in Low
CCDC42B	-0.4179755	0.84927013	-4.8609349	1.57E-06	1.44E-05	3.36613706	5.803915009	up-regulated in High
DDX54	0.42861639	4.00387722	9.13873818	1.62E-18	8.91E-17	30.4475234	17.79014228	up-regulated in Low
RITA1	0.2994263	3.58912554	7.53996003	2.26E-13	6.22E-12	18.7531203	12.64617267	up-regulated in Low
IQCD	0.23193316	1.65242702	3.35408794	0.00085729	0.00415493	-2.6205195	3.066873797	up-regulated in Low
PLBD2	0.21345811	4.42970414	3.91523704	0.00010296	0.00064063	-0.6317236	3.987319816	up-regulated in Low
SDS	0.21207798	1.89001737	2.30929622	0.02133749	0.06554252	-5.5340294	1.670856616	up-regulated in Low
SDSL	0.29037357	2.94509361	4.13698567	4.13E-05	0.00028145	0.23383245	4.383623423	up-regulated in Low
LHX5	0.13561661	0.17312139	4.19033609	3.30E-05	0.00022935	0.44872207	4.481702597	up-regulated in Low
RBM19	0.26456434	2.6660291	6.63238289	8.66E-11	1.66E-09	12.9085994	10.06237943	up-regulated in Low
TBX5	-0.5729586	1.46900686	-10.790502	1.59E-24	1.67E-22	44.1623037	23.7985509	up-regulated in High
TBX3	-0.2866289	1.37250401	-4.8413441	1.73E-06	1.57E-05	3.27529685	5.762961799	up-regulated in High

RNFT2	0.49122094	0.89308562	8.72950197	3.93E-17	1.78E-15	27.2951547	16.40597579	up-regulated in Low
FBXO21	0.14926072	3.20727797	3.5723054	0.00038841	0.00207136	-1.8817474	3.410709873	up-regulated in Low
NOS1	0.12690716	0.17830554	2.94642408	0.0033661	0.01373803	-3.8810196	2.47287354	up-regulated in Low
RFC5	0.72840202	2.68369902	15.9495714	1.60E-46	6.95E-44	94.5806553	45.79708608	up-regulated in Low
WSB2	0.3863488	4.25369636	8.31414512	8.97E-16	3.46E-14	24.2046114	15.0473911	up-regulated in Low
VSIG10	0.16813039	1.98842635	2.71280993	0.00690364	0.02545037	-4.5323429	2.160921701	up-regulated in Low
TAOK3	-0.1514371	2.46580726	-3.4794363	0.00054679	0.00279606	-2.2015739	3.262179798	up-regulated in High
SUDS3	0.37953287	3.4911533	8.30165806	9.83E-16	3.77E-14	24.1134476	15.00728953	up-regulated in Low
HSPB8	-0.5972327	3.10737103	-6.3262813	5.62E-10	9.48E-09	11.0780458	9.250288006	up-regulated in High
CCDC60	-0.2783624	0.32654831	-5.8565813	8.61E-09	1.19E-07	8.41325256	8.064803541	up-regulated in High
TMEM233	-0.2376766	0.73591646	-3.498579	0.00050988	0.00263061	-2.1363053	3.292529059	up-regulated in High
PRKAB1	-0.1495874	3.5762069	-2.5286479	0.01175958	0.03994221	-5.0089715	1.929608203	up-regulated in High
CIT	-0.28075	2.86031301	-2.1198174	0.03451788	0.09698515	-5.9500428	1.461955835	up-regulated in High
CCDC64	0.3791654	2.18464856	4.52388316	7.61E-06	6.04E-05	1.85005559	5.118757236	up-regulated in Low
RAB35	0.28793945	3.8444288	8.27191958	1.22E-15	4.63E-14	23.8967539	14.91196281	up-regulated in Low
GCN1L1	0.42297786	4.00444887	8.32302908	8.40E-16	3.25E-14	24.2695332	15.07594824	up-regulated in Low
RPLP0	0.40866321	8.2889901	7.04680656	6.16E-12	1.41E-10	15.5014969	11.21015615	up-regulated in Low
PXN	0.12293502	4.14087795	2.14660631	0.03230938	0.09197607	-5.8933453	1.490671363	up-regulated in Low
SIRT4	-0.1136466	1.10049379	-3.0507225	0.00240525	0.01026123	-3.5734648	2.618840124	up-regulated in High
PLA2G1B	-1.2603214	1.83038821	-9.015726	4.27E-18	2.21E-16	29.4890212	17.36943025	up-regulated in High
MSI1	0.56835989	0.85513571	6.03746908	3.07E-09	4.62E-08	9.41853544	8.512539437	up-regulated in Low
COX6A1	0.46785208	6.21610825	8.11502686	3.86E-15	1.36E-13	22.7633249	14.41319687	up-regulated in Low
TRIAP1	0.38441786	4.23808673	9.15519973	1.42E-18	7.86E-17	30.5764929	17.84674067	up-regulated in Low
GATC	0.30455419	2.68996105	9.39047226	2.17E-19	1.32E-17	32.4376296	18.66325815	up-regulated in Low
SRSF9	0.48587228	4.51343836	13.4624218	1.99E-35	4.53E-33	69.1264181	34.70205723	up-regulated in Low
DYNLL1	0.40198927	5.58657084	9.88027249	3.91E-21	2.97E-19	36.4165153	20.40748105	up-regulated in Low
COQ5	0.32301877	3.54200948	7.48167969	3.37E-13	9.05E-12	18.359663	12.47258112	up-regulated in Low
POP5	0.3008658	3.37489207	6.50209998	1.94E-10	3.52E-09	12.1205662	9.712982159	up-regulated in Low
ACADS	-0.2823981	3.39748836	-4.4346577	1.14E-05	8.71E-05	1.46547115	4.94432553	up-regulated in High
SPPL3	0.10405218	3.4105446	2.90226005	0.00386981	0.01549848	-4.0081378	2.412310926	up-regulated in Low
HNF1A	0.21674539	0.43400603	3.73174646	0.00021218	0.00121898	-1.3140283	3.673297444	up-regulated in Low
C12orf43	0.22560959	1.81529926	9.36051722	2.76E-19	1.65E-17	32.1988276	18.5585158	up-regulated in Low
OASL	0.30899612	2.03481736	3.39170283	0.00075014	0.00369857	-2.4963414	3.124857399	up-regulated in Low
P2RX7	-0.3040724	1.4046716	-4.9772612	8.91E-07	8.58E-06	3.91238231	6.049937968	up-regulated in High
P2RX4	-0.1833736	2.79526038	-3.7540855	0.0001946	0.00112821	-1.2326131	3.710852403	up-regulated in High
CAMKK2	0.17175582	3.25025168	5.05790112	5.98E-07	5.94E-06	4.29790735	6.223340397	up-regulated in Low
ANAPC5	0.30588633	3.3823973	8.68210468	5.64E-17	2.53E-15	26.9368502	16.24855185	up-regulated in Low
RNF34	0.33903208	2.9408546	9.87877082	3.96E-21	3.00E-19	36.4041069	20.4020443	up-regulated in Low
KDM2B	0.13251027	1.70699349	3.90377221	0.00010781	0.00066683	-0.6752582	3.967329444	up-regulated in Low
ORAI1	0.14182289	3.83870993	3.28989286	0.00107361	0.00506748	-2.8293886	2.969154281	up-regulated in Low
MORN3	-0.1138966	0.71353046	-2.2519976	0.02475942	0.07413479	-5.6635119	1.606259484	up-regulated in High
TMEM120B	0.15289352	1.78581349	3.47433149	0.00055705	0.00284277	-2.2189217	3.25410981	up-regulated in Low
RHOF	0.37447374	1.03040622	7.1148779	3.95E-12	9.23E-11	15.939731	11.40389352	up-regulated in Low
HPD	0.19537628	0.40603869	4.04198233	6.14E-05	0.0004019	-0.1424617	4.211590422	up-regulated in Low
PSMD9	0.24837988	1.81553495	8.78950071	2.48E-17	1.16E-15	27.75077	16.60612404	up-regulated in Low
WDR66	-0.1860234	1.14159755	-2.7377578	0.00640845	0.02392088	-4.4652731	2.193246796	up-regulated in High
LRRC43	-0.1155285	0.74248955	-2.0422751	0.04165303	0.11294042	-6.1102141	1.380353386	up-regulated in High
B3GNT4	0.13664259	0.57988116	3.18009981	0.00156443	0.00706873	-3.1776544	2.805644831	up-regulated in Low
DIABLO	0.26601361	1.41627158	9.64112074	2.83E-20	1.91E-18	34.456484	19.54848551	up-regulated in Low
VPS33A	0.27542625	1.9630483	10.2310194	2.03E-22	1.74E-20	39.3489222	21.69189874	up-regulated in Low
CLIP1	0.26883409	2.76894865	5.52271114	5.40E-08	6.54E-07	6.62805673	7.267884622	up-regulated in Low
ZCCHC8	0.21080289	2.08333236	7.22405273	1.92E-12	4.66E-11	16.649734	11.71763691	up-regulated in Low
RSRC2	0.10761905	3.0999911	3.30714249	0.00101098	0.00480918	-2.7736435	2.995258731	up-regulated in Low
KNTC1	0.67644027	1.77044296	12.095341	1.09E-29	1.73E-27	55.9795392	28.96377041	up-regulated in Low
DENR	0.5024779	4.44525695	13.1711544	3.51E-34	7.37E-32	66.2671175	33.45461597	up-regulated in Low
PITPNM2	0.11663677	1.81438673	2.0665365	0.03929678	0.1077547	-6.0607303	1.405643074	up-regulated in Low
MPHOSPH9	0.36817284	1.30523888	9.26110468	6.13E-19	3.53E-17	31.4101439	18.21253545	up-regulated in Low
C12orf65	0.14151908	2.21938936	4.06396142	5.61E-05	0.0003712	-0.0561325	4.251091538	up-regulated in Low
CDK2AP1	0.24413544	4.50028578	4.97901539	8.84E-07	8.51E-06	3.92070928	6.053685282	up-regulated in Low
SBNO1	0.34271888	2.46592814	8.23007779	1.67E-15	6.22E-14	23.5928657	14.77826228	up-regulated in Low
SETD8	0.37487913	2.83389795	10.074608	7.66E-22	6.21E-20	38.0329072	21.11557591	up-regulated in Low
RILPL2	-0.4522444	2.32085252	-10.088847	6.80E-22	5.53E-20	38.1521643	21.16780889	up-regulated in High
TMED2	0.40632896	6.65635851	7.8730874	2.20E-14	6.95E-13	21.0482124	13.65793667	up-regulated in Low
DDX55	0.43029167	2.34081405	10.6253829	6.77E-24	6.78E-22	42.7248905	23.16964079	up-regulated in Low
EIF2B1	0.34506061	4.01082091	10.8010517	1.45E-24	1.52E-22	44.2546065	23.83893072	up-regulated in Low
GTF2H3	0.32482876	3.17365645	8.62184743	8.93E-17	3.91E-15	26.4834041	16.04929625	up-regulated in Low
DNAH10	-0.196123	0.32582429	-4.8445841	1.70E-06	1.54E-05	3.29029759	5.769725344	up-regulated in High
CCDC92	-0.3011994	2.69195321	-6.8992748	1.60E-11	3.43E-10	14.5635677	10.7952768	up-regulated in High
ZNF664	0.18494072	4.68348424	3.40674319	0.0007109	0.00352473	-2.4463186	3.148192098	up-regulated in Low
SCARB1	0.62841496	2.64457334	8.81310363	2.06E-17	9.77E-16	27.9306293	16.68512576	up-regulated in Low
UBC	0.10465166	7.2961706	2.73877792	0.00638891	0.02385401	-4.462518	2.194573576	up-regulated in Low

DHX37	0.50219749	2.45781798	11.8845755	7.81E-29	1.16E-26	54.0186984	28.10725919	up-regulated in Low
BRI3BP	0.6694257	2.48719328	13.4836612	1.61E-35	3.68E-33	69.3360649	34.7935099	up-regulated in Low
AACS	0.36324129	1.88341282	8.36505607	6.15E-16	2.43E-14	24.5773645	15.21134283	up-regulated in Low
TMEM132C	-0.1034113	0.1258906	-4.2671759	2.37E-05	0.00017006	0.76272796	4.624820055	up-regulated in High
SLC15A4	0.1278246	2.77056234	3.61616551	0.00032962	0.00179377	-1.7279184	3.481989197	up-regulated in Low
TMEM132D	-0.2989121	0.33124876	-5.4362741	8.55E-08	9.99E-07	6.18094141	7.06787465	up-regulated in High
FZD10	0.63299828	0.78945749	5.62506053	3.10E-08	3.90E-07	7.16553419	7.508084794	up-regulated in Low
RAN	0.75802484	5.64761836	15.9058695	2.53E-46	1.09E-43	94.1198679	45.59636456	up-regulated in Low
GPR133	-0.6802523	1.50523865	-7.9349343	1.41E-14	4.62E-13	21.4828296	13.84938585	up-regulated in High
SFSWAP	0.17731499	2.37084831	4.48011356	9.27E-06	7.22E-05	1.66051473	5.03282441	up-regulated in Low
ULK1	0.24175255	3.30909299	4.24421791	2.62E-05	0.00018599	0.66835428	4.581830664	up-regulated in Low
PUS1	0.553214	2.33777776	11.9462753	4.39E-29	6.68E-27	54.5907511	28.35715674	up-regulated in Low
EP400	0.19812703	2.25679579	4.23925574	2.68E-05	0.0001895	0.64801844	4.572564535	up-regulated in Low
DDX51	0.23762694	2.26835637	5.41777471	9.43E-08	1.09E-06	6.08606154	7.025408037	up-regulated in Low
NOC4L	0.44551347	3.5039312	9.98948189	1.57E-21	1.23E-19	37.3222917	20.80430753	up-regulated in Low
FBRSL1	0.29338427	2.8412799	5.16469083	3.50E-07	3.64E-06	4.81703707	6.45655202	up-regulated in Low
P2RX2	-0.1358276	0.21848546	-3.9222522	0.0001001	0.0006248	-0.6050263	3.999575889	up-regulated in High
POLE	0.53959164	1.79951388	11.3001221	1.68E-26	2.09E-24	48.6837219	25.77583699	up-regulated in Low
PXMP2	0.37236482	2.80842394	7.52420484	2.52E-13	6.89E-12	18.6465158	12.59914355	up-regulated in Low
PGAM5	0.76164164	3.83028038	16.4392918	8.75E-49	4.22E-46	99.7710318	48.05778897	up-regulated in Low
ANKLE2	0.28477832	2.58772715	6.62687463	8.96E-11	1.72E-09	12.8750166	10.04749531	up-regulated in Low
GOLGA3	0.11327168	3.70166114	2.07364375	0.0386284	0.1062463	-6.0461254	1.413093276	up-regulated in Low
CHFR	0.18892951	1.55196484	5.62889696	3.04E-08	3.83E-07	7.18584987	7.517159171	up-regulated in Low
ZNF605	0.17925402	1.27364746	4.77350862	2.39E-06	2.10E-05	2.96333286	5.622229144	up-regulated in Low
ZNF140	0.10868705	2.05512533	3.12894104	0.00185773	0.00819671	-3.3360559	2.731018421	up-regulated in Low
MPHOSPH8	-0.2047421	3.42351015	-4.7492929	2.68E-06	2.33E-05	2.85294111	5.572394716	up-regulated in High
PSPC1	0.23833893	3.41390205	6.18032684	1.34E-09	2.13E-08	10.2310918	8.873967314	up-regulated in Low
ZMYM2	0.10812694	2.66231723	2.43685492	0.01516655	0.04941293	-5.2343517	1.819113325	up-regulated in Low
GJB2	0.46608022	3.20189211	3.14703909	0.00174862	0.00777109	-3.2803015	2.7573049	up-regulated in Low
CRYL1	-0.3437316	3.58601533	-5.5984229	3.59E-08	4.47E-07	7.02481205	7.445219277	up-regulated in High
LATS2	-0.1416385	2.51230468	-2.9298125	0.00354808	0.01438322	-3.9290506	2.450006981	up-regulated in High
SAP18	0.11109996	4.89401008	2.60024077	0.00959394	0.0336494	-4.8275539	2.018002858	up-regulated in Low
SKA3	1.14143018	1.59824943	21.3191908	4.75E-72	5.54E-68	153.23015	71.32365409	up-regulated in Low
MRPL57	0.16515897	4.18339132	3.25747175	0.00120117	0.00559361	-2.9334076	2.920395483	up-regulated in Low
SGCG	-0.149517	0.28301291	-4.8212436	1.90E-06	1.71E-05	3.18244035	5.721087401	up-regulated in High
SACS	0.21979468	0.95096327	5.7317774	1.73E-08	2.27E-07	7.7351835	7.762403774	up-regulated in Low
TNFRSF19	-0.5362205	2.55358195	-5.3477759	1.36E-07	1.53E-06	5.72965451	6.865809526	up-regulated in High
SPATA13	-0.2790896	2.23447702	-5.6935183	2.13E-08	2.76E-07	7.52987822	7.670775154	up-regulated in High
PARP4	-0.2423006	4.142581	-4.5396017	7.08E-06	5.66E-05	1.91853766	5.149788882	up-regulated in High
CENPJ	0.3228308	1.47411602	7.43861199	4.52E-13	1.19E-11	18.0704717	12.34496312	up-regulated in Low
MTMR6	-0.1496583	2.91528508	-3.1979726	0.00147248	0.00670152	-3.1217339	2.831950387	up-regulated in High
ATP8A2	-0.1854777	0.48306773	-3.9799284	7.92E-05	0.0005066	-0.3838288	4.101038776	up-regulated in High
SHISA2	-0.7315938	1.86682977	-5.3764861	1.17E-07	1.33E-06	5.87533679	6.931060682	up-regulated in High
CDK8	0.33312899	2.38823928	8.77890788	2.69E-17	1.25E-15	27.6701649	16.57071723	up-regulated in Low
WASF3	-0.1505864	1.33454729	-2.4175983	0.01598314	0.05163126	-5.2806005	1.796338041	up-regulated in High
USP12	-0.1287	2.98965966	-3.0385932	0.00250235	0.01062232	-3.6097614	2.60165275	up-regulated in High
RASL11A	-0.5871844	2.56801307	-6.4153116	3.29E-10	5.77E-09	11.6029296	9.483311968	up-regulated in High
GTF3A	0.29516761	5.04454479	5.40726912	9.97E-08	1.15E-06	6.0323083	7.001345238	up-regulated in Low
LNX2	-0.3547018	3.06706847	-6.317254	5.93E-10	9.97E-09	11.0251729	9.226806898	up-regulated in High
PDX1	0.26341922	0.38465309	3.73626895	0.0002085	0.00119986	-1.2975831	3.680885214	up-regulated in Low
CDX2	0.27923533	0.22749724	5.73009701	1.74E-08	2.29E-07	7.72614092	7.758368719	up-regulated in Low
FLT3	-0.1657465	0.37667491	-6.8642195	2.01E-11	4.23E-10	14.3431078	10.6977113	up-regulated in High
POMP	0.36288603	5.82957682	8.1406954	3.20E-15	1.15E-13	22.9476281	14.49431758	up-regulated in Low
SLC46A3	-0.4486413	2.73167443	-6.1953948	1.22E-09	1.96E-08	10.3177471	8.912488714	up-regulated in High
SLC7A1	0.63574995	2.85914021	10.1989019	2.67E-22	2.26E-20	39.07761	21.57309602	up-regulated in Low
UBL3	-0.4928872	4.03755425	-9.1389183	1.62E-18	8.91E-17	30.4489337	17.79076119	up-regulated in High
HMGB1	0.17741651	4.54362159	4.30087007	2.05E-05	0.00014898	0.90209129	4.688266421	up-regulated in Low
ALOX5AP	-0.7644723	4.25487623	-7.2285962	1.86E-12	4.54E-11	16.6794718	11.73077411	up-regulated in High
TEX26	-0.1861374	0.22218909	-4.6206334	4.88E-06	4.03E-05	2.27503629	5.311196303	up-regulated in High
HSPH1	0.25479475	3.76265957	4.31872271	1.90E-05	0.00013878	0.97634425	4.722053159	up-regulated in Low
FRY	-0.4955915	1.35291845	-10.81249	1.31E-24	1.38E-22	44.3547493	23.88273963	up-regulated in High
BRCA2	0.36088557	0.73994098	12.0129249	2.36E-29	3.65E-27	55.2105331	28.6278855	up-regulated in Low
N4BP2L1	-0.4881403	1.69011348	-9.8542229	4.86E-21	3.63E-19	36.2014464	20.31324575	up-regulated in High
N4BP2L2	-0.2491343	2.30050132	-5.6708248	2.42E-08	3.09E-07	7.40867044	7.616664113	up-regulated in High
KL	-0.1681168	0.64312203	-2.907082	0.00381165	0.0153082	-3.9943488	2.418887334	up-regulated in High
STARD13	-0.2490853	1.3397987	-5.8739211	7.81E-09	1.09E-07	8.508468	8.107240297	up-regulated in High
RFC3	0.79893251	2.49698959	15.2013276	4.11E-43	1.48E-40	86.7504617	42.38566157	up-regulated in Low
NBEA	-0.2064795	1.04441386	-3.4843243	0.00053714	0.00275393	-2.1849404	3.269916104	up-regulated in High
SOHLH2	0.14674878	0.31122901	2.2863604	0.02265423	0.06877307	-5.5862418	1.64485074	up-regulated in Low
EXOSC8	0.28689161	2.67677777	6.79823901	3.05E-11	6.29E-10	13.9306817	10.51513864	up-regulated in Low
FREM2	-0.2151114	0.9024523	-3.2432657	0.00126137	0.00584089	-2.9786748	2.899156335	up-regulated in High

STOML3	-0.4255666	0.53944283	-5.7756255	1.36E-08	1.81E-07	7.97195987	7.868038013	up-regulated in High
PROSER1	0.20512901	2.97671885	3.9318302	9.63E-05	0.00060314	-0.5685031	4.016339201	up-regulated in Low
NHLRC3	-0.1307446	2.55303763	-3.3016903	0.0010304	0.00488763	-2.7912934	2.986995529	up-regulated in High
LHFP	-0.6277954	3.58133351	-10.00758	1.35E-21	1.07E-19	37.4730371	20.87034211	up-regulated in High
FOXO1	-0.3647757	2.45191465	-5.774429	1.36E-08	1.83E-07	7.9654783	7.86514692	up-regulated in High
SLC25A15	0.38460855	2.06826239	6.96400237	1.06E-11	2.32E-10	14.9730647	10.97645206	up-regulated in Low
ELF1	-0.1668764	4.13212881	-3.5830666	0.00037314	0.00199783	-1.84417	3.428131294	up-regulated in High
RGCC	-0.8127947	5.23775537	-10.598391	8.56E-24	8.47E-22	42.4912349	23.06739447	up-regulated in High
AKAP11	-0.1178184	2.60785541	-2.5957961	0.00971721	0.03403063	-4.8389604	2.012458466	up-regulated in High
TNFSF11	0.22647707	0.70348881	3.07334829	0.00223331	0.00962549	-3.5053856	2.651050409	up-regulated in Low
FAM216B	-0.6960225	0.89431888	-6.795648	3.10E-11	6.39E-10	13.9145535	10.50799765	up-regulated in High
EPSTI1	0.16540798	2.56549843	2.02854457	0.04303893	0.11585589	-6.1379644	1.36613851	up-regulated in Low
DNAJC15	0.1408029	2.51585312	2.08496663	0.0375836	0.10391598	-6.022756	1.425001613	up-regulated in Low
SERP2	-0.2306867	0.79861074	-5.3864283	1.11E-07	1.27E-06	5.92594789	6.953724573	up-regulated in High
TSC22D1	-0.3518298	4.66890072	-5.4874627	6.52E-08	7.79E-07	6.44497173	7.186006279	up-regulated in High
NUFIP1	0.24187878	1.78993987	7.93982709	1.37E-14	4.47E-13	21.5173251	13.86457925	up-regulated in Low
GTF2F2	0.24124113	2.90047602	6.78554037	3.31E-11	6.77E-10	13.8516848	10.48016049	up-regulated in Low
TPT1	-0.2959568	8.60824023	-4.3924323	1.37E-05	0.00010336	1.28593504	4.862796757	up-regulated in High
SLC25A30	-0.1848092	1.70068252	-5.1042088	4.74E-07	4.81E-06	4.52182133	6.323969301	up-regulated in High
CPB2	-0.6334853	1.13663559	-5.0370934	6.63E-07	6.53E-06	4.19789396	6.178373922	up-regulated in High
LCP1	-0.3777804	5.13880795	-3.8110938	0.00015576	0.00092527	-1.022766	3.807540817	up-regulated in High
KIAA0226L	-0.2657812	0.81955303	-6.7940926	3.14E-11	6.45E-10	13.9048736	10.50371166	up-regulated in High
NUDT15	0.46751269	3.4500714	10.7315189	2.67E-24	2.77E-22	43.6472493	23.57321777	up-regulated in Low
ITM2B	-0.4432554	5.82396132	-8.2780433	1.17E-15	4.45E-14	23.9413274	14.93157208	up-regulated in High
RB1	-0.1100479	3.04822711	-2.2079381	0.02770582	0.08119967	-5.7609089	1.557428972	up-regulated in High
LPAR6	-0.2944228	2.29771815	-4.9566953	9.86E-07	9.42E-06	3.81495728	6.006087978	up-regulated in High
RCBTB2	-0.4455859	2.09075903	-9.7882623	8.41E-21	6.03E-19	35.6585765	20.0753586	up-regulated in High
FNDC3A	-0.1280265	3.57227365	-2.3704028	0.0181505	0.05733663	-5.3924346	1.741111511	up-regulated in High
CAB39L	-0.2658583	1.732866	-5.3959576	1.06E-07	1.21E-06	5.97453522	6.975479888	up-regulated in High
SETDB2	-0.3262723	1.68778069	-9.7693803	9.83E-21	6.97E-19	35.5036261	20.00745315	up-regulated in High
PHF11	-0.2241812	2.75131428	-5.8066581	1.14E-08	1.55E-07	8.14048672	7.943198227	up-regulated in High
EBPL	0.14162386	4.23658637	2.24847715	0.02498441	0.07469736	-5.6713635	1.602330925	up-regulated in Low
KPNA3	0.17577682	3.56729142	4.05383694	5.85E-05	0.0003851	-0.0959537	4.232873322	up-regulated in Low
TRIM13	-0.2231609	2.18572549	-6.4305642	3.00E-10	5.29E-09	11.6934754	9.523495788	up-regulated in High
KCNRG	-0.1904573	0.43858446	-4.6185796	4.93E-06	4.07E-05	2.26592909	5.307075696	up-regulated in High
INTS6	0.103429	1.17950974	4.25720624	2.48E-05	0.00017669	0.72168745	4.606127662	up-regulated in Low
WDFY2	-0.1396922	1.18512072	-4.5400761	7.07E-06	5.65E-05	1.92060769	5.150726753	up-regulated in High
DHRS12	-0.2783872	2.06959703	-7.1199139	3.82E-12	8.95E-11	15.9722882	11.41828396	up-regulated in High
ATP7B	0.23556318	1.21762895	3.24265494	0.00126402	0.00585131	-2.9806169	2.898244846	up-regulated in Low
NEK5	-0.1846735	0.43919451	-4.2105005	3.03E-05	0.00021199	0.53060993	4.519047819	up-regulated in High
THSD1	-0.2944596	1.13350854	-6.7047121	5.51E-11	1.09E-09	13.3517389	10.25873496	up-regulated in High
VPS36	-0.1149786	3.23077886	-2.7454751	0.0062619	0.02345029	-4.4444052	2.203294029	up-regulated in High
CKAP2	0.79748479	2.67767706	14.6964551	7.64E-41	2.37E-38	81.5443129	40.11675695	up-regulated in Low
LECT1	-0.4509474	0.57147245	-3.1707396	0.00161467	0.00725421	-3.2068205	2.79191664	up-regulated in High
PCDH17	-0.2896235	1.67657473	-4.8527862	1.63E-06	1.49E-05	3.3283122	5.786863967	up-regulated in High
DIAPH3	0.65089035	0.98350652	15.1643514	6.05E-43	2.15E-40	86.366948	42.21854216	up-regulated in Low
TDRD3	-0.1517553	1.73007246	-4.6367469	4.53E-06	3.76E-05	2.34661781	5.343578937	up-regulated in High
DACH1	-0.1123771	0.67011206	-2.159804	0.03126675	0.08964701	-5.865156	1.504917249	up-regulated in High
MZT1	0.57572481	3.37998537	10.4043216	4.60E-23	4.26E-21	40.8224141	22.33699997	up-regulated in Low
BORA	0.55449902	1.40436516	13.4157626	3.15E-35	7.08E-33	68.6663996	34.50138165	up-regulated in Low
PIBF1	-0.1165898	2.45808154	-2.6790475	0.00762866	0.02765176	-4.6221606	2.117551722	up-regulated in High
KLF12	-0.137612	0.97891606	-3.1733791	0.00160035	0.00719986	-3.1986044	2.795784456	up-regulated in High
COMMD6	-0.1070483	4.26589766	-2.3149581	0.02102293	0.0647295	-5.521062	1.677306842	up-regulated in High
UCHL3	0.30600237	2.31799502	6.79392559	3.14E-11	6.45E-10	13.9038346	10.50325161	up-regulated in Low
LMO7	-0.4323888	3.94359092	-5.6163923	3.25E-08	4.08E-07	7.11967661	7.487600404	up-regulated in High
KCTD12	-0.6181125	4.13849762	-7.4343156	4.65E-13	1.22E-11	18.0416954	12.33226303	up-regulated in High
CLN5	-0.3297043	2.17001745	-8.0035608	8.65E-15	2.90E-13	21.9681677	14.06312631	up-regulated in High
FBXL3	-0.3020477	2.93915183	-7.3735273	7.03E-13	1.81E-11	17.6359745	12.1531766	up-regulated in High
MYCBP2	-0.1749393	2.17523291	-3.3396725	0.000902	0.00434315	-2.6677586	3.044794258	up-regulated in High
SCEL	-0.6488905	3.28652308	-4.885804	1.39E-06	1.29E-05	3.48193242	5.856101855	up-regulated in High
SLAIN1	-0.3871211	2.07057547	-5.3023259	1.73E-07	1.90E-06	5.50045119	6.763106541	up-regulated in High
EDNRB	-0.6896429	1.79974573	-9.2172825	8.69E-19	4.93E-17	31.064367	18.06082444	up-regulated in High
POU4F1	0.15144914	0.24726439	3.68435788	0.00025454	0.00142968	-1.4852157	3.594252126	up-regulated in Low
RNF219	0.1609563	1.92643774	4.52178537	7.68E-06	6.09E-05	1.84093256	5.11462262	up-regulated in Low
NDFIP2	0.18901966	3.26723186	3.40923266	0.00070459	0.00349732	-2.4380185	3.152062696	up-regulated in Low
SPRY2	-0.5810067	3.36247426	-9.1277122	1.77E-18	9.68E-17	30.3612318	17.75227185	up-regulated in High
GPC5	-0.1853947	0.42434198	-3.2783185	0.00111763	0.00524517	-2.8666368	2.951701556	up-regulated in High
GPC6	0.50987802	1.65967628	5.41537885	9.55E-08	1.11E-06	6.07379463	7.019916961	up-regulated in Low
GPR180	0.15373271	1.77791701	3.5625901	0.0004027	0.00214037	-1.9155804	3.395019204	up-regulated in Low
SOX21	0.37972186	0.86748366	3.77197448	0.00018153	0.00105956	-1.1670853	3.741061448	up-regulated in Low
ABCC4	-0.3478829	2.4534789	-4.151898	3.88E-05	0.00026591	0.29363924	4.410931778	up-regulated in High

DNAJC3	-0.2061858	4.61589946	-3.8191899	0.00015088	0.00089983	-0.9927224	3.821371046	up-regulated in High
MBNL2	-0.4052848	3.83155321	-8.1792756	2.42E-15	8.83E-14	23.2254743	14.61659766	up-regulated in High
IPO5	0.30944835	3.88907274	6.40260664	3.55E-10	6.19E-09	11.5276462	9.44989834	up-regulated in Low
STK24	0.20263336	2.87482074	3.97365431	8.13E-05	0.00051863	-0.4080379	4.089941234	up-regulated in Low
SLC15A1	0.38043723	0.65055041	4.78943409	2.21E-06	1.96E-05	3.03621121	5.655118732	up-regulated in Low
DOCK9	-0.5546805	2.7816945	-8.5698408	1.32E-16	5.71E-15	26.0939228	15.87812064	up-regulated in High
GPR18	-0.1492526	0.62089326	-3.5402179	0.0004375	0.0023017	-1.9931572	3.359022576	up-regulated in High
GPR183	-0.5607678	3.38715065	-5.8075599	1.13E-08	1.54E-07	8.1453958	7.945387287	up-regulated in High
TM9SF2	-0.1644181	6.07323038	-3.2562771	0.00120613	0.00561267	-2.9372215	2.918606499	up-regulated in High
ZIC5	0.14466455	0.14655814	4.85195027	1.64E-06	1.50E-05	3.32443521	5.785116145	up-regulated in Low
ZIC2	0.22536717	0.49451366	3.5412247	0.00043588	0.00229398	-1.9896762	3.360638397	up-regulated in Low
PCCA	-0.2637834	1.94594476	-4.6288426	4.70E-06	3.89E-05	2.31147567	5.327682126	up-regulated in High
NALCN	-0.199482	0.45558848	-6.6657862	7.03E-11	1.37E-09	13.1127528	10.15285067	up-regulated in High
ITGBL1	-0.6667186	1.99345002	-8.0865794	4.75E-15	1.65E-13	22.5595893	14.3235146	up-regulated in High
FGF14	-0.1612988	0.29732498	-6.7146993	5.18E-11	1.03E-09	13.4132425	10.28598053	up-regulated in High
TPP2	-0.1056801	3.01564466	-2.4159113	0.01605649	0.05180935	-5.2846352	1.794349449	up-regulated in High
TEX30	0.50696137	2.0589972	9.76172801	1.05E-20	7.39E-19	35.4408878	19.979958	up-regulated in Low
KDELC1	0.13852607	1.6700544	2.32761541	0.02033432	0.06294554	-5.4919603	1.691770444	up-regulated in Low
BIVM	-0.1142653	1.57833111	-2.5387197	0.01143072	0.03899764	-4.9837476	1.941926276	up-regulated in High
ERCC5	-0.2139237	2.08289306	-5.3811454	1.14E-07	1.30E-06	5.89904472	6.941677515	up-regulated in High
SLC10A2	-0.1666106	0.14167047	-2.6907993	0.00736888	0.02683842	-4.5910214	2.132598567	up-regulated in High
EFNB2	-0.1796756	3.40012028	-1.9782575	0.04845304	0.12731898	-6.2380255	1.314678969	up-regulated in High
ARGLU1	-0.267369	3.98606427	-3.8597111	0.00012854	0.00078007	-0.8414505	3.890961385	up-regulated in High
LIG4	-0.1991808	2.16027549	-4.5632655	6.36E-06	5.13E-05	2.0220472	5.19667664	up-regulated in High
ABHD13	-0.1225481	2.36463328	-3.177014	0.00158083	0.0071274	-3.1872788	2.801115339	up-regulated in High
TNFSF13B	-0.2406608	2.66971536	-2.8512861	0.00453596	0.01776264	-4.1525496	2.34333093	up-regulated in High
RAB20	-0.3374254	4.53084092	-5.377743	1.16E-07	1.32E-06	5.88173084	6.933924105	up-regulated in High
ANKRD10	-0.2938319	4.15098566	-4.8057861	2.05E-06	1.82E-05	3.11127159	5.688984804	up-regulated in High
ARHGEF7	-0.1047741	2.44017069	-2.6494819	0.00831901	0.0297533	-4.6999158	2.079928283	up-regulated in High
TUBGCP3	0.11272639	2.2872612	2.84596619	0.00461117	0.01801248	-4.1674784	2.33618871	up-regulated in Low
ATP11A	-0.7319451	4.32201871	-7.1313582	3.54E-12	8.33E-11	16.0463448	11.45101598	up-regulated in High
F7	0.20334915	0.27735071	4.00237517	7.23E-05	0.00046635	-0.2969231	4.140862703	up-regulated in Low
F10	-0.2647937	1.12076453	-4.1383561	4.11E-05	0.00028005	0.23932012	4.38612952	up-regulated in High
PCID2	0.10759823	2.77596526	2.25618523	0.0244941	0.07348744	-5.6541567	1.61093859	up-regulated in Low
TFDP1	0.43694534	4.4189749	7.94446998	1.32E-14	4.33E-13	21.5500742	13.87900319	up-regulated in Low
TMEM255B	-0.1567031	0.94518881	-3.8986052	0.00011007	0.00067884	-0.6948392	3.958336232	up-regulated in High
GAS6	-0.7275154	3.93270017	-8.8056368	2.19E-17	1.03E-15	27.8736924	16.66011723	up-regulated in High
CHAMP1	0.10223133	2.67979777	2.15444951	0.03168622	0.09058893	-5.8766132	1.49912961	up-regulated in Low
AL589743.1	0.14652908	0.21660844	5.21652179	2.68E-07	2.85E-06	5.07250965	6.57120652	up-regulated in Low
TTC5	0.10294785	1.51542903	2.77477927	0.00573256	0.02173846	-4.3646472	2.241651235	up-regulated in Low
CCNB1IP1	0.23683454	3.22171621	4.01584423	6.84E-05	0.00044394	-0.2445559	4.164849142	up-regulated in Low
PARP2	0.35457063	2.65179283	7.4191077	5.16E-13	1.35E-11	17.9399429	12.28735373	up-regulated in Low
TEP1	-0.281636	2.42053487	-5.6590903	2.58E-08	3.28E-07	7.34616232	7.588753914	up-regulated in High
APEX1	0.30918313	6.28700739	6.5229013	1.70E-10	3.13E-09	12.245504	9.768396279	up-regulated in Low
PNP	0.50457716	3.87319486	8.27774577	1.17E-15	4.46E-14	23.939161	14.93061902	up-regulated in Low
RNASE4	-0.2220478	0.87591916	-4.9879751	8.46E-07	8.17E-06	3.96328105	6.072842078	up-regulated in High
ANG	-0.3812595	2.99765649	-4.0543524	5.84E-05	0.00038454	-0.0939286	4.23379993	up-regulated in High
RNASE6	-0.6421785	3.85805609	-7.9287522	1.48E-14	4.81E-13	21.4392677	13.83019878	up-regulated in High
RNASE1	-1.6221933	7.92892725	-13.439622	2.49E-35	5.61E-33	68.9015409	34.60395908	up-regulated in High
RNASE2	-0.1592974	1.39909463	-2.1434399	0.03256393	0.09250317	-5.9000832	1.487263217	up-regulated in High
NDRG2	-0.762334	2.4636934	-10.065586	8.27E-22	6.67E-20	37.9574072	21.08250721	up-regulated in High
ARHGEF40	-0.3441675	2.26429911	-5.9453974	5.21E-09	7.50E-08	8.90353585	8.283251839	up-regulated in High
ZNF219	-0.1953154	2.18465481	-2.9141751	0.00372754	0.01502209	-3.9740251	2.428577236	up-regulated in High
HNRNPC	0.37015045	5.74522506	11.6533287	6.65E-28	9.50E-26	51.889468	27.17696524	up-regulated in Low
SUPT16H	0.34672963	4.21905491	7.14766915	3.18E-12	7.52E-11	16.1520614	11.49773799	up-regulated in Low
SALL2	-0.3692399	2.01901137	-4.6527262	4.21E-06	3.52E-05	2.41782847	5.375785279	up-regulated in High
DAD1	0.24616668	7.31486219	5.40399097	1.01E-07	1.17E-06	6.01555419	6.993844653	up-regulated in Low
SLC7A7	-0.195308	2.84546637	-2.5707962	0.01043728	0.03605925	-4.9027642	1.981412794	up-regulated in High
MRPL52	0.40929254	3.69476993	7.97855024	1.03E-14	3.44E-13	21.7909149	13.98507124	up-regulated in Low
MMP14	0.22130232	5.97325406	2.30667588	0.02148446	0.06591768	-5.5400203	1.66787549	up-regulated in Low
LRP10	-0.1146213	5.44529224	-2.1081624	0.03551839	0.09925871	-5.9744917	1.449546732	up-regulated in High
PRMT5	0.47033754	4.04476831	9.27646901	5.42E-19	3.13E-17	31.5316493	18.26584272	up-regulated in Low
PSMB5	0.54876309	6.03436778	11.357576	9.95E-27	1.26E-24	49.2012647	26.00208017	up-regulated in Low
CDH24	0.50722389	2.07195976	6.8493379	2.21E-11	4.63E-10	14.2497992	10.65641139	up-regulated in Low
SLC7A8	-0.6291973	2.83814974	-6.1789129	1.35E-09	2.15E-08	10.2229694	8.870356427	up-regulated in High
BCL2L2	-0.1333561	3.40467653	-2.6901452	0.00738313	0.0268702	-4.5927581	2.131759684	up-regulated in High
CL2L2-PABP	0.14406982	0.31759165	7.90350519	1.77E-14	5.68E-13	21.2616378	13.75195621	up-regulated in Low
PABPN1	0.18744918	4.47185498	3.39965713	0.00072914	0.00360386	-2.4699125	3.137187608	up-regulated in Low
SLC22A17	-0.4555863	2.13429813	-5.2798773	1.94E-07	2.12E-06	5.38788774	6.712648793	up-regulated in High
NGDN	0.20422359	2.84454293	4.83495346	1.78E-06	1.61E-05	3.24573654	5.749632679	up-regulated in Low
AP1G2	-0.1319062	2.94603833	-1.9911543	0.04701253	0.12430017	-6.2125992	1.327786378	up-regulated in High

JPH4	-0.1490702	0.26858565	-7.6694822	9.21E-14	2.70E-12	19.6361942	13.03562601	up-regulated in High
LRRC16B	0.10141946	0.39453105	2.61207275	0.00927259	0.03267147	-4.7970968	2.032798745	up-regulated in Low
PCK2	0.11016359	3.40660028	2.05897546	0.04001862	0.10940607	-6.0762135	1.39773795	up-regulated in Low
EMC9	0.4876215	3.08685914	9.09017888	2.38E-18	1.28E-16	30.0680443	17.6235942	up-regulated in Low
PSME2	0.33240057	5.39583591	5.77037199	1.40E-08	1.86E-07	7.94350835	7.855347022	up-regulated in Low
IPO4	0.30803225	1.21388267	8.5436384	1.61E-16	6.88E-15	25.8983529	15.79215848	up-regulated in Low
TM9SF1	0.12505203	1.9343215	3.95864528	8.64E-05	0.00054623	-0.4658059	4.063453136	up-regulated in Low
TSSK4	-0.1161233	0.80488498	-3.1764373	0.00158391	0.00713854	-3.1890766	2.800269199	up-regulated in High
NEDD8	0.26145481	4.24399767	6.01860614	3.43E-09	5.11E-08	9.31246919	8.465330758	up-regulated in Low
DHRS1	-0.2110768	2.62710011	-4.4272861	1.17E-05	8.97E-05	1.43401364	4.93004508	up-regulated in High
NOP9	0.13332706	3.09043265	2.89140575	0.00400369	0.01596744	-4.0390959	2.397539748	up-regulated in Low
ADCY4	-0.2184181	1.00431267	-5.1937032	3.02E-07	3.17E-06	4.95975612	6.520612328	up-regulated in High
RIPK3	-0.441622	2.26409092	-8.2996584	9.98E-16	3.81E-14	24.0988586	15.00087192	up-regulated in High
NYNRIN	-0.3765194	2.61584645	-4.4301261	1.16E-05	8.87E-05	1.44612736	4.935544452	up-regulated in High
CBLN3	-0.149244	1.50677727	-2.8683237	0.00430252	0.01698677	-4.1045565	2.36627708	up-regulated in High
CMA1	-0.2020153	0.25805799	-6.6515865	7.69E-11	1.49E-09	13.0258628	10.11434732	up-regulated in High
CTSG	-0.5655147	0.79996633	-8.6114725	9.66E-17	4.22E-15	26.4055665	16.01508898	up-regulated in High
GZMB	0.6900948	2.66084807	5.95020545	5.07E-09	7.32E-08	8.9302602	8.295154308	up-regulated in Low
STXBP6	-0.1307464	0.58868974	-2.4907058	0.01307528	0.04373182	-5.1031165	1.883549	up-regulated in High
FOXG1	0.16716819	0.09231018	5.22149634	2.62E-07	2.79E-06	5.09714927	6.582260842	up-regulated in Low
PRKD1	-0.1550751	1.45907223	-2.408001	0.01640441	0.05271061	-5.3035169	1.785039336	up-regulated in High
G2E3	0.38778542	1.68445365	7.88065355	2.08E-14	6.62E-13	21.1012403	13.68129787	up-regulated in Low
SCFD1	0.20165093	3.2674748	4.79567617	2.15E-06	1.91E-05	3.06483663	5.668035059	up-regulated in Low
COCH	0.60602095	1.13150077	6.36206963	4.53E-10	7.77E-09	11.28829	9.343643785	up-regulated in Low
STRN3	0.16261627	3.17641426	3.19595003	0.00148263	0.00674087	-3.1280773	2.828967421	up-regulated in Low
AP4S1	-0.1004076	1.33690977	-2.5671397	0.01054649	0.03637416	-4.9120457	1.976891937	up-regulated in High
DTD2	0.15273962	2.75205873	2.93088394	0.00353607	0.01434154	-3.9259606	2.451478685	up-regulated in Low
AKAP6	-0.136089	0.50743467	-3.9240352	9.94E-05	0.00062067	-0.5982336	4.002693884	up-regulated in High
EGLN3	0.95303592	2.68136854	7.59846513	1.51E-13	4.26E-12	19.1505312	12.82146485	up-regulated in Low
SPTSSA	0.27359942	5.57917596	3.13317167	0.00183167	0.00809705	-3.3230503	2.737152051	up-regulated in Low
SNX6	0.26448447	4.31853622	4.32439472	1.85E-05	0.00013574	0.99999506	4.732812277	up-regulated in Low
CFL2	0.18970693	2.20769081	3.19962133	0.00146426	0.0066698	-3.1165603	2.834383093	up-regulated in Low
BAZ1A	0.15787363	3.44698103	2.54093009	0.01135966	0.0388028	-4.9781988	1.944634755	up-regulated in Low
SRP54	0.25208838	4.46677802	4.27939372	2.25E-05	0.00016217	0.8131448	4.647777783	up-regulated in Low
PPP2R3C	0.13492119	2.78162124	2.51328906	0.01227728	0.04140674	-5.0472483	1.910897864	up-regulated in Low
PSMA6	0.34044215	2.87302237	5.27309487	2.01E-07	2.19E-06	5.35396276	6.697438994	up-regulated in Low
NFKBIA	-0.2054516	6.48912202	-2.7069571	0.0070247	0.02581677	-4.5479914	2.153372353	up-regulated in High
MBIP	-0.8301531	4.418992	-9.1807633	1.16E-18	6.47E-17	30.7770996	17.93477265	up-regulated in High
SFTA3	-1.7454561	4.4203955	-12.731139	2.54E-32	4.87E-30	62.0054201	31.59478855	up-regulated in High
NKX2-1	-1.4451908	5.28295417	-10.32029	9.48E-23	8.47E-21	40.1059478	22.02335165	up-regulated in High
NKX2-8	-0.1518365	0.86018865	-2.0124574	0.04471224	0.11953907	-6.1702434	1.349573539	up-regulated in High
PAX9	0.39813508	1.30982391	4.08144077	5.22E-05	0.00034772	0.0128352	4.282634153	up-regulated in Low
SLC25A21	0.25153709	0.39821034	5.14611199	3.84E-07	3.96E-06	4.72602025	6.415686621	up-regulated in Low
FOXA1	0.4012216	4.10969238	3.76161772	0.00018899	0.00109907	-1.2050582	3.723557382	up-regulated in Low
CLEC14A	-0.5881362	3.35536089	-8.3885782	5.16E-16	2.07E-14	24.7501643	15.28733815	up-regulated in High
SEC23A	0.2855948	3.07601018	4.49325479	8.74E-06	6.84E-05	1.71724342	5.058550748	up-regulated in Low
GEMIN2	0.40410185	2.24186127	6.76206049	3.84E-11	7.79E-10	13.7059418	10.41562187	up-regulated in Low
PNN	0.22024491	4.49381368	3.37009786	0.00081005	0.00395098	-2.5678276	3.091487706	up-regulated in Low
C14orf28	-0.1913386	1.08285887	-6.994387	8.67E-12	1.93E-10	15.166376	11.0619576	up-regulated in High
FKBP3	0.40118405	4.4894887	7.32422464	9.80E-13	2.47E-11	17.3088759	12.00875823	up-regulated in Low
FANCM	0.23343901	0.81158572	8.04149108	6.58E-15	2.24E-13	22.2377995	14.18184802	up-regulated in Low
MIS18BP1	0.2614799	2.36934954	5.19434579	3.01E-07	3.16E-06	4.96292511	6.522034492	up-regulated in Low
LRR1	0.57191756	2.30179939	11.4900634	2.98E-27	3.97E-25	50.4005489	26.52628266	up-regulated in Low
MGAT2	0.10593822	1.24051632	2.50775394	0.01246876	0.04195317	-5.060987	1.904176823	up-regulated in Low
DNAAF2	0.20436019	2.7385257	4.17286274	3.55E-05	0.0002453	0.3780587	4.44946328	up-regulated in Low
POLE2	0.77083552	1.55273763	14.2156839	1.04E-38	2.86E-36	76.6514003	37.98376711	up-regulated in Low
KLHDC1	-0.3125168	0.94907602	-10.079844	7.33E-22	5.96E-20	38.076749	21.13477814	up-regulated in High
KLHDC2	-0.2113795	2.88434644	-4.3485789	1.66E-05	0.0001234	1.10115967	4.778820038	up-regulated in High
ARF6	0.14086403	5.36074866	3.11289299	0.00195969	0.00858817	-3.3852363	2.70781345	up-regulated in Low
SOS2	-0.1584267	2.88163806	-3.7216119	0.00022064	0.00126161	-1.3508127	3.656321667	up-regulated in High
L2HGDH	0.24763993	1.37122273	6.40509409	3.50E-10	6.10E-09	11.5423756	9.456436039	up-regulated in Low
ATP5S	-0.1140741	1.64503945	-3.2581966	0.00119817	0.00558321	-2.9310927	2.921481272	up-regulated in High
CDKL1	-0.1332621	0.44588393	-5.2261728	2.55E-07	2.73E-06	5.1203313	6.592660626	up-regulated in High
PYGL	0.59228267	3.6917881	6.72193339	4.95E-11	9.84E-10	13.4578397	10.30573569	up-regulated in Low
TRIM9	0.20805192	0.35471244	4.09358489	4.96E-05	0.0003323	0.06091477	4.304615983	up-regulated in Low
TMX1	0.21137959	3.86548666	4.0409276	6.17E-05	0.00040338	-0.1465934	4.209699366	up-regulated in Low
GNG2	-0.3340995	1.77976723	-6.4399878	2.83E-10	5.03E-09	11.7495091	9.548361257	up-regulated in High
C14orf166	0.2238585	3.79074242	5.76485075	1.44E-08	1.92E-07	7.91363102	7.84201939	up-regulated in Low
PTGER2	-0.4505778	1.6097407	-5.5655899	4.28E-08	5.28E-07	6.85217061	7.368071635	up-regulated in High
ERO1L	0.66644246	4.7539293	8.2118098	1.91E-15	7.06E-14	23.460556	14.72004472	up-regulated in Low
PSMC6	0.24473721	3.45984755	5.46723809	7.26E-08	8.58E-07	6.34039141	7.139222903	up-regulated in Low

STYX	0.11569763	2.82892491	2.77365848	0.00575204	0.02180099	-4.3677128	2.240178194	up-regulated in Low
GNPNAT1	0.75080538	3.37030736	12.4594287	3.45E-31	6.09E-29	59.4104519	30.46197283	up-regulated in Low
BMP4	-0.5500981	2.26762016	-5.1338272	4.09E-07	4.19E-06	4.66599956	6.388732926	up-regulated in High
CDKN3	1.32962881	2.30741425	17.2462444	1.50E-52	9.83E-50	108.420698	51.82427399	up-regulated in Low
CNIH1	0.22240938	4.17805017	4.09125607	5.01E-05	0.00033512	0.05168445	4.300396385	up-regulated in Low
GMFB	0.21686024	3.2172224	4.43191642	1.15E-05	8.80E-05	1.45376734	4.939012696	up-regulated in Low
SAMD4A	-0.325193	1.62150074	-5.1408325	3.94E-07	4.05E-06	4.70021009	6.404096468	up-regulated in High
GCH1	0.46586168	2.88849511	7.26390012	1.47E-12	3.64E-11	16.9110565	11.833071	up-regulated in Low
WDHD1	0.68934301	1.73682262	12.0484101	1.69E-29	2.64E-27	55.5412855	28.77235448	up-regulated in Low
SOCS4	0.1295263	2.34426946	3.25743903	0.00120131	0.00559376	-2.9335121	2.920346486	up-regulated in Low
MAPK1IP1L	0.13915293	3.96223973	3.43903736	0.00063302	0.00318127	-2.3381981	3.198584797	up-regulated in Low
LGALS3	-0.4544358	7.0756272	-5.7309966	1.74E-08	2.28E-07	7.73098156	7.760528758	up-regulated in High
DLGAP5	1.35337833	2.21346793	18.1185251	1.14E-56	1.02E-53	117.884164	55.94398983	up-regulated in Low
AL158801.1	0.20933613	1.08627375	5.46566358	7.32E-08	8.65E-07	6.33226403	7.135586756	up-regulated in Low
FBXO34	-0.1909428	3.76174922	-3.9556812	8.75E-05	0.00055248	-0.47719	4.058232082	up-regulated in High
KTN1	0.17526678	3.64594188	3.30460872	0.00101996	0.00484638	-2.7818493	2.99141723	up-regulated in Low
PELI2	-0.2647681	1.80779139	-3.6395721	0.00030177	0.0016597	-1.6450965	3.520325306	up-regulated in High
EXOC5	0.18992163	2.54933291	4.50923111	8.13E-06	6.41E-05	1.78641698	5.089912628	up-regulated in Low
C14orf105	-0.1380761	0.32254661	-2.3211106	0.0206857	0.06383931	-5.5069356	1.684329795	up-regulated in High
PSMA3	0.32473317	4.89975287	7.05333278	5.91E-12	1.35E-10	15.5433628	11.22866739	up-regulated in Low
ARID4A	-0.2936306	2.08413507	-7.3781278	6.81E-13	1.75E-11	17.6665867	12.16669066	up-regulated in High
TIMM9	0.31456518	3.99446629	6.67559374	6.61E-11	1.29E-09	13.1728568	10.17948247	up-regulated in Low
DAAM1	-0.1481689	2.19168384	-3.1823017	0.00155282	0.00702716	-3.1707812	2.808879144	up-regulated in High
L3HYPDH	0.12834587	1.66998813	2.5012971	0.01269547	0.04262509	-5.0769762	1.89635125	up-regulated in Low
RTN1	-0.3177343	0.98943544	-6.0560793	2.76E-09	4.18E-08	9.52346114	8.559233474	up-regulated in High
DHRS7	-0.2137776	4.6149353	-3.4312936	0.00065093	0.00325962	-2.3642127	3.186465292	up-regulated in High
PPM1A	-0.110382	2.71899985	-3.0161177	0.00269183	0.0112981	-3.6766508	2.569952274	up-regulated in High
SIX1	-0.3651389	2.28724738	-3.3159789	0.00098022	0.00467814	-2.7449791	3.008674837	up-regulated in High
MNAT1	0.21346395	2.42281786	5.29714714	1.77E-07	1.95E-06	5.4744457	6.751450465	up-regulated in Low
TRMT5	0.1317026	2.22744074	3.51452804	0.00048093	0.00250266	-2.0816654	3.317920826	up-regulated in Low
SLC38A6	-0.1108241	1.80233613	-2.6392134	0.00857159	0.03053876	-4.7267249	2.066938769	up-regulated in High
PRKCH	-0.2458346	2.26552956	-5.4610247	7.50E-08	8.85E-07	6.30833116	7.124878952	up-regulated in High
TMEM30B	-0.3157311	4.18609795	-4.7460524	2.72E-06	2.36E-05	2.8382073	5.56574199	up-regulated in High
HIF1A	0.26622227	5.41628922	3.81194496	0.00015524	0.00092311	-1.0196102	3.808993686	up-regulated in Low
SNAPC1	0.19779503	2.44268772	4.09310327	4.97E-05	0.00033286	0.05900545	4.303743163	up-regulated in Low
RHOJ	-0.3554396	1.85107139	-6.7444115	4.29E-11	8.66E-10	13.5966682	10.367227	up-regulated in High
SYNE2	-0.1585598	2.33589033	-2.4397693	0.01504623	0.04909763	-5.2273211	1.822572432	up-regulated in High
MTHFD1	0.42306349	3.08551305	9.44464916	1.40E-19	8.72E-18	32.8708789	18.85327016	up-regulated in Low
HSPA2	0.19749531	1.55398758	2.54058368	0.01137077	0.0388294	-4.9790688	1.944210161	up-regulated in Low
PLEKHG3	0.1628613	1.87743978	2.50743748	0.01247978	0.0419833	-5.0617716	1.903792914	up-regulated in Low
SPTB	-0.2767188	1.19634456	-2.6326233	0.00873729	0.03104954	-4.7438772	2.058623391	up-regulated in High
CHURC1	-0.2265521	3.15419886	-4.7525816	2.64E-06	2.29E-05	2.86790285	5.579150021	up-regulated in High
GPX2	1.11949896	3.48710207	3.99935392	7.32E-05	0.00047163	-0.308647	4.135491596	up-regulated in Low
FUT8	-0.2399858	3.27489292	-3.2812649	0.00110627	0.00519894	-2.8571666	2.95613964	up-regulated in High
EIF2S1	0.37579524	3.76229765	8.9791299	5.69E-18	2.90E-16	29.2056645	17.24503248	up-regulated in Low
PLEK2	0.4922483	3.38034395	5.38273544	1.13E-07	1.29E-06	5.90713961	6.945302425	up-regulated in Low
PLEKHH1	0.39971056	0.73743222	7.12188418	3.77E-12	8.85E-11	15.9850314	11.42391639	up-regulated in Low
ARG2	0.29199742	1.71511996	3.56778747	0.000395	0.00210382	-1.8974917	3.403408767	up-regulated in Low
RDH11	0.26148564	3.80178855	5.24739739	2.29E-07	2.47E-06	5.22578022	6.639959226	up-regulated in Low
ZFYVE26	-0.1037389	1.84890555	-2.5885803	0.00992034	0.03459883	-4.8574379	2.003473396	up-regulated in High
ZFP36L1	-0.6239927	6.43820867	-8.3581119	6.47E-16	2.55E-14	24.526421	15.18893742	up-regulated in High
ACTN1	0.19746804	4.24708523	2.78449359	0.00556623	0.02121243	-4.3380262	2.254438618	up-regulated in Low
DCAF5	-0.1697474	3.19153019	-4.2371798	2.70E-05	0.00019099	0.6395175	4.568690752	up-regulated in High
GALNT16	-0.1081705	0.29456778	-3.4892276	0.00052761	0.00271033	-2.1682323	3.277685833	up-regulated in High
ERH	0.38690186	6.06072765	8.03403554	6.94E-15	2.36E-13	22.1847235	14.15847934	up-regulated in Low
SUSD6	-0.4562718	4.09501176	-8.7260453	4.03E-17	1.83E-15	27.268975	16.39447421	up-regulated in High
SRSF5	-0.2373354	4.92296683	-4.9380957	1.08E-06	1.02E-05	3.72716137	5.966561305	up-regulated in High
SMOC1	0.68535737	1.48806863	4.48854712	8.93E-06	6.97E-05	1.69690351	5.04932734	up-regulated in Low
SYNJ2BP	-0.1374394	2.68283104	-3.4827986	0.00054013	0.0027654	-2.1901345	3.267500441	up-regulated in High
MED6	0.1948006	2.55615198	6.04301908	2.98E-09	4.48E-08	9.44979754	8.526452424	up-regulated in Low
PCNX	-0.1304227	2.01821284	-3.0810551	0.00217738	0.0094122	-3.4820858	2.662066291	up-regulated in High
RGS6	-0.1110622	0.15612838	-4.2249532	2.85E-05	0.00020028	0.58952791	4.545907649	up-regulated in High
ZFYVE1	-0.1943618	2.47480524	-5.3243516	1.54E-07	1.71E-06	5.61130862	6.812786893	up-regulated in High
PAPLN	-0.5652158	2.08240028	-8.1383923	3.26E-15	1.16E-13	22.9310734	14.48703135	up-regulated in High
NUMB	-0.1263476	3.20440319	-3.6834426	0.00025543	0.00143386	-1.4885015	3.592733798	up-regulated in High
C14orf169	0.12142037	2.8811133	3.17498662	0.00159169	0.00716807	-3.1935971	2.798141447	up-regulated in Low
ACOT1	-0.2086369	1.5470178	-3.9613063	8.55E-05	0.00054115	-0.4555791	4.068143113	up-regulated in High
PNMA1	0.29535916	3.91099781	4.87573816	1.46E-06	1.35E-05	3.43499939	5.834952253	up-regulated in Low
FAM161B	-0.2437844	1.162848	-6.7760103	3.52E-11	7.17E-10	13.7924798	10.45394409	up-regulated in High
CCDC176	-0.2372248	1.58436118	-4.730778	2.92E-06	2.52E-05	2.76888322	5.534435735	up-regulated in High
ALDH6A1	-0.3012573	2.73931555	-4.7082021	3.25E-06	2.77E-05	2.66679424	5.488319455	up-regulated in High

ABCD4	-0.2037264	2.33071484	-5.2414414	2.36E-07	2.54E-06	5.19615101	6.62667039	up-regulated in High
SYNDIG1L	-0.2723152	0.35006723	-6.2771079	7.54E-10	1.24E-08	10.7908157	9.12270964	up-regulated in High
NPC2	-1.2398745	7.4201988	-12.946707	3.15E-33	6.19E-31	64.084334	32.50212473	up-regulated in High
LTBP2	-0.9867254	4.41400946	-10.605892	8.02E-24	7.95E-22	42.5561368	23.09579561	up-regulated in High
EIF2B2	0.16498464	2.68377704	4.57085286	6.14E-06	4.97E-05	2.05534045	5.211753757	up-regulated in Low
ACYP1	0.35948179	1.87198895	7.04175782	6.37E-12	1.45E-10	15.4691309	11.19584489	up-regulated in Low
NEK9	-0.1746211	3.09039054	-4.2585766	2.46E-05	0.00017578	0.72732329	4.6086948	up-regulated in High
FOS	-0.865026	6.09273651	-6.984378	9.25E-12	2.05E-10	15.1026209	11.03375894	up-regulated in High
JDP2	-0.3662392	2.45484279	-7.5936562	1.56E-13	4.39E-12	19.1177734	12.80701749	up-regulated in High
BATF	-0.2016381	3.68675763	-2.2248915	0.02653801	0.07840847	-5.7236553	1.576131679	up-regulated in High
FLVCR2	-0.196927	2.02573848	-3.181289	0.00155815	0.00704472	-3.1739429	2.807391372	up-regulated in High
TTLL5	0.11402307	1.66341729	3.33432568	0.00091913	0.00441485	-2.6852304	3.036624808	up-regulated in Low
TGFB3	-0.248197	2.39847385	-3.6377077	0.0003039	0.00166963	-1.6517122	3.517264104	up-regulated in High
VASH1	-0.2876704	2.21393364	-4.8449708	1.70E-06	1.54E-05	3.29208846	5.770532792	up-regulated in High
CIPC	-0.1009	2.70065367	-2.4101425	0.01630957	0.05246099	-5.298411	1.787557534	up-regulated in High
TMEM63C	-0.2605747	1.25146557	-2.6830656	0.00753893	0.02737068	-4.6115286	2.122690568	up-regulated in High
GSTZ1	0.11819053	1.65906365	2.3105041	0.02127004	0.06535252	-5.5312656	1.672231665	up-regulated in Low
AHSA1	0.34866699	5.02343716	8.56106119	1.41E-16	6.08E-15	26.0283437	15.84929629	up-regulated in Low
SPTLC2	-0.2533786	3.41488369	-4.7118929	3.19E-06	2.73E-05	2.6834535	5.495845997	up-regulated in High
SLIRP	0.37495507	3.40837651	8.1593059	2.80E-15	1.01E-13	23.0815317	14.55325054	up-regulated in Low
SNW1	0.19870757	4.73382414	5.77476106	1.36E-08	1.82E-07	7.96727697	7.865949215	up-regulated in Low
NRXN3	-0.1676078	0.54074494	-3.6999538	0.00023979	0.00135743	-1.4291054	3.620173154	up-regulated in High
CEP128	0.1697229	0.72977825	5.1525243	3.72E-07	3.85E-06	4.75740053	6.429777028	up-regulated in Low
GTF2A1	0.11551307	3.00641889	2.79038518	0.0054675	0.02087704	-4.3218371	2.262211429	up-regulated in Low
STON2	0.11297076	1.20179951	2.17816048	0.0298648	0.08635677	-5.8256661	1.524840437	up-regulated in Low
GALC	-0.3030495	2.78281247	-5.7416811	1.64E-08	2.16E-07	7.78852499	7.786204992	up-regulated in High
GPR65	-0.2718155	1.20377513	-5.2302171	2.50E-07	2.68E-06	5.14039502	6.601661031	up-regulated in High
PTPN21	-0.3946339	1.74061701	-7.6704925	9.15E-14	2.68E-12	19.6431289	13.03868351	up-regulated in High
TTC8	0.15057912	1.91659914	4.18278556	3.41E-05	0.00023612	0.4181535	4.46775755	up-regulated in Low
FOXN3	-0.2990514	2.57532804	-5.5520098	4.61E-08	5.65E-07	6.78102634	7.336272282	up-regulated in High
EFCAB11	0.18385358	0.77182118	5.59734869	3.61E-08	4.49E-07	7.01914947	7.442689257	up-regulated in Low
TDP1	0.39020763	1.75708979	7.71764445	6.58E-14	1.96E-12	19.9675848	13.18172276	up-regulated in Low
PSMC1	0.28016323	2.01016107	7.41868073	5.17E-13	1.35E-11	17.9370885	12.28609391	up-regulated in Low
C14orf159	-0.3315378	2.56709938	-5.8631281	8.30E-09	1.15E-07	8.44917363	8.080814022	up-regulated in High
GPR68	-0.4403173	2.27524633	-5.1604345	3.57E-07	3.71E-06	4.79615928	6.447178986	up-regulated in High
CCDC88C	-0.1532499	2.45138338	-2.4467548	0.01476126	0.04829481	-5.2104357	1.830876716	up-regulated in High
SMEK1	0.11457466	3.87932142	2.72434191	0.00667061	0.02471144	-4.5014145	2.175834368	up-regulated in Low
CATSPERB	0.12497301	0.69648493	2.00605077	0.0453938	0.12090173	-6.183028	1.343003488	up-regulated in Low
FBLN5	-0.8240765	3.11747245	-9.6317699	3.05E-20	2.04E-18	34.3805111	19.51518146	up-regulated in High
NDUFB1	0.14224028	4.08796064	2.52191045	0.01198424	0.04056296	-5.0257904	1.921389521	up-regulated in Low
CPSF2	0.30252703	2.60136146	8.38095758	5.46E-16	2.18E-14	24.6941411	15.2627004	up-regulated in Low
RIN3	-0.2876136	2.28412867	-5.3357786	1.45E-07	1.62E-06	5.66898307	6.838628583	up-regulated in High
LGMN	-0.3783126	5.85171053	-6.159685	1.51E-09	2.39E-08	10.1126753	8.821319871	up-regulated in High
GOLGA5	0.19161982	4.51226282	4.8070454	2.03E-06	1.81E-05	3.11706186	5.691596941	up-regulated in Low
CHGA	0.21064199	0.31738188	2.23585135	0.02580596	0.07668188	-5.6994234	1.588279971	up-regulated in Low
ITPK1	0.11989091	3.31605928	2.36593419	0.01836851	0.05788681	-5.4029118	1.735926106	up-regulated in Low
MOAP1	-0.3601346	3.80272538	-6.9028857	1.57E-11	3.36E-10	14.5863292	10.80534893	up-regulated in High
UBR7	0.14003942	3.48823262	3.36060768	0.00083775	0.00406733	-2.5990906	3.076885637	up-regulated in Low
ASB2	-0.101846	0.71380933	-2.3941285	0.01703063	0.05436376	-5.3364838	1.768769213	up-regulated in High
OTUB2	0.23515161	0.94691226	5.89605034	6.89E-09	9.70E-08	8.6303387	8.161547843	up-regulated in Low
DDX24	-0.1237452	4.13685426	-3.1379103	0.00180289	0.00798506	-3.308463	2.744030267	up-regulated in High
IFI27L1	0.13731724	2.14027072	2.61954666	0.00907461	0.03206676	-4.7777886	2.042172233	up-regulated in Low
IFI27L2	-0.2385694	3.35212503	-3.0070621	0.00277183	0.01158887	-3.7034658	2.557234136	up-regulated in High
PPP4R4	-0.1164459	0.36730879	-3.0878713	0.00212898	0.00922965	-3.4614317	2.67182801	up-regulated in High
SERPINA1	-0.8192322	6.78027998	-4.924914	1.15E-06	1.08E-05	3.66512112	5.938623941	up-regulated in High
SERPINA5	0.20687052	0.65179071	2.40985974	0.01632207	0.05249512	-5.2990855	1.787224902	up-regulated in Low
SYNE3	-0.1661665	0.59049738	-5.2845646	1.89E-07	2.07E-06	5.41135598	6.723169758	up-regulated in High
GLRX5	0.33138943	3.7737388	8.65184756	7.11E-17	3.15E-15	26.7088692	16.14837555	up-regulated in Low
TCL1A	-0.1615279	0.57882547	-2.6745551	0.00773012	0.02796745	-4.6340292	2.111813651	up-regulated in High
BDKRB1	0.2354588	0.54467289	5.04430768	6.40E-07	6.32E-06	4.23252758	6.193946743	up-regulated in Low
ATG2B	-0.1295239	1.74999642	-3.2779775	0.00111895	0.00525011	-2.8677323	2.951188151	up-regulated in High
GSKIP	0.26743556	3.02559499	4.94987864	1.02E-06	9.70E-06	3.7827458	5.991587201	up-regulated in Low
AK7	-0.1464168	0.90753473	-2.3824313	0.01757491	0.05579915	-5.3641366	1.755106887	up-regulated in High
PAPOLA	0.23673987	4.03825787	5.48656699	6.55E-08	7.82E-07	6.44033301	7.183931387	up-regulated in Low
VRK1	0.67554318	2.48390648	13.7768637	8.66E-37	2.14E-34	72.2455543	36.06254798	up-regulated in Low
BCL11B	-0.1768136	0.88690644	-3.497021	0.0005128	0.00264401	-2.14163	3.290053864	up-regulated in High
CCNK	0.15710675	1.28616599	5.01675022	7.34E-07	7.17E-06	4.10047301	6.134561143	up-regulated in Low
CCDC85C	0.21483781	1.18720026	5.71148497	1.93E-08	2.52E-07	7.62614058	7.713741531	up-regulated in Low
EML1	-0.2067687	1.17941777	-4.11001	4.63E-05	0.00031255	0.12615538	4.334434037	up-regulated in High
EVL	-0.3074844	2.93032281	-5.0307533	6.84E-07	6.72E-06	4.16749375	6.164703412	up-regulated in High
YY1	0.13825364	3.90752903	4.61130026	5.10E-06	4.19E-05	2.23368024	5.292483363	up-regulated in Low

SLC25A29	-0.4980592	2.73408644	-6.1110341	2.01E-09	3.11E-08	9.83492324	8.697800096	up-regulated in High
WARS	0.33958959	5.34873167	3.60162626	0.00034811	0.00188032	-1.7791087	3.458280339	up-regulated in Low
PPP2R5C	-0.1355695	3.42412058	-3.7280641	0.00021522	0.00123388	-1.3274048	3.667124839	up-regulated in High
DYNC1H1	0.14698948	4.62880475	2.59513473	0.00973567	0.03408506	-4.840656	2.01163415	up-regulated in Low
HSP90AA1	0.44735826	7.62465684	8.15720325	2.84E-15	1.03E-13	23.0663914	14.54658725	up-regulated in Low
AMN	-0.2617586	1.3852963	-2.7144208	0.00687066	0.02534957	-4.5280302	2.163001794	up-regulated in High
CDC42BPB	0.12371692	4.04836712	2.16070474	0.03119666	0.08949879	-5.8632259	1.505891913	up-regulated in Low
EXOC3L4	-0.1098898	0.47856245	-2.6182006	0.00910998	0.03217245	-4.7812699	2.040482539	up-regulated in High
CKB	-0.3520773	4.89115136	-2.7415496	0.00633606	0.02368853	-4.4550271	2.198180539	up-regulated in High
TRMT61A	0.17854308	2.98891915	3.437084	0.00063749	0.00320045	-2.3447655	3.195525516	up-regulated in Low
BAG5	0.10667211	3.4356727	2.85564347	0.00447519	0.01755999	-4.1403016	2.349188928	up-regulated in Low
APOPT1	0.15983179	2.54215467	3.8832953	0.00011702	0.00071708	-0.7527149	3.931747804	up-regulated in Low
XRCC3	0.30641057	1.50943513	6.84445263	2.28E-11	4.77E-10	14.2192047	10.64286902	up-regulated in Low
ZFYVE21	-0.1467473	2.60524827	-4.0542297	5.84E-05	0.00038464	-0.0944106	4.233579398	up-regulated in High
PPP1R13B	-0.4656918	2.75617351	-7.2191923	1.98E-12	4.81E-11	16.6179386	11.70359044	up-regulated in High
C14orf2	0.38196121	4.01984366	7.83831953	2.81E-14	8.78E-13	20.805049	13.55080359	up-regulated in Low
ADSSL1	0.24251714	1.77754998	3.01651952	0.00268833	0.01128666	-3.675459	2.570517384	up-regulated in Low
SIVA1	0.23396775	3.62933736	4.05065855	5.93E-05	0.00038953	-0.1084357	4.227161933	up-regulated in Low
PLD4	-0.5153267	0.82451957	-11.217498	3.53E-26	4.31E-24	47.9421635	25.45163616	up-regulated in High
CDCA4	0.83066942	3.04530105	13.6771816	2.35E-36	5.66E-34	71.2532152	35.62974753	up-regulated in Low
GPR132	-0.406414	1.76521917	-6.1175141	1.93E-09	3.01E-08	9.87180874	8.714206193	up-regulated in High
MTA1	0.19271728	3.09706146	3.4408384	0.00062892	0.0031642	-2.3321396	3.2014068	up-regulated in Low
CRIP2	-0.444219	4.5974708	-4.5000243	8.48E-06	6.66E-05	1.74652623	5.071828066	up-regulated in High
CRIP1	-0.4188003	2.02128595	-6.0338614	3.14E-09	4.70E-08	9.39822744	8.50350117	up-regulated in High
CRIP1	-0.4188003	2.02128595	-6.0338614	3.14E-09	4.70E-08	9.39822744	8.50350117	up-regulated in High
C14orf80	0.38435618	1.39768226	8.06747466	5.45E-15	1.87E-13	22.4230725	14.2634164	up-regulated in Low
NIPA2	0.20282576	3.64604017	4.37459796	1.48E-05	0.00011114	1.21058349	4.828559544	up-regulated in Low
MKRN3	0.14735057	0.17725472	5.02972584	6.88E-07	6.75E-06	4.16257044	6.162489364	up-regulated in Low
NDN	-0.5762532	3.08104223	-6.9700439	1.02E-11	2.24E-10	15.0114466	10.99343022	up-regulated in High
SNRPN	-0.3042696	4.00623848	-3.6397535	0.00030156	0.00165919	-1.6444527	3.520623202	up-regulated in High
UBE3A	0.11494331	2.81470628	3.20208361	0.00145205	0.00662351	-3.1088289	2.838018164	up-regulated in Low
ATP10A	-0.4695708	1.78540541	-6.0544554	2.79E-09	4.21E-08	9.51429453	8.555154424	up-regulated in High
ARHGAP11E	0.34771744	0.59470963	12.3973398	6.24E-31	1.09E-28	58.8215549	30.20485327	up-regulated in Low
MTMR10	-0.2750206	1.81482068	-7.3022174	1.14E-12	2.85E-11	17.1634385	11.94453509	up-regulated in High
KLF13	-0.3164266	3.37079882	-5.0620375	5.86E-07	5.83E-06	4.31783356	6.23229785	up-regulated in High
ARHGAP11A	1.08940294	1.7606228	19.6758236	4.07E-64	8.49E-61	134.9946	63.3902642	up-regulated in Low
GREM1	0.28637349	1.86304351	2.41710468	0.01600457	0.05167323	-5.2817814	1.795756029	up-regulated in Low
FMN1	-0.3651308	1.07947822	-6.9749239	9.84E-12	2.17E-10	15.0424692	11.00715265	up-regulated in High
AVEN	0.32742788	2.80752242	7.13802581	3.39E-12	7.98E-11	16.0895362	11.47010512	up-regulated in Low
EMC7	0.14119803	5.21625947	3.19426728	0.00149112	0.00677421	-3.1333519	2.826486833	up-regulated in Low
KATNBL1	0.11247995	2.09021432	2.24700928	0.02507874	0.07493717	-5.6746337	1.600694288	up-regulated in Low
EMC4	0.24322341	4.03574839	6.29670018	6.71E-10	1.12E-08	10.9050287	9.173444711	up-regulated in Low
SLC12A6	-0.164108	2.14775664	-3.6824431	0.0002564	0.0014386	-1.4920893	3.591075918	up-regulated in High
NOP10	0.3858328	6.63655212	7.44446451	4.34E-13	1.15E-11	18.109692	12.36227216	up-regulated in Low
LPCAT4	0.13657938	2.95009858	2.10533932	0.03576443	0.09982208	-5.9803938	1.44654865	up-regulated in Low
GOLGA8A	-0.3616158	1.884877	-3.7876068	0.00017078	0.00100407	-1.1095831	3.767558015	up-regulated in High
GOLGA8B	-0.2320832	1.68733394	-2.811107	0.0051327	0.01977712	-4.2646336	2.289654551	up-regulated in High
AQR	0.12810643	2.2245097	3.66558609	0.00027342	0.00152183	-1.5524546	3.563174017	up-regulated in Low
ZNF770	0.24817081	2.27542449	4.19987885	3.17E-05	0.00022108	0.48742978	4.49935738	up-regulated in Low
DPH6	0.15629945	0.83924695	6.73298812	4.61E-11	9.24E-10	13.5260675	10.33595683	up-regulated in Low
C15orf41	0.23201433	0.75269239	9.36697365	2.62E-19	1.57E-17	32.2502532	18.5810725	up-regulated in Low
SPRED1	-0.4798713	3.30328329	-7.6891562	8.03E-14	2.37E-12	19.7713686	13.09522235	up-regulated in High
FAM98B	0.23763258	2.41956214	6.33462286	5.35E-10	9.06E-09	11.1269596	9.272009524	up-regulated in Low
RASGRP1	-0.4078814	1.76954806	-6.0069584	3.66E-09	5.43E-08	9.24711731	8.436239926	up-regulated in High
THBS1	-0.2729957	4.94818786	-2.3918491	0.01713551	0.05462688	-5.3418827	1.766102858	up-regulated in High
SRP14	0.10138507	6.48180688	2.69143015	0.00735516	0.02680125	-4.589346	2.133407812	up-regulated in Low
BMF	-0.3601735	3.17330496	-5.0807881	5.33E-07	5.35E-06	4.40834407	6.272979119	up-regulated in High
BUB1B	1.27664194	1.94941766	20.9331979	3.50E-70	2.04E-66	148.93728	69.45622688	up-regulated in Low
PLCB2	-0.4965071	2.3289145	-6.614801	9.66E-11	1.84E-09	12.8014878	10.01490515	up-regulated in High
C15orf52	-0.230241	1.60841657	-3.3786649	0.00078578	0.00384701	-2.5395334	3.104698634	up-regulated in High
KNSTRN	0.93666456	2.31850275	19.3108551	2.31E-62	3.85E-59	130.965159	61.63693279	up-regulated in Low
IVD	-0.4342181	3.53336277	-7.0222048	7.24E-12	1.63E-10	15.3439612	11.14049513	up-regulated in High
BAHD1	-0.1465262	3.12822006	-3.012056	0.00272745	0.01142779	-3.6886875	2.564244063	up-regulated in High
RPUSD2	0.19399138	2.24079708	5.94991746	5.08E-09	7.33E-08	8.92865896	8.294441162	up-regulated in Low
CASC5	0.70880108	0.96393757	17.720153	8.77E-55	6.57E-52	113.54917	54.05697756	up-regulated in Low
RAD51	0.99575138	1.60310349	20.6436026	8.78E-69	4.66E-65	145.719548	68.05642123	up-regulated in Low
RMDN3	-0.1671844	3.45317789	-4.3980312	1.34E-05	0.00010095	1.30964966	4.873569474	up-regulated in High
DNAJC17	-0.1115062	1.8331913	-3.2145943	0.00139148	0.0063756	-3.0694582	2.856523243	up-regulated in High
C15orf62	-0.2299621	1.12218851	-5.0865047	5.18E-07	5.22E-06	4.43599852	6.285406854	up-regulated in High
RHOV	1.08791696	3.20107868	7.39647832	6.02E-13	1.56E-11	17.7888437	12.22065928	up-regulated in Low
VPS18	0.15301972	3.4629953	3.1400899	0.00178979	0.00793415	-3.3017463	2.747196853	up-regulated in Low

DLL4	-0.2062657	2.49378453	-3.1621553	0.00166205	0.00743789	-3.2334963	2.779355745	up-regulated in High
CHAC1	0.47345914	1.72379019	7.04544052	6.22E-12	1.42E-10	15.4927378	11.20628317	up-regulated in Low
OIP5	1.11431262	1.50238437	21.2438015	1.10E-71	9.13E-68	152.391396	70.95879857	up-regulated in Low
NUSAP1	1.34865729	3.31264807	20.0537556	6.17E-66	1.71E-62	139.176746	65.20990834	up-regulated in Low
RPAP1	0.20188195	2.31403398	5.04188071	6.48E-07	6.39E-06	4.22087138	6.188705762	up-regulated in Low
MGA	0.10502141	1.81443109	2.57417004	0.0103374	0.03579254	-4.8941886	1.985588758	up-regulated in Low
MAPKBP1	-0.1373213	1.4668677	-3.5219094	0.00046805	0.00244351	-2.0562975	3.329704899	up-regulated in High
1JD7-PLA2G	-0.2072514	0.71042396	-5.1268142	4.23E-07	4.33E-06	4.63179351	6.373370043	up-regulated in High
SPTBN5	-0.1159509	0.57850371	-2.9536845	0.00328927	0.01345551	-3.8599444	2.482900754	up-regulated in High
EHD4	-0.1255271	3.41912322	-2.9722881	0.00309967	0.01277477	-3.8057142	2.508685151	up-regulated in High
PLA2G4E	-0.2112499	0.60366378	-3.1363945	0.00181205	0.00802125	-3.3131315	2.741829126	up-regulated in High
PLA2G4F	-0.7006065	1.42125996	-8.1530889	2.93E-15	1.05E-13	23.0367743	14.53355256	up-regulated in High
VPS39	-0.1515908	3.32676011	-3.5513782	0.0004198	0.00221919	-1.9545165	3.376955697	up-regulated in High
TMEM87A	-0.2556371	4.39080204	-5.0945088	4.98E-07	5.03E-06	4.47476627	6.302827305	up-regulated in High
GANC	-0.417677	1.61409029	-9.8511664	4.99E-21	3.72E-19	36.1762364	20.30219935	up-regulated in High
CAPN3	-0.3245577	0.50916235	-8.2713947	1.23E-15	4.64E-14	23.8929348	14.91028264	up-regulated in High
ZNF106	-0.1306395	2.63696087	-2.415914	0.01605637	0.05180935	-5.2846288	1.794352615	up-regulated in High
HAUS2	0.27495198	2.49490387	7.52451829	2.51E-13	6.87E-12	18.648635	12.60007846	up-regulated in Low
STARD9	-0.2055498	0.53614823	-6.5043066	1.91E-10	3.48E-09	12.1338037	9.718853813	up-regulated in High
TTBK2	-0.1347775	0.9622328	-5.1752183	3.31E-07	3.47E-06	4.8687412	6.479762357	up-regulated in High
CCNDBP1	-0.2389296	2.99078077	-5.355696	1.31E-07	1.47E-06	5.76977324	6.883780801	up-regulated in High
ZSCAN29	0.14103221	2.07013713	3.65006052	0.00029002	0.0016034	-1.6078201	3.537570515	up-regulated in Low
TUBGCP4	0.13834626	1.28861916	4.57042573	6.15E-06	4.98E-05	2.05346486	5.210904435	up-regulated in Low
TP53BP1	0.10371228	2.13383431	2.26461083	0.02396773	0.07213422	-5.6352822	1.620373106	up-regulated in Low
MAP1A	-0.1434646	1.42170859	-2.051331	0.04075986	0.11104688	-6.0918108	1.389767358	up-regulated in High
CKMT1B	0.28870386	0.78852491	4.40799004	1.28E-05	9.71E-05	1.35189949	4.892759262	up-regulated in Low
CKMT1A	0.27864346	0.74210724	4.60253314	5.31E-06	4.35E-05	2.19490199	5.27493418	up-regulated in Low
PDIA3	0.23539169	7.26280731	3.6845189	0.00025438	0.00142894	-1.4846375	3.594519288	up-regulated in Low
SERF2	0.13612595	5.83817946	2.34613902	0.01936215	0.06046736	-5.4490911	1.713046427	up-regulated in Low
MFAP1	0.17915368	3.8825926	4.43301432	1.15E-05	8.77E-05	1.45845392	4.941140159	up-regulated in Low
WDR76	0.73479198	1.79296637	15.0097259	3.01E-42	1.02E-39	84.766911	41.52127773	up-regulated in Low
FRMD5	0.15565434	1.03303551	2.65567004	0.00817005	0.02929586	-4.6837109	2.087775484	up-regulated in Low
CTDSPL2	0.18473848	2.21300501	5.27497504	1.99E-07	2.17E-06	5.36336328	6.70165371	up-regulated in Low
EIF3J	0.4124539	4.27261445	10.8152557	1.28E-24	1.36E-22	44.3789748	23.89333727	up-regulated in Low
PATL2	-0.1613194	0.59958394	-4.1564564	3.81E-05	0.00026124	0.31196085	4.419295802	up-regulated in High
B2M	-0.2172807	9.52585012	-2.8306576	0.00483397	0.01875184	-4.2102872	2.315696231	up-regulated in High
TRIM69	0.17074847	2.24689527	3.15032351	0.00172946	0.00769939	-3.2701501	2.762088677	up-regulated in Low
SORD	0.3273257	2.25341587	5.53129137	5.15E-08	6.26E-07	6.67278039	7.287881203	up-regulated in Low
DUOX2	-0.3030927	0.7378862	-3.1991082	0.00146681	0.00667936	-3.1181708	2.833625809	up-regulated in High
DUOXA1	-0.8581703	1.85283028	-8.7972799	2.33E-17	1.10E-15	27.8100101	16.63214533	up-regulated in High
DUOX1	-0.8954619	2.02512693	-8.3887429	5.15E-16	2.07E-14	24.7513754	15.28787075	up-regulated in High
SHF	-0.2708664	1.36570021	-4.1087875	4.65E-05	0.0003139	0.1212914	4.332211354	up-regulated in High
SPATA5L1	0.29557271	1.84255949	9.00876277	4.51E-18	2.33E-16	29.4350422	17.34573358	up-regulated in Low
C15orf48	0.62150756	4.60912301	3.90413093	0.00010766	0.00066594	-0.6738978	3.967954169	up-regulated in Low
SEMA6D	-0.2232814	0.61440579	-4.3802356	1.45E-05	0.00010863	1.23437229	4.839369638	up-regulated in High
DUT	0.38797145	3.5395718	9.6900685	1.89E-20	1.29E-18	34.8549941	19.72316867	up-regulated in Low
FBN1	-0.3895696	2.81588354	-4.0990234	4.85E-05	0.00032548	0.08248951	4.314477898	up-regulated in High
CEP152	0.29978651	0.79218705	10.3231608	9.25E-23	8.29E-21	40.1303595	22.03403912	up-regulated in Low
EID1	-0.1808575	5.77381715	-4.2586206	2.46E-05	0.00017577	0.72750442	4.608777307	up-regulated in High
SECISBP2L	-0.3593681	3.09565967	-5.3599896	1.28E-07	1.44E-06	5.79154474	6.893532732	up-regulated in High
COPS2	0.21253936	3.16973868	5.4516851	7.88E-08	9.26E-07	6.26020034	7.103343157	up-regulated in Low
GALK2	0.11139683	1.68917175	3.30348587	0.00102396	0.00486145	-2.7854838	2.989715632	up-regulated in Low
FGF7	-0.3824976	1.31443128	-5.8191488	1.06E-08	1.45E-07	8.20854114	7.973543482	up-regulated in High
ATP8B4	-0.158566	0.50636603	-6.318722	5.88E-10	9.89E-09	11.0337667	9.230623525	up-regulated in High
SLC27A2	0.27295782	2.24711628	2.81542983	0.00506524	0.01954686	-4.2526484	2.295400148	up-regulated in Low
HDC	-0.3920183	0.72715519	-7.5416512	2.23E-13	6.16E-12	18.7645738	12.65122525	up-regulated in High
GABPB1	0.15090714	1.88992626	6.78111612	3.40E-11	6.95E-10	13.8241908	10.46798614	up-regulated in Low
TNFAIP8L3	-0.168217	1.44196628	-2.8694212	0.00428787	0.0169411	-4.1014556	2.367758907	up-regulated in High
GLDN	-0.3040614	0.77980045	-5.9728459	4.45E-09	6.49E-08	9.05635196	8.351306548	up-regulated in High
SCG3	0.38084125	0.36048874	4.4319782	1.15E-05	8.80E-05	1.45403105	4.939132408	up-regulated in Low
LYSMD2	-0.1052959	2.70226505	-2.0401573	0.04186428	0.11334183	-6.1145063	1.37815633	up-regulated in High
TMOD2	-0.1442843	0.97727598	-3.779809	0.00017606	0.0010316	-1.1382947	3.754329394	up-regulated in High
TMOD3	0.13564381	3.34185723	2.90740038	0.00380784	0.0152957	-3.9934377	2.419321816	up-regulated in Low
LEO1	0.32775642	3.19709838	8.95576125	6.83E-18	3.44E-16	29.0251597	17.16578213	up-regulated in Low
MAPK6	0.59147996	3.20511611	11.3623661	9.53E-27	1.21E-24	49.2444833	26.02097236	up-regulated in Low
BCL2L10	0.30788302	0.52648932	5.5045951	5.95E-08	7.15E-07	6.53383022	7.225748847	up-regulated in Low
MYO5C	-0.3076518	2.79835943	-4.4120794	1.26E-05	9.54E-05	1.36927419	4.900649769	up-regulated in High
MYO5A	-0.117896	1.87368582	-2.2951019	0.02214426	0.06750331	-5.5664024	1.654738902	up-regulated in High
ARPP19	0.18281646	4.53966419	4.70400308	3.31E-06	2.82E-05	2.64785524	5.479762403	up-regulated in Low
FAM214A	-0.2969634	2.43823811	-6.2588605	8.40E-10	1.38E-08	10.6847151	9.07557183	up-regulated in High
WDR72	0.64107337	0.89578465	6.40523361	3.49E-10	6.10E-09	11.5432019	9.456802781	up-regulated in Low

RSL24D1	0.25201699	4.71372316	4.42429199	1.19E-05	9.09E-05	1.42125063	4.924250622	up-regulated in Low
RAB27A	-0.5440263	3.69908284	-8.7813399	2.64E-17	1.23E-15	27.6886646	16.57884356	up-regulated in High
CCPG1	-0.3138383	2.2360804	-7.3095504	1.08E-12	2.72E-11	17.2118607	11.96591841	up-regulated in High
NEDD4	0.15498276	1.40764265	3.23453246	0.00129976	0.00600055	-3.0064091	2.886137347	up-regulated in Low
TEX9	-0.2016454	0.85858847	-4.1240813	4.36E-05	0.00029594	0.18224089	4.360058922	up-regulated in High
MNS1	0.28275208	1.29566844	4.79161842	2.19E-06	1.94E-05	3.04622444	5.659637027	up-regulated in Low
CGNL1	-0.7348767	2.23269974	-10.387773	5.31E-23	4.90E-21	40.6810259	22.27510776	up-regulated in High
MYZAP	-0.2272616	0.75572692	-4.9434707	1.05E-06	9.98E-06	3.7525023	5.97797111	up-regulated in High
ADAM10	0.17253867	3.04155821	3.80287983	0.00016087	0.00095124	-1.0531855	3.79353436	up-regulated in Low
FAM63B	-0.1562517	2.02013708	-3.834192	0.00014221	0.00085355	-0.9368924	3.847063702	up-regulated in High
CCNB2	1.45185871	2.78384677	21.2322375	1.25E-71	9.13E-68	152.262751	70.90283822	up-regulated in Low
MYO1E	0.28103084	3.00559418	4.05234617	5.89E-05	0.00038722	-0.1018093	4.230194021	up-regulated in Low
FAM81A	0.19403409	0.79081136	4.41723466	1.23E-05	9.35E-05	1.39119856	4.910605589	up-regulated in Low
GTF2A2	0.34790669	3.62016816	7.71028636	6.93E-14	2.06E-12	19.9168504	13.15935782	up-regulated in Low
BNIP2	-0.1090721	3.01740826	-2.7898944	0.00547566	0.02090411	-4.3231869	2.261563454	up-regulated in High
RORA	-0.2983262	1.03729843	-7.2142942	2.04E-12	4.96E-11	16.5859142	11.68944241	up-regulated in High
VPS13C	-0.3277188	2.43381221	-6.2008575	1.18E-09	1.90E-08	10.3492073	8.926472863	up-regulated in High
C2CD4A	0.36499896	1.88093952	2.94564535	0.00337443	0.01376724	-3.8832771	2.471799244	up-regulated in Low
TLN2	-0.101923	0.90059565	-2.6128226	0.00925256	0.03261071	-4.7951621	2.033738197	up-regulated in High
TPM1	-0.1759806	3.67476544	-2.4427788	0.01492286	0.04875196	-5.2200521	1.826147893	up-regulated in High
RPS27L	-0.1567993	2.65810419	-2.9680364	0.00314209	0.01292994	-3.818137	2.502780767	up-regulated in High
RAB8B	-0.1464951	2.82729978	-2.4549096	0.01443463	0.04740926	-5.1906642	1.840594476	up-regulated in High
APH1B	-0.2060081	1.88605215	-4.0755429	5.35E-05	0.00035575	-0.0104668	4.271978326	up-regulated in High
CA12	0.51021952	2.1100069	3.47494935	0.00055579	0.00283752	-2.2168234	3.255086024	up-regulated in Low
HERC1	-0.2144204	1.99921065	-4.6217966	4.86E-06	4.01E-05	2.28019632	5.313530931	up-regulated in High
DAPK2	-0.5302524	1.20900762	-9.9838847	1.64E-21	1.29E-19	37.2757073	20.78390059	up-regulated in High
FAM96A	0.24680313	4.07232065	5.39907893	1.04E-07	1.20E-06	5.9904664	6.98261269	up-regulated in Low
SNX1	-0.2158439	3.58453663	-5.9941799	3.94E-09	5.81E-08	9.17554689	8.404377807	up-regulated in High
SNX22	-0.1752829	1.00108662	-3.9626627	8.50E-05	0.00053857	-0.4503635	4.070534851	up-regulated in High
PPIB	0.24631149	7.1474749	4.24131233	2.65E-05	0.00018799	0.65644401	4.576403803	up-regulated in Low
CSNK1G1	0.10591002	1.95054845	2.87173403	0.00425713	0.01683903	-4.0949168	2.370883346	up-regulated in Low
KIAA0101	1.24691137	2.32308651	20.0627187	5.58E-66	1.63E-62	139.276036	65.25310762	up-regulated in Low
TRIP4	0.17060923	3.2463159	4.87613069	1.46E-06	1.35E-05	3.43682798	5.835776333	up-regulated in Low
OAZ2	-0.1146492	3.56378743	-3.513162	0.00048335	0.00251346	-2.0863547	3.315742198	up-regulated in High
RBPMS2	0.50338242	1.57523165	6.29285027	6.86E-10	1.14E-08	10.8825617	9.163465101	up-regulated in Low
PIF1	0.62434336	0.87339317	13.7034886	1.80E-36	4.37E-34	71.5147876	35.74383313	up-regulated in Low
PLEKHO2	-0.3082385	3.71369957	-4.9009659	1.29E-06	1.21E-05	3.55279269	5.88802812	up-regulated in High
RASL12	-0.4871505	2.30989627	-7.4135894	5.36E-13	1.40E-11	17.9030625	12.27107552	up-regulated in High
UBAP1L	-0.1019117	0.42712876	-4.1830816	3.40E-05	0.00023591	0.41935119	4.468303966	up-regulated in High
CLPX	0.1132567	3.22855552	3.0340323	0.00253978	0.01075657	-3.6233739	2.595204296	up-regulated in Low
CILP	-0.3969591	1.66439484	-3.3311796	0.00092935	0.00445901	-2.6954984	3.031822968	up-regulated in High
PARP16	-0.118756	2.17509276	-3.3524263	0.00086233	0.00417661	-2.6259744	3.064324788	up-regulated in High
PTPLAD1	0.19334257	4.18592776	3.66099463	0.00027823	0.00154568	-1.5688513	3.555592706	up-regulated in Low
VWA9	0.19646525	3.49981319	6.01331242	3.53E-09	5.25E-08	9.28275413	8.452103692	up-regulated in Low
DENND4A	-0.1006651	1.79240707	-2.7686489	0.00583983	0.02208641	-4.3814003	2.233600027	up-regulated in High
RAB11A	0.15421145	4.7066431	3.73828842	0.00020688	0.00119101	-1.2902335	3.684275946	up-regulated in Low
MEGF11	-0.301986	0.30869851	-5.4923273	6.35E-08	7.60E-07	6.47017746	7.197280435	up-regulated in High
TIPIN	0.4719992	1.6575673	14.0605962	4.99E-38	1.30E-35	75.087482	37.30186673	up-regulated in Low
MAP2K1	0.21447212	4.19020094	5.32809193	1.51E-07	1.68E-06	5.63017446	6.821240314	up-regulated in Low
SNAPC5	0.21846206	3.15363859	5.70250383	2.03E-08	2.64E-07	7.57798791	7.692249657	up-regulated in Low
RPL4	0.21019887	7.58838552	4.21109533	3.02E-05	0.00021148	0.53303126	4.520151827	up-regulated in Low
ZWILCH	0.66185214	2.07484507	16.4112758	1.18E-48	5.60E-46	99.4728214	47.92791354	up-regulated in Low
SMAD6	-0.332375	1.24019183	-7.0425821	6.34E-12	1.44E-10	15.4744139	11.19818087	up-regulated in High
AAGAB	0.35284376	3.82822509	8.93258288	8.18E-18	4.07E-16	28.8464598	17.08731949	up-regulated in Low
CLN6	0.26116202	3.51586169	5.63952402	2.87E-08	3.63E-07	7.24218867	7.542322177	up-regulated in Low
FEM1B	0.13619883	3.23222095	3.46963305	0.00056664	0.00288495	-2.234867	3.246691012	up-regulated in Low
CORO2B	-0.3635553	0.76821907	-8.0424283	6.53E-15	2.23E-13	22.2444743	14.1847868	up-regulated in High
ANP32A	0.26622404	4.07477262	7.21565611	2.03E-12	4.92E-11	16.5948165	11.6933754	up-regulated in Low
SPESP1	0.16466403	0.66825927	2.03867422	0.04201277	0.1136572	-6.1175095	1.376618682	up-regulated in Low
GLCE	0.12917783	2.75670765	2.27310796	0.02344689	0.07082221	-5.6161777	1.629914822	up-regulated in Low
KIF23	1.2139018	1.80719384	21.2708062	8.14E-72	7.92E-68	152.691825	71.08948499	up-regulated in Low
LARP6	0.29537819	1.05043462	5.28811629	1.86E-07	2.04E-06	5.42915084	6.73114693	up-regulated in Low
THAP10	0.19017658	0.95662251	5.68694882	2.21E-08	2.85E-07	7.49474646	7.655092404	up-regulated in Low
THSD4	-0.1653149	1.18076845	-3.0080235	0.00276323	0.01155956	-3.7006224	2.558582979	up-regulated in High
MYO9A	-0.2529498	1.49006498	-6.8190547	2.68E-11	5.55E-10	14.060439	10.57258692	up-regulated in High
SENP8	-0.1149115	0.76520137	-4.8040424	2.06E-06	1.84E-05	3.10325637	5.685368858	up-regulated in High
GRAMD2	-0.6692004	2.11530505	-7.6963065	7.64E-14	2.26E-12	19.8205633	13.11691039	up-regulated in High
PKM	0.41207582	7.25492675	8.04549559	6.39E-15	2.18E-13	22.2663233	14.19440641	up-regulated in Low
HEXA	-0.1448332	3.13593187	-3.1930199	0.00149745	0.00680029	-3.13726	2.824648801	up-regulated in High
BBS4	-0.1274407	2.43860491	-2.8857207	0.00407548	0.01620727	-4.0552659	2.38982101	up-regulated in High
CD276	0.22350569	4.22173926	4.58965379	5.63E-06	4.59E-05	2.13805764	5.249204466	up-regulated in Low

C15orf59	-0.2714181	0.37906713	-7.0092357	7.87E-12	1.76E-10	15.2610962	11.10384929	up-regulated in High
LOXL1	-0.5183928	2.97220539	-6.5893415	1.13E-10	2.14E-09	12.6468067	9.946337834	up-regulated in High
STOML1	-0.1020577	1.3889858	-2.7516085	0.00614759	0.02307697	-4.4277798	2.211295217	up-regulated in High
ISLR2	-0.1873387	0.40250987	-5.8581782	8.54E-09	1.18E-07	8.42201155	8.06870762	up-regulated in High
ISLR	-0.5239956	4.55788287	-4.8712688	1.49E-06	1.37E-05	3.41418858	5.825573219	up-regulated in High
CCDC33	-0.2065027	0.302768	-4.4474447	1.07E-05	8.26E-05	1.52015222	4.969143911	up-regulated in High
UBL7	0.10694825	4.4005084	2.73161212	0.00652737	0.0242732	-4.4818505	2.185261854	up-regulated in Low
ARID3B	0.13967052	1.25762338	3.47490184	0.00055589	0.00283763	-2.2169847	3.255010955	up-regulated in Low
EDC3	0.22832	2.90683047	7.21060492	2.10E-12	5.07E-11	16.5618046	11.67879087	up-regulated in Low
SCAMP2	-0.2335146	4.84940147	-5.2727663	2.01E-07	2.19E-06	5.35232051	6.696702684	up-regulated in High
FAM219B	-0.1749747	1.73012349	-5.2432877	2.34E-07	2.52E-06	5.20533271	6.630788524	up-regulated in High
COX5A	0.45207233	5.65795299	9.24688333	6.87E-19	3.92E-17	31.2978041	18.16324765	up-regulated in Low
RPP25	0.55603671	2.67439884	8.68815454	5.39E-17	2.42E-15	26.9825049	16.26861178	up-regulated in Low
PPCDC	0.17270903	1.98043461	4.53726971	7.16E-06	5.71E-05	1.90836383	5.145179299	up-regulated in Low
COMMD4	0.26319121	3.05599195	6.55219079	1.42E-10	2.65E-09	12.421993	9.8466625	up-regulated in Low
NEIL1	-0.5122289	1.61988449	-7.6088485	1.40E-13	3.98E-12	19.2213173	12.85268307	up-regulated in High
MAN2C1	-0.1382276	2.69592359	-2.3746502	0.01794539	0.05680242	-5.3824584	1.746047131	up-regulated in High
SNUPN	0.20813291	2.51249062	6.36300342	4.51E-10	7.74E-09	11.2937891	9.346085284	up-regulated in Low
SNX33	-0.2400987	3.02497611	-5.6003421	3.55E-08	4.43E-07	7.03493094	7.449740285	up-regulated in High
ODF3L1	-0.1306158	0.49829092	-3.7088248	0.00023176	0.00131748	-1.3970892	3.634958128	up-regulated in High
FBXO22	0.22911119	1.72378042	7.53772562	2.29E-13	6.30E-12	18.7379908	12.63949841	up-regulated in Low
NRG4	0.17985749	0.3171674	4.61159206	5.09E-06	4.19E-05	2.23497207	5.29306794	up-regulated in Low
ETFA	0.28249529	3.92592986	6.68884506	6.09E-11	1.19E-09	13.2541829	10.21551514	up-regulated in Low
ISL2	0.12590779	0.23386239	4.7754421	2.36E-06	2.08E-05	2.97216906	5.626217297	up-regulated in Low
SCAPER	-0.179408	1.27692902	-5.3741335	1.19E-07	1.35E-06	5.8633733	6.925703014	up-regulated in High
RCN2	0.18416941	2.97173798	3.81674831	0.00015234	0.00090732	-1.0017891	3.817197632	up-regulated in Low
PSTPIP1	-0.2278585	1.45806099	-3.7777132	0.00017751	0.001039	-1.1460019	3.750777862	up-regulated in High
TSPAN3	-0.5177762	5.37252143	-8.7363616	3.73E-17	1.70E-15	27.3471293	16.4288095	up-regulated in High
PEAK1	-0.2958224	1.47425707	-7.1139079	3.97E-12	9.29E-11	15.9334618	11.40112248	up-regulated in High
TBC1D2B	-0.2722127	2.68253543	-5.3632334	1.26E-07	1.42E-06	5.80800337	6.900904601	up-regulated in High
CIB2	0.43515925	1.80676502	5.4753041	6.95E-08	8.25E-07	6.38205937	7.157864002	up-regulated in Low
IDH3A	0.16252866	1.9166278	4.35263748	1.63E-05	0.00012139	1.11818842	4.786562249	up-regulated in Low
CRABP1	0.65224528	0.89606806	5.35564828	1.31E-07	1.47E-06	5.76953157	6.88367255	up-regulated in Low
IREB2	0.15721673	2.57434909	3.85048531	0.00013333	0.00080556	-0.876024	3.875063077	up-regulated in Low
PSMA4	0.41758573	4.12200836	10.3427851	7.81E-23	7.06E-21	40.2973748	22.10715721	up-regulated in Low
CHRNA5	0.44791867	0.99618137	6.0809104	2.39E-09	3.66E-08	9.66389407	8.62171818	up-regulated in Low
CTSH	-1.485283	5.98485586	-13.526521	1.05E-35	2.47E-33	69.7595814	34.97825288	up-regulated in High
RASGRF1	-0.7925504	1.05957836	-9.3880706	2.21E-19	1.34E-17	32.418464	18.65485208	up-regulated in High
MTHFS	0.15918867	2.01483857	3.54081342	0.00043654	0.00229685	-1.9910984	3.359978263	up-regulated in Low
ST20	0.11654042	1.47227942	2.20061656	0.02822375	0.08242384	-5.776911	1.549385366	up-regulated in Low
FAH	-0.1465771	2.31791733	-2.7402189	0.00636138	0.02376189	-4.4586243	2.196448501	up-regulated in High
ARNT2	-0.3566665	1.69239643	-3.9827644	7.83E-05	0.00050127	-0.3728741	4.106059922	up-regulated in High
MESDC2	0.21387851	3.36614307	6.22036617	1.06E-09	1.70E-08	10.4617537	8.976495293	up-regulated in Low
MESDC1	0.15342901	2.76182589	3.62438974	0.00031957	0.00174527	-1.6988755	3.495435586	up-regulated in Low
C15orf26	-0.1972642	0.3181617	-4.5134687	7.98E-06	6.30E-05	1.80480269	5.098246835	up-regulated in High
IL16	-0.4868572	1.46374354	-8.6080133	9.92E-17	4.33E-15	26.3796289	16.00368997	up-regulated in High
FAM154B	-0.4059109	0.69320409	-5.9140785	6.22E-09	8.84E-08	8.72991781	8.205914274	up-regulated in High
GOLGA6L9	-0.1459769	0.61113579	-4.1634268	3.70E-05	0.00025437	0.34001366	4.432100607	up-regulated in High
RPS17	0.17674542	7.12956903	3.1877443	0.00152447	0.00691234	-3.1537731	2.816881314	up-regulated in Low
WHAMM	-0.1413944	1.97960556	-4.1454116	3.99E-05	0.0002724	0.26760054	4.399043369	up-regulated in High
HOMER2	-0.2748691	1.09594528	-4.8659229	1.53E-06	1.41E-05	3.38931908	5.814364219	up-regulated in High
FAM103A1	0.27005704	2.95328338	7.04041642	6.43E-12	1.46E-10	15.4605348	11.19204389	up-regulated in Low
TM6SF1	-0.3156479	1.22188631	-6.2754682	7.61E-10	1.26E-08	10.7812705	9.118469213	up-regulated in High
HDGFRP3	0.17400383	1.31568003	3.1282387	0.00186208	0.00821345	-3.3382133	2.730000812	up-regulated in Low
BNC1	0.11221619	0.14821389	2.32137683	0.02067121	0.06380135	-5.5063234	1.684634036	up-regulated in Low
ADAMTSL3	-0.2919396	0.67818573	-6.4920048	2.06E-10	3.73E-09	12.060053	9.686139848	up-regulated in High
ZSCAN2	0.16289192	1.34887728	4.88275091	1.41E-06	1.31E-05	3.46768786	5.849683098	up-regulated in Low
NMB	0.39696717	3.08949741	4.35322338	1.63E-05	0.00012109	1.12064791	4.787680419	up-regulated in Low
SEC11A	0.11194525	4.40317725	2.72362449	0.0066849	0.02475337	-4.5033423	2.174905162	up-regulated in Low
AKAP13	-0.5401387	3.25865185	-8.6808948	5.69E-17	2.55E-15	26.9277225	16.24454123	up-regulated in High
KLHL25	0.21440036	1.19644202	5.43886	8.44E-08	9.86E-07	6.19422672	7.073820256	up-regulated in Low
MRPL46	0.16421867	1.40256254	5.95081552	5.05E-09	7.30E-08	8.9336524	8.296665091	up-regulated in Low
MRPS11	0.28668023	2.20178949	9.00414885	4.68E-18	2.41E-16	29.3992918	17.33003898	up-regulated in Low
AEN	0.28067176	2.46101382	6.07786103	2.43E-09	3.72E-08	9.64662161	8.614033572	up-regulated in Low
MFGE8	-0.3230018	3.80520131	-4.5630305	6.36E-06	5.13E-05	2.02101668	5.19620993	up-regulated in High
FANCI	1.01479908	2.20227505	19.4402503	5.52E-63	1.01E-59	132.392611	62.25807541	up-regulated in Low
RHCG	0.52845037	0.40373814	6.90491193	1.55E-11	3.32E-10	14.5991057	10.81100254	up-regulated in Low
TICRR	0.65709923	0.71716853	19.165268	1.15E-61	1.68E-58	129.360688	60.93874112	up-regulated in Low
PEX11A	-0.1351478	1.89746419	-2.6758635	0.00770045	0.02787909	-4.6305745	2.113484058	up-regulated in High
WDR93	-0.1354025	0.34706782	-3.9769891	8.02E-05	0.00051218	-0.3951748	4.09583797	up-regulated in High
AP3S2	-0.1304712	1.85352347	-3.8949104	0.00011171	0.00068809	-0.7088262	3.951911466	up-regulated in High

ARPIN	-0.2707054	2.78051451	-5.6827531	2.26E-08	2.90E-07	7.47232754	7.645084132	up-regulated in High
IDH2	0.45068176	5.6792459	6.80622386	2.90E-11	5.99E-10	13.9804173	10.53715922	up-regulated in Low
SEMA4B	0.41259348	4.73358317	5.09703497	4.92E-07	4.97E-06	4.48701292	6.308330017	up-regulated in Low
CIB1	0.12804159	6.79727602	2.04561056	0.04132214	0.11223837	-6.1034451	1.383817178	up-regulated in Low
NGRN	0.19447672	2.57909187	4.89996909	1.30E-06	1.21E-05	3.5481277	5.885926512	up-regulated in Low
IQGAP1	-0.2300848	5.04132393	-4.54435	6.93E-06	5.55E-05	1.93926796	5.159180835	up-regulated in High
BLM	0.70566993	1.17809645	17.1079799	6.67E-52	4.06E-49	106.930845	51.17559588	up-regulated in Low
FURIN	0.42809629	5.84299537	3.93505006	9.51E-05	0.00059593	-0.5562062	4.021982261	up-regulated in Low
FES	-0.1644008	2.79254751	-2.8155883	0.00506278	0.01953867	-4.2522088	2.295610863	up-regulated in High
HDDC3	0.21131648	2.12837544	4.79578558	2.15E-06	1.90E-05	3.06533867	5.668261578	up-regulated in Low
RCCD1	0.53136677	1.85654241	13.2428593	1.74E-34	3.74E-32	66.9682743	33.76053955	up-regulated in Low
PRC1	1.33758246	2.62046998	22.0045176	2.28E-75	4.44E-71	160.859216	74.64210824	up-regulated in Low
VPS33B	0.20435526	2.40075119	6.89276265	1.67E-11	3.57E-10	14.5225434	10.77712272	up-regulated in Low
SLCO3A1	-0.4493677	2.1402446	-7.0223256	7.23E-12	1.63E-10	15.3447335	11.14083665	up-regulated in High
FAM174B	-0.3609184	3.19436659	-4.4372828	1.12E-05	8.62E-05	1.47668488	4.949415641	up-regulated in High
CHD2	-0.1724121	2.95224724	-3.3717386	0.00080535	0.00393094	-2.562414	3.094015741	up-regulated in High
RGMA	-0.3001187	0.73918588	-5.3601031	1.28E-07	1.44E-06	5.79212055	6.893790644	up-regulated in High
MCTP2	-0.2306821	1.85050573	-4.3024735	2.04E-05	0.00014805	0.90874855	4.691296115	up-regulated in High
ARRDC4	-0.4772897	2.58581934	-6.70617	5.46E-11	1.08E-09	13.3607127	10.26271035	up-regulated in High
MEF2A	-0.199561	2.9548974	-5.2201067	2.64E-07	2.80E-06	5.09026418	6.57917198	up-regulated in High
ADAMTS17	-0.1274109	0.34374403	-4.8751792	1.47E-06	1.35E-05	3.43239584	5.833778917	up-regulated in High
ASB7	0.10598837	2.06103005	3.4328234	0.00064736	0.00324383	-2.3590779	3.188857711	up-regulated in Low
ALDH1A3	-0.1805357	1.90926069	-2.0096059	0.04501452	0.12013268	-6.1759387	1.346647373	up-regulated in High
LRRK1	-0.2118451	1.27096323	-4.9498607	1.02E-06	9.70E-06	3.78266131	5.991549161	up-regulated in High
VIMP	0.18590591	4.28412874	3.93601198	9.47E-05	0.00059382	-0.5525307	4.023668864	up-regulated in Low
SNRPA1	0.59970265	3.19386615	14.8481205	1.60E-41	5.14E-39	83.1012324	40.79534703	up-regulated in Low
POLR3K	0.24164423	3.38846918	5.32764965	1.51E-07	1.69E-06	5.62794304	6.820240475	up-regulated in Low
SNRNP25	0.30395036	3.40386985	6.25349779	8.67E-10	1.42E-08	10.6535837	9.061739741	up-regulated in Low
RHBDF1	-0.331084	3.28992003	-5.4203129	9.31E-08	1.08E-06	6.09906217	7.031227396	up-regulated in High
MPG	-0.1559593	4.33606515	-2.6203909	0.00905248	0.03200213	-4.7756042	2.043232364	up-regulated in High
HBA2	-0.6272669	2.47679363	-4.6136721	5.04E-06	4.15E-05	2.24418299	5.297235961	up-regulated in High
HBQ1	0.19659813	0.2715425	3.78141424	0.00017496	0.00102577	-1.1323887	3.75705078	up-regulated in Low
LUC7L	-0.161355	2.87634717	-2.6608562	0.00804705	0.0289241	-4.6701014	2.094363329	up-regulated in High
ITFG3	-0.1187344	3.90121722	-2.0952603	0.03665479	0.10183011	-6.0014023	1.435869226	up-regulated in High
RGS11	-0.3076639	0.74496609	-5.5442907	4.81E-08	5.87E-07	6.74065479	7.318225472	up-regulated in High
MRPL28	0.27893127	4.50060033	6.16240352	1.49E-09	2.35E-08	10.1282511	8.828245292	up-regulated in Low
NME4	0.28954345	4.60329545	4.33268587	1.78E-05	0.00013142	1.03461885	4.748560992	up-regulated in Low
RAB11FIP3	-0.31961	2.98491369	-5.3204624	1.57E-07	1.74E-06	5.59170394	6.804002033	up-regulated in High
PIGQ	-0.279382	3.16105319	-5.0928528	5.02E-07	5.06E-06	4.46674071	6.299221131	up-regulated in High
NHLRC4	-0.3448043	0.79644297	-6.2949948	6.78E-10	1.12E-08	10.8950752	9.169023516	up-regulated in High
RAB40C	-0.190947	3.79470103	-2.8225723	0.00495554	0.01917296	-4.2328068	2.304908886	up-regulated in High
FAM195A	0.14847297	2.78817909	2.29618742	0.02208163	0.06734292	-5.5639335	1.65596885	up-regulated in Low
MSLN	-1.0178752	5.34687973	-3.7049234	0.00023526	0.0013349	-1.4111788	3.628452074	up-regulated in High
JMJD8	-0.2171429	4.20343755	-4.0234461	6.63E-05	0.00043115	-0.2149275	4.178416764	up-regulated in High
FBXL16	-0.3753849	1.61459338	-3.6465236	0.00029393	0.00162257	-1.6204017	3.53175049	up-regulated in High
METRN	-0.321314	2.57162928	-3.9100446	0.00010513	0.00065213	-0.6514554	3.978260023	up-regulated in High
CCDC78	-0.3238498	1.10077925	-3.9732017	8.14E-05	0.00051944	-0.4097831	4.089141181	up-regulated in High
RPUSD1	0.28833812	3.30385768	5.35877512	1.29E-07	1.45E-06	5.78538498	6.890773685	up-regulated in Low
CHTF18	0.48645345	1.91664715	7.58961373	1.60E-13	4.51E-12	19.0902496	12.79487829	up-regulated in Low
LMF1	-0.3857791	1.55807421	-8.2110232	1.92E-15	7.09E-14	23.4548638	14.71753998	up-regulated in High
SOX8	-0.1494527	0.31987993	-3.9236467	9.95E-05	0.00062157	-0.599714	4.002014371	up-regulated in High
TPSB2	-1.1983899	2.9179152	-9.8917703	3.56E-21	2.72E-19	36.5115637	20.44912629	up-regulated in High
TPSAB1	-1.1402649	2.97291275	-10.356205	6.96E-23	6.34E-21	40.4117081	22.15721011	up-regulated in High
TPSD1	-0.5808916	1.03957785	-6.5533713	1.41E-10	2.63E-09	12.42912	9.849822743	up-regulated in High
BAIAP3	-0.3660311	1.8469071	-4.0912593	5.01E-05	0.00033512	0.05169717	4.300402201	up-regulated in High
GNPTG	-0.2969707	4.20446183	-6.1844573	1.30E-09	2.08E-08	10.2548279	8.884519293	up-regulated in High
C16orf91	0.22599798	3.63018181	4.48251745	9.17E-06	7.15E-05	1.6708805	5.037525719	up-regulated in Low
CLCN7	-0.1131	3.4256961	-2.1662976	0.03076446	0.08846317	-5.8512239	1.51195065	up-regulated in High
IFT140	-0.4250437	2.28474048	-7.0912534	4.61E-12	1.07E-10	15.7872503	11.3364915	up-regulated in High
TMEM204	-0.5449868	3.10492504	-8.8647749	1.38E-17	6.67E-16	28.3256004	16.85859594	up-regulated in High
HN1L	0.24749105	5.15681668	4.91568055	1.20E-06	1.13E-05	3.62175267	5.91909162	up-regulated in Low
MAPK8IP3	-0.2279202	2.72626618	-2.6494849	0.00831894	0.0297533	-4.699908	2.079932017	up-regulated in High
NME3	-0.2805184	4.65062692	-3.8112317	0.00015568	0.00092503	-1.0222546	3.807776241	up-regulated in High
MRPS34	0.24940806	5.84108588	5.07486932	5.49E-07	5.50E-06	4.3797411	6.260124166	up-regulated in Low
IGFALS	-0.3560964	0.79917296	-4.649573	4.27E-06	3.57E-05	2.40375872	5.369422642	up-regulated in High
HAGH	-0.1754425	2.97226475	-3.8597499	0.00012852	0.00078003	-0.8413048	3.891028359	up-regulated in High
FAHD1	0.15166334	3.2936178	3.74075945	0.00020492	0.00118097	-1.2812355	3.688426932	up-regulated in Low
HS3ST6	0.15843717	0.46681099	2.25924901	0.02430154	0.0729959	-5.6473014	1.614366145	up-regulated in Low
MSRB1	0.37553219	4.68857758	4.67102165	3.87E-06	3.25E-05	2.49963657	5.412774169	up-regulated in Low
NDUFB10	0.14065521	5.87105696	3.15533834	0.00170059	0.00758188	-3.2546308	2.769400697	up-regulated in Low
RPS2	0.19323977	8.36670898	3.3834831	0.00077243	0.00379086	-2.5235901	3.112140895	up-regulated in Low

ZNF598	0.38437453	3.33881047	7.41194284	5.42E-13	1.41E-11	17.8920623	12.2662202	up-regulated in Low
NPW	0.59549448	1.09318434	4.77267348	2.40E-06	2.10E-05	2.95951719	5.620506935	up-regulated in Low
SLC9A3R2	-0.5144229	4.90627049	-6.7060763	5.46E-11	1.08E-09	13.3601359	10.26245487	up-regulated in High
TSC2	-0.1565348	3.28114679	-3.0603298	0.00233081	0.00998012	-3.5446165	2.632493346	up-regulated in High
PKD1	-0.1915549	1.97296179	-3.4681376	0.00056973	0.00289889	-2.2399381	3.24433139	up-regulated in High
RAB26	0.282595	1.15179035	3.88641248	0.00011557	0.0007091	-0.7409485	3.937154221	up-regulated in Low
TRAF7	0.22671561	4.53465322	5.1086854	4.64E-07	4.71E-06	4.54356455	6.333737723	up-regulated in Low
CASKIN1	0.13801613	0.25074801	5.33719348	1.44E-07	1.61E-06	5.67613168	6.841831365	up-regulated in Low
MLST8	0.20287492	3.38090747	4.93623706	1.09E-06	1.03E-05	3.71840455	5.962618331	up-regulated in Low
PGP	0.40679832	3.08910554	8.75773761	3.16E-17	1.46E-15	27.5092846	16.50004553	up-regulated in Low
ECI1	0.21743335	3.81570689	3.96017232	8.59E-05	0.0005435	-0.4599379	4.066144215	up-regulated in Low
RNPS1	0.17974865	3.90649061	5.26844812	2.06E-07	2.23E-06	5.33074271	6.687027912	up-regulated in Low
ABCA3	-1.4436526	4.3490495	-10.386455	5.37E-23	4.94E-21	40.669766	22.27017872	up-regulated in High
CCNF	0.68447065	1.97551229	12.8593284	7.35E-33	1.44E-30	63.2395638	32.13344773	up-regulated in Low
C16orf59	0.83463909	1.61168809	15.8713808	3.65E-46	1.52E-43	93.7565116	45.43808189	up-regulated in Low
TBC1D24	-0.3501056	2.05751444	-5.8691392	8.03E-09	1.12E-07	8.48218569	8.095527122	up-regulated in High
PDPK1	-0.2542965	2.06280411	-6.8361367	2.40E-11	5.02E-10	14.1671669	10.61983415	up-regulated in High
KCTD5	0.23397309	3.86336268	4.45222668	1.05E-05	8.10E-05	1.540639	4.978440844	up-regulated in Low
FLYWCH1	-0.1536871	2.28730392	-3.3267209	0.00094401	0.0045223	-2.7100347	3.025023995	up-regulated in High
KREMEN2	0.25329917	0.55040478	4.89719193	1.32E-06	1.23E-05	3.535136	5.880073502	up-regulated in Low
PKMYT1	1.00880846	1.63683031	17.6330299	2.26E-54	1.65E-51	112.603925	53.64548567	up-regulated in Low
PAQR4	0.27432205	2.69621235	3.94869059	9.00E-05	0.0005667	-0.5040072	4.04593143	up-regulated in Low
CLDN9	-0.684425	1.67802314	-5.7858797	1.28E-08	1.73E-07	8.02755964	7.892836917	up-regulated in High
HCFC1R1	-0.1280786	4.87767486	-1.9957535	0.04650764	0.12327786	-6.2034924	1.33247573	up-regulated in High
THOC6	0.11578056	4.11680927	2.52005598	0.01204674	0.04074691	-5.0304121	1.919130373	up-regulated in Low
CCDC64B	-0.5433153	2.68873943	-6.5595555	1.36E-10	2.54E-09	12.4664748	9.866386203	up-regulated in High
ZNF213	-0.1010993	2.24241064	-2.4354635	0.01522429	0.04956787	-5.2377054	1.817463	up-regulated in High
ZNF200	0.11312753	1.29857794	4.17207612	3.56E-05	0.00024603	0.37488404	4.448014591	up-regulated in Low
ZNF597	-0.2240458	1.21694281	-5.4302587	8.83E-08	1.03E-06	6.15005784	7.054052617	up-regulated in High
NAA60	-0.1298698	2.70588472	-2.9976629	0.00285715	0.01190216	-3.7312158	2.544066434	up-regulated in High
CLUAP1	-0.1957171	1.88697275	-5.1670616	3.45E-07	3.60E-06	4.82867232	6.461775424	up-regulated in High
NLRC3	-0.2372848	1.18647877	-4.4453305	1.08E-05	8.33E-05	1.51110155	4.965036439	up-regulated in High
TRAP1	0.35185974	3.66645383	8.09854597	4.35E-15	1.52E-13	22.6452249	14.36121159	up-regulated in Low
CREBBP	-0.1481603	3.08185985	-3.015712	0.00269537	0.01131051	-3.6778536	2.569381924	up-regulated in High
ADCY9	-0.5628054	2.2729369	-8.6901604	5.31E-17	2.39E-15	26.9976472	16.27526495	up-regulated in High
TFAP4	0.17588529	1.58584701	5.01385561	7.44E-07	7.27E-06	4.08664005	6.128339136	up-regulated in Low
GLIS2	-0.3549034	3.10335449	-5.4155919	9.54E-08	1.10E-06	6.07488512	7.020405107	up-regulated in High
CORO7	-0.139739	1.45791808	-3.4368873	0.00063794	0.00320217	-2.3454266	3.195217576	up-regulated in High
VASN	-0.3491943	3.74892772	-3.5477584	0.00042547	0.00224487	-1.967062	3.371134058	up-regulated in High
DNAJA3	0.23733555	3.30923303	6.76870986	3.68E-11	7.49E-10	13.7471728	10.43388088	up-regulated in Low
HMOX2	-0.1646767	3.90531697	-3.4686224	0.00056873	0.00289455	-2.2382941	3.245096339	up-regulated in High
CDIP1	-0.4245844	2.94551836	-6.8693656	1.94E-11	4.11E-10	14.3754132	10.71200938	up-regulated in High
MGRN1	-0.3723808	3.66535009	-6.4815223	2.20E-10	3.97E-09	11.9973024	9.658303145	up-regulated in High
NUDT16L1	-0.1911372	4.04011143	-3.3941893	0.00074352	0.00367025	-2.4880861	3.128709233	up-regulated in High
ANKS3	-0.1282621	1.62472539	-2.5734908	0.01035744	0.0358506	-4.895916	1.984747671	up-regulated in High
C16orf71	-0.2326608	0.60389594	-5.3985158	1.04E-07	1.20E-06	5.98759134	6.981325467	up-regulated in High
ZNF500	-0.1490406	1.71135328	-4.6479011	4.30E-06	3.59E-05	2.39630226	5.366050538	up-regulated in High
SMIM22	-0.2523688	4.41870362	-2.2583421	0.0243584	0.07313283	-5.6493316	1.613351167	up-regulated in High
ROGDI	-0.3841968	3.19348577	-7.1425321	3.29E-12	7.76E-11	16.1187456	11.48301431	up-regulated in High
GLYR1	-0.1029948	4.44486707	-2.8074309	0.0051907	0.01997162	-4.2748116	2.284774169	up-regulated in High
PPL	-0.4993989	4.09331783	-5.5699774	4.18E-08	5.16E-07	6.87518886	7.378359175	up-regulated in High
C16orf89	-2.7218758	5.41366593	-14.594	2.19E-40	6.66E-38	80.4961052	39.65985815	up-regulated in High
ABAT	-0.4127708	1.43212405	-6.1026782	2.11E-09	3.26E-08	9.78740935	8.676665445	up-regulated in High
PMM2	0.14169181	1.8758295	3.91055539	0.00010492	0.00065106	-0.6495154	3.979150836	up-regulated in Low
CARHSP1	0.22372673	3.78610417	4.28337153	2.21E-05	0.00015971	0.8295881	4.655264139	up-regulated in Low
GRIN2A	-0.102737	0.1482699	-3.2070621	0.00142766	0.00652857	-3.0931793	2.845374993	up-regulated in High
ATF7IP2	-0.1778531	1.27144216	-4.0798393	5.25E-05	0.00034984	0.00650491	4.279739506	up-regulated in High
EMP2	-0.7170869	4.65438498	-9.0995831	2.21E-18	1.19E-16	30.1414231	17.65580067	up-regulated in High
NUBP1	-0.1501483	3.49975946	-4.3219851	1.87E-05	0.00013702	0.98994393	4.728240008	up-regulated in High
CIITA	-0.6231579	2.09445704	-8.0009847	8.81E-15	2.95E-13	21.9498912	14.05507837	up-regulated in High
DEXI	-0.2176557	2.11780892	-5.684735	2.24E-08	2.88E-07	7.48291554	7.649810885	up-regulated in High
CLEC16A	-0.2263364	2.20620791	-5.4491206	7.99E-08	9.38E-07	6.24699718	7.097435124	up-regulated in High
RMI2	0.66288635	2.62948011	9.99707764	1.47E-21	1.17E-19	37.385537	20.83201264	up-regulated in Low
SOCS1	-0.2947156	2.84445984	-3.6271199	0.0003163	0.00172902	-1.6892206	3.499904896	up-regulated in High
LITAF	-0.2119229	5.50245744	-4.0482828	5.99E-05	0.00039303	-0.1177597	4.222895235	up-regulated in High
SNN	-0.1346603	3.71131977	-2.4564028	0.01437551	0.04725114	-5.1870368	1.842376652	up-regulated in High
TXNDC11	-0.4235592	4.77106939	-7.9969578	9.07E-15	3.03E-13	21.92133	14.04250154	up-regulated in High
ZC3H7A	-0.1290925	3.08934563	-3.3046589	0.00101978	0.00484593	-2.781687	2.991493224	up-regulated in High
RSL1D1	0.19488502	4.8509781	4.61037005	5.12E-06	4.21E-05	2.22956256	5.290620023	up-regulated in Low
TNFRSF17	-0.3917758	1.79573102	-3.747104	0.00019994	0.00115619	-1.2581067	3.699095385	up-regulated in High
SNX29	-0.3277665	1.95026349	-7.8177135	3.25E-14	1.01E-12	20.661328	13.48747624	up-regulated in High

SHISA9	0.11183158	0.07304203	3.46719134	0.00057169	0.0029071	-2.2431455	3.242838866	up-regulated in Low
MKL2	-0.2038989	2.21973952	-4.3255682	1.84E-05	0.0001352	1.00489174	4.735039702	up-regulated in High
PARN	-0.152929	3.94867677	-3.3004058	0.00103502	0.00490797	-2.7954473	2.98505051	up-regulated in High
BFAR	-0.1866053	4.41920083	-3.6524658	0.00028739	0.0015905	-1.5992571	3.541531195	up-regulated in High
PLA2G10	-0.7130929	1.80144371	-5.5999155	3.56E-08	4.44E-07	7.03268133	7.448735192	up-regulated in High
NPIPA1	-0.1012401	0.49891826	-3.5058101	0.00049656	0.00257277	-2.1115614	3.30402953	up-regulated in High
NOMO1	0.1345012	3.39888955	2.76627618	0.00588183	0.02221504	-4.3878749	2.230487652	up-regulated in Low
MPV17L	-0.1577619	0.83880385	-2.3462186	0.01935807	0.06046107	-5.4489063	1.713138066	up-regulated in High
C16orf45	-0.2615026	1.87262482	-3.5061768	0.00049589	0.00257	-2.1103054	3.304613222	up-regulated in High
KIAA0430	-0.3082465	3.14348373	-6.7189576	5.04E-11	1.00E-09	13.4394893	10.29760717	up-regulated in High
NDE1	0.22606757	2.26989619	4.52035208	7.73E-06	6.13E-05	1.83470157	5.11179861	up-regulated in Low
MYH11	-1.0153166	2.42115016	-9.9409948	2.36E-21	1.82E-19	36.9193222	20.6277742	up-regulated in High
FOPNL	0.23598544	4.17365349	4.84528663	1.69E-06	1.54E-05	3.29355126	5.771192318	up-regulated in Low
ABCC1	0.35469071	3.67091947	5.48422881	6.63E-08	7.90E-07	6.42822665	7.178516135	up-regulated in Low
ABCC6	-0.6646434	1.75525905	-9.4673263	1.17E-19	7.35E-18	33.0527407	18.93302329	up-regulated in High
XYLT1	-0.1789756	1.32694608	-2.8078197	0.00518454	0.01995054	-4.2737357	2.285290102	up-regulated in High
NOMO2	0.13601613	1.65105918	2.66281646	0.008001	0.02878692	-4.6649507	2.096855997	up-regulated in Low
ARL6IP1	0.45106992	5.92246901	9.44682745	1.38E-19	8.60E-18	32.8883348	18.8609254	up-regulated in Low
TMC5	-0.4111378	4.22144191	-3.7424524	0.00020358	0.00117406	-1.2750676	3.691272156	up-regulated in High
GDE1	-0.3606338	4.88293465	-6.986857	9.10E-12	2.02E-10	15.1184048	11.04074024	up-regulated in High
KNOP1	0.3202949	1.85501587	9.61528834	3.50E-20	2.33E-18	34.2467248	19.45653238	up-regulated in Low
GP2	0.24308391	0.30575788	2.59444378	0.00975499	0.03414043	-4.842427	2.010773094	up-regulated in Low
ACSM5	-0.1982719	0.30418686	-7.1621479	2.89E-12	6.87E-11	16.246067	11.53928112	up-regulated in High
ACSM3	-0.4576153	1.45462294	-6.6239269	9.13E-11	1.75E-09	12.8570547	10.03953429	up-regulated in High
ACSM1	-0.2330875	0.45024856	-7.0170027	7.49E-12	1.68E-10	15.310708	11.12578967	up-regulated in High
THUMPD1	-0.1732584	3.63483808	-4.6069504	5.20E-06	4.27E-05	2.21443182	5.283772773	up-regulated in High
AC004381.6	0.31397446	1.12998994	7.86787735	2.28E-14	7.20E-13	21.0117205	13.64185993	up-regulated in Low
DCUN1D3	-0.2874923	2.05563308	-6.4220928	3.15E-10	5.55E-09	11.6431633	9.501168005	up-regulated in High
LYRM1	-0.127164	2.93102083	-2.4358319	0.01520898	0.04952356	-5.2368177	1.817899841	up-regulated in High
DNAH3	-0.205895	0.50616971	-4.2614225	2.43E-05	0.00017387	0.73903286	4.614028305	up-regulated in High
TMEM159	-0.6099199	4.53253228	-7.7767026	4.35E-14	1.32E-12	20.3761681	13.36181218	up-regulated in High
CRYM	-1.1387336	2.16093727	-8.1967812	2.13E-15	7.83E-14	23.3518757	14.67222164	up-regulated in High
METTL9	0.23456589	3.89856789	5.34713507	1.37E-07	1.54E-06	5.72641049	6.86435629	up-regulated in Low
IGSF6	-0.5479445	2.79594136	-6.3733332	4.24E-10	7.30E-09	11.3546678	9.373113068	up-regulated in High
UQCRC2	0.19308375	4.815099	4.66895506	3.90E-06	3.28E-05	2.49038111	5.408589936	up-regulated in Low
VWA3A	-0.3421621	0.46284911	-5.0789622	5.38E-07	5.40E-06	4.39951727	6.269012218	up-regulated in High
POLR3E	0.12053348	2.464501	3.22410113	0.00134702	0.00619818	-3.039442	2.870624918	up-regulated in Low
HS3ST2	-0.6499265	1.65834005	-6.9049031	1.55E-11	3.32E-10	14.5990499	10.81097783	up-regulated in High
USP31	0.20111424	1.22339516	5.45319346	7.82E-08	9.19E-07	6.26796856	7.106819136	up-regulated in Low
SCNN1G	-0.6119729	1.94745276	-5.5650724	4.30E-08	5.30E-07	6.84945686	7.366858748	up-regulated in High
SCNN1B	-1.4029807	3.51873926	-11.030845	1.89E-25	2.19E-23	46.278937	24.7243618	up-regulated in High
GGA2	-0.368532	3.89173839	-8.2264451	1.71E-15	6.38E-14	23.5665375	14.76667791	up-regulated in High
UBFD1	0.18028866	3.20071309	4.15650901	3.81E-05	0.00026121	0.31217247	4.419392407	up-regulated in Low
NDUFAB1	0.34974699	4.94163133	8.13751942	3.28E-15	1.17E-13	22.9248001	14.48427025	up-regulated in Low
PALB2	0.2229556	2.14236895	6.80813679	2.87E-11	5.93E-10	13.9923397	10.54243774	up-regulated in Low
PLK1	1.44477956	2.48813731	23.2127774	3.22E-81	9.39E-77	174.311114	80.49271984	up-regulated in Low
ERN2	-0.3877828	1.53878396	-2.5740934	0.01033966	0.03579612	-4.8943835	1.985493818	up-regulated in High
CHP2	-0.1028002	0.22985554	-2.0761212	0.03839771	0.10572634	-6.041023	1.415694687	up-regulated in High
PRKCB	-0.3931857	1.33862865	-6.6512973	7.70E-11	1.49E-09	13.0240944	10.11356365	up-regulated in High
SLC5A11	0.1317842	0.13920574	5.30336547	1.72E-07	1.89E-06	5.50567429	6.765447538	up-regulated in Low
LCMT1	0.11854408	3.64438931	3.67223703	0.00026658	0.00148942	-1.5286687	3.574169971	up-regulated in Low
IL4R	-0.1891529	4.0839165	-3.3734983	0.00080034	0.00390974	-2.5566052	3.096728125	up-regulated in High
IL21R	-0.1570831	1.19387305	-2.6437518	0.00845912	0.0301915	-4.7148886	2.072674776	up-regulated in High
GTF3C1	0.11880173	3.41212119	2.50706213	0.01249288	0.04201979	-5.0627021	1.903337601	up-regulated in Low
XPO6	0.22037568	3.99150947	5.64185204	2.83E-08	3.58E-07	7.254543	7.547839735	up-regulated in Low
APOBR	-0.5217667	2.34155912	-6.7328316	4.62E-11	9.25E-10	13.5251009	10.33552871	up-regulated in High
NUPR1	-0.3592979	3.73522753	-3.7582686	0.00019147	0.00111192	-1.2173167	3.717905596	up-regulated in High
CCDC101	-0.1972272	2.82313717	-4.5374263	7.15E-06	5.71E-05	1.90904666	5.145488683	up-regulated in High
SULT1A2	-0.3544709	1.46975835	-4.7849799	2.26E-06	1.99E-05	3.01580555	5.645910574	up-regulated in High
SULT1A1	-0.4064752	1.76035922	-6.3069781	6.31E-10	1.06E-08	10.965065	9.200110883	up-regulated in High
ATXN2L	0.27823489	4.54430469	6.16652456	1.45E-09	2.30E-08	10.1518742	8.838748435	up-regulated in Low
TUFM	0.20528071	6.55356321	5.21798704	2.66E-07	2.83E-06	5.07976504	6.574461646	up-regulated in Low
SH2B1	-0.2003086	2.97776052	-3.3526512	0.00086165	0.00417399	-2.6252363	3.064669694	up-regulated in High
ATP2A1	0.136627	0.41214204	4.85825155	1.59E-06	1.46E-05	3.35367518	5.798297551	up-regulated in Low
RABEP2	-0.1879415	2.89512064	-3.5495487	0.00042266	0.00223186	-1.9608588	3.374012677	up-regulated in High
CD19	-0.3630349	1.23425328	-4.2620749	2.43E-05	0.00017349	0.7417185	4.615251526	up-regulated in High
RP11-345J4.5	0.30934495	1.10580155	7.89016431	1.95E-14	6.21E-13	21.1679533	13.71068703	up-regulated in Low
SPN	-0.5668073	1.91886009	-7.7374103	5.73E-14	1.72E-12	20.1040595	13.24188076	up-regulated in High
QPRT	0.37299066	3.34801969	2.66978338	0.00783922	0.02829029	-4.6466146	2.105727097	up-regulated in Low
C16orf54	-0.5847288	1.72447477	-7.9062417	1.74E-14	5.58E-13	21.2808695	13.76042776	up-regulated in High
KIF22	0.55971583	3.64089603	11.075386	1.27E-25	1.49E-23	46.6743061	24.89726053	up-regulated in Low

MAZ	0.42803558	3.9158291	9.21550029	8.82E-19	4.99E-17	31.0503288	18.0546648	up-regulated in Low
PRRT2	-0.2770552	0.87423498	-4.5439829	6.94E-06	5.56E-05	1.93766438	5.158454356	up-regulated in High
PAGR1	0.11207301	2.36977104	2.30493385	0.02158266	0.06615986	-5.5439995	1.665895036	up-regulated in Low
PAGR1	0.11207301	2.36977104	2.30493385	0.02158266	0.06615986	-5.5439995	1.665895036	up-regulated in Low
MVP	-0.5770295	5.92285855	-8.3733757	5.78E-16	2.30E-14	24.6384409	15.23820417	up-regulated in High
CDIPT	-0.1590412	4.83789042	-3.9298128	9.71E-05	0.00060755	-0.5762027	4.012805593	up-regulated in High
SEZ6L2	-0.4664647	4.27997515	-3.7770531	0.00017797	0.00104118	-1.1484285	3.749659628	up-regulated in High
TMEM219	-0.1372844	5.540714	-2.8503929	0.00454851	0.01780223	-4.1550579	2.342131044	up-regulated in High
TAOK2	-0.1167468	3.23446809	-2.4132806	0.01617147	0.05209973	-5.2909214	1.791250563	up-regulated in High
ALDOA	0.56386703	7.75460149	9.72396119	1.43E-20	1.00E-18	35.131738	19.84446645	up-regulated in Low
PPP4C	0.33893563	5.42001923	7.54260027	2.22E-13	6.13E-12	18.7710023	12.6540611	up-regulated in Low
YPEL3	-0.5048368	4.33282721	-7.9442286	1.32E-14	4.34E-13	21.5483711	13.87825312	up-regulated in High
CORO1A	-0.398003	3.92359479	-4.7572974	2.58E-06	2.25E-05	2.88937415	5.588843825	up-regulated in High
BOLA2B	0.15611054	0.4931956	6.0151507	3.49E-09	5.20E-08	9.29307034	8.45669581	up-regulated in Low
TBC1D10B	0.15918552	3.56013506	4.08268685	5.19E-05	0.00034612	0.01776239	4.284887133	up-regulated in Low
ZNF48	0.2233921	2.36724584	5.13022586	4.16E-07	4.26E-06	4.64842865	6.380841513	up-regulated in Low
1-Sep	-0.2712378	1.93249205	-3.8715976	0.00012261	0.00074763	-0.796791	3.911491872	up-regulated in High
ZNF771	0.11316886	0.67276873	4.77606597	2.36E-06	2.07E-05	2.97502094	5.627504443	up-regulated in Low
DCTPP1	0.44933154	4.542149	7.76609608	4.68E-14	1.42E-12	20.3026092	13.329393	up-regulated in Low
SEPHS2	0.37770163	5.37220625	6.19258226	1.24E-09	1.99E-08	10.3015584	8.90529259	up-regulated in Low
ITGAL	-0.4365676	2.62386754	-5.060834	5.89E-07	5.87E-06	4.31203408	6.229690853	up-regulated in High
ZNF768	0.25538642	4.25369575	4.88173085	1.42E-06	1.31E-05	3.46293043	5.847539284	up-regulated in Low
ZNF688	-0.2036428	2.27572285	-4.3769626	1.47E-05	0.00011005	1.22055784	4.833092225	up-regulated in High
ZNF785	-0.1278924	1.66480114	-2.9920226	0.00290951	0.01209263	-3.7478281	2.536180742	up-regulated in High
RNF40	0.13088137	3.6012536	2.61038046	0.00931796	0.03280359	-4.8014612	2.030679283	up-regulated in Low
ZNF629	-0.1735025	2.86789154	-3.2060343	0.00143267	0.00654889	-3.0964122	2.843855358	up-regulated in High
CTF1	-0.5682727	2.58636886	-7.3204067	1.01E-12	2.53E-11	17.2836192	11.99760569	up-regulated in High
FBXL19	0.3328428	2.79879478	5.76485221	1.44E-08	1.92E-07	7.91363891	7.84202291	up-regulated in Low
ORAI3	-0.1900474	2.68629238	-3.9317376	9.63E-05	0.00060331	-0.5688567	4.016176934	up-regulated in High
SETD1A	0.1343317	2.88490178	2.99799068	0.00285414	0.01189214	-3.7302496	2.544525013	up-regulated in Low
ZNF668	0.10404283	1.14152073	4.07943602	5.26E-05	0.00035039	0.00491105	4.279010667	up-regulated in Low
KAT8	-0.1489087	3.38037714	-3.4815205	0.00054265	0.0027771	-2.1944842	3.265477421	up-regulated in High
PRSS8	-0.4740199	6.19530838	-5.0935426	5.00E-07	5.05E-06	4.47008363	6.300723234	up-regulated in High
FUS	0.13913768	4.80866715	2.99550252	0.0028771	0.01197245	-3.7375824	2.541044538	up-regulated in Low
PYCARD	-0.1738228	3.61670442	-2.0697733	0.03899117	0.10703233	-6.054085	1.409033705	up-regulated in High
TRIM72	0.10743435	0.1984929	2.0659305	0.03935422	0.10788181	-6.0619732	1.405008714	up-regulated in Low
ITGAM	-0.5316089	2.51062198	-6.0209099	3.38E-09	5.04E-08	9.32540759	8.471089853	up-regulated in High
ITGAX	-0.3842398	2.69968209	-4.6375017	4.52E-06	3.75E-05	2.34997643	5.345098124	up-regulated in High
ARMC5	-0.1271963	1.81947012	-2.8420529	0.00466722	0.01820097	-4.1784429	2.330941754	up-regulated in High
TGFB1I1	-0.2787354	2.35299064	-4.5213926	7.69E-06	6.10E-05	1.83922477	5.113848625	up-regulated in High
SLC5A2	-0.1945676	0.3832696	-5.3647818	1.25E-07	1.41E-06	5.81586259	6.904424675	up-regulated in High
C16orf58	-0.1795708	3.94704226	-3.4163482	0.00068685	0.00341797	-2.4142628	3.163138785	up-regulated in High
ZNF720	-0.1070966	1.446791	-3.6112998	0.0003357	0.00182213	-1.7450714	3.474045983	up-regulated in High
RP11-812E19.	-0.4006561	1.29766978	-3.8433176	0.00013717	0.00082662	-0.902831	3.862733412	up-regulated in High
SHCBP1	0.89959189	1.70425296	16.3996477	1.34E-48	6.29E-46	99.3490926	47.87402727	up-regulated in Low
VPS35	0.10336174	4.00344288	2.73924974	0.00637988	0.02382184	-4.4612434	2.195187375	up-regulated in Low
ORC6	0.90526522	1.40219628	18.8115609	5.68E-60	6.38E-57	125.470279	59.2457211	up-regulated in Low
C16orf87	0.17620773	1.29284448	6.1495926	1.60E-09	2.52E-08	10.0549014	8.795630899	up-regulated in Low
GPT2	0.54114076	3.21657425	5.1048854	4.73E-07	4.80E-06	4.52510647	6.325445228	up-regulated in Low
NETO2	0.48853427	2.0585302	6.86154982	2.04E-11	4.30E-10	14.3263564	10.69029711	up-regulated in Low
ITFG1	-0.1086392	3.36711261	-2.4115158	0.01624901	0.05230336	-5.2951345	1.789173226	up-regulated in High
PHKB	-0.3486167	3.10394184	-7.6048086	1.44E-13	4.09E-12	19.1937667	12.84053281	up-regulated in High
LONP2	-0.1231044	3.36095972	-2.9877834	0.00294943	0.01223853	-3.7602938	2.53026194	up-regulated in High
N4BP1	-0.2395783	3.22814129	-6.3698297	4.33E-10	7.45E-09	11.3340107	9.363942327	up-regulated in High
ZNF423	-0.1826822	0.46701251	-6.5363626	1.57E-10	2.90E-09	12.3265346	9.80433211	up-regulated in High
HEATR3	0.16501098	2.93972208	4.49518124	8.66E-06	6.79E-05	1.72557248	5.062327444	up-regulated in Low
ADCY7	-0.1759146	1.76796092	-2.6579607	0.00811552	0.02912895	-4.6777031	2.090683933	up-regulated in High
BRD7	0.10874602	3.02003197	3.17701939	0.0015808	0.0071274	-3.187262	2.801123253	up-regulated in Low
NKD1	-0.2078144	0.74238442	-3.1473576	0.00174675	0.00776516	-3.2793175	2.757768595	up-regulated in High
SNX20	-0.358845	1.5544393	-6.0617213	2.67E-09	4.06E-08	9.55532645	8.573412809	up-regulated in High
NOD2	-0.1031295	1.15359002	-2.4224805	0.01577252	0.05105388	-5.2689089	1.802099018	up-regulated in High
CYLD	-0.29422	2.32489332	-6.703452	5.56E-11	1.10E-09	13.3439848	10.25529984	up-regulated in High
SALL1	0.15844844	0.27348005	2.63805082	0.00860061	0.03062536	-4.7297539	2.0654706	up-regulated in Low
CHD9	-0.2348918	1.89207336	-5.8179148	1.07E-08	1.46E-07	8.20181197	7.970543118	up-regulated in High
RBL2	-0.4297852	3.59655473	-8.4808948	2.59E-16	1.07E-14	25.4318583	15.58708505	up-regulated in High
AKTIP	-0.1837714	2.62038758	-4.5614213	6.41E-06	5.17E-05	2.01396258	5.193015158	up-regulated in High
FTO	-0.1769006	2.1637165	-4.4475188	1.07E-05	8.26E-05	1.5204696	4.969287943	up-regulated in High
IRX3	-0.8387497	3.80039329	-7.1128999	4.00E-12	9.34E-11	15.9269482	11.39824336	up-regulated in High
IRX5	-0.8092473	2.41773576	-7.6239441	1.26E-13	3.62E-12	19.3243634	12.89812626	up-regulated in High
IRX6	-0.3102738	0.80728285	-3.3495241	0.00087121	0.00421543	-2.6354961	3.059875063	up-regulated in High
MMP2	-0.5791816	5.84011678	-5.306205	1.69E-07	1.87E-06	5.51994516	6.771843598	up-regulated in High

LPCAT2	-0.4240879	2.14200247	-7.3348863	9.12E-13	2.31E-11	17.3794614	12.03992545	up-regulated in High
CAPNS2	-0.1614508	0.4188882	-1.9658049	0.04987905	0.13031539	-6.2624218	1.302081788	up-regulated in High
CES1	-0.3972298	3.91799974	-2.210913	0.02749774	0.08074791	-5.7543921	1.560702961	up-regulated in High
GNAO1	-0.1460113	0.5346977	-3.545031	0.00042978	0.00226478	-1.9765068	3.366750842	up-regulated in High
AMFR	0.1033016	4.11405542	2.05578771	0.04032631	0.1100763	-6.0827245	1.39441154	up-regulated in Low
NUDT21	0.22170065	3.99057842	5.65453056	2.64E-08	3.36E-07	7.3219041	7.577921652	up-regulated in Low
OGFOD1	0.12589428	2.97997921	3.96420284	8.45E-05	0.00053562	-0.4444394	4.073251357	up-regulated in Low
BBS2	-0.1695173	2.22245785	-4.3902711	1.38E-05	0.00010425	1.27678887	4.858641664	up-regulated in High
MT2A	0.3401364	6.1164426	2.76504054	0.00590381	0.02228873	-4.3912445	2.228867687	up-regulated in Low
MT1E	-0.4808855	4.53322305	-3.6041156	0.00034488	0.00186552	-1.7703579	3.462334054	up-regulated in High
MT1A	0.34991047	1.46494831	2.88812698	0.00404495	0.0161122	-4.0484254	2.393086599	up-regulated in Low
MT1G	0.54521935	2.16092293	4.46031869	1.01E-05	7.84E-05	1.57535255	4.994192052	up-regulated in Low
MT1H	0.49689434	0.84376439	6.30530962	6.37E-10	1.07E-08	10.9553133	9.195779606	up-regulated in Low
MT1X	0.22744627	3.74771729	2.19382775	0.02871144	0.08356386	-5.7917021	1.541945067	up-regulated in Low
NUP93	0.23600457	2.38674026	7.22314972	1.93E-12	4.69E-11	16.6438254	11.71502667	up-regulated in Low
HERPUD1	-0.4424616	4.9659895	-7.7579544	4.96E-14	1.50E-12	20.2461975	13.30453014	up-regulated in High
CPNE2	-0.4269784	2.99983238	-7.7992368	3.71E-14	1.14E-12	20.5327099	13.43079935	up-regulated in High
FAM192A	0.10718132	4.00200789	3.04412375	0.00245764	0.01045493	-3.5932288	2.609482623	up-regulated in Low
ARL2BP	-0.1416387	2.18077066	-3.3701048	0.00081003	0.00395098	-2.5678046	3.091498435	up-regulated in High
PLLP	-0.7960681	1.97039499	-10.814046	1.29E-24	1.37E-22	44.3683753	23.88870045	up-regulated in High
CCL22	-0.4462566	2.20066197	-4.1850167	3.37E-05	0.00023409	0.42718088	4.471875977	up-regulated in High
CX3CL1	-1.2270459	3.56180728	-9.8400073	5.47E-21	4.06E-19	36.0842414	20.2618888	up-regulated in High
CCL17	-0.9502555	1.96258104	-7.6425508	1.11E-13	3.21E-12	19.4515992	12.95423312	up-regulated in High
CIAPIN1	0.23981478	3.83628836	6.56680426	1.30E-10	2.44E-09	12.5102976	9.885816749	up-regulated in Low
COQ9	0.21234136	3.64223072	4.90206636	1.29E-06	1.20E-05	3.5579433	5.890348471	up-regulated in Low
POLR2C	-0.1150963	4.57336705	-2.2084681	0.02766866	0.08111517	-5.7597486	1.558011945	up-regulated in High
DOK4	-0.8905717	3.51295279	-9.8735321	4.14E-21	3.13E-19	36.3608288	20.38308173	up-regulated in High
CCDC102A	-0.2761266	2.05955079	-5.2921963	1.82E-07	2.00E-06	5.44960568	6.740316126	up-regulated in High
GPR97	0.29955544	0.62064838	6.108446	2.04E-09	3.16E-08	9.82020066	8.691251492	up-regulated in Low
DRC7	-0.1959386	0.35053947	-4.0523525	5.89E-05	0.00038722	-0.1017844	4.230205409	up-regulated in High
TEPP	-0.4773435	0.55318024	-7.6216548	1.28E-13	3.67E-12	19.3087256	12.89123019	up-regulated in High
ZNF319	-0.2851805	2.49259965	-5.993508	3.96E-09	5.83E-08	9.1717871	8.40270391	up-regulated in High
MMP15	-0.3648197	4.36297467	-4.0598628	5.71E-05	0.00037696	-0.0722644	4.243711705	up-regulated in High
CFAP20	0.15841	3.7021308	4.40853703	1.28E-05	9.68E-05	1.35422266	4.893814338	up-regulated in Low
CSNK2A2	0.19735827	3.3716777	5.74851273	1.58E-08	2.09E-07	7.82536684	7.802642723	up-regulated in Low
GINS3	0.53040799	1.26147075	13.9172404	2.12E-37	5.35E-35	73.6484322	36.67435019	up-regulated in Low
NDRG4	0.14627727	0.86115809	2.51196474	0.01232285	0.04155084	-5.0505381	1.909288741	up-regulated in Low
SETD6	0.10677987	1.97248943	2.57373104	0.01035035	0.035831	-4.895305	1.985045153	up-regulated in Low
SLC38A7	0.29923194	2.32367959	6.99198169	8.81E-12	1.96E-10	15.1510477	11.05517809	up-regulated in Low
GOT2	0.3589709	4.64626972	8.56362022	1.39E-16	5.98E-15	26.0474531	15.85769561	up-regulated in Low
CDH11	-0.5692375	2.95890899	-6.2778187	7.51E-10	1.24E-08	10.7949537	9.124547919	up-regulated in High
CDH5	-0.4076742	2.9449757	-5.7048948	2.00E-08	2.60E-07	7.59080077	7.697968568	up-regulated in High
BEAN1	-0.2121836	1.08223139	-2.9617431	0.00320587	0.01315769	-3.8364934	2.494053827	up-regulated in High
TK2	-0.4854339	2.61870298	-10.355833	6.99E-23	6.35E-21	40.4085306	22.15581907	up-regulated in High
CMTM3	-0.2240883	4.10688591	-2.7672392	0.00586475	0.02216774	-4.3852478	2.231750584	up-regulated in High
DYNC1LI2	-0.1421254	3.67208924	-2.7538039	0.00610713	0.02294862	-4.4218202	2.214162611	up-regulated in High
NAE1	0.2401565	3.2310715	6.38521333	3.94E-10	6.83E-09	11.4247872	9.404240974	up-regulated in Low
RRAD	-1.0737663	3.38619614	-7.9776494	1.04E-14	3.46E-13	21.7845383	13.9822631	up-regulated in High
FAM96B	0.14819078	5.79063685	2.85714981	0.00445435	0.01749344	-4.1360634	2.351215706	up-regulated in Low
CES2	-0.2905575	3.21590789	-6.0106826	3.58E-09	5.32E-08	9.26800087	8.445536362	up-regulated in High
CES3	-0.2947244	1.00394226	-3.2822761	0.00110239	0.0051824	-2.8539147	2.957663423	up-regulated in High
CES4A	-0.3481261	1.32696205	-5.0579736	5.98E-07	5.94E-06	4.2982564	6.22349731	up-regulated in High
B3GNT9	-0.4679143	2.79769332	-8.0870078	4.73E-15	1.65E-13	22.5626537	14.3248636	up-regulated in High
TRADD	-0.3396219	3.826856	-6.3293904	5.52E-10	9.33E-09	11.096271	9.258381563	up-regulated in High
FBXL8	-0.2476582	1.83653115	-4.0110887	6.98E-05	0.00045188	-0.263064	4.156372516	up-regulated in High
E2F4	0.19918453	4.33423897	5.37944777	1.15E-07	1.31E-06	5.89040471	6.937808429	up-regulated in Low
ELMO3	-0.1734551	4.10721709	-2.2635991	0.02403041	0.07229678	-5.6375523	1.6192388	up-regulated in High
LRRC29	-0.1722715	1.10160377	-4.3412169	1.72E-05	0.00012709	1.07030863	4.764791883	up-regulated in High
TMEM208	0.14442574	4.6675846	2.5879287	0.00993887	0.0346531	-4.859104	2.002662979	up-regulated in Low
FHOD1	-0.4355536	3.52956233	-6.5184193	1.75E-10	3.21E-09	12.2185556	9.756444401	up-regulated in High
SLC9A5	-0.2453881	0.89795515	-4.8176641	1.93E-06	1.74E-05	3.165941	5.71364558	up-regulated in High
LRRC36	-0.5166331	0.69523358	-7.5041317	2.89E-13	7.82E-12	18.5109512	12.53933394	up-regulated in High
TPPP3	-1.0767153	3.3843612	-8.1195566	3.74E-15	1.32E-13	22.7958165	14.42749851	up-regulated in High
ZDHHC1	-0.4506816	2.47459721	-8.0134836	8.05E-15	2.71E-13	22.03861	14.09414434	up-regulated in High
ATP6V0D1	-0.2421499	3.95122538	-5.4980797	6.16E-08	7.38E-07	6.5000086	7.21062273	up-regulated in High
AGRP	-0.3627813	0.58907631	-7.3596689	7.72E-13	1.97E-11	17.543853	12.11250705	up-regulated in High
CTCF	0.13984605	3.90198129	4.15073469	3.90E-05	0.000267	0.2889664	4.408798438	up-regulated in Low
ACD	0.16704879	2.62165696	3.59989125	0.00035038	0.00189074	-1.7852044	3.455456413	up-regulated in Low
ENKD1	-0.2640758	2.55938437	-4.0271536	6.53E-05	0.00042485	-0.2004581	4.185041766	up-regulated in High
C16orf86	-0.1630682	0.88456685	-4.3853058	1.42E-05	0.00010636	1.25579093	4.849101686	up-regulated in High
GFOD2	0.11773616	1.6890899	3.08437892	0.00215365	0.00931999	-3.4720197	2.666824217	up-regulated in Low

TSNAXIP1	-0.1541302	0.40287429	-3.675201	0.00026359	0.00147485	-1.5180554	3.579075668	up-regulated in High
NUTF2	0.31978304	4.58771404	7.5087149	2.80E-13	7.61E-12	18.5418789	12.55297935	up-regulated in Low
LCAT	-0.3216388	1.68633082	-5.0422581	6.46E-07	6.38E-06	4.22268362	6.189520609	up-regulated in High
SLC12A4	-0.3533539	2.76430497	-7.0140299	7.63E-12	1.71E-10	15.2917136	11.11738969	up-regulated in High
DPEP2	-0.5347922	1.24318158	-9.955914	2.08E-21	1.61E-19	37.043175	20.68203347	up-regulated in High
DDX28	0.13454831	2.81269892	3.48036623	0.00054494	0.00278831	-2.1984112	3.263650851	up-regulated in Low
NFATC3	-0.2310296	1.95645131	-5.1914173	3.05E-07	3.21E-06	4.94848478	6.515553941	up-regulated in High
ESRP2	-0.216265	3.83854022	-3.1457704	0.00175607	0.00780243	-3.28422	2.755458135	up-regulated in High
PLA2G15	-0.292148	3.0100728	-6.1691089	1.43E-09	2.27E-08	10.1666955	8.845338038	up-regulated in High
SLC7A6	-0.1202241	1.48547529	-2.7065188	0.00703384	0.02584874	-4.5491619	2.152807566	up-regulated in High
SMPD3	-0.2746045	1.20570391	-3.2571876	0.00120235	0.00559731	-2.934315	2.919969857	up-regulated in High
ZFP90	-0.2068323	2.14569352	-5.494487	6.28E-08	7.52E-07	6.48137438	7.202288468	up-regulated in High
CDH1	-0.2266547	6.11680207	-2.8919555	0.00399681	0.01594218	-4.0375306	2.398286802	up-regulated in High
HAS3	-1.0700398	2.510402	-8.3657358	6.12E-16	2.42E-14	24.5823532	15.21353689	up-regulated in High
CIRH1A	0.27430061	3.07329766	7.53713359	2.30E-13	6.32E-12	18.7339827	12.63773025	up-regulated in Low
PDF	0.20085672	1.33190119	5.23731215	2.41E-07	2.59E-06	5.1756267	6.617464594	up-regulated in Low
NIP7	0.31947733	3.45142199	7.64858603	1.07E-13	3.09E-12	19.4929219	12.97245419	up-regulated in Low
CYB5B	0.21646705	3.63935312	5.2630017	2.11E-07	2.29E-06	5.30354991	6.674834867	up-regulated in Low
NFAT5	-0.1292568	1.72587244	-2.6802169	0.00760245	0.02757557	-4.6190679	2.119046705	up-regulated in High
NQO1	0.5968892	5.70299596	3.80917024	0.00015694	0.00093124	-1.0298952	3.804258508	up-regulated in Low
NOB1	0.25166657	4.40528313	5.84815348	9.03E-09	1.24E-07	8.36706288	8.044214863	up-regulated in Low
WWP2	-0.3092099	3.19216912	-6.8949618	1.65E-11	3.52E-10	14.5363937	10.78325182	up-regulated in High
PDPR	-0.1677423	2.34478776	-3.1861168	0.0015329	0.00694777	-3.1588621	2.814487182	up-regulated in High
AARS	0.28214577	5.24959347	5.33701604	1.44E-07	1.61E-06	5.67523508	6.841429668	up-regulated in Low
DDX19B	-0.1076382	2.37153239	-3.1942811	0.00149105	0.00677421	-3.1333087	2.826507142	up-regulated in High
ST3GAL2	-0.183705	1.91655161	-4.2823883	2.22E-05	0.00016027	0.82552245	4.653413172	up-regulated in High
FUK	-0.1774161	2.2805929	-3.6525861	0.00028726	0.00158992	-1.5988287	3.541729336	up-regulated in High
SF3B3	0.2378525	3.84516656	5.6050778	3.46E-08	4.32E-07	7.0599136	7.460901908	up-regulated in Low
IL34	-0.4512413	1.38066034	-7.1071192	4.15E-12	9.69E-11	15.8896078	11.38173811	up-regulated in High
MTSS1L	-0.32696	2.47176593	-5.1652483	3.49E-07	3.63E-06	4.81977276	6.457780163	up-regulated in High
HYDIN	-0.1099701	0.17545148	-3.8110531	0.00015579	0.00092532	-1.0229169	3.807471347	up-regulated in High
CMTR2	-0.143021	2.30482139	-3.6345704	0.00030753	0.00168661	-1.662837	3.512116044	up-regulated in High
CALB2	0.38468573	0.91314316	3.76864705	0.00018389	0.00107241	-1.1792961	3.735433317	up-regulated in Low
ZNF19	-0.1530072	0.60499985	-7.780227	4.24E-14	1.29E-12	20.4006284	13.37259211	up-regulated in High
MARVELD3	0.14715275	1.9719961	2.82396035	0.00493448	0.01910114	-4.2289452	2.306759028	up-regulated in Low
ATXN1L	-0.1792332	2.69840283	-3.9699005	8.25E-05	0.00052518	-0.4225052	4.083308527	up-regulated in High
HP	-0.638089	2.24187349	-3.4166381	0.00068614	0.00341558	-2.413294	3.163590426	up-regulated in High
ZFHX3	-0.1270552	1.84349087	-2.4738349	0.01370112	0.04541914	-5.1445322	1.863243905	up-regulated in High
PSMD7	0.36443154	4.68420733	9.06648851	2.87E-18	1.51E-16	29.8834336	17.54256393	up-regulated in Low
NPIPB15	-0.1924655	1.16042872	-1.9811369	0.04812824	0.1266993	-6.2323628	1.317599986	up-regulated in High
RFWD3	0.49233879	2.85410829	11.5105027	2.47E-27	3.32E-25	50.5862856	26.60745976	up-regulated in Low
ZNRF1	0.15034231	2.16815411	3.56036242	0.00040604	0.00215584	-1.9233258	3.391426423	up-regulated in Low
LDHD	-0.3488113	2.21930218	-3.6656765	0.00027332	0.00152145	-1.5521314	3.563323437	up-regulated in High
BCAR1	0.12569573	3.20353467	2.17192555	0.03033478	0.08745579	-5.839116	1.518059166	up-regulated in Low
TMEM231	-0.3013921	1.45918547	-4.8551122	1.62E-06	1.48E-05	3.33910319	5.791728634	up-regulated in High
GABARAPL2	-0.1402422	4.6504189	-3.1747676	0.00159287	0.00717227	-3.1942796	2.797820228	up-regulated in High
KARS	0.30129318	5.16124237	8.02748904	7.28E-15	2.47E-13	22.1381502	14.13797323	up-regulated in Low
TERF2IP	-0.2560812	4.33384449	-6.5542055	1.41E-10	2.62E-09	12.4341577	9.852056538	up-regulated in High
NUDT7	-0.2213748	1.38207799	-4.4219854	1.20E-05	9.17E-05	1.41142382	4.919788996	up-regulated in High
MAF	-0.2930865	2.68905961	-4.563006	6.37E-06	5.13E-05	2.02090953	5.196161404	up-regulated in High
DYNLRB2	-0.4306398	0.68705346	-6.0842368	2.34E-09	3.60E-08	9.68274446	8.630104605	up-regulated in High
CMC2	0.31538077	1.44412357	9.88391009	3.80E-21	2.89E-19	36.4465781	20.42065311	up-regulated in Low
CENPN	0.85025749	1.90072024	17.6198196	2.61E-54	1.88E-51	112.460693	53.58313159	up-regulated in Low
GCSH	0.33799107	1.62014356	8.41246764	4.32E-16	1.75E-14	24.9260348	15.36467817	up-regulated in Low
PKD1L2	0.17718765	0.26531851	4.97088611	9.20E-07	8.84E-06	3.88214315	6.036328957	up-regulated in Low
BCO1	-0.1605156	0.81047238	-2.4052025	0.01652908	0.05304112	-5.310182	1.781751409	up-regulated in High
CMIP	0.16208833	3.06527461	2.80883521	0.00516847	0.01989527	-4.2709249	2.286637975	up-regulated in Low
PLCG2	-0.3346433	1.53026513	-6.4448027	2.75E-10	4.89E-09	11.7781659	9.561077367	up-regulated in High
SDR42E1	-0.231738	0.91703845	-5.3918843	1.08E-07	1.24E-06	5.95375694	6.966176544	up-regulated in High
MPHOSPH6	0.27219074	2.93203582	6.32349372	5.71E-10	9.62E-09	11.0617122	9.243034322	up-regulated in Low
HSBP1	0.14648815	3.71177693	2.75677913	0.00605269	0.02277345	-4.4137361	2.218051588	up-regulated in Low
OSGIN1	0.51501817	2.44270404	4.31809904	1.90E-05	0.00013912	0.97374547	4.720870858	up-regulated in Low
NECAB2	0.33785281	0.329293	6.78239638	3.38E-11	6.90E-10	13.8321452	10.47150841	up-regulated in Low
SLC38A8	0.20594377	0.232827	3.52218204	0.00046758	0.00244128	-2.0553596	3.330140532	up-regulated in Low
DNAAF1	-0.3330631	0.59468699	-4.9179959	1.19E-06	1.12E-05	3.63262076	5.923986636	up-regulated in High
WFDC1	-0.2053004	0.62925035	-5.1487536	3.79E-07	3.91E-06	4.73894365	6.421489634	up-regulated in High
TLDC1	0.26322774	1.87290521	5.91638861	6.14E-09	8.74E-08	8.74269694	8.211607392	up-regulated in Low
USP10	0.3059359	3.84876647	7.81362868	3.35E-14	1.03E-12	20.6328727	13.47493744	up-regulated in Low
ZDHHC7	-0.2854636	4.52014594	-5.2457527	2.31E-07	2.49E-06	5.21759556	6.636288465	up-regulated in High
KIAA0513	-0.4089006	1.98996513	-7.6072252	1.42E-13	4.02E-12	19.2102454	12.8478002	up-regulated in High
FAM92B	-0.5336696	0.99271607	-5.5164707	5.58E-08	6.75E-07	6.5955676	7.253357167	up-regulated in High

GINS2	1.06220077	2.04237786	15.8628089	3.99E-46	1.65E-43	93.6662415	45.39875871	up-regulated in Low
C16orf74	0.38980241	1.20965385	4.62882518	4.70E-06	3.89E-05	2.31139839	5.327647166	up-regulated in Low
EMC8	0.32160948	2.8232266	9.3790364	2.38E-19	1.44E-17	32.3463999	18.62324419	up-regulated in Low
COX4I1	0.17828243	5.58530942	3.40933241	0.00070434	0.00349637	-2.4376858	3.152217835	up-regulated in Low
IRF8	-0.5557212	2.43139274	-6.9527432	1.14E-11	2.48E-10	14.9016078	10.94484165	up-regulated in High
FOXF1	-0.4931402	1.61534741	-8.4338515	3.68E-16	1.50E-14	25.0837778	15.43404162	up-regulated in High
FBXO31	-0.1477996	2.34888654	-4.1765285	3.50E-05	0.00024188	0.39286051	4.456217441	up-regulated in High
MAP1LC3B	-0.1138353	4.09347848	-2.8035014	0.00525336	0.02017282	-4.2856768	2.279563091	up-regulated in High
ZCCHC14	-0.1666853	2.82948817	-3.5585043	0.00040886	0.00216918	-1.9297829	3.38843105	up-regulated in High
SLC7A5	1.30895102	4.40462085	12.3246441	1.25E-30	2.13E-28	58.1340251	29.90464972	up-regulated in Low
BANP	0.21071231	1.39667798	6.90685677	1.53E-11	3.28E-10	14.6113721	10.81643034	up-regulated in Low
ZFPM1	-0.1503286	0.8887441	-3.5844935	0.00037116	0.00198868	-1.8391792	3.43044464	up-regulated in High
ZC3H18	0.16483475	2.7804063	4.1497024	3.92E-05	0.00026798	0.28482097	4.406905837	up-regulated in Low
IL17C	0.14764163	0.41543908	2.38901953	0.0172665	0.05499519	-5.3485779	1.762795597	up-regulated in Low
MVD	0.13432251	3.14196414	2.2272097	0.02638169	0.07801598	-5.7185396	1.578697471	up-regulated in Low
SNAI3	-0.3244779	1.19888559	-7.6433289	1.11E-13	3.19E-12	19.4569252	12.95658166	up-regulated in High
RNF166	-0.167101	2.56834391	-3.2955024	0.00105286	0.00498081	-2.8112911	2.977630977	up-regulated in High
CTU2	0.24409482	2.47403011	5.77274486	1.38E-08	1.84E-07	7.95635643	7.86107807	up-regulated in Low
CDT1	1.24672183	2.39600747	18.4499635	3.02E-58	2.94E-55	121.505617	57.52024032	up-regulated in Low
APRT	0.19722077	5.77975014	3.29898052	0.00104018	0.00492841	-2.8000551	2.982892885	up-regulated in Low
CBFA2T3	-0.3033568	0.99251399	-5.4366044	8.54E-08	9.97E-07	6.1826381	7.068633984	up-regulated in High
ACSF3	-0.1241554	1.65711762	-3.4392781	0.00063247	0.00317908	-2.3373884	3.19896193	up-regulated in High
CDH15	-0.304849	0.84601939	-4.4887453	8.92E-06	6.97E-05	1.69775929	5.049715422	up-regulated in High
SLC22A31	-1.7325244	4.60789033	-10.559372	1.20E-23	1.17E-21	42.154142	22.91987704	up-regulated in High
ZNF778	-0.1756251	0.93530984	-4.9401685	1.07E-06	1.01E-05	3.73693092	5.970960167	up-regulated in High
DPEP1	-0.1849052	0.86723068	-2.5125355	0.01230319	0.04148935	-5.0491205	1.909982158	up-regulated in High
CHMP1A	0.15240585	5.09830225	3.36934163	0.00081223	0.00396054	-2.5703219	3.090322886	up-regulated in Low
CDK10	-0.1643097	3.03385501	-2.5272497	0.01180589	0.04007387	-5.0124655	1.927901159	up-regulated in High
ZNF276	-0.1606597	1.81255082	-3.1752282	0.00159039	0.00716278	-3.1928444	2.798495745	up-regulated in High
FANCA	0.59028179	1.3661348	13.1716568	3.49E-34	7.36E-32	66.2720239	33.45675677	up-regulated in Low
TCF25	-0.1828321	4.09938486	-3.9719365	8.19E-05	0.00052164	-0.4146598	4.086905418	up-regulated in High
TUBB3	0.42954333	1.37365654	5.69660888	2.10E-08	2.71E-07	7.54641806	7.67815817	up-regulated in Low
TUBB3	0.42954333	1.37365654	5.69660888	2.10E-08	2.71E-07	7.54641806	7.67815817	up-regulated in Low
DBNDD1	0.2520423	2.88645684	3.51242036	0.00048466	0.00251941	-2.0888997	3.314559766	up-regulated in Low
FAM101B	-0.1349515	2.47024432	-2.1564904	0.03152577	0.09023014	-5.8722495	1.501334356	up-regulated in High
GEMIN4	0.11040766	2.65877577	2.5169643	0.01215159	0.04104448	-5.0381097	1.915366935	up-regulated in Low
GLOD4	0.1903852	3.43920165	5.50125795	6.05E-08	7.27E-07	6.51650266	7.217999529	up-regulated in Low
RNMTL1	0.24789727	2.74406078	6.22846464	1.01E-09	1.63E-08	10.5085629	8.997298058	up-regulated in Low
NXN	-0.3020966	3.38474323	-4.1430496	4.03E-05	0.0002749	0.25812781	4.394718006	up-regulated in High
TIMM22	0.17902169	2.77693876	5.70480495	2.01E-08	2.61E-07	7.5903191	7.697753582	up-regulated in Low
ABR	-0.2728614	3.43774508	-4.8765834	1.46E-06	1.34E-05	3.43893722	5.836726889	up-regulated in High
YWHAE	0.29875025	6.78819088	7.08367937	4.84E-12	1.12E-10	15.7384524	11.3149193	up-regulated in Low
MYO1C	-0.1158103	4.82326399	-2.3334478	0.02002371	0.06213212	-5.4784983	1.698455461	up-regulated in High
INPP5K	-0.2692269	3.10033861	-7.0594645	5.67E-12	1.30E-10	15.5827267	11.24607182	up-regulated in High
PITPNA	-0.1138177	4.22446228	-3.2726836	0.00113966	0.00533741	-2.8847251	2.943223307	up-regulated in High
SLC43A2	-0.1738728	2.68929752	-3.3178213	0.00097392	0.00465224	-2.7389933	3.011475847	up-regulated in High
SCARF1	-0.556718	2.13455434	-8.1984263	2.10E-15	7.75E-14	23.3637651	14.67745353	up-regulated in High
RILP	-0.4025097	2.81619725	-6.9541602	1.13E-11	2.46E-10	14.9105955	10.94881766	up-regulated in High
TLCD2	-0.2146259	1.71585326	-3.8909792	0.00011348	0.00069749	-0.7236945	3.945081278	up-regulated in High
WDR81	-0.2057317	2.70089988	-3.8696584	0.00012356	0.00075256	-0.8040857	3.908138818	up-regulated in High
SERPINF2	-0.3139098	2.79547272	-2.8203478	0.00498947	0.01928891	-4.2389918	2.301945278	up-regulated in High
SERPINF1	-0.3733422	5.20108218	-3.4084202	0.00070664	0.00350602	-2.4407279	3.150799263	up-regulated in High
RPA1	0.21762967	3.93829465	5.50527602	5.92E-08	7.13E-07	6.53736695	7.227330527	up-regulated in Low
RTN4RL1	-0.5258121	0.96064578	-7.0883118	4.70E-12	1.09E-10	15.7682934	11.32811126	up-regulated in High
HIC1	-0.1498159	1.08743661	-3.6164237	0.0003293	0.00179253	-1.7270075	3.482410977	up-regulated in High
SMG6	-0.1146419	1.58281666	-3.0591721	0.00233967	0.0100129	-3.5480974	2.630846253	up-regulated in High
TSR1	0.3633991	3.06795552	9.26619813	5.89E-19	3.39E-17	31.4504086	18.23020075	up-regulated in Low
SGSM2	-0.2954888	2.79612906	-4.4038432	1.30E-05	9.86E-05	1.33429574	4.884764114	up-regulated in High
CLUH	0.324463	3.65710638	5.58497105	3.86E-08	4.78E-07	6.95397165	7.413566133	up-regulated in Low
RAP1GAP2	0.29345971	2.24760978	3.92811935	9.78E-05	0.00061119	-0.5826633	4.00984045	up-regulated in Low
ASPA	-0.1602168	0.24580019	-7.4666924	3.73E-13	9.97E-12	18.2588749	12.42810681	up-regulated in High
TRPV3	0.12811175	0.24296449	4.90735525	1.25E-06	1.17E-05	3.58271311	5.901506775	up-regulated in Low
TAX1BP3	-0.1245956	4.53421236	-2.5187853	0.01208974	0.04086629	-5.0335769	1.91758319	up-regulated in High
EMC6	0.37749576	2.08851447	7.62682859	1.24E-13	3.55E-12	19.344072	12.90681738	up-regulated in Low
ITGAE	0.14268359	1.95787775	2.90258978	0.0038658	0.01548841	-4.0071956	2.412760347	up-regulated in Low
GSG2	0.66853737	0.93589776	17.3621392	4.28E-53	2.90E-50	109.671805	52.36898003	up-regulated in Low
C17orf85	0.12022656	2.05933875	2.75709333	0.00604697	0.02275778	-4.4128819	2.218462476	up-regulated in Low
CAMKK1	-0.1512391	1.33541686	-3.2766773	0.00112401	0.0052717	-2.8719082	2.949230994	up-regulated in High
P2RX1	-0.2809561	0.91905426	-5.5423003	4.86E-08	5.92E-07	6.73025332	7.313575596	up-regulated in High
CYB5D2	-0.235042	2.9417463	-4.3274121	1.83E-05	0.00013426	1.0125887	4.738540821	up-regulated in High
ANKFY1	-0.1340086	2.49560999	-3.34003	0.00090086	0.0043384	-2.6665893	3.045340924	up-regulated in High

UBE2G1	0.2754244	3.43795293	7.18600766	2.47E-12	5.93E-11	16.4013172	11.60788311	up-regulated in Low
SPNS3	-0.2834235	0.5973404	-8.3139986	8.98E-16	3.46E-14	24.2035414	15.04692044	up-regulated in High
SPNS2	-0.4366354	2.75747054	-3.963042	8.49E-05	0.00053791	-0.4489045	4.071203868	up-regulated in High
MYBBP1A	0.28788313	3.44441714	5.48547621	6.59E-08	7.86E-07	6.43468472	7.181404889	up-regulated in Low
GGT6	-0.9642585	1.84812074	-7.8745271	2.18E-14	6.89E-13	21.0582998	13.66238067	up-regulated in High
ALOX15	-0.4166299	0.95911357	-3.8133126	0.00015441	0.00091873	-1.0145384	3.811328602	up-regulated in High
PELP1	0.30015378	3.5078858	6.2386601	9.47E-10	1.54E-08	10.5675668	9.023518541	up-regulated in Low
ARRB2	-0.2425447	3.76799293	-4.9778811	8.89E-07	8.56E-06	3.91532473	6.051262131	up-regulated in High
CXCL16	-0.5925175	5.56127416	-8.6344944	8.11E-17	3.57E-15	26.5783815	16.09103455	up-regulated in High
ZMYND15	-0.414455	1.47852606	-7.9526232	1.25E-14	4.11E-13	21.6076197	13.90434787	up-regulated in High
VMO1	-0.2326316	2.47859156	-2.4298124	0.01546081	0.05020357	-5.2513073	1.810767705	up-regulated in High
PSMB6	0.43688745	5.86732756	9.84625589	5.19E-21	3.87E-19	36.1357457	20.28445715	up-regulated in Low
PLD2	-0.1226296	2.8964768	-2.8866982	0.00406306	0.01616776	-4.0524877	2.391147332	up-regulated in High
C17orf107	-0.2035358	1.23891143	-4.0053826	7.14E-05	0.00046145	-0.2852448	4.146212543	up-regulated in High
GP1BA	-0.1722185	0.64144762	-4.3903046	1.38E-05	0.00010425	1.27693038	4.858705955	up-regulated in High
SLC25A11	0.21434873	4.39705481	5.20365334	2.87E-07	3.03E-06	5.00886819	6.542651358	up-regulated in Low
PFN1	0.28265588	8.17386459	7.38299938	6.59E-13	1.70E-11	17.6990188	12.18100776	up-regulated in Low
ENO3	0.36762178	1.45961998	3.29758675	0.00104524	0.00494919	-2.804559	2.980783775	up-regulated in Low
CAMTA2	-0.1803161	3.16897662	-3.984916	7.76E-05	0.00049738	-0.3645581	4.109871339	up-regulated in High
INCA1	-0.1586899	1.62475623	-3.6976015	0.00024196	0.00136799	-1.4375826	3.616257713	up-regulated in High
KIF1C	-0.2379445	4.06034836	-4.4153409	1.24E-05	9.42E-05	1.38314216	4.906947306	up-regulated in High
SLC52A1	-0.3139004	0.81477632	-5.610073	3.37E-08	4.21E-07	7.08628537	7.472683586	up-regulated in High
ZFP3	-0.2489812	1.34486067	-5.4761762	6.92E-08	8.22E-07	6.38656759	7.159880761	up-regulated in High
ZNF232	0.12570069	1.97693552	2.84695836	0.00459706	0.01796937	-4.1646962	2.337519934	up-regulated in Low
SCIMP	-0.3139772	1.43677829	-4.9631573	9.55E-07	9.15E-06	3.8455298	6.01984968	up-regulated in High
NUP88	0.25408637	3.08870699	6.14234304	1.67E-09	2.62E-08	10.0134516	8.777199156	up-regulated in Low
C1QBP	0.55139426	4.90364571	9.56380967	5.32E-20	3.49E-18	33.8298695	19.27377868	up-regulated in Low
DHX33	0.34532234	2.33033284	8.58187614	1.21E-16	5.24E-15	26.1839009	15.91766799	up-regulated in Low
DERL2	0.11317099	2.91657748	3.07907857	0.0021916	0.00946526	-3.4880668	2.659238944	up-regulated in Low
MIS12	0.14777276	2.89284915	3.71505968	0.00022627	0.00129005	-1.374544	3.645367112	up-regulated in Low
NLRP1	-0.5976494	1.68834401	-9.3585968	2.81E-19	1.68E-17	32.1835363	18.55180855	up-regulated in High
FAM64A	1.17351087	1.54400175	18.5390511	1.14E-58	1.14E-55	122.481136	57.94481511	up-regulated in Low
PITPNM3	-0.3927035	1.39547265	-7.2090006	2.12E-12	5.12E-11	16.5513235	11.67416029	up-regulated in High
SLC13A5	0.15556126	0.13830247	4.51803938	7.81E-06	6.18E-05	1.82465138	5.107243511	up-regulated in Low
XAF1	-0.1583887	1.46804049	-2.3061305	0.02151516	0.06598413	-5.5412663	1.667255375	up-regulated in High
TEKT1	-0.5067638	0.69726051	-5.6557682	2.63E-08	3.34E-07	7.32848657	7.580861033	up-regulated in High
BCL6B	-0.1795652	1.90863869	-3.1581196	0.00168477	0.00752283	-3.2460134	2.773460127	up-regulated in High
SLC16A13	0.15066255	2.17466041	2.71943768	0.00676883	0.02502132	-4.514583	2.169486257	up-regulated in Low
SLC16A11	-0.4635577	1.0050565	-6.7266647	4.80E-11	9.58E-10	13.487029	10.31866518	up-regulated in High
CLEC10A	-0.5384343	1.91777556	-6.2416854	9.30E-10	1.52E-08	10.585091	9.031305656	up-regulated in High
ASGR2	0.13193588	0.46026733	2.5069678	0.01249617	0.04202602	-5.0629359	1.90322319	up-regulated in Low
DLG4	-0.1824638	1.58872595	-3.6107738	0.00033637	0.00182505	-1.7469244	3.473187824	up-regulated in High
DVL2	0.2906503	2.84904609	7.142219	3.30E-12	7.77E-11	16.1167154	11.48211706	up-regulated in Low
PHF23	0.14229516	3.87818066	3.90549432	0.00010707	0.00066266	-0.6687266	3.970329011	up-regulated in Low
GABARAP	-0.1017958	4.72834562	-2.6662697	0.00792044	0.02854185	-4.6558681	2.101250717	up-regulated in High
CTDNEP1	0.10624324	5.1828514	3.33439251	0.00091891	0.00441455	-2.6850122	3.036726855	up-regulated in Low
ELP5	0.1985495	3.36933361	4.91874822	1.19E-06	1.11E-05	3.63615292	5.925577496	up-regulated in Low
YBX2	0.56579493	0.91480328	6.69347038	5.91E-11	1.16E-09	13.2826011	10.22810556	up-regulated in Low
EIF5A	0.62914255	6.944866	13.2079747	2.45E-34	5.19E-32	66.6269323	33.61160991	up-regulated in Low
ACAP1	-0.2953898	1.74058953	-4.2607133	2.44E-05	0.00017434	0.73611411	4.612698898	up-regulated in High
TMEM256	0.29182022	4.97440081	4.13798704	4.12E-05	0.00028043	0.23784224	4.385454613	up-regulated in Low
ZBTB4	-0.5462665	4.19791218	-11.111766	9.15E-26	1.09E-23	46.9979484	25.03878455	up-regulated in High
POLR2A	0.12698697	4.77996084	2.06648112	0.03930202	0.10775897	-6.0608438	1.405585105	up-regulated in Low
TNFSF12	-0.6193951	3.82472691	-11.563609	1.52E-27	2.08E-25	51.0697651	26.81875722	up-regulated in High
TNFSF13	-0.5983569	3.27691628	-9.920225	2.80E-21	2.15E-19	36.7471079	20.55232539	up-regulated in High
SENP3	0.31212055	2.70196517	9.78732911	8.47E-21	6.06E-19	35.6509135	20.07200042	up-regulated in Low
CD68	-0.1058249	1.04275266	-2.118479	0.03463153	0.09723641	-5.952857	1.460528316	up-regulated in High
MPDU1	0.17184468	4.43997935	3.39344422	0.0007455	0.00367847	-2.4905605	3.127554727	up-regulated in Low
SOX15	0.4978445	1.11015994	6.18859772	1.27E-09	2.03E-08	10.2786348	8.895102452	up-regulated in Low
FXR2	0.19955803	3.39456787	4.81802661	1.93E-06	1.73E-05	3.1676116	5.714399101	up-regulated in Low
ATP1B2	-0.35954	0.70209104	-8.7465728	3.44E-17	1.58E-15	27.4245537	16.46282314	up-regulated in High
WRAP53	0.27808798	1.78608859	7.84118963	2.76E-14	8.61E-13	20.8250905	13.55963402	up-regulated in Low
EFNB3	-0.2107388	1.20930377	-2.6934518	0.00731136	0.02666717	-4.5839746	2.136002099	up-regulated in High
DNAH2	-0.3814723	1.14597353	-5.8696102	8.00E-09	1.11E-07	8.48477354	8.096680465	up-regulated in High
TMEM88	-0.1858851	1.61847407	-2.9737788	0.00308491	0.01272295	-3.8013544	2.51075698	up-regulated in High
NAA38	0.16891432	4.15137017	2.57119642	0.01042538	0.0360286	-4.9017474	1.981907988	up-regulated in Low
CNTROB	-0.1524054	2.94482181	-2.905902	0.00382581	0.01535344	-3.9977252	2.417277195	up-regulated in High
GUCY2D	-0.1574833	0.45180465	-4.6762393	3.77E-06	3.18E-05	2.52302115	5.423345315	up-regulated in High
ALOX15B	-1.5086289	3.5610047	-9.9857284	1.62E-21	1.27E-19	37.2910505	20.79062192	up-regulated in High
ALOXE3	0.10529271	0.20632399	4.27265295	2.32E-05	0.00016649	0.78531247	4.635104808	up-regulated in Low
PER1	-0.4381279	3.72258548	-4.533642	7.28E-06	5.80E-05	1.89254671	5.138012469	up-regulated in High

VAMP2	-0.467327	4.1824305	-9.0488703	3.29E-18	1.73E-16	29.7463642	17.48239759	up-regulated in High
AURKB	1.51478934	2.56857464	19.5394621	1.84E-63	3.71E-60	133.487951	62.73469135	up-regulated in Low
PFAS	0.22866794	2.33069531	4.70815	3.25E-06	2.77E-05	2.66655905	5.488213191	up-regulated in Low
ARHGEF15	-0.3968316	1.46219553	-7.4346769	4.64E-13	1.22E-11	18.0441148	12.33333079	up-regulated in High
MYH10	-0.2963534	3.51779994	-3.7468349	0.00020015	0.00115728	-1.2590885	3.698642555	up-regulated in High
PIK3R6	-0.2935149	1.04917185	-6.2838773	7.24E-10	1.20E-08	10.8302436	9.1402249	up-regulated in High
PIK3R5	-0.3769729	1.47936302	-6.8047434	2.93E-11	6.04E-10	13.971192	10.53307477	up-regulated in High
NTN1	-0.1954211	1.52946627	-2.1435984	0.03255115	0.09248036	-5.8997462	1.487433716	up-regulated in High
CFAP52	-0.3797342	0.61331832	-5.3237049	1.54E-07	1.72E-06	5.60804762	6.811325668	up-regulated in High
GAS7	-0.6133792	2.00059775	-9.3314232	3.49E-19	2.06E-17	31.9674003	18.45700132	up-regulated in High
SCO1	0.15683483	1.9873982	5.28489942	1.89E-07	2.07E-06	5.41303308	6.723921588	up-regulated in Low
ADPRM	-0.1041094	2.12322943	-2.7009139	0.00715169	0.02616984	-4.5641144	2.145591126	up-regulated in High
TMEM220	-0.251128	1.19180124	-4.7422521	2.77E-06	2.40E-05	2.82094041	5.557945081	up-regulated in High
DNAH9	-0.2986534	0.31907556	-5.2628721	2.12E-07	2.29E-06	5.30290336	6.67454495	up-regulated in High
MYOCD	-0.2062012	0.26871716	-7.7641998	4.75E-14	1.44E-12	20.2894664	13.32360054	up-regulated in High
ARHGAP44	-0.5017513	1.67351957	-7.5090311	2.79E-13	7.60E-12	18.5440129	12.5539209	up-regulated in High
ELAC2	0.2083275	3.36032477	5.84681334	9.10E-09	1.25E-07	8.35972336	8.040943193	up-regulated in Low
COX10	0.18482588	2.46228387	5.40105493	1.03E-07	1.19E-06	6.00055619	6.987130023	up-regulated in Low
HS3ST3B1	-0.107513	0.9483862	-2.2605689	0.024219	0.07278539	-5.6443453	1.615843836	up-regulated in High
PMP22	-0.5134859	4.53055018	-6.8902968	1.70E-11	3.62E-10	14.5070176	10.7702521	up-regulated in High
TEKT3	-0.1845457	0.2630901	-6.2565488	8.52E-10	1.40E-08	10.6712926	9.069608123	up-regulated in High
TRIM16	0.5077527	2.0590812	6.85493193	2.13E-11	4.47E-10	14.2848545	10.67192784	up-regulated in Low
ZNF286A	0.14669149	0.72983515	5.07756002	5.42E-07	5.43E-06	4.39274041	6.265966532	up-regulated in Low
ZNF286A	0.14669149	0.72983515	5.07756002	5.42E-07	5.43E-06	4.39274041	6.265966532	up-regulated in Low
ADORA2B	-0.2071624	2.30376115	-2.7792167	0.00565603	0.02149713	-4.3524981	2.247487991	up-regulated in High
ZSWIM7	-0.1663861	2.7814044	-3.6035204	0.00034565	0.00186917	-1.7724509	3.46136453	up-regulated in High
TTC19	-0.1710617	3.10765718	-3.7853772	0.00017228	0.00101184	-1.1177979	3.763773395	up-regulated in High
CENPV	0.21330316	2.28390227	2.551885	0.01101322	0.03778421	-4.950629	1.958085724	up-regulated in Low
TRPV2	-0.3392648	2.92871029	-4.7663707	2.47E-06	2.16E-05	2.93074026	5.607517709	up-regulated in High
ZNF287	-0.1060206	1.03521159	-2.764656	0.00591067	0.02231097	-4.3922929	2.228363642	up-regulated in High
TNFRSF13B	-0.2967027	0.47135082	-6.7400073	4.41E-11	8.86E-10	13.5694368	10.35516603	up-regulated in High
MPRIP	-0.2476592	3.29045527	-4.3021302	2.04E-05	0.00014825	0.90732321	4.690647456	up-regulated in High
FLCN	-0.1107229	2.02030912	-2.6445408	0.0084397	0.0301336	-4.7128289	2.073672773	up-regulated in High
COPS3	0.33733045	4.15292247	7.42944751	4.81E-13	1.26E-11	18.009106	12.31787976	up-regulated in Low
NT5M	0.1624571	1.14756802	3.15030294	0.00172958	0.00769939	-3.2702137	2.762058709	up-regulated in Low
PEMT	0.15441335	3.39032003	2.58511782	0.01001916	0.03488511	-4.8662865	1.999168904	up-regulated in Low
TOM1L2	-0.4515882	2.72756371	-8.5425925	1.63E-16	6.92E-15	25.8905556	15.78873109	up-regulated in High
LRRC48	-0.3086398	0.83004991	-5.6544097	2.64E-08	3.36E-07	7.32126134	7.577634627	up-regulated in High
DRG2	0.17050523	2.16760422	5.15068454	3.75E-07	3.88E-06	4.74839361	6.425732848	up-regulated in Low
ALKBH5	0.10599331	4.9080182	2.7022771	0.00712287	0.02608397	-4.5604805	2.147345188	up-regulated in Low
LLGL1	0.1388875	2.60342029	2.62103973	0.00903551	0.03194794	-4.7739249	2.044047332	up-regulated in Low
MIEF2	-0.1770124	2.2680881	-4.131288	4.23E-05	0.00028781	0.21103481	4.373211414	up-regulated in High
TOP3A	0.27570301	2.28714952	6.68584667	6.20E-11	1.21E-09	13.2357694	10.20735703	up-regulated in Low
SHMT1	0.12110934	2.57274851	1.99598967	0.04648183	0.12322624	-6.2030242	1.332716768	up-regulated in Low
EVPLL	-0.1321679	0.48330631	-3.3622835	0.0008328	0.0040463	-2.5935761	3.079461726	up-regulated in High
TRIM16L	0.84472603	1.74772178	8.23444477	1.61E-15	6.02E-14	23.6245276	14.79219329	up-regulated in Low
TVP23B	0.12675797	2.45518235	2.87303355	0.00423995	0.01678016	-4.0912406	2.37263974	up-regulated in Low
PRPSAP2	0.22220009	2.79185898	4.90169716	1.29E-06	1.20E-05	3.55621507	5.889569906	up-regulated in Low
FAM83G	0.32343585	2.12431537	5.85481736	8.70E-09	1.20E-07	8.40358048	8.060492413	up-regulated in Low
GRAP	-0.103679	0.54561504	-3.9199344	0.00010104	0.00062992	-0.6138521	3.995524416	up-regulated in High
MFAP4	-1.6509975	4.8307442	-14.3184	3.66E-39	1.03E-36	77.6911619	38.43708726	up-regulated in High
SLC47A1	-0.6358859	1.5010243	-7.2252318	1.90E-12	4.63E-11	16.6574499	11.72104559	up-regulated in High
ALDH3A2	-0.3231132	4.60485317	-3.3769692	0.00079053	0.00386572	-2.545139	3.102081656	up-regulated in High
ULK2	-0.1630053	1.6933325	-3.6859589	0.00025298	0.00142206	-1.479466	3.596908835	up-regulated in High
SPECC1	0.2575671	1.59496409	5.01431607	7.42E-07	7.25E-06	4.08884005	6.129328702	up-regulated in Low
DHRS7B	-0.1000417	2.49656137	-2.1070093	0.03561871	0.09950573	-5.9769034	1.448321802	up-regulated in High
TMEM11	0.26182803	2.95961645	6.9581779	1.10E-11	2.40E-10	14.9360877	10.96009476	up-regulated in Low
NATD1	-0.5012783	2.01921123	-9.3832825	2.30E-19	1.39E-17	32.3802638	18.63809725	up-regulated in High
KCNJ18	0.17457269	0.08117716	4.53680266	7.17E-06	5.72E-05	1.9063268	5.144256333	up-regulated in Low
WSB1	-0.302223	4.77315143	-3.0575467	0.00235215	0.01005823	-3.5529824	2.628534583	up-regulated in High
LGALS9	-0.5517332	3.83904344	-6.4528043	2.62E-10	4.68E-09	11.8258281	9.582226008	up-regulated in High
LYRM9	-0.2706195	1.05818677	-8.0071929	8.42E-15	2.83E-13	21.9939441	14.07447663	up-regulated in High
TMEM97	0.36355345	3.87950866	4.73755574	2.83E-06	2.45E-05	2.79961928	5.548316836	up-regulated in Low
POLDIP2	0.2758079	5.82378101	7.59931326	1.50E-13	4.24E-12	19.1563102	12.82401358	up-regulated in Low
TMEM199	0.1518199	2.27157724	5.36307195	1.26E-07	1.42E-06	5.80718378	6.900537509	up-regulated in Low
SARM1	-0.2004429	0.76732035	-6.8672589	1.97E-11	4.16E-10	14.3621854	10.7061549	up-regulated in High
VTN	0.20189747	0.58238905	2.35567221	0.01887789	0.05922747	-5.4268989	1.724046616	up-regulated in Low
SLC46A1	-0.14392	1.41047949	-3.5619329	0.00040368	0.00214467	-1.9178659	3.393959083	up-regulated in High
SLC13A2	-0.2757622	0.57148969	-2.5749164	0.01031542	0.03573128	-4.8922901	1.986513105	up-regulated in High
SPAG5	1.29191876	2.55427865	18.8986831	2.18E-60	2.70E-57	126.427467	59.66228098	up-regulated in Low
KIAA0100	0.12217455	4.2762234	2.37902529	0.01773624	0.05624408	-5.3721638	1.751138374	up-regulated in Low

RPL23A	0.13534885	7.18830646	2.65166867	0.00826609	0.02959483	-4.6941935	2.082699652	up-regulated in Low
TLCD1	0.17797043	4.00614177	2.32229684	0.02062123	0.06368073	-5.5042077	1.685685527	up-regulated in Low
NEK8	-0.2393369	1.79989796	-4.9232636	1.16E-06	1.09E-05	3.65736377	5.935130367	up-regulated in High
TRAF4	0.25252464	4.6547477	4.51316928	7.99E-06	6.31E-05	1.80350304	5.097657726	up-regulated in Low
ERAL1	0.31680131	4.55612678	8.68066821	5.70E-17	2.55E-15	26.9260135	16.24379031	up-regulated in Low
DHRS13	0.1344118	1.50882853	3.03585439	0.00252476	0.01070384	-3.6179381	2.597779499	up-regulated in Low
TIAF1	-0.1281447	0.65712384	-3.6877371	0.00025127	0.00141392	-1.473077	3.599860804	up-regulated in High
TAOK1	0.11015199	2.63381213	2.60667974	0.00941785	0.03311533	-4.8109958	2.026048204	up-regulated in Low
ABHD15	-0.1321072	2.82390093	-3.2007973	0.00145842	0.00664527	-3.1128686	2.836118878	up-regulated in High
GIT1	0.24977088	3.92580291	5.00930916	7.61E-07	7.42E-06	4.0649277	6.118572525	up-regulated in Low
ANKRD13B	0.15625793	1.41458546	2.471113	0.01380454	0.04569711	-5.1511883	1.859978052	up-regulated in Low
CORO6	0.10181337	0.40284577	3.2230941	0.00135167	0.00621808	-3.0426256	2.869129541	up-regulated in Low
SLC6A4	-0.2185634	0.30658646	-3.8722131	0.00012231	0.00074619	-0.7944751	3.91255634	up-regulated in High
BLMH	0.23878967	2.72736676	3.94152874	9.26E-05	0.00058204	-0.531435	4.033348434	up-regulated in Low
CPD	0.34247296	4.50395102	3.64494751	0.00029569	0.00163151	-1.6260047	3.529158434	up-regulated in Low
GOSR1	0.14922042	2.71525516	4.4390611	1.11E-05	8.56E-05	1.48428497	4.952865316	up-regulated in Low
ATAD5	0.42946083	1.08465825	10.9195238	5.08E-25	5.69E-23	45.2950107	24.29403438	up-regulated in Low
TEFM	0.18761133	1.66336843	5.66621705	2.48E-08	3.16E-07	7.38411217	7.605699092	up-regulated in Low
ADAP2	-0.3042176	2.48664405	-5.1455368	3.85E-07	3.97E-06	4.72320737	6.414423522	up-regulated in High
RNF135	-0.18146	3.23000384	-4.1043424	4.74E-05	0.00031927	0.10361601	4.324133738	up-regulated in High
OMG	-0.442853	0.68879373	-6.3607578	4.57E-10	7.83E-09	11.2805656	9.340214304	up-regulated in High
EVI2B	-0.6449984	3.53426012	-7.6853495	8.25E-14	2.43E-12	19.7451922	13.08368197	up-regulated in High
EVI2A	-0.4842894	2.70473841	-5.9437441	5.26E-09	7.56E-08	8.89435122	8.279161086	up-regulated in High
RAB11FIP4	-0.1806599	1.9050731	-3.6530096	0.0002868	0.00158767	-1.5973206	3.542426865	up-regulated in High
COPRS	0.30794589	4.38703982	6.74342463	4.32E-11	8.70E-10	13.5905653	10.364524	up-regulated in Low
UTP6	0.29532419	3.11553889	9.1102391	2.03E-18	1.10E-16	30.2246353	17.69232222	up-regulated in Low
SUZ12	0.39130642	3.15814293	8.58779311	1.16E-16	5.02E-15	26.2281715	15.9371254	up-regulated in Low
C17orf75	0.22313172	2.16748573	5.84090783	9.41E-09	1.29E-07	8.32739826	8.026533466	up-regulated in Low
PSMD11	0.53300604	4.1933309	11.8762721	8.44E-29	1.25E-26	53.9418385	28.07368213	up-regulated in Low
CDK5R1	0.34635812	1.21045239	6.03883367	3.05E-09	4.59E-08	9.4262196	8.515959278	up-regulated in Low
MYO1D	-0.3634522	4.29560761	-5.195624	2.99E-07	3.15E-06	4.96922992	6.524863898	up-regulated in High
TMEM98	-0.798535	3.77395789	-9.4256218	1.64E-19	1.01E-17	32.7185205	18.78645232	up-regulated in High
CCL2	-0.2742588	4.22499034	-2.5906561	0.00986152	0.03444306	-4.8521275	2.006056152	up-regulated in High
CCL7	0.27877646	0.92058578	3.49296729	0.00052045	0.00267875	-2.1554742	3.283617769	up-regulated in Low
CCL8	0.3928877	1.78255025	3.69172402	0.00024747	0.00139508	-1.458742	3.606483561	up-regulated in Low
CCL13	-0.7580096	3.34475328	-5.4525451	7.85E-08	9.22E-07	6.26462921	7.105324914	up-regulated in High
TMEM132E	-0.1965999	0.24908081	-8.7179002	4.29E-17	1.94E-15	27.2073176	16.36738576	up-regulated in High
CCT6B	-0.1519784	0.56900673	-5.855886	8.65E-09	1.19E-07	8.40944003	8.063104191	up-regulated in High
LIG3	0.2814249	1.88532316	7.4312248	4.75E-13	1.25E-11	18.0210021	12.3231301	up-regulated in Low
RFFL	0.21794018	2.11702802	5.15119656	3.74E-07	3.87E-06	4.75090001	6.426858251	up-regulated in Low
RAD51D	0.11007506	1.34032914	4.01167858	6.96E-05	0.00045099	-0.2607694	4.157423492	up-regulated in Low
NLE1	0.35317619	2.21689862	9.54239869	6.34E-20	4.11E-18	33.6569445	19.19796068	up-regulated in Low
SLFN5	-0.355846	3.46548555	-4.9190188	1.19E-06	1.11E-05	3.63742346	5.926149735	up-regulated in High
SLFN12	-0.326628	1.56710936	-6.4207283	3.18E-10	5.59E-09	11.6350646	9.497573826	up-regulated in High
SLFN12L	-0.1355028	0.59389642	-3.9621129	8.52E-05	0.00053965	-0.4524777	4.069565325	up-regulated in High
AP2B1	0.12005157	5.22382408	2.33285875	0.02005489	0.06221811	-5.4798594	1.697779734	up-regulated in Low
TAF15	0.15924128	4.59177028	2.79627433	0.00537039	0.02056677	-4.3056215	2.269994198	up-regulated in Low
RASL10B	0.21012556	0.79260792	3.07864797	0.00219471	0.00947589	-3.4893693	2.65862319	up-regulated in Low
GAS2L2	-0.5009166	0.66494306	-6.9982472	8.46E-12	1.89E-10	15.1909846	11.07284151	up-regulated in High
MMP28	-1.132623	2.47795541	-7.786706	4.05E-14	1.24E-12	20.4456164	13.39241841	up-regulated in High
C17orf50	-0.2006776	0.31831738	-6.0537842	2.80E-09	4.22E-08	9.51050641	8.553468732	up-regulated in High
RDM1	0.34764216	0.50832155	10.2233069	2.17E-22	1.85E-20	39.2837204	21.66334866	up-regulated in Low
CCL14	-0.3361298	0.4506945	-8.7848377	2.57E-17	1.20E-15	27.7152789	16.59053425	up-regulated in High
CCL23	-0.3936121	1.11457549	-5.589007	3.78E-08	4.69E-07	6.97521013	7.423056423	up-regulated in High
ZNHIT3	0.19359217	2.45829887	5.75119754	1.55E-08	2.06E-07	7.83985614	7.809107137	up-regulated in Low
MYO19	0.49188496	2.24183629	10.2448352	1.81E-22	1.56E-20	39.4658021	21.74307629	up-regulated in Low
PIGW	0.26759348	2.39784451	6.05744093	2.74E-09	4.15E-08	9.53114905	8.562654475	up-regulated in Low
DHRS11	0.11026603	1.6478577	2.32730457	0.02035099	0.06298686	-5.4926769	1.691414515	up-regulated in Low
MRM1	0.2291973	2.53152164	4.59485462	5.50E-06	4.49E-05	2.16099444	5.259587126	up-regulated in Low
LHX1	0.17943486	0.13453901	4.56004789	6.45E-06	5.19E-05	2.00794377	5.190289196	up-regulated in Low
AATF	0.28636884	3.91314024	6.68331733	6.30E-11	1.23E-09	13.2202418	10.20047739	up-regulated in Low
ACACA	0.11182659	2.99825258	2.13165764	0.0335263	0.0947453	-5.9250696	1.474614415	up-regulated in Low
TADA2A	0.13130606	2.04548384	3.98947479	7.62E-05	0.00048875	-0.3469247	4.117952505	up-regulated in Low
DUSP14	0.32535665	3.09282752	5.76102925	1.47E-08	1.96E-07	7.8929662	7.832800868	up-regulated in Low
DDX52	0.2731635	2.39644976	7.6281367	1.23E-13	3.52E-12	19.3530118	12.91075964	up-regulated in Low
HNF1B	-0.814422	2.37004874	-9.2341623	7.60E-19	4.32E-17	31.1974194	18.11920355	up-regulated in High
MRPL45	0.33084168	4.55374299	7.69859824	7.52E-14	2.23E-12	19.8363384	13.12386487	up-regulated in Low
ARHGAP23	-0.3267801	2.47293542	-4.8367362	1.76E-06	1.60E-05	3.25397935	5.753349595	up-regulated in High
SRCIN1	-0.1583718	1.15095935	-2.4560402	0.01438985	0.0472888	-5.187918	1.841943753	up-regulated in High
C17orf96	0.51684153	1.54477346	9.16628517	1.30E-18	7.21E-17	30.6634357	17.88489434	up-regulated in Low
MLLT6	-0.1922812	3.76392782	-3.1806994	0.00156126	0.0070566	-3.1757833	2.806525321	up-regulated in High

CISD3	0.11238381	3.61661311	2.15640569	0.03153241	0.09024032	-5.8724308	1.501242782	up-regulated in Low
PSMB3	0.41678347	6.71170816	7.58560489	1.65E-13	4.63E-12	19.0629661	12.78284486	up-regulated in Low
PLXDC1	-0.3435112	1.40175557	-6.7715062	3.62E-11	7.36E-10	13.7645223	10.44156384	up-regulated in High
CACNB1	-0.7167678	1.7359817	-10.186597	2.97E-22	2.49E-20	38.9738139	21.5276438	up-regulated in High
RPL19	0.11821399	8.68657235	2.40379317	0.01659218	0.05321144	-5.313536	1.780096611	up-regulated in Low
FBXL20	-0.2416299	2.08397362	-5.7617753	1.46E-08	1.95E-07	7.89699929	7.834600047	up-regulated in High
MED1	0.19301769	2.98029031	3.49173477	0.0005228	0.00268871	-2.1596804	3.281662145	up-regulated in Low
CDK12	0.21322128	2.71633744	4.59741761	5.44E-06	4.45E-05	2.17230655	5.264707373	up-regulated in Low
PPP1R1B	-1.3526901	2.90316901	-8.1296156	3.47E-15	1.23E-13	22.8680191	14.45927869	up-regulated in High
PNMT	-0.1892321	0.49766586	-3.4268127	0.00066151	0.0033071	-2.3792407	3.179462637	up-regulated in High
PGAP3	-0.4690861	4.03139583	-7.0681523	5.36E-12	1.23E-10	15.638548	11.27075184	up-regulated in High
ERBB2	-0.3589822	4.90110564	-4.3677489	1.53E-05	0.00011429	1.18172086	4.815442225	up-regulated in High
MIEN1	0.32299957	4.33558322	5.06349273	5.81E-07	5.79E-06	4.32484708	6.235450532	up-regulated in Low
IKZF3	-0.3660591	1.88034702	-5.1558547	3.66E-07	3.78E-06	4.7737126	6.437101051	up-regulated in High
GSDMB	-0.3038531	2.32137078	-3.5183307	0.00047425	0.00247257	-2.068603	3.323989024	up-regulated in High
ORMDL3	-0.4908065	4.91863157	-9.2547765	6.45E-19	3.70E-17	31.36014	18.19059702	up-regulated in High
GSDMA	-0.1169401	0.64838095	-2.4324579	0.01534969	0.04990379	-5.2449437	1.813900482	up-regulated in High
PSMD3	0.3679796	4.99891048	8.51842369	1.95E-16	8.23E-15	25.7105753	15.70961515	up-regulated in Low
MED24	0.18392932	3.77350014	3.8903754	0.00011375	0.00069884	-0.7259768	3.944032747	up-regulated in Low
THRA	-0.3862298	2.7389996	-7.1834343	2.51E-12	6.02E-11	16.3845528	11.60047562	up-regulated in High
MSL1	0.16660777	3.66627381	4.15392001	3.85E-05	0.0002639	0.30176397	4.414640913	up-regulated in Low
CDC6	1.27081132	2.17988811	18.1705655	6.44E-57	5.97E-54	118.451932	56.19112195	up-regulated in Low
RARA	-0.3455596	3.73187998	-6.2265013	1.02E-09	1.65E-08	10.4972101	8.992252791	up-regulated in High
TOP2A	1.50315593	3.93477097	15.6057087	5.99E-45	2.36E-42	90.9662686	44.22253443	up-regulated in Low
IGFBP4	-0.6987042	7.57229024	-9.5495845	5.98E-20	3.90E-18	33.7149507	19.22339357	up-regulated in High
TNS4	0.59619058	1.95714343	3.8175121	0.00015188	0.00090496	-0.9989534	3.818502953	up-regulated in Low
CCR7	-0.5575236	1.86414937	-6.5328337	1.60E-10	2.96E-09	12.3052792	9.794905958	up-regulated in High
KRT15	-0.5626044	2.34405827	-4.1792621	3.46E-05	0.00023932	0.40390601	4.461257225	up-regulated in High
KRT16	0.35689742	1.51260359	2.43740543	0.01514375	0.04935244	-5.2330243	1.819766484	up-regulated in Low
EIF1	0.10128853	7.18566184	2.72818581	0.00659453	0.02447306	-4.4910769	2.180816366	up-regulated in Low
GAST	0.1685016	0.0895871	6.47212382	2.33E-10	4.18E-09	11.9411138	9.633375835	up-regulated in Low
HAP1	0.15198834	0.21850258	4.46200645	1.01E-05	7.78E-05	1.58260012	4.997480329	up-regulated in Low
JUP	0.26548329	6.29739777	3.66340322	0.0002757	0.0015332	-1.5602524	3.559568714	up-regulated in Low
P3H4	0.43997655	3.44617303	5.74542563	1.60E-08	2.12E-07	7.80871378	7.795212753	up-regulated in Low
FKBP10	0.53660015	4.6400569	4.5682714	6.21E-06	5.02E-05	2.04400733	5.206621685	up-regulated in Low
NT5C3B	0.23578583	4.0293757	4.5019981	8.40E-06	6.60E-05	1.75507179	5.075702464	up-regulated in Low
ACLY	0.41038267	5.16583305	7.88787045	1.98E-14	6.31E-13	21.1518574	13.70359636	up-regulated in Low
TTC25	-0.3580001	1.22157038	-4.9020102	1.29E-06	1.20E-05	3.55768035	5.890230012	up-regulated in High
CNP	0.22810931	4.01505884	5.5932178	3.69E-08	4.59E-07	6.997383	7.432963837	up-regulated in Low
DNAJC7	0.21269461	3.08699411	6.90145579	1.58E-11	3.38E-10	14.5773145	10.80135987	up-regulated in Low
NKIRAS2	0.33829294	3.40557648	8.75810984	3.15E-17	1.46E-15	27.5121109	16.50128709	up-regulated in Low
DHX58	-0.3125448	2.77919999	-4.8087667	2.02E-06	1.80E-05	3.12497864	5.695168317	up-regulated in High
KAT2A	0.25666514	4.1993134	3.80691498	0.00015834	0.00093848	-1.0382495	3.800411931	up-regulated in Low
GHDC	-0.2994537	3.45928029	-6.0169702	3.46E-09	5.15E-08	9.30328379	8.461242121	up-regulated in High
STAT5B	-0.1443049	3.39185237	-3.5699745	0.00039179	0.00208796	-1.8898725	3.406942138	up-regulated in High
STAT5A	-0.3005672	2.73459434	-5.4401856	8.38E-08	9.80E-07	6.20103924	7.076869014	up-regulated in High
STAT3	-0.1857082	5.28338208	-4.3320734	1.79E-05	0.00013169	1.03205916	4.7473968	up-regulated in High
PTRF	-0.4533554	5.46457301	-5.5446874	4.80E-08	5.86E-07	6.74272854	7.319152509	up-regulated in High
ATP6V0A1	-0.2915415	2.90600901	-5.9140064	6.23E-09	8.84E-08	8.72951931	8.205736743	up-regulated in High
NAGLU	-0.1427017	4.07542318	-2.6547406	0.00819227	0.02936471	-4.686147	2.086595983	up-regulated in High
COASY	0.25122397	4.52198515	6.24354786	9.20E-10	1.50E-08	10.5958828	9.036101085	up-regulated in Low
PSMC3IP	0.55558986	1.40297662	13.1657879	3.70E-34	7.72E-32	66.2147152	33.43175148	up-regulated in Low
TUBG1	0.65275457	4.25299078	12.0877317	1.17E-29	1.85E-27	55.9084178	28.9327074	up-regulated in Low
CCR10	0.12651022	0.54966718	2.46409141	0.01407453	0.04642241	-5.1683259	1.851566161	up-regulated in Low
CNTNAP1	-0.1254639	1.4428283	-2.5679163	0.01052321	0.03629815	-4.9100755	1.97785167	up-regulated in High
EZH1	-0.2861033	2.5460273	-6.7134794	5.22E-11	1.03E-09	13.4057259	10.28265082	up-regulated in High
RAMP2	-0.4870517	3.44542562	-6.5484483	1.46E-10	2.71E-09	12.3994048	9.836646275	up-regulated in High
VPS25	0.30694052	4.51304535	7.66958485	9.21E-14	2.70E-12	19.6368988	13.03593668	up-regulated in Low
CNTD1	-0.186937	0.66727408	-4.9865488	8.52E-07	8.22E-06	3.9564992	6.06979048	up-regulated in High
PSME3	0.37654444	4.58479878	10.2859921	1.27E-22	1.11E-20	39.814591	21.89579125	up-regulated in Low
AOC2	0.1258826	0.54834521	3.05750663	0.00235246	0.01005882	-3.5531028	2.62847763	up-regulated in Low
AOC3	-1.0046146	3.33311222	-11.147064	6.66E-26	8.00E-24	47.3125796	25.17636144	up-regulated in High
G6PC	0.10087752	0.04364289	2.73716917	0.00641976	0.02394623	-4.4668625	2.19248137	up-regulated in Low
PTGES3L	0.12992144	0.26776637	5.96819857	4.57E-09	6.66E-08	9.03043559	8.33976611	up-regulated in Low
RPL27	0.18450622	8.12061119	2.80597039	0.00521391	0.02004507	-4.2788516	2.282836676	up-regulated in Low
BRCA1	0.76978975	1.45325071	15.2278986	3.12E-43	1.15E-40	87.0262643	42.50584297	up-regulated in Low
NBR1	-0.133056	4.36455403	-3.2572777	0.00120197	0.00559602	-2.9340272	2.920104838	up-regulated in High
TMEM106A	-0.1651891	2.13815963	-3.731711	0.00021221	0.001219	-1.3141571	3.673238008	up-regulated in High
ARL4D	0.4136779	2.67453078	4.63908831	4.48E-06	3.73E-05	2.35703806	5.348292213	up-regulated in Low
DHX8	0.11715039	3.03139979	3.28048706	0.00110926	0.0052109	-2.8596675	2.954967669	up-regulated in Low
ETV4	-0.2878781	3.59464116	-2.6956086	0.00726488	0.02652497	-4.5782399	2.138771437	up-regulated in High

MEOX1	-0.2030369	0.47036283	-4.6787351	3.73E-06	3.15E-05	2.53421532	5.428405392	up-regulated in High
DUSP3	-0.1782611	4.27958626	-4.1995368	3.17E-05	0.00022132	0.48604091	4.498723971	up-regulated in High
CD300LG	-0.1796824	0.15365981	-6.3990268	3.63E-10	6.32E-09	11.5064567	9.440493136	up-regulated in High
MPP2	0.2563471	0.61628261	5.52558866	5.31E-08	6.44E-07	6.64304874	7.274587955	up-regulated in Low
NAGS	-0.1883631	1.83803036	-2.8910241	0.00400847	0.01598215	-4.0401825	2.397021132	up-regulated in High
LSM12	0.35513378	2.30717253	10.3331243	8.49E-23	7.64E-21	40.2151293	22.07115103	up-regulated in Low
G6PC3	0.11747317	3.80486018	2.65234107	0.00824988	0.02955129	-4.692433	2.083552182	up-regulated in Low
HDAC5	-0.3027789	3.21943532	-6.4170585	3.25E-10	5.71E-09	11.6132905	9.487910301	up-regulated in High
C17orf53	0.87492207	1.5658843	15.1401747	7.77E-43	2.75E-40	86.1163733	42.10935037	up-regulated in Low
ATXN7L3	0.21988685	4.05083293	5.65568988	2.63E-08	3.34E-07	7.32807019	7.580675101	up-regulated in Low
UBTF	0.15056423	4.1986618	4.32708004	1.83E-05	0.00013442	1.01120223	4.737910166	up-regulated in Low
RUNDC3A	0.30193667	0.4288046	4.93847873	1.08E-06	1.02E-05	3.72896648	5.967374087	up-regulated in Low
SLC25A39	0.4156356	5.69172642	8.27409433	1.21E-15	4.56E-14	23.9125806	14.91892552	up-regulated in Low
GRN	-0.2689123	7.44714175	-4.6762354	3.77E-06	3.18E-05	2.52300378	5.423337464	up-regulated in High
FAM171A2	0.20025318	1.14562324	2.69941079	0.0071836	0.02627342	-4.5681192	2.143657895	up-regulated in Low
FZD2	-0.1604853	2.2175987	-1.9903993	0.04709585	0.12446562	-6.2140922	1.327017323	up-regulated in High
C17orf104	0.12332531	0.21068616	3.47154197	0.00056272	0.0028675	-2.2283911	3.249704182	up-regulated in Low
CCDC43	0.3473802	3.51413982	9.41036806	1.85E-19	1.13E-17	32.5965334	18.73295196	up-regulated in Low
DBF4B	0.48416919	1.21765409	12.1173122	8.84E-30	1.42E-27	56.18503	29.05351932	up-regulated in Low
GJC1	0.22385278	0.98952979	4.24903785	2.57E-05	0.00018245	0.68812843	4.590839942	up-regulated in Low
HIGD1B	-0.6654764	1.81737315	-9.318302	3.88E-19	2.28E-17	31.863194	18.41128951	up-regulated in High
EFTUD2	0.35276282	3.65230084	9.94559206	2.27E-21	1.76E-19	36.9574735	20.64448827	up-regulated in Low
KIF18B	1.22980003	1.7331578	17.9244538	9.47E-56	7.90E-53	115.769766	55.02362452	up-regulated in Low
C1QL1	0.27802316	0.42163878	4.90349422	1.28E-06	1.19E-05	3.56462811	5.893359929	up-regulated in Low
NMT1	0.1646068	4.2608684	4.87452363	1.47E-06	1.35E-05	3.42934248	5.832402849	up-regulated in Low
PLCD3	0.31221053	1.68328797	4.0830942	5.18E-05	0.00034561	0.01937342	4.285623769	up-regulated in Low
ACBD4	-0.2192303	2.26400685	-4.3655236	1.54E-05	0.00011531	1.17235259	4.811184217	up-regulated in High
MAP3K14	-0.1739778	2.11612121	-2.9641929	0.00318091	0.01306992	-3.8293523	2.497449152	up-regulated in High
ARHGAP27	-0.2494839	2.60566008	-4.3260972	1.84E-05	0.00013492	1.00709948	4.736043949	up-regulated in High
PLEKHM1	-0.355769	2.41200796	-8.0183789	7.77E-15	2.62E-13	22.0733873	14.10945752	up-regulated in High
NSF	0.12301743	3.73367589	2.81182884	0.00512137	0.01973829	-4.2626334	2.290613519	up-regulated in Low
CDC27	0.29692732	3.32765983	8.04242037	6.53E-15	2.23E-13	22.2444178	14.18476192	up-regulated in Low
ITGB3	-0.363565	0.99112268	-4.7621751	2.52E-06	2.20E-05	2.91160308	5.598878942	up-regulated in High
NPEPPS	0.1412306	3.57495358	3.01815742	0.00267411	0.01123985	-3.6706	2.572821323	up-regulated in Low
KPNB1	0.48819534	5.18036103	10.9926211	2.65E-25	3.04E-23	45.9404029	24.57630907	up-regulated in Low
OSBPL7	-0.3214677	1.98730738	-5.0658709	5.75E-07	5.73E-06	4.33631297	6.240604495	up-regulated in High
MRPL10	0.25821358	4.22249294	7.54896291	2.12E-13	5.87E-12	18.8141161	12.67307985	up-regulated in Low
LRRC46	-0.3651497	1.27452732	-4.0218483	6.67E-05	0.0004337	-0.221159	4.175563409	up-regulated in High
SP6	0.3661213	1.06425041	4.83605713	1.77E-06	1.60E-05	3.25083916	5.751933605	up-regulated in Low
PNPO	0.14384074	3.38394511	3.16723222	0.00163388	0.00732809	-3.2177282	2.786781104	up-regulated in Low
PRR15L	-1.0300009	5.07254855	-8.9119744	9.60E-18	4.73E-16	28.6878546	17.01767594	up-regulated in High
COPZ2	-0.2283277	2.54929413	-3.0393236	0.0024964	0.01060053	-3.6075799	2.602686043	up-regulated in High
CBX1	0.39050773	4.66634038	7.35053648	8.21E-13	2.09E-11	17.4832226	12.08573867	up-regulated in Low
HOXB6	0.26365356	1.6750922	2.52511507	0.01187691	0.04025876	-5.0177961	1.925296505	up-regulated in Low
HOXB7	0.60418611	2.54669997	4.83715299	1.76E-06	1.60E-05	3.25590667	5.754218662	up-regulated in Low
HOXB8	0.422298	0.71666694	4.02291153	6.65E-05	0.000432	-0.2170125	4.177462053	up-regulated in Low
HOXB9	0.70758498	1.05315324	4.79033452	2.20E-06	1.95E-05	3.04033838	5.656981065	up-regulated in Low
PRAC2	0.25242171	0.27510166	4.77619958	2.36E-06	2.07E-05	2.97563173	5.627780115	up-regulated in Low
HOXB13	0.36322273	0.55446889	4.38924881	1.39E-05	0.00010465	1.27246379	4.856676726	up-regulated in Low
CALCOCO2	-0.1486621	3.81237405	-3.8603202	0.00012823	0.00077843	-0.8391651	3.892012173	up-regulated in High
ATP5G1	0.43694353	4.32785057	7.7707308	4.53E-14	1.38E-12	20.3347426	13.34355511	up-regulated in Low
UBE2Z	0.14400983	4.64584628	3.97121316	8.21E-05	0.00052302	-0.4174476	4.08562733	up-regulated in Low
SNF8	0.34965107	3.77313677	10.0996707	6.20E-22	5.09E-20	38.2428844	21.20754214	up-regulated in Low
GIP	0.16862151	0.16296074	4.69862611	3.40E-06	2.89E-05	2.62362603	5.468814287	up-regulated in Low
IGF2BP1	0.76354132	0.5005128	8.99206132	5.14E-18	2.64E-16	29.3056953	17.2889488	up-regulated in Low
B4GALNT2	0.40522624	0.51767039	4.79682025	2.14E-06	1.90E-05	3.07008693	5.67040396	up-regulated in Low
GNGT2	-0.2004291	1.23228151	-4.1017979	4.79E-05	0.00032206	0.09350652	4.319513356	up-regulated in High
ABI3	-0.3369873	2.72523249	-5.1034388	4.76E-07	4.83E-06	4.51808278	6.322289654	up-regulated in High
ZNF652	-0.1043322	2.61582929	-2.5234014	0.0119342	0.04042714	-5.0220724	1.923206709	up-regulated in High
PHB	0.39476387	4.86836453	10.2938004	1.19E-22	1.05E-20	39.8808658	21.92480798	up-regulated in Low
AC091180.1	0.1604724	1.06930537	2.39293857	0.01708531	0.05449065	-5.3393028	1.767377035	up-regulated in Low
NGFR	-0.283785	0.71708348	-4.6001505	5.37E-06	4.39E-05	2.18437498	5.270169715	up-regulated in High
NXPH3	-0.2188494	0.5171567	-6.748807	4.18E-11	8.43E-10	13.6238611	10.37927059	up-regulated in High
SPOP	-0.1677548	3.53841355	-5.2015994	2.90E-07	3.06E-06	4.99872338	6.538099091	up-regulated in High
SLC35B1	0.17787792	3.73637804	4.60853659	5.16E-06	4.24E-05	2.2214488	5.286948275	up-regulated in Low
FAM117A	-0.4627952	2.67858156	-8.5529683	1.50E-16	6.45E-15	25.9679389	15.82274554	up-regulated in High
DLX4	-0.163168	0.92749088	-2.9947165	0.00288439	0.01199851	-3.7398978	2.539945476	up-regulated in High
DLX3	-0.5952388	1.10549341	-7.5697569	1.84E-13	5.13E-12	18.9552191	12.7353209	up-regulated in High
ITGA3	-0.5682941	5.4802012	-5.4772189	6.88E-08	8.18E-07	6.39195898	7.162292583	up-regulated in High
PDK2	-0.3964637	2.27654899	-8.5802838	1.22E-16	5.30E-15	26.1719912	15.9124335	up-regulated in High
PPP1R9B	-0.1478878	4.40851444	-2.8735395	0.00423327	0.01675603	-4.089809	2.3733237	up-regulated in High

SGCA	-0.6791629	1.27571126	-10.482041	2.35E-23	2.24E-21	41.4883754	22.62849943	up-regulated in High
TMEM92	-0.361778	2.75485055	-4.1517859	3.88E-05	0.00026601	0.29318893	4.410726196	up-regulated in High
XYLT2	-0.1167913	3.30025166	-2.4892332	0.01312888	0.04387841	-5.1067424	1.881772373	up-regulated in High
MRPL27	0.2809081	3.76478609	6.33623388	5.29E-10	8.98E-09	11.1364127	9.276207293	up-regulated in Low
EME1	0.72674287	1.16156596	15.0748478	1.53E-42	5.33E-40	85.4400407	41.8146212	up-regulated in Low
LRRC59	0.46375887	5.46087848	10.6660337	4.74E-24	4.83E-22	43.0774824	23.32392469	up-regulated in Low
ACSF2	-0.3107132	3.23107751	-4.1399883	4.08E-05	0.00027831	0.24585844	4.38911533	up-regulated in High
CHAD	-0.5845131	1.16957347	-6.3559485	4.70E-10	8.04E-09	11.2522588	9.327646328	up-regulated in High
EPN3	0.35293614	2.24562873	4.44729933	1.07E-05	8.27E-05	1.51952993	4.9688615	up-regulated in Low
SPATA20	-0.2048805	3.80329512	-3.1981977	0.00147136	0.00669692	-3.1210278	2.832282432	up-regulated in High
ABCC3	-0.7901761	4.40396961	-6.8052053	2.92E-11	6.03E-10	13.9740701	10.53434902	up-regulated in High
TOB1	-0.2261509	4.78536386	-2.7017534	0.00713393	0.0261212	-4.5618767	2.14667128	up-regulated in High
SPAG9	-0.3199136	3.39548202	-5.7834388	1.30E-08	1.75E-07	8.01431708	7.88693061	up-regulated in High
NME1	0.78662413	4.14374281	12.2880442	1.76E-30	2.95E-28	57.7886879	29.75385323	up-regulated in Low
NME2	0.2239077	0.66980362	6.28988681	6.99E-10	1.16E-08	10.8652758	9.155786689	up-regulated in Low
NME1-NME2	0.44736087	4.63717877	6.96958897	1.02E-11	2.24E-10	15.0085556	10.99215144	up-regulated in Low
NME1-NME2	0.44736087	4.63717877	6.96958897	1.02E-11	2.24E-10	15.0085556	10.99215144	up-regulated in Low
UTP18	0.31504205	3.90648134	7.90923507	1.70E-14	5.47E-13	21.3019128	13.76969722	up-regulated in Low
CA10	-0.4155565	0.40968812	-5.6690171	2.44E-08	3.12E-07	7.39903395	7.612361575	up-regulated in High
HLF	-1.1255539	1.62627988	-11.944617	4.46E-29	6.77E-27	54.575351	28.35042951	up-regulated in High
MMD	0.39433305	3.16718318	4.86806091	1.52E-06	1.39E-05	3.39926245	5.818845927	up-regulated in Low
TMEM100	-0.8991903	1.41625683	-9.4379994	1.48E-19	9.17E-18	32.8176079	18.82990811	up-regulated in High
PCTP	-0.1070486	2.53590014	-2.0438314	0.04149836	0.11259652	-6.107057	1.381969096	up-regulated in High
ANKFN1	-0.2582343	0.37881522	-7.018276	7.42E-12	1.67E-10	15.3188455	11.12938835	up-regulated in High
NOG	-0.187321	0.72439162	-2.7680946	0.00584961	0.0221177	-4.3829133	2.232872758	up-regulated in High
TRIM25	0.20865727	3.01597389	3.85636103	0.00013026	0.00078945	-0.8540138	3.88518471	up-regulated in Low
COIL	0.23464549	3.19398733	6.50901074	1.86E-10	3.39E-09	12.1620366	9.731376499	up-regulated in Low
SCPEP1	-0.4784668	5.15671876	-5.6380693	2.89E-08	3.65E-07	7.23447126	7.53887545	up-regulated in High
AKAP1	-0.1662456	4.20011842	-3.0142521	0.00270814	0.01135756	-3.6821814	2.567329622	up-regulated in High
MRPS23	0.21262399	3.36924816	4.09367823	4.96E-05	0.00033221	0.06128484	4.304785155	up-regulated in Low
VEZF1	-0.1286542	3.84449537	-2.8722573	0.0042502	0.01681733	-4.0934368	2.371590445	up-regulated in High
SRSF1	0.27676613	5.63808762	9.78944743	8.32E-21	5.98E-19	35.6683085	20.07962342	up-regulated in Low
BZRAP1	-0.4401081	1.26849202	-6.6019889	1.05E-10	1.99E-09	12.7235842	9.980373255	up-regulated in High
SUPT4H1	0.10060644	5.0411501	2.42487168	0.01567026	0.05076509	-5.2631741	1.804923947	up-regulated in Low
RNF43	-0.2413393	2.54744257	-3.0534179	0.00238415	0.01018386	-3.5653801	2.622667052	up-regulated in High
4-Sep	-0.4026464	1.05974999	-11.616263	9.36E-28	1.32E-25	51.5503856	27.02879174	up-regulated in High
RAD51C	0.20749979	1.71470825	6.57059237	1.27E-10	2.39E-09	12.5332149	9.895977695	up-regulated in Low
PPM1E	0.16599071	0.31513072	4.02264214	6.65E-05	0.00043238	-0.2180632	4.176980936	up-regulated in Low
TRIM37	0.2625817	2.84175786	5.41320485	9.66E-08	1.12E-06	6.06266787	7.014936126	up-regulated in Low
SKA2	0.41472643	3.60862718	7.21071148	2.09E-12	5.07E-11	16.5625008	11.67909845	up-regulated in Low
PRR11	1.23721789	2.18721686	17.8179796	3.02E-55	2.49E-52	114.611779	54.51954871	up-regulated in Low
SMG8	0.19833964	2.74825673	4.81329292	1.97E-06	1.77E-05	3.14580816	5.704564381	up-regulated in Low
YPEL2	-0.275998	2.04885476	-6.9186739	1.41E-11	3.05E-10	14.6859655	10.84943637	up-regulated in High
DHX40	-0.105816	3.53359966	-2.0904994	0.03708191	0.10279181	-6.0112915	1.43083792	up-regulated in High
PTRH2	0.32843088	2.59505988	8.08360436	4.85E-15	1.68E-13	22.5383142	14.31414905	up-regulated in Low
TUBD1	0.18961478	1.95667937	4.66684746	3.94E-06	3.31E-05	2.48094578	5.404324242	up-regulated in Low
RPS6KB1	0.19886075	2.67723532	4.85398841	1.62E-06	1.48E-05	3.33388906	5.78937808	up-regulated in Low
RNFT1	-0.1210696	2.48028804	-2.3583393	0.01874432	0.05887169	-5.4206745	1.72713024	up-regulated in High
CA4	-0.4191838	0.69039436	-5.874255	7.80E-09	1.09E-07	8.51030419	8.108058609	up-regulated in High
USP32	0.19072359	2.66759474	3.98306801	7.82E-05	0.00050081	-0.371701	4.106597587	up-regulated in Low
C17orf64	0.10869741	0.20685789	4.11244305	4.58E-05	0.00030952	0.13584038	4.338859594	up-regulated in Low
BCAS3	-0.1017555	1.51264291	-2.8102039	0.00514689	0.01982265	-4.2671351	2.288455161	up-regulated in High
TBX2	-0.5647276	2.20693002	-8.0537076	6.02E-15	2.06E-13	22.3248506	14.22017409	up-regulated in High
TBX4	-0.787452	1.58448447	-11.297428	1.72E-26	2.13E-24	48.6594954	25.76524599	up-regulated in High
BRIP1	0.52159588	0.93478521	12.3560014	9.25E-31	1.59E-28	58.4303293	30.03403088	up-regulated in Low
INTS2	0.19924872	1.85981293	5.1608334	3.56E-07	3.70E-06	4.79811549	6.44805724	up-regulated in Low
METTL2A	0.28386456	2.6747424	7.76245907	4.81E-14	1.45E-12	20.2774037	13.31828403	up-regulated in Low
TLK2	0.1555769	2.32563982	5.0231649	7.11E-07	6.95E-06	4.13115366	6.148360321	up-regulated in Low
MRC2	-0.527493	4.13921191	-6.1579027	1.52E-09	2.41E-08	10.1024665	8.816780717	up-regulated in High
TANC2	-0.1801427	2.33132594	-2.9288565	0.00355882	0.01441776	-3.9318068	2.448694196	up-regulated in High
CYB561	-0.1241608	4.91238822	-2.2522978	0.02474032	0.07409659	-5.6628418	1.606594703	up-regulated in High
ACE	-0.3292728	2.36320539	-4.7090175	3.24E-06	2.76E-05	2.67047339	5.489981706	up-regulated in High
KCNH6	0.15898312	0.16702539	3.21753195	0.0013776	0.00632243	-3.0601921	2.860877078	up-regulated in Low
TACO1	0.39205755	3.44064819	10.8795622	7.24E-25	7.98E-23	44.9432944	24.14019204	up-regulated in Low
MAP3K3	-0.4716625	2.83169819	-10.441608	3.34E-23	3.13E-21	41.1415159	22.47667938	up-regulated in High
STRADA	-0.1070105	1.3296172	-3.3220789	0.0009595	0.00458862	-2.7251487	3.017953557	up-regulated in High
FTSJ3	0.2302126	3.78613077	5.3014078	1.73E-07	1.91E-06	5.4958393	6.761039471	up-regulated in Low
PSMC5	0.25815241	4.89104447	7.03075164	6.84E-12	1.55E-10	15.3986393	11.16467436	up-regulated in Low
SMARCD2	0.16208491	4.98333246	4.02948991	6.47E-05	0.00042116	-0.1913339	4.18921914	up-regulated in Low
CD79B	-0.4872555	1.99255248	-5.1300149	4.17E-07	4.26E-06	4.64739957	6.380379325	up-regulated in High
PRR29	-0.4499471	1.27750046	-6.3279502	5.56E-10	9.40E-09	11.0878276	9.254631956	up-regulated in High

ICAM2	-0.3525726	2.10756438	-6.3030879	6.46E-10	1.08E-08	10.9423315	9.190013625	up-regulated in High
ERN1	-0.2756831	2.55111064	-5.3128514	1.63E-07	1.81E-06	5.55337601	6.786826162	up-regulated in High
PECAM1	-0.5327904	4.50586113	-8.4149502	4.24E-16	1.72E-14	24.9443323	15.37272426	up-regulated in High
MILR1	-0.2147134	2.157796	-2.7411017	0.00634457	0.02370896	-4.456238	2.197597503	up-regulated in High
DDX5	-0.1552383	6.40779953	-3.7919194	0.00016793	0.00098927	-1.0936799	3.774884023	up-regulated in High
SMURF2	-0.3444073	3.29957162	-5.3087712	1.67E-07	1.84E-06	5.53284865	6.777626636	up-regulated in High
GNA13	0.16376133	4.00788154	3.25365167	0.00121709	0.00565918	-2.9455988	2.914676631	up-regulated in Low
RGS9	-0.1599631	0.46786095	-5.805084	1.15E-08	1.56E-07	8.13191944	7.939377868	up-regulated in High
AXIN2	-0.5372133	1.88328799	-7.1268558	3.65E-12	8.57E-11	16.0171986	11.43813394	up-regulated in High
CEP112	-0.4497211	1.42216694	-10.841532	1.01E-24	1.10E-22	44.6093098	23.99409734	up-regulated in High
APOH	-0.8636227	1.66939409	-5.9852252	4.15E-09	6.09E-08	9.12547115	8.382082836	up-regulated in High
PRKCA	-0.1701472	1.98567943	-2.1621669	0.03108317	0.08924775	-5.8600912	1.507474713	up-regulated in High
PSMD12	0.37552907	3.87941912	10.6144662	7.44E-24	7.42E-22	42.630347	23.12826962	up-regulated in Low
PITPNC1	0.45594127	2.45218713	5.83260575	9.86E-09	1.35E-07	8.28200317	8.006296207	up-regulated in Low
NOL11	0.40772522	3.6130501	10.4543115	2.99E-23	2.81E-21	41.2504053	22.52434124	up-regulated in Low
C17orf58	0.3488293	3.03221191	6.28056057	7.39E-10	1.22E-08	10.8109208	9.131641139	up-regulated in Low
KPNA2	1.23279729	4.94060735	16.9908376	2.36E-51	1.38E-48	105.671003	50.62704007	up-regulated in Low
AMZ2	0.13896656	4.04005331	3.51545744	0.00047929	0.00249588	-2.0784741	3.319403445	up-regulated in Low
PRKAR1A	-0.2134468	5.55195028	-4.8077161	2.03E-06	1.81E-05	3.12014623	5.692988358	up-regulated in High
FAM20A	-0.7555587	3.14752395	-8.3682844	6.00E-16	2.38E-14	24.601059	15.22176371	up-regulated in High
ABCA8	-0.5269084	0.69914426	-10.133095	4.67E-22	3.89E-20	38.523449	21.33041788	up-regulated in High
ABCA9	-0.1987703	0.40698503	-7.617185	1.33E-13	3.78E-12	19.2782042	12.87777044	up-regulated in High
ABCA6	-0.2400667	0.48860771	-8.3498982	6.88E-16	2.70E-14	24.4662045	15.16245298	up-regulated in High
ABCA10	-0.1235353	0.20660181	-7.3839738	6.55E-13	1.69E-11	17.7055079	12.18387232	up-regulated in High
ABCA5	-0.1107874	1.09632907	-2.4081908	0.01639598	0.05269222	-5.3030644	1.785262508	up-regulated in High
MAP2K6	0.44231354	0.99628571	7.15068931	3.12E-12	7.38E-11	16.1716574	11.50639817	up-regulated in Low
KCNJ2	-0.163308	1.11205155	-2.6996349	0.00717884	0.02625764	-4.5675223	2.143946045	up-regulated in High
SSTR2	0.1428446	0.3876927	4.6931068	3.49E-06	2.95E-05	2.59878179	5.457587299	up-regulated in Low
FAM104A	0.29460074	2.95552396	9.175674	1.21E-18	6.73E-17	30.7371305	17.91723348	up-regulated in Low
C17orf80	0.19182327	2.64757504	5.27896803	1.95E-07	2.12E-06	5.38333749	6.710608823	up-regulated in Low
CDC42EP4	-0.2397181	3.83880614	-4.0552196	5.82E-05	0.00038355	-0.0905211	4.235358986	up-regulated in High
SDK2	-0.2098951	0.64410327	-3.3905618	0.0007532	0.00371051	-2.5001277	3.123090608	up-regulated in High
RPL38	0.25519773	6.39553894	4.49490791	8.67E-06	6.80E-05	1.72439052	5.061791508	up-regulated in Low
TTYH2	-0.3363461	1.26925147	-6.5349743	1.58E-10	2.92E-09	12.3181715	9.800623346	up-regulated in High
DNAI2	-0.4298758	0.5139405	-5.9074793	6.46E-09	9.15E-08	8.69343614	8.189661039	up-regulated in High
GPRC5C	-0.6927145	3.41689471	-6.7428708	4.34E-11	8.72E-10	13.5871408	10.36300729	up-regulated in High
CD300A	-0.1794201	2.63483433	-2.373874	0.01798272	0.05688973	-5.3842827	1.74514473	up-regulated in High
CD300LB	-0.1679268	0.53244654	-5.6860901	2.22E-08	2.86E-07	7.49015703	7.65304362	up-regulated in High
CD300C	-0.3578942	1.56398514	-5.4237236	9.14E-08	1.06E-06	6.11654067	7.039050896	up-regulated in High
RAB37	-0.4481475	1.2892282	-6.1046021	2.08E-09	3.22E-08	9.79834394	8.681529381	up-regulated in High
CD300LF	-0.5502217	2.14373427	-6.7925218	3.17E-11	6.50E-10	13.8951002	10.49938424	up-regulated in High
NAT9	0.17377146	3.38765786	3.63079902	0.00031194	0.00170807	-1.6761985	3.505932252	up-regulated in Low
FADS6	-0.2116099	0.32981233	-3.5427628	0.0004334	0.00228221	-1.9843563	3.363107641	up-regulated in High
CDR2L	0.18619229	3.38851561	2.40391641	0.01658665	0.05319664	-5.3132428	1.780241282	up-regulated in Low
ICT1	0.36399461	4.50839906	7.64899811	1.06E-13	3.09E-12	19.4957443	12.97369871	up-regulated in Low
KCTD2	-0.1508088	3.2542381	-3.733359	0.00021086	0.00121213	-1.3081668	3.676002013	up-regulated in High
ATP5H	0.23370677	5.19290771	5.38546922	1.12E-07	1.28E-06	5.92106208	6.951536791	up-regulated in Low
SLC16A5	-0.4108789	3.16167451	-4.7394989	2.80E-06	2.43E-05	2.80843889	5.552299699	up-regulated in High
NT5C	0.21989562	3.91149507	4.09343213	4.96E-05	0.00033244	0.06030915	4.304339135	up-regulated in Low
HN1	0.62542525	5.43071076	8.90564072	1.01E-17	4.96E-16	28.6391627	16.99629457	up-regulated in Low
SUMO2	0.26649564	5.58015498	6.65571897	7.49E-11	1.45E-09	13.0511339	10.12554598	up-regulated in Low
NUP85	0.40468579	3.30325224	9.69786968	1.77E-20	1.22E-18	34.9186347	19.75106333	up-regulated in Low
MRPS7	0.31711885	4.36072718	7.46181211	3.86E-13	1.03E-11	18.2260905	12.41363956	up-regulated in Low
SLC25A19	0.17069723	2.0467616	3.93663182	9.45E-05	0.00059259	-0.5501619	4.02475586	up-regulated in Low
CASKIN2	-0.1149993	2.80505966	-2.1368253	0.03310124	0.09378311	-5.9141274	1.480155708	up-regulated in High
TSEN54	0.10709747	3.67020024	2.21443232	0.02725333	0.08015918	-5.7466715	1.564580484	up-regulated in Low
LLGL2	-0.1568244	4.20078604	-2.1124859	0.03514438	0.09839719	-5.9654376	1.454144078	up-regulated in High
SMIM5	-0.2836861	0.82628599	-5.0404009	6.52E-07	6.44E-06	4.21376668	6.185511188	up-regulated in High
SMIM6	-0.5293325	1.88794575	-5.6490769	2.72E-08	3.45E-07	7.29291251	7.564975123	up-regulated in High
GALK1	0.1805165	3.18675631	3.08708881	0.00213449	0.00924939	-3.4638051	2.670706436	up-regulated in Low
UNK	0.10923268	2.939367	2.76432562	0.00591656	0.02232815	-4.3931935	2.227930662	up-regulated in Low
WBP2	-0.1235953	4.84490646	-2.8777085	0.00417865	0.01655579	-4.0780025	2.378963553	up-regulated in High
ACOX1	-0.1437609	3.5631128	-3.2801062	0.00111072	0.00521695	-2.8608919	2.954393877	up-regulated in High
EVPL	-0.5618232	3.86126552	-5.6348834	2.94E-08	3.71E-07	7.21757496	7.531329095	up-regulated in High
SRP68	0.15230693	4.05370777	4.38354504	1.43E-05	0.00010714	1.24835022	4.845720936	up-regulated in Low
FOXJ1	-0.5081608	3.44348141	-3.4604031	0.00058595	0.00297157	-2.2661313	3.232141292	up-regulated in High
RNF157	0.33717702	0.92958607	5.38401481	1.13E-07	1.28E-06	5.91365437	6.948219705	up-regulated in Low
UBALD2	0.22835234	5.00329067	4.15059214	3.90E-05	0.00026713	0.28839391	4.408537073	up-regulated in Low
PRPSAP1	0.16736319	2.72806154	4.69524075	3.45E-06	2.93E-05	2.60838423	5.461926713	up-regulated in Low
SPHK1	0.49929842	1.99626135	6.05865897	2.72E-09	4.12E-08	9.53802752	8.565715256	up-regulated in Low
UBE2O	0.22524905	3.12435555	5.00049983	7.95E-07	7.72E-06	4.0229079	6.099669567	up-regulated in Low

RHBDF2	0.15249311	3.00235282	2.40912378	0.01635463	0.05257889	-5.3008404	1.786359401	up-regulated in Low
CYGB	-0.3694498	2.56646462	-6.1331062	1.76E-09	2.76E-08	9.96070038	8.75374045	up-regulated in High
ST6GALNAC	-0.3156543	1.4451524	-4.1871624	3.34E-05	0.00023208	0.43586701	4.475838536	up-regulated in High
ST6GALNAC	-0.9371378	3.80837081	-6.5694968	1.28E-10	2.40E-09	12.5265858	9.893038554	up-regulated in High
MXRA7	-0.3292601	2.98602631	-4.507591	8.19E-06	6.45E-05	1.77930528	5.086688739	up-regulated in High
JMJD6	0.24859272	2.54990954	6.7700608	3.65E-11	7.43E-10	13.7555537	10.43759226	up-regulated in Low
METTL23	0.13502191	3.75953067	3.80775677	0.00015782	0.00093576	-1.0351317	3.801847468	up-regulated in Low
SRSF2	0.22415817	5.5083524	6.49765632	1.99E-10	3.61E-09	12.0939201	9.701162735	up-regulated in Low
MGAT5B	0.2700007	0.32639337	6.0012047	3.79E-09	5.60E-08	9.21487526	8.42188664	up-regulated in Low
TNRC6C	-0.209524	1.41881937	-4.9332388	1.11E-06	1.04E-05	3.70428484	5.956260364	up-regulated in High
TMC6	-0.2556088	3.77999297	-4.1502346	3.91E-05	0.00026744	0.2869581	4.407881555	up-regulated in High
TMC8	-0.4299268	2.60090358	-5.7084248	1.97E-08	2.56E-07	7.6097258	7.706415359	up-regulated in High
SYNGR2	-0.1813291	6.37173654	-2.8929528	0.00398435	0.01590261	-4.0346904	2.399642309	up-regulated in High
TK1	1.27388541	4.7852632	14.3257016	3.39E-39	9.62E-37	77.7651952	38.46936353	up-regulated in Low
AFMID	0.14790922	3.11112311	2.83583792	0.00475751	0.01851103	-4.1958261	2.322620743	up-regulated in Low
BIRC5	1.65058965	3.18103259	18.7120604	1.70E-59	1.83E-56	124.377995	58.7703577	up-regulated in Low
PGS1	-0.1271373	3.03406207	-2.6904174	0.00737719	0.02686033	-4.5920354	2.132108792	up-regulated in High
CYTH1	-0.2542574	3.54244803	-5.3713619	1.20E-07	1.36E-06	5.84928445	6.919393352	up-regulated in High
TIMP2	-0.2909901	5.62658254	-3.2880265	0.0010806	0.00509635	-2.8354035	2.966336554	up-regulated in High
LGALS3BP	-0.3500222	8.26063938	-4.2631743	2.41E-05	0.00017273	0.74624467	4.617313006	up-regulated in High
CANT1	0.11973121	4.77000532	2.16031902	0.03122666	0.08957052	-5.8640526	1.505474497	up-regulated in Low
C1QTNF1	-0.2857632	2.35501954	-3.6651322	0.00027389	0.00152403	-1.5540763	3.562424251	up-regulated in High
ENGASE	-0.252922	2.52284217	-3.3823712	0.00077549	0.00380301	-2.5272712	3.110422681	up-regulated in High
CBX2	0.79493333	1.76923723	8.87313357	1.30E-17	6.28E-16	28.3896514	16.88672469	up-regulated in Low
CBX8	0.11515602	2.03346047	2.60168258	0.00955426	0.0335223	-4.8238497	2.019802995	up-regulated in Low
CBX4	0.16101334	4.00657377	3.06004648	0.00233297	0.00998866	-3.5454684	2.632090238	up-regulated in Low
TBC1D16	0.15293359	2.77672534	2.42898521	0.01549571	0.05029729	-5.2532958	1.80978866	up-regulated in Low
CCDC40	-0.1549602	0.97953415	-2.9290013	0.00355719	0.01441416	-3.9313895	2.448892965	up-regulated in High
EIF4A3	0.54642885	4.29942529	12.3743832	7.76E-31	1.35E-28	58.6042089	30.10995346	up-regulated in Low
CARD14	0.50674062	0.95975193	7.10049656	4.34E-12	1.01E-10	15.8468596	11.36284193	up-regulated in Low
SGSH	-0.2549839	3.06952909	-5.4036995	1.02E-07	1.17E-06	6.01406497	6.993177938	up-regulated in High
RPTOR	0.22368962	2.58468033	4.91501301	1.21E-06	1.13E-05	3.61862015	5.917680694	up-regulated in Low
CHMP6	-0.1900097	3.77985381	-4.0677814	5.52E-05	0.00036586	-0.0410839	4.257975146	up-regulated in High
BAIAP2	-0.4319932	2.95032047	-6.5890744	1.13E-10	2.14E-09	12.6451863	9.945619491	up-regulated in High
AATK	-0.3100797	1.32594678	-3.5824667	0.00037397	0.00200139	-1.8462674	3.427159027	up-regulated in High
CEP131	0.19260275	2.69829502	2.96663068	0.00315624	0.01297953	-3.8222405	2.50083013	up-regulated in Low
C17orf89	0.25429449	3.60934918	4.99646456	8.11E-07	7.86E-06	4.00368233	6.091020038	up-regulated in Low
SLC38A10	-0.184424	4.73789768	-3.2068411	0.00142874	0.00653298	-3.0938747	2.845048147	up-regulated in High
TMEM105	-0.1660793	0.92173938	-3.3776303	0.00078868	0.00385913	-2.542954	3.103101733	up-regulated in High
BAHCC1	-0.165275	1.75550962	-2.7787044	0.00566482	0.02151932	-4.3539016	2.24681376	up-regulated in High
ACTG1	0.31522819	10.1411748	6.67816169	6.51E-11	1.27E-09	13.1886064	10.18646076	up-regulated in Low
NPLOC4	0.18408008	4.21752589	4.29799827	2.08E-05	0.00015064	0.89017348	4.682842425	up-regulated in Low
PDE6G	-0.1372104	0.94285195	-2.5189174	0.01208526	0.04085352	-5.0332479	1.917744031	up-regulated in High
OXLD1	0.12332853	3.8199797	2.06795689	0.03916242	0.10743674	-6.0578154	1.407130494	up-regulated in Low
CCDC137	0.40231657	3.65073734	7.930601	1.46E-14	4.75E-13	21.4522922	13.83593551	up-regulated in Low
ARL16	0.11124601	3.44809392	2.00853642	0.04512833	0.1203814	-6.1780726	1.34555071	up-regulated in Low
HGS	0.24797619	3.53456511	5.33976828	1.42E-07	1.59E-06	5.68914552	6.847661814	up-regulated in Low
MRPL12	0.65659805	4.50860495	10.1747009	3.28E-22	2.75E-20	38.8735402	21.48373309	up-regulated in Low
SLC25A10	0.56339808	2.87916646	8.25289907	1.41E-15	5.29E-14	23.7584671	14.85112358	up-regulated in Low
SLC25A10	0.56339808	2.87916646	8.25289907	1.41E-15	5.29E-14	23.7584671	14.85112358	up-regulated in Low
P4HB	0.12908706	7.95698009	2.62558503	0.00891742	0.03159151	-4.7621498	2.0497608	up-regulated in Low
ALYREF	0.75473154	5.2967212	14.0356756	6.42E-38	1.67E-35	74.8368637	37.19258565	up-regulated in Low
ANAPC11	0.32502704	4.29859563	5.58244454	3.91E-08	4.85E-07	6.9406833	7.407628118	up-regulated in Low
SIRT7	0.11445423	2.64824312	2.3324684	0.02007558	0.06225694	-5.4807612	1.697332003	up-regulated in Low
MAFG	0.33092295	3.13263538	5.95253151	5.00E-09	7.23E-08	8.94319561	8.300915298	up-regulated in Low
PYCR1	0.57801192	5.25109297	6.78053957	3.42E-11	6.97E-10	13.820609	10.46640008	up-regulated in Low
STRA13	0.45274335	4.71615628	6.44234752	2.79E-10	4.96E-09	11.763551	9.55459224	up-regulated in Low
LRRC45	0.2713267	3.07531795	4.57846055	5.93E-06	4.81E-05	2.08877392	5.226892407	up-regulated in Low
RAC3	0.48156432	3.08583179	4.74485752	2.73E-06	2.37E-05	2.83277695	5.563289949	up-regulated in Low
RFNG	0.10598055	3.61767353	2.12675895	0.03393354	0.09570466	-5.9354184	1.469370789	up-regulated in Low
GPS1	0.26080575	4.27511313	6.16549156	1.46E-09	2.31E-08	10.1459514	8.836115143	up-regulated in Low
DUS1L	0.17459066	4.25038937	3.10729942	0.00199642	0.00872296	-3.4023211	2.699748302	up-regulated in Low
FASN	0.3394511	5.0091455	3.18132684	0.00155795	0.00704437	-3.1738248	2.80744693	up-regulated in Low
CCDC57	-0.1570384	2.03714316	-2.9773793	0.00304955	0.01259668	-3.7908158	2.515764434	up-regulated in High
SLC16A3	0.44413973	4.23753346	4.80215457	2.08E-06	1.85E-05	3.09458172	5.681455318	up-regulated in Low
TEX19	0.14972485	0.16527286	2.93455986	0.00349517	0.01419735	-3.9153508	2.456531217	up-regulated in Low
HEXDC	-0.1513964	2.90579726	-2.1461841	0.03234322	0.09203742	-5.8942443	1.490216688	up-regulated in High
FOXK2	0.27975258	3.11632221	6.86909551	1.94E-11	4.11E-10	14.3737171	10.71125871	up-regulated in Low
WDR45B	0.28796559	5.41321789	5.17894774	3.25E-07	3.41E-06	4.88708061	6.487994323	up-regulated in Low
RAB40B	-0.2059463	2.00856533	-3.5804639	0.00037678	0.00201493	-1.8532682	3.423913791	up-regulated in High
FN3KRP	0.11698358	4.06869727	2.73233964	0.00651319	0.02422818	-4.47989	2.186206357	up-regulated in Low

TBCD	-0.1173065	3.22781261	-2.3371627	0.01982804	0.06163879	-5.4699064	1.70272021	up-regulated in High
ZNF750	-0.9260736	1.90870159	-8.8303854	1.81E-17	8.58E-16	28.0625432	16.74306466	up-regulated in High
USP14	0.37381437	3.86837965	8.70361968	4.79E-17	2.16E-15	27.0993174	16.31993572	up-regulated in Low
THOC1	0.15786181	1.95248069	3.60229956	0.00034724	0.00187601	-1.7767425	3.459376514	up-regulated in Low
COLEC12	-0.9891687	2.46486808	-10.998529	2.52E-25	2.91E-23	45.9926833	24.59917361	up-regulated in High
CLUL1	-0.3494683	0.58994502	-7.1430076	3.28E-12	7.74E-11	16.1218283	11.4843767	up-regulated in High
TYMS	0.99513171	3.23245842	14.1498189	2.02E-38	5.40E-36	75.9863226	37.69378796	up-regulated in Low
YES1	0.38536675	3.85227824	7.19499767	2.32E-12	5.60E-11	16.459922	11.63377728	up-regulated in Low
METTL4	0.24918162	1.62170985	6.7036043	5.55E-11	1.09E-09	13.3449217	10.25571488	up-regulated in Low
NDC80	1.22633177	1.90911506	18.7155754	1.63E-59	1.80E-56	124.416564	58.78714338	up-regulated in Low
SMCHD1	0.18923651	2.57264994	3.55489002	0.00041437	0.00219467	-1.9423333	3.382608524	up-regulated in Low
EMILIN2	-0.1666538	2.42870232	-2.4620453	0.01415408	0.04664629	-5.1733109	1.849118452	up-regulated in High
LPIN2	-0.352959	4.18963452	-4.2252446	2.84E-05	0.00020006	0.59071784	4.546450038	up-regulated in High
TGIF1	0.34555651	3.486195	6.69900271	5.71E-11	1.12E-09	13.3166136	10.243174	up-regulated in Low
LAMA1	0.11524174	0.31355103	2.42874489	0.01550586	0.05032464	-5.2538733	1.809504284	up-regulated in Low
PTPRM	-0.302158	2.50082966	-4.2502989	2.55E-05	0.00018152	0.6933053	4.593198421	up-regulated in High
NDUFV2	0.27174525	2.51057554	5.61965415	3.20E-08	4.01E-07	7.13692564	7.495305677	up-regulated in Low
ANKRD12	-0.1373836	2.22383036	-2.8997156	0.00390082	0.01560835	-4.0154049	2.408844301	up-regulated in High
RALBP1	0.22824446	4.11340196	4.71218223	3.19E-06	2.73E-05	2.68475995	5.496436228	up-regulated in Low
RAB31	-0.1667701	4.55451315	-2.1214973	0.03437568	0.09666317	-5.9465078	1.463748724	up-regulated in High
APCDD1	-0.2922559	1.84205394	-3.9688997	8.29E-05	0.00052692	-0.4263602	4.081541054	up-regulated in High
PIEZO2	-0.2759403	0.79804925	-5.892741	7.02E-09	9.86E-08	8.61208808	8.153415728	up-regulated in High
CHMP1B	0.19031008	4.80205628	3.29931437	0.00103897	0.00492388	-2.798976	2.983398197	up-regulated in Low
IMPA2	0.21115801	4.23525364	2.2666595	0.02384124	0.071835	-5.6306825	1.62267114	up-regulated in Low
TUBB6	0.17066091	3.49836059	2.03803322	0.04207708	0.11377552	-6.1188069	1.375954361	up-regulated in Low
AFG3L2	0.27916697	3.12325641	6.37760016	4.13E-10	7.13E-09	11.3798397	9.384287845	up-regulated in Low
SLMO1	0.22952873	0.97914384	5.77928196	1.33E-08	1.78E-07	7.99177596	7.876876755	up-regulated in Low
PSMG2	0.17123031	3.91924204	4.81555927	1.95E-06	1.75E-05	3.15624457	5.709271945	up-regulated in Low
CEP76	0.2631831	1.13927889	8.68345822	5.58E-17	2.51E-15	26.9470625	16.25303901	up-regulated in Low
SEH1L	0.35536735	2.36425611	8.73915512	3.65E-17	1.66E-15	27.3683041	16.43811198	up-regulated in Low
LDLRAD4	-0.3654573	0.98538798	-8.3950725	4.92E-16	1.98E-14	24.7979368	15.30834693	up-regulated in High
FAM210A	0.35706404	2.04529331	8.76098942	3.08E-17	1.42E-15	27.5339778	16.51089303	up-regulated in Low
GREB1L	0.23192787	0.45083227	5.09122471	5.06E-07	5.10E-06	4.45885318	6.295676908	up-regulated in Low
ESCO1	0.135839	2.47226484	3.50233392	0.00050292	0.00259998	-2.1234626	3.298498455	up-regulated in Low
SNRPD1	0.65044952	3.33874712	14.1979192	1.24E-38	3.38E-36	76.4718917	37.90550114	up-regulated in Low
ABHD3	0.30723303	2.86244857	5.30299803	1.72E-07	1.89E-06	5.50382808	6.76462007	up-regulated in Low
MIB1	0.1595275	2.81063381	3.14977975	0.00173262	0.00770997	-3.2718314	2.76129641	up-regulated in Low
GATA6	-0.6748895	1.93159145	-8.4680675	2.85E-16	1.17E-14	25.3368036	15.54529375	up-regulated in High
RBBP8	0.22570483	3.24162721	3.9706345	8.23E-05	0.00052407	-0.4196773	4.084605077	up-regulated in Low
CABLES1	-0.432022	2.43431297	-5.3728353	1.19E-07	1.35E-06	5.85677357	6.922747361	up-regulated in High
ANKRD29	-0.4776201	1.54059312	-5.1806201	3.22E-07	3.38E-06	4.8953082	6.491687298	up-regulated in High
LAMA3	0.36331595	2.32861319	2.86797853	0.00430714	0.01700028	-4.1055316	2.36581112	up-regulated in Low
TTC39C	0.27445056	2.28120374	3.79645558	0.00016497	0.00097343	-1.0769339	3.782597356	up-regulated in Low
CABYR	0.72007499	1.57863742	6.15446201	1.56E-09	2.46E-08	10.0827661	8.808021079	up-regulated in Low
OSBPL1A	-0.1618705	2.44734696	-2.6365547	0.0086381	0.03074195	-4.7336499	2.063582029	up-regulated in High
ZNF521	-0.3238363	1.19064183	-5.7108983	1.94E-08	2.53E-07	7.62299308	7.712336769	up-regulated in High
AQP4	-1.5260163	2.92046293	-9.5493961	5.99E-20	3.90E-18	33.7134295	19.22272659	up-regulated in High
CHST9	-0.2326491	0.40758957	-3.5002456	0.00050678	0.00261739	-2.1306069	3.295177816	up-regulated in High
CDH2	0.47724292	0.87886339	5.04868643	6.26E-07	6.20E-06	4.25357051	6.203407865	up-regulated in Low
DSC2	0.40079017	2.24582432	4.99153504	8.31E-07	8.04E-06	3.98021522	6.08046161	up-regulated in Low
DSG3	0.20453602	0.20327928	3.16917587	0.00162321	0.00728695	-3.211685	2.78962645	up-regulated in Low
DSG2	0.56078254	4.82708518	6.03833255	3.06E-09	4.60E-08	9.42339754	8.514703323	up-regulated in Low
B4GALT6	0.36111683	0.87204031	6.84482166	2.27E-11	4.76E-10	14.2215152	10.64389175	up-regulated in Low
RNF125	-0.2504849	1.20565841	-5.776423	1.35E-08	1.81E-07	7.9762813	7.869965561	up-regulated in High
RNF138	0.17652555	2.95746935	3.59382528	0.00035843	0.00192741	-1.8064941	3.445592287	up-regulated in Low
GAREM	-0.1551734	1.96960633	-2.5397975	0.01139602	0.03889533	-4.9810426	1.943246711	up-regulated in High
NOL4	0.15276946	0.15160093	3.70714614	0.00023326	0.00132523	-1.4031533	3.632158053	up-regulated in Low
MAPRE2	-0.1301696	3.10324018	-2.3824856	0.01757235	0.05579652	-5.3640086	1.755170186	up-regulated in High
ZNF397	-0.1350384	2.05041629	-2.5784959	0.01021059	0.03541858	-4.8831772	1.990949356	up-regulated in High
ZNF396	-0.1384088	0.76311357	-4.7075459	3.26E-06	2.78E-05	2.66383333	5.486981687	up-regulated in High
INO80C	0.12211688	1.75006823	3.05395109	0.00237999	0.0101676	-3.5637799	2.623424438	up-regulated in Low
C18orf21	0.17621788	2.74433795	3.91228316	0.00010419	0.00064711	-0.6429516	3.982164648	up-regulated in Low
RPRD1A	0.21629661	2.9415084	4.04858594	5.98E-05	0.00039261	-0.1165702	4.22343959	up-regulated in Low
SLC39A6	0.25615203	4.46015642	3.70188872	0.00023802	0.00134818	-1.4221282	3.623395531	up-regulated in Low
MOCOS	0.33744733	1.85511383	5.22905047	2.52E-07	2.69E-06	5.13460606	6.599064199	up-regulated in Low
FHOD3	0.14930937	0.8411406	2.07393668	0.03860106	0.10620019	-6.0455224	1.413400752	up-regulated in Low
KIAA1328	-0.1180616	0.72162212	-4.0736056	5.39E-05	0.00035821	-0.0181139	4.268481067	up-regulated in High
SYT4	0.17449051	0.16916583	2.47952802	0.01348702	0.0448418	-5.1305871	1.870083854	up-regulated in Low
SETBP1	-0.2360675	1.06022767	-4.2844171	2.20E-05	0.0001591	0.83391271	4.657232957	up-regulated in High
SLC14A1	-0.2197885	0.34721756	-6.3358111	5.31E-10	9.00E-09	11.1339317	9.275105582	up-regulated in High
EPG5	-0.1001648	1.94478847	-2.286098	0.02266969	0.06881286	-5.5868361	1.644554394	up-regulated in High

PSTPIP2	-0.1380911	2.06685312	-2.2351905	0.0258496	0.07679089	-5.7008878	1.58754619	up-regulated in High
ATP5A1	0.13691625	5.01732754	3.00733473	0.00276939	0.01158116	-3.7026595	2.557616605	up-regulated in Low
HAUS1	0.34803787	2.91893089	6.46720359	2.40E-10	4.30E-09	11.9117257	9.620337553	up-regulated in Low
HDHD2	-0.1543304	2.34753554	-4.0041154	7.18E-05	0.0004635	-0.2901663	4.143958036	up-regulated in High
ZBTB7C	-0.5331296	1.30401879	-6.7234603	4.90E-11	9.75E-10	13.4672579	10.30990758	up-regulated in High
CTIF	-0.2195614	2.13544461	-4.5663048	6.27E-06	5.07E-05	2.03537747	5.202713595	up-regulated in High
SMAD7	-0.5019639	3.11736315	-9.5638314	5.32E-20	3.49E-18	33.8300453	19.27385576	up-regulated in High
RPL17	0.23258661	3.82788126	4.04731851	6.01E-05	0.00039451	-0.1215426	4.221164115	up-regulated in Low
CFAP53	-0.2779169	1.11724261	-3.8514249	0.00013284	0.00080273	-0.8725066	3.876680685	up-regulated in High
SKA1	1.08841817	1.42961937	18.8704403	2.97E-60	3.54E-57	126.117093	59.52720969	up-regulated in Low
MAPK4	0.21190209	0.41760574	3.04330507	0.00246421	0.01047907	-3.5956779	2.608322838	up-regulated in Low
SMAD4	-0.1059561	2.50348415	-2.7570465	0.00604782	0.02275953	-4.4130093	2.218401164	up-regulated in High
POLI	-0.1223753	1.56168574	-3.0796701	0.00218733	0.00945034	-3.4862771	2.660084978	up-regulated in High
C18orf54	0.37358662	0.7740616	11.0898544	1.11E-25	1.32E-23	46.8029432	24.95351251	up-regulated in Low
RAB27B	-0.3610472	2.66322613	-3.4007504	0.0007263	0.00359225	-2.4662755	3.138884131	up-regulated in High
CCDC68	-0.3359716	1.59567432	-5.0293331	6.89E-07	6.76E-06	4.16068856	6.161643062	up-regulated in High
TCF4	-0.2936677	1.60115672	-5.498022	6.16E-08	7.38E-07	6.49970935	7.210488891	up-regulated in High
TXNL1	0.18522111	2.70436991	4.53626747	7.19E-06	5.74E-05	1.90399283	5.14319882	up-regulated in Low
FECH	0.31527697	2.87155825	5.47921589	6.81E-08	8.10E-07	6.40228674	7.166912602	up-regulated in Low
NARS	0.32776437	5.07624971	7.38084106	6.69E-13	1.72E-11	17.6846477	12.17466371	up-regulated in Low
NEDD4L	-0.3492233	2.84527245	-4.8670806	1.52E-06	1.40E-05	3.39470267	5.816790747	up-regulated in High
GRP	-0.2571587	1.0340382	-2.2687398	0.02371339	0.07150696	-5.6260075	1.625006315	up-regulated in High
LMAN1	0.28208431	4.89130631	5.09696374	4.92E-07	4.98E-06	4.48666752	6.308174824	up-regulated in Low
PMAIP1	0.56061791	2.98579578	5.20039216	2.91E-07	3.08E-06	4.99276227	6.535424122	up-regulated in Low
CDH20	-0.1518154	0.16931159	-5.562509	4.36E-08	5.36E-07	6.83601688	7.360851781	up-regulated in High
RNF152	-0.1004937	0.88648953	-2.3968828	0.01690465	0.05403746	-5.3299531	1.771993832	up-regulated in High
TNFRSF11A	-0.1555359	1.00096073	-2.9771063	0.00305222	0.01260502	-3.7916154	2.51538453	up-regulated in High
PHLPP1	0.18169565	1.6499105	3.6475441	0.0002928	0.00161723	-1.6167728	3.533429199	up-regulated in Low
BCL2	-0.166587	1.4549314	-2.3527343	0.01902598	0.05962481	-5.4337473	1.720652978	up-regulated in High
SERPINB5	1.09443694	1.22048767	7.84579187	2.67E-14	8.36E-13	20.8572392	13.57379878	up-regulated in Low
SERPINB4	0.28937343	0.47616115	3.33895993	0.00090426	0.0043519	-2.6700885	3.043704923	up-regulated in Low
SERPINB3	0.3828886	0.92334773	2.75996866	0.00599481	0.02258188	-4.4050604	2.222224352	up-regulated in Low
SERPINB7	0.21221926	0.34870355	3.10342322	0.00202225	0.00882457	-3.414143	2.694166333	up-regulated in Low
SERPINB8	-0.142525	2.03336112	-2.8314044	0.00482288	0.01871798	-4.2082041	2.316693823	up-regulated in High
DSEL	-0.1052809	0.90257566	-2.0418578	0.04169459	0.1130344	-6.1110603	1.379920317	up-regulated in High
CCDC102B	-0.103799	1.1291805	-2.5074351	0.01247987	0.0419833	-5.0617776	1.903789987	up-regulated in High
RTTN	0.21972519	1.01776157	6.71830955	5.06E-11	1.00E-09	13.4354943	10.2958375	up-regulated in Low
FBXO15	-0.1079619	0.30178168	-3.7226482	0.00021976	0.00125745	-1.3470554	3.658055835	up-regulated in High
TIMM21	0.23436904	1.983914	6.90475385	1.55E-11	3.32E-10	14.5981088	10.81056141	up-regulated in Low
CYB5A	-0.8075391	3.92457004	-8.9800075	5.65E-18	2.89E-16	29.2124498	17.24801146	up-regulated in High
TSHZ1	-0.1142959	2.04953987	-2.00378	0.04563747	0.12143438	-6.1875498	1.340678484	up-regulated in High
ZNF516	-0.1552825	1.89459427	-2.7045602	0.00707482	0.02596014	-4.5543905	2.150284409	up-regulated in High
MBP	-0.12548	1.8748204	-2.7349232	0.00646306	0.0240693	-4.4729236	2.189562096	up-regulated in High
SALL3	0.12665742	0.10362361	3.63960459	0.00030173	0.00165965	-1.6449813	3.520378617	up-regulated in Low
ATP9B	-0.1295595	1.34835397	-3.9405451	9.30E-05	0.00058404	-0.5351984	4.031621743	up-regulated in High
NFATC1	-0.3735624	1.48597434	-8.5583057	1.44E-16	6.21E-15	26.0077721	15.84025419	up-regulated in High
TXNL4A	0.2665028	3.16907281	6.25438559	8.63E-10	1.41E-08	10.658736	9.064029003	up-regulated in Low
RBFA	0.11503312	2.47821508	2.64737472	0.00837029	0.02991652	-4.7054255	2.077259435	up-regulated in Low
ADNP2	0.16337856	2.57688138	3.83929145	0.00013938	0.00083842	-0.9178681	3.855816135	up-regulated in Low
PARD6G	0.13154309	1.41021139	2.64218285	0.00849785	0.03030974	-4.7189827	2.070690934	up-regulated in Low
SHC2	-0.4746745	2.31889106	-5.2525104	2.23E-07	2.41E-06	5.25124019	6.651377397	up-regulated in High
CDC34	0.34134853	4.65332961	6.20938958	1.13E-09	1.81E-08	10.398392	8.948334407	up-regulated in Low
BSG	0.1936062	7.14403997	3.58092812	0.00037613	0.00201188	-1.851646	3.4246658	up-regulated in Low
HCN2	0.10849544	0.46901026	2.22365513	0.02662171	0.07861827	-5.7263816	1.57476406	up-regulated in Low
POLRMT	0.17767031	2.99904799	3.91519921	0.00010298	0.00064066	-0.6318674	3.987253777	up-regulated in Low
RNF126	0.28778179	3.12694293	6.32740714	5.58E-10	9.42E-09	11.0846445	9.253218407	up-regulated in Low
PALM	-0.4069311	2.08424301	-5.7404356	1.65E-08	2.17E-07	7.78181215	7.783209809	up-regulated in High
MISP	0.2626266	4.34147647	2.29187013	0.02233162	0.06797739	-5.5737457	1.65107986	up-regulated in Low
PTBP1	0.34562463	5.30573892	10.4447801	3.25E-23	3.05E-21	41.1687001	22.48857827	up-regulated in Low
AZU1	-0.2689231	0.3698031	-4.7629983	2.51E-06	2.19E-05	2.91535648	5.600573319	up-regulated in High
ELANE	-0.3337731	0.37630111	-5.7276686	1.77E-08	2.32E-07	7.71307737	7.752539281	up-regulated in High
CFD	-0.6716049	3.86378368	-5.7874058	1.27E-08	1.71E-07	8.03584187	7.896530806	up-regulated in High
R3HDM4	0.10474339	4.98592196	2.13563347	0.03319886	0.0940095	-5.9166533	1.478876818	up-regulated in Low
KISS1R	0.18386586	0.78187524	2.32090824	0.02069671	0.06386655	-5.5074008	1.684098604	up-regulated in Low
ARID3A	0.39721133	1.7927691	6.31265824	6.10E-10	1.02E-08	10.9982802	9.214863138	up-regulated in Low
WDR18	0.35242681	3.68710887	6.64949715	7.79E-11	1.50E-09	13.0130907	10.10868737	up-regulated in Low
GRIN3B	-0.1142351	0.50894693	-2.2505556	0.02485137	0.07435289	-5.6667295	1.604649729	up-regulated in High
CNN2	-0.19954	5.913173	-3.3118107	0.00099462	0.00473987	-2.7585095	3.002342665	up-regulated in High
ABCA7	-0.1783484	2.37518216	-2.4646459	0.01405304	0.04636987	-5.1669744	1.852229744	up-regulated in High
HMHA1	-0.4385436	3.51942563	-6.5226697	1.71E-10	3.14E-09	12.2441111	9.767778525	up-regulated in High
POLR2E	0.1672384	4.64698386	4.08619309	5.11E-05	0.00034154	0.03163414	4.291229702	up-regulated in Low

STK11	-0.1779878	2.59984649	-3.087934	0.00212854	0.00922842	-3.4612417	2.671917817	up-regulated in High
C19orf26	0.14559297	0.50715136	4.16738428	3.64E-05	0.00025047	0.35596011	4.439378551	up-regulated in Low
ATP5D	0.26296637	4.27975722	4.35912849	1.59E-05	0.00011832	1.14545342	4.798957152	up-regulated in Low
CIRBP	-0.5842501	4.93483887	-10.027671	1.14E-21	9.07E-20	37.6405953	20.94373899	up-regulated in High
C19orf24	0.20282335	3.88657743	3.63116241	0.00031151	0.00170621	-1.6749117	3.506527843	up-regulated in Low
EFNA2	0.1875465	0.42552654	3.45861212	0.00058976	0.00298937	-2.2721886	3.229321808	up-regulated in Low
DAZAP1	0.23550705	3.58887937	6.47508579	2.28E-10	4.11E-09	11.9588145	9.641228664	up-regulated in Low
RPS15	0.14047269	6.62083094	2.18012821	0.02971778	0.08600833	-5.8214135	1.526983626	up-regulated in Low
PCSK4	-0.1579111	1.04246754	-2.9476146	0.00335339	0.01368903	-3.8775671	2.474516423	up-regulated in High
REEP6	-0.3015301	2.83728227	-2.5237428	0.01192277	0.04039543	-5.0212206	1.92362301	up-regulated in High
MEX3D	0.22051275	3.33088415	3.41892086	0.00068054	0.00339132	-2.4056622	3.167148136	up-regulated in Low
MBD3	0.1968386	3.02389133	3.89397493	0.00011213	0.00069026	-0.7123656	3.950285637	up-regulated in Low
TCF3	0.27797665	3.80940154	4.96553492	9.44E-07	9.05E-06	3.8567877	6.024916928	up-regulated in Low
ATP8B3	0.17508693	0.66313059	2.98928704	0.00293521	0.01218733	-3.7558743	2.532360504	up-regulated in Low
REXO1	0.13955529	2.7722667	3.10061918	0.00204112	0.00889364	-3.4226862	2.690131897	up-regulated in Low
KLF16	0.34693462	2.94710059	6.74715629	4.22E-11	8.51E-10	13.6136471	10.37474693	up-regulated in Low
ABHD17A	-0.101627	2.46071384	-2.3103977	0.02127597	0.06536731	-5.5315091	1.672110544	up-regulated in High
SCAMP4	-0.1177536	3.77945193	-2.6913953	0.00735592	0.02680125	-4.5894386	2.133363081	up-regulated in High
CSNK1G2	0.11978813	4.03795983	2.42686264	0.01558556	0.05054199	-5.258395	1.807277684	up-regulated in Low
BTBD2	0.11263869	3.97753539	2.45200176	0.01455035	0.04770885	-5.1977217	1.837126452	up-regulated in Low
MKNK2	-0.116422	5.13786824	-2.0489028	0.04099774	0.11154403	-6.0967533	1.387240113	up-regulated in High
IZUMO4	-0.1376312	0.95478796	-2.4051484	0.0165315	0.05304597	-5.3103109	1.781687855	up-regulated in High
AP3D1	0.11585643	4.40720299	2.38518531	0.01744541	0.05546338	-5.3576379	1.758318919	up-regulated in Low
DOT1L	0.2210673	1.91495767	4.69738057	3.42E-06	2.90E-05	2.61801712	5.466279735	up-regulated in Low
PLEKHJ1	0.16274396	3.24616733	3.41837375	0.00068188	0.00339669	-2.4074917	3.166295281	up-regulated in Low
SF3A2	0.25034162	4.19521163	4.79469408	2.16E-06	1.91E-05	3.06033066	5.66600196	up-regulated in Low
C19orf35	-0.133382	0.37594182	-6.4754441	2.28E-10	4.11E-09	11.9609563	9.642178858	up-regulated in High
LSM7	0.23662642	4.32246013	4.21410361	2.98E-05	0.00020906	0.54528095	4.525736886	up-regulated in Low
TIMM13	0.32620329	4.45526524	6.29833539	6.64E-10	1.11E-08	10.9145749	9.177684939	up-regulated in Low
LMNB2	0.78710929	3.82961405	12.6305588	6.70E-32	1.23E-29	61.0414793	31.17401999	up-regulated in Low
GADD45B	-0.3185969	4.67333742	-3.827403	0.00014607	0.00087442	-0.962183	3.83542642	up-regulated in High
GNG7	-0.6623687	1.43277019	-12.523033	1.88E-31	3.36E-29	60.0153164	30.72604806	up-regulated in High
DIRAS1	-0.2055626	1.24848716	-2.2554189	0.02454246	0.07361352	-5.6558699	1.610081833	up-regulated in High
SGTA	0.2744473	4.28332191	7.15318104	3.07E-12	7.28E-11	16.18783	11.51354526	up-regulated in Low
THOP1	0.49362853	2.44571428	11.401345	6.69E-27	8.60E-25	49.5965673	26.17487504	up-regulated in Low
ZNF554	-0.1872651	1.07261	-6.368897	4.35E-10	7.49E-09	11.3285128	9.361501487	up-regulated in High
TLE2	-0.5591608	3.49912656	-7.1755271	2.64E-12	6.32E-11	16.3330709	11.5777274	up-regulated in High
AES	-0.1495325	5.84424535	-3.1893089	0.00151641	0.00687838	-3.1488785	2.819183839	up-regulated in High
GNA15	-0.1960557	3.53961131	-2.7038443	0.00708986	0.02599896	-4.5563005	2.149362636	up-regulated in High
S1PR4	-0.4642188	2.0827229	-6.9132674	1.46E-11	3.16E-10	14.6518248	10.8343301	up-regulated in High
NCLN	0.25777054	4.45422224	5.41153877	9.75E-08	1.13E-06	6.05414335	7.011120096	up-regulated in Low
NFIC	-0.3850477	3.87332409	-6.5470819	1.47E-10	2.73E-09	12.3911607	9.832990577	up-regulated in High
DOHH	0.21260326	2.47130116	4.19874293	3.18E-05	0.00022202	0.48281789	4.497254066	up-regulated in Low
FZR1	0.10272794	3.16945799	2.42535765	0.01564954	0.05070643	-5.262008	1.805498322	up-regulated in Low
MFSD12	0.37319398	3.38642179	5.94475382	5.23E-09	7.52E-08	8.89996034	8.281659345	up-regulated in Low
HMG20B	0.16161456	4.0267823	3.46342841	0.00057955	0.00294247	-2.2558925	3.236906724	up-regulated in Low
MATK	-0.1768381	1.05075691	-3.3910291	0.00075195	0.00370496	-2.4985772	3.123814142	up-regulated in High
PIAS4	0.12465073	2.70910546	3.16538754	0.00164406	0.00736868	-3.2234603	2.78408198	up-regulated in Low
ZBTB7A	-0.1575303	3.1705545	-3.4361355	0.00063968	0.00321031	-2.3479532	3.194040538	up-regulated in High
MAP2K2	0.17741466	4.33786093	4.38704847	1.40E-05	0.00010557	1.26315807	4.852448895	up-regulated in Low
SIRT6	0.12940162	2.88626127	2.96631902	0.00315938	0.01299154	-3.82315	2.500397761	up-regulated in Low
ANKRD24	-0.2207652	0.56506509	-5.0822342	5.30E-07	5.32E-06	4.41533713	6.276121843	up-regulated in High
EBI3	-0.2209116	1.5539858	-3.2584366	0.00119718	0.00557948	-2.9303264	2.921840724	up-regulated in High
FSD1	0.24114178	0.29545811	6.08314348	2.36E-09	3.62E-08	9.6765476	8.627347686	up-regulated in Low
MPND	-0.2619238	3.3718123	-5.353534	1.32E-07	1.49E-06	5.75881648	6.878872854	up-regulated in High
SH3GL1	0.23759059	4.5065662	5.06945317	5.64E-07	5.64E-06	4.35359341	6.248371815	up-regulated in Low
CHAF1A	0.70093072	2.36584125	14.2153335	1.04E-38	2.86E-36	76.6478584	37.98222286	up-regulated in Low
UBXN6	-0.1376084	4.31147602	-3.5190499	0.000473	0.0024667	-2.0661308	3.325137387	up-regulated in High
HDGFRP2	0.19725182	3.50716306	4.15253165	3.87E-05	0.0002653	0.29618489	4.41209395	up-regulated in Low
PLIN5	-0.3355562	0.78823777	-6.1066814	2.06E-09	3.19E-08	9.81016561	8.686787827	up-regulated in High
CTB-50L17.1[illegible]	-0.1311607	0.26779856	-4.7877222	2.23E-06	1.97E-05	3.0283666	5.651578887	up-regulated in High
LRG1	-0.4159376	3.82144357	-3.8304502	0.00014433	0.00086492	-0.950837	3.840647453	up-regulated in High
TNFAIP8L1	0.1098132	2.02610649	2.07490877	0.03851046	0.10599741	-6.0435207	1.414421328	up-regulated in Low
MYDGF	0.1554713	6.09143808	3.01420125	0.00270858	0.01135861	-3.6823321	2.56725817	up-regulated in Low
TICAM1	0.13391761	3.41006761	2.46692989	0.01396483	0.0461205	-5.1614038	1.854964427	up-regulated in Low
PLIN3	0.22703441	4.82127829	3.74833005	0.000199	0.00115094	-1.2536329	3.701158769	up-regulated in Low
UHRF1	1.01717396	1.86910631	15.8953145	2.83E-46	1.19E-43	94.0086376	45.54791146	up-regulated in Low
PTPRS	-0.2102933	2.52067243	-2.7965504	0.00536588	0.02055083	-4.3048607	2.270359314	up-regulated in High
SAFB	0.13918399	3.71874659	3.2039959	0.00144264	0.00658623	-3.1028205	2.840842875	up-regulated in Low
HSD11B1L	-0.1562641	1.55277996	-3.4827657	0.0005402	0.00276549	-2.1902465	3.267448348	up-regulated in High
LONP1	0.3210618	4.25651874	6.74017212	4.41E-11	8.86E-10	13.5704558	10.35561735	up-regulated in Low

CATSPERD	-0.1707597	0.21511862	-4.5555788	6.59E-06	5.29E-05	1.98836987	5.181423586	up-regulated in High
PRR22	0.10513692	0.88455249	2.21650529	0.02711024	0.0798107	-5.7421182	1.566866608	up-regulated in Low
DUS3L	0.2764683	2.59165127	5.99006521	4.04E-09	5.93E-08	9.15252891	8.39412983	up-regulated in Low
NRTN	0.17584059	0.94164078	2.99286833	0.0029016	0.01206458	-3.7453392	2.537362346	up-regulated in Low
FUT6	-0.207244	0.99183256	-2.045789	0.04130451	0.11220091	-6.1030828	1.384002552	up-regulated in High
FUT3	-0.489798	3.11320541	-4.718619	3.09E-06	2.66E-05	2.71384441	5.509575289	up-regulated in High
NDUFA11	0.11197287	3.11803542	2.04517381	0.04136534	0.1123348	-6.1043321	1.383363388	up-regulated in Low
VMAC	-0.2899485	1.77346719	-7.8263827	3.06E-14	9.50E-13	20.7217571	13.51410355	up-regulated in High
CAPS	-0.8323164	3.09247224	-7.4998279	2.97E-13	8.04E-12	18.4819234	12.52652652	up-regulated in High
RFX2	-0.3231342	1.28983177	-6.2002246	1.19E-09	1.91E-08	10.3455608	8.924852015	up-regulated in High
MLLT1	-0.1574592	4.03296716	-2.9593522	0.00323041	0.01324723	-3.8434575	2.490742274	up-regulated in High
CLPP	0.2037796	4.08410383	4.72665077	2.98E-06	2.57E-05	2.75018625	5.525991041	up-regulated in Low
KHSRP	0.31819264	5.1280936	6.9277572	1.33E-11	2.89E-10	14.743373	10.87483654	up-regulated in Low
SLC25A23	-0.4351732	3.9788589	-7.9857707	9.83E-15	3.27E-13	21.8420434	14.00758691	up-regulated in High
CRB3	-0.2329176	3.99130516	-3.4279034	0.00065892	0.00329613	-2.3755843	3.181166529	up-regulated in High
DENND1C	-0.3087071	2.87693922	-5.9060862	6.51E-09	9.21E-08	8.68573975	8.18623204	up-regulated in High
TUBB4A	0.35791328	0.68771524	4.57549194	6.01E-06	4.87E-05	2.07572173	5.220982621	up-regulated in Low
CD70	0.1432202	0.72146006	2.47478817	0.01366506	0.04532277	-5.1421994	1.864388351	up-regulated in Low
TNFSF14	-0.1350254	0.84289257	-2.5003512	0.01272899	0.04272878	-5.0793152	1.895206149	up-regulated in High
C3	-1.1431496	6.52126326	-8.3836064	5.35E-16	2.14E-14	24.7136094	15.27126219	up-regulated in High
GPR108	-0.2300735	4.4465198	-5.8410703	9.40E-09	1.29E-07	8.32828745	8.026929856	up-regulated in High
VAV1	-0.4129421	2.63216502	-5.7659809	1.43E-08	1.91E-07	7.91974477	7.844746659	up-regulated in High
ZNF557	-0.105043	1.21283191	-3.5108749	0.00048742	0.00253284	-2.0942016	3.312096348	up-regulated in High
CTB-133G6.1	-0.1916602	0.43787514	-5.9662912	4.62E-09	6.73E-08	9.01980394	8.335031759	up-regulated in High
PEX11G	-0.2282198	1.78034399	-4.6349138	4.57E-06	3.79E-05	2.33846289	5.339890179	up-regulated in High
C19orf45	0.1596185	0.3242902	4.6603154	4.06E-06	3.41E-05	2.45172792	5.391113984	up-regulated in Low
MCOLN1	-0.1070704	2.90020841	-2.4344412	0.01526684	0.04968906	-5.2401685	1.81625082	up-regulated in High
PCP2	-0.2185475	0.93439761	-3.2503233	0.00123112	0.00571896	-2.9562098	2.909698369	up-regulated in High
STXBP2	-0.1003347	3.50298623	-2.1372175	0.03306917	0.09370589	-5.9132959	1.480576656	up-regulated in High
RETN	-0.7162503	1.56487575	-6.1531994	1.57E-09	2.47E-08	10.0755391	8.804807598	up-regulated in High
MCEMP1	-0.764754	1.90963285	-6.3257235	5.64E-10	9.51E-09	11.074777	9.248836347	up-regulated in High
FCER2	-0.3162677	0.46081647	-6.0373432	3.07E-09	4.62E-08	9.41782638	8.512223868	up-regulated in High
EVI5L	-0.1156226	2.01952736	-2.7951748	0.0053884	0.02062628	-4.3086517	2.268540061	up-regulated in High
CTXN1	0.27049319	2.42705221	2.69702659	0.00723447	0.02643461	-4.574467	2.140593169	up-regulated in Low
TIMM44	0.28668985	2.71568407	7.79393515	3.85E-14	1.18E-12	20.495848	13.41455506	up-regulated in Low
ELAVL1	0.2295256	3.56952228	8.77071707	2.86E-17	1.33E-15	27.6078864	16.54335993	up-regulated in Low
CERS4	-0.3628294	3.30651753	-3.610213	0.00033708	0.00182839	-1.7488999	3.472272878	up-regulated in High
CD320	0.26444722	4.01736526	3.89255848	0.00011277	0.00069371	-0.7177231	3.947824482	up-regulated in Low
NDUFA7	0.12905254	1.38693507	3.73495406	0.00020957	0.00120539	-1.3023664	3.678678315	up-regulated in Low
NDUFA7	0.12905254	1.38693507	3.73495406	0.00020957	0.00120539	-1.3023664	3.678678315	up-regulated in Low
KANK3	-0.3407397	1.1156361	-6.8902958	1.70E-11	3.62E-10	14.5070116	10.77024944	up-regulated in High
ANGPTL4	0.64429513	3.64930681	4.18197404	3.42E-05	0.00023685	0.41487106	4.466260003	up-regulated in Low
RAB11B	-0.102859	5.41026649	-2.2436299	0.02529709	0.07544986	-5.6821544	1.59692945	up-regulated in High
2-Mar	-0.6042363	2.74940947	-8.5835591	1.19E-16	5.18E-15	26.1964909	15.92320144	up-regulated in High
HNRNPM	0.26836825	5.10925424	6.66054302	7.27E-11	1.41E-09	13.0806509	10.13862581	up-regulated in Low
PRAM1	-0.3881769	1.20663825	-6.6888081	6.09E-11	1.19E-09	13.253956	10.21541463	up-regulated in High
MYO1F	-0.3528349	2.24190735	-5.3313443	1.48E-07	1.66E-06	5.64658896	6.828595043	up-regulated in High
ADAMTS10	-0.2902157	1.2062037	-5.2475528	2.29E-07	2.46E-06	5.22655379	6.640306162	up-regulated in High
MUC16	0.19797683	0.96722284	2.16664374	0.03073789	0.08840919	-5.8504802	1.512325953	up-regulated in Low
ZNF266	-0.2167704	2.60429545	-4.1024069	4.78E-05	0.00032146	0.09592572	4.320619037	up-regulated in High
ZNF121	0.13990844	2.42945678	2.79113499	0.00545505	0.02084176	-4.3197744	2.263201602	up-regulated in Low
ZNF561	-0.1623468	2.4031463	-4.2739015	2.30E-05	0.00016566	0.79046476	4.637450948	up-regulated in High
ZNF846	-0.3128125	1.29431166	-8.9073042	9.96E-18	4.90E-16	28.6519488	17.00190921	up-regulated in High
UBL5	0.15329106	5.61630946	2.97837741	0.00303981	0.01255912	-3.7878921	2.517153464	up-regulated in Low
OLFM2	-0.2824471	2.62367677	-3.0810815	0.00217719	0.0094122	-3.482006	2.662104026	up-regulated in High
C19orf66	-0.2196354	2.74525855	-4.5029177	8.37E-06	6.58E-05	1.75905462	5.077508161	up-regulated in High
PPAN	0.22499453	1.52243381	5.16630892	3.47E-07	3.61E-06	4.82497797	6.460116936	up-regulated in Low
DNMT1	0.37347703	3.32558268	6.3523179	4.81E-10	8.21E-09	11.2309021	9.318163847	up-regulated in Low
S1PR2	-0.2822273	2.67717386	-5.6959544	2.11E-08	2.72E-07	7.54291469	7.676594365	up-regulated in High
MRPL4	0.2264538	3.43787903	4.81004136	2.01E-06	1.79E-05	3.13084274	5.697813641	up-regulated in Low
ICAM1	-0.9715276	6.08361922	-8.5733805	1.29E-16	5.57E-15	26.120376	15.88974754	up-regulated in High
ICAM5	-0.7698075	1.55005419	-8.8951608	1.09E-17	5.36E-16	28.558651	16.96093986	up-regulated in High
RAVER1	0.20085022	2.6376957	3.88042352	0.00011837	0.00072443	-0.7635472	3.926770214	up-regulated in Low
ICAM3	-0.3089364	1.11308139	-5.6875046	2.21E-08	2.84E-07	7.49771714	7.656418549	up-regulated in High
TYK2	-0.1354368	3.64671742	-2.621719	0.00901777	0.03189682	-4.7721665	2.044900644	up-regulated in High
CDC37	0.18545242	5.53131578	4.5115571	8.04E-06	6.35E-05	1.7965068	5.094486403	up-regulated in Low
PDE4A	-0.3404425	2.72602669	-6.1617321	1.49E-09	2.36E-08	10.1244038	8.826534686	up-regulated in High
KEAP1	0.11900985	4.25679572	2.25475534	0.02458442	0.07371631	-5.657353	1.609340128	up-regulated in Low
S1PR5	0.13614113	0.41047319	3.40587309	0.00071312	0.00353423	-2.4492183	3.146839824	up-regulated in Low
ATG4D	0.25263075	2.99423045	5.42694449	8.99E-08	1.04E-06	6.13305565	7.046442853	up-regulated in Low
KRI1	0.15431603	3.07291186	3.38715777	0.00076239	0.00374947	-2.5114163	3.117822707	up-regulated in Low

CDKN2D	0.34942322	2.55398829	6.42439411	3.11E-10	5.48E-09	11.6568252	9.507231107	up-regulated in Low
SLC44A2	-0.2365576	5.26747313	-4.8781248	1.45E-06	1.33E-05	3.44611934	5.83996355	up-regulated in High
ILF3	0.26892976	4.57708488	6.30153711	6.52E-10	1.09E-08	10.9332722	9.185989809	up-regulated in Low
DNM2	-0.1762524	3.73680959	-4.2221481	2.88E-05	0.00020256	0.578078	4.540688449	up-regulated in High
C19orf38	-0.2158492	1.38953779	-3.794453	0.00016627	0.0009805	-1.0843292	3.779191179	up-regulated in High
CARM1	0.23483136	3.29336307	5.41577275	9.53E-08	1.10E-06	6.07581107	7.020819594	up-regulated in Low
LDLR	-0.2176845	3.5927261	-2.6896957	0.00739293	0.02690086	-4.5939513	2.131183291	up-regulated in High
SPC24	1.03383137	1.87702248	17.2721033	1.13E-52	7.52E-50	108.69967	51.94573438	up-regulated in Low
KANK2	-0.4851224	3.52501458	-9.1528021	1.45E-18	7.99E-17	30.5576986	17.8384929	up-regulated in High
CCDC159	-0.338215	2.13979873	-6.422234	3.15E-10	5.55E-09	11.6440015	9.501540012	up-regulated in High
LPPR2	-0.1577369	3.2888033	-2.8587734	0.00443199	0.01741859	-4.1314926	2.353401273	up-regulated in High
EPOR	-0.2535676	2.32468823	-4.0280681	6.51E-05	0.00042335	-0.196887	4.186676757	up-regulated in High
RGL3	-0.1994158	2.7439154	-2.3612017	0.0186019	0.0585062	-5.4139866	1.730442681	up-regulated in High
CCDC151	-0.1993292	0.69653768	-3.2627491	0.00117949	0.00550584	-2.9165432	2.928305028	up-regulated in High
PRKCSH	0.19720355	5.85340664	4.50643017	8.23E-06	6.48E-05	1.77427326	5.08440756	up-regulated in Low
ECSIT	0.17449461	3.55058969	3.81807876	0.00015154	0.00090313	-0.9968492	3.819471526	up-regulated in Low
CNN1	-0.4268133	2.23244288	-4.9313898	1.12E-06	1.05E-05	3.6955811	5.952341025	up-regulated in High
ACP5	-0.4724701	5.13024839	-5.526775	5.28E-08	6.41E-07	6.64923164	7.277352442	up-regulated in High
ZNF441	-0.2869079	1.11149097	-8.5567812	1.46E-16	6.27E-15	25.9963926	15.83525234	up-regulated in High
ZNF491	-0.1886841	0.53009616	-8.3008901	9.89E-16	3.79E-14	24.1078445	15.00482477	up-regulated in High
ZNF440	-0.22688	1.60347364	-4.4687574	9.76E-06	7.58E-05	1.61161543	5.010643784	up-regulated in High
ZNF439	-0.2035611	0.90441905	-4.8470284	1.68E-06	1.53E-05	3.30162026	5.774830305	up-regulated in High
ZNF69	-0.3601336	1.37159325	-7.4170858	5.23E-13	1.37E-11	17.9264273	12.28138831	up-regulated in High
ZNF700	-0.1952979	2.07433261	-4.2365028	2.71E-05	0.00019152	0.63674606	4.567427805	up-regulated in High
ZNF763	-0.2399004	0.59771053	-10.386787	5.35E-23	4.94E-21	40.6726057	22.27142179	up-regulated in High
ZNF433	-0.1361361	1.13632592	-3.5664695	0.00039694	0.00211247	-1.9020812	3.401280311	up-regulated in High
ZNF844	-0.211337	1.37441255	-4.7348603	2.87E-06	2.48E-05	2.78739104	5.54279446	up-regulated in High
ZNF136	-0.1694047	1.47749508	-5.2844291	1.89E-07	2.07E-06	5.41067724	6.722865479	up-regulated in High
ZNF44	-0.2590105	1.56618345	-7.1806339	2.56E-12	6.13E-11	16.3663149	11.59241699	up-regulated in High
ZNF563	-0.1837888	0.86555096	-6.7725635	3.59E-11	7.32E-10	13.7710838	10.44446946	up-regulated in High
ZNF791	-0.1752722	2.2858015	-5.7030633	2.02E-08	2.63E-07	7.58098545	7.693587595	up-regulated in High
MAN2B1	-0.3525545	4.50466159	-7.2049412	2.18E-12	5.26E-11	16.5248119	11.66244723	up-regulated in High
DHPS	0.13067581	3.86742381	3.42507047	0.00066567	0.00332475	-2.3850785	3.176742073	up-regulated in Low
FBXW9	0.11270991	2.47440524	2.28809672	0.02255213	0.06852367	-5.582307	1.646812535	up-regulated in Low
TNPO2	0.20790393	3.42289406	4.88943592	1.37E-06	1.27E-05	3.49888834	5.863742012	up-regulated in Low
C19orf43	0.11804847	5.79823216	2.97854218	0.00303821	0.01255515	-3.7874094	2.517382801	up-regulated in Low
ASNA1	0.14112906	5.19854063	3.05239717	0.00239212	0.01021417	-3.5684425	2.621217488	up-regulated in Low
HOOK2	-0.1191972	2.92011753	-1.9926362	0.04684934	0.12396413	-6.2096671	1.329296517	up-regulated in High
JUNB	-0.2812264	6.49454648	-3.6254507	0.00031829	0.00173912	-1.6951244	3.497172027	up-regulated in High
PRDX2	0.22517144	5.98326153	3.38077221	0.00077992	0.00382149	-2.532563	3.107952556	up-regulated in Low
RNASEH2A	0.83692622	3.49067342	14.5745741	2.67E-40	8.04E-38	80.2976946	39.57337077	up-regulated in Low
MAST1	0.15659701	0.40937704	4.46387899	9.97E-06	7.73E-05	1.59064414	5.001129843	up-regulated in Low
DNASE2	-0.152922	5.08329097	-3.8625994	0.00012707	0.00077205	-0.8306106	3.895945175	up-regulated in High
SYCE2	0.1830235	0.62948671	5.95270939	5.00E-09	7.23E-08	8.94418501	8.301355938	up-regulated in Low
FARSA	0.31863007	4.58107999	8.02208192	7.57E-15	2.56E-13	22.0997046	14.12104539	up-regulated in Low
CALR	0.14803932	8.75411158	3.62049933	0.00032429	0.00176805	-1.7126218	3.489071699	up-regulated in Low
RAD23A	0.24875516	5.17549393	7.50125162	2.95E-13	7.97E-12	18.4915245	12.53076265	up-regulated in Low
GADD45GIP1	0.33497458	4.38403808	5.7462307	1.60E-08	2.11E-07	7.81305586	7.797150048	up-regulated in Low
NFIX	-1.148064	3.4588432	-13.487018	1.56E-35	3.59E-33	69.3692139	34.80797009	up-regulated in High
LYL1	-0.3531749	1.49254976	-7.4008561	5.84E-13	1.52E-11	17.8180455	12.23354935	up-regulated in High
TRMT1	0.20550877	3.23511406	4.03449265	6.34E-05	0.00041335	-0.1717794	4.198170996	up-regulated in Low
NACC1	0.23386728	4.23063311	4.26523135	2.39E-05	0.00017135	0.75471618	4.621171295	up-regulated in Low
IER2	-0.1966997	4.81938007	-2.8009218	0.00529486	0.02031281	-4.2928015	2.276145333	up-regulated in High
CACNA1A	0.10151134	0.18216725	3.03105029	0.00256453	0.01084589	-3.6322633	2.590992483	up-regulated in Low
C19orf53	0.19702563	5.86619437	3.85683261	0.00013002	0.00078806	-0.8522459	3.885997626	up-regulated in Low
ZSWIM4	-0.2219836	2.78920257	-3.8347997	0.00014187	0.00085188	-0.9346266	3.848106164	up-regulated in High
C19orf57	0.15107501	0.70476274	3.34043511	0.00089958	0.00433329	-2.6652643	3.045960375	up-regulated in Low
PODNL1	-0.6106064	1.84872637	-5.9476147	5.14E-09	7.42E-08	8.91585809	8.288739966	up-regulated in High
RFX1	-0.1321076	2.36809044	-3.3645159	0.00082624	0.00401912	-2.5862261	3.08289493	up-regulated in High
RLN3	-0.1265403	0.44926457	-2.4846356	0.01329748	0.04432265	-5.1180497	1.876230732	up-regulated in High
IL27RA	-0.4709607	3.62219238	-5.5429323	4.84E-08	5.91E-07	6.73355558	7.315051848	up-regulated in High
PALM3	-0.519039	1.96868368	-4.5067654	8.22E-06	6.47E-05	1.77572621	5.085066234	up-regulated in High
CTB-55O6.8	-0.3526773	1.98393223	-4.9689866	9.28E-07	8.92E-06	3.87313993	6.032276868	up-regulated in High
SAMD1	0.30499714	4.12764245	6.21046701	1.12E-09	1.80E-08	10.4046071	8.951096804	up-regulated in Low
ASF1B	1.10872359	3.0530965	15.8432363	4.91E-46	2.00E-43	93.460185	45.30899641	up-regulated in Low
LPHN1	-0.1478718	2.35203649	-2.2054804	0.02787876	0.081597	-5.7662864	1.55472657	up-regulated in High
CD97	-0.4713282	4.39908174	-6.3052377	6.37E-10	1.07E-08	10.9548929	9.195592873	up-regulated in High
DDX39A	0.59060583	3.98505179	11.7250008	3.43E-28	4.97E-26	52.546874	27.46422256	up-regulated in Low
PTGER1	-0.1956164	0.6786227	-3.6477391	0.00029258	0.00161649	-1.6160793	3.533750001	up-regulated in High
NDUFB7	0.15498224	6.59384218	2.35671468	0.01882558	0.05908242	-5.4244668	1.725251587	up-regulated in Low
CLEC17A	-0.1825162	0.37881535	-5.070806	5.61E-07	5.61E-06	4.36012196	6.251306217	up-regulated in High

EMR3	-0.1268309	0.34916995	-4.3122442	1.95E-05	0.00014245	0.94936572	4.709778724	up-regulated in High
ZNF333	-0.1118093	0.9165095	-4.5664542	6.27E-06	5.06E-05	2.03603314	5.203010528	up-regulated in High
CASP14	0.33154844	0.19317816	4.87742717	1.45E-06	1.34E-05	3.44286847	5.838498538	up-regulated in Low
SYDE1	-0.1206656	1.97735577	-2.0412671	0.04175347	0.11312574	-6.1122576	1.379307465	up-regulated in High
ILVBL	0.15444039	3.38386626	3.16166362	0.0016648	0.0074485	-3.2350222	2.778637083	up-regulated in Low
NOTCH3	0.17950234	4.34514992	2.1459546	0.03236163	0.09207486	-5.8947329	1.489969583	up-regulated in Low
BRD4	0.17530506	3.21301689	3.96977743	8.26E-05	0.00052527	-0.4229792	4.083091211	up-regulated in Low
WIZ	0.11329891	2.75302598	2.52660216	0.0118274	0.04012704	-5.014083	1.927110856	up-regulated in Low
RASAL3	-0.3614279	1.99746973	-4.9517736	1.01E-06	9.62E-06	3.7916964	5.995616666	up-regulated in High
CYP4F3	0.7515842	0.72475516	6.71445302	5.18E-11	1.03E-09	13.4117252	10.28530838	up-regulated in Low
CYP4F11	0.59017264	1.22192838	4.42981859	1.16E-05	8.88E-05	1.44481534	4.934948837	up-regulated in Low
RAB8A	0.10750669	3.04404627	3.34446359	0.0008869	0.00428176	-2.6520798	3.052123864	up-regulated in Low
KLF2	-0.7646867	3.42880292	-8.7976464	2.33E-17	1.10E-15	27.812802	16.63337164	up-regulated in High
C19orf44	-0.102242	1.32744121	-2.8963241	0.00394251	0.01575465	-4.0250819	2.404227405	up-regulated in High
CHERP	0.19074565	3.02973862	4.24889127	2.57E-05	0.00018252	0.68752675	4.590565823	up-regulated in Low
SLC35E1	-0.1794836	3.67148868	-4.1838934	3.39E-05	0.00023516	0.42263537	4.469802271	up-regulated in High
MED26	-0.1175723	1.38011287	-4.1977123	3.20E-05	0.00022286	0.47863462	4.495346181	up-regulated in High
NWD1	-0.4209065	0.53724789	-6.4193178	3.21E-10	5.64E-09	11.6266943	9.493859052	up-regulated in High
CPAMD8	-0.9653187	1.604442	-10.350028	7.34E-23	6.66E-21	40.3590695	22.13416611	up-regulated in High
HAUS8	0.23148988	1.29097748	7.35690005	7.86E-13	2.01E-11	17.5254641	12.10438843	up-regulated in Low
MYO9B	-0.1242106	3.47210449	-2.3036363	0.02165606	0.06633004	-5.5469614	1.664420637	up-regulated in High
OCEL1	-0.1333367	3.24254363	-2.5347418	0.01155962	0.03937527	-4.9937217	1.937056514	up-regulated in High
NR2F6	0.22224354	4.50542539	3.61030029	0.00033697	0.00182796	-1.7485924	3.47241532	up-regulated in Low
USHBP1	-0.1931248	0.57740427	-6.4133486	3.33E-10	5.83E-09	11.5912893	9.478145759	up-regulated in High
ANKLE1	0.14848066	0.42525182	4.05449705	5.83E-05	0.00038435	-0.0933603	4.234059959	up-regulated in Low
MRPL34	0.12932595	4.06254539	2.45348336	0.01449129	0.0475686	-5.1941268	1.838893077	up-regulated in Low
DDA1	0.15073263	3.31768404	3.64563851	0.00029492	0.0016274	-1.6235485	3.530294725	up-regulated in Low
GTPBP3	0.20102167	2.23152151	4.35131388	1.64E-05	0.00012203	1.11263336	4.784036679	up-regulated in Low
BST2	-0.2809193	6.75835145	-2.6138888	0.00922414	0.03252035	-4.7924102	2.03507437	up-regulated in High
SLC27A1	-0.5856261	2.17652425	-11.21497	3.62E-26	4.39E-24	47.9195328	25.4417417	up-regulated in High
FAM129C	-0.1755041	0.31828574	-4.872891	1.48E-06	1.36E-05	3.42174011	5.828976617	up-regulated in High
COLGALT1	0.30698857	4.66807314	5.38136346	1.14E-07	1.30E-06	5.90015485	6.942174639	up-regulated in Low
UNC13A	0.17135393	0.26351076	3.18570363	0.00153504	0.00695642	-3.1601535	2.8138796	up-regulated in Low
MAP1S	-0.1151379	3.40872896	-2.0828868	0.03777369	0.10436248	-6.0270581	1.4228106	up-regulated in High
JAK3	-0.1616523	2.31050138	-2.3929123	0.01708653	0.05449153	-5.3393652	1.767346261	up-regulated in High
SLC5A5	0.23390941	0.59777198	2.3748353	0.01793649	0.05678069	-5.3820231	1.746262433	up-regulated in Low
CCDC124	0.25682692	4.86606652	4.70182725	3.35E-06	2.85E-05	2.63804769	5.475330913	up-regulated in Low
ARRDC2	-0.2790332	3.4063542	-4.7872233	2.24E-06	1.98E-05	3.02608091	5.650547459	up-regulated in High
IL12RB1	-0.1203029	1.25673566	-2.1685806	0.03058953	0.08806859	-5.846316	1.514427151	up-regulated in High
MAST3	-0.23341	1.94191171	-5.2895546	1.84E-07	2.02E-06	5.43636039	6.734378771	up-regulated in High
MPV17L2	0.16137184	2.74263898	3.82444719	0.00014779	0.00088374	-0.973181	3.830365098	up-regulated in Low
PDE4C	-0.3138518	0.84491507	-5.2582132	2.17E-07	2.34E-06	5.27966275	6.664123404	up-regulated in High
KIAA1683	-0.2365796	0.6858311	-4.6036355	5.28E-06	4.33E-05	2.19977424	5.27713927	up-regulated in High
JUND	-0.2914585	6.05087291	-4.1491546	3.93E-05	0.00026851	0.28262162	4.405901707	up-regulated in High
LSM4	0.44626621	4.80230706	9.35610985	2.86E-19	1.71E-17	32.1637369	18.54312389	up-regulated in Low
PGPEP1	-0.3589788	2.50697429	-7.7303226	6.02E-14	1.81E-12	20.0550907	13.22029586	up-regulated in High
GDF15	-0.7448277	4.71077563	-4.9864589	8.52E-07	8.22E-06	3.95607184	6.069598184	up-regulated in High
LRRC25	-0.2861513	2.24676297	-3.9408891	9.28E-05	0.00058336	-0.5338825	4.032225514	up-regulated in High
SSBP4	-0.1246405	3.70126613	-2.0946427	0.03670996	0.10195911	-6.0026864	1.435216086	up-regulated in High
ELL	-0.1063537	2.45528536	-2.8254685	0.00491168	0.0190209	-4.2247473	2.308770139	up-regulated in High
CRLF1	-0.7509061	3.32701525	-3.2657116	0.00116748	0.00545676	-2.9070647	2.932749769	up-regulated in High
TMEM59L	-0.7309348	1.9378561	-4.2504409	2.55E-05	0.00018146	0.69388826	4.593464001	up-regulated in High
KLHL26	-0.1939989	1.92416785	-4.8626823	1.56E-06	1.43E-05	3.37425604	5.807574698	up-regulated in High
CRTC1	-0.2450916	2.01736277	-4.6352565	4.56E-06	3.79E-05	2.33998722	5.340579696	up-regulated in High
COMP	-1.0153163	3.08465952	-6.4470463	2.71E-10	4.83E-09	11.7915251	9.567005228	up-regulated in High
CERS1	0.10305106	0.16249851	4.82224287	1.89E-06	1.70E-05	3.18704823	5.723165652	up-regulated in Low
COPE	0.20095596	5.35869674	3.95442086	8.79E-05	0.00055519	-0.4820282	4.056013007	up-regulated in Low
DDX49	0.25349777	3.8520987	5.69281849	2.14E-08	2.77E-07	7.52613413	7.669103847	up-regulated in Low
HOMER3	0.17211935	2.95264789	2.46468296	0.0140516	0.04636775	-5.166884	1.852274122	up-regulated in Low
ARMC6	0.19656813	3.02832779	4.59007005	5.62E-06	4.58E-05	2.13989255	5.250035095	up-regulated in Low
SLC25A42	-0.4024429	1.91782954	-8.2733775	1.21E-15	4.58E-14	23.9073635	14.91663034	up-regulated in High
TMEM161A	0.13711503	3.22261886	2.81282033	0.00510586	0.01968282	-4.2598854	2.29193097	up-regulated in Low
MEF2BNB	-0.1093237	1.8865721	-2.945201	0.0033792	0.01378187	-3.8845651	2.471186284	up-regulated in High
NR2C2AP	0.2480656	3.48592376	5.01645354	7.35E-07	7.18E-06	4.0990549	6.133923298	up-regulated in Low
MAU2	-0.1565064	2.6173363	-4.0220344	6.67E-05	0.00043341	-0.2204335	4.175895587	up-regulated in High
GATAD2A	0.1104673	3.63525846	2.52950876	0.01173115	0.03985259	-5.0068194	1.930659514	up-regulated in Low
CILP2	-0.4231622	1.6401976	-4.3474483	1.67E-05	0.00012392	1.09641892	4.776664519	up-regulated in High
PBX4	-0.1575991	0.98136722	-3.550455	0.00042124	0.00222579	-1.9577171	3.375470516	up-regulated in High
GMIP	-0.1987928	3.23540364	-4.1124935	4.58E-05	0.00030949	0.13604122	4.338951368	up-regulated in High
ZNF14	-0.2167134	1.89729048	-4.9622442	9.60E-07	9.18E-06	3.84120784	6.017904295	up-regulated in High
ZNF506	-0.17259	1.42115981	-4.0825387	5.19E-05	0.00034629	0.01717653	4.284619247	up-regulated in High

ZNF253	-0.1787124	1.79140566	-3.6789621	0.00025983	0.00145594	-1.5045763	3.585305363	up-regulated in High
ZNF93	0.17494628	1.0371567	3.77727134	0.00017782	0.0010405	-1.1476264	3.750029288	up-regulated in Low
ZNF682	-0.2640337	1.00896683	-4.2222464	2.88E-05	0.0002025	0.57847904	4.54087126	up-regulated in High
ZNF90	-0.21538	0.90357149	-2.9671149	0.00315136	0.01296129	-3.8208273	2.501501902	up-regulated in High
ZNF486	-0.460328	2.06771273	-3.7458806	0.00020089	0.00116065	-1.2625693	3.69703713	up-regulated in High
ZNF737	-0.3826188	1.42490883	-5.8405717	9.43E-09	1.29E-07	8.32555943	8.025713741	up-regulated in High
ZNF626	-0.2450492	1.15960413	-3.5157105	0.00047884	0.00249382	-2.077605	3.319807196	up-regulated in High
ZNF430	-0.1198351	1.31385775	-3.1355121	0.00181741	0.00804251	-3.3158481	2.740548208	up-regulated in High
ZNF714	0.14327003	1.14884353	2.43801241	0.01511866	0.04928991	-5.2315603	1.82048679	up-regulated in Low
ZNF431	-0.2613313	1.42627559	-5.0308335	6.84E-07	6.72E-06	4.16787794	6.164876181	up-regulated in High
ZNF708	-0.1722353	1.33458249	-4.1072935	4.68E-05	0.00031568	0.1153485	4.329495553	up-regulated in High
ZNF738	0.16889297	1.41638544	2.68861206	0.00741662	0.02697527	-4.596827	2.129794119	up-regulated in Low
ZNF493	-0.2784695	0.89586818	-6.4305541	3.00E-10	5.29E-09	11.6934156	9.523469274	up-regulated in High
ZNF429	-0.263528	1.37408508	-6.2521145	8.75E-10	1.43E-08	10.6455571	9.058173356	up-regulated in High
ZNF100	-0.1163524	1.31705628	-2.5532104	0.01097195	0.03766355	-4.9472856	1.959716183	up-regulated in High
ZNF257	0.10030796	0.4120405	2.34441694	0.01945078	0.06069221	-5.4530905	1.711063005	up-regulated in Low
ZNF724P	0.25318714	0.49993095	7.46542103	3.76E-13	1.00E-11	18.2503327	12.4243373	up-regulated in Low
ZNF91	-0.1610281	1.506133	-3.5485718	0.00042419	0.00223913	-1.9642439	3.372441838	up-regulated in High
ZNF254	-0.268149	1.96519709	-4.8175774	1.93E-06	1.74E-05	3.16554175	5.713465496	up-regulated in High
UQCRFS1	0.32841074	3.93055558	6.54896764	1.45E-10	2.70E-09	12.4025388	9.838035988	up-regulated in Low
POP4	0.17856554	2.50280568	4.07455856	5.37E-05	0.000357	-0.0143528	4.270201169	up-regulated in Low
CCNE1	0.92167583	1.87843699	11.3303452	1.27E-26	1.60E-24	48.955778	25.89476805	up-regulated in Low
URI1	0.10083558	3.73156256	2.17217774	0.03031565	0.08743527	-5.8385727	1.518333171	up-regulated in Low
TSHZ3	-0.1553144	1.14975214	-3.002741	0.00281076	0.01173904	-3.7162338	2.551176318	up-regulated in High
DPY19L3	-0.1657983	1.98302521	-4.0271183	6.53E-05	0.00042487	-0.2005961	4.184978589	up-regulated in High
PDCD5	0.46134267	4.39531477	8.5504971	1.53E-16	6.56E-15	25.949502	15.81464154	up-regulated in Low
ANKRD27	0.16371083	2.59662364	3.50612161	0.00049599	0.00257029	-2.1104945	3.30452534	up-regulated in Low
NUDT19	0.33731785	3.30231395	6.17344361	1.39E-09	2.21E-08	10.1915668	8.856395609	up-regulated in Low
TDRD12	0.13815984	0.16011854	3.38135713	0.0007783	0.00381515	-2.5306275	3.108856034	up-regulated in Low
SLC7A9	0.17094367	0.37859544	2.90670749	0.00381614	0.01531885	-3.9954206	2.418376201	up-regulated in Low
CEP89	0.19151809	2.01845142	4.68779952	3.57E-06	3.03E-05	2.57491715	5.446802053	up-regulated in Low
C19orf40	0.48059835	1.59312951	9.81106974	6.96E-21	5.07E-19	35.8460085	20.15749531	up-regulated in Low
RHPN2	0.51955241	2.89568955	6.01052801	3.59E-09	5.33E-08	9.26713355	8.445150278	up-regulated in Low
GPATCH1	0.12498386	1.96276452	3.12290676	0.00189548	0.00833995	-3.3545769	2.722281567	up-regulated in Low
SLC7A10	-0.4095984	0.65643678	-4.7648177	2.49E-06	2.18E-05	2.92365473	5.604319274	up-regulated in High
CEBPA	-0.7686488	2.96001032	-7.5129389	2.72E-13	7.42E-12	18.5703958	12.56556089	up-regulated in High
CEBPG	0.43457036	3.46392772	8.31399076	8.98E-16	3.46E-14	24.2034839	15.04689514	up-regulated in Low
LSM14A	0.1819839	4.74598788	4.09215448	4.99E-05	0.00033402	0.05524473	4.302023979	up-regulated in Low
KIAA0355	-0.1700361	2.43660947	-3.8877912	0.00011494	0.0007055	-0.7357415	3.939546609	up-regulated in High
GPI	0.58561017	5.32737031	10.9847488	2.85E-25	3.25E-23	45.8707709	24.54585558	up-regulated in Low
PDCD2L	0.36375702	2.76140535	6.53265084	1.61E-10	2.96E-09	12.3041777	9.794417481	up-regulated in Low
UBA2	0.42125607	4.53063192	8.1127633	3.93E-15	1.38E-13	22.7470937	14.40605236	up-regulated in Low
WTIP	-0.3536089	1.05530449	-6.743094	4.33E-11	8.72E-10	13.5885206	10.36361839	up-regulated in High
ZNF302	-0.1965936	2.4784335	-4.0565587	5.78E-05	0.00038173	-0.0852577	4.237767201	up-regulated in High
ZNF181	-0.112515	1.23616715	-3.6572384	0.00028223	0.00156522	-1.5822507	3.549396454	up-regulated in High
SCN1B	-0.1409819	1.55303828	-2.0077134	0.04521609	0.12053287	-6.1797141	1.344707046	up-regulated in High
HPN	-0.466414	3.79709643	-3.6680643	0.00027085	0.00151	-1.5435966	3.567269219	up-regulated in High
LGI4	-0.1259533	0.51856643	-4.781015	2.30E-06	2.03E-05	2.9976561	5.637720021	up-regulated in High
FXYD1	-0.2680626	0.4078766	-9.2145554	8.88E-19	5.02E-17	31.042887	18.05139948	up-regulated in High
FXYD5	-0.3684483	4.97941701	-4.053266	5.86E-05	0.00038588	-0.0981964	4.231847147	up-regulated in High
CD22	-0.5822461	1.1826206	-8.149725	3.00E-15	1.08E-13	23.0125672	14.52289873	up-regulated in High
FFAR2	0.17755985	0.82492341	2.11268667	0.0351271	0.09835824	-5.9650168	1.454357697	up-regulated in Low
DMKN	-0.2470204	2.5303555	-2.4012021	0.01670874	0.05351765	-5.3196971	1.777056305	up-regulated in High
SBSN	0.20057737	0.39862741	2.49875184	0.01278584	0.04289151	-5.083268	1.893270782	up-regulated in Low
TMEM147	0.29782147	5.36886954	5.38025586	1.15E-07	1.31E-06	5.89451721	6.939650058	up-regulated in Low
HAUS5	0.2463614	2.3489183	4.21628834	2.95E-05	0.00020726	0.55418219	4.52979505	up-regulated in Low
RBM42	0.34235769	5.08303196	6.22858277	1.01E-09	1.63E-08	10.5092461	8.997601664	up-regulated in Low
COX6B1	0.3577053	7.19932635	5.96946996	4.54E-09	6.61E-08	9.03752387	8.342922531	up-regulated in Low
KMT2B	0.1166007	2.96624083	2.09211029	0.03693692	0.10246274	-6.0079479	1.432539345	up-regulated in Low
PSENEN	0.23885669	4.87128876	3.85449107	0.00013123	0.00079423	-0.861022	3.88196207	up-regulated in Low
HSPB6	-0.621894	2.02455655	-6.5973614	1.08E-10	2.04E-09	12.6954782	9.967914191	up-regulated in High
PROSER3	0.13748943	1.63422801	2.93363124	0.00350547	0.0142342	-3.9180323	2.455254353	up-regulated in Low
ARHGAP33	0.16237972	1.44046016	2.2790706	0.02308731	0.06993508	-5.6027298	1.636626707	up-regulated in Low
NFKBID	-0.2362487	1.55469111	-4.3577491	1.60E-05	0.00011896	1.13965634	4.796321883	up-regulated in High
HCST	-0.326537	3.19012979	-3.892274	0.00011289	0.00069443	-0.7187989	3.947330291	up-regulated in High
TYROBP	-0.5132099	6.12563505	-5.0668865	5.72E-07	5.71E-06	4.34121122	6.242806227	up-regulated in High
CLIP3	-0.246178	2.26282554	-3.4918075	0.00052266	0.00268823	-2.1594324	3.281777462	up-regulated in High
WDR62	0.55385353	1.13588171	9.53064799	6.98E-20	4.50E-18	33.562154	19.15639881	up-regulated in Low
OVOL3	0.10906764	0.29165992	3.40235519	0.00072215	0.00357368	-2.4609345	3.141375388	up-regulated in Low
POLR2I	0.2044633	4.17898295	3.64727096	0.0002931	0.00161875	-1.6177443	3.532979826	up-regulated in Low
TBCB	0.22945201	4.1911719	4.3211575	1.88E-05	0.00013748	0.98649317	4.726670206	up-regulated in Low

COX7A1	-0.4872901	2.43455128	-7.8722893	2.21E-14	6.99E-13	21.0426213	13.65547349	up-regulated in High
ZNF146	0.30735673	4.58599369	5.5860265	3.84E-08	4.76E-07	6.95952449	7.416047424	up-regulated in Low
ZFP14	-0.1234173	1.12589903	-3.9351386	9.50E-05	0.00059578	-0.5558679	4.022137486	up-regulated in High
ZNF850	0.15933686	0.54129712	4.68613488	3.60E-06	3.05E-05	2.56743705	5.443421352	up-regulated in Low
ZNF790	-0.1315431	1.21569434	-3.657106	0.00028237	0.00156585	-1.5827229	3.5491781	up-regulated in High
ZNF345	-0.1387861	0.97509419	-4.0637561	5.61E-05	0.00037147	-0.0569411	4.250721674	up-regulated in High
ZNF540	-0.3451634	0.55709121	-11.592796	1.16E-27	1.61E-25	51.3360276	26.93511742	up-regulated in High
DPF1	0.1137804	0.20536272	3.79776793	0.00016412	0.00096902	-1.0720856	3.78483032	up-regulated in Low
PPP1R14A	-0.2243577	2.42068489	-2.8020028	0.00527743	0.02025062	-4.2898166	2.277577265	up-regulated in High
YIF1B	0.29195326	3.61313857	5.83994683	9.46E-09	1.30E-07	8.32214074	8.024189728	up-regulated in Low
PSMD8	0.2699022	5.55197443	5.83014294	9.99E-09	1.37E-07	8.26854757	8.000297377	up-regulated in Low
SPRED3	0.1162648	0.6339116	2.7937498	0.00541182	0.02070372	-4.3125768	2.266656305	up-regulated in Low
FAM98C	-0.1005204	2.03257766	-2.4363849	0.01518603	0.04946537	-5.2354848	1.818555755	up-regulated in High
RASGRP4	-0.3317148	0.74628888	-9.7090497	1.62E-20	1.12E-18	35.0099	19.79106548	up-regulated in High
RYR1	-0.2171461	0.85061668	-3.2166697	0.00138166	0.00633857	-3.0629126	2.859598849	up-regulated in High
MAP4K1	-0.3143634	2.01235438	-4.2174039	2.94E-05	0.00020639	0.55872922	4.531868009	up-regulated in High
EIF3K	0.12210071	5.60003732	2.54693118	0.0111687	0.03822655	-4.9631104	1.951997548	up-regulated in Low
CAPN12	-0.2954111	1.4546272	-4.0696308	5.48E-05	0.00036339	-0.0337934	4.261309793	up-regulated in High
LGALS7B	0.21308421	0.66096496	2.20812717	0.02769256	0.08118117	-5.760495	1.557636924	up-regulated in Low
HNRNPL	0.24658517	4.9995527	9.81346948	6.82E-21	4.99E-19	35.8657466	20.16614479	up-regulated in Low
RINL	-0.1256609	2.09110348	-2.1811539	0.02964139	0.0858298	-5.8191953	1.528101391	up-regulated in High
SIRT2	-0.2072662	3.15922101	-4.8137748	1.97E-06	1.76E-05	3.14802668	5.705565107	up-regulated in High
NFKBIB	0.19666356	3.58012649	3.79008181	0.00016914	0.00099576	-1.1004582	3.771761615	up-regulated in Low
SARS2	0.23673035	1.52229214	5.20929183	2.79E-07	2.95E-06	5.03673627	6.555155974	up-regulated in Low
MRPS12	0.52063334	3.96898542	8.91785597	9.17E-18	4.53E-16	28.7330926	17.03754033	up-regulated in Low
FBXO17	0.2270358	1.20821764	3.36325615	0.00082993	0.00403407	-2.5903744	3.08095728	up-regulated in Low
PAPL	0.15321324	0.13973151	5.26981863	2.04E-07	2.22E-06	5.33758933	6.690097762	up-regulated in Low
PAK4	0.27519287	3.39885207	5.21197322	2.75E-07	2.92E-06	5.04999841	6.561106498	up-regulated in Low
GMFG	-0.5129341	3.81604319	-6.7190022	5.04E-11	1.00E-09	13.4397646	10.2977291	up-regulated in High
SAMD4B	0.19679424	3.90844132	4.03881486	6.22E-05	0.00040646	-0.1548667	4.205912641	up-regulated in Low
PAF1	0.11257322	4.72389423	2.68841547	0.00742092	0.02698925	-4.5973486	2.129542145	up-regulated in Low
MED29	-0.1168432	4.29370761	-2.8314129	0.00482275	0.01871798	-4.2081803	2.316705216	up-regulated in High
ZFP36	-0.6342142	6.57017824	-6.0182581	3.43E-09	5.12E-08	9.310515	8.464460908	up-regulated in High
PLEKHG2	0.32647409	2.78081958	3.78960516	0.00016945	0.00099746	-1.102216	3.770951905	up-regulated in Low
RPS16	0.27529575	8.14363137	3.91205207	0.00010429	0.00064757	-0.6438297	3.981761493	up-regulated in Low
SUPT5H	0.11540134	4.44663093	2.4131116	0.01617888	0.05212073	-5.2913249	1.791051615	up-regulated in Low
TIMM50	0.35039033	3.2669253	7.20682287	2.15E-12	5.20E-11	16.5370993	11.66787594	up-regulated in Low
DLL3	0.48870261	0.70369738	4.79148372	2.19E-06	1.94E-05	3.04560681	5.659358336	up-regulated in Low
FBL	0.43135008	5.59013235	8.93751582	7.87E-18	3.94E-16	28.8844636	17.10400641	up-regulated in Low
FCGBP	-1.3710245	2.17296757	-11.392883	7.22E-27	9.27E-25	49.5200747	26.14143933	up-regulated in High
PSMC4	0.39990611	5.33606761	8.89143838	1.13E-17	5.48E-16	28.5300701	16.94838905	up-regulated in Low
ZNF546	-0.1241536	0.57921082	-5.4108375	9.78E-08	1.13E-06	6.05055602	7.009514194	up-regulated in High
ZNF780B	-0.1278948	1.17618157	-3.4553249	0.00059683	0.0030202	-2.2832986	3.224149976	up-regulated in High
MAP3K10	0.19621219	1.98698481	3.77502152	0.00017938	0.00104873	-1.1558946	3.746218943	up-regulated in Low
CNTD2	0.29681247	1.23580938	3.08346307	0.00216017	0.00934402	-3.4747944	2.665512792	up-regulated in Low
AKT2	0.14250381	3.01115965	3.52984591	0.00045457	0.00238154	-2.0289652	3.342398186	up-regulated in Low
C19orf47	0.25150751	2.12111658	6.45018497	2.66E-10	4.74E-09	11.8102204	9.575300701	up-regulated in Low
PLD3	-0.407866	6.33629698	-5.5483617	4.70E-08	5.75E-07	6.76194003	7.32774053	up-regulated in High
PRX	-0.56631	1.48526012	-8.8420993	1.65E-17	7.89E-16	28.1520632	16.78238186	up-regulated in High
SERTAD1	-0.1737689	3.61162716	-2.7411826	0.00634304	0.02370853	-4.4560195	2.19770271	up-regulated in High
SHKBP1	0.20707433	4.42592587	4.05386798	5.85E-05	0.00038509	-0.0958318	4.232929103	up-regulated in Low
LTBP4	-0.5927128	3.51414675	-7.19397	2.34E-12	5.64E-11	16.4532197	11.63081598	up-regulated in High
NUMBL	0.20552076	2.26832153	3.57348536	0.00038671	0.00206292	-1.8776323	3.412617994	up-regulated in Low
C19orf54	0.13845116	2.55489649	3.09077315	0.00210868	0.00915078	-3.4526255	2.675989132	up-regulated in Low
SNRPA	0.40628954	4.63050453	11.3458333	1.11E-26	1.40E-24	49.0953611	25.95578589	up-regulated in Low
MIA	-0.2292559	0.3665273	-2.8065659	0.00520443	0.02001392	-4.2772046	2.283626528	up-regulated in High
RAB4B	-0.1721602	2.2523316	-3.7809749	0.00017527	0.00102732	-1.1340053	3.7563059	up-regulated in High
EGLN2	-0.2031304	2.31163256	-4.5220987	7.67E-06	6.09E-05	1.84229485	5.11524003	up-regulated in High
CYP2B6	0.13332855	0.14396565	2.70842292	0.0069942	0.02571926	-4.5440754	2.15526185	up-regulated in Low
CYP2A13	-0.113757	0.15268891	-3.3129113	0.0009908	0.00472398	-2.7549385	3.004013986	up-regulated in High
CYP2F1	-0.2391051	0.32333304	-4.3106224	1.96E-05	0.00014334	0.94261782	4.706708382	up-regulated in High
CYP2S1	-0.6699096	2.81427342	-5.8547302	8.70E-09	1.20E-07	8.4031024	8.060279318	up-regulated in High
AXL	-0.408694	3.41527213	-4.9541402	9.99E-07	9.52E-06	3.80287858	6.00065062	up-regulated in High
HNRNPUL1	0.1450374	5.49294809	3.34032312	0.00089993	0.00433464	-2.6656306	3.04578912	up-regulated in Low
TGFB1	-0.471847	4.5310544	-6.7146564	5.18E-11	1.03E-09	13.4129784	10.28586355	up-regulated in High
TMEM91	-0.471555	1.97005505	-7.199374	2.26E-12	5.45E-11	16.488472	11.64639155	up-regulated in High
B9D2	-0.141921	2.48317982	-2.8070428	0.00519686	0.01999136	-4.2758854	2.284259225	up-regulated in High
EXOSC5	0.42227948	3.83527234	7.20909666	2.12E-12	5.12E-11	16.5519509	11.6744375	up-regulated in Low
BCKDHA	-0.1298454	1.46610075	-3.5175469	0.00047562	0.00247882	-2.0712966	3.322737774	up-regulated in High
B3GNT8	-1.1745162	3.08333275	-11.487242	3.05E-27	4.06E-25	50.3749275	26.51508454	up-regulated in High
ATP5SL	0.13914139	3.39213442	3.62181954	0.00032268	0.00176093	-1.7079586	3.491230635	up-regulated in Low

CEACAM21	-0.4048617	1.20423936	-6.9730628	9.96E-12	2.20E-10	15.0306359	11.0019184	up-regulated in High
CEACAM4	-0.4532185	0.95943612	-6.9673335	1.03E-11	2.27E-10	14.9942241	10.98581197	up-regulated in High
CEACAM6	-1.5758557	8.29817141	-8.4497235	3.27E-16	1.34E-14	25.2010555	15.48560854	up-regulated in High
RPS19	0.29764412	7.51279578	4.44010507	1.11E-05	8.52E-05	1.48874796	4.95489101	up-regulated in Low
CD79A	-0.6413525	3.79466434	-4.4004177	1.32E-05	1.00E-04	1.31976616	4.878164697	up-regulated in High
ARHGEF1	-0.2866877	3.92436497	-4.5626249	6.38E-06	5.14E-05	2.01923845	5.195404587	up-regulated in High
GRIK5	0.12846245	0.41590497	2.11417496	0.03499919	0.09808476	-5.9618956	1.455941977	up-regulated in Low
ZNF574	0.14354871	2.38733852	3.50084348	0.00050568	0.00261236	-2.1285618	3.296128381	up-regulated in Low
POU2F2	-0.1866744	1.23379012	-3.1821269	0.00155374	0.00703023	-3.171327	2.808622302	up-regulated in High
ZNF526	0.10791671	2.20206626	3.21236374	0.00140211	0.00642026	-3.0764885	2.853219592	up-regulated in Low
GSK3A	0.27855493	4.05575205	6.84843092	2.22E-11	4.65E-10	14.2441177	10.65389659	up-regulated in Low
ERF	0.22215641	3.68174555	4.41413618	1.25E-05	9.47E-05	1.3780184	4.904620617	up-regulated in Low
PAFAH1B3	0.51675894	4.62651658	6.7942939	3.13E-11	6.44E-10	13.9061264	10.50426637	up-regulated in Low
PRR19	0.17268211	0.82455166	3.87715067	0.00011992	0.00073295	-0.7758831	3.921101231	up-regulated in Low
TMEM145	0.18079005	0.44948944	3.46202252	0.00058252	0.00295571	-2.2606516	3.234691761	up-regulated in Low
CXCL17	-1.6392622	7.05206895	-10.009558	1.33E-21	1.05E-19	37.4895213	20.87756295	up-regulated in High
CEACAM1	-0.2354085	3.24988069	-2.3881709	0.01730596	0.05510165	-5.3505844	1.761804303	up-regulated in High
CEACAM8	-0.2068638	0.19250201	-4.9684734	9.31E-07	8.93E-06	3.87070819	6.031182391	up-regulated in High
PSG4	0.11015263	0.06594865	2.54528989	0.01122064	0.03838407	-4.9672405	1.949982476	up-regulated in Low
TEX101	0.10380241	0.19455462	1.98128363	0.04811174	0.1266787	-6.232074	1.317748924	up-regulated in Low
LYPD3	1.01672998	2.21862959	7.73976716	5.64E-14	1.70E-12	20.1203509	13.24906169	up-regulated in Low
ETHE1	0.19343546	3.91889925	2.77557393	0.00571879	0.02169892	-4.3624729	2.242695931	up-regulated in Low
XRCC1	0.11411122	3.51622059	2.63552267	0.00866404	0.03081924	-4.7363361	2.062279782	up-regulated in Low
PINLYP	-0.1899633	0.60744297	-4.9386719	1.08E-06	1.02E-05	3.72987666	5.967783908	up-regulated in High
ZNF576	0.12062373	2.1650711	3.82668585	0.00014649	0.00087669	-0.9648522	3.834198076	up-regulated in Low
ZNF428	0.13286914	3.67366685	2.34706406	0.01931469	0.06034819	-5.4469416	1.714112304	up-regulated in Low
KCNN4	-0.5279597	3.61570517	-3.827907	0.00014578	0.00087284	-0.9603072	3.836289633	up-regulated in High
LYPD5	0.18298137	0.81373881	3.15065197	0.00172756	0.00769213	-3.2691343	2.762567299	up-regulated in Low
ZNF404	-0.1551436	0.94323747	-2.9320026	0.00352358	0.0143008	-3.9227331	2.453015768	up-regulated in High
ZNF224	-0.1459748	1.30565705	-4.1336808	4.19E-05	0.0002852	0.22060526	4.377582535	up-regulated in High
ZNF229	0.26761299	0.92423534	3.96573452	8.40E-05	0.0005327	-0.4385458	4.075953817	up-regulated in Low
PVR	0.44724758	3.96254804	7.50459989	2.88E-13	7.80E-12	18.5141102	12.5407277	up-regulated in Low
CEACAM19	0.21601445	1.3040122	3.48407801	0.00053762	0.00275574	-2.185779	3.269526091	up-regulated in Low
BCL3	0.17995365	4.60133908	2.31805382	0.02085265	0.06428998	-5.5139586	1.680838795	up-regulated in Low
CBLC	0.46566169	3.50956048	4.67304713	3.83E-06	3.22E-05	2.50871158	5.416876689	up-regulated in Low
BCAM	-0.9117494	5.69799905	-8.9403488	7.70E-18	3.86E-16	28.9062963	17.11359271	up-regulated in High
PVRL2	0.14468147	5.35859266	2.47878818	0.01351468	0.04491329	-5.1324011	1.869194278	up-regulated in Low
TOMM40	0.70790423	4.16892078	14.5225683	4.55E-40	1.35E-37	79.7670309	39.34204902	up-regulated in Low
APOC1	-0.3347989	5.8763829	-2.7056381	0.00705224	0.02589029	-4.5515134	2.151672841	up-regulated in High
CLPTM1	0.13780019	4.78782918	3.24545499	0.00125192	0.00580375	-2.9717111	2.902424454	up-regulated in Low
RELB	0.15003927	3.31835702	2.49667985	0.01285983	0.04311246	-5.0883853	1.890764905	up-regulated in Low
CLASRP	0.13684195	2.92721671	2.31612373	0.02095867	0.06456911	-5.5183884	1.678636339	up-regulated in Low
ZNF296	0.10344228	1.37603163	2.23224686	0.02604476	0.07724742	-5.7074057	1.584279623	up-regulated in Low
GEMIN7	0.3223357	3.22790895	6.72444261	4.87E-11	9.70E-10	13.4733179	10.31259188	up-regulated in Low
MARK4	0.17192547	2.65076485	4.21358165	2.99E-05	0.00020947	0.54315495	4.524767596	up-regulated in Low
TRAPPC6A	-0.2083133	4.46019629	-2.9320672	0.00352286	0.01429887	-3.9225466	2.45310455	up-regulated in High
ERCC2	0.15374363	2.43360495	3.61205275	0.00033475	0.00181766	-1.7424186	3.475274517	up-regulated in Low
CD3EAP	0.43027744	1.39096689	11.4118044	6.08E-27	7.87E-25	49.6911634	26.21622354	up-regulated in Low
FOSB	-0.9495829	2.85186851	-5.5563396	4.50E-08	5.53E-07	6.80369262	7.3464039	up-regulated in High
PPM1N	-0.1246194	0.79346331	-2.2726358	0.02347557	0.07089784	-5.6172412	1.629383879	up-regulated in High
VASP	0.18424961	5.17585054	3.61662357	0.00032905	0.00179135	-1.7263024	3.482737442	up-regulated in Low
GIPR	-0.4413772	1.0209081	-7.2338535	1.79E-12	4.39E-11	16.7139007	11.74598336	up-regulated in High
SNRPD2	0.43584554	6.24693475	7.64617448	1.08E-13	3.14E-12	19.4764072	12.96517215	up-regulated in Low
FBXO46	0.23660868	2.86199395	4.83423668	1.79E-06	1.61E-05	3.24242324	5.748138592	up-regulated in Low
DMWD	0.12599433	2.42592094	3.13942137	0.0017938	0.00794845	-3.3038069	2.746225395	up-regulated in Low
SYMPK	0.22027534	3.38904967	4.56585449	6.28E-06	5.07E-05	2.03340198	5.201818964	up-regulated in Low
IRF2BP1	0.11017256	3.49755627	2.12438247	0.03413263	0.09613613	-5.9404304	1.46683021	up-regulated in Low
MYPOP	0.1899223	2.30226724	4.6145389	5.02E-06	4.14E-05	2.24802235	5.298973263	up-regulated in Low
CCDC61	-0.1220301	2.09809505	-2.7260009	0.00663768	0.02461053	-4.4969546	2.177983878	up-regulated in High
IGFL2	-0.2559907	0.81217585	-3.2874996	0.00108258	0.00510445	-2.8371007	2.965541428	up-regulated in High
HIF3A	-0.1974539	1.14676832	-2.0286744	0.04302565	0.11584148	-6.1377029	1.366272602	up-regulated in High
PPP5C	0.2071905	3.23543637	5.49243747	6.35E-08	7.59E-07	6.47074867	7.197535922	up-regulated in Low
CCDC8	-0.3825322	1.70416742	-4.958941	9.75E-07	9.32E-06	3.82557786	6.010868791	up-regulated in High
PNMAL1	-0.3292608	1.74149895	-2.9060142	0.00382446	0.01535014	-3.9974042	2.417430265	up-regulated in High
PNMAL2	-0.2386977	0.51209978	-7.0686104	5.34E-12	1.23E-10	15.6414925	11.27205363	up-regulated in High
CALM3	0.20904943	5.90712969	4.77202849	2.40E-06	2.11E-05	2.95657071	5.61917702	up-regulated in Low
PTGIR	-0.321938	1.23662679	-7.3630492	7.54E-13	1.93E-11	17.5663103	12.12242169	up-regulated in High
GNG8	-0.1680704	0.5738907	-4.4094671	1.27E-05	9.65E-05	1.35817334	4.895608526	up-regulated in High
DACT3	-0.342292	1.05234326	-7.8247601	3.10E-14	9.61E-13	20.7104426	13.50911802	up-regulated in High
STRN4	0.27035308	3.65169947	5.93533063	5.52E-09	7.90E-08	8.84764362	8.258357058	up-regulated in Low
SLC1A5	0.29572416	5.46857923	5.01162762	7.52E-07	7.34E-06	4.07599768	6.123552079	up-regulated in Low

AP2S1	0.36167851	5.32111783	8.28286421	1.13E-15	4.30E-14	23.9764351	14.94701682	up-regulated in Low
NPAS1	0.11309881	0.55013246	3.26403959	0.00117425	0.00548486	-2.9124152	2.930240823	up-regulated in Low
TMEM160	0.15469423	2.83974285	2.52937997	0.0117354	0.03986471	-5.0071415	1.930502202	up-regulated in Low
SAE1	0.35717726	4.70481152	8.7472596	3.43E-17	1.58E-15	27.4297637	16.46511194	up-regulated in Low
INAFM1	-0.1628509	2.83567409	-3.0589977	0.002341	0.01001599	-3.5486216	2.630598203	up-regulated in High
C5AR1	-0.2916499	3.05916017	-3.4216408	0.00067392	0.00336138	-2.3965626	3.171389669	up-regulated in High
DHX34	0.28151576	2.50758153	5.62754236	3.06E-08	3.86E-07	7.17867525	7.513954534	up-regulated in Low
KPTN	0.29707065	2.28621875	5.6651722	2.49E-08	3.18E-07	7.3785458	7.603213698	up-regulated in Low
NAPA	-0.1440023	3.6524977	-3.6998803	0.00023986	0.00135754	-1.42937	3.62005091	up-regulated in High
EHD2	-0.6867597	5.1300355	-8.1785207	2.43E-15	8.87E-14	23.2200281	14.61420096	up-regulated in High
GLTSCR2	-0.2712	5.36462818	-4.8599032	1.58E-06	1.45E-05	3.36134514	5.801754975	up-regulated in High
SEPW1	-0.6154124	5.42007443	-9.0670315	2.85E-18	1.51E-16	29.8876609	17.54441944	up-regulated in High
PLA2G4C	-0.2407151	0.99685786	-5.3677131	1.23E-07	1.39E-06	5.8307468	6.911091013	up-regulated in High
LIG1	0.48623204	2.58755665	10.2623284	1.56E-22	1.35E-20	39.6139392	21.80793846	up-regulated in Low
ZNF114	0.21427976	0.78006287	2.59606226	0.00970979	0.03400872	-4.8382778	2.012790321	up-regulated in Low
CARD8	-0.2514434	1.76980294	-6.6119699	9.83E-11	1.88E-09	12.7842622	10.00726989	up-regulated in High
CCDC114	-0.3701058	0.96217069	-4.8078587	2.03E-06	1.81E-05	3.1208022	5.693284274	up-regulated in High
EMP3	-0.1764996	4.43474441	-2.1528369	0.0318135	0.09086658	-5.8800584	1.497388621	up-regulated in High
SYNGR4	0.18747259	0.37161269	3.90223127	0.00010848	0.00067045	-0.6811003	3.964646367	up-regulated in Low
KDELR1	0.1030126	6.76303826	2.58802742	0.00993606	0.03464537	-4.8588516	2.002785751	up-regulated in Low
GRIN2D	0.40914003	0.99965094	4.78117748	2.30E-06	2.03E-05	2.99839955	5.638055538	up-regulated in Low
GRWD1	0.24884108	2.96524248	7.11876804	3.85E-12	9.01E-11	15.9648789	11.41500906	up-regulated in Low
KCNJ14	0.12942897	0.50026694	4.36610284	1.54E-05	0.00011508	1.17479063	4.812292359	up-regulated in Low
SULT2B1	0.37968589	2.08661278	3.68723987	0.00025175	0.00141593	-1.4748639	3.59903522	up-regulated in Low
FAM83E	-0.4831327	3.70627229	-4.6638257	4.00E-06	3.35E-05	2.46742499	5.398211267	up-regulated in High
SPACA4	-0.2740715	0.98411073	-4.8641833	1.55E-06	1.42E-05	3.38123209	5.810719123	up-regulated in High
DBP	-0.2680437	1.60049346	-5.8621427	8.35E-09	1.16E-07	8.44376435	8.078403095	up-regulated in High
CA11	-0.2096442	2.75788219	-2.3401262	0.01967315	0.06125378	-5.4630429	1.706126004	up-regulated in High
MAMSTR	0.25755144	0.91489619	4.63822102	4.50E-06	3.74E-05	2.3531777	5.346546119	up-regulated in Low
BCAT2	-0.1829927	3.38936804	-3.2805565	0.00110899	0.00521006	-2.8594443	2.955072284	up-regulated in High
PLEKHA4	-0.4510689	2.64503861	-6.1167266	1.94E-09	3.02E-08	9.8673244	8.71221167	up-regulated in High
PPP1R15A	-0.2627617	4.70019139	-3.9931271	7.51E-05	0.00048258	-0.332784	4.124432375	up-regulated in High
NUCB1	-0.2119077	6.45686597	-4.7898033	2.21E-06	1.95E-05	3.03790341	5.655882319	up-regulated in High
DHDH	-0.3443362	1.47228928	-4.7087987	3.24E-06	2.77E-05	2.66948641	5.489535787	up-regulated in High
BAX	0.2084787	4.48137348	4.56178107	6.40E-06	5.16E-05	2.01553954	5.193729366	up-regulated in Low
RUVBL2	0.46481698	4.42081283	10.539798	1.43E-23	1.38E-21	41.9853288	22.84599814	up-regulated in Low
NTF4	-0.2826966	0.73798894	-5.8964072	6.88E-09	9.68E-08	8.63230713	8.162424921	up-regulated in High
PPFIA3	0.24627737	1.32911649	4.29339842	2.12E-05	0.00015336	0.8710998	4.674161024	up-regulated in Low
TRPM4	-0.3518858	3.02737668	-4.4233028	1.20E-05	9.12E-05	1.41703568	4.92233695	up-regulated in High
CD37	-0.7074029	3.0524058	-8.502701	2.20E-16	9.21E-15	25.5936954	15.65823385	up-regulated in High
GFY	0.26143523	0.19740789	5.39205871	1.08E-07	1.24E-06	5.95464653	6.966574864	up-regulated in Low
PIH1D1	0.21912445	4.05006821	5.69245017	2.15E-08	2.77E-07	7.52416374	7.668224287	up-regulated in Low
FLT3LG	-0.1322371	0.75530651	-4.4026184	1.31E-05	9.92E-05	1.32909964	4.882404065	up-regulated in High
FCGRT	-0.7414429	5.33858433	-12.246789	2.61E-30	4.30E-28	57.4000866	29.58415824	up-regulated in High
NOSIP	0.15442172	3.29771191	3.31294926	0.00099067	0.00472374	-2.7548152	3.004071679	up-regulated in Low
PRRG2	-0.1954374	2.81808508	-3.4065196	0.00071147	0.00352716	-2.4470637	3.14784465	up-regulated in High
RRAS	-0.325984	5.28466508	-4.7130089	3.18E-06	2.72E-05	2.68849334	5.498122881	up-regulated in High
SCAF1	0.20090349	4.2162305	3.71595697	0.00022549	0.00128602	-1.3712964	3.646866345	up-regulated in Low
BCL2L12	0.43096897	3.0883818	8.33935234	7.44E-16	2.91E-14	24.388956	15.12847655	up-regulated in Low
PRMT1	0.44138112	4.55552301	10.6561201	5.17E-24	5.25E-22	42.9914172	23.28626597	up-regulated in Low
CPT1C	0.23501387	0.72772694	4.31229646	1.95E-05	0.00014244	0.94958323	4.709877692	up-regulated in Low
AP2A1	0.19850463	3.63271478	4.62483378	4.79E-06	3.96E-05	2.29367388	5.31962856	up-regulated in Low
PTOV1	0.14833272	4.1834799	3.1843312	0.00154219	0.00698611	-3.1644424	2.811861726	up-regulated in Low
PNKP	0.16682851	3.15255671	3.59594242	0.0003556	0.00191467	-1.7990674	3.449033475	up-regulated in Low
AKT1S1	0.24164124	3.22706232	5.69762922	2.09E-08	2.70E-07	7.55188034	7.680596366	up-regulated in Low
TBC1D17	-0.2312861	3.3317093	-5.2697577	2.04E-07	2.22E-06	5.33728488	6.689961256	up-regulated in High
NUP62	0.28708267	3.77512826	7.11768916	3.87E-12	9.07E-11	15.9579034	11.41192584	up-regulated in Low
ATF5	0.27484545	4.03763398	4.26404385	2.40E-05	0.00017215	0.74982518	4.618943745	up-regulated in Low
SIGLEC11	-0.1325253	0.36860885	-4.8512031	1.65E-06	1.50E-05	3.32097053	5.783554185	up-regulated in High
ZNF473	0.16959825	1.38446644	4.94883117	1.02E-06	9.75E-06	3.77779969	5.989360468	up-regulated in Low
MYH14	-0.2406882	4.33627363	-2.639908	0.00855429	0.03048643	-4.7249146	2.067816153	up-regulated in High
KCNC3	-0.4323841	1.58376183	-7.0766057	5.07E-12	1.17E-10	15.6929169	11.29478861	up-regulated in High
NR1H2	-0.1645337	4.56909513	-3.829859	0.00014467	0.00086667	-0.9530388	3.839634284	up-regulated in High
NAPSA	-2.8375825	7.68469205	-14.806627	2.46E-41	7.80E-39	82.6746697	40.60943353	up-regulated in High
POLD1	0.41145616	2.90296578	7.93391377	1.42E-14	4.65E-13	21.4756366	13.84621764	up-regulated in Low
SPIB	-0.4495517	0.94290654	-5.8966235	6.87E-09	9.67E-08	8.63350071	8.162956748	up-regulated in High
MYBPC2	-0.2371113	0.48413254	-5.3732716	1.19E-07	1.35E-06	5.85899119	6.923740513	up-regulated in High
LRRC4B	-0.1043989	0.60595085	-2.4043137	0.01656885	0.05314836	-5.3122976	1.780707644	up-regulated in High
CLEC11A	-0.2721613	2.96052556	-3.6911952	0.00024797	0.0013975	-1.4606443	3.605604744	up-regulated in High
C19orf48	0.83300274	3.78280188	14.1213213	2.70E-38	7.14E-36	75.6989705	37.56849637	up-regulated in Low
KLK1	0.14279132	0.4756951	2.15746496	0.0314494	0.0900513	-5.8701644	1.502387661	up-regulated in Low

KLK5	0.2273209	0.4378604	2.22632668	0.02644114	0.07816407	-5.7204889	1.577719892	up-regulated in Low
KLK6	0.77162781	0.99229082	5.24017984	2.38E-07	2.55E-06	5.18987884	6.623857174	up-regulated in Low
KLK8	0.51851893	0.62455738	4.784893	2.26E-06	1.99E-05	3.01540767	5.645731023	up-regulated in Low
KLK11	-0.4036038	2.1800099	-2.1598479	0.03126333	0.08964161	-5.865062	1.504964763	up-regulated in High
KLK12	0.70516035	1.26597421	3.47085777	0.00056413	0.00287339	-2.2307126	3.248624037	up-regulated in Low
KLK14	0.49395019	0.87822792	3.33440519	0.00091887	0.00441455	-2.6849708	3.036746224	up-regulated in Low
CTU1	0.21253753	1.12614529	5.90612644	6.51E-09	9.21E-08	8.68596184	8.186330989	up-regulated in Low
SIGLEC9	-0.2787507	1.49637707	-4.6303211	4.67E-06	3.87E-05	2.31804476	5.330653859	up-regulated in High
SIGLEC7	-0.2011933	1.2300223	-3.5981656	0.00035266	0.00190142	-1.7912644	3.452648844	up-regulated in High
CD33	-0.3153056	0.92824099	-7.3463445	8.44E-13	2.15E-11	17.4554118	12.07345983	up-regulated in High
NKG7	0.25391504	3.27884892	2.17871255	0.02982349	0.0862544	-5.8244734	1.525441584	up-regulated in Low
SIGLEC8	-0.2762194	0.73728378	-5.4769047	6.89E-08	8.19E-07	6.39033453	7.161565897	up-regulated in High
SIGLEC6	-0.2530275	0.43835616	-6.3303956	5.48E-10	9.28E-09	11.1021648	9.260998882	up-regulated in High
ZNF175	-0.2096716	0.97304152	-6.9464307	1.18E-11	2.58E-10	14.8615871	10.9271369	up-regulated in High
SIGLEC14	-0.2819163	1.56283116	-3.941642	9.26E-05	0.00058184	-0.5310017	4.033547221	up-regulated in High
FPR1	-0.2685912	2.36705632	-2.9772316	0.00305099	0.01260175	-3.7912485	2.515558874	up-regulated in High
FPR3	-0.3548286	3.48233664	-3.6776894	0.0002611	0.0014619	-1.5091387	3.583196829	up-regulated in High
ZNF577	-0.1521042	0.96319262	-3.1773915	0.00157881	0.00712106	-3.1861018	2.801669303	up-regulated in High
ZNF615	-0.1945448	1.49596308	-3.9545856	8.78E-05	0.00055488	-0.4813957	4.056303101	up-regulated in High
ZNF836	-0.2074385	1.17533705	-6.5229541	1.70E-10	3.13E-09	12.2458215	9.768537089	up-regulated in High
PPP2R1A	0.13166145	5.13738471	3.76814047	0.00018426	0.00107432	-1.1811542	3.734576839	up-regulated in Low
ZNF480	0.10379002	2.23100289	2.83751304	0.00473302	0.01843417	-4.1911444	2.324862071	up-regulated in Low
ZNF528	-0.1300256	1.50797171	-2.3928008	0.01709165	0.05450193	-5.339629	1.767215933	up-regulated in High
ZNF83	-0.2575475	2.2307591	-4.6352549	4.56E-06	3.79E-05	2.33998004	5.340576451	up-regulated in High
ZNF468	0.11020026	2.35526447	2.21417916	0.02727085	0.08020126	-5.7472273	1.564301396	up-regulated in Low
ZNF816	-0.162164	1.83431782	-3.7819423	0.0001746	0.00102383	-1.1304455	3.757946137	up-regulated in High
ZNF160	-0.228681	2.01236676	-5.3910321	1.09E-07	1.24E-06	5.94941201	6.96423108	up-regulated in High
ZNF415	-0.1434223	1.31110912	-2.733812	0.00648458	0.02414073	-4.4759207	2.188118441	up-regulated in High
ZNF525	0.14207257	1.2485592	3.91280517	0.00010397	0.00064596	-0.640968	3.983075442	up-regulated in Low
ZNF761	0.1264671	2.18125141	3.01973339	0.00266049	0.01119229	-3.6659223	2.575039104	up-regulated in Low
ZNF813	0.18041842	1.12641728	5.13651417	4.03E-07	4.13E-06	4.67911648	6.394623736	up-regulated in Low
ZNF331	-0.1309044	1.9744569	-2.1569923	0.03148642	0.09013963	-5.8711759	1.501876698	up-regulated in High
NLRP12	-0.1053091	0.45563334	-2.4529767	0.01451146	0.04761074	-5.1953564	1.838288852	up-regulated in High
MYADM	-0.2906493	5.10676592	-3.6255701	0.00031815	0.0017385	-1.694702	3.49736758	up-regulated in High
CACNG6	-0.2161815	0.54934268	-2.6908794	0.00736714	0.02683542	-4.5908087	2.132701313	up-regulated in High
OSCAR	-0.5160712	2.47265827	-5.9788656	4.30E-09	6.30E-08	9.08994698	8.366265589	up-regulated in High
NDUFA3	0.10621824	4.0323422	2.16062838	0.0312026	0.08951142	-5.8633896	1.505809277	up-regulated in Low
TFPT	0.16045182	3.58379003	3.05573602	0.00236613	0.01011432	-3.5584214	2.625960603	up-regulated in Low
PRPF31	0.17877321	4.19216101	4.52469136	7.58E-06	6.02E-05	1.85357139	5.120350578	up-regulated in Low
CNOT3	0.20328142	3.10902618	4.57749515	5.96E-06	4.83E-05	2.08452845	5.224970169	up-regulated in Low
TMC4	-0.5079044	5.04134981	-6.4348768	2.92E-10	5.16E-09	11.7191098	9.534871507	up-regulated in High
LILRB3	-0.1705679	1.10336936	-3.7683156	0.00018413	0.00107369	-1.180512	3.734872857	up-regulated in High
LILRA6	-0.1303594	0.90382402	-2.6224777	0.008998	0.03183459	-4.7702018	2.045854055	up-regulated in High
LILRB5	-0.2137425	0.92799168	-3.7198483	0.00022214	0.00126922	-1.357204	3.65337159	up-regulated in High
LILRA4	-0.1831388	0.53164703	-4.5089736	8.14E-06	6.42E-05	1.78530036	5.089406445	up-regulated in High
LAIR1	-0.372289	2.39588462	-4.8533805	1.63E-06	1.49E-05	3.33106873	5.788106646	up-regulated in High
LENG8	-0.2518259	4.35938098	-2.6953076	0.00727135	0.02654282	-4.5790405	2.138384848	up-regulated in High
KIR2DL1	0.12435124	0.13448738	2.578122	0.01022149	0.03545009	-4.8841297	1.99048572	up-regulated in Low
KIR2DL4	0.27055513	0.42874501	5.64836667	2.73E-08	3.46E-07	7.28913869	7.563289831	up-regulated in Low
EPS8L1	-0.2534475	2.87457826	-2.7620068	0.00595809	0.02246385	-4.3995114	2.224892809	up-regulated in High
TNNT1	0.83587497	2.40656622	4.87776108	1.45E-06	1.34E-05	3.44442443	5.839199737	up-regulated in Low
TNNI3	0.37287655	0.98135723	3.70537321	0.00023485	0.00133273	-1.4095549	3.629201951	up-regulated in Low
DNAAF3	-0.1599724	0.86685682	-2.5066736	0.01250644	0.04205329	-5.063665	1.902866393	up-regulated in High
SYT5	0.21268239	0.24857941	4.41410366	1.25E-05	9.47E-05	1.37788011	4.904557817	up-regulated in Low
PTPRH	0.38186635	1.80194119	3.38453294	0.00076955	0.00377926	-2.5201134	3.113763648	up-regulated in Low
PPP6R1	0.19025146	4.02055879	3.85598297	0.00013046	0.00079055	-0.8554309	3.884533069	up-regulated in Low
HSPBP1	0.34063622	4.23530883	6.42814464	3.04E-10	5.37E-09	11.6790998	9.517116248	up-regulated in Low
SUV420H2	0.1113056	1.71851435	2.0458541	0.04129807	0.11219386	-6.1029504	1.384070252	up-regulated in Low
COX6B2	0.10089004	0.25456944	2.75146663	0.00615021	0.02308431	-4.4281648	2.211109976	up-regulated in Low
IL11	0.27498509	0.60061157	5.03133469	6.82E-07	6.71E-06	4.17028003	6.165956405	up-regulated in Low
TMEM190	-0.6579936	1.13184056	-5.4202241	9.31E-08	1.08E-06	6.09860745	7.031023859	up-regulated in High
UBE2S	1.18079641	2.79258589	16.6555668	8.66E-50	4.51E-47	102.078075	49.06249571	up-regulated in Low
ISOC2	0.13656961	3.440872	2.73157367	0.00652812	0.02427444	-4.4819541	2.185211938	up-regulated in Low
SSC5D	-0.6400239	1.95478781	-8.9422976	7.58E-18	3.80E-16	28.9213173	17.1201881	up-regulated in High
SBK2	0.11994198	0.08484432	3.87551793	0.00012071	0.00073735	-0.7820335	3.918274629	up-regulated in Low
SBK3	0.14953886	0.32252532	4.67195428	3.85E-06	3.24E-05	2.50381469	5.414662982	up-regulated in Low
ZNF579	0.15687245	2.0958054	2.97281766	0.00309442	0.01275494	-3.8041656	2.509421062	up-regulated in Low
ZNF784	-0.1457031	2.19688523	-3.1467205	0.00175049	0.0077788	-3.2812856	2.756841107	up-regulated in High
ZNF580	-0.1147538	2.68451007	-2.3142272	0.02106331	0.06482648	-5.5227378	1.676473493	up-regulated in High
U2AF2	0.3487539	5.21275715	9.12245269	1.84E-18	1.01E-16	30.3200957	17.73421826	up-regulated in Low
EPN1	0.11177506	3.10600176	2.50519818	0.01255806	0.04220743	-5.0673207	1.901077395	up-regulated in Low

NLRP11	0.15986371	0.13461936	3.81285078	0.00015469	0.00092011	-1.0162511	3.810540135	up-regulated in Low
ZNF787	0.18179341	3.17982907	3.35971763	0.00084039	0.0040788	-2.6020184	3.075517901	up-regulated in Low
ZNF444	-0.2278999	2.65930884	-4.2929269	2.12E-05	0.00015364	0.86914582	4.673271622	up-regulated in High
ZNF582	-0.1340661	0.70594931	-4.4930048	8.75E-06	6.85E-05	1.71616291	5.058060796	up-regulated in High
ZNF471	-0.1507904	0.6976229	-3.7688077	0.00018378	0.00107185	-1.1787068	3.735704923	up-regulated in High
ZNF835	-0.1157939	0.43908656	-3.9002643	0.00010934	0.00067468	-0.6885543	3.961222873	up-regulated in High
PEG3	-0.124657	0.3620442	-3.2749368	0.00113081	0.00529933	-2.8774956	2.946612108	up-regulated in High
ZNF264	-0.2551958	1.79865968	-6.696522	5.80E-11	1.14E-09	13.3013592	10.23641599	up-regulated in High
ZNF304	-0.1178858	1.81302056	-2.9338879	0.00350262	0.01422363	-3.9172913	2.455607172	up-regulated in High
ZNF547	-0.1125433	0.64467821	-4.8637466	1.55E-06	1.42E-05	3.37920221	5.80980417	up-regulated in High
ZNF548	-0.1068488	1.77865254	-2.7638233	0.00592554	0.02235698	-4.3945624	2.227272463	up-regulated in High
ZNF17	-0.1127873	1.13174254	-4.3323373	1.79E-05	0.0001316	1.03316191	4.74789835	up-regulated in High
ZNF530	0.10497132	0.85510268	3.437304	0.00063699	0.00319819	-2.344026	3.195869997	up-regulated in Low
ZNF134	-0.1567812	2.13714787	-3.3856457	0.00076651	0.00376686	-2.516427	3.11548417	up-regulated in High
ZNF211	-0.3778713	2.08599728	-7.1834956	2.51E-12	6.02E-11	16.3849522	11.60065211	up-regulated in High
ZSCAN4	-0.213181	0.21496172	-6.3622957	4.53E-10	7.77E-09	11.2896209	9.344234712	up-regulated in High
ZNF154	-0.2511874	0.90136619	-5.319484	1.58E-07	1.75E-06	5.58677399	6.801792863	up-regulated in High
ZNF671	-0.4112008	1.44920596	-8.7410083	3.60E-17	1.64E-15	27.3823539	16.44428426	up-regulated in High
ZNF776	-0.1758568	1.94218238	-4.5962597	5.47E-06	4.47E-05	2.16719513	5.262393797	up-regulated in High
ZNF586	-0.1051005	1.74277331	-2.8709945	0.00426693	0.01687096	-4.0970081	2.369884107	up-regulated in High
ZNF552	-0.4757812	2.25720982	-8.5712821	1.31E-16	5.65E-15	26.1046927	15.88285432	up-regulated in High
ZNF814	-0.100341	0.83196013	-3.4228943	0.0006709	0.00334857	-2.3923664	3.17334547	up-regulated in High
ZNF417	-0.1337676	1.34557061	-3.9235621	9.96E-05	0.00062168	-0.6000364	4.001866379	up-regulated in High
C19orf18	-0.1684873	0.77820279	-3.6161097	0.00032969	0.00179398	-1.7281153	3.481898015	up-regulated in High
ZSCAN18	-0.1737185	1.89647938	-2.758272	0.00602554	0.02268444	-4.4096765	2.220004241	up-regulated in High
ZNF544	0.22901608	2.22438886	5.09549846	4.95E-07	5.01E-06	4.47956332	6.304982753	up-regulated in Low
RNF225	-0.1835722	0.45059527	-3.8890029	0.00011438	0.00070231	-0.7311637	3.941649796	up-regulated in High
ZNF584	-0.1080715	1.6649638	-3.0675435	0.00227631	0.00978409	-3.5228978	2.642768164	up-regulated in High
ZNF132	-0.2677021	1.11552454	-6.7872949	3.27E-11	6.70E-10	13.8625922	10.48499024	up-regulated in High
SLC27A5	0.13159051	0.68252049	4.31109184	1.96E-05	0.00014308	0.94457091	4.707597061	up-regulated in Low
TRIM28	0.44782947	6.07545306	9.62651326	3.19E-20	2.13E-18	34.3378239	19.49646853	up-regulated in Low
UBE2M	0.42485336	5.01537598	10.5498199	1.31E-23	1.27E-21	42.0717354	22.88381314	up-regulated in Low
MZF1	-0.1550093	1.8891666	-2.6326974	0.00873541	0.03104854	-4.7436847	2.058716762	up-regulated in High
SOX12	0.28066809	2.95602073	3.9773843	8.01E-05	0.00051148	-0.3936498	4.096537024	up-regulated in Low
TRIB3	0.75463906	3.33894395	9.79037522	8.26E-21	5.94E-19	35.6759279	20.08296247	up-regulated in Low
RBCK1	0.17522312	4.69352047	3.55192265	0.00041896	0.00221552	-1.9526283	3.377831806	up-regulated in Low
TBC1D20	-0.1275076	3.27668204	-3.5198952	0.00047153	0.00246024	-2.0632249	3.326487228	up-regulated in High
CSNK2A1	0.22483733	4.02418731	5.14346302	3.89E-07	4.00E-06	4.71306711	6.409870057	up-regulated in Low
SRXN1	0.59930976	1.11011297	7.71607073	6.65E-14	1.98E-12	19.9567308	13.17693807	up-regulated in Low
SLC52A3	-0.1614707	1.87963396	-2.2057535	0.02785949	0.08156407	-5.7656891	1.555026786	up-regulated in High
RSPO4	-0.2308112	0.72367284	-3.4555166	0.00059642	0.00301915	-2.2826511	3.224451391	up-regulated in High
SDCBP2	0.32279865	2.64419665	2.48057122	0.01344812	0.04473537	-5.1280284	1.871338523	up-regulated in Low
NSFL1C	0.12765455	3.71226204	3.26369457	0.00117565	0.00548964	-2.913519	2.929723212	up-regulated in Low
SIRPB2	-0.2036329	1.07296276	-4.1029726	4.77E-05	0.00032081	0.09817294	4.321646103	up-regulated in High
SIRPB1	-0.1609874	0.78319619	-3.2273955	0.00133193	0.00613451	-3.0290207	2.87551954	up-regulated in High
SIRPA	-0.2582165	3.45776398	-3.1792544	0.0015689	0.00708513	-3.1802921	2.804403509	up-regulated in High
STK35	0.20112388	3.05322397	4.14618025	3.98E-05	0.00027168	0.2706841	4.400451306	up-regulated in Low
TGM3	0.10357752	0.26991166	2.41782988	0.01597309	0.05160898	-5.2800465	1.796611076	up-regulated in Low
SNRPB	0.64774117	6.97656542	10.9825699	2.90E-25	3.31E-23	45.8515033	24.53742886	up-regulated in Low
TMC2	0.10157717	0.11458258	2.88691084	0.00406036	0.01616253	-4.0518832	2.391435901	up-regulated in Low
NOP56	0.55472518	4.17874959	11.252862	2.57E-26	3.15E-24	48.2591653	25.59022998	up-regulated in Low
IDH3B	0.24528967	4.57221273	5.70941451	1.96E-08	2.55E-07	7.61503382	7.708784436	up-regulated in Low
EBF4	-0.5555732	2.03372619	-7.3140752	1.05E-12	2.64E-11	17.2417586	11.979121	up-regulated in High
C20orf141	0.11089916	0.07608295	5.13703253	4.02E-07	4.12E-06	4.68164766	6.395760467	up-regulated in Low
PTPRA	-0.1459279	4.23893095	-3.4526216	0.0006027	0.00304726	-2.2924277	3.219899817	up-regulated in High
MRPS26	0.25485046	5.20497888	3.80168438	0.00016162	0.00095532	-1.0576076	3.791497983	up-regulated in Low
OXT	-0.1621148	0.37021285	-3.4472381	0.00061455	0.00310099	-2.3105872	3.211444275	up-regulated in High
LZTS3	-0.4121512	3.34553016	-5.1115733	4.57E-07	4.65E-06	4.55760058	6.340043294	up-regulated in High
ITPA	0.17471205	4.52587146	3.34686353	0.00087943	0.00425096	-2.644218	3.055798634	up-regulated in Low
C20orf194	-0.3688341	1.80770362	-8.3276942	8.11E-16	3.15E-14	24.3036458	15.090953	up-regulated in High
ADAM33	-0.1534908	0.48868207	-3.4201679	0.0006775	0.00337748	-2.4014911	3.169092425	up-regulated in High
SIGLEC1	-0.4262861	2.10638835	-4.8655737	1.54E-06	1.41E-05	3.38769546	5.813632403	up-regulated in High
HSPA12B	-0.3256759	1.4426151	-6.2348156	9.69E-10	1.57E-08	10.545308	9.013627272	up-regulated in High
C20orf27	0.31885905	3.62291547	5.96360151	4.70E-09	6.82E-08	9.00481683	8.328357765	up-regulated in Low
SPEF1	-0.3290451	0.82207699	-4.5957942	5.48E-06	4.48E-05	2.16514069	5.261463885	up-regulated in High
CDC25B	0.18576245	4.5178091	2.24215287	0.02539304	0.07568653	-5.6854381	1.59528528	up-regulated in Low
SMOX	0.58680596	2.3322338	7.55813933	1.99E-13	5.53E-12	18.8763471	12.70053085	up-regulated in Low
PRNP	-0.259701	5.04749815	-3.699173	0.00024051	0.0013607	-1.4319198	3.61887327	up-regulated in High
PRND	-0.1182953	0.30609418	-3.0615411	0.00232158	0.00994569	-3.540973	2.634217294	up-regulated in High
RASSF2	-0.5854069	2.69733049	-8.1814027	2.38E-15	8.71E-14	23.2408226	14.62335198	up-regulated in High
TMEM230	0.11585403	5.15721727	2.58777336	0.00994329	0.03466324	-4.8595011	2.002469799	up-regulated in Low

PCNA	0.84978115	5.83973507	14.4215128	1.28E-39	3.71E-37	78.7380571	38.8934885	up-regulated in Low
CDS2	-0.1675887	2.73857729	-4.0403832	6.18E-05	0.00040406	-0.1487258	4.20872338	up-regulated in High
GPCPD1	-0.2187051	3.56783008	-2.7473325	0.00622708	0.02333786	-4.4393743	2.205715568	up-regulated in High
CHGB	0.30559236	0.92648751	2.01213354	0.04474649	0.11959333	-6.1708907	1.349241009	up-regulated in Low
TRMT6	0.34988539	2.85707504	7.38587475	6.46E-13	1.67E-11	17.7181695	12.1894617	up-regulated in Low
MCM8	0.5416229	1.64524375	10.7587357	2.10E-24	2.20E-22	43.8846934	23.67710057	up-regulated in Low
CRLS1	-0.3368387	3.80852661	-5.382493	1.14E-07	1.29E-06	5.90590534	6.944749722	up-regulated in High
LRRN4	-1.0700525	2.27192406	-7.5092505	2.79E-13	7.59E-12	18.5454938	12.55457424	up-regulated in High
FERMT1	0.23738604	2.45663683	2.58438377	0.01004022	0.03493761	-4.868161	1.998256927	up-regulated in Low
BMP2	-0.4530623	2.53664016	-4.2619274	2.43E-05	0.00017356	0.74111137	4.614975	up-regulated in High
TMX4	-0.3909561	3.64191423	-6.5708529	1.27E-10	2.38E-09	12.5347913	9.89667664	up-regulated in High
PLCB1	0.23139509	1.12829118	3.94657763	9.07E-05	0.00057107	-0.5121041	4.042217073	up-regulated in Low
PLCB4	-0.2101416	0.97990699	-2.8308168	0.0048316	0.01874562	-4.2098434	2.315908791	up-regulated in High
LAMP5	-0.7771328	1.74445975	-9.7475794	1.18E-20	8.29E-19	35.324976	19.92915836	up-regulated in High
ANKEF1	0.10028052	1.8558247	2.21806016	0.02700335	0.07958253	-5.7387002	1.568582429	up-regulated in Low
SNAP25	0.2135627	0.65407401	2.91927494	0.00366813	0.01480716	-3.9593831	2.435555897	up-regulated in Low
MKKS	0.23344971	2.56534262	5.70853178	1.96E-08	2.56E-07	7.61029962	7.706671468	up-regulated in Low
BTBD3	0.1610162	2.54732133	2.67474802	0.00772574	0.02795679	-4.6335199	2.112059895	up-regulated in Low
SPTLC3	-0.561325	1.38553689	-7.8989045	1.83E-14	5.85E-13	21.2293159	13.73771825	up-regulated in High
ISM1	-0.8043182	1.8703852	-9.4466774	1.38E-19	8.60E-18	32.8871322	18.86039804	up-regulated in High
ESF1	0.33450364	2.77457576	6.78144846	3.40E-11	6.94E-10	13.8262555	10.46890041	up-regulated in Low
NDUFAF5	0.18213059	1.20699963	5.34792937	1.36E-07	1.53E-06	5.73043118	6.866157449	up-regulated in Low
MACROD2	-0.7102279	1.11651638	-7.8850875	2.02E-14	6.43E-13	21.1323345	13.69499594	up-regulated in High
FLRT3	-0.7781475	2.81493962	-7.0207764	7.30E-12	1.65E-10	15.3348283	11.13645637	up-regulated in High
KIF16B	-0.3100761	2.47448408	-6.1295571	1.80E-09	2.81E-08	9.94044952	8.744734365	up-regulated in High
SNRPB2	0.27221016	4.37137891	6.04686552	2.91E-09	4.39E-08	9.47147839	8.536100976	up-regulated in Low
PCSK2	-0.5162019	0.81311452	-3.1325645	0.00183539	0.00811103	-3.3249178	2.736271363	up-regulated in High
DSTN	-0.2077451	6.81215323	-3.582664	0.0003737	0.00200029	-1.8455779	3.427478644	up-regulated in High
RRBP1	-0.1916243	5.803083	-2.5887965	0.0099142	0.03458774	-4.856885	2.00374229	up-regulated in High
OVOL2	0.17008694	2.43598329	2.69735962	0.00722735	0.02641356	-4.5735807	2.141021127	up-regulated in Low
MGME1	0.33543776	3.30975972	7.23766871	1.75E-12	4.28E-11	16.738898	11.75702584	up-regulated in Low
PET117	0.25017453	2.38536198	5.80208862	1.17E-08	1.58E-07	8.11562193	7.932110254	up-regulated in Low
POLR3F	0.18984256	2.37719418	4.722049	3.04E-06	2.62E-05	2.72935733	5.516582788	up-regulated in Low
RBBP9	-0.1213103	3.23259348	-2.3235149	0.02055521	0.06350669	-5.5014053	1.68707812	up-regulated in High
SEC23B	0.11840489	4.5760426	2.22009515	0.02686399	0.07922558	-5.7342232	1.570829438	up-regulated in Low
LINC00493	0.11949705	4.50564802	2.29628256	0.02207615	0.06733324	-5.563717	1.656076671	up-regulated in Low
DTD1	0.20437919	3.24691021	4.44416144	1.09E-05	8.37E-05	1.50609824	4.962765714	up-regulated in Low
SLC24A3	-0.312979	1.7447143	-3.7537085	0.00019489	0.00112964	-1.2339911	3.71021698	up-regulated in High
RIN2	-0.2960567	3.78381691	-5.7803584	1.32E-08	1.77E-07	7.99761177	7.879479695	up-regulated in High
NAA20	0.33137862	4.65044901	6.36060935	4.57E-10	7.84E-09	11.2796916	9.339826267	up-regulated in Low
CRNKL1	0.12885004	3.11790686	3.17114341	0.00161247	0.00724489	-3.205564	2.792508217	up-regulated in Low
CFAP61	-0.1313484	0.22534576	-3.9667683	8.36E-05	0.00053081	-0.4345668	4.0777783	up-regulated in High
INSM1	0.3729176	0.30352402	3.92122908	0.00010051	0.00062725	-0.6089227	3.997787276	up-regulated in Low
RALGAPA2	-0.3863483	2.98703095	-5.6827099	2.26E-08	2.90E-07	7.47209668	7.644981071	up-regulated in High
KIZ	-0.1646831	1.75516569	-4.253228	2.52E-05	0.00017955	0.70533558	4.598678933	up-regulated in High
XRN2	0.14975811	4.93376254	2.98738589	0.0029532	0.01225069	-3.7614619	2.529707223	up-regulated in Low
FOXA2	-1.1641942	3.00216313	-8.5152418	2.00E-16	8.42E-15	25.6869087	15.6992113	up-regulated in High
THBD	-0.4552427	3.19199447	-5.1806235	3.22E-07	3.38E-06	4.89532494	6.491694814	up-regulated in High
CD93	-0.4797985	3.60912659	-6.0024347	3.76E-09	5.56E-08	9.2217656	8.424954089	up-regulated in High
NXT1	0.30553117	4.07775507	5.02454984	7.06E-07	6.91E-06	4.1377823	6.151341515	up-regulated in Low
CST3	-0.6539036	6.38341827	-9.0184602	4.18E-18	2.17E-16	29.5102244	17.37873832	up-regulated in High
CST1	-0.6507242	2.88136568	-3.2688326	0.00115495	0.00540338	-2.8970702	2.937435909	up-regulated in High
CST2	-0.6272043	1.59163631	-5.6526029	2.67E-08	3.39E-07	7.31165404	7.573344448	up-regulated in High
CST5	-0.4445357	0.51976838	-8.073966	5.20E-15	1.80E-13	22.46943	14.28382464	up-regulated in High
GGTLC1	-2.1164473	2.55475346	-14.088229	3.77E-38	9.93E-36	75.365597	37.42313559	up-regulated in High
CST7	-0.30146	3.3398275	-2.9295965	0.0035505	0.01439034	-3.9296733	2.449710368	up-regulated in High
ACSS1	-0.7786779	2.59747975	-9.9237338	2.72E-21	2.09E-19	36.7761842	20.56506422	up-regulated in High
GINS1	1.0245732	2.09811299	14.8698823	1.28E-41	4.13E-39	83.3251334	40.89293052	up-regulated in Low
NANP	0.16312237	1.82843876	4.0445096	6.08E-05	0.00039836	-0.1325574	4.2161233	up-regulated in Low
ZNF337	-0.1524687	0.84739123	-4.3155327	1.92E-05	0.0001406	0.96305527	4.716007261	up-regulated in High
FAM182B	-0.1148511	0.25725047	-3.3743885	0.00079781	0.00389805	-2.5536655	3.098100728	up-regulated in High
REM1	-0.2702408	0.77395058	-7.2336069	1.80E-12	4.39E-11	16.7122848	11.74526953	up-regulated in High
HM13	0.16733106	4.50069923	4.17674095	3.49E-05	0.00024172	0.39371866	4.456609004	up-regulated in Low
COX4I2	-0.5206642	2.52572922	-6.5924956	1.11E-10	2.10E-09	12.6659427	9.954821053	up-regulated in High
TPX2	1.76953552	3.64597188	19.1850632	9.25E-62	1.39E-58	129.578741	61.03362909	up-regulated in Low
FOXS1	-0.2273723	1.24010983	-4.1821339	3.42E-05	0.00023672	0.41551761	4.46655498	up-regulated in High
TTLL9	-0.1627244	0.25123817	-4.7045845	3.30E-06	2.81E-05	2.65047668	5.48094686	up-regulated in High
PDRG1	0.38937994	3.53429429	8.25252716	1.41E-15	5.30E-14	23.7557656	14.84993499	up-regulated in Low
CCM2L	-0.2350248	0.99206007	-5.1622834	3.54E-07	3.68E-06	4.8052264	6.451249706	up-regulated in High
HCK	-0.4345048	3.36020647	-4.6289693	4.70E-06	3.89E-05	2.31203873	5.327936848	up-regulated in High
TM9SF4	0.15117097	4.50682498	3.59041561	0.00036303	0.00195035	-1.818446	3.440053771	up-regulated in Low

POFUT1	0.31825817	3.6408839	7.09270277	4.56E-12	1.06E-10	15.796593	11.34062155	up-regulated in Low
KIF3B	0.1850608	3.61971102	3.63668262	0.00030508	0.00167525	-1.6553481	3.515581608	up-regulated in Low
DNMT3B	0.64696012	1.18504483	10.8170138	1.26E-24	1.34E-22	44.3943753	23.90007433	up-regulated in Low
MAPRE1	0.37086606	5.77637271	9.53039244	6.99E-20	4.50E-18	33.5600934	19.15549532	up-regulated in Low
BPIFA2	0.38774891	1.12262523	2.82328372	0.00494473	0.01913824	-4.2308279	2.305857054	up-regulated in Low
BPIFB1	-0.951986	3.64966562	-4.0119704	6.95E-05	0.0004505	-0.2596339	4.157943545	up-regulated in High
CDK5RAP1	0.21205192	2.9650262	5.4350783	8.61E-08	1.00E-06	6.17479956	7.065125918	up-regulated in Low
E2F1	1.05868834	3.11545072	13.4937155	1.46E-35	3.39E-33	69.4353612	34.83682465	up-regulated in Low
PXMP4	-0.8052306	2.78227085	-10.064626	8.34E-22	6.71E-20	37.9493699	21.0789869	up-regulated in High
EIF2S2	0.43025467	5.18208538	12.1241516	8.29E-30	1.33E-27	56.2490385	29.08147485	up-regulated in Low
AHCY	0.53999204	5.431541	10.0397176	1.03E-21	8.23E-20	37.7411662	20.98779155	up-regulated in Low
ITCH	0.14491038	3.58838626	3.41658984	0.00068625	0.00341588	-2.4134553	3.163515262	up-regulated in Low
DYNLRB1	0.12844501	5.37329731	3.03149376	0.00256083	0.01083395	-3.6309418	2.591618628	up-regulated in Low
MAP1LC3A	-0.4644541	3.28302595	-5.6286862	3.04E-08	3.83E-07	7.18473353	7.516660547	up-regulated in High
PIGU	0.28575411	3.73430425	6.22872782	1.00E-09	1.63E-08	10.5100849	8.997974446	up-regulated in Low
NCOA6	0.19287818	2.947942	3.86527083	0.00012573	0.00076461	-0.8205778	3.900557593	up-regulated in Low
GGT7	-0.3041046	3.35801914	-4.1942873	3.24E-05	0.00022587	0.46473917	4.489008518	up-regulated in High
ACSS2	-0.3884959	3.75439045	-5.7773041	1.34E-08	1.80E-07	7.98105577	7.872095174	up-regulated in High
GSS	0.36058186	4.95704693	8.05723445	5.87E-15	2.01E-13	22.350001	14.23124681	up-regulated in Low
MMP24	-0.6818131	2.13992812	-7.5239295	2.52E-13	6.90E-12	18.644654	12.59832218	up-regulated in High
EIF6	0.25291061	6.33804202	5.32491491	1.53E-07	1.71E-06	5.61414893	6.814059603	up-regulated in Low
UQCC1	0.11601042	2.6102995	3.19437976	0.00149055	0.00677268	-3.1329994	2.826652619	up-regulated in Low
GDF5	-0.2126277	0.49383276	-3.9880116	7.67E-05	0.00049151	-0.3525865	4.115357899	up-regulated in High
CEP250	0.2271886	1.97385002	4.38833958	1.40E-05	0.000105	1.26861797	4.854929491	up-regulated in Low
SPAG4	0.25811797	2.82975089	2.99569931	0.00287528	0.01196742	-3.7370026	2.541319728	up-regulated in Low
CPNE1	0.20836813	5.03196376	3.00942666	0.00275073	0.01151304	-3.6964714	2.56055207	up-regulated in Low
RBM12	0.17123546	3.82121012	5.04139568	6.49E-07	6.41E-06	4.21854252	6.187658612	up-regulated in Low
NFS1	0.17355275	2.38579863	5.05975046	5.92E-07	5.89E-06	4.30681427	6.227344394	up-regulated in Low
ROMO1	0.36777767	6.25635599	5.33450885	1.46E-07	1.63E-06	5.66256869	6.835754713	up-regulated in Low
SCAND1	0.18055224	4.39675533	2.73622517	0.00643792	0.02399713	-4.4694106	2.191254134	up-regulated in Low
EPB41L1	-0.2605521	3.26040089	-4.4647928	9.93E-06	7.70E-05	1.59457068	5.00291124	up-regulated in High
MYL9	-0.4851837	5.63242263	-5.8373395	9.60E-09	1.32E-07	8.30788027	8.017832472	up-regulated in High
TGIF2	0.19054956	3.17438608	3.38103933	0.00077918	0.00381883	-2.5316792	3.108365133	up-regulated in Low
C20orf24	0.62067028	4.45472926	10.4400882	3.38E-23	3.16E-21	41.1284976	22.47098112	up-regulated in Low
NDRG3	0.15979309	3.97386182	4.11579242	4.52E-05	0.0003056	0.14918143	4.344955384	up-regulated in Low
DSN1	0.603334	3.20742483	10.9145485	5.31E-25	5.93E-23	45.2511784	24.27486241	up-regulated in Low
SOGA1	0.13831653	2.09483718	2.43257572	0.01534475	0.04989331	-5.2446601	1.814040081	up-regulated in Low
SAMHD1	-0.2975534	4.56147616	-3.8742646	0.00012131	0.00074066	-0.7867532	3.91610547	up-regulated in High
RBL1	0.45535118	1.70949873	9.93433787	2.49E-21	1.92E-19	36.8640997	20.60358097	up-regulated in Low
MROH8	-0.1161433	0.41835434	-6.3848778	3.95E-10	6.84E-09	11.4228051	9.403361135	up-regulated in High
RPN2	0.29721994	7.36749017	6.06960224	2.55E-09	3.89E-08	9.59987912	8.593236666	up-regulated in Low
MANBAL	0.1415096	4.75226858	3.35237659	0.00086249	0.004177	-2.6261377	3.064248473	up-regulated in Low
SRC	0.11889466	3.89941946	2.11988478	0.03451217	0.09698515	-5.949901	1.462027765	up-regulated in Low
CTNNBL1	0.19425008	3.57478855	4.85213263	1.64E-06	1.49E-05	3.32528091	5.785497405	up-regulated in Low
VSTM2L	-0.6140572	4.43719287	-3.625615	0.0003181	0.00173837	-1.6945432	3.497441072	up-regulated in High
TTI1	0.35441548	3.15460043	8.40632287	4.52E-16	1.82E-14	24.8807621	15.34476977	up-regulated in Low
RPRD1B	0.1979491	3.18026872	4.52335847	7.63E-06	6.06E-05	1.84777339	5.117722942	up-regulated in Low
TGM2	-0.2147536	5.72275539	-2.2203056	0.02684962	0.07919185	-5.7337601	1.571061886	up-regulated in High
LBP	0.57695209	0.55569727	4.87666953	1.46E-06	1.34E-05	3.43933832	5.836907646	up-regulated in Low
ARHGAP40	-0.6627545	1.78576222	-6.3178037	5.91E-10	9.94E-09	11.0283904	9.228235849	up-regulated in High
ACTR5	0.18553714	2.57772208	4.56260384	6.38E-06	5.14E-05	2.0191463	5.195362849	up-regulated in Low
PPP1R16B	-0.3407763	1.71216925	-4.6175444	4.95E-06	4.08E-05	2.2613404	5.304999457	up-regulated in High
FAM83D	1.23821358	2.56015331	15.9945235	9.91E-47	4.42E-44	95.055047	46.00372972	up-regulated in Low
DHX35	0.1318116	1.94596637	3.74361855	0.00020266	0.00117016	-1.2708173	3.69323268	up-regulated in Low
MAFB	-0.294321	3.65765748	-3.5604778	0.00040587	0.00215511	-1.9229247	3.391612505	up-regulated in High
TOP1	0.14072841	5.52626115	2.64621134	0.00839872	0.0299961	-4.7084656	2.075786688	up-regulated in Low
ZHX3	-0.119543	1.78973043	-2.8967232	0.00393758	0.01573819	-4.0239436	2.404770526	up-regulated in High
EMILIN3	0.11090272	0.1396039	3.72331869	0.00021919	0.00125457	-1.3446242	3.659177962	up-regulated in Low
PTPRT	-0.2141462	0.27574036	-4.6760028	3.78E-06	3.18E-05	2.52196083	5.422866008	up-regulated in High
SGK2	0.17648813	0.82466931	2.62886596	0.00883304	0.03134022	-4.7536378	2.053889815	up-regulated in Low
MYBL2	1.92492365	3.76735537	17.5667449	4.65E-54	3.31E-51	111.885476	53.3327171	up-regulated in Low
OSER1	0.22055463	4.38955258	4.58160335	5.85E-06	4.75E-05	2.10260049	5.233152494	up-regulated in Low
FITM2	-0.2069858	2.36575354	-3.9102548	0.00010505	0.00065178	-0.650657	3.978626638	up-regulated in High
HNF4A	0.23843678	0.51822921	2.81035181	0.00514456	0.0198163	-4.2667254	2.288651597	up-regulated in Low
TTPAL	0.24197395	2.22456142	5.69098048	2.16E-08	2.79E-07	7.51630245	7.664715075	up-regulated in Low
ADA	0.5484053	1.97817162	8.76680614	2.95E-17	1.37E-15	27.5781648	16.5303038	up-regulated in Low
WISP2	-0.2752271	1.48755199	-3.7415025	0.00020433	0.00117781	-1.2785287	3.689675594	up-regulated in High
KCNK15	-0.1532062	0.90515063	-2.2892401	0.02248511	0.06835207	-5.5797143	1.64810498	up-regulated in High
YWHAB	0.15541265	6.35576077	4.30257331	2.03E-05	0.00014801	0.9091631	4.691484772	up-regulated in Low
TOMM34	0.32295866	4.35439431	6.63883789	8.32E-11	1.60E-09	12.947984	10.07983427	up-regulated in Low
KCNS1	-0.1349279	0.35095225	-2.653461	0.00822295	0.02946022	-4.6895	2.084972477	up-regulated in High

WFDC12	-0.2221425	0.36304509	-2.8783677	0.00417008	0.01652605	-4.0761342	2.379855897	up-regulated in High
PI3	0.76602503	2.02669178	4.52921573	7.43E-06	5.91E-05	1.87326385	5.129274677	up-regulated in Low
SLPI	-1.178339	8.04306887	-6.2750388	7.63E-10	1.26E-08	10.7787714	9.117358954	up-regulated in High
SDC4	-0.4760994	7.52632815	-6.576958	1.22E-10	2.30E-09	12.5717507	9.913062958	up-regulated in High
SYS1	-0.1104758	3.15976603	-3.1740287	0.00159685	0.00718686	-3.1965811	2.796736858	up-regulated in High
WFDC2	-1.2666836	7.53196773	-8.9635391	6.42E-18	3.26E-16	29.0852002	17.19214337	up-regulated in High
WFDC6	-0.2179731	0.236167	-4.8048093	2.06E-06	1.83E-05	3.10678136	5.686959118	up-regulated in High
WFDC10B	-0.3250027	1.27774272	-3.4449838	0.00061957	0.00312447	-2.3181835	3.207906751	up-regulated in High
WFDC3	-0.2487738	1.88476825	-2.0054109	0.04546234	0.12104012	-6.1843026	1.342348178	up-regulated in High
UBE2C	1.83410111	4.37399906	15.5950113	6.70E-45	2.63E-42	90.8542494	44.17373105	up-regulated in Low
TNNC2	-0.6543381	2.03645504	-3.8112095	0.00015569	0.00092503	-1.0223368	3.807738383	up-regulated in High
SNX21	-0.3229976	2.88174298	-4.0015805	7.25E-05	0.00046771	-0.3000075	4.139449667	up-regulated in High
ZNF335	0.12385303	2.33884362	2.94239319	0.00340945	0.01389071	-3.8926987	2.467315214	up-regulated in Low
NCOA5	0.11521028	3.91701229	2.92905183	0.00355662	0.01441286	-3.9312438	2.448962385	up-regulated in Low
CD40	-0.3327041	3.68416998	-3.6418567	0.00029917	0.00164884	-1.6369856	3.524078136	up-regulated in High
SLC35C2	0.1241928	3.28414653	3.25834359	0.00119756	0.00558082	-2.9306233	2.921701456	up-regulated in Low
ELMO2	-0.1194767	3.08151566	-3.0631782	0.00230915	0.00990117	-3.5360465	2.636548115	up-regulated in High
ZNF334	-0.1753055	0.66105514	-3.382557	0.00077498	0.00380082	-2.5266563	3.110709731	up-regulated in High
TP53RK	0.10628688	2.77799345	2.6566792	0.00814598	0.02922034	-4.6810647	2.089056583	up-regulated in Low
EYA2	0.3138547	1.7361127	2.27473464	0.02334831	0.07058449	-5.6125124	1.63174457	up-regulated in Low
NCOA3	0.19772165	3.36202341	3.88337127	0.00011698	0.00071694	-0.7524283	3.931879517	up-regulated in Low
PREX1	-0.3147442	2.86979771	-4.4932657	8.74E-06	6.84E-05	1.71729068	5.058572176	up-regulated in High
ARFGEF2	0.16923154	3.17479419	3.42379403	0.00066873	0.0033389	-2.3893538	3.174749526	up-regulated in Low
CSE1L	0.73387289	4.66132215	14.6057349	1.94E-40	5.93E-38	80.6160208	39.71212902	up-regulated in Low
STAU1	0.15372881	5.41115347	3.77470417	0.00017961	0.00104981	-1.1570605	3.745681625	up-regulated in Low
DDX27	0.29510686	3.83065627	6.50812103	1.87E-10	3.41E-09	12.1566955	9.729007488	up-regulated in Low
ZNFX1	-0.1422576	3.471054	-2.5439496	0.01126321	0.03851167	-4.9706113	1.948337701	up-regulated in High
PTGIS	-0.443437	2.22084435	-4.7458341	2.72E-06	2.36E-05	2.83721496	5.565293907	up-regulated in High
B4GALT5	0.19525539	4.74496739	3.32461869	0.000951	0.0045513	-2.7168818	3.021821046	up-regulated in Low
SPATA2	0.11638602	2.48343256	3.00354221	0.0028035	0.01171125	-3.7138676	2.552299052	up-regulated in Low
RNF114	0.11515972	4.35385036	2.76634061	0.00588068	0.02221216	-4.3876992	2.230572145	up-regulated in Low
SNAI1	0.37475046	1.98137073	4.97827389	8.87E-07	8.54E-06	3.91718913	6.052101152	up-regulated in Low
UBE2V1	0.1552776	1.57297587	5.59976207	3.56E-08	4.44E-07	7.03187254	7.448373834	up-regulated in Low
TMEM189	0.31064838	2.7447361	7.29476309	1.19E-12	2.99E-11	17.1142559	11.92281526	up-regulated in Low
CEBPB	0.17188315	5.39781474	2.47841699	0.01352857	0.04494922	-5.133311	1.868748053	up-regulated in Low
PARD6B	-0.2838895	2.20824052	-4.8356043	1.77E-06	1.61E-05	3.24874554	5.750989534	up-regulated in High
BCAS4	-0.2474279	1.6464207	-4.3667515	1.54E-05	0.00011476	1.17752157	4.813533614	up-regulated in High
ADNP	0.15970169	4.10269069	3.83059291	0.00014425	0.00086452	-0.9503052	3.840892133	up-regulated in Low
DPM1	0.31251516	4.80602391	6.70134111	5.63E-11	1.11E-09	13.330997	10.24954608	up-regulated in Low
MOCS3	0.15963848	1.77351511	4.85107107	1.65E-06	1.50E-05	3.32035818	5.78327812	up-regulated in Low
KCNG1	0.1430001	0.15368703	4.20831477	3.05E-05	0.00021367	0.52171612	4.514992545	up-regulated in Low
NFATC2	-0.4206762	1.85144116	-7.6949326	7.71E-14	2.28E-12	19.8111076	13.11274179	up-regulated in High
ATP9A	-0.332955	3.66889566	-4.7195631	3.08E-06	2.65E-05	2.71811327	5.511503657	up-regulated in High
ZFP64	0.24676298	1.94860098	7.64283222	1.11E-13	3.20E-12	19.4535256	12.9550826	up-regulated in Low
CYP24A1	1.13886529	2.94385855	5.91157257	6.31E-09	8.95E-08	8.71606048	8.199740693	up-regulated in Low
PFDN4	0.51777968	3.43544456	9.39238389	2.14E-19	1.30E-17	32.4528873	18.66995016	up-regulated in Low
DOK5	-0.1474429	1.00009953	-1.9885372	0.04730188	0.12495205	-6.2177719	1.325121558	up-regulated in High
CBLN4	-0.1935981	0.28585455	-6.0533921	2.80E-09	4.23E-08	9.50829313	8.552483834	up-regulated in High
FAM210B	-0.4168425	4.5644744	-7.5040135	2.89E-13	7.82E-12	18.5101538	12.53898213	up-regulated in High
AURKA	1.43673473	2.92576823	19.7565173	1.67E-64	3.74E-61	135.886783	63.77846272	up-regulated in Low
CSTF1	0.1732102	3.07549614	5.36842138	1.22E-07	1.38E-06	5.83434444	6.912702292	up-regulated in Low
CASS4	-0.5535813	1.25175285	-10.995537	2.58E-25	2.97E-23	45.9662062	24.58759401	up-regulated in High
RTFDC1	0.10373834	4.53358897	3.16096329	0.00166873	0.00746424	-3.2371951	2.777613688	up-regulated in Low
RAE1	0.38992678	2.79906953	9.35336834	2.93E-19	1.74E-17	32.1419154	18.53355216	up-regulated in Low
RBM38	0.19730508	3.99775058	2.66311254	0.00799406	0.02876551	-4.6641724	2.097232632	up-regulated in Low
PCK1	0.26697784	0.20843957	4.40661754	1.29E-05	9.76E-05	1.34607149	4.890112409	up-regulated in Low
PMEPA1	-0.243439	4.30370409	-2.5225481	0.01196282	0.04050058	-5.0242005	1.922166591	up-regulated in High
C20orf85	-1.0949242	1.84410555	-6.2565637	8.52E-10	1.40E-08	10.6713792	9.06964656	up-regulated in High
VAPB	0.21370446	2.83657104	5.10070565	4.83E-07	4.89E-06	4.504818	6.316329947	up-regulated in Low
APCDD1L	0.25982932	0.50353061	4.4858317	9.04E-06	7.05E-05	1.68518023	5.044010906	up-regulated in Low
TX16-NPEPL	-0.1423791	0.44222167	-4.1607219	3.74E-05	0.00025711	0.32912253	4.427129547	up-regulated in High
NPEPL1	-0.1281856	1.53852523	-2.1859779	0.02928441	0.08495622	-5.8087492	1.533363494	up-regulated in High
NELFCD	0.3138637	3.90869067	6.58779007	1.14E-10	2.16E-09	12.637397	9.942166333	up-regulated in Low
CTSZ	-0.3715687	7.47387031	-6.0891007	2.28E-09	3.51E-08	9.71032311	8.642373793	up-regulated in High
ATP5E	0.16892328	5.42243706	3.33532164	0.00091591	0.0044018	-2.6819779	3.038145739	up-regulated in Low
SLMO2	0.44226364	5.04914114	7.59580604	1.54E-13	4.33E-12	19.1324159	12.81347538	up-regulated in Low
ZNF831	-0.143519	0.36519556	-5.1841928	3.17E-07	3.32E-06	4.91289283	6.499579956	up-regulated in High
PHACTR3	-0.512846	1.16533787	-4.676847	3.76E-06	3.17E-05	2.52574647	5.424577252	up-regulated in High
PPP1R3D	-0.1244182	2.29662997	-2.5733945	0.01036028	0.03585477	-4.8961608	1.984628427	up-regulated in High
CDH26	-0.2148145	1.13420997	-2.4655947	0.01401633	0.04627229	-5.1646608	1.853365585	up-regulated in High
TAF4	0.19192143	1.91080101	4.82326765	1.88E-06	1.69E-05	3.19177468	5.725297355	up-regulated in Low

LSM14B	0.14389993	3.99473273	2.78502004	0.00555735	0.02118403	-4.336581	2.255132629	up-regulated in Low
PSMA7	0.44816156	6.57327549	9.35640604	2.86E-19	1.70E-17	32.1660948	18.54415813	up-regulated in Low
SS18L1	0.11784702	2.46883644	2.03390554	0.04249324	0.11472545	-6.1271515	1.371680209	up-regulated in Low
MTG2	0.18488437	2.83562711	3.92003722	0.00010099	0.00062973	-0.6134606	3.995704142	up-regulated in Low
ADRM1	0.41392891	5.64376114	8.42970234	3.80E-16	1.55E-14	25.0531471	15.42057294	up-regulated in Low
LAMA5	0.2937663	3.69385463	3.09258615	0.00209609	0.00910326	-3.4471195	2.678590553	up-regulated in Low
RPS21	0.27364814	8.34542371	3.68751376	0.00025148	0.00141486	-1.4738797	3.599489955	up-regulated in Low
CABLES2	0.22811804	2.39736786	3.7820943	0.0001745	0.00102336	-1.1298859	3.758203969	up-regulated in Low
GATA5	-0.2268986	0.41373376	-6.83553	2.41E-11	5.03E-10	14.1633722	10.61815438	up-regulated in High
SLCO4A1	0.26143492	1.60122936	2.65459843	0.00819567	0.02937331	-4.6865197	2.086415518	up-regulated in Low
NTSR1	0.28039199	0.22253357	4.29895109	2.07E-05	0.0001501	0.89412681	4.684641689	up-regulated in Low
MRGBP	0.47467233	3.30656934	9.97269051	1.81E-21	1.41E-19	37.1825933	20.7431101	up-regulated in Low
COL9A3	0.16531588	0.55776834	2.84772637	0.00458616	0.01793518	-4.1625419	2.338550648	up-regulated in Low
TCFL5	0.14258766	2.25085192	2.97171872	0.00310532	0.01279445	-3.8073787	2.507894067	up-regulated in Low
GID8	0.19340227	4.29620379	4.24854938	2.57E-05	0.00018276	0.68612348	4.589926511	up-regulated in Low
SLC17A9	-0.2191898	2.21470021	-2.1403026	0.03281784	0.09310666	-5.9067497	1.48389005	up-regulated in High
YTHDF1	0.11569884	4.69895592	2.69044773	0.00737653	0.0268596	-4.5919549	2.132147689	up-regulated in Low
BIRC7	-0.3279024	0.99718504	-4.5690686	6.19E-06	5.01E-05	2.04750645	5.208206243	up-regulated in High
ARFGAP1	0.15237706	4.01326674	2.57302371	0.01037124	0.03587992	-4.8971035	1.984169424	up-regulated in Low
PPDPF	-0.2051815	7.6414125	-2.5768095	0.01025986	0.03557258	-4.8874721	1.98885868	up-regulated in High
PTK6	-0.2353243	3.12577078	-2.130263	0.03364181	0.09500167	-5.9280183	1.473120617	up-regulated in High
SRMS	0.17714361	0.95647677	2.32312966	0.02057607	0.06355808	-5.5022917	1.686637641	up-regulated in Low
GMEB2	0.23296446	2.67072537	5.46997161	7.15E-08	8.47E-07	6.35450631	7.145537698	up-regulated in Low
ZBTB46	-0.2272342	1.55050722	-5.4333245	8.69E-08	1.01E-06	6.16579422	7.061095593	up-regulated in High
TPD52L2	0.27262969	5.12468905	6.40844516	3.43E-10	6.00E-09	11.5622266	9.465246786	up-regulated in Low
SOX18	-0.1788122	2.06809789	-2.6905075	0.00737523	0.02685821	-4.5917961	2.132224357	up-regulated in High
RGS19	-0.1167272	3.5309637	-2.0797971	0.03805758	0.10498001	-6.0334409	1.419558863	up-regulated in High
OPRL1	-0.1931838	1.12686177	-4.129501	4.27E-05	0.00028972	0.20389061	4.369948281	up-regulated in High
PCMTD2	-0.1496696	3.48117884	-3.1796734	0.00156668	0.00707565	-3.1789848	2.805018705	up-regulated in High
CBS	0.13172063	0.22567836	5.79974813	1.18E-08	1.60E-07	8.10289286	7.926433794	up-regulated in Low
HSPA13	0.30417107	3.51685341	5.07090058	5.60E-07	5.60E-06	4.36057862	6.251511474	up-regulated in Low
SAMSN1	-0.2701785	2.39845886	-3.4107439	0.00070079	0.00348021	-2.4329771	3.154413503	up-regulated in High
NRIP1	0.1671294	2.85881574	2.41543174	0.01607739	0.05186328	-5.2857816	1.793784332	up-regulated in Low
USP25	-0.1848357	2.85941347	-3.7593884	0.00019064	0.00110753	-1.2132193	3.719794785	up-regulated in High
CXADR	-0.1893743	3.19994834	-2.6421784	0.00849796	0.03030974	-4.7189943	2.070685292	up-regulated in High
BTG3	-0.3119048	4.63232615	-4.7851083	2.26E-06	1.99E-05	3.01639345	5.646175878	up-regulated in High
CHODL	0.18264534	0.65969446	2.71172862	0.00692587	0.02551135	-4.5352365	2.159525985	up-regulated in Low
NCAM2	-0.3335611	0.83173998	-6.5557476	1.39E-10	2.60E-09	12.4434704	9.856185906	up-regulated in High
MRPL39	0.26332871	3.89249616	5.44453819	8.19E-08	9.59E-07	6.2234189	7.086884125	up-regulated in Low
JAM2	-0.4989948	1.43021218	-10.313647	1.00E-22	8.96E-21	40.0494615	21.99862167	up-regulated in High
ATP5J	0.21323577	5.0077604	4.36868899	1.52E-05	0.00011387	1.18568015	4.817241722	up-regulated in Low
CYYR1	-0.4543932	2.13346881	-8.0128599	8.09E-15	2.72E-13	22.0341805	14.09219392	up-regulated in High
ADAMTS1	-0.4360365	2.46169498	-5.0706329	5.61E-07	5.61E-06	4.35928648	6.250930698	up-regulated in High
ADAMTS5	0.19883215	0.70748736	4.18942022	3.31E-05	0.0002301	0.44501144	4.480009967	up-regulated in Low
N6AMT1	-0.1382384	1.61318398	-3.9938888	7.49E-05	0.00048123	-0.3298331	4.1257845	up-regulated in High
CCT8	0.39171761	5.68693688	9.63602222	2.95E-20	1.99E-18	34.4150535	19.53032382	up-regulated in Low
MAP3K7CL	-0.1448116	1.1879505	-3.6330214	0.00030933	0.00169602	-1.6683267	3.509575453	up-regulated in High
CLDN8	-0.2573129	0.92557617	-2.3074501	0.02144094	0.06580144	-5.5382508	1.668756095	up-regulated in High
SOD1	0.25968318	6.2985557	4.96628215	9.41E-07	9.02E-06	3.86032679	6.026509858	up-regulated in Low
MIS18A	0.71333397	3.03904907	14.2470432	7.55E-39	2.09E-36	76.9685097	38.12202503	up-regulated in Low
URB1	0.15387487	2.56494585	2.66922719	0.00785203	0.028333	-4.6480802	2.105018205	up-regulated in Low
EVA1C	-0.6282163	2.5136072	-7.622813	1.27E-13	3.64E-12	19.3166365	12.8947188	up-regulated in High
SYNJ1	-0.2469787	1.5872703	-6.3057617	6.35E-10	1.06E-08	10.9579554	9.196953138	up-regulated in High
OLIG1	-0.1304796	0.24993757	-3.1127486	0.00196063	0.008591	-3.3856778	2.707605068	up-regulated in High
IFNAR2	-0.3955192	2.93309807	-7.390162	6.28E-13	1.62E-11	17.7467351	12.20207154	up-regulated in High
IL10RB	-0.1243609	4.37572585	-2.47015	0.0138413	0.0457745	-5.1535416	1.858823243	up-regulated in High
IFNAR1	-0.2668965	3.99976999	-6.8048584	2.93E-11	6.04E-10	13.9719089	10.53339216	up-regulated in High
IFNGR2	-0.2243395	5.49936915	-4.0655301	5.57E-05	0.00036904	-0.0499542	4.253917758	up-regulated in High
TMEM50B	-0.4810809	3.86714684	-8.6938831	5.16E-17	2.33E-15	27.0257562	16.28761536	up-regulated in High
DNAJC28	-0.1463208	0.71146305	-5.3898222	1.09E-07	1.25E-06	5.94324357	6.961469096	up-regulated in High
GART	0.37922537	3.36441502	8.9294144	8.38E-18	4.16E-16	28.8220576	17.07660473	up-regulated in Low
SON	-0.1467519	4.34261968	-3.1953555	0.00148563	0.00675239	-3.1299411	2.828090914	up-regulated in High
DONSON	0.53440625	2.38390397	10.6547209	5.24E-24	5.31E-22	42.9792735	23.28095235	up-regulated in Low
CRYZL1	-0.1232343	1.88459243	-3.8300206	0.00014457	0.00086621	-0.9524371	3.839911149	up-regulated in High
ATP5O	0.11807978	4.19575488	2.46040386	0.01421818	0.04682206	-5.1773071	1.847155942	up-regulated in Low
KCNE1	-0.1067705	0.19627098	-4.9630433	9.56E-07	9.15E-06	3.84498994	6.019606684	up-regulated in High
RCAN1	-0.3706797	2.5301555	-6.2448027	9.13E-10	1.49E-08	10.6031555	9.039332709	up-regulated in High
CLIC6	-1.4210179	4.54647459	-8.8706041	1.32E-17	6.40E-16	28.3702642	16.87821065	up-regulated in High
RUNX1	-0.416062	3.31311389	-7.9875156	9.70E-15	3.23E-13	21.8544039	14.01303008	up-regulated in High
SETD4	-0.1323171	1.80530883	-3.2667332	0.00116337	0.00544013	-2.9037941	2.934283325	up-regulated in High
CBR3	0.22024549	2.6144246	1.97483524	0.04884148	0.12809754	-6.2447453	1.311211188	up-regulated in Low

MORC3	-0.1918063	2.55819494	-5.1155147	4.48E-07	4.57E-06	4.57676838	6.348653901	up-regulated in High
CHAF1B	0.65808348	1.79973077	13.165706	3.70E-34	7.72E-32	66.2139163	33.4314029	up-regulated in Low
SIM2	0.28512617	0.47313628	5.83147997	9.92E-09	1.36E-07	8.27585185	8.003553818	up-regulated in Low
RIPPLY3	0.45953225	0.74994599	6.59963088	1.06E-10	2.02E-09	12.7092603	9.974023688	up-regulated in Low
TTC3	-0.2215509	3.5602538	-4.5116281	8.04E-06	6.35E-05	1.7968149	5.094626061	up-regulated in High
DYRK1A	-0.1347478	2.93577149	-3.7519247	0.00019624	0.00113681	-1.2405082	3.707211649	up-regulated in High
DSCR8	0.20536385	0.23010443	3.05255593	0.00239088	0.01021036	-3.5679663	2.621442921	up-regulated in Low
KCNJ15	-0.9677412	2.10916563	-8.2552138	1.39E-15	5.20E-14	23.7752833	14.85852207	up-regulated in High
ERG	-0.4692857	1.56355631	-9.5556314	5.69E-20	3.73E-18	33.7637866	19.24480541	up-regulated in High
PSMG1	0.42868082	3.28582451	8.33723486	7.56E-16	2.95E-14	24.3734543	15.12165828	up-regulated in Low
BRWD1	-0.1032744	1.90579623	-2.6817441	0.00756833	0.02746208	-4.6150271	2.120999769	up-regulated in High
HMGN1	0.1703752	4.98069911	3.96035058	8.58E-05	0.00054317	-0.4592527	4.066458423	up-regulated in Low
WRB	-0.1566134	2.60219185	-3.4213673	0.00067459	0.0033644	-2.3974779	3.17096305	up-regulated in High
LCA5L	-0.109812	0.57355522	-3.9031542	0.00010808	0.00066834	-0.6776015	3.966253245	up-regulated in High
SH3BGR	-0.1678541	1.70061931	-3.0624608	0.00231459	0.00991866	-3.5382057	2.635526592	up-regulated in High
MX2	-0.3740538	2.36840314	-4.7978536	2.13E-06	1.89E-05	3.07483021	5.672544064	up-regulated in High
TMPRSS2	-1.6369111	3.84109246	-15.462461	2.69E-44	1.03E-41	89.4684329	43.56995239	up-regulated in High
RIPK4	-0.1850323	2.90087698	-2.4876746	0.01318582	0.04403596	-5.110578	1.879892787	up-regulated in High
C2CD2	-0.1577202	2.41216173	-3.407888	0.00070799	0.00351151	-2.4425025	3.149971721	up-regulated in High
ZBTB21	-0.1518917	1.90099458	-3.7962599	0.0001651	0.00097407	-1.0776567	3.782264449	up-regulated in High
ABCG1	-0.1863228	2.79637436	-2.4640278	0.014077	0.04642793	-5.1684811	1.851489991	up-regulated in High
TFF1	0.95993347	2.23539706	3.61784387	0.00032754	0.00178431	-1.7219965	3.484731195	up-regulated in Low
UBASH3A	-0.1317608	0.90180488	-2.5873987	0.00995396	0.03468709	-4.8604589	2.002003923	up-regulated in High
RSPH1	-0.5806206	1.46111813	-5.8987649	6.79E-09	9.56E-08	8.64531595	8.168221233	up-regulated in High
PDE9A	-0.2785999	2.06614427	-3.9656797	8.40E-05	0.00053271	-0.4387566	4.075857142	up-regulated in High
WDR4	0.3665743	2.33054874	8.20671016	1.98E-15	7.30E-14	23.4236607	14.70380979	up-regulated in Low
CBS	0.13172063	0.22567836	5.79974813	1.18E-08	1.60E-07	8.10289286	7.926433794	up-regulated in Low
HSF2BP	0.24773775	0.56433097	7.57901345	1.73E-13	4.83E-12	19.0181306	12.76306966	up-regulated in Low
RRP1B	0.33693936	3.28335432	7.72756198	6.14E-14	1.84E-12	20.0360269	13.2118926	up-regulated in Low
RRP1	0.32966852	3.179338	7.45538976	4.03E-13	1.07E-11	18.1829728	12.3946119	up-regulated in Low
AGPAT3	-0.1208978	3.34533038	-2.7304393	0.00655029	0.02433517	-4.4850099	2.183739697	up-regulated in High
C21orf2	-0.381746	2.02451143	-8.0065092	8.47E-15	2.84E-13	21.9890919	14.07234001	up-regulated in High
TRPM2	0.22568965	1.72341185	3.31007679	0.00100067	0.00476635	-2.764133	2.99971054	up-regulated in Low
LRRC3	-0.243043	1.01906134	-5.3905679	1.09E-07	1.25E-06	5.94704522	6.963171329	up-regulated in High
UBE2G2	-0.2046101	3.61044129	-4.3671685	1.53E-05	0.00011457	1.17927727	4.814331599	up-regulated in High
PTTG1IP	-0.2606565	6.87653136	-5.1970956	2.96E-07	3.12E-06	4.97649082	6.528122317	up-regulated in High
ITGB2	-0.6908954	4.64154077	-6.6625502	7.18E-11	1.39E-09	13.0929375	10.14407024	up-regulated in High
FAM207A	0.31277937	3.10502838	6.29490509	6.78E-10	1.13E-08	10.8945516	9.168790917	up-regulated in Low
ADARB1	-0.3101162	1.63186388	-6.3285139	5.54E-10	9.37E-09	11.0911319	9.256099371	up-regulated in High
COL18A1	-0.2159564	4.43687374	-2.2693151	0.02367815	0.07143205	-5.6247141	1.625652297	up-regulated in High
SLC19A1	0.11961337	1.92489888	2.44269152	0.01492643	0.04875849	-5.2202631	1.826044126	up-regulated in Low
SPATC1L	0.19033139	2.05584682	2.63957586	0.00856256	0.03051218	-4.7257804	2.067396555	up-regulated in Low
C21orf58	0.18120914	1.20752356	3.38731547	0.00076196	0.00374863	-2.5108935	3.118066653	up-regulated in Low
S100B	-1.0131738	2.41105557	-8.5904187	1.13E-16	4.93E-15	26.2478237	15.9457626	up-regulated in High
PRMT2	-0.2459731	3.44818525	-6.8960028	1.64E-11	3.50E-10	14.5429511	10.78615364	up-regulated in High
BAGE5	0.10871924	0.1354607	2.06061609	0.03986104	0.10908166	-6.0728586	1.399451421	up-regulated in Low
IL17RA	-0.2225057	2.39854637	-5.2449664	2.32E-07	2.50E-06	5.21368302	6.63453369	up-regulated in High
CECR5	0.18872797	2.96650872	4.21328649	2.99E-05	0.00020968	0.54195282	4.524219511	up-regulated in Low
CECR1	-0.5049815	3.88832302	-5.7297745	1.75E-08	2.30E-07	7.72440586	7.757594478	up-regulated in High
BCL2L13	0.14338105	3.15420543	3.86507849	0.00012583	0.00076512	-0.8213004	3.900225414	up-regulated in Low
BID	0.22576183	2.9574769	3.72764557	0.00021556	0.00123551	-1.3289242	3.666423678	up-regulated in Low
MICAL3	0.15556124	0.67254549	4.55196499	6.69E-06	5.37E-05	1.97255504	5.174260075	up-regulated in Low
USP18	0.2132801	2.58135187	3.27924832	0.00111403	0.00522996	-2.863649	2.953101797	up-regulated in Low
DGCR6	-0.1895126	1.12741855	-3.4972142	0.00051244	0.00264237	-2.1409699	3.290360739	up-regulated in High
PRODH	-0.8151364	1.84971681	-6.9834305	9.31E-12	2.06E-10	15.0965893	11.0310911	up-regulated in High
DGCR2	-0.2361072	4.8852889	-4.3810942	1.44E-05	0.00010824	1.23799785	4.841017057	up-regulated in High
SLC25A1	0.18481618	5.23454025	3.47365574	0.00055842	0.00284828	-2.2212163	3.2530423	up-regulated in Low
CLTCL1	0.14912158	0.67641025	4.20066023	3.16E-05	0.0002204	0.49060288	4.500804486	up-regulated in Low
HIRA	0.14120097	2.21948003	3.33955341	0.00090238	0.00434461	-2.6681479	3.044612216	up-regulated in Low
MRPL40	0.18419102	3.97418363	3.81339554	0.00015436	0.00091852	-1.0142306	3.811470301	up-regulated in Low
UFD1L	0.3444518	2.89697124	8.68334768	5.59E-17	2.51E-15	26.9462285	16.25267255	up-regulated in Low
CDC45	1.30323626	2.03785658	19.7307671	2.22E-64	4.79E-61	135.602031	63.65456464	up-regulated in Low
CLDN5	-0.3548292	2.68938291	-3.8772527	0.00011987	0.00073273	-0.7754988	3.921277833	up-regulated in High
GNB1L	0.21879578	0.89422014	6.96387413	1.06E-11	2.32E-10	14.9722503	10.97609179	up-regulated in Low
C22orf29	-0.1377262	2.53089648	-2.5914326	0.0098396	0.03437572	-4.8501398	2.007022771	up-regulated in High
ARVCF	-0.2448625	2.08331545	-3.523838	0.00046474	0.0024284	-2.0496612	3.33278714	up-regulated in High
TRMT2A	0.21506709	3.26576756	4.01529585	6.86E-05	0.00044489	-0.2466912	4.163871232	up-regulated in Low
RANBP1	0.71663501	3.80319687	15.2840127	1.74E-43	6.46E-41	87.6092895	42.75989178	up-regulated in Low
ZDHHC8	-0.1996482	2.93788144	-3.5033491	0.00050106	0.00259148	-2.1199882	3.300113249	up-regulated in High
RTN4R	-0.1454321	1.60741123	-2.3788899	0.01774268	0.05625839	-5.3724827	1.75098069	up-regulated in High
USP41	0.10321319	0.15083584	3.29770713	0.0010448	0.00494792	-2.80417	2.98096591	up-regulated in Low

ZNF74	0.11994744	1.745187	2.75624312	0.00606247	0.02280288	-4.4151931	2.21735072	up-regulated in Low
SCARF2	-0.4098869	2.02681248	-5.8775008	7.66E-09	1.07E-07	8.52815544	8.116014039	up-regulated in High
SERPIND1	-0.9617853	1.46755971	-6.5094108	1.85E-10	3.39E-09	12.1644386	9.732441907	up-regulated in High
CRKL	0.18229212	4.16390793	3.91507689	0.00010303	0.00064084	-0.6323325	3.987040246	up-regulated in Low
THAP7	0.1291079	3.33244798	2.54464285	0.01124117	0.03844531	-4.968868	1.949188373	up-regulated in Low
LRRC74B	-0.1318992	0.14556892	-4.0363313	6.29E-05	0.00041042	-0.164587	4.201463345	up-regulated in High
GGT2	-0.248671	0.38327351	-6.1325165	1.77E-09	2.77E-08	9.9573346	8.752243616	up-regulated in High
HIC2	0.20545077	1.05728215	4.52286804	7.64E-06	6.07E-05	1.84564045	5.116756286	up-regulated in Low
UBE2L3	0.20142497	4.59398657	5.51790211	5.54E-08	6.70E-07	6.60301698	7.256688226	up-regulated in Low
YDJC	0.4579082	3.72750627	7.59474221	1.55E-13	4.36E-12	19.1251699	12.81027961	up-regulated in Low
SDF2L1	0.26703941	4.86954611	3.88266988	0.00011731	0.00071857	-0.7550746	3.930663512	up-regulated in Low
YPEL1	-0.2139164	1.06125596	-5.0380448	6.60E-07	6.50E-06	4.2024586	6.180426473	up-regulated in High
ZNF280B	0.25110174	0.68567194	5.48250977	6.69E-08	7.97E-07	6.41932892	7.174536039	up-regulated in Low
ZNF280A	0.16974471	0.1224664	6.81486856	2.75E-11	5.69E-10	14.0343176	10.56102257	up-regulated in Low
PRAME	1.20797048	1.62104634	7.34953341	8.26E-13	2.11E-11	17.4765668	12.08280009	up-regulated in Low
GGTLC2	-0.2040511	0.23493692	-6.5718015	1.26E-10	2.37E-09	12.5405321	9.899221912	up-regulated in High
IGLL5	-0.4361181	4.96314227	-2.6017399	0.00955269	0.03351879	-4.8237024	2.019874561	up-regulated in High
RAB36	-0.4500403	1.83024305	-6.0034403	3.74E-09	5.54E-08	9.22739991	8.427462354	up-regulated in High
BCR	-0.1253731	3.32782679	-2.2213742	0.02677673	0.07900813	-5.7314073	1.572242505	up-regulated in High
IGLL1	-0.110372	0.40650795	-2.1311332	0.03356969	0.09485258	-5.9261787	1.474052623	up-regulated in High
ZNF70	-0.1107317	1.10148887	-3.5448732	0.00043003	0.0022659	-1.9770531	3.366497299	up-regulated in High
VPREB3	-0.2348018	1.65264404	-2.3989291	0.01681159	0.05380279	-5.3250965	1.774391333	up-regulated in High
C22orf15	-0.2732695	0.40731476	-4.8822435	1.42E-06	1.31E-05	3.46532132	5.848616684	up-regulated in High
CHCHD10	0.31207831	4.24606053	4.53356964	7.28E-06	5.80E-05	1.89223146	5.137869623	up-regulated in Low
SMARCB1	0.20435453	4.91808005	5.18197479	3.20E-07	3.36E-06	4.90197469	6.494679515	up-regulated in Low
DERL3	-0.3947317	3.23467083	-3.6236371	0.00032048	0.00174973	-1.7015361	3.494203932	up-regulated in High
SLC2A11	-0.203316	1.20796311	-5.2855438	1.88E-07	2.06E-06	5.41626112	6.725368692	up-regulated in High
MIF	0.57291172	5.30200738	7.62140795	1.29E-13	3.68E-12	19.3070397	12.89048673	up-regulated in Low
GSTT2B	-0.1784149	1.2444547	-2.3171643	0.02090145	0.06442002	-5.5160005	1.679823616	up-regulated in High
DDT	0.19393491	3.71721839	3.43597546	0.00064004	0.00321122	-2.348491	3.193790004	up-regulated in Low
CABIN1	-0.2322556	3.32818489	-4.2432878	2.63E-05	0.00018663	0.66454092	4.580093162	up-regulated in High
SUSD2	-2.2205571	3.93544267	-14.331555	3.20E-39	9.11E-37	77.8245556	38.49524278	up-regulated in High
GGT5	-0.5908112	2.86420206	-7.5163387	2.66E-13	7.25E-12	18.5933575	12.57569131	up-regulated in High
SPECC1L	-0.1514199	2.5582283	-3.8838887	0.00011674	0.00071553	-0.7504759	3.932776643	up-regulated in High
GUCD1	-0.1268951	4.45600529	-3.1267229	0.00187152	0.00824885	-3.3428681	2.727805168	up-regulated in High
SNRPD3	0.25217625	4.93873169	6.41291434	3.33E-10	5.84E-09	11.5887148	9.477003099	up-regulated in Low
GGT1	-0.4823919	2.64643916	-5.3249396	1.53E-07	1.71E-06	5.61427332	6.814115342	up-regulated in High
LRRC75B	-0.4950876	1.92995083	-7.1511344	3.11E-12	7.36E-11	16.1745459	11.50767465	up-regulated in High
SGSM1	-0.1377576	0.812489	-2.9462947	0.00336748	0.01374272	-3.8813948	2.472694988	up-regulated in High
KIAA1671	-0.3486527	2.35290102	-6.1964524	1.22E-09	1.95E-08	10.3238361	8.915195357	up-regulated in High
LRP5L	-0.1618592	1.25541196	-2.8646956	0.0043513	0.01714993	-4.1147997	2.361381506	up-regulated in High
ADRBK2	-0.2552687	2.14462797	-4.0581941	5.75E-05	0.00037936	-0.0788276	4.240709047	up-regulated in High
TPST2	-0.1234037	2.68453481	-2.9160698	0.00370537	0.01494303	-3.9685881	2.431168831	up-regulated in High
CRYBB1	-0.1564132	0.63619148	-5.1118205	4.56E-07	4.65E-06	4.55880236	6.340583173	up-regulated in High
PITPNB	0.1742697	3.73290758	5.09883487	4.87E-07	4.93E-06	4.49574213	6.312252153	up-regulated in Low
TTC28	-0.2257829	1.50936096	-4.7191028	3.09E-06	2.65E-05	2.7160315	5.510563261	up-regulated in High
CHEK2	0.59881315	2.10975325	10.6065908	7.97E-24	7.92E-22	42.5621801	23.09844014	up-regulated in Low
RHBDD3	0.21844327	3.11314351	3.99842299	7.35E-05	0.00047317	-0.3122578	4.133837305	up-regulated in Low
EWSR1	0.14905247	4.41112358	3.90723961	0.00010632	0.0006586	-0.6621043	3.973370105	up-regulated in Low
GAS2L1	0.13913909	2.63395905	2.63728621	0.00861975	0.03068228	-4.7317452	2.06450532	up-regulated in Low
NEFH	0.32464171	0.82226924	2.82871411	0.00486294	0.01885063	-4.2157061	2.313100935	up-regulated in Low
NIPSNAP1	0.3714751	4.74221663	6.59441813	1.10E-10	2.08E-09	12.6776101	9.959993309	up-regulated in Low
ZMAT5	-0.1041331	2.31412249	-2.5978639	0.00965969	0.03385761	-4.8336561	2.015036956	up-regulated in High
MTMR3	-0.2731381	0.9731325	-8.2750611	1.20E-15	4.54E-14	23.9196172	14.92202112	up-regulated in High
GATSL3	-0.1868262	0.97646793	-4.5370071	7.17E-06	5.72E-05	1.90721845	5.144660338	up-regulated in High
TBC1D10A	-0.2715388	2.05657038	-7.024428	7.13E-12	1.61E-10	15.3581787	11.14678235	up-regulated in High
MTFP1	0.36551245	2.05782901	7.61619618	1.33E-13	3.80E-12	19.271454	12.87479362	up-regulated in Low
SEC14L3	-0.1659449	0.16429762	-4.4476504	1.07E-05	8.26E-05	1.52103308	4.969543664	up-regulated in High
SEC14L4	-0.3697257	1.19904751	-4.7075139	3.26E-06	2.78E-05	2.66368926	5.486916596	up-regulated in High
SEC14L6	-0.6859243	1.26585065	-8.3404427	7.38E-16	2.89E-14	24.3969391	15.13198784	up-regulated in High
PES1	0.34433461	4.13615848	9.16674501	1.30E-18	7.19E-17	30.6670438	17.88647769	up-regulated in Low
TCN2	-0.2253399	4.13049493	-2.8489507	0.00456884	0.01787462	-4.1591066	2.340194178	up-regulated in High
DUSP18	-0.1995201	1.22243669	-5.9177369	6.10E-09	8.68E-08	8.75015762	8.214931083	up-regulated in High
MORC2	0.11031575	3.32376966	2.60477955	0.00946951	0.03326894	-4.8158864	2.02367233	up-regulated in Low
SMTN	0.22562634	2.43462961	3.99914607	7.33E-05	0.00047198	-0.3094532	4.135122214	up-regulated in Low
INPP5J	-0.3031335	1.20649459	-4.923129	1.16E-06	1.09E-05	3.65673133	5.934845536	up-regulated in High
PLA2G3	-0.2437679	0.62539783	-3.4654538	0.00057531	0.00292346	-2.249033	3.240099079	up-regulated in High
RNF185	-0.113353	3.37496004	-3.4445634	0.00062052	0.00312867	-2.3195995	3.207247311	up-regulated in High
PIK3IP1	-0.8241762	3.83434336	-13.808746	6.29E-37	1.56E-34	72.5636187	36.20126269	up-regulated in High
DRG1	0.30413054	4.49387251	7.3971438	5.99E-13	1.55E-11	17.7932819	12.22261838	up-regulated in Low
SFI1	-0.1718492	1.55376155	-3.7537886	0.00019483	0.0011294	-1.2336982	3.71035203	up-regulated in High

SLC5A1	-0.3600433	0.98771725	-3.3060801	0.00101473	0.00482431	-2.777085	2.993647678	up-regulated in High
SLC5A4	-0.1045414	0.2355749	-4.4615086	1.01E-05	7.80E-05	1.58046185	4.99651019	up-regulated in High
TIMP3	-0.4035151	1.59794593	-5.271204	2.03E-07	2.20E-06	5.34451185	6.693201597	up-regulated in High
LARGE	-0.3602529	1.51411872	-5.4250609	9.08E-08	1.05E-06	6.12339663	7.042119596	up-regulated in High
HMGXB4	0.26450318	2.58283722	7.45672348	3.99E-13	1.06E-11	18.1919246	12.39856232	up-regulated in Low
TOM1	-0.2003294	3.94607389	-4.1379089	4.12E-05	0.00028049	0.23752941	4.385311751	up-regulated in High
MCM5	0.52189473	3.12672698	9.84587289	5.21E-21	3.88E-19	36.1325882	20.28307361	up-regulated in Low
RBFOX2	-0.1276788	3.16797409	-2.7468644	0.00623584	0.02336618	-4.4406425	2.205105173	up-regulated in High
APOL3	-0.1633015	2.65091359	-2.0382798	0.04205234	0.11374415	-6.1183079	1.376209859	up-regulated in High
APOL2	0.18651303	4.01108953	2.71163614	0.00692777	0.02551676	-4.5354839	2.159406631	up-regulated in Low
APOL1	0.31581912	5.38329609	2.99583134	0.00287406	0.01196319	-3.7366137	2.541504364	up-regulated in Low
FOXRED2	0.1577104	2.58653487	2.17508156	0.03009609	0.08690937	-5.8323126	1.521489897	up-regulated in Low
EIF3D	0.17453946	5.5509086	4.63786124	4.51E-06	3.75E-05	2.35157646	5.345821848	up-regulated in Low
IFT27	-0.109088	2.10518207	-2.3004911	0.02183487	0.06677901	-5.5541342	1.66084944	up-regulated in High
PVALB	-0.1401521	0.42742616	-3.0804619	0.00218164	0.00942782	-3.4838814	2.661217531	up-regulated in High
NCF4	-0.5790958	3.36895224	-7.271647	1.39E-12	3.46E-11	16.961996	11.85556996	up-regulated in High
CSF2RB	-0.4291187	2.43145624	-5.4450826	8.16E-08	9.57E-07	6.22621919	7.088137248	up-regulated in High
TST	-0.2701267	4.59843477	-3.1177751	0.00192814	0.00846643	-3.3703005	2.71486245	up-regulated in High
TMPRSS6	-0.6287573	1.36009687	-5.1893123	3.08E-07	3.24E-06	4.9381099	6.510897748	up-regulated in High
C1QTNF6	0.33919865	2.28799686	4.41244351	1.26E-05	9.53E-05	1.3708218	4.901352569	up-regulated in Low
RAC2	-0.336899	4.67835552	-3.920521	0.0001008	0.00062863	-0.6116187	3.996549653	up-regulated in High
CYTH4	-0.3026533	2.16813163	-4.5189603	7.78E-06	6.16E-05	1.82865289	5.109057158	up-regulated in High
ELFN2	-0.5814523	1.17903692	-7.142645	3.29E-12	7.75E-11	16.1194775	11.48333776	up-regulated in High
MFNG	-0.4966169	2.60001209	-8.3044354	9.63E-16	3.70E-14	24.1337148	15.01620491	up-regulated in High
CDC42EP1	-0.7150644	5.3825316	-7.1957569	2.31E-12	5.58E-11	16.4648738	11.63596518	up-regulated in High
LGALS2	-0.4606367	1.97810358	-4.9968609	8.09E-07	7.85E-06	4.00557	6.091869316	up-regulated in High
SH3BP1	0.18155869	2.22258351	3.41237191	0.00069671	0.00346233	-2.4275438	3.15694691	up-regulated in Low
TRIOBP	-0.201821	1.68251295	-5.6830101	2.26E-08	2.90E-07	7.4737005	7.645697065	up-regulated in High
H1F0	0.23715968	7.04182234	3.50442593	0.00049908	0.00258242	-2.1163017	3.30182654	up-regulated in Low
MICALL1	0.22701639	2.74083618	4.27535971	2.29E-05	0.00016469	0.79648368	4.640191637	up-regulated in Low
BAIAP2L2	0.30881583	1.47421338	2.90416867	0.00384669	0.01542347	-4.0026825	2.41491292	up-regulated in Low
CSNK1E	0.29828069	4.2751774	5.63367153	2.96E-08	3.74E-07	7.21115043	7.528459663	up-regulated in Low
KDELR3	-0.1738939	4.61629096	-1.9758819	0.04872241	0.12785529	-6.2426914	1.312271266	up-regulated in High
DDX17	-0.2631656	5.51570276	-5.1463378	3.84E-07	3.95E-06	4.72712471	6.416182566	up-regulated in High
DMC1	0.1773892	0.31552817	7.22996913	1.84E-12	4.50E-11	16.6884607	11.73474507	up-regulated in Low
FAM227A	-0.1723711	0.74043827	-3.1776003	0.0015777	0.00711659	-3.1854509	2.801975631	up-regulated in High
CBY1	-0.1579242	3.6547977	-3.0062958	0.00277869	0.01161426	-3.7057314	2.556159302	up-regulated in High
TOMM22	0.28889131	5.04454963	6.02710888	3.26E-09	4.88E-08	9.36024461	8.486595851	up-regulated in Low
GTPBP1	-0.1259342	3.16889828	-2.9171678	0.00369257	0.01489808	-3.9654359	2.432671242	up-regulated in High
SUN2	-0.1833646	4.42719093	-3.284445	0.00109412	0.00514933	-2.8469362	2.960933331	up-regulated in High
NPTXR	-0.1735042	1.34972343	-2.1260607	0.03399194	0.09583694	-5.9368916	1.468624103	up-regulated in High
CBX6	-0.2118269	2.99786736	-2.6701753	0.00783021	0.02826475	-4.6455818	2.106226679	up-regulated in High
APOBEC3B	0.51407932	2.06139542	4.83222387	1.80E-06	1.63E-05	3.23312142	5.743943983	up-regulated in Low
APOBEC3C	-0.4032989	4.10975673	-5.1258462	4.25E-07	4.35E-06	4.62707547	6.371250939	up-regulated in High
APOBEC3H	-0.2647335	1.04998053	-4.5615343	6.41E-06	5.16E-05	2.01445798	5.193239527	up-regulated in High
CBX7	-0.6984272	2.07343307	-12.674807	4.38E-32	8.24E-30	61.4650628	31.35892263	up-regulated in High
SYNGR1	-0.1094571	0.96001603	-1.9868194	0.04749263	0.12535951	-6.2211637	1.323373752	up-regulated in High
MGAT3	-0.3945084	1.08306115	-5.0639044	5.80E-07	5.78E-06	4.32683147	6.236342536	up-regulated in High
MIEF1	0.17445479	2.72838264	4.6492697	4.28E-06	3.57E-05	2.40240578	5.368810796	up-regulated in Low
ATF4	0.29567495	6.66714419	6.34176719	5.12E-10	8.70E-09	11.1688965	9.290631736	up-regulated in Low
RPS19BP1	0.12330356	3.88939714	2.48627024	0.01323732	0.04417508	-5.1140318	1.878200087	up-regulated in Low
GRAP2	-0.1564842	0.79679413	-3.6185413	0.00032669	0.00178014	-1.719535	3.485870891	up-regulated in High
FAM83F	0.41642425	0.55507789	7.06217712	5.57E-12	1.28E-10	15.6001499	11.25377518	up-regulated in Low
ADSL	0.31500816	3.33205729	6.86819318	1.96E-11	4.13E-10	14.3680514	10.70875111	up-regulated in Low
SGSM3	-0.1959841	3.90652257	-3.3446763	0.00088624	0.00427891	-2.6513832	3.052449474	up-regulated in High
CHADL	-0.3995188	1.19511173	-7.7272181	6.15E-14	1.84E-12	20.0336526	13.21084601	up-regulated in High
RANGAP1	0.3967461	4.32539463	8.12384191	3.62E-15	1.28E-13	22.8265678	14.44103393	up-regulated in Low
ZC3H7B	-0.2132347	3.54716096	-4.3043979	2.02E-05	0.00014691	0.91674167	4.694933626	up-regulated in High
TEF	-0.7681152	2.69656527	-11.935979	4.84E-29	7.30E-27	54.495179	28.31540786	up-regulated in High
TOB2	-0.2166545	4.07269908	-4.746457	2.71E-06	2.35E-05	2.84004641	5.566572415	up-regulated in High
PHF5A	0.31972052	4.405227	6.48269427	2.18E-10	3.94E-09	12.004314	9.66141366	up-regulated in Low
POLR3H	-0.2095455	3.0176336	-3.0227898	0.00263425	0.01109551	-3.6568439	2.579342885	up-regulated in High
CSDC2	-0.4209002	1.16858592	-7.9181627	1.59E-14	5.16E-13	21.3647096	13.79735825	up-regulated in High
PMM1	-0.4085284	3.13916593	-5.754363	1.52E-08	2.03E-07	7.8569468	7.816731941	up-regulated in High
XRCC6	0.17070673	6.63207173	4.50758647	8.19E-06	6.45E-05	1.77928564	5.086679836	up-regulated in Low
NHP2L1	0.10595947	5.32582421	2.68656977	0.00746144	0.02711635	-4.6022437	2.127177142	up-regulated in Low
MEI1	-0.2714707	1.16734665	-4.2193117	2.91E-05	0.00020486	0.5665073	4.535413874	up-regulated in High
CCDC134	0.11736494	2.02154114	2.90789788	0.00380189	0.01527741	-3.9920136	2.420000898	up-regulated in Low
TNFRSF13C	-0.2746143	1.10595921	-3.6775791	0.00026121	0.00146238	-1.5095342	3.58301406	up-regulated in High
CENPM	0.96558015	2.38003029	15.1293512	8.70E-43	3.06E-40	86.0042428	42.06048733	up-regulated in Low
3-Sep	0.16465611	1.0925266	2.07357352	0.03863496	0.10625573	-6.04627	1.413019566	up-regulated in Low

NAGA	-0.1986453	4.23444522	-4.5883333	5.67E-06	4.62E-05	2.13223786	5.246569914	up-regulated in High
FAM109B	-0.1115783	3.33216001	-2.1342796	0.03331005	0.09426493	-5.919521	1.477424702	up-regulated in High
SMDT1	-0.2677975	4.39745728	-4.9770092	8.92E-07	8.59E-06	3.9111864	6.049399776	up-regulated in High
NDUFA6	0.11680107	5.14011895	2.325024	0.02047367	0.06330855	-5.4979311	1.688804307	up-regulated in Low
CYP2D6	-0.1993359	0.8131259	-3.1142508	0.00195086	0.008554	-3.3810846	2.709773008	up-regulated in High
NFAM1	-0.3485576	1.80328448	-5.038171	6.60E-07	6.50E-06	4.2030643	6.180698829	up-regulated in High
RRP7A	0.18276838	3.68292655	4.00865779	7.05E-05	0.00045591	-0.272517	4.152042713	up-regulated in Low
POLDIP3	-0.1192561	4.61872892	-3.1147366	0.00194772	0.00854196	-3.3795989	2.710474223	up-regulated in High
CYB5R3	-0.3271326	5.24067839	-6.5056501	1.89E-10	3.45E-09	12.1418655	9.722429654	up-regulated in High
A4GALT	0.21179685	2.80502958	2.3475395	0.01929033	0.06028703	-5.4458365	1.714660259	up-regulated in Low
PACSIN2	-0.2234034	4.78890059	-4.0402225	6.19E-05	0.00040429	-0.1493548	4.208435502	up-regulated in High
TTLL1	-0.258762	2.24367916	-5.8681859	8.07E-09	1.12E-07	8.47694812	8.093192845	up-regulated in High
BIK	0.54112464	2.86726964	5.69097238	2.16E-08	2.79E-07	7.5162591	7.664695721	up-regulated in Low
MCAT	0.11518192	2.87299221	2.98001914	0.00302386	0.01250293	-3.7830812	2.519438991	up-regulated in Low
TTLL12	0.39258777	3.69850087	6.34305976	5.08E-10	8.65E-09	11.1764881	9.294002731	up-regulated in Low
EFCAB6	-0.1849884	0.36028094	-6.8779224	1.84E-11	3.90E-10	14.429174	10.73580253	up-regulated in High
PARVB	0.11935669	2.06519207	2.23267679	0.02601618	0.07718137	-5.7064543	1.58475651	up-regulated in Low
PARVG	-0.4191925	1.67735439	-6.933237	1.29E-11	2.79E-10	14.7780364	10.89017289	up-regulated in High
ARHGAP8	-0.143527	0.6268196	-4.5351576	7.23E-06	5.76E-05	1.89915335	5.141006032	up-regulated in High
NUP50	0.21424937	2.96548604	5.48293501	6.68E-08	7.95E-07	6.42152977	7.175520521	up-regulated in Low
UPK3A	-0.2654724	0.93363402	-2.833237	0.00479576	0.01863626	-4.2030898	2.319142863	up-regulated in High
SMC1B	0.16986565	0.4788339	2.73044814	0.00655011	0.02433517	-4.4849861	2.183751138	up-regulated in Low
RIBC2	0.27266044	1.29828902	3.81127504	0.00015565	0.00092501	-1.022094	3.807850166	up-regulated in Low
FBLN1	-0.5499457	4.26152342	-5.409196	9.87E-08	1.14E-06	6.04216069	7.005755899	up-regulated in High
WNT7B	-0.349333	2.81115686	-3.5983062	0.00035247	0.00190077	-1.7907706	3.452877587	up-regulated in High
PPARA	-0.1093607	1.63130925	-2.853663	0.00450272	0.01765852	-4.1458707	2.346525488	up-regulated in High
TTC38	-0.1181093	3.23731851	-2.5020441	0.01266905	0.04254619	-5.0751284	1.897255801	up-regulated in High
GTSE1	1.08430517	1.70635149	18.5343823	1.20E-58	1.18E-55	122.429991	57.9225555	up-regulated in Low
TRMU	0.22398071	1.93781249	4.9812536	8.74E-07	8.42E-06	3.93133757	6.058468105	up-regulated in Low
CELSR1	-0.2941218	3.53821863	-3.8518146	0.00013263	0.00080185	-0.8710472	3.87735182	up-regulated in High
CERK	-0.1174842	3.60587365	-2.1874938	0.029173	0.08468771	-5.8054618	1.535018922	up-regulated in High
TBC1D22A	-0.1799056	3.08206825	-4.7590574	2.56E-06	2.23E-05	2.89739242	5.592463713	up-regulated in High
FAM19A5	-0.2016357	1.43074746	-2.5732487	0.01036459	0.0358633	-4.8965315	1.984447933	up-regulated in High
BRD1	-0.1396605	3.10757352	-2.9522339	0.00330449	0.01350953	-3.8641592	2.480895726	up-regulated in High
ZBED4	0.14392868	2.58108966	3.58959005	0.00036416	0.00195548	-1.8213383	3.438713432	up-regulated in Low
PIM3	0.2008731	5.59375028	2.83643111	0.00474882	0.01848093	-4.1941686	2.323414313	up-regulated in Low
PANX2	0.44553412	2.30597164	4.19047171	3.30E-05	0.00022924	0.44927161	4.48195327	up-regulated in Low
TUBGCP6	-0.3550208	2.78355433	-5.2779434	1.96E-07	2.13E-06	5.37821052	6.708310268	up-regulated in High
HDAC10	-0.1146699	1.47377806	-2.2894519	0.02247271	0.06832861	-5.5792338	1.648344511	up-regulated in High
MAPK12	0.28971001	1.06096124	5.74120403	1.64E-08	2.16E-07	7.78595342	7.785057595	up-regulated in Low
PLXNB2	-0.3800115	5.77196133	-5.1138781	4.52E-07	4.60E-06	4.56880752	6.345077756	up-regulated in High
DENND6B	-0.4089388	1.75091134	-7.845703	2.67E-14	8.36E-13	20.8566182	13.57352515	up-regulated in High
PPP6R2	-0.1629683	3.55908235	-3.3880112	0.00076008	0.00374061	-2.5085871	3.119143039	up-regulated in High
LMF2	-0.117999	4.800596	-2.1581143	0.0313986	0.08994995	-5.8687745	1.503089736	up-regulated in High
NCAPH2	0.18165492	3.90688044	3.83661329	0.00014086	0.00084647	-0.9278625	3.851218222	up-regulated in Low
ODF3B	-0.2174318	3.40805201	-2.4707914	0.01381681	0.04572216	-5.1519742	1.859592382	up-regulated in High
KLHDC7B	0.2038252	1.38527866	2.330518	0.02017921	0.06253735	-5.4852648	1.695095763	up-regulated in Low
CHKB	-0.1619135	1.40005742	-3.1003005	0.00204327	0.0089017	-3.4236565	2.689673619	up-regulated in High
MAPK8IP2	0.30020095	1.19980647	3.69970512	0.00024002	0.00135833	-1.4300017	3.619759189	up-regulated in Low
ARSA	-0.2993856	4.32380298	-4.349474	1.66E-05	0.00012294	1.10491393	4.780526984	up-regulated in High
RABL2B	-0.2275663	1.93924435	-5.0473926	6.30E-07	6.24E-06	4.24735125	6.200611677	up-regulated in High
PPP2R3B	0.18821393	1.26552897	4.09905537	4.85E-05	0.00032548	0.08261648	4.314535935	up-regulated in Low
CRLF2	-0.1369452	0.47402125	-2.7260959	0.0066358	0.02460589	-4.4966993	2.178106924	up-regulated in High
CSF2RA	-0.3813583	2.31131166	-4.7106884	3.21E-06	2.75E-05	2.67801543	5.493389163	up-regulated in High
IL3RA	-0.328733	2.77800004	-5.3848583	1.12E-07	1.28E-06	5.91795004	6.950143263	up-regulated in High
SLC25A6	0.13188687	7.7572299	2.22078217	0.02681709	0.07910723	-5.7327109	1.571588383	up-regulated in Low
ASMTL	-0.2465969	3.12723833	-4.9433083	1.05E-06	9.99E-06	3.7517363	5.977626233	up-regulated in High
P2RY8	-0.3678854	1.50245993	-5.7575758	1.50E-08	1.99E-07	7.87430175	7.82447443	up-regulated in High
DHRSX	0.17532314	3.05920541	3.16352181	0.00165442	0.00741	-3.2292546	2.781353358	up-regulated in Low
ZBED1	0.12359996	4.15639524	2.21024637	0.02754425	0.08085602	-5.7558532	1.559969022	up-regulated in Low
CD99	0.22683919	5.66678825	3.51237646	0.00048474	0.00251959	-2.0890503	3.314489777	up-regulated in Low
ARSD	-0.4974801	4.05941856	-6.2561322	8.54E-10	1.40E-08	10.6688744	9.06853365	up-regulated in High
ARSE	-0.390592	2.78494207	-2.5874794	0.00995167	0.03468173	-4.8602527	2.002104223	up-regulated in High
PRKX	0.14000563	2.2238576	2.6095896	0.00933923	0.03286459	-4.8034999	2.029689166	up-regulated in Low
NLGN4X	0.16719285	0.33801978	3.36661662	0.00082011	0.00399331	-2.5793055	3.086127332	up-regulated in Low
STS	-0.5353645	2.73553385	-6.7230248	4.91E-11	9.78E-10	13.4645717	10.3087177	up-regulated in High
PNPLA4	-0.120822	2.55822331	-2.1318182	0.03351302	0.09472454	-5.9247301	1.474786426	up-regulated in High
KAL1	-0.876927	2.19363951	-10.245044	1.80E-22	1.56E-20	39.4675678	21.74384939	up-regulated in High
TBL1X	0.30261069	2.81588443	4.58643926	5.72E-06	4.65E-05	2.1238928	5.242792096	up-regulated in Low
GPR143	-0.1603302	1.2887506	-2.2290549	0.02625783	0.07773633	-5.714464	1.580741152	up-regulated in High
SHROOM2	-0.1436941	1.53967303	-2.6304801	0.00879179	0.03122042	-4.7494463	2.055922738	up-regulated in High

WWC3	-0.3954094	3.50955326	-6.001566	3.78E-09	5.59E-08	9.21689933	8.422787723	up-regulated in High
MID1	0.14120098	1.84563345	2.16347373	0.03098204	0.08899238	-5.8572877	1.508890062	up-regulated in Low
HCCS	0.3115575	3.41740393	8.27428336	1.20E-15	4.56E-14	23.9139564	14.91953078	up-regulated in Low
ARHGAP6	-0.2720097	0.58736708	-8.960994	6.55E-18	3.31E-16	29.0655492	17.18351548	up-regulated in High
PRPS2	0.3448944	4.32826097	6.36844784	4.36E-10	7.50E-09	11.3258654	9.360326175	up-regulated in Low
TLR7	-0.438181	1.11442246	-8.1333166	3.38E-15	1.20E-13	22.8946016	14.47097878	up-regulated in High
TLR8	-0.2145591	1.27177293	-3.0998348	0.00204643	0.00891411	-3.4250745	2.689003925	up-regulated in High
TMSB4X	-0.3595107	9.94905359	-5.0660496	5.74E-07	5.73E-06	4.33717485	6.240991904	up-regulated in High
EGFL6	-0.1957403	2.60117378	-2.4261398	0.01561626	0.05061544	-5.2601306	1.806422968	up-regulated in High
TCEANC	-0.1262569	0.72138529	-5.9715179	4.49E-09	6.54E-08	9.04894437	8.348008027	up-regulated in High
OFD1	-0.1560731	3.05305143	-3.0070777	0.00277169	0.01158887	-3.7034196	2.557256034	up-regulated in High
GPM6B	-0.187958	1.22765862	-3.1225505	0.00189773	0.00834734	-3.3556691	2.721766252	up-regulated in High
GEMIN8	-0.192316	2.80123438	-4.5942855	5.52E-06	4.50E-05	2.15848335	5.25845049	up-regulated in High
FANCB	0.35913561	0.49537946	14.9866346	3.82E-42	1.29E-39	84.528491	41.41737413	up-regulated in Low
PIGA	-0.2316564	3.00223669	-3.631938	0.0003106	0.00170202	-1.6721647	3.507799199	up-regulated in High
FIGF	-0.9285679	1.21211405	-9.8358481	5.66E-21	4.19E-19	36.0499707	20.24687174	up-regulated in High
PIR	0.6042237	2.80264881	5.99016305	4.03E-09	5.93E-08	9.15307608	8.394373443	up-regulated in Low
BMX	-0.1965218	0.44521987	-3.5711986	0.00039001	0.00207923	-1.885606	3.408920601	up-regulated in High
ACE2	-0.4216585	1.33029265	-4.2418094	2.65E-05	0.00018768	0.65848103	4.577331986	up-regulated in High
TMEM27	-0.7480322	1.71390924	-6.5140327	1.80E-10	3.29E-09	12.1921956	9.744753126	up-regulated in High
CA5B	-0.183082	0.8807716	-6.6576878	7.40E-11	1.43E-09	13.0631782	10.13088322	up-regulated in High
ZRSR2	-0.2534111	2.81360616	-5.7042753	2.01E-08	2.61E-07	7.58748068	7.696486685	up-regulated in High
AP1S2	-0.2192548	2.2344156	-3.5645903	0.00039972	0.00212611	-1.9086219	3.398246746	up-regulated in High
SYAP1	0.1224343	3.91970646	2.67721829	0.00766983	0.02778029	-4.6269955	2.115214407	up-regulated in Low
RBBP7	0.38077916	3.964729	9.83144972	5.87E-21	4.34E-19	36.0137405	20.23099596	up-regulated in Low
REPS2	-0.4160258	1.39561885	-7.9403775	1.36E-14	4.46E-13	21.5212069	13.86628894	up-regulated in High
NHS	-0.2485356	1.26451888	-3.667195	0.00027175	0.00151413	-1.5467043	3.565832508	up-regulated in High
RAI2	-0.8053017	2.09797078	-12.97002	2.51E-33	5.00E-31	64.3102027	32.60069432	up-regulated in High
SCML2	0.41712116	0.49099715	10.0518918	9.28E-22	7.44E-20	37.842887	21.0323468	up-regulated in Low
CDKL5	-0.2434963	1.15085153	-6.3894246	3.84E-10	6.67E-09	11.4496696	9.41528638	up-regulated in High
RS1	-0.1145022	0.12953376	-5.5407547	4.90E-08	5.97E-07	6.72217822	7.30996564	up-regulated in High
GPR64	-0.1449398	0.44267779	-3.4543156	0.00059901	0.00303021	-2.2867078	3.22256282	up-regulated in High
PDHA1	0.28719441	3.76179499	7.16141966	2.90E-12	6.90E-11	16.2413351	11.53719006	up-regulated in Low
CXorf23	-0.2321126	1.44771583	-6.3282696	5.55E-10	9.38E-09	11.0897003	9.255463602	up-regulated in High
RPS6KA3	-0.359727	3.81656098	-6.3512203	4.84E-10	8.25E-09	11.2244477	9.315298032	up-regulated in High
CNKSR2	-0.1134291	0.27528167	-4.2852046	2.19E-05	0.00015858	0.83717042	4.658716033	up-regulated in High
SMPX	-0.1118294	0.34574453	-2.2754438	0.02330544	0.07048249	-5.6109136	1.632542628	up-regulated in High
MBTPS2	0.15928854	2.02680994	4.13221038	4.22E-05	0.00028683	0.21472336	4.374896116	up-regulated in Low
SMS	0.56742372	5.18096273	10.4185549	4.07E-23	3.79E-21	40.9441396	22.39028351	up-regulated in Low
DDX53	0.10697159	0.06951698	4.62443491	4.80E-06	3.96E-05	2.29190341	5.318827566	up-regulated in Low
PRDX4	0.38290351	5.93717646	5.46215926	7.46E-08	8.80E-07	6.31418273	7.127497052	up-regulated in Low
ACOT9	0.12531982	3.13869322	3.36913579	0.00081282	0.00396277	-2.5710008	3.090005863	up-regulated in Low
SAT1	-0.1582308	7.62005054	-2.2311765	0.02611604	0.07741863	-5.7097737	1.583092649	up-regulated in High
APOO	0.5033499	3.45134972	10.1959573	2.74E-22	2.31E-20	39.0527637	21.56221594	up-regulated in Low
KLHL15	0.10564751	1.45024656	3.1331411	0.00183186	0.00809727	-3.3231443	2.737107711	up-regulated in Low
EIF2S3	0.34658491	5.84707345	7.3596995	7.72E-13	1.97E-11	17.5440562	12.11259675	up-regulated in Low
PDK3	0.23445544	2.11345046	3.83479106	0.00014188	0.00085188	-0.9346589	3.848091316	up-regulated in Low
POLA1	0.30914843	1.93908796	7.47855114	3.44E-13	9.23E-12	18.3386105	12.46329164	up-regulated in Low
ARX	-0.3351431	0.80815159	-3.6067385	0.0003415	0.00185017	-1.7611315	3.466607753	up-regulated in High
MAGEB2	0.50804054	0.33219587	5.46093458	7.50E-08	8.85E-07	6.30786616	7.124670901	up-regulated in Low
NR0B1	0.72032886	0.62413856	5.70217132	2.03E-08	2.64E-07	7.5762064	7.691454482	up-regulated in Low
CXorf21	-0.473559	1.57304194	-8.0724095	5.26E-15	1.82E-13	22.458312	14.27893011	up-regulated in High
DMD	-0.166417	0.85641399	-3.4565367	0.00059422	0.00300958	-2.279204	3.226056098	up-regulated in High
TMEM47	-0.3674	2.34906645	-5.0801313	5.35E-07	5.37E-06	4.40516851	6.271551984	up-regulated in High
LANCL3	-0.1597322	0.24669581	-5.9499847	5.07E-09	7.33E-08	8.92903276	8.294607641	up-regulated in High
CYBB	-0.5388508	4.30812901	-4.7490505	2.68E-06	2.33E-05	2.85183841	5.571896826	up-regulated in High
SRPX	-0.3931735	2.09076538	-4.5094848	8.12E-06	6.41E-05	1.78751736	5.090411444	up-regulated in High
TSPAN7	-0.4831875	2.49593112	-3.7877919	0.00017066	0.00100355	-1.1089009	3.767872317	up-regulated in High
MID1IP1	-0.2662823	4.22285592	-3.8178258	0.00015169	0.00090394	-0.9977885	3.819039151	up-regulated in High
MED14	0.13310048	2.9929876	3.00625003	0.0027791	0.01161514	-3.7058666	2.556095167	up-regulated in Low
GPR34	-0.6394285	2.13825198	-7.912064	1.67E-14	5.38E-13	21.3218058	13.77845987	up-regulated in High
GPR82	-0.1620146	0.44387459	-5.5095762	5.79E-08	6.97E-07	6.55971119	7.237322961	up-regulated in High
MAOA	-0.7499691	4.19054894	-6.3115916	6.14E-10	1.03E-08	10.9920408	9.212092025	up-regulated in High
MAOB	-0.7245237	2.58325308	-7.6878265	8.11E-14	2.39E-12	19.7622235	13.09119057	up-regulated in High
EFHC2	-0.3797042	0.9703228	-5.2989342	1.76E-07	1.93E-06	5.48341685	6.755471558	up-regulated in High
FUNDC1	0.10624938	3.64416145	2.22267222	0.02668841	0.07878736	-5.7285479	1.573677236	up-regulated in Low
KDM6A	-0.1722852	2.47570691	-3.656521	0.000283	0.00156904	-1.5848084	3.548213641	up-regulated in High
CXorf36	-0.2698839	1.46678489	-5.3552572	1.31E-07	1.47E-06	5.76754935	6.882784645	up-regulated in High
CHST7	-0.2091416	1.40705836	-4.8972771	1.32E-06	1.23E-05	3.53553455	5.88025306	up-regulated in High
JADE3	0.21472806	2.15955275	4.77897744	2.33E-06	2.05E-05	2.9883344	5.633513095	up-regulated in Low
RGN	-0.4804337	1.49655836	-6.7292135	4.72E-11	9.44E-10	13.5027604	10.32563333	up-regulated in High

NDUFB11	0.27791458	5.88832746	5.40838866	9.91E-08	1.14E-06	6.03803213	7.003907661	up-regulated in Low
RBM10	0.19324354	4.01962883	3.51081687	0.00048752	0.00253316	-2.0944006	3.31200386	up-regulated in Low
UBA1	0.14684953	5.79004404	3.21549995	0.00138719	0.00636043	-3.0666024	2.857865156	up-regulated in Low
CDK16	0.37985465	4.16201768	8.01893301	7.74E-15	2.61E-13	22.0773245	14.11119114	up-regulated in Low
ARAF	-0.1376339	4.61519381	-3.2971165	0.00104695	0.0049565	-2.8060781	2.980072372	up-regulated in High
CFP	-0.2401825	0.7807716	-5.4328305	8.71E-08	1.01E-06	6.16325777	7.059960393	up-regulated in High
ELK1	0.29888297	3.55800198	7.99506781	9.19E-15	3.07E-13	21.9079289	14.03660032	up-regulated in Low
UXT	0.17490811	5.28165419	3.6788424	0.00025995	0.00145646	-1.5050054	3.585107079	up-regulated in Low
SLC38A5	-0.172739	2.26094207	-2.0165433	0.04428212	0.11862268	-6.1620689	1.353771641	up-regulated in High
FTSJ1	0.30125425	4.04647117	6.91979897	1.40E-11	3.03E-10	14.6930724	10.85258093	up-regulated in Low
PORCN	0.11121439	2.26821854	2.19128421	0.02889603	0.08400897	-5.7972323	1.539161898	up-regulated in Low
EBP	0.41363784	4.36638922	6.80664083	2.90E-11	5.98E-10	13.9830159	10.53830972	up-regulated in Low
WDR13	-0.1382499	3.4943637	-2.7583282	0.00602452	0.02268207	-4.4095237	2.220077742	up-regulated in High
WAS	-0.4129643	2.8967943	-5.5549555	4.54E-08	5.57E-07	6.79644518	7.343164411	up-regulated in High
SUV39H1	0.57604403	2.08587066	13.4011083	3.64E-35	8.15E-33	68.5220748	34.43842083	up-regulated in Low
GLOD5	-0.4460465	1.12086227	-8.1192788	3.74E-15	1.32E-13	22.7938238	14.42662137	up-regulated in High
PCSK1N	-0.3812819	2.36025926	-2.4243107	0.01569419	0.05083136	-5.2645201	1.804260998	up-regulated in High
TIMM17B	0.34571296	4.24407899	6.03423252	3.13E-09	4.69E-08	9.40031587	8.50443066	up-regulated in Low
PQBP1	0.12209429	4.8644558	2.57841578	0.01021292	0.03542458	-4.8833814	1.990849978	up-regulated in Low
SLC35A2	0.27220835	4.28826504	4.81249755	1.98E-06	1.77E-05	3.14214659	5.702912719	up-regulated in Low
PIM2	-0.2855485	4.52730379	-2.9455585	0.00337536	0.01376987	-3.8835289	2.471679392	up-regulated in High
GPKOW	0.19245705	3.94819754	4.7122685	3.19E-06	2.73E-05	2.68514953	5.49661223	up-regulated in Low
PRICKLE3	0.13887994	2.34724965	3.1027699	0.00202663	0.00884106	-3.4161342	2.693226082	up-regulated in Low
CACNA1F	-0.1317489	0.35489433	-3.9301875	9.69E-05	0.00060683	-0.5747728	4.013461811	up-regulated in High
FOXP3	-0.156806	1.61792116	-2.4743992	0.01367977	0.04536638	-5.1431513	1.863921351	up-regulated in High
GAGE2A	0.2230965	0.14740049	3.34406269	0.00088816	0.0042864	-2.6533925	3.051510226	up-regulated in Low
PAGE1	0.19010278	0.24076633	2.37848564	0.01776192	0.05631328	-5.3734345	1.750510015	up-regulated in Low
USP27X	-0.4421286	2.15390994	-5.4421892	8.29E-08	9.70E-07	6.21133938	7.081478481	up-regulated in High
SHROOM4	-0.7551575	1.60028428	-12.377466	7.54E-31	1.31E-28	58.6333852	30.12269283	up-regulated in High
NUDT11	0.14723525	0.43747874	3.01884748	0.00266813	0.01122042	-3.6685521	2.573792288	up-regulated in Low
GSPT2	-0.3413521	2.09887685	-4.4625276	1.00E-05	7.77E-05	1.58483857	4.998495913	up-regulated in High
TSPYL2	-0.2570808	2.74001562	-4.1504209	3.91E-05	0.0002673	0.28770633	4.408223159	up-regulated in High
IQSEC2	-0.1820145	2.11010906	-3.8586207	0.0001291	0.00078329	-0.8455409	3.889080622	up-regulated in High
SMC1A	0.33330223	3.50023318	5.94350314	5.27E-09	7.57E-08	8.89301253	8.27856484	up-regulated in Low
RIBC1	-0.2614168	0.95159586	-4.9267073	1.14E-06	1.08E-05	3.67355253	5.942420996	up-regulated in High
HSD17B10	0.3932285	5.76996069	8.73361537	3.80E-17	1.73E-15	27.3263179	16.41966658	up-regulated in Low
HUWE1	0.22623554	4.12793863	3.87249654	0.00012217	0.00074542	-0.7934084	3.913046616	up-regulated in Low
PHF8	0.2124376	2.6607214	3.7974508	0.00016433	0.00097003	-1.0732574	3.78429066	up-regulated in Low
WNK3	0.1569664	0.29878139	4.82380908	1.88E-06	1.69E-05	3.19427226	5.726423785	up-regulated in Low
TSR2	0.13805825	4.90974994	3.30111308	0.00103247	0.00489668	-2.7931603	2.98612141	up-regulated in Low
FGD1	0.4502178	2.07939217	7.61272324	1.37E-13	3.89E-12	19.2477516	12.8643408	up-regulated in Low
GNL3L	0.19496726	2.35976535	3.38020776	0.00078148	0.00382789	-2.5344304	3.10708084	up-regulated in Low
APEX2	0.24724918	3.57664092	6.34094298	5.15E-10	8.74E-09	11.1640564	9.288482518	up-regulated in Low
MAGEH1	-0.2665617	3.78996897	-3.9621124	8.52E-05	0.00053965	-0.4524795	4.069564524	up-regulated in High
USP51	-0.1720735	0.88237266	-4.0432446	6.11E-05	0.00040012	-0.1375154	4.213854191	up-regulated in High
KLF8	-0.3137746	0.96246205	-6.760668	3.87E-11	7.85E-10	13.6973118	10.41180003	up-regulated in High
SPIN4	0.48631892	1.79251255	9.00170409	4.77E-18	2.45E-16	29.3803541	17.32172517	up-regulated in Low
ARHGEF9	-0.2937483	1.90568807	-7.0044835	8.12E-12	1.82E-10	15.2307638	11.09043462	up-regulated in High
LAS1L	0.23736742	3.15626813	6.65713904	7.42E-11	1.44E-09	13.059821	10.12939554	up-regulated in Low
MSN	-0.4342846	6.65607384	-6.0230383	3.34E-09	4.99E-08	9.33736522	8.476412294	up-regulated in High
VSIG4	-0.451954	4.06925858	-3.7594674	0.00019058	0.0011073	-1.2129299	3.719928189	up-regulated in High
HEPH	-0.2837283	1.7033146	-4.0423511	6.13E-05	0.00040143	-0.1410168	4.212251697	up-regulated in High
EDA2R	-0.3927729	0.82539772	-6.3291963	5.52E-10	9.34E-09	11.0951329	9.257876123	up-regulated in High
AR	-0.2261955	0.6746375	-4.1295135	4.27E-05	0.00028972	0.20394042	4.369971035	up-regulated in High
OPHN1	-0.2901849	1.48203066	-5.5109936	5.75E-08	6.92E-07	6.56707925	7.24061789	up-regulated in High
YIPF6	0.14387195	3.39617616	3.64876344	0.00029145	0.00161069	-1.6124355	3.535435592	up-regulated in Low
STARD8	-0.2942076	1.51085	-6.0017112	3.77E-09	5.58E-08	9.21771221	8.4231496	up-regulated in High
EFNB1	-0.1684768	3.15056039	-1.9922244	0.04689464	0.12407819	-6.2104821	1.328876794	up-regulated in High
EDA	-0.2816857	0.70733046	-5.3221177	1.56E-07	1.73E-06	5.60004652	6.807740392	up-regulated in High
PDZD11	0.31327026	4.88796911	6.38683296	3.90E-10	6.77E-09	11.4343551	9.408488259	up-regulated in Low
KIF4A	1.38862956	2.18242786	19.3187523	2.11E-62	3.63E-59	131.052241	61.67482598	up-regulated in Low
SNX12	0.14573949	4.61311142	4.0808149	5.23E-05	0.00034851	0.01036094	4.281502762	up-regulated in Low
FOXO4	-0.2092395	2.59796729	-4.0601902	5.70E-05	0.00037654	-0.0709761	4.24430109	up-regulated in High
CXorf65	-0.1262178	0.4744144	-3.5053283	0.00049744	0.00257549	-2.1132117	3.303262588	up-regulated in High
IL2RG	-0.3592039	3.9290275	-3.6143016	0.00033194	0.00180387	-1.7344917	3.478945372	up-regulated in High
GJB1	-0.6719974	2.38860554	-4.0129947	6.92E-05	0.00044881	-0.2556487	4.159768812	up-regulated in High
NONO	0.25513509	6.14552251	5.93452768	5.54E-09	7.93E-08	8.843189	8.256372847	up-regulated in Low
OGT	-0.2612183	4.15888787	-3.4374411	0.00063667	0.00319688	-2.3435651	3.196084717	up-regulated in High
RGAG4	-0.4361674	1.49040098	-6.7922602	3.17E-11	6.51E-10	13.8934726	10.49866357	up-regulated in High
NHSL2	-0.1682368	0.42095316	-6.2534766	8.68E-10	1.42E-08	10.6534608	9.06168515	up-regulated in High
PIN4	0.11695458	2.36626318	3.02204096	0.00264066	0.01111929	-3.659069	2.578288115	up-regulated in Low

ERCC6L	0.73360959	0.98226014	18.0852466	1.64E-56	1.45E-53	117.521263	55.78602782	up-regulated in Low
PHKA1	0.39726892	2.27381686	7.5171826	2.64E-13	7.21E-12	18.5990582	12.57820638	up-regulated in Low
NAP1L2	-0.1681321	0.75950189	-3.0004703	0.00283142	0.01181012	-3.7229363	2.547995811	up-regulated in High
CHIC1	-0.1957731	1.31909598	-5.3148304	1.62E-07	1.79E-06	5.56333713	6.791290175	up-regulated in High
SLC16A2	-0.3470185	2.10108442	-4.7329866	2.89E-06	2.50E-05	2.77889417	5.538957069	up-regulated in High
RLIM	0.10824322	3.06384491	2.80995178	0.00515086	0.01983532	-4.2678333	2.288120386	up-regulated in Low
KIAA2022	-0.1471188	0.26634301	-4.8179866	1.93E-06	1.73E-05	3.16742725	5.714315948	up-regulated in High
ABCB7	0.14686956	3.03314109	4.02078327	6.70E-05	0.00043555	-0.2253117	4.173661825	up-regulated in Low
UPRT	-0.1885518	2.34129087	-5.2685474	2.05E-07	2.23E-06	5.33123884	6.687250365	up-regulated in High
ZDHHC15	-0.1819972	0.46201932	-5.3247377	1.54E-07	1.71E-06	5.6132555	6.81365927	up-regulated in High
MAGEE2	-0.1119418	0.16877362	-4.3238167	1.85E-05	0.000136	0.99758346	4.731715258	up-regulated in High
PBDC1	0.11712781	4.42420215	2.96802712	0.00314219	0.01292994	-3.8181641	2.502767882	up-regulated in Low
MAGEE1	-0.2131088	1.1066736	-4.8292173	1.83E-06	1.65E-05	3.21923367	5.737681135	up-regulated in High
ATRX	-0.1142817	2.34097264	-2.5461712	0.01119272	0.03829755	-4.9650232	1.951064325	up-regulated in High
COX7B	0.33088237	4.63746327	5.70969943	1.95E-08	2.54E-07	7.61656207	7.709466516	up-regulated in Low
ATP7A	-0.2038723	1.97743918	-4.5293198	7.42E-06	5.91E-05	1.873717	5.129480022	up-regulated in High
PGK1	0.5134176	6.76405272	7.5669281	1.88E-13	5.22E-12	18.9360054	12.72684598	up-regulated in Low
CYSLTR1	-0.539581	1.12702666	-9.111683	2.01E-18	1.09E-16	30.2359158	17.69727309	up-regulated in High
P2RY10	-0.2552887	1.35039806	-3.6464218	0.00029405	0.00162304	-1.620764	3.531582902	up-regulated in High
GPR174	-0.1616859	0.82411743	-2.7988951	0.00532768	0.02042598	-4.2983948	2.273461851	up-regulated in High
ITM2A	-0.6124258	3.06818984	-7.3286608	9.51E-13	2.41E-11	17.3382356	12.02172227	up-regulated in High
BRWD3	0.11841591	1.52573548	2.88288579	0.00411172	0.01633024	-4.0633176	2.385976624	up-regulated in Low
SH3BGRL	-0.4289734	5.45965508	-7.6536795	1.03E-13	3.00E-12	19.5278164	12.9878404	up-regulated in High
RPS6KA6	0.11438203	0.69968302	2.45648236	0.01437237	0.04724732	-5.1868436	1.842471569	up-regulated in Low
ZNF711	0.2078494	0.87682039	3.52963268	0.00045493	0.00238266	-2.0297003	3.342056842	up-regulated in Low
NAP1L3	-0.2530847	0.78196897	-5.8340956	9.77E-09	1.34E-07	8.29014524	8.009926072	up-regulated in High
FAM133A	0.6302363	0.62668015	5.8901261	7.13E-09	1.00E-07	8.59767361	8.146992765	up-regulated in Low
DIAPH2	-0.155589	1.7772125	-3.3546253	0.00085566	0.00414774	-2.6187548	3.067698375	up-regulated in High
CSTF2	0.40231472	3.48607087	9.69295166	1.85E-20	1.27E-18	34.8785103	19.73347625	up-regulated in Low
NOX1	-0.1344799	0.77715522	-2.7738583	0.00574856	0.02179064	-4.3671662	2.240440841	up-regulated in High
XKRX	-0.7829327	1.98740492	-6.9560375	1.11E-11	2.43E-10	14.9225057	10.95408648	up-regulated in High
CENPI	0.75447151	1.08961227	16.510166	4.11E-49	2.03E-46	100.526102	48.38662761	up-regulated in Low
DRP2	0.1701375	0.18399579	6.83570714	2.41E-11	5.03E-10	14.1644803	10.61864487	up-regulated in Low
TIMM8A	0.47039016	1.97409554	12.9636296	2.67E-33	5.28E-31	64.2482691	32.57366655	up-regulated in Low
BTK	-0.5484384	1.88943242	-8.424924	3.93E-16	1.60E-14	25.0178857	15.40506783	up-regulated in High
RPL36A	0.19780019	4.16895062	3.26895544	0.00115446	0.00540152	-2.8966765	2.937620471	up-regulated in Low
GLA	0.54955167	3.88324687	9.61272849	3.57E-20	2.37E-18	34.2259598	19.44742927	up-regulated in Low
ARMCX4	-0.1852912	0.80541081	-5.6383339	2.89E-08	3.65E-07	7.23587464	7.539502226	up-regulated in High
ARMCX1	-0.2318412	2.61622533	-3.4115461	0.00069878	0.0034717	-2.4303001	3.155661754	up-regulated in High
ARMCX6	-0.1150973	2.56004802	-2.5379253	0.01145636	0.03907598	-4.9857407	1.940953288	up-regulated in High
ARMCX3	-0.1709289	3.95213464	-3.5080234	0.00049255	0.00255584	-2.1039783	3.307553438	up-regulated in High
ZMAT1	-0.5580693	1.27035453	-8.3090489	9.31E-16	3.59E-14	24.1673929	15.03101946	up-regulated in High
TCEAL2	-0.2701997	0.524655	-4.367862	1.53E-05	0.00011425	1.18219707	4.815658667	up-regulated in High
BEX5	-0.3977765	1.74669329	-4.3723233	1.50E-05	0.00011216	1.20099338	4.824201281	up-regulated in High
TMSB15A	0.26799814	0.73167471	3.67909375	0.0002597	0.00145535	-1.5041042	3.585523573	up-regulated in Low
GPRASP1	-0.3396989	0.92483125	-7.6744565	8.90E-14	2.61E-12	19.6703455	13.05068323	up-regulated in High
GPRASP2	-0.206616	1.66383873	-4.5552065	6.60E-06	5.30E-05	1.98674002	5.18068535	up-regulated in High
BEX1	0.37855971	0.63662541	3.34570467	0.00088303	0.00426496	-2.6480149	3.054023926	up-regulated in Low
NXF3	-0.2223445	0.29996243	-4.6548504	4.17E-06	3.49E-05	2.42731184	5.380073676	up-regulated in High
BEX4	-0.4922898	4.21499441	-5.5709561	4.16E-08	5.14E-07	6.88032592	7.380655016	up-regulated in High
TCEAL8	-0.1897678	5.22917848	-3.5067794	0.0004948	0.00256547	-2.1082409	3.305572608	up-regulated in High
BEX2	-0.2744833	3.05776717	-2.2372277	0.02571528	0.07646353	-5.696372	1.5898088	up-regulated in High
TCEAL7	-0.2442906	1.04828878	-4.939632	1.07E-06	1.02E-05	3.73440216	5.969821572	up-regulated in High
NGFRAP1	0.19539654	5.82907652	2.12663251	0.03394411	0.09572984	-5.9356852	1.469235569	up-regulated in Low
TCEAL4	-0.1683337	4.62127553	-3.3062144	0.00101426	0.00482401	-2.7766499	2.99385135	up-regulated in High
TCEAL3	-0.2864682	3.13736494	-5.0348591	6.71E-07	6.60E-06	4.18717692	6.173554761	up-regulated in High
TCEAL1	-0.2185964	3.37989	-4.3033112	2.03E-05	0.00014755	0.91222778	4.69287946	up-regulated in High
MORF4L2	0.35948291	6.45389029	8.37604046	5.66E-16	2.26E-14	24.6580132	15.2468119	up-regulated in Low
FAM199X	0.14788492	3.0650325	3.85819263	0.00012932	0.00078446	-0.8471462	3.888342514	up-regulated in Low
MUM1L1	-0.2546305	0.59713303	-3.7736902	0.00018032	0.00105323	-1.160785	3.743965133	up-regulated in High
RNF128	-0.4243677	2.49228201	-3.1279001	0.00186419	0.00822087	-3.3392532	2.729510331	up-regulated in High
TBC1D8B	-0.2214939	1.38235892	-5.7795222	1.33E-08	1.78E-07	7.99307819	7.877457587	up-regulated in High
MORC4	0.10779227	2.50140381	2.11308613	0.03509273	0.09828557	-5.9641792	1.45478284	up-regulated in Low
CLDN2	-1.2121369	2.02662385	-6.2828095	7.29E-10	1.20E-08	10.8240217	9.137460966	up-regulated in High
NUP62CL	0.4016068	1.39950939	6.79381161	3.14E-11	6.45E-10	13.9031254	10.5029376	up-regulated in Low
PIH1D3	-0.2611523	0.31296484	-5.2476449	2.29E-07	2.46E-06	5.22701226	6.64051178	up-regulated in High
PRPS1	0.2665472	4.28159817	5.79648477	1.21E-08	1.63E-07	8.08515215	7.918522224	up-regulated in Low
TSC22D3	-0.9765427	5.27388907	-10.814507	1.29E-24	1.36E-22	44.3724186	23.89046921	up-regulated in High
MID2	-0.2426186	1.70618856	-4.7234338	3.02E-06	2.60E-05	2.73562337	5.519413177	up-regulated in High
VSIG1	-0.9270717	1.96817109	-4.9873507	8.48E-07	8.20E-06	3.96031215	6.071506184	up-regulated in High
PSMD10	0.16837454	4.38427155	3.83267209	0.00014307	0.00085816	-0.9425582	3.844456777	up-regulated in Low

AMMECR1	0.22104099	2.10138975	5.31227681	1.64E-07	1.81E-06	5.55048422	6.785530205	up-regulated in Low
CHRDL1	-1.1075812	2.06107399	-10.277108	1.37E-22	1.20E-20	39.7392214	21.86279206	up-regulated in High
CAPN6	-0.2668342	0.87687709	-2.2358342	0.0258071	0.07668188	-5.6994615	1.58826088	up-regulated in High
AMOT	-0.642803	1.79861794	-6.6226273	9.20E-11	1.76E-09	12.8491375	10.03602518	up-regulated in High
HTR2C	0.15231431	0.12525752	4.46684391	9.84E-06	7.64E-05	1.60338712	5.006910987	up-regulated in Low
IL13RA2	-0.2353629	0.73306319	-2.8021043	0.0052758	0.02024569	-4.2895364	2.277711698	up-regulated in High
PLS3	-0.4493703	5.42779412	-5.3047635	1.70E-07	1.88E-06	5.5126999	6.768596372	up-regulated in High
AGTR2	-0.4773718	0.89210552	-4.4637339	9.98E-06	7.73E-05	1.5900207	5.000846996	up-regulated in High
CT83	0.68020379	1.81007298	3.73372392	0.00021057	0.00121078	-1.3068399	3.676614257	up-regulated in Low
DOCK11	-0.2857872	2.09204219	-4.2291424	2.79E-05	0.0001971	0.60664057	4.553707523	up-regulated in High
IL13RA1	-0.1367055	5.31049671	-2.5057756	0.01253783	0.04214915	-5.0658902	1.901777484	up-regulated in High
SLC25A43	0.11351844	3.33455563	2.14653166	0.03231536	0.09197607	-5.8935043	1.490590967	up-regulated in Low
SLC25A5	0.29256462	7.63940567	5.63006306	3.02E-08	3.81E-07	7.1920273	7.519918368	up-regulated in Low
CXorf56	0.12579777	3.40072141	3.40128378	0.00072492	0.00358601	-2.4645005	3.139712076	up-regulated in Low
NKRF	0.25181343	2.07163348	8.12818655	3.51E-15	1.24E-13	22.8577574	14.45476206	up-regulated in Low
6-Sep	-0.1679067	3.02454359	-2.8161067	0.00505475	0.01951154	-4.2507702	2.296300443	up-regulated in High
SOWAHD	-0.1785116	0.95823896	-4.0860826	5.12E-05	0.00034165	0.03119673	4.291029714	up-regulated in High
UPF3B	0.3022161	2.83682698	5.89529158	6.92E-09	9.74E-08	8.62615348	8.159683009	up-regulated in Low
NDUFA1	0.19367879	6.46419199	3.73979113	0.00020568	0.00118481	-1.2847622	3.686800018	up-regulated in Low
AKAP14	-0.344088	0.47567892	-5.1500651	3.76E-07	3.89E-06	4.74536186	6.424371542	up-regulated in High
TMEM255A	-0.1759903	0.81231509	-3.1066969	0.00200041	0.00873649	-3.4041595	2.698880315	up-regulated in High
CUL4B	0.16163686	3.5782786	4.21138193	3.02E-05	0.00021124	0.53419795	4.520683777	up-regulated in Low
MCTS1	0.31720491	3.76975178	7.57797501	1.74E-13	4.86E-12	19.0110699	12.7599554	up-regulated in Low
GLUD2	0.11814272	0.81089146	2.6389283	0.0085787	0.03056223	-4.7274679	2.066578651	up-regulated in Low
SH2D1A	-0.2009127	1.3221777	-2.8043116	0.00524038	0.02013359	-4.2834378	2.280637024	up-regulated in High
DCAF12L2	0.12422963	0.19653558	3.55837201	0.00040906	0.00217005	-1.9302424	3.388217888	up-regulated in Low
PRR32	-0.1219637	0.17225902	-3.8580803	0.00012938	0.00078465	-0.8475673	3.888148884	up-regulated in High
SMARCA1	0.18946462	4.15673987	2.37586334	0.01788718	0.0566458	-5.3796056	1.747458162	up-regulated in Low
OCRL	0.23615177	3.30556579	5.54299223	4.84E-08	5.91E-07	6.73386891	7.31519192	up-regulated in Low
XPNPEP2	-0.1083596	0.33541775	-3.7615012	0.00018908	0.00109935	-1.2054849	3.72336069	up-regulated in High
SASH3	-0.548615	3.29868298	-6.5201445	1.73E-10	3.18E-09	12.2289267	9.761044105	up-regulated in High
ZDHHC9	-0.439911	5.17766278	-5.814678	1.09E-08	1.48E-07	8.18416766	7.96267581	up-regulated in High
UTP14A	0.28966368	2.93690821	8.07728195	5.08E-15	1.76E-13	22.4931215	14.2942543	up-regulated in Low
AIFM1	0.19125292	3.9803352	4.38840826	1.40E-05	0.00010498	1.26890843	4.855061457	up-regulated in Low
ZNF280C	0.1319115	1.34787323	3.73252243	0.00021154	0.00121559	-1.311208	3.674598812	up-regulated in Low
RBMX2	0.20768359	3.30498969	5.7049169	2.00E-08	2.60E-07	7.5909191	7.698021382	up-regulated in Low
ENOX2	0.14360942	2.22671303	3.74234227	0.00020366	0.00117445	-1.2754689	3.691087045	up-regulated in Low
IGSF1	0.11106545	0.3112386	2.81303767	0.00510247	0.01967103	-4.2592829	2.292219807	up-regulated in Low
STK26	0.30954111	3.24443764	4.30951161	1.97E-05	0.00014401	0.93799766	4.704606117	up-regulated in Low
RAP2C	0.10489873	3.49810503	2.41685231	0.01601554	0.05170293	-5.282385	1.795458526	up-regulated in Low
MBNL3	-0.150189	1.46306903	-2.993909	0.0028919	0.01202802	-3.7422756	2.538816718	up-regulated in High
HS6ST2	-0.6104198	2.89009616	-5.0729158	5.55E-07	5.55E-06	4.37030695	6.255883982	up-regulated in High
GPC4	-1.082994	4.37136881	-11.215048	3.61E-26	4.39E-24	47.9202304	25.44204672	up-regulated in High
GPC3	-0.5704375	2.94591524	-4.354709	1.62E-05	0.00012042	1.1268855	4.790516186	up-regulated in High
PHF6	0.28749449	2.31625936	6.5583369	1.37E-10	2.56E-09	12.4591119	9.863121442	up-regulated in Low
HPRT1	0.51646505	4.50071032	8.41178188	4.34E-16	1.76E-14	24.9209811	15.36245586	up-regulated in Low
PLAC1	0.24340387	0.45144118	4.49448881	8.69E-06	6.81E-05	1.72257835	5.060969813	up-regulated in Low
FAM122B	0.24836137	3.35424595	5.11373258	4.52E-07	4.60E-06	4.56809981	6.344759836	up-regulated in Low
SMIM10	-0.1286561	1.81600671	-2.0073528	0.04525458	0.12061346	-6.1804331	1.344337475	up-regulated in High
FAM127B	0.19614693	5.23536991	3.28495411	0.00109219	0.00514231	-2.8452976	2.961701068	up-regulated in Low
ZNF75D	-0.291559	1.91437371	-6.6692085	6.88E-11	1.34E-09	13.1337171	10.16214004	up-regulated in High
DDX26B	-0.139571	1.25840636	-3.0423841	0.00247162	0.01050753	-3.5984324	2.607018395	up-regulated in High
CT45A1	0.14962904	0.11287685	3.12857321	0.00186001	0.00820615	-3.3371858	2.730485454	up-regulated in Low
CT45A10	0.15760495	0.13868513	3.33092149	0.00093019	0.00446195	-2.6963404	3.031429139	up-regulated in Low
SAGE1	0.1240072	0.11795726	2.90133026	0.00388111	0.01553589	-4.0107941	2.411043869	up-regulated in Low
SLC9A6	-0.1303788	2.52352526	-3.0350339	0.00253151	0.01072826	-3.6203863	2.596619711	up-regulated in High
FHL1	-0.8953742	2.32674168	-9.6248501	3.23E-20	2.16E-18	34.3243211	19.49054919	up-regulated in High
HTATSF1	0.10574216	4.72558549	2.17298834	0.03025422	0.0872926	-5.836826	1.519214053	up-regulated in Low
VGLL1	-0.409574	1.03898854	-3.6115572	0.00033538	0.00182071	-1.7441646	3.474465939	up-regulated in High
CD40LG	-0.4670617	0.9327116	-8.8136363	2.06E-17	9.74E-16	27.9346923	16.68691036	up-regulated in High
ARHGEF6	-0.4960195	2.17700979	-7.9853448	9.86E-15	3.28E-13	21.8390268	14.00625848	up-regulated in High
RBMX	0.17881355	4.85314783	5.91872633	6.06E-09	8.63E-08	8.75563314	8.217370372	up-regulated in Low
SPANXB2	0.4087344	0.23485917	4.93231315	1.11E-06	1.05E-05	3.69992701	5.954298022	up-regulated in Low
LDOC1	-0.4415749	4.41296939	-3.5602877	0.00040616	0.00215624	-1.9235855	3.391305949	up-regulated in High
SPANXC	0.19664804	0.11727164	3.83210257	0.00014339	0.00085992	-0.9446806	3.843480219	up-regulated in Low
SPANXD	0.13804646	0.06696071	4.01598361	6.84E-05	0.00044378	-0.2440131	4.165097703	up-regulated in Low
MAGEC1	0.23746693	0.24877093	3.21805024	0.00137516	0.00631423	-3.0585565	2.861645562	up-regulated in Low
MAGEC2	0.35490464	0.48873438	3.15013967	0.00173053	0.00770302	-3.2707186	2.761820802	up-regulated in Low
IDS	-0.3650843	4.10902762	-4.9300758	1.12E-06	1.06E-05	3.68939731	5.949556373	up-regulated in High
IDS	-0.3650843	4.10902762	-4.9300758	1.12E-06	1.06E-05	3.68939731	5.949556373	up-regulated in High
MAGEA9	0.16415586	0.1277918	3.85252501	0.00013226	0.00080006	-0.8683869	3.878575247	up-regulated in Low

MAGEA11	0.12313425	0.13059665	2.69372378	0.00730548	0.02664908	-4.5832517	2.136351196	up-regulated in Low
MAGEA8	0.18781283	0.20193012	2.72990108	0.00656083	0.02436814	-4.4864594	2.183041293	up-regulated in Low
CXorf40B	0.18379942	2.94884929	4.81491619	1.96E-06	1.76E-05	3.15328277	5.707935977	up-regulated in Low
MTM1	-0.2380586	2.30997849	-5.5215012	5.43E-08	6.58E-07	6.62175491	7.265066847	up-regulated in High
HMGB3	0.68924828	5.69122325	5.42826418	8.92E-08	1.04E-06	6.1398247	7.049472547	up-regulated in Low
VMA21	0.21595092	3.4878361	4.96738961	9.36E-07	8.98E-06	3.86557295	6.028871104	up-regulated in Low
FATE1	0.11209805	0.34802483	4.19562284	3.22E-05	0.00022475	0.47015628	4.491479294	up-regulated in Low
MAGEA4	0.4775236	0.4496815	3.6873582	0.00025163	0.00141556	-1.4744386	3.599231686	up-regulated in Low
GABRE	-0.4207868	1.53207524	-4.1901085	3.30E-05	0.00022951	0.44779992	4.481281955	up-regulated in High
MAGEA10	0.3099215	0.36070736	3.04733244	0.00243203	0.01035582	-3.5836236	2.614030681	up-regulated in Low
GABRA3	0.34706226	0.45272336	4.64705628	4.32E-06	3.61E-05	2.39253516	5.364346875	up-regulated in Low
GABRQ	0.14011415	0.10458957	5.85788608	8.55E-09	1.18E-07	8.420409	8.067993328	up-regulated in Low
MAGEA3	1.10925579	1.03602089	6.51283007	1.81E-10	3.32E-09	12.1849717	9.741549115	up-regulated in Low
CSAG1	0.71526153	0.80559458	4.79410433	2.16E-06	1.92E-05	3.05762515	5.664781218	up-regulated in Low
MAGEA12	0.64519175	0.71394434	4.6276583	4.73E-06	3.91E-05	2.30621522	5.325302344	up-regulated in Low
MAGEA6	0.92204816	0.909149	5.78357454	1.30E-08	1.74E-07	8.01505323	7.887258945	up-regulated in Low
NSDHL	0.23560941	3.96892525	5.51928315	5.50E-08	6.66E-07	6.61020583	7.259902741	up-regulated in Low
MAGEA1	0.58999602	0.45829684	5.26631566	2.08E-07	2.26E-06	5.32009278	6.682252657	up-regulated in Low
ZNF275	-0.1290559	2.21091532	-2.7851225	0.00555562	0.02117889	-4.3362998	2.255267668	up-regulated in High
ZFP92	-0.2220997	0.56014693	-7.8225358	3.15E-14	9.75E-13	20.6949358	13.50228516	up-regulated in High
BGN	-0.4955834	7.74358418	-5.2994073	1.75E-07	1.93E-06	5.48579272	6.756536469	up-regulated in High
FAM58A	0.19274607	3.67488087	3.59197376	0.00036092	0.00193956	-1.8129856	3.442584208	up-regulated in Low
DUSP9	0.18261872	0.66269922	2.23290137	0.02600126	0.07714495	-5.7059572	1.585005649	up-regulated in Low
PNCK	0.13691604	0.32017791	2.80326654	0.00525712	0.02018463	-4.2863257	2.27925182	up-regulated in Low
SLC6A8	0.57731911	2.79273478	5.71216128	1.93E-08	2.52E-07	7.62976928	7.715361048	up-regulated in Low
ABCD1	0.21837376	2.73266047	3.83106507	0.00014398	0.00086309	-0.9485463	3.841701496	up-regulated in Low
PLXNB3	-0.4204117	1.77469328	-4.1955178	3.23E-05	0.00022482	0.46973003	4.491284882	up-regulated in High
IDH3G	0.15595958	4.52600072	3.55102015	0.00042036	0.00222173	-1.9557579	3.376379664	up-regulated in Low
PDZD4	-0.2253363	0.76483042	-3.6718294	0.000267	0.00149135	-1.5301276	3.573495616	up-regulated in High
L1CAM	0.27976048	0.43190934	4.00406944	7.18E-05	0.00046353	-0.2903449	4.143876229	up-regulated in Low
NAA10	0.3140935	3.16222153	6.42589456	3.08E-10	5.44E-09	11.6657351	9.511185235	up-regulated in Low
RENBP	-0.313	3.06279729	-3.636404	0.00030541	0.00167654	-1.6563363	3.515124312	up-regulated in High
HCFC1	0.25778507	3.77500677	4.93935291	1.07E-06	1.02E-05	3.73308645	5.969229158	up-regulated in Low
IRAK1	0.40970522	5.46298377	7.35827931	7.79E-13	1.99E-11	17.5346235	12.10843229	up-regulated in Low
FLNA	-0.4284323	6.53361438	-4.5767202	5.98E-06	4.85E-05	2.08112094	5.223427319	up-regulated in High
EMD	0.16423034	4.89685399	3.95174297	8.89E-05	0.00056052	-0.4923032	4.051300126	up-regulated in Low
GDI1	0.16442352	4.70672375	3.50498383	0.00049807	0.0025776	-2.1143914	3.302714363	up-regulated in Low
FAM50A	0.18481608	5.11333154	3.24544483	0.00125196	0.00580375	-2.9717435	2.902409276	up-regulated in Low
LAGE3	0.47140041	4.53876616	6.94874618	1.17E-11	2.54E-10	14.8762636	10.93362968	up-regulated in Low
UBL4A	0.31259846	4.63119998	6.41840288	3.23E-10	5.67E-09	11.6212659	9.491449866	up-regulated in Low
G6PD	0.86605051	5.06154268	7.51428614	2.69E-13	7.35E-12	18.5794937	12.56957478	up-regulated in Low
CTAG2	0.37802647	0.42316117	3.32138828	0.00096183	0.00459861	-2.7273955	3.016902384	up-regulated in Low
GAB3	-0.3504296	1.2243929	-7.4129941	5.38E-13	1.40E-11	17.8990853	12.26932008	up-regulated in High
DKC1	0.62156557	4.24931595	14.6171656	1.73E-40	5.33E-38	80.7328596	39.76305836	up-regulated in Low
F8	-0.3720695	1.41031583	-8.4573148	3.09E-16	1.27E-14	25.2572053	15.51029668	up-regulated in High
BRCC3	0.1398005	3.41158685	3.06249223	0.00231435	0.00991837	-3.5381111	2.635571331	up-regulated in Low
VBP1	0.39211518	4.62932349	7.90306811	1.78E-14	5.70E-13	21.2585665	13.75060329	up-regulated in Low
RAB39B	0.129954	0.55697299	3.07630571	0.00221169	0.00954076	-3.4964512	2.655274986	up-regulated in Low
CLIC2	-0.4876294	3.53953382	-5.16806	3.44E-07	3.59E-06	4.83357422	6.463975983	up-regulated in High
TMLHE	0.29434715	1.20500682	5.68232878	2.27E-08	2.91E-07	7.47006108	7.644072318	up-regulated in Low
VAMP7	0.10942755	4.25201787	2.30216669	0.02173945	0.06654963	-5.5503141	1.662751512	up-regulated in Low
PPP2R3B	0.18821393	1.26552897	4.09905537	4.85E-05	0.00032548	0.08261648	4.314535935	up-regulated in Low
CRLF2	-0.1369452	0.47402125	-2.7260959	0.0066358	0.02460589	-4.4966993	2.178106924	up-regulated in High
CSF2RA	-0.3813583	2.31131166	-4.7106884	3.21E-06	2.75E-05	2.67801543	5.493389163	up-regulated in High
IL3RA	-0.328733	2.77800004	-5.3848583	1.12E-07	1.28E-06	5.91795004	6.950143263	up-regulated in High
SLC25A6	0.13188687	7.7572299	2.22078217	0.02681709	0.07910723	-5.7327109	1.571588383	up-regulated in Low
ASMTL	-0.2465969	3.12723833	-4.9433083	1.05E-06	9.99E-06	3.7517363	5.977626233	up-regulated in High
P2RY8	-0.3678854	1.50245993	-5.7575758	1.50E-08	1.99E-07	7.87430175	7.82447443	up-regulated in High
DHRSX	0.17532314	3.05920541	3.16352181	0.00165442	0.00741	-3.2292546	2.781353358	up-regulated in Low
ZBED1	0.12359996	4.15639524	2.21024637	0.02754425	0.08085602	-5.7558532	1.559969022	up-regulated in Low
CD99	0.22683919	5.66678825	3.51237646	0.00048474	0.00251959	-2.0890503	3.314489777	up-regulated in Low
VAMP7	0.10942755	4.25201787	2.30216669	0.02173945	0.06654963	-5.5503141	1.662751512	up-regulated in Low
RPS4Y1	0.69135245	2.75176453	2.44182941	0.01496168	0.04886272	-5.2223462	1.825019577	up-regulated in Low
DDX3Y	0.30780784	1.36736998	2.09122861	0.03701621	0.10263395	-6.0097782	1.431607999	up-regulated in Low
TMSB4Y	0.16967036	0.46513617	2.74607242	0.00625068	0.02341279	-4.4427878	2.204072574	up-regulated in Low
EIF1AY	0.29201263	1.0322626	2.45827878	0.01430156	0.04705161	-5.1824768	1.844616734	up-regulated in Low
MT-ND5	-0.4328183	10.0144795	-4.1572602	3.80E-05	0.00026045	0.31519377	4.420771581	up-regulated in High
MT-ND6	-0.6118461	10.3079446	-5.2635431	2.11E-07	2.28E-06	5.30625203	6.676046509	up-regulated in High
