# Supplementary material for: Identification and Validation of a Proliferation-Associated Score Model Predicting Survival in Lung Adenocarcinomas
Source: Dis Markers. 2021 Oct 21;2021:3219594. doi: 10.1155/2021/3219594 (PMC8554523; doi:10.1155/2021/3219594)
Supplement: Supplementary 2 — Table S1: the table showed genes associated with microenvironment of the 24 immune cell subsets. Table S2: the table showed the sequences of all the siRNAs and primers used in this study. Table S3: the table showed 55 genes selected for LASSO Cox regression; all the 55 genes showed the same tendency in cell proliferation (the CERES dependency score) and survival (HR). Table S4: the table showed six genes used in the model and their LASSO coefficient after LASSO Cox regression. Table S5: the table showed the summary of genomic alterations in the two groups, including the somatic mutation numbers of each gene in high and low score groups. Table S6: the table showed the differentially expressed genes (DEGs) between high score group and low score group identified by limma. Table S7: the table showed the differentially expressed miRNAs between high score group and low score group identified by limma. Table S8: the table showed the comparison the abundance of 24 types of immune cells between the two groups by Wilcoxon test. [file 3219594.f2.zip › Table S7.pdf]

Table S7. Differentially expressed miRNAs between high-score and low-score group identified by limma.

miRNAs	logFC	AveExpr	t	P.Value	adj.P.Val	logP	Group
MIMAT0000731	0.001471	5.409728	0.015918	0.987307	0.987307	0.005548	not-significant
MIMAT0000080	-0.00099	10.92871	-0.01777	0.985828	0.987307	0.006199	not-significant
MIMAT0004774	-0.00915	5.124315	-0.09689	0.922858	0.93469	0.034865	not-significant
MIMAT0000259	0.008971	14.26073	0.09269	0.926193	0.93469	0.033299	not-significant
MIMAT0002813	0.012906	2.520396	0.110942	0.911713	0.931725	0.040142	not-significant
MIMAT0003239	0.008914	6.433101	0.107068	0.914785	0.931725	0.038681	not-significant
MIMAT0000446	0.027016	8.283671	0.201048	0.840755	0.864328	0.07533	not-significant
MIMAT0004497	0.022602	4.239901	0.257127	0.797203	0.827286	0.098431	not-significant
MIMAT0000646	0.030918	8.522664	0.296216	0.767207	0.799932	0.115087	not-significant
MIMAT0000438	-0.0265	7.683897	-0.31121	0.755793	0.791783	0.121597	not-significant
MIMAT0002872	-0.02569	2.203194	-0.31496	0.752946	0.791783	0.123236	not-significant
MIMAT0000266	0.099988	6.414042	0.337604	0.735825	0.778276	0.133225	not-significant
MIMAT0000068	0.026273	7.028657	0.370706	0.711038	0.755692	0.148107	not-significant
MIMAT0004808	-0.03598	7.869634	-0.38515	0.700316	0.74791	0.154706	not-significant
MIMAT0004584	0.04745	4.867195	0.41505	0.678311	0.728575	0.168571	not-significant
MIMAT0000085	0.023097	6.709036	0.414245	0.6789	0.728575	0.168194	not-significant
MIMAT0000456	0.026935	7.998619	0.437659	0.661851	0.717277	0.179239	not-significant
MIMAT0000253	-0.04577	14.4143	-0.46363	0.643142	0.700452	0.191693	not-significant
MIMAT0000084	0.033218	10.48389	0.469739	0.638777	0.699159	0.194651	not-significant
MIMAT0003322	-0.04187	5.368383	-0.51163	0.609173	0.670091	0.215259	not-significant
MIMAT0000415	-0.02893	8.892073	-0.52851	0.597416	0.66046	0.223723	not-significant
MIMAT0000617	0.050628	13.14462	0.536074	0.592182	0.65798	0.227545	not-significant
MIMAT0000425	-0.05548	5.977919	-0.54502	0.586018	0.654436	0.232089	not-significant
MIMAT0004784	0.067243	6.864224	0.551298	0.581713	0.652944	0.235291	not-significant
MIMAT0000432	0.061056	9.5867	0.564229	0.57289	0.646337	0.241929	not-significant
MIMAT0000264	-0.09437	11.73939	-0.56992	0.569027	0.645289	0.244867	not-significant
MIMAT0000751	-0.04325	2.435699	-0.58018	0.562097	0.640732	0.250189	not-significant
MIMAT0000450	0.104308	4.454836	0.652315	0.514543	0.589581	0.288578	not-significant
MIMAT0002809	-0.06834	9.977014	-0.657	0.51153	0.589197	0.291129	not-significant
MIMAT0004495	0.062363	4.613013	0.691932	0.48935	0.566616	0.31038	not-significant
MIMAT0000732	0.065112	7.463417	0.697647	0.485772	0.565449	0.313568	not-significant
MIMAT0004604	0.108166	6.52453	0.727666	0.467211	0.546736	0.330487	not-significant
MIMAT0000689	-0.05331	14.50265	-0.73561	0.462365	0.543958	0.335015	not-significant
MIMAT0004599	-0.09696	5.194454	-0.7583	0.448684	0.533919	0.34806	not-significant
MIMAT0000760	0.069628	5.187949	0.753757	0.451404	0.533919	0.345435	not-significant
MIMAT0004911	0.063839	4.509874	0.757351	0.449251	0.533919	0.347511	not-significant
MIMAT0004748	0.057481	5.746955	0.780808	0.435341	0.526236	0.36117	not-significant
MIMAT0004501	0.082811	3.454556	0.788073	0.431085	0.52397	0.365437	not-significant
MIMAT0004502	0.049861	11.85981	0.789248	0.430398	0.52397	0.36613	not-significant
MIMAT0000078	-0.04756	11.52648	-0.81199	0.417241	0.512811	0.379613	not-significant
MIMAT0004678	0.070071	6.338724	0.817289	0.414212	0.511948	0.382777	not-significant
MIMAT0004602	0.061337	2.610493	0.826687	0.408869	0.508198	0.388416	not-significant
MIMAT0004694	-0.06907	3.011336	-0.89509	0.371234	0.464042	0.430353	not-significant
MIMAT0002871	-0.08815	7.407741	-0.90318	0.366931	0.461285	0.435415	not-significant
MIMAT0000101	0.050852	14.03149	0.935667	0.349965	0.442485	0.455975	not-significant
MIMAT0000754	-0.11261	3.758086	-0.94825	0.343532	0.436862	0.464033	not-significant
MIMAT0000281	-0.16713	4.977709	-0.96492	0.335124	0.428647	0.474795	not-significant
MIMAT0000104	0.053137	6.522541	0.970688	0.332244	0.427449	0.478542	not-significant
MIMAT0000065	-0.07184	7.660688	-1.04084	0.298529	0.390931	0.525013	not-significant
MIMAT0000764	0.113871	4.939862	1.076577	0.282267	0.371849	0.549339	not-significant
MIMAT0001620	-0.12257	8.85959	-1.08238	0.279684	0.370666	0.553332	not-significant
MIMAT0004500	-0.10003	4.457389	-1.08621	0.277991	0.370655	0.555969	not-significant
MIMAT0004780	-0.11294	5.036879	-1.12225	0.262377	0.351969	0.581074	not-significant
MIMAT0014990	0.094424	2.620584	1.165233	0.244565	0.330088	0.611606	not-significant
MIMAT0001635	-0.16378	6.017984	-1.18911	0.235048	0.3192	0.628844	not-significant

MIMAT0000445	-0.13403	10.89855	-1.19547	0.232557	0.31778	0.63347	not-significant
MIMAT0004600	-0.19436	6.031223	-1.24723	0.212985	0.292854	0.671652	not-significant
MIMAT0000733	0.186947	8.494578	1.312011	0.19021	0.263184	0.720766	not-significant
MIMAT0004697	0.099885	6.458493	1.343417	0.179839	0.250409	0.745115	not-significant
MIMAT0003389	0.15446	7.723787	1.388156	0.165802	0.232334	0.780411	not-significant
MIMAT0002888	-0.11702	9.516897	-1.39223	0.164565	0.232079	0.783662	not-significant
MIMAT0000069	0.094219	8.75564	1.473596	0.141316	0.200577	0.84981	not-significant
MIMAT0004945	0.1089	4.950544	1.50517	0.133008	0.190012	0.876122	not-significant
MIMAT0000077	-0.08382	16.17622	-1.51127	0.131447	0.189009	0.881248	not-significant
MIMAT0000279	-0.14815	6.019933	-1.52409	0.128215	0.185575	0.89206	not-significant
MIMAT0000076	0.087658	18.37682	1.557487	0.120084	0.174957	0.920515	not-significant
MIMAT0000066	-0.13873	10.34691	-1.64786	0.100106	0.146822	0.999541	not-significant
MIMAT0005920	0.180108	3.917936	1.672296	0.095187	0.140544	1.021423	not-significant
MIMAT0003249	0.21599	4.570847	1.679882	0.0937	0.139284	1.02826	not-significant
MIMAT0000510	-0.1234	8.361396	-1.68202	0.093285	0.139284	1.030186	not-significant
MIMAT0000757	0.13408	11.44216	1.716574	0.086771	0.130751	1.061626	not-significant
MIMAT0001631	-0.30098	8.372664	-1.83033	0.067886	0.103	1.168219	not-significant
MIMAT0004484	-0.13664	8.228464	-1.83079	0.067818	0.103	1.168656	not-significant
MIMAT0004688	0.14466	9.274702	1.848628	0.065192	0.100295	1.185808	not-significant
MIMAT0000460	0.368106	7.905992	1.887054	0.05982	0.09268	1.22315	not-significant
MIMAT0000761	0.198577	4.481683	1.927034	0.054628	0.085235	1.262583	not-significant
MIMAT0004588	-0.12398	4.072948	-1.9298	0.054283	0.085235	1.265335	not-significant
MIMAT0001639	0.272852	3.336204	1.961961	0.050406	0.079779	1.297518	not-significant
MIMAT0002177	-0.31614	6.527627	-2.01894	0.044108	0.070318	1.355479	-regulated in Hi
MIMAT0000444	-0.17764	7.602406	-2.03992	0.041964	0.067387	1.377128	-regulated in Hi
MIMAT0004569	0.203707	2.738717	2.047959	0.041166	0.066591	1.385466	-regulated in Lo
MIMAT0000447	0.311992	6.915336	2.083634	0.037778	0.061775	1.422758	-regulated in Lo
MIMAT0000083	-0.15644	9.185833	-2.08222	0.037907	0.061775	1.421275	-regulated in Hi
MIMAT0000280	0.248658	7.784308	2.102569	0.036079	0.05968	1.442745	-regulated in Lo
MIMAT0027587	-0.1594	2.087139	-2.11673	0.034851	0.058085	1.457782	-regulated in Hi
MIMAT0004775	-0.15733	3.347979	-2.13834	0.033048	0.0555	1.480861	-regulated in Hi
MIMAT0004598	0.217484	9.134323	2.160374	0.031291	0.052953	1.504586	-regulated in Lo
MIMAT0004693	0.16493	5.221823	2.186181	0.029336	0.05003	1.532598	-regulated in Lo
MIMAT0004585	-0.21456	6.589898	-2.23119	0.026179	0.044995	1.582047	-regulated in Hi
MIMAT0000435	-0.2442	16.01815	-2.2433	0.025381	0.043968	1.595483	-regulated in Hi
MIMAT0002808	-0.25634	4.240601	-2.26303	0.024128	0.042128	1.617486	-regulated in Hi
MIMAT0000067	-0.22805	13.11321	-2.26673	0.023899	0.042062	1.621628	-regulated in Hi
MIMAT0000254	0.358716	11.93148	2.273383	0.023491	0.041678	1.629096	-regulated in Lo
MIMAT0002174	0.173659	5.66547	2.293856	0.022276	0.039843	1.652164	-regulated in Lo
MIMAT0004563	-0.21323	10.79941	-2.32449	0.02056	0.037075	1.686976	-regulated in Hi
MIMAT0002876	0.19842	5.064872	2.326178	0.020469	0.037075	1.688904	-regulated in Lo
MIMAT0000232	-0.21382	10.80313	-2.33269	0.020121	0.036888	1.696352	-regulated in Hi
MIMAT0000098	-0.26625	12.61757	-2.35409	0.019013	0.03539	1.720942	-regulated in Hi
MIMAT0000703	0.12829	7.752151	2.351531	0.019143	0.03539	1.717997	-regulated in Lo
MIMAT0000227	0.210324	7.989854	2.45153	0.014618	0.027488	1.835099	-regulated in Lo
MIMAT0004766	-0.24712	7.550229	-2.46145	0.014226	0.02698	1.84692	-regulated in Hi
MIMAT0004559	-0.20137	5.656368	-2.49006	0.013146	0.02527	1.881222	-regulated in Hi
MIMAT0000759	0.169486	7.527036	2.488319	0.013209	0.02527	1.879127	-regulated in Lo
MIMAT0006789	-0.23215	2.18175	-2.64081	0.008569	0.016683	2.06706	-regulated in Hi
MIMAT0000278	-0.267	7.982253	-2.71018	0.006991	0.013733	2.155445	-regulated in Hi
MIMAT0005792	0.228202	2.264982	2.726096	0.006668	0.013217	2.17598	-regulated in Lo
MIMAT0003218	0.261575	7.484547	2.863534	0.004393	0.008785	2.357281	-regulated in Lo
MIMAT0004682	-0.19614	7.7304	-2.87395	0.004253	0.008584	2.371317	-regulated in Hi
MIMAT0000417	0.21263	7.492125	2.908398	0.003819	0.00778	2.418004	-regulated in Lo
MIMAT0005878	-0.28707	5.529532	-3.00341	0.002824	0.005807	2.549103	-regulated in Hi
MIMAT0000423	-0.27148	8.897505	-3.0239	0.002643	0.005486	2.577824	-regulated in Hi
MIMAT0000728	-0.57298	13.99865	-3.02932	0.002598	0.005442	2.585444	-regulated in Hi
MIMAT0000752	-0.30323	4.901765	-3.06053	0.002347	0.004964	2.629552	-regulated in Hi

MIMAT0000763	-0.52554	8.752212	-3.20987	0.001427	0.003047	2.845707	-regulated in Hi
MIMAT0000261	0.348414	13.18262	3.216213	0.001396	0.003022	2.855069	-regulated in Lo
MIMAT0000414	-0.20782	9.182383	-3.21521	0.001401	0.003022	2.853583	-regulated in Hi
MIMAT0001341	0.386313	6.015706	3.23407	0.001314	0.00289	2.881518	-regulated in Lo
MIMAT0003886	0.214914	4.018901	3.24567	0.001263	0.002806	2.898763	-regulated in Lo
MIMAT0000440	0.256327	8.657749	3.249463	0.001246	0.002798	2.904414	-regulated in Lo
MIMAT0000455	0.20868	6.310353	3.269016	0.001165	0.002643	2.933624	-regulated in Lo
MIMAT0002821	-0.28014	4.539284	-3.29763	0.001055	0.002418	2.976638	-regulated in Hi
MIMAT0004614	0.273517	7.464158	3.309806	0.001012	0.002342	2.995024	-regulated in Lo
MIMAT0001340	0.234606	6.678301	3.330683	0.00094	0.002201	3.02669	-regulated in Lo
MIMAT0000222	0.685315	9.0381	3.373811	0.000808	0.001911	3.092625	-regulated in Lo
MIMAT0026477	0.248831	2.40359	3.392095	0.000757	0.001811	3.120789	-regulated in Lo
MIMAT0004564	-0.34697	4.124411	-3.42148	0.000682	0.001648	3.166312	-regulated in Hi
MIMAT0017994	0.245637	2.409866	3.458573	0.000597	0.001459	3.22424	-regulated in Lo
MIMAT0004701	-0.47451	3.091523	-3.5471	0.000432	0.001068	3.364573	-regulated in Hi
MIMAT0004611	0.293179	3.033524	3.555549	0.000419	0.001047	3.378114	-regulated in Lo
MIMAT0004558	-0.29752	9.942094	-3.67432	0.000268	0.000679	3.57135	-regulated in Hi
MIMAT0001343	0.263207	3.427372	3.70515	0.000239	0.00061	3.622368	-regulated in Lo
MIMAT0000250	-0.41568	4.99223	-3.74909	0.000202	0.000522	3.695686	-regulated in Hi
MIMAT0000257	-0.29787	9.406495	-3.77605	0.000182	0.000475	3.741037	-regulated in Hi
MIMAT0004552	-0.39993	3.434041	-3.80872	0.00016	0.000424	3.796346	-regulated in Hi
MIMAT0004549	-0.41645	5.85015	-3.82065	0.000153	0.000409	3.816639	-regulated in Hi
MIMAT0004603	-0.38157	3.354207	-3.85424	0.000134	0.000363	3.87406	-regulated in Hi
MIMAT0000772	0.461319	3.917429	3.899748	0.000112	0.000307	3.952521	-regulated in Lo
MIMAT0000318	-0.43953	9.21896	-3.91148	0.000106	0.000296	3.972866	-regulated in Hi
MIMAT0000451	-0.51181	9.631605	-3.92976	9.89E-05	0.000279	4.004693	-regulated in Hi
MIMAT0000762	0.282337	4.41006	3.961848	8.69E-05	0.000248	4.060826	-regulated in Lo
MIMAT0000756	-0.50509	4.148615	-3.98067	8.06E-05	0.000233	4.093928	-regulated in Hi
MIMAT0019927	0.382097	2.754555	3.993881	7.63E-05	0.000224	4.117249	-regulated in Lo
MIMAT0000100	-0.40003	9.596472	-4.00284	7.36E-05	0.000219	4.1331	-regulated in Hi
MIMAT0003888	0.347664	3.283227	4.103787	4.86E-05	0.000146	4.313703	-regulated in Lo
MIMAT0000255	-0.36729	7.660419	-4.20267	3.20E-05	9.79E-05	4.494239	-regulated in Hi
MIMAT0000062	-0.3309	14.79532	-4.20974	3.11E-05	9.64E-05	4.507289	-regulated in Hi
MIMAT0000710	0.407087	5.051394	4.26432	2.46E-05	7.79E-05	4.608601	-regulated in Lo
MIMAT0022834	0.406942	5.051396	4.262792	2.48E-05	7.79E-05	4.60575	-regulated in Lo
MIMAT0000071	0.336408	8.359605	4.323718	1.91E-05	6.16E-05	4.720095	-regulated in Lo
MIMAT0004946	0.485985	3.426824	4.382683	1.47E-05	4.83E-05	4.832045	-regulated in Lo
MIMAT0000243	-0.43689	15.21487	-4.3899	1.43E-05	4.75E-05	4.845824	-regulated in Hi
MIMAT0004485	-0.3465	3.671432	-4.67072	4.01E-06	1.36E-05	5.396938	-regulated in Hi
MIMAT0000426	0.286616	6.687608	4.677444	3.89E-06	1.34E-05	5.410479	-regulated in Lo
MIMAT0000419	-0.31185	10.74525	-4.75567	2.70E-06	9.42E-06	5.569221	-regulated in Hi
MIMAT0000267	0.82996	9.383735	4.761638	2.62E-06	9.30E-06	5.581416	-regulated in Lo
MIMAT0000270	-0.37773	8.384288	-4.78069	2.40E-06	8.64E-06	5.620446	-regulated in Hi
MIMAT0022727	0.617092	8.208841	4.803125	2.15E-06	7.90E-06	5.666573	-regulated in Lo
MIMAT0004494	0.431491	11.58352	4.808324	2.10E-06	7.84E-06	5.677286	-regulated in Lo
MIMAT0003266	-0.44608	3.790133	-4.81705	2.02E-06	7.65E-06	5.695283	-regulated in Hi
MIMAT0003247	0.641003	3.648131	4.864484	1.61E-06	6.21E-06	5.793638	-regulated in Lo
MIMAT0000075	0.485643	7.671487	4.943279	1.10E-06	4.32E-06	5.958763	-regulated in Lo
MIMAT0004799	0.372056	6.507229	4.965588	9.86E-07	3.95E-06	6.005911	-regulated in Lo
MIMAT0000693	-0.3588	12.14032	-5.0282	7.26E-07	2.96E-06	6.139171	-regulated in Hi
MIMAT0000437	-0.52815	9.923492	-5.06002	6.20E-07	2.57E-06	6.207418	-regulated in Hi
MIMAT0004509	0.451862	3.213631	5.061618	6.15E-07	2.57E-06	6.210859	-regulated in Lo
MIMAT0000263	-0.55248	6.994181	-5.19134	3.21E-07	1.39E-06	6.492836	-regulated in Hi
MIMAT0003241	0.423074	3.256937	5.239941	2.51E-07	1.11E-06	6.599973	-regulated in Lo
MIMAT0004680	0.520182	3.776895	5.264393	2.22E-07	9.98E-07	6.65419	-regulated in Lo
MIMAT0004673	-0.47517	4.491462	-5.26393	2.22E-07	9.98E-07	6.653167	-regulated in Hi
MIMAT0000231	-0.52448	9.386931	-5.33623	1.53E-07	7.17E-07	6.814668	-regulated in Hi
MIMAT0000092	0.412853	13.08614	5.361038	1.35E-07	6.44E-07	6.870494	-regulated in Lo

MIMAT0004550	-0.59767	4.68481	-5.40641	1.06E-07	5.20E-07	6.973156	-regulated in Hi
MIMAT0009451	-0.37604	3.861532	-5.44428	8.72E-08	4.36E-07	7.059359	-regulated in Hi
MIMAT0000244	-0.50197	8.528468	-5.45092	8.42E-08	4.31E-07	7.074542	-regulated in Hi
MIMAT0000692	-0.35564	12.47447	-5.55584	4.83E-08	2.53E-07	7.316207	-regulated in Hi
MIMAT0000269	0.507739	2.999836	5.582054	4.20E-08	2.25E-07	7.377172	-regulated in Lo
MIMAT0000420	-0.5726	8.842332	-5.59513	3.91E-08	2.15E-07	7.407659	-regulated in Hi
MIMAT0005825	0.517178	4.646873	5.738266	1.80E-08	1.01E-07	7.745269	-regulated in Lo
MIMAT0000086	-0.45736	13.45171	-5.8839	8.02E-09	4.64E-08	8.095796	-regulated in Hi
MIMAT0004597	-0.37293	9.773451	-5.9709	4.91E-09	2.92E-08	8.308557	-regulated in Hi
MIMAT0004482	-0.43854	4.360654	-5.9805	4.65E-09	2.84E-08	8.332189	-regulated in Hi
MIMAT0004797	0.879019	7.676654	6.030233	3.51E-09	2.20E-08	8.455084	-regulated in Lo
MIMAT0002819	0.797234	5.660669	6.046927	3.19E-09	2.06E-08	8.496519	-regulated in Lo
MIMAT0000088	-0.72416	11.54978	-6.06601	2.86E-09	1.91E-08	8.543998	-regulated in Hi
MIMAT0000258	-0.54562	5.742258	-6.08287	2.59E-09	1.78E-08	8.58603	-regulated in Hi
MIMAT0000081	0.397602	12.81074	6.104412	2.29E-09	1.63E-08	8.639897	-regulated in Lo
MIMAT0000418	-0.37107	10.30317	-6.16961	1.57E-09	1.15E-08	8.803822	-regulated in Hi
MIMAT0000443	-0.4767	9.024999	-6.19071	1.39E-09	1.05E-08	8.857147	-regulated in Hi
MIMAT0004703	0.533778	6.621902	6.218167	1.18E-09	9.30E-09	8.926789	-regulated in Lo
MIMAT0019729	0.574812	2.041526	6.271798	8.64E-10	7.04E-09	9.063499	-regulated in Lo
MIMAT0005797	0.56506	3.726692	6.404357	3.93E-10	3.38E-09	9.405364	-regulated in Lo
MIMAT0005949	-0.49446	4.611662	-6.4019	3.99E-10	3.38E-09	9.398985	-regulated in Hi
MIMAT0015378	-1.01995	6.359898	-6.44988	2.99E-10	2.74E-09	9.52406	-regulated in Hi
MIMAT0000441	1.483642	9.591951	6.46119	2.79E-10	2.67E-09	9.553651	-regulated in Lo
MIMAT0000064	-0.65002	10.03192	-6.53067	1.84E-10	1.84E-09	9.736328	-regulated in Hi
MIMAT0003393	0.558448	7.224637	6.553793	1.59E-10	1.67E-09	9.797456	-regulated in Lo
MIMAT0000070	0.634196	8.251375	6.670571	7.78E-11	8.56E-10	10.10875	-regulated in Lo
MIMAT0022272	-0.4562	2.384391	-6.83459	2.80E-11	3.24E-10	10.55313	-regulated in Hi
MIMAT0000063	-0.55083	13.70984	-6.86071	2.37E-11	2.90E-10	10.62467	-regulated in Hi
MIMAT0004515	-0.73418	4.882747	-6.96998	1.19E-11	1.53E-10	10.92618	-regulated in Hi
MIMAT0000245	-0.66183	13.35176	-7.06459	6.45E-12	8.87E-11	11.19014	-regulated in Hi
MIMAT0004601	-0.5506	4.872039	-7.09188	5.41E-12	7.94E-11	11.26678	-regulated in Hi
MIMAT0000087	-0.81224	13.39824	-7.18138	3.02E-12	4.75E-11	11.51971	-regulated in Hi
MIMAT0000256	-0.55923	11.41737	-7.19334	2.79E-12	4.73E-11	11.5537	-regulated in Hi
MIMAT0005951	0.563436	9.846924	7.319402	1.22E-12	2.23E-11	11.91444	-regulated in Lo
MIMAT0000093	0.647202	11.80115	7.35364	9.70E-13	1.94E-11	12.01322	-regulated in Lo
MIMAT0000099	-0.54785	13.33054	-7.53008	2.97E-13	6.75E-12	12.52771	-regulated in Hi
MIMAT0000082	-0.4749	11.38957	-7.52512	3.07E-13	6.75E-12	12.51313	-regulated in Hi
MIMAT0000681	-0.72513	11.45656	-7.72482	7.84E-14	2.15E-12	13.10591	-regulated in Hi
MIMAT0004672	0.656825	8.359937	7.741254	6.99E-14	2.15E-12	13.15521	-regulated in Lo
MIMAT0002820	-0.61479	4.23465	-7.82963	3.79E-14	1.39E-12	13.42157	-regulated in Hi
MIMAT0004810	0.593417	6.762254	7.861957	3.02E-14	1.33E-12	13.51955	-regulated in Lo
MIMAT0000097	-0.76153	8.038139	-7.98455	1.28E-14	7.02E-13	13.89373	-regulated in Hi
MIMAT0004985	0.609604	2.577637	8.173844	3.31E-15	2.43E-13	14.47961	-regulated in Lo
MIMAT0000424	0.561186	7.076364	8.532947	2.41E-16	2.65E-14	15.61739	-regulated in Lo
MIMAT0000680	0.59582	7.943196	8.723224	5.84E-17	1.28E-14	16.23388	-regulated in Lo

gh
gh
ow
ow
gh
ow
gh
gh
ow
ow
gh
gh
gh
gh
ow
ow
gh
ow
gh
gh
ow
ow
gh
gh
ow
gh
gh
ow
ow
gh
ow
gh
gh
gh
gh

gh
ow
gh
ow
ow
ow
ow
gh
ow
ow
ow
ow
gh
ow
gh
ow
gh
ow
gh
gh
gh
gh
gh
ow
gh
gh
ow
gh
ow
gh
ow
gh
gh
ow
ow
ow
ow
gh
gh
ow
gh
ow
gh
ow
ow
gh
ow
ow
ow
gh
gh
ow
gh
ow
ow
gh
gh
ow

gh
gh
gh
gh
ow
gh
ow
gh
gh
gh
ow
ow
gh
gh
ow
gh
gh
ow
ow
ow
gh
gh
ow
gh
ow
ow
gh
gh
gh
gh
gh
gh
gh
ow
ow
gh
gh
gh
ow
gh
ow
gh
ow
ow
ow
