# Supplementary material for: Identification and Validation of a Proliferation-Associated Score Model Predicting Survival in Lung Adenocarcinomas
Source: Dis Markers. 2021 Oct 21;2021:3219594. doi: 10.1155/2021/3219594 (PMC8554523; doi:10.1155/2021/3219594)
Supplement: Supplementary 2 — Table S1: the table showed genes associated with microenvironment of the 24 immune cell subsets. Table S2: the table showed the sequences of all the siRNAs and primers used in this study. Table S3: the table showed 55 genes selected for LASSO Cox regression; all the 55 genes showed the same tendency in cell proliferation (the CERES dependency score) and survival (HR). Table S4: the table showed six genes used in the model and their LASSO coefficient after LASSO Cox regression. Table S5: the table showed the summary of genomic alterations in the two groups, including the somatic mutation numbers of each gene in high and low score groups. Table S6: the table showed the differentially expressed genes (DEGs) between high score group and low score group identified by limma. Table S7: the table showed the differentially expressed miRNAs between high score group and low score group identified by limma. Table S8: the table showed the comparison the abundance of 24 types of immune cells between the two groups by Wilcoxon test. [file 3219594.f2.zip › Table S8.pdf]

Table S8. Compare the abundance of 24 types of immune cells between the two groups by Wilcoxon test.

Cells	P-value
Th2.cells	4.45E-26
Mast.cells	2.26E-25
Eosinophils	4.71E-22
iDC	1.42E-21
CD8.T.cells	7.35E-19
TFH	3.59E-17
Macrophages	1.80E-14
DC	4.33E-12
Tem	8.43E-10
NK.cells	5.72E-08
pDC	3.54E-07
Th17.cells	5.04E-07
T.cells	1.26E-06
B.cells	4.19E-06
Tcm	1.52E-05
Th1.cells	1.62E-05
Cytotoxic.cells	0.001491371
NK.CD56dim.cells	0.002449679
NK.CD56bright.cells	0.003427445
Neutrophils	0.005699242
Tgd	0.039358431
aDC	0.057893432
TReg	0.128802163
T.helper.cells	0.71814262
Cells	P-value
