# Supplementary figures and images for: Identification and Validation of a Proliferation-Associated Score Model Predicting Survival in Lung Adenocarcinomas
Source: Dis Markers. 2021 Oct 21;2021:3219594. doi: 10.1155/2021/3219594 (PMC8554523; doi:10.1155/2021/3219594)

A

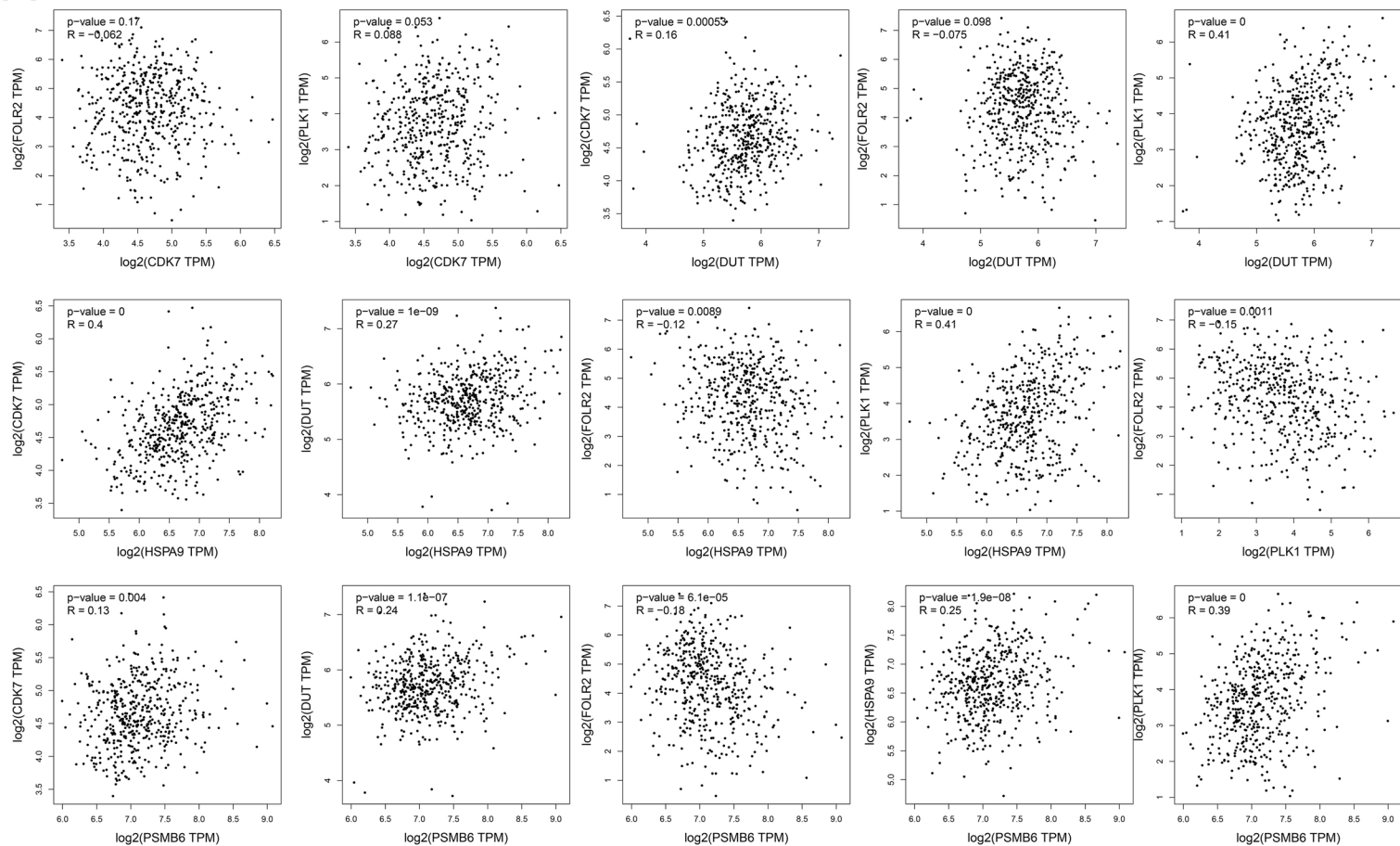

B

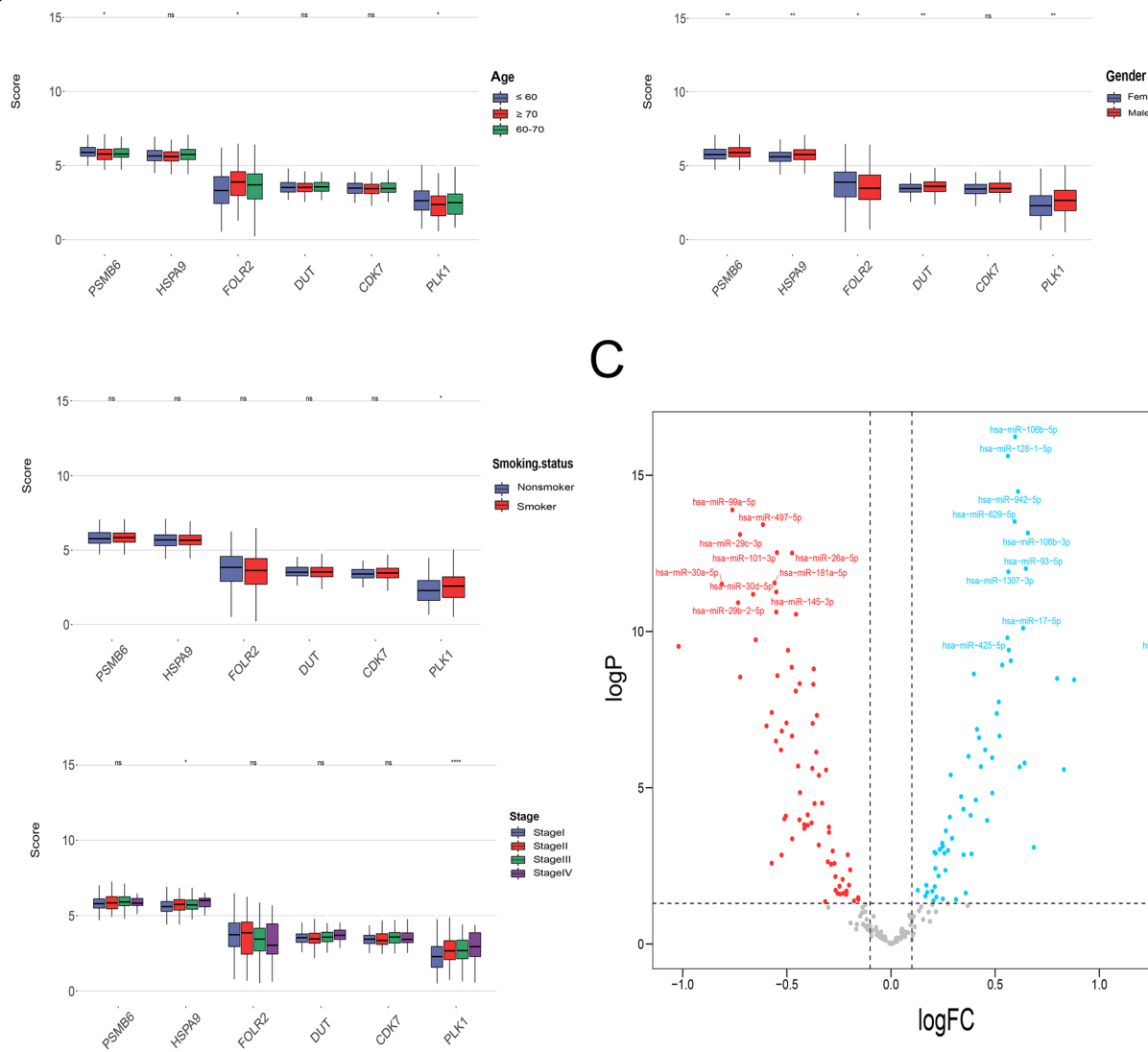

C

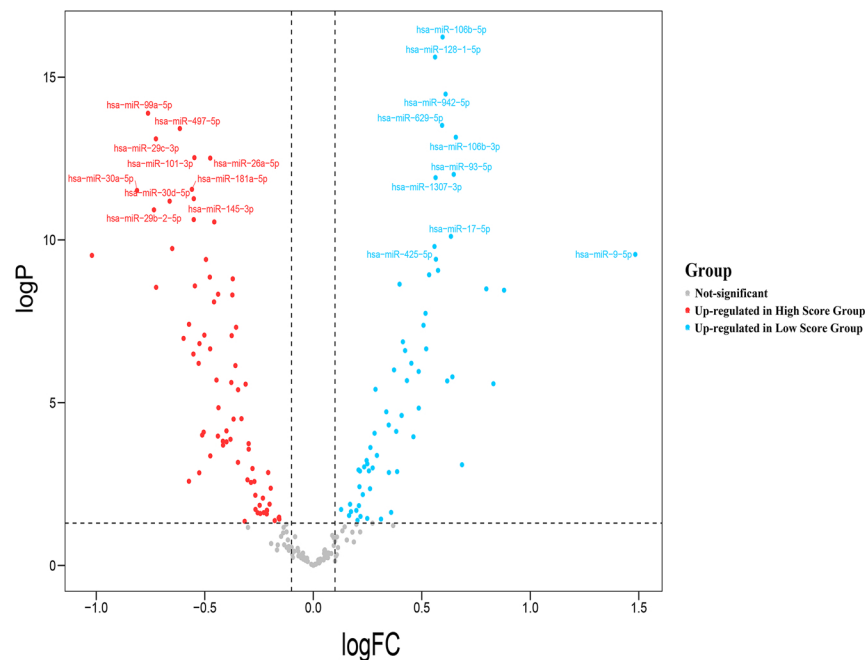

Supplement: Supplementary 3 — Figure S1 (A–C) (A) The scatter dot plots show the relationships of expression values between the six genes we selected in the score models. (B) The correlation between clinical characteristics (sex, age, stage, and smoking) and gene expression. The scattered dots show the immune cells' score. The median, third, and first quartile values are shown in the boxplots. ∗P < 0.05; ∗∗P < 0.01; ∗∗∗P < 0.001; ∗∗∗∗P < 0.0001. (C) The volcano plot displays the differentially expressed miRNAs of the two groups. [file 3219594.f3.pdf]
